# Vascular-associated bacterial burden and neuroinflammatory transcriptional responses observed in models of pneumonic plague

**DOI:** 10.3389/fmicb.2026.1865125

**Published:** 2026-06-24

**Authors:** Brian A. Smith, Christopher P. Klimko, Carlos I. Rodriguez, Nathaniel O. Rill, Michael L. Davies, Jennifer L. Dankmeyer, Melissa Hunter, Christopher T. Braun, Yunuen Hernandez-Viezcas, Christian J. Xander, Elsie I. Martinez, Ronald G. Toothman, Christina E. Douglas, Christopher P. Stefan, Kevin D. Mlynek, Joel A. Bozue, Sara I. Ruiz, Nancy A. Twenhafel, Charles J. Shoemaker, Ju Qiu, Sergei S. Biryukov, Christopher K. Cote

**Affiliations:** 1 Bacteriology Division, United States Army Medical Research Institute of Infectious Diseases (USAMRIID), Frederick, MD, United States; 2 Diagnostic Systems Division, United States Army Medical Research Institute of Infectious Diseases (USAMRIID), Frederick, MD, United States; 3 Pathology Division, United States Army Medical Research Institute of Infectious Diseases (USAMRIID), Frederick, MD, United States; 4 Biostatistics Branch, United States Army Medical Research Institute of Infectious Diseases (USAMRIID), Frederick, MD, United States

**Keywords:** astrocyte, brain, *Burkholderia pseudomallei*, expression, melioidosis, meningitis, neuroinflammation, plague

## Abstract

**Introduction:**

*Yersinia pestis* is the etiologic agent of plague, and the disease is categorized into several forms, including bubonic, septicemic, and pneumonic. Plague meningitis is a rare but severe complication and estimated to occur in 6–11% of documented cases. It is most frequently observed in bubonic plague patients under 15 years old that receive inadequate or no antibiotic treatment. To date, there are no reports describing plague meningitis in laboratory animal models of pneumonic plague.

**Methods:**

Therefore, we sought to use the BALB/c mouse pneumonic plague model to investigate central nervous system (CNS) involvement after exposure to aerosolized *Y. pestis*. We used a multifaceted approach analyzing bacterial burden, histopathological analyses, transcriptomic data, and cytokine expression in mice exposed to aerosolized *Y. pestis* CO92 collected at intervals post-exposure for 3 days.

**Results:**

*Y. pestis* was detected in brain homogenates as early as 2 days post challenge. CNS involvement is further supported by increased pro-inflammatory cytokine expression in the brain homogenates including IL-6. Histopathological analyses conducted in mice and confirmed in non-human primate tissue sections did not demonstrate meningitis but rather indicated that the bacteria remain within the blood vessels of the cerebellum, cerebrum, and nasal turbinates. However, transcriptomic data targeting mouse neuroinflammatory responses indicated alterations in several transcriptional signatures of gene sets, including those that regulate astrocyte, oligodendrocyte, and microglial cell functions.

**Discussion:**

While *Y. pestis* does not appear to breach the blood vessels resulting in meningitis in our acute models of pneumonic plague, we found evidence of a neuroinflammatory response within the brain homogenates of infected mice. We also compared this mouse model of pneumonic plague to a mouse model of inhalational melioidosis, a known neuroinvasive disease caused by *Burkholderia pseudomallei*. The establishment of a murine model of plague-induced neuroinflammation described herein will contribute to the refinement of animal models, development of medical countermeasures for neurological infections or neurological impacts associated with systemic infection, and improvement of diagnostic strategies for *Y. pestis*.

## 1 Introduction

*Yersinia pestis* is a gram-negative, nonmotile, facultative intracellular, coccobacillus and the etiologic agent of plague (Perry and Fetherston, 1997; Barbieri et al., 2020). Although it primarily spreads via fleas and is maintained in rodent reservoir populations, humans are accidental hosts and can become infected through flea bites, contaminated body fluids, or aerosolized droplets. Historically, *Y. pestis* has caused three pandemics including the “Black Death” period (1346–1353) during the second plague pandemic which killed approximately 30–40% of the European population (Perry and Fetherston, 1997; Barbieri et al., 2020; Mas Fiol et al., 2023; Riedel, 2005). Today, plague remains endemic in several regions of the world, including in the western United States, with notable recent outbreaks occurring in the Democratic Republic of the Congo and Madagascar (Barbieri et al., 2020; Andrianaivoarimanana et al., 2024).

Clinical presentations of *Y. pestis* include bubonic, septicemic, and pneumonic plague attacking the lymphatic, vascular, and respiratory systems, respectively (Perry and Fetherston, 1997; Ditchburn and Hodgkins, 2019). After initial infection, plague can progress into secondary infections (e.g., primary bubonic plague resulting in secondary pneumonic plague). Bubonic plague is the most common form and is typically characterized by swollen and tender lymph nodes referred to as “buboes.” Primary septicemic plague is similar to other examples of bacterial septicemia and is typically diagnosed from a positive blood culture in the absence of lymphadenopathy (Perry and Fetherston, 1997; Dennis, 2005). Primary pneumonic plague is rare, extremely lethal, can spread person-to-person, and causes rapid febrile flu-like symptoms often resulting in death in as little as 72 h (Perry and Fetherston, 1997; Pechous et al., 2016; Venugopal and Pechous, 2024). *Y. pestis* may also cause meningitis in 6–11% of cases, with higher incidence in younger patients (Becker et al., 1987; Valerio et al., 2021). Plague meningitis is not well characterized but existing case reports resemble other forms of bacterial meningitis with patients experiencing fever, nuchal rigidity, headache, and focal neurological deficits (Feeley and Kriz, 1965; Landsborough and Tunnell, 1947; Cooley et al., 2023). Most neurological cases were secondary to bubonic plague, with only 6% secondary to pneumonic plague. There are only five reported cases of primary plague meningitis (Landsborough and Tunnell, 1947; Tuan et al., 1971; Lewillon et al., 1940; Tovar Padua et al., 2017; Singh, 1951). Without antibiotic treatment, plague meningitis is fatal in 96% of cases. Even with medical intervention, the fatality rate remains high at 42% (Cooley et al., 2023). Given its potential for rapid, fatal disease and classification as a biothreat agent, the U.S. Department of Health and Human Services designates *Y. pestis* as a Tier 1 select agent, underscoring the critical need to understand pathogenesis of all plague forms (Riedel, 2005; Dennis, 2005; Select Agents and Toxins List, 2025).

Each of the clinical manifestations and the acute lethality of plague are due to several virulence factors encoded on multiple plasmids within the *Y. pestis* genome including but not limited to: a type three secretion system (T3SS) with effectors (Yops), an antiphagocytic capsular protein (F1), and a plasminogen associated protease (Perry and Fetherston, 1997; Demeure et al., 2019; Atkinson and Williams, 2016). Depending on the route of infection these virulence factors can have differential effects with varying degrees of importance and ultimately result in evasion and modulation of the host immune responses resulting in rapid progression of *Y. pestis* infection (Pechous et al., 2016; Venugopal and Pechous, 2024; Sebbane et al., 2020; Lathem et al., 2007; Pechous et al., 2013).

Well-characterized laboratory animal models of plague include mice, guinea pigs, rats, and non-human primates (Lawrenz, 2010). Mouse models have been particularly useful for bacterial pathogenesis studies as well as medical countermeasure discovery and evaluation. While flea-borne transmission is likely the most relevant animal model in context of naturally acquired disease, several mouse models of infection using intranasal instillation or small-particle aerosol delivery have been described in strains of mice including inbred strains (e.g., BALB/c and C57BL/6) and outbred strains (e.g., Swiss Webster) that recapitulate pneumonic plague (Jarrett et al., 2004). To date, however, these mouse models have not attempted or failed to detect plague meningitis or other neuroinflammatory responses associated with plague, and very little data exist regarding this topic.

In this study, we identified brain-associated involvement in a mouse model of pneumonic plague using a multifaceted approach that included analyzing bacterial burden, histopathology, cytokine levels, and host transcriptomic patterns. Furthermore, we compared infection with *Y. pestis* to infection with *Burkholderia pseudomallei* ATS2021, which causes highly invasive and destructive neurologic melioidosis in a mouse aerosol challenge model. We examined the differences and similarities between these animal models using two mouse strains with distinct immunological profiles. *Y. pestis* was identified in brain homogenates of infected mice; however, histopathological analysis revealed that the bacteria were confined within blood vessels of the brain and meninges and did not disseminate into surrounding parenchymal tissues. Despite the absence of direct tissue invasion or meningitis, we observed changes in cytokine and transcriptomic profiles of brain homogenates suggesting alterations to glial cell function tied to the CNS immune response and blood brain barrier (BBB) permeability. To our knowledge, this report is among the first to describe neuroinflammatory transcriptional responses in laboratory animal models exposed to aerosolized *Y. pestis*. These data will help with efforts toward refining animal models, discovering new medical countermeasures, and developing novel diagnostic strategies for *Y. pestis* infections.

## 2 Materials and methods

### 2.1 Animal research

The animal research was conducted under an Institutional Animal Care and Use Committee (IACUC) approved protocol in compliance with the Animal Welfare Act, Public Health Service Policy on Humane Care and Use of Laboratory Animals, and other federal statutes and regulations relating to animals and experiments involving animals. USAMRIID is accredited by the AAALAC International and adheres to the principles stated in *The Guide for the Care and Use of Laboratory Animals* (National Research Council (US), 2011). Mice were checked daily for food and water and at least daily for assessment of clinical impact of the *Y. pestis* infection. Whenever possible, euthanasia of moribund animals was conducted in accordance with approved early endpoint intervention criteria. Mice were evaluated daily after exposure to aerosolized *Y. pestis*; scores of 0–2 represented normal mice, scores of 3–7 indicated significant clinical manifestations and these mice warranted multiple clinical assessments per day, and final scores of 8 or greater indicated severe clinical condition and mice were euthanized immediately. When mice met pre-determined euthanasia criteria and they were not in the sampling cohorts, mice were euthanized by CO_2_ exposure (flow rate 6–11 ft^3^/h) or by barbiturate overdose through intraperitoneal injection (approximately 0.15 mL for 20 g of body weight) of Euthasol® euthanasia solution (or equivalent) and then death was confirmed by cervical dislocation. Non-human primates (NHPs) were purchased via an approved vendor. NHPs were checked daily for food and water and at least once daily for assessment of clinical impact of the *Y. pestis* infection. NHPs were individually housed upon transfer to ABSL-3 to prevent spread of disease between animals and to increase personnel safety while working under ABSL-3 laboratory conditions. Whenever possible, euthanasia of moribund animals was conducted in accordance with approved early endpoint intervention criteria. NHPs were evaluated daily after exposure to aerosolized *Y. pestis*; scores of 0–1 represented normal NHPs, scores of 2–3 indicated significant clinical manifestations and these NHPs warranted multiple clinical assessments per day, and final scores of 4 or greater indicated severe clinical condition and were euthanized immediately. When NHPs met pre-determined euthanasia criteria, NHPs were deeply anesthetized via an intramuscular injection of Telazol (>6 mg/kg) and then euthanized by barbiturate overdose through intracardiac injection (approximately 0.3–0.4 mL/kg) of Euthasol® euthanasia solution (or equivalent) and then death was confirmed at minimum 10 min post-administration of euthanasia solution.

### 2.2 Bacterial growth conditions and exposure of mice to aerosolized *Y. pestis*

Culture of *Y. pestis* and exposure of mice to aerosolized bacteria were conducted using previously published methods (Cote et al., 2021). Briefly, colonies of *Y. pestis* CO92 were taken from a tryptose blood agar (Difco, Becton Dickinson, Sparks, MD) slant and suspended in heart infusion broth (HIB) (Difco, Becton Dickinson, Sparks, MD) with 0.2% xylose (Sigma Aldrich, St. Louis, MO) and incubated at 28–30 °C with shaking at 150 RPM for 24 h. Cultures were then harvested by centrifugation and suspended in HIB without xylose and mice were exposed via aerosolization of the bacteria as previously described (Cote et al., 2021; Heine et al., 2007; Doll et al., 1994). Female BALB/c mice (Charles River, Frederick, MD, 7–9 weeks at time of exposure to *Y. pestis*) were placed in wire mesh cages inside whole-body aerosol chambers inside a class three biological safety cabinet within a BSL-3 laboratory. The aerosol exposure was generated using a 3-jet collision nebulizer (CH Technologies, Westwood, NJ) and controlled by an automated bioaerosol exposure system (Biaera Technologies, Hagerstown, MD). The system generated a target aerosol of 1–3 μm mass median aerodynamic diameter determined by aerodynamic particle sizer (TSI, Inc., Shoreview, MN). Samples of the aerosol were collected from the exposure chamber using an all-glass impinger (Ace Glass Inc., Vineland, NJ) and samples were assessed to determine the inhaled dose for each animal. Inhaled doses, collected by using an all-glass impinger, were estimated by serially diluting samples onto sheep blood agar and using the mouse weight and Guyton’s formula (Guyton, 1947). The LD_50_ value used for BALB/c mice is 6.8 × 10^4^ inhaled CFU of *Y. pestis* CO92 (Heine et al., 2007) the average inhaled dose and LD_50_ equivalents are described in the legend of each figure. At one, two, and approximately 3 days post challenge (3DPC ranged from 60 h to 66 h post-exposure to aerosolized *Y. pestis* as indicated in figure legends), mice underwent a terminal blood collection under deep anesthesia, euthanized and then, spleens, lungs, and brains were harvested. Organs were homogenized 1 mL of PBS using 15 mL capacity Covidien™ Precision disposable tissue grinder systems (Covidien, Dublin, Ireland) and homogenates were diluted and plated on 5% sheep blood agar (SBA) plates for CFU enumeration and subsequent analysis. Importantly, the mice were not perfused prior to tissue collection.

### 2.3 Non-human primate tissue sharing efforts for histopathological analyses

To confirm our findings are not mouse specific we employed tissue sharing protocols to examine brain samples from NHPs exposed to aerosolized *Y. pestis* CO92. In this previously conducted study four (2 male and 2 female) *Chlorocebus aethiops* (African Green Monkeys) weighing 3.236–5.914 kg were assigned to the study. All animals were housed individually and provided LabDiet Laboratory Fiber-Plus Monkey Diet daily and *ad libitum* water. *Y. pestis* CO92 was grown as described above. NHPs were anesthetized immediately prior to plethysmography and aerosol exposure. The time-calculated aerosol exposure was generated using a 3-jet collision nebulizer (CH Technologies, Westwood, NJ) and controlled by an automated bioaerosol exposure system (Biaera Technologies). The system generated a target aerosol of 1 to 3 μm mass median aerodynamic diameter determined by aerodynamic particle sizer (TSI, Inc., Shoreview, MN). Samples of the aerosol were collected from the exposure chamber using an all-glass impinger (Ace Glass Inc., Vineland, NJ) and samples were assessed to determine the inhaled dose for each animal. The NHPs described here received an inhaled dose of either 696 CFU or 4.99×10^6^ (as indicated in figure legends). NHPs were monitored a minimum of once-a-day post-exposure for clinical signs of disease to include respiratory rate, responsiveness, appearance, and food consumption up to 14 days post-exposure. Early end-point euthanasia criteria were utilized whenever possible, and a full necropsy was performed on all major organ systems. Select tissues were collected for homogenization to determine bacterial burden.

### 2.4 Luminex cytokine assay

Cytokine concentrations were measured in brain homogenates using the Mouse ProcartaPlex Cytokine and Chemokine 36-Plex (Thermo Fisher, Waltham, MA) on a MagPix (Thermo Fisher) according to the manufacturer’s instructions. Homogenates were pelleted via microcentrifugation and cleared supernatants were analyzed undiluted. All tissue homogenate samples were run in duplicate. Cytokine concentrations were log10-transformed prior to analysis and analyzed using linear mixed effects model. This transformation ensured that the residuals of the model met the assumptions of normality of the residuals and homogeneity of variance. Linear mixed models are relatively robust to non-normality in the outcome variable. Analytes below or above the limit of quantitation were imputed as the lower or upper limit of quantitation, respectively. Statistical analysis was done using Statistical Analysis Software (SAS Institute Inc., Cary, NC). Pairwise treatment groups were compared by linear mixed effects model.

### 2.5 Histopathology

Non-perfused tissues were collected from euthanized BALB/c mice including head, nasal turbinates, brain (coronal sections of olfactory bulb, cerebrum, and cerebellum), spinal cord, lung, and spleen. In a “tissue sharing” effort, sections of brains obtained from African Green monkeys (AGMs) exposure to aerosolized *Y. pestis* CO92 were also collected for confirmatory analysis. These tissues were immersed in 10% neutral buffered formalin for at least 21 days and then embedded in paraffin, sectioned and stained with hematoxylin and eosin (HE). Immunohistochemistry (IHC) was performed on tissue types listed above using the Dako Envision system (Dako Agilent Pathology Solution, Carpinteria, CA, USA) or using Bond RX stainer (Leica Biosystems, Nussloch Germany). Rabbit polyclonal anti-*Y. pestis* antibodies (971, USAMRIID, Frederick, MD, USA) were used at a dilution of 1:5,000. The sections were dehydrated, cleared with Xyless II (LabChem Inc., Zelienople, PA, USA), and then cover slipped.

### 2.6 RNA isolation and differential gene expression analysis

Homogenates of non-perfused brains were inactivated using TRIzol LS (Thermo Fisher Scientific, Rockville, MD) at a 3:1 ratio, and total RNA was extracted as previously described (Cote et al., 2024). Host gene expression was analyzed with the NanoString nCounter Mouse Neuroinflammation Panel on the SPRINT Profiler platform (Bruker Inc., Billerica, MA) that encompasses 770 targets. Briefly, to prepare a master mix, 70 μL of hybridization buffer was combined with the reporter code set. Then, 8 μL of this mixture was added to 50 ng of extracted host RNA and 2 μL of the capture code set. The reaction was incubated at 65 °C for 17 h, followed by incubation at 4 °C until placement on the NanoString SPRINT Profiler, where total fluorescent counts corresponding to target hybridization were recorded. Count data were extracted from NanoString RCC files and analyzed using nCounter Advanced Analysis Software according to the manual. Housekeeping probes used for normalization were selected based on the geNorm algorithm (Vandesompele et al., 2002). Any housekeeping genes with <100 counts were removed from the normalization process. Filtering thresholds for the removal of target genes were set to <20 counts and <0.5 observation frequency. Unless otherwise stated, differentially expressed genes were determined by a ≥ | ± 1| log2 fold change (LFC) cutoff and an adjusted *p*-value of <0.05 calculated using the Benjamini-Yekutieli method when compared to unchallenged mice (Benjamini and Yekutieli, 2001). Gene set analysis was conducted using a directed global significance score to summarize the change in regulation of genes tied to functional pathways when compared to unchallenged mice via a composite score. The NanoString gene sets are provided as Supplementary Table S1. Pathview was used to visualize gene expression data in Kyoto Encyclopedia of Genes and Genomes (KEGG) pathways. Pathview superimposes significant differentially expressed genes onto KEGG pathway maps (Luo and Brouwer, 2013).

### 2.7 *Burkholderia pseudomallei* ATS2021

All raw data regarding *B. pseudomallei* utilized for this data comparison analysis was originally generated in the study described in our previous publication (Cote et al., 2024). Female C57BL/6 mice (Charles River, Frederick, MD, 7–9 weeks at time of exposure to *B. pseudomallei*) were exposed to aerosolized *B. pseudomallei* ATS2021 and samples were collected at intervals post-challenge. We analyzed data using the study group challenged with an inhaled dose of approximately 1,150 CFUs, following the methods described above.

## 3 Results

### 3.1 *Y. pestis* is present in the brain homogenates from mice exposed to aerosolized bacteria

**Figure 1:**
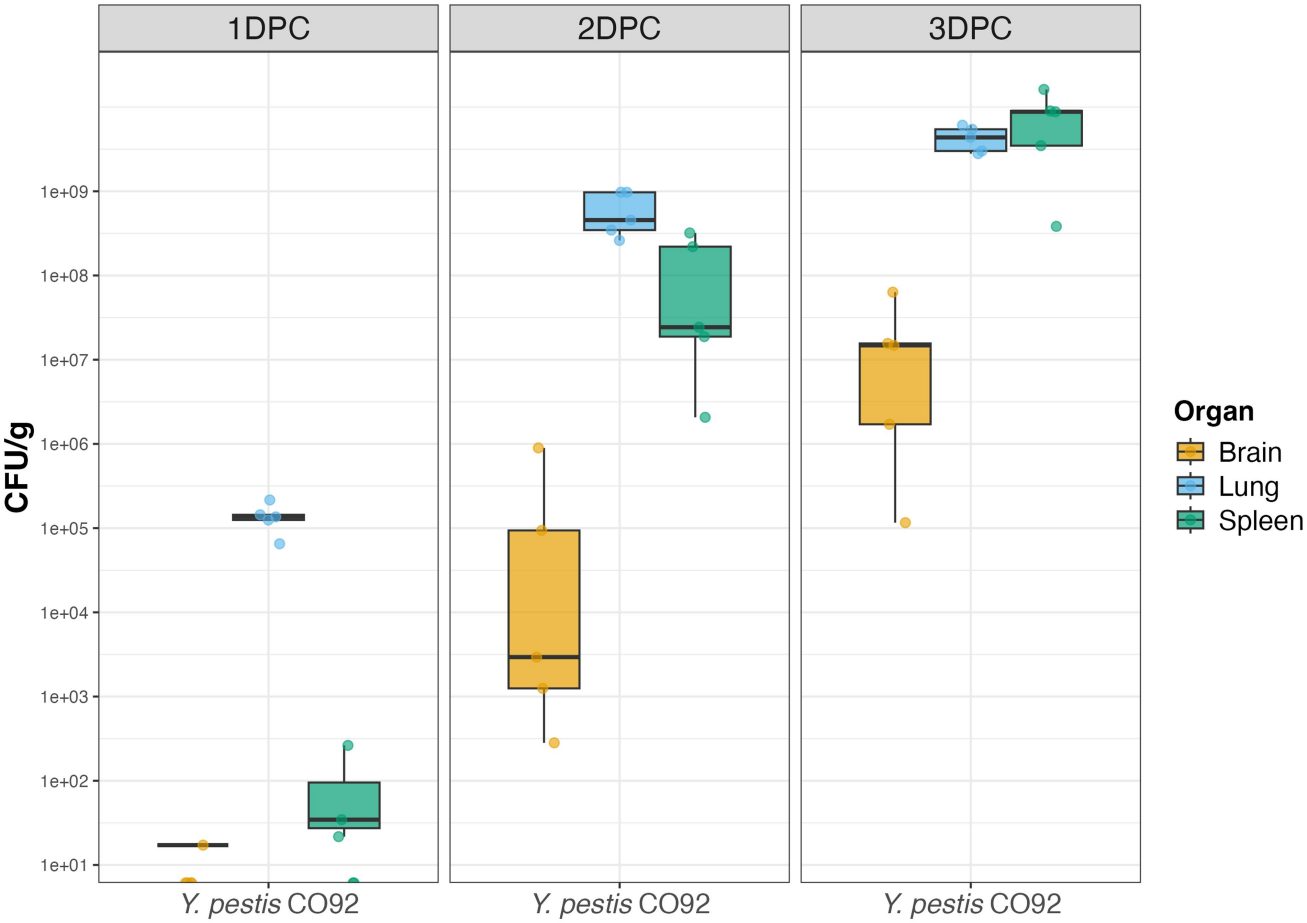
Mice infected with aerosolized *Y. pestis* develop a systemic infection including bacteria observed in brain homogenates. Colony counts from homogenates of brain, lung, and spleen tissues collected on days 1 (24 h), 2 (48 h), 3 (66 h) post aerosol challenge with *Y. pestis* CO92. Solid lines through boxes indicate the median values. Lower and upper hinges of box plots correspond to first and third quartiles. Upper and lower whiskers extend to the largest or smallest value no further than 1.5 * inter-quartile range, respectively. Data beyond the whiskers are outliers. Mice in this experiment had an average inhaled dose of 1.49 × 10^6^ CFU/mouse aerosolized *Y. pestis* CO92. Each individual data point is represented by a color-coded dot. *n* = 5 mice per time point, 1 of 5 mice was found dead 3DPC.

Although there are documented clinical cases of *Y. pestis* meningitis, to our knowledge there has been no attempted modeling or documenting of the pathological process of potentially neurological sequalae associated with plague in laboratory animals (Cooley et al., 2023). To this end, we quantified the bacterial burden in various tissue homogenates from BALB/c mice exposed to aerosolized *Y. pestis.* Lungs, spleens, and brains demonstrated detectable levels of bacterial burden in mice exposed to aerosolized *Y. pestis* at all three collection time points (Figure 1). As expected, the only tissue with substantial colony counts at 1 day post challenge (DPC) are the lungs since they are the main portal of entry for aerosolized bacteria, and the mice showed no clinical signs of disease at this early time point. However, at 2DPC *Y. pestis* infection appears to become systemic, and the bacterial burden further increases within all tissues at 3DPC (Figure 1). Blood from only one out of five mice showed bacteria at 1DPC, while acute bacteremia was observed by 2DPC with four out of five blood samples collected from mice on 2DPC, and all mice on 3DPC exceeding the limit of detection on the SBA plates (data not shown).

### 3.2 *Y. pestis* induces a pro-inflammatory brain cytokine profile in mice

**Figure 2:**
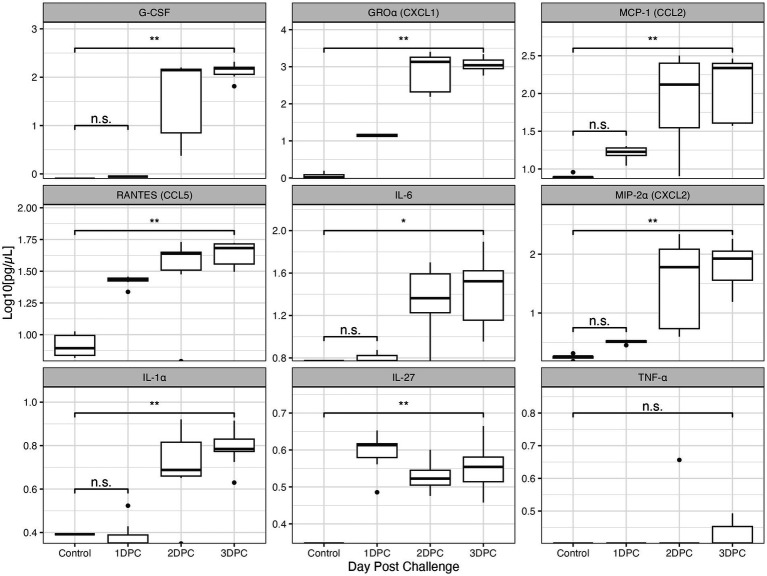
Selected cytokine expression in BALB/c mouse brain homogenates when exposed to aerosolized challenge with *Y. pestis*. Nine cytokines selected based on biological and statistical significance are shown out of 36 total tested cytokines. Solid lines through boxes indicate the median value. Lower and upper hinges of box plots correspond to first and third quartiles. Upper and lower whiskers extend to the largest or smallest value no further than 1.5 * inter-quartile range, respectively. Outliers are represented by a black dot. Pairwise treatment groups were compared by linear mixed effects model. No multiplicity adjustment was applied. N.S., not significant, *p < 0.05, **p < 0.001. Significance brackets are inclusive of days post challenge compared to control unless a sub bracket is below indicating a separation in significance values between specific days post challenge and control. Mice in this experiment had an average inhaled dose of 4.42 × 10^5^ CFU/mouse aerosolized *Y. pestis* CO92. *n* = 5 control, *n* = 7 1DPC, *n* = 7 2DPC, and *n* = 9 3DPC (approximately 60 h post-challenge).

To understand the effect of *Y. pestis* presence in the brain, the immunological response in brain homogenate was evaluated for levels of 36 cytokines using a multiplex Luminex kit. Six cytokines were upregulated at least two-fold by 1DPC and 36% (14/36) were upregulated at least two-fold by 2DPC; all cytokines remained at least as high at subsequent timepoints (Supplementary Figure S1 contains all cytokines evaluated and a subset of which are featured in Figure 2). At 2DPC, relative to the uninfected brain homogenates the levels of G-CSF and CXCL1 increased 2 log10 units (100-fold, *p* < 0.001) and 3 log10 units (1,000-fold, *p* < 0.001), respectively (Figure 2; Supplementary Table S2 details statistical analyses for all cytokines examined on each of the three collection time-points). These cytokines stimulated neutrophil production and migration into sites of inflammation, suggesting a strong neutrophil response at the expected proinflammatory phase of *Y. pestis* infection. Furthermore, CCL2, CCL5, IL-6, CXCL2, IL-1α and CXCL1 are associated with neuroinflammatory responses, and all increased significantly throughout disease progression with *Y. pestis* infection (Jiang et al., 2023; Bose and Cho, 2013; Chi et al., 2024). Of particular interest to this study is the significant increase of IL-6 at 2DPC, which is associated with an increased endothelial permeability at the blood–brain barrier (BBB) (Abbott, 2002). The expression of IL-27, a pleiotropic cytokine, increased 1 day post challenge. IL-27 is known to play an anti-inflammatory role but may also induce inflammatory cytokine production (e.g., TNF-*α* and IL-6) by microglia (Kawanokuchi et al., 2013). Although TNF-α protein levels did not show an increase, the transcriptomic data from brain homogenate indicated several upregulated genes within the tumor necrosis factor (TNF) pathway (Supplementary Figure S2) (Xu et al., 2024; Rodriguez et al., 2026). The immune response profile of mouse brains through 3 days of *Y. pestis* infection indicated pre- and pro-inflammatory responses in CNS immune associated cells and a robust neutrophil response after exposure to *Y. pestis*.

### 3.3 Neuroinflammatory transcriptional signatures in mice exposed to aerosolized *Y. pestis*

**Figure 3:**
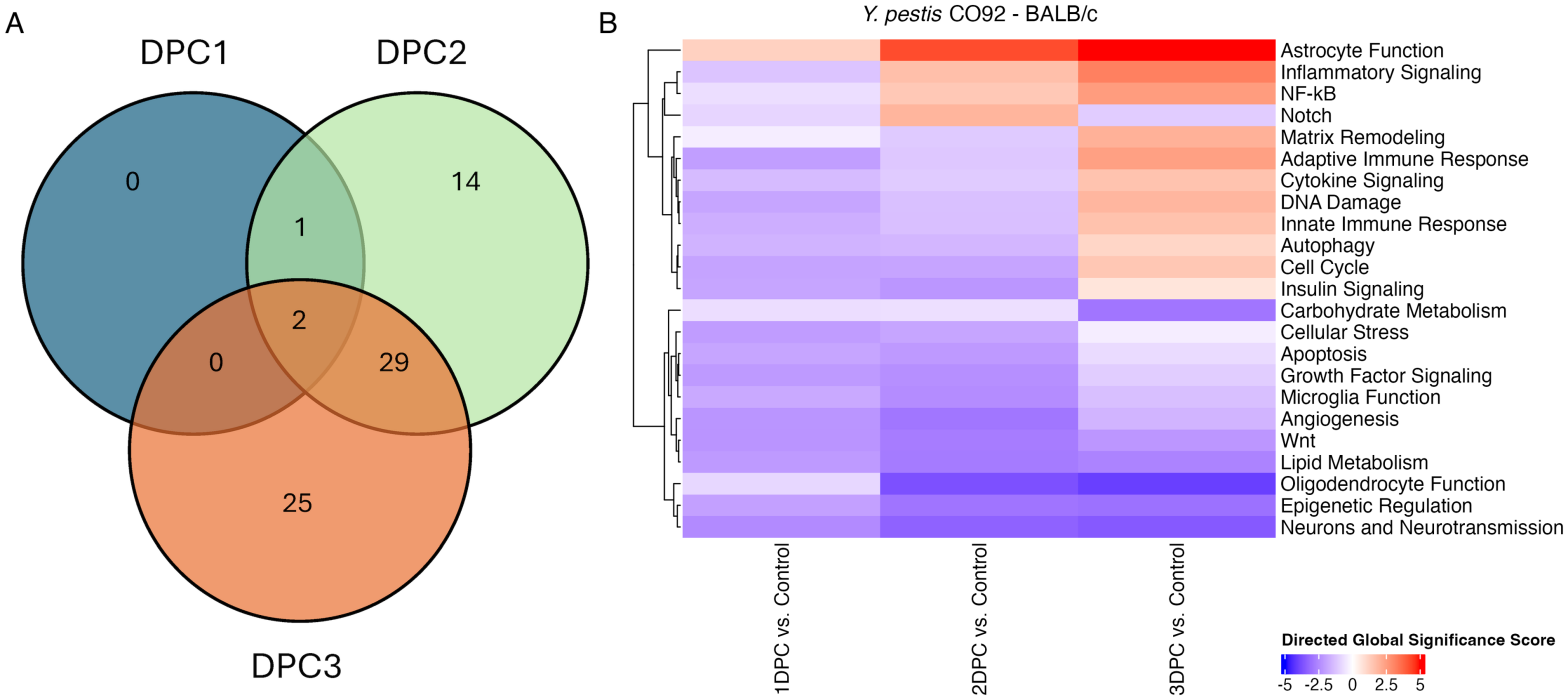
Neuroinflammatory-associated transcriptional signatures in brain homogenates are altered after 2DPC with aerosolized *Y. pestis* notably in astrocyte, oligodendrocyte, and microglia functions. **(A)** Venn diagram indicates genes that are unique or shared and are significantly expressed for each day post challenge with thresholds of ≥ | ± 1| LFC and *p*-adj < 0.05. **(B)** Directed global significance score heat map of genes grouped to functional gene sets. Blue indicates a downregulated gene set, red is upregulated. All genes are compared to naïve control mouse expression profiles. Mice in this experiment had an average inhaled dose of 1.49 × 10^6^ CFU/mouse aerosolized *Y. pestis* CO92. *n* = 5.

Next, to further understand the pre- and pro-inflammatory responses in CNS described above, a targeted transcriptomic approach was used to analyze differentially expressed genes (DEGs) associated with neuroinflammatory responses in mouse brain homogenates. Given our use of brain homogenates for this analysis, it is important to note that some transcriptional changes observed could be affected by various cells, blood, and tissues found in whole brains that may be generally inflammatory and not tied solely to neuroinflammation. Similar to the bacterial burdens mentioned above, there were minimal transcriptomic changes occurring at 1DPC with only three genes considered differentially expressed (≥| ± 1| LFC and *p*-adj < 0.05) in mice infected with *Y. pestis* when compared to unchallenged control mice (Figure 3A). However, as disease progressed during 2DPC and 3DPC, the number of significantly differentially expressed genes also increased to 46 and 56 genes, respectively with 31 of those genes shared between 2DPC and 3DPC (Figure 3A).

Gene Set Analysis was utilized to group genes to known functional sets, and a directed global significance score (DGSS) was used to determine the degree of regulatory changes in each gene set (Figure 3B). Overall, this analysis indicates high levels of neuroinflammatory-associated transcriptional alterations in mice after exposure to aerosolized *Y. pestis* compared to control mice. Furthermore, the inflammatory response seen here is similar to the pre- and pro-inflammatory phases of pneumonic plague discussed in previous literature (Pechous et al., 2016; Venugopal and Pechous, 2024; Price et al., 2012; Lathem et al., 2005). We observed that the transcriptional signatures of neuroinflammatory gene sets were mostly downregulated during the first 48 h barring astrocytes, inflammatory signaling, and the NF-κB pathway which were upregulated. By 3DPC 11 of 23 gene sets tested had a positive DGSS indicating upregulation (10 of the 11 gene sets DGSS ≥ 1), the most upregulated transcriptional signature being associated with astrocyte function (Figure 3B). Astrocytes, the most abundant cells found in the CNS, have extensive radiating processes that interact with both neurons and endothelial cells, playing a critical role in maintaining the blood brain barrier and responding to inflammation (Abbott, 2002). Conversely, the oligodendrocyte-associated transcriptional signature was highly downregulated (DGSS = −4.127). Oligodendrocytes are glial cells found in the CNS that form myelin sheaths that protect neurons from damage and degradation and properly insulating electrical signals in the nervous system (Michalski and Kothary, 2015). Taken together, these data suggest clear alteration of the transcription of genes involved with cells associated with or present in the CNS.

### 3.4 *Y. pestis* is confined to blood vessels in the brain in mice and non-human primates during acute pneumonic plague

**Figure 4:**
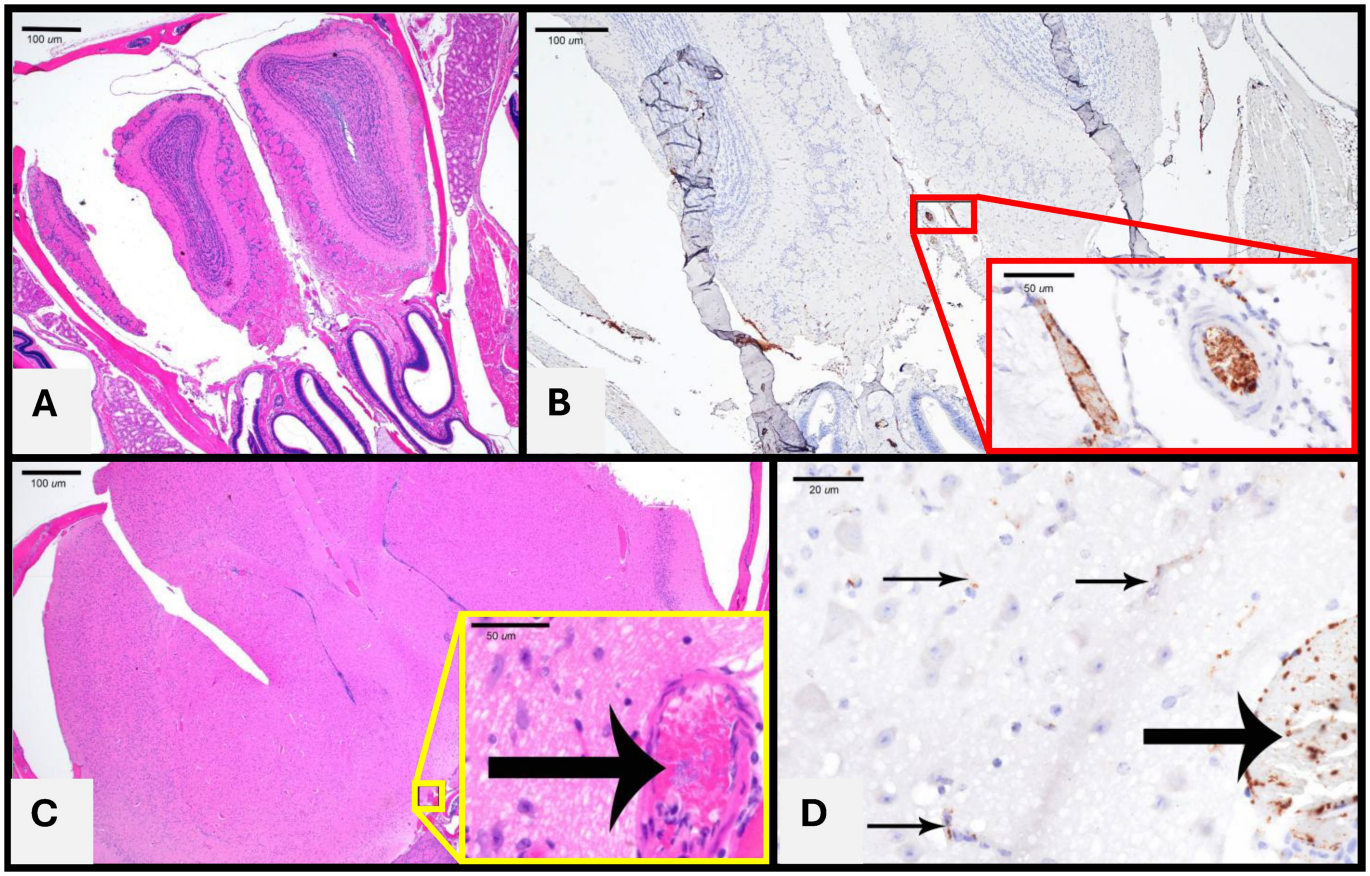
*Y. pestis* is contained within brain blood vessels and capillaries in mice after exposure (approximately 66 h) to aerosolized bacteria. **(A)** Nasal turbinates and the olfactory bulb appear normal at low magnification. Coronal section, HE 2X. **(B)** There is intravascular *Y. pestis* antigen IHC immunopositivity in meningeal blood vessels. Inset: *Y. pestis* antigen immunopositivity within the boundaries of the blood vessel. Coronal section, *Y. pestis* antigen IHC 2X and 40X. **(C)** Cerebrum appears normal but with noticeable congestion. Inset: There are intravascular *Y. pestis* (arrow) within the meningeal blood vessel. Coronal section, HE 2X and 60X. **(D)** Cerebrum. There is *Y. pestis* antigen immunopositivity in meningeal and cerebral blood vessels (arrows). *Y. pestis* antigen coronal section, IHC 20X. All images were collected from Mouse 10 in Supplementary Table S3.

To further characterize the inflammatory regulatory changes associated with the CNS described above, we performed histopathological and immunohistochemical analysis of brain tissues from *Y. pestis* challenged mice. By 3DPC (approximately 66 h), several mice exhibited histopathologic changes consistent with pneumonic plague, including necrosis, inflammation, fibrin, and hemorrhage in the lungs, lymph nodes, and spleen (data not shown). The mice used for histopathological analyses (Figure 4) were either euthanized at described collection points or did not survive disease as shown in Supplementary Table S3 which provides a detailed description of mouse samples and histological analyses based upon severity scores determined during histological analyses. Control mice in these uniformly lethal infection models typically succumb to disease or meet early endpoint euthanasia criteria by 3DPC or 4DPC after exposure to aerosolized *Y. pestis* CO92.

The progression of the infection was traced using immunohistochemistry (IHC) to detect *Y. pestis* antigen. *Y. pestis* antigen IHC positivity was first detected in the lungs and spleen as early as 1DPC, with the IHC signal intensifying over the course of the infection. By 2DPC, *Y. pestis* antigen IHC positivity was also present in the nasal turbinates and lymph nodes. The most significant finding occurred at 3DPC: one euthanized mouse showed low levels of *Y. pestis* antigen IHC positivity within the blood vessels of the meninges (Figure 4B). In contrast, mice that had terminal disease displayed strong (marked to severe) IHC positivity for *Y. pestis*, which was contained within the blood vessels of the meninges, cerebrum, cerebellum, nasal turbinates, lymph nodes, and cranial bone marrow (Supplementary Table S3 showing representative examples of additional mouse histopathology).

**Figure 5:**
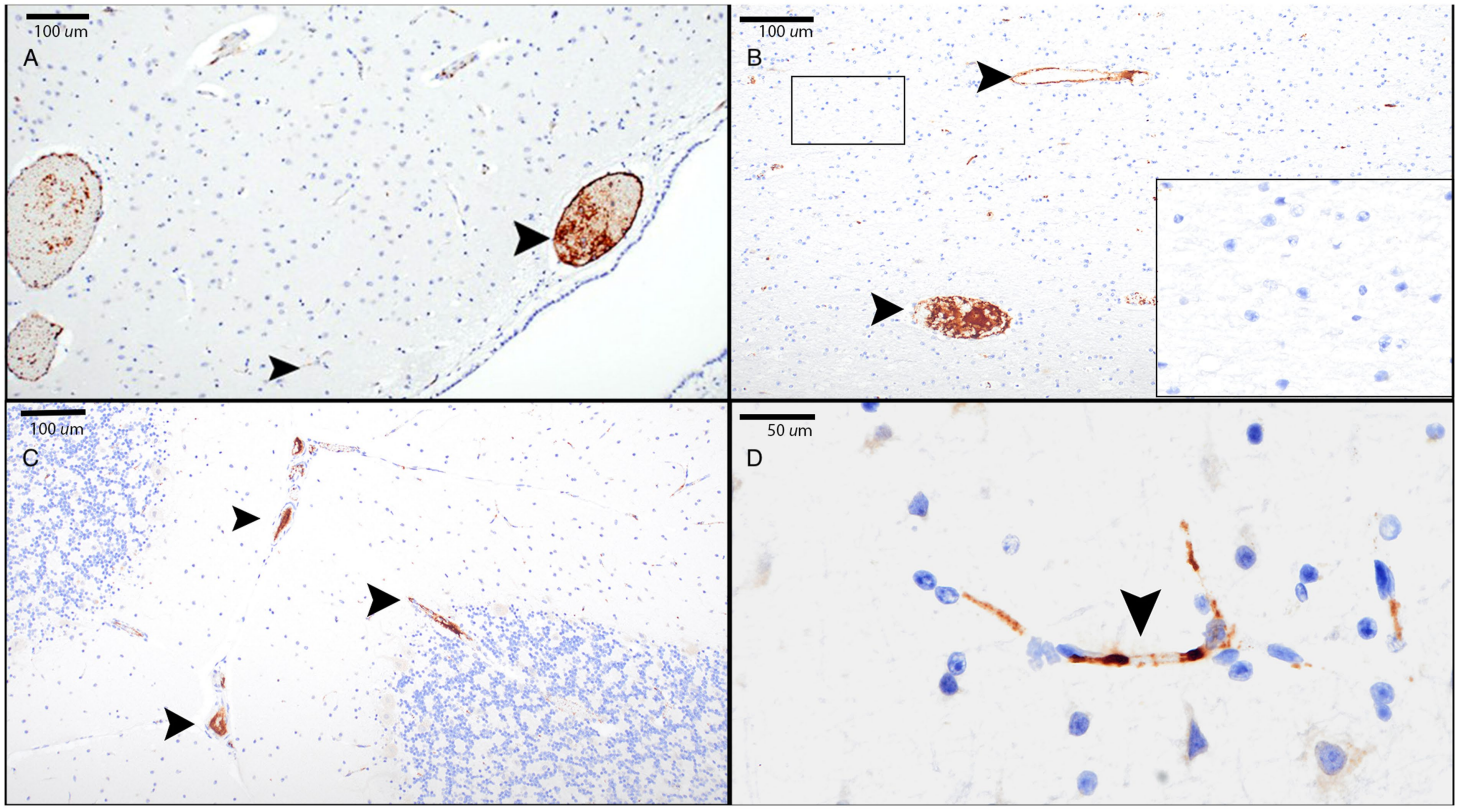
*Y. pestis* is contained within brain blood vessels and capillaries in NHPs after exposure to aerosolized bacteria. IHC staining in African green monkeys following aerosol challenge *Y. pestis* CO92. **(A)** Female NHP non-survivor on 5DPC and had significant histopathological changes. Cerebrum, corpus striatum. There is *Y. pestis* IHC immunopositivity in blood vessels of the cerebrum (arrowheads). *Y. pestis* IHC 4X. **(B)** Male NHP non-survivor on 3DPC and had significant histopathological changes. Cerebrum, frontal cortex. There is *Y. pestis* IHC immunopositivity in blood vessels of the frontal cortex (arrowheads). Inset: The neuropil of the frontal cortex appears normal and no immunopositivity is present. *Y. pestis* IHC 10X and 40X. **(C)** Same male NHP, Cerebellum. There is *Y. pestis* IHC immunopositivity in blood vessels of the cerebellum (arrowheads). *Y. pestis* IHC 4X. **(D)** Same male NHP, Cerebrum, frontal cortex. There is *Y. pestis* IHC immunopositivity in capillaries of the frontal cortex (arrowhead). *Y. pestis* IHC 60X. These tissues were obtained via a tissue sharing effort in order to leverage existing animal specimens to avoid unnecessary duplication.

In a “tissue sharing” effort, we also examined the brains of non-survivor NHPs on 3DPC or 5DPC after exposure to aerosolized *Y. pestis* CO92. *Y. pestis* was recovered from brain homogenates from all NHPs at this time post-infection (data not shown). Mirroring the results obtained in the mouse model of pneumonic plague, there was no evidence of infection in the parenchymal brain tissue, but rather the *Y. pestis* was confined to the blood vessels and capillaries in the cerebrum and cerebellum (Figure 5).

### 3.5 Comparison of neurological impacts as observed from data collected from brain homogenates collected from mice exposed to aerosolized *Y. pestis* or *B. pseudomallei*

**Figure 6:**
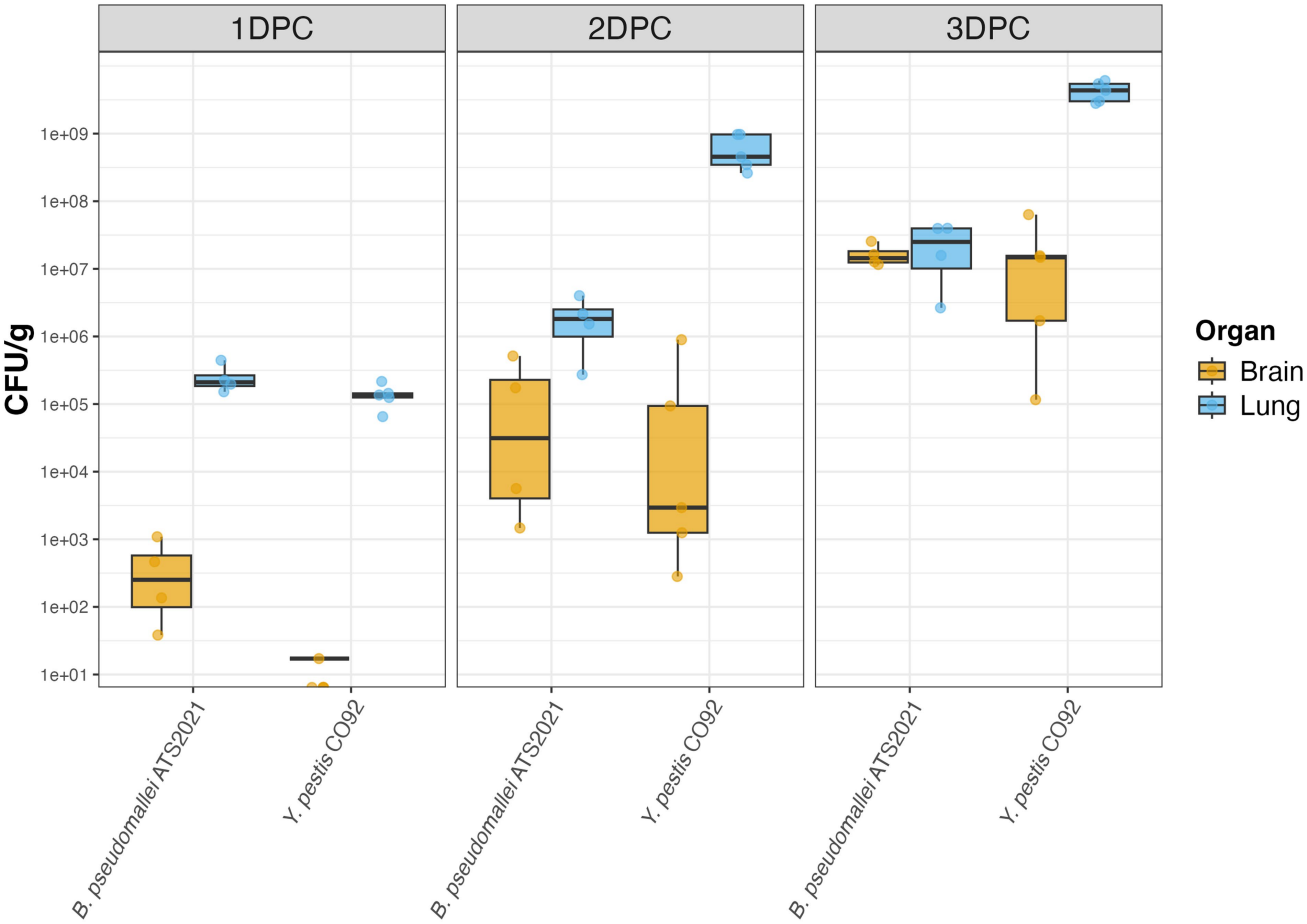
Similar levels of infection in the brain and lung homogenates in mice infected with *Y. pestis* or *B. pseudomallei*. Colony counts of brain and lung homogenates on days 1, 2, 3 post aerosol challenge (#DPC) with *Y. pestis* CO92 or *B. pseudomallei* ATS2021. Solid lines through boxes indicate the median value. Lower and upper hinges of box plots correspond to first and third quartiles. Upper and lower whiskers extend to the largest or smallest value, no further than 1.5 * inter-quartile range, respectively. Data beyond the whiskers are outliers. Mice in this experiment had an average inhaled dose of 1.49 × 10^6^ CFU/mouse aerosolized *Y. pestis* CO92 or average inhaled dose of 1.15 × 10^3^ CFU/mouse of aerosolized *B. pseudomallei* ATS2021. Each individual data point is represented by a color-coded dot. For *Y. pestis* data *n* = 5 mice per time point, 1 of 5 mice was found dead 3 DPC (approximately 66 h) and for *B. pseudomallei* data *n* = 4 mice per time point.

To better understand the neuroinflammatory response to bacterial disease, we compared mice exposed to aerosolized *Y. pestis* to mice exposed to *B. pseudomallei*, a pathogen with documented neuroinvasive potential causing severe brain damage (Cote et al., 2024; St John et al., 2014, 2016). Importantly in this study, the BALB/c mouse strain was used for pneumonic plague, and the C57BL/6 strain was used for inhalational melioidosis. Thus, there were inherent differences between the immune responses in these two animal models. Nevertheless, this comparison allowed us to begin to characterize the immune response from two distinct bacterial diseases, each with differing levels of neurological involvement. We first compared bacterial burden in mouse lungs and brains on days one, two, and three after infection with either *Y. pestis* CO92 or *B. pseudomallei* ATS2021 (Figure 6). Although the total aerosolized bacteria challenge dose of *Y. pestis* was three times higher than that of *B. pseudomallei*, the calculated number of median lethal doses were comparable and averaged 22 LD_50_s and 21 LD_50_s, respectively, and the lung bacterial burden was similar between the two models at 1DPC. However, at 2 and 3 DPC, the burden was markedly higher in the lungs of mice infected with *Y. pestis* (Figure 6). In contrast, the bacterial burden in brain homogenates was relatively similar in both models at 2 and 3DPC. Despite the similar bacterial burden in the brain, histopathological and IHC analyses revealed a stark contrast between the two disease models from data generated here and in a previously published study with *B. pseudomallei* (Cote et al., 2024). *Y. pestis* appears restricted to the blood vessels of the brain and the meninges (Figures 4, 5). In contrast, *B. pseudomallei* can readily spread cell-to-cell via actin-polymerization and can bypass the BBB and gain direct access to the brain parenchyma through the cribriform plate via olfactory nerves or through respiratory epithelium via trigeminal nerves causing necrotizing meningoencephalitis (Cote et al., 2024; St John et al., 2014, 2016; Jitprasutwit et al., 2023).

**Figure 7:**
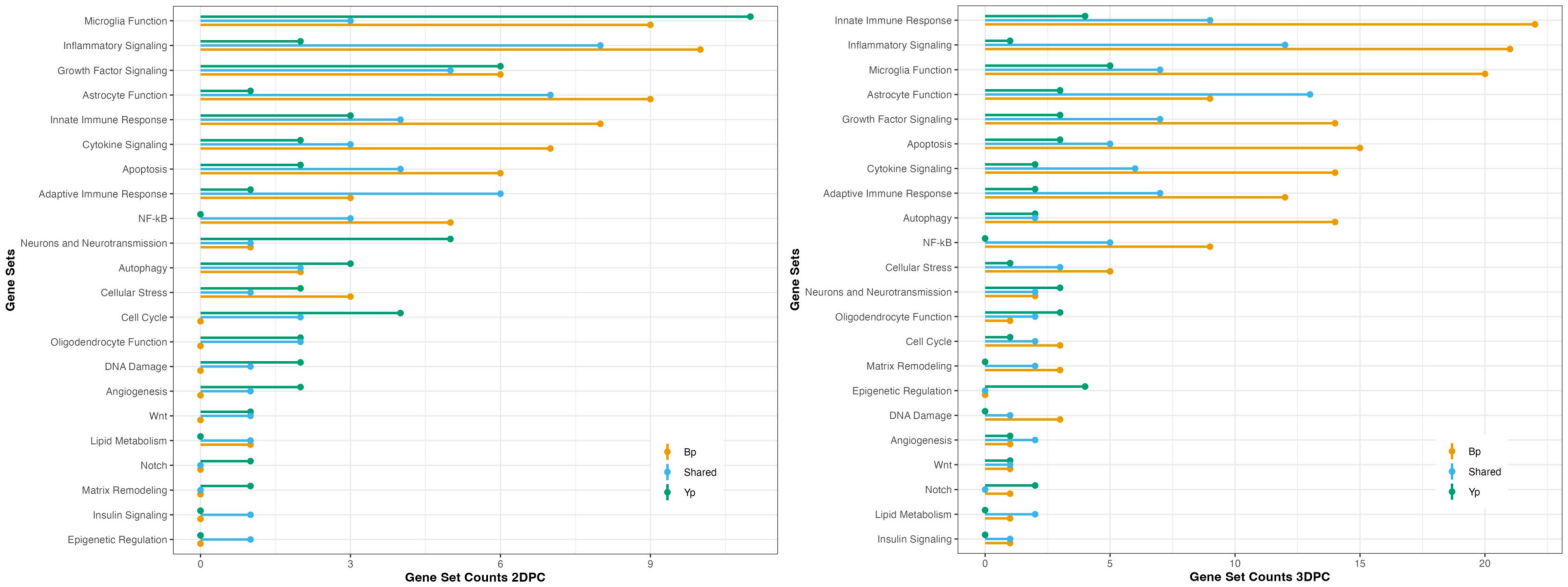
Most gene sets share similar gene counts between brain homogenates from mice infected with either *Y. pestis* or *B. pseudomallei* at two and 3 days post challenge. Each line indicates the number of genes that passed a threshold of ≥| ± 1| LFC and *p*-adj < 0.05 for a specific gene set. Orange lines are genes unique to mice infected with *B. pseudomallei*, green lines are genes unique to mice infected with *Y. pestis*, and blue lines are genes shared by both mouse models. Each plot represents significant gene counts expressed for either day two or three post challenge. Note: The *x*-axes scales are different for 2DPC and 3DPC due to some gene sets increasing in counts at 3DPC. Mice in this experiment had an average inhaled dose of 1.49 × 10^6^ CFU/mouse aerosolized *Y. pestis* CO92 or average inhaled dose of 1.15 × 10^3^ CFU/mouse of aerosolized *B. pseudomallei* ATS2021. For *Y. pestis* data *n* = 5 mice per time point and for *B. pseudomallei* data *n* = 4 mice per time point.

We also compared the neuroinflammatory transcriptomic responses between mice infected with each bacterium. Using the same approach described above to analyze transcriptomic data, we found that overall neuroinflammatory gene sets share similar quantities in mice infected with *Y. pestis* or *B. pseudomallei* at 2DPC and 3DPC (Figure 7). At 2DPC *Y. pestis* infected mice had higher gene set counts in several groups including microglia function and neurons and neurotransmission, while *B. pseudomallei* infected mice had higher gene counts in cytokine signaling, various cellular stress responses, and transcriptional signatured associated with astrocyte function. This bias shifts dramatically at 3DPC with higher gene set counts in nearly all gene sets in *B. pseudomallei* infected mice but an overall lower unique gene set count for *Y. pestis* infected mice. The lower unique gene set count in mice infected with *Y. pestis* at 3DPC is due to 69% of DEGs being shared with *B. pseudomallei* infected mice.

**Figure 8:**
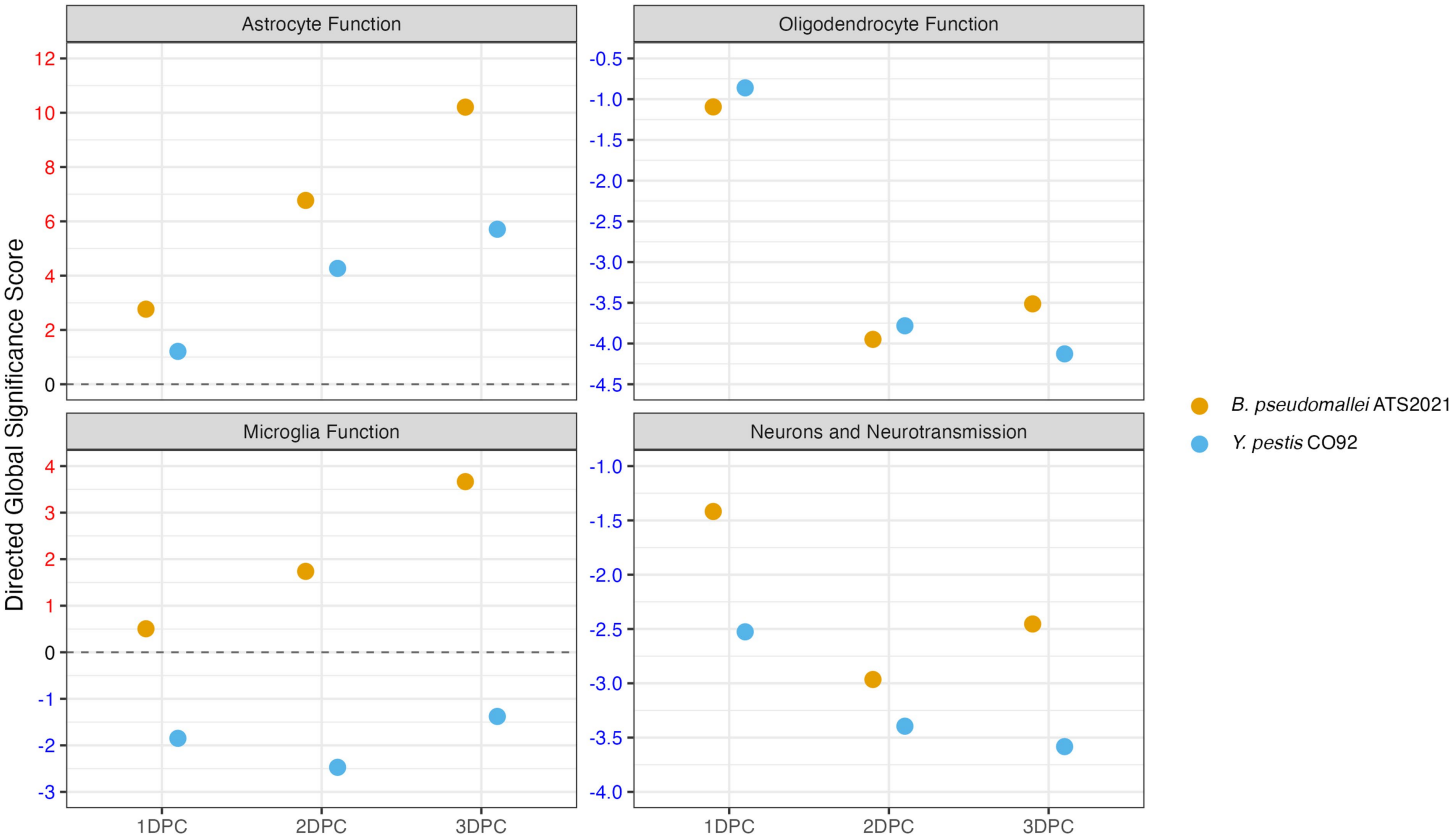
Comparison of glial and neuronal specific gene set directed global significance scores (DGSS) for brain homogenates from mice infected with *Y. pestis* and *B. pseudomallei*. Pathway scores above 0 indicate upregulation of a gene set and scores below 0 indicate downregulation. Blue and orange dots are expression data from mice aerosolized with *Y. pestis* and *B. pseudomallei, respectively.* Y-axes are unique to each gene set group. If present, dashed lines indicate 0 DGSS. Y-axis values are color coded red or blue to indicate values above or below 0, respectively. All genes are compared to control mice expression profiles. Mice in this experiment had an average inhaled dose of 1.49 × 10^6^ CFU/mouse aerosolized *Y. pestis* CO92 or average inhaled dose of 1.15 × 10^3^ CFU/mouse of aerosolized *B. pseudomallei* ATS2021. For *Y. pestis* data *n* = 5 mice per time point and for *B. pseudomallei* data *n* = 4 mice per time point.

**Figure 9:**
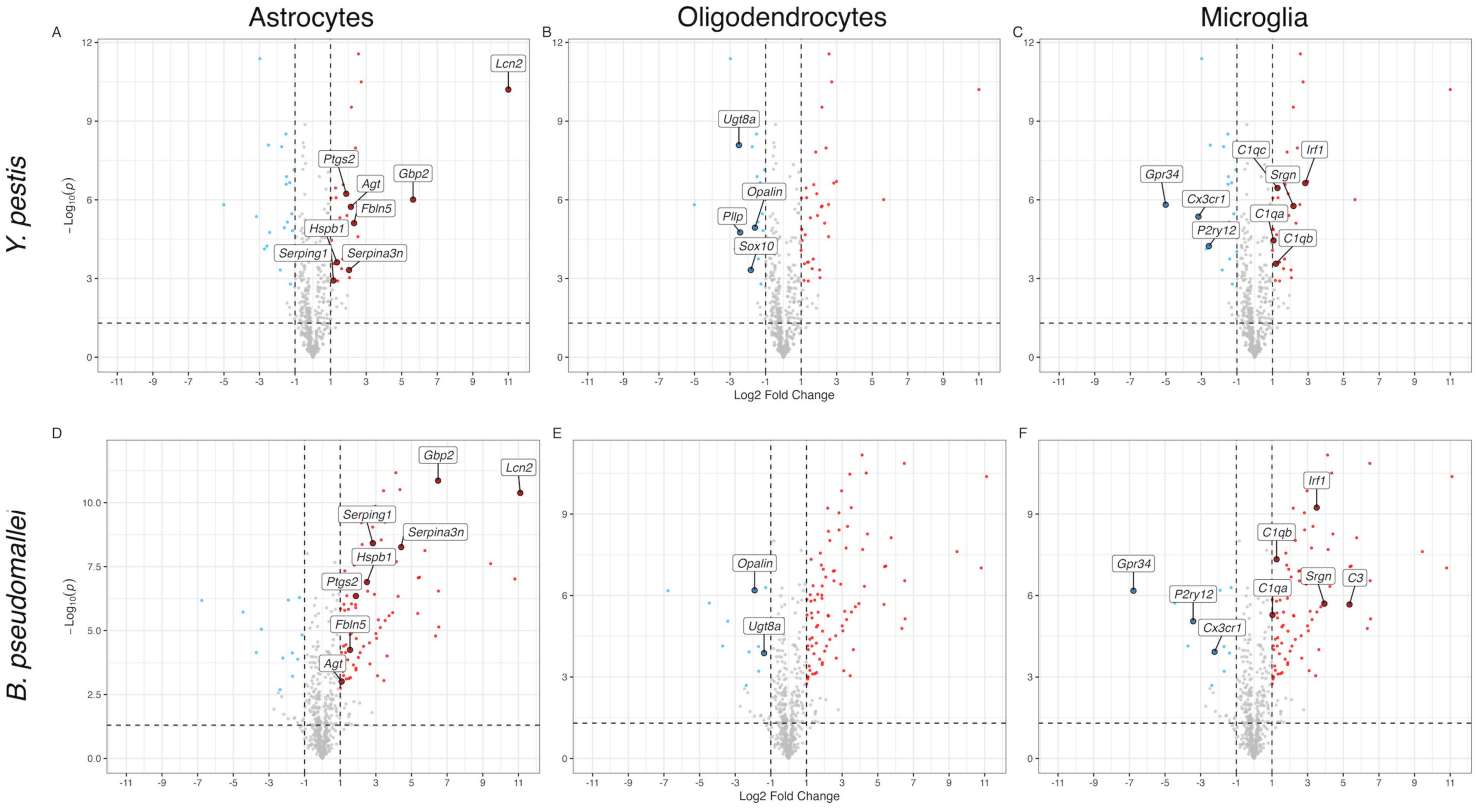
Volcano plots of differentially expressed genes in brain homogenates from mice exposed to either *Y. pestis* or *B. pseudomallei* at 3 DPC with selected genes in astrocyte, microglia, and oligodendrocyte-associated gene sets. **(A)** Astrocyte-associated transcriptional signatures in mice aerosolized with *Y. pestis*. **(B)** Oligodendrocyte-associated transcriptional signatures in mice aerosolized with *Y. pestis*. **(C)** Microglia-associated transcriptional signatures in mice exposed to aerosolized *Y. pestis*. **(D)** Astrocyte-associated transcriptional signatures in mice aerosolized with *B. pseudomallei*. **(E)** Oligodendrocyte-associated transcriptional signatures in mice exposed to aerosolized *B. pseudomallei*. **(F)** Microglia-associated transcriptional signatures in mice aerosolized with *B. pseudomallei*. Genes labeled were based on expression magnitude, statistical significance, and biological relevance. Vertical dashed lines indicate ±1 LFC, the horizontal dashed line indicates *p* < 0.05 and dots colored blue (downregulated) or red (upregulated) passed both ≥| ± 1| LFC and a *p*-adj < 0.05, thresholds. Mice in this experiment had an average inhaled dose of 1.49 × 10^6^ CFU/mouse aerosolized *Y. pestis* CO92 or average inhaled dose of 1.15 × 10^3^ CFU/mouse of aerosolized *B. pseudomallei* ATS2021. For *Y. pestis* data *n* = 5 mice per time point and for *B. pseudomallei* data *n* = 4 mice per time point.

The directed global significance score statistic was used to measure which gene sets were up or down-regulated relative to the naïve mice. Overall, the gene expression profiles were quite different (Supplementary Figures S4–S7 provide detailed transcription expression comparisons between mice with pneumonic plague or inhalational melioidosis). Neuroinflammatory transcriptional signatures in mice infected with *B. pseudomallei* were heavily upregulated, in contrast to the pre- and pro-inflammatory response in *Y. pestis* infected mice mentioned previously (Figure 8; Supplementary Figure S4). Specifically, the astrocyte-associated transcriptional signature was highly upregulated in both models as soon as 2DPC (Figure 8). Genes encoding Lipocalin 2 (*Lcn2*), angiotensinogen (*Agt*), and fibulin-5 (*Fbln5*) were highly upregulated associated with astrocyte function in both disease models and were involved with BBB modulation, anti-inflammatory and cardiovascular stress responses (Figures 9A,D; Supplementary Figure S5). In addition, infection with either bacterium resulted in downregulation of genes involved in oligodendrocyte and neuron function (Figure 8). While *Ugt8a* (UDP galatosyltransferase 8A), *Opalin* (oligodendrocytic myelin paranodal and inner loop protein)*, Sox10* (SRY-box transcription factor 10), and *Pllp* (plasmolipin), are genes that were highly downregulated in *Y. pestis* infected mice, *Ugt8a* and *Opalin* were also downregulated in *B. pseudomallei* infected mice indicating downregulation of genes tied to myelin synthesis and maintenance in both models (Figures 9B,E; Supplementary Figure S7). Gene counts involved in microglia function were higher in *Y. pestis* infection at 2DPC. However, at 3DPC mice aerosolized with *B. pseudomallei* had far higher gene counts (Figure 7). Interestingly, microglia function was overall downregulated throughout each timepoint in the plague model and upregulated in the melioidosis model (Figure 8). Despite microglia related gene sets not sharing the same overall gene expression pattern between both models, several genes associated with phagocytosis and complement mediated synapse degradation displayed a similar altered transcription pattern at 2DPC and 3DPC (Figures 9C,F; Supplementary Figure S7). Neuron-function and neurotransmission was another gene set directly tied to the nervous system that was downregulated in both models (Figure 8). In this gene set *Arc* (encoding activity-regulated cytoskeleton-associated protein), a gene important for synaptic strength, was downregulated in mice exposed to *Y. pestis,* but did not meet cutoff thresholds in mice exposed to *B. pseudomallei* (Data not shown). These data demonstrate that while neuroinflammatory gene set counts were overall similar between the two infection models, the direction of differential expression was quite different overall. For example, the magnitude of upregulation in genes associated with astrocyte function in mice aerosolized with *B. pseudomallei* was nearly twice that of mice aerosolized with *Y. pestis* (Figure 8). However, in both models, there are similarities in individual genes tied to neuroinflammatory cells contained within the CNS.

## 4 Discussion

In this report, we characterize the impact of exposure to aerosolized *Y. pestis* on the brains of mice. Understanding the inflammatory response and disease progression will help to refine disease models, inform how plague meningitis manifests, and advance the development of medical countermeasures. Furthermore, by comparing the pneumonic plague model with our previously described inhalational neuroinvasive melioidosis model, we demonstrated similarities and differences that spanned two different mouse strains with different immune profiles. BALB/c mice exhibit a Th2-skewed immune response and are used as an acute pneumonic plague model for pathogenesis, vaccines, and therapeutic studies. BALB/c mice are also routinely used to test for therapeutic efficacy as an inhalational melioidosis model (Nelson et al., 2023; Biryukov et al., 2025; Biryukov et al., 2023; Velappan et al., 2024). C57BL/6 mice have more pronounced Th1 cell-mediated immune responses and are used for vaccine development and less-acute disease models for inhalational melioidosis (Cote et al., 2024; Gessner et al., 1993; Locksley et al., 1987; Limmathurotsakul et al., 2015). It is also critical to note that due to extreme sensitivity to inhalational melioidosis in BALB/c mice, it is not necessarily a feasible mouse strain for direct comparison (Cote et al., 2024). Although we did not notice any differentially expressed genes between naïve control mice of either mouse strain, in future studies we could infect C57BL/6 mice with aerosolized *Y. pestis* for a more direct comparison to the inhalational melioidosis model.

There is increasing evidence that bacteria or bacterial components (e.g., lipopolysaccharide) found in humans or animals can result in neurological sequalae. This association of neurodegeneration with either commensal bacteria or pathogenic bacteria has been demonstrated using animal models (e.g., sepsis models) and human post-mortem samples (Tran et al., 2022; Singer et al., 2018; Singer et al., 2016; Denstaedt et al., 2018; Denstaedt et al., 2020). To date there is no readily accessible information pertaining to neuroinflammatory response in mice infected with aerosolized *Y. pestis*. Plague meningitis has been documented in several cases in the literature; however, it remains a relatively rare manifestation of the disease (Cooley et al., 2023). In our mouse model, we observed high bacterial burdens within brain homogenates as early as 2DPC. However, histopathological analysis did not confirm the presence of bacterial meningitis. Instead, at 3DPC, *Y. pestis* bacteria were confined to the blood vessels within the brain and meninges. It is possible that subtle damage to the vasculature occurred but was not detectable using standard light microscopy. More sensitive techniques, such as electron or immunofluorescence microscopy may be required to identify these potential vasculature changes. However, these data clearly support the concept of systemic *Y. pestis* infection with cerebral vascular involvement. The histopathological data presented here, along with findings from other studies, support that despite considerable anatomical differences between mice and humans, not all aerosolized bacteria gain access to the brain through the same mechanisms in mice (Stucki et al., 2024; Harkema et al., 2013). While case reports have documented melioidosis-associated meningitis (Chlebicki et al., 2008; Chatterjee et al., 2021; Prasad et al., 2017), *B. pseudomallei* primarily gains direct access to the CNS by invading the olfactory or trigeminal nerves (Cote et al., 2024; St John et al., 2014; St John et al., 2016). *B. pseudomallei* infected mice will demonstrate neurological manifestations of the infection (i.e., uncoordinated movements, sensitivity to touch, etc.) (Cote et al., 2024, 2026). In contrast, even large numbers of aerosolized *Y. pestis* bacteria remain confined within the blood vessels of the brain and we have not identified any outward clinical signs of neurological manifestations in mice or NHPs associated with pneumonic plague. Importantly, we have also observed that vascular containment of the *Y. pestis* bacteria within the brain’s blood vessels was also observed in the brains of NHPs after exposure to aerosolized *Y. pestis* (Figure 5). Despite the confirmed retention of *Y. pestis* in the blood vessels of the cerebrum, cerebellum, and the meninges, our cytokine and transcriptomic data revealed a significant increase in the pro-inflammatory cytokine response within the brain homogenates, highlighting the potential for indirect effects of the systemic infection on CNS function via cerebral vascular involvement. In future work, we could infect mice at a much lower dose or possibly provide suboptimal antibiotic regimens in order to slow disease progression in hopes of defining a plague meningitis model.

As mentioned previously it is important to note that all transcriptomic data were taken from non-perfused brain homogenates which indeed is a limitation. However, our transcriptomic results discussed herein provide a framework to begin modeling the potential signal for neurological impacts of plague and better inform the execution of more precise experiments such as spatial transcriptomics. Differential gene expression data demonstrate an overall response in neuroinflammatory associated genes, with transcripts related to astrocyte function being the most upregulated and shared gene set in both *Y. pestis* and *B. pseudomallei* infected mice. However, the magnitude at which upregulation of the astrocyte related genes occurs in melioidosis model is twice that of the pneumonic plague model at 3DPC. Notwithstanding the differences in mouse strains used in the two disease models, these data suggest a more fulminant neuroinflammatory state which has been supported by histopathology. Astrocytes are glial cells and are the predominant cell type in the CNS providing metabolic, structural, homeostatic, inflammatory, and neuroprotective roles including regulation of the BBB by maintaining tight junctions and modulating transport and metabolic barriers. High upregulation of transcripts associated with astrocyte function suggests that there is a substantial neuroinflammatory response in plague infected mice as early as 2DPC. Furthermore, some of the most upregulated transcriptional signatures are associated with astrocyte function like *Lcn2*, *Agt*, and *Fbln5* following *Y. pestis* infection. LCN2 is an iron chelating protein that has diverse functionality in mammals. Specifically, LCN2 is a well-known mediator of a neuroinflammation that increases through activation of the NF-κB pathway and is induced in response to CNS injury and neurodegeneration (Bi et al., 2013; Fujino et al., 2006; Jung and Ryu, 2023; Zhao and Stephens, 2013; Wang et al., 2024). *Agt* is expressed in astrocytes and some neurons in areas associated with the vasculature of the brain and overexpression results in hypertension (Lavoie et al., 2004; Morimoto et al., 2001; Nakagawa and Sigmund, 2017; Sherrod et al., 2005; Stornetta et al., 1988; Yang et al., 1999). Previous reports indicate that AGT regulates the BBB by increasing tight junctions in endothelial cells via its cleavage into angiotensin II such that *Agt* knockout mice demonstrated reduced formation of tight junctions in brain endothelial cells (Lv et al., 2025; Wosik et al., 2007). Additionally, *Y. pestis* is known to cause hypoxia and ischemia and FBLN5 is an extracellular matrix protein that is upregulated during hypoxia and is associated with astrocytes (Guadall et al., 2011; Guarner et al., 2005; Riedel, 2005; Saglam et al., 2021). Additional astrocyte-related transcripts shared between both pneumonic plague and inhalational melioidosis models indicate several upregulated genes (*Ptgs2, Gbp2, Hspb1, Serpina3n,* and *Serping1*) tied to reactive astrocytes and modulation of the BBB in response to neuroinflammation (Zamanian et al., 2012; Yang et al., 2024; Shiow et al., 2017; Taylor et al., 2022; Kim et al., 2022).

Although some genes are shared in oligodendrocyte function, neurons, and neurotransmission in both disease models, mice infected with *Y. pestis* have a more pronounced regulatory shift of these gene sets. Oligodendrocytes are glial cells whose primary role is the formation and maintenance of a protective and insulating layer of myelin sheaths along axons (Michalski and Kothary, 2015). Oligodendrocyte death in the CNS is most commonly due to trauma and ischemia followed by demyelination (Traka et al., 2016; Fancy et al., 2011; Kuhn et al., 2019). Demyelination is the process where myelin sheaths are broken down and can be repaired through remyelination but can also lead to neuron degradation (Kuhn et al., 2019; Franklin and ffrench-Constant, 2008). In our pneumonic plague model oligodendrocyte function is the most downregulated gene set, that encompasses *Opalin*, *Sox10*, and *Ugt8a* genes which play a role in myelin development. Knockouts in *Opalin* have been shown to downregulate *Sox10* resulting in hypomyelination (Teng et al., 2024). *Sox10* encodes a transcription factor and mutations within *Sox10* are associated with Shah-Waardenburg syndrome, a rare disease involving central dysmyelinating leukodystrophy (Anderson et al., 2015; Verheij et al., 2006; Sánchez-Mejías et al., 2010; Bhattarai et al., 2022). *Ugt8a* encodes a key enzyme for the biosynthesis of critical glycosphingolipids necessary for myelin development and is a direct target of *Sox10* (Bosio et al., 1996; Cao et al., 2018). Additionally, Pllp is found in oligodendrocytes and is important for myelin development. Downregulation of *Pllp* is associated with demyelinating disorders and myelin defects (Shulgin et al., 2021). Lastly, Arc is a retrotransposon Gag protein that is important for synaptic strength. Knockdown of *Arc* promotes glial cell activation, inflammatory cytokine expression, and aggravates brain damage in rats with subarachnoid hemorrhage (Chen et al., 2023). *Arc* and *Pllp* are highly downregulated genes in our plague model, but neither meet differentially expressed cutoffs in our analysis for mice infected with *B. pseudomallei* making them potential markers for neuroinflammation in plague cases or this might be mouse strain dependent. Furthermore, the downregulation of these transcriptional signatures in the pneumonic plague model indicates a potential issue with myelin development and maintenance that requires further investigation.

Microglia patrol and phagocytize cellular debris and pathogens in infected hosts (Gao et al., 2023; Lannes et al., 2017). Mice infected with *B. pseudomallei* show both an increased total count and overall upregulation of genes associated with microglia function, while these genes are overall downregulated in the pneumonic plague mice. The most significant and differentially expressed genes are shared in both models specifically *Gpr34, P2ry12, Cx3cr1* are downregulated while *Irf1 and Srgn* are upregulated. These genes all regulate microglia response to neuroinflammation, migration, and phagocytosis (Figure 8). Given the bidirectional expression of these genes and previous reports, the data contradictorily suggest simultaneous activation and impairment of microglia migration and uptake as well as an indication of pathological neuroinflammation (Yang et al., 2022; Izume et al., 2024; Qian et al., 2024; Gómez Morillas et al., 2021; Puntambekar et al., 2022). This may be due to the heterogeneity of the tissue homogenates with different cell subsets attempting to mitigate and resolve the pathology in different ways.

The complement component 1q (C1q) subunit genes (i.e., *C1qA*, *C1qB*, and *C1qC*) are traditionally associated with innate immunity, but our analysis also closely associates them with microglia (Figure 8). C1q initiates the classical complement pathway leading to various innate immune responses (Kouser et al., 2015; Thielens et al., 2017). *C1q* genes are upregulated in mice infected with aerosolized *Y. pestis* and higher levels of *C1q* were previously shown to be required for microglia-mediated synaptic pruning (phagocytosis of synapses) in response to neuroinvasive disease (Li and Barres, 2018). Furthermore, previous research demonstrated that microglia are activated in response to injury or lipopolysaccharides and increase C1q, IL-1α, and TNF inducing A1 reactive astrocyte contributing to the death of neurons and oligodendrocytes (Liddelow et al., 2017). In our study, we observe upregulation of *C1q* and TNF pathway associated genes, as well as increased IL-1α cytokine concentrations, which are of interest due to the role they play in A1 astrocytes. Ideally, validation through protein analyses would have been completed on glia associated genes with significant and biologically relevant changes. However, this was not feasible within the experimental constraints and we recognize the lack of direct evidence of protein function as a limitation.

*Y. pestis* has a multitude of virulence factors that enable it to evade host immune responses and spread systemically throughout the host including plasminogen activator (Pla) and a T3SS with *Yersinia* outer protein (Yop) effectors. Yop effectors are translocated directly into host cells via a T3SS to inhibit phagocytosis, down regulate pro-inflammatory cytokines, and induce cell death (Demeure et al., 2019). A recent study demonstrated that these effectors and the T3SS are necessary for causing hemorrhage of endothelial cells directly allowing *Y. pestis* to cause septicemia (Mikaty et al., 2021). Pla contributes to rapid lethality often observed in primary pneumonic plague and is necessary to cause bubonic plague (Sebbane et al., 2020). Although the exact mechanisms of how Pla causes bubonic plague are unknown, the current model suggests that Pla degrades fibrin clots, extracellular matrix, and basement membranes to gain entry to the lymphatic system (Sebbane et al., 2020). Pla also targets and disrupts the hemostasis pathway, by cleaving plasminogen and inducing fibrinolysis while also inducing coagulation by inactivating the tissue factor pathway inhibitor (TFPI) (Sebbane et al., 2020). Previous case reports and studies have indicated that patients treated with tissue plasminogen activator for myocardial infarction resulted in intracranial hemorrhage (Kase et al., 1990; Whiteley et al., 2012). Furthermore, *Streptococcus agalactiae* is capable of hijacking the fibrinolytic system to invade the brain, causing meningoencephalitis via a plasminogen binding surface protein (Lentini et al., 2018; Quan et al., 2025). By modulating the hemostatic pathway with Pla, *Y. pestis* may have the ability to invade the brain via hemorrhage of the vascular system. Pla and Yop effectors are important for dissemination and the breakdown of endothelial cells and basement membranes by *Y. pestis*, both of which are key components of the BBB. Therefore, these virulence factors in combination with increased proinflammatory cytokine levels, (e.g., IL-6) and neuroinflammatory responses may play an important role in the breakdown of the BBB resulting in meningitis found in previous case studies (Scheller et al., 2011; Gryka-Marton et al., 2025; Yang et al., 2022).

While we demonstrate congestion of cerebral blood vessels and a neuroinflammatory response within the CNS, we did not observe *Y. pestis* causing meningitis in our acute model of pneumonic plague caused by the fully virulent *Y. pestis* CO92 strain. We have previously described a strain of *Y. pestis* CO92 that had a mutation in the twin arginine translocation (Tat) system (Bozue et al., 2014). In this study we observed that infection with the *Y. pestis* Δ*tatA* mutant resulted in a meningitis in the cerebrum/cerebellum when delivered to mice via intranasal instillation, but not small-particle aerosol delivery. However, during this study we did not perform IHC on the samples collected from mice exposed via small-particle exposure because there were no clinical lesions observed after HE stains, and the brain-associated vascular involvement was likely missed without the use of IHC.

Of the reported cases of plague causing meningitis, the majority of these cases were secondary to bubonic plague and the individuals did not receive antibiotic treatment or they received treatment three or more days after symptom onset (Cooley et al., 2023). Our results demonstrate that pneumonic plague without treatment causes lethal disease due to respiratory failure and bacteremia usually by 3 or 4 days post-challenge, and disease progression is likely too rapid for meningitis to develop. Experiments with lower dosing of *Y. pestis* or suboptimal antibiotic treatment might extend disease progression leading to BBB breakdown and the development of meningitis. In addition, mouse strain difference may allow for C57BL/6 mice to better model meningeal complications.

The differential gene and cytokine expression analyses described here offer new data that could be leveraged for the development of medical countermeasures or diagnostic strategies. Given the strong signal of myelin dysfunction in mice with pneumonic plague, targeting genes like *Opalin* or *Pllp* for upregulation or gene silencing could equilibrate downstream gene expression of oligodendrocyte-associated genes. Activation and upregulation of genes associated with astrocyte function can be due to an infection response resulting in increased inflammation and restricting passage through the BBB. Of the transcripts associated with glial cells examined, astrocyte associated genes have the highest gene count and is the most upregulated gene set in both models presented here indicating possible disruption to this vital component protecting the brain. Although this is a critical function in the defense of neurological infections, some immune responses can negatively impact BBB permeability. For example, increased expression of *Serpina3n* was previously shown to cause BBB dysfunction through vascular inflammation when activated by STAT3, a signal transducer and activator of transcription (Kim et al., 2022; Darnell et al., 1994). STAT3 is activated by IL-6 which is released by activation of NF-κB in astrocyte cells and we observe that IL-6 expression is increased in mice after exposure to aerosolized *Y. pestis* (Abbott, 2002; Levy and Lee, 2002). Silencing *Serpina3n* expression, limiting the expression of IL-6 or blocking the IL-6 receptor may prevent potential breaches of the BBB (Pons-Espinal et al., 2024). More work is required to develop a true plague meningitis model as this current model may represent early or indirect brain-associated inflammatory changes rather than confirmed meningitis. However, the data sets presented here, and the direct comparison of neuroinflammation in brain homogenates caused by *Y. pestis* and *B. pseudomallei* lay the groundwork for model refinement. By understanding the impact of exposure to aerosolized *Y. pestis* on the host-immune response in the CNS, we can begin to more precisely characterize the immune mechanisms required to clear *Y. pestis* and prevent plague.

## Data Availability

The datasets presented in this study can be found in online repositories. The names of the repository/repositories and accession number(s) can be found in the article/Supplementary material.

## Author disclaimer

All opinions expressed in this paper are the author’s and do not necessarily reflect the policies and views of DOD, DOE or ORAU/ORISE. The opinions, interpretations, conclusions, and recommendations presented are those of the author and are not necessarily endorsed by the U. S. Army or the Department of Defense. The use of either trade or manufacturers’ names in this report does not constitute an official endorsement of any commercial products. This report may not be cited for purposes of advertisement.
