# Supplementary material for: Vascular-associated bacterial burden and neuroinflammatory transcriptional responses observed in models of pneumonic plague
Source: Front Microbiol. 2026 Jun 24;17:1865125. doi: 10.3389/fmicb.2026.1865125 (PMC13341613; doi:10.3389/fmicb.2026.1865125)
Supplement: Supplementary file 1 [file Data_Sheet_1.ZIP › Bp_final 2026-04-14 12-30/AdvAnalysisReport.html]

nCounter Advanced Analysis Report


- #

- Overview
- Diff Expr
- GSA
- PathView
- Cell Type Profiling
- Analysis Parameters
- Share

This module generates a series of high level plots that describe the data overall and may be useful for identifying anomalous data and/or covariates.

Gene sets:

- Summary
- Adaptive Immune Response
- Angiogenesis
- Apoptosis
- Astrocyte Function
- Autophagy
- Carbohydrate Metabolism
- Cell Cycle
- Cellular Stress
- Cytokine Signaling
- DNA Damage
- Epigenetic Regulation
- Growth Factor Signaling
- Inflammatory Signaling
- Innate Immune Response
- Insulin Signaling
- Lipid Metabolism
- Matrix Remodeling
- Microglia Function
- Neurons and Neurotransmission
- NF-kB
- Notch
- Oligodendrocyte Function
- Wnt

- Heatmaps
- PCA
- Study design
- Other QC

- Heatmaps

- DPI

- Covariates

- Other QC

- **Heatmap of Raw Data  
  More Plot Information  Download detected/undetected calls data**

  ##### Heatmap of Raw Data

  Heatmap of the raw counts. The plot is meant to provide an overview of how robust the raw expression levels are across samples and gene sets. Datasets that entirely lack higher level expressions (e.g. counts > 100) may indicate experimental failure or low input. The detected/undetected calls links to a .csv file stating whether each probe is above background, with 0/1 indicating below/above background. If the user has not specified a detection threshold, probes are called detected if they have more than double the counts of the median negative control.
- **Heatmap of All Data  
  More Plot Information**

  ##### Heatmap of All Data

  Heatmap of the normalized data, scaled to give all genes equal variance, generated via unsupervised clustering. Orange indicates high expression; blue indicates low expression. This plot is meant to provide a high level exploratory view of the data.

- **Principal Components of All Data  
  More Plot Information**

  ##### Principal Components of All Data

  Principal component analysis maps high-dimensional datasets onto a smaller number of highly informative dimensions. Here, the first four principal components of the gene expression data are plotted against each other and colored by the values of the selected covariate. This plot may be used to identify clusters in the data and to identify variables associated with prominent signal in the data. Variables that are associated with these leading principal components should be considered in downstream analyses.

- **More Plot Information**

  ##### 

  Pairwise comparisons of all covariates in the analysis. The type of plot is dependent on the types of variables compared; A categorical vs. categorical covariate plot is shown as a bar chart of counts (Y axis). Continuous vs. categorical covariates generate a boxplot with whiskers denoting 1.5 IQR. Continuous vs. continuous covariates are compared via a scatter plot. Variables that are correlated with a biological variable of interest are potential confounders that may influence downstream analyses. Additionally, bar plots and histograms show the distributions of categorical and continuous variables, respectively.

- **Variance vs. Mean normalized signal plot across all targets/probes  
  More Plot Information  Mean and Variance statistics across all genes**

  ##### Variance vs. Mean normalized signal plot across all targets/probes

  Each gene's variance in the log-scaled, normalized data is plotted against its mean value across all samples. Highly variable genes are indicated by gene name. Housekeeping genes are color coded according to their use in (or omission from) normalization.
- **p-value distribution plots  
  More Plot Information**

  ##### p-value distribution plots

  For each covariate included in the analysis, a histogram of p-values testing each gene's univariate association with the chosen covariate is displayed. Covariates with largely flat histograms have minimal association with gene expression; covariates with histograms with significantly more mass on the left are either associated with the expression of many genes or are confounded with a covariate that is associated with the expression. Low p-values indicate strong evidence for an association.

- Heatmaps
- PCA

- Heatmaps

- DPI

- **Heatmap of Adaptive Immune Response Data  
  More Plot Information**

  ##### Heatmap of Adaptive Immune Response Data

  Heatmap of the normalized data, scaled to give all genes equal variance, generated via unsupervised clustering. Orange indicates high expression; blue indicates low expression. This plot is meant to provide a high level exploratory view of the data.

- **Principal Components of Adaptive Immune Response Data  
  More Plot Information**

  ##### Principal Components of Adaptive Immune Response Data

  Principal component analysis maps high-dimensional datasets onto a smaller number of highly informative dimensions. Here, the first four principal components of the gene expression data are plotted against each other and colored by the values of the selected covariate. This plot may be used to identify clusters in the data and to identify variables associated with prominent signal in the data. Variables that are associated with these leading principal components should be considered in downstream analyses.

- Heatmaps
- PCA

- Heatmaps

- DPI

- **Heatmap of Angiogenesis Data  
  More Plot Information**

  ##### Heatmap of Angiogenesis Data

  Heatmap of the normalized data, scaled to give all genes equal variance, generated via unsupervised clustering. Orange indicates high expression; blue indicates low expression. This plot is meant to provide a high level exploratory view of the data.

- **Principal Components of Angiogenesis Data  
  More Plot Information**

  ##### Principal Components of Angiogenesis Data

  Principal component analysis maps high-dimensional datasets onto a smaller number of highly informative dimensions. Here, the first four principal components of the gene expression data are plotted against each other and colored by the values of the selected covariate. This plot may be used to identify clusters in the data and to identify variables associated with prominent signal in the data. Variables that are associated with these leading principal components should be considered in downstream analyses.

- Heatmaps
- PCA

- Heatmaps

- DPI

- **Heatmap of Apoptosis Data  
  More Plot Information**

  ##### Heatmap of Apoptosis Data

  Heatmap of the normalized data, scaled to give all genes equal variance, generated via unsupervised clustering. Orange indicates high expression; blue indicates low expression. This plot is meant to provide a high level exploratory view of the data.

- **Principal Components of Apoptosis Data  
  More Plot Information**

  ##### Principal Components of Apoptosis Data

  Principal component analysis maps high-dimensional datasets onto a smaller number of highly informative dimensions. Here, the first four principal components of the gene expression data are plotted against each other and colored by the values of the selected covariate. This plot may be used to identify clusters in the data and to identify variables associated with prominent signal in the data. Variables that are associated with these leading principal components should be considered in downstream analyses.

- Heatmaps
- PCA

- Heatmaps

- DPI

- **Heatmap of Astrocyte Function Data  
  More Plot Information**

  ##### Heatmap of Astrocyte Function Data

  Heatmap of the normalized data, scaled to give all genes equal variance, generated via unsupervised clustering. Orange indicates high expression; blue indicates low expression. This plot is meant to provide a high level exploratory view of the data.

- **Principal Components of Astrocyte Function Data  
  More Plot Information**

  ##### Principal Components of Astrocyte Function Data

  Principal component analysis maps high-dimensional datasets onto a smaller number of highly informative dimensions. Here, the first four principal components of the gene expression data are plotted against each other and colored by the values of the selected covariate. This plot may be used to identify clusters in the data and to identify variables associated with prominent signal in the data. Variables that are associated with these leading principal components should be considered in downstream analyses.

- Heatmaps
- PCA

- Heatmaps

- DPI

- **Heatmap of Autophagy Data  
  More Plot Information**

  ##### Heatmap of Autophagy Data

  Heatmap of the normalized data, scaled to give all genes equal variance, generated via unsupervised clustering. Orange indicates high expression; blue indicates low expression. This plot is meant to provide a high level exploratory view of the data.

- **Principal Components of Autophagy Data  
  More Plot Information**

  ##### Principal Components of Autophagy Data

  Principal component analysis maps high-dimensional datasets onto a smaller number of highly informative dimensions. Here, the first four principal components of the gene expression data are plotted against each other and colored by the values of the selected covariate. This plot may be used to identify clusters in the data and to identify variables associated with prominent signal in the data. Variables that are associated with these leading principal components should be considered in downstream analyses.

- Heatmaps
- PCA

- Heatmaps

- DPI

- **Heatmap of Carbohydrate Metabolism Data  
  More Plot Information**

  ##### Heatmap of Carbohydrate Metabolism Data

  Heatmap of the normalized data, scaled to give all genes equal variance, generated via unsupervised clustering. Orange indicates high expression; blue indicates low expression. This plot is meant to provide a high level exploratory view of the data.

- **Principal Components of Carbohydrate Metabolism Data  
  More Plot Information**

  ##### Principal Components of Carbohydrate Metabolism Data

  Principal component analysis maps high-dimensional datasets onto a smaller number of highly informative dimensions. Here, the first four principal components of the gene expression data are plotted against each other and colored by the values of the selected covariate. This plot may be used to identify clusters in the data and to identify variables associated with prominent signal in the data. Variables that are associated with these leading principal components should be considered in downstream analyses.

- Heatmaps
- PCA

- Heatmaps

- DPI

- **Heatmap of Cell Cycle Data  
  More Plot Information**

  ##### Heatmap of Cell Cycle Data

  Heatmap of the normalized data, scaled to give all genes equal variance, generated via unsupervised clustering. Orange indicates high expression; blue indicates low expression. This plot is meant to provide a high level exploratory view of the data.

- **Principal Components of Cell Cycle Data  
  More Plot Information**

  ##### Principal Components of Cell Cycle Data

  Principal component analysis maps high-dimensional datasets onto a smaller number of highly informative dimensions. Here, the first four principal components of the gene expression data are plotted against each other and colored by the values of the selected covariate. This plot may be used to identify clusters in the data and to identify variables associated with prominent signal in the data. Variables that are associated with these leading principal components should be considered in downstream analyses.

- Heatmaps
- PCA

- Heatmaps

- DPI

- **Heatmap of Cellular Stress Data  
  More Plot Information**

  ##### Heatmap of Cellular Stress Data

  Heatmap of the normalized data, scaled to give all genes equal variance, generated via unsupervised clustering. Orange indicates high expression; blue indicates low expression. This plot is meant to provide a high level exploratory view of the data.

- **Principal Components of Cellular Stress Data  
  More Plot Information**

  ##### Principal Components of Cellular Stress Data

  Principal component analysis maps high-dimensional datasets onto a smaller number of highly informative dimensions. Here, the first four principal components of the gene expression data are plotted against each other and colored by the values of the selected covariate. This plot may be used to identify clusters in the data and to identify variables associated with prominent signal in the data. Variables that are associated with these leading principal components should be considered in downstream analyses.

- Heatmaps
- PCA

- Heatmaps

- DPI

- **Heatmap of Cytokine Signaling Data  
  More Plot Information**

  ##### Heatmap of Cytokine Signaling Data

  Heatmap of the normalized data, scaled to give all genes equal variance, generated via unsupervised clustering. Orange indicates high expression; blue indicates low expression. This plot is meant to provide a high level exploratory view of the data.

- **Principal Components of Cytokine Signaling Data  
  More Plot Information**

  ##### Principal Components of Cytokine Signaling Data

  Principal component analysis maps high-dimensional datasets onto a smaller number of highly informative dimensions. Here, the first four principal components of the gene expression data are plotted against each other and colored by the values of the selected covariate. This plot may be used to identify clusters in the data and to identify variables associated with prominent signal in the data. Variables that are associated with these leading principal components should be considered in downstream analyses.

- Heatmaps
- PCA

- Heatmaps

- DPI

- **Heatmap of DNA Damage Data  
  More Plot Information**

  ##### Heatmap of DNA Damage Data

  Heatmap of the normalized data, scaled to give all genes equal variance, generated via unsupervised clustering. Orange indicates high expression; blue indicates low expression. This plot is meant to provide a high level exploratory view of the data.

- **Principal Components of DNA Damage Data  
  More Plot Information**

  ##### Principal Components of DNA Damage Data

  Principal component analysis maps high-dimensional datasets onto a smaller number of highly informative dimensions. Here, the first four principal components of the gene expression data are plotted against each other and colored by the values of the selected covariate. This plot may be used to identify clusters in the data and to identify variables associated with prominent signal in the data. Variables that are associated with these leading principal components should be considered in downstream analyses.

- Heatmaps
- PCA

- Heatmaps

- DPI

- **Heatmap of Epigenetic Regulation Data  
  More Plot Information**

  ##### Heatmap of Epigenetic Regulation Data

  Heatmap of the normalized data, scaled to give all genes equal variance, generated via unsupervised clustering. Orange indicates high expression; blue indicates low expression. This plot is meant to provide a high level exploratory view of the data.

- **Principal Components of Epigenetic Regulation Data  
  More Plot Information**

  ##### Principal Components of Epigenetic Regulation Data

  Principal component analysis maps high-dimensional datasets onto a smaller number of highly informative dimensions. Here, the first four principal components of the gene expression data are plotted against each other and colored by the values of the selected covariate. This plot may be used to identify clusters in the data and to identify variables associated with prominent signal in the data. Variables that are associated with these leading principal components should be considered in downstream analyses.

- Heatmaps
- PCA

- Heatmaps

- DPI

- **Heatmap of Growth Factor Signaling Data  
  More Plot Information**

  ##### Heatmap of Growth Factor Signaling Data

  Heatmap of the normalized data, scaled to give all genes equal variance, generated via unsupervised clustering. Orange indicates high expression; blue indicates low expression. This plot is meant to provide a high level exploratory view of the data.

- **Principal Components of Growth Factor Signaling Data  
  More Plot Information**

  ##### Principal Components of Growth Factor Signaling Data

  Principal component analysis maps high-dimensional datasets onto a smaller number of highly informative dimensions. Here, the first four principal components of the gene expression data are plotted against each other and colored by the values of the selected covariate. This plot may be used to identify clusters in the data and to identify variables associated with prominent signal in the data. Variables that are associated with these leading principal components should be considered in downstream analyses.

- Heatmaps
- PCA

- Heatmaps

- DPI

- **Heatmap of Inflammatory Signaling Data  
  More Plot Information**

  ##### Heatmap of Inflammatory Signaling Data

  Heatmap of the normalized data, scaled to give all genes equal variance, generated via unsupervised clustering. Orange indicates high expression; blue indicates low expression. This plot is meant to provide a high level exploratory view of the data.

- **Principal Components of Inflammatory Signaling Data  
  More Plot Information**

  ##### Principal Components of Inflammatory Signaling Data

  Principal component analysis maps high-dimensional datasets onto a smaller number of highly informative dimensions. Here, the first four principal components of the gene expression data are plotted against each other and colored by the values of the selected covariate. This plot may be used to identify clusters in the data and to identify variables associated with prominent signal in the data. Variables that are associated with these leading principal components should be considered in downstream analyses.

- Heatmaps
- PCA

- Heatmaps

- DPI

- **Heatmap of Innate Immune Response Data  
  More Plot Information**

  ##### Heatmap of Innate Immune Response Data

  Heatmap of the normalized data, scaled to give all genes equal variance, generated via unsupervised clustering. Orange indicates high expression; blue indicates low expression. This plot is meant to provide a high level exploratory view of the data.

- **Principal Components of Innate Immune Response Data  
  More Plot Information**

  ##### Principal Components of Innate Immune Response Data

  Principal component analysis maps high-dimensional datasets onto a smaller number of highly informative dimensions. Here, the first four principal components of the gene expression data are plotted against each other and colored by the values of the selected covariate. This plot may be used to identify clusters in the data and to identify variables associated with prominent signal in the data. Variables that are associated with these leading principal components should be considered in downstream analyses.

- Heatmaps
- PCA

- Heatmaps

- DPI

- **Heatmap of Insulin Signaling Data  
  More Plot Information**

  ##### Heatmap of Insulin Signaling Data

  Heatmap of the normalized data, scaled to give all genes equal variance, generated via unsupervised clustering. Orange indicates high expression; blue indicates low expression. This plot is meant to provide a high level exploratory view of the data.

- **Principal Components of Insulin Signaling Data  
  More Plot Information**

  ##### Principal Components of Insulin Signaling Data

  Principal component analysis maps high-dimensional datasets onto a smaller number of highly informative dimensions. Here, the first four principal components of the gene expression data are plotted against each other and colored by the values of the selected covariate. This plot may be used to identify clusters in the data and to identify variables associated with prominent signal in the data. Variables that are associated with these leading principal components should be considered in downstream analyses.

- Heatmaps
- PCA

- Heatmaps

- DPI

- **Heatmap of Lipid Metabolism Data  
  More Plot Information**

  ##### Heatmap of Lipid Metabolism Data

  Heatmap of the normalized data, scaled to give all genes equal variance, generated via unsupervised clustering. Orange indicates high expression; blue indicates low expression. This plot is meant to provide a high level exploratory view of the data.

- **Principal Components of Lipid Metabolism Data  
  More Plot Information**

  ##### Principal Components of Lipid Metabolism Data

  Principal component analysis maps high-dimensional datasets onto a smaller number of highly informative dimensions. Here, the first four principal components of the gene expression data are plotted against each other and colored by the values of the selected covariate. This plot may be used to identify clusters in the data and to identify variables associated with prominent signal in the data. Variables that are associated with these leading principal components should be considered in downstream analyses.

- Heatmaps
- PCA

- Heatmaps

- DPI

- **Heatmap of Matrix Remodeling Data  
  More Plot Information**

  ##### Heatmap of Matrix Remodeling Data

  Heatmap of the normalized data, scaled to give all genes equal variance, generated via unsupervised clustering. Orange indicates high expression; blue indicates low expression. This plot is meant to provide a high level exploratory view of the data.

- **Principal Components of Matrix Remodeling Data  
  More Plot Information**

  ##### Principal Components of Matrix Remodeling Data

  Principal component analysis maps high-dimensional datasets onto a smaller number of highly informative dimensions. Here, the first four principal components of the gene expression data are plotted against each other and colored by the values of the selected covariate. This plot may be used to identify clusters in the data and to identify variables associated with prominent signal in the data. Variables that are associated with these leading principal components should be considered in downstream analyses.

- Heatmaps
- PCA

- Heatmaps

- DPI

- **Heatmap of Microglia Function Data  
  More Plot Information**

  ##### Heatmap of Microglia Function Data

  Heatmap of the normalized data, scaled to give all genes equal variance, generated via unsupervised clustering. Orange indicates high expression; blue indicates low expression. This plot is meant to provide a high level exploratory view of the data.

- **Principal Components of Microglia Function Data  
  More Plot Information**

  ##### Principal Components of Microglia Function Data

  Principal component analysis maps high-dimensional datasets onto a smaller number of highly informative dimensions. Here, the first four principal components of the gene expression data are plotted against each other and colored by the values of the selected covariate. This plot may be used to identify clusters in the data and to identify variables associated with prominent signal in the data. Variables that are associated with these leading principal components should be considered in downstream analyses.

- Heatmaps
- PCA

- Heatmaps

- DPI

- **Heatmap of Neurons and Neurotransmission Data  
  More Plot Information**

  ##### Heatmap of Neurons and Neurotransmission Data

  Heatmap of the normalized data, scaled to give all genes equal variance, generated via unsupervised clustering. Orange indicates high expression; blue indicates low expression. This plot is meant to provide a high level exploratory view of the data.

- **Principal Components of Neurons and Neurotransmission Data  
  More Plot Information**

  ##### Principal Components of Neurons and Neurotransmission Data

  Principal component analysis maps high-dimensional datasets onto a smaller number of highly informative dimensions. Here, the first four principal components of the gene expression data are plotted against each other and colored by the values of the selected covariate. This plot may be used to identify clusters in the data and to identify variables associated with prominent signal in the data. Variables that are associated with these leading principal components should be considered in downstream analyses.

- Heatmaps
- PCA

- Heatmaps

- DPI

- **Heatmap of NF-kB Data  
  More Plot Information**

  ##### Heatmap of NF-kB Data

  Heatmap of the normalized data, scaled to give all genes equal variance, generated via unsupervised clustering. Orange indicates high expression; blue indicates low expression. This plot is meant to provide a high level exploratory view of the data.

- **Principal Components of NF-kB Data  
  More Plot Information**

  ##### Principal Components of NF-kB Data

  Principal component analysis maps high-dimensional datasets onto a smaller number of highly informative dimensions. Here, the first four principal components of the gene expression data are plotted against each other and colored by the values of the selected covariate. This plot may be used to identify clusters in the data and to identify variables associated with prominent signal in the data. Variables that are associated with these leading principal components should be considered in downstream analyses.

- Heatmaps
- PCA

- Heatmaps

- DPI

- **Heatmap of Notch Data  
  More Plot Information**

  ##### Heatmap of Notch Data

  Heatmap of the normalized data, scaled to give all genes equal variance, generated via unsupervised clustering. Orange indicates high expression; blue indicates low expression. This plot is meant to provide a high level exploratory view of the data.

- **Principal Components of Notch Data  
  More Plot Information**

  ##### Principal Components of Notch Data

  Principal component analysis maps high-dimensional datasets onto a smaller number of highly informative dimensions. Here, the first four principal components of the gene expression data are plotted against each other and colored by the values of the selected covariate. This plot may be used to identify clusters in the data and to identify variables associated with prominent signal in the data. Variables that are associated with these leading principal components should be considered in downstream analyses.

- Heatmaps
- PCA

- Heatmaps

- DPI

- **Heatmap of Oligodendrocyte Function Data  
  More Plot Information**

  ##### Heatmap of Oligodendrocyte Function Data

  Heatmap of the normalized data, scaled to give all genes equal variance, generated via unsupervised clustering. Orange indicates high expression; blue indicates low expression. This plot is meant to provide a high level exploratory view of the data.

- **Principal Components of Oligodendrocyte Function Data  
  More Plot Information**

  ##### Principal Components of Oligodendrocyte Function Data

  Principal component analysis maps high-dimensional datasets onto a smaller number of highly informative dimensions. Here, the first four principal components of the gene expression data are plotted against each other and colored by the values of the selected covariate. This plot may be used to identify clusters in the data and to identify variables associated with prominent signal in the data. Variables that are associated with these leading principal components should be considered in downstream analyses.

- Heatmaps
- PCA

- Heatmaps

- DPI

- **Heatmap of Wnt Data  
  More Plot Information**

  ##### Heatmap of Wnt Data

  Heatmap of the normalized data, scaled to give all genes equal variance, generated via unsupervised clustering. Orange indicates high expression; blue indicates low expression. This plot is meant to provide a high level exploratory view of the data.

- **Principal Components of Wnt Data  
  More Plot Information**

  ##### Principal Components of Wnt Data

  Principal component analysis maps high-dimensional datasets onto a smaller number of highly informative dimensions. Here, the first four principal components of the gene expression data are plotted against each other and colored by the values of the selected covariate. This plot may be used to identify clusters in the data and to identify variables associated with prominent signal in the data. Variables that are associated with these leading principal components should be considered in downstream analyses.

Genes are tested for differential expression in response to each selected covariate. For each gene, a single linear
regression is fit using all selected covariates to predict expression. This approach eliminates confounding due to measured
covariates and isolates the independent association of each covariate with gene expression, measuring each variable's
association with a gene after holding all other variables constant.

DPI:
- DPI: differential expression in 1 vs. baseline of CTRL
- DPI: differential expression in 2 vs. baseline of CTRL
- DPI: differential expression in 3 vs. baseline of CTRL

- **Volcano Plot: DPI: 1 vs.CTRL  
  More Plot Information**

  ##### Volcano Plot: DPI: 1 vs.CTRL

  Volcano plot displaying each gene's -log10(p-value) and log2 fold change with the selected covariate. Highly statistically significant genes fall at the top of the plot above the horizontal lines, and highly differentially expressed genes fall to either side. Horizontal lines indicate various False Discovery Rate (FDR) thresholds or p-value thresholds if there is no adjustment to the p-values. Genes are colored if the resulting p-value is below the given FDR or p-value threshold. The 40 most statistically significant genes are labeled in the plot.
- **DE Results: DPI: 1 vs.CTRL  
  More Plot Information  Download CSV Data**

  |  | Log2 fold change | std error (log2) | Lower confidence limit (log2) | Upper confidence limit (log2) | Linear fold change | Lower confidence limit (linear) | Upper confidence limit (linear) | P-value | BY.p.value | method | Gene.sets | probe.ID |
  | --- | --- | --- | --- | --- | --- | --- | --- | --- | --- | --- | --- | --- |
  | Lcn2-mRNA | 6.98 | 0.503 | 6 | 7.97 | 127 | 63.9 | 251 | 9.34e-09 | 3.73e-05 | lm.nb | Astrocyte Function, Inflammatory Signaling | NM\_008491.1:190 |
  | Homer1-mRNA | -0.513 | 0.0603 | -0.631 | -0.395 | 0.701 | 0.646 | 0.76 | 1.99e-06 | 0.00397 | loglinear | Neurons and Neurotransmission | NM\_147176.2:1165 |
  | Tmem119-mRNA | -0.702 | 0.112 | -0.922 | -0.481 | 0.615 | 0.528 | 0.716 | 4.29e-05 | 0.0571 | loglinear | Microglia Function | NM\_146162.2:1550 |
  | Cldn5-mRNA | -1.21 | 0.202 | -1.6 | -0.81 | 0.433 | 0.329 | 0.57 | 6.54e-05 | 0.0653 | lm.nb | Matrix Remodeling | NM\_013805.4:975 |
  | Kat2b-mRNA | 0.472 | 0.0862 | 0.303 | 0.641 | 1.39 | 1.23 | 1.56 | 0.000141 | 0.0959 | loglinear | Epigenetic Regulation, Notch | NM\_020005.3:3030 |
  | F3-mRNA | 0.475 | 0.087 | 0.305 | 0.646 | 1.39 | 1.24 | 1.56 | 0.000144 | 0.0959 | loglinear | Microglia Function | NM\_010171.3:1170 |
  | Ifitm2-mRNA | 0.897 | 0.17 | 0.563 | 1.23 | 1.86 | 1.48 | 2.35 | 2e-04 | 0.114 | lm.nb | Adaptive Immune Response, Inflammatory Signaling | NM\_030694.1:87 |
  | Ifitm3-mRNA | 0.83 | 0.163 | 0.511 | 1.15 | 1.78 | 1.42 | 2.22 | 0.000264 | 0.125 | lm.nb | Adaptive Immune Response, Inflammatory Signaling | NM\_025378.2:370 |
  | Cp-mRNA | 0.816 | 0.163 | 0.497 | 1.13 | 1.76 | 1.41 | 2.2 | 0.000303 | 0.125 | lm.nb | Astrocyte Function | NM\_001042611.1:1750 |
  | Osmr-mRNA | 1.68 | 0.337 | 1.02 | 2.34 | 3.21 | 2.03 | 5.07 | 0.000313 | 0.125 | lm.nb | Astrocyte Function, Cytokine Signaling, Growth Factor Signaling | NM\_011019.3:395 |
  | Ralb-mRNA | 0.207 | 0.047 | 0.115 | 0.299 | 1.15 | 1.08 | 1.23 | 0.000866 | 0.314 | loglinear | Autophagy, Growth Factor Signaling | NM\_022327.5:1120 |
  | Clic4-mRNA | 0.499 | 0.117 | 0.271 | 0.728 | 1.41 | 1.21 | 1.66 | 0.00106 | 0.335 | lm.nb | Autophagy, Microglia Function | NM\_013885.2:3280 |
  | Ccl2-mRNA | 4.15 | 0.972 | 2.24 | 6.05 | 17.8 | 4.74 | 66.5 | 0.00109 | 0.335 | lm.nb | Astrocyte Function, Cytokine Signaling, Inflammatory Signaling, Innate Immune Response | NM\_011333.3:415 |
  | Nlgn1-mRNA | -0.294 | 0.0713 | -0.434 | -0.155 | 0.815 | 0.74 | 0.898 | 0.0014 | 0.399 | loglinear | Matrix Remodeling, Neurons and Neurotransmission | NM\_138666.3:1028 |
  | Lingo1-mRNA | -0.371 | 0.0936 | -0.555 | -0.188 | 0.773 | 0.681 | 0.878 | 0.00187 | 0.437 | loglinear | Growth Factor Signaling, Oligodendrocyte Function | NM\_181074.4:1087 |
  | Ms4a4a-mRNA | 2 | 0.508 | 1 | 3 | 4 | 2.01 | 7.98 | 0.00198 | 0.437 | lm.nb |  | XM\_003086124.1:252 |
  | Grm3-mRNA | -0.323 | 0.0825 | -0.484 | -0.161 | 0.8 | 0.715 | 0.894 | 0.00206 | 0.437 | loglinear | Astrocyte Function, Neurons and Neurotransmission | NM\_181850.2:2525 |
  | Cd14-mRNA | 2.57 | 0.657 | 1.28 | 3.85 | 5.92 | 2.43 | 14.5 | 0.00208 | 0.437 | lm.nb | Apoptosis, Astrocyte Function, Autophagy, Growth Factor Signaling, Innate Immune Response, NF-kB | NM\_009841.3:235 |
  | Ets2-mRNA | 0.213 | 0.0545 | 0.106 | 0.319 | 1.16 | 1.08 | 1.25 | 0.00211 | 0.437 | loglinear | Cellular Stress, Growth Factor Signaling, Microglia Function | NM\_011809.2:3284 |
  | Sesn1-mRNA | 0.237 | 0.0612 | 0.117 | 0.357 | 1.18 | 1.08 | 1.28 | 0.00219 | 0.437 | loglinear | DNA Damage | NM\_001013370.2:497 |

  ##### DE Results: DPI: 1 vs.CTRL

  Table presenting the most statistically significantly differentially expressed genes with the selected covariate. 'Estimated log fold-change' estimates a gene's differential expression. For categorical covariates, a gene is estimated to have 2^(log fold change) times its expression in baseline samples, holding all other variables in the analysis constant. If the covariate is continuous, for each unit increase in the selected covariate, a gene's expression is estimated to increase by 2^(log fold change)-fold, holding all other variables in the analysis constant. The 95% confidence interval for the log fold change is also presented, along with a p-value and an adjusted p-value or FDR if requested. Method column indicates the model used to estimate differential expression. Mixed negative binomial model uses the mle function to run the Wald test. The simplified negative binomial model uses the glm.nb function . The loglinear model uses the lm function.

- **Volcano Plot: DPI: 2 vs.CTRL  
  More Plot Information**

  ##### Volcano Plot: DPI: 2 vs.CTRL

  Volcano plot displaying each gene's -log10(p-value) and log2 fold change with the selected covariate. Highly statistically significant genes fall at the top of the plot above the horizontal lines, and highly differentially expressed genes fall to either side. Horizontal lines indicate various False Discovery Rate (FDR) thresholds or p-value thresholds if there is no adjustment to the p-values. Genes are colored if the resulting p-value is below the given FDR or p-value threshold. The 40 most statistically significant genes are labeled in the plot.
- **DE Results: DPI: 2 vs.CTRL  
  More Plot Information  Download CSV Data**

  |  | Log2 fold change | std error (log2) | Lower confidence limit (log2) | Upper confidence limit (log2) | Linear fold change | Lower confidence limit (linear) | Upper confidence limit (linear) | P-value | BY.p.value | method | Gene.sets | probe.ID |
  | --- | --- | --- | --- | --- | --- | --- | --- | --- | --- | --- | --- | --- |
  | Lcn2-mRNA | 9.93 | 0.502 | 8.94 | 10.9 | 972 | 492 | 1920 | 1.58e-10 | 3.57e-07 | lm.nb | Astrocyte Function, Inflammatory Signaling | NM\_008491.1:190 |
  | Gbp2-mRNA | 5.23 | 0.267 | 4.71 | 5.76 | 37.6 | 26.2 | 54.1 | 1.79e-10 | 3.57e-07 | lm.nb | Astrocyte Function, Inflammatory Signaling | NM\_010260.1:1996 |
  | Ifitm3-mRNA | 2.95 | 0.16 | 2.64 | 3.26 | 7.72 | 6.21 | 9.59 | 3.56e-10 | 4.74e-07 | lm.nb | Adaptive Immune Response, Inflammatory Signaling | NM\_025378.2:370 |
  | Fkbp5-mRNA | 1.85 | 0.124 | 1.61 | 2.1 | 3.61 | 3.05 | 4.27 | 4.19e-09 | 3.71e-06 | loglinear | Astrocyte Function | NM\_010220.3:2125 |
  | Cdkn1a-mRNA | 2.21 | 0.15 | 1.92 | 2.51 | 4.64 | 3.78 | 5.68 | 4.64e-09 | 3.71e-06 | lm.nb | Adaptive Immune Response, Cell Cycle, Cellular Stress, Cytokine Signaling, DNA Damage, Growth Factor Signaling | NM\_007669.4:1670 |
  | Sesn1-mRNA | 0.872 | 0.0612 | 0.752 | 0.992 | 1.83 | 1.68 | 1.99 | 6.94e-09 | 4.29e-06 | loglinear | DNA Damage | NM\_001013370.2:497 |
  | Psmb8-mRNA | 2.78 | 0.196 | 2.39 | 3.16 | 6.85 | 5.25 | 8.94 | 7.51e-09 | 4.29e-06 | lm.nb | Adaptive Immune Response, Angiogenesis, Apoptosis, Astrocyte Function, Cell Cycle, Cytokine Signaling, Growth Factor Signaling, Inflammatory Signaling, Insulin Signaling, Microglia Function, NF-kB, Wnt | NM\_010724.2:362 |
  | Homer1-mRNA | -0.828 | 0.0603 | -0.946 | -0.71 | 0.563 | 0.519 | 0.611 | 1.06e-08 | 5.31e-06 | loglinear | Neurons and Neurotransmission | NM\_147176.2:1165 |
  | Cp-mRNA | 2.04 | 0.156 | 1.73 | 2.34 | 4.11 | 3.33 | 5.08 | 1.8e-08 | 8e-06 | lm.nb | Astrocyte Function | NM\_001042611.1:1750 |
  | Zbp1-mRNA | 4.74 | 0.408 | 3.94 | 5.54 | 26.7 | 15.3 | 46.5 | 7.04e-08 | 2.81e-05 | lm.nb | Innate Immune Response, Microglia Function | NM\_021394.2:473 |
  | Irf1-mRNA | 2.25 | 0.201 | 1.86 | 2.64 | 4.76 | 3.62 | 6.25 | 1.02e-07 | 3.7e-05 | lm.nb | Apoptosis, Inflammatory Signaling, Innate Immune Response | NM\_008390.1:365 |
  | Opalin-mRNA | -2.22 | 0.203 | -2.62 | -1.82 | 0.215 | 0.163 | 0.283 | 1.38e-07 | 4.59e-05 | lm.nb | Oligodendrocyte Function | NM\_153520.1:201 |
  | Ifitm2-mRNA | 1.8 | 0.167 | 1.47 | 2.13 | 3.48 | 2.78 | 4.37 | 1.6e-07 | 4.92e-05 | lm.nb | Adaptive Immune Response, Inflammatory Signaling | NM\_030694.1:87 |
  | C4a-mRNA | 1.63 | 0.152 | 1.33 | 1.92 | 3.09 | 2.51 | 3.8 | 1.77e-07 | 5.06e-05 | lm.nb | Astrocyte Function | NM\_011413.2:56 |
  | Tgm2-mRNA | 2.15 | 0.218 | 1.73 | 2.58 | 4.45 | 3.31 | 5.99 | 4.07e-07 | 0.000109 | lm.nb | Autophagy, Microglia Function | NM\_009373.3:1260 |
  | Ctsf-mRNA | 0.546 | 0.0593 | 0.43 | 0.662 | 1.46 | 1.35 | 1.58 | 8.67e-07 | 0.000216 | loglinear | Adaptive Immune Response | NM\_019861.1:625 |
  | Ets2-mRNA | 0.499 | 0.0545 | 0.392 | 0.606 | 1.41 | 1.31 | 1.52 | 9.29e-07 | 0.000218 | loglinear | Cellular Stress, Growth Factor Signaling, Microglia Function | NM\_011809.2:3284 |
  | Cxcl10-mRNA | 6.62 | 0.741 | 5.17 | 8.07 | 98.6 | 36 | 270 | 1.18e-06 | 0.000256 | lm.nb | Astrocyte Function, Cytokine Signaling, Inflammatory Signaling, Innate Immune Response, Microglia Function | NM\_021274.1:115 |
  | Pik3r2-mRNA | -0.347 | 0.0389 | -0.423 | -0.27 | 0.786 | 0.746 | 0.829 | 1.22e-06 | 0.000256 | loglinear | Adaptive Immune Response, Angiogenesis, Apoptosis, Autophagy, Cytokine Signaling, Growth Factor Signaling, Innate Immune Response, Insulin Signaling | NM\_008841.2:230 |
  | Serping1-mRNA | 1.72 | 0.194 | 1.34 | 2.1 | 3.29 | 2.53 | 4.29 | 1.29e-06 | 0.000258 | lm.nb | Astrocyte Function | NM\_009776.3:1480 |

  ##### DE Results: DPI: 2 vs.CTRL

  Table presenting the most statistically significantly differentially expressed genes with the selected covariate. 'Estimated log fold-change' estimates a gene's differential expression. For categorical covariates, a gene is estimated to have 2^(log fold change) times its expression in baseline samples, holding all other variables in the analysis constant. If the covariate is continuous, for each unit increase in the selected covariate, a gene's expression is estimated to increase by 2^(log fold change)-fold, holding all other variables in the analysis constant. The 95% confidence interval for the log fold change is also presented, along with a p-value and an adjusted p-value or FDR if requested. Method column indicates the model used to estimate differential expression. Mixed negative binomial model uses the mle function to run the Wald test. The simplified negative binomial model uses the glm.nb function . The loglinear model uses the lm function.

- **Volcano Plot: DPI: 3 vs.CTRL  
  More Plot Information**

  ##### Volcano Plot: DPI: 3 vs.CTRL

  Volcano plot displaying each gene's -log10(p-value) and log2 fold change with the selected covariate. Highly statistically significant genes fall at the top of the plot above the horizontal lines, and highly differentially expressed genes fall to either side. Horizontal lines indicate various False Discovery Rate (FDR) thresholds or p-value thresholds if there is no adjustment to the p-values. Genes are colored if the resulting p-value is below the given FDR or p-value threshold. The 40 most statistically significant genes are labeled in the plot.
- **DE Results: DPI: 3 vs.CTRL  
  More Plot Information  Download CSV Data**

  |  | Log2 fold change | std error (log2) | Lower confidence limit (log2) | Upper confidence limit (log2) | Linear fold change | Lower confidence limit (linear) | Upper confidence limit (linear) | P-value | BY.p.value | method | Gene.sets | probe.ID |
  | --- | --- | --- | --- | --- | --- | --- | --- | --- | --- | --- | --- | --- |
  | Ifitm3-mRNA | 4.12 | 0.159 | 3.81 | 4.44 | 17.4 | 14 | 21.6 | 6.72e-12 | 2.69e-08 | lm.nb | Adaptive Immune Response, Inflammatory Signaling | NM\_025378.2:370 |
  | Gbp2-mRNA | 6.49 | 0.266 | 5.97 | 7.01 | 89.9 | 62.6 | 129 | 1.37e-11 | 2.73e-08 | lm.nb | Astrocyte Function, Inflammatory Signaling | NM\_010260.1:1996 |
  | Psmb8-mRNA | 4.35 | 0.191 | 3.98 | 4.73 | 20.4 | 15.7 | 26.5 | 3.09e-11 | 3.33e-08 | lm.nb | Adaptive Immune Response, Angiogenesis, Apoptosis, Astrocyte Function, Cell Cycle, Cytokine Signaling, Growth Factor Signaling, Inflammatory Signaling, Insulin Signaling, Microglia Function, NF-kB, Wnt | NM\_010724.2:362 |
  | Cp-mRNA | 3.43 | 0.152 | 3.14 | 3.73 | 10.8 | 8.79 | 13.3 | 3.4e-11 | 3.33e-08 | lm.nb | Astrocyte Function | NM\_001042611.1:1750 |
  | Lcn2-mRNA | 11.1 | 0.501 | 10.1 | 12.1 | 2230 | 1130 | 4400 | 4.16e-11 | 3.33e-08 | lm.nb | Astrocyte Function, Inflammatory Signaling | NM\_008491.1:190 |
  | C4a-mRNA | 2.97 | 0.148 | 2.68 | 3.26 | 7.83 | 6.4 | 9.58 | 1.4e-10 | 9.31e-08 | lm.nb | Astrocyte Function | NM\_011413.2:56 |
  | Irf1-mRNA | 3.51 | 0.198 | 3.12 | 3.9 | 11.4 | 8.72 | 14.9 | 5.78e-10 | 3.03e-07 | lm.nb | Apoptosis, Inflammatory Signaling, Innate Immune Response | NM\_008390.1:365 |
  | Fkbp5-mRNA | 2.19 | 0.124 | 1.95 | 2.43 | 4.56 | 3.86 | 5.4 | 6.07e-10 | 3.03e-07 | loglinear | Astrocyte Function | NM\_010220.3:2125 |
  | Ifitm2-mRNA | 2.82 | 0.165 | 2.49 | 3.14 | 7.04 | 5.62 | 8.81 | 9.04e-10 | 4.01e-07 | lm.nb | Adaptive Immune Response, Inflammatory Signaling | NM\_030694.1:87 |
  | Tgm2-mRNA | 3.3 | 0.214 | 2.88 | 3.72 | 9.83 | 7.35 | 13.1 | 2.85e-09 | 1.14e-06 | lm.nb | Autophagy, Microglia Function | NM\_009373.3:1260 |
  | Serping1-mRNA | 2.83 | 0.188 | 2.46 | 3.2 | 7.1 | 5.49 | 9.16 | 3.84e-09 | 1.39e-06 | lm.nb | Astrocyte Function | NM\_009776.3:1480 |
  | Cdkn1a-mRNA | 2.23 | 0.15 | 1.93 | 2.52 | 4.68 | 3.82 | 5.74 | 4.31e-09 | 1.43e-06 | lm.nb | Adaptive Immune Response, Cell Cycle, Cellular Stress, Cytokine Signaling, DNA Damage, Growth Factor Signaling | NM\_007669.4:1670 |
  | Serpina3n-mRNA | 4.42 | 0.304 | 3.83 | 5.02 | 21.5 | 14.2 | 32.4 | 5.44e-09 | 1.67e-06 | lm.nb | Astrocyte Function | NM\_009252.2:119 |
  | Zbp1-mRNA | 5.75 | 0.406 | 4.96 | 6.55 | 53.8 | 31 | 93.5 | 7.44e-09 | 2.12e-06 | lm.nb | Innate Immune Response, Microglia Function | NM\_021394.2:473 |
  | Ddx58-mRNA | 2.31 | 0.166 | 1.98 | 2.63 | 4.95 | 3.95 | 6.21 | 9.41e-09 | 2.44e-06 | loglinear | Inflammatory Signaling, Innate Immune Response, NF-kB | NM\_172689.3:1751 |
  | Homer1-mRNA | -0.834 | 0.0603 | -0.953 | -0.716 | 0.561 | 0.517 | 0.609 | 9.76e-09 | 2.44e-06 | loglinear | Neurons and Neurotransmission | NM\_147176.2:1165 |
  | Cd74-mRNA | 3.22 | 0.246 | 2.74 | 3.7 | 9.34 | 6.69 | 13 | 1.79e-08 | 4.22e-06 | lm.nb | Adaptive Immune Response, Inflammatory Signaling | NM\_001042605.1:391 |
  | Osmr-mRNA | 4.15 | 0.32 | 3.52 | 4.78 | 17.8 | 11.5 | 27.4 | 2.02e-08 | 4.49e-06 | lm.nb | Astrocyte Function, Cytokine Signaling, Growth Factor Signaling | NM\_011019.3:395 |
  | Ets2-mRNA | 0.703 | 0.0545 | 0.596 | 0.81 | 1.63 | 1.51 | 1.75 | 2.16e-08 | 4.54e-06 | loglinear | Cellular Stress, Growth Factor Signaling, Microglia Function | NM\_011809.2:3284 |
  | Cxcl10-mRNA | 9.44 | 0.739 | 7.99 | 10.9 | 693 | 254 | 1890 | 2.42e-08 | 4.84e-06 | lm.nb | Astrocyte Function, Cytokine Signaling, Inflammatory Signaling, Innate Immune Response, Microglia Function | NM\_021274.1:115 |

  ##### DE Results: DPI: 3 vs.CTRL

  Table presenting the most statistically significantly differentially expressed genes with the selected covariate. 'Estimated log fold-change' estimates a gene's differential expression. For categorical covariates, a gene is estimated to have 2^(log fold change) times its expression in baseline samples, holding all other variables in the analysis constant. If the covariate is continuous, for each unit increase in the selected covariate, a gene's expression is estimated to increase by 2^(log fold change)-fold, holding all other variables in the analysis constant. The 95% confidence interval for the log fold change is also presented, along with a p-value and an adjusted p-value or FDR if requested. Method column indicates the model used to estimate differential expression. Mixed negative binomial model uses the mle function to run the Wald test. The simplified negative binomial model uses the glm.nb function . The loglinear model uses the lm function.

The results of differential expression testing are summarized at the gene set level. Each gene set's most differentially expressed genes are identified, and the extent of differential expression in each gene set is summarized using a 'global significance score'.

Gene sets:

- Summary
- Adaptive Immune Response
- Angiogenesis
- Apoptosis
- Astrocyte Function
- Autophagy
- Carbohydrate Metabolism
- Cell Cycle
- Cellular Stress
- Cytokine Signaling
- DNA Damage
- Epigenetic Regulation
- Growth Factor Signaling
- Inflammatory Signaling
- Innate Immune Response
- Insulin Signaling
- Lipid Metabolism
- Matrix Remodeling
- Microglia Function
- Neurons and Neurotransmission
- NF-kB
- Notch
- Oligodendrocyte Function
- Wnt

- **Global Significance Scores  
  More Plot Information**

  ##### Global Significance Scores

  Heatmap displaying each sample's global significance scores. Global significance statistics measure the extent of differential expression of a gene set's genes with a covariate, ignoring whether each gene within the set is up- or down-regulated. Orange denotes gene sets whose genes exhibit extensive differential expression with the covariate, blue denotes gene sets with less differential expression.
- **Directed Global Significance Scores  
  More Plot Information**

  ##### Directed Global Significance Scores

  Heatmap displaying each sample's directed global significance scores. Directed global significance statistics measure the extent to which a gene set's genes are up- or down-regulated with the variable. Red denotes gene sets whose genes exhibit extensive over-expression with the covariate, blue denotes gene sets with extensive under-expression.

- **Undirected and Directed global significance scores table  
  More Plot Information  Download CSV Data**

  |  | Undirected DPI: differential expression in 1 vs. baseline of CTRL | Undirected DPI: differential expression in 2 vs. baseline of CTRL | Undirected DPI: differential expression in 3 vs. baseline of CTRL | Directed DPI: differential expression in 1 vs. baseline of CTRL | Directed DPI: differential expression in 2 vs. baseline of CTRL | Directed DPI: differential expression in 3 vs. baseline of CTRL |
  | --- | --- | --- | --- | --- | --- | --- |
  | Adaptive Immune Response | 1.696 | 4.225 | 6.12 | 0.974 | 3.192 | 5.275 |
  | Angiogenesis | 1.423 | 3.548 | 5.069 | 0.157 | 1.137 | 3.694 |
  | Apoptosis | 1.587 | 3.3 | 5.013 | 1.16 | 2.002 | 4.111 |
  | Astrocyte Function | 2.949 | 7.113 | 10.374 | 2.765 | 6.771 | 10.206 |
  | Autophagy | 1.682 | 3.114 | 4.683 | 1.007 | 1.496 | 3.475 |
  | Carbohydrate Metabolism | 1.149 | 1.691 | 2.244 | -0.622 | 0.423 | 1.663 |
  | Cell Cycle | 1.291 | 3.35 | 4.545 | 0.974 | 1.971 | 3.505 |
  | Cellular Stress | 1.582 | 3.146 | 3.999 | 1.235 | 2.368 | 3.344 |
  | Cytokine Signaling | 1.676 | 3.95 | 5.736 | 0.768 | 2.795 | 5.043 |
  | DNA Damage | 1.215 | 2.9 | 3.131 | 0.904 | 2.403 | 2.623 |
  | Epigenetic Regulation | 1.33 | 2.345 | 2.463 | 0.847 | -1.153 | -1.681 |
  | Growth Factor Signaling | 1.685 | 3.7 | 5.249 | 1.107 | 1.609 | 3.857 |
  | Inflammatory Signaling | 2.485 | 5.892 | 8.84 | 2.13 | 5.379 | 8.482 |
  | Innate Immune Response | 1.584 | 3.456 | 5.609 | 0.744 | 2.707 | 4.925 |
  | Insulin Signaling | 1.362 | 3.923 | 5.598 | -0.316 | 1.911 | 4.29 |
  | Lipid Metabolism | 1.087 | 2.907 | 3.35 | -0.596 | 0.188 | 2.035 |
  | Matrix Remodeling | 2.044 | 2.116 | 3.984 | -1.62 | 0.502 | 3.669 |
  | Microglia Function | 1.66 | 3.633 | 5.141 | 0.505 | 1.738 | 3.666 |
  | Neurons and Neurotransmission | 1.979 | 3.424 | 3.848 | -1.418 | -2.965 | -2.454 |
  | NF-kB | 1.654 | 3.752 | 6.412 | 1.26 | 3.424 | 6.248 |

  ##### Undirected and Directed global significance scores table

  Table displaying each sample's global significance scores and directed global significance scores as defined in the heatmaps above. The global significance score is calculated as the square root of the mean squared t-statistic for the genes in a gene set, with t-statistics coming from the linear regression underlying our differential expression analysis. The directed global significance score is calculated as the square root of the mean signed squared t-statistic for the genes in a gene set, with t-statistics coming from the linear regression underlying our differential expression analysis.

DPI:
- DPI: differential expression in 1 vs. baseline of CTRL
- DPI: differential expression in 2 vs. baseline of CTRL
- DPI: differential expression in 3 vs. baseline of CTRL

- **Volcano Plot: DPI: 1 vs.CTRL  
  More Plot Information**

  ##### Volcano Plot: DPI: 1 vs.CTRL

  Volcano plot displaying each gene's -log10(p-value) and log2 fold change for the selected covariate. Highly statistically significant genes fall at the top of the plot, and highly differentially expressed genes fall to either side. Genes within the selected gene set are highlighted in orange. Horizontal lines indicate various False Discovery Rate (FDR) thresholds.
- **DE Results - Adaptive Immune Response genes - DPI: 1 vs.CTRL  
  More Plot Information  Download CSV Data**

  | Probe Label | Log2 fold change | std error (log2) | Lower confidence limit (log2) | Upper confidence limit (log2) | Linear fold change | Lower confidence limit (linear) | Upper confidence limit (linear) | P-value | BY.p.value | method | Gene.sets | Codeset.Name | Probe.Label | Analyte.Type | Is.Control | Control.Type | Probe.Annotation | KEGG.Pathways | Cell.Type | Official.Gene.Name | volcanocol | volcanopch | tstats.all |
  | --- | --- | --- | --- | --- | --- | --- | --- | --- | --- | --- | --- | --- | --- | --- | --- | --- | --- | --- | --- | --- | --- | --- | --- |
  | Ifitm2-mRNA | 0.897 | 0.17 | 0.563 | 1.23 | 1.86 | 1.48 | 2.35 | 2e-04 | 0.114 | lm.nb | Adaptive Immune Response, Inflammatory Signaling | NS\_MM\_NEUROINFLAM\_V1.0 | Ifitm2 | mRNA | 0 |  | Inflammatory Signaling;Adaptive Immune Response |  |  | Ifitm2 | slateblue4 | 16 | 5.27647058823529 |
  | Ifitm3-mRNA | 0.83 | 0.163 | 0.511 | 1.15 | 1.78 | 1.42 | 2.22 | 0.000264 | 0.125 | lm.nb | Adaptive Immune Response, Inflammatory Signaling | NS\_MM\_NEUROINFLAM\_V1.0 | Ifitm3 | mRNA | 0 |  | Inflammatory Signaling;Adaptive Immune Response |  |  | Ifitm3 | slateblue4 | 16 | 5.0920245398773 |
  | Calr-mRNA | -0.485 | 0.129 | -0.739 | -0.231 | 0.715 | 0.599 | 0.852 | 0.0028 | 0.509 | lm.nb | Adaptive Immune Response, Autophagy, Neurons and Neurotransmission | NS\_MM\_NEUROINFLAM\_V1.0 | Calr | mRNA | 0 |  | Neurons and Neurotransmission;Autophagy;Adaptive Immune Response | mmu04141;mmu04145;mmu04612;mmu05142;mmu05166 |  | Calr | azure4 | 1 | -3.75968992248062 |
  | Nfkbia-mRNA | 0.866 | 0.242 | 0.392 | 1.34 | 1.82 | 1.31 | 2.53 | 0.00379 | 0.558 | lm.nb | Adaptive Immune Response, Apoptosis, Growth Factor Signaling, Inflammatory Signaling, Innate Immune Response, NF-kB | NS\_MM\_NEUROINFLAM\_V1.0 | Nfkbia | mRNA | 0 |  | NF-kB;Innate Immune Response;Inflammatory Signaling;Growth Factor Signaling;Apoptosis;Adaptive Immune Response | mmu04024;mmu04062;mmu04064;mmu04210;mmu04380;mmu04620;mmu04621;mmu04622;mmu04623;mmu04660;mmu04662;mmu04668;mmu04722;mmu04920;mmu04931;mmu05134;mmu05140;mmu05142;mmu05145;mmu05160;mmu05161;mmu05162;mmu05164;mmu05166;mmu05168;mmu05169;mmu05200;mmu05203;mmu05215;mmu05220;mmu05222 |  | Nfkbia | azure4 | 1 | 3.57851239669422 |
  | Ctss-mRNA | -0.633 | 0.178 | -0.981 | -0.285 | 0.645 | 0.507 | 0.821 | 0.00391 | 0.558 | lm.nb | Adaptive Immune Response, Innate Immune Response, Matrix Remodeling, Microglia Function | NS\_MM\_NEUROINFLAM\_V1.0 | Ctss | mRNA | 0 |  | Microglia Function;Matrix Remodeling;Innate Immune Response;Adaptive Immune Response | mmu04142;mmu04145;mmu04612;mmu05152 |  | Ctss | azure4 | 1 | -3.5561797752809 |
  | Braf-mRNA | 0.0984 | 0.0282 | 0.0432 | 0.154 | 1.07 | 1.03 | 1.11 | 0.00445 | 0.577 | loglinear | Adaptive Immune Response, Angiogenesis, Apoptosis, Cytokine Signaling, Growth Factor Signaling, Innate Immune Response, Insulin Signaling, Neurons and Neurotransmission | NS\_MM\_NEUROINFLAM\_V1.0 | Braf | mRNA | 0 |  | Neurons and Neurotransmission;Insulin Signaling;Innate Immune Response;Growth Factor Signaling;Cytokine Signaling;Apoptosis;Angiogenesis;Adaptive Immune Response | mmu04010;mmu04012;mmu04015;mmu04024;mmu04062;mmu04068;mmu04150;mmu04270;mmu04510;mmu04650;mmu04720;mmu04722;mmu04726;mmu04730;mmu04810;mmu04910;mmu04914;mmu05034;mmu05160;mmu05200;mmu05205;mmu05210;mmu05211;mmu05212;mmu05213;mmu05214;mmu05215;mmu05216;mmu05218;mmu05219;mmu05220;mmu05221;mmu05223 |  | Braf | azure4 | 1 | 3.48936170212766 |
  | Jun-mRNA | 0.323 | 0.0979 | 0.131 | 0.515 | 1.25 | 1.09 | 1.43 | 0.0064 | 0.711 | loglinear | Adaptive Immune Response, Apoptosis, Cellular Stress, Growth Factor Signaling, Innate Immune Response, Notch, Wnt | NS\_MM\_NEUROINFLAM\_V1.0 | Jun | mRNA | 0 |  | Wnt;Notch;Innate Immune Response;Growth Factor Signaling;Cellular Stress;Apoptosis;Adaptive Immune Response | mmu04010;mmu04012;mmu04024;mmu04310;mmu04380;mmu04510;mmu04620;mmu04660;mmu04662;mmu04668;mmu04722;mmu04912;mmu04915;mmu04921;mmu04932;mmu04933;mmu05030;mmu05031;mmu05132;mmu05133;mmu05140;mmu05142;mmu05161;mmu05164;mmu05166;mmu05168;mmu05169;mmu05200;mmu05203;mmu05210;mmu05211;mmu05231;mmu05321;mmu05323 |  | Jun | azure4 | 1 | 3.29928498467824 |
  | Fcgr2b-mRNA | 0.79 | 0.259 | 0.284 | 1.3 | 1.73 | 1.22 | 2.46 | 0.00996 | 0.861 | lm.nb | Adaptive Immune Response, Autophagy | NS\_MM\_NEUROINFLAM\_V1.0 | Fcgr2b | mRNA | 0 |  | Autophagy;Adaptive Immune Response | mmu04145;mmu04380;mmu04662;mmu04666;mmu05150;mmu05152;mmu05162 |  | Fcgr2b | azure4 | 1 | 3.05019305019305 |
  | Lgmn-mRNA | -0.262 | 0.0924 | -0.444 | -0.0813 | 0.834 | 0.735 | 0.945 | 0.0149 | 0.992 | loglinear | Adaptive Immune Response, Innate Immune Response | NS\_MM\_NEUROINFLAM\_V1.0 | Lgmn | mRNA | 0 |  | Innate Immune Response;Adaptive Immune Response | mmu04142;mmu04612 |  | Lgmn | azure4 | 1 | -2.83549783549784 |
  | Grin2b-mRNA | -0.218 | 0.0772 | -0.369 | -0.0665 | 0.86 | 0.774 | 0.955 | 0.0154 | 1 | loglinear | Adaptive Immune Response, Angiogenesis, Cytokine Signaling, Growth Factor Signaling, Insulin Signaling, Neurons and Neurotransmission | NS\_MM\_NEUROINFLAM\_V1.0 | Grin2b | mRNA | 0 |  | Neurons and Neurotransmission;Insulin Signaling;Growth Factor Signaling;Cytokine Signaling;Angiogenesis;Adaptive Immune Response | mmu04014;mmu04015;mmu04024;mmu04080;mmu04713;mmu04720;mmu04724;mmu04728;mmu05010;mmu05014;mmu05016;mmu05030;mmu05031;mmu05033;mmu05034;mmu05322 |  | Grin2b | azure4 | 1 | -2.82383419689119 |
  | Blnk-mRNA | -0.706 | 0.264 | -1.22 | -0.19 | 0.613 | 0.428 | 0.877 | 0.0201 | 1 | lm.nb | Adaptive Immune Response, Microglia Function, NF-kB | NS\_MM\_NEUROINFLAM\_V1.0 | Blnk | mRNA | 0 |  | NF-kB;Microglia Function;Adaptive Immune Response | mmu04064;mmu04380;mmu04662;mmu05340 |  | Blnk | azure4 | 1 | -2.67424242424242 |
  | Rab7-mRNA | 0.0707 | 0.0271 | 0.0176 | 0.124 | 1.05 | 1.01 | 1.09 | 0.0229 | 1 | loglinear | Adaptive Immune Response, Autophagy | NS\_MM\_NEUROINFLAM\_V1.0 | Rab7 | mRNA | 0 |  | Autophagy;Adaptive Immune Response | mmu04144;mmu04145;mmu05132;mmu05146;mmu05152 |  | Rab7 | azure4 | 1 | 2.60885608856089 |
  | Pik3r2-mRNA | -0.099 | 0.0389 | -0.175 | -0.0229 | 0.934 | 0.886 | 0.984 | 0.0256 | 1 | loglinear | Adaptive Immune Response, Angiogenesis, Apoptosis, Autophagy, Cytokine Signaling, Growth Factor Signaling, Innate Immune Response, Insulin Signaling | NS\_MM\_NEUROINFLAM\_V1.0 | Pik3r2 | mRNA | 0 |  | Insulin Signaling;Innate Immune Response;Growth Factor Signaling;Cytokine Signaling;Autophagy;Apoptosis;Angiogenesis;Adaptive Immune Response | mmu04012;mmu04014;mmu04015;mmu04022;mmu04024;mmu04062;mmu04066;mmu04068;mmu04070;mmu04071;mmu04072;mmu04150;mmu04151;mmu04152;mmu04210;mmu04211;mmu04213;mmu04261;mmu04370;mmu04380;mmu04510;mmu04550;mmu04611;mmu04620;mmu04630;mmu04650;mmu04660;mmu04662;mmu04664;mmu04666;mmu04668;mmu04670;mmu04722;mmu04725;mmu04750;mmu04810;mmu04910;mmu04914;mmu04915;mmu04917;mmu04919;mmu04921;mmu04923;mmu04930;mmu04931;mmu04932;mmu04933;mmu04960;mmu04973;mmu05100;mmu05142;mmu05145;mmu05146;mmu05160;mmu05161;mmu05162;mmu05164;mmu05166;mmu05169;mmu05200;mmu05203;mmu05205;mmu05210;mmu05211;mmu05212;mmu05213;mmu05214;mmu05215;mmu05218;mmu05220;mmu05221;mmu05222;mmu05223;mmu05230;mmu05231 |  | Pik3r2 | azure4 | 1 | -2.54498714652956 |
  | Lilrb4a-mRNA | 2.12 | 0.888 | 0.376 | 3.86 | 4.33 | 1.3 | 14.5 | 0.0346 | 1 | lm.nb | Adaptive Immune Response, Inflammatory Signaling | NS\_MM\_NEUROINFLAM\_V1.0 | Lilrb4a | mRNA | 0 |  | Inflammatory Signaling;Adaptive Immune Response |  |  | Lilrb4a | azure4 | 1 | 2.38738738738739 |
  | Cdkn1a-mRNA | 0.349 | 0.153 | 0.0486 | 0.649 | 1.27 | 1.03 | 1.57 | 0.0419 | 1 | lm.nb | Adaptive Immune Response, Cell Cycle, Cellular Stress, Cytokine Signaling, DNA Damage, Growth Factor Signaling | NS\_MM\_NEUROINFLAM\_V1.0 | Cdkn1a | mRNA | 0 |  | Growth Factor Signaling;DNA Damage;Cytokine Signaling;Cellular Stress;Cell Cycle;Adaptive Immune Response | mmu04012;mmu04066;mmu04068;mmu04110;mmu04115;mmu04151;mmu04630;mmu04921;mmu05160;mmu05161;mmu05166;mmu05169;mmu05200;mmu05202;mmu05203;mmu05205;mmu05206;mmu05214;mmu05215;mmu05218;mmu05219;mmu05220 |  | Cdkn1a | azure4 | 1 | 2.28104575163399 |
  | Casp9-mRNA | 0.284 | 0.132 | 0.025 | 0.544 | 1.22 | 1.02 | 1.46 | 0.0528 | 1 | loglinear | Adaptive Immune Response, Apoptosis, DNA Damage, Growth Factor Signaling, Innate Immune Response | NS\_MM\_NEUROINFLAM\_V1.0 | Casp9 | mRNA | 0 |  | Innate Immune Response;Growth Factor Signaling;DNA Damage;Apoptosis;Adaptive Immune Response | mmu04115;mmu04151;mmu04210;mmu04370;mmu04919;mmu05010;mmu05012;mmu05014;mmu05016;mmu05134;mmu05145;mmu05152;mmu05161;mmu05164;mmu05200;mmu05210;mmu05212;mmu05213;mmu05215;mmu05222;mmu05223;mmu05416 |  | Casp9 | azure4 | 1 | 2.15151515151515 |
  | Chuk-mRNA | -0.302 | 0.162 | -0.619 | 0.0144 | 0.811 | 0.651 | 1.01 | 0.0859 | 1 | lm.nb | Adaptive Immune Response, Apoptosis, Cytokine Signaling, Growth Factor Signaling, Inflammatory Signaling, Innate Immune Response, NF-kB | NS\_MM\_NEUROINFLAM\_V1.0 | Chuk | mRNA | 0 |  | NF-kB;Innate Immune Response;Inflammatory Signaling;Growth Factor Signaling;Cytokine Signaling;Apoptosis;Adaptive Immune Response | mmu04010;mmu04014;mmu04062;mmu04064;mmu04068;mmu04151;mmu04210;mmu04380;mmu04620;mmu04621;mmu04622;mmu04623;mmu04660;mmu04662;mmu04668;mmu04920;mmu05142;mmu05145;mmu05160;mmu05161;mmu05162;mmu05166;mmu05168;mmu05169;mmu05200;mmu05212;mmu05215;mmu05220;mmu05221;mmu05222 |  | Chuk | azure4 | 1 | -1.8641975308642 |
  | Tubb4a-mRNA | 0.183 | 0.098 | -0.00886 | 0.375 | 1.14 | 0.994 | 1.3 | 0.0861 | 1 | loglinear | Adaptive Immune Response, Apoptosis, Cell Cycle, Neurons and Neurotransmission | NS\_MM\_NEUROINFLAM\_V1.0 | Tubb4a | mRNA | 0 |  | Neurons and Neurotransmission;Cell Cycle;Apoptosis;Adaptive Immune Response | mmu04145;mmu04540 |  | Tubb4a | azure4 | 1 | 1.86734693877551 |
  | Cd74-mRNA | 0.468 | 0.259 | -0.0394 | 0.976 | 1.38 | 0.973 | 1.97 | 0.0957 | 1 | lm.nb | Adaptive Immune Response, Inflammatory Signaling | NS\_MM\_NEUROINFLAM\_V1.0 | Cd74 | mRNA | 0 |  | Inflammatory Signaling;Adaptive Immune Response | mmu04612;mmu05152;mmu05168 |  | Cd74 | azure4 | 1 | 1.80694980694981 |
  | Ctsf-mRNA | -0.103 | 0.0593 | -0.219 | 0.013 | 0.931 | 0.859 | 1.01 | 0.107 | 1 | loglinear | Adaptive Immune Response | NS\_MM\_NEUROINFLAM\_V1.0 | Ctsf | mRNA | 0 |  | Adaptive Immune Response | mmu04142 |  | Ctsf | azure4 | 1 | -1.73693086003373 |

  ##### DE Results - Adaptive Immune Response genes - DPI: 1 vs.CTRL

  Table displaying each sample's global significance scores and directed global significance scores as defined in the heatmaps above. The global significance score is calculated as the square root of the mean squared t-statistic for the genes in a gene set, with t-statistics coming from the linear regression underlying our differential expression analysis. The directed global significance score is calculated as the square root of the mean signed squared t-statistic for the genes in a gene set, with t-statistics coming from the linear regression underlying our differential expression analysis.

- **Volcano Plot: DPI: 2 vs.CTRL  
  More Plot Information**

  ##### Volcano Plot: DPI: 2 vs.CTRL

  Volcano plot displaying each gene's -log10(p-value) and log2 fold change for the selected covariate. Highly statistically significant genes fall at the top of the plot, and highly differentially expressed genes fall to either side. Genes within the selected gene set are highlighted in orange. Horizontal lines indicate various False Discovery Rate (FDR) thresholds.
- **DE Results - Adaptive Immune Response genes - DPI: 2 vs.CTRL  
  More Plot Information  Download CSV Data**

  | Probe Label | Log2 fold change | std error (log2) | Lower confidence limit (log2) | Upper confidence limit (log2) | Linear fold change | Lower confidence limit (linear) | Upper confidence limit (linear) | P-value | BY.p.value | method | Gene.sets | Codeset.Name | Probe.Label | Analyte.Type | Is.Control | Control.Type | Probe.Annotation | KEGG.Pathways | Cell.Type | Official.Gene.Name | volcanocol | volcanopch | tstats.all |
  | --- | --- | --- | --- | --- | --- | --- | --- | --- | --- | --- | --- | --- | --- | --- | --- | --- | --- | --- | --- | --- | --- | --- | --- |
  | Ifitm3-mRNA | 2.95 | 0.16 | 2.64 | 3.26 | 7.72 | 6.21 | 9.59 | 3.56e-10 | 4.74e-07 | lm.nb | Adaptive Immune Response, Inflammatory Signaling | NS\_MM\_NEUROINFLAM\_V1.0 | Ifitm3 | mRNA | 0 |  | Inflammatory Signaling;Adaptive Immune Response |  |  | Ifitm3 | slateblue1 | 16 | 18.4375 |
  | Cdkn1a-mRNA | 2.21 | 0.15 | 1.92 | 2.51 | 4.64 | 3.78 | 5.68 | 4.64e-09 | 3.71e-06 | lm.nb | Adaptive Immune Response, Cell Cycle, Cellular Stress, Cytokine Signaling, DNA Damage, Growth Factor Signaling | NS\_MM\_NEUROINFLAM\_V1.0 | Cdkn1a | mRNA | 0 |  | Growth Factor Signaling;DNA Damage;Cytokine Signaling;Cellular Stress;Cell Cycle;Adaptive Immune Response | mmu04012;mmu04066;mmu04068;mmu04110;mmu04115;mmu04151;mmu04630;mmu04921;mmu05160;mmu05161;mmu05166;mmu05169;mmu05200;mmu05202;mmu05203;mmu05205;mmu05206;mmu05214;mmu05215;mmu05218;mmu05219;mmu05220 |  | Cdkn1a | slateblue1 | 16 | 14.7333333333333 |
  | Psmb8-mRNA | 2.78 | 0.196 | 2.39 | 3.16 | 6.85 | 5.25 | 8.94 | 7.51e-09 | 4.29e-06 | lm.nb | Adaptive Immune Response, Angiogenesis, Apoptosis, Astrocyte Function, Cell Cycle, Cytokine Signaling, Growth Factor Signaling, Inflammatory Signaling, Insulin Signaling, Microglia Function, NF-kB, Wnt | NS\_MM\_NEUROINFLAM\_V1.0 | Psmb8 | mRNA | 0 |  | Wnt;NF-kB;Microglia Function;Insulin Signaling;Inflammatory Signaling;Growth Factor Signaling;Cytokine Signaling;Cell Cycle;Astrocyte Function;Apoptosis;Angiogenesis;Adaptive Immune Response |  |  | Psmb8 | slateblue1 | 16 | 14.1836734693878 |
  | Ifitm2-mRNA | 1.8 | 0.167 | 1.47 | 2.13 | 3.48 | 2.78 | 4.37 | 1.6e-07 | 4.92e-05 | lm.nb | Adaptive Immune Response, Inflammatory Signaling | NS\_MM\_NEUROINFLAM\_V1.0 | Ifitm2 | mRNA | 0 |  | Inflammatory Signaling;Adaptive Immune Response |  |  | Ifitm2 | slateblue1 | 16 | 10.7784431137725 |
  | Ctsf-mRNA | 0.546 | 0.0593 | 0.43 | 0.662 | 1.46 | 1.35 | 1.58 | 8.67e-07 | 0.000216 | loglinear | Adaptive Immune Response | NS\_MM\_NEUROINFLAM\_V1.0 | Ctsf | mRNA | 0 |  | Adaptive Immune Response | mmu04142 |  | Ctsf | slateblue1 | 16 | 9.20741989881956 |
  | Pik3r2-mRNA | -0.347 | 0.0389 | -0.423 | -0.27 | 0.786 | 0.746 | 0.829 | 1.22e-06 | 0.000256 | loglinear | Adaptive Immune Response, Angiogenesis, Apoptosis, Autophagy, Cytokine Signaling, Growth Factor Signaling, Innate Immune Response, Insulin Signaling | NS\_MM\_NEUROINFLAM\_V1.0 | Pik3r2 | mRNA | 0 |  | Insulin Signaling;Innate Immune Response;Growth Factor Signaling;Cytokine Signaling;Autophagy;Apoptosis;Angiogenesis;Adaptive Immune Response | mmu04012;mmu04014;mmu04015;mmu04022;mmu04024;mmu04062;mmu04066;mmu04068;mmu04070;mmu04071;mmu04072;mmu04150;mmu04151;mmu04152;mmu04210;mmu04211;mmu04213;mmu04261;mmu04370;mmu04380;mmu04510;mmu04550;mmu04611;mmu04620;mmu04630;mmu04650;mmu04660;mmu04662;mmu04664;mmu04666;mmu04668;mmu04670;mmu04722;mmu04725;mmu04750;mmu04810;mmu04910;mmu04914;mmu04915;mmu04917;mmu04919;mmu04921;mmu04923;mmu04930;mmu04931;mmu04932;mmu04933;mmu04960;mmu04973;mmu05100;mmu05142;mmu05145;mmu05146;mmu05160;mmu05161;mmu05162;mmu05164;mmu05166;mmu05169;mmu05200;mmu05203;mmu05205;mmu05210;mmu05211;mmu05212;mmu05213;mmu05214;mmu05215;mmu05218;mmu05220;mmu05221;mmu05222;mmu05223;mmu05230;mmu05231 |  | Pik3r2 | slateblue1 | 16 | -8.92030848329049 |
  | Tubb3-mRNA | -0.383 | 0.0494 | -0.48 | -0.286 | 0.767 | 0.717 | 0.82 | 5.22e-06 | 0.000718 | loglinear | Adaptive Immune Response, Apoptosis, Cell Cycle, Neurons and Neurotransmission | NS\_MM\_NEUROINFLAM\_V1.0 | Tubb3 | mRNA | 0 |  | Neurons and Neurotransmission;Cell Cycle;Apoptosis;Adaptive Immune Response | mmu04145;mmu04540 |  | Tubb3 | slateblue1 | 16 | -7.75303643724696 |
  | Nfkbia-mRNA | 1.42 | 0.238 | 0.952 | 1.88 | 2.67 | 1.93 | 3.69 | 6.55e-05 | 0.00556 | lm.nb | Adaptive Immune Response, Apoptosis, Growth Factor Signaling, Inflammatory Signaling, Innate Immune Response, NF-kB | NS\_MM\_NEUROINFLAM\_V1.0 | Nfkbia | mRNA | 0 |  | NF-kB;Innate Immune Response;Inflammatory Signaling;Growth Factor Signaling;Apoptosis;Adaptive Immune Response | mmu04024;mmu04062;mmu04064;mmu04210;mmu04380;mmu04620;mmu04621;mmu04622;mmu04623;mmu04660;mmu04662;mmu04668;mmu04722;mmu04920;mmu04931;mmu05134;mmu05140;mmu05142;mmu05145;mmu05160;mmu05161;mmu05162;mmu05164;mmu05166;mmu05168;mmu05169;mmu05200;mmu05203;mmu05215;mmu05220;mmu05222 |  | Nfkbia | slateblue1 | 16 | 5.96638655462185 |
  | Fcgr2b-mRNA | 1.48 | 0.253 | 0.983 | 1.98 | 2.79 | 1.98 | 3.93 | 7.95e-05 | 0.00599 | lm.nb | Adaptive Immune Response, Autophagy | NS\_MM\_NEUROINFLAM\_V1.0 | Fcgr2b | mRNA | 0 |  | Autophagy;Adaptive Immune Response | mmu04145;mmu04380;mmu04662;mmu04666;mmu05150;mmu05152;mmu05162 |  | Fcgr2b | slateblue1 | 16 | 5.8498023715415 |
  | Grin2b-mRNA | -0.381 | 0.0772 | -0.533 | -0.23 | 0.768 | 0.691 | 0.853 | 0.00034 | 0.02 | loglinear | Adaptive Immune Response, Angiogenesis, Cytokine Signaling, Growth Factor Signaling, Insulin Signaling, Neurons and Neurotransmission | NS\_MM\_NEUROINFLAM\_V1.0 | Grin2b | mRNA | 0 |  | Neurons and Neurotransmission;Insulin Signaling;Growth Factor Signaling;Cytokine Signaling;Angiogenesis;Adaptive Immune Response | mmu04014;mmu04015;mmu04024;mmu04080;mmu04713;mmu04720;mmu04724;mmu04728;mmu05010;mmu05014;mmu05016;mmu05030;mmu05031;mmu05033;mmu05034;mmu05322 |  | Grin2b | slateblue2 | 16 | -4.93523316062176 |
  | Ifi30-mRNA | 1.43 | 0.292 | 0.86 | 2 | 2.7 | 1.81 | 4.01 | 0.000362 | 0.0209 | lm.nb | Adaptive Immune Response, Inflammatory Signaling | NS\_MM\_NEUROINFLAM\_V1.0 | Ifi30 | mRNA | 0 |  | Inflammatory Signaling;Adaptive Immune Response | mmu04612 |  | Ifi30 | slateblue2 | 16 | 4.8972602739726 |
  | Lilrb4a-mRNA | 4.26 | 0.874 | 2.55 | 5.97 | 19.1 | 5.84 | 62.7 | 0.000383 | 0.0215 | lm.nb | Adaptive Immune Response, Inflammatory Signaling | NS\_MM\_NEUROINFLAM\_V1.0 | Lilrb4a | mRNA | 0 |  | Inflammatory Signaling;Adaptive Immune Response |  |  | Lilrb4a | slateblue2 | 16 | 4.87414187643021 |
  | Ctss-mRNA | 0.799 | 0.168 | 0.47 | 1.13 | 1.74 | 1.38 | 2.19 | 0.000473 | 0.0239 | lm.nb | Adaptive Immune Response, Innate Immune Response, Matrix Remodeling, Microglia Function | NS\_MM\_NEUROINFLAM\_V1.0 | Ctss | mRNA | 0 |  | Microglia Function;Matrix Remodeling;Innate Immune Response;Adaptive Immune Response | mmu04142;mmu04145;mmu04612;mmu05152 |  | Ctss | slateblue2 | 16 | 4.75595238095238 |
  | Rab7-mRNA | -0.119 | 0.0271 | -0.172 | -0.0655 | 0.921 | 0.888 | 0.956 | 0.000901 | 0.039 | loglinear | Adaptive Immune Response, Autophagy | NS\_MM\_NEUROINFLAM\_V1.0 | Rab7 | mRNA | 0 |  | Autophagy;Adaptive Immune Response | mmu04144;mmu04145;mmu05132;mmu05146;mmu05152 |  | Rab7 | slateblue2 | 16 | -4.39114391143911 |
  | Fcgr3-mRNA | 1.25 | 0.287 | 0.685 | 1.81 | 2.37 | 1.61 | 3.51 | 0.000959 | 0.0399 | lm.nb | Adaptive Immune Response, Autophagy, Inflammatory Signaling, Innate Immune Response | NS\_MM\_NEUROINFLAM\_V1.0 | Fcgr3 | mRNA | 0 |  | Innate Immune Response;Inflammatory Signaling;Autophagy;Adaptive Immune Response | mmu04145;mmu04380;mmu05140;mmu05150;mmu05152 |  | Fcgr3 | slateblue2 | 16 | 4.35540069686411 |
  | Prkcq-mRNA | -0.84 | 0.198 | -1.23 | -0.451 | 0.559 | 0.427 | 0.732 | 0.00116 | 0.0479 | lm.nb | Adaptive Immune Response, Apoptosis, Autophagy, NF-kB, Oligodendrocyte Function | NS\_MM\_NEUROINFLAM\_V1.0 | Prkcq | mRNA | 0 |  | Oligodendrocyte Function;NF-kB;Autophagy;Apoptosis;Adaptive Immune Response | mmu04064;mmu04270;mmu04530;mmu04660;mmu04750;mmu04920;mmu04931;mmu05162 | Oligodendrocytes | Prkcq | slateblue2 | 16 | -4.24242424242424 |
  | Calr-mRNA | -0.51 | 0.13 | -0.764 | -0.257 | 0.702 | 0.589 | 0.837 | 0.00196 | 0.0717 | lm.nb | Adaptive Immune Response, Autophagy, Neurons and Neurotransmission | NS\_MM\_NEUROINFLAM\_V1.0 | Calr | mRNA | 0 |  | Neurons and Neurotransmission;Autophagy;Adaptive Immune Response | mmu04141;mmu04145;mmu04612;mmu05142;mmu05166 |  | Calr | slateblue3 | 16 | -3.92307692307692 |
  | Pak1-mRNA | -0.131 | 0.0351 | -0.2 | -0.0621 | 0.913 | 0.871 | 0.958 | 0.0029 | 0.0964 | loglinear | Adaptive Immune Response, Angiogenesis, Growth Factor Signaling, Innate Immune Response, Microglia Function | NS\_MM\_NEUROINFLAM\_V1.0 | Pak1 | mRNA | 0 |  | Microglia Function;Innate Immune Response;Growth Factor Signaling;Angiogenesis;Adaptive Immune Response | mmu04010;mmu04012;mmu04014;mmu04024;mmu04062;mmu04360;mmu04510;mmu04650;mmu04660;mmu04666;mmu04810;mmu05205;mmu05211 |  | Pak1 | slateblue3 | 16 | -3.73219373219373 |
  | Dusp7-mRNA | -0.315 | 0.0861 | -0.484 | -0.147 | 0.804 | 0.715 | 0.903 | 0.00325 | 0.104 | loglinear | Adaptive Immune Response, Angiogenesis, Cytokine Signaling, Growth Factor Signaling, Innate Immune Response, Insulin Signaling, Microglia Function | NS\_MM\_NEUROINFLAM\_V1.0 | Dusp7 | mRNA | 0 |  | Microglia Function;Insulin Signaling;Innate Immune Response;Growth Factor Signaling;Cytokine Signaling;Angiogenesis;Adaptive Immune Response | mmu04010 |  | Dusp7 | slateblue4 | 16 | -3.65853658536585 |
  | Cd24a-mRNA | -0.546 | 0.155 | -0.85 | -0.242 | 0.685 | 0.555 | 0.845 | 0.00421 | 0.125 | loglinear | Adaptive Immune Response | NS\_MM\_NEUROINFLAM\_V1.0 | Cd24a | mRNA | 0 |  | Adaptive Immune Response | mmu04640 |  | Cd24a | slateblue4 | 16 | -3.52258064516129 |

  ##### DE Results - Adaptive Immune Response genes - DPI: 2 vs.CTRL

  Table displaying each sample's global significance scores and directed global significance scores as defined in the heatmaps above. The global significance score is calculated as the square root of the mean squared t-statistic for the genes in a gene set, with t-statistics coming from the linear regression underlying our differential expression analysis. The directed global significance score is calculated as the square root of the mean signed squared t-statistic for the genes in a gene set, with t-statistics coming from the linear regression underlying our differential expression analysis.

- **Volcano Plot: DPI: 3 vs.CTRL  
  More Plot Information**

  ##### Volcano Plot: DPI: 3 vs.CTRL

  Volcano plot displaying each gene's -log10(p-value) and log2 fold change for the selected covariate. Highly statistically significant genes fall at the top of the plot, and highly differentially expressed genes fall to either side. Genes within the selected gene set are highlighted in orange. Horizontal lines indicate various False Discovery Rate (FDR) thresholds.
- **DE Results - Adaptive Immune Response genes - DPI: 3 vs.CTRL  
  More Plot Information  Download CSV Data**

  | Probe Label | Log2 fold change | std error (log2) | Lower confidence limit (log2) | Upper confidence limit (log2) | Linear fold change | Lower confidence limit (linear) | Upper confidence limit (linear) | P-value | BY.p.value | method | Gene.sets | Codeset.Name | Probe.Label | Analyte.Type | Is.Control | Control.Type | Probe.Annotation | KEGG.Pathways | Cell.Type | Official.Gene.Name | volcanocol | volcanopch | tstats.all |
  | --- | --- | --- | --- | --- | --- | --- | --- | --- | --- | --- | --- | --- | --- | --- | --- | --- | --- | --- | --- | --- | --- | --- | --- |
  | Ifitm3-mRNA | 4.12 | 0.159 | 3.81 | 4.44 | 17.4 | 14 | 21.6 | 6.72e-12 | 2.69e-08 | lm.nb | Adaptive Immune Response, Inflammatory Signaling | NS\_MM\_NEUROINFLAM\_V1.0 | Ifitm3 | mRNA | 0 |  | Inflammatory Signaling;Adaptive Immune Response |  |  | Ifitm3 | slateblue1 | 16 | 25.9119496855346 |
  | Psmb8-mRNA | 4.35 | 0.191 | 3.98 | 4.73 | 20.4 | 15.7 | 26.5 | 3.09e-11 | 3.33e-08 | lm.nb | Adaptive Immune Response, Angiogenesis, Apoptosis, Astrocyte Function, Cell Cycle, Cytokine Signaling, Growth Factor Signaling, Inflammatory Signaling, Insulin Signaling, Microglia Function, NF-kB, Wnt | NS\_MM\_NEUROINFLAM\_V1.0 | Psmb8 | mRNA | 0 |  | Wnt;NF-kB;Microglia Function;Insulin Signaling;Inflammatory Signaling;Growth Factor Signaling;Cytokine Signaling;Cell Cycle;Astrocyte Function;Apoptosis;Angiogenesis;Adaptive Immune Response |  |  | Psmb8 | slateblue1 | 16 | 22.7748691099476 |
  | Ifitm2-mRNA | 2.82 | 0.165 | 2.49 | 3.14 | 7.04 | 5.62 | 8.81 | 9.04e-10 | 4.01e-07 | lm.nb | Adaptive Immune Response, Inflammatory Signaling | NS\_MM\_NEUROINFLAM\_V1.0 | Ifitm2 | mRNA | 0 |  | Inflammatory Signaling;Adaptive Immune Response |  |  | Ifitm2 | slateblue1 | 16 | 17.0909090909091 |
  | Cdkn1a-mRNA | 2.23 | 0.15 | 1.93 | 2.52 | 4.68 | 3.82 | 5.74 | 4.31e-09 | 1.43e-06 | lm.nb | Adaptive Immune Response, Cell Cycle, Cellular Stress, Cytokine Signaling, DNA Damage, Growth Factor Signaling | NS\_MM\_NEUROINFLAM\_V1.0 | Cdkn1a | mRNA | 0 |  | Growth Factor Signaling;DNA Damage;Cytokine Signaling;Cellular Stress;Cell Cycle;Adaptive Immune Response | mmu04012;mmu04066;mmu04068;mmu04110;mmu04115;mmu04151;mmu04630;mmu04921;mmu05160;mmu05161;mmu05166;mmu05169;mmu05200;mmu05202;mmu05203;mmu05205;mmu05206;mmu05214;mmu05215;mmu05218;mmu05219;mmu05220 |  | Cdkn1a | slateblue1 | 16 | 14.8666666666667 |
  | Cd74-mRNA | 3.22 | 0.246 | 2.74 | 3.7 | 9.34 | 6.69 | 13 | 1.79e-08 | 4.22e-06 | lm.nb | Adaptive Immune Response, Inflammatory Signaling | NS\_MM\_NEUROINFLAM\_V1.0 | Cd74 | mRNA | 0 |  | Inflammatory Signaling;Adaptive Immune Response | mmu04612;mmu05152;mmu05168 |  | Cd74 | slateblue1 | 16 | 13.0894308943089 |
  | Nfkbia-mRNA | 2.57 | 0.233 | 2.11 | 3.02 | 5.93 | 4.32 | 8.13 | 1.22e-07 | 1.74e-05 | lm.nb | Adaptive Immune Response, Apoptosis, Growth Factor Signaling, Inflammatory Signaling, Innate Immune Response, NF-kB | NS\_MM\_NEUROINFLAM\_V1.0 | Nfkbia | mRNA | 0 |  | NF-kB;Innate Immune Response;Inflammatory Signaling;Growth Factor Signaling;Apoptosis;Adaptive Immune Response | mmu04024;mmu04062;mmu04064;mmu04210;mmu04380;mmu04620;mmu04621;mmu04622;mmu04623;mmu04660;mmu04662;mmu04668;mmu04722;mmu04920;mmu04931;mmu05134;mmu05140;mmu05142;mmu05145;mmu05160;mmu05161;mmu05162;mmu05164;mmu05166;mmu05168;mmu05169;mmu05200;mmu05203;mmu05215;mmu05220;mmu05222 |  | Nfkbia | slateblue1 | 16 | 11.0300429184549 |
  | Fcgr2b-mRNA | 2.54 | 0.249 | 2.05 | 3.03 | 5.81 | 4.14 | 8.14 | 2.88e-07 | 3.6e-05 | lm.nb | Adaptive Immune Response, Autophagy | NS\_MM\_NEUROINFLAM\_V1.0 | Fcgr2b | mRNA | 0 |  | Autophagy;Adaptive Immune Response | mmu04145;mmu04380;mmu04662;mmu04666;mmu05150;mmu05152;mmu05162 |  | Fcgr2b | slateblue1 | 16 | 10.2008032128514 |
  | Pik3r2-mRNA | -0.374 | 0.0389 | -0.451 | -0.298 | 0.771 | 0.732 | 0.813 | 5.36e-07 | 5.35e-05 | loglinear | Adaptive Immune Response, Angiogenesis, Apoptosis, Autophagy, Cytokine Signaling, Growth Factor Signaling, Innate Immune Response, Insulin Signaling | NS\_MM\_NEUROINFLAM\_V1.0 | Pik3r2 | mRNA | 0 |  | Insulin Signaling;Innate Immune Response;Growth Factor Signaling;Cytokine Signaling;Autophagy;Apoptosis;Angiogenesis;Adaptive Immune Response | mmu04012;mmu04014;mmu04015;mmu04022;mmu04024;mmu04062;mmu04066;mmu04068;mmu04070;mmu04071;mmu04072;mmu04150;mmu04151;mmu04152;mmu04210;mmu04211;mmu04213;mmu04261;mmu04370;mmu04380;mmu04510;mmu04550;mmu04611;mmu04620;mmu04630;mmu04650;mmu04660;mmu04662;mmu04664;mmu04666;mmu04668;mmu04670;mmu04722;mmu04725;mmu04750;mmu04810;mmu04910;mmu04914;mmu04915;mmu04917;mmu04919;mmu04921;mmu04923;mmu04930;mmu04931;mmu04932;mmu04933;mmu04960;mmu04973;mmu05100;mmu05142;mmu05145;mmu05146;mmu05160;mmu05161;mmu05162;mmu05164;mmu05166;mmu05169;mmu05200;mmu05203;mmu05205;mmu05210;mmu05211;mmu05212;mmu05213;mmu05214;mmu05215;mmu05218;mmu05220;mmu05221;mmu05222;mmu05223;mmu05230;mmu05231 |  | Pik3r2 | slateblue1 | 16 | -9.61439588688946 |
  | Tubb3-mRNA | -0.448 | 0.0494 | -0.545 | -0.351 | 0.733 | 0.686 | 0.784 | 1.03e-06 | 8.78e-05 | loglinear | Adaptive Immune Response, Apoptosis, Cell Cycle, Neurons and Neurotransmission | NS\_MM\_NEUROINFLAM\_V1.0 | Tubb3 | mRNA | 0 |  | Neurons and Neurotransmission;Cell Cycle;Apoptosis;Adaptive Immune Response | mmu04145;mmu04540 |  | Tubb3 | slateblue1 | 16 | -9.06882591093117 |
  | Ctss-mRNA | 1.46 | 0.166 | 1.13 | 1.78 | 2.74 | 2.19 | 3.44 | 1.48e-06 | 0.000114 | lm.nb | Adaptive Immune Response, Innate Immune Response, Matrix Remodeling, Microglia Function | NS\_MM\_NEUROINFLAM\_V1.0 | Ctss | mRNA | 0 |  | Microglia Function;Matrix Remodeling;Innate Immune Response;Adaptive Immune Response | mmu04142;mmu04145;mmu04612;mmu05152 |  | Ctss | slateblue1 | 16 | 8.79518072289157 |
  | Ctsf-mRNA | 0.51 | 0.0593 | 0.393 | 0.626 | 1.42 | 1.31 | 1.54 | 1.79e-06 | 0.000132 | loglinear | Adaptive Immune Response | NS\_MM\_NEUROINFLAM\_V1.0 | Ctsf | mRNA | 0 |  | Adaptive Immune Response | mmu04142 |  | Ctsf | slateblue1 | 16 | 8.60033726812816 |
  | C3-mRNA | 5.35 | 0.633 | 4.11 | 6.59 | 40.8 | 17.2 | 96.3 | 2.14e-06 | 0.000149 | lm.nb | Adaptive Immune Response, Autophagy, Inflammatory Signaling, Innate Immune Response, Microglia Function | NS\_MM\_NEUROINFLAM\_V1.0 | C3 | mRNA | 0 |  | Microglia Function;Innate Immune Response;Inflammatory Signaling;Autophagy;Adaptive Immune Response | mmu04145;mmu04610;mmu05133;mmu05134;mmu05140;mmu05142;mmu05150;mmu05152;mmu05168;mmu05203;mmu05322 |  | C3 | slateblue1 | 16 | 8.45181674565561 |
  | Ifi30-mRNA | 2.21 | 0.287 | 1.65 | 2.77 | 4.63 | 3.14 | 6.84 | 5.43e-06 | 0.000319 | lm.nb | Adaptive Immune Response, Inflammatory Signaling | NS\_MM\_NEUROINFLAM\_V1.0 | Ifi30 | mRNA | 0 |  | Inflammatory Signaling;Adaptive Immune Response | mmu04612 |  | Ifi30 | slateblue1 | 16 | 7.70034843205575 |
  | Lilrb4a-mRNA | 6.53 | 0.87 | 4.82 | 8.23 | 92.2 | 28.3 | 301 | 7.25e-06 | 0.000402 | lm.nb | Adaptive Immune Response, Inflammatory Signaling | NS\_MM\_NEUROINFLAM\_V1.0 | Lilrb4a | mRNA | 0 |  | Inflammatory Signaling;Adaptive Immune Response |  |  | Lilrb4a | slateblue1 | 16 | 7.50574712643678 |
  | Fcgr3-mRNA | 1.9 | 0.282 | 1.35 | 2.46 | 3.74 | 2.55 | 5.49 | 2.05e-05 | 0.000986 | lm.nb | Adaptive Immune Response, Autophagy, Inflammatory Signaling, Innate Immune Response | NS\_MM\_NEUROINFLAM\_V1.0 | Fcgr3 | mRNA | 0 |  | Innate Immune Response;Inflammatory Signaling;Autophagy;Adaptive Immune Response | mmu04145;mmu04380;mmu05140;mmu05150;mmu05152 |  | Fcgr3 | slateblue1 | 16 | 6.73758865248227 |
  | Fcer1g-mRNA | 2.64 | 0.408 | 1.84 | 3.44 | 6.25 | 3.59 | 10.9 | 3.03e-05 | 0.00141 | lm.nb | Adaptive Immune Response, Autophagy, Inflammatory Signaling, Innate Immune Response | NS\_MM\_NEUROINFLAM\_V1.0 | Fcer1g | mRNA | 0 |  | Innate Immune Response;Inflammatory Signaling;Autophagy;Adaptive Immune Response | mmu04071;mmu04072;mmu04611;mmu04650;mmu04664;mmu05152;mmu05310 |  | Fcer1g | slateblue1 | 16 | 6.47058823529412 |
  | Pak1-mRNA | -0.221 | 0.0351 | -0.29 | -0.152 | 0.858 | 0.818 | 0.9 | 4.08e-05 | 0.00181 | loglinear | Adaptive Immune Response, Angiogenesis, Growth Factor Signaling, Innate Immune Response, Microglia Function | NS\_MM\_NEUROINFLAM\_V1.0 | Pak1 | mRNA | 0 |  | Microglia Function;Innate Immune Response;Growth Factor Signaling;Angiogenesis;Adaptive Immune Response | mmu04010;mmu04012;mmu04014;mmu04024;mmu04062;mmu04360;mmu04510;mmu04650;mmu04660;mmu04666;mmu04810;mmu05205;mmu05211 |  | Pak1 | slateblue1 | 16 | -6.2962962962963 |
  | H2-T23-mRNA | 2.12 | 0.359 | 1.42 | 2.82 | 4.35 | 2.67 | 7.08 | 7.29e-05 | 0.00285 | lm.nb | Adaptive Immune Response, Astrocyte Function, Inflammatory Signaling, Innate Immune Response, Matrix Remodeling | NS\_MM\_NEUROINFLAM\_V1.0 | H2-T23 | mRNA | 0 |  | Matrix Remodeling;Innate Immune Response;Inflammatory Signaling;Astrocyte Function;Adaptive Immune Response | mmu04144;mmu04145;mmu04514;mmu04612;mmu04650;mmu04940;mmu05166;mmu05168;mmu05169;mmu05203;mmu05320;mmu05330;mmu05332;mmu05416 |  | H2-T23 | slateblue1 | 16 | 5.90529247910864 |
  | Tubb4a-mRNA | -0.523 | 0.098 | -0.715 | -0.331 | 0.696 | 0.609 | 0.795 | 0.000177 | 0.00596 | loglinear | Adaptive Immune Response, Apoptosis, Cell Cycle, Neurons and Neurotransmission | NS\_MM\_NEUROINFLAM\_V1.0 | Tubb4a | mRNA | 0 |  | Neurons and Neurotransmission;Cell Cycle;Apoptosis;Adaptive Immune Response | mmu04145;mmu04540 |  | Tubb4a | slateblue1 | 16 | -5.33673469387755 |
  | Tyrobp-mRNA | 1.74 | 0.335 | 1.08 | 2.4 | 3.34 | 2.12 | 5.26 | 0.000221 | 0.00713 | lm.nb | Adaptive Immune Response, Innate Immune Response | NS\_MM\_NEUROINFLAM\_V1.0 | Tyrobp | mRNA | 0 |  | Innate Immune Response;Adaptive Immune Response | mmu04380;mmu04650 |  | Tyrobp | slateblue1 | 16 | 5.19402985074627 |

  ##### DE Results - Adaptive Immune Response genes - DPI: 3 vs.CTRL

  Table displaying each sample's global significance scores and directed global significance scores as defined in the heatmaps above. The global significance score is calculated as the square root of the mean squared t-statistic for the genes in a gene set, with t-statistics coming from the linear regression underlying our differential expression analysis. The directed global significance score is calculated as the square root of the mean signed squared t-statistic for the genes in a gene set, with t-statistics coming from the linear regression underlying our differential expression analysis.

DPI:
- DPI: differential expression in 1 vs. baseline of CTRL
- DPI: differential expression in 2 vs. baseline of CTRL
- DPI: differential expression in 3 vs. baseline of CTRL

- **Volcano Plot: DPI: 1 vs.CTRL  
  More Plot Information**

  ##### Volcano Plot: DPI: 1 vs.CTRL

  Volcano plot displaying each gene's -log10(p-value) and log2 fold change for the selected covariate. Highly statistically significant genes fall at the top of the plot, and highly differentially expressed genes fall to either side. Genes within the selected gene set are highlighted in orange. Horizontal lines indicate various False Discovery Rate (FDR) thresholds.
- **DE Results - Angiogenesis genes - DPI: 1 vs.CTRL  
  More Plot Information  Download CSV Data**

  | Probe Label | Log2 fold change | std error (log2) | Lower confidence limit (log2) | Upper confidence limit (log2) | Linear fold change | Lower confidence limit (linear) | Upper confidence limit (linear) | P-value | BY.p.value | method | Gene.sets | Codeset.Name | Probe.Label | Analyte.Type | Is.Control | Control.Type | Probe.Annotation | KEGG.Pathways | Cell.Type | Official.Gene.Name | volcanocol | volcanopch | tstats.all |
  | --- | --- | --- | --- | --- | --- | --- | --- | --- | --- | --- | --- | --- | --- | --- | --- | --- | --- | --- | --- | --- | --- | --- | --- |
  | Braf-mRNA | 0.0984 | 0.0282 | 0.0432 | 0.154 | 1.07 | 1.03 | 1.11 | 0.00445 | 0.577 | loglinear | Adaptive Immune Response, Angiogenesis, Apoptosis, Cytokine Signaling, Growth Factor Signaling, Innate Immune Response, Insulin Signaling, Neurons and Neurotransmission | NS\_MM\_NEUROINFLAM\_V1.0 | Braf | mRNA | 0 |  | Neurons and Neurotransmission;Insulin Signaling;Innate Immune Response;Growth Factor Signaling;Cytokine Signaling;Apoptosis;Angiogenesis;Adaptive Immune Response | mmu04010;mmu04012;mmu04015;mmu04024;mmu04062;mmu04068;mmu04150;mmu04270;mmu04510;mmu04650;mmu04720;mmu04722;mmu04726;mmu04730;mmu04810;mmu04910;mmu04914;mmu05034;mmu05160;mmu05200;mmu05205;mmu05210;mmu05211;mmu05212;mmu05213;mmu05214;mmu05215;mmu05216;mmu05218;mmu05219;mmu05220;mmu05221;mmu05223 |  | Braf | azure4 | 1 | 3.48936170212766 |
  | Plxdc2-mRNA | -0.365 | 0.106 | -0.572 | -0.158 | 0.776 | 0.673 | 0.896 | 0.00471 | 0.577 | loglinear | Angiogenesis | NS\_MM\_NEUROINFLAM\_V1.0 | Plxdc2 | mRNA | 0 |  | Angiogenesis |  |  | Plxdc2 | azure4 | 1 | -3.44339622641509 |
  | Grin2b-mRNA | -0.218 | 0.0772 | -0.369 | -0.0665 | 0.86 | 0.774 | 0.955 | 0.0154 | 1 | loglinear | Adaptive Immune Response, Angiogenesis, Cytokine Signaling, Growth Factor Signaling, Insulin Signaling, Neurons and Neurotransmission | NS\_MM\_NEUROINFLAM\_V1.0 | Grin2b | mRNA | 0 |  | Neurons and Neurotransmission;Insulin Signaling;Growth Factor Signaling;Cytokine Signaling;Angiogenesis;Adaptive Immune Response | mmu04014;mmu04015;mmu04024;mmu04080;mmu04713;mmu04720;mmu04724;mmu04728;mmu05010;mmu05014;mmu05016;mmu05030;mmu05031;mmu05033;mmu05034;mmu05322 |  | Grin2b | azure4 | 1 | -2.82383419689119 |
  | Rhoa-mRNA | 0.267 | 0.102 | 0.068 | 0.467 | 1.2 | 1.05 | 1.38 | 0.022 | 1 | loglinear | Angiogenesis, Autophagy, Growth Factor Signaling, Wnt | NS\_MM\_NEUROINFLAM\_V1.0 | Rhoa | mRNA | 0 |  | Wnt;Growth Factor Signaling;Autophagy;Angiogenesis | mmu04014;mmu04015;mmu04022;mmu04024;mmu04062;mmu04071;mmu04072;mmu04144;mmu04270;mmu04310;mmu04350;mmu04360;mmu04510;mmu04520;mmu04530;mmu04611;mmu04660;mmu04670;mmu04722;mmu04810;mmu04921;mmu05100;mmu05133;mmu05152;mmu05200;mmu05203;mmu05205;mmu05206;mmu05210 |  | Rhoa | azure4 | 1 | 2.61764705882353 |
  | Pik3r2-mRNA | -0.099 | 0.0389 | -0.175 | -0.0229 | 0.934 | 0.886 | 0.984 | 0.0256 | 1 | loglinear | Adaptive Immune Response, Angiogenesis, Apoptosis, Autophagy, Cytokine Signaling, Growth Factor Signaling, Innate Immune Response, Insulin Signaling | NS\_MM\_NEUROINFLAM\_V1.0 | Pik3r2 | mRNA | 0 |  | Insulin Signaling;Innate Immune Response;Growth Factor Signaling;Cytokine Signaling;Autophagy;Apoptosis;Angiogenesis;Adaptive Immune Response | mmu04012;mmu04014;mmu04015;mmu04022;mmu04024;mmu04062;mmu04066;mmu04068;mmu04070;mmu04071;mmu04072;mmu04150;mmu04151;mmu04152;mmu04210;mmu04211;mmu04213;mmu04261;mmu04370;mmu04380;mmu04510;mmu04550;mmu04611;mmu04620;mmu04630;mmu04650;mmu04660;mmu04662;mmu04664;mmu04666;mmu04668;mmu04670;mmu04722;mmu04725;mmu04750;mmu04810;mmu04910;mmu04914;mmu04915;mmu04917;mmu04919;mmu04921;mmu04923;mmu04930;mmu04931;mmu04932;mmu04933;mmu04960;mmu04973;mmu05100;mmu05142;mmu05145;mmu05146;mmu05160;mmu05161;mmu05162;mmu05164;mmu05166;mmu05169;mmu05200;mmu05203;mmu05205;mmu05210;mmu05211;mmu05212;mmu05213;mmu05214;mmu05215;mmu05218;mmu05220;mmu05221;mmu05222;mmu05223;mmu05230;mmu05231 |  | Pik3r2 | azure4 | 1 | -2.54498714652956 |
  | Pik3r1-mRNA | 0.15 | 0.0891 | -0.0245 | 0.325 | 1.11 | 0.983 | 1.25 | 0.118 | 1 | loglinear | Adaptive Immune Response, Angiogenesis, Apoptosis, Autophagy, Carbohydrate Metabolism, Cytokine Signaling, Growth Factor Signaling, Innate Immune Response, Insulin Signaling, Lipid Metabolism | NS\_MM\_NEUROINFLAM\_V1.0 | Pik3r1 | mRNA | 0 |  | Lipid Metabolism;Insulin Signaling;Innate Immune Response;Growth Factor Signaling;Cytokine Signaling;Carbohydrate Metabolism;Autophagy;Apoptosis;Angiogenesis;Adaptive Immune Response | mmu04012;mmu04014;mmu04015;mmu04022;mmu04024;mmu04062;mmu04066;mmu04068;mmu04070;mmu04071;mmu04072;mmu04150;mmu04151;mmu04152;mmu04210;mmu04211;mmu04213;mmu04261;mmu04370;mmu04380;mmu04510;mmu04550;mmu04611;mmu04620;mmu04630;mmu04650;mmu04660;mmu04662;mmu04664;mmu04666;mmu04668;mmu04670;mmu04722;mmu04725;mmu04750;mmu04810;mmu04910;mmu04914;mmu04915;mmu04917;mmu04919;mmu04921;mmu04923;mmu04930;mmu04931;mmu04932;mmu04933;mmu04960;mmu04973;mmu05100;mmu05142;mmu05145;mmu05146;mmu05160;mmu05161;mmu05162;mmu05164;mmu05166;mmu05169;mmu05200;mmu05203;mmu05205;mmu05210;mmu05211;mmu05212;mmu05213;mmu05214;mmu05215;mmu05218;mmu05220;mmu05221;mmu05222;mmu05223;mmu05230;mmu05231 |  | Pik3r1 | azure4 | 1 | 1.68350168350168 |
  | Psmb8-mRNA | 0.333 | 0.226 | -0.11 | 0.775 | 1.26 | 0.927 | 1.71 | 0.166 | 1 | lm.nb | Adaptive Immune Response, Angiogenesis, Apoptosis, Astrocyte Function, Cell Cycle, Cytokine Signaling, Growth Factor Signaling, Inflammatory Signaling, Insulin Signaling, Microglia Function, NF-kB, Wnt | NS\_MM\_NEUROINFLAM\_V1.0 | Psmb8 | mRNA | 0 |  | Wnt;NF-kB;Microglia Function;Insulin Signaling;Inflammatory Signaling;Growth Factor Signaling;Cytokine Signaling;Cell Cycle;Astrocyte Function;Apoptosis;Angiogenesis;Adaptive Immune Response |  |  | Psmb8 | azure4 | 1 | 1.47345132743363 |
  | Hspb1-mRNA | 0.338 | 0.236 | -0.124 | 0.801 | 1.26 | 0.918 | 1.74 | 0.177 | 1 | lm.nb | Angiogenesis, Astrocyte Function, Cellular Stress, Growth Factor Signaling | NS\_MM\_NEUROINFLAM\_V1.0 | Hspb1 | mRNA | 0 |  | Growth Factor Signaling;Cellular Stress;Astrocyte Function;Angiogenesis | mmu04010;mmu04370;mmu05146;mmu05169 |  | Hspb1 | azure4 | 1 | 1.43220338983051 |
  | Rac1-mRNA | 0.064 | 0.0461 | -0.0264 | 0.154 | 1.05 | 0.982 | 1.11 | 0.19 | 1 | loglinear | Adaptive Immune Response, Angiogenesis, Autophagy, Growth Factor Signaling, Innate Immune Response, Neurons and Neurotransmission, Wnt | NS\_MM\_NEUROINFLAM\_V1.0 | Rac1 | mRNA | 0 |  | Wnt;Neurons and Neurotransmission;Innate Immune Response;Growth Factor Signaling;Autophagy;Angiogenesis;Adaptive Immune Response | mmu04010;mmu04014;mmu04015;mmu04024;mmu04062;mmu04071;mmu04145;mmu04151;mmu04310;mmu04360;mmu04370;mmu04380;mmu04510;mmu04520;mmu04620;mmu04650;mmu04662;mmu04664;mmu04666;mmu04670;mmu04722;mmu04810;mmu04932;mmu04933;mmu05014;mmu05100;mmu05132;mmu05200;mmu05203;mmu05205;mmu05210;mmu05211;mmu05212;mmu05231;mmu05416 |  | Rac1 | azure4 | 1 | 1.3882863340564 |
  | Pak1-mRNA | -0.0458 | 0.0351 | -0.115 | 0.0231 | 0.969 | 0.924 | 1.02 | 0.217 | 1 | loglinear | Adaptive Immune Response, Angiogenesis, Growth Factor Signaling, Innate Immune Response, Microglia Function | NS\_MM\_NEUROINFLAM\_V1.0 | Pak1 | mRNA | 0 |  | Microglia Function;Innate Immune Response;Growth Factor Signaling;Angiogenesis;Adaptive Immune Response | mmu04010;mmu04012;mmu04014;mmu04024;mmu04062;mmu04360;mmu04510;mmu04650;mmu04660;mmu04666;mmu04810;mmu05205;mmu05211 |  | Pak1 | azure4 | 1 | -1.3048433048433 |
  | Grin2a-mRNA | -0.174 | 0.136 | -0.44 | 0.0922 | 0.886 | 0.737 | 1.07 | 0.224 | 1 | lm.nb | Adaptive Immune Response, Angiogenesis, Cytokine Signaling, Growth Factor Signaling, Insulin Signaling, Neurons and Neurotransmission | NS\_MM\_NEUROINFLAM\_V1.0 | Grin2a | mRNA | 0 |  | Neurons and Neurotransmission;Insulin Signaling;Growth Factor Signaling;Cytokine Signaling;Angiogenesis;Adaptive Immune Response | mmu04014;mmu04015;mmu04020;mmu04024;mmu04080;mmu04713;mmu04720;mmu04724;mmu04728;mmu05010;mmu05014;mmu05030;mmu05031;mmu05033;mmu05034;mmu05322 |  | Grin2a | azure4 | 1 | -1.27941176470588 |
  | Erbb3-mRNA | -0.62 | 0.518 | -1.64 | 0.396 | 0.651 | 0.322 | 1.32 | 0.255 | 1 | lm.nb | Adaptive Immune Response, Angiogenesis, Cytokine Signaling, Growth Factor Signaling, Insulin Signaling, Oligodendrocyte Function | NS\_MM\_NEUROINFLAM\_V1.0 | Erbb3 | mRNA | 0 |  | Oligodendrocyte Function;Insulin Signaling;Growth Factor Signaling;Cytokine Signaling;Angiogenesis;Adaptive Immune Response | mmu04012;mmu04020;mmu04144;mmu05205;mmu05206 | Oligodendrocytes | Erbb3 | azure4 | 1 | -1.1969111969112 |
  | Flt1-mRNA | -0.558 | 0.493 | -1.52 | 0.407 | 0.679 | 0.348 | 1.33 | 0.279 | 1 | lm.nb | Angiogenesis, Cytokine Signaling, Growth Factor Signaling, Microglia Function | NS\_MM\_NEUROINFLAM\_V1.0 | Flt1 | mRNA | 0 |  | Microglia Function;Growth Factor Signaling;Cytokine Signaling;Angiogenesis | mmu04014;mmu04015;mmu04060;mmu04066;mmu04144;mmu04151;mmu04510;mmu05202;mmu05323 | Endothelial Cells | Flt1 | azure4 | 1 | -1.13184584178499 |
  | Axl-mRNA | 0.221 | 0.201 | -0.172 | 0.615 | 1.17 | 0.887 | 1.53 | 0.292 | 1 | lm.nb | Angiogenesis, Autophagy, Microglia Function | NS\_MM\_NEUROINFLAM\_V1.0 | Axl | mRNA | 0 |  | Microglia Function;Autophagy;Angiogenesis |  |  | Axl | azure4 | 1 | 1.09950248756219 |
  | Prkacb-mRNA | 0.0484 | 0.0442 | -0.0382 | 0.135 | 1.03 | 0.974 | 1.1 | 0.295 | 1 | loglinear | Adaptive Immune Response, Angiogenesis, Apoptosis, Autophagy, Growth Factor Signaling, Lipid Metabolism, Neurons and Neurotransmission, Wnt | NS\_MM\_NEUROINFLAM\_V1.0 | Prkacb | mRNA | 0 |  | Wnt;Neurons and Neurotransmission;Lipid Metabolism;Growth Factor Signaling;Autophagy;Apoptosis;Angiogenesis;Adaptive Immune Response | mmu04010;mmu04014;mmu04020;mmu04024;mmu04062;mmu04114;mmu04210;mmu04211;mmu04213;mmu04261;mmu04270;mmu04310;mmu04540;mmu04611;mmu04713;mmu04720;mmu04723;mmu04724;mmu04725;mmu04726;mmu04728;mmu04750;mmu04910;mmu04911;mmu04912;mmu04913;mmu04914;mmu04915;mmu04916;mmu04919;mmu04921;mmu04922;mmu04923;mmu04924;mmu04925;mmu05012;mmu05020;mmu05030;mmu05031;mmu05146;mmu05166;mmu05169;mmu05200;mmu05203;mmu05205;mmu05414 |  | Prkacb | azure4 | 1 | 1.09502262443439 |
  | Pik3cb-mRNA | -0.0707 | 0.0676 | -0.203 | 0.0618 | 0.952 | 0.869 | 1.04 | 0.316 | 1 | loglinear | Adaptive Immune Response, Angiogenesis, Apoptosis, Autophagy, Carbohydrate Metabolism, Cytokine Signaling, Growth Factor Signaling, Innate Immune Response, Insulin Signaling, Lipid Metabolism | NS\_MM\_NEUROINFLAM\_V1.0 | Pik3cb | mRNA | 0 |  | Lipid Metabolism;Insulin Signaling;Innate Immune Response;Growth Factor Signaling;Cytokine Signaling;Carbohydrate Metabolism;Autophagy;Apoptosis;Angiogenesis;Adaptive Immune Response | mmu00562;mmu04012;mmu04014;mmu04015;mmu04022;mmu04024;mmu04062;mmu04066;mmu04068;mmu04070;mmu04071;mmu04072;mmu04150;mmu04151;mmu04152;mmu04210;mmu04211;mmu04213;mmu04261;mmu04370;mmu04380;mmu04510;mmu04550;mmu04611;mmu04620;mmu04630;mmu04650;mmu04660;mmu04662;mmu04664;mmu04666;mmu04668;mmu04670;mmu04722;mmu04725;mmu04750;mmu04810;mmu04910;mmu04914;mmu04915;mmu04917;mmu04919;mmu04921;mmu04923;mmu04930;mmu04931;mmu04932;mmu04933;mmu04960;mmu04973;mmu05100;mmu05142;mmu05145;mmu05146;mmu05160;mmu05161;mmu05162;mmu05164;mmu05166;mmu05169;mmu05200;mmu05203;mmu05205;mmu05210;mmu05211;mmu05212;mmu05213;mmu05214;mmu05215;mmu05218;mmu05220;mmu05221;mmu05222;mmu05223;mmu05230;mmu05231 |  | Pik3cb | azure4 | 1 | -1.04585798816568 |
  | Map2k1-mRNA | 0.037 | 0.0377 | -0.037 | 0.111 | 1.03 | 0.975 | 1.08 | 0.346 | 1 | loglinear | Adaptive Immune Response, Angiogenesis, Autophagy, Cytokine Signaling, Growth Factor Signaling, Innate Immune Response, Insulin Signaling, Microglia Function | NS\_MM\_NEUROINFLAM\_V1.0 | Map2k1 | mRNA | 0 |  | Microglia Function;Insulin Signaling;Innate Immune Response;Growth Factor Signaling;Cytokine Signaling;Autophagy;Angiogenesis;Adaptive Immune Response | mmu04010;mmu04012;mmu04014;mmu04015;mmu04022;mmu04024;mmu04062;mmu04066;mmu04068;mmu04071;mmu04072;mmu04114;mmu04151;mmu04270;mmu04370;mmu04380;mmu04510;mmu04540;mmu04550;mmu04620;mmu04650;mmu04660;mmu04662;mmu04664;mmu04666;mmu04668;mmu04720;mmu04722;mmu04725;mmu04726;mmu04730;mmu04810;mmu04910;mmu04912;mmu04914;mmu04915;mmu04916;mmu04917;mmu04919;mmu04921;mmu05020;mmu05034;mmu05161;mmu05164;mmu05200;mmu05205;mmu05206;mmu05210;mmu05211;mmu05212;mmu05213;mmu05214;mmu05215;mmu05216;mmu05218;mmu05219;mmu05220;mmu05221;mmu05223;mmu05230;mmu05231 |  | Map2k1 | azure4 | 1 | 0.981432360742706 |
  | Dusp7-mRNA | -0.0829 | 0.0861 | -0.252 | 0.0858 | 0.944 | 0.84 | 1.06 | 0.354 | 1 | loglinear | Adaptive Immune Response, Angiogenesis, Cytokine Signaling, Growth Factor Signaling, Innate Immune Response, Insulin Signaling, Microglia Function | NS\_MM\_NEUROINFLAM\_V1.0 | Dusp7 | mRNA | 0 |  | Microglia Function;Insulin Signaling;Innate Immune Response;Growth Factor Signaling;Cytokine Signaling;Angiogenesis;Adaptive Immune Response | mmu04010 |  | Dusp7 | azure4 | 1 | -0.962833914053426 |
  | Prkaca-mRNA | 0.0525 | 0.0605 | -0.066 | 0.171 | 1.04 | 0.955 | 1.13 | 0.402 | 1 | loglinear | Adaptive Immune Response, Angiogenesis, Apoptosis, Autophagy, Cell Cycle, Cytokine Signaling, Growth Factor Signaling, Lipid Metabolism, Neurons and Neurotransmission, Wnt | NS\_MM\_NEUROINFLAM\_V1.0 | Prkaca | mRNA | 0 |  | Wnt;Neurons and Neurotransmission;Lipid Metabolism;Growth Factor Signaling;Cytokine Signaling;Cell Cycle;Autophagy;Apoptosis;Angiogenesis;Adaptive Immune Response | mmu04010;mmu04014;mmu04020;mmu04024;mmu04062;mmu04114;mmu04210;mmu04211;mmu04213;mmu04261;mmu04270;mmu04310;mmu04540;mmu04611;mmu04713;mmu04720;mmu04723;mmu04724;mmu04725;mmu04726;mmu04728;mmu04750;mmu04910;mmu04911;mmu04912;mmu04913;mmu04914;mmu04915;mmu04916;mmu04919;mmu04921;mmu04922;mmu04923;mmu04924;mmu04925;mmu05012;mmu05020;mmu05030;mmu05031;mmu05034;mmu05146;mmu05166;mmu05169;mmu05200;mmu05203;mmu05205;mmu05414 |  | Prkaca | azure4 | 1 | 0.867768595041322 |
  | Nefl-mRNA | 0.124 | 0.154 | -0.178 | 0.425 | 1.09 | 0.884 | 1.34 | 0.437 | 1 | lm.nb | Adaptive Immune Response, Angiogenesis, Cytokine Signaling, Growth Factor Signaling, Insulin Signaling, Neurons and Neurotransmission | NS\_MM\_NEUROINFLAM\_V1.0 | Nefl | mRNA | 0 |  | Neurons and Neurotransmission;Insulin Signaling;Growth Factor Signaling;Cytokine Signaling;Angiogenesis;Adaptive Immune Response | mmu05014 |  | Nefl | azure4 | 1 | 0.805194805194805 |

  ##### DE Results - Angiogenesis genes - DPI: 1 vs.CTRL

  Table displaying each sample's global significance scores and directed global significance scores as defined in the heatmaps above. The global significance score is calculated as the square root of the mean squared t-statistic for the genes in a gene set, with t-statistics coming from the linear regression underlying our differential expression analysis. The directed global significance score is calculated as the square root of the mean signed squared t-statistic for the genes in a gene set, with t-statistics coming from the linear regression underlying our differential expression analysis.

- **Volcano Plot: DPI: 2 vs.CTRL  
  More Plot Information**

  ##### Volcano Plot: DPI: 2 vs.CTRL

  Volcano plot displaying each gene's -log10(p-value) and log2 fold change for the selected covariate. Highly statistically significant genes fall at the top of the plot, and highly differentially expressed genes fall to either side. Genes within the selected gene set are highlighted in orange. Horizontal lines indicate various False Discovery Rate (FDR) thresholds.
- **DE Results - Angiogenesis genes - DPI: 2 vs.CTRL  
  More Plot Information  Download CSV Data**

  | Probe Label | Log2 fold change | std error (log2) | Lower confidence limit (log2) | Upper confidence limit (log2) | Linear fold change | Lower confidence limit (linear) | Upper confidence limit (linear) | P-value | BY.p.value | method | Gene.sets | Codeset.Name | Probe.Label | Analyte.Type | Is.Control | Control.Type | Probe.Annotation | KEGG.Pathways | Cell.Type | Official.Gene.Name | volcanocol | volcanopch | tstats.all |
  | --- | --- | --- | --- | --- | --- | --- | --- | --- | --- | --- | --- | --- | --- | --- | --- | --- | --- | --- | --- | --- | --- | --- | --- |
  | Psmb8-mRNA | 2.78 | 0.196 | 2.39 | 3.16 | 6.85 | 5.25 | 8.94 | 7.51e-09 | 4.29e-06 | lm.nb | Adaptive Immune Response, Angiogenesis, Apoptosis, Astrocyte Function, Cell Cycle, Cytokine Signaling, Growth Factor Signaling, Inflammatory Signaling, Insulin Signaling, Microglia Function, NF-kB, Wnt | NS\_MM\_NEUROINFLAM\_V1.0 | Psmb8 | mRNA | 0 |  | Wnt;NF-kB;Microglia Function;Insulin Signaling;Inflammatory Signaling;Growth Factor Signaling;Cytokine Signaling;Cell Cycle;Astrocyte Function;Apoptosis;Angiogenesis;Adaptive Immune Response |  |  | Psmb8 | slateblue1 | 16 | 14.1836734693878 |
  | Pik3r2-mRNA | -0.347 | 0.0389 | -0.423 | -0.27 | 0.786 | 0.746 | 0.829 | 1.22e-06 | 0.000256 | loglinear | Adaptive Immune Response, Angiogenesis, Apoptosis, Autophagy, Cytokine Signaling, Growth Factor Signaling, Innate Immune Response, Insulin Signaling | NS\_MM\_NEUROINFLAM\_V1.0 | Pik3r2 | mRNA | 0 |  | Insulin Signaling;Innate Immune Response;Growth Factor Signaling;Cytokine Signaling;Autophagy;Apoptosis;Angiogenesis;Adaptive Immune Response | mmu04012;mmu04014;mmu04015;mmu04022;mmu04024;mmu04062;mmu04066;mmu04068;mmu04070;mmu04071;mmu04072;mmu04150;mmu04151;mmu04152;mmu04210;mmu04211;mmu04213;mmu04261;mmu04370;mmu04380;mmu04510;mmu04550;mmu04611;mmu04620;mmu04630;mmu04650;mmu04660;mmu04662;mmu04664;mmu04666;mmu04668;mmu04670;mmu04722;mmu04725;mmu04750;mmu04810;mmu04910;mmu04914;mmu04915;mmu04917;mmu04919;mmu04921;mmu04923;mmu04930;mmu04931;mmu04932;mmu04933;mmu04960;mmu04973;mmu05100;mmu05142;mmu05145;mmu05146;mmu05160;mmu05161;mmu05162;mmu05164;mmu05166;mmu05169;mmu05200;mmu05203;mmu05205;mmu05210;mmu05211;mmu05212;mmu05213;mmu05214;mmu05215;mmu05218;mmu05220;mmu05221;mmu05222;mmu05223;mmu05230;mmu05231 |  | Pik3r2 | slateblue1 | 16 | -8.92030848329049 |
  | Grin2b-mRNA | -0.381 | 0.0772 | -0.533 | -0.23 | 0.768 | 0.691 | 0.853 | 0.00034 | 0.02 | loglinear | Adaptive Immune Response, Angiogenesis, Cytokine Signaling, Growth Factor Signaling, Insulin Signaling, Neurons and Neurotransmission | NS\_MM\_NEUROINFLAM\_V1.0 | Grin2b | mRNA | 0 |  | Neurons and Neurotransmission;Insulin Signaling;Growth Factor Signaling;Cytokine Signaling;Angiogenesis;Adaptive Immune Response | mmu04014;mmu04015;mmu04024;mmu04080;mmu04713;mmu04720;mmu04724;mmu04728;mmu05010;mmu05014;mmu05016;mmu05030;mmu05031;mmu05033;mmu05034;mmu05322 |  | Grin2b | slateblue2 | 16 | -4.93523316062176 |
  | Dock1-mRNA | -0.39 | 0.101 | -0.588 | -0.191 | 0.763 | 0.665 | 0.876 | 0.00231 | 0.0803 | loglinear | Angiogenesis, Autophagy | NS\_MM\_NEUROINFLAM\_V1.0 | Dock1 | mRNA | 0 |  | Autophagy;Angiogenesis | mmu04510;mmu04810;mmu05100 |  | Dock1 | slateblue3 | 16 | -3.86138613861386 |
  | Hspb1-mRNA | 0.891 | 0.232 | 0.435 | 1.35 | 1.85 | 1.35 | 2.54 | 0.00238 | 0.082 | lm.nb | Angiogenesis, Astrocyte Function, Cellular Stress, Growth Factor Signaling | NS\_MM\_NEUROINFLAM\_V1.0 | Hspb1 | mRNA | 0 |  | Growth Factor Signaling;Cellular Stress;Astrocyte Function;Angiogenesis | mmu04010;mmu04370;mmu05146;mmu05169 |  | Hspb1 | slateblue3 | 16 | 3.84051724137931 |
  | Pak1-mRNA | -0.131 | 0.0351 | -0.2 | -0.0621 | 0.913 | 0.871 | 0.958 | 0.0029 | 0.0964 | loglinear | Adaptive Immune Response, Angiogenesis, Growth Factor Signaling, Innate Immune Response, Microglia Function | NS\_MM\_NEUROINFLAM\_V1.0 | Pak1 | mRNA | 0 |  | Microglia Function;Innate Immune Response;Growth Factor Signaling;Angiogenesis;Adaptive Immune Response | mmu04010;mmu04012;mmu04014;mmu04024;mmu04062;mmu04360;mmu04510;mmu04650;mmu04660;mmu04666;mmu04810;mmu05205;mmu05211 |  | Pak1 | slateblue3 | 16 | -3.73219373219373 |
  | Dusp7-mRNA | -0.315 | 0.0861 | -0.484 | -0.147 | 0.804 | 0.715 | 0.903 | 0.00325 | 0.104 | loglinear | Adaptive Immune Response, Angiogenesis, Cytokine Signaling, Growth Factor Signaling, Innate Immune Response, Insulin Signaling, Microglia Function | NS\_MM\_NEUROINFLAM\_V1.0 | Dusp7 | mRNA | 0 |  | Microglia Function;Insulin Signaling;Innate Immune Response;Growth Factor Signaling;Cytokine Signaling;Angiogenesis;Adaptive Immune Response | mmu04010 |  | Dusp7 | slateblue4 | 16 | -3.65853658536585 |
  | Plxdc2-mRNA | -0.384 | 0.106 | -0.591 | -0.177 | 0.766 | 0.664 | 0.884 | 0.00339 | 0.108 | loglinear | Angiogenesis | NS\_MM\_NEUROINFLAM\_V1.0 | Plxdc2 | mRNA | 0 |  | Angiogenesis |  |  | Plxdc2 | slateblue4 | 16 | -3.62264150943396 |
  | Akt2-mRNA | -0.687 | 0.21 | -1.1 | -0.276 | 0.621 | 0.467 | 0.826 | 0.00664 | 0.18 | lm.nb | Adaptive Immune Response, Angiogenesis, Apoptosis, Autophagy, Carbohydrate Metabolism, Cytokine Signaling, Growth Factor Signaling, Innate Immune Response, Insulin Signaling, Neurons and Neurotransmission, Wnt | NS\_MM\_NEUROINFLAM\_V1.0 | Akt2 | mRNA | 0 |  | Wnt;Neurons and Neurotransmission;Insulin Signaling;Innate Immune Response;Growth Factor Signaling;Cytokine Signaling;Carbohydrate Metabolism;Autophagy;Apoptosis;Angiogenesis;Adaptive Immune Response | mmu04010;mmu04012;mmu04014;mmu04015;mmu04022;mmu04024;mmu04062;mmu04066;mmu04068;mmu04071;mmu04072;mmu04150;mmu04151;mmu04152;mmu04210;mmu04211;mmu04213;mmu04261;mmu04370;mmu04380;mmu04510;mmu04530;mmu04550;mmu04611;mmu04620;mmu04630;mmu04660;mmu04662;mmu04664;mmu04666;mmu04668;mmu04722;mmu04725;mmu04728;mmu04910;mmu04914;mmu04915;mmu04917;mmu04919;mmu04920;mmu04922;mmu04923;mmu04931;mmu04932;mmu04933;mmu04973;mmu05142;mmu05145;mmu05152;mmu05160;mmu05161;mmu05162;mmu05164;mmu05166;mmu05169;mmu05200;mmu05205;mmu05210;mmu05211;mmu05212;mmu05213;mmu05214;mmu05215;mmu05218;mmu05220;mmu05221;mmu05222;mmu05223;mmu05230;mmu05231 |  | Akt2 | slateblue4 | 16 | -3.27142857142857 |
  | Dlg4-mRNA | -0.431 | 0.165 | -0.754 | -0.108 | 0.742 | 0.593 | 0.928 | 0.0227 | 0.48 | lm.nb | Adaptive Immune Response, Angiogenesis, Cytokine Signaling, Growth Factor Signaling, Insulin Signaling, Neurons and Neurotransmission | NS\_MM\_NEUROINFLAM\_V1.0 | Dlg4 | mRNA | 0 |  | Neurons and Neurotransmission;Insulin Signaling;Growth Factor Signaling;Cytokine Signaling;Angiogenesis;Adaptive Immune Response | mmu04390;mmu04724;mmu05016;mmu05030 |  | Dlg4 | slateblue4 | 16 | -2.61212121212121 |
  | Vegfa-mRNA | -0.325 | 0.124 | -0.569 | -0.0809 | 0.798 | 0.674 | 0.945 | 0.0228 | 0.48 | loglinear | Angiogenesis, Cellular Stress, Cytokine Signaling, Growth Factor Signaling, Microglia Function | NS\_MM\_NEUROINFLAM\_V1.0 | Vegfa | mRNA | 0 |  | Microglia Function;Growth Factor Signaling;Cytokine Signaling;Cellular Stress;Angiogenesis | mmu04014;mmu04015;mmu04060;mmu04066;mmu04150;mmu04151;mmu04370;mmu04510;mmu04933;mmu05200;mmu05205;mmu05206;mmu05211;mmu05212;mmu05219;mmu05323 |  | Vegfa | slateblue4 | 16 | -2.62096774193548 |
  | Fyn-mRNA | -0.146 | 0.0568 | -0.258 | -0.035 | 0.903 | 0.836 | 0.976 | 0.0242 | 0.495 | loglinear | Adaptive Immune Response, Angiogenesis, Autophagy, Cytokine Signaling, Growth Factor Signaling, Innate Immune Response, Insulin Signaling, Microglia Function | NS\_MM\_NEUROINFLAM\_V1.0 | Fyn | mRNA | 0 |  | Microglia Function;Insulin Signaling;Innate Immune Response;Growth Factor Signaling;Cytokine Signaling;Autophagy;Angiogenesis;Adaptive Immune Response | mmu04071;mmu04072;mmu04360;mmu04380;mmu04510;mmu04520;mmu04611;mmu04650;mmu04660;mmu04664;mmu04725;mmu05020;mmu05162;mmu05416 |  | Fyn | slateblue4 | 16 | -2.57042253521127 |
  | Rhoa-mRNA | 0.258 | 0.102 | 0.0584 | 0.457 | 1.2 | 1.04 | 1.37 | 0.0262 | 0.518 | loglinear | Angiogenesis, Autophagy, Growth Factor Signaling, Wnt | NS\_MM\_NEUROINFLAM\_V1.0 | Rhoa | mRNA | 0 |  | Wnt;Growth Factor Signaling;Autophagy;Angiogenesis | mmu04014;mmu04015;mmu04022;mmu04024;mmu04062;mmu04071;mmu04072;mmu04144;mmu04270;mmu04310;mmu04350;mmu04360;mmu04510;mmu04520;mmu04530;mmu04611;mmu04660;mmu04670;mmu04722;mmu04810;mmu04921;mmu05100;mmu05133;mmu05152;mmu05200;mmu05203;mmu05205;mmu05206;mmu05210 |  | Rhoa | azure4 | 1 | 2.52941176470588 |
  | Pik3r1-mRNA | 0.218 | 0.0891 | 0.043 | 0.392 | 1.16 | 1.03 | 1.31 | 0.031 | 0.577 | loglinear | Adaptive Immune Response, Angiogenesis, Apoptosis, Autophagy, Carbohydrate Metabolism, Cytokine Signaling, Growth Factor Signaling, Innate Immune Response, Insulin Signaling, Lipid Metabolism | NS\_MM\_NEUROINFLAM\_V1.0 | Pik3r1 | mRNA | 0 |  | Lipid Metabolism;Insulin Signaling;Innate Immune Response;Growth Factor Signaling;Cytokine Signaling;Carbohydrate Metabolism;Autophagy;Apoptosis;Angiogenesis;Adaptive Immune Response | mmu04012;mmu04014;mmu04015;mmu04022;mmu04024;mmu04062;mmu04066;mmu04068;mmu04070;mmu04071;mmu04072;mmu04150;mmu04151;mmu04152;mmu04210;mmu04211;mmu04213;mmu04261;mmu04370;mmu04380;mmu04510;mmu04550;mmu04611;mmu04620;mmu04630;mmu04650;mmu04660;mmu04662;mmu04664;mmu04666;mmu04668;mmu04670;mmu04722;mmu04725;mmu04750;mmu04810;mmu04910;mmu04914;mmu04915;mmu04917;mmu04919;mmu04921;mmu04923;mmu04930;mmu04931;mmu04932;mmu04933;mmu04960;mmu04973;mmu05100;mmu05142;mmu05145;mmu05146;mmu05160;mmu05161;mmu05162;mmu05164;mmu05166;mmu05169;mmu05200;mmu05203;mmu05205;mmu05210;mmu05211;mmu05212;mmu05213;mmu05214;mmu05215;mmu05218;mmu05220;mmu05221;mmu05222;mmu05223;mmu05230;mmu05231 |  | Pik3r1 | azure4 | 1 | 2.44668911335578 |
  | Ncf1-mRNA | 1.46 | 0.674 | 0.141 | 2.78 | 2.75 | 1.1 | 6.88 | 0.0509 | 0.857 | lm.nb | Adaptive Immune Response, Angiogenesis, Cellular Stress | NS\_MM\_NEUROINFLAM\_V1.0 | Ncf1 | mRNA | 0 |  | Cellular Stress;Angiogenesis;Adaptive Immune Response | mmu04062;mmu04145;mmu04380;mmu04666;mmu04670;mmu05140 | Microglia | Ncf1 | azure4 | 1 | 2.16617210682493 |
  | Pik3ca-mRNA | 0.153 | 0.0873 | -0.0183 | 0.324 | 1.11 | 0.987 | 1.25 | 0.106 | 1 | loglinear | Adaptive Immune Response, Angiogenesis, Apoptosis, Autophagy, Carbohydrate Metabolism, Cytokine Signaling, Growth Factor Signaling, Innate Immune Response, Insulin Signaling, Lipid Metabolism | NS\_MM\_NEUROINFLAM\_V1.0 | Pik3ca | mRNA | 0 |  | Lipid Metabolism;Insulin Signaling;Innate Immune Response;Growth Factor Signaling;Cytokine Signaling;Carbohydrate Metabolism;Autophagy;Apoptosis;Angiogenesis;Adaptive Immune Response | mmu00562;mmu04012;mmu04014;mmu04015;mmu04022;mmu04024;mmu04062;mmu04066;mmu04068;mmu04070;mmu04071;mmu04072;mmu04150;mmu04151;mmu04152;mmu04210;mmu04211;mmu04213;mmu04261;mmu04370;mmu04380;mmu04510;mmu04550;mmu04611;mmu04620;mmu04630;mmu04650;mmu04660;mmu04662;mmu04664;mmu04666;mmu04668;mmu04670;mmu04722;mmu04725;mmu04750;mmu04810;mmu04910;mmu04914;mmu04915;mmu04917;mmu04919;mmu04921;mmu04923;mmu04930;mmu04931;mmu04932;mmu04933;mmu04960;mmu04973;mmu05100;mmu05142;mmu05145;mmu05146;mmu05160;mmu05161;mmu05162;mmu05164;mmu05166;mmu05169;mmu05200;mmu05203;mmu05205;mmu05210;mmu05211;mmu05212;mmu05213;mmu05214;mmu05215;mmu05218;mmu05220;mmu05221;mmu05222;mmu05223;mmu05230;mmu05231 |  | Pik3ca | azure4 | 1 | 1.75257731958763 |
  | Mapk14-mRNA | 0.14 | 0.0867 | -0.0296 | 0.31 | 1.1 | 0.98 | 1.24 | 0.132 | 1 | loglinear | Angiogenesis, Autophagy, Cellular Stress, Growth Factor Signaling, Innate Immune Response | NS\_MM\_NEUROINFLAM\_V1.0 | Mapk14 | mRNA | 0 |  | Innate Immune Response;Growth Factor Signaling;Cellular Stress;Autophagy;Angiogenesis | mmu04010;mmu04015;mmu04068;mmu04071;mmu04261;mmu04370;mmu04380;mmu04550;mmu04611;mmu04620;mmu04621;mmu04622;mmu04660;mmu04664;mmu04668;mmu04670;mmu04722;mmu04723;mmu04728;mmu04750;mmu04912;mmu04914;mmu04917;mmu04933;mmu05014;mmu05132;mmu05133;mmu05140;mmu05142;mmu05145;mmu05152;mmu05160;mmu05164;mmu05169;mmu05205 |  | Mapk14 | azure4 | 1 | 1.61476355247982 |
  | Erbb3-mRNA | -0.841 | 0.52 | -1.86 | 0.179 | 0.558 | 0.275 | 1.13 | 0.132 | 1 | lm.nb | Adaptive Immune Response, Angiogenesis, Cytokine Signaling, Growth Factor Signaling, Insulin Signaling, Oligodendrocyte Function | NS\_MM\_NEUROINFLAM\_V1.0 | Erbb3 | mRNA | 0 |  | Oligodendrocyte Function;Insulin Signaling;Growth Factor Signaling;Cytokine Signaling;Angiogenesis;Adaptive Immune Response | mmu04012;mmu04020;mmu04144;mmu05205;mmu05206 | Oligodendrocytes | Erbb3 | azure4 | 1 | -1.61730769230769 |
  | Nrp2-mRNA | -0.223 | 0.19 | -0.595 | 0.15 | 0.857 | 0.662 | 1.11 | 0.264 | 1 | lm.nb | Angiogenesis, Microglia Function | NS\_MM\_NEUROINFLAM\_V1.0 | Nrp2 | mRNA | 0 |  | Microglia Function;Angiogenesis |  |  | Nrp2 | azure4 | 1 | -1.17368421052632 |
  | Nefl-mRNA | -0.155 | 0.154 | -0.457 | 0.147 | 0.898 | 0.729 | 1.11 | 0.334 | 1 | lm.nb | Adaptive Immune Response, Angiogenesis, Cytokine Signaling, Growth Factor Signaling, Insulin Signaling, Neurons and Neurotransmission | NS\_MM\_NEUROINFLAM\_V1.0 | Nefl | mRNA | 0 |  | Neurons and Neurotransmission;Insulin Signaling;Growth Factor Signaling;Cytokine Signaling;Angiogenesis;Adaptive Immune Response | mmu05014 |  | Nefl | azure4 | 1 | -1.00649350649351 |

  ##### DE Results - Angiogenesis genes - DPI: 2 vs.CTRL

  Table displaying each sample's global significance scores and directed global significance scores as defined in the heatmaps above. The global significance score is calculated as the square root of the mean squared t-statistic for the genes in a gene set, with t-statistics coming from the linear regression underlying our differential expression analysis. The directed global significance score is calculated as the square root of the mean signed squared t-statistic for the genes in a gene set, with t-statistics coming from the linear regression underlying our differential expression analysis.

- **Volcano Plot: DPI: 3 vs.CTRL  
  More Plot Information**

  ##### Volcano Plot: DPI: 3 vs.CTRL

  Volcano plot displaying each gene's -log10(p-value) and log2 fold change for the selected covariate. Highly statistically significant genes fall at the top of the plot, and highly differentially expressed genes fall to either side. Genes within the selected gene set are highlighted in orange. Horizontal lines indicate various False Discovery Rate (FDR) thresholds.
- **DE Results - Angiogenesis genes - DPI: 3 vs.CTRL  
  More Plot Information  Download CSV Data**

  | Probe Label | Log2 fold change | std error (log2) | Lower confidence limit (log2) | Upper confidence limit (log2) | Linear fold change | Lower confidence limit (linear) | Upper confidence limit (linear) | P-value | BY.p.value | method | Gene.sets | Codeset.Name | Probe.Label | Analyte.Type | Is.Control | Control.Type | Probe.Annotation | KEGG.Pathways | Cell.Type | Official.Gene.Name | volcanocol | volcanopch | tstats.all |
  | --- | --- | --- | --- | --- | --- | --- | --- | --- | --- | --- | --- | --- | --- | --- | --- | --- | --- | --- | --- | --- | --- | --- | --- |
  | Psmb8-mRNA | 4.35 | 0.191 | 3.98 | 4.73 | 20.4 | 15.7 | 26.5 | 3.09e-11 | 3.33e-08 | lm.nb | Adaptive Immune Response, Angiogenesis, Apoptosis, Astrocyte Function, Cell Cycle, Cytokine Signaling, Growth Factor Signaling, Inflammatory Signaling, Insulin Signaling, Microglia Function, NF-kB, Wnt | NS\_MM\_NEUROINFLAM\_V1.0 | Psmb8 | mRNA | 0 |  | Wnt;NF-kB;Microglia Function;Insulin Signaling;Inflammatory Signaling;Growth Factor Signaling;Cytokine Signaling;Cell Cycle;Astrocyte Function;Apoptosis;Angiogenesis;Adaptive Immune Response |  |  | Psmb8 | slateblue1 | 16 | 22.7748691099476 |
  | Hspb1-mRNA | 2.5 | 0.227 | 2.05 | 2.95 | 5.66 | 4.15 | 7.7 | 1.27e-07 | 1.76e-05 | lm.nb | Angiogenesis, Astrocyte Function, Cellular Stress, Growth Factor Signaling | NS\_MM\_NEUROINFLAM\_V1.0 | Hspb1 | mRNA | 0 |  | Growth Factor Signaling;Cellular Stress;Astrocyte Function;Angiogenesis | mmu04010;mmu04370;mmu05146;mmu05169 |  | Hspb1 | slateblue1 | 16 | 11.0132158590308 |
  | Pik3r2-mRNA | -0.374 | 0.0389 | -0.451 | -0.298 | 0.771 | 0.732 | 0.813 | 5.36e-07 | 5.35e-05 | loglinear | Adaptive Immune Response, Angiogenesis, Apoptosis, Autophagy, Cytokine Signaling, Growth Factor Signaling, Innate Immune Response, Insulin Signaling | NS\_MM\_NEUROINFLAM\_V1.0 | Pik3r2 | mRNA | 0 |  | Insulin Signaling;Innate Immune Response;Growth Factor Signaling;Cytokine Signaling;Autophagy;Apoptosis;Angiogenesis;Adaptive Immune Response | mmu04012;mmu04014;mmu04015;mmu04022;mmu04024;mmu04062;mmu04066;mmu04068;mmu04070;mmu04071;mmu04072;mmu04150;mmu04151;mmu04152;mmu04210;mmu04211;mmu04213;mmu04261;mmu04370;mmu04380;mmu04510;mmu04550;mmu04611;mmu04620;mmu04630;mmu04650;mmu04660;mmu04662;mmu04664;mmu04666;mmu04668;mmu04670;mmu04722;mmu04725;mmu04750;mmu04810;mmu04910;mmu04914;mmu04915;mmu04917;mmu04919;mmu04921;mmu04923;mmu04930;mmu04931;mmu04932;mmu04933;mmu04960;mmu04973;mmu05100;mmu05142;mmu05145;mmu05146;mmu05160;mmu05161;mmu05162;mmu05164;mmu05166;mmu05169;mmu05200;mmu05203;mmu05205;mmu05210;mmu05211;mmu05212;mmu05213;mmu05214;mmu05215;mmu05218;mmu05220;mmu05221;mmu05222;mmu05223;mmu05230;mmu05231 |  | Pik3r2 | slateblue1 | 16 | -9.61439588688946 |
  | Pak1-mRNA | -0.221 | 0.0351 | -0.29 | -0.152 | 0.858 | 0.818 | 0.9 | 4.08e-05 | 0.00181 | loglinear | Adaptive Immune Response, Angiogenesis, Growth Factor Signaling, Innate Immune Response, Microglia Function | NS\_MM\_NEUROINFLAM\_V1.0 | Pak1 | mRNA | 0 |  | Microglia Function;Innate Immune Response;Growth Factor Signaling;Angiogenesis;Adaptive Immune Response | mmu04010;mmu04012;mmu04014;mmu04024;mmu04062;mmu04360;mmu04510;mmu04650;mmu04660;mmu04666;mmu04810;mmu05205;mmu05211 |  | Pak1 | slateblue1 | 16 | -6.2962962962963 |
  | Ncf1-mRNA | 3.09 | 0.668 | 1.78 | 4.4 | 8.52 | 3.44 | 21.1 | 0.000586 | 0.0167 | lm.nb | Adaptive Immune Response, Angiogenesis, Cellular Stress | NS\_MM\_NEUROINFLAM\_V1.0 | Ncf1 | mRNA | 0 |  | Cellular Stress;Angiogenesis;Adaptive Immune Response | mmu04062;mmu04145;mmu04380;mmu04666;mmu04670;mmu05140 | Microglia | Ncf1 | slateblue2 | 16 | 4.62574850299401 |
  | Fyn-mRNA | -0.238 | 0.0568 | -0.349 | -0.126 | 0.848 | 0.785 | 0.916 | 0.00126 | 0.0312 | loglinear | Adaptive Immune Response, Angiogenesis, Autophagy, Cytokine Signaling, Growth Factor Signaling, Innate Immune Response, Insulin Signaling, Microglia Function | NS\_MM\_NEUROINFLAM\_V1.0 | Fyn | mRNA | 0 |  | Microglia Function;Insulin Signaling;Innate Immune Response;Growth Factor Signaling;Cytokine Signaling;Autophagy;Angiogenesis;Adaptive Immune Response | mmu04071;mmu04072;mmu04360;mmu04380;mmu04510;mmu04520;mmu04611;mmu04650;mmu04660;mmu04664;mmu04725;mmu05020;mmu05162;mmu05416 |  | Fyn | slateblue2 | 16 | -4.19014084507042 |
  | Dlg4-mRNA | -0.5 | 0.165 | -0.823 | -0.177 | 0.707 | 0.565 | 0.885 | 0.0105 | 0.188 | lm.nb | Adaptive Immune Response, Angiogenesis, Cytokine Signaling, Growth Factor Signaling, Insulin Signaling, Neurons and Neurotransmission | NS\_MM\_NEUROINFLAM\_V1.0 | Dlg4 | mRNA | 0 |  | Neurons and Neurotransmission;Insulin Signaling;Growth Factor Signaling;Cytokine Signaling;Angiogenesis;Adaptive Immune Response | mmu04390;mmu04724;mmu05016;mmu05030 |  | Dlg4 | slateblue4 | 16 | -3.03030303030303 |
  | Vegfa-mRNA | -0.374 | 0.124 | -0.618 | -0.13 | 0.772 | 0.652 | 0.914 | 0.011 | 0.193 | loglinear | Angiogenesis, Cellular Stress, Cytokine Signaling, Growth Factor Signaling, Microglia Function | NS\_MM\_NEUROINFLAM\_V1.0 | Vegfa | mRNA | 0 |  | Microglia Function;Growth Factor Signaling;Cytokine Signaling;Cellular Stress;Angiogenesis | mmu04014;mmu04015;mmu04060;mmu04066;mmu04150;mmu04151;mmu04370;mmu04510;mmu04933;mmu05200;mmu05205;mmu05206;mmu05211;mmu05212;mmu05219;mmu05323 |  | Vegfa | slateblue4 | 16 | -3.01612903225806 |
  | Dusp7-mRNA | -0.246 | 0.0861 | -0.415 | -0.0773 | 0.843 | 0.75 | 0.948 | 0.0144 | 0.244 | loglinear | Adaptive Immune Response, Angiogenesis, Cytokine Signaling, Growth Factor Signaling, Innate Immune Response, Insulin Signaling, Microglia Function | NS\_MM\_NEUROINFLAM\_V1.0 | Dusp7 | mRNA | 0 |  | Microglia Function;Insulin Signaling;Innate Immune Response;Growth Factor Signaling;Cytokine Signaling;Angiogenesis;Adaptive Immune Response | mmu04010 |  | Dusp7 | slateblue4 | 16 | -2.85714285714286 |
  | Vav1-mRNA | 1.49 | 0.55 | 0.415 | 2.57 | 2.81 | 1.33 | 5.94 | 0.0188 | 0.303 | lm.nb | Adaptive Immune Response, Angiogenesis, Autophagy, Cytokine Signaling, Growth Factor Signaling, Innate Immune Response | NS\_MM\_NEUROINFLAM\_V1.0 | Vav1 | mRNA | 0 |  | Innate Immune Response;Growth Factor Signaling;Cytokine Signaling;Autophagy;Angiogenesis;Adaptive Immune Response | mmu04024;mmu04062;mmu04510;mmu04650;mmu04660;mmu04662;mmu04664;mmu04666;mmu04670;mmu04810 |  | Vav1 | slateblue4 | 16 | 2.70909090909091 |
  | Pik3r1-mRNA | 0.238 | 0.0891 | 0.0639 | 0.413 | 1.18 | 1.05 | 1.33 | 0.0201 | 0.319 | loglinear | Adaptive Immune Response, Angiogenesis, Apoptosis, Autophagy, Carbohydrate Metabolism, Cytokine Signaling, Growth Factor Signaling, Innate Immune Response, Insulin Signaling, Lipid Metabolism | NS\_MM\_NEUROINFLAM\_V1.0 | Pik3r1 | mRNA | 0 |  | Lipid Metabolism;Insulin Signaling;Innate Immune Response;Growth Factor Signaling;Cytokine Signaling;Carbohydrate Metabolism;Autophagy;Apoptosis;Angiogenesis;Adaptive Immune Response | mmu04012;mmu04014;mmu04015;mmu04022;mmu04024;mmu04062;mmu04066;mmu04068;mmu04070;mmu04071;mmu04072;mmu04150;mmu04151;mmu04152;mmu04210;mmu04211;mmu04213;mmu04261;mmu04370;mmu04380;mmu04510;mmu04550;mmu04611;mmu04620;mmu04630;mmu04650;mmu04660;mmu04662;mmu04664;mmu04666;mmu04668;mmu04670;mmu04722;mmu04725;mmu04750;mmu04810;mmu04910;mmu04914;mmu04915;mmu04917;mmu04919;mmu04921;mmu04923;mmu04930;mmu04931;mmu04932;mmu04933;mmu04960;mmu04973;mmu05100;mmu05142;mmu05145;mmu05146;mmu05160;mmu05161;mmu05162;mmu05164;mmu05166;mmu05169;mmu05200;mmu05203;mmu05205;mmu05210;mmu05211;mmu05212;mmu05213;mmu05214;mmu05215;mmu05218;mmu05220;mmu05221;mmu05222;mmu05223;mmu05230;mmu05231 |  | Pik3r1 | slateblue4 | 16 | 2.67115600448934 |
  | Plxdc2-mRNA | -0.248 | 0.106 | -0.455 | -0.0409 | 0.842 | 0.73 | 0.972 | 0.0369 | 0.526 | loglinear | Angiogenesis | NS\_MM\_NEUROINFLAM\_V1.0 | Plxdc2 | mRNA | 0 |  | Angiogenesis |  |  | Plxdc2 | azure4 | 1 | -2.33962264150943 |
  | Map2k1-mRNA | -0.0853 | 0.0377 | -0.159 | -0.0114 | 0.943 | 0.895 | 0.992 | 0.0431 | 0.581 | loglinear | Adaptive Immune Response, Angiogenesis, Autophagy, Cytokine Signaling, Growth Factor Signaling, Innate Immune Response, Insulin Signaling, Microglia Function | NS\_MM\_NEUROINFLAM\_V1.0 | Map2k1 | mRNA | 0 |  | Microglia Function;Insulin Signaling;Innate Immune Response;Growth Factor Signaling;Cytokine Signaling;Autophagy;Angiogenesis;Adaptive Immune Response | mmu04010;mmu04012;mmu04014;mmu04015;mmu04022;mmu04024;mmu04062;mmu04066;mmu04068;mmu04071;mmu04072;mmu04114;mmu04151;mmu04270;mmu04370;mmu04380;mmu04510;mmu04540;mmu04550;mmu04620;mmu04650;mmu04660;mmu04662;mmu04664;mmu04666;mmu04668;mmu04720;mmu04722;mmu04725;mmu04726;mmu04730;mmu04810;mmu04910;mmu04912;mmu04914;mmu04915;mmu04916;mmu04917;mmu04919;mmu04921;mmu05020;mmu05034;mmu05161;mmu05164;mmu05200;mmu05205;mmu05206;mmu05210;mmu05211;mmu05212;mmu05213;mmu05214;mmu05215;mmu05216;mmu05218;mmu05219;mmu05220;mmu05221;mmu05223;mmu05230;mmu05231 |  | Map2k1 | azure4 | 1 | -2.26259946949602 |
  | Rhoa-mRNA | 0.225 | 0.102 | 0.0256 | 0.424 | 1.17 | 1.02 | 1.34 | 0.0471 | 0.619 | loglinear | Angiogenesis, Autophagy, Growth Factor Signaling, Wnt | NS\_MM\_NEUROINFLAM\_V1.0 | Rhoa | mRNA | 0 |  | Wnt;Growth Factor Signaling;Autophagy;Angiogenesis | mmu04014;mmu04015;mmu04022;mmu04024;mmu04062;mmu04071;mmu04072;mmu04144;mmu04270;mmu04310;mmu04350;mmu04360;mmu04510;mmu04520;mmu04530;mmu04611;mmu04660;mmu04670;mmu04722;mmu04810;mmu04921;mmu05100;mmu05133;mmu05152;mmu05200;mmu05203;mmu05205;mmu05206;mmu05210 |  | Rhoa | azure4 | 1 | 2.20588235294118 |
  | Rac1-mRNA | -0.0922 | 0.0461 | -0.183 | -0.00186 | 0.938 | 0.881 | 0.999 | 0.0686 | 0.841 | loglinear | Adaptive Immune Response, Angiogenesis, Autophagy, Growth Factor Signaling, Innate Immune Response, Neurons and Neurotransmission, Wnt | NS\_MM\_NEUROINFLAM\_V1.0 | Rac1 | mRNA | 0 |  | Wnt;Neurons and Neurotransmission;Innate Immune Response;Growth Factor Signaling;Autophagy;Angiogenesis;Adaptive Immune Response | mmu04010;mmu04014;mmu04015;mmu04024;mmu04062;mmu04071;mmu04145;mmu04151;mmu04310;mmu04360;mmu04370;mmu04380;mmu04510;mmu04520;mmu04620;mmu04650;mmu04662;mmu04664;mmu04666;mmu04670;mmu04722;mmu04810;mmu04932;mmu04933;mmu05014;mmu05100;mmu05132;mmu05200;mmu05203;mmu05205;mmu05210;mmu05211;mmu05212;mmu05231;mmu05416 |  | Rac1 | azure4 | 1 | -2 |
  | Kit-mRNA | -0.27 | 0.142 | -0.549 | 0.00831 | 0.829 | 0.683 | 1.01 | 0.0815 | 0.96 | lm.nb | Adaptive Immune Response, Angiogenesis, Cytokine Signaling, Growth Factor Signaling, Insulin Signaling | NS\_MM\_NEUROINFLAM\_V1.0 | Kit | mRNA | 0 |  | Insulin Signaling;Growth Factor Signaling;Cytokine Signaling;Angiogenesis;Adaptive Immune Response | mmu04014;mmu04015;mmu04060;mmu04072;mmu04144;mmu04151;mmu04640;mmu04916;mmu05200;mmu05221;mmu05230 |  | Kit | azure4 | 1 | -1.90140845070423 |
  | Pik3cb-mRNA | -0.128 | 0.0676 | -0.261 | 0.00424 | 0.915 | 0.835 | 1 | 0.0821 | 0.961 | loglinear | Adaptive Immune Response, Angiogenesis, Apoptosis, Autophagy, Carbohydrate Metabolism, Cytokine Signaling, Growth Factor Signaling, Innate Immune Response, Insulin Signaling, Lipid Metabolism | NS\_MM\_NEUROINFLAM\_V1.0 | Pik3cb | mRNA | 0 |  | Lipid Metabolism;Insulin Signaling;Innate Immune Response;Growth Factor Signaling;Cytokine Signaling;Carbohydrate Metabolism;Autophagy;Apoptosis;Angiogenesis;Adaptive Immune Response | mmu00562;mmu04012;mmu04014;mmu04015;mmu04022;mmu04024;mmu04062;mmu04066;mmu04068;mmu04070;mmu04071;mmu04072;mmu04150;mmu04151;mmu04152;mmu04210;mmu04211;mmu04213;mmu04261;mmu04370;mmu04380;mmu04510;mmu04550;mmu04611;mmu04620;mmu04630;mmu04650;mmu04660;mmu04662;mmu04664;mmu04666;mmu04668;mmu04670;mmu04722;mmu04725;mmu04750;mmu04810;mmu04910;mmu04914;mmu04915;mmu04917;mmu04919;mmu04921;mmu04923;mmu04930;mmu04931;mmu04932;mmu04933;mmu04960;mmu04973;mmu05100;mmu05142;mmu05145;mmu05146;mmu05160;mmu05161;mmu05162;mmu05164;mmu05166;mmu05169;mmu05200;mmu05203;mmu05205;mmu05210;mmu05211;mmu05212;mmu05213;mmu05214;mmu05215;mmu05218;mmu05220;mmu05221;mmu05222;mmu05223;mmu05230;mmu05231 |  | Pik3cb | azure4 | 1 | -1.89349112426036 |
  | Akt2-mRNA | -0.394 | 0.208 | -0.803 | 0.0139 | 0.761 | 0.573 | 1.01 | 0.0827 | 0.961 | lm.nb | Adaptive Immune Response, Angiogenesis, Apoptosis, Autophagy, Carbohydrate Metabolism, Cytokine Signaling, Growth Factor Signaling, Innate Immune Response, Insulin Signaling, Neurons and Neurotransmission, Wnt | NS\_MM\_NEUROINFLAM\_V1.0 | Akt2 | mRNA | 0 |  | Wnt;Neurons and Neurotransmission;Insulin Signaling;Innate Immune Response;Growth Factor Signaling;Cytokine Signaling;Carbohydrate Metabolism;Autophagy;Apoptosis;Angiogenesis;Adaptive Immune Response | mmu04010;mmu04012;mmu04014;mmu04015;mmu04022;mmu04024;mmu04062;mmu04066;mmu04068;mmu04071;mmu04072;mmu04150;mmu04151;mmu04152;mmu04210;mmu04211;mmu04213;mmu04261;mmu04370;mmu04380;mmu04510;mmu04530;mmu04550;mmu04611;mmu04620;mmu04630;mmu04660;mmu04662;mmu04664;mmu04666;mmu04668;mmu04722;mmu04725;mmu04728;mmu04910;mmu04914;mmu04915;mmu04917;mmu04919;mmu04920;mmu04922;mmu04923;mmu04931;mmu04932;mmu04933;mmu04973;mmu05142;mmu05145;mmu05152;mmu05160;mmu05161;mmu05162;mmu05164;mmu05166;mmu05169;mmu05200;mmu05205;mmu05210;mmu05211;mmu05212;mmu05213;mmu05214;mmu05215;mmu05218;mmu05220;mmu05221;mmu05222;mmu05223;mmu05230;mmu05231 |  | Akt2 | azure4 | 1 | -1.89423076923077 |
  | Axl-mRNA | 0.366 | 0.2 | -0.0255 | 0.757 | 1.29 | 0.982 | 1.69 | 0.0918 | 1 | lm.nb | Angiogenesis, Autophagy, Microglia Function | NS\_MM\_NEUROINFLAM\_V1.0 | Axl | mRNA | 0 |  | Microglia Function;Autophagy;Angiogenesis |  |  | Axl | azure4 | 1 | 1.83 |
  | Mapk14-mRNA | -0.127 | 0.0867 | -0.297 | 0.043 | 0.916 | 0.814 | 1.03 | 0.169 | 1 | loglinear | Angiogenesis, Autophagy, Cellular Stress, Growth Factor Signaling, Innate Immune Response | NS\_MM\_NEUROINFLAM\_V1.0 | Mapk14 | mRNA | 0 |  | Innate Immune Response;Growth Factor Signaling;Cellular Stress;Autophagy;Angiogenesis | mmu04010;mmu04015;mmu04068;mmu04071;mmu04261;mmu04370;mmu04380;mmu04550;mmu04611;mmu04620;mmu04621;mmu04622;mmu04660;mmu04664;mmu04668;mmu04670;mmu04722;mmu04723;mmu04728;mmu04750;mmu04912;mmu04914;mmu04917;mmu04933;mmu05014;mmu05132;mmu05133;mmu05140;mmu05142;mmu05145;mmu05152;mmu05160;mmu05164;mmu05169;mmu05205 |  | Mapk14 | azure4 | 1 | -1.46482122260669 |

  ##### DE Results - Angiogenesis genes - DPI: 3 vs.CTRL

  Table displaying each sample's global significance scores and directed global significance scores as defined in the heatmaps above. The global significance score is calculated as the square root of the mean squared t-statistic for the genes in a gene set, with t-statistics coming from the linear regression underlying our differential expression analysis. The directed global significance score is calculated as the square root of the mean signed squared t-statistic for the genes in a gene set, with t-statistics coming from the linear regression underlying our differential expression analysis.

DPI:
- DPI: differential expression in 1 vs. baseline of CTRL
- DPI: differential expression in 2 vs. baseline of CTRL
- DPI: differential expression in 3 vs. baseline of CTRL

- **Volcano Plot: DPI: 1 vs.CTRL  
  More Plot Information**

  ##### Volcano Plot: DPI: 1 vs.CTRL

  Volcano plot displaying each gene's -log10(p-value) and log2 fold change for the selected covariate. Highly statistically significant genes fall at the top of the plot, and highly differentially expressed genes fall to either side. Genes within the selected gene set are highlighted in orange. Horizontal lines indicate various False Discovery Rate (FDR) thresholds.
- **DE Results - Apoptosis genes - DPI: 1 vs.CTRL  
  More Plot Information  Download CSV Data**

  | Probe Label | Log2 fold change | std error (log2) | Lower confidence limit (log2) | Upper confidence limit (log2) | Linear fold change | Lower confidence limit (linear) | Upper confidence limit (linear) | P-value | BY.p.value | method | Gene.sets | Codeset.Name | Probe.Label | Analyte.Type | Is.Control | Control.Type | Probe.Annotation | KEGG.Pathways | Cell.Type | Official.Gene.Name | volcanocol | volcanopch | tstats.all |
  | --- | --- | --- | --- | --- | --- | --- | --- | --- | --- | --- | --- | --- | --- | --- | --- | --- | --- | --- | --- | --- | --- | --- | --- |
  | Cd14-mRNA | 2.57 | 0.657 | 1.28 | 3.85 | 5.92 | 2.43 | 14.5 | 0.00208 | 0.437 | lm.nb | Apoptosis, Astrocyte Function, Autophagy, Growth Factor Signaling, Innate Immune Response, NF-kB | NS\_MM\_NEUROINFLAM\_V1.0 | Cd14 | mRNA | 0 |  | NF-kB;Innate Immune Response;Growth Factor Signaling;Autophagy;Astrocyte Function;Apoptosis | mmu04010;mmu04064;mmu04145;mmu04620;mmu04640;mmu04810;mmu05132;mmu05133;mmu05134;mmu05146;mmu05152;mmu05202 |  | Cd14 | slateblue4 | 16 | 3.9117199391172 |
  | Nfkbia-mRNA | 0.866 | 0.242 | 0.392 | 1.34 | 1.82 | 1.31 | 2.53 | 0.00379 | 0.558 | lm.nb | Adaptive Immune Response, Apoptosis, Growth Factor Signaling, Inflammatory Signaling, Innate Immune Response, NF-kB | NS\_MM\_NEUROINFLAM\_V1.0 | Nfkbia | mRNA | 0 |  | NF-kB;Innate Immune Response;Inflammatory Signaling;Growth Factor Signaling;Apoptosis;Adaptive Immune Response | mmu04024;mmu04062;mmu04064;mmu04210;mmu04380;mmu04620;mmu04621;mmu04622;mmu04623;mmu04660;mmu04662;mmu04668;mmu04722;mmu04920;mmu04931;mmu05134;mmu05140;mmu05142;mmu05145;mmu05160;mmu05161;mmu05162;mmu05164;mmu05166;mmu05168;mmu05169;mmu05200;mmu05203;mmu05215;mmu05220;mmu05222 |  | Nfkbia | azure4 | 1 | 3.57851239669422 |
  | Il1rap-mRNA | -0.445 | 0.125 | -0.689 | -0.2 | 0.735 | 0.62 | 0.871 | 0.00391 | 0.558 | loglinear | Apoptosis, Cytokine Signaling | NS\_MM\_NEUROINFLAM\_V1.0 | Il1rap | mRNA | 0 |  | Cytokine Signaling;Apoptosis | mmu04060;mmu04210;mmu04750 |  | Il1rap | azure4 | 1 | -3.56 |
  | Braf-mRNA | 0.0984 | 0.0282 | 0.0432 | 0.154 | 1.07 | 1.03 | 1.11 | 0.00445 | 0.577 | loglinear | Adaptive Immune Response, Angiogenesis, Apoptosis, Cytokine Signaling, Growth Factor Signaling, Innate Immune Response, Insulin Signaling, Neurons and Neurotransmission | NS\_MM\_NEUROINFLAM\_V1.0 | Braf | mRNA | 0 |  | Neurons and Neurotransmission;Insulin Signaling;Innate Immune Response;Growth Factor Signaling;Cytokine Signaling;Apoptosis;Angiogenesis;Adaptive Immune Response | mmu04010;mmu04012;mmu04015;mmu04024;mmu04062;mmu04068;mmu04150;mmu04270;mmu04510;mmu04650;mmu04720;mmu04722;mmu04726;mmu04730;mmu04810;mmu04910;mmu04914;mmu05034;mmu05160;mmu05200;mmu05205;mmu05210;mmu05211;mmu05212;mmu05213;mmu05214;mmu05215;mmu05216;mmu05218;mmu05219;mmu05220;mmu05221;mmu05223 |  | Braf | azure4 | 1 | 3.48936170212766 |
  | Il1r1-mRNA | 0.625 | 0.181 | 0.27 | 0.98 | 1.54 | 1.21 | 1.97 | 0.00477 | 0.577 | lm.nb | Apoptosis, Cellular Stress, Cytokine Signaling, Growth Factor Signaling, Microglia Function, NF-kB | NS\_MM\_NEUROINFLAM\_V1.0 | Il1r1 | mRNA | 0 |  | NF-kB;Microglia Function;Growth Factor Signaling;Cytokine Signaling;Cellular Stress;Apoptosis | mmu04010;mmu04060;mmu04064;mmu04210;mmu04380;mmu04640;mmu04750;mmu05146;mmu05166 |  | Il1r1 | azure4 | 1 | 3.45303867403315 |
  | Jun-mRNA | 0.323 | 0.0979 | 0.131 | 0.515 | 1.25 | 1.09 | 1.43 | 0.0064 | 0.711 | loglinear | Adaptive Immune Response, Apoptosis, Cellular Stress, Growth Factor Signaling, Innate Immune Response, Notch, Wnt | NS\_MM\_NEUROINFLAM\_V1.0 | Jun | mRNA | 0 |  | Wnt;Notch;Innate Immune Response;Growth Factor Signaling;Cellular Stress;Apoptosis;Adaptive Immune Response | mmu04010;mmu04012;mmu04024;mmu04310;mmu04380;mmu04510;mmu04620;mmu04660;mmu04662;mmu04668;mmu04722;mmu04912;mmu04915;mmu04921;mmu04932;mmu04933;mmu05030;mmu05031;mmu05132;mmu05133;mmu05140;mmu05142;mmu05161;mmu05164;mmu05166;mmu05168;mmu05169;mmu05200;mmu05203;mmu05210;mmu05211;mmu05231;mmu05321;mmu05323 |  | Jun | azure4 | 1 | 3.29928498467824 |
  | Vim-mRNA | 0.712 | 0.223 | 0.275 | 1.15 | 1.64 | 1.21 | 2.22 | 0.00772 | 0.76 | lm.nb | Apoptosis, Astrocyte Function | NS\_MM\_NEUROINFLAM\_V1.0 | Vim | mRNA | 0 |  | Astrocyte Function;Apoptosis | mmu05169;mmu05206 |  | Vim | azure4 | 1 | 3.19282511210762 |
  | Bag3-mRNA | 0.567 | 0.182 | 0.21 | 0.924 | 1.48 | 1.16 | 1.9 | 0.00895 | 0.813 | loglinear | Apoptosis, Cellular Stress | NS\_MM\_NEUROINFLAM\_V1.0 | Bag3 | mRNA | 0 |  | Cellular Stress;Apoptosis |  |  | Bag3 | azure4 | 1 | 3.11538461538461 |
  | Bcl2l1-mRNA | 0.173 | 0.0572 | 0.0603 | 0.285 | 1.13 | 1.04 | 1.22 | 0.0108 | 0.861 | loglinear | Apoptosis, Autophagy, Cytokine Signaling, Growth Factor Signaling, Innate Immune Response, NF-kB | NS\_MM\_NEUROINFLAM\_V1.0 | Bcl2l1 | mRNA | 0 |  | NF-kB;Innate Immune Response;Growth Factor Signaling;Cytokine Signaling;Autophagy;Apoptosis | mmu04014;mmu04064;mmu04151;mmu04210;mmu04630;mmu05014;mmu05145;mmu05166;mmu05200;mmu05202;mmu05212;mmu05220;mmu05222 |  | Bcl2l1 | azure4 | 1 | 3.02447552447552 |
  | Bnip3-mRNA | 0.118 | 0.0396 | 0.0406 | 0.196 | 1.09 | 1.03 | 1.15 | 0.0114 | 0.868 | loglinear | Apoptosis, Autophagy, Cellular Stress, Microglia Function | NS\_MM\_NEUROINFLAM\_V1.0 | Bnip3 | mRNA | 0 |  | Microglia Function;Cellular Stress;Autophagy;Apoptosis | mmu04068;mmu05134 |  | Bnip3 | azure4 | 1 | 2.97979797979798 |
  | Casp3-mRNA | 0.393 | 0.132 | 0.134 | 0.652 | 1.31 | 1.1 | 1.57 | 0.0115 | 0.868 | lm.nb | Apoptosis, Cellular Stress, DNA Damage, Growth Factor Signaling, Innate Immune Response, Matrix Remodeling | NS\_MM\_NEUROINFLAM\_V1.0 | Casp3 | mRNA | 0 |  | Matrix Remodeling;Innate Immune Response;Growth Factor Signaling;DNA Damage;Cellular Stress;Apoptosis | mmu04010;mmu04115;mmu04210;mmu04650;mmu04668;mmu04726;mmu04932;mmu04933;mmu05010;mmu05012;mmu05014;mmu05016;mmu05133;mmu05134;mmu05145;mmu05146;mmu05152;mmu05161;mmu05168;mmu05200;mmu05203;mmu05205;mmu05206;mmu05210;mmu05416 |  | Casp3 | azure4 | 1 | 2.97727272727273 |
  | Myd88-mRNA | 0.604 | 0.215 | 0.184 | 1.02 | 1.52 | 1.14 | 2.03 | 0.0156 | 1 | lm.nb | Apoptosis, Cytokine Signaling, Growth Factor Signaling, Inflammatory Signaling, Innate Immune Response, NF-kB | NS\_MM\_NEUROINFLAM\_V1.0 | Myd88 | mRNA | 0 |  | NF-kB;Innate Immune Response;Inflammatory Signaling;Growth Factor Signaling;Cytokine Signaling;Apoptosis | mmu04064;mmu04210;mmu04620;mmu05132;mmu05133;mmu05134;mmu05140;mmu05142;mmu05143;mmu05144;mmu05145;mmu05152;mmu05161;mmu05162;mmu05164;mmu05168 |  | Myd88 | azure4 | 1 | 2.8093023255814 |
  | Tnfrsf1a-mRNA | 0.827 | 0.299 | 0.241 | 1.41 | 1.77 | 1.18 | 2.66 | 0.017 | 1 | lm.nb | Apoptosis, Cytokine Signaling, Growth Factor Signaling, Innate Immune Response, NF-kB | NS\_MM\_NEUROINFLAM\_V1.0 | Tnfrsf1a | mRNA | 0 |  | NF-kB;Innate Immune Response;Growth Factor Signaling;Cytokine Signaling;Apoptosis | mmu04010;mmu04060;mmu04064;mmu04071;mmu04210;mmu04380;mmu04668;mmu04920;mmu04931;mmu04932;mmu05010;mmu05014;mmu05142;mmu05145;mmu05152;mmu05160;mmu05164;mmu05166;mmu05168 |  | Tnfrsf1a | azure4 | 1 | 2.76588628762542 |
  | Pik3r2-mRNA | -0.099 | 0.0389 | -0.175 | -0.0229 | 0.934 | 0.886 | 0.984 | 0.0256 | 1 | loglinear | Adaptive Immune Response, Angiogenesis, Apoptosis, Autophagy, Cytokine Signaling, Growth Factor Signaling, Innate Immune Response, Insulin Signaling | NS\_MM\_NEUROINFLAM\_V1.0 | Pik3r2 | mRNA | 0 |  | Insulin Signaling;Innate Immune Response;Growth Factor Signaling;Cytokine Signaling;Autophagy;Apoptosis;Angiogenesis;Adaptive Immune Response | mmu04012;mmu04014;mmu04015;mmu04022;mmu04024;mmu04062;mmu04066;mmu04068;mmu04070;mmu04071;mmu04072;mmu04150;mmu04151;mmu04152;mmu04210;mmu04211;mmu04213;mmu04261;mmu04370;mmu04380;mmu04510;mmu04550;mmu04611;mmu04620;mmu04630;mmu04650;mmu04660;mmu04662;mmu04664;mmu04666;mmu04668;mmu04670;mmu04722;mmu04725;mmu04750;mmu04810;mmu04910;mmu04914;mmu04915;mmu04917;mmu04919;mmu04921;mmu04923;mmu04930;mmu04931;mmu04932;mmu04933;mmu04960;mmu04973;mmu05100;mmu05142;mmu05145;mmu05146;mmu05160;mmu05161;mmu05162;mmu05164;mmu05166;mmu05169;mmu05200;mmu05203;mmu05205;mmu05210;mmu05211;mmu05212;mmu05213;mmu05214;mmu05215;mmu05218;mmu05220;mmu05221;mmu05222;mmu05223;mmu05230;mmu05231 |  | Pik3r2 | azure4 | 1 | -2.54498714652956 |
  | Il1a-mRNA | 2.22 | 0.937 | 0.385 | 4.06 | 4.67 | 1.31 | 16.7 | 0.0354 | 1 | lm.nb | Apoptosis, Cellular Stress, Cytokine Signaling, Growth Factor Signaling | NS\_MM\_NEUROINFLAM\_V1.0 | Il1a | mRNA | 0 |  | Growth Factor Signaling;Cytokine Signaling;Cellular Stress;Apoptosis | mmu04010;mmu04060;mmu04210;mmu04380;mmu04640;mmu04932;mmu04933;mmu04940;mmu05020;mmu05132;mmu05133;mmu05140;mmu05152;mmu05162;mmu05164;mmu05321;mmu05323;mmu05332 |  | Il1a | azure4 | 1 | 2.3692636072572 |
  | Bag4-mRNA | 0.147 | 0.0629 | 0.0237 | 0.27 | 1.11 | 1.02 | 1.21 | 0.0375 | 1 | loglinear | Apoptosis, Cellular Stress, Innate Immune Response | NS\_MM\_NEUROINFLAM\_V1.0 | Bag4 | mRNA | 0 |  | Innate Immune Response;Cellular Stress;Apoptosis | mmu04668 |  | Bag4 | azure4 | 1 | 2.33704292527822 |
  | Cidea-mRNA | 0.413 | 0.177 | 0.0655 | 0.761 | 1.33 | 1.05 | 1.69 | 0.0381 | 1 | lm.nb | Apoptosis | NS\_MM\_NEUROINFLAM\_V1.0 | Cidea | mRNA | 0 |  | Apoptosis |  |  | Cidea | azure4 | 1 | 2.33333333333333 |
  | Traf3-mRNA | -0.322 | 0.139 | -0.594 | -0.0498 | 0.8 | 0.662 | 0.966 | 0.0389 | 1 | loglinear | Apoptosis, Cytokine Signaling, Inflammatory Signaling, Innate Immune Response, NF-kB | NS\_MM\_NEUROINFLAM\_V1.0 | Traf3 | mRNA | 0 |  | NF-kB;Innate Immune Response;Inflammatory Signaling;Cytokine Signaling;Apoptosis | mmu04064;mmu04620;mmu04622;mmu04668;mmu05160;mmu05168;mmu05169;mmu05200;mmu05203;mmu05222 |  | Traf3 | azure4 | 1 | -2.31654676258993 |
  | Apc-mRNA | -0.18 | 0.0782 | -0.333 | -0.0269 | 0.883 | 0.794 | 0.982 | 0.0399 | 1 | loglinear | Apoptosis, Astrocyte Function, Wnt | NS\_MM\_NEUROINFLAM\_V1.0 | Apc | mRNA | 0 |  | Wnt;Astrocyte Function;Apoptosis | mmu04310;mmu04390;mmu04550;mmu04810;mmu05166;mmu05200;mmu05206;mmu05210;mmu05213 |  | Apc | azure4 | 1 | -2.30179028132992 |
  | Casp9-mRNA | 0.284 | 0.132 | 0.025 | 0.544 | 1.22 | 1.02 | 1.46 | 0.0528 | 1 | loglinear | Adaptive Immune Response, Apoptosis, DNA Damage, Growth Factor Signaling, Innate Immune Response | NS\_MM\_NEUROINFLAM\_V1.0 | Casp9 | mRNA | 0 |  | Innate Immune Response;Growth Factor Signaling;DNA Damage;Apoptosis;Adaptive Immune Response | mmu04115;mmu04151;mmu04210;mmu04370;mmu04919;mmu05010;mmu05012;mmu05014;mmu05016;mmu05134;mmu05145;mmu05152;mmu05161;mmu05164;mmu05200;mmu05210;mmu05212;mmu05213;mmu05215;mmu05222;mmu05223;mmu05416 |  | Casp9 | azure4 | 1 | 2.15151515151515 |

  ##### DE Results - Apoptosis genes - DPI: 1 vs.CTRL

  Table displaying each sample's global significance scores and directed global significance scores as defined in the heatmaps above. The global significance score is calculated as the square root of the mean squared t-statistic for the genes in a gene set, with t-statistics coming from the linear regression underlying our differential expression analysis. The directed global significance score is calculated as the square root of the mean signed squared t-statistic for the genes in a gene set, with t-statistics coming from the linear regression underlying our differential expression analysis.

- **Volcano Plot: DPI: 2 vs.CTRL  
  More Plot Information**

  ##### Volcano Plot: DPI: 2 vs.CTRL

  Volcano plot displaying each gene's -log10(p-value) and log2 fold change for the selected covariate. Highly statistically significant genes fall at the top of the plot, and highly differentially expressed genes fall to either side. Genes within the selected gene set are highlighted in orange. Horizontal lines indicate various False Discovery Rate (FDR) thresholds.
- **DE Results - Apoptosis genes - DPI: 2 vs.CTRL  
  More Plot Information  Download CSV Data**

  | Probe Label | Log2 fold change | std error (log2) | Lower confidence limit (log2) | Upper confidence limit (log2) | Linear fold change | Lower confidence limit (linear) | Upper confidence limit (linear) | P-value | BY.p.value | method | Gene.sets | Codeset.Name | Probe.Label | Analyte.Type | Is.Control | Control.Type | Probe.Annotation | KEGG.Pathways | Cell.Type | Official.Gene.Name | volcanocol | volcanopch | tstats.all |
  | --- | --- | --- | --- | --- | --- | --- | --- | --- | --- | --- | --- | --- | --- | --- | --- | --- | --- | --- | --- | --- | --- | --- | --- |
  | Psmb8-mRNA | 2.78 | 0.196 | 2.39 | 3.16 | 6.85 | 5.25 | 8.94 | 7.51e-09 | 4.29e-06 | lm.nb | Adaptive Immune Response, Angiogenesis, Apoptosis, Astrocyte Function, Cell Cycle, Cytokine Signaling, Growth Factor Signaling, Inflammatory Signaling, Insulin Signaling, Microglia Function, NF-kB, Wnt | NS\_MM\_NEUROINFLAM\_V1.0 | Psmb8 | mRNA | 0 |  | Wnt;NF-kB;Microglia Function;Insulin Signaling;Inflammatory Signaling;Growth Factor Signaling;Cytokine Signaling;Cell Cycle;Astrocyte Function;Apoptosis;Angiogenesis;Adaptive Immune Response |  |  | Psmb8 | slateblue1 | 16 | 14.1836734693878 |
  | Irf1-mRNA | 2.25 | 0.201 | 1.86 | 2.64 | 4.76 | 3.62 | 6.25 | 1.02e-07 | 3.7e-05 | lm.nb | Apoptosis, Inflammatory Signaling, Innate Immune Response | NS\_MM\_NEUROINFLAM\_V1.0 | Irf1 | mRNA | 0 |  | Innate Immune Response;Inflammatory Signaling;Apoptosis | mmu04917;mmu05133;mmu05160 |  | Irf1 | slateblue1 | 16 | 11.1940298507463 |
  | Pik3r2-mRNA | -0.347 | 0.0389 | -0.423 | -0.27 | 0.786 | 0.746 | 0.829 | 1.22e-06 | 0.000256 | loglinear | Adaptive Immune Response, Angiogenesis, Apoptosis, Autophagy, Cytokine Signaling, Growth Factor Signaling, Innate Immune Response, Insulin Signaling | NS\_MM\_NEUROINFLAM\_V1.0 | Pik3r2 | mRNA | 0 |  | Insulin Signaling;Innate Immune Response;Growth Factor Signaling;Cytokine Signaling;Autophagy;Apoptosis;Angiogenesis;Adaptive Immune Response | mmu04012;mmu04014;mmu04015;mmu04022;mmu04024;mmu04062;mmu04066;mmu04068;mmu04070;mmu04071;mmu04072;mmu04150;mmu04151;mmu04152;mmu04210;mmu04211;mmu04213;mmu04261;mmu04370;mmu04380;mmu04510;mmu04550;mmu04611;mmu04620;mmu04630;mmu04650;mmu04660;mmu04662;mmu04664;mmu04666;mmu04668;mmu04670;mmu04722;mmu04725;mmu04750;mmu04810;mmu04910;mmu04914;mmu04915;mmu04917;mmu04919;mmu04921;mmu04923;mmu04930;mmu04931;mmu04932;mmu04933;mmu04960;mmu04973;mmu05100;mmu05142;mmu05145;mmu05146;mmu05160;mmu05161;mmu05162;mmu05164;mmu05166;mmu05169;mmu05200;mmu05203;mmu05205;mmu05210;mmu05211;mmu05212;mmu05213;mmu05214;mmu05215;mmu05218;mmu05220;mmu05221;mmu05222;mmu05223;mmu05230;mmu05231 |  | Pik3r2 | slateblue1 | 16 | -8.92030848329049 |
  | Bnip3-mRNA | 0.317 | 0.0396 | 0.239 | 0.395 | 1.25 | 1.18 | 1.31 | 3.73e-06 | 0.000532 | loglinear | Apoptosis, Autophagy, Cellular Stress, Microglia Function | NS\_MM\_NEUROINFLAM\_V1.0 | Bnip3 | mRNA | 0 |  | Microglia Function;Cellular Stress;Autophagy;Apoptosis | mmu04068;mmu05134 |  | Bnip3 | slateblue1 | 16 | 8.00505050505051 |
  | Tubb3-mRNA | -0.383 | 0.0494 | -0.48 | -0.286 | 0.767 | 0.717 | 0.82 | 5.22e-06 | 0.000718 | loglinear | Adaptive Immune Response, Apoptosis, Cell Cycle, Neurons and Neurotransmission | NS\_MM\_NEUROINFLAM\_V1.0 | Tubb3 | mRNA | 0 |  | Neurons and Neurotransmission;Cell Cycle;Apoptosis;Adaptive Immune Response | mmu04145;mmu04540 |  | Tubb3 | slateblue1 | 16 | -7.75303643724696 |
  | Cd14-mRNA | 4.52 | 0.641 | 3.26 | 5.78 | 22.9 | 9.59 | 54.8 | 1.34e-05 | 0.00162 | lm.nb | Apoptosis, Astrocyte Function, Autophagy, Growth Factor Signaling, Innate Immune Response, NF-kB | NS\_MM\_NEUROINFLAM\_V1.0 | Cd14 | mRNA | 0 |  | NF-kB;Innate Immune Response;Growth Factor Signaling;Autophagy;Astrocyte Function;Apoptosis | mmu04010;mmu04064;mmu04145;mmu04620;mmu04640;mmu04810;mmu05132;mmu05133;mmu05134;mmu05146;mmu05152;mmu05202 |  | Cd14 | slateblue1 | 16 | 7.05148205928237 |
  | Il1r1-mRNA | 1.09 | 0.175 | 0.752 | 1.44 | 2.14 | 1.68 | 2.71 | 4.23e-05 | 0.00403 | lm.nb | Apoptosis, Cellular Stress, Cytokine Signaling, Growth Factor Signaling, Microglia Function, NF-kB | NS\_MM\_NEUROINFLAM\_V1.0 | Il1r1 | mRNA | 0 |  | NF-kB;Microglia Function;Growth Factor Signaling;Cytokine Signaling;Cellular Stress;Apoptosis | mmu04010;mmu04060;mmu04064;mmu04210;mmu04380;mmu04640;mmu04750;mmu05146;mmu05166 |  | Il1r1 | slateblue1 | 16 | 6.22857142857143 |
  | Prkar2b-mRNA | -0.548 | 0.0919 | -0.728 | -0.368 | 0.684 | 0.604 | 0.775 | 6.54e-05 | 0.00556 | loglinear | Apoptosis, Cell Cycle, Growth Factor Signaling | NS\_MM\_NEUROINFLAM\_V1.0 | Prkar2b | mRNA | 0 |  | Growth Factor Signaling;Cell Cycle;Apoptosis | mmu04210;mmu04910 |  | Prkar2b | slateblue1 | 16 | -5.96300326441785 |
  | Nfkbia-mRNA | 1.42 | 0.238 | 0.952 | 1.88 | 2.67 | 1.93 | 3.69 | 6.55e-05 | 0.00556 | lm.nb | Adaptive Immune Response, Apoptosis, Growth Factor Signaling, Inflammatory Signaling, Innate Immune Response, NF-kB | NS\_MM\_NEUROINFLAM\_V1.0 | Nfkbia | mRNA | 0 |  | NF-kB;Innate Immune Response;Inflammatory Signaling;Growth Factor Signaling;Apoptosis;Adaptive Immune Response | mmu04024;mmu04062;mmu04064;mmu04210;mmu04380;mmu04620;mmu04621;mmu04622;mmu04623;mmu04660;mmu04662;mmu04668;mmu04722;mmu04920;mmu04931;mmu05134;mmu05140;mmu05142;mmu05145;mmu05160;mmu05161;mmu05162;mmu05164;mmu05166;mmu05168;mmu05169;mmu05200;mmu05203;mmu05215;mmu05220;mmu05222 |  | Nfkbia | slateblue1 | 16 | 5.96638655462185 |
  | Irf7-mRNA | 2.67 | 0.455 | 1.78 | 3.57 | 6.39 | 3.44 | 11.8 | 7.48e-05 | 0.00586 | lm.nb | Apoptosis, Inflammatory Signaling, Innate Immune Response | NS\_MM\_NEUROINFLAM\_V1.0 | Irf7 | mRNA | 0 |  | Innate Immune Response;Inflammatory Signaling;Apoptosis | mmu04620;mmu04622;mmu04623;mmu05160;mmu05161;mmu05162;mmu05164;mmu05168;mmu05203 |  | Irf7 | slateblue1 | 16 | 5.86813186813187 |
  | Bcl2l1-mRNA | 0.33 | 0.0572 | 0.217 | 0.442 | 1.26 | 1.16 | 1.36 | 9.06e-05 | 0.00658 | loglinear | Apoptosis, Autophagy, Cytokine Signaling, Growth Factor Signaling, Innate Immune Response, NF-kB | NS\_MM\_NEUROINFLAM\_V1.0 | Bcl2l1 | mRNA | 0 |  | NF-kB;Innate Immune Response;Growth Factor Signaling;Cytokine Signaling;Autophagy;Apoptosis | mmu04014;mmu04064;mmu04151;mmu04210;mmu04630;mmu05014;mmu05145;mmu05166;mmu05200;mmu05202;mmu05212;mmu05220;mmu05222 |  | Bcl2l1 | slateblue1 | 16 | 5.76923076923077 |
  | Bcl2a1a-mRNA | 2.13 | 0.44 | 1.26 | 2.99 | 4.37 | 2.4 | 7.95 | 0.00041 | 0.0228 | lm.nb | Apoptosis, NF-kB | NS\_MM\_NEUROINFLAM\_V1.0 | Bcl2a1a | mRNA | 0 |  | NF-kB;Apoptosis | mmu04064;mmu05202 |  | Bcl2a1a | slateblue2 | 16 | 4.84090909090909 |
  | Myd88-mRNA | 1.01 | 0.21 | 0.601 | 1.42 | 2.02 | 1.52 | 2.68 | 0.000418 | 0.0228 | lm.nb | Apoptosis, Cytokine Signaling, Growth Factor Signaling, Inflammatory Signaling, Innate Immune Response, NF-kB | NS\_MM\_NEUROINFLAM\_V1.0 | Myd88 | mRNA | 0 |  | NF-kB;Innate Immune Response;Inflammatory Signaling;Growth Factor Signaling;Cytokine Signaling;Apoptosis | mmu04064;mmu04210;mmu04620;mmu05132;mmu05133;mmu05134;mmu05140;mmu05142;mmu05143;mmu05144;mmu05145;mmu05152;mmu05161;mmu05162;mmu05164;mmu05168 |  | Myd88 | slateblue2 | 16 | 4.80952380952381 |
  | Il1a-mRNA | 4.25 | 0.922 | 2.44 | 6.06 | 19 | 5.43 | 66.5 | 0.000605 | 0.0291 | lm.nb | Apoptosis, Cellular Stress, Cytokine Signaling, Growth Factor Signaling | NS\_MM\_NEUROINFLAM\_V1.0 | Il1a | mRNA | 0 |  | Growth Factor Signaling;Cytokine Signaling;Cellular Stress;Apoptosis | mmu04010;mmu04060;mmu04210;mmu04380;mmu04640;mmu04932;mmu04933;mmu04940;mmu05020;mmu05132;mmu05133;mmu05140;mmu05152;mmu05162;mmu05164;mmu05321;mmu05323;mmu05332 |  | Il1a | slateblue2 | 16 | 4.60954446854664 |
  | Tnfrsf1a-mRNA | 1.3 | 0.295 | 0.72 | 1.88 | 2.46 | 1.65 | 3.67 | 0.000868 | 0.0385 | lm.nb | Apoptosis, Cytokine Signaling, Growth Factor Signaling, Innate Immune Response, NF-kB | NS\_MM\_NEUROINFLAM\_V1.0 | Tnfrsf1a | mRNA | 0 |  | NF-kB;Innate Immune Response;Growth Factor Signaling;Cytokine Signaling;Apoptosis | mmu04010;mmu04060;mmu04064;mmu04071;mmu04210;mmu04380;mmu04668;mmu04920;mmu04931;mmu04932;mmu05010;mmu05014;mmu05142;mmu05145;mmu05152;mmu05160;mmu05164;mmu05166;mmu05168 |  | Tnfrsf1a | slateblue2 | 16 | 4.40677966101695 |
  | Prkar2a-mRNA | -0.21 | 0.0484 | -0.305 | -0.115 | 0.865 | 0.81 | 0.923 | 0.000956 | 0.0399 | loglinear | Apoptosis, Growth Factor Signaling | NS\_MM\_NEUROINFLAM\_V1.0 | Prkar2a | mRNA | 0 |  | Growth Factor Signaling;Apoptosis | mmu04210;mmu04910 |  | Prkar2a | slateblue2 | 16 | -4.33884297520661 |
  | Prkcq-mRNA | -0.84 | 0.198 | -1.23 | -0.451 | 0.559 | 0.427 | 0.732 | 0.00116 | 0.0479 | lm.nb | Adaptive Immune Response, Apoptosis, Autophagy, NF-kB, Oligodendrocyte Function | NS\_MM\_NEUROINFLAM\_V1.0 | Prkcq | mRNA | 0 |  | Oligodendrocyte Function;NF-kB;Autophagy;Apoptosis;Adaptive Immune Response | mmu04064;mmu04270;mmu04530;mmu04660;mmu04750;mmu04920;mmu04931;mmu05162 | Oligodendrocytes | Prkcq | slateblue2 | 16 | -4.24242424242424 |
  | Ngf-mRNA | -1.09 | 0.264 | -1.61 | -0.575 | 0.469 | 0.327 | 0.671 | 0.00138 | 0.0545 | lm.nb | Apoptosis, Growth Factor Signaling | NS\_MM\_NEUROINFLAM\_V1.0 | Ngf | mRNA | 0 |  | Growth Factor Signaling;Apoptosis | mmu04010;mmu04014;mmu04015;mmu04151;mmu04210;mmu04722;mmu04750 |  | Ngf | slateblue3 | 16 | -4.12878787878788 |
  | Vim-mRNA | 0.876 | 0.223 | 0.439 | 1.31 | 1.84 | 1.36 | 2.48 | 0.00199 | 0.0722 | lm.nb | Apoptosis, Astrocyte Function | NS\_MM\_NEUROINFLAM\_V1.0 | Vim | mRNA | 0 |  | Astrocyte Function;Apoptosis | mmu05169;mmu05206 |  | Vim | slateblue3 | 16 | 3.92825112107623 |
  | Bbc3-mRNA | 0.635 | 0.167 | 0.308 | 0.961 | 1.55 | 1.24 | 1.95 | 0.00249 | 0.0848 | loglinear | Apoptosis, DNA Damage | NS\_MM\_NEUROINFLAM\_V1.0 | Bbc3 | mRNA | 0 |  | DNA Damage;Apoptosis | mmu04115;mmu04390;mmu05016;mmu05162 |  | Bbc3 | slateblue3 | 16 | 3.80239520958084 |

  ##### DE Results - Apoptosis genes - DPI: 2 vs.CTRL

  Table displaying each sample's global significance scores and directed global significance scores as defined in the heatmaps above. The global significance score is calculated as the square root of the mean squared t-statistic for the genes in a gene set, with t-statistics coming from the linear regression underlying our differential expression analysis. The directed global significance score is calculated as the square root of the mean signed squared t-statistic for the genes in a gene set, with t-statistics coming from the linear regression underlying our differential expression analysis.

- **Volcano Plot: DPI: 3 vs.CTRL  
  More Plot Information**

  ##### Volcano Plot: DPI: 3 vs.CTRL

  Volcano plot displaying each gene's -log10(p-value) and log2 fold change for the selected covariate. Highly statistically significant genes fall at the top of the plot, and highly differentially expressed genes fall to either side. Genes within the selected gene set are highlighted in orange. Horizontal lines indicate various False Discovery Rate (FDR) thresholds.
- **DE Results - Apoptosis genes - DPI: 3 vs.CTRL  
  More Plot Information  Download CSV Data**

  | Probe Label | Log2 fold change | std error (log2) | Lower confidence limit (log2) | Upper confidence limit (log2) | Linear fold change | Lower confidence limit (linear) | Upper confidence limit (linear) | P-value | BY.p.value | method | Gene.sets | Codeset.Name | Probe.Label | Analyte.Type | Is.Control | Control.Type | Probe.Annotation | KEGG.Pathways | Cell.Type | Official.Gene.Name | volcanocol | volcanopch | tstats.all |
  | --- | --- | --- | --- | --- | --- | --- | --- | --- | --- | --- | --- | --- | --- | --- | --- | --- | --- | --- | --- | --- | --- | --- | --- |
  | Psmb8-mRNA | 4.35 | 0.191 | 3.98 | 4.73 | 20.4 | 15.7 | 26.5 | 3.09e-11 | 3.33e-08 | lm.nb | Adaptive Immune Response, Angiogenesis, Apoptosis, Astrocyte Function, Cell Cycle, Cytokine Signaling, Growth Factor Signaling, Inflammatory Signaling, Insulin Signaling, Microglia Function, NF-kB, Wnt | NS\_MM\_NEUROINFLAM\_V1.0 | Psmb8 | mRNA | 0 |  | Wnt;NF-kB;Microglia Function;Insulin Signaling;Inflammatory Signaling;Growth Factor Signaling;Cytokine Signaling;Cell Cycle;Astrocyte Function;Apoptosis;Angiogenesis;Adaptive Immune Response |  |  | Psmb8 | slateblue1 | 16 | 22.7748691099476 |
  | Irf1-mRNA | 3.51 | 0.198 | 3.12 | 3.9 | 11.4 | 8.72 | 14.9 | 5.78e-10 | 3.03e-07 | lm.nb | Apoptosis, Inflammatory Signaling, Innate Immune Response | NS\_MM\_NEUROINFLAM\_V1.0 | Irf1 | mRNA | 0 |  | Innate Immune Response;Inflammatory Signaling;Apoptosis | mmu04917;mmu05133;mmu05160 |  | Irf1 | slateblue1 | 16 | 17.7272727272727 |
  | Nfkbia-mRNA | 2.57 | 0.233 | 2.11 | 3.02 | 5.93 | 4.32 | 8.13 | 1.22e-07 | 1.74e-05 | lm.nb | Adaptive Immune Response, Apoptosis, Growth Factor Signaling, Inflammatory Signaling, Innate Immune Response, NF-kB | NS\_MM\_NEUROINFLAM\_V1.0 | Nfkbia | mRNA | 0 |  | NF-kB;Innate Immune Response;Inflammatory Signaling;Growth Factor Signaling;Apoptosis;Adaptive Immune Response | mmu04024;mmu04062;mmu04064;mmu04210;mmu04380;mmu04620;mmu04621;mmu04622;mmu04623;mmu04660;mmu04662;mmu04668;mmu04722;mmu04920;mmu04931;mmu05134;mmu05140;mmu05142;mmu05145;mmu05160;mmu05161;mmu05162;mmu05164;mmu05166;mmu05168;mmu05169;mmu05200;mmu05203;mmu05215;mmu05220;mmu05222 |  | Nfkbia | slateblue1 | 16 | 11.0300429184549 |
  | Cd14-mRNA | 6.51 | 0.637 | 5.26 | 7.76 | 90.9 | 38.3 | 216 | 2.85e-07 | 3.6e-05 | lm.nb | Apoptosis, Astrocyte Function, Autophagy, Growth Factor Signaling, Innate Immune Response, NF-kB | NS\_MM\_NEUROINFLAM\_V1.0 | Cd14 | mRNA | 0 |  | NF-kB;Innate Immune Response;Growth Factor Signaling;Autophagy;Astrocyte Function;Apoptosis | mmu04010;mmu04064;mmu04145;mmu04620;mmu04640;mmu04810;mmu05132;mmu05133;mmu05134;mmu05146;mmu05152;mmu05202 |  | Cd14 | slateblue1 | 16 | 10.2197802197802 |
  | Myc-mRNA | 1.37 | 0.14 | 1.1 | 1.65 | 2.59 | 2.14 | 3.13 | 4.35e-07 | 4.81e-05 | loglinear | Apoptosis, Cell Cycle, Cytokine Signaling, Growth Factor Signaling, Notch, Wnt | NS\_MM\_NEUROINFLAM\_V1.0 | Myc | mRNA | 0 |  | Wnt;Notch;Growth Factor Signaling;Cytokine Signaling;Cell Cycle;Apoptosis | mmu04010;mmu04012;mmu04110;mmu04151;mmu04310;mmu04350;mmu04390;mmu04550;mmu04630;mmu04919;mmu05161;mmu05166;mmu05169;mmu05200;mmu05202;mmu05205;mmu05206;mmu05210;mmu05213;mmu05216;mmu05219;mmu05220;mmu05221;mmu05222;mmu05230 |  | Myc | slateblue1 | 16 | 9.78571428571428 |
  | Bcl2a1a-mRNA | 4.25 | 0.435 | 3.4 | 5.11 | 19.1 | 10.6 | 34.4 | 4.59e-07 | 4.82e-05 | lm.nb | Apoptosis, NF-kB | NS\_MM\_NEUROINFLAM\_V1.0 | Bcl2a1a | mRNA | 0 |  | NF-kB;Apoptosis | mmu04064;mmu05202 |  | Bcl2a1a | slateblue1 | 16 | 9.77011494252874 |
  | Pik3r2-mRNA | -0.374 | 0.0389 | -0.451 | -0.298 | 0.771 | 0.732 | 0.813 | 5.36e-07 | 5.35e-05 | loglinear | Adaptive Immune Response, Angiogenesis, Apoptosis, Autophagy, Cytokine Signaling, Growth Factor Signaling, Innate Immune Response, Insulin Signaling | NS\_MM\_NEUROINFLAM\_V1.0 | Pik3r2 | mRNA | 0 |  | Insulin Signaling;Innate Immune Response;Growth Factor Signaling;Cytokine Signaling;Autophagy;Apoptosis;Angiogenesis;Adaptive Immune Response | mmu04012;mmu04014;mmu04015;mmu04022;mmu04024;mmu04062;mmu04066;mmu04068;mmu04070;mmu04071;mmu04072;mmu04150;mmu04151;mmu04152;mmu04210;mmu04211;mmu04213;mmu04261;mmu04370;mmu04380;mmu04510;mmu04550;mmu04611;mmu04620;mmu04630;mmu04650;mmu04660;mmu04662;mmu04664;mmu04666;mmu04668;mmu04670;mmu04722;mmu04725;mmu04750;mmu04810;mmu04910;mmu04914;mmu04915;mmu04917;mmu04919;mmu04921;mmu04923;mmu04930;mmu04931;mmu04932;mmu04933;mmu04960;mmu04973;mmu05100;mmu05142;mmu05145;mmu05146;mmu05160;mmu05161;mmu05162;mmu05164;mmu05166;mmu05169;mmu05200;mmu05203;mmu05205;mmu05210;mmu05211;mmu05212;mmu05213;mmu05214;mmu05215;mmu05218;mmu05220;mmu05221;mmu05222;mmu05223;mmu05230;mmu05231 |  | Pik3r2 | slateblue1 | 16 | -9.61439588688946 |
  | Myd88-mRNA | 1.86 | 0.204 | 1.46 | 2.25 | 3.62 | 2.74 | 4.77 | 9.7e-07 | 8.42e-05 | lm.nb | Apoptosis, Cytokine Signaling, Growth Factor Signaling, Inflammatory Signaling, Innate Immune Response, NF-kB | NS\_MM\_NEUROINFLAM\_V1.0 | Myd88 | mRNA | 0 |  | NF-kB;Innate Immune Response;Inflammatory Signaling;Growth Factor Signaling;Cytokine Signaling;Apoptosis | mmu04064;mmu04210;mmu04620;mmu05132;mmu05133;mmu05134;mmu05140;mmu05142;mmu05143;mmu05144;mmu05145;mmu05152;mmu05161;mmu05162;mmu05164;mmu05168 |  | Myd88 | slateblue1 | 16 | 9.11764705882353 |
  | Tubb3-mRNA | -0.448 | 0.0494 | -0.545 | -0.351 | 0.733 | 0.686 | 0.784 | 1.03e-06 | 8.78e-05 | loglinear | Adaptive Immune Response, Apoptosis, Cell Cycle, Neurons and Neurotransmission | NS\_MM\_NEUROINFLAM\_V1.0 | Tubb3 | mRNA | 0 |  | Neurons and Neurotransmission;Cell Cycle;Apoptosis;Adaptive Immune Response | mmu04145;mmu04540 |  | Tubb3 | slateblue1 | 16 | -9.06882591093117 |
  | Irf7-mRNA | 3.74 | 0.451 | 2.85 | 4.62 | 13.3 | 7.22 | 24.6 | 2.61e-06 | 0.000174 | lm.nb | Apoptosis, Inflammatory Signaling, Innate Immune Response | NS\_MM\_NEUROINFLAM\_V1.0 | Irf7 | mRNA | 0 |  | Innate Immune Response;Inflammatory Signaling;Apoptosis | mmu04620;mmu04622;mmu04623;mmu05160;mmu05161;mmu05162;mmu05164;mmu05168;mmu05203 |  | Irf7 | slateblue1 | 16 | 8.29268292682927 |
  | Birc3-mRNA | 3.52 | 0.444 | 2.65 | 4.39 | 11.5 | 6.29 | 21 | 4.05e-06 | 0.000261 | lm.nb | Apoptosis, Cytokine Signaling, Inflammatory Signaling, Innate Immune Response, NF-kB | NS\_MM\_NEUROINFLAM\_V1.0 | Birc3 | mRNA | 0 |  | NF-kB;Innate Immune Response;Inflammatory Signaling;Cytokine Signaling;Apoptosis | mmu04064;mmu04120;mmu04210;mmu04510;mmu04621;mmu04668;mmu05145;mmu05200;mmu05202;mmu05222 |  | Birc3 | slateblue1 | 16 | 7.92792792792793 |
  | Casp7-mRNA | 1.28 | 0.162 | 0.96 | 1.6 | 2.43 | 1.95 | 3.02 | 4.4e-06 | 0.000271 | lm.nb | Apoptosis, Innate Immune Response | NS\_MM\_NEUROINFLAM\_V1.0 | Casp7 | mRNA | 0 |  | Innate Immune Response;Apoptosis | mmu04210;mmu04668;mmu04932;mmu05010;mmu05133;mmu05134 |  | Casp7 | slateblue1 | 16 | 7.90123456790123 |
  | Tnfrsf1a-mRNA | 2.23 | 0.291 | 1.66 | 2.8 | 4.7 | 3.17 | 6.98 | 5.68e-06 | 0.000329 | lm.nb | Apoptosis, Cytokine Signaling, Growth Factor Signaling, Innate Immune Response, NF-kB | NS\_MM\_NEUROINFLAM\_V1.0 | Tnfrsf1a | mRNA | 0 |  | NF-kB;Innate Immune Response;Growth Factor Signaling;Cytokine Signaling;Apoptosis | mmu04010;mmu04060;mmu04064;mmu04071;mmu04210;mmu04380;mmu04668;mmu04920;mmu04931;mmu04932;mmu05010;mmu05014;mmu05142;mmu05145;mmu05152;mmu05160;mmu05164;mmu05166;mmu05168 |  | Tnfrsf1a | slateblue1 | 16 | 7.66323024054983 |
  | Il1r1-mRNA | 1.31 | 0.173 | 0.968 | 1.65 | 2.47 | 1.96 | 3.13 | 6.67e-06 | 0.00038 | lm.nb | Apoptosis, Cellular Stress, Cytokine Signaling, Growth Factor Signaling, Microglia Function, NF-kB | NS\_MM\_NEUROINFLAM\_V1.0 | Il1r1 | mRNA | 0 |  | NF-kB;Microglia Function;Growth Factor Signaling;Cytokine Signaling;Cellular Stress;Apoptosis | mmu04010;mmu04060;mmu04064;mmu04210;mmu04380;mmu04640;mmu04750;mmu05146;mmu05166 |  | Il1r1 | slateblue1 | 16 | 7.57225433526012 |
  | Fas-mRNA | 3.25 | 0.436 | 2.39 | 4.1 | 9.5 | 5.26 | 17.2 | 7.68e-06 | 0.00042 | lm.nb | Apoptosis, Cellular Stress, Cytokine Signaling, DNA Damage, Growth Factor Signaling, Innate Immune Response | NS\_MM\_NEUROINFLAM\_V1.0 | Fas | mRNA | 0 |  | Innate Immune Response;Growth Factor Signaling;DNA Damage;Cytokine Signaling;Cellular Stress;Apoptosis | mmu04010;mmu04060;mmu04115;mmu04210;mmu04650;mmu04668;mmu04932;mmu04940;mmu05010;mmu05142;mmu05143;mmu05161;mmu05162;mmu05164;mmu05168;mmu05200;mmu05205;mmu05320;mmu05330;mmu05332 |  | Fas | slateblue1 | 16 | 7.45412844036697 |
  | Vim-mRNA | 1.56 | 0.222 | 1.12 | 1.99 | 2.94 | 2.17 | 3.97 | 1.43e-05 | 0.000732 | lm.nb | Apoptosis, Astrocyte Function | NS\_MM\_NEUROINFLAM\_V1.0 | Vim | mRNA | 0 |  | Astrocyte Function;Apoptosis | mmu05169;mmu05206 |  | Vim | slateblue1 | 16 | 7.02702702702703 |
  | Il1a-mRNA | 6.35 | 0.919 | 4.55 | 8.15 | 81.3 | 23.3 | 283 | 1.63e-05 | 0.000805 | lm.nb | Apoptosis, Cellular Stress, Cytokine Signaling, Growth Factor Signaling | NS\_MM\_NEUROINFLAM\_V1.0 | Il1a | mRNA | 0 |  | Growth Factor Signaling;Cytokine Signaling;Cellular Stress;Apoptosis | mmu04010;mmu04060;mmu04210;mmu04380;mmu04640;mmu04932;mmu04933;mmu04940;mmu05020;mmu05132;mmu05133;mmu05140;mmu05152;mmu05162;mmu05164;mmu05321;mmu05323;mmu05332 |  | Il1a | slateblue1 | 16 | 6.90968443960827 |
  | Irak3-mRNA | 3.04 | 0.449 | 2.16 | 3.92 | 8.21 | 4.46 | 15.1 | 2e-05 | 0.000972 | lm.nb | Apoptosis, Cytokine Signaling, Innate Immune Response | NS\_MM\_NEUROINFLAM\_V1.0 | Irak3 | mRNA | 0 |  | Innate Immune Response;Cytokine Signaling;Apoptosis | mmu04210;mmu04722 |  | Irak3 | slateblue1 | 16 | 6.77060133630289 |
  | Dapk1-mRNA | 0.614 | 0.0918 | 0.434 | 0.794 | 1.53 | 1.35 | 1.73 | 2.24e-05 | 0.00106 | loglinear | Apoptosis, Autophagy | NS\_MM\_NEUROINFLAM\_V1.0 | Dapk1 | mRNA | 0 |  | Autophagy;Apoptosis | mmu05200;mmu05219 |  | Dapk1 | slateblue1 | 16 | 6.68845315904139 |
  | Cflar-mRNA | 1.32 | 0.208 | 0.915 | 1.73 | 2.5 | 1.89 | 3.32 | 3.58e-05 | 0.00164 | lm.nb | Apoptosis, Autophagy, Innate Immune Response, NF-kB | NS\_MM\_NEUROINFLAM\_V1.0 | Cflar | mRNA | 0 |  | NF-kB;Innate Immune Response;Autophagy;Apoptosis | mmu04064;mmu04210;mmu04668;mmu05142 |  | Cflar | slateblue1 | 16 | 6.34615384615385 |

  ##### DE Results - Apoptosis genes - DPI: 3 vs.CTRL

  Table displaying each sample's global significance scores and directed global significance scores as defined in the heatmaps above. The global significance score is calculated as the square root of the mean squared t-statistic for the genes in a gene set, with t-statistics coming from the linear regression underlying our differential expression analysis. The directed global significance score is calculated as the square root of the mean signed squared t-statistic for the genes in a gene set, with t-statistics coming from the linear regression underlying our differential expression analysis.

DPI:
- DPI: differential expression in 1 vs. baseline of CTRL
- DPI: differential expression in 2 vs. baseline of CTRL
- DPI: differential expression in 3 vs. baseline of CTRL

- **Volcano Plot: DPI: 1 vs.CTRL  
  More Plot Information**

  ##### Volcano Plot: DPI: 1 vs.CTRL

  Volcano plot displaying each gene's -log10(p-value) and log2 fold change for the selected covariate. Highly statistically significant genes fall at the top of the plot, and highly differentially expressed genes fall to either side. Genes within the selected gene set are highlighted in orange. Horizontal lines indicate various False Discovery Rate (FDR) thresholds.
- **DE Results - Astrocyte Function genes - DPI: 1 vs.CTRL  
  More Plot Information  Download CSV Data**

  | Probe Label | Log2 fold change | std error (log2) | Lower confidence limit (log2) | Upper confidence limit (log2) | Linear fold change | Lower confidence limit (linear) | Upper confidence limit (linear) | P-value | BY.p.value | method | Gene.sets | Codeset.Name | Probe.Label | Analyte.Type | Is.Control | Control.Type | Probe.Annotation | KEGG.Pathways | Cell.Type | Official.Gene.Name | volcanocol | volcanopch | tstats.all |
  | --- | --- | --- | --- | --- | --- | --- | --- | --- | --- | --- | --- | --- | --- | --- | --- | --- | --- | --- | --- | --- | --- | --- | --- |
  | Lcn2-mRNA | 6.98 | 0.503 | 6 | 7.97 | 127 | 63.9 | 251 | 9.34e-09 | 3.73e-05 | lm.nb | Astrocyte Function, Inflammatory Signaling | NS\_MM\_NEUROINFLAM\_V1.0 | Lcn2 | mRNA | 0 |  | Inflammatory Signaling;Astrocyte Function |  |  | Lcn2 | slateblue1 | 16 | 13.8767395626243 |
  | Cp-mRNA | 0.816 | 0.163 | 0.497 | 1.13 | 1.76 | 1.41 | 2.2 | 0.000303 | 0.125 | lm.nb | Astrocyte Function | NS\_MM\_NEUROINFLAM\_V1.0 | Cp | mRNA | 0 |  | Astrocyte Function |  |  | Cp | slateblue4 | 16 | 5.00613496932515 |
  | Osmr-mRNA | 1.68 | 0.337 | 1.02 | 2.34 | 3.21 | 2.03 | 5.07 | 0.000313 | 0.125 | lm.nb | Astrocyte Function, Cytokine Signaling, Growth Factor Signaling | NS\_MM\_NEUROINFLAM\_V1.0 | Osmr | mRNA | 0 |  | Growth Factor Signaling;Cytokine Signaling;Astrocyte Function | mmu04060;mmu04151;mmu04630 |  | Osmr | slateblue4 | 16 | 4.98516320474777 |
  | Ccl2-mRNA | 4.15 | 0.972 | 2.24 | 6.05 | 17.8 | 4.74 | 66.5 | 0.00109 | 0.335 | lm.nb | Astrocyte Function, Cytokine Signaling, Inflammatory Signaling, Innate Immune Response | NS\_MM\_NEUROINFLAM\_V1.0 | Ccl2 | mRNA | 0 |  | Innate Immune Response;Inflammatory Signaling;Cytokine Signaling;Astrocyte Function | mmu04060;mmu04062;mmu04621;mmu04668;mmu04933;mmu05142;mmu05144;mmu05164;mmu05168;mmu05323 | DC | Ccl2 | slateblue4 | 16 | 4.26954732510288 |
  | Grm3-mRNA | -0.323 | 0.0825 | -0.484 | -0.161 | 0.8 | 0.715 | 0.894 | 0.00206 | 0.437 | loglinear | Astrocyte Function, Neurons and Neurotransmission | NS\_MM\_NEUROINFLAM\_V1.0 | Grm3 | mRNA | 0 |  | Neurons and Neurotransmission;Astrocyte Function | mmu04072;mmu04080;mmu04724;mmu05030 |  | Grm3 | slateblue4 | 16 | -3.91515151515152 |
  | Cd14-mRNA | 2.57 | 0.657 | 1.28 | 3.85 | 5.92 | 2.43 | 14.5 | 0.00208 | 0.437 | lm.nb | Apoptosis, Astrocyte Function, Autophagy, Growth Factor Signaling, Innate Immune Response, NF-kB | NS\_MM\_NEUROINFLAM\_V1.0 | Cd14 | mRNA | 0 |  | NF-kB;Innate Immune Response;Growth Factor Signaling;Autophagy;Astrocyte Function;Apoptosis | mmu04010;mmu04064;mmu04145;mmu04620;mmu04640;mmu04810;mmu05132;mmu05133;mmu05134;mmu05146;mmu05152;mmu05202 |  | Cd14 | slateblue4 | 16 | 3.9117199391172 |
  | Fkbp5-mRNA | 0.453 | 0.124 | 0.209 | 0.697 | 1.37 | 1.16 | 1.62 | 0.00335 | 0.558 | loglinear | Astrocyte Function | NS\_MM\_NEUROINFLAM\_V1.0 | Fkbp5 | mRNA | 0 |  | Astrocyte Function | mmu04915 |  | Fkbp5 | azure4 | 1 | 3.65322580645161 |
  | Aldh1l1-mRNA | 0.291 | 0.0861 | 0.123 | 0.46 | 1.22 | 1.09 | 1.38 | 0.00542 | 0.637 | loglinear | Astrocyte Function | NS\_MM\_NEUROINFLAM\_V1.0 | Aldh1l1 | mRNA | 0 |  | Astrocyte Function |  | Astrocytes | Aldh1l1 | azure4 | 1 | 3.37979094076655 |
  | Vim-mRNA | 0.712 | 0.223 | 0.275 | 1.15 | 1.64 | 1.21 | 2.22 | 0.00772 | 0.76 | lm.nb | Apoptosis, Astrocyte Function | NS\_MM\_NEUROINFLAM\_V1.0 | Vim | mRNA | 0 |  | Astrocyte Function;Apoptosis | mmu05169;mmu05206 |  | Vim | azure4 | 1 | 3.19282511210762 |
  | Serping1-mRNA | 0.624 | 0.205 | 0.222 | 1.03 | 1.54 | 1.17 | 2.04 | 0.0103 | 0.861 | lm.nb | Astrocyte Function | NS\_MM\_NEUROINFLAM\_V1.0 | Serping1 | mRNA | 0 |  | Astrocyte Function | mmu04610;mmu05133 |  | Serping1 | azure4 | 1 | 3.04390243902439 |
  | S100b-mRNA | 0.307 | 0.109 | 0.0927 | 0.521 | 1.24 | 1.07 | 1.44 | 0.0158 | 1 | lm.nb | Astrocyte Function, Growth Factor Signaling, Inflammatory Signaling, Innate Immune Response | NS\_MM\_NEUROINFLAM\_V1.0 | S100b | mRNA | 0 |  | Innate Immune Response;Inflammatory Signaling;Growth Factor Signaling;Astrocyte Function |  |  | S100b | azure4 | 1 | 2.81651376146789 |
  | Gbp2-mRNA | 0.742 | 0.303 | 0.148 | 1.34 | 1.67 | 1.11 | 2.52 | 0.0308 | 1 | lm.nb | Astrocyte Function, Inflammatory Signaling | NS\_MM\_NEUROINFLAM\_V1.0 | Gbp2 | mRNA | 0 |  | Inflammatory Signaling;Astrocyte Function |  |  | Gbp2 | azure4 | 1 | 2.44884488448845 |
  | Apc-mRNA | -0.18 | 0.0782 | -0.333 | -0.0269 | 0.883 | 0.794 | 0.982 | 0.0399 | 1 | loglinear | Apoptosis, Astrocyte Function, Wnt | NS\_MM\_NEUROINFLAM\_V1.0 | Apc | mRNA | 0 |  | Wnt;Astrocyte Function;Apoptosis | mmu04310;mmu04390;mmu04550;mmu04810;mmu05166;mmu05200;mmu05206;mmu05210;mmu05213 |  | Apc | azure4 | 1 | -2.30179028132992 |
  | Serpina3n-mRNA | 0.637 | 0.321 | 0.00874 | 1.27 | 1.56 | 1.01 | 2.41 | 0.0702 | 1 | lm.nb | Astrocyte Function | NS\_MM\_NEUROINFLAM\_V1.0 | Serpina3n | mRNA | 0 |  | Astrocyte Function |  |  | Serpina3n | azure4 | 1 | 1.98442367601246 |
  | B3gnt5-mRNA | 1.07 | 0.598 | -0.105 | 2.24 | 2.1 | 0.93 | 4.72 | 0.0995 | 1 | lm.nb | Astrocyte Function | NS\_MM\_NEUROINFLAM\_V1.0 | B3gnt5 | mRNA | 0 |  | Astrocyte Function |  |  | B3gnt5 | azure4 | 1 | 1.78929765886288 |
  | Slc1a3-mRNA | 0.177 | 0.107 | -0.0324 | 0.386 | 1.13 | 0.978 | 1.31 | 0.124 | 1 | loglinear | Astrocyte Function, Neurons and Neurotransmission | NS\_MM\_NEUROINFLAM\_V1.0 | Slc1a3 | mRNA | 0 |  | Neurons and Neurotransmission;Astrocyte Function | mmu04724 |  | Slc1a3 | azure4 | 1 | 1.65420560747664 |
  | Sox9-mRNA | 0.191 | 0.124 | -0.0519 | 0.433 | 1.14 | 0.965 | 1.35 | 0.149 | 1 | loglinear | Astrocyte Function, Wnt | NS\_MM\_NEUROINFLAM\_V1.0 | Sox9 | mRNA | 0 |  | Wnt;Astrocyte Function | mmu04024 | Astrocytes | Sox9 | azure4 | 1 | 1.54032258064516 |
  | Psmb8-mRNA | 0.333 | 0.226 | -0.11 | 0.775 | 1.26 | 0.927 | 1.71 | 0.166 | 1 | lm.nb | Adaptive Immune Response, Angiogenesis, Apoptosis, Astrocyte Function, Cell Cycle, Cytokine Signaling, Growth Factor Signaling, Inflammatory Signaling, Insulin Signaling, Microglia Function, NF-kB, Wnt | NS\_MM\_NEUROINFLAM\_V1.0 | Psmb8 | mRNA | 0 |  | Wnt;NF-kB;Microglia Function;Insulin Signaling;Inflammatory Signaling;Growth Factor Signaling;Cytokine Signaling;Cell Cycle;Astrocyte Function;Apoptosis;Angiogenesis;Adaptive Immune Response |  |  | Psmb8 | azure4 | 1 | 1.47345132743363 |
  | Hspb1-mRNA | 0.338 | 0.236 | -0.124 | 0.801 | 1.26 | 0.918 | 1.74 | 0.177 | 1 | lm.nb | Angiogenesis, Astrocyte Function, Cellular Stress, Growth Factor Signaling | NS\_MM\_NEUROINFLAM\_V1.0 | Hspb1 | mRNA | 0 |  | Growth Factor Signaling;Cellular Stress;Astrocyte Function;Angiogenesis | mmu04010;mmu04370;mmu05146;mmu05169 |  | Hspb1 | azure4 | 1 | 1.43220338983051 |
  | C4a-mRNA | 0.193 | 0.163 | -0.126 | 0.512 | 1.14 | 0.916 | 1.43 | 0.26 | 1 | lm.nb | Astrocyte Function | NS\_MM\_NEUROINFLAM\_V1.0 | C4a | mRNA | 0 |  | Astrocyte Function |  |  | C4a | azure4 | 1 | 1.1840490797546 |

  ##### DE Results - Astrocyte Function genes - DPI: 1 vs.CTRL

  Table displaying each sample's global significance scores and directed global significance scores as defined in the heatmaps above. The global significance score is calculated as the square root of the mean squared t-statistic for the genes in a gene set, with t-statistics coming from the linear regression underlying our differential expression analysis. The directed global significance score is calculated as the square root of the mean signed squared t-statistic for the genes in a gene set, with t-statistics coming from the linear regression underlying our differential expression analysis.

- **Volcano Plot: DPI: 2 vs.CTRL  
  More Plot Information**

  ##### Volcano Plot: DPI: 2 vs.CTRL

  Volcano plot displaying each gene's -log10(p-value) and log2 fold change for the selected covariate. Highly statistically significant genes fall at the top of the plot, and highly differentially expressed genes fall to either side. Genes within the selected gene set are highlighted in orange. Horizontal lines indicate various False Discovery Rate (FDR) thresholds.
- **DE Results - Astrocyte Function genes - DPI: 2 vs.CTRL  
  More Plot Information  Download CSV Data**

  | Probe Label | Log2 fold change | std error (log2) | Lower confidence limit (log2) | Upper confidence limit (log2) | Linear fold change | Lower confidence limit (linear) | Upper confidence limit (linear) | P-value | BY.p.value | method | Gene.sets | Codeset.Name | Probe.Label | Analyte.Type | Is.Control | Control.Type | Probe.Annotation | KEGG.Pathways | Cell.Type | Official.Gene.Name | volcanocol | volcanopch | tstats.all |
  | --- | --- | --- | --- | --- | --- | --- | --- | --- | --- | --- | --- | --- | --- | --- | --- | --- | --- | --- | --- | --- | --- | --- | --- |
  | Lcn2-mRNA | 9.93 | 0.502 | 8.94 | 10.9 | 972 | 492 | 1920 | 1.58e-10 | 3.57e-07 | lm.nb | Astrocyte Function, Inflammatory Signaling | NS\_MM\_NEUROINFLAM\_V1.0 | Lcn2 | mRNA | 0 |  | Inflammatory Signaling;Astrocyte Function |  |  | Lcn2 | slateblue1 | 16 | 19.7808764940239 |
  | Gbp2-mRNA | 5.23 | 0.267 | 4.71 | 5.76 | 37.6 | 26.2 | 54.1 | 1.79e-10 | 3.57e-07 | lm.nb | Astrocyte Function, Inflammatory Signaling | NS\_MM\_NEUROINFLAM\_V1.0 | Gbp2 | mRNA | 0 |  | Inflammatory Signaling;Astrocyte Function |  |  | Gbp2 | slateblue1 | 16 | 19.5880149812734 |
  | Fkbp5-mRNA | 1.85 | 0.124 | 1.61 | 2.1 | 3.61 | 3.05 | 4.27 | 4.19e-09 | 3.71e-06 | loglinear | Astrocyte Function | NS\_MM\_NEUROINFLAM\_V1.0 | Fkbp5 | mRNA | 0 |  | Astrocyte Function | mmu04915 |  | Fkbp5 | slateblue1 | 16 | 14.9193548387097 |
  | Psmb8-mRNA | 2.78 | 0.196 | 2.39 | 3.16 | 6.85 | 5.25 | 8.94 | 7.51e-09 | 4.29e-06 | lm.nb | Adaptive Immune Response, Angiogenesis, Apoptosis, Astrocyte Function, Cell Cycle, Cytokine Signaling, Growth Factor Signaling, Inflammatory Signaling, Insulin Signaling, Microglia Function, NF-kB, Wnt | NS\_MM\_NEUROINFLAM\_V1.0 | Psmb8 | mRNA | 0 |  | Wnt;NF-kB;Microglia Function;Insulin Signaling;Inflammatory Signaling;Growth Factor Signaling;Cytokine Signaling;Cell Cycle;Astrocyte Function;Apoptosis;Angiogenesis;Adaptive Immune Response |  |  | Psmb8 | slateblue1 | 16 | 14.1836734693878 |
  | Cp-mRNA | 2.04 | 0.156 | 1.73 | 2.34 | 4.11 | 3.33 | 5.08 | 1.8e-08 | 8e-06 | lm.nb | Astrocyte Function | NS\_MM\_NEUROINFLAM\_V1.0 | Cp | mRNA | 0 |  | Astrocyte Function |  |  | Cp | slateblue1 | 16 | 13.0769230769231 |
  | C4a-mRNA | 1.63 | 0.152 | 1.33 | 1.92 | 3.09 | 2.51 | 3.8 | 1.77e-07 | 5.06e-05 | lm.nb | Astrocyte Function | NS\_MM\_NEUROINFLAM\_V1.0 | C4a | mRNA | 0 |  | Astrocyte Function |  |  | C4a | slateblue1 | 16 | 10.7236842105263 |
  | Cxcl10-mRNA | 6.62 | 0.741 | 5.17 | 8.07 | 98.6 | 36 | 270 | 1.18e-06 | 0.000256 | lm.nb | Astrocyte Function, Cytokine Signaling, Inflammatory Signaling, Innate Immune Response, Microglia Function | NS\_MM\_NEUROINFLAM\_V1.0 | Cxcl10 | mRNA | 0 |  | Microglia Function;Innate Immune Response;Inflammatory Signaling;Cytokine Signaling;Astrocyte Function | mmu04060;mmu04062;mmu04620;mmu04622;mmu04623;mmu04668;mmu05164 |  | Cxcl10 | slateblue1 | 16 | 8.93387314439946 |
  | Serping1-mRNA | 1.72 | 0.194 | 1.34 | 2.1 | 3.29 | 2.53 | 4.29 | 1.29e-06 | 0.000258 | lm.nb | Astrocyte Function | NS\_MM\_NEUROINFLAM\_V1.0 | Serping1 | mRNA | 0 |  | Astrocyte Function | mmu04610;mmu05133 |  | Serping1 | slateblue1 | 16 | 8.8659793814433 |
  | Osmr-mRNA | 2.76 | 0.326 | 2.12 | 3.4 | 6.77 | 4.35 | 10.5 | 2.1e-06 | 0.000373 | lm.nb | Astrocyte Function, Cytokine Signaling, Growth Factor Signaling | NS\_MM\_NEUROINFLAM\_V1.0 | Osmr | mRNA | 0 |  | Growth Factor Signaling;Cytokine Signaling;Astrocyte Function | mmu04060;mmu04151;mmu04630 |  | Osmr | slateblue1 | 16 | 8.46625766871166 |
  | Ccl2-mRNA | 7.94 | 0.958 | 6.06 | 9.81 | 245 | 66.6 | 899 | 2.63e-06 | 0.00042 | lm.nb | Astrocyte Function, Cytokine Signaling, Inflammatory Signaling, Innate Immune Response | NS\_MM\_NEUROINFLAM\_V1.0 | Ccl2 | mRNA | 0 |  | Innate Immune Response;Inflammatory Signaling;Cytokine Signaling;Astrocyte Function | mmu04060;mmu04062;mmu04621;mmu04668;mmu04933;mmu05142;mmu05144;mmu05164;mmu05168;mmu05323 | DC | Ccl2 | slateblue1 | 16 | 8.28810020876827 |
  | Serpina3n-mRNA | 2.31 | 0.308 | 1.7 | 2.91 | 4.95 | 3.26 | 7.52 | 7.4e-06 | 0.000953 | lm.nb | Astrocyte Function | NS\_MM\_NEUROINFLAM\_V1.0 | Serpina3n | mRNA | 0 |  | Astrocyte Function |  |  | Serpina3n | slateblue1 | 16 | 7.5 |
  | Cd14-mRNA | 4.52 | 0.641 | 3.26 | 5.78 | 22.9 | 9.59 | 54.8 | 1.34e-05 | 0.00162 | lm.nb | Apoptosis, Astrocyte Function, Autophagy, Growth Factor Signaling, Innate Immune Response, NF-kB | NS\_MM\_NEUROINFLAM\_V1.0 | Cd14 | mRNA | 0 |  | NF-kB;Innate Immune Response;Growth Factor Signaling;Autophagy;Astrocyte Function;Apoptosis | mmu04010;mmu04064;mmu04145;mmu04620;mmu04640;mmu04810;mmu05132;mmu05133;mmu05134;mmu05146;mmu05152;mmu05202 |  | Cd14 | slateblue1 | 16 | 7.05148205928237 |
  | Aldh1l1-mRNA | 0.54 | 0.0861 | 0.371 | 0.709 | 1.45 | 1.29 | 1.63 | 4.12e-05 | 0.00402 | loglinear | Astrocyte Function | NS\_MM\_NEUROINFLAM\_V1.0 | Aldh1l1 | mRNA | 0 |  | Astrocyte Function |  | Astrocytes | Aldh1l1 | slateblue1 | 16 | 6.27177700348432 |
  | Grm3-mRNA | -0.48 | 0.0825 | -0.642 | -0.318 | 0.717 | 0.641 | 0.802 | 8.23e-05 | 0.00609 | loglinear | Astrocyte Function, Neurons and Neurotransmission | NS\_MM\_NEUROINFLAM\_V1.0 | Grm3 | mRNA | 0 |  | Neurons and Neurotransmission;Astrocyte Function | mmu04072;mmu04080;mmu04724;mmu05030 |  | Grm3 | slateblue1 | 16 | -5.81818181818182 |
  | Ptgs2-mRNA | 1.09 | 0.194 | 0.713 | 1.47 | 2.13 | 1.64 | 2.78 | 0.00011 | 0.00772 | lm.nb | Astrocyte Function, Cellular Stress, Innate Immune Response, Lipid Metabolism, Microglia Function, NF-kB | NS\_MM\_NEUROINFLAM\_V1.0 | Ptgs2 | mRNA | 0 |  | NF-kB;Microglia Function;Lipid Metabolism;Innate Immune Response;Cellular Stress;Astrocyte Function | mmu00590;mmu04064;mmu04370;mmu04668;mmu04723;mmu04726;mmu04913;mmu04921;mmu04923;mmu05140;mmu05200;mmu05206;mmu05222 |  | Ptgs2 | slateblue1 | 16 | 5.61855670103093 |
  | Agt-mRNA | 1.39 | 0.249 | 0.907 | 1.88 | 2.63 | 1.88 | 3.69 | 0.000115 | 0.00789 | lm.nb | Astrocyte Function | NS\_MM\_NEUROINFLAM\_V1.0 | Agt | mRNA | 0 |  | Astrocyte Function | mmu04924 |  | Agt | slateblue1 | 16 | 5.58232931726908 |
  | Srgn-mRNA | 2.27 | 0.464 | 1.36 | 3.18 | 4.82 | 2.56 | 9.06 | 0.000376 | 0.0215 | lm.nb | Astrocyte Function, Inflammatory Signaling | NS\_MM\_NEUROINFLAM\_V1.0 | Srgn | mRNA | 0 |  | Inflammatory Signaling;Astrocyte Function |  |  | Srgn | slateblue2 | 16 | 4.89224137931034 |
  | Tgfa-mRNA | -0.548 | 0.115 | -0.772 | -0.323 | 0.684 | 0.585 | 0.799 | 0.000447 | 0.0232 | loglinear | Astrocyte Function, Microglia Function, Neurons and Neurotransmission | NS\_MM\_NEUROINFLAM\_V1.0 | Tgfa | mRNA | 0 |  | Neurons and Neurotransmission;Microglia Function;Astrocyte Function | mmu04012;mmu05200;mmu05211;mmu05212;mmu05214;mmu05215;mmu05223 |  | Tgfa | slateblue2 | 16 | -4.76521739130435 |
  | Padi2-mRNA | -1.07 | 0.235 | -1.53 | -0.606 | 0.478 | 0.347 | 0.657 | 0.000682 | 0.032 | lm.nb | Astrocyte Function, Epigenetic Regulation | NS\_MM\_NEUROINFLAM\_V1.0 | Padi2 | mRNA | 0 |  | Epigenetic Regulation;Astrocyte Function |  |  | Padi2 | slateblue2 | 16 | -4.5531914893617 |
  | Fbln5-mRNA | 1.08 | 0.259 | 0.57 | 1.59 | 2.11 | 1.48 | 3 | 0.00132 | 0.0526 | lm.nb | Astrocyte Function, Matrix Remodeling | NS\_MM\_NEUROINFLAM\_V1.0 | Fbln5 | mRNA | 0 |  | Matrix Remodeling;Astrocyte Function |  |  | Fbln5 | slateblue3 | 16 | 4.16988416988417 |

  ##### DE Results - Astrocyte Function genes - DPI: 2 vs.CTRL

  Table displaying each sample's global significance scores and directed global significance scores as defined in the heatmaps above. The global significance score is calculated as the square root of the mean squared t-statistic for the genes in a gene set, with t-statistics coming from the linear regression underlying our differential expression analysis. The directed global significance score is calculated as the square root of the mean signed squared t-statistic for the genes in a gene set, with t-statistics coming from the linear regression underlying our differential expression analysis.

- **Volcano Plot: DPI: 3 vs.CTRL  
  More Plot Information**

  ##### Volcano Plot: DPI: 3 vs.CTRL

  Volcano plot displaying each gene's -log10(p-value) and log2 fold change for the selected covariate. Highly statistically significant genes fall at the top of the plot, and highly differentially expressed genes fall to either side. Genes within the selected gene set are highlighted in orange. Horizontal lines indicate various False Discovery Rate (FDR) thresholds.
- **DE Results - Astrocyte Function genes - DPI: 3 vs.CTRL  
  More Plot Information  Download CSV Data**

  | Probe Label | Log2 fold change | std error (log2) | Lower confidence limit (log2) | Upper confidence limit (log2) | Linear fold change | Lower confidence limit (linear) | Upper confidence limit (linear) | P-value | BY.p.value | method | Gene.sets | Codeset.Name | Probe.Label | Analyte.Type | Is.Control | Control.Type | Probe.Annotation | KEGG.Pathways | Cell.Type | Official.Gene.Name | volcanocol | volcanopch | tstats.all |
  | --- | --- | --- | --- | --- | --- | --- | --- | --- | --- | --- | --- | --- | --- | --- | --- | --- | --- | --- | --- | --- | --- | --- | --- |
  | Gbp2-mRNA | 6.49 | 0.266 | 5.97 | 7.01 | 89.9 | 62.6 | 129 | 1.37e-11 | 2.73e-08 | lm.nb | Astrocyte Function, Inflammatory Signaling | NS\_MM\_NEUROINFLAM\_V1.0 | Gbp2 | mRNA | 0 |  | Inflammatory Signaling;Astrocyte Function |  |  | Gbp2 | slateblue1 | 16 | 24.3984962406015 |
  | Psmb8-mRNA | 4.35 | 0.191 | 3.98 | 4.73 | 20.4 | 15.7 | 26.5 | 3.09e-11 | 3.33e-08 | lm.nb | Adaptive Immune Response, Angiogenesis, Apoptosis, Astrocyte Function, Cell Cycle, Cytokine Signaling, Growth Factor Signaling, Inflammatory Signaling, Insulin Signaling, Microglia Function, NF-kB, Wnt | NS\_MM\_NEUROINFLAM\_V1.0 | Psmb8 | mRNA | 0 |  | Wnt;NF-kB;Microglia Function;Insulin Signaling;Inflammatory Signaling;Growth Factor Signaling;Cytokine Signaling;Cell Cycle;Astrocyte Function;Apoptosis;Angiogenesis;Adaptive Immune Response |  |  | Psmb8 | slateblue1 | 16 | 22.7748691099476 |
  | Cp-mRNA | 3.43 | 0.152 | 3.14 | 3.73 | 10.8 | 8.79 | 13.3 | 3.4e-11 | 3.33e-08 | lm.nb | Astrocyte Function | NS\_MM\_NEUROINFLAM\_V1.0 | Cp | mRNA | 0 |  | Astrocyte Function |  |  | Cp | slateblue1 | 16 | 22.5657894736842 |
  | Lcn2-mRNA | 11.1 | 0.501 | 10.1 | 12.1 | 2230 | 1130 | 4400 | 4.16e-11 | 3.33e-08 | lm.nb | Astrocyte Function, Inflammatory Signaling | NS\_MM\_NEUROINFLAM\_V1.0 | Lcn2 | mRNA | 0 |  | Inflammatory Signaling;Astrocyte Function |  |  | Lcn2 | slateblue1 | 16 | 22.1556886227545 |
  | C4a-mRNA | 2.97 | 0.148 | 2.68 | 3.26 | 7.83 | 6.4 | 9.58 | 1.4e-10 | 9.31e-08 | lm.nb | Astrocyte Function | NS\_MM\_NEUROINFLAM\_V1.0 | C4a | mRNA | 0 |  | Astrocyte Function |  |  | C4a | slateblue1 | 16 | 20.0675675675676 |
  | Fkbp5-mRNA | 2.19 | 0.124 | 1.95 | 2.43 | 4.56 | 3.86 | 5.4 | 6.07e-10 | 3.03e-07 | loglinear | Astrocyte Function | NS\_MM\_NEUROINFLAM\_V1.0 | Fkbp5 | mRNA | 0 |  | Astrocyte Function | mmu04915 |  | Fkbp5 | slateblue1 | 16 | 17.6612903225806 |
  | Serping1-mRNA | 2.83 | 0.188 | 2.46 | 3.2 | 7.1 | 5.49 | 9.16 | 3.84e-09 | 1.39e-06 | lm.nb | Astrocyte Function | NS\_MM\_NEUROINFLAM\_V1.0 | Serping1 | mRNA | 0 |  | Astrocyte Function | mmu04610;mmu05133 |  | Serping1 | slateblue1 | 16 | 15.0531914893617 |
  | Serpina3n-mRNA | 4.42 | 0.304 | 3.83 | 5.02 | 21.5 | 14.2 | 32.4 | 5.44e-09 | 1.67e-06 | lm.nb | Astrocyte Function | NS\_MM\_NEUROINFLAM\_V1.0 | Serpina3n | mRNA | 0 |  | Astrocyte Function |  |  | Serpina3n | slateblue1 | 16 | 14.5394736842105 |
  | Osmr-mRNA | 4.15 | 0.32 | 3.52 | 4.78 | 17.8 | 11.5 | 27.4 | 2.02e-08 | 4.49e-06 | lm.nb | Astrocyte Function, Cytokine Signaling, Growth Factor Signaling | NS\_MM\_NEUROINFLAM\_V1.0 | Osmr | mRNA | 0 |  | Growth Factor Signaling;Cytokine Signaling;Astrocyte Function | mmu04060;mmu04151;mmu04630 |  | Osmr | slateblue1 | 16 | 12.96875 |
  | Cxcl10-mRNA | 9.44 | 0.739 | 7.99 | 10.9 | 693 | 254 | 1890 | 2.42e-08 | 4.84e-06 | lm.nb | Astrocyte Function, Cytokine Signaling, Inflammatory Signaling, Innate Immune Response, Microglia Function | NS\_MM\_NEUROINFLAM\_V1.0 | Cxcl10 | mRNA | 0 |  | Microglia Function;Innate Immune Response;Inflammatory Signaling;Cytokine Signaling;Astrocyte Function | mmu04060;mmu04062;mmu04620;mmu04622;mmu04623;mmu04668;mmu05164 |  | Cxcl10 | slateblue1 | 16 | 12.7740189445196 |
  | Ccl2-mRNA | 10.8 | 0.957 | 8.91 | 12.7 | 1770 | 483 | 6500 | 9.65e-08 | 1.48e-05 | lm.nb | Astrocyte Function, Cytokine Signaling, Inflammatory Signaling, Innate Immune Response | NS\_MM\_NEUROINFLAM\_V1.0 | Ccl2 | mRNA | 0 |  | Innate Immune Response;Inflammatory Signaling;Cytokine Signaling;Astrocyte Function | mmu04060;mmu04062;mmu04621;mmu04668;mmu04933;mmu05142;mmu05144;mmu05164;mmu05168;mmu05323 | DC | Ccl2 | slateblue1 | 16 | 11.2852664576803 |
  | Hspb1-mRNA | 2.5 | 0.227 | 2.05 | 2.95 | 5.66 | 4.15 | 7.7 | 1.27e-07 | 1.76e-05 | lm.nb | Angiogenesis, Astrocyte Function, Cellular Stress, Growth Factor Signaling | NS\_MM\_NEUROINFLAM\_V1.0 | Hspb1 | mRNA | 0 |  | Growth Factor Signaling;Cellular Stress;Astrocyte Function;Angiogenesis | mmu04010;mmu04370;mmu05146;mmu05169 |  | Hspb1 | slateblue1 | 16 | 11.0132158590308 |
  | Cd14-mRNA | 6.51 | 0.637 | 5.26 | 7.76 | 90.9 | 38.3 | 216 | 2.85e-07 | 3.6e-05 | lm.nb | Apoptosis, Astrocyte Function, Autophagy, Growth Factor Signaling, Innate Immune Response, NF-kB | NS\_MM\_NEUROINFLAM\_V1.0 | Cd14 | mRNA | 0 |  | NF-kB;Innate Immune Response;Growth Factor Signaling;Autophagy;Astrocyte Function;Apoptosis | mmu04010;mmu04064;mmu04145;mmu04620;mmu04640;mmu04810;mmu05132;mmu05133;mmu05134;mmu05146;mmu05152;mmu05202 |  | Cd14 | slateblue1 | 16 | 10.2197802197802 |
  | Amigo2-mRNA | 0.915 | 0.0919 | 0.734 | 1.09 | 1.89 | 1.66 | 2.14 | 3.79e-07 | 4.49e-05 | loglinear | Astrocyte Function | NS\_MM\_NEUROINFLAM\_V1.0 | Amigo2 | mRNA | 0 |  | Astrocyte Function |  |  | Amigo2 | slateblue1 | 16 | 9.95647442872688 |
  | Ptgs2-mRNA | 1.88 | 0.191 | 1.5 | 2.25 | 3.67 | 2.83 | 4.76 | 4.46e-07 | 4.81e-05 | lm.nb | Astrocyte Function, Cellular Stress, Innate Immune Response, Lipid Metabolism, Microglia Function, NF-kB | NS\_MM\_NEUROINFLAM\_V1.0 | Ptgs2 | mRNA | 0 |  | NF-kB;Microglia Function;Lipid Metabolism;Innate Immune Response;Cellular Stress;Astrocyte Function | mmu00590;mmu04064;mmu04370;mmu04668;mmu04723;mmu04726;mmu04913;mmu04921;mmu04923;mmu05140;mmu05200;mmu05206;mmu05222 |  | Ptgs2 | slateblue1 | 16 | 9.84293193717278 |
  | S1pr3-mRNA | 1.63 | 0.178 | 1.28 | 1.98 | 3.1 | 2.43 | 3.95 | 9.05e-07 | 8.22e-05 | lm.nb | Astrocyte Function, Neurons and Neurotransmission | NS\_MM\_NEUROINFLAM\_V1.0 | S1pr3 | mRNA | 0 |  | Neurons and Neurotransmission;Astrocyte Function | mmu04071;mmu04080 |  | S1pr3 | slateblue1 | 16 | 9.15730337078652 |
  | Csf1-mRNA | 1.21 | 0.132 | 0.948 | 1.47 | 2.31 | 1.93 | 2.76 | 9.38e-07 | 8.32e-05 | lm.nb | Astrocyte Function, Autophagy, Cytokine Signaling, Growth Factor Signaling, Innate Immune Response, Microglia Function | NS\_MM\_NEUROINFLAM\_V1.0 | Csf1 | mRNA | 0 |  | Microglia Function;Innate Immune Response;Growth Factor Signaling;Cytokine Signaling;Autophagy;Astrocyte Function | mmu04014;mmu04015;mmu04060;mmu04151;mmu04380;mmu04640;mmu04668;mmu05323 |  | Csf1 | slateblue1 | 16 | 9.16666666666667 |
  | Srgn-mRNA | 3.94 | 0.463 | 3.03 | 4.84 | 15.3 | 8.17 | 28.7 | 1.99e-06 | 0.000142 | lm.nb | Astrocyte Function, Inflammatory Signaling | NS\_MM\_NEUROINFLAM\_V1.0 | Srgn | mRNA | 0 |  | Inflammatory Signaling;Astrocyte Function |  |  | Srgn | slateblue1 | 16 | 8.5097192224622 |
  | Tm4sf1-mRNA | 1.63 | 0.229 | 1.18 | 2.07 | 3.08 | 2.26 | 4.21 | 1.25e-05 | 0.000657 | lm.nb | Astrocyte Function | NS\_MM\_NEUROINFLAM\_V1.0 | Tm4sf1 | mRNA | 0 |  | Astrocyte Function |  |  | Tm4sf1 | slateblue1 | 16 | 7.117903930131 |
  | Vim-mRNA | 1.56 | 0.222 | 1.12 | 1.99 | 2.94 | 2.17 | 3.97 | 1.43e-05 | 0.000732 | lm.nb | Apoptosis, Astrocyte Function | NS\_MM\_NEUROINFLAM\_V1.0 | Vim | mRNA | 0 |  | Astrocyte Function;Apoptosis | mmu05169;mmu05206 |  | Vim | slateblue1 | 16 | 7.02702702702703 |

  ##### DE Results - Astrocyte Function genes - DPI: 3 vs.CTRL

  Table displaying each sample's global significance scores and directed global significance scores as defined in the heatmaps above. The global significance score is calculated as the square root of the mean squared t-statistic for the genes in a gene set, with t-statistics coming from the linear regression underlying our differential expression analysis. The directed global significance score is calculated as the square root of the mean signed squared t-statistic for the genes in a gene set, with t-statistics coming from the linear regression underlying our differential expression analysis.

DPI:
- DPI: differential expression in 1 vs. baseline of CTRL
- DPI: differential expression in 2 vs. baseline of CTRL
- DPI: differential expression in 3 vs. baseline of CTRL

- **Volcano Plot: DPI: 1 vs.CTRL  
  More Plot Information**

  ##### Volcano Plot: DPI: 1 vs.CTRL

  Volcano plot displaying each gene's -log10(p-value) and log2 fold change for the selected covariate. Highly statistically significant genes fall at the top of the plot, and highly differentially expressed genes fall to either side. Genes within the selected gene set are highlighted in orange. Horizontal lines indicate various False Discovery Rate (FDR) thresholds.
- **DE Results - Autophagy genes - DPI: 1 vs.CTRL  
  More Plot Information  Download CSV Data**

  | Probe Label | Log2 fold change | std error (log2) | Lower confidence limit (log2) | Upper confidence limit (log2) | Linear fold change | Lower confidence limit (linear) | Upper confidence limit (linear) | P-value | BY.p.value | method | Gene.sets | Codeset.Name | Probe.Label | Analyte.Type | Is.Control | Control.Type | Probe.Annotation | KEGG.Pathways | Cell.Type | Official.Gene.Name | volcanocol | volcanopch | tstats.all |
  | --- | --- | --- | --- | --- | --- | --- | --- | --- | --- | --- | --- | --- | --- | --- | --- | --- | --- | --- | --- | --- | --- | --- | --- |
  | Ralb-mRNA | 0.207 | 0.047 | 0.115 | 0.299 | 1.15 | 1.08 | 1.23 | 0.000866 | 0.314 | loglinear | Autophagy, Growth Factor Signaling | NS\_MM\_NEUROINFLAM\_V1.0 | Ralb | mRNA | 0 |  | Growth Factor Signaling;Autophagy | mmu04014;mmu04015;mmu04072;mmu05200;mmu05212 |  | Ralb | slateblue4 | 16 | 4.40425531914894 |
  | Clic4-mRNA | 0.499 | 0.117 | 0.271 | 0.728 | 1.41 | 1.21 | 1.66 | 0.00106 | 0.335 | lm.nb | Autophagy, Microglia Function | NS\_MM\_NEUROINFLAM\_V1.0 | Clic4 | mRNA | 0 |  | Microglia Function;Autophagy |  |  | Clic4 | slateblue4 | 16 | 4.26495726495726 |
  | Cd14-mRNA | 2.57 | 0.657 | 1.28 | 3.85 | 5.92 | 2.43 | 14.5 | 0.00208 | 0.437 | lm.nb | Apoptosis, Astrocyte Function, Autophagy, Growth Factor Signaling, Innate Immune Response, NF-kB | NS\_MM\_NEUROINFLAM\_V1.0 | Cd14 | mRNA | 0 |  | NF-kB;Innate Immune Response;Growth Factor Signaling;Autophagy;Astrocyte Function;Apoptosis | mmu04010;mmu04064;mmu04145;mmu04620;mmu04640;mmu04810;mmu05132;mmu05133;mmu05134;mmu05146;mmu05152;mmu05202 |  | Cd14 | slateblue4 | 16 | 3.9117199391172 |
  | Calr-mRNA | -0.485 | 0.129 | -0.739 | -0.231 | 0.715 | 0.599 | 0.852 | 0.0028 | 0.509 | lm.nb | Adaptive Immune Response, Autophagy, Neurons and Neurotransmission | NS\_MM\_NEUROINFLAM\_V1.0 | Calr | mRNA | 0 |  | Neurons and Neurotransmission;Autophagy;Adaptive Immune Response | mmu04141;mmu04145;mmu04612;mmu05142;mmu05166 |  | Calr | azure4 | 1 | -3.75968992248062 |
  | Optn-mRNA | 0.299 | 0.0828 | 0.137 | 0.461 | 1.23 | 1.1 | 1.38 | 0.00357 | 0.558 | loglinear | Autophagy, Cell Cycle, Microglia Function | NS\_MM\_NEUROINFLAM\_V1.0 | Optn | mRNA | 0 |  | Microglia Function;Cell Cycle;Autophagy |  |  | Optn | azure4 | 1 | 3.61111111111111 |
  | Atg9a-mRNA | -0.287 | 0.08 | -0.444 | -0.13 | 0.82 | 0.735 | 0.914 | 0.00374 | 0.558 | loglinear | Autophagy, Cellular Stress | NS\_MM\_NEUROINFLAM\_V1.0 | Atg9a | mRNA | 0 |  | Cellular Stress;Autophagy |  |  | Atg9a | azure4 | 1 | -3.5875 |
  | Pex14-mRNA | -0.235 | 0.0665 | -0.365 | -0.104 | 0.85 | 0.776 | 0.93 | 0.00413 | 0.569 | loglinear | Autophagy | NS\_MM\_NEUROINFLAM\_V1.0 | Pex14 | mRNA | 0 |  | Autophagy |  |  | Pex14 | azure4 | 1 | -3.53383458646617 |
  | Fcgr2b-mRNA | 0.79 | 0.259 | 0.284 | 1.3 | 1.73 | 1.22 | 2.46 | 0.00996 | 0.861 | lm.nb | Adaptive Immune Response, Autophagy | NS\_MM\_NEUROINFLAM\_V1.0 | Fcgr2b | mRNA | 0 |  | Autophagy;Adaptive Immune Response | mmu04145;mmu04380;mmu04662;mmu04666;mmu05150;mmu05152;mmu05162 |  | Fcgr2b | azure4 | 1 | 3.05019305019305 |
  | Bcl2l1-mRNA | 0.173 | 0.0572 | 0.0603 | 0.285 | 1.13 | 1.04 | 1.22 | 0.0108 | 0.861 | loglinear | Apoptosis, Autophagy, Cytokine Signaling, Growth Factor Signaling, Innate Immune Response, NF-kB | NS\_MM\_NEUROINFLAM\_V1.0 | Bcl2l1 | mRNA | 0 |  | NF-kB;Innate Immune Response;Growth Factor Signaling;Cytokine Signaling;Autophagy;Apoptosis | mmu04014;mmu04064;mmu04151;mmu04210;mmu04630;mmu05014;mmu05145;mmu05166;mmu05200;mmu05202;mmu05212;mmu05220;mmu05222 |  | Bcl2l1 | azure4 | 1 | 3.02447552447552 |
  | Bnip3-mRNA | 0.118 | 0.0396 | 0.0406 | 0.196 | 1.09 | 1.03 | 1.15 | 0.0114 | 0.868 | loglinear | Apoptosis, Autophagy, Cellular Stress, Microglia Function | NS\_MM\_NEUROINFLAM\_V1.0 | Bnip3 | mRNA | 0 |  | Microglia Function;Cellular Stress;Autophagy;Apoptosis | mmu04068;mmu05134 |  | Bnip3 | azure4 | 1 | 2.97979797979798 |
  | Vps4a-mRNA | 0.123 | 0.0417 | 0.0416 | 0.205 | 1.09 | 1.03 | 1.15 | 0.012 | 0.868 | loglinear | Autophagy, Neurons and Neurotransmission | NS\_MM\_NEUROINFLAM\_V1.0 | Vps4a | mRNA | 0 |  | Neurons and Neurotransmission;Autophagy | mmu04144 |  | Vps4a | azure4 | 1 | 2.94964028776978 |
  | Hif1a-mRNA | 0.21 | 0.0727 | 0.0677 | 0.353 | 1.16 | 1.05 | 1.28 | 0.0136 | 0.934 | loglinear | Autophagy, Cellular Stress, Microglia Function, Notch | NS\_MM\_NEUROINFLAM\_V1.0 | Hif1a | mRNA | 0 |  | Notch;Microglia Function;Cellular Stress;Autophagy | mmu04066;mmu04150;mmu04919;mmu05200;mmu05205;mmu05211;mmu05230;mmu05231 |  | Hif1a | azure4 | 1 | 2.88858321870701 |
  | Cd47-mRNA | -0.156 | 0.0561 | -0.266 | -0.046 | 0.898 | 0.832 | 0.969 | 0.0167 | 1 | loglinear | Autophagy, Matrix Remodeling | NS\_MM\_NEUROINFLAM\_V1.0 | Cd47 | mRNA | 0 |  | Matrix Remodeling;Autophagy | mmu04512 |  | Cd47 | azure4 | 1 | -2.7807486631016 |
  | Rhoa-mRNA | 0.267 | 0.102 | 0.068 | 0.467 | 1.2 | 1.05 | 1.38 | 0.022 | 1 | loglinear | Angiogenesis, Autophagy, Growth Factor Signaling, Wnt | NS\_MM\_NEUROINFLAM\_V1.0 | Rhoa | mRNA | 0 |  | Wnt;Growth Factor Signaling;Autophagy;Angiogenesis | mmu04014;mmu04015;mmu04022;mmu04024;mmu04062;mmu04071;mmu04072;mmu04144;mmu04270;mmu04310;mmu04350;mmu04360;mmu04510;mmu04520;mmu04530;mmu04611;mmu04660;mmu04670;mmu04722;mmu04810;mmu04921;mmu05100;mmu05133;mmu05152;mmu05200;mmu05203;mmu05205;mmu05206;mmu05210 |  | Rhoa | azure4 | 1 | 2.61764705882353 |
  | Rab7-mRNA | 0.0707 | 0.0271 | 0.0176 | 0.124 | 1.05 | 1.01 | 1.09 | 0.0229 | 1 | loglinear | Adaptive Immune Response, Autophagy | NS\_MM\_NEUROINFLAM\_V1.0 | Rab7 | mRNA | 0 |  | Autophagy;Adaptive Immune Response | mmu04144;mmu04145;mmu05132;mmu05146;mmu05152 |  | Rab7 | azure4 | 1 | 2.60885608856089 |
  | Pik3r2-mRNA | -0.099 | 0.0389 | -0.175 | -0.0229 | 0.934 | 0.886 | 0.984 | 0.0256 | 1 | loglinear | Adaptive Immune Response, Angiogenesis, Apoptosis, Autophagy, Cytokine Signaling, Growth Factor Signaling, Innate Immune Response, Insulin Signaling | NS\_MM\_NEUROINFLAM\_V1.0 | Pik3r2 | mRNA | 0 |  | Insulin Signaling;Innate Immune Response;Growth Factor Signaling;Cytokine Signaling;Autophagy;Apoptosis;Angiogenesis;Adaptive Immune Response | mmu04012;mmu04014;mmu04015;mmu04022;mmu04024;mmu04062;mmu04066;mmu04068;mmu04070;mmu04071;mmu04072;mmu04150;mmu04151;mmu04152;mmu04210;mmu04211;mmu04213;mmu04261;mmu04370;mmu04380;mmu04510;mmu04550;mmu04611;mmu04620;mmu04630;mmu04650;mmu04660;mmu04662;mmu04664;mmu04666;mmu04668;mmu04670;mmu04722;mmu04725;mmu04750;mmu04810;mmu04910;mmu04914;mmu04915;mmu04917;mmu04919;mmu04921;mmu04923;mmu04930;mmu04931;mmu04932;mmu04933;mmu04960;mmu04973;mmu05100;mmu05142;mmu05145;mmu05146;mmu05160;mmu05161;mmu05162;mmu05164;mmu05166;mmu05169;mmu05200;mmu05203;mmu05205;mmu05210;mmu05211;mmu05212;mmu05213;mmu05214;mmu05215;mmu05218;mmu05220;mmu05221;mmu05222;mmu05223;mmu05230;mmu05231 |  | Pik3r2 | azure4 | 1 | -2.54498714652956 |
  | Map1lc3a-mRNA | 0.212 | 0.0991 | 0.018 | 0.407 | 1.16 | 1.01 | 1.33 | 0.0534 | 1 | loglinear | Autophagy, Cellular Stress | NS\_MM\_NEUROINFLAM\_V1.0 | Map1lc3a | mRNA | 0 |  | Cellular Stress;Autophagy |  |  | Map1lc3a | azure4 | 1 | 2.13925327951564 |
  | Anxa1-mRNA | 0.75 | 0.368 | 0.0283 | 1.47 | 1.68 | 1.02 | 2.77 | 0.0643 | 1 | lm.nb | Autophagy | NS\_MM\_NEUROINFLAM\_V1.0 | Anxa1 | mRNA | 0 |  | Autophagy |  |  | Anxa1 | azure4 | 1 | 2.03804347826087 |
  | Tgm2-mRNA | 0.459 | 0.234 | 0.000621 | 0.918 | 1.38 | 1 | 1.89 | 0.0733 | 1 | lm.nb | Autophagy, Microglia Function | NS\_MM\_NEUROINFLAM\_V1.0 | Tgm2 | mRNA | 0 |  | Microglia Function;Autophagy | mmu05016 |  | Tgm2 | azure4 | 1 | 1.96153846153846 |
  | Msn-mRNA | 0.732 | 0.38 | -0.0126 | 1.48 | 1.66 | 0.991 | 2.78 | 0.078 | 1 | lm.nb | Autophagy | NS\_MM\_NEUROINFLAM\_V1.0 | Msn | mRNA | 0 |  | Autophagy | mmu04670;mmu04810;mmu05162;mmu05205 |  | Msn | azure4 | 1 | 1.92631578947368 |

  ##### DE Results - Autophagy genes - DPI: 1 vs.CTRL

  Table displaying each sample's global significance scores and directed global significance scores as defined in the heatmaps above. The global significance score is calculated as the square root of the mean squared t-statistic for the genes in a gene set, with t-statistics coming from the linear regression underlying our differential expression analysis. The directed global significance score is calculated as the square root of the mean signed squared t-statistic for the genes in a gene set, with t-statistics coming from the linear regression underlying our differential expression analysis.

- **Volcano Plot: DPI: 2 vs.CTRL  
  More Plot Information**

  ##### Volcano Plot: DPI: 2 vs.CTRL

  Volcano plot displaying each gene's -log10(p-value) and log2 fold change for the selected covariate. Highly statistically significant genes fall at the top of the plot, and highly differentially expressed genes fall to either side. Genes within the selected gene set are highlighted in orange. Horizontal lines indicate various False Discovery Rate (FDR) thresholds.
- **DE Results - Autophagy genes - DPI: 2 vs.CTRL  
  More Plot Information  Download CSV Data**

  | Probe Label | Log2 fold change | std error (log2) | Lower confidence limit (log2) | Upper confidence limit (log2) | Linear fold change | Lower confidence limit (linear) | Upper confidence limit (linear) | P-value | BY.p.value | method | Gene.sets | Codeset.Name | Probe.Label | Analyte.Type | Is.Control | Control.Type | Probe.Annotation | KEGG.Pathways | Cell.Type | Official.Gene.Name | volcanocol | volcanopch | tstats.all |
  | --- | --- | --- | --- | --- | --- | --- | --- | --- | --- | --- | --- | --- | --- | --- | --- | --- | --- | --- | --- | --- | --- | --- | --- |
  | Tgm2-mRNA | 2.15 | 0.218 | 1.73 | 2.58 | 4.45 | 3.31 | 5.99 | 4.07e-07 | 0.000109 | lm.nb | Autophagy, Microglia Function | NS\_MM\_NEUROINFLAM\_V1.0 | Tgm2 | mRNA | 0 |  | Microglia Function;Autophagy | mmu05016 |  | Tgm2 | slateblue1 | 16 | 9.86238532110092 |
  | Pik3r2-mRNA | -0.347 | 0.0389 | -0.423 | -0.27 | 0.786 | 0.746 | 0.829 | 1.22e-06 | 0.000256 | loglinear | Adaptive Immune Response, Angiogenesis, Apoptosis, Autophagy, Cytokine Signaling, Growth Factor Signaling, Innate Immune Response, Insulin Signaling | NS\_MM\_NEUROINFLAM\_V1.0 | Pik3r2 | mRNA | 0 |  | Insulin Signaling;Innate Immune Response;Growth Factor Signaling;Cytokine Signaling;Autophagy;Apoptosis;Angiogenesis;Adaptive Immune Response | mmu04012;mmu04014;mmu04015;mmu04022;mmu04024;mmu04062;mmu04066;mmu04068;mmu04070;mmu04071;mmu04072;mmu04150;mmu04151;mmu04152;mmu04210;mmu04211;mmu04213;mmu04261;mmu04370;mmu04380;mmu04510;mmu04550;mmu04611;mmu04620;mmu04630;mmu04650;mmu04660;mmu04662;mmu04664;mmu04666;mmu04668;mmu04670;mmu04722;mmu04725;mmu04750;mmu04810;mmu04910;mmu04914;mmu04915;mmu04917;mmu04919;mmu04921;mmu04923;mmu04930;mmu04931;mmu04932;mmu04933;mmu04960;mmu04973;mmu05100;mmu05142;mmu05145;mmu05146;mmu05160;mmu05161;mmu05162;mmu05164;mmu05166;mmu05169;mmu05200;mmu05203;mmu05205;mmu05210;mmu05211;mmu05212;mmu05213;mmu05214;mmu05215;mmu05218;mmu05220;mmu05221;mmu05222;mmu05223;mmu05230;mmu05231 |  | Pik3r2 | slateblue1 | 16 | -8.92030848329049 |
  | Rala-mRNA | -0.405 | 0.0487 | -0.5 | -0.309 | 0.755 | 0.707 | 0.807 | 2.56e-06 | 0.00042 | loglinear | Autophagy, Growth Factor Signaling, Neurons and Neurotransmission | NS\_MM\_NEUROINFLAM\_V1.0 | Rala | mRNA | 0 |  | Neurons and Neurotransmission;Growth Factor Signaling;Autophagy | mmu04014;mmu04015;mmu04072;mmu05200;mmu05212 |  | Rala | slateblue1 | 16 | -8.31622176591376 |
  | Bnip3-mRNA | 0.317 | 0.0396 | 0.239 | 0.395 | 1.25 | 1.18 | 1.31 | 3.73e-06 | 0.000532 | loglinear | Apoptosis, Autophagy, Cellular Stress, Microglia Function | NS\_MM\_NEUROINFLAM\_V1.0 | Bnip3 | mRNA | 0 |  | Microglia Function;Cellular Stress;Autophagy;Apoptosis | mmu04068;mmu05134 |  | Bnip3 | slateblue1 | 16 | 8.00505050505051 |
  | Cd14-mRNA | 4.52 | 0.641 | 3.26 | 5.78 | 22.9 | 9.59 | 54.8 | 1.34e-05 | 0.00162 | lm.nb | Apoptosis, Astrocyte Function, Autophagy, Growth Factor Signaling, Innate Immune Response, NF-kB | NS\_MM\_NEUROINFLAM\_V1.0 | Cd14 | mRNA | 0 |  | NF-kB;Innate Immune Response;Growth Factor Signaling;Autophagy;Astrocyte Function;Apoptosis | mmu04010;mmu04064;mmu04145;mmu04620;mmu04640;mmu04810;mmu05132;mmu05133;mmu05134;mmu05146;mmu05152;mmu05202 |  | Cd14 | slateblue1 | 16 | 7.05148205928237 |
  | Fcgr2b-mRNA | 1.48 | 0.253 | 0.983 | 1.98 | 2.79 | 1.98 | 3.93 | 7.95e-05 | 0.00599 | lm.nb | Adaptive Immune Response, Autophagy | NS\_MM\_NEUROINFLAM\_V1.0 | Fcgr2b | mRNA | 0 |  | Autophagy;Adaptive Immune Response | mmu04145;mmu04380;mmu04662;mmu04666;mmu05150;mmu05152;mmu05162 |  | Fcgr2b | slateblue1 | 16 | 5.8498023715415 |
  | Bcl2l1-mRNA | 0.33 | 0.0572 | 0.217 | 0.442 | 1.26 | 1.16 | 1.36 | 9.06e-05 | 0.00658 | loglinear | Apoptosis, Autophagy, Cytokine Signaling, Growth Factor Signaling, Innate Immune Response, NF-kB | NS\_MM\_NEUROINFLAM\_V1.0 | Bcl2l1 | mRNA | 0 |  | NF-kB;Innate Immune Response;Growth Factor Signaling;Cytokine Signaling;Autophagy;Apoptosis | mmu04014;mmu04064;mmu04151;mmu04210;mmu04630;mmu05014;mmu05145;mmu05166;mmu05200;mmu05202;mmu05212;mmu05220;mmu05222 |  | Bcl2l1 | slateblue1 | 16 | 5.76923076923077 |
  | Mertk-mRNA | 0.998 | 0.215 | 0.576 | 1.42 | 2 | 1.49 | 2.67 | 0.000576 | 0.0281 | lm.nb | Autophagy, Microglia Function | NS\_MM\_NEUROINFLAM\_V1.0 | Mertk | mRNA | 0 |  | Microglia Function;Autophagy |  |  | Mertk | slateblue2 | 16 | 4.64186046511628 |
  | Pros1-mRNA | 0.65 | 0.144 | 0.369 | 0.932 | 1.57 | 1.29 | 1.91 | 0.000695 | 0.0323 | loglinear | Autophagy | NS\_MM\_NEUROINFLAM\_V1.0 | Pros1 | mRNA | 0 |  | Autophagy | mmu04610 |  | Pros1 | slateblue2 | 16 | 4.51388888888889 |
  | Rab7-mRNA | -0.119 | 0.0271 | -0.172 | -0.0655 | 0.921 | 0.888 | 0.956 | 0.000901 | 0.039 | loglinear | Adaptive Immune Response, Autophagy | NS\_MM\_NEUROINFLAM\_V1.0 | Rab7 | mRNA | 0 |  | Autophagy;Adaptive Immune Response | mmu04144;mmu04145;mmu05132;mmu05146;mmu05152 |  | Rab7 | slateblue2 | 16 | -4.39114391143911 |
  | Fcgr3-mRNA | 1.25 | 0.287 | 0.685 | 1.81 | 2.37 | 1.61 | 3.51 | 0.000959 | 0.0399 | lm.nb | Adaptive Immune Response, Autophagy, Inflammatory Signaling, Innate Immune Response | NS\_MM\_NEUROINFLAM\_V1.0 | Fcgr3 | mRNA | 0 |  | Innate Immune Response;Inflammatory Signaling;Autophagy;Adaptive Immune Response | mmu04145;mmu04380;mmu05140;mmu05150;mmu05152 |  | Fcgr3 | slateblue2 | 16 | 4.35540069686411 |
  | Prkcq-mRNA | -0.84 | 0.198 | -1.23 | -0.451 | 0.559 | 0.427 | 0.732 | 0.00116 | 0.0479 | lm.nb | Adaptive Immune Response, Apoptosis, Autophagy, NF-kB, Oligodendrocyte Function | NS\_MM\_NEUROINFLAM\_V1.0 | Prkcq | mRNA | 0 |  | Oligodendrocyte Function;NF-kB;Autophagy;Apoptosis;Adaptive Immune Response | mmu04064;mmu04270;mmu04530;mmu04660;mmu04750;mmu04920;mmu04931;mmu05162 | Oligodendrocytes | Prkcq | slateblue2 | 16 | -4.24242424242424 |
  | Sqstm1-mRNA | 0.107 | 0.0261 | 0.0554 | 0.158 | 1.08 | 1.04 | 1.12 | 0.00152 | 0.0584 | loglinear | Autophagy, Cytokine Signaling, Growth Factor Signaling | NS\_MM\_NEUROINFLAM\_V1.0 | Sqstm1 | mRNA | 0 |  | Growth Factor Signaling;Cytokine Signaling;Autophagy | mmu04380 |  | Sqstm1 | slateblue3 | 16 | 4.09961685823755 |
  | Calr-mRNA | -0.51 | 0.13 | -0.764 | -0.257 | 0.702 | 0.589 | 0.837 | 0.00196 | 0.0717 | lm.nb | Adaptive Immune Response, Autophagy, Neurons and Neurotransmission | NS\_MM\_NEUROINFLAM\_V1.0 | Calr | mRNA | 0 |  | Neurons and Neurotransmission;Autophagy;Adaptive Immune Response | mmu04141;mmu04145;mmu04612;mmu05142;mmu05166 |  | Calr | slateblue3 | 16 | -3.92307692307692 |
  | Dock1-mRNA | -0.39 | 0.101 | -0.588 | -0.191 | 0.763 | 0.665 | 0.876 | 0.00231 | 0.0803 | loglinear | Angiogenesis, Autophagy | NS\_MM\_NEUROINFLAM\_V1.0 | Dock1 | mRNA | 0 |  | Autophagy;Angiogenesis | mmu04510;mmu04810;mmu05100 |  | Dock1 | slateblue3 | 16 | -3.86138613861386 |
  | Ralb-mRNA | 0.179 | 0.047 | 0.0864 | 0.271 | 1.13 | 1.06 | 1.21 | 0.00254 | 0.0854 | loglinear | Autophagy, Growth Factor Signaling | NS\_MM\_NEUROINFLAM\_V1.0 | Ralb | mRNA | 0 |  | Growth Factor Signaling;Autophagy | mmu04014;mmu04015;mmu04072;mmu05200;mmu05212 |  | Ralb | slateblue3 | 16 | 3.80851063829787 |
  | Prkce-mRNA | -0.171 | 0.0471 | -0.263 | -0.0788 | 0.888 | 0.833 | 0.947 | 0.00343 | 0.108 | loglinear | Autophagy, Growth Factor Signaling | NS\_MM\_NEUROINFLAM\_V1.0 | Prkce | mRNA | 0 |  | Growth Factor Signaling;Autophagy | mmu04022;mmu04071;mmu04270;mmu04530;mmu04666;mmu04750;mmu04925;mmu04930;mmu04931;mmu04933;mmu05206 |  | Prkce | slateblue4 | 16 | -3.63057324840764 |
  | Lamp2-mRNA | 0.233 | 0.0688 | 0.0983 | 0.368 | 1.18 | 1.07 | 1.29 | 0.00537 | 0.155 | loglinear | Autophagy | NS\_MM\_NEUROINFLAM\_V1.0 | Lamp2 | mRNA | 0 |  | Autophagy | mmu04142;mmu04145;mmu05152 |  | Lamp2 | slateblue4 | 16 | 3.38662790697674 |
  | Dapk1-mRNA | 0.302 | 0.0918 | 0.122 | 0.482 | 1.23 | 1.09 | 1.4 | 0.00647 | 0.179 | loglinear | Apoptosis, Autophagy | NS\_MM\_NEUROINFLAM\_V1.0 | Dapk1 | mRNA | 0 |  | Autophagy;Apoptosis | mmu05200;mmu05219 |  | Dapk1 | slateblue4 | 16 | 3.28976034858388 |
  | Akt2-mRNA | -0.687 | 0.21 | -1.1 | -0.276 | 0.621 | 0.467 | 0.826 | 0.00664 | 0.18 | lm.nb | Adaptive Immune Response, Angiogenesis, Apoptosis, Autophagy, Carbohydrate Metabolism, Cytokine Signaling, Growth Factor Signaling, Innate Immune Response, Insulin Signaling, Neurons and Neurotransmission, Wnt | NS\_MM\_NEUROINFLAM\_V1.0 | Akt2 | mRNA | 0 |  | Wnt;Neurons and Neurotransmission;Insulin Signaling;Innate Immune Response;Growth Factor Signaling;Cytokine Signaling;Carbohydrate Metabolism;Autophagy;Apoptosis;Angiogenesis;Adaptive Immune Response | mmu04010;mmu04012;mmu04014;mmu04015;mmu04022;mmu04024;mmu04062;mmu04066;mmu04068;mmu04071;mmu04072;mmu04150;mmu04151;mmu04152;mmu04210;mmu04211;mmu04213;mmu04261;mmu04370;mmu04380;mmu04510;mmu04530;mmu04550;mmu04611;mmu04620;mmu04630;mmu04660;mmu04662;mmu04664;mmu04666;mmu04668;mmu04722;mmu04725;mmu04728;mmu04910;mmu04914;mmu04915;mmu04917;mmu04919;mmu04920;mmu04922;mmu04923;mmu04931;mmu04932;mmu04933;mmu04973;mmu05142;mmu05145;mmu05152;mmu05160;mmu05161;mmu05162;mmu05164;mmu05166;mmu05169;mmu05200;mmu05205;mmu05210;mmu05211;mmu05212;mmu05213;mmu05214;mmu05215;mmu05218;mmu05220;mmu05221;mmu05222;mmu05223;mmu05230;mmu05231 |  | Akt2 | slateblue4 | 16 | -3.27142857142857 |

  ##### DE Results - Autophagy genes - DPI: 2 vs.CTRL

  Table displaying each sample's global significance scores and directed global significance scores as defined in the heatmaps above. The global significance score is calculated as the square root of the mean squared t-statistic for the genes in a gene set, with t-statistics coming from the linear regression underlying our differential expression analysis. The directed global significance score is calculated as the square root of the mean signed squared t-statistic for the genes in a gene set, with t-statistics coming from the linear regression underlying our differential expression analysis.

- **Volcano Plot: DPI: 3 vs.CTRL  
  More Plot Information**

  ##### Volcano Plot: DPI: 3 vs.CTRL

  Volcano plot displaying each gene's -log10(p-value) and log2 fold change for the selected covariate. Highly statistically significant genes fall at the top of the plot, and highly differentially expressed genes fall to either side. Genes within the selected gene set are highlighted in orange. Horizontal lines indicate various False Discovery Rate (FDR) thresholds.
- **DE Results - Autophagy genes - DPI: 3 vs.CTRL  
  More Plot Information  Download CSV Data**

  | Probe Label | Log2 fold change | std error (log2) | Lower confidence limit (log2) | Upper confidence limit (log2) | Linear fold change | Lower confidence limit (linear) | Upper confidence limit (linear) | P-value | BY.p.value | method | Gene.sets | Codeset.Name | Probe.Label | Analyte.Type | Is.Control | Control.Type | Probe.Annotation | KEGG.Pathways | Cell.Type | Official.Gene.Name | volcanocol | volcanopch | tstats.all |
  | --- | --- | --- | --- | --- | --- | --- | --- | --- | --- | --- | --- | --- | --- | --- | --- | --- | --- | --- | --- | --- | --- | --- | --- |
  | Tgm2-mRNA | 3.3 | 0.214 | 2.88 | 3.72 | 9.83 | 7.35 | 13.1 | 2.85e-09 | 1.14e-06 | lm.nb | Autophagy, Microglia Function | NS\_MM\_NEUROINFLAM\_V1.0 | Tgm2 | mRNA | 0 |  | Microglia Function;Autophagy | mmu05016 |  | Tgm2 | slateblue1 | 16 | 15.4205607476636 |
  | Cd14-mRNA | 6.51 | 0.637 | 5.26 | 7.76 | 90.9 | 38.3 | 216 | 2.85e-07 | 3.6e-05 | lm.nb | Apoptosis, Astrocyte Function, Autophagy, Growth Factor Signaling, Innate Immune Response, NF-kB | NS\_MM\_NEUROINFLAM\_V1.0 | Cd14 | mRNA | 0 |  | NF-kB;Innate Immune Response;Growth Factor Signaling;Autophagy;Astrocyte Function;Apoptosis | mmu04010;mmu04064;mmu04145;mmu04620;mmu04640;mmu04810;mmu05132;mmu05133;mmu05134;mmu05146;mmu05152;mmu05202 |  | Cd14 | slateblue1 | 16 | 10.2197802197802 |
  | Fcgr2b-mRNA | 2.54 | 0.249 | 2.05 | 3.03 | 5.81 | 4.14 | 8.14 | 2.88e-07 | 3.6e-05 | lm.nb | Adaptive Immune Response, Autophagy | NS\_MM\_NEUROINFLAM\_V1.0 | Fcgr2b | mRNA | 0 |  | Autophagy;Adaptive Immune Response | mmu04145;mmu04380;mmu04662;mmu04666;mmu05150;mmu05152;mmu05162 |  | Fcgr2b | slateblue1 | 16 | 10.2008032128514 |
  | Pik3r2-mRNA | -0.374 | 0.0389 | -0.451 | -0.298 | 0.771 | 0.732 | 0.813 | 5.36e-07 | 5.35e-05 | loglinear | Adaptive Immune Response, Angiogenesis, Apoptosis, Autophagy, Cytokine Signaling, Growth Factor Signaling, Innate Immune Response, Insulin Signaling | NS\_MM\_NEUROINFLAM\_V1.0 | Pik3r2 | mRNA | 0 |  | Insulin Signaling;Innate Immune Response;Growth Factor Signaling;Cytokine Signaling;Autophagy;Apoptosis;Angiogenesis;Adaptive Immune Response | mmu04012;mmu04014;mmu04015;mmu04022;mmu04024;mmu04062;mmu04066;mmu04068;mmu04070;mmu04071;mmu04072;mmu04150;mmu04151;mmu04152;mmu04210;mmu04211;mmu04213;mmu04261;mmu04370;mmu04380;mmu04510;mmu04550;mmu04611;mmu04620;mmu04630;mmu04650;mmu04660;mmu04662;mmu04664;mmu04666;mmu04668;mmu04670;mmu04722;mmu04725;mmu04750;mmu04810;mmu04910;mmu04914;mmu04915;mmu04917;mmu04919;mmu04921;mmu04923;mmu04930;mmu04931;mmu04932;mmu04933;mmu04960;mmu04973;mmu05100;mmu05142;mmu05145;mmu05146;mmu05160;mmu05161;mmu05162;mmu05164;mmu05166;mmu05169;mmu05200;mmu05203;mmu05205;mmu05210;mmu05211;mmu05212;mmu05213;mmu05214;mmu05215;mmu05218;mmu05220;mmu05221;mmu05222;mmu05223;mmu05230;mmu05231 |  | Pik3r2 | slateblue1 | 16 | -9.61439588688946 |
  | Csf1-mRNA | 1.21 | 0.132 | 0.948 | 1.47 | 2.31 | 1.93 | 2.76 | 9.38e-07 | 8.32e-05 | lm.nb | Astrocyte Function, Autophagy, Cytokine Signaling, Growth Factor Signaling, Innate Immune Response, Microglia Function | NS\_MM\_NEUROINFLAM\_V1.0 | Csf1 | mRNA | 0 |  | Microglia Function;Innate Immune Response;Growth Factor Signaling;Cytokine Signaling;Autophagy;Astrocyte Function | mmu04014;mmu04015;mmu04060;mmu04151;mmu04380;mmu04640;mmu04668;mmu05323 |  | Csf1 | slateblue1 | 16 | 9.16666666666667 |
  | Sqstm1-mRNA | 0.231 | 0.0261 | 0.18 | 0.283 | 1.17 | 1.13 | 1.22 | 1.29e-06 | 0.000105 | loglinear | Autophagy, Cytokine Signaling, Growth Factor Signaling | NS\_MM\_NEUROINFLAM\_V1.0 | Sqstm1 | mRNA | 0 |  | Growth Factor Signaling;Cytokine Signaling;Autophagy | mmu04380 |  | Sqstm1 | slateblue1 | 16 | 8.85057471264368 |
  | Clic4-mRNA | 1.02 | 0.116 | 0.791 | 1.24 | 2.02 | 1.73 | 2.37 | 1.41e-06 | 0.000112 | lm.nb | Autophagy, Microglia Function | NS\_MM\_NEUROINFLAM\_V1.0 | Clic4 | mRNA | 0 |  | Microglia Function;Autophagy |  |  | Clic4 | slateblue1 | 16 | 8.79310344827586 |
  | Pros1-mRNA | 1.25 | 0.144 | 0.965 | 1.53 | 2.37 | 1.95 | 2.88 | 1.62e-06 | 0.000122 | loglinear | Autophagy | NS\_MM\_NEUROINFLAM\_V1.0 | Pros1 | mRNA | 0 |  | Autophagy | mmu04610 |  | Pros1 | slateblue1 | 16 | 8.68055555555556 |
  | C3-mRNA | 5.35 | 0.633 | 4.11 | 6.59 | 40.8 | 17.2 | 96.3 | 2.14e-06 | 0.000149 | lm.nb | Adaptive Immune Response, Autophagy, Inflammatory Signaling, Innate Immune Response, Microglia Function | NS\_MM\_NEUROINFLAM\_V1.0 | C3 | mRNA | 0 |  | Microglia Function;Innate Immune Response;Inflammatory Signaling;Autophagy;Adaptive Immune Response | mmu04145;mmu04610;mmu05133;mmu05134;mmu05140;mmu05142;mmu05150;mmu05152;mmu05168;mmu05203;mmu05322 |  | C3 | slateblue1 | 16 | 8.45181674565561 |
  | Prkce-mRNA | -0.39 | 0.0471 | -0.482 | -0.298 | 0.763 | 0.716 | 0.813 | 2.61e-06 | 0.000174 | loglinear | Autophagy, Growth Factor Signaling | NS\_MM\_NEUROINFLAM\_V1.0 | Prkce | mRNA | 0 |  | Growth Factor Signaling;Autophagy | mmu04022;mmu04071;mmu04270;mmu04530;mmu04666;mmu04750;mmu04925;mmu04930;mmu04931;mmu04933;mmu05206 |  | Prkce | slateblue1 | 16 | -8.28025477707006 |
  | Ralb-mRNA | 0.37 | 0.047 | 0.278 | 0.463 | 1.29 | 1.21 | 1.38 | 4.39e-06 | 0.000271 | loglinear | Autophagy, Growth Factor Signaling | NS\_MM\_NEUROINFLAM\_V1.0 | Ralb | mRNA | 0 |  | Growth Factor Signaling;Autophagy | mmu04014;mmu04015;mmu04072;mmu05200;mmu05212 |  | Ralb | slateblue1 | 16 | 7.87234042553191 |
  | Fcgr3-mRNA | 1.9 | 0.282 | 1.35 | 2.46 | 3.74 | 2.55 | 5.49 | 2.05e-05 | 0.000986 | lm.nb | Adaptive Immune Response, Autophagy, Inflammatory Signaling, Innate Immune Response | NS\_MM\_NEUROINFLAM\_V1.0 | Fcgr3 | mRNA | 0 |  | Innate Immune Response;Inflammatory Signaling;Autophagy;Adaptive Immune Response | mmu04145;mmu04380;mmu05140;mmu05150;mmu05152 |  | Fcgr3 | slateblue1 | 16 | 6.73758865248227 |
  | Dapk1-mRNA | 0.614 | 0.0918 | 0.434 | 0.794 | 1.53 | 1.35 | 1.73 | 2.24e-05 | 0.00106 | loglinear | Apoptosis, Autophagy | NS\_MM\_NEUROINFLAM\_V1.0 | Dapk1 | mRNA | 0 |  | Autophagy;Apoptosis | mmu05200;mmu05219 |  | Dapk1 | slateblue1 | 16 | 6.68845315904139 |
  | Fcer1g-mRNA | 2.64 | 0.408 | 1.84 | 3.44 | 6.25 | 3.59 | 10.9 | 3.03e-05 | 0.00141 | lm.nb | Adaptive Immune Response, Autophagy, Inflammatory Signaling, Innate Immune Response | NS\_MM\_NEUROINFLAM\_V1.0 | Fcer1g | mRNA | 0 |  | Innate Immune Response;Inflammatory Signaling;Autophagy;Adaptive Immune Response | mmu04071;mmu04072;mmu04611;mmu04650;mmu04664;mmu05152;mmu05310 |  | Fcer1g | slateblue1 | 16 | 6.47058823529412 |
  | Cflar-mRNA | 1.32 | 0.208 | 0.915 | 1.73 | 2.5 | 1.89 | 3.32 | 3.58e-05 | 0.00164 | lm.nb | Apoptosis, Autophagy, Innate Immune Response, NF-kB | NS\_MM\_NEUROINFLAM\_V1.0 | Cflar | mRNA | 0 |  | NF-kB;Innate Immune Response;Autophagy;Apoptosis | mmu04064;mmu04210;mmu04668;mmu05142 |  | Cflar | slateblue1 | 16 | 6.34615384615385 |
  | Tcirg1-mRNA | 1.19 | 0.189 | 0.816 | 1.56 | 2.27 | 1.76 | 2.94 | 4.07e-05 | 0.00181 | lm.nb | Autophagy, Growth Factor Signaling, Insulin Signaling | NS\_MM\_NEUROINFLAM\_V1.0 | Tcirg1 | mRNA | 0 |  | Insulin Signaling;Growth Factor Signaling;Autophagy | mmu04142;mmu04145;mmu04721;mmu05152;mmu05323 |  | Tcirg1 | slateblue1 | 16 | 6.2962962962963 |
  | Msn-mRNA | 2.31 | 0.369 | 1.59 | 3.04 | 4.97 | 3.01 | 8.21 | 4.14e-05 | 0.00182 | lm.nb | Autophagy | NS\_MM\_NEUROINFLAM\_V1.0 | Msn | mRNA | 0 |  | Autophagy | mmu04670;mmu04810;mmu05162;mmu05205 |  | Msn | slateblue1 | 16 | 6.26016260162602 |
  | Rala-mRNA | -0.304 | 0.0487 | -0.4 | -0.209 | 0.81 | 0.758 | 0.865 | 4.3e-05 | 0.00185 | loglinear | Autophagy, Growth Factor Signaling, Neurons and Neurotransmission | NS\_MM\_NEUROINFLAM\_V1.0 | Rala | mRNA | 0 |  | Neurons and Neurotransmission;Growth Factor Signaling;Autophagy | mmu04014;mmu04015;mmu04072;mmu05200;mmu05212 |  | Rala | slateblue1 | 16 | -6.24229979466119 |
  | Hmgb1-mRNA | -0.34 | 0.056 | -0.449 | -0.23 | 0.79 | 0.732 | 0.853 | 5.6e-05 | 0.00238 | loglinear | Apoptosis, Autophagy, Inflammatory Signaling, Innate Immune Response | NS\_MM\_NEUROINFLAM\_V1.0 | Hmgb1 | mRNA | 0 |  | Innate Immune Response;Inflammatory Signaling;Autophagy;Apoptosis | mmu03410 |  | Hmgb1 | slateblue1 | 16 | -6.07142857142857 |
  | Bnip3-mRNA | 0.236 | 0.0396 | 0.159 | 0.314 | 1.18 | 1.12 | 1.24 | 6.56e-05 | 0.00271 | loglinear | Apoptosis, Autophagy, Cellular Stress, Microglia Function | NS\_MM\_NEUROINFLAM\_V1.0 | Bnip3 | mRNA | 0 |  | Microglia Function;Cellular Stress;Autophagy;Apoptosis | mmu04068;mmu05134 |  | Bnip3 | slateblue1 | 16 | 5.95959595959596 |

  ##### DE Results - Autophagy genes - DPI: 3 vs.CTRL

  Table displaying each sample's global significance scores and directed global significance scores as defined in the heatmaps above. The global significance score is calculated as the square root of the mean squared t-statistic for the genes in a gene set, with t-statistics coming from the linear regression underlying our differential expression analysis. The directed global significance score is calculated as the square root of the mean signed squared t-statistic for the genes in a gene set, with t-statistics coming from the linear regression underlying our differential expression analysis.

DPI:
- DPI: differential expression in 1 vs. baseline of CTRL
- DPI: differential expression in 2 vs. baseline of CTRL
- DPI: differential expression in 3 vs. baseline of CTRL

- **Volcano Plot: DPI: 1 vs.CTRL  
  More Plot Information**

  ##### Volcano Plot: DPI: 1 vs.CTRL

  Volcano plot displaying each gene's -log10(p-value) and log2 fold change for the selected covariate. Highly statistically significant genes fall at the top of the plot, and highly differentially expressed genes fall to either side. Genes within the selected gene set are highlighted in orange. Horizontal lines indicate various False Discovery Rate (FDR) thresholds.
- **DE Results - Carbohydrate Metabolism genes - DPI: 1 vs.CTRL  
  More Plot Information  Download CSV Data**

  | Probe Label | Log2 fold change | std error (log2) | Lower confidence limit (log2) | Upper confidence limit (log2) | Linear fold change | Lower confidence limit (linear) | Upper confidence limit (linear) | P-value | BY.p.value | method | Gene.sets | Codeset.Name | Probe.Label | Analyte.Type | Is.Control | Control.Type | Probe.Annotation | KEGG.Pathways | Cell.Type | Official.Gene.Name | volcanocol | volcanopch | tstats.all |
  | --- | --- | --- | --- | --- | --- | --- | --- | --- | --- | --- | --- | --- | --- | --- | --- | --- | --- | --- | --- | --- | --- | --- | --- |
  | Slco2b1-mRNA | -0.89 | 0.361 | -1.6 | -0.183 | 0.54 | 0.331 | 0.881 | 0.0296 | 1 | lm.nb | Carbohydrate Metabolism | NS\_MM\_NEUROINFLAM\_V1.0 | Slco2b1 | mRNA | 0 |  | Carbohydrate Metabolism |  |  | Slco2b1 | azure4 | 1 | -2.46537396121884 |
  | Pik3r1-mRNA | 0.15 | 0.0891 | -0.0245 | 0.325 | 1.11 | 0.983 | 1.25 | 0.118 | 1 | loglinear | Adaptive Immune Response, Angiogenesis, Apoptosis, Autophagy, Carbohydrate Metabolism, Cytokine Signaling, Growth Factor Signaling, Innate Immune Response, Insulin Signaling, Lipid Metabolism | NS\_MM\_NEUROINFLAM\_V1.0 | Pik3r1 | mRNA | 0 |  | Lipid Metabolism;Insulin Signaling;Innate Immune Response;Growth Factor Signaling;Cytokine Signaling;Carbohydrate Metabolism;Autophagy;Apoptosis;Angiogenesis;Adaptive Immune Response | mmu04012;mmu04014;mmu04015;mmu04022;mmu04024;mmu04062;mmu04066;mmu04068;mmu04070;mmu04071;mmu04072;mmu04150;mmu04151;mmu04152;mmu04210;mmu04211;mmu04213;mmu04261;mmu04370;mmu04380;mmu04510;mmu04550;mmu04611;mmu04620;mmu04630;mmu04650;mmu04660;mmu04662;mmu04664;mmu04666;mmu04668;mmu04670;mmu04722;mmu04725;mmu04750;mmu04810;mmu04910;mmu04914;mmu04915;mmu04917;mmu04919;mmu04921;mmu04923;mmu04930;mmu04931;mmu04932;mmu04933;mmu04960;mmu04973;mmu05100;mmu05142;mmu05145;mmu05146;mmu05160;mmu05161;mmu05162;mmu05164;mmu05166;mmu05169;mmu05200;mmu05203;mmu05205;mmu05210;mmu05211;mmu05212;mmu05213;mmu05214;mmu05215;mmu05218;mmu05220;mmu05221;mmu05222;mmu05223;mmu05230;mmu05231 |  | Pik3r1 | azure4 | 1 | 1.68350168350168 |
  | Pik3cb-mRNA | -0.0707 | 0.0676 | -0.203 | 0.0618 | 0.952 | 0.869 | 1.04 | 0.316 | 1 | loglinear | Adaptive Immune Response, Angiogenesis, Apoptosis, Autophagy, Carbohydrate Metabolism, Cytokine Signaling, Growth Factor Signaling, Innate Immune Response, Insulin Signaling, Lipid Metabolism | NS\_MM\_NEUROINFLAM\_V1.0 | Pik3cb | mRNA | 0 |  | Lipid Metabolism;Insulin Signaling;Innate Immune Response;Growth Factor Signaling;Cytokine Signaling;Carbohydrate Metabolism;Autophagy;Apoptosis;Angiogenesis;Adaptive Immune Response | mmu00562;mmu04012;mmu04014;mmu04015;mmu04022;mmu04024;mmu04062;mmu04066;mmu04068;mmu04070;mmu04071;mmu04072;mmu04150;mmu04151;mmu04152;mmu04210;mmu04211;mmu04213;mmu04261;mmu04370;mmu04380;mmu04510;mmu04550;mmu04611;mmu04620;mmu04630;mmu04650;mmu04660;mmu04662;mmu04664;mmu04666;mmu04668;mmu04670;mmu04722;mmu04725;mmu04750;mmu04810;mmu04910;mmu04914;mmu04915;mmu04917;mmu04919;mmu04921;mmu04923;mmu04930;mmu04931;mmu04932;mmu04933;mmu04960;mmu04973;mmu05100;mmu05142;mmu05145;mmu05146;mmu05160;mmu05161;mmu05162;mmu05164;mmu05166;mmu05169;mmu05200;mmu05203;mmu05205;mmu05210;mmu05211;mmu05212;mmu05213;mmu05214;mmu05215;mmu05218;mmu05220;mmu05221;mmu05222;mmu05223;mmu05230;mmu05231 |  | Pik3cb | azure4 | 1 | -1.04585798816568 |
  | Sirt1-mRNA | 0.123 | 0.136 | -0.144 | 0.391 | 1.09 | 0.905 | 1.31 | 0.384 | 1 | lm.nb | Carbohydrate Metabolism, Cellular Stress, Epigenetic Regulation | NS\_MM\_NEUROINFLAM\_V1.0 | Sirt1 | mRNA | 0 |  | Epigenetic Regulation;Cellular Stress;Carbohydrate Metabolism | mmu04068;mmu04152;mmu04211;mmu04213;mmu04922;mmu05031;mmu05206 |  | Sirt1 | azure4 | 1 | 0.904411764705882 |
  | Igf1r-mRNA | -0.0784 | 0.111 | -0.296 | 0.139 | 0.947 | 0.815 | 1.1 | 0.493 | 1 | loglinear | Apoptosis, Autophagy, Carbohydrate Metabolism, Growth Factor Signaling, Insulin Signaling | NS\_MM\_NEUROINFLAM\_V1.0 | Igf1r | mRNA | 0 |  | Insulin Signaling;Growth Factor Signaling;Carbohydrate Metabolism;Autophagy;Apoptosis | mmu04014;mmu04015;mmu04066;mmu04068;mmu04114;mmu04144;mmu04151;mmu04152;mmu04211;mmu04213;mmu04510;mmu04520;mmu04550;mmu04730;mmu04913;mmu04914;mmu05200;mmu05202;mmu05205;mmu05214;mmu05215;mmu05218 |  | Igf1r | azure4 | 1 | -0.706306306306306 |
  | Creb1-mRNA | 0.068 | 0.124 | -0.175 | 0.311 | 1.05 | 0.886 | 1.24 | 0.594 | 1 | loglinear | Adaptive Immune Response, Carbohydrate Metabolism, DNA Damage, Growth Factor Signaling, Innate Immune Response, Neurons and Neurotransmission, Notch | NS\_MM\_NEUROINFLAM\_V1.0 | Creb1 | mRNA | 0 |  | Notch;Neurons and Neurotransmission;Innate Immune Response;Growth Factor Signaling;DNA Damage;Carbohydrate Metabolism;Adaptive Immune Response | mmu04022;mmu04024;mmu04151;mmu04152;mmu04211;mmu04261;mmu04380;mmu04612;mmu04668;mmu04713;mmu04725;mmu04728;mmu04911;mmu04915;mmu04916;mmu04922;mmu04924;mmu04925;mmu04931;mmu05016;mmu05030;mmu05031;mmu05034;mmu05152;mmu05161;mmu05166;mmu05203;mmu05215 |  | Creb1 | azure4 | 1 | 0.548387096774194 |
  | Akt1-mRNA | 0.0436 | 0.118 | -0.187 | 0.274 | 1.03 | 0.878 | 1.21 | 0.718 | 1 | lm.nb | Adaptive Immune Response, Angiogenesis, Apoptosis, Autophagy, Carbohydrate Metabolism, Cellular Stress, Cytokine Signaling, Growth Factor Signaling, Innate Immune Response, Insulin Signaling, Neurons and Neurotransmission, Wnt | NS\_MM\_NEUROINFLAM\_V1.0 | Akt1 | mRNA | 0 |  | Wnt;Neurons and Neurotransmission;Insulin Signaling;Innate Immune Response;Growth Factor Signaling;Cytokine Signaling;Cellular Stress;Carbohydrate Metabolism;Autophagy;Apoptosis;Angiogenesis;Adaptive Immune Response | mmu04010;mmu04012;mmu04014;mmu04015;mmu04022;mmu04024;mmu04062;mmu04066;mmu04068;mmu04071;mmu04072;mmu04150;mmu04151;mmu04152;mmu04210;mmu04211;mmu04213;mmu04261;mmu04370;mmu04380;mmu04510;mmu04530;mmu04550;mmu04611;mmu04620;mmu04630;mmu04660;mmu04662;mmu04664;mmu04666;mmu04668;mmu04722;mmu04725;mmu04728;mmu04910;mmu04914;mmu04915;mmu04917;mmu04919;mmu04920;mmu04922;mmu04923;mmu04931;mmu04932;mmu04933;mmu04973;mmu05142;mmu05145;mmu05152;mmu05160;mmu05161;mmu05162;mmu05164;mmu05166;mmu05169;mmu05200;mmu05205;mmu05210;mmu05211;mmu05212;mmu05213;mmu05214;mmu05215;mmu05218;mmu05220;mmu05221;mmu05222;mmu05223;mmu05230;mmu05231 |  | Akt1 | azure4 | 1 | 0.369491525423729 |
  | Pik3ca-mRNA | 0.0296 | 0.0873 | -0.142 | 0.201 | 1.02 | 0.906 | 1.15 | 0.741 | 1 | loglinear | Adaptive Immune Response, Angiogenesis, Apoptosis, Autophagy, Carbohydrate Metabolism, Cytokine Signaling, Growth Factor Signaling, Innate Immune Response, Insulin Signaling, Lipid Metabolism | NS\_MM\_NEUROINFLAM\_V1.0 | Pik3ca | mRNA | 0 |  | Lipid Metabolism;Insulin Signaling;Innate Immune Response;Growth Factor Signaling;Cytokine Signaling;Carbohydrate Metabolism;Autophagy;Apoptosis;Angiogenesis;Adaptive Immune Response | mmu00562;mmu04012;mmu04014;mmu04015;mmu04022;mmu04024;mmu04062;mmu04066;mmu04068;mmu04070;mmu04071;mmu04072;mmu04150;mmu04151;mmu04152;mmu04210;mmu04211;mmu04213;mmu04261;mmu04370;mmu04380;mmu04510;mmu04550;mmu04611;mmu04620;mmu04630;mmu04650;mmu04660;mmu04662;mmu04664;mmu04666;mmu04668;mmu04670;mmu04722;mmu04725;mmu04750;mmu04810;mmu04910;mmu04914;mmu04915;mmu04917;mmu04919;mmu04921;mmu04923;mmu04930;mmu04931;mmu04932;mmu04933;mmu04960;mmu04973;mmu05100;mmu05142;mmu05145;mmu05146;mmu05160;mmu05161;mmu05162;mmu05164;mmu05166;mmu05169;mmu05200;mmu05203;mmu05205;mmu05210;mmu05211;mmu05212;mmu05213;mmu05214;mmu05215;mmu05218;mmu05220;mmu05221;mmu05222;mmu05223;mmu05230;mmu05231 |  | Pik3ca | azure4 | 1 | 0.339060710194731 |
  | Akt2-mRNA | -0.025 | 0.207 | -0.431 | 0.381 | 0.983 | 0.742 | 1.3 | 0.906 | 1 | lm.nb | Adaptive Immune Response, Angiogenesis, Apoptosis, Autophagy, Carbohydrate Metabolism, Cytokine Signaling, Growth Factor Signaling, Innate Immune Response, Insulin Signaling, Neurons and Neurotransmission, Wnt | NS\_MM\_NEUROINFLAM\_V1.0 | Akt2 | mRNA | 0 |  | Wnt;Neurons and Neurotransmission;Insulin Signaling;Innate Immune Response;Growth Factor Signaling;Cytokine Signaling;Carbohydrate Metabolism;Autophagy;Apoptosis;Angiogenesis;Adaptive Immune Response | mmu04010;mmu04012;mmu04014;mmu04015;mmu04022;mmu04024;mmu04062;mmu04066;mmu04068;mmu04071;mmu04072;mmu04150;mmu04151;mmu04152;mmu04210;mmu04211;mmu04213;mmu04261;mmu04370;mmu04380;mmu04510;mmu04530;mmu04550;mmu04611;mmu04620;mmu04630;mmu04660;mmu04662;mmu04664;mmu04666;mmu04668;mmu04722;mmu04725;mmu04728;mmu04910;mmu04914;mmu04915;mmu04917;mmu04919;mmu04920;mmu04922;mmu04923;mmu04931;mmu04932;mmu04933;mmu04973;mmu05142;mmu05145;mmu05152;mmu05160;mmu05161;mmu05162;mmu05164;mmu05166;mmu05169;mmu05200;mmu05205;mmu05210;mmu05211;mmu05212;mmu05213;mmu05214;mmu05215;mmu05218;mmu05220;mmu05221;mmu05222;mmu05223;mmu05230;mmu05231 |  | Akt2 | azure4 | 1 | -0.120772946859903 |

  ##### DE Results - Carbohydrate Metabolism genes - DPI: 1 vs.CTRL

  Table displaying each sample's global significance scores and directed global significance scores as defined in the heatmaps above. The global significance score is calculated as the square root of the mean squared t-statistic for the genes in a gene set, with t-statistics coming from the linear regression underlying our differential expression analysis. The directed global significance score is calculated as the square root of the mean signed squared t-statistic for the genes in a gene set, with t-statistics coming from the linear regression underlying our differential expression analysis.

- **Volcano Plot: DPI: 2 vs.CTRL  
  More Plot Information**

  ##### Volcano Plot: DPI: 2 vs.CTRL

  Volcano plot displaying each gene's -log10(p-value) and log2 fold change for the selected covariate. Highly statistically significant genes fall at the top of the plot, and highly differentially expressed genes fall to either side. Genes within the selected gene set are highlighted in orange. Horizontal lines indicate various False Discovery Rate (FDR) thresholds.
- **DE Results - Carbohydrate Metabolism genes - DPI: 2 vs.CTRL  
  More Plot Information  Download CSV Data**

  | Probe Label | Log2 fold change | std error (log2) | Lower confidence limit (log2) | Upper confidence limit (log2) | Linear fold change | Lower confidence limit (linear) | Upper confidence limit (linear) | P-value | BY.p.value | method | Gene.sets | Codeset.Name | Probe.Label | Analyte.Type | Is.Control | Control.Type | Probe.Annotation | KEGG.Pathways | Cell.Type | Official.Gene.Name | volcanocol | volcanopch | tstats.all |
  | --- | --- | --- | --- | --- | --- | --- | --- | --- | --- | --- | --- | --- | --- | --- | --- | --- | --- | --- | --- | --- | --- | --- | --- |
  | Akt2-mRNA | -0.687 | 0.21 | -1.1 | -0.276 | 0.621 | 0.467 | 0.826 | 0.00664 | 0.18 | lm.nb | Adaptive Immune Response, Angiogenesis, Apoptosis, Autophagy, Carbohydrate Metabolism, Cytokine Signaling, Growth Factor Signaling, Innate Immune Response, Insulin Signaling, Neurons and Neurotransmission, Wnt | NS\_MM\_NEUROINFLAM\_V1.0 | Akt2 | mRNA | 0 |  | Wnt;Neurons and Neurotransmission;Insulin Signaling;Innate Immune Response;Growth Factor Signaling;Cytokine Signaling;Carbohydrate Metabolism;Autophagy;Apoptosis;Angiogenesis;Adaptive Immune Response | mmu04010;mmu04012;mmu04014;mmu04015;mmu04022;mmu04024;mmu04062;mmu04066;mmu04068;mmu04071;mmu04072;mmu04150;mmu04151;mmu04152;mmu04210;mmu04211;mmu04213;mmu04261;mmu04370;mmu04380;mmu04510;mmu04530;mmu04550;mmu04611;mmu04620;mmu04630;mmu04660;mmu04662;mmu04664;mmu04666;mmu04668;mmu04722;mmu04725;mmu04728;mmu04910;mmu04914;mmu04915;mmu04917;mmu04919;mmu04920;mmu04922;mmu04923;mmu04931;mmu04932;mmu04933;mmu04973;mmu05142;mmu05145;mmu05152;mmu05160;mmu05161;mmu05162;mmu05164;mmu05166;mmu05169;mmu05200;mmu05205;mmu05210;mmu05211;mmu05212;mmu05213;mmu05214;mmu05215;mmu05218;mmu05220;mmu05221;mmu05222;mmu05223;mmu05230;mmu05231 |  | Akt2 | slateblue4 | 16 | -3.27142857142857 |
  | Pik3r1-mRNA | 0.218 | 0.0891 | 0.043 | 0.392 | 1.16 | 1.03 | 1.31 | 0.031 | 0.577 | loglinear | Adaptive Immune Response, Angiogenesis, Apoptosis, Autophagy, Carbohydrate Metabolism, Cytokine Signaling, Growth Factor Signaling, Innate Immune Response, Insulin Signaling, Lipid Metabolism | NS\_MM\_NEUROINFLAM\_V1.0 | Pik3r1 | mRNA | 0 |  | Lipid Metabolism;Insulin Signaling;Innate Immune Response;Growth Factor Signaling;Cytokine Signaling;Carbohydrate Metabolism;Autophagy;Apoptosis;Angiogenesis;Adaptive Immune Response | mmu04012;mmu04014;mmu04015;mmu04022;mmu04024;mmu04062;mmu04066;mmu04068;mmu04070;mmu04071;mmu04072;mmu04150;mmu04151;mmu04152;mmu04210;mmu04211;mmu04213;mmu04261;mmu04370;mmu04380;mmu04510;mmu04550;mmu04611;mmu04620;mmu04630;mmu04650;mmu04660;mmu04662;mmu04664;mmu04666;mmu04668;mmu04670;mmu04722;mmu04725;mmu04750;mmu04810;mmu04910;mmu04914;mmu04915;mmu04917;mmu04919;mmu04921;mmu04923;mmu04930;mmu04931;mmu04932;mmu04933;mmu04960;mmu04973;mmu05100;mmu05142;mmu05145;mmu05146;mmu05160;mmu05161;mmu05162;mmu05164;mmu05166;mmu05169;mmu05200;mmu05203;mmu05205;mmu05210;mmu05211;mmu05212;mmu05213;mmu05214;mmu05215;mmu05218;mmu05220;mmu05221;mmu05222;mmu05223;mmu05230;mmu05231 |  | Pik3r1 | azure4 | 1 | 2.44668911335578 |
  | Igf1r-mRNA | 0.227 | 0.111 | 0.00951 | 0.444 | 1.17 | 1.01 | 1.36 | 0.0633 | 1 | loglinear | Apoptosis, Autophagy, Carbohydrate Metabolism, Growth Factor Signaling, Insulin Signaling | NS\_MM\_NEUROINFLAM\_V1.0 | Igf1r | mRNA | 0 |  | Insulin Signaling;Growth Factor Signaling;Carbohydrate Metabolism;Autophagy;Apoptosis | mmu04014;mmu04015;mmu04066;mmu04068;mmu04114;mmu04144;mmu04151;mmu04152;mmu04211;mmu04213;mmu04510;mmu04520;mmu04550;mmu04730;mmu04913;mmu04914;mmu05200;mmu05202;mmu05205;mmu05214;mmu05215;mmu05218 |  | Igf1r | azure4 | 1 | 2.04504504504505 |
  | Pik3ca-mRNA | 0.153 | 0.0873 | -0.0183 | 0.324 | 1.11 | 0.987 | 1.25 | 0.106 | 1 | loglinear | Adaptive Immune Response, Angiogenesis, Apoptosis, Autophagy, Carbohydrate Metabolism, Cytokine Signaling, Growth Factor Signaling, Innate Immune Response, Insulin Signaling, Lipid Metabolism | NS\_MM\_NEUROINFLAM\_V1.0 | Pik3ca | mRNA | 0 |  | Lipid Metabolism;Insulin Signaling;Innate Immune Response;Growth Factor Signaling;Cytokine Signaling;Carbohydrate Metabolism;Autophagy;Apoptosis;Angiogenesis;Adaptive Immune Response | mmu00562;mmu04012;mmu04014;mmu04015;mmu04022;mmu04024;mmu04062;mmu04066;mmu04068;mmu04070;mmu04071;mmu04072;mmu04150;mmu04151;mmu04152;mmu04210;mmu04211;mmu04213;mmu04261;mmu04370;mmu04380;mmu04510;mmu04550;mmu04611;mmu04620;mmu04630;mmu04650;mmu04660;mmu04662;mmu04664;mmu04666;mmu04668;mmu04670;mmu04722;mmu04725;mmu04750;mmu04810;mmu04910;mmu04914;mmu04915;mmu04917;mmu04919;mmu04921;mmu04923;mmu04930;mmu04931;mmu04932;mmu04933;mmu04960;mmu04973;mmu05100;mmu05142;mmu05145;mmu05146;mmu05160;mmu05161;mmu05162;mmu05164;mmu05166;mmu05169;mmu05200;mmu05203;mmu05205;mmu05210;mmu05211;mmu05212;mmu05213;mmu05214;mmu05215;mmu05218;mmu05220;mmu05221;mmu05222;mmu05223;mmu05230;mmu05231 |  | Pik3ca | azure4 | 1 | 1.75257731958763 |
  | Akt1-mRNA | -0.0961 | 0.118 | -0.327 | 0.135 | 0.936 | 0.797 | 1.1 | 0.431 | 1 | lm.nb | Adaptive Immune Response, Angiogenesis, Apoptosis, Autophagy, Carbohydrate Metabolism, Cellular Stress, Cytokine Signaling, Growth Factor Signaling, Innate Immune Response, Insulin Signaling, Neurons and Neurotransmission, Wnt | NS\_MM\_NEUROINFLAM\_V1.0 | Akt1 | mRNA | 0 |  | Wnt;Neurons and Neurotransmission;Insulin Signaling;Innate Immune Response;Growth Factor Signaling;Cytokine Signaling;Cellular Stress;Carbohydrate Metabolism;Autophagy;Apoptosis;Angiogenesis;Adaptive Immune Response | mmu04010;mmu04012;mmu04014;mmu04015;mmu04022;mmu04024;mmu04062;mmu04066;mmu04068;mmu04071;mmu04072;mmu04150;mmu04151;mmu04152;mmu04210;mmu04211;mmu04213;mmu04261;mmu04370;mmu04380;mmu04510;mmu04530;mmu04550;mmu04611;mmu04620;mmu04630;mmu04660;mmu04662;mmu04664;mmu04666;mmu04668;mmu04722;mmu04725;mmu04728;mmu04910;mmu04914;mmu04915;mmu04917;mmu04919;mmu04920;mmu04922;mmu04923;mmu04931;mmu04932;mmu04933;mmu04973;mmu05142;mmu05145;mmu05152;mmu05160;mmu05161;mmu05162;mmu05164;mmu05166;mmu05169;mmu05200;mmu05205;mmu05210;mmu05211;mmu05212;mmu05213;mmu05214;mmu05215;mmu05218;mmu05220;mmu05221;mmu05222;mmu05223;mmu05230;mmu05231 |  | Akt1 | azure4 | 1 | -0.814406779661017 |
  | Slco2b1-mRNA | -0.249 | 0.351 | -0.937 | 0.439 | 0.842 | 0.522 | 1.36 | 0.492 | 1 | lm.nb | Carbohydrate Metabolism | NS\_MM\_NEUROINFLAM\_V1.0 | Slco2b1 | mRNA | 0 |  | Carbohydrate Metabolism |  |  | Slco2b1 | azure4 | 1 | -0.709401709401709 |
  | Sirt1-mRNA | 0.0855 | 0.137 | -0.182 | 0.353 | 1.06 | 0.881 | 1.28 | 0.543 | 1 | lm.nb | Carbohydrate Metabolism, Cellular Stress, Epigenetic Regulation | NS\_MM\_NEUROINFLAM\_V1.0 | Sirt1 | mRNA | 0 |  | Epigenetic Regulation;Cellular Stress;Carbohydrate Metabolism | mmu04068;mmu04152;mmu04211;mmu04213;mmu04922;mmu05031;mmu05206 |  | Sirt1 | azure4 | 1 | 0.624087591240876 |
  | Pik3cb-mRNA | -0.0295 | 0.0676 | -0.162 | 0.103 | 0.98 | 0.894 | 1.07 | 0.671 | 1 | loglinear | Adaptive Immune Response, Angiogenesis, Apoptosis, Autophagy, Carbohydrate Metabolism, Cytokine Signaling, Growth Factor Signaling, Innate Immune Response, Insulin Signaling, Lipid Metabolism | NS\_MM\_NEUROINFLAM\_V1.0 | Pik3cb | mRNA | 0 |  | Lipid Metabolism;Insulin Signaling;Innate Immune Response;Growth Factor Signaling;Cytokine Signaling;Carbohydrate Metabolism;Autophagy;Apoptosis;Angiogenesis;Adaptive Immune Response | mmu00562;mmu04012;mmu04014;mmu04015;mmu04022;mmu04024;mmu04062;mmu04066;mmu04068;mmu04070;mmu04071;mmu04072;mmu04150;mmu04151;mmu04152;mmu04210;mmu04211;mmu04213;mmu04261;mmu04370;mmu04380;mmu04510;mmu04550;mmu04611;mmu04620;mmu04630;mmu04650;mmu04660;mmu04662;mmu04664;mmu04666;mmu04668;mmu04670;mmu04722;mmu04725;mmu04750;mmu04810;mmu04910;mmu04914;mmu04915;mmu04917;mmu04919;mmu04921;mmu04923;mmu04930;mmu04931;mmu04932;mmu04933;mmu04960;mmu04973;mmu05100;mmu05142;mmu05145;mmu05146;mmu05160;mmu05161;mmu05162;mmu05164;mmu05166;mmu05169;mmu05200;mmu05203;mmu05205;mmu05210;mmu05211;mmu05212;mmu05213;mmu05214;mmu05215;mmu05218;mmu05220;mmu05221;mmu05222;mmu05223;mmu05230;mmu05231 |  | Pik3cb | azure4 | 1 | -0.436390532544379 |
  | Creb1-mRNA | 0.0241 | 0.124 | -0.219 | 0.267 | 1.02 | 0.859 | 1.2 | 0.849 | 1 | loglinear | Adaptive Immune Response, Carbohydrate Metabolism, DNA Damage, Growth Factor Signaling, Innate Immune Response, Neurons and Neurotransmission, Notch | NS\_MM\_NEUROINFLAM\_V1.0 | Creb1 | mRNA | 0 |  | Notch;Neurons and Neurotransmission;Innate Immune Response;Growth Factor Signaling;DNA Damage;Carbohydrate Metabolism;Adaptive Immune Response | mmu04022;mmu04024;mmu04151;mmu04152;mmu04211;mmu04261;mmu04380;mmu04612;mmu04668;mmu04713;mmu04725;mmu04728;mmu04911;mmu04915;mmu04916;mmu04922;mmu04924;mmu04925;mmu04931;mmu05016;mmu05030;mmu05031;mmu05034;mmu05152;mmu05161;mmu05166;mmu05203;mmu05215 |  | Creb1 | azure4 | 1 | 0.194354838709677 |

  ##### DE Results - Carbohydrate Metabolism genes - DPI: 2 vs.CTRL

  Table displaying each sample's global significance scores and directed global significance scores as defined in the heatmaps above. The global significance score is calculated as the square root of the mean squared t-statistic for the genes in a gene set, with t-statistics coming from the linear regression underlying our differential expression analysis. The directed global significance score is calculated as the square root of the mean signed squared t-statistic for the genes in a gene set, with t-statistics coming from the linear regression underlying our differential expression analysis.

- **Volcano Plot: DPI: 3 vs.CTRL  
  More Plot Information**

  ##### Volcano Plot: DPI: 3 vs.CTRL

  Volcano plot displaying each gene's -log10(p-value) and log2 fold change for the selected covariate. Highly statistically significant genes fall at the top of the plot, and highly differentially expressed genes fall to either side. Genes within the selected gene set are highlighted in orange. Horizontal lines indicate various False Discovery Rate (FDR) thresholds.
- **DE Results - Carbohydrate Metabolism genes - DPI: 3 vs.CTRL  
  More Plot Information  Download CSV Data**

  | Probe Label | Log2 fold change | std error (log2) | Lower confidence limit (log2) | Upper confidence limit (log2) | Linear fold change | Lower confidence limit (linear) | Upper confidence limit (linear) | P-value | BY.p.value | method | Gene.sets | Codeset.Name | Probe.Label | Analyte.Type | Is.Control | Control.Type | Probe.Annotation | KEGG.Pathways | Cell.Type | Official.Gene.Name | volcanocol | volcanopch | tstats.all |
  | --- | --- | --- | --- | --- | --- | --- | --- | --- | --- | --- | --- | --- | --- | --- | --- | --- | --- | --- | --- | --- | --- | --- | --- |
  | Igf1r-mRNA | 0.584 | 0.111 | 0.367 | 0.802 | 1.5 | 1.29 | 1.74 | 0.000199 | 0.0065 | loglinear | Apoptosis, Autophagy, Carbohydrate Metabolism, Growth Factor Signaling, Insulin Signaling | NS\_MM\_NEUROINFLAM\_V1.0 | Igf1r | mRNA | 0 |  | Insulin Signaling;Growth Factor Signaling;Carbohydrate Metabolism;Autophagy;Apoptosis | mmu04014;mmu04015;mmu04066;mmu04068;mmu04114;mmu04144;mmu04151;mmu04152;mmu04211;mmu04213;mmu04510;mmu04520;mmu04550;mmu04730;mmu04913;mmu04914;mmu05200;mmu05202;mmu05205;mmu05214;mmu05215;mmu05218 |  | Igf1r | slateblue1 | 16 | 5.26126126126126 |
  | Pik3r1-mRNA | 0.238 | 0.0891 | 0.0639 | 0.413 | 1.18 | 1.05 | 1.33 | 0.0201 | 0.319 | loglinear | Adaptive Immune Response, Angiogenesis, Apoptosis, Autophagy, Carbohydrate Metabolism, Cytokine Signaling, Growth Factor Signaling, Innate Immune Response, Insulin Signaling, Lipid Metabolism | NS\_MM\_NEUROINFLAM\_V1.0 | Pik3r1 | mRNA | 0 |  | Lipid Metabolism;Insulin Signaling;Innate Immune Response;Growth Factor Signaling;Cytokine Signaling;Carbohydrate Metabolism;Autophagy;Apoptosis;Angiogenesis;Adaptive Immune Response | mmu04012;mmu04014;mmu04015;mmu04022;mmu04024;mmu04062;mmu04066;mmu04068;mmu04070;mmu04071;mmu04072;mmu04150;mmu04151;mmu04152;mmu04210;mmu04211;mmu04213;mmu04261;mmu04370;mmu04380;mmu04510;mmu04550;mmu04611;mmu04620;mmu04630;mmu04650;mmu04660;mmu04662;mmu04664;mmu04666;mmu04668;mmu04670;mmu04722;mmu04725;mmu04750;mmu04810;mmu04910;mmu04914;mmu04915;mmu04917;mmu04919;mmu04921;mmu04923;mmu04930;mmu04931;mmu04932;mmu04933;mmu04960;mmu04973;mmu05100;mmu05142;mmu05145;mmu05146;mmu05160;mmu05161;mmu05162;mmu05164;mmu05166;mmu05169;mmu05200;mmu05203;mmu05205;mmu05210;mmu05211;mmu05212;mmu05213;mmu05214;mmu05215;mmu05218;mmu05220;mmu05221;mmu05222;mmu05223;mmu05230;mmu05231 |  | Pik3r1 | slateblue4 | 16 | 2.67115600448934 |
  | Pik3cb-mRNA | -0.128 | 0.0676 | -0.261 | 0.00424 | 0.915 | 0.835 | 1 | 0.0821 | 0.961 | loglinear | Adaptive Immune Response, Angiogenesis, Apoptosis, Autophagy, Carbohydrate Metabolism, Cytokine Signaling, Growth Factor Signaling, Innate Immune Response, Insulin Signaling, Lipid Metabolism | NS\_MM\_NEUROINFLAM\_V1.0 | Pik3cb | mRNA | 0 |  | Lipid Metabolism;Insulin Signaling;Innate Immune Response;Growth Factor Signaling;Cytokine Signaling;Carbohydrate Metabolism;Autophagy;Apoptosis;Angiogenesis;Adaptive Immune Response | mmu00562;mmu04012;mmu04014;mmu04015;mmu04022;mmu04024;mmu04062;mmu04066;mmu04068;mmu04070;mmu04071;mmu04072;mmu04150;mmu04151;mmu04152;mmu04210;mmu04211;mmu04213;mmu04261;mmu04370;mmu04380;mmu04510;mmu04550;mmu04611;mmu04620;mmu04630;mmu04650;mmu04660;mmu04662;mmu04664;mmu04666;mmu04668;mmu04670;mmu04722;mmu04725;mmu04750;mmu04810;mmu04910;mmu04914;mmu04915;mmu04917;mmu04919;mmu04921;mmu04923;mmu04930;mmu04931;mmu04932;mmu04933;mmu04960;mmu04973;mmu05100;mmu05142;mmu05145;mmu05146;mmu05160;mmu05161;mmu05162;mmu05164;mmu05166;mmu05169;mmu05200;mmu05203;mmu05205;mmu05210;mmu05211;mmu05212;mmu05213;mmu05214;mmu05215;mmu05218;mmu05220;mmu05221;mmu05222;mmu05223;mmu05230;mmu05231 |  | Pik3cb | azure4 | 1 | -1.89349112426036 |
  | Akt2-mRNA | -0.394 | 0.208 | -0.803 | 0.0139 | 0.761 | 0.573 | 1.01 | 0.0827 | 0.961 | lm.nb | Adaptive Immune Response, Angiogenesis, Apoptosis, Autophagy, Carbohydrate Metabolism, Cytokine Signaling, Growth Factor Signaling, Innate Immune Response, Insulin Signaling, Neurons and Neurotransmission, Wnt | NS\_MM\_NEUROINFLAM\_V1.0 | Akt2 | mRNA | 0 |  | Wnt;Neurons and Neurotransmission;Insulin Signaling;Innate Immune Response;Growth Factor Signaling;Cytokine Signaling;Carbohydrate Metabolism;Autophagy;Apoptosis;Angiogenesis;Adaptive Immune Response | mmu04010;mmu04012;mmu04014;mmu04015;mmu04022;mmu04024;mmu04062;mmu04066;mmu04068;mmu04071;mmu04072;mmu04150;mmu04151;mmu04152;mmu04210;mmu04211;mmu04213;mmu04261;mmu04370;mmu04380;mmu04510;mmu04530;mmu04550;mmu04611;mmu04620;mmu04630;mmu04660;mmu04662;mmu04664;mmu04666;mmu04668;mmu04722;mmu04725;mmu04728;mmu04910;mmu04914;mmu04915;mmu04917;mmu04919;mmu04920;mmu04922;mmu04923;mmu04931;mmu04932;mmu04933;mmu04973;mmu05142;mmu05145;mmu05152;mmu05160;mmu05161;mmu05162;mmu05164;mmu05166;mmu05169;mmu05200;mmu05205;mmu05210;mmu05211;mmu05212;mmu05213;mmu05214;mmu05215;mmu05218;mmu05220;mmu05221;mmu05222;mmu05223;mmu05230;mmu05231 |  | Akt2 | azure4 | 1 | -1.89423076923077 |
  | Creb1-mRNA | -0.182 | 0.124 | -0.425 | 0.0616 | 0.882 | 0.745 | 1.04 | 0.169 | 1 | loglinear | Adaptive Immune Response, Carbohydrate Metabolism, DNA Damage, Growth Factor Signaling, Innate Immune Response, Neurons and Neurotransmission, Notch | NS\_MM\_NEUROINFLAM\_V1.0 | Creb1 | mRNA | 0 |  | Notch;Neurons and Neurotransmission;Innate Immune Response;Growth Factor Signaling;DNA Damage;Carbohydrate Metabolism;Adaptive Immune Response | mmu04022;mmu04024;mmu04151;mmu04152;mmu04211;mmu04261;mmu04380;mmu04612;mmu04668;mmu04713;mmu04725;mmu04728;mmu04911;mmu04915;mmu04916;mmu04922;mmu04924;mmu04925;mmu04931;mmu05016;mmu05030;mmu05031;mmu05034;mmu05152;mmu05161;mmu05166;mmu05203;mmu05215 |  | Creb1 | azure4 | 1 | -1.46774193548387 |
  | Pik3ca-mRNA | -0.0826 | 0.0873 | -0.254 | 0.0886 | 0.944 | 0.839 | 1.06 | 0.363 | 1 | loglinear | Adaptive Immune Response, Angiogenesis, Apoptosis, Autophagy, Carbohydrate Metabolism, Cytokine Signaling, Growth Factor Signaling, Innate Immune Response, Insulin Signaling, Lipid Metabolism | NS\_MM\_NEUROINFLAM\_V1.0 | Pik3ca | mRNA | 0 |  | Lipid Metabolism;Insulin Signaling;Innate Immune Response;Growth Factor Signaling;Cytokine Signaling;Carbohydrate Metabolism;Autophagy;Apoptosis;Angiogenesis;Adaptive Immune Response | mmu00562;mmu04012;mmu04014;mmu04015;mmu04022;mmu04024;mmu04062;mmu04066;mmu04068;mmu04070;mmu04071;mmu04072;mmu04150;mmu04151;mmu04152;mmu04210;mmu04211;mmu04213;mmu04261;mmu04370;mmu04380;mmu04510;mmu04550;mmu04611;mmu04620;mmu04630;mmu04650;mmu04660;mmu04662;mmu04664;mmu04666;mmu04668;mmu04670;mmu04722;mmu04725;mmu04750;mmu04810;mmu04910;mmu04914;mmu04915;mmu04917;mmu04919;mmu04921;mmu04923;mmu04930;mmu04931;mmu04932;mmu04933;mmu04960;mmu04973;mmu05100;mmu05142;mmu05145;mmu05146;mmu05160;mmu05161;mmu05162;mmu05164;mmu05166;mmu05169;mmu05200;mmu05203;mmu05205;mmu05210;mmu05211;mmu05212;mmu05213;mmu05214;mmu05215;mmu05218;mmu05220;mmu05221;mmu05222;mmu05223;mmu05230;mmu05231 |  | Pik3ca | azure4 | 1 | -0.946162657502864 |
  | Slco2b1-mRNA | 0.187 | 0.346 | -0.492 | 0.866 | 1.14 | 0.711 | 1.82 | 0.599 | 1 | lm.nb | Carbohydrate Metabolism | NS\_MM\_NEUROINFLAM\_V1.0 | Slco2b1 | mRNA | 0 |  | Carbohydrate Metabolism |  |  | Slco2b1 | azure4 | 1 | 0.540462427745665 |
  | Sirt1-mRNA | 0.0105 | 0.137 | -0.258 | 0.279 | 1.01 | 0.836 | 1.21 | 0.94 | 1 | lm.nb | Carbohydrate Metabolism, Cellular Stress, Epigenetic Regulation | NS\_MM\_NEUROINFLAM\_V1.0 | Sirt1 | mRNA | 0 |  | Epigenetic Regulation;Cellular Stress;Carbohydrate Metabolism | mmu04068;mmu04152;mmu04211;mmu04213;mmu04922;mmu05031;mmu05206 |  | Sirt1 | azure4 | 1 | 0.0766423357664234 |
  | Akt1-mRNA | -0.00678 | 0.118 | -0.238 | 0.224 | 0.995 | 0.848 | 1.17 | 0.955 | 1 | lm.nb | Adaptive Immune Response, Angiogenesis, Apoptosis, Autophagy, Carbohydrate Metabolism, Cellular Stress, Cytokine Signaling, Growth Factor Signaling, Innate Immune Response, Insulin Signaling, Neurons and Neurotransmission, Wnt | NS\_MM\_NEUROINFLAM\_V1.0 | Akt1 | mRNA | 0 |  | Wnt;Neurons and Neurotransmission;Insulin Signaling;Innate Immune Response;Growth Factor Signaling;Cytokine Signaling;Cellular Stress;Carbohydrate Metabolism;Autophagy;Apoptosis;Angiogenesis;Adaptive Immune Response | mmu04010;mmu04012;mmu04014;mmu04015;mmu04022;mmu04024;mmu04062;mmu04066;mmu04068;mmu04071;mmu04072;mmu04150;mmu04151;mmu04152;mmu04210;mmu04211;mmu04213;mmu04261;mmu04370;mmu04380;mmu04510;mmu04530;mmu04550;mmu04611;mmu04620;mmu04630;mmu04660;mmu04662;mmu04664;mmu04666;mmu04668;mmu04722;mmu04725;mmu04728;mmu04910;mmu04914;mmu04915;mmu04917;mmu04919;mmu04920;mmu04922;mmu04923;mmu04931;mmu04932;mmu04933;mmu04973;mmu05142;mmu05145;mmu05152;mmu05160;mmu05161;mmu05162;mmu05164;mmu05166;mmu05169;mmu05200;mmu05205;mmu05210;mmu05211;mmu05212;mmu05213;mmu05214;mmu05215;mmu05218;mmu05220;mmu05221;mmu05222;mmu05223;mmu05230;mmu05231 |  | Akt1 | azure4 | 1 | -0.0574576271186441 |

  ##### DE Results - Carbohydrate Metabolism genes - DPI: 3 vs.CTRL

  Table displaying each sample's global significance scores and directed global significance scores as defined in the heatmaps above. The global significance score is calculated as the square root of the mean squared t-statistic for the genes in a gene set, with t-statistics coming from the linear regression underlying our differential expression analysis. The directed global significance score is calculated as the square root of the mean signed squared t-statistic for the genes in a gene set, with t-statistics coming from the linear regression underlying our differential expression analysis.

DPI:
- DPI: differential expression in 1 vs. baseline of CTRL
- DPI: differential expression in 2 vs. baseline of CTRL
- DPI: differential expression in 3 vs. baseline of CTRL

- **Volcano Plot: DPI: 1 vs.CTRL  
  More Plot Information**

  ##### Volcano Plot: DPI: 1 vs.CTRL

  Volcano plot displaying each gene's -log10(p-value) and log2 fold change for the selected covariate. Highly statistically significant genes fall at the top of the plot, and highly differentially expressed genes fall to either side. Genes within the selected gene set are highlighted in orange. Horizontal lines indicate various False Discovery Rate (FDR) thresholds.
- **DE Results - Cell Cycle genes - DPI: 1 vs.CTRL  
  More Plot Information  Download CSV Data**

  | Probe Label | Log2 fold change | std error (log2) | Lower confidence limit (log2) | Upper confidence limit (log2) | Linear fold change | Lower confidence limit (linear) | Upper confidence limit (linear) | P-value | BY.p.value | method | Gene.sets | Codeset.Name | Probe.Label | Analyte.Type | Is.Control | Control.Type | Probe.Annotation | KEGG.Pathways | Cell.Type | Official.Gene.Name | volcanocol | volcanopch | tstats.all |
  | --- | --- | --- | --- | --- | --- | --- | --- | --- | --- | --- | --- | --- | --- | --- | --- | --- | --- | --- | --- | --- | --- | --- | --- |
  | Optn-mRNA | 0.299 | 0.0828 | 0.137 | 0.461 | 1.23 | 1.1 | 1.38 | 0.00357 | 0.558 | loglinear | Autophagy, Cell Cycle, Microglia Function | NS\_MM\_NEUROINFLAM\_V1.0 | Optn | mRNA | 0 |  | Microglia Function;Cell Cycle;Autophagy |  |  | Optn | azure4 | 1 | 3.61111111111111 |
  | Cdkn1c-mRNA | 0.665 | 0.226 | 0.222 | 1.11 | 1.59 | 1.17 | 2.15 | 0.0123 | 0.875 | lm.nb | Cell Cycle, DNA Damage | NS\_MM\_NEUROINFLAM\_V1.0 | Cdkn1c | mRNA | 0 |  | DNA Damage;Cell Cycle | mmu04110 |  | Cdkn1c | azure4 | 1 | 2.94247787610619 |
  | Cks1b-mRNA | 0.429 | 0.148 | 0.139 | 0.718 | 1.35 | 1.1 | 1.65 | 0.0132 | 0.927 | loglinear | Cell Cycle | NS\_MM\_NEUROINFLAM\_V1.0 | Cks1b | mRNA | 0 |  | Cell Cycle | mmu05200;mmu05222 |  | Cks1b | azure4 | 1 | 2.89864864864865 |
  | Prkdc-mRNA | -0.365 | 0.133 | -0.626 | -0.103 | 0.777 | 0.648 | 0.931 | 0.0181 | 1 | loglinear | Cell Cycle, DNA Damage, Innate Immune Response | NS\_MM\_NEUROINFLAM\_V1.0 | Prkdc | mRNA | 0 |  | Innate Immune Response;DNA Damage;Cell Cycle | mmu03450;mmu04110 |  | Prkdc | azure4 | 1 | -2.74436090225564 |
  | Rpa1-mRNA | 0.133 | 0.0503 | 0.0349 | 0.232 | 1.1 | 1.02 | 1.17 | 0.021 | 1 | loglinear | Cell Cycle, Cellular Stress, DNA Damage | NS\_MM\_NEUROINFLAM\_V1.0 | Rpa1 | mRNA | 0 |  | DNA Damage;Cellular Stress;Cell Cycle | mmu03030;mmu03420;mmu03430;mmu03440;mmu03460 |  | Rpa1 | azure4 | 1 | 2.6441351888668 |
  | Cdkn1a-mRNA | 0.349 | 0.153 | 0.0486 | 0.649 | 1.27 | 1.03 | 1.57 | 0.0419 | 1 | lm.nb | Adaptive Immune Response, Cell Cycle, Cellular Stress, Cytokine Signaling, DNA Damage, Growth Factor Signaling | NS\_MM\_NEUROINFLAM\_V1.0 | Cdkn1a | mRNA | 0 |  | Growth Factor Signaling;DNA Damage;Cytokine Signaling;Cellular Stress;Cell Cycle;Adaptive Immune Response | mmu04012;mmu04066;mmu04068;mmu04110;mmu04115;mmu04151;mmu04630;mmu04921;mmu05160;mmu05161;mmu05166;mmu05169;mmu05200;mmu05202;mmu05203;mmu05205;mmu05206;mmu05214;mmu05215;mmu05218;mmu05219;mmu05220 |  | Cdkn1a | azure4 | 1 | 2.28104575163399 |
  | Gadd45g-mRNA | 0.422 | 0.214 | 0.00309 | 0.841 | 1.34 | 1 | 1.79 | 0.0718 | 1 | lm.nb | Cell Cycle, DNA Damage, Growth Factor Signaling | NS\_MM\_NEUROINFLAM\_V1.0 | Gadd45g | mRNA | 0 |  | Growth Factor Signaling;DNA Damage;Cell Cycle | mmu04010;mmu04068;mmu04110;mmu04115 |  | Gadd45g | azure4 | 1 | 1.97196261682243 |
  | Tubb4a-mRNA | 0.183 | 0.098 | -0.00886 | 0.375 | 1.14 | 0.994 | 1.3 | 0.0861 | 1 | loglinear | Adaptive Immune Response, Apoptosis, Cell Cycle, Neurons and Neurotransmission | NS\_MM\_NEUROINFLAM\_V1.0 | Tubb4a | mRNA | 0 |  | Neurons and Neurotransmission;Cell Cycle;Apoptosis;Adaptive Immune Response | mmu04145;mmu04540 |  | Tubb4a | azure4 | 1 | 1.86734693877551 |
  | Smarca5-mRNA | -0.0842 | 0.0459 | -0.174 | 0.00583 | 0.943 | 0.886 | 1 | 0.0917 | 1 | loglinear | Cell Cycle, DNA Damage, Epigenetic Regulation | NS\_MM\_NEUROINFLAM\_V1.0 | Smarca5 | mRNA | 0 |  | Epigenetic Regulation;DNA Damage;Cell Cycle |  |  | Smarca5 | azure4 | 1 | -1.83442265795207 |
  | Hdac1-mRNA | 0.25 | 0.14 | -0.0256 | 0.525 | 1.19 | 0.982 | 1.44 | 0.101 | 1 | loglinear | Cell Cycle, Epigenetic Regulation, Growth Factor Signaling, Notch, Wnt | NS\_MM\_NEUROINFLAM\_V1.0 | Hdac1 | mRNA | 0 |  | Wnt;Notch;Growth Factor Signaling;Epigenetic Regulation;Cell Cycle | mmu04110;mmu04213;mmu04330;mmu04919;mmu05016;mmu05031;mmu05034;mmu05169;mmu05200;mmu05202;mmu05203;mmu05220 |  | Hdac1 | azure4 | 1 | 1.78571428571429 |
  | Rnf8-mRNA | 0.192 | 0.122 | -0.0461 | 0.43 | 1.14 | 0.969 | 1.35 | 0.14 | 1 | lm.nb | Cell Cycle, DNA Damage | NS\_MM\_NEUROINFLAM\_V1.0 | Rnf8 | mRNA | 0 |  | DNA Damage;Cell Cycle |  |  | Rnf8 | azure4 | 1 | 1.57377049180328 |
  | E2f1-mRNA | -0.235 | 0.153 | -0.534 | 0.0648 | 0.85 | 0.691 | 1.05 | 0.15 | 1 | loglinear | Apoptosis, Cell Cycle, Cellular Stress, Notch | NS\_MM\_NEUROINFLAM\_V1.0 | E2f1 | mRNA | 0 |  | Notch;Cellular Stress;Cell Cycle;Apoptosis | mmu04110;mmu05161;mmu05166;mmu05200;mmu05206;mmu05212;mmu05214;mmu05215;mmu05218;mmu05219;mmu05220;mmu05222;mmu05223 |  | E2f1 | azure4 | 1 | -1.5359477124183 |
  | Mcm2-mRNA | 0.512 | 0.335 | -0.145 | 1.17 | 1.43 | 0.904 | 2.25 | 0.153 | 1 | lm.nb | Cell Cycle, Inflammatory Signaling | NS\_MM\_NEUROINFLAM\_V1.0 | Mcm2 | mRNA | 0 |  | Inflammatory Signaling;Cell Cycle | mmu03030;mmu04110 |  | Mcm2 | azure4 | 1 | 1.52835820895522 |
  | Myc-mRNA | 0.209 | 0.14 | -0.065 | 0.483 | 1.16 | 0.956 | 1.4 | 0.161 | 1 | loglinear | Apoptosis, Cell Cycle, Cytokine Signaling, Growth Factor Signaling, Notch, Wnt | NS\_MM\_NEUROINFLAM\_V1.0 | Myc | mRNA | 0 |  | Wnt;Notch;Growth Factor Signaling;Cytokine Signaling;Cell Cycle;Apoptosis | mmu04010;mmu04012;mmu04110;mmu04151;mmu04310;mmu04350;mmu04390;mmu04550;mmu04630;mmu04919;mmu05161;mmu05166;mmu05169;mmu05200;mmu05202;mmu05205;mmu05206;mmu05210;mmu05213;mmu05216;mmu05219;mmu05220;mmu05221;mmu05222;mmu05230 |  | Myc | azure4 | 1 | 1.49285714285714 |
  | Prkar2b-mRNA | -0.136 | 0.0919 | -0.316 | 0.0445 | 0.91 | 0.803 | 1.03 | 0.166 | 1 | loglinear | Apoptosis, Cell Cycle, Growth Factor Signaling | NS\_MM\_NEUROINFLAM\_V1.0 | Prkar2b | mRNA | 0 |  | Growth Factor Signaling;Cell Cycle;Apoptosis | mmu04210;mmu04910 |  | Prkar2b | azure4 | 1 | -1.47986942328618 |
  | Psmb8-mRNA | 0.333 | 0.226 | -0.11 | 0.775 | 1.26 | 0.927 | 1.71 | 0.166 | 1 | lm.nb | Adaptive Immune Response, Angiogenesis, Apoptosis, Astrocyte Function, Cell Cycle, Cytokine Signaling, Growth Factor Signaling, Inflammatory Signaling, Insulin Signaling, Microglia Function, NF-kB, Wnt | NS\_MM\_NEUROINFLAM\_V1.0 | Psmb8 | mRNA | 0 |  | Wnt;NF-kB;Microglia Function;Insulin Signaling;Inflammatory Signaling;Growth Factor Signaling;Cytokine Signaling;Cell Cycle;Astrocyte Function;Apoptosis;Angiogenesis;Adaptive Immune Response |  |  | Psmb8 | azure4 | 1 | 1.47345132743363 |
  | Mdm2-mRNA | 0.11 | 0.0766 | -0.0405 | 0.26 | 1.08 | 0.972 | 1.2 | 0.178 | 1 | loglinear | Adaptive Immune Response, Apoptosis, Cell Cycle, Cellular Stress, DNA Damage, Growth Factor Signaling, Neurons and Neurotransmission | NS\_MM\_NEUROINFLAM\_V1.0 | Mdm2 | mRNA | 0 |  | Neurons and Neurotransmission;Growth Factor Signaling;DNA Damage;Cellular Stress;Cell Cycle;Apoptosis;Adaptive Immune Response | mmu04068;mmu04110;mmu04115;mmu04120;mmu04144;mmu04151;mmu04919;mmu05169;mmu05200;mmu05202;mmu05203;mmu05205;mmu05206;mmu05214;mmu05215;mmu05218;mmu05219;mmu05220 |  | Mdm2 | azure4 | 1 | 1.43603133159269 |
  | Ccni-mRNA | 0.0845 | 0.0602 | -0.0334 | 0.202 | 1.06 | 0.977 | 1.15 | 0.185 | 1 | loglinear | Cell Cycle | NS\_MM\_NEUROINFLAM\_V1.0 | Ccni | mRNA | 0 |  | Cell Cycle |  |  | Ccni | azure4 | 1 | 1.40365448504983 |
  | Cables1-mRNA | 0.159 | 0.118 | -0.0726 | 0.392 | 1.12 | 0.951 | 1.31 | 0.203 | 1 | loglinear | Cell Cycle | NS\_MM\_NEUROINFLAM\_V1.0 | Cables1 | mRNA | 0 |  | Cell Cycle |  |  | Cables1 | azure4 | 1 | 1.34745762711864 |
  | Anapc15-mRNA | -0.0965 | 0.0737 | -0.241 | 0.0479 | 0.935 | 0.846 | 1.03 | 0.215 | 1 | loglinear | Cell Cycle, Cellular Stress | NS\_MM\_NEUROINFLAM\_V1.0 | Anapc15 | mRNA | 0 |  | Cellular Stress;Cell Cycle |  |  | Anapc15 | azure4 | 1 | -1.30936227951153 |

  ##### DE Results - Cell Cycle genes - DPI: 1 vs.CTRL

  Table displaying each sample's global significance scores and directed global significance scores as defined in the heatmaps above. The global significance score is calculated as the square root of the mean squared t-statistic for the genes in a gene set, with t-statistics coming from the linear regression underlying our differential expression analysis. The directed global significance score is calculated as the square root of the mean signed squared t-statistic for the genes in a gene set, with t-statistics coming from the linear regression underlying our differential expression analysis.

- **Volcano Plot: DPI: 2 vs.CTRL  
  More Plot Information**

  ##### Volcano Plot: DPI: 2 vs.CTRL

  Volcano plot displaying each gene's -log10(p-value) and log2 fold change for the selected covariate. Highly statistically significant genes fall at the top of the plot, and highly differentially expressed genes fall to either side. Genes within the selected gene set are highlighted in orange. Horizontal lines indicate various False Discovery Rate (FDR) thresholds.
- **DE Results - Cell Cycle genes - DPI: 2 vs.CTRL  
  More Plot Information  Download CSV Data**

  | Probe Label | Log2 fold change | std error (log2) | Lower confidence limit (log2) | Upper confidence limit (log2) | Linear fold change | Lower confidence limit (linear) | Upper confidence limit (linear) | P-value | BY.p.value | method | Gene.sets | Codeset.Name | Probe.Label | Analyte.Type | Is.Control | Control.Type | Probe.Annotation | KEGG.Pathways | Cell.Type | Official.Gene.Name | volcanocol | volcanopch | tstats.all |
  | --- | --- | --- | --- | --- | --- | --- | --- | --- | --- | --- | --- | --- | --- | --- | --- | --- | --- | --- | --- | --- | --- | --- | --- |
  | Cdkn1a-mRNA | 2.21 | 0.15 | 1.92 | 2.51 | 4.64 | 3.78 | 5.68 | 4.64e-09 | 3.71e-06 | lm.nb | Adaptive Immune Response, Cell Cycle, Cellular Stress, Cytokine Signaling, DNA Damage, Growth Factor Signaling | NS\_MM\_NEUROINFLAM\_V1.0 | Cdkn1a | mRNA | 0 |  | Growth Factor Signaling;DNA Damage;Cytokine Signaling;Cellular Stress;Cell Cycle;Adaptive Immune Response | mmu04012;mmu04066;mmu04068;mmu04110;mmu04115;mmu04151;mmu04630;mmu04921;mmu05160;mmu05161;mmu05166;mmu05169;mmu05200;mmu05202;mmu05203;mmu05205;mmu05206;mmu05214;mmu05215;mmu05218;mmu05219;mmu05220 |  | Cdkn1a | slateblue1 | 16 | 14.7333333333333 |
  | Psmb8-mRNA | 2.78 | 0.196 | 2.39 | 3.16 | 6.85 | 5.25 | 8.94 | 7.51e-09 | 4.29e-06 | lm.nb | Adaptive Immune Response, Angiogenesis, Apoptosis, Astrocyte Function, Cell Cycle, Cytokine Signaling, Growth Factor Signaling, Inflammatory Signaling, Insulin Signaling, Microglia Function, NF-kB, Wnt | NS\_MM\_NEUROINFLAM\_V1.0 | Psmb8 | mRNA | 0 |  | Wnt;NF-kB;Microglia Function;Insulin Signaling;Inflammatory Signaling;Growth Factor Signaling;Cytokine Signaling;Cell Cycle;Astrocyte Function;Apoptosis;Angiogenesis;Adaptive Immune Response |  |  | Psmb8 | slateblue1 | 16 | 14.1836734693878 |
  | Tubb3-mRNA | -0.383 | 0.0494 | -0.48 | -0.286 | 0.767 | 0.717 | 0.82 | 5.22e-06 | 0.000718 | loglinear | Adaptive Immune Response, Apoptosis, Cell Cycle, Neurons and Neurotransmission | NS\_MM\_NEUROINFLAM\_V1.0 | Tubb3 | mRNA | 0 |  | Neurons and Neurotransmission;Cell Cycle;Apoptosis;Adaptive Immune Response | mmu04145;mmu04540 |  | Tubb3 | slateblue1 | 16 | -7.75303643724696 |
  | Prkar2b-mRNA | -0.548 | 0.0919 | -0.728 | -0.368 | 0.684 | 0.604 | 0.775 | 6.54e-05 | 0.00556 | loglinear | Apoptosis, Cell Cycle, Growth Factor Signaling | NS\_MM\_NEUROINFLAM\_V1.0 | Prkar2b | mRNA | 0 |  | Growth Factor Signaling;Cell Cycle;Apoptosis | mmu04210;mmu04910 |  | Prkar2b | slateblue1 | 16 | -5.96300326441785 |
  | Sumo1-mRNA | -0.199 | 0.0361 | -0.269 | -0.128 | 0.871 | 0.83 | 0.915 | 0.000135 | 0.00884 | loglinear | Cell Cycle, Cytokine Signaling, DNA Damage, Inflammatory Signaling | NS\_MM\_NEUROINFLAM\_V1.0 | Sumo1 | mRNA | 0 |  | Inflammatory Signaling;DNA Damage;Cytokine Signaling;Cell Cycle |  |  | Sumo1 | slateblue1 | 16 | -5.51246537396122 |
  | Anapc15-mRNA | -0.364 | 0.0737 | -0.508 | -0.22 | 0.777 | 0.703 | 0.859 | 0.000341 | 0.02 | loglinear | Cell Cycle, Cellular Stress | NS\_MM\_NEUROINFLAM\_V1.0 | Anapc15 | mRNA | 0 |  | Cellular Stress;Cell Cycle |  |  | Anapc15 | slateblue2 | 16 | -4.93894165535957 |
  | Mcm5-mRNA | -0.68 | 0.193 | -1.06 | -0.302 | 0.624 | 0.48 | 0.811 | 0.00417 | 0.124 | lm.nb | Cell Cycle, Inflammatory Signaling | NS\_MM\_NEUROINFLAM\_V1.0 | Mcm5 | mRNA | 0 |  | Inflammatory Signaling;Cell Cycle | mmu03030;mmu04110 |  | Mcm5 | slateblue4 | 16 | -3.52331606217617 |
  | Hdac2-mRNA | -0.239 | 0.0775 | -0.391 | -0.0871 | 0.847 | 0.763 | 0.941 | 0.00946 | 0.24 | loglinear | Cell Cycle, Cellular Stress, Epigenetic Regulation, Growth Factor Signaling, Notch | NS\_MM\_NEUROINFLAM\_V1.0 | Hdac2 | mRNA | 0 |  | Notch;Growth Factor Signaling;Epigenetic Regulation;Cellular Stress;Cell Cycle | mmu04110;mmu04213;mmu04330;mmu04919;mmu05016;mmu05034;mmu05169;mmu05200;mmu05202;mmu05203;mmu05220 |  | Hdac2 | slateblue4 | 16 | -3.08387096774194 |
  | Timeless-mRNA | 0.559 | 0.194 | 0.18 | 0.938 | 1.47 | 1.13 | 1.92 | 0.0136 | 0.322 | loglinear | Cell Cycle, DNA Damage | NS\_MM\_NEUROINFLAM\_V1.0 | Timeless | mRNA | 0 |  | DNA Damage;Cell Cycle |  |  | Timeless | slateblue4 | 16 | 2.88144329896907 |
  | Fen1-mRNA | 0.405 | 0.146 | 0.119 | 0.691 | 1.32 | 1.09 | 1.61 | 0.0168 | 0.376 | loglinear | Cell Cycle, DNA Damage | NS\_MM\_NEUROINFLAM\_V1.0 | Fen1 | mRNA | 0 |  | DNA Damage;Cell Cycle | mmu03030;mmu03410;mmu03450 |  | Fen1 | slateblue4 | 16 | 2.77397260273973 |
  | Mcm6-mRNA | -0.439 | 0.16 | -0.752 | -0.126 | 0.737 | 0.594 | 0.916 | 0.0176 | 0.388 | lm.nb | Cell Cycle | NS\_MM\_NEUROINFLAM\_V1.0 | Mcm6 | mRNA | 0 |  | Cell Cycle | mmu03030;mmu04110 |  | Mcm6 | slateblue4 | 16 | -2.74375 |
  | Cables1-mRNA | 0.293 | 0.118 | 0.061 | 0.525 | 1.23 | 1.04 | 1.44 | 0.0292 | 0.561 | loglinear | Cell Cycle | NS\_MM\_NEUROINFLAM\_V1.0 | Cables1 | mRNA | 0 |  | Cell Cycle |  |  | Cables1 | azure4 | 1 | 2.48305084745763 |
  | Tubb4a-mRNA | -0.241 | 0.098 | -0.433 | -0.0492 | 0.846 | 0.741 | 0.966 | 0.0299 | 0.566 | loglinear | Adaptive Immune Response, Apoptosis, Cell Cycle, Neurons and Neurotransmission | NS\_MM\_NEUROINFLAM\_V1.0 | Tubb4a | mRNA | 0 |  | Neurons and Neurotransmission;Cell Cycle;Apoptosis;Adaptive Immune Response | mmu04145;mmu04540 |  | Tubb4a | azure4 | 1 | -2.45918367346939 |
  | Smarca5-mRNA | -0.112 | 0.0459 | -0.202 | -0.0221 | 0.925 | 0.869 | 0.985 | 0.0311 | 0.577 | loglinear | Cell Cycle, DNA Damage, Epigenetic Regulation | NS\_MM\_NEUROINFLAM\_V1.0 | Smarca5 | mRNA | 0 |  | Epigenetic Regulation;DNA Damage;Cell Cycle |  |  | Smarca5 | azure4 | 1 | -2.4400871459695 |
  | Myc-mRNA | 0.32 | 0.14 | 0.0462 | 0.594 | 1.25 | 1.03 | 1.51 | 0.0409 | 0.723 | loglinear | Apoptosis, Cell Cycle, Cytokine Signaling, Growth Factor Signaling, Notch, Wnt | NS\_MM\_NEUROINFLAM\_V1.0 | Myc | mRNA | 0 |  | Wnt;Notch;Growth Factor Signaling;Cytokine Signaling;Cell Cycle;Apoptosis | mmu04010;mmu04012;mmu04110;mmu04151;mmu04310;mmu04350;mmu04390;mmu04550;mmu04630;mmu04919;mmu05161;mmu05166;mmu05169;mmu05200;mmu05202;mmu05205;mmu05206;mmu05210;mmu05213;mmu05216;mmu05219;mmu05220;mmu05221;mmu05222;mmu05230 |  | Myc | azure4 | 1 | 2.28571428571429 |
  | Mcm2-mRNA | -0.827 | 0.369 | -1.55 | -0.104 | 0.564 | 0.342 | 0.93 | 0.0446 | 0.778 | lm.nb | Cell Cycle, Inflammatory Signaling | NS\_MM\_NEUROINFLAM\_V1.0 | Mcm2 | mRNA | 0 |  | Inflammatory Signaling;Cell Cycle | mmu03030;mmu04110 |  | Mcm2 | azure4 | 1 | -2.24119241192412 |
  | Gadd45g-mRNA | 0.469 | 0.214 | 0.051 | 0.888 | 1.38 | 1.04 | 1.85 | 0.0483 | 0.833 | lm.nb | Cell Cycle, DNA Damage, Growth Factor Signaling | NS\_MM\_NEUROINFLAM\_V1.0 | Gadd45g | mRNA | 0 |  | Growth Factor Signaling;DNA Damage;Cell Cycle | mmu04010;mmu04068;mmu04110;mmu04115 |  | Gadd45g | azure4 | 1 | 2.19158878504673 |
  | Rpa1-mRNA | 0.0941 | 0.0503 | -0.00438 | 0.193 | 1.07 | 0.997 | 1.14 | 0.0856 | 1 | loglinear | Cell Cycle, Cellular Stress, DNA Damage | NS\_MM\_NEUROINFLAM\_V1.0 | Rpa1 | mRNA | 0 |  | DNA Damage;Cellular Stress;Cell Cycle | mmu03030;mmu03420;mmu03430;mmu03440;mmu03460 |  | Rpa1 | azure4 | 1 | 1.87077534791253 |
  | Rad50-mRNA | -0.386 | 0.208 | -0.795 | 0.0225 | 0.765 | 0.576 | 1.02 | 0.0888 | 1 | loglinear | Cell Cycle, Cellular Stress, DNA Damage | NS\_MM\_NEUROINFLAM\_V1.0 | Rad50 | mRNA | 0 |  | DNA Damage;Cellular Stress;Cell Cycle | mmu03440;mmu03450 |  | Rad50 | azure4 | 1 | -1.85576923076923 |
  | Atm-mRNA | -0.267 | 0.151 | -0.564 | 0.0294 | 0.831 | 0.676 | 1.02 | 0.103 | 1 | lm.nb | Apoptosis, Cell Cycle, Cellular Stress, DNA Damage, NF-kB | NS\_MM\_NEUROINFLAM\_V1.0 | Atm | mRNA | 0 |  | NF-kB;DNA Damage;Cellular Stress;Cell Cycle;Apoptosis | mmu04064;mmu04068;mmu04110;mmu04115;mmu04210;mmu05166;mmu05202;mmu05206 |  | Atm | azure4 | 1 | -1.7682119205298 |

  ##### DE Results - Cell Cycle genes - DPI: 2 vs.CTRL

  Table displaying each sample's global significance scores and directed global significance scores as defined in the heatmaps above. The global significance score is calculated as the square root of the mean squared t-statistic for the genes in a gene set, with t-statistics coming from the linear regression underlying our differential expression analysis. The directed global significance score is calculated as the square root of the mean signed squared t-statistic for the genes in a gene set, with t-statistics coming from the linear regression underlying our differential expression analysis.

- **Volcano Plot: DPI: 3 vs.CTRL  
  More Plot Information**

  ##### Volcano Plot: DPI: 3 vs.CTRL

  Volcano plot displaying each gene's -log10(p-value) and log2 fold change for the selected covariate. Highly statistically significant genes fall at the top of the plot, and highly differentially expressed genes fall to either side. Genes within the selected gene set are highlighted in orange. Horizontal lines indicate various False Discovery Rate (FDR) thresholds.
- **DE Results - Cell Cycle genes - DPI: 3 vs.CTRL  
  More Plot Information  Download CSV Data**

  | Probe Label | Log2 fold change | std error (log2) | Lower confidence limit (log2) | Upper confidence limit (log2) | Linear fold change | Lower confidence limit (linear) | Upper confidence limit (linear) | P-value | BY.p.value | method | Gene.sets | Codeset.Name | Probe.Label | Analyte.Type | Is.Control | Control.Type | Probe.Annotation | KEGG.Pathways | Cell.Type | Official.Gene.Name | volcanocol | volcanopch | tstats.all |
  | --- | --- | --- | --- | --- | --- | --- | --- | --- | --- | --- | --- | --- | --- | --- | --- | --- | --- | --- | --- | --- | --- | --- | --- |
  | Psmb8-mRNA | 4.35 | 0.191 | 3.98 | 4.73 | 20.4 | 15.7 | 26.5 | 3.09e-11 | 3.33e-08 | lm.nb | Adaptive Immune Response, Angiogenesis, Apoptosis, Astrocyte Function, Cell Cycle, Cytokine Signaling, Growth Factor Signaling, Inflammatory Signaling, Insulin Signaling, Microglia Function, NF-kB, Wnt | NS\_MM\_NEUROINFLAM\_V1.0 | Psmb8 | mRNA | 0 |  | Wnt;NF-kB;Microglia Function;Insulin Signaling;Inflammatory Signaling;Growth Factor Signaling;Cytokine Signaling;Cell Cycle;Astrocyte Function;Apoptosis;Angiogenesis;Adaptive Immune Response |  |  | Psmb8 | slateblue1 | 16 | 22.7748691099476 |
  | Cdkn1a-mRNA | 2.23 | 0.15 | 1.93 | 2.52 | 4.68 | 3.82 | 5.74 | 4.31e-09 | 1.43e-06 | lm.nb | Adaptive Immune Response, Cell Cycle, Cellular Stress, Cytokine Signaling, DNA Damage, Growth Factor Signaling | NS\_MM\_NEUROINFLAM\_V1.0 | Cdkn1a | mRNA | 0 |  | Growth Factor Signaling;DNA Damage;Cytokine Signaling;Cellular Stress;Cell Cycle;Adaptive Immune Response | mmu04012;mmu04066;mmu04068;mmu04110;mmu04115;mmu04151;mmu04630;mmu04921;mmu05160;mmu05161;mmu05166;mmu05169;mmu05200;mmu05202;mmu05203;mmu05205;mmu05206;mmu05214;mmu05215;mmu05218;mmu05219;mmu05220 |  | Cdkn1a | slateblue1 | 16 | 14.8666666666667 |
  | Myc-mRNA | 1.37 | 0.14 | 1.1 | 1.65 | 2.59 | 2.14 | 3.13 | 4.35e-07 | 4.81e-05 | loglinear | Apoptosis, Cell Cycle, Cytokine Signaling, Growth Factor Signaling, Notch, Wnt | NS\_MM\_NEUROINFLAM\_V1.0 | Myc | mRNA | 0 |  | Wnt;Notch;Growth Factor Signaling;Cytokine Signaling;Cell Cycle;Apoptosis | mmu04010;mmu04012;mmu04110;mmu04151;mmu04310;mmu04350;mmu04390;mmu04550;mmu04630;mmu04919;mmu05161;mmu05166;mmu05169;mmu05200;mmu05202;mmu05205;mmu05206;mmu05210;mmu05213;mmu05216;mmu05219;mmu05220;mmu05221;mmu05222;mmu05230 |  | Myc | slateblue1 | 16 | 9.78571428571428 |
  | Tubb3-mRNA | -0.448 | 0.0494 | -0.545 | -0.351 | 0.733 | 0.686 | 0.784 | 1.03e-06 | 8.78e-05 | loglinear | Adaptive Immune Response, Apoptosis, Cell Cycle, Neurons and Neurotransmission | NS\_MM\_NEUROINFLAM\_V1.0 | Tubb3 | mRNA | 0 |  | Neurons and Neurotransmission;Cell Cycle;Apoptosis;Adaptive Immune Response | mmu04145;mmu04540 |  | Tubb3 | slateblue1 | 16 | -9.06882591093117 |
  | Gadd45g-mRNA | 1.65 | 0.209 | 1.24 | 2.06 | 3.15 | 2.37 | 4.18 | 4.12e-06 | 0.000261 | lm.nb | Cell Cycle, DNA Damage, Growth Factor Signaling | NS\_MM\_NEUROINFLAM\_V1.0 | Gadd45g | mRNA | 0 |  | Growth Factor Signaling;DNA Damage;Cell Cycle | mmu04010;mmu04068;mmu04110;mmu04115 |  | Gadd45g | slateblue1 | 16 | 7.89473684210526 |
  | Timeless-mRNA | 1.1 | 0.194 | 0.724 | 1.48 | 2.15 | 1.65 | 2.8 | 9.89e-05 | 0.00366 | loglinear | Cell Cycle, DNA Damage | NS\_MM\_NEUROINFLAM\_V1.0 | Timeless | mRNA | 0 |  | DNA Damage;Cell Cycle |  |  | Timeless | slateblue1 | 16 | 5.67010309278351 |
  | Prkar2b-mRNA | -0.52 | 0.0919 | -0.7 | -0.34 | 0.697 | 0.615 | 0.79 | 0.000105 | 0.00382 | loglinear | Apoptosis, Cell Cycle, Growth Factor Signaling | NS\_MM\_NEUROINFLAM\_V1.0 | Prkar2b | mRNA | 0 |  | Growth Factor Signaling;Cell Cycle;Apoptosis | mmu04210;mmu04910 |  | Prkar2b | slateblue1 | 16 | -5.65832426550599 |
  | Sumo1-mRNA | -0.196 | 0.0361 | -0.267 | -0.126 | 0.873 | 0.831 | 0.917 | 0.00015 | 0.00512 | loglinear | Cell Cycle, Cytokine Signaling, DNA Damage, Inflammatory Signaling | NS\_MM\_NEUROINFLAM\_V1.0 | Sumo1 | mRNA | 0 |  | Inflammatory Signaling;DNA Damage;Cytokine Signaling;Cell Cycle |  |  | Sumo1 | slateblue1 | 16 | -5.42936288088643 |
  | Tubb4a-mRNA | -0.523 | 0.098 | -0.715 | -0.331 | 0.696 | 0.609 | 0.795 | 0.000177 | 0.00596 | loglinear | Adaptive Immune Response, Apoptosis, Cell Cycle, Neurons and Neurotransmission | NS\_MM\_NEUROINFLAM\_V1.0 | Tubb4a | mRNA | 0 |  | Neurons and Neurotransmission;Cell Cycle;Apoptosis;Adaptive Immune Response | mmu04145;mmu04540 |  | Tubb4a | slateblue1 | 16 | -5.33673469387755 |
  | Gadd45a-mRNA | 0.534 | 0.105 | 0.329 | 0.74 | 1.45 | 1.26 | 1.67 | 0.000264 | 0.00845 | loglinear | Cell Cycle, DNA Damage, Growth Factor Signaling | NS\_MM\_NEUROINFLAM\_V1.0 | Gadd45a | mRNA | 0 |  | Growth Factor Signaling;DNA Damage;Cell Cycle | mmu04010;mmu04068;mmu04110;mmu04115 |  | Gadd45a | slateblue1 | 16 | 5.08571428571429 |
  | Anapc15-mRNA | -0.268 | 0.0737 | -0.412 | -0.123 | 0.831 | 0.751 | 0.918 | 0.00341 | 0.0762 | loglinear | Cell Cycle, Cellular Stress | NS\_MM\_NEUROINFLAM\_V1.0 | Anapc15 | mRNA | 0 |  | Cellular Stress;Cell Cycle |  |  | Anapc15 | slateblue3 | 16 | -3.63636363636364 |
  | Hdac2-mRNA | -0.267 | 0.0775 | -0.419 | -0.115 | 0.831 | 0.748 | 0.923 | 0.00484 | 0.0986 | loglinear | Cell Cycle, Cellular Stress, Epigenetic Regulation, Growth Factor Signaling, Notch | NS\_MM\_NEUROINFLAM\_V1.0 | Hdac2 | mRNA | 0 |  | Notch;Growth Factor Signaling;Epigenetic Regulation;Cellular Stress;Cell Cycle | mmu04110;mmu04213;mmu04330;mmu04919;mmu05016;mmu05034;mmu05169;mmu05200;mmu05202;mmu05203;mmu05220 |  | Hdac2 | slateblue3 | 16 | -3.44516129032258 |
  | Fen1-mRNA | 0.496 | 0.146 | 0.21 | 0.782 | 1.41 | 1.16 | 1.72 | 0.0053 | 0.105 | loglinear | Cell Cycle, DNA Damage | NS\_MM\_NEUROINFLAM\_V1.0 | Fen1 | mRNA | 0 |  | DNA Damage;Cell Cycle | mmu03030;mmu03410;mmu03450 |  | Fen1 | slateblue4 | 16 | 3.3972602739726 |
  | Pttg1-mRNA | 0.168 | 0.0532 | 0.064 | 0.272 | 1.12 | 1.05 | 1.21 | 0.00816 | 0.154 | loglinear | Cell Cycle, DNA Damage | NS\_MM\_NEUROINFLAM\_V1.0 | Pttg1 | mRNA | 0 |  | DNA Damage;Cell Cycle | mmu04110;mmu04114;mmu05166 |  | Pttg1 | slateblue4 | 16 | 3.15789473684211 |
  | Cdkn1c-mRNA | 0.706 | 0.225 | 0.264 | 1.15 | 1.63 | 1.2 | 2.22 | 0.00865 | 0.161 | lm.nb | Cell Cycle, DNA Damage | NS\_MM\_NEUROINFLAM\_V1.0 | Cdkn1c | mRNA | 0 |  | DNA Damage;Cell Cycle | mmu04110 |  | Cdkn1c | slateblue4 | 16 | 3.13777777777778 |
  | Ccni-mRNA | -0.184 | 0.0602 | -0.302 | -0.0665 | 0.88 | 0.811 | 0.955 | 0.00979 | 0.179 | loglinear | Cell Cycle | NS\_MM\_NEUROINFLAM\_V1.0 | Ccni | mRNA | 0 |  | Cell Cycle |  |  | Ccni | slateblue4 | 16 | -3.05647840531561 |
  | Trp53-mRNA | 0.573 | 0.192 | 0.197 | 0.95 | 1.49 | 1.15 | 1.93 | 0.0114 | 0.198 | lm.nb | Apoptosis, Cell Cycle, Cellular Stress, DNA Damage, Growth Factor Signaling, Notch, Wnt | NS\_MM\_NEUROINFLAM\_V1.0 | Trp53 | mRNA | 0 |  | Wnt;Notch;Growth Factor Signaling;DNA Damage;Cellular Stress;Cell Cycle;Apoptosis | mmu04010;mmu04071;mmu04110;mmu04115;mmu04151;mmu04210;mmu04211;mmu04310;mmu04722;mmu04919;mmu05014;mmu05016;mmu05160;mmu05161;mmu05162;mmu05166;mmu05168;mmu05169;mmu05200;mmu05202;mmu05203;mmu05205;mmu05206;mmu05210;mmu05212;mmu05213;mmu05214;mmu05215;mmu05216;mmu05218;mmu05219;mmu05220;mmu05222;mmu05223;mmu05230 |  | Trp53 | slateblue4 | 16 | 2.984375 |
  | Ccng2-mRNA | -0.418 | 0.155 | -0.723 | -0.114 | 0.748 | 0.606 | 0.924 | 0.0196 | 0.314 | lm.nb | Cell Cycle, DNA Damage | NS\_MM\_NEUROINFLAM\_V1.0 | Ccng2 | mRNA | 0 |  | DNA Damage;Cell Cycle | mmu04068;mmu04115 |  | Ccng2 | slateblue4 | 16 | -2.69677419354839 |
  | Rad51-mRNA | -1.58 | 0.629 | -2.82 | -0.352 | 0.333 | 0.142 | 0.784 | 0.0269 | 0.409 | lm.nb | Cell Cycle, DNA Damage | NS\_MM\_NEUROINFLAM\_V1.0 | Rad51 | mRNA | 0 |  | DNA Damage;Cell Cycle | mmu03440;mmu03460;mmu05200;mmu05212 |  | Rad51 | slateblue4 | 16 | -2.51192368839428 |
  | Rad17-mRNA | -0.362 | 0.163 | -0.682 | -0.0414 | 0.778 | 0.623 | 0.972 | 0.047 | 0.619 | loglinear | Cell Cycle, DNA Damage | NS\_MM\_NEUROINFLAM\_V1.0 | Rad17 | mRNA | 0 |  | DNA Damage;Cell Cycle |  |  | Rad17 | azure4 | 1 | -2.22085889570552 |

  ##### DE Results - Cell Cycle genes - DPI: 3 vs.CTRL

  Table displaying each sample's global significance scores and directed global significance scores as defined in the heatmaps above. The global significance score is calculated as the square root of the mean squared t-statistic for the genes in a gene set, with t-statistics coming from the linear regression underlying our differential expression analysis. The directed global significance score is calculated as the square root of the mean signed squared t-statistic for the genes in a gene set, with t-statistics coming from the linear regression underlying our differential expression analysis.

DPI:
- DPI: differential expression in 1 vs. baseline of CTRL
- DPI: differential expression in 2 vs. baseline of CTRL
- DPI: differential expression in 3 vs. baseline of CTRL

- **Volcano Plot: DPI: 1 vs.CTRL  
  More Plot Information**

  ##### Volcano Plot: DPI: 1 vs.CTRL

  Volcano plot displaying each gene's -log10(p-value) and log2 fold change for the selected covariate. Highly statistically significant genes fall at the top of the plot, and highly differentially expressed genes fall to either side. Genes within the selected gene set are highlighted in orange. Horizontal lines indicate various False Discovery Rate (FDR) thresholds.
- **DE Results - Cellular Stress genes - DPI: 1 vs.CTRL  
  More Plot Information  Download CSV Data**

  | Probe Label | Log2 fold change | std error (log2) | Lower confidence limit (log2) | Upper confidence limit (log2) | Linear fold change | Lower confidence limit (linear) | Upper confidence limit (linear) | P-value | BY.p.value | method | Gene.sets | Codeset.Name | Probe.Label | Analyte.Type | Is.Control | Control.Type | Probe.Annotation | KEGG.Pathways | Cell.Type | Official.Gene.Name | volcanocol | volcanopch | tstats.all |
  | --- | --- | --- | --- | --- | --- | --- | --- | --- | --- | --- | --- | --- | --- | --- | --- | --- | --- | --- | --- | --- | --- | --- | --- |
  | Ets2-mRNA | 0.213 | 0.0545 | 0.106 | 0.319 | 1.16 | 1.08 | 1.25 | 0.00211 | 0.437 | loglinear | Cellular Stress, Growth Factor Signaling, Microglia Function | NS\_MM\_NEUROINFLAM\_V1.0 | Ets2 | mRNA | 0 |  | Microglia Function;Growth Factor Signaling;Cellular Stress | mmu04014;mmu05166 |  | Ets2 | slateblue4 | 16 | 3.90825688073395 |
  | Atg9a-mRNA | -0.287 | 0.08 | -0.444 | -0.13 | 0.82 | 0.735 | 0.914 | 0.00374 | 0.558 | loglinear | Autophagy, Cellular Stress | NS\_MM\_NEUROINFLAM\_V1.0 | Atg9a | mRNA | 0 |  | Cellular Stress;Autophagy |  |  | Atg9a | azure4 | 1 | -3.5875 |
  | Il1r1-mRNA | 0.625 | 0.181 | 0.27 | 0.98 | 1.54 | 1.21 | 1.97 | 0.00477 | 0.577 | lm.nb | Apoptosis, Cellular Stress, Cytokine Signaling, Growth Factor Signaling, Microglia Function, NF-kB | NS\_MM\_NEUROINFLAM\_V1.0 | Il1r1 | mRNA | 0 |  | NF-kB;Microglia Function;Growth Factor Signaling;Cytokine Signaling;Cellular Stress;Apoptosis | mmu04010;mmu04060;mmu04064;mmu04210;mmu04380;mmu04640;mmu04750;mmu05146;mmu05166 |  | Il1r1 | azure4 | 1 | 3.45303867403315 |
  | Jun-mRNA | 0.323 | 0.0979 | 0.131 | 0.515 | 1.25 | 1.09 | 1.43 | 0.0064 | 0.711 | loglinear | Adaptive Immune Response, Apoptosis, Cellular Stress, Growth Factor Signaling, Innate Immune Response, Notch, Wnt | NS\_MM\_NEUROINFLAM\_V1.0 | Jun | mRNA | 0 |  | Wnt;Notch;Innate Immune Response;Growth Factor Signaling;Cellular Stress;Apoptosis;Adaptive Immune Response | mmu04010;mmu04012;mmu04024;mmu04310;mmu04380;mmu04510;mmu04620;mmu04660;mmu04662;mmu04668;mmu04722;mmu04912;mmu04915;mmu04921;mmu04932;mmu04933;mmu05030;mmu05031;mmu05132;mmu05133;mmu05140;mmu05142;mmu05161;mmu05164;mmu05166;mmu05168;mmu05169;mmu05200;mmu05203;mmu05210;mmu05211;mmu05231;mmu05321;mmu05323 |  | Jun | azure4 | 1 | 3.29928498467824 |
  | Nqo1-mRNA | 0.576 | 0.178 | 0.227 | 0.924 | 1.49 | 1.17 | 1.9 | 0.0071 | 0.76 | lm.nb | Cellular Stress | NS\_MM\_NEUROINFLAM\_V1.0 | Nqo1 | mRNA | 0 |  | Cellular Stress |  |  | Nqo1 | azure4 | 1 | 3.23595505617978 |
  | Bag3-mRNA | 0.567 | 0.182 | 0.21 | 0.924 | 1.48 | 1.16 | 1.9 | 0.00895 | 0.813 | loglinear | Apoptosis, Cellular Stress | NS\_MM\_NEUROINFLAM\_V1.0 | Bag3 | mRNA | 0 |  | Cellular Stress;Apoptosis |  |  | Bag3 | azure4 | 1 | 3.11538461538461 |
  | Bnip3-mRNA | 0.118 | 0.0396 | 0.0406 | 0.196 | 1.09 | 1.03 | 1.15 | 0.0114 | 0.868 | loglinear | Apoptosis, Autophagy, Cellular Stress, Microglia Function | NS\_MM\_NEUROINFLAM\_V1.0 | Bnip3 | mRNA | 0 |  | Microglia Function;Cellular Stress;Autophagy;Apoptosis | mmu04068;mmu05134 |  | Bnip3 | azure4 | 1 | 2.97979797979798 |
  | Casp3-mRNA | 0.393 | 0.132 | 0.134 | 0.652 | 1.31 | 1.1 | 1.57 | 0.0115 | 0.868 | lm.nb | Apoptosis, Cellular Stress, DNA Damage, Growth Factor Signaling, Innate Immune Response, Matrix Remodeling | NS\_MM\_NEUROINFLAM\_V1.0 | Casp3 | mRNA | 0 |  | Matrix Remodeling;Innate Immune Response;Growth Factor Signaling;DNA Damage;Cellular Stress;Apoptosis | mmu04010;mmu04115;mmu04210;mmu04650;mmu04668;mmu04726;mmu04932;mmu04933;mmu05010;mmu05012;mmu05014;mmu05016;mmu05133;mmu05134;mmu05145;mmu05146;mmu05152;mmu05161;mmu05168;mmu05200;mmu05203;mmu05205;mmu05206;mmu05210;mmu05416 |  | Casp3 | azure4 | 1 | 2.97727272727273 |
  | Hif1a-mRNA | 0.21 | 0.0727 | 0.0677 | 0.353 | 1.16 | 1.05 | 1.28 | 0.0136 | 0.934 | loglinear | Autophagy, Cellular Stress, Microglia Function, Notch | NS\_MM\_NEUROINFLAM\_V1.0 | Hif1a | mRNA | 0 |  | Notch;Microglia Function;Cellular Stress;Autophagy | mmu04066;mmu04150;mmu04919;mmu05200;mmu05205;mmu05211;mmu05230;mmu05231 |  | Hif1a | azure4 | 1 | 2.88858321870701 |
  | Rpa1-mRNA | 0.133 | 0.0503 | 0.0349 | 0.232 | 1.1 | 1.02 | 1.17 | 0.021 | 1 | loglinear | Cell Cycle, Cellular Stress, DNA Damage | NS\_MM\_NEUROINFLAM\_V1.0 | Rpa1 | mRNA | 0 |  | DNA Damage;Cellular Stress;Cell Cycle | mmu03030;mmu03420;mmu03430;mmu03440;mmu03460 |  | Rpa1 | azure4 | 1 | 2.6441351888668 |
  | Txnrd1-mRNA | 0.0667 | 0.026 | 0.0158 | 0.118 | 1.05 | 1.01 | 1.08 | 0.0247 | 1 | loglinear | Cellular Stress | NS\_MM\_NEUROINFLAM\_V1.0 | Txnrd1 | mRNA | 0 |  | Cellular Stress |  |  | Txnrd1 | azure4 | 1 | 2.56538461538462 |
  | Il1a-mRNA | 2.22 | 0.937 | 0.385 | 4.06 | 4.67 | 1.31 | 16.7 | 0.0354 | 1 | lm.nb | Apoptosis, Cellular Stress, Cytokine Signaling, Growth Factor Signaling | NS\_MM\_NEUROINFLAM\_V1.0 | Il1a | mRNA | 0 |  | Growth Factor Signaling;Cytokine Signaling;Cellular Stress;Apoptosis | mmu04010;mmu04060;mmu04210;mmu04380;mmu04640;mmu04932;mmu04933;mmu04940;mmu05020;mmu05132;mmu05133;mmu05140;mmu05152;mmu05162;mmu05164;mmu05321;mmu05323;mmu05332 |  | Il1a | azure4 | 1 | 2.3692636072572 |
  | Bag4-mRNA | 0.147 | 0.0629 | 0.0237 | 0.27 | 1.11 | 1.02 | 1.21 | 0.0375 | 1 | loglinear | Apoptosis, Cellular Stress, Innate Immune Response | NS\_MM\_NEUROINFLAM\_V1.0 | Bag4 | mRNA | 0 |  | Innate Immune Response;Cellular Stress;Apoptosis | mmu04668 |  | Bag4 | azure4 | 1 | 2.33704292527822 |
  | Cdkn1a-mRNA | 0.349 | 0.153 | 0.0486 | 0.649 | 1.27 | 1.03 | 1.57 | 0.0419 | 1 | lm.nb | Adaptive Immune Response, Cell Cycle, Cellular Stress, Cytokine Signaling, DNA Damage, Growth Factor Signaling | NS\_MM\_NEUROINFLAM\_V1.0 | Cdkn1a | mRNA | 0 |  | Growth Factor Signaling;DNA Damage;Cytokine Signaling;Cellular Stress;Cell Cycle;Adaptive Immune Response | mmu04012;mmu04066;mmu04068;mmu04110;mmu04115;mmu04151;mmu04630;mmu04921;mmu05160;mmu05161;mmu05166;mmu05169;mmu05200;mmu05202;mmu05203;mmu05205;mmu05206;mmu05214;mmu05215;mmu05218;mmu05219;mmu05220 |  | Cdkn1a | azure4 | 1 | 2.28104575163399 |
  | Map1lc3a-mRNA | 0.212 | 0.0991 | 0.018 | 0.407 | 1.16 | 1.01 | 1.33 | 0.0534 | 1 | loglinear | Autophagy, Cellular Stress | NS\_MM\_NEUROINFLAM\_V1.0 | Map1lc3a | mRNA | 0 |  | Cellular Stress;Autophagy |  |  | Map1lc3a | azure4 | 1 | 2.13925327951564 |
  | Parp1-mRNA | 0.246 | 0.118 | 0.0149 | 0.477 | 1.19 | 1.01 | 1.39 | 0.0589 | 1 | loglinear | Apoptosis, Cellular Stress, DNA Damage, NF-kB | NS\_MM\_NEUROINFLAM\_V1.0 | Parp1 | mRNA | 0 |  | NF-kB;DNA Damage;Cellular Stress;Apoptosis | mmu03410;mmu04064 |  | Parp1 | azure4 | 1 | 2.08474576271186 |
  | Ezh2-mRNA | 0.268 | 0.138 | -0.0014 | 0.538 | 1.2 | 0.999 | 1.45 | 0.075 | 1 | loglinear | Cellular Stress, Epigenetic Regulation | NS\_MM\_NEUROINFLAM\_V1.0 | Ezh2 | mRNA | 0 |  | Epigenetic Regulation;Cellular Stress | mmu05206 |  | Ezh2 | azure4 | 1 | 1.94202898550725 |
  | Hira-mRNA | -0.127 | 0.0655 | -0.256 | 0.00127 | 0.916 | 0.838 | 1 | 0.0762 | 1 | loglinear | Cellular Stress, Epigenetic Regulation | NS\_MM\_NEUROINFLAM\_V1.0 | Hira | mRNA | 0 |  | Epigenetic Regulation;Cellular Stress |  |  | Hira | azure4 | 1 | -1.93893129770992 |
  | Ambra1-mRNA | -0.158 | 0.0832 | -0.321 | 0.00507 | 0.896 | 0.8 | 1 | 0.0818 | 1 | loglinear | Autophagy, Cellular Stress | NS\_MM\_NEUROINFLAM\_V1.0 | Ambra1 | mRNA | 0 |  | Cellular Stress;Autophagy |  |  | Ambra1 | azure4 | 1 | -1.89903846153846 |
  | Suz12-mRNA | 0.162 | 0.0894 | -0.013 | 0.337 | 1.12 | 0.991 | 1.26 | 0.0946 | 1 | loglinear | Cellular Stress, Epigenetic Regulation | NS\_MM\_NEUROINFLAM\_V1.0 | Suz12 | mRNA | 0 |  | Epigenetic Regulation;Cellular Stress |  |  | Suz12 | azure4 | 1 | 1.81208053691275 |

  ##### DE Results - Cellular Stress genes - DPI: 1 vs.CTRL

  Table displaying each sample's global significance scores and directed global significance scores as defined in the heatmaps above. The global significance score is calculated as the square root of the mean squared t-statistic for the genes in a gene set, with t-statistics coming from the linear regression underlying our differential expression analysis. The directed global significance score is calculated as the square root of the mean signed squared t-statistic for the genes in a gene set, with t-statistics coming from the linear regression underlying our differential expression analysis.

- **Volcano Plot: DPI: 2 vs.CTRL  
  More Plot Information**

  ##### Volcano Plot: DPI: 2 vs.CTRL

  Volcano plot displaying each gene's -log10(p-value) and log2 fold change for the selected covariate. Highly statistically significant genes fall at the top of the plot, and highly differentially expressed genes fall to either side. Genes within the selected gene set are highlighted in orange. Horizontal lines indicate various False Discovery Rate (FDR) thresholds.
- **DE Results - Cellular Stress genes - DPI: 2 vs.CTRL  
  More Plot Information  Download CSV Data**

  | Probe Label | Log2 fold change | std error (log2) | Lower confidence limit (log2) | Upper confidence limit (log2) | Linear fold change | Lower confidence limit (linear) | Upper confidence limit (linear) | P-value | BY.p.value | method | Gene.sets | Codeset.Name | Probe.Label | Analyte.Type | Is.Control | Control.Type | Probe.Annotation | KEGG.Pathways | Cell.Type | Official.Gene.Name | volcanocol | volcanopch | tstats.all |
  | --- | --- | --- | --- | --- | --- | --- | --- | --- | --- | --- | --- | --- | --- | --- | --- | --- | --- | --- | --- | --- | --- | --- | --- |
  | Cdkn1a-mRNA | 2.21 | 0.15 | 1.92 | 2.51 | 4.64 | 3.78 | 5.68 | 4.64e-09 | 3.71e-06 | lm.nb | Adaptive Immune Response, Cell Cycle, Cellular Stress, Cytokine Signaling, DNA Damage, Growth Factor Signaling | NS\_MM\_NEUROINFLAM\_V1.0 | Cdkn1a | mRNA | 0 |  | Growth Factor Signaling;DNA Damage;Cytokine Signaling;Cellular Stress;Cell Cycle;Adaptive Immune Response | mmu04012;mmu04066;mmu04068;mmu04110;mmu04115;mmu04151;mmu04630;mmu04921;mmu05160;mmu05161;mmu05166;mmu05169;mmu05200;mmu05202;mmu05203;mmu05205;mmu05206;mmu05214;mmu05215;mmu05218;mmu05219;mmu05220 |  | Cdkn1a | slateblue1 | 16 | 14.7333333333333 |
  | Ets2-mRNA | 0.499 | 0.0545 | 0.392 | 0.606 | 1.41 | 1.31 | 1.52 | 9.29e-07 | 0.000218 | loglinear | Cellular Stress, Growth Factor Signaling, Microglia Function | NS\_MM\_NEUROINFLAM\_V1.0 | Ets2 | mRNA | 0 |  | Microglia Function;Growth Factor Signaling;Cellular Stress | mmu04014;mmu05166 |  | Ets2 | slateblue1 | 16 | 9.15596330275229 |
  | Bnip3-mRNA | 0.317 | 0.0396 | 0.239 | 0.395 | 1.25 | 1.18 | 1.31 | 3.73e-06 | 0.000532 | loglinear | Apoptosis, Autophagy, Cellular Stress, Microglia Function | NS\_MM\_NEUROINFLAM\_V1.0 | Bnip3 | mRNA | 0 |  | Microglia Function;Cellular Stress;Autophagy;Apoptosis | mmu04068;mmu05134 |  | Bnip3 | slateblue1 | 16 | 8.00505050505051 |
  | Il1r1-mRNA | 1.09 | 0.175 | 0.752 | 1.44 | 2.14 | 1.68 | 2.71 | 4.23e-05 | 0.00403 | lm.nb | Apoptosis, Cellular Stress, Cytokine Signaling, Growth Factor Signaling, Microglia Function, NF-kB | NS\_MM\_NEUROINFLAM\_V1.0 | Il1r1 | mRNA | 0 |  | NF-kB;Microglia Function;Growth Factor Signaling;Cytokine Signaling;Cellular Stress;Apoptosis | mmu04010;mmu04060;mmu04064;mmu04210;mmu04380;mmu04640;mmu04750;mmu05146;mmu05166 |  | Il1r1 | slateblue1 | 16 | 6.22857142857143 |
  | Snca-mRNA | -0.215 | 0.0364 | -0.286 | -0.144 | 0.862 | 0.82 | 0.905 | 7.2e-05 | 0.00586 | loglinear | Cellular Stress, Microglia Function | NS\_MM\_NEUROINFLAM\_V1.0 | Snca | mRNA | 0 |  | Microglia Function;Cellular Stress | mmu05010;mmu05012 |  | Snca | slateblue1 | 16 | -5.90659340659341 |
  | Ptgs2-mRNA | 1.09 | 0.194 | 0.713 | 1.47 | 2.13 | 1.64 | 2.78 | 0.00011 | 0.00772 | lm.nb | Astrocyte Function, Cellular Stress, Innate Immune Response, Lipid Metabolism, Microglia Function, NF-kB | NS\_MM\_NEUROINFLAM\_V1.0 | Ptgs2 | mRNA | 0 |  | NF-kB;Microglia Function;Lipid Metabolism;Innate Immune Response;Cellular Stress;Astrocyte Function | mmu00590;mmu04064;mmu04370;mmu04668;mmu04723;mmu04726;mmu04913;mmu04921;mmu04923;mmu05140;mmu05200;mmu05206;mmu05222 |  | Ptgs2 | slateblue1 | 16 | 5.61855670103093 |
  | Anapc15-mRNA | -0.364 | 0.0737 | -0.508 | -0.22 | 0.777 | 0.703 | 0.859 | 0.000341 | 0.02 | loglinear | Cell Cycle, Cellular Stress | NS\_MM\_NEUROINFLAM\_V1.0 | Anapc15 | mRNA | 0 |  | Cellular Stress;Cell Cycle |  |  | Anapc15 | slateblue2 | 16 | -4.93894165535957 |
  | Il1a-mRNA | 4.25 | 0.922 | 2.44 | 6.06 | 19 | 5.43 | 66.5 | 0.000605 | 0.0291 | lm.nb | Apoptosis, Cellular Stress, Cytokine Signaling, Growth Factor Signaling | NS\_MM\_NEUROINFLAM\_V1.0 | Il1a | mRNA | 0 |  | Growth Factor Signaling;Cytokine Signaling;Cellular Stress;Apoptosis | mmu04010;mmu04060;mmu04210;mmu04380;mmu04640;mmu04932;mmu04933;mmu04940;mmu05020;mmu05132;mmu05133;mmu05140;mmu05152;mmu05162;mmu05164;mmu05321;mmu05323;mmu05332 |  | Il1a | slateblue2 | 16 | 4.60954446854664 |
  | Apoe-mRNA | 0.313 | 0.0758 | 0.165 | 0.462 | 1.24 | 1.12 | 1.38 | 0.00139 | 0.0545 | loglinear | Astrocyte Function, Cellular Stress, Lipid Metabolism, Microglia Function, Neurons and Neurotransmission | NS\_MM\_NEUROINFLAM\_V1.0 | Apoe | mRNA | 0 |  | Neurons and Neurotransmission;Microglia Function;Lipid Metabolism;Cellular Stress;Astrocyte Function | mmu05010 |  | Apoe | slateblue3 | 16 | 4.12928759894459 |
  | Hspb1-mRNA | 0.891 | 0.232 | 0.435 | 1.35 | 1.85 | 1.35 | 2.54 | 0.00238 | 0.082 | lm.nb | Angiogenesis, Astrocyte Function, Cellular Stress, Growth Factor Signaling | NS\_MM\_NEUROINFLAM\_V1.0 | Hspb1 | mRNA | 0 |  | Growth Factor Signaling;Cellular Stress;Astrocyte Function;Angiogenesis | mmu04010;mmu04370;mmu05146;mmu05169 |  | Hspb1 | slateblue3 | 16 | 3.84051724137931 |
  | Mapt-mRNA | -0.249 | 0.0702 | -0.387 | -0.112 | 0.841 | 0.765 | 0.926 | 0.00401 | 0.121 | loglinear | Apoptosis, Cellular Stress, Growth Factor Signaling, Neurons and Neurotransmission | NS\_MM\_NEUROINFLAM\_V1.0 | Mapt | mRNA | 0 |  | Neurons and Neurotransmission;Growth Factor Signaling;Cellular Stress;Apoptosis | mmu04010;mmu05010 |  | Mapt | slateblue4 | 16 | -3.54700854700855 |
  | Map1lc3a-mRNA | -0.322 | 0.0991 | -0.516 | -0.128 | 0.8 | 0.699 | 0.915 | 0.00698 | 0.187 | loglinear | Autophagy, Cellular Stress | NS\_MM\_NEUROINFLAM\_V1.0 | Map1lc3a | mRNA | 0 |  | Cellular Stress;Autophagy |  |  | Map1lc3a | slateblue4 | 16 | -3.24924318869828 |
  | Ulk1-mRNA | 0.268 | 0.0847 | 0.101 | 0.434 | 1.2 | 1.07 | 1.35 | 0.00826 | 0.217 | loglinear | Autophagy, Cellular Stress | NS\_MM\_NEUROINFLAM\_V1.0 | Ulk1 | mRNA | 0 |  | Cellular Stress;Autophagy | mmu04140;mmu04150;mmu04152;mmu04211 |  | Ulk1 | slateblue4 | 16 | 3.16410861865407 |
  | Hdac2-mRNA | -0.239 | 0.0775 | -0.391 | -0.0871 | 0.847 | 0.763 | 0.941 | 0.00946 | 0.24 | loglinear | Cell Cycle, Cellular Stress, Epigenetic Regulation, Growth Factor Signaling, Notch | NS\_MM\_NEUROINFLAM\_V1.0 | Hdac2 | mRNA | 0 |  | Notch;Growth Factor Signaling;Epigenetic Regulation;Cellular Stress;Cell Cycle | mmu04110;mmu04213;mmu04330;mmu04919;mmu05016;mmu05034;mmu05169;mmu05200;mmu05202;mmu05203;mmu05220 |  | Hdac2 | slateblue4 | 16 | -3.08387096774194 |
  | Jun-mRNA | 0.301 | 0.0979 | 0.11 | 0.493 | 1.23 | 1.08 | 1.41 | 0.00956 | 0.24 | loglinear | Adaptive Immune Response, Apoptosis, Cellular Stress, Growth Factor Signaling, Innate Immune Response, Notch, Wnt | NS\_MM\_NEUROINFLAM\_V1.0 | Jun | mRNA | 0 |  | Wnt;Notch;Innate Immune Response;Growth Factor Signaling;Cellular Stress;Apoptosis;Adaptive Immune Response | mmu04010;mmu04012;mmu04024;mmu04310;mmu04380;mmu04510;mmu04620;mmu04660;mmu04662;mmu04668;mmu04722;mmu04912;mmu04915;mmu04921;mmu04932;mmu04933;mmu05030;mmu05031;mmu05132;mmu05133;mmu05140;mmu05142;mmu05161;mmu05164;mmu05166;mmu05168;mmu05169;mmu05200;mmu05203;mmu05210;mmu05211;mmu05231;mmu05321;mmu05323 |  | Jun | slateblue4 | 16 | 3.07456588355465 |
  | Hira-mRNA | -0.195 | 0.0655 | -0.323 | -0.0662 | 0.874 | 0.799 | 0.955 | 0.0117 | 0.284 | loglinear | Cellular Stress, Epigenetic Regulation | NS\_MM\_NEUROINFLAM\_V1.0 | Hira | mRNA | 0 |  | Epigenetic Regulation;Cellular Stress |  |  | Hira | slateblue4 | 16 | -2.97709923664122 |
  | Fas-mRNA | 1.3 | 0.457 | 0.407 | 2.2 | 2.47 | 1.33 | 4.6 | 0.0146 | 0.342 | lm.nb | Apoptosis, Cellular Stress, Cytokine Signaling, DNA Damage, Growth Factor Signaling, Innate Immune Response | NS\_MM\_NEUROINFLAM\_V1.0 | Fas | mRNA | 0 |  | Innate Immune Response;Growth Factor Signaling;DNA Damage;Cytokine Signaling;Cellular Stress;Apoptosis | mmu04010;mmu04060;mmu04115;mmu04210;mmu04650;mmu04668;mmu04932;mmu04940;mmu05010;mmu05142;mmu05143;mmu05161;mmu05162;mmu05164;mmu05168;mmu05200;mmu05205;mmu05320;mmu05330;mmu05332 |  | Fas | slateblue4 | 16 | 2.84463894967177 |
  | Vegfa-mRNA | -0.325 | 0.124 | -0.569 | -0.0809 | 0.798 | 0.674 | 0.945 | 0.0228 | 0.48 | loglinear | Angiogenesis, Cellular Stress, Cytokine Signaling, Growth Factor Signaling, Microglia Function | NS\_MM\_NEUROINFLAM\_V1.0 | Vegfa | mRNA | 0 |  | Microglia Function;Growth Factor Signaling;Cytokine Signaling;Cellular Stress;Angiogenesis | mmu04014;mmu04015;mmu04060;mmu04066;mmu04150;mmu04151;mmu04370;mmu04510;mmu04933;mmu05200;mmu05205;mmu05206;mmu05211;mmu05212;mmu05219;mmu05323 |  | Vegfa | slateblue4 | 16 | -2.62096774193548 |
  | Parp1-mRNA | 0.3 | 0.118 | 0.0689 | 0.531 | 1.23 | 1.05 | 1.45 | 0.0257 | 0.511 | loglinear | Apoptosis, Cellular Stress, DNA Damage, NF-kB | NS\_MM\_NEUROINFLAM\_V1.0 | Parp1 | mRNA | 0 |  | NF-kB;DNA Damage;Cellular Stress;Apoptosis | mmu03410;mmu04064 |  | Parp1 | azure4 | 1 | 2.54237288135593 |
  | Map2k4-mRNA | -0.164 | 0.0645 | -0.291 | -0.0378 | 0.892 | 0.818 | 0.974 | 0.0257 | 0.511 | loglinear | Adaptive Immune Response, Apoptosis, Cellular Stress, Growth Factor Signaling, Innate Immune Response | NS\_MM\_NEUROINFLAM\_V1.0 | Map2k4 | mRNA | 0 |  | Innate Immune Response;Growth Factor Signaling;Cellular Stress;Apoptosis;Adaptive Immune Response | mmu04010;mmu04012;mmu04620;mmu04664;mmu04668;mmu04912;mmu05142;mmu05161;mmu05164;mmu05166;mmu05169 |  | Map2k4 | azure4 | 1 | -2.54263565891473 |

  ##### DE Results - Cellular Stress genes - DPI: 2 vs.CTRL

  Table displaying each sample's global significance scores and directed global significance scores as defined in the heatmaps above. The global significance score is calculated as the square root of the mean squared t-statistic for the genes in a gene set, with t-statistics coming from the linear regression underlying our differential expression analysis. The directed global significance score is calculated as the square root of the mean signed squared t-statistic for the genes in a gene set, with t-statistics coming from the linear regression underlying our differential expression analysis.

- **Volcano Plot: DPI: 3 vs.CTRL  
  More Plot Information**

  ##### Volcano Plot: DPI: 3 vs.CTRL

  Volcano plot displaying each gene's -log10(p-value) and log2 fold change for the selected covariate. Highly statistically significant genes fall at the top of the plot, and highly differentially expressed genes fall to either side. Genes within the selected gene set are highlighted in orange. Horizontal lines indicate various False Discovery Rate (FDR) thresholds.
- **DE Results - Cellular Stress genes - DPI: 3 vs.CTRL  
  More Plot Information  Download CSV Data**

  | Probe Label | Log2 fold change | std error (log2) | Lower confidence limit (log2) | Upper confidence limit (log2) | Linear fold change | Lower confidence limit (linear) | Upper confidence limit (linear) | P-value | BY.p.value | method | Gene.sets | Codeset.Name | Probe.Label | Analyte.Type | Is.Control | Control.Type | Probe.Annotation | KEGG.Pathways | Cell.Type | Official.Gene.Name | volcanocol | volcanopch | tstats.all |
  | --- | --- | --- | --- | --- | --- | --- | --- | --- | --- | --- | --- | --- | --- | --- | --- | --- | --- | --- | --- | --- | --- | --- | --- |
  | Cdkn1a-mRNA | 2.23 | 0.15 | 1.93 | 2.52 | 4.68 | 3.82 | 5.74 | 4.31e-09 | 1.43e-06 | lm.nb | Adaptive Immune Response, Cell Cycle, Cellular Stress, Cytokine Signaling, DNA Damage, Growth Factor Signaling | NS\_MM\_NEUROINFLAM\_V1.0 | Cdkn1a | mRNA | 0 |  | Growth Factor Signaling;DNA Damage;Cytokine Signaling;Cellular Stress;Cell Cycle;Adaptive Immune Response | mmu04012;mmu04066;mmu04068;mmu04110;mmu04115;mmu04151;mmu04630;mmu04921;mmu05160;mmu05161;mmu05166;mmu05169;mmu05200;mmu05202;mmu05203;mmu05205;mmu05206;mmu05214;mmu05215;mmu05218;mmu05219;mmu05220 |  | Cdkn1a | slateblue1 | 16 | 14.8666666666667 |
  | Ets2-mRNA | 0.703 | 0.0545 | 0.596 | 0.81 | 1.63 | 1.51 | 1.75 | 2.16e-08 | 4.54e-06 | loglinear | Cellular Stress, Growth Factor Signaling, Microglia Function | NS\_MM\_NEUROINFLAM\_V1.0 | Ets2 | mRNA | 0 |  | Microglia Function;Growth Factor Signaling;Cellular Stress | mmu04014;mmu05166 |  | Ets2 | slateblue1 | 16 | 12.8990825688073 |
  | Hspb1-mRNA | 2.5 | 0.227 | 2.05 | 2.95 | 5.66 | 4.15 | 7.7 | 1.27e-07 | 1.76e-05 | lm.nb | Angiogenesis, Astrocyte Function, Cellular Stress, Growth Factor Signaling | NS\_MM\_NEUROINFLAM\_V1.0 | Hspb1 | mRNA | 0 |  | Growth Factor Signaling;Cellular Stress;Astrocyte Function;Angiogenesis | mmu04010;mmu04370;mmu05146;mmu05169 |  | Hspb1 | slateblue1 | 16 | 11.0132158590308 |
  | Ptgs2-mRNA | 1.88 | 0.191 | 1.5 | 2.25 | 3.67 | 2.83 | 4.76 | 4.46e-07 | 4.81e-05 | lm.nb | Astrocyte Function, Cellular Stress, Innate Immune Response, Lipid Metabolism, Microglia Function, NF-kB | NS\_MM\_NEUROINFLAM\_V1.0 | Ptgs2 | mRNA | 0 |  | NF-kB;Microglia Function;Lipid Metabolism;Innate Immune Response;Cellular Stress;Astrocyte Function | mmu00590;mmu04064;mmu04370;mmu04668;mmu04723;mmu04726;mmu04913;mmu04921;mmu04923;mmu05140;mmu05200;mmu05206;mmu05222 |  | Ptgs2 | slateblue1 | 16 | 9.84293193717278 |
  | Il1r1-mRNA | 1.31 | 0.173 | 0.968 | 1.65 | 2.47 | 1.96 | 3.13 | 6.67e-06 | 0.00038 | lm.nb | Apoptosis, Cellular Stress, Cytokine Signaling, Growth Factor Signaling, Microglia Function, NF-kB | NS\_MM\_NEUROINFLAM\_V1.0 | Il1r1 | mRNA | 0 |  | NF-kB;Microglia Function;Growth Factor Signaling;Cytokine Signaling;Cellular Stress;Apoptosis | mmu04010;mmu04060;mmu04064;mmu04210;mmu04380;mmu04640;mmu04750;mmu05146;mmu05166 |  | Il1r1 | slateblue1 | 16 | 7.57225433526012 |
  | Snca-mRNA | -0.274 | 0.0364 | -0.345 | -0.203 | 0.827 | 0.787 | 0.869 | 6.97e-06 | 0.000392 | loglinear | Cellular Stress, Microglia Function | NS\_MM\_NEUROINFLAM\_V1.0 | Snca | mRNA | 0 |  | Microglia Function;Cellular Stress | mmu05010;mmu05012 |  | Snca | slateblue1 | 16 | -7.52747252747253 |
  | Fas-mRNA | 3.25 | 0.436 | 2.39 | 4.1 | 9.5 | 5.26 | 17.2 | 7.68e-06 | 0.00042 | lm.nb | Apoptosis, Cellular Stress, Cytokine Signaling, DNA Damage, Growth Factor Signaling, Innate Immune Response | NS\_MM\_NEUROINFLAM\_V1.0 | Fas | mRNA | 0 |  | Innate Immune Response;Growth Factor Signaling;DNA Damage;Cytokine Signaling;Cellular Stress;Apoptosis | mmu04010;mmu04060;mmu04115;mmu04210;mmu04650;mmu04668;mmu04932;mmu04940;mmu05010;mmu05142;mmu05143;mmu05161;mmu05162;mmu05164;mmu05168;mmu05200;mmu05205;mmu05320;mmu05330;mmu05332 |  | Fas | slateblue1 | 16 | 7.45412844036697 |
  | Hmox1-mRNA | 3 | 0.425 | 2.17 | 3.83 | 8 | 4.49 | 14.2 | 1.31e-05 | 0.000679 | lm.nb | Cellular Stress | NS\_MM\_NEUROINFLAM\_V1.0 | Hmox1 | mRNA | 0 |  | Cellular Stress | mmu04066 |  | Hmox1 | slateblue1 | 16 | 7.05882352941176 |
  | Il1a-mRNA | 6.35 | 0.919 | 4.55 | 8.15 | 81.3 | 23.3 | 283 | 1.63e-05 | 0.000805 | lm.nb | Apoptosis, Cellular Stress, Cytokine Signaling, Growth Factor Signaling | NS\_MM\_NEUROINFLAM\_V1.0 | Il1a | mRNA | 0 |  | Growth Factor Signaling;Cytokine Signaling;Cellular Stress;Apoptosis | mmu04010;mmu04060;mmu04210;mmu04380;mmu04640;mmu04932;mmu04933;mmu04940;mmu05020;mmu05132;mmu05133;mmu05140;mmu05152;mmu05162;mmu05164;mmu05321;mmu05323;mmu05332 |  | Il1a | slateblue1 | 16 | 6.90968443960827 |
  | Bnip3-mRNA | 0.236 | 0.0396 | 0.159 | 0.314 | 1.18 | 1.12 | 1.24 | 6.56e-05 | 0.00271 | loglinear | Apoptosis, Autophagy, Cellular Stress, Microglia Function | NS\_MM\_NEUROINFLAM\_V1.0 | Bnip3 | mRNA | 0 |  | Microglia Function;Cellular Stress;Autophagy;Apoptosis | mmu04068;mmu05134 |  | Bnip3 | slateblue1 | 16 | 5.95959595959596 |
  | Jun-mRNA | 0.495 | 0.0979 | 0.303 | 0.687 | 1.41 | 1.23 | 1.61 | 0.000281 | 0.00876 | loglinear | Adaptive Immune Response, Apoptosis, Cellular Stress, Growth Factor Signaling, Innate Immune Response, Notch, Wnt | NS\_MM\_NEUROINFLAM\_V1.0 | Jun | mRNA | 0 |  | Wnt;Notch;Innate Immune Response;Growth Factor Signaling;Cellular Stress;Apoptosis;Adaptive Immune Response | mmu04010;mmu04012;mmu04024;mmu04310;mmu04380;mmu04510;mmu04620;mmu04660;mmu04662;mmu04668;mmu04722;mmu04912;mmu04915;mmu04921;mmu04932;mmu04933;mmu05030;mmu05031;mmu05132;mmu05133;mmu05140;mmu05142;mmu05161;mmu05164;mmu05166;mmu05168;mmu05169;mmu05200;mmu05203;mmu05210;mmu05211;mmu05231;mmu05321;mmu05323 |  | Jun | slateblue1 | 16 | 5.0561797752809 |
  | Bag3-mRNA | 0.888 | 0.182 | 0.531 | 1.25 | 1.85 | 1.45 | 2.37 | 0.000381 | 0.0114 | loglinear | Apoptosis, Cellular Stress | NS\_MM\_NEUROINFLAM\_V1.0 | Bag3 | mRNA | 0 |  | Cellular Stress;Apoptosis |  |  | Bag3 | slateblue2 | 16 | 4.87912087912088 |
  | Ncf1-mRNA | 3.09 | 0.668 | 1.78 | 4.4 | 8.52 | 3.44 | 21.1 | 0.000586 | 0.0167 | lm.nb | Adaptive Immune Response, Angiogenesis, Cellular Stress | NS\_MM\_NEUROINFLAM\_V1.0 | Ncf1 | mRNA | 0 |  | Cellular Stress;Angiogenesis;Adaptive Immune Response | mmu04062;mmu04145;mmu04380;mmu04666;mmu04670;mmu05140 | Microglia | Ncf1 | slateblue2 | 16 | 4.62574850299401 |
  | Anapc15-mRNA | -0.268 | 0.0737 | -0.412 | -0.123 | 0.831 | 0.751 | 0.918 | 0.00341 | 0.0762 | loglinear | Cell Cycle, Cellular Stress | NS\_MM\_NEUROINFLAM\_V1.0 | Anapc15 | mRNA | 0 |  | Cellular Stress;Cell Cycle |  |  | Anapc15 | slateblue3 | 16 | -3.63636363636364 |
  | Sod2-mRNA | 0.171 | 0.0482 | 0.0761 | 0.265 | 1.13 | 1.05 | 1.2 | 0.00407 | 0.0864 | loglinear | Cellular Stress, Microglia Function | NS\_MM\_NEUROINFLAM\_V1.0 | Sod2 | mRNA | 0 |  | Microglia Function;Cellular Stress | mmu04068;mmu04211;mmu04213;mmu05016 |  | Sod2 | slateblue3 | 16 | 3.54771784232365 |
  | Hira-mRNA | -0.23 | 0.0655 | -0.359 | -0.102 | 0.853 | 0.78 | 0.932 | 0.00429 | 0.0896 | loglinear | Cellular Stress, Epigenetic Regulation | NS\_MM\_NEUROINFLAM\_V1.0 | Hira | mRNA | 0 |  | Epigenetic Regulation;Cellular Stress |  |  | Hira | slateblue3 | 16 | -3.51145038167939 |
  | Mapk10-mRNA | -0.152 | 0.0439 | -0.238 | -0.066 | 0.9 | 0.848 | 0.955 | 0.00469 | 0.0961 | loglinear | Adaptive Immune Response, Apoptosis, Autophagy, Cellular Stress, Growth Factor Signaling, Innate Immune Response, Wnt | NS\_MM\_NEUROINFLAM\_V1.0 | Mapk10 | mRNA | 0 |  | Wnt;Innate Immune Response;Growth Factor Signaling;Cellular Stress;Autophagy;Apoptosis;Adaptive Immune Response | mmu04010;mmu04012;mmu04014;mmu04024;mmu04068;mmu04071;mmu04141;mmu04310;mmu04380;mmu04510;mmu04620;mmu04621;mmu04622;mmu04664;mmu04668;mmu04722;mmu04723;mmu04728;mmu04750;mmu04910;mmu04912;mmu04914;mmu04917;mmu04920;mmu04930;mmu04931;mmu04932;mmu04933;mmu05132;mmu05133;mmu05142;mmu05145;mmu05152;mmu05160;mmu05161;mmu05164;mmu05168;mmu05169;mmu05200;mmu05210;mmu05212;mmu05231 |  | Mapk10 | slateblue3 | 16 | -3.4624145785877 |
  | Hdac2-mRNA | -0.267 | 0.0775 | -0.419 | -0.115 | 0.831 | 0.748 | 0.923 | 0.00484 | 0.0986 | loglinear | Cell Cycle, Cellular Stress, Epigenetic Regulation, Growth Factor Signaling, Notch | NS\_MM\_NEUROINFLAM\_V1.0 | Hdac2 | mRNA | 0 |  | Notch;Growth Factor Signaling;Epigenetic Regulation;Cellular Stress;Cell Cycle | mmu04110;mmu04213;mmu04330;mmu04919;mmu05016;mmu05034;mmu05169;mmu05200;mmu05202;mmu05203;mmu05220 |  | Hdac2 | slateblue3 | 16 | -3.44516129032258 |
  | Txnrd1-mRNA | 0.0803 | 0.026 | 0.0293 | 0.131 | 1.06 | 1.02 | 1.1 | 0.00938 | 0.174 | loglinear | Cellular Stress | NS\_MM\_NEUROINFLAM\_V1.0 | Txnrd1 | mRNA | 0 |  | Cellular Stress |  |  | Txnrd1 | slateblue4 | 16 | 3.08846153846154 |
  | Rela-mRNA | 0.444 | 0.144 | 0.161 | 0.727 | 1.36 | 1.12 | 1.65 | 0.00964 | 0.177 | lm.nb | Adaptive Immune Response, Apoptosis, Cellular Stress, Cytokine Signaling, Growth Factor Signaling, Inflammatory Signaling, Innate Immune Response, NF-kB | NS\_MM\_NEUROINFLAM\_V1.0 | Rela | mRNA | 0 |  | NF-kB;Innate Immune Response;Inflammatory Signaling;Growth Factor Signaling;Cytokine Signaling;Cellular Stress;Apoptosis;Adaptive Immune Response | mmu04010;mmu04014;mmu04024;mmu04062;mmu04064;mmu04066;mmu04071;mmu04151;mmu04210;mmu04211;mmu04380;mmu04620;mmu04621;mmu04622;mmu04623;mmu04660;mmu04662;mmu04668;mmu04722;mmu04917;mmu04920;mmu04931;mmu04932;mmu04933;mmu05030;mmu05132;mmu05133;mmu05134;mmu05140;mmu05142;mmu05145;mmu05146;mmu05152;mmu05160;mmu05161;mmu05162;mmu05164;mmu05166;mmu05168;mmu05169;mmu05200;mmu05202;mmu05203;mmu05212;mmu05215;mmu05220;mmu05221;mmu05222;mmu05321 |  | Rela | slateblue4 | 16 | 3.08333333333333 |

  ##### DE Results - Cellular Stress genes - DPI: 3 vs.CTRL

  Table displaying each sample's global significance scores and directed global significance scores as defined in the heatmaps above. The global significance score is calculated as the square root of the mean squared t-statistic for the genes in a gene set, with t-statistics coming from the linear regression underlying our differential expression analysis. The directed global significance score is calculated as the square root of the mean signed squared t-statistic for the genes in a gene set, with t-statistics coming from the linear regression underlying our differential expression analysis.

DPI:
- DPI: differential expression in 1 vs. baseline of CTRL
- DPI: differential expression in 2 vs. baseline of CTRL
- DPI: differential expression in 3 vs. baseline of CTRL

- **Volcano Plot: DPI: 1 vs.CTRL  
  More Plot Information**

  ##### Volcano Plot: DPI: 1 vs.CTRL

  Volcano plot displaying each gene's -log10(p-value) and log2 fold change for the selected covariate. Highly statistically significant genes fall at the top of the plot, and highly differentially expressed genes fall to either side. Genes within the selected gene set are highlighted in orange. Horizontal lines indicate various False Discovery Rate (FDR) thresholds.
- **DE Results - Cytokine Signaling genes - DPI: 1 vs.CTRL  
  More Plot Information  Download CSV Data**

  | Probe Label | Log2 fold change | std error (log2) | Lower confidence limit (log2) | Upper confidence limit (log2) | Linear fold change | Lower confidence limit (linear) | Upper confidence limit (linear) | P-value | BY.p.value | method | Gene.sets | Codeset.Name | Probe.Label | Analyte.Type | Is.Control | Control.Type | Probe.Annotation | KEGG.Pathways | Cell.Type | Official.Gene.Name | volcanocol | volcanopch | tstats.all |
  | --- | --- | --- | --- | --- | --- | --- | --- | --- | --- | --- | --- | --- | --- | --- | --- | --- | --- | --- | --- | --- | --- | --- | --- |
  | Osmr-mRNA | 1.68 | 0.337 | 1.02 | 2.34 | 3.21 | 2.03 | 5.07 | 0.000313 | 0.125 | lm.nb | Astrocyte Function, Cytokine Signaling, Growth Factor Signaling | NS\_MM\_NEUROINFLAM\_V1.0 | Osmr | mRNA | 0 |  | Growth Factor Signaling;Cytokine Signaling;Astrocyte Function | mmu04060;mmu04151;mmu04630 |  | Osmr | slateblue4 | 16 | 4.98516320474777 |
  | Ccl2-mRNA | 4.15 | 0.972 | 2.24 | 6.05 | 17.8 | 4.74 | 66.5 | 0.00109 | 0.335 | lm.nb | Astrocyte Function, Cytokine Signaling, Inflammatory Signaling, Innate Immune Response | NS\_MM\_NEUROINFLAM\_V1.0 | Ccl2 | mRNA | 0 |  | Innate Immune Response;Inflammatory Signaling;Cytokine Signaling;Astrocyte Function | mmu04060;mmu04062;mmu04621;mmu04668;mmu04933;mmu05142;mmu05144;mmu05164;mmu05168;mmu05323 | DC | Ccl2 | slateblue4 | 16 | 4.26954732510288 |
  | Cx3cl1-mRNA | -0.475 | 0.126 | -0.723 | -0.228 | 0.719 | 0.606 | 0.854 | 0.00266 | 0.507 | lm.nb | Cytokine Signaling, Inflammatory Signaling, Innate Immune Response | NS\_MM\_NEUROINFLAM\_V1.0 | Cx3cl1 | mRNA | 0 |  | Innate Immune Response;Inflammatory Signaling;Cytokine Signaling | mmu04060;mmu04062;mmu04668 |  | Cx3cl1 | azure4 | 1 | -3.76984126984127 |
  | Il1rap-mRNA | -0.445 | 0.125 | -0.689 | -0.2 | 0.735 | 0.62 | 0.871 | 0.00391 | 0.558 | loglinear | Apoptosis, Cytokine Signaling | NS\_MM\_NEUROINFLAM\_V1.0 | Il1rap | mRNA | 0 |  | Cytokine Signaling;Apoptosis | mmu04060;mmu04210;mmu04750 |  | Il1rap | azure4 | 1 | -3.56 |
  | Braf-mRNA | 0.0984 | 0.0282 | 0.0432 | 0.154 | 1.07 | 1.03 | 1.11 | 0.00445 | 0.577 | loglinear | Adaptive Immune Response, Angiogenesis, Apoptosis, Cytokine Signaling, Growth Factor Signaling, Innate Immune Response, Insulin Signaling, Neurons and Neurotransmission | NS\_MM\_NEUROINFLAM\_V1.0 | Braf | mRNA | 0 |  | Neurons and Neurotransmission;Insulin Signaling;Innate Immune Response;Growth Factor Signaling;Cytokine Signaling;Apoptosis;Angiogenesis;Adaptive Immune Response | mmu04010;mmu04012;mmu04015;mmu04024;mmu04062;mmu04068;mmu04150;mmu04270;mmu04510;mmu04650;mmu04720;mmu04722;mmu04726;mmu04730;mmu04810;mmu04910;mmu04914;mmu05034;mmu05160;mmu05200;mmu05205;mmu05210;mmu05211;mmu05212;mmu05213;mmu05214;mmu05215;mmu05216;mmu05218;mmu05219;mmu05220;mmu05221;mmu05223 |  | Braf | azure4 | 1 | 3.48936170212766 |
  | Il1r1-mRNA | 0.625 | 0.181 | 0.27 | 0.98 | 1.54 | 1.21 | 1.97 | 0.00477 | 0.577 | lm.nb | Apoptosis, Cellular Stress, Cytokine Signaling, Growth Factor Signaling, Microglia Function, NF-kB | NS\_MM\_NEUROINFLAM\_V1.0 | Il1r1 | mRNA | 0 |  | NF-kB;Microglia Function;Growth Factor Signaling;Cytokine Signaling;Cellular Stress;Apoptosis | mmu04010;mmu04060;mmu04064;mmu04210;mmu04380;mmu04640;mmu04750;mmu05146;mmu05166 |  | Il1r1 | azure4 | 1 | 3.45303867403315 |
  | Csf1r-mRNA | -0.635 | 0.198 | -1.02 | -0.248 | 0.644 | 0.492 | 0.842 | 0.00748 | 0.76 | lm.nb | Cytokine Signaling, Growth Factor Signaling, Microglia Function | NS\_MM\_NEUROINFLAM\_V1.0 | Csf1r | mRNA | 0 |  | Microglia Function;Growth Factor Signaling;Cytokine Signaling | mmu04014;mmu04015;mmu04060;mmu04144;mmu04151;mmu04380;mmu04640;mmu05200;mmu05202 |  | Csf1r | azure4 | 1 | -3.20707070707071 |
  | Bcl2l1-mRNA | 0.173 | 0.0572 | 0.0603 | 0.285 | 1.13 | 1.04 | 1.22 | 0.0108 | 0.861 | loglinear | Apoptosis, Autophagy, Cytokine Signaling, Growth Factor Signaling, Innate Immune Response, NF-kB | NS\_MM\_NEUROINFLAM\_V1.0 | Bcl2l1 | mRNA | 0 |  | NF-kB;Innate Immune Response;Growth Factor Signaling;Cytokine Signaling;Autophagy;Apoptosis | mmu04014;mmu04064;mmu04151;mmu04210;mmu04630;mmu05014;mmu05145;mmu05166;mmu05200;mmu05202;mmu05212;mmu05220;mmu05222 |  | Bcl2l1 | azure4 | 1 | 3.02447552447552 |
  | Grin2b-mRNA | -0.218 | 0.0772 | -0.369 | -0.0665 | 0.86 | 0.774 | 0.955 | 0.0154 | 1 | loglinear | Adaptive Immune Response, Angiogenesis, Cytokine Signaling, Growth Factor Signaling, Insulin Signaling, Neurons and Neurotransmission | NS\_MM\_NEUROINFLAM\_V1.0 | Grin2b | mRNA | 0 |  | Neurons and Neurotransmission;Insulin Signaling;Growth Factor Signaling;Cytokine Signaling;Angiogenesis;Adaptive Immune Response | mmu04014;mmu04015;mmu04024;mmu04080;mmu04713;mmu04720;mmu04724;mmu04728;mmu05010;mmu05014;mmu05016;mmu05030;mmu05031;mmu05033;mmu05034;mmu05322 |  | Grin2b | azure4 | 1 | -2.82383419689119 |
  | Myd88-mRNA | 0.604 | 0.215 | 0.184 | 1.02 | 1.52 | 1.14 | 2.03 | 0.0156 | 1 | lm.nb | Apoptosis, Cytokine Signaling, Growth Factor Signaling, Inflammatory Signaling, Innate Immune Response, NF-kB | NS\_MM\_NEUROINFLAM\_V1.0 | Myd88 | mRNA | 0 |  | NF-kB;Innate Immune Response;Inflammatory Signaling;Growth Factor Signaling;Cytokine Signaling;Apoptosis | mmu04064;mmu04210;mmu04620;mmu05132;mmu05133;mmu05134;mmu05140;mmu05142;mmu05143;mmu05144;mmu05145;mmu05152;mmu05161;mmu05162;mmu05164;mmu05168 |  | Myd88 | azure4 | 1 | 2.8093023255814 |
  | Tnfrsf1a-mRNA | 0.827 | 0.299 | 0.241 | 1.41 | 1.77 | 1.18 | 2.66 | 0.017 | 1 | lm.nb | Apoptosis, Cytokine Signaling, Growth Factor Signaling, Innate Immune Response, NF-kB | NS\_MM\_NEUROINFLAM\_V1.0 | Tnfrsf1a | mRNA | 0 |  | NF-kB;Innate Immune Response;Growth Factor Signaling;Cytokine Signaling;Apoptosis | mmu04010;mmu04060;mmu04064;mmu04071;mmu04210;mmu04380;mmu04668;mmu04920;mmu04931;mmu04932;mmu05010;mmu05014;mmu05142;mmu05145;mmu05152;mmu05160;mmu05164;mmu05166;mmu05168 |  | Tnfrsf1a | azure4 | 1 | 2.76588628762542 |
  | Tgfbr1-mRNA | -0.354 | 0.135 | -0.618 | -0.0896 | 0.783 | 0.652 | 0.94 | 0.0222 | 1 | lm.nb | Cytokine Signaling, Growth Factor Signaling | NS\_MM\_NEUROINFLAM\_V1.0 | Tgfbr1 | mRNA | 0 |  | Growth Factor Signaling;Cytokine Signaling | mmu04010;mmu04060;mmu04068;mmu04144;mmu04350;mmu04380;mmu04390;mmu04520;mmu04933;mmu05142;mmu05161;mmu05166;mmu05200;mmu05210;mmu05212;mmu05220 |  | Tgfbr1 | azure4 | 1 | -2.62222222222222 |
  | Pik3r2-mRNA | -0.099 | 0.0389 | -0.175 | -0.0229 | 0.934 | 0.886 | 0.984 | 0.0256 | 1 | loglinear | Adaptive Immune Response, Angiogenesis, Apoptosis, Autophagy, Cytokine Signaling, Growth Factor Signaling, Innate Immune Response, Insulin Signaling | NS\_MM\_NEUROINFLAM\_V1.0 | Pik3r2 | mRNA | 0 |  | Insulin Signaling;Innate Immune Response;Growth Factor Signaling;Cytokine Signaling;Autophagy;Apoptosis;Angiogenesis;Adaptive Immune Response | mmu04012;mmu04014;mmu04015;mmu04022;mmu04024;mmu04062;mmu04066;mmu04068;mmu04070;mmu04071;mmu04072;mmu04150;mmu04151;mmu04152;mmu04210;mmu04211;mmu04213;mmu04261;mmu04370;mmu04380;mmu04510;mmu04550;mmu04611;mmu04620;mmu04630;mmu04650;mmu04660;mmu04662;mmu04664;mmu04666;mmu04668;mmu04670;mmu04722;mmu04725;mmu04750;mmu04810;mmu04910;mmu04914;mmu04915;mmu04917;mmu04919;mmu04921;mmu04923;mmu04930;mmu04931;mmu04932;mmu04933;mmu04960;mmu04973;mmu05100;mmu05142;mmu05145;mmu05146;mmu05160;mmu05161;mmu05162;mmu05164;mmu05166;mmu05169;mmu05200;mmu05203;mmu05205;mmu05210;mmu05211;mmu05212;mmu05213;mmu05214;mmu05215;mmu05218;mmu05220;mmu05221;mmu05222;mmu05223;mmu05230;mmu05231 |  | Pik3r2 | azure4 | 1 | -2.54498714652956 |
  | Il1a-mRNA | 2.22 | 0.937 | 0.385 | 4.06 | 4.67 | 1.31 | 16.7 | 0.0354 | 1 | lm.nb | Apoptosis, Cellular Stress, Cytokine Signaling, Growth Factor Signaling | NS\_MM\_NEUROINFLAM\_V1.0 | Il1a | mRNA | 0 |  | Growth Factor Signaling;Cytokine Signaling;Cellular Stress;Apoptosis | mmu04010;mmu04060;mmu04210;mmu04380;mmu04640;mmu04932;mmu04933;mmu04940;mmu05020;mmu05132;mmu05133;mmu05140;mmu05152;mmu05162;mmu05164;mmu05321;mmu05323;mmu05332 |  | Il1a | azure4 | 1 | 2.3692636072572 |
  | Traf3-mRNA | -0.322 | 0.139 | -0.594 | -0.0498 | 0.8 | 0.662 | 0.966 | 0.0389 | 1 | loglinear | Apoptosis, Cytokine Signaling, Inflammatory Signaling, Innate Immune Response, NF-kB | NS\_MM\_NEUROINFLAM\_V1.0 | Traf3 | mRNA | 0 |  | NF-kB;Innate Immune Response;Inflammatory Signaling;Cytokine Signaling;Apoptosis | mmu04064;mmu04620;mmu04622;mmu04668;mmu05160;mmu05168;mmu05169;mmu05200;mmu05203;mmu05222 |  | Traf3 | azure4 | 1 | -2.31654676258993 |
  | Cdkn1a-mRNA | 0.349 | 0.153 | 0.0486 | 0.649 | 1.27 | 1.03 | 1.57 | 0.0419 | 1 | lm.nb | Adaptive Immune Response, Cell Cycle, Cellular Stress, Cytokine Signaling, DNA Damage, Growth Factor Signaling | NS\_MM\_NEUROINFLAM\_V1.0 | Cdkn1a | mRNA | 0 |  | Growth Factor Signaling;DNA Damage;Cytokine Signaling;Cellular Stress;Cell Cycle;Adaptive Immune Response | mmu04012;mmu04066;mmu04068;mmu04110;mmu04115;mmu04151;mmu04630;mmu04921;mmu05160;mmu05161;mmu05166;mmu05169;mmu05200;mmu05202;mmu05203;mmu05205;mmu05206;mmu05214;mmu05215;mmu05218;mmu05219;mmu05220 |  | Cdkn1a | azure4 | 1 | 2.28104575163399 |
  | Cx3cr1-mRNA | -0.733 | 0.388 | -1.49 | 0.0282 | 0.602 | 0.355 | 1.02 | 0.0835 | 1 | lm.nb | Cytokine Signaling, Microglia Function | NS\_MM\_NEUROINFLAM\_V1.0 | Cx3cr1 | mRNA | 0 |  | Microglia Function;Cytokine Signaling | mmu04060;mmu04062 |  | Cx3cr1 | azure4 | 1 | -1.88917525773196 |
  | Chuk-mRNA | -0.302 | 0.162 | -0.619 | 0.0144 | 0.811 | 0.651 | 1.01 | 0.0859 | 1 | lm.nb | Adaptive Immune Response, Apoptosis, Cytokine Signaling, Growth Factor Signaling, Inflammatory Signaling, Innate Immune Response, NF-kB | NS\_MM\_NEUROINFLAM\_V1.0 | Chuk | mRNA | 0 |  | NF-kB;Innate Immune Response;Inflammatory Signaling;Growth Factor Signaling;Cytokine Signaling;Apoptosis;Adaptive Immune Response | mmu04010;mmu04014;mmu04062;mmu04064;mmu04068;mmu04151;mmu04210;mmu04380;mmu04620;mmu04621;mmu04622;mmu04623;mmu04660;mmu04662;mmu04668;mmu04920;mmu05142;mmu05145;mmu05160;mmu05161;mmu05162;mmu05166;mmu05168;mmu05169;mmu05200;mmu05212;mmu05215;mmu05220;mmu05221;mmu05222 |  | Chuk | azure4 | 1 | -1.8641975308642 |
  | Ltbr-mRNA | 0.428 | 0.237 | -0.0364 | 0.892 | 1.35 | 0.975 | 1.86 | 0.096 | 1 | loglinear | Apoptosis, Cytokine Signaling, Inflammatory Signaling, NF-kB | NS\_MM\_NEUROINFLAM\_V1.0 | Ltbr | mRNA | 0 |  | NF-kB;Inflammatory Signaling;Cytokine Signaling;Apoptosis | mmu04060;mmu04064;mmu04066;mmu04672;mmu05166;mmu05203 |  | Ltbr | azure4 | 1 | 1.80590717299578 |
  | Pik3r1-mRNA | 0.15 | 0.0891 | -0.0245 | 0.325 | 1.11 | 0.983 | 1.25 | 0.118 | 1 | loglinear | Adaptive Immune Response, Angiogenesis, Apoptosis, Autophagy, Carbohydrate Metabolism, Cytokine Signaling, Growth Factor Signaling, Innate Immune Response, Insulin Signaling, Lipid Metabolism | NS\_MM\_NEUROINFLAM\_V1.0 | Pik3r1 | mRNA | 0 |  | Lipid Metabolism;Insulin Signaling;Innate Immune Response;Growth Factor Signaling;Cytokine Signaling;Carbohydrate Metabolism;Autophagy;Apoptosis;Angiogenesis;Adaptive Immune Response | mmu04012;mmu04014;mmu04015;mmu04022;mmu04024;mmu04062;mmu04066;mmu04068;mmu04070;mmu04071;mmu04072;mmu04150;mmu04151;mmu04152;mmu04210;mmu04211;mmu04213;mmu04261;mmu04370;mmu04380;mmu04510;mmu04550;mmu04611;mmu04620;mmu04630;mmu04650;mmu04660;mmu04662;mmu04664;mmu04666;mmu04668;mmu04670;mmu04722;mmu04725;mmu04750;mmu04810;mmu04910;mmu04914;mmu04915;mmu04917;mmu04919;mmu04921;mmu04923;mmu04930;mmu04931;mmu04932;mmu04933;mmu04960;mmu04973;mmu05100;mmu05142;mmu05145;mmu05146;mmu05160;mmu05161;mmu05162;mmu05164;mmu05166;mmu05169;mmu05200;mmu05203;mmu05205;mmu05210;mmu05211;mmu05212;mmu05213;mmu05214;mmu05215;mmu05218;mmu05220;mmu05221;mmu05222;mmu05223;mmu05230;mmu05231 |  | Pik3r1 | azure4 | 1 | 1.68350168350168 |

  ##### DE Results - Cytokine Signaling genes - DPI: 1 vs.CTRL

  Table displaying each sample's global significance scores and directed global significance scores as defined in the heatmaps above. The global significance score is calculated as the square root of the mean squared t-statistic for the genes in a gene set, with t-statistics coming from the linear regression underlying our differential expression analysis. The directed global significance score is calculated as the square root of the mean signed squared t-statistic for the genes in a gene set, with t-statistics coming from the linear regression underlying our differential expression analysis.

- **Volcano Plot: DPI: 2 vs.CTRL  
  More Plot Information**

  ##### Volcano Plot: DPI: 2 vs.CTRL

  Volcano plot displaying each gene's -log10(p-value) and log2 fold change for the selected covariate. Highly statistically significant genes fall at the top of the plot, and highly differentially expressed genes fall to either side. Genes within the selected gene set are highlighted in orange. Horizontal lines indicate various False Discovery Rate (FDR) thresholds.
- **DE Results - Cytokine Signaling genes - DPI: 2 vs.CTRL  
  More Plot Information  Download CSV Data**

  | Probe Label | Log2 fold change | std error (log2) | Lower confidence limit (log2) | Upper confidence limit (log2) | Linear fold change | Lower confidence limit (linear) | Upper confidence limit (linear) | P-value | BY.p.value | method | Gene.sets | Codeset.Name | Probe.Label | Analyte.Type | Is.Control | Control.Type | Probe.Annotation | KEGG.Pathways | Cell.Type | Official.Gene.Name | volcanocol | volcanopch | tstats.all |
  | --- | --- | --- | --- | --- | --- | --- | --- | --- | --- | --- | --- | --- | --- | --- | --- | --- | --- | --- | --- | --- | --- | --- | --- |
  | Cdkn1a-mRNA | 2.21 | 0.15 | 1.92 | 2.51 | 4.64 | 3.78 | 5.68 | 4.64e-09 | 3.71e-06 | lm.nb | Adaptive Immune Response, Cell Cycle, Cellular Stress, Cytokine Signaling, DNA Damage, Growth Factor Signaling | NS\_MM\_NEUROINFLAM\_V1.0 | Cdkn1a | mRNA | 0 |  | Growth Factor Signaling;DNA Damage;Cytokine Signaling;Cellular Stress;Cell Cycle;Adaptive Immune Response | mmu04012;mmu04066;mmu04068;mmu04110;mmu04115;mmu04151;mmu04630;mmu04921;mmu05160;mmu05161;mmu05166;mmu05169;mmu05200;mmu05202;mmu05203;mmu05205;mmu05206;mmu05214;mmu05215;mmu05218;mmu05219;mmu05220 |  | Cdkn1a | slateblue1 | 16 | 14.7333333333333 |
  | Psmb8-mRNA | 2.78 | 0.196 | 2.39 | 3.16 | 6.85 | 5.25 | 8.94 | 7.51e-09 | 4.29e-06 | lm.nb | Adaptive Immune Response, Angiogenesis, Apoptosis, Astrocyte Function, Cell Cycle, Cytokine Signaling, Growth Factor Signaling, Inflammatory Signaling, Insulin Signaling, Microglia Function, NF-kB, Wnt | NS\_MM\_NEUROINFLAM\_V1.0 | Psmb8 | mRNA | 0 |  | Wnt;NF-kB;Microglia Function;Insulin Signaling;Inflammatory Signaling;Growth Factor Signaling;Cytokine Signaling;Cell Cycle;Astrocyte Function;Apoptosis;Angiogenesis;Adaptive Immune Response |  |  | Psmb8 | slateblue1 | 16 | 14.1836734693878 |
  | Cxcl10-mRNA | 6.62 | 0.741 | 5.17 | 8.07 | 98.6 | 36 | 270 | 1.18e-06 | 0.000256 | lm.nb | Astrocyte Function, Cytokine Signaling, Inflammatory Signaling, Innate Immune Response, Microglia Function | NS\_MM\_NEUROINFLAM\_V1.0 | Cxcl10 | mRNA | 0 |  | Microglia Function;Innate Immune Response;Inflammatory Signaling;Cytokine Signaling;Astrocyte Function | mmu04060;mmu04062;mmu04620;mmu04622;mmu04623;mmu04668;mmu05164 |  | Cxcl10 | slateblue1 | 16 | 8.93387314439946 |
  | Pik3r2-mRNA | -0.347 | 0.0389 | -0.423 | -0.27 | 0.786 | 0.746 | 0.829 | 1.22e-06 | 0.000256 | loglinear | Adaptive Immune Response, Angiogenesis, Apoptosis, Autophagy, Cytokine Signaling, Growth Factor Signaling, Innate Immune Response, Insulin Signaling | NS\_MM\_NEUROINFLAM\_V1.0 | Pik3r2 | mRNA | 0 |  | Insulin Signaling;Innate Immune Response;Growth Factor Signaling;Cytokine Signaling;Autophagy;Apoptosis;Angiogenesis;Adaptive Immune Response | mmu04012;mmu04014;mmu04015;mmu04022;mmu04024;mmu04062;mmu04066;mmu04068;mmu04070;mmu04071;mmu04072;mmu04150;mmu04151;mmu04152;mmu04210;mmu04211;mmu04213;mmu04261;mmu04370;mmu04380;mmu04510;mmu04550;mmu04611;mmu04620;mmu04630;mmu04650;mmu04660;mmu04662;mmu04664;mmu04666;mmu04668;mmu04670;mmu04722;mmu04725;mmu04750;mmu04810;mmu04910;mmu04914;mmu04915;mmu04917;mmu04919;mmu04921;mmu04923;mmu04930;mmu04931;mmu04932;mmu04933;mmu04960;mmu04973;mmu05100;mmu05142;mmu05145;mmu05146;mmu05160;mmu05161;mmu05162;mmu05164;mmu05166;mmu05169;mmu05200;mmu05203;mmu05205;mmu05210;mmu05211;mmu05212;mmu05213;mmu05214;mmu05215;mmu05218;mmu05220;mmu05221;mmu05222;mmu05223;mmu05230;mmu05231 |  | Pik3r2 | slateblue1 | 16 | -8.92030848329049 |
  | Osmr-mRNA | 2.76 | 0.326 | 2.12 | 3.4 | 6.77 | 4.35 | 10.5 | 2.1e-06 | 0.000373 | lm.nb | Astrocyte Function, Cytokine Signaling, Growth Factor Signaling | NS\_MM\_NEUROINFLAM\_V1.0 | Osmr | mRNA | 0 |  | Growth Factor Signaling;Cytokine Signaling;Astrocyte Function | mmu04060;mmu04151;mmu04630 |  | Osmr | slateblue1 | 16 | 8.46625766871166 |
  | Ccl2-mRNA | 7.94 | 0.958 | 6.06 | 9.81 | 245 | 66.6 | 899 | 2.63e-06 | 0.00042 | lm.nb | Astrocyte Function, Cytokine Signaling, Inflammatory Signaling, Innate Immune Response | NS\_MM\_NEUROINFLAM\_V1.0 | Ccl2 | mRNA | 0 |  | Innate Immune Response;Inflammatory Signaling;Cytokine Signaling;Astrocyte Function | mmu04060;mmu04062;mmu04621;mmu04668;mmu04933;mmu05142;mmu05144;mmu05164;mmu05168;mmu05323 | DC | Ccl2 | slateblue1 | 16 | 8.28810020876827 |
  | Stat1-mRNA | 1.36 | 0.2 | 0.967 | 1.75 | 2.56 | 1.95 | 3.36 | 1.9e-05 | 0.00217 | lm.nb | Cytokine Signaling, Growth Factor Signaling, Inflammatory Signaling, Microglia Function | NS\_MM\_NEUROINFLAM\_V1.0 | Stat1 | mRNA | 0 |  | Microglia Function;Inflammatory Signaling;Growth Factor Signaling;Cytokine Signaling | mmu04062;mmu04380;mmu04620;mmu04630;mmu04917;mmu04919;mmu04933;mmu05140;mmu05145;mmu05152;mmu05160;mmu05161;mmu05162;mmu05164;mmu05168;mmu05200;mmu05212;mmu05321 |  | Stat1 | slateblue1 | 16 | 6.8 |
  | Il1r1-mRNA | 1.09 | 0.175 | 0.752 | 1.44 | 2.14 | 1.68 | 2.71 | 4.23e-05 | 0.00403 | lm.nb | Apoptosis, Cellular Stress, Cytokine Signaling, Growth Factor Signaling, Microglia Function, NF-kB | NS\_MM\_NEUROINFLAM\_V1.0 | Il1r1 | mRNA | 0 |  | NF-kB;Microglia Function;Growth Factor Signaling;Cytokine Signaling;Cellular Stress;Apoptosis | mmu04010;mmu04060;mmu04064;mmu04210;mmu04380;mmu04640;mmu04750;mmu05146;mmu05166 |  | Il1r1 | slateblue1 | 16 | 6.22857142857143 |
  | Tgfbr1-mRNA | -0.817 | 0.139 | -1.09 | -0.544 | 0.568 | 0.47 | 0.686 | 7.66e-05 | 0.00588 | lm.nb | Cytokine Signaling, Growth Factor Signaling | NS\_MM\_NEUROINFLAM\_V1.0 | Tgfbr1 | mRNA | 0 |  | Growth Factor Signaling;Cytokine Signaling | mmu04010;mmu04060;mmu04068;mmu04144;mmu04350;mmu04380;mmu04390;mmu04520;mmu04933;mmu05142;mmu05161;mmu05166;mmu05200;mmu05210;mmu05212;mmu05220 |  | Tgfbr1 | slateblue1 | 16 | -5.87769784172662 |
  | Bcl2l1-mRNA | 0.33 | 0.0572 | 0.217 | 0.442 | 1.26 | 1.16 | 1.36 | 9.06e-05 | 0.00658 | loglinear | Apoptosis, Autophagy, Cytokine Signaling, Growth Factor Signaling, Innate Immune Response, NF-kB | NS\_MM\_NEUROINFLAM\_V1.0 | Bcl2l1 | mRNA | 0 |  | NF-kB;Innate Immune Response;Growth Factor Signaling;Cytokine Signaling;Autophagy;Apoptosis | mmu04014;mmu04064;mmu04151;mmu04210;mmu04630;mmu05014;mmu05145;mmu05166;mmu05200;mmu05202;mmu05212;mmu05220;mmu05222 |  | Bcl2l1 | slateblue1 | 16 | 5.76923076923077 |
  | Cx3cl1-mRNA | -0.706 | 0.127 | -0.955 | -0.458 | 0.613 | 0.516 | 0.728 | 0.000121 | 0.00818 | lm.nb | Cytokine Signaling, Inflammatory Signaling, Innate Immune Response | NS\_MM\_NEUROINFLAM\_V1.0 | Cx3cl1 | mRNA | 0 |  | Innate Immune Response;Inflammatory Signaling;Cytokine Signaling | mmu04060;mmu04062;mmu04668 |  | Cx3cl1 | slateblue1 | 16 | -5.55905511811024 |
  | Sumo1-mRNA | -0.199 | 0.0361 | -0.269 | -0.128 | 0.871 | 0.83 | 0.915 | 0.000135 | 0.00884 | loglinear | Cell Cycle, Cytokine Signaling, DNA Damage, Inflammatory Signaling | NS\_MM\_NEUROINFLAM\_V1.0 | Sumo1 | mRNA | 0 |  | Inflammatory Signaling;DNA Damage;Cytokine Signaling;Cell Cycle |  |  | Sumo1 | slateblue1 | 16 | -5.51246537396122 |
  | Grin2b-mRNA | -0.381 | 0.0772 | -0.533 | -0.23 | 0.768 | 0.691 | 0.853 | 0.00034 | 0.02 | loglinear | Adaptive Immune Response, Angiogenesis, Cytokine Signaling, Growth Factor Signaling, Insulin Signaling, Neurons and Neurotransmission | NS\_MM\_NEUROINFLAM\_V1.0 | Grin2b | mRNA | 0 |  | Neurons and Neurotransmission;Insulin Signaling;Growth Factor Signaling;Cytokine Signaling;Angiogenesis;Adaptive Immune Response | mmu04014;mmu04015;mmu04024;mmu04080;mmu04713;mmu04720;mmu04724;mmu04728;mmu05010;mmu05014;mmu05016;mmu05030;mmu05031;mmu05033;mmu05034;mmu05322 |  | Grin2b | slateblue2 | 16 | -4.93523316062176 |
  | Myd88-mRNA | 1.01 | 0.21 | 0.601 | 1.42 | 2.02 | 1.52 | 2.68 | 0.000418 | 0.0228 | lm.nb | Apoptosis, Cytokine Signaling, Growth Factor Signaling, Inflammatory Signaling, Innate Immune Response, NF-kB | NS\_MM\_NEUROINFLAM\_V1.0 | Myd88 | mRNA | 0 |  | NF-kB;Innate Immune Response;Inflammatory Signaling;Growth Factor Signaling;Cytokine Signaling;Apoptosis | mmu04064;mmu04210;mmu04620;mmu05132;mmu05133;mmu05134;mmu05140;mmu05142;mmu05143;mmu05144;mmu05145;mmu05152;mmu05161;mmu05162;mmu05164;mmu05168 |  | Myd88 | slateblue2 | 16 | 4.80952380952381 |
  | Il1a-mRNA | 4.25 | 0.922 | 2.44 | 6.06 | 19 | 5.43 | 66.5 | 0.000605 | 0.0291 | lm.nb | Apoptosis, Cellular Stress, Cytokine Signaling, Growth Factor Signaling | NS\_MM\_NEUROINFLAM\_V1.0 | Il1a | mRNA | 0 |  | Growth Factor Signaling;Cytokine Signaling;Cellular Stress;Apoptosis | mmu04010;mmu04060;mmu04210;mmu04380;mmu04640;mmu04932;mmu04933;mmu04940;mmu05020;mmu05132;mmu05133;mmu05140;mmu05152;mmu05162;mmu05164;mmu05321;mmu05323;mmu05332 |  | Il1a | slateblue2 | 16 | 4.60954446854664 |
  | Tnfrsf1a-mRNA | 1.3 | 0.295 | 0.72 | 1.88 | 2.46 | 1.65 | 3.67 | 0.000868 | 0.0385 | lm.nb | Apoptosis, Cytokine Signaling, Growth Factor Signaling, Innate Immune Response, NF-kB | NS\_MM\_NEUROINFLAM\_V1.0 | Tnfrsf1a | mRNA | 0 |  | NF-kB;Innate Immune Response;Growth Factor Signaling;Cytokine Signaling;Apoptosis | mmu04010;mmu04060;mmu04064;mmu04071;mmu04210;mmu04380;mmu04668;mmu04920;mmu04931;mmu04932;mmu05010;mmu05014;mmu05142;mmu05145;mmu05152;mmu05160;mmu05164;mmu05166;mmu05168 |  | Tnfrsf1a | slateblue2 | 16 | 4.40677966101695 |
  | Sqstm1-mRNA | 0.107 | 0.0261 | 0.0554 | 0.158 | 1.08 | 1.04 | 1.12 | 0.00152 | 0.0584 | loglinear | Autophagy, Cytokine Signaling, Growth Factor Signaling | NS\_MM\_NEUROINFLAM\_V1.0 | Sqstm1 | mRNA | 0 |  | Growth Factor Signaling;Cytokine Signaling;Autophagy | mmu04380 |  | Sqstm1 | slateblue3 | 16 | 4.09961685823755 |
  | Il6ra-mRNA | 1.06 | 0.263 | 0.547 | 1.58 | 2.09 | 1.46 | 2.99 | 0.00164 | 0.0619 | lm.nb | Cytokine Signaling, Growth Factor Signaling, Microglia Function | NS\_MM\_NEUROINFLAM\_V1.0 | Il6ra | mRNA | 0 |  | Microglia Function;Growth Factor Signaling;Cytokine Signaling | mmu04060;mmu04066;mmu04151;mmu04630;mmu04640;mmu04932 |  | Il6ra | slateblue3 | 16 | 4.03041825095057 |
  | Cx3cr1-mRNA | -1.45 | 0.391 | -2.22 | -0.687 | 0.365 | 0.215 | 0.621 | 0.00296 | 0.0968 | lm.nb | Cytokine Signaling, Microglia Function | NS\_MM\_NEUROINFLAM\_V1.0 | Cx3cr1 | mRNA | 0 |  | Microglia Function;Cytokine Signaling | mmu04060;mmu04062 |  | Cx3cr1 | slateblue4 | 16 | -3.70843989769821 |
  | Dusp7-mRNA | -0.315 | 0.0861 | -0.484 | -0.147 | 0.804 | 0.715 | 0.903 | 0.00325 | 0.104 | loglinear | Adaptive Immune Response, Angiogenesis, Cytokine Signaling, Growth Factor Signaling, Innate Immune Response, Insulin Signaling, Microglia Function | NS\_MM\_NEUROINFLAM\_V1.0 | Dusp7 | mRNA | 0 |  | Microglia Function;Insulin Signaling;Innate Immune Response;Growth Factor Signaling;Cytokine Signaling;Angiogenesis;Adaptive Immune Response | mmu04010 |  | Dusp7 | slateblue4 | 16 | -3.65853658536585 |

  ##### DE Results - Cytokine Signaling genes - DPI: 2 vs.CTRL

  Table displaying each sample's global significance scores and directed global significance scores as defined in the heatmaps above. The global significance score is calculated as the square root of the mean squared t-statistic for the genes in a gene set, with t-statistics coming from the linear regression underlying our differential expression analysis. The directed global significance score is calculated as the square root of the mean signed squared t-statistic for the genes in a gene set, with t-statistics coming from the linear regression underlying our differential expression analysis.

- **Volcano Plot: DPI: 3 vs.CTRL  
  More Plot Information**

  ##### Volcano Plot: DPI: 3 vs.CTRL

  Volcano plot displaying each gene's -log10(p-value) and log2 fold change for the selected covariate. Highly statistically significant genes fall at the top of the plot, and highly differentially expressed genes fall to either side. Genes within the selected gene set are highlighted in orange. Horizontal lines indicate various False Discovery Rate (FDR) thresholds.
- **DE Results - Cytokine Signaling genes - DPI: 3 vs.CTRL  
  More Plot Information  Download CSV Data**

  | Probe Label | Log2 fold change | std error (log2) | Lower confidence limit (log2) | Upper confidence limit (log2) | Linear fold change | Lower confidence limit (linear) | Upper confidence limit (linear) | P-value | BY.p.value | method | Gene.sets | Codeset.Name | Probe.Label | Analyte.Type | Is.Control | Control.Type | Probe.Annotation | KEGG.Pathways | Cell.Type | Official.Gene.Name | volcanocol | volcanopch | tstats.all |
  | --- | --- | --- | --- | --- | --- | --- | --- | --- | --- | --- | --- | --- | --- | --- | --- | --- | --- | --- | --- | --- | --- | --- | --- |
  | Psmb8-mRNA | 4.35 | 0.191 | 3.98 | 4.73 | 20.4 | 15.7 | 26.5 | 3.09e-11 | 3.33e-08 | lm.nb | Adaptive Immune Response, Angiogenesis, Apoptosis, Astrocyte Function, Cell Cycle, Cytokine Signaling, Growth Factor Signaling, Inflammatory Signaling, Insulin Signaling, Microglia Function, NF-kB, Wnt | NS\_MM\_NEUROINFLAM\_V1.0 | Psmb8 | mRNA | 0 |  | Wnt;NF-kB;Microglia Function;Insulin Signaling;Inflammatory Signaling;Growth Factor Signaling;Cytokine Signaling;Cell Cycle;Astrocyte Function;Apoptosis;Angiogenesis;Adaptive Immune Response |  |  | Psmb8 | slateblue1 | 16 | 22.7748691099476 |
  | Cdkn1a-mRNA | 2.23 | 0.15 | 1.93 | 2.52 | 4.68 | 3.82 | 5.74 | 4.31e-09 | 1.43e-06 | lm.nb | Adaptive Immune Response, Cell Cycle, Cellular Stress, Cytokine Signaling, DNA Damage, Growth Factor Signaling | NS\_MM\_NEUROINFLAM\_V1.0 | Cdkn1a | mRNA | 0 |  | Growth Factor Signaling;DNA Damage;Cytokine Signaling;Cellular Stress;Cell Cycle;Adaptive Immune Response | mmu04012;mmu04066;mmu04068;mmu04110;mmu04115;mmu04151;mmu04630;mmu04921;mmu05160;mmu05161;mmu05166;mmu05169;mmu05200;mmu05202;mmu05203;mmu05205;mmu05206;mmu05214;mmu05215;mmu05218;mmu05219;mmu05220 |  | Cdkn1a | slateblue1 | 16 | 14.8666666666667 |
  | Osmr-mRNA | 4.15 | 0.32 | 3.52 | 4.78 | 17.8 | 11.5 | 27.4 | 2.02e-08 | 4.49e-06 | lm.nb | Astrocyte Function, Cytokine Signaling, Growth Factor Signaling | NS\_MM\_NEUROINFLAM\_V1.0 | Osmr | mRNA | 0 |  | Growth Factor Signaling;Cytokine Signaling;Astrocyte Function | mmu04060;mmu04151;mmu04630 |  | Osmr | slateblue1 | 16 | 12.96875 |
  | Cxcl10-mRNA | 9.44 | 0.739 | 7.99 | 10.9 | 693 | 254 | 1890 | 2.42e-08 | 4.84e-06 | lm.nb | Astrocyte Function, Cytokine Signaling, Inflammatory Signaling, Innate Immune Response, Microglia Function | NS\_MM\_NEUROINFLAM\_V1.0 | Cxcl10 | mRNA | 0 |  | Microglia Function;Innate Immune Response;Inflammatory Signaling;Cytokine Signaling;Astrocyte Function | mmu04060;mmu04062;mmu04620;mmu04622;mmu04623;mmu04668;mmu05164 |  | Cxcl10 | slateblue1 | 16 | 12.7740189445196 |
  | Ccl2-mRNA | 10.8 | 0.957 | 8.91 | 12.7 | 1770 | 483 | 6500 | 9.65e-08 | 1.48e-05 | lm.nb | Astrocyte Function, Cytokine Signaling, Inflammatory Signaling, Innate Immune Response | NS\_MM\_NEUROINFLAM\_V1.0 | Ccl2 | mRNA | 0 |  | Innate Immune Response;Inflammatory Signaling;Cytokine Signaling;Astrocyte Function | mmu04060;mmu04062;mmu04621;mmu04668;mmu04933;mmu05142;mmu05144;mmu05164;mmu05168;mmu05323 | DC | Ccl2 | slateblue1 | 16 | 11.2852664576803 |
  | Stat1-mRNA | 2.08 | 0.198 | 1.69 | 2.47 | 4.24 | 3.24 | 5.55 | 2.09e-07 | 2.78e-05 | lm.nb | Cytokine Signaling, Growth Factor Signaling, Inflammatory Signaling, Microglia Function | NS\_MM\_NEUROINFLAM\_V1.0 | Stat1 | mRNA | 0 |  | Microglia Function;Inflammatory Signaling;Growth Factor Signaling;Cytokine Signaling | mmu04062;mmu04380;mmu04620;mmu04630;mmu04917;mmu04919;mmu04933;mmu05140;mmu05145;mmu05152;mmu05160;mmu05161;mmu05162;mmu05164;mmu05168;mmu05200;mmu05212;mmu05321 |  | Stat1 | slateblue1 | 16 | 10.5050505050505 |
  | Myc-mRNA | 1.37 | 0.14 | 1.1 | 1.65 | 2.59 | 2.14 | 3.13 | 4.35e-07 | 4.81e-05 | loglinear | Apoptosis, Cell Cycle, Cytokine Signaling, Growth Factor Signaling, Notch, Wnt | NS\_MM\_NEUROINFLAM\_V1.0 | Myc | mRNA | 0 |  | Wnt;Notch;Growth Factor Signaling;Cytokine Signaling;Cell Cycle;Apoptosis | mmu04010;mmu04012;mmu04110;mmu04151;mmu04310;mmu04350;mmu04390;mmu04550;mmu04630;mmu04919;mmu05161;mmu05166;mmu05169;mmu05200;mmu05202;mmu05205;mmu05206;mmu05210;mmu05213;mmu05216;mmu05219;mmu05220;mmu05221;mmu05222;mmu05230 |  | Myc | slateblue1 | 16 | 9.78571428571428 |
  | Pik3r2-mRNA | -0.374 | 0.0389 | -0.451 | -0.298 | 0.771 | 0.732 | 0.813 | 5.36e-07 | 5.35e-05 | loglinear | Adaptive Immune Response, Angiogenesis, Apoptosis, Autophagy, Cytokine Signaling, Growth Factor Signaling, Innate Immune Response, Insulin Signaling | NS\_MM\_NEUROINFLAM\_V1.0 | Pik3r2 | mRNA | 0 |  | Insulin Signaling;Innate Immune Response;Growth Factor Signaling;Cytokine Signaling;Autophagy;Apoptosis;Angiogenesis;Adaptive Immune Response | mmu04012;mmu04014;mmu04015;mmu04022;mmu04024;mmu04062;mmu04066;mmu04068;mmu04070;mmu04071;mmu04072;mmu04150;mmu04151;mmu04152;mmu04210;mmu04211;mmu04213;mmu04261;mmu04370;mmu04380;mmu04510;mmu04550;mmu04611;mmu04620;mmu04630;mmu04650;mmu04660;mmu04662;mmu04664;mmu04666;mmu04668;mmu04670;mmu04722;mmu04725;mmu04750;mmu04810;mmu04910;mmu04914;mmu04915;mmu04917;mmu04919;mmu04921;mmu04923;mmu04930;mmu04931;mmu04932;mmu04933;mmu04960;mmu04973;mmu05100;mmu05142;mmu05145;mmu05146;mmu05160;mmu05161;mmu05162;mmu05164;mmu05166;mmu05169;mmu05200;mmu05203;mmu05205;mmu05210;mmu05211;mmu05212;mmu05213;mmu05214;mmu05215;mmu05218;mmu05220;mmu05221;mmu05222;mmu05223;mmu05230;mmu05231 |  | Pik3r2 | slateblue1 | 16 | -9.61439588688946 |
  | Csf1-mRNA | 1.21 | 0.132 | 0.948 | 1.47 | 2.31 | 1.93 | 2.76 | 9.38e-07 | 8.32e-05 | lm.nb | Astrocyte Function, Autophagy, Cytokine Signaling, Growth Factor Signaling, Innate Immune Response, Microglia Function | NS\_MM\_NEUROINFLAM\_V1.0 | Csf1 | mRNA | 0 |  | Microglia Function;Innate Immune Response;Growth Factor Signaling;Cytokine Signaling;Autophagy;Astrocyte Function | mmu04014;mmu04015;mmu04060;mmu04151;mmu04380;mmu04640;mmu04668;mmu05323 |  | Csf1 | slateblue1 | 16 | 9.16666666666667 |
  | Myd88-mRNA | 1.86 | 0.204 | 1.46 | 2.25 | 3.62 | 2.74 | 4.77 | 9.7e-07 | 8.42e-05 | lm.nb | Apoptosis, Cytokine Signaling, Growth Factor Signaling, Inflammatory Signaling, Innate Immune Response, NF-kB | NS\_MM\_NEUROINFLAM\_V1.0 | Myd88 | mRNA | 0 |  | NF-kB;Innate Immune Response;Inflammatory Signaling;Growth Factor Signaling;Cytokine Signaling;Apoptosis | mmu04064;mmu04210;mmu04620;mmu05132;mmu05133;mmu05134;mmu05140;mmu05142;mmu05143;mmu05144;mmu05145;mmu05152;mmu05161;mmu05162;mmu05164;mmu05168 |  | Myd88 | slateblue1 | 16 | 9.11764705882353 |
  | Sqstm1-mRNA | 0.231 | 0.0261 | 0.18 | 0.283 | 1.17 | 1.13 | 1.22 | 1.29e-06 | 0.000105 | loglinear | Autophagy, Cytokine Signaling, Growth Factor Signaling | NS\_MM\_NEUROINFLAM\_V1.0 | Sqstm1 | mRNA | 0 |  | Growth Factor Signaling;Cytokine Signaling;Autophagy | mmu04380 |  | Sqstm1 | slateblue1 | 16 | 8.85057471264368 |
  | Birc3-mRNA | 3.52 | 0.444 | 2.65 | 4.39 | 11.5 | 6.29 | 21 | 4.05e-06 | 0.000261 | lm.nb | Apoptosis, Cytokine Signaling, Inflammatory Signaling, Innate Immune Response, NF-kB | NS\_MM\_NEUROINFLAM\_V1.0 | Birc3 | mRNA | 0 |  | NF-kB;Innate Immune Response;Inflammatory Signaling;Cytokine Signaling;Apoptosis | mmu04064;mmu04120;mmu04210;mmu04510;mmu04621;mmu04668;mmu05145;mmu05200;mmu05202;mmu05222 |  | Birc3 | slateblue1 | 16 | 7.92792792792793 |
  | Tnfrsf1a-mRNA | 2.23 | 0.291 | 1.66 | 2.8 | 4.7 | 3.17 | 6.98 | 5.68e-06 | 0.000329 | lm.nb | Apoptosis, Cytokine Signaling, Growth Factor Signaling, Innate Immune Response, NF-kB | NS\_MM\_NEUROINFLAM\_V1.0 | Tnfrsf1a | mRNA | 0 |  | NF-kB;Innate Immune Response;Growth Factor Signaling;Cytokine Signaling;Apoptosis | mmu04010;mmu04060;mmu04064;mmu04071;mmu04210;mmu04380;mmu04668;mmu04920;mmu04931;mmu04932;mmu05010;mmu05014;mmu05142;mmu05145;mmu05152;mmu05160;mmu05164;mmu05166;mmu05168 |  | Tnfrsf1a | slateblue1 | 16 | 7.66323024054983 |
  | Il1r1-mRNA | 1.31 | 0.173 | 0.968 | 1.65 | 2.47 | 1.96 | 3.13 | 6.67e-06 | 0.00038 | lm.nb | Apoptosis, Cellular Stress, Cytokine Signaling, Growth Factor Signaling, Microglia Function, NF-kB | NS\_MM\_NEUROINFLAM\_V1.0 | Il1r1 | mRNA | 0 |  | NF-kB;Microglia Function;Growth Factor Signaling;Cytokine Signaling;Cellular Stress;Apoptosis | mmu04010;mmu04060;mmu04064;mmu04210;mmu04380;mmu04640;mmu04750;mmu05146;mmu05166 |  | Il1r1 | slateblue1 | 16 | 7.57225433526012 |
  | Fas-mRNA | 3.25 | 0.436 | 2.39 | 4.1 | 9.5 | 5.26 | 17.2 | 7.68e-06 | 0.00042 | lm.nb | Apoptosis, Cellular Stress, Cytokine Signaling, DNA Damage, Growth Factor Signaling, Innate Immune Response | NS\_MM\_NEUROINFLAM\_V1.0 | Fas | mRNA | 0 |  | Innate Immune Response;Growth Factor Signaling;DNA Damage;Cytokine Signaling;Cellular Stress;Apoptosis | mmu04010;mmu04060;mmu04115;mmu04210;mmu04650;mmu04668;mmu04932;mmu04940;mmu05010;mmu05142;mmu05143;mmu05161;mmu05162;mmu05164;mmu05168;mmu05200;mmu05205;mmu05320;mmu05330;mmu05332 |  | Fas | slateblue1 | 16 | 7.45412844036697 |
  | Il1a-mRNA | 6.35 | 0.919 | 4.55 | 8.15 | 81.3 | 23.3 | 283 | 1.63e-05 | 0.000805 | lm.nb | Apoptosis, Cellular Stress, Cytokine Signaling, Growth Factor Signaling | NS\_MM\_NEUROINFLAM\_V1.0 | Il1a | mRNA | 0 |  | Growth Factor Signaling;Cytokine Signaling;Cellular Stress;Apoptosis | mmu04010;mmu04060;mmu04210;mmu04380;mmu04640;mmu04932;mmu04933;mmu04940;mmu05020;mmu05132;mmu05133;mmu05140;mmu05152;mmu05162;mmu05164;mmu05321;mmu05323;mmu05332 |  | Il1a | slateblue1 | 16 | 6.90968443960827 |
  | Irak3-mRNA | 3.04 | 0.449 | 2.16 | 3.92 | 8.21 | 4.46 | 15.1 | 2e-05 | 0.000972 | lm.nb | Apoptosis, Cytokine Signaling, Innate Immune Response | NS\_MM\_NEUROINFLAM\_V1.0 | Irak3 | mRNA | 0 |  | Innate Immune Response;Cytokine Signaling;Apoptosis | mmu04210;mmu04722 |  | Irak3 | slateblue1 | 16 | 6.77060133630289 |
  | Bcl2l1-mRNA | 0.341 | 0.0572 | 0.229 | 0.453 | 1.27 | 1.17 | 1.37 | 6.59e-05 | 0.00271 | loglinear | Apoptosis, Autophagy, Cytokine Signaling, Growth Factor Signaling, Innate Immune Response, NF-kB | NS\_MM\_NEUROINFLAM\_V1.0 | Bcl2l1 | mRNA | 0 |  | NF-kB;Innate Immune Response;Growth Factor Signaling;Cytokine Signaling;Autophagy;Apoptosis | mmu04014;mmu04064;mmu04151;mmu04210;mmu04630;mmu05014;mmu05145;mmu05166;mmu05200;mmu05202;mmu05212;mmu05220;mmu05222 |  | Bcl2l1 | slateblue1 | 16 | 5.96153846153846 |
  | Cx3cr1-mRNA | -2.22 | 0.397 | -2.99 | -1.44 | 0.215 | 0.126 | 0.369 | 0.000119 | 0.0042 | lm.nb | Cytokine Signaling, Microglia Function | NS\_MM\_NEUROINFLAM\_V1.0 | Cx3cr1 | mRNA | 0 |  | Microglia Function;Cytokine Signaling | mmu04060;mmu04062 |  | Cx3cr1 | slateblue1 | 16 | -5.5919395465995 |
  | Il6ra-mRNA | 1.42 | 0.259 | 0.912 | 1.93 | 2.67 | 1.88 | 3.8 | 0.000139 | 0.00483 | lm.nb | Cytokine Signaling, Growth Factor Signaling, Microglia Function | NS\_MM\_NEUROINFLAM\_V1.0 | Il6ra | mRNA | 0 |  | Microglia Function;Growth Factor Signaling;Cytokine Signaling | mmu04060;mmu04066;mmu04151;mmu04630;mmu04640;mmu04932 |  | Il6ra | slateblue1 | 16 | 5.48262548262548 |

  ##### DE Results - Cytokine Signaling genes - DPI: 3 vs.CTRL

  Table displaying each sample's global significance scores and directed global significance scores as defined in the heatmaps above. The global significance score is calculated as the square root of the mean squared t-statistic for the genes in a gene set, with t-statistics coming from the linear regression underlying our differential expression analysis. The directed global significance score is calculated as the square root of the mean signed squared t-statistic for the genes in a gene set, with t-statistics coming from the linear regression underlying our differential expression analysis.

DPI:
- DPI: differential expression in 1 vs. baseline of CTRL
- DPI: differential expression in 2 vs. baseline of CTRL
- DPI: differential expression in 3 vs. baseline of CTRL

- **Volcano Plot: DPI: 1 vs.CTRL  
  More Plot Information**

  ##### Volcano Plot: DPI: 1 vs.CTRL

  Volcano plot displaying each gene's -log10(p-value) and log2 fold change for the selected covariate. Highly statistically significant genes fall at the top of the plot, and highly differentially expressed genes fall to either side. Genes within the selected gene set are highlighted in orange. Horizontal lines indicate various False Discovery Rate (FDR) thresholds.
- **DE Results - DNA Damage genes - DPI: 1 vs.CTRL  
  More Plot Information  Download CSV Data**

  | Probe Label | Log2 fold change | std error (log2) | Lower confidence limit (log2) | Upper confidence limit (log2) | Linear fold change | Lower confidence limit (linear) | Upper confidence limit (linear) | P-value | BY.p.value | method | Gene.sets | Codeset.Name | Probe.Label | Analyte.Type | Is.Control | Control.Type | Probe.Annotation | KEGG.Pathways | Cell.Type | Official.Gene.Name | volcanocol | volcanopch | tstats.all |
  | --- | --- | --- | --- | --- | --- | --- | --- | --- | --- | --- | --- | --- | --- | --- | --- | --- | --- | --- | --- | --- | --- | --- | --- |
  | Sesn1-mRNA | 0.237 | 0.0612 | 0.117 | 0.357 | 1.18 | 1.08 | 1.28 | 0.00219 | 0.437 | loglinear | DNA Damage | NS\_MM\_NEUROINFLAM\_V1.0 | Sesn1 | mRNA | 0 |  | DNA Damage | mmu04115;mmu04211 |  | Sesn1 | azure4 | 1 | 3.87254901960784 |
  | Casp3-mRNA | 0.393 | 0.132 | 0.134 | 0.652 | 1.31 | 1.1 | 1.57 | 0.0115 | 0.868 | lm.nb | Apoptosis, Cellular Stress, DNA Damage, Growth Factor Signaling, Innate Immune Response, Matrix Remodeling | NS\_MM\_NEUROINFLAM\_V1.0 | Casp3 | mRNA | 0 |  | Matrix Remodeling;Innate Immune Response;Growth Factor Signaling;DNA Damage;Cellular Stress;Apoptosis | mmu04010;mmu04115;mmu04210;mmu04650;mmu04668;mmu04726;mmu04932;mmu04933;mmu05010;mmu05012;mmu05014;mmu05016;mmu05133;mmu05134;mmu05145;mmu05146;mmu05152;mmu05161;mmu05168;mmu05200;mmu05203;mmu05205;mmu05206;mmu05210;mmu05416 |  | Casp3 | azure4 | 1 | 2.97727272727273 |
  | Cdkn1c-mRNA | 0.665 | 0.226 | 0.222 | 1.11 | 1.59 | 1.17 | 2.15 | 0.0123 | 0.875 | lm.nb | Cell Cycle, DNA Damage | NS\_MM\_NEUROINFLAM\_V1.0 | Cdkn1c | mRNA | 0 |  | DNA Damage;Cell Cycle | mmu04110 |  | Cdkn1c | azure4 | 1 | 2.94247787610619 |
  | Prkdc-mRNA | -0.365 | 0.133 | -0.626 | -0.103 | 0.777 | 0.648 | 0.931 | 0.0181 | 1 | loglinear | Cell Cycle, DNA Damage, Innate Immune Response | NS\_MM\_NEUROINFLAM\_V1.0 | Prkdc | mRNA | 0 |  | Innate Immune Response;DNA Damage;Cell Cycle | mmu03450;mmu04110 |  | Prkdc | azure4 | 1 | -2.74436090225564 |
  | Rpa1-mRNA | 0.133 | 0.0503 | 0.0349 | 0.232 | 1.1 | 1.02 | 1.17 | 0.021 | 1 | loglinear | Cell Cycle, Cellular Stress, DNA Damage | NS\_MM\_NEUROINFLAM\_V1.0 | Rpa1 | mRNA | 0 |  | DNA Damage;Cellular Stress;Cell Cycle | mmu03030;mmu03420;mmu03430;mmu03440;mmu03460 |  | Rpa1 | azure4 | 1 | 2.6441351888668 |
  | Cdkn1a-mRNA | 0.349 | 0.153 | 0.0486 | 0.649 | 1.27 | 1.03 | 1.57 | 0.0419 | 1 | lm.nb | Adaptive Immune Response, Cell Cycle, Cellular Stress, Cytokine Signaling, DNA Damage, Growth Factor Signaling | NS\_MM\_NEUROINFLAM\_V1.0 | Cdkn1a | mRNA | 0 |  | Growth Factor Signaling;DNA Damage;Cytokine Signaling;Cellular Stress;Cell Cycle;Adaptive Immune Response | mmu04012;mmu04066;mmu04068;mmu04110;mmu04115;mmu04151;mmu04630;mmu04921;mmu05160;mmu05161;mmu05166;mmu05169;mmu05200;mmu05202;mmu05203;mmu05205;mmu05206;mmu05214;mmu05215;mmu05218;mmu05219;mmu05220 |  | Cdkn1a | azure4 | 1 | 2.28104575163399 |
  | Casp9-mRNA | 0.284 | 0.132 | 0.025 | 0.544 | 1.22 | 1.02 | 1.46 | 0.0528 | 1 | loglinear | Adaptive Immune Response, Apoptosis, DNA Damage, Growth Factor Signaling, Innate Immune Response | NS\_MM\_NEUROINFLAM\_V1.0 | Casp9 | mRNA | 0 |  | Innate Immune Response;Growth Factor Signaling;DNA Damage;Apoptosis;Adaptive Immune Response | mmu04115;mmu04151;mmu04210;mmu04370;mmu04919;mmu05010;mmu05012;mmu05014;mmu05016;mmu05134;mmu05145;mmu05152;mmu05161;mmu05164;mmu05200;mmu05210;mmu05212;mmu05213;mmu05215;mmu05222;mmu05223;mmu05416 |  | Casp9 | azure4 | 1 | 2.15151515151515 |
  | Parp1-mRNA | 0.246 | 0.118 | 0.0149 | 0.477 | 1.19 | 1.01 | 1.39 | 0.0589 | 1 | loglinear | Apoptosis, Cellular Stress, DNA Damage, NF-kB | NS\_MM\_NEUROINFLAM\_V1.0 | Parp1 | mRNA | 0 |  | NF-kB;DNA Damage;Cellular Stress;Apoptosis | mmu03410;mmu04064 |  | Parp1 | azure4 | 1 | 2.08474576271186 |
  | Gadd45g-mRNA | 0.422 | 0.214 | 0.00309 | 0.841 | 1.34 | 1 | 1.79 | 0.0718 | 1 | lm.nb | Cell Cycle, DNA Damage, Growth Factor Signaling | NS\_MM\_NEUROINFLAM\_V1.0 | Gadd45g | mRNA | 0 |  | Growth Factor Signaling;DNA Damage;Cell Cycle | mmu04010;mmu04068;mmu04110;mmu04115 |  | Gadd45g | azure4 | 1 | 1.97196261682243 |
  | Smarca5-mRNA | -0.0842 | 0.0459 | -0.174 | 0.00583 | 0.943 | 0.886 | 1 | 0.0917 | 1 | loglinear | Cell Cycle, DNA Damage, Epigenetic Regulation | NS\_MM\_NEUROINFLAM\_V1.0 | Smarca5 | mRNA | 0 |  | Epigenetic Regulation;DNA Damage;Cell Cycle |  |  | Smarca5 | azure4 | 1 | -1.83442265795207 |
  | Kdm4a-mRNA | 0.325 | 0.186 | -0.0383 | 0.689 | 1.25 | 0.974 | 1.61 | 0.105 | 1 | lm.nb | DNA Damage, Epigenetic Regulation | NS\_MM\_NEUROINFLAM\_V1.0 | Kdm4a | mRNA | 0 |  | Epigenetic Regulation;DNA Damage |  |  | Kdm4a | azure4 | 1 | 1.74731182795699 |
  | Fas-mRNA | 0.755 | 0.471 | -0.168 | 1.68 | 1.69 | 0.89 | 3.2 | 0.135 | 1 | lm.nb | Apoptosis, Cellular Stress, Cytokine Signaling, DNA Damage, Growth Factor Signaling, Innate Immune Response | NS\_MM\_NEUROINFLAM\_V1.0 | Fas | mRNA | 0 |  | Innate Immune Response;Growth Factor Signaling;DNA Damage;Cytokine Signaling;Cellular Stress;Apoptosis | mmu04010;mmu04060;mmu04115;mmu04210;mmu04650;mmu04668;mmu04932;mmu04940;mmu05010;mmu05142;mmu05143;mmu05161;mmu05162;mmu05164;mmu05168;mmu05200;mmu05205;mmu05320;mmu05330;mmu05332 |  | Fas | azure4 | 1 | 1.60297239915074 |
  | Rnf8-mRNA | 0.192 | 0.122 | -0.0461 | 0.43 | 1.14 | 0.969 | 1.35 | 0.14 | 1 | lm.nb | Cell Cycle, DNA Damage | NS\_MM\_NEUROINFLAM\_V1.0 | Rnf8 | mRNA | 0 |  | DNA Damage;Cell Cycle |  |  | Rnf8 | azure4 | 1 | 1.57377049180328 |
  | Mdm2-mRNA | 0.11 | 0.0766 | -0.0405 | 0.26 | 1.08 | 0.972 | 1.2 | 0.178 | 1 | loglinear | Adaptive Immune Response, Apoptosis, Cell Cycle, Cellular Stress, DNA Damage, Growth Factor Signaling, Neurons and Neurotransmission | NS\_MM\_NEUROINFLAM\_V1.0 | Mdm2 | mRNA | 0 |  | Neurons and Neurotransmission;Growth Factor Signaling;DNA Damage;Cellular Stress;Cell Cycle;Apoptosis;Adaptive Immune Response | mmu04068;mmu04110;mmu04115;mmu04120;mmu04144;mmu04151;mmu04919;mmu05169;mmu05200;mmu05202;mmu05203;mmu05205;mmu05206;mmu05214;mmu05215;mmu05218;mmu05219;mmu05220 |  | Mdm2 | azure4 | 1 | 1.43603133159269 |
  | Pms2-mRNA | -0.117 | 0.0874 | -0.288 | 0.0545 | 0.922 | 0.819 | 1.04 | 0.206 | 1 | loglinear | DNA Damage | NS\_MM\_NEUROINFLAM\_V1.0 | Pms2 | mRNA | 0 |  | DNA Damage | mmu03430;mmu03460 |  | Pms2 | azure4 | 1 | -1.33867276887872 |
  | Mdc1-mRNA | 0.196 | 0.154 | -0.105 | 0.497 | 1.15 | 0.93 | 1.41 | 0.225 | 1 | lm.nb | Cell Cycle, DNA Damage | NS\_MM\_NEUROINFLAM\_V1.0 | Mdc1 | mRNA | 0 |  | DNA Damage;Cell Cycle |  |  | Mdc1 | azure4 | 1 | 1.27272727272727 |
  | Ung-mRNA | -0.218 | 0.175 | -0.561 | 0.125 | 0.86 | 0.678 | 1.09 | 0.237 | 1 | loglinear | DNA Damage | NS\_MM\_NEUROINFLAM\_V1.0 | Ung | mRNA | 0 |  | DNA Damage | mmu03410;mmu05340 |  | Ung | azure4 | 1 | -1.24571428571429 |
  | Pcna-mRNA | -0.058 | 0.0477 | -0.152 | 0.0356 | 0.961 | 0.9 | 1.02 | 0.248 | 1 | loglinear | Cell Cycle, DNA Damage | NS\_MM\_NEUROINFLAM\_V1.0 | Pcna | mRNA | 0 |  | DNA Damage;Cell Cycle | mmu03030;mmu03410;mmu03420;mmu03430;mmu04110;mmu05161;mmu05166 |  | Pcna | azure4 | 1 | -1.21593291404612 |
  | Bbc3-mRNA | 0.192 | 0.167 | -0.135 | 0.519 | 1.14 | 0.911 | 1.43 | 0.271 | 1 | loglinear | Apoptosis, DNA Damage | NS\_MM\_NEUROINFLAM\_V1.0 | Bbc3 | mRNA | 0 |  | DNA Damage;Apoptosis | mmu04115;mmu04390;mmu05016;mmu05162 |  | Bbc3 | azure4 | 1 | 1.1497005988024 |
  | Mgmt-mRNA | 0.541 | 0.498 | -0.435 | 1.52 | 1.45 | 0.739 | 2.86 | 0.299 | 1 | lm.nb | Cellular Stress, DNA Damage | NS\_MM\_NEUROINFLAM\_V1.0 | Mgmt | mRNA | 0 |  | DNA Damage;Cellular Stress |  |  | Mgmt | azure4 | 1 | 1.0863453815261 |

  ##### DE Results - DNA Damage genes - DPI: 1 vs.CTRL

  Table displaying each sample's global significance scores and directed global significance scores as defined in the heatmaps above. The global significance score is calculated as the square root of the mean squared t-statistic for the genes in a gene set, with t-statistics coming from the linear regression underlying our differential expression analysis. The directed global significance score is calculated as the square root of the mean signed squared t-statistic for the genes in a gene set, with t-statistics coming from the linear regression underlying our differential expression analysis.

- **Volcano Plot: DPI: 2 vs.CTRL  
  More Plot Information**

  ##### Volcano Plot: DPI: 2 vs.CTRL

  Volcano plot displaying each gene's -log10(p-value) and log2 fold change for the selected covariate. Highly statistically significant genes fall at the top of the plot, and highly differentially expressed genes fall to either side. Genes within the selected gene set are highlighted in orange. Horizontal lines indicate various False Discovery Rate (FDR) thresholds.
- **DE Results - DNA Damage genes - DPI: 2 vs.CTRL  
  More Plot Information  Download CSV Data**

  | Probe Label | Log2 fold change | std error (log2) | Lower confidence limit (log2) | Upper confidence limit (log2) | Linear fold change | Lower confidence limit (linear) | Upper confidence limit (linear) | P-value | BY.p.value | method | Gene.sets | Codeset.Name | Probe.Label | Analyte.Type | Is.Control | Control.Type | Probe.Annotation | KEGG.Pathways | Cell.Type | Official.Gene.Name | volcanocol | volcanopch | tstats.all |
  | --- | --- | --- | --- | --- | --- | --- | --- | --- | --- | --- | --- | --- | --- | --- | --- | --- | --- | --- | --- | --- | --- | --- | --- |
  | Cdkn1a-mRNA | 2.21 | 0.15 | 1.92 | 2.51 | 4.64 | 3.78 | 5.68 | 4.64e-09 | 3.71e-06 | lm.nb | Adaptive Immune Response, Cell Cycle, Cellular Stress, Cytokine Signaling, DNA Damage, Growth Factor Signaling | NS\_MM\_NEUROINFLAM\_V1.0 | Cdkn1a | mRNA | 0 |  | Growth Factor Signaling;DNA Damage;Cytokine Signaling;Cellular Stress;Cell Cycle;Adaptive Immune Response | mmu04012;mmu04066;mmu04068;mmu04110;mmu04115;mmu04151;mmu04630;mmu04921;mmu05160;mmu05161;mmu05166;mmu05169;mmu05200;mmu05202;mmu05203;mmu05205;mmu05206;mmu05214;mmu05215;mmu05218;mmu05219;mmu05220 |  | Cdkn1a | slateblue1 | 16 | 14.7333333333333 |
  | Sesn1-mRNA | 0.872 | 0.0612 | 0.752 | 0.992 | 1.83 | 1.68 | 1.99 | 6.94e-09 | 4.29e-06 | loglinear | DNA Damage | NS\_MM\_NEUROINFLAM\_V1.0 | Sesn1 | mRNA | 0 |  | DNA Damage | mmu04115;mmu04211 |  | Sesn1 | slateblue1 | 16 | 14.2483660130719 |
  | Sumo1-mRNA | -0.199 | 0.0361 | -0.269 | -0.128 | 0.871 | 0.83 | 0.915 | 0.000135 | 0.00884 | loglinear | Cell Cycle, Cytokine Signaling, DNA Damage, Inflammatory Signaling | NS\_MM\_NEUROINFLAM\_V1.0 | Sumo1 | mRNA | 0 |  | Inflammatory Signaling;DNA Damage;Cytokine Signaling;Cell Cycle |  |  | Sumo1 | slateblue1 | 16 | -5.51246537396122 |
  | Bbc3-mRNA | 0.635 | 0.167 | 0.308 | 0.961 | 1.55 | 1.24 | 1.95 | 0.00249 | 0.0848 | loglinear | Apoptosis, DNA Damage | NS\_MM\_NEUROINFLAM\_V1.0 | Bbc3 | mRNA | 0 |  | DNA Damage;Apoptosis | mmu04115;mmu04390;mmu05016;mmu05162 |  | Bbc3 | slateblue3 | 16 | 3.80239520958084 |
  | Timeless-mRNA | 0.559 | 0.194 | 0.18 | 0.938 | 1.47 | 1.13 | 1.92 | 0.0136 | 0.322 | loglinear | Cell Cycle, DNA Damage | NS\_MM\_NEUROINFLAM\_V1.0 | Timeless | mRNA | 0 |  | DNA Damage;Cell Cycle |  |  | Timeless | slateblue4 | 16 | 2.88144329896907 |
  | Fas-mRNA | 1.3 | 0.457 | 0.407 | 2.2 | 2.47 | 1.33 | 4.6 | 0.0146 | 0.342 | lm.nb | Apoptosis, Cellular Stress, Cytokine Signaling, DNA Damage, Growth Factor Signaling, Innate Immune Response | NS\_MM\_NEUROINFLAM\_V1.0 | Fas | mRNA | 0 |  | Innate Immune Response;Growth Factor Signaling;DNA Damage;Cytokine Signaling;Cellular Stress;Apoptosis | mmu04010;mmu04060;mmu04115;mmu04210;mmu04650;mmu04668;mmu04932;mmu04940;mmu05010;mmu05142;mmu05143;mmu05161;mmu05162;mmu05164;mmu05168;mmu05200;mmu05205;mmu05320;mmu05330;mmu05332 |  | Fas | slateblue4 | 16 | 2.84463894967177 |
  | Fen1-mRNA | 0.405 | 0.146 | 0.119 | 0.691 | 1.32 | 1.09 | 1.61 | 0.0168 | 0.376 | loglinear | Cell Cycle, DNA Damage | NS\_MM\_NEUROINFLAM\_V1.0 | Fen1 | mRNA | 0 |  | DNA Damage;Cell Cycle | mmu03030;mmu03410;mmu03450 |  | Fen1 | slateblue4 | 16 | 2.77397260273973 |
  | Parp1-mRNA | 0.3 | 0.118 | 0.0689 | 0.531 | 1.23 | 1.05 | 1.45 | 0.0257 | 0.511 | loglinear | Apoptosis, Cellular Stress, DNA Damage, NF-kB | NS\_MM\_NEUROINFLAM\_V1.0 | Parp1 | mRNA | 0 |  | NF-kB;DNA Damage;Cellular Stress;Apoptosis | mmu03410;mmu04064 |  | Parp1 | azure4 | 1 | 2.54237288135593 |
  | Bax-mRNA | -0.273 | 0.111 | -0.491 | -0.0561 | 0.827 | 0.712 | 0.962 | 0.0297 | 0.565 | loglinear | Apoptosis, DNA Damage | NS\_MM\_NEUROINFLAM\_V1.0 | Bax | mRNA | 0 |  | DNA Damage;Apoptosis | mmu04071;mmu04115;mmu04141;mmu04210;mmu04211;mmu04722;mmu04932;mmu04933;mmu05014;mmu05016;mmu05020;mmu05152;mmu05161;mmu05166;mmu05200;mmu05203;mmu05210 |  | Bax | azure4 | 1 | -2.45945945945946 |
  | Smarca5-mRNA | -0.112 | 0.0459 | -0.202 | -0.0221 | 0.925 | 0.869 | 0.985 | 0.0311 | 0.577 | loglinear | Cell Cycle, DNA Damage, Epigenetic Regulation | NS\_MM\_NEUROINFLAM\_V1.0 | Smarca5 | mRNA | 0 |  | Epigenetic Regulation;DNA Damage;Cell Cycle |  |  | Smarca5 | azure4 | 1 | -2.4400871459695 |
  | Pten-mRNA | 0.158 | 0.0683 | 0.0243 | 0.292 | 1.12 | 1.02 | 1.22 | 0.0391 | 0.697 | loglinear | Adaptive Immune Response, Autophagy, DNA Damage, Growth Factor Signaling, Lipid Metabolism | NS\_MM\_NEUROINFLAM\_V1.0 | Pten | mRNA | 0 |  | Lipid Metabolism;Growth Factor Signaling;DNA Damage;Autophagy;Adaptive Immune Response | mmu00562;mmu04068;mmu04070;mmu04071;mmu04115;mmu04150;mmu04151;mmu04510;mmu04530;mmu04931;mmu05161;mmu05200;mmu05206;mmu05213;mmu05214;mmu05215;mmu05218;mmu05222;mmu05230 |  | Pten | azure4 | 1 | 2.31332357247438 |
  | Gadd45g-mRNA | 0.469 | 0.214 | 0.051 | 0.888 | 1.38 | 1.04 | 1.85 | 0.0483 | 0.833 | lm.nb | Cell Cycle, DNA Damage, Growth Factor Signaling | NS\_MM\_NEUROINFLAM\_V1.0 | Gadd45g | mRNA | 0 |  | Growth Factor Signaling;DNA Damage;Cell Cycle | mmu04010;mmu04068;mmu04110;mmu04115 |  | Gadd45g | azure4 | 1 | 2.19158878504673 |
  | Cycs-mRNA | -1.05 | 0.51 | -2.04 | -0.0462 | 0.485 | 0.242 | 0.968 | 0.0628 | 1 | lm.nb | Apoptosis, Cellular Stress, DNA Damage | NS\_MM\_NEUROINFLAM\_V1.0 | Cycs | mRNA | 0 |  | DNA Damage;Cellular Stress;Apoptosis | mmu04115;mmu04210;mmu04932;mmu05010;mmu05012;mmu05014;mmu05016;mmu05134;mmu05145;mmu05152;mmu05161;mmu05164;mmu05168;mmu05200;mmu05210;mmu05222;mmu05416 |  | Cycs | azure4 | 1 | -2.05882352941176 |
  | Kdm4a-mRNA | 0.372 | 0.185 | 0.00904 | 0.736 | 1.29 | 1.01 | 1.67 | 0.0676 | 1 | lm.nb | DNA Damage, Epigenetic Regulation | NS\_MM\_NEUROINFLAM\_V1.0 | Kdm4a | mRNA | 0 |  | Epigenetic Regulation;DNA Damage |  |  | Kdm4a | azure4 | 1 | 2.01081081081081 |
  | Casp3-mRNA | 0.256 | 0.133 | -0.00405 | 0.517 | 1.19 | 0.997 | 1.43 | 0.0776 | 1 | lm.nb | Apoptosis, Cellular Stress, DNA Damage, Growth Factor Signaling, Innate Immune Response, Matrix Remodeling | NS\_MM\_NEUROINFLAM\_V1.0 | Casp3 | mRNA | 0 |  | Matrix Remodeling;Innate Immune Response;Growth Factor Signaling;DNA Damage;Cellular Stress;Apoptosis | mmu04010;mmu04115;mmu04210;mmu04650;mmu04668;mmu04726;mmu04932;mmu04933;mmu05010;mmu05012;mmu05014;mmu05016;mmu05133;mmu05134;mmu05145;mmu05146;mmu05152;mmu05161;mmu05168;mmu05200;mmu05203;mmu05205;mmu05206;mmu05210;mmu05416 |  | Casp3 | azure4 | 1 | 1.92481203007519 |
  | Rpa1-mRNA | 0.0941 | 0.0503 | -0.00438 | 0.193 | 1.07 | 0.997 | 1.14 | 0.0856 | 1 | loglinear | Cell Cycle, Cellular Stress, DNA Damage | NS\_MM\_NEUROINFLAM\_V1.0 | Rpa1 | mRNA | 0 |  | DNA Damage;Cellular Stress;Cell Cycle | mmu03030;mmu03420;mmu03430;mmu03440;mmu03460 |  | Rpa1 | azure4 | 1 | 1.87077534791253 |
  | Bcl2-mRNA | -0.462 | 0.247 | -0.947 | 0.0223 | 0.726 | 0.519 | 1.02 | 0.0861 | 1 | lm.nb | Apoptosis, Autophagy, Cellular Stress, Cytokine Signaling, DNA Damage, Growth Factor Signaling, Innate Immune Response, Microglia Function, NF-kB | NS\_MM\_NEUROINFLAM\_V1.0 | Bcl2 | mRNA | 0 |  | NF-kB;Microglia Function;Innate Immune Response;Growth Factor Signaling;DNA Damage;Cytokine Signaling;Cellular Stress;Autophagy;Apoptosis | mmu04064;mmu04066;mmu04071;mmu04141;mmu04151;mmu04210;mmu04261;mmu04510;mmu04630;mmu04722;mmu04725;mmu04933;mmu05014;mmu05145;mmu05152;mmu05161;mmu05169;mmu05200;mmu05206;mmu05210;mmu05215;mmu05222 |  | Bcl2 | azure4 | 1 | -1.87044534412955 |
  | Rad50-mRNA | -0.386 | 0.208 | -0.795 | 0.0225 | 0.765 | 0.576 | 1.02 | 0.0888 | 1 | loglinear | Cell Cycle, Cellular Stress, DNA Damage | NS\_MM\_NEUROINFLAM\_V1.0 | Rad50 | mRNA | 0 |  | DNA Damage;Cellular Stress;Cell Cycle | mmu03440;mmu03450 |  | Rad50 | azure4 | 1 | -1.85576923076923 |
  | Atm-mRNA | -0.267 | 0.151 | -0.564 | 0.0294 | 0.831 | 0.676 | 1.02 | 0.103 | 1 | lm.nb | Apoptosis, Cell Cycle, Cellular Stress, DNA Damage, NF-kB | NS\_MM\_NEUROINFLAM\_V1.0 | Atm | mRNA | 0 |  | NF-kB;DNA Damage;Cellular Stress;Cell Cycle;Apoptosis | mmu04064;mmu04068;mmu04110;mmu04115;mmu04210;mmu05166;mmu05202;mmu05206 |  | Atm | azure4 | 1 | -1.7682119205298 |
  | Trp73-mRNA | -1.05 | 0.597 | -2.22 | 0.119 | 0.483 | 0.215 | 1.09 | 0.104 | 1 | lm.nb | DNA Damage | NS\_MM\_NEUROINFLAM\_V1.0 | Trp73 | mRNA | 0 |  | DNA Damage | mmu04115;mmu04390;mmu04722;mmu05162 |  | Trp73 | azure4 | 1 | -1.75879396984925 |

  ##### DE Results - DNA Damage genes - DPI: 2 vs.CTRL

  Table displaying each sample's global significance scores and directed global significance scores as defined in the heatmaps above. The global significance score is calculated as the square root of the mean squared t-statistic for the genes in a gene set, with t-statistics coming from the linear regression underlying our differential expression analysis. The directed global significance score is calculated as the square root of the mean signed squared t-statistic for the genes in a gene set, with t-statistics coming from the linear regression underlying our differential expression analysis.

- **Volcano Plot: DPI: 3 vs.CTRL  
  More Plot Information**

  ##### Volcano Plot: DPI: 3 vs.CTRL

  Volcano plot displaying each gene's -log10(p-value) and log2 fold change for the selected covariate. Highly statistically significant genes fall at the top of the plot, and highly differentially expressed genes fall to either side. Genes within the selected gene set are highlighted in orange. Horizontal lines indicate various False Discovery Rate (FDR) thresholds.
- **DE Results - DNA Damage genes - DPI: 3 vs.CTRL  
  More Plot Information  Download CSV Data**

  | Probe Label | Log2 fold change | std error (log2) | Lower confidence limit (log2) | Upper confidence limit (log2) | Linear fold change | Lower confidence limit (linear) | Upper confidence limit (linear) | P-value | BY.p.value | method | Gene.sets | Codeset.Name | Probe.Label | Analyte.Type | Is.Control | Control.Type | Probe.Annotation | KEGG.Pathways | Cell.Type | Official.Gene.Name | volcanocol | volcanopch | tstats.all |
  | --- | --- | --- | --- | --- | --- | --- | --- | --- | --- | --- | --- | --- | --- | --- | --- | --- | --- | --- | --- | --- | --- | --- | --- |
  | Cdkn1a-mRNA | 2.23 | 0.15 | 1.93 | 2.52 | 4.68 | 3.82 | 5.74 | 4.31e-09 | 1.43e-06 | lm.nb | Adaptive Immune Response, Cell Cycle, Cellular Stress, Cytokine Signaling, DNA Damage, Growth Factor Signaling | NS\_MM\_NEUROINFLAM\_V1.0 | Cdkn1a | mRNA | 0 |  | Growth Factor Signaling;DNA Damage;Cytokine Signaling;Cellular Stress;Cell Cycle;Adaptive Immune Response | mmu04012;mmu04066;mmu04068;mmu04110;mmu04115;mmu04151;mmu04630;mmu04921;mmu05160;mmu05161;mmu05166;mmu05169;mmu05200;mmu05202;mmu05203;mmu05205;mmu05206;mmu05214;mmu05215;mmu05218;mmu05219;mmu05220 |  | Cdkn1a | slateblue1 | 16 | 14.8666666666667 |
  | Sesn1-mRNA | 0.605 | 0.0612 | 0.485 | 0.725 | 1.52 | 1.4 | 1.65 | 4.01e-07 | 4.58e-05 | loglinear | DNA Damage | NS\_MM\_NEUROINFLAM\_V1.0 | Sesn1 | mRNA | 0 |  | DNA Damage | mmu04115;mmu04211 |  | Sesn1 | slateblue1 | 16 | 9.88562091503268 |
  | Gadd45g-mRNA | 1.65 | 0.209 | 1.24 | 2.06 | 3.15 | 2.37 | 4.18 | 4.12e-06 | 0.000261 | lm.nb | Cell Cycle, DNA Damage, Growth Factor Signaling | NS\_MM\_NEUROINFLAM\_V1.0 | Gadd45g | mRNA | 0 |  | Growth Factor Signaling;DNA Damage;Cell Cycle | mmu04010;mmu04068;mmu04110;mmu04115 |  | Gadd45g | slateblue1 | 16 | 7.89473684210526 |
  | Fas-mRNA | 3.25 | 0.436 | 2.39 | 4.1 | 9.5 | 5.26 | 17.2 | 7.68e-06 | 0.00042 | lm.nb | Apoptosis, Cellular Stress, Cytokine Signaling, DNA Damage, Growth Factor Signaling, Innate Immune Response | NS\_MM\_NEUROINFLAM\_V1.0 | Fas | mRNA | 0 |  | Innate Immune Response;Growth Factor Signaling;DNA Damage;Cytokine Signaling;Cellular Stress;Apoptosis | mmu04010;mmu04060;mmu04115;mmu04210;mmu04650;mmu04668;mmu04932;mmu04940;mmu05010;mmu05142;mmu05143;mmu05161;mmu05162;mmu05164;mmu05168;mmu05200;mmu05205;mmu05320;mmu05330;mmu05332 |  | Fas | slateblue1 | 16 | 7.45412844036697 |
  | Timeless-mRNA | 1.1 | 0.194 | 0.724 | 1.48 | 2.15 | 1.65 | 2.8 | 9.89e-05 | 0.00366 | loglinear | Cell Cycle, DNA Damage | NS\_MM\_NEUROINFLAM\_V1.0 | Timeless | mRNA | 0 |  | DNA Damage;Cell Cycle |  |  | Timeless | slateblue1 | 16 | 5.67010309278351 |
  | Sumo1-mRNA | -0.196 | 0.0361 | -0.267 | -0.126 | 0.873 | 0.831 | 0.917 | 0.00015 | 0.00512 | loglinear | Cell Cycle, Cytokine Signaling, DNA Damage, Inflammatory Signaling | NS\_MM\_NEUROINFLAM\_V1.0 | Sumo1 | mRNA | 0 |  | Inflammatory Signaling;DNA Damage;Cytokine Signaling;Cell Cycle |  |  | Sumo1 | slateblue1 | 16 | -5.42936288088643 |
  | Gadd45a-mRNA | 0.534 | 0.105 | 0.329 | 0.74 | 1.45 | 1.26 | 1.67 | 0.000264 | 0.00845 | loglinear | Cell Cycle, DNA Damage, Growth Factor Signaling | NS\_MM\_NEUROINFLAM\_V1.0 | Gadd45a | mRNA | 0 |  | Growth Factor Signaling;DNA Damage;Cell Cycle | mmu04010;mmu04068;mmu04110;mmu04115 |  | Gadd45a | slateblue1 | 16 | 5.08571428571429 |
  | Bbc3-mRNA | 0.578 | 0.167 | 0.252 | 0.905 | 1.49 | 1.19 | 1.87 | 0.00462 | 0.0956 | loglinear | Apoptosis, DNA Damage | NS\_MM\_NEUROINFLAM\_V1.0 | Bbc3 | mRNA | 0 |  | DNA Damage;Apoptosis | mmu04115;mmu04390;mmu05016;mmu05162 |  | Bbc3 | slateblue3 | 16 | 3.46107784431138 |
  | Fen1-mRNA | 0.496 | 0.146 | 0.21 | 0.782 | 1.41 | 1.16 | 1.72 | 0.0053 | 0.105 | loglinear | Cell Cycle, DNA Damage | NS\_MM\_NEUROINFLAM\_V1.0 | Fen1 | mRNA | 0 |  | DNA Damage;Cell Cycle | mmu03030;mmu03410;mmu03450 |  | Fen1 | slateblue4 | 16 | 3.3972602739726 |
  | Pttg1-mRNA | 0.168 | 0.0532 | 0.064 | 0.272 | 1.12 | 1.05 | 1.21 | 0.00816 | 0.154 | loglinear | Cell Cycle, DNA Damage | NS\_MM\_NEUROINFLAM\_V1.0 | Pttg1 | mRNA | 0 |  | DNA Damage;Cell Cycle | mmu04110;mmu04114;mmu05166 |  | Pttg1 | slateblue4 | 16 | 3.15789473684211 |
  | Cdkn1c-mRNA | 0.706 | 0.225 | 0.264 | 1.15 | 1.63 | 1.2 | 2.22 | 0.00865 | 0.161 | lm.nb | Cell Cycle, DNA Damage | NS\_MM\_NEUROINFLAM\_V1.0 | Cdkn1c | mRNA | 0 |  | DNA Damage;Cell Cycle | mmu04110 |  | Cdkn1c | slateblue4 | 16 | 3.13777777777778 |
  | Trp53-mRNA | 0.573 | 0.192 | 0.197 | 0.95 | 1.49 | 1.15 | 1.93 | 0.0114 | 0.198 | lm.nb | Apoptosis, Cell Cycle, Cellular Stress, DNA Damage, Growth Factor Signaling, Notch, Wnt | NS\_MM\_NEUROINFLAM\_V1.0 | Trp53 | mRNA | 0 |  | Wnt;Notch;Growth Factor Signaling;DNA Damage;Cellular Stress;Cell Cycle;Apoptosis | mmu04010;mmu04071;mmu04110;mmu04115;mmu04151;mmu04210;mmu04211;mmu04310;mmu04722;mmu04919;mmu05014;mmu05016;mmu05160;mmu05161;mmu05162;mmu05166;mmu05168;mmu05169;mmu05200;mmu05202;mmu05203;mmu05205;mmu05206;mmu05210;mmu05212;mmu05213;mmu05214;mmu05215;mmu05216;mmu05218;mmu05219;mmu05220;mmu05222;mmu05223;mmu05230 |  | Trp53 | slateblue4 | 16 | 2.984375 |
  | Ccng2-mRNA | -0.418 | 0.155 | -0.723 | -0.114 | 0.748 | 0.606 | 0.924 | 0.0196 | 0.314 | lm.nb | Cell Cycle, DNA Damage | NS\_MM\_NEUROINFLAM\_V1.0 | Ccng2 | mRNA | 0 |  | DNA Damage;Cell Cycle | mmu04068;mmu04115 |  | Ccng2 | slateblue4 | 16 | -2.69677419354839 |
  | Rad51-mRNA | -1.58 | 0.629 | -2.82 | -0.352 | 0.333 | 0.142 | 0.784 | 0.0269 | 0.409 | lm.nb | Cell Cycle, DNA Damage | NS\_MM\_NEUROINFLAM\_V1.0 | Rad51 | mRNA | 0 |  | DNA Damage;Cell Cycle | mmu03440;mmu03460;mmu05200;mmu05212 |  | Rad51 | slateblue4 | 16 | -2.51192368839428 |
  | Ercc2-mRNA | -0.243 | 0.0963 | -0.431 | -0.0539 | 0.845 | 0.742 | 0.963 | 0.0269 | 0.409 | loglinear | DNA Damage, Inflammatory Signaling | NS\_MM\_NEUROINFLAM\_V1.0 | Ercc2 | mRNA | 0 |  | Inflammatory Signaling;DNA Damage | mmu03420 |  | Ercc2 | slateblue4 | 16 | -2.52336448598131 |
  | Ung-mRNA | -0.433 | 0.175 | -0.775 | -0.0897 | 0.741 | 0.584 | 0.94 | 0.0293 | 0.434 | loglinear | DNA Damage | NS\_MM\_NEUROINFLAM\_V1.0 | Ung | mRNA | 0 |  | DNA Damage | mmu03410;mmu05340 |  | Ung | slateblue4 | 16 | -2.47428571428571 |
  | Xrcc6-mRNA | -0.328 | 0.139 | -0.6 | -0.0553 | 0.797 | 0.66 | 0.962 | 0.0362 | 0.521 | lm.nb | DNA Damage, Innate Immune Response | NS\_MM\_NEUROINFLAM\_V1.0 | Xrcc6 | mRNA | 0 |  | Innate Immune Response;DNA Damage | mmu03450 |  | Xrcc6 | azure4 | 1 | -2.35971223021583 |
  | Casp8-mRNA | 1.32 | 0.589 | 0.167 | 2.48 | 2.5 | 1.12 | 5.57 | 0.0445 | 0.596 | lm.nb | Apoptosis, DNA Damage, Innate Immune Response, Microglia Function | NS\_MM\_NEUROINFLAM\_V1.0 | Casp8 | mRNA | 0 |  | Microglia Function;Innate Immune Response;DNA Damage;Apoptosis | mmu04115;mmu04210;mmu04620;mmu04621;mmu04622;mmu04668;mmu04932;mmu05010;mmu05016;mmu05134;mmu05142;mmu05145;mmu05152;mmu05161;mmu05168;mmu05200;mmu05203;mmu05416 |  | Casp8 | azure4 | 1 | 2.24108658743633 |
  | Rad17-mRNA | -0.362 | 0.163 | -0.682 | -0.0414 | 0.778 | 0.623 | 0.972 | 0.047 | 0.619 | loglinear | Cell Cycle, DNA Damage | NS\_MM\_NEUROINFLAM\_V1.0 | Rad17 | mRNA | 0 |  | DNA Damage;Cell Cycle |  |  | Rad17 | azure4 | 1 | -2.22085889570552 |
  | Smc1a-mRNA | 0.17 | 0.0806 | 0.0117 | 0.328 | 1.12 | 1.01 | 1.25 | 0.0571 | 0.724 | loglinear | Cell Cycle, DNA Damage | NS\_MM\_NEUROINFLAM\_V1.0 | Smc1a | mRNA | 0 |  | DNA Damage;Cell Cycle | mmu04110;mmu04114 |  | Smc1a | azure4 | 1 | 2.10918114143921 |

  ##### DE Results - DNA Damage genes - DPI: 3 vs.CTRL

  Table displaying each sample's global significance scores and directed global significance scores as defined in the heatmaps above. The global significance score is calculated as the square root of the mean squared t-statistic for the genes in a gene set, with t-statistics coming from the linear regression underlying our differential expression analysis. The directed global significance score is calculated as the square root of the mean signed squared t-statistic for the genes in a gene set, with t-statistics coming from the linear regression underlying our differential expression analysis.

DPI:
- DPI: differential expression in 1 vs. baseline of CTRL
- DPI: differential expression in 2 vs. baseline of CTRL
- DPI: differential expression in 3 vs. baseline of CTRL

- **Volcano Plot: DPI: 1 vs.CTRL  
  More Plot Information**

  ##### Volcano Plot: DPI: 1 vs.CTRL

  Volcano plot displaying each gene's -log10(p-value) and log2 fold change for the selected covariate. Highly statistically significant genes fall at the top of the plot, and highly differentially expressed genes fall to either side. Genes within the selected gene set are highlighted in orange. Horizontal lines indicate various False Discovery Rate (FDR) thresholds.
- **DE Results - Epigenetic Regulation genes - DPI: 1 vs.CTRL  
  More Plot Information  Download CSV Data**

  | Probe Label | Log2 fold change | std error (log2) | Lower confidence limit (log2) | Upper confidence limit (log2) | Linear fold change | Lower confidence limit (linear) | Upper confidence limit (linear) | P-value | BY.p.value | method | Gene.sets | Codeset.Name | Probe.Label | Analyte.Type | Is.Control | Control.Type | Probe.Annotation | KEGG.Pathways | Cell.Type | Official.Gene.Name | volcanocol | volcanopch | tstats.all |
  | --- | --- | --- | --- | --- | --- | --- | --- | --- | --- | --- | --- | --- | --- | --- | --- | --- | --- | --- | --- | --- | --- | --- | --- |
  | Kat2b-mRNA | 0.472 | 0.0862 | 0.303 | 0.641 | 1.39 | 1.23 | 1.56 | 0.000141 | 0.0959 | loglinear | Epigenetic Regulation, Notch | NS\_MM\_NEUROINFLAM\_V1.0 | Kat2b | mRNA | 0 |  | Notch;Epigenetic Regulation | mmu04330;mmu04919;mmu05166;mmu05203 |  | Kat2b | slateblue3 | 16 | 5.47563805104408 |
  | Mbd2-mRNA | 0.143 | 0.0548 | 0.0358 | 0.25 | 1.1 | 1.03 | 1.19 | 0.0227 | 1 | loglinear | Epigenetic Regulation | NS\_MM\_NEUROINFLAM\_V1.0 | Mbd2 | mRNA | 0 |  | Epigenetic Regulation |  |  | Mbd2 | azure4 | 1 | 2.60948905109489 |
  | Jarid2-mRNA | 0.204 | 0.0957 | 0.0162 | 0.391 | 1.15 | 1.01 | 1.31 | 0.0546 | 1 | loglinear | Epigenetic Regulation | NS\_MM\_NEUROINFLAM\_V1.0 | Jarid2 | mRNA | 0 |  | Epigenetic Regulation | mmu04550 |  | Jarid2 | azure4 | 1 | 2.13166144200627 |
  | Eif1-mRNA | 0.179 | 0.089 | 0.00442 | 0.353 | 1.13 | 1 | 1.28 | 0.0675 | 1 | loglinear | Epigenetic Regulation | NS\_MM\_NEUROINFLAM\_V1.0 | Eif1 | mRNA | 0 |  | Epigenetic Regulation |  |  | Eif1 | azure4 | 1 | 2.01123595505618 |
  | Ezh2-mRNA | 0.268 | 0.138 | -0.0014 | 0.538 | 1.2 | 0.999 | 1.45 | 0.075 | 1 | loglinear | Cellular Stress, Epigenetic Regulation | NS\_MM\_NEUROINFLAM\_V1.0 | Ezh2 | mRNA | 0 |  | Epigenetic Regulation;Cellular Stress | mmu05206 |  | Ezh2 | azure4 | 1 | 1.94202898550725 |
  | Hira-mRNA | -0.127 | 0.0655 | -0.256 | 0.00127 | 0.916 | 0.838 | 1 | 0.0762 | 1 | loglinear | Cellular Stress, Epigenetic Regulation | NS\_MM\_NEUROINFLAM\_V1.0 | Hira | mRNA | 0 |  | Epigenetic Regulation;Cellular Stress |  |  | Hira | azure4 | 1 | -1.93893129770992 |
  | Ash2l-mRNA | -0.101 | 0.0548 | -0.209 | 0.00632 | 0.932 | 0.865 | 1 | 0.0899 | 1 | loglinear | Epigenetic Regulation, Wnt | NS\_MM\_NEUROINFLAM\_V1.0 | Ash2l | mRNA | 0 |  | Wnt;Epigenetic Regulation |  |  | Ash2l | azure4 | 1 | -1.84306569343066 |
  | Smarca5-mRNA | -0.0842 | 0.0459 | -0.174 | 0.00583 | 0.943 | 0.886 | 1 | 0.0917 | 1 | loglinear | Cell Cycle, DNA Damage, Epigenetic Regulation | NS\_MM\_NEUROINFLAM\_V1.0 | Smarca5 | mRNA | 0 |  | Epigenetic Regulation;DNA Damage;Cell Cycle |  |  | Smarca5 | azure4 | 1 | -1.83442265795207 |
  | Suz12-mRNA | 0.162 | 0.0894 | -0.013 | 0.337 | 1.12 | 0.991 | 1.26 | 0.0946 | 1 | loglinear | Cellular Stress, Epigenetic Regulation | NS\_MM\_NEUROINFLAM\_V1.0 | Suz12 | mRNA | 0 |  | Epigenetic Regulation;Cellular Stress |  |  | Suz12 | azure4 | 1 | 1.81208053691275 |
  | Ehmt2-mRNA | -0.124 | 0.0695 | -0.26 | 0.0125 | 0.918 | 0.835 | 1.01 | 0.1 | 1 | loglinear | Cellular Stress, Epigenetic Regulation | NS\_MM\_NEUROINFLAM\_V1.0 | Ehmt2 | mRNA | 0 |  | Epigenetic Regulation;Cellular Stress | mmu00310;mmu04211 |  | Ehmt2 | azure4 | 1 | -1.7841726618705 |
  | Hdac1-mRNA | 0.25 | 0.14 | -0.0256 | 0.525 | 1.19 | 0.982 | 1.44 | 0.101 | 1 | loglinear | Cell Cycle, Epigenetic Regulation, Growth Factor Signaling, Notch, Wnt | NS\_MM\_NEUROINFLAM\_V1.0 | Hdac1 | mRNA | 0 |  | Wnt;Notch;Growth Factor Signaling;Epigenetic Regulation;Cell Cycle | mmu04110;mmu04213;mmu04330;mmu04919;mmu05016;mmu05031;mmu05034;mmu05169;mmu05200;mmu05202;mmu05203;mmu05220 |  | Hdac1 | azure4 | 1 | 1.78571428571429 |
  | Kdm4a-mRNA | 0.325 | 0.186 | -0.0383 | 0.689 | 1.25 | 0.974 | 1.61 | 0.105 | 1 | lm.nb | DNA Damage, Epigenetic Regulation | NS\_MM\_NEUROINFLAM\_V1.0 | Kdm4a | mRNA | 0 |  | Epigenetic Regulation;DNA Damage |  |  | Kdm4a | azure4 | 1 | 1.74731182795699 |
  | Dot1l-mRNA | -0.324 | 0.188 | -0.692 | 0.0449 | 0.799 | 0.619 | 1.03 | 0.111 | 1 | lm.nb | Epigenetic Regulation | NS\_MM\_NEUROINFLAM\_V1.0 | Dot1l | mRNA | 0 |  | Epigenetic Regulation | mmu00310;mmu05202 |  | Dot1l | azure4 | 1 | -1.72340425531915 |
  | Kmt2a-mRNA | -0.129 | 0.0803 | -0.287 | 0.028 | 0.914 | 0.82 | 1.02 | 0.133 | 1 | loglinear | Epigenetic Regulation | NS\_MM\_NEUROINFLAM\_V1.0 | Kmt2a | mRNA | 0 |  | Epigenetic Regulation | mmu00310;mmu05202 |  | Kmt2a | azure4 | 1 | -1.60647571606476 |
  | Hdac4-mRNA | -0.196 | 0.126 | -0.443 | 0.0505 | 0.873 | 0.735 | 1.04 | 0.145 | 1 | loglinear | Epigenetic Regulation, Notch | NS\_MM\_NEUROINFLAM\_V1.0 | Hdac4 | mRNA | 0 |  | Notch;Epigenetic Regulation | mmu05034;mmu05169;mmu05203 |  | Hdac4 | azure4 | 1 | -1.55555555555556 |
  | Kdm6a-mRNA | 0.169 | 0.109 | -0.0449 | 0.383 | 1.12 | 0.969 | 1.3 | 0.148 | 1 | loglinear | Epigenetic Regulation | NS\_MM\_NEUROINFLAM\_V1.0 | Kdm6a | mRNA | 0 |  | Epigenetic Regulation | mmu05202 |  | Kdm6a | azure4 | 1 | 1.55045871559633 |
  | Ezh1-mRNA | 0.142 | 0.092 | -0.0383 | 0.322 | 1.1 | 0.974 | 1.25 | 0.149 | 1 | loglinear | Epigenetic Regulation | NS\_MM\_NEUROINFLAM\_V1.0 | Ezh1 | mRNA | 0 |  | Epigenetic Regulation |  |  | Ezh1 | azure4 | 1 | 1.54347826086957 |
  | Ncor2-mRNA | -0.124 | 0.0816 | -0.284 | 0.0364 | 0.918 | 0.822 | 1.03 | 0.156 | 1 | loglinear | Epigenetic Regulation, Notch | NS\_MM\_NEUROINFLAM\_V1.0 | Ncor2 | mRNA | 0 |  | Notch;Epigenetic Regulation | mmu04330;mmu05169 |  | Ncor2 | azure4 | 1 | -1.51960784313725 |
  | Kdm3a-mRNA | -0.139 | 0.099 | -0.333 | 0.0555 | 0.908 | 0.794 | 1.04 | 0.187 | 1 | loglinear | Epigenetic Regulation | NS\_MM\_NEUROINFLAM\_V1.0 | Kdm3a | mRNA | 0 |  | Epigenetic Regulation |  |  | Kdm3a | azure4 | 1 | -1.4040404040404 |
  | Setd1b-mRNA | 0.203 | 0.154 | -0.0991 | 0.506 | 1.15 | 0.934 | 1.42 | 0.212 | 1 | lm.nb | Epigenetic Regulation | NS\_MM\_NEUROINFLAM\_V1.0 | Setd1b | mRNA | 0 |  | Epigenetic Regulation | mmu00310 |  | Setd1b | azure4 | 1 | 1.31818181818182 |

  ##### DE Results - Epigenetic Regulation genes - DPI: 1 vs.CTRL

  Table displaying each sample's global significance scores and directed global significance scores as defined in the heatmaps above. The global significance score is calculated as the square root of the mean squared t-statistic for the genes in a gene set, with t-statistics coming from the linear regression underlying our differential expression analysis. The directed global significance score is calculated as the square root of the mean signed squared t-statistic for the genes in a gene set, with t-statistics coming from the linear regression underlying our differential expression analysis.

- **Volcano Plot: DPI: 2 vs.CTRL  
  More Plot Information**

  ##### Volcano Plot: DPI: 2 vs.CTRL

  Volcano plot displaying each gene's -log10(p-value) and log2 fold change for the selected covariate. Highly statistically significant genes fall at the top of the plot, and highly differentially expressed genes fall to either side. Genes within the selected gene set are highlighted in orange. Horizontal lines indicate various False Discovery Rate (FDR) thresholds.
- **DE Results - Epigenetic Regulation genes - DPI: 2 vs.CTRL  
  More Plot Information  Download CSV Data**

  | Probe Label | Log2 fold change | std error (log2) | Lower confidence limit (log2) | Upper confidence limit (log2) | Linear fold change | Lower confidence limit (linear) | Upper confidence limit (linear) | P-value | BY.p.value | method | Gene.sets | Codeset.Name | Probe.Label | Analyte.Type | Is.Control | Control.Type | Probe.Annotation | KEGG.Pathways | Cell.Type | Official.Gene.Name | volcanocol | volcanopch | tstats.all |
  | --- | --- | --- | --- | --- | --- | --- | --- | --- | --- | --- | --- | --- | --- | --- | --- | --- | --- | --- | --- | --- | --- | --- | --- |
  | Ezh1-mRNA | 0.641 | 0.092 | 0.46 | 0.821 | 1.56 | 1.38 | 1.77 | 1.51e-05 | 0.00177 | loglinear | Epigenetic Regulation | NS\_MM\_NEUROINFLAM\_V1.0 | Ezh1 | mRNA | 0 |  | Epigenetic Regulation |  |  | Ezh1 | slateblue1 | 16 | 6.96739130434783 |
  | Kat2b-mRNA | 0.575 | 0.0862 | 0.406 | 0.744 | 1.49 | 1.32 | 1.67 | 2.31e-05 | 0.0024 | loglinear | Epigenetic Regulation, Notch | NS\_MM\_NEUROINFLAM\_V1.0 | Kat2b | mRNA | 0 |  | Notch;Epigenetic Regulation | mmu04330;mmu04919;mmu05166;mmu05203 |  | Kat2b | slateblue1 | 16 | 6.67053364269141 |
  | Padi2-mRNA | -1.07 | 0.235 | -1.53 | -0.606 | 0.478 | 0.347 | 0.657 | 0.000682 | 0.032 | lm.nb | Astrocyte Function, Epigenetic Regulation | NS\_MM\_NEUROINFLAM\_V1.0 | Padi2 | mRNA | 0 |  | Epigenetic Regulation;Astrocyte Function |  |  | Padi2 | slateblue2 | 16 | -4.5531914893617 |
  | Hat1-mRNA | -0.323 | 0.0739 | -0.468 | -0.178 | 0.799 | 0.723 | 0.884 | 0.000908 | 0.039 | loglinear | Epigenetic Regulation | NS\_MM\_NEUROINFLAM\_V1.0 | Hat1 | mRNA | 0 |  | Epigenetic Regulation | mmu05034 |  | Hat1 | slateblue2 | 16 | -4.37077131258457 |
  | Arid1a-mRNA | -0.244 | 0.0559 | -0.353 | -0.134 | 0.845 | 0.783 | 0.911 | 0.00094 | 0.0399 | loglinear | Epigenetic Regulation | NS\_MM\_NEUROINFLAM\_V1.0 | Arid1a | mRNA | 0 |  | Epigenetic Regulation |  |  | Arid1a | slateblue2 | 16 | -4.3649373881932 |
  | Dnmt3a-mRNA | -0.412 | 0.0978 | -0.603 | -0.22 | 0.752 | 0.658 | 0.859 | 0.00121 | 0.0495 | loglinear | Epigenetic Regulation | NS\_MM\_NEUROINFLAM\_V1.0 | Dnmt3a | mRNA | 0 |  | Epigenetic Regulation | mmu00270;mmu05206 |  | Dnmt3a | slateblue2 | 16 | -4.21267893660532 |
  | Ash2l-mRNA | -0.226 | 0.0548 | -0.333 | -0.118 | 0.855 | 0.794 | 0.921 | 0.00142 | 0.055 | loglinear | Epigenetic Regulation, Wnt | NS\_MM\_NEUROINFLAM\_V1.0 | Ash2l | mRNA | 0 |  | Wnt;Epigenetic Regulation |  |  | Ash2l | slateblue3 | 16 | -4.12408759124088 |
  | Kdm1a-mRNA | -0.105 | 0.0263 | -0.156 | -0.0532 | 0.93 | 0.897 | 0.964 | 0.00182 | 0.0671 | loglinear | Epigenetic Regulation | NS\_MM\_NEUROINFLAM\_V1.0 | Kdm1a | mRNA | 0 |  | Epigenetic Regulation |  |  | Kdm1a | slateblue3 | 16 | -3.99239543726236 |
  | Dot1l-mRNA | -0.69 | 0.193 | -1.07 | -0.312 | 0.62 | 0.477 | 0.805 | 0.00379 | 0.116 | lm.nb | Epigenetic Regulation | NS\_MM\_NEUROINFLAM\_V1.0 | Dot1l | mRNA | 0 |  | Epigenetic Regulation | mmu00310;mmu05202 |  | Dot1l | slateblue4 | 16 | -3.57512953367876 |
  | Ncor2-mRNA | -0.289 | 0.0816 | -0.449 | -0.129 | 0.819 | 0.733 | 0.915 | 0.0041 | 0.123 | loglinear | Epigenetic Regulation, Notch | NS\_MM\_NEUROINFLAM\_V1.0 | Ncor2 | mRNA | 0 |  | Notch;Epigenetic Regulation | mmu04330;mmu05169 |  | Ncor2 | slateblue4 | 16 | -3.54166666666667 |
  | Hdac2-mRNA | -0.239 | 0.0775 | -0.391 | -0.0871 | 0.847 | 0.763 | 0.941 | 0.00946 | 0.24 | loglinear | Cell Cycle, Cellular Stress, Epigenetic Regulation, Growth Factor Signaling, Notch | NS\_MM\_NEUROINFLAM\_V1.0 | Hdac2 | mRNA | 0 |  | Notch;Growth Factor Signaling;Epigenetic Regulation;Cellular Stress;Cell Cycle | mmu04110;mmu04213;mmu04330;mmu04919;mmu05016;mmu05034;mmu05169;mmu05200;mmu05202;mmu05203;mmu05220 |  | Hdac2 | slateblue4 | 16 | -3.08387096774194 |
  | Kat2a-mRNA | -0.386 | 0.128 | -0.637 | -0.136 | 0.765 | 0.643 | 0.91 | 0.0106 | 0.262 | loglinear | Epigenetic Regulation, Notch | NS\_MM\_NEUROINFLAM\_V1.0 | Kat2a | mRNA | 0 |  | Notch;Epigenetic Regulation | mmu04330;mmu04919;mmu05166;mmu05203 |  | Kat2a | slateblue4 | 16 | -3.015625 |
  | Hira-mRNA | -0.195 | 0.0655 | -0.323 | -0.0662 | 0.874 | 0.799 | 0.955 | 0.0117 | 0.284 | loglinear | Cellular Stress, Epigenetic Regulation | NS\_MM\_NEUROINFLAM\_V1.0 | Hira | mRNA | 0 |  | Epigenetic Regulation;Cellular Stress |  |  | Hira | slateblue4 | 16 | -2.97709923664122 |
  | Sall1-mRNA | -0.609 | 0.22 | -1.04 | -0.179 | 0.655 | 0.486 | 0.883 | 0.0168 | 0.376 | lm.nb | Epigenetic Regulation | NS\_MM\_NEUROINFLAM\_V1.0 | Sall1 | mRNA | 0 |  | Epigenetic Regulation |  |  | Sall1 | slateblue4 | 16 | -2.76818181818182 |
  | Kdm4d-mRNA | -1.69 | 0.615 | -2.9 | -0.487 | 0.31 | 0.134 | 0.714 | 0.0175 | 0.388 | lm.nb | Epigenetic Regulation | NS\_MM\_NEUROINFLAM\_V1.0 | Kdm4d | mRNA | 0 |  | Epigenetic Regulation |  |  | Kdm4d | slateblue4 | 16 | -2.7479674796748 |
  | Suv39h1-mRNA | 0.188 | 0.0696 | 0.052 | 0.325 | 1.14 | 1.04 | 1.25 | 0.019 | 0.414 | loglinear | Epigenetic Regulation | NS\_MM\_NEUROINFLAM\_V1.0 | Suv39h1 | mRNA | 0 |  | Epigenetic Regulation | mmu00310 |  | Suv39h1 | slateblue4 | 16 | 2.70114942528736 |
  | Mbd2-mRNA | 0.142 | 0.0548 | 0.0346 | 0.249 | 1.1 | 1.02 | 1.19 | 0.0236 | 0.489 | loglinear | Epigenetic Regulation | NS\_MM\_NEUROINFLAM\_V1.0 | Mbd2 | mRNA | 0 |  | Epigenetic Regulation |  |  | Mbd2 | slateblue4 | 16 | 2.59124087591241 |
  | Smarca5-mRNA | -0.112 | 0.0459 | -0.202 | -0.0221 | 0.925 | 0.869 | 0.985 | 0.0311 | 0.577 | loglinear | Cell Cycle, DNA Damage, Epigenetic Regulation | NS\_MM\_NEUROINFLAM\_V1.0 | Smarca5 | mRNA | 0 |  | Epigenetic Regulation;DNA Damage;Cell Cycle |  |  | Smarca5 | azure4 | 1 | -2.4400871459695 |
  | Brd4-mRNA | -0.13 | 0.059 | -0.245 | -0.0141 | 0.914 | 0.844 | 0.99 | 0.0483 | 0.833 | loglinear | Epigenetic Regulation | NS\_MM\_NEUROINFLAM\_V1.0 | Brd4 | mRNA | 0 |  | Epigenetic Regulation |  |  | Brd4 | azure4 | 1 | -2.20338983050847 |
  | Kdm5a-mRNA | 0.173 | 0.0792 | 0.0181 | 0.329 | 1.13 | 1.01 | 1.26 | 0.0492 | 0.839 | loglinear | Epigenetic Regulation | NS\_MM\_NEUROINFLAM\_V1.0 | Kdm5a | mRNA | 0 |  | Epigenetic Regulation |  |  | Kdm5a | azure4 | 1 | 2.18434343434343 |

  ##### DE Results - Epigenetic Regulation genes - DPI: 2 vs.CTRL

  Table displaying each sample's global significance scores and directed global significance scores as defined in the heatmaps above. The global significance score is calculated as the square root of the mean squared t-statistic for the genes in a gene set, with t-statistics coming from the linear regression underlying our differential expression analysis. The directed global significance score is calculated as the square root of the mean signed squared t-statistic for the genes in a gene set, with t-statistics coming from the linear regression underlying our differential expression analysis.

- **Volcano Plot: DPI: 3 vs.CTRL  
  More Plot Information**

  ##### Volcano Plot: DPI: 3 vs.CTRL

  Volcano plot displaying each gene's -log10(p-value) and log2 fold change for the selected covariate. Highly statistically significant genes fall at the top of the plot, and highly differentially expressed genes fall to either side. Genes within the selected gene set are highlighted in orange. Horizontal lines indicate various False Discovery Rate (FDR) thresholds.
- **DE Results - Epigenetic Regulation genes - DPI: 3 vs.CTRL  
  More Plot Information  Download CSV Data**

  | Probe Label | Log2 fold change | std error (log2) | Lower confidence limit (log2) | Upper confidence limit (log2) | Linear fold change | Lower confidence limit (linear) | Upper confidence limit (linear) | P-value | BY.p.value | method | Gene.sets | Codeset.Name | Probe.Label | Analyte.Type | Is.Control | Control.Type | Probe.Annotation | KEGG.Pathways | Cell.Type | Official.Gene.Name | volcanocol | volcanopch | tstats.all |
  | --- | --- | --- | --- | --- | --- | --- | --- | --- | --- | --- | --- | --- | --- | --- | --- | --- | --- | --- | --- | --- | --- | --- | --- |
  | Kdm1a-mRNA | -0.222 | 0.0263 | -0.274 | -0.171 | 0.857 | 0.827 | 0.889 | 2.16e-06 | 0.000149 | loglinear | Epigenetic Regulation | NS\_MM\_NEUROINFLAM\_V1.0 | Kdm1a | mRNA | 0 |  | Epigenetic Regulation |  |  | Kdm1a | slateblue1 | 16 | -8.44106463878327 |
  | Ezh1-mRNA | 0.582 | 0.092 | 0.401 | 0.762 | 1.5 | 1.32 | 1.7 | 3.81e-05 | 0.00173 | loglinear | Epigenetic Regulation | NS\_MM\_NEUROINFLAM\_V1.0 | Ezh1 | mRNA | 0 |  | Epigenetic Regulation |  |  | Ezh1 | slateblue1 | 16 | 6.32608695652174 |
  | Mbd2-mRNA | 0.324 | 0.0548 | 0.217 | 0.432 | 1.25 | 1.16 | 1.35 | 7e-05 | 0.00285 | loglinear | Epigenetic Regulation | NS\_MM\_NEUROINFLAM\_V1.0 | Mbd2 | mRNA | 0 |  | Epigenetic Regulation |  |  | Mbd2 | slateblue1 | 16 | 5.91240875912409 |
  | Sall1-mRNA | -0.928 | 0.221 | -1.36 | -0.494 | 0.525 | 0.389 | 0.71 | 0.00125 | 0.031 | lm.nb | Epigenetic Regulation | NS\_MM\_NEUROINFLAM\_V1.0 | Sall1 | mRNA | 0 |  | Epigenetic Regulation |  |  | Sall1 | slateblue2 | 16 | -4.19909502262443 |
  | Dnmt3a-mRNA | -0.385 | 0.0978 | -0.577 | -0.194 | 0.766 | 0.67 | 0.874 | 0.00196 | 0.0469 | loglinear | Epigenetic Regulation | NS\_MM\_NEUROINFLAM\_V1.0 | Dnmt3a | mRNA | 0 |  | Epigenetic Regulation | mmu00270;mmu05206 |  | Dnmt3a | slateblue2 | 16 | -3.93660531697342 |
  | Kdm2b-mRNA | -0.632 | 0.176 | -0.977 | -0.288 | 0.645 | 0.508 | 0.819 | 0.00366 | 0.0794 | lm.nb | Epigenetic Regulation | NS\_MM\_NEUROINFLAM\_V1.0 | Kdm2b | mRNA | 0 |  | Epigenetic Regulation |  |  | Kdm2b | slateblue3 | 16 | -3.59090909090909 |
  | Dicer1-mRNA | -0.233 | 0.0654 | -0.361 | -0.105 | 0.851 | 0.779 | 0.93 | 0.00388 | 0.0828 | loglinear | Epigenetic Regulation | NS\_MM\_NEUROINFLAM\_V1.0 | Dicer1 | mRNA | 0 |  | Epigenetic Regulation | mmu05206 |  | Dicer1 | slateblue3 | 16 | -3.56269113149847 |
  | Smarcd1-mRNA | -0.536 | 0.151 | -0.832 | -0.239 | 0.69 | 0.562 | 0.847 | 0.00409 | 0.0864 | lm.nb | Epigenetic Regulation | NS\_MM\_NEUROINFLAM\_V1.0 | Smarcd1 | mRNA | 0 |  | Epigenetic Regulation |  |  | Smarcd1 | slateblue3 | 16 | -3.54966887417219 |
  | Hira-mRNA | -0.23 | 0.0655 | -0.359 | -0.102 | 0.853 | 0.78 | 0.932 | 0.00429 | 0.0896 | loglinear | Cellular Stress, Epigenetic Regulation | NS\_MM\_NEUROINFLAM\_V1.0 | Hira | mRNA | 0 |  | Epigenetic Regulation;Cellular Stress |  |  | Hira | slateblue3 | 16 | -3.51145038167939 |
  | Hdac2-mRNA | -0.267 | 0.0775 | -0.419 | -0.115 | 0.831 | 0.748 | 0.923 | 0.00484 | 0.0986 | loglinear | Cell Cycle, Cellular Stress, Epigenetic Regulation, Growth Factor Signaling, Notch | NS\_MM\_NEUROINFLAM\_V1.0 | Hdac2 | mRNA | 0 |  | Notch;Growth Factor Signaling;Epigenetic Regulation;Cellular Stress;Cell Cycle | mmu04110;mmu04213;mmu04330;mmu04919;mmu05016;mmu05034;mmu05169;mmu05200;mmu05202;mmu05203;mmu05220 |  | Hdac2 | slateblue3 | 16 | -3.44516129032258 |
  | Suv39h2-mRNA | -0.719 | 0.212 | -1.14 | -0.303 | 0.607 | 0.455 | 0.81 | 0.00537 | 0.106 | loglinear | Epigenetic Regulation | NS\_MM\_NEUROINFLAM\_V1.0 | Suv39h2 | mRNA | 0 |  | Epigenetic Regulation | mmu00310 |  | Suv39h2 | slateblue4 | 16 | -3.39150943396226 |
  | Dot1l-mRNA | -0.581 | 0.191 | -0.956 | -0.207 | 0.668 | 0.515 | 0.867 | 0.0103 | 0.186 | lm.nb | Epigenetic Regulation | NS\_MM\_NEUROINFLAM\_V1.0 | Dot1l | mRNA | 0 |  | Epigenetic Regulation | mmu00310;mmu05202 |  | Dot1l | slateblue4 | 16 | -3.04188481675393 |
  | Ash2l-mRNA | -0.165 | 0.0548 | -0.273 | -0.0578 | 0.892 | 0.828 | 0.961 | 0.0108 | 0.191 | loglinear | Epigenetic Regulation, Wnt | NS\_MM\_NEUROINFLAM\_V1.0 | Ash2l | mRNA | 0 |  | Wnt;Epigenetic Regulation |  |  | Ash2l | slateblue4 | 16 | -3.01094890510949 |
  | Padi2-mRNA | -0.661 | 0.233 | -1.12 | -0.204 | 0.632 | 0.461 | 0.868 | 0.015 | 0.251 | lm.nb | Astrocyte Function, Epigenetic Regulation | NS\_MM\_NEUROINFLAM\_V1.0 | Padi2 | mRNA | 0 |  | Epigenetic Regulation;Astrocyte Function |  |  | Padi2 | slateblue4 | 16 | -2.83690987124464 |
  | Tet1-mRNA | -0.358 | 0.129 | -0.61 | -0.106 | 0.78 | 0.655 | 0.929 | 0.0166 | 0.273 | lm.nb | Epigenetic Regulation | NS\_MM\_NEUROINFLAM\_V1.0 | Tet1 | mRNA | 0 |  | Epigenetic Regulation |  |  | Tet1 | slateblue4 | 16 | -2.77519379844961 |
  | Smarca4-mRNA | -0.203 | 0.0783 | -0.357 | -0.05 | 0.868 | 0.781 | 0.966 | 0.0233 | 0.364 | loglinear | Epigenetic Regulation, Wnt | NS\_MM\_NEUROINFLAM\_V1.0 | Smarca4 | mRNA | 0 |  | Wnt;Epigenetic Regulation |  |  | Smarca4 | slateblue4 | 16 | -2.59259259259259 |
  | Hat1-mRNA | -0.185 | 0.0739 | -0.33 | -0.0407 | 0.879 | 0.795 | 0.972 | 0.0274 | 0.413 | loglinear | Epigenetic Regulation | NS\_MM\_NEUROINFLAM\_V1.0 | Hat1 | mRNA | 0 |  | Epigenetic Regulation | mmu05034 |  | Hat1 | slateblue4 | 16 | -2.50338294993234 |
  | Kdm5c-mRNA | -0.337 | 0.138 | -0.607 | -0.0662 | 0.792 | 0.656 | 0.955 | 0.0312 | 0.458 | lm.nb | Epigenetic Regulation | NS\_MM\_NEUROINFLAM\_V1.0 | Kdm5c | mRNA | 0 |  | Epigenetic Regulation |  |  | Kdm5c | slateblue4 | 16 | -2.44202898550725 |
  | Brd2-mRNA | 0.191 | 0.0789 | 0.0367 | 0.346 | 1.14 | 1.03 | 1.27 | 0.032 | 0.468 | loglinear | Epigenetic Regulation | NS\_MM\_NEUROINFLAM\_V1.0 | Brd2 | mRNA | 0 |  | Epigenetic Regulation |  |  | Brd2 | slateblue4 | 16 | 2.42078580481622 |
  | Ehmt2-mRNA | -0.166 | 0.0695 | -0.302 | -0.0298 | 0.891 | 0.811 | 0.98 | 0.0342 | 0.497 | loglinear | Cellular Stress, Epigenetic Regulation | NS\_MM\_NEUROINFLAM\_V1.0 | Ehmt2 | mRNA | 0 |  | Epigenetic Regulation;Cellular Stress | mmu00310;mmu04211 |  | Ehmt2 | azure4 | 1 | -2.38848920863309 |

  ##### DE Results - Epigenetic Regulation genes - DPI: 3 vs.CTRL

  Table displaying each sample's global significance scores and directed global significance scores as defined in the heatmaps above. The global significance score is calculated as the square root of the mean squared t-statistic for the genes in a gene set, with t-statistics coming from the linear regression underlying our differential expression analysis. The directed global significance score is calculated as the square root of the mean signed squared t-statistic for the genes in a gene set, with t-statistics coming from the linear regression underlying our differential expression analysis.

DPI:
- DPI: differential expression in 1 vs. baseline of CTRL
- DPI: differential expression in 2 vs. baseline of CTRL
- DPI: differential expression in 3 vs. baseline of CTRL

- **Volcano Plot: DPI: 1 vs.CTRL  
  More Plot Information**

  ##### Volcano Plot: DPI: 1 vs.CTRL

  Volcano plot displaying each gene's -log10(p-value) and log2 fold change for the selected covariate. Highly statistically significant genes fall at the top of the plot, and highly differentially expressed genes fall to either side. Genes within the selected gene set are highlighted in orange. Horizontal lines indicate various False Discovery Rate (FDR) thresholds.
- **DE Results - Growth Factor Signaling genes - DPI: 1 vs.CTRL  
  More Plot Information  Download CSV Data**

  | Probe Label | Log2 fold change | std error (log2) | Lower confidence limit (log2) | Upper confidence limit (log2) | Linear fold change | Lower confidence limit (linear) | Upper confidence limit (linear) | P-value | BY.p.value | method | Gene.sets | Codeset.Name | Probe.Label | Analyte.Type | Is.Control | Control.Type | Probe.Annotation | KEGG.Pathways | Cell.Type | Official.Gene.Name | volcanocol | volcanopch | tstats.all |
  | --- | --- | --- | --- | --- | --- | --- | --- | --- | --- | --- | --- | --- | --- | --- | --- | --- | --- | --- | --- | --- | --- | --- | --- |
  | Osmr-mRNA | 1.68 | 0.337 | 1.02 | 2.34 | 3.21 | 2.03 | 5.07 | 0.000313 | 0.125 | lm.nb | Astrocyte Function, Cytokine Signaling, Growth Factor Signaling | NS\_MM\_NEUROINFLAM\_V1.0 | Osmr | mRNA | 0 |  | Growth Factor Signaling;Cytokine Signaling;Astrocyte Function | mmu04060;mmu04151;mmu04630 |  | Osmr | slateblue4 | 16 | 4.98516320474777 |
  | Ralb-mRNA | 0.207 | 0.047 | 0.115 | 0.299 | 1.15 | 1.08 | 1.23 | 0.000866 | 0.314 | loglinear | Autophagy, Growth Factor Signaling | NS\_MM\_NEUROINFLAM\_V1.0 | Ralb | mRNA | 0 |  | Growth Factor Signaling;Autophagy | mmu04014;mmu04015;mmu04072;mmu05200;mmu05212 |  | Ralb | slateblue4 | 16 | 4.40425531914894 |
  | Lingo1-mRNA | -0.371 | 0.0936 | -0.555 | -0.188 | 0.773 | 0.681 | 0.878 | 0.00187 | 0.437 | loglinear | Growth Factor Signaling, Oligodendrocyte Function | NS\_MM\_NEUROINFLAM\_V1.0 | Lingo1 | mRNA | 0 |  | Oligodendrocyte Function;Growth Factor Signaling |  |  | Lingo1 | slateblue4 | 16 | -3.96367521367521 |
  | Cd14-mRNA | 2.57 | 0.657 | 1.28 | 3.85 | 5.92 | 2.43 | 14.5 | 0.00208 | 0.437 | lm.nb | Apoptosis, Astrocyte Function, Autophagy, Growth Factor Signaling, Innate Immune Response, NF-kB | NS\_MM\_NEUROINFLAM\_V1.0 | Cd14 | mRNA | 0 |  | NF-kB;Innate Immune Response;Growth Factor Signaling;Autophagy;Astrocyte Function;Apoptosis | mmu04010;mmu04064;mmu04145;mmu04620;mmu04640;mmu04810;mmu05132;mmu05133;mmu05134;mmu05146;mmu05152;mmu05202 |  | Cd14 | slateblue4 | 16 | 3.9117199391172 |
  | Ets2-mRNA | 0.213 | 0.0545 | 0.106 | 0.319 | 1.16 | 1.08 | 1.25 | 0.00211 | 0.437 | loglinear | Cellular Stress, Growth Factor Signaling, Microglia Function | NS\_MM\_NEUROINFLAM\_V1.0 | Ets2 | mRNA | 0 |  | Microglia Function;Growth Factor Signaling;Cellular Stress | mmu04014;mmu05166 |  | Ets2 | slateblue4 | 16 | 3.90825688073395 |
  | Nfkbia-mRNA | 0.866 | 0.242 | 0.392 | 1.34 | 1.82 | 1.31 | 2.53 | 0.00379 | 0.558 | lm.nb | Adaptive Immune Response, Apoptosis, Growth Factor Signaling, Inflammatory Signaling, Innate Immune Response, NF-kB | NS\_MM\_NEUROINFLAM\_V1.0 | Nfkbia | mRNA | 0 |  | NF-kB;Innate Immune Response;Inflammatory Signaling;Growth Factor Signaling;Apoptosis;Adaptive Immune Response | mmu04024;mmu04062;mmu04064;mmu04210;mmu04380;mmu04620;mmu04621;mmu04622;mmu04623;mmu04660;mmu04662;mmu04668;mmu04722;mmu04920;mmu04931;mmu05134;mmu05140;mmu05142;mmu05145;mmu05160;mmu05161;mmu05162;mmu05164;mmu05166;mmu05168;mmu05169;mmu05200;mmu05203;mmu05215;mmu05220;mmu05222 |  | Nfkbia | azure4 | 1 | 3.57851239669422 |
  | Braf-mRNA | 0.0984 | 0.0282 | 0.0432 | 0.154 | 1.07 | 1.03 | 1.11 | 0.00445 | 0.577 | loglinear | Adaptive Immune Response, Angiogenesis, Apoptosis, Cytokine Signaling, Growth Factor Signaling, Innate Immune Response, Insulin Signaling, Neurons and Neurotransmission | NS\_MM\_NEUROINFLAM\_V1.0 | Braf | mRNA | 0 |  | Neurons and Neurotransmission;Insulin Signaling;Innate Immune Response;Growth Factor Signaling;Cytokine Signaling;Apoptosis;Angiogenesis;Adaptive Immune Response | mmu04010;mmu04012;mmu04015;mmu04024;mmu04062;mmu04068;mmu04150;mmu04270;mmu04510;mmu04650;mmu04720;mmu04722;mmu04726;mmu04730;mmu04810;mmu04910;mmu04914;mmu05034;mmu05160;mmu05200;mmu05205;mmu05210;mmu05211;mmu05212;mmu05213;mmu05214;mmu05215;mmu05216;mmu05218;mmu05219;mmu05220;mmu05221;mmu05223 |  | Braf | azure4 | 1 | 3.48936170212766 |
  | Il1r1-mRNA | 0.625 | 0.181 | 0.27 | 0.98 | 1.54 | 1.21 | 1.97 | 0.00477 | 0.577 | lm.nb | Apoptosis, Cellular Stress, Cytokine Signaling, Growth Factor Signaling, Microglia Function, NF-kB | NS\_MM\_NEUROINFLAM\_V1.0 | Il1r1 | mRNA | 0 |  | NF-kB;Microglia Function;Growth Factor Signaling;Cytokine Signaling;Cellular Stress;Apoptosis | mmu04010;mmu04060;mmu04064;mmu04210;mmu04380;mmu04640;mmu04750;mmu05146;mmu05166 |  | Il1r1 | azure4 | 1 | 3.45303867403315 |
  | Jun-mRNA | 0.323 | 0.0979 | 0.131 | 0.515 | 1.25 | 1.09 | 1.43 | 0.0064 | 0.711 | loglinear | Adaptive Immune Response, Apoptosis, Cellular Stress, Growth Factor Signaling, Innate Immune Response, Notch, Wnt | NS\_MM\_NEUROINFLAM\_V1.0 | Jun | mRNA | 0 |  | Wnt;Notch;Innate Immune Response;Growth Factor Signaling;Cellular Stress;Apoptosis;Adaptive Immune Response | mmu04010;mmu04012;mmu04024;mmu04310;mmu04380;mmu04510;mmu04620;mmu04660;mmu04662;mmu04668;mmu04722;mmu04912;mmu04915;mmu04921;mmu04932;mmu04933;mmu05030;mmu05031;mmu05132;mmu05133;mmu05140;mmu05142;mmu05161;mmu05164;mmu05166;mmu05168;mmu05169;mmu05200;mmu05203;mmu05210;mmu05211;mmu05231;mmu05321;mmu05323 |  | Jun | azure4 | 1 | 3.29928498467824 |
  | Csf1r-mRNA | -0.635 | 0.198 | -1.02 | -0.248 | 0.644 | 0.492 | 0.842 | 0.00748 | 0.76 | lm.nb | Cytokine Signaling, Growth Factor Signaling, Microglia Function | NS\_MM\_NEUROINFLAM\_V1.0 | Csf1r | mRNA | 0 |  | Microglia Function;Growth Factor Signaling;Cytokine Signaling | mmu04014;mmu04015;mmu04060;mmu04144;mmu04151;mmu04380;mmu04640;mmu05200;mmu05202 |  | Csf1r | azure4 | 1 | -3.20707070707071 |
[truncated: 434,413 more chars]
