# Supplementary figures and images for: Vascular-associated bacterial burden and neuroinflammatory transcriptional responses observed in models of pneumonic plague
Source: Front Microbiol. 2026 Jun 24;17:1865125. doi: 10.3389/fmicb.2026.1865125 (PMC13341613; doi:10.3389/fmicb.2026.1865125)

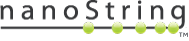

Supplement: Supplementary file 1 [file Data_Sheet_1.ZIP › Bp_final 2026-04-14 12-30/resources/img/logo_nanostring.png]

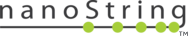

Supplement: Supplementary file 1 [file Data_Sheet_1.ZIP › Bp_final 2026-04-14 12-30/resources/img/logo_nanostring_Flat_189x40.png]

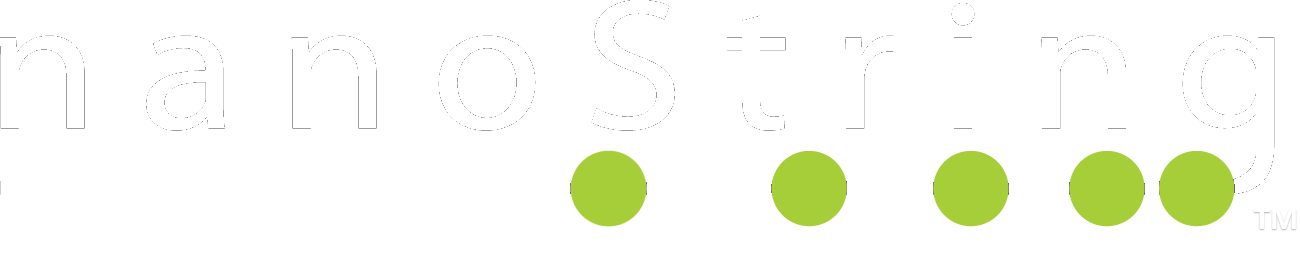

Supplement: Supplementary file 1 [file Data_Sheet_1.ZIP › Bp_final 2026-04-14 12-30/resources/img/logo_nanostring_white_Flat.png]

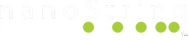

Supplement: Supplementary file 1 [file Data_Sheet_1.ZIP › Bp_final 2026-04-14 12-30/resources/img/logo_nanostring_white_Flat_189x40.png]

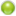

Supplement: Supplementary file 1 [file Data_Sheet_1.ZIP › Bp_final 2026-04-14 12-30/resources/img/nanostring_icon.png]

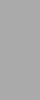

Supplement: Supplementary file 1 [file Data_Sheet_1.ZIP › Bp_final 2026-04-14 12-30/resources/img/ui-bg_flat_0_aaaaaa_40x100.png]

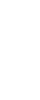

Supplement: Supplementary file 1 [file Data_Sheet_1.ZIP › Bp_final 2026-04-14 12-30/resources/img/ui-bg_flat_75_ffffff_40x100.png]

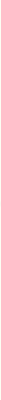

Supplement: Supplementary file 1 [file Data_Sheet_1.ZIP › Bp_final 2026-04-14 12-30/resources/img/ui-bg_glass_55_fbf9ee_1x400.png]

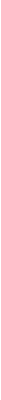

Supplement: Supplementary file 1 [file Data_Sheet_1.ZIP › Bp_final 2026-04-14 12-30/resources/img/ui-bg_glass_65_ffffff_1x400.png]

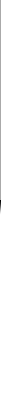

Supplement: Supplementary file 1 [file Data_Sheet_1.ZIP › Bp_final 2026-04-14 12-30/resources/img/ui-bg_glass_75_dadada_1x400.png]

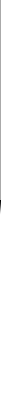

Supplement: Supplementary file 1 [file Data_Sheet_1.ZIP › Bp_final 2026-04-14 12-30/resources/img/ui-bg_glass_75_e6e6e6_1x400.png]

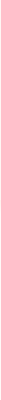

Supplement: Supplementary file 1 [file Data_Sheet_1.ZIP › Bp_final 2026-04-14 12-30/resources/img/ui-bg_glass_95_fef1ec_1x400.png]

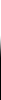

Supplement: Supplementary file 1 [file Data_Sheet_1.ZIP › Bp_final 2026-04-14 12-30/resources/img/ui-bg_highlight-soft_75_cccccc_1x100.png]

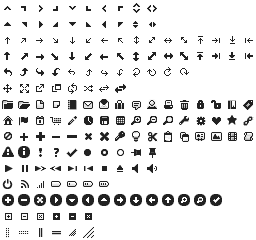

Supplement: Supplementary file 1 [file Data_Sheet_1.ZIP › Bp_final 2026-04-14 12-30/resources/img/ui-icons_222222_256x240.png]

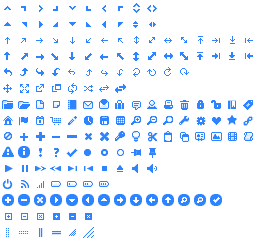

Supplement: Supplementary file 1 [file Data_Sheet_1.ZIP › Bp_final 2026-04-14 12-30/resources/img/ui-icons_2e83ff_256x240.png]

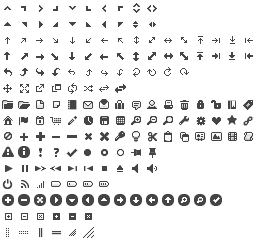

Supplement: Supplementary file 1 [file Data_Sheet_1.ZIP › Bp_final 2026-04-14 12-30/resources/img/ui-icons_454545_256x240.png]

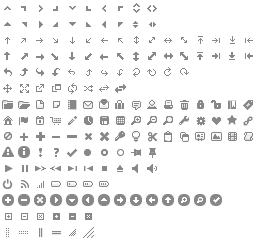

Supplement: Supplementary file 1 [file Data_Sheet_1.ZIP › Bp_final 2026-04-14 12-30/resources/img/ui-icons_888888_256x240.png]

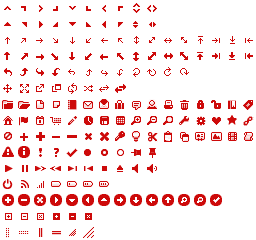

Supplement: Supplementary file 1 [file Data_Sheet_1.ZIP › Bp_final 2026-04-14 12-30/resources/img/ui-icons_cd0a0a_256x240.png]

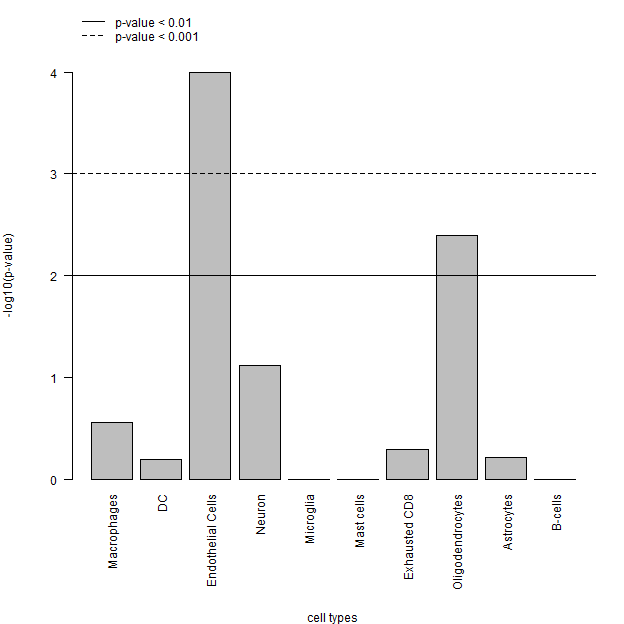

Supplement: Supplementary file 1 [file Data_Sheet_1.ZIP › Bp_final 2026-04-14 12-30/results/cell types/barplot of p-values of cell types.png]

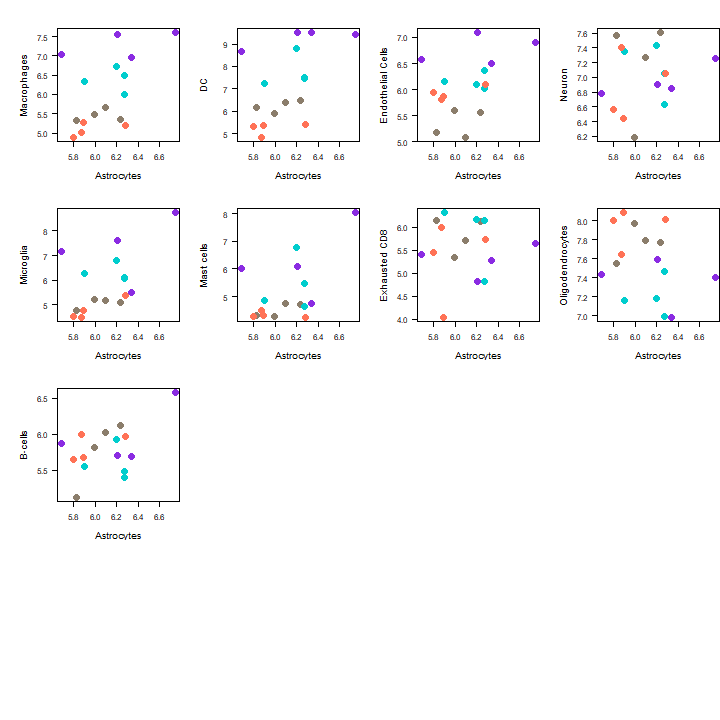

Supplement: Supplementary file 1 [file Data_Sheet_1.ZIP › Bp_final 2026-04-14 12-30/results/cell types/cell scores pairs plot - raw - Astrocytes - colored by DPI.png]

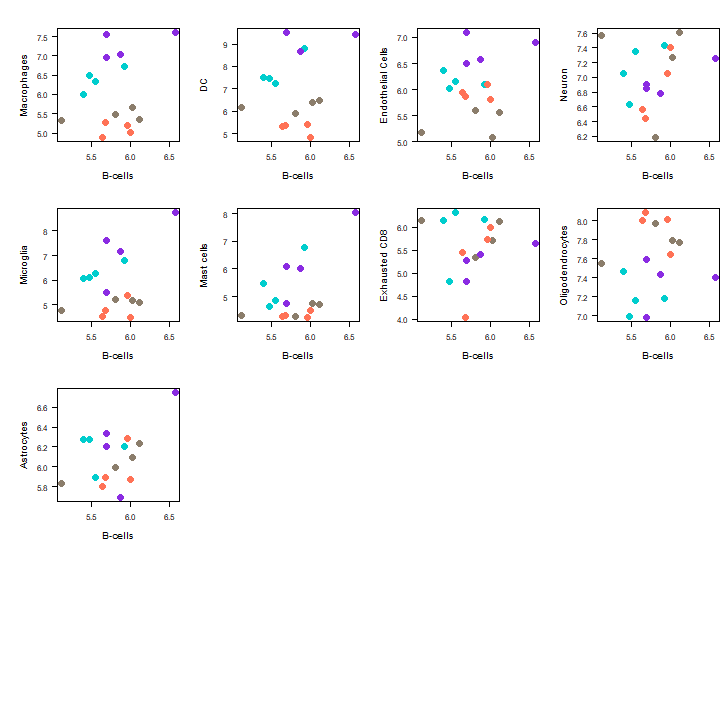

Supplement: Supplementary file 1 [file Data_Sheet_1.ZIP › Bp_final 2026-04-14 12-30/results/cell types/cell scores pairs plot - raw - B-cells - colored by DPI.png]

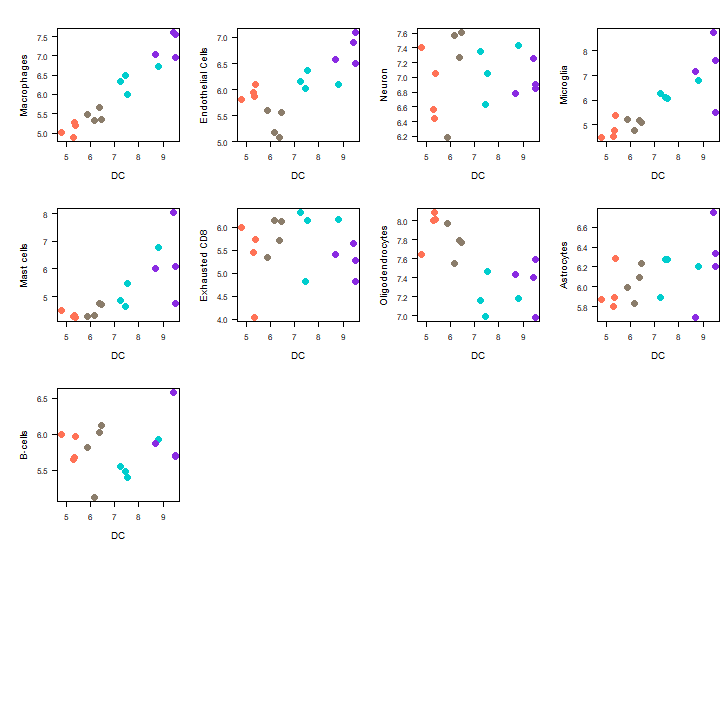

Supplement: Supplementary file 1 [file Data_Sheet_1.ZIP › Bp_final 2026-04-14 12-30/results/cell types/cell scores pairs plot - raw - DC - colored by DPI.png]

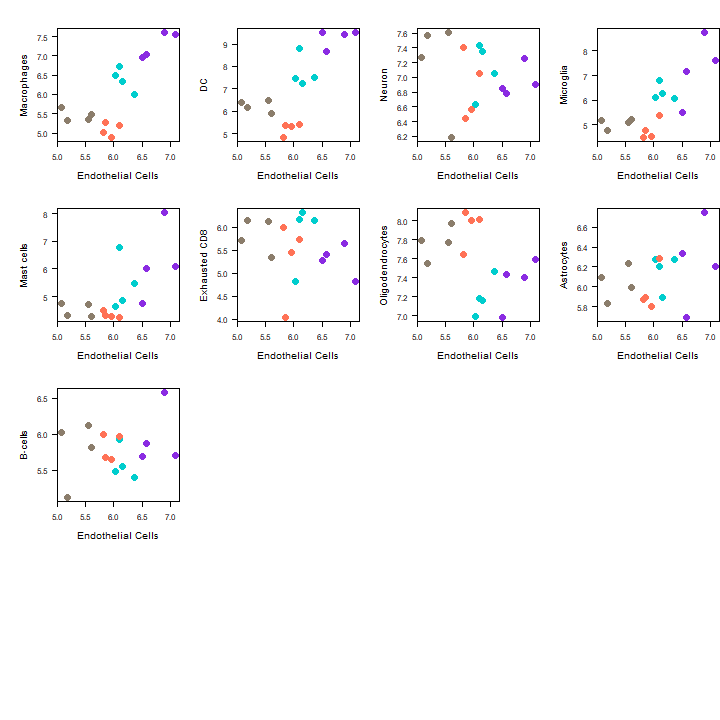

Supplement: Supplementary file 1 [file Data_Sheet_1.ZIP › Bp_final 2026-04-14 12-30/results/cell types/cell scores pairs plot - raw - Endothelial Cells - colored by DPI.png]

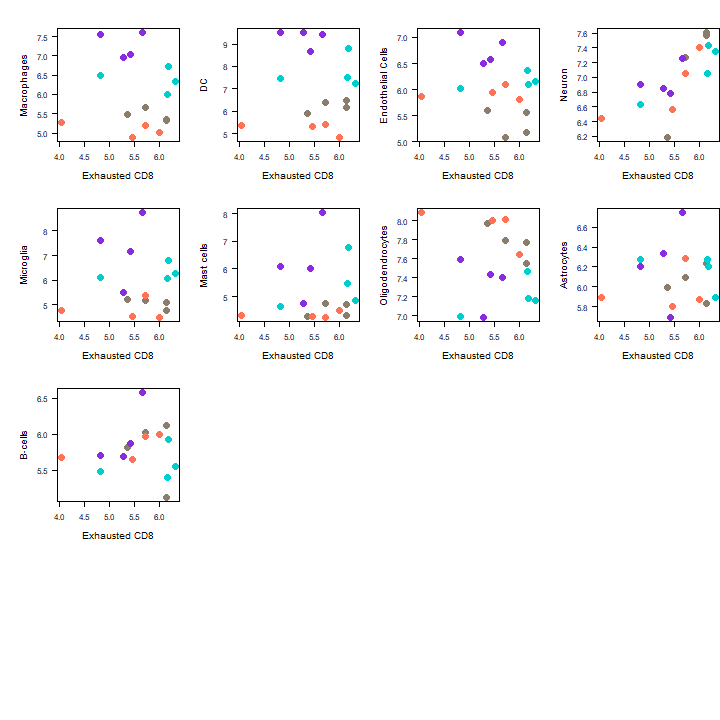

Supplement: Supplementary file 1 [file Data_Sheet_1.ZIP › Bp_final 2026-04-14 12-30/results/cell types/cell scores pairs plot - raw - Exhausted CD8 - colored by DPI.png]

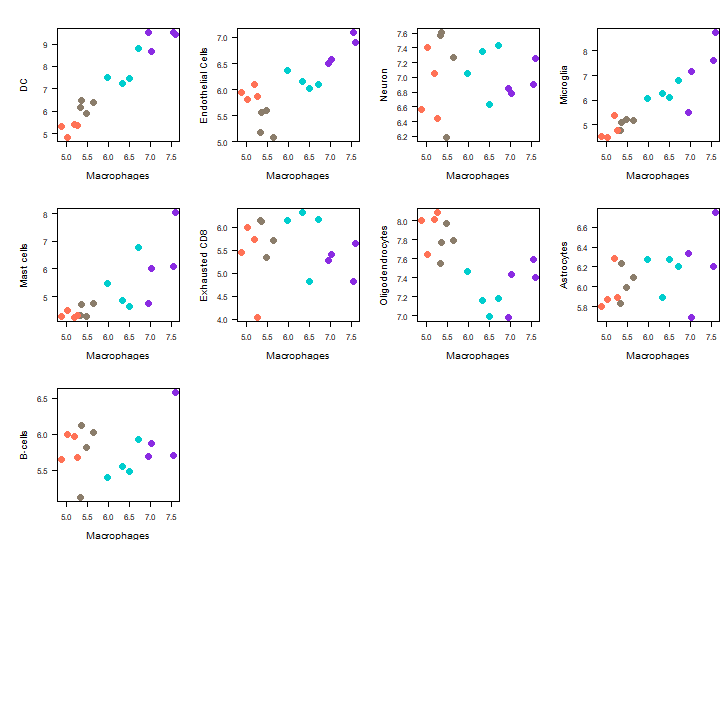

Supplement: Supplementary file 1 [file Data_Sheet_1.ZIP › Bp_final 2026-04-14 12-30/results/cell types/cell scores pairs plot - raw - Macrophages - colored by DPI.png]

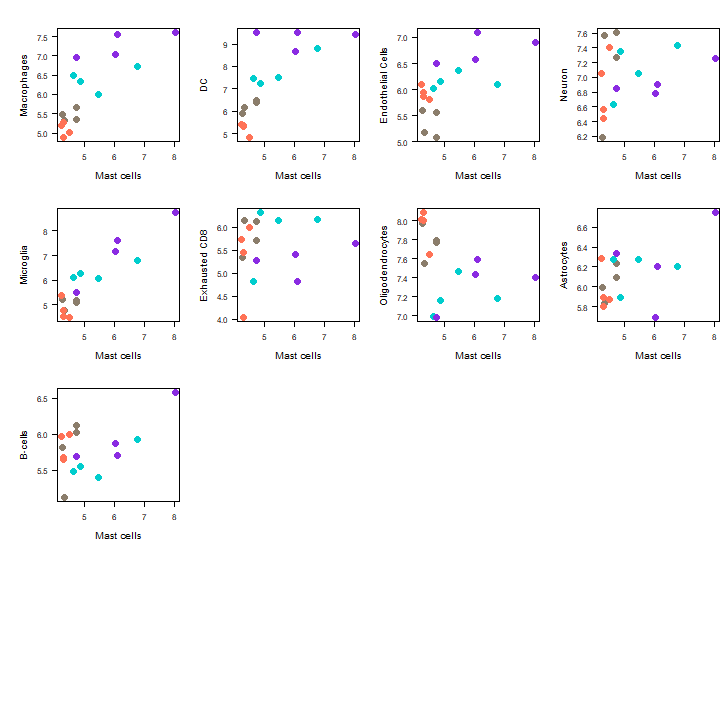

Supplement: Supplementary file 1 [file Data_Sheet_1.ZIP › Bp_final 2026-04-14 12-30/results/cell types/cell scores pairs plot - raw - Mast cells - colored by DPI.png]

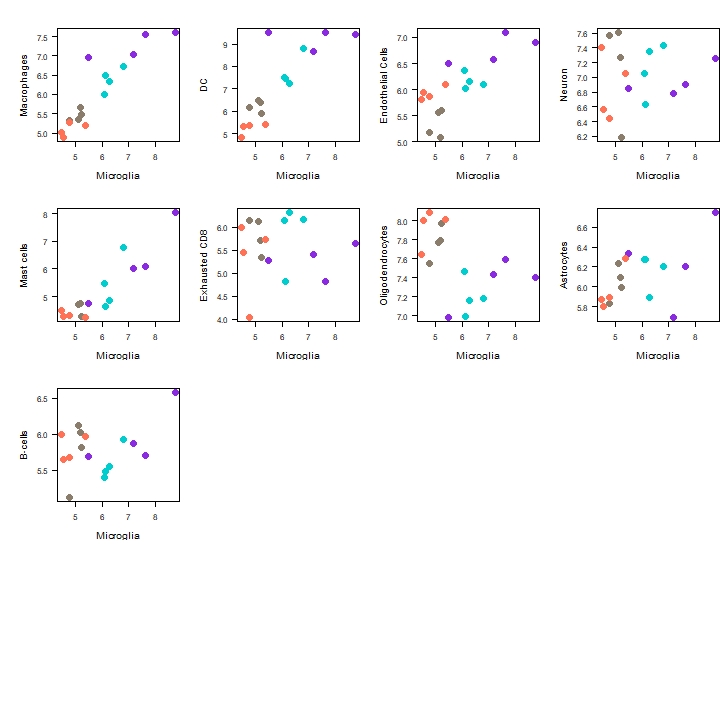

Supplement: Supplementary file 1 [file Data_Sheet_1.ZIP › Bp_final 2026-04-14 12-30/results/cell types/cell scores pairs plot - raw - Microglia - colored by DPI.png]

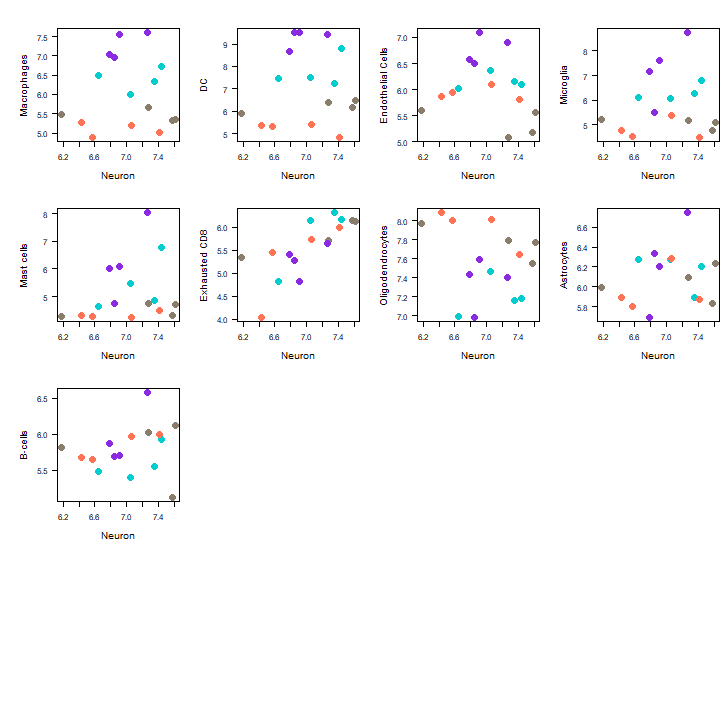

Supplement: Supplementary file 1 [file Data_Sheet_1.ZIP › Bp_final 2026-04-14 12-30/results/cell types/cell scores pairs plot - raw - Neuron - colored by DPI.png]

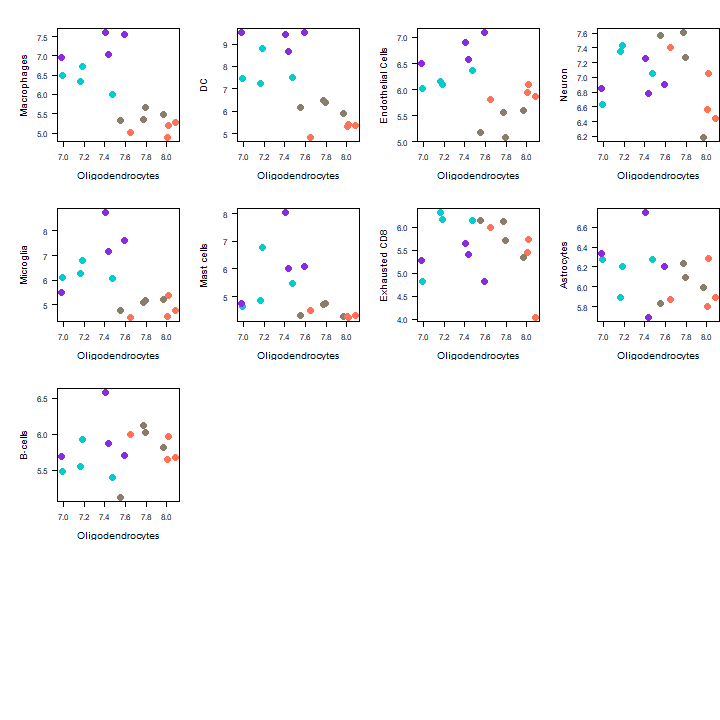

Supplement: Supplementary file 1 [file Data_Sheet_1.ZIP › Bp_final 2026-04-14 12-30/results/cell types/cell scores pairs plot - raw - Oligodendrocytes - colored by DPI.png]

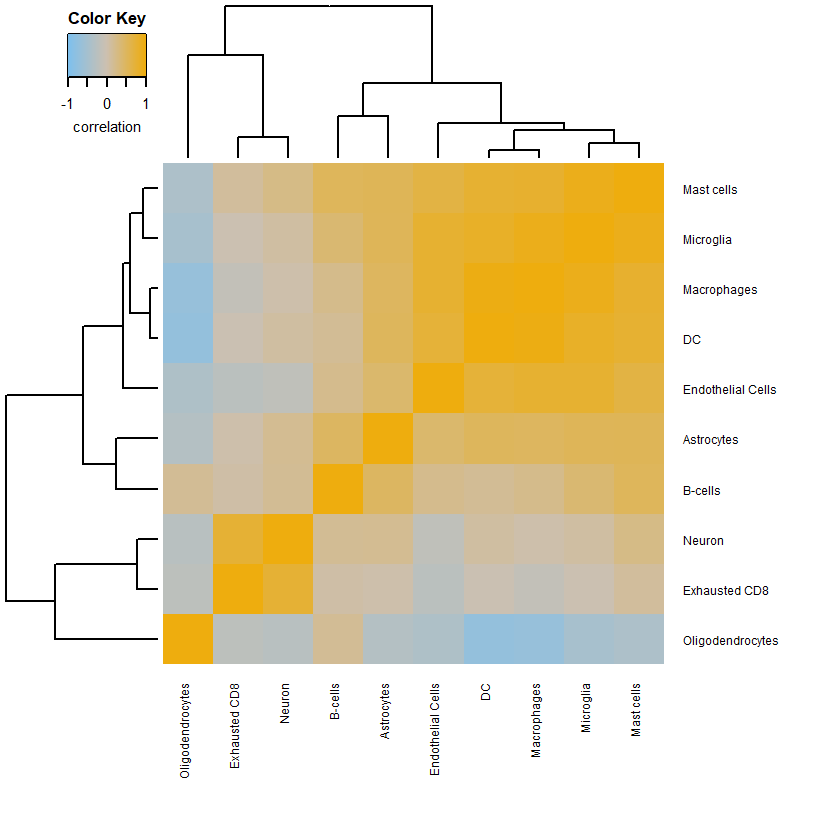

Supplement: Supplementary file 1 [file Data_Sheet_1.ZIP › Bp_final 2026-04-14 12-30/results/cell types/cell type scores correlation heatmap - raw.png]

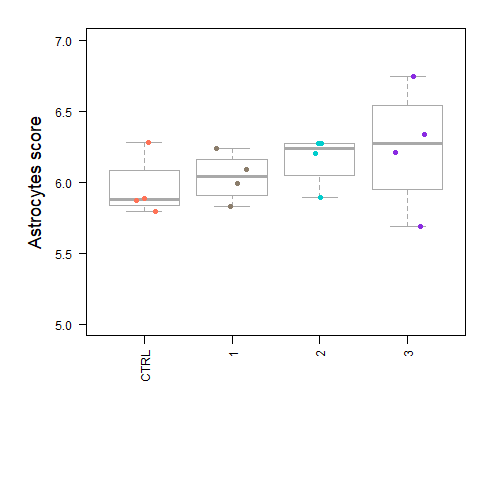

Supplement: Supplementary file 1 [file Data_Sheet_1.ZIP › Bp_final 2026-04-14 12-30/results/cell types/DPI - raw - Astrocytes.png]

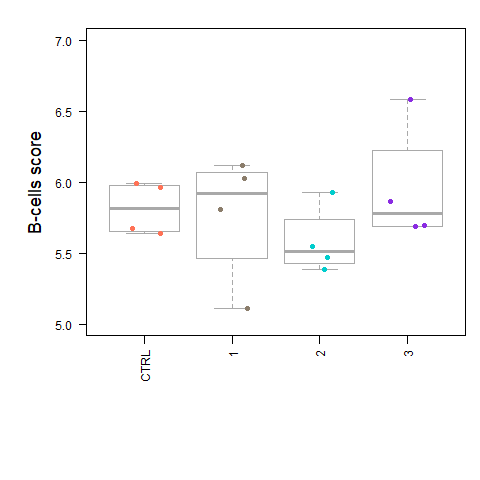

Supplement: Supplementary file 1 [file Data_Sheet_1.ZIP › Bp_final 2026-04-14 12-30/results/cell types/DPI - raw - B-cells.png]

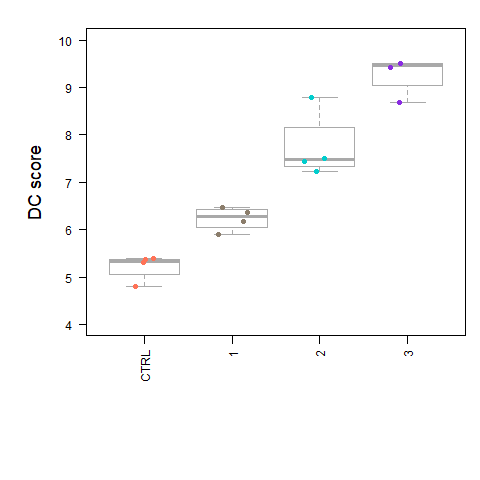

Supplement: Supplementary file 1 [file Data_Sheet_1.ZIP › Bp_final 2026-04-14 12-30/results/cell types/DPI - raw - DC.png]

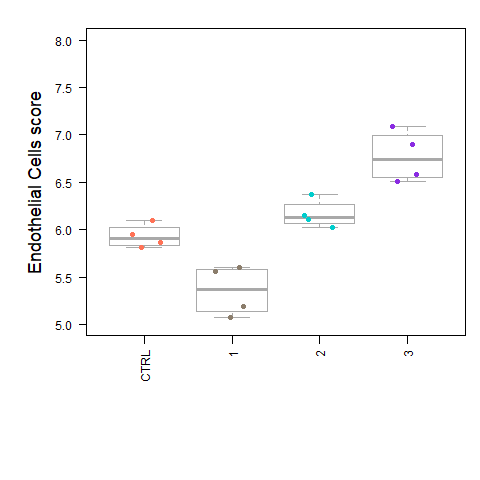

Supplement: Supplementary file 1 [file Data_Sheet_1.ZIP › Bp_final 2026-04-14 12-30/results/cell types/DPI - raw - Endothelial Cells.png]

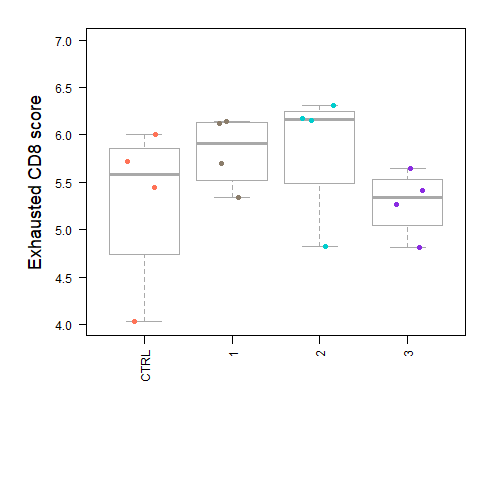

Supplement: Supplementary file 1 [file Data_Sheet_1.ZIP › Bp_final 2026-04-14 12-30/results/cell types/DPI - raw - Exhausted CD8.png]

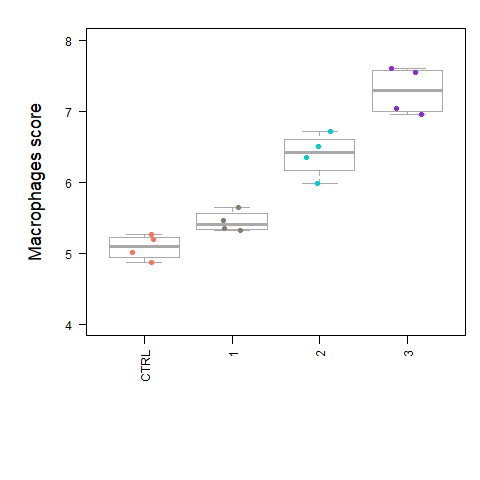

Supplement: Supplementary file 1 [file Data_Sheet_1.ZIP › Bp_final 2026-04-14 12-30/results/cell types/DPI - raw - Macrophages.png]

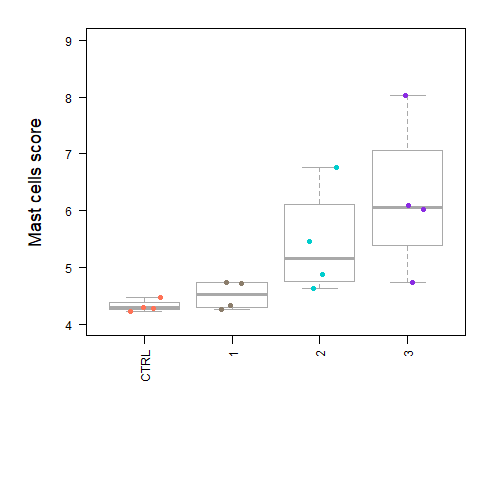

Supplement: Supplementary file 1 [file Data_Sheet_1.ZIP › Bp_final 2026-04-14 12-30/results/cell types/DPI - raw - Mast cells.png]

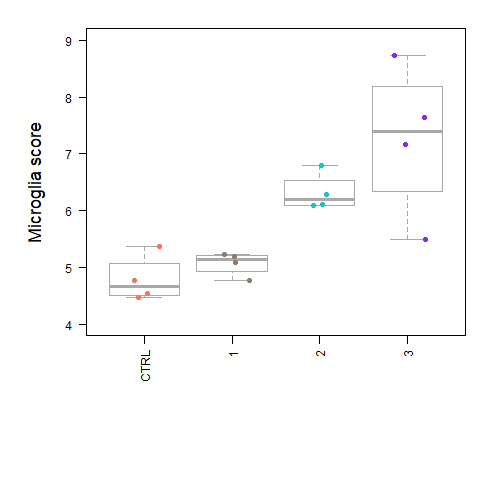

Supplement: Supplementary file 1 [file Data_Sheet_1.ZIP › Bp_final 2026-04-14 12-30/results/cell types/DPI - raw - Microglia.png]

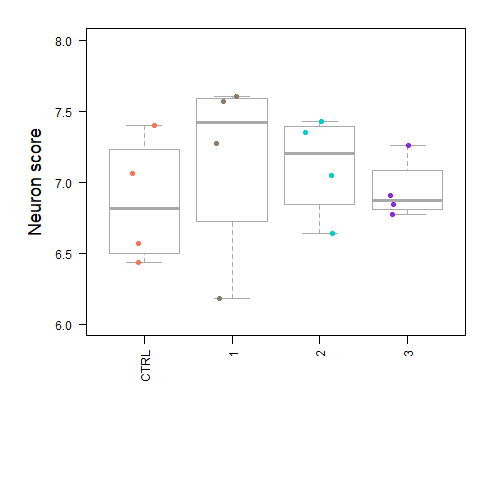

Supplement: Supplementary file 1 [file Data_Sheet_1.ZIP › Bp_final 2026-04-14 12-30/results/cell types/DPI - raw - Neuron.png]

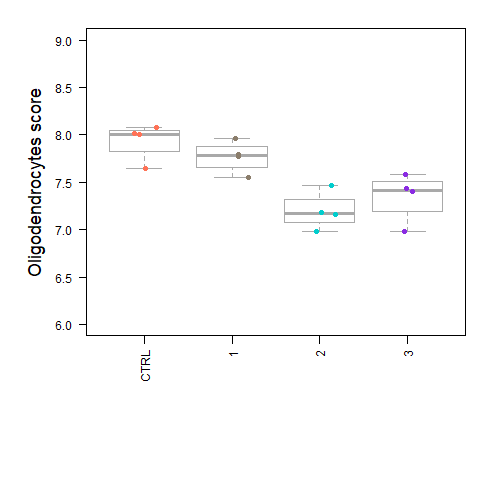

Supplement: Supplementary file 1 [file Data_Sheet_1.ZIP › Bp_final 2026-04-14 12-30/results/cell types/DPI - raw - Oligodendrocytes.png]

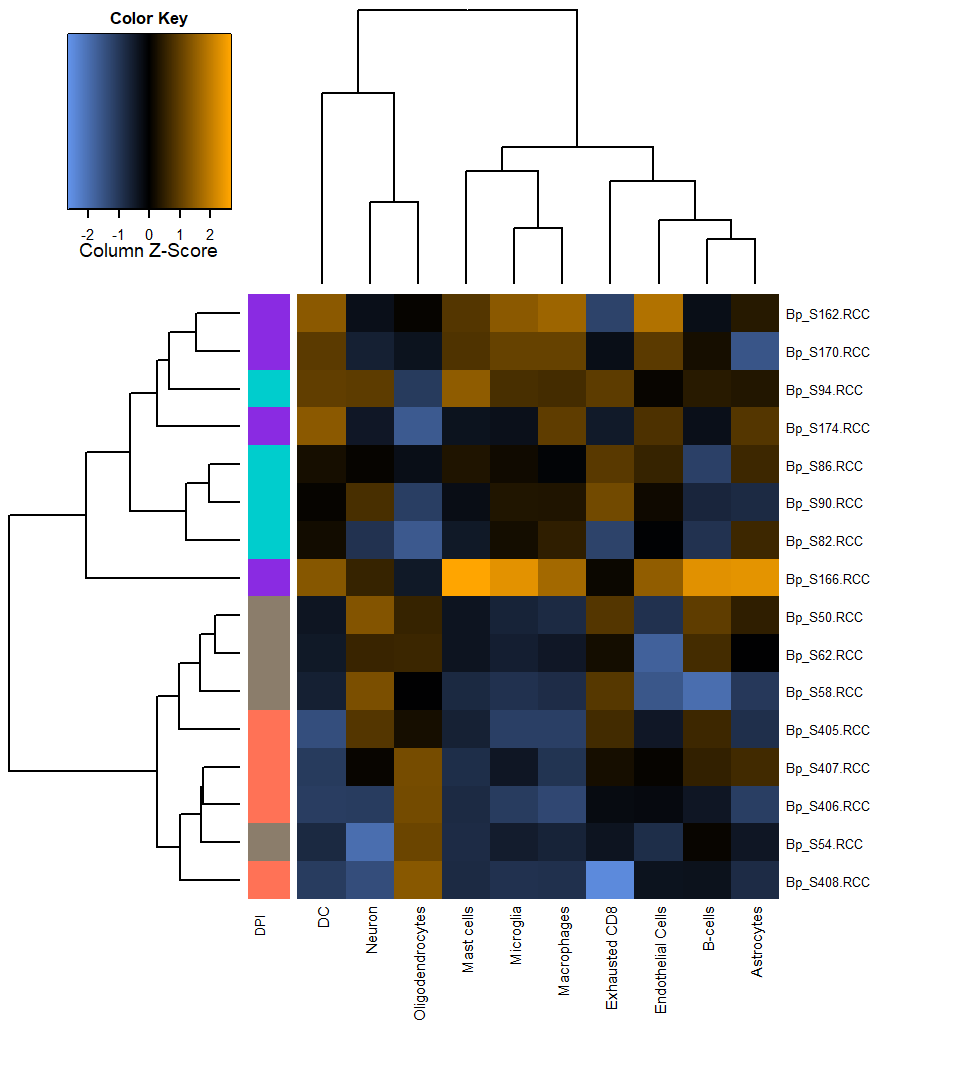

Supplement: Supplementary file 1 [file Data_Sheet_1.ZIP › Bp_final 2026-04-14 12-30/results/cell types/heatmap of cell types scores - raw.png]

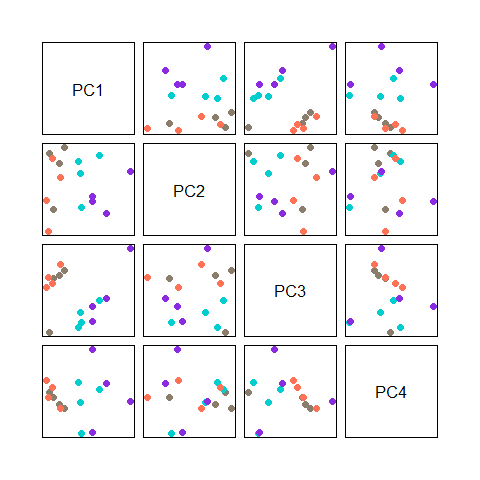

Supplement: Supplementary file 1 [file Data_Sheet_1.ZIP › Bp_final 2026-04-14 12-30/results/cell types/principal components fit to celltype scores pairs plot.png]

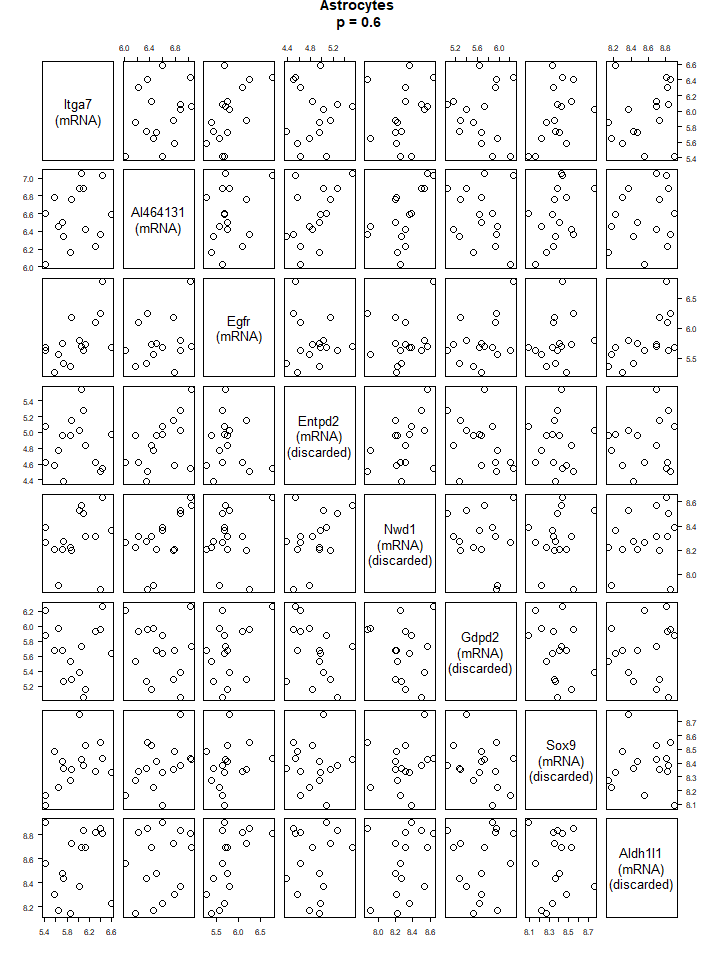

Supplement: Supplementary file 1 [file Data_Sheet_1.ZIP › Bp_final 2026-04-14 12-30/results/cell types/QC for cell scores - Astrocytes.png]

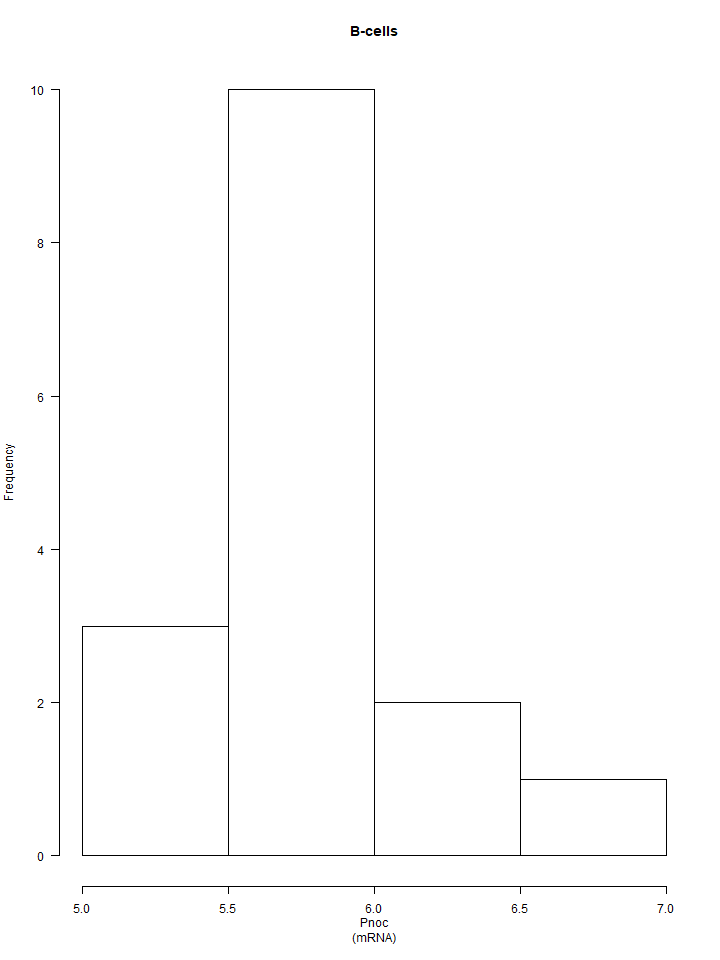

Supplement: Supplementary file 1 [file Data_Sheet_1.ZIP › Bp_final 2026-04-14 12-30/results/cell types/QC for cell scores - B-cells.png]

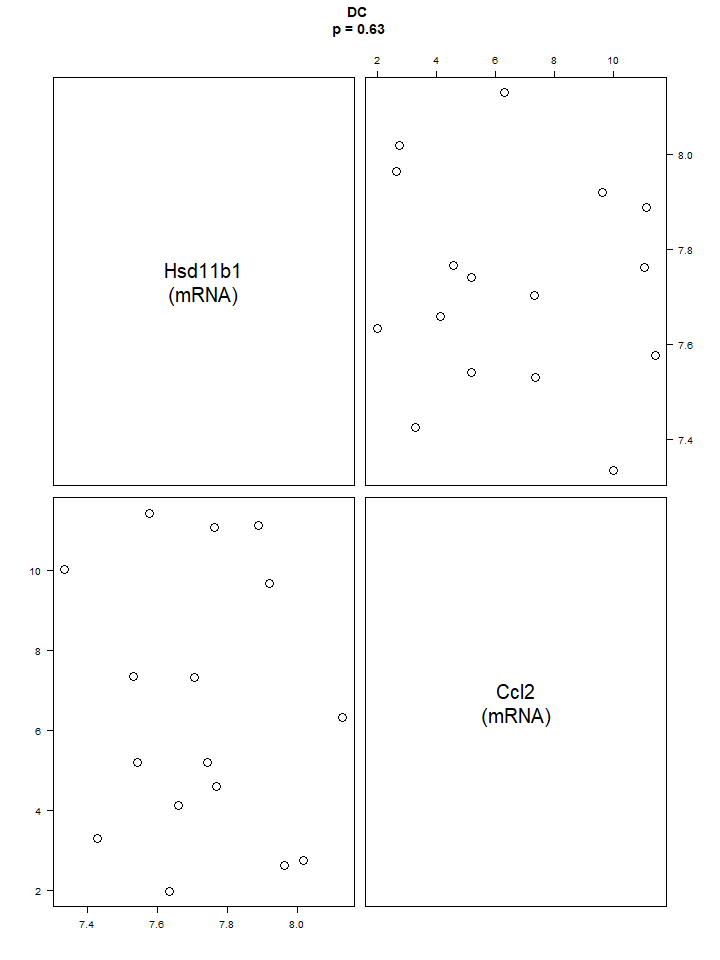

Supplement: Supplementary file 1 [file Data_Sheet_1.ZIP › Bp_final 2026-04-14 12-30/results/cell types/QC for cell scores - DC.png]

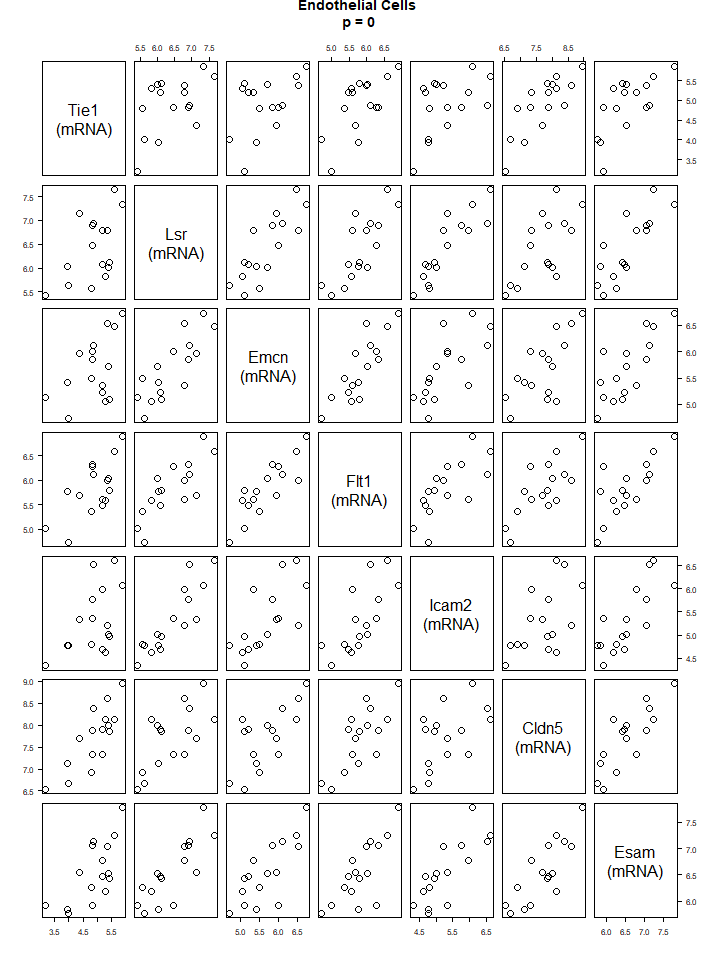

Supplement: Supplementary file 1 [file Data_Sheet_1.ZIP › Bp_final 2026-04-14 12-30/results/cell types/QC for cell scores - Endothelial Cells.png]

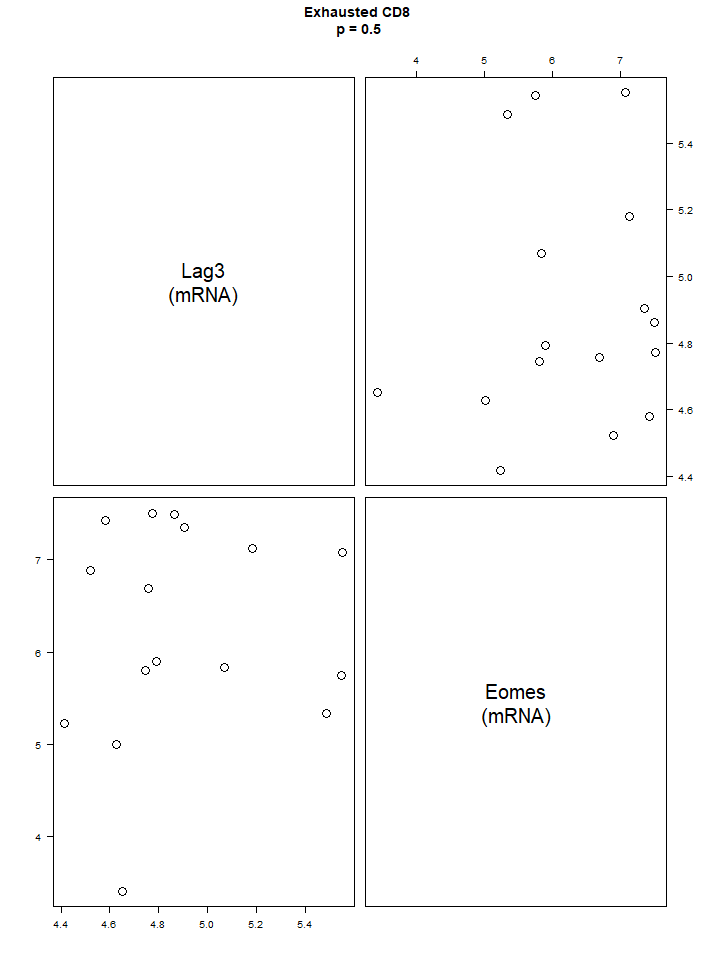

Supplement: Supplementary file 1 [file Data_Sheet_1.ZIP › Bp_final 2026-04-14 12-30/results/cell types/QC for cell scores - Exhausted CD8.png]

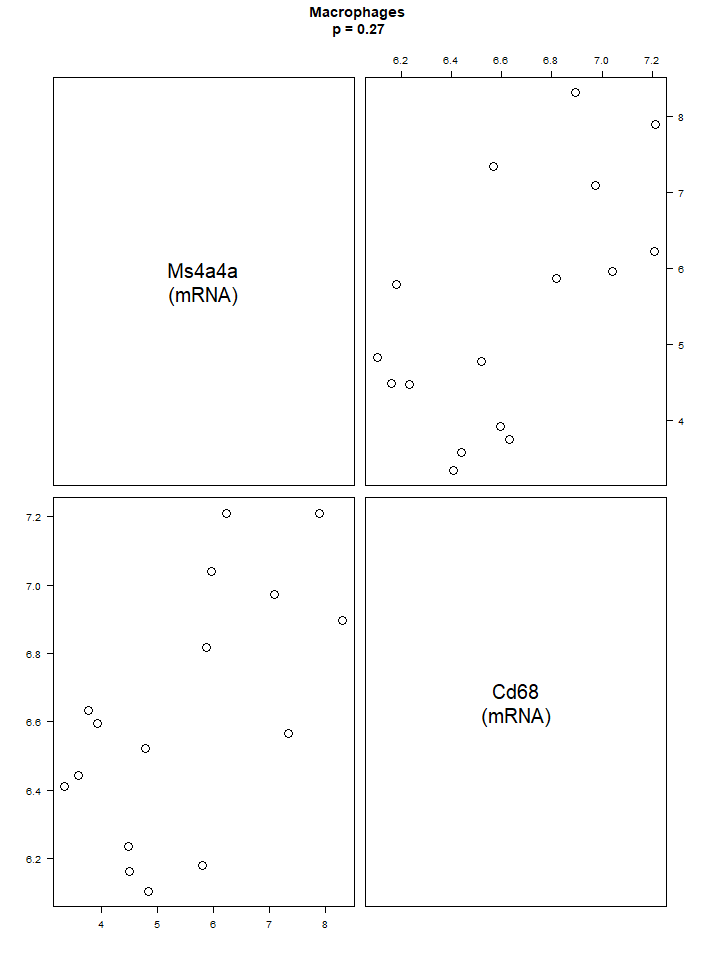

Supplement: Supplementary file 1 [file Data_Sheet_1.ZIP › Bp_final 2026-04-14 12-30/results/cell types/QC for cell scores - Macrophages.png]

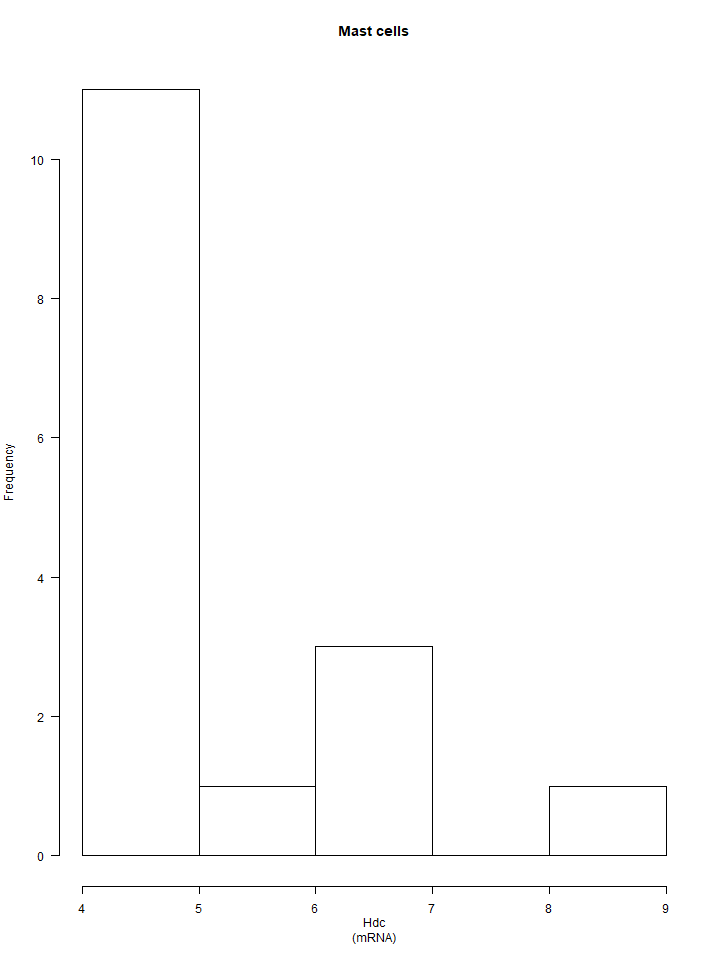

Supplement: Supplementary file 1 [file Data_Sheet_1.ZIP › Bp_final 2026-04-14 12-30/results/cell types/QC for cell scores - Mast cells.png]

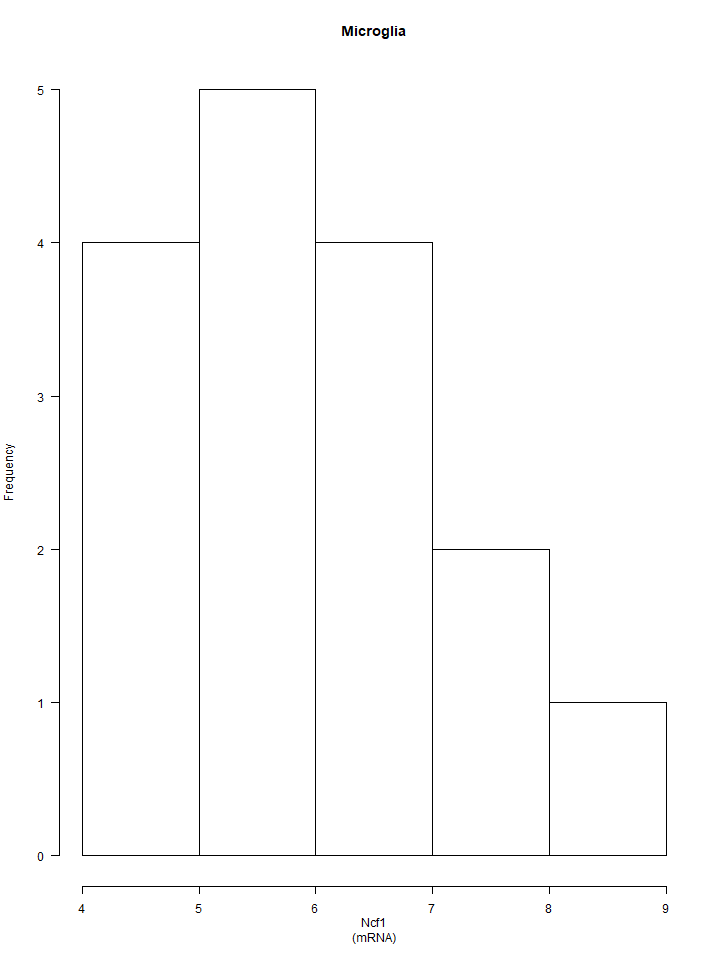

Supplement: Supplementary file 1 [file Data_Sheet_1.ZIP › Bp_final 2026-04-14 12-30/results/cell types/QC for cell scores - Microglia.png]

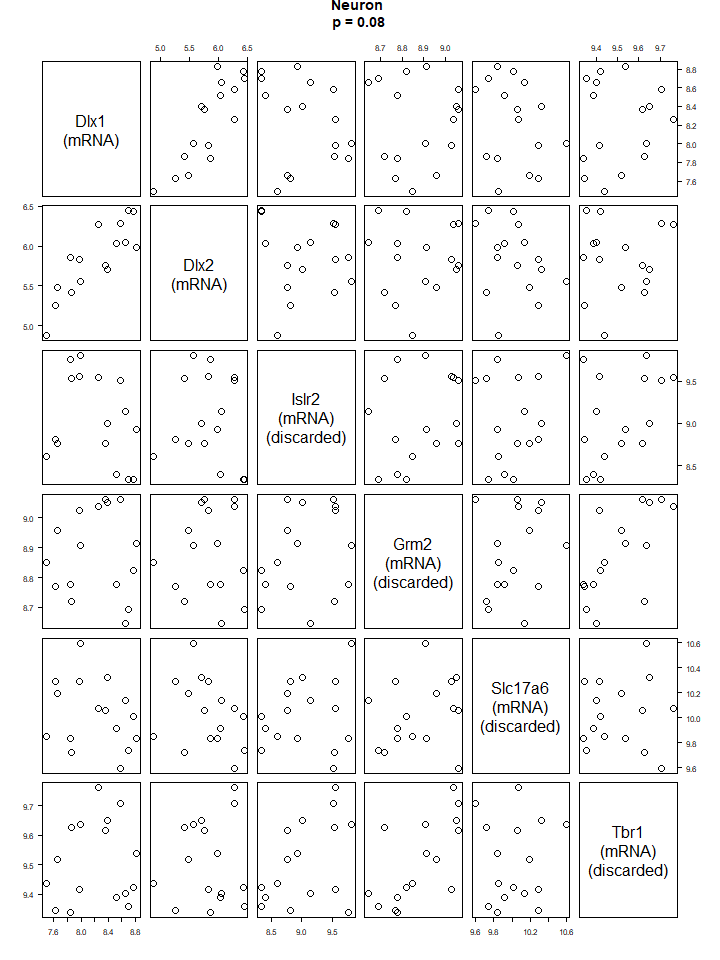

Supplement: Supplementary file 1 [file Data_Sheet_1.ZIP › Bp_final 2026-04-14 12-30/results/cell types/QC for cell scores - Neuron.png]

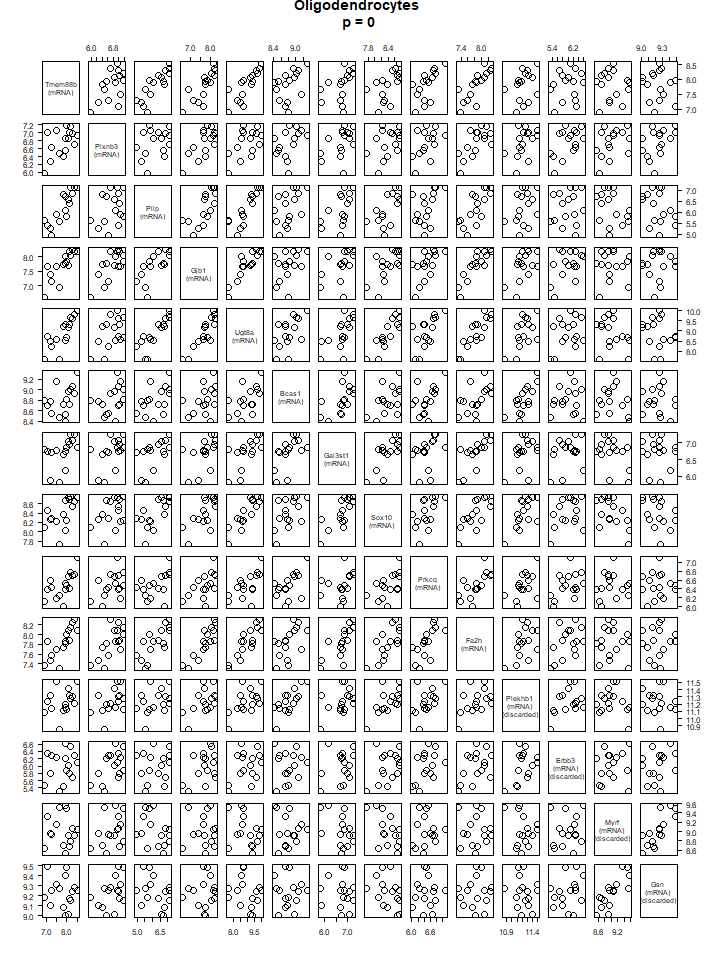

Supplement: Supplementary file 1 [file Data_Sheet_1.ZIP › Bp_final 2026-04-14 12-30/results/cell types/QC for cell scores - Oligodendrocytes.png]

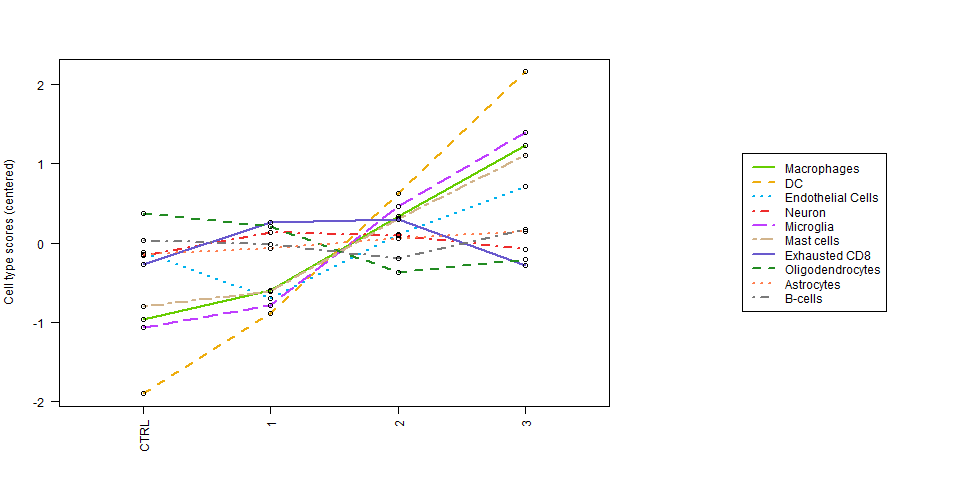

Supplement: Supplementary file 1 [file Data_Sheet_1.ZIP › Bp_final 2026-04-14 12-30/results/cell types/trend plot of cell type scores vs. DPI - raw - legend.png]

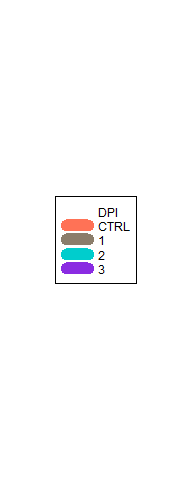

Supplement: Supplementary file 1 [file Data_Sheet_1.ZIP › Bp_final 2026-04-14 12-30/results/color legend - DPI.png]

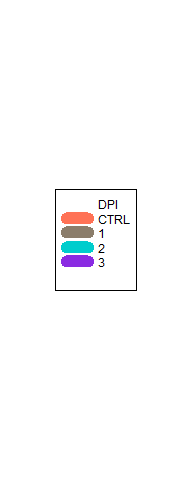

Supplement: Supplementary file 1 [file Data_Sheet_1.ZIP › Bp_final 2026-04-14 12-30/results/color legend.png]

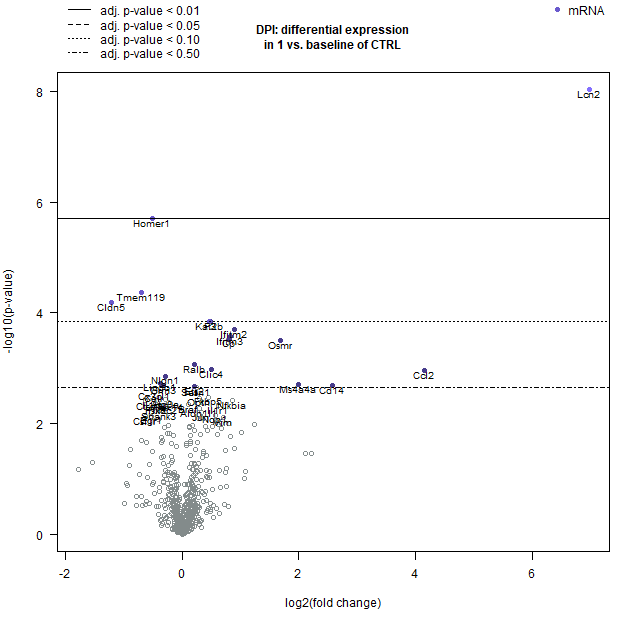

Supplement: Supplementary file 1 [file Data_Sheet_1.ZIP › Bp_final 2026-04-14 12-30/results/DE/volcano plotDPI1.png]

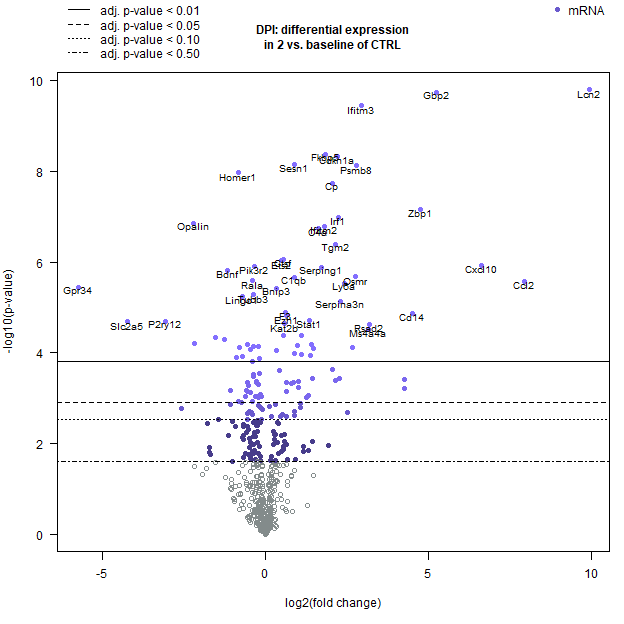

Supplement: Supplementary file 1 [file Data_Sheet_1.ZIP › Bp_final 2026-04-14 12-30/results/DE/volcano plotDPI2.png]

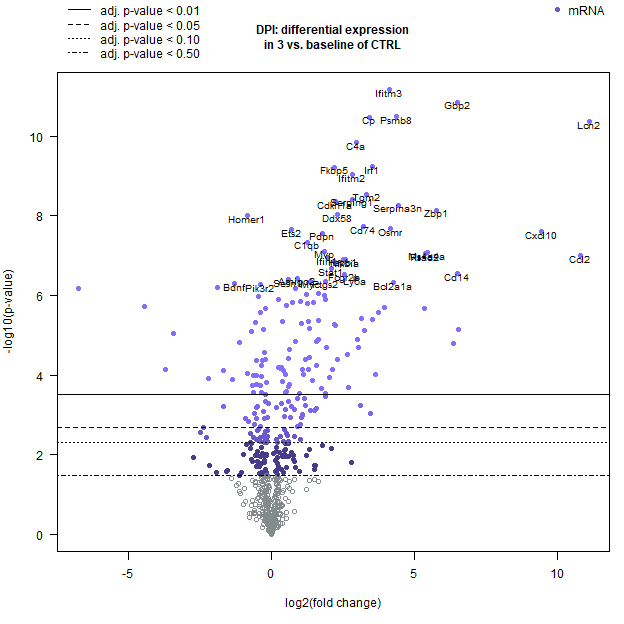

Supplement: Supplementary file 1 [file Data_Sheet_1.ZIP › Bp_final 2026-04-14 12-30/results/DE/volcano plotDPI3.png]

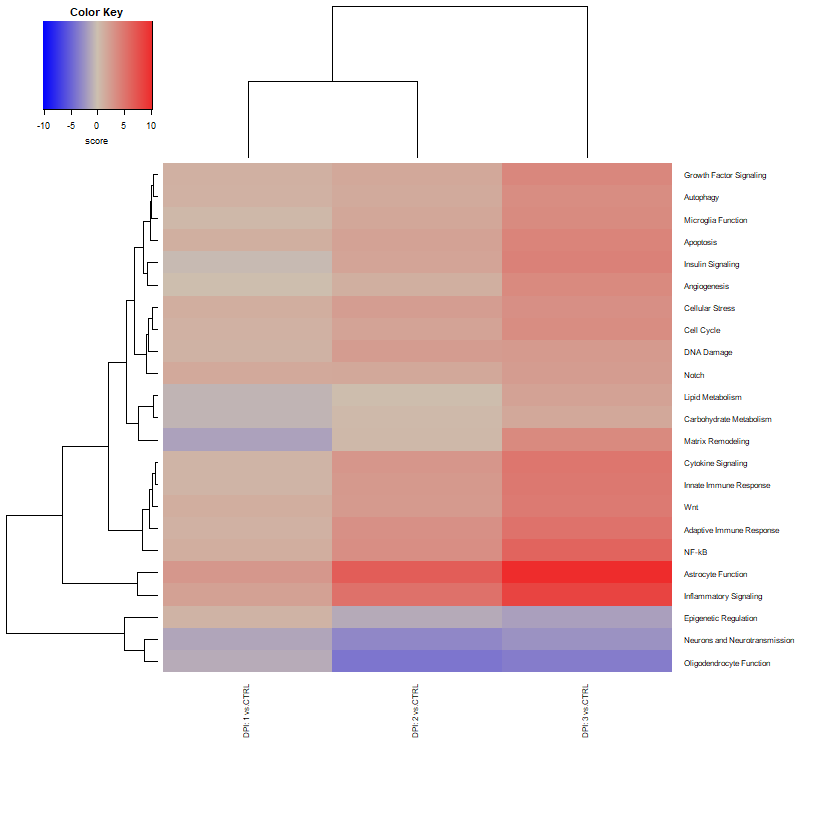

Supplement: Supplementary file 1 [file Data_Sheet_1.ZIP › Bp_final 2026-04-14 12-30/results/Gene set analysis/heatmap of directed global significance scores - directed.png]

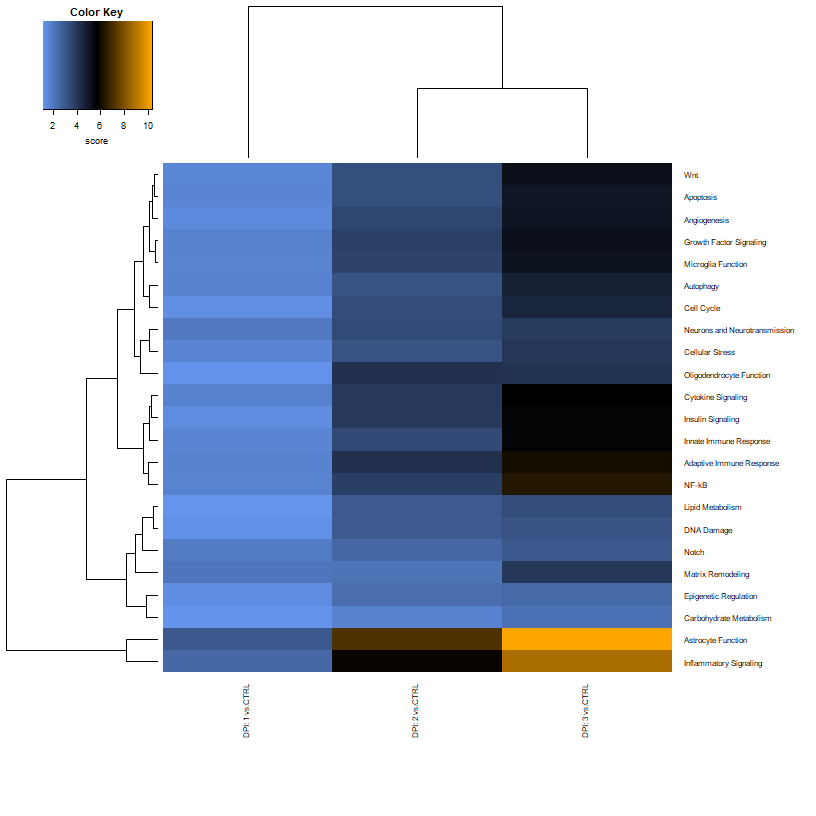

Supplement: Supplementary file 1 [file Data_Sheet_1.ZIP › Bp_final 2026-04-14 12-30/results/Gene set analysis/heatmap of global significance scores.png]

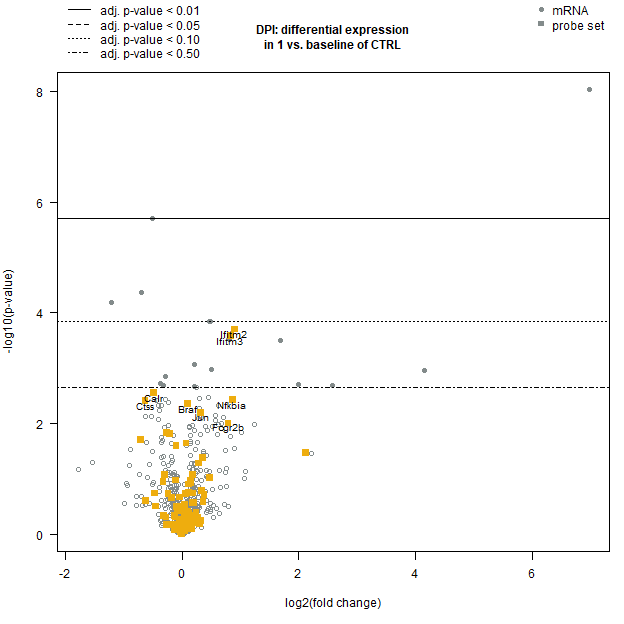

Supplement: Supplementary file 1 [file Data_Sheet_1.ZIP › Bp_final 2026-04-14 12-30/results/Gene set analysis/volcano plot - DPI1 - Adaptive.Immune.Response.png]

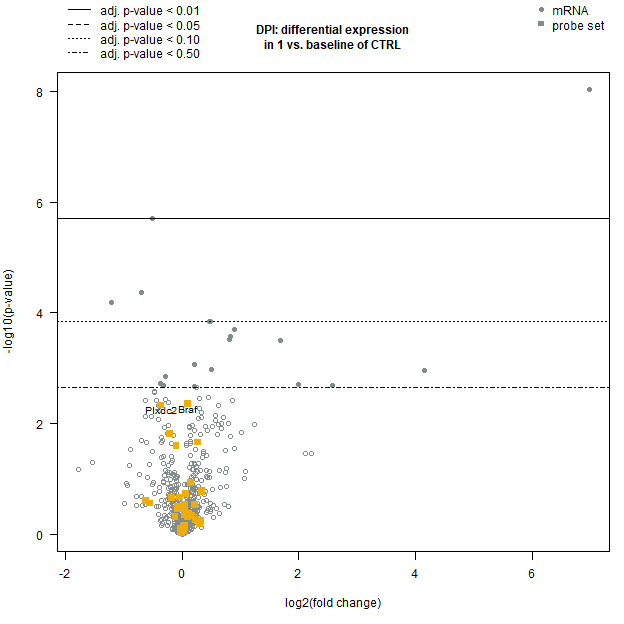

Supplement: Supplementary file 1 [file Data_Sheet_1.ZIP › Bp_final 2026-04-14 12-30/results/Gene set analysis/volcano plot - DPI1 - Angiogenesis.png]

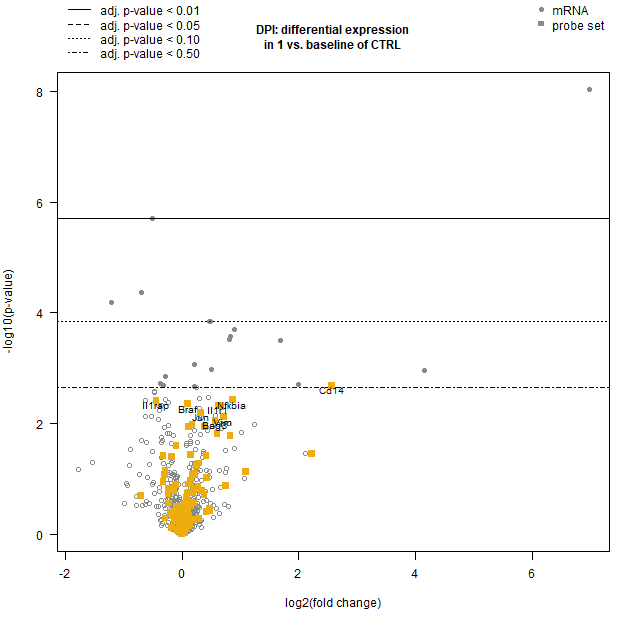

Supplement: Supplementary file 1 [file Data_Sheet_1.ZIP › Bp_final 2026-04-14 12-30/results/Gene set analysis/volcano plot - DPI1 - Apoptosis.png]

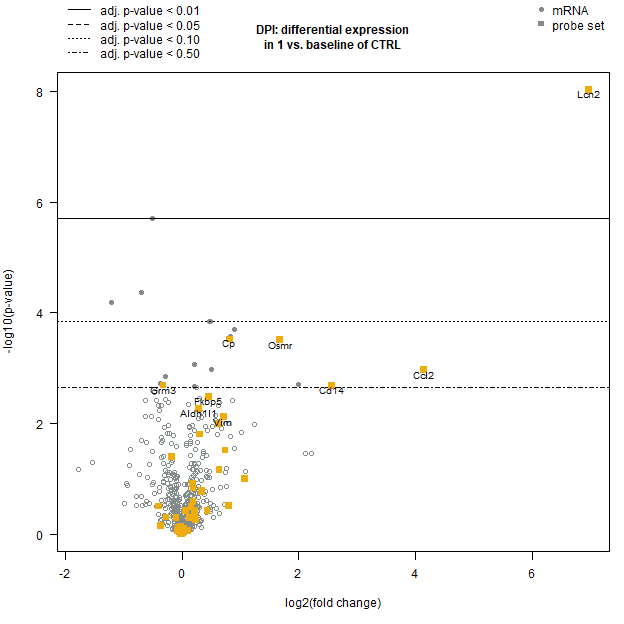

Supplement: Supplementary file 1 [file Data_Sheet_1.ZIP › Bp_final 2026-04-14 12-30/results/Gene set analysis/volcano plot - DPI1 - Astrocyte.Function.png]

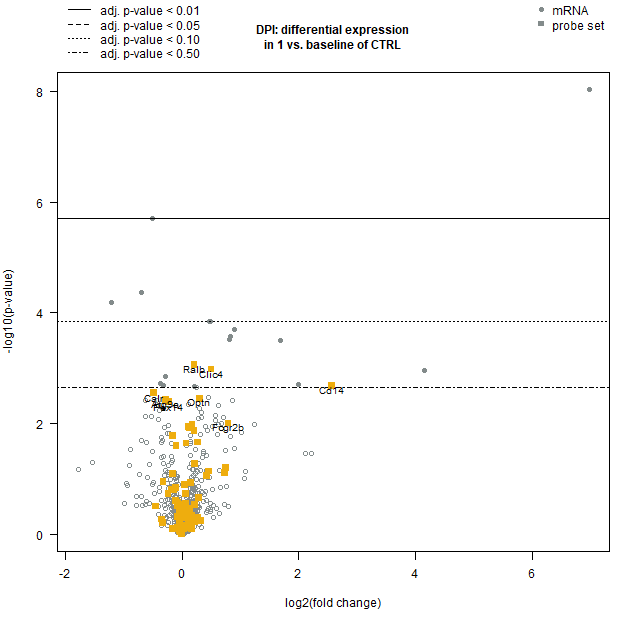

Supplement: Supplementary file 1 [file Data_Sheet_1.ZIP › Bp_final 2026-04-14 12-30/results/Gene set analysis/volcano plot - DPI1 - Autophagy.png]

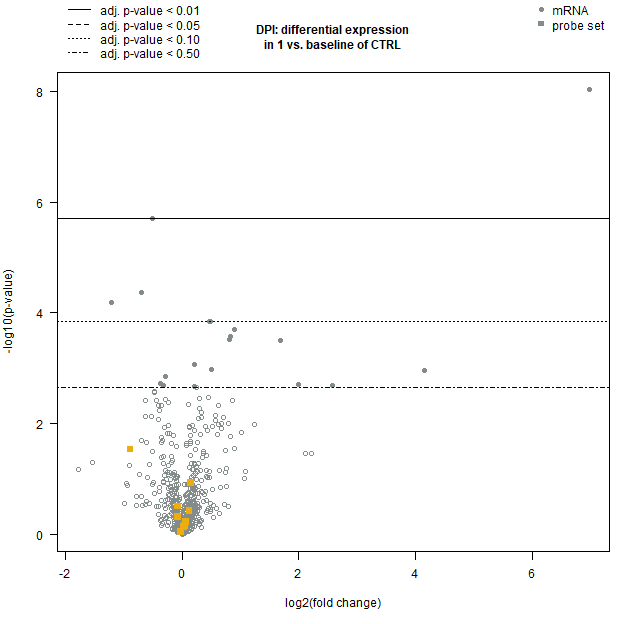

Supplement: Supplementary file 1 [file Data_Sheet_1.ZIP › Bp_final 2026-04-14 12-30/results/Gene set analysis/volcano plot - DPI1 - Carbohydrate.Metabolism.png]

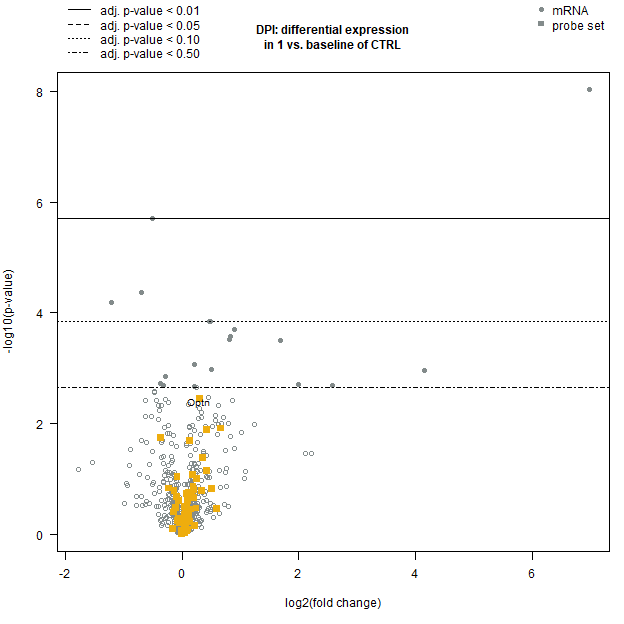

Supplement: Supplementary file 1 [file Data_Sheet_1.ZIP › Bp_final 2026-04-14 12-30/results/Gene set analysis/volcano plot - DPI1 - Cell.Cycle.png]

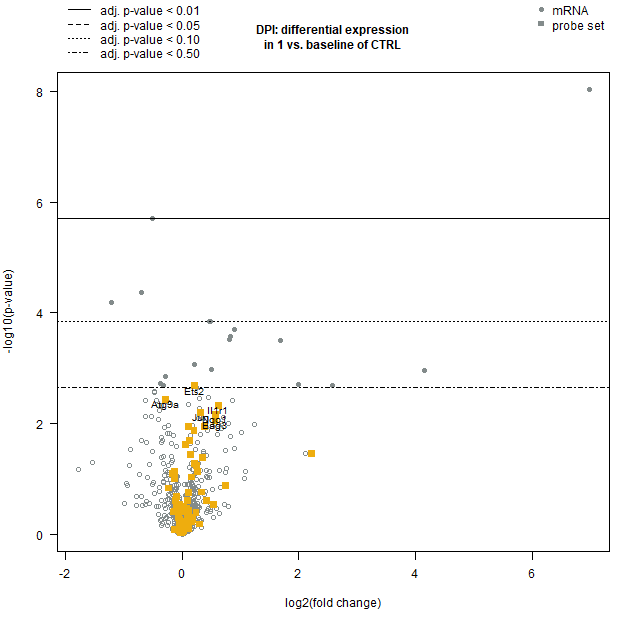

Supplement: Supplementary file 1 [file Data_Sheet_1.ZIP › Bp_final 2026-04-14 12-30/results/Gene set analysis/volcano plot - DPI1 - Cellular.Stress.png]

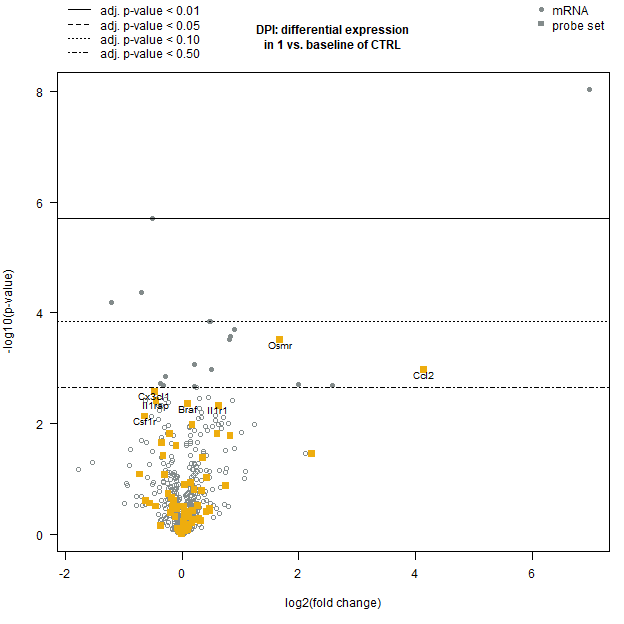

Supplement: Supplementary file 1 [file Data_Sheet_1.ZIP › Bp_final 2026-04-14 12-30/results/Gene set analysis/volcano plot - DPI1 - Cytokine.Signaling.png]

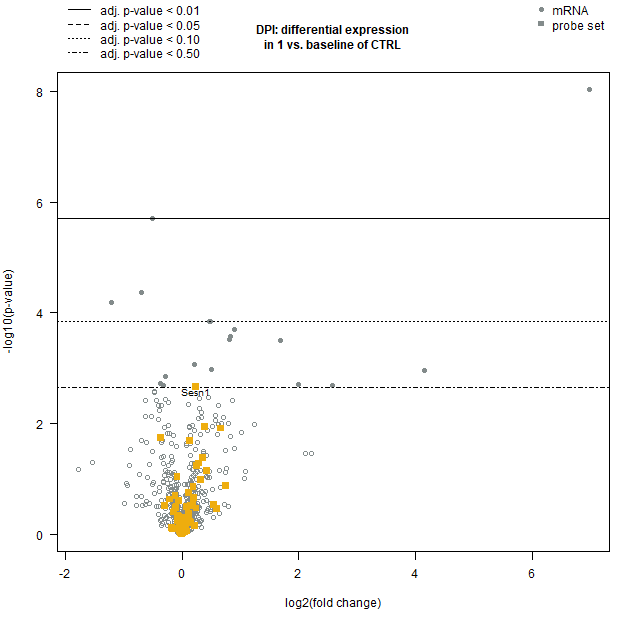

Supplement: Supplementary file 1 [file Data_Sheet_1.ZIP › Bp_final 2026-04-14 12-30/results/Gene set analysis/volcano plot - DPI1 - DNA.Damage.png]

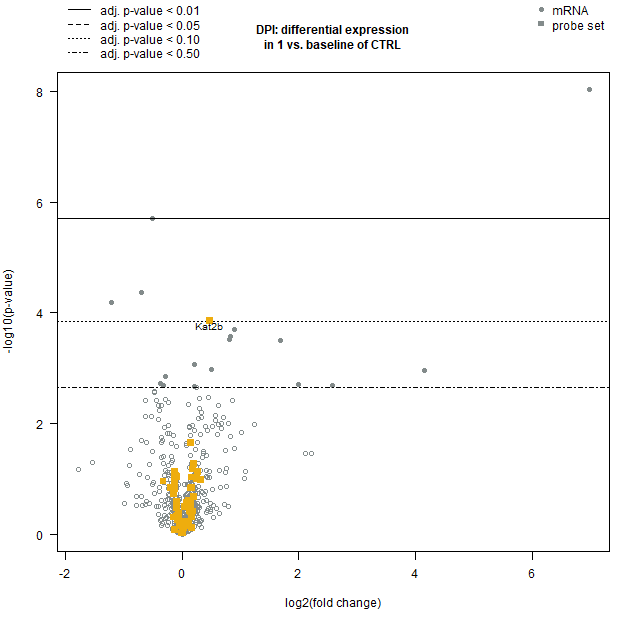

Supplement: Supplementary file 1 [file Data_Sheet_1.ZIP › Bp_final 2026-04-14 12-30/results/Gene set analysis/volcano plot - DPI1 - Epigenetic.Regulation.png]

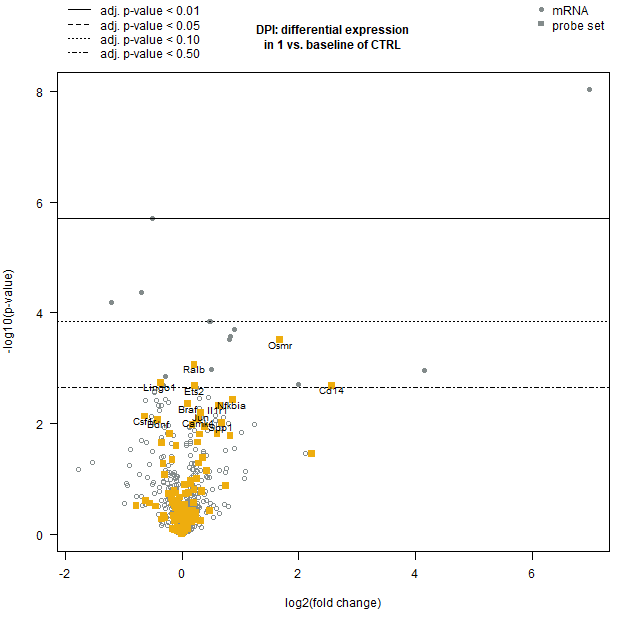

Supplement: Supplementary file 1 [file Data_Sheet_1.ZIP › Bp_final 2026-04-14 12-30/results/Gene set analysis/volcano plot - DPI1 - Growth.Factor.Signaling.png]

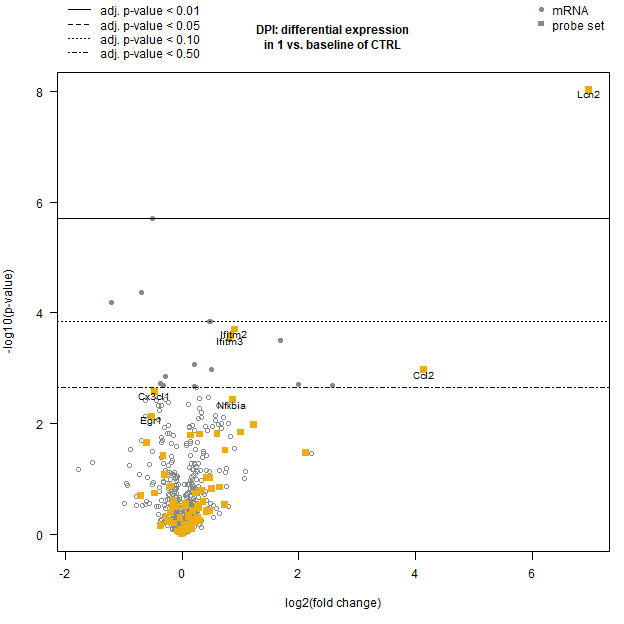

Supplement: Supplementary file 1 [file Data_Sheet_1.ZIP › Bp_final 2026-04-14 12-30/results/Gene set analysis/volcano plot - DPI1 - Inflammatory.Signaling.png]

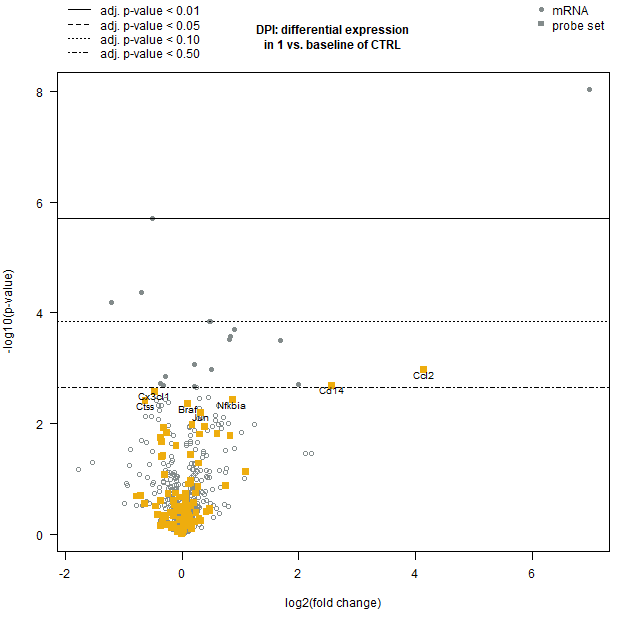

Supplement: Supplementary file 1 [file Data_Sheet_1.ZIP › Bp_final 2026-04-14 12-30/results/Gene set analysis/volcano plot - DPI1 - Innate.Immune.Response.png]

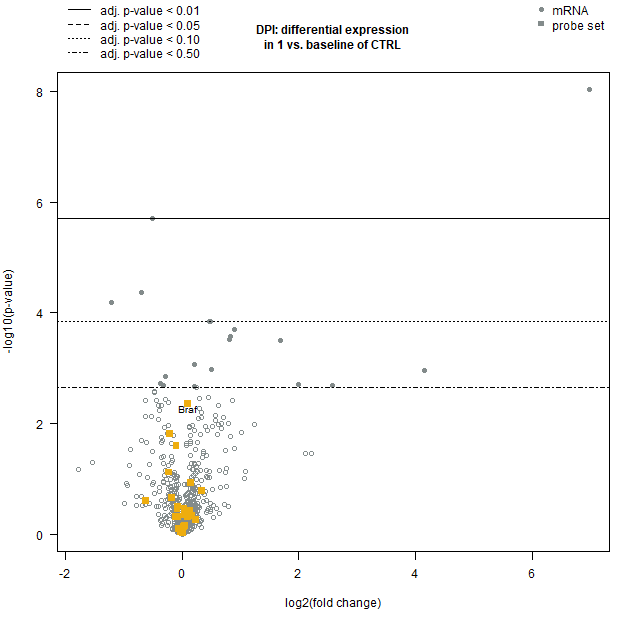

Supplement: Supplementary file 1 [file Data_Sheet_1.ZIP › Bp_final 2026-04-14 12-30/results/Gene set analysis/volcano plot - DPI1 - Insulin.Signaling.png]

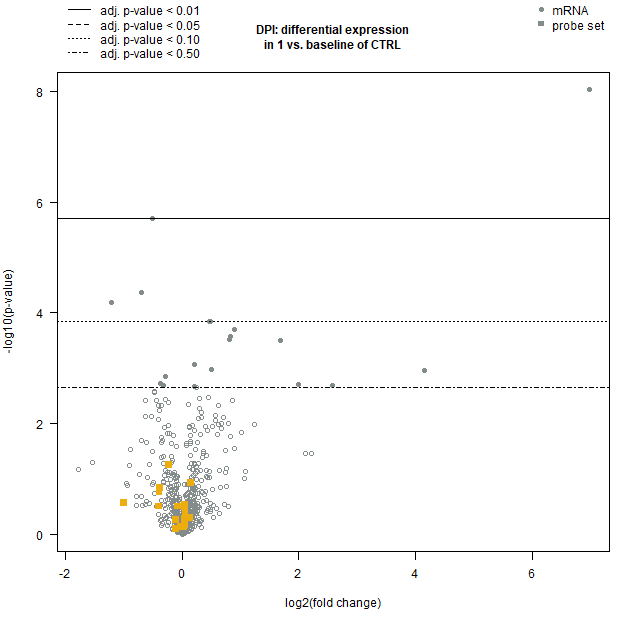

Supplement: Supplementary file 1 [file Data_Sheet_1.ZIP › Bp_final 2026-04-14 12-30/results/Gene set analysis/volcano plot - DPI1 - Lipid.Metabolism.png]

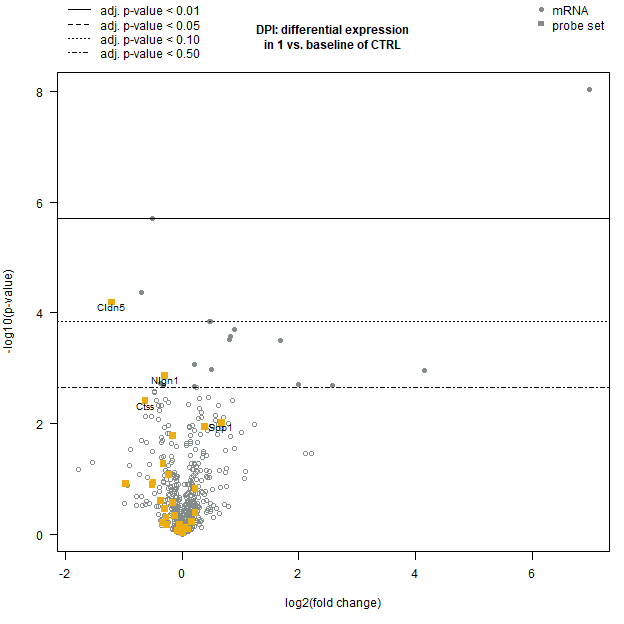

Supplement: Supplementary file 1 [file Data_Sheet_1.ZIP › Bp_final 2026-04-14 12-30/results/Gene set analysis/volcano plot - DPI1 - Matrix.Remodeling.png]

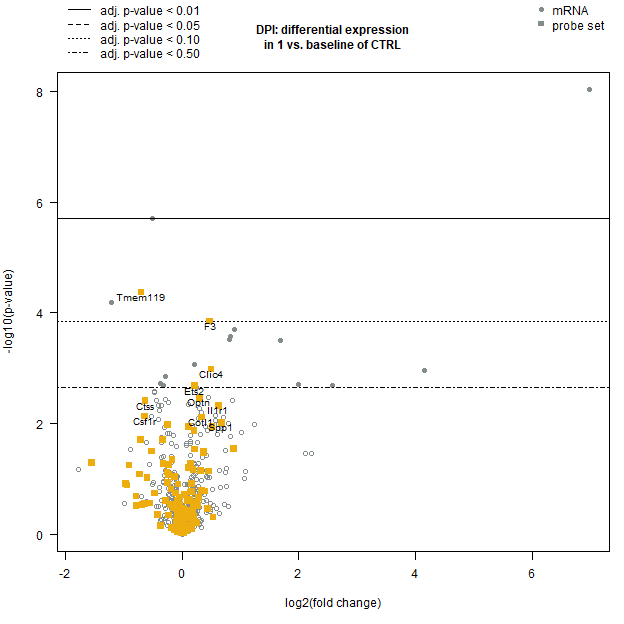

Supplement: Supplementary file 1 [file Data_Sheet_1.ZIP › Bp_final 2026-04-14 12-30/results/Gene set analysis/volcano plot - DPI1 - Microglia.Function.png]

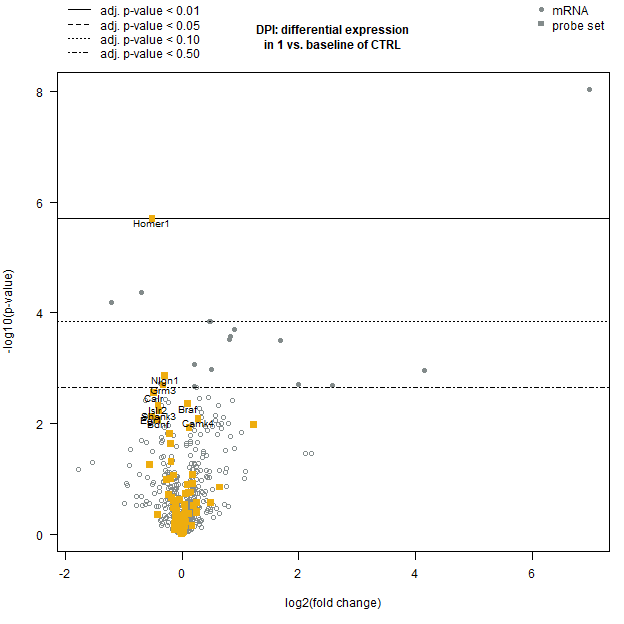

Supplement: Supplementary file 1 [file Data_Sheet_1.ZIP › Bp_final 2026-04-14 12-30/results/Gene set analysis/volcano plot - DPI1 - Neurons.and.Neurotransmission.png]

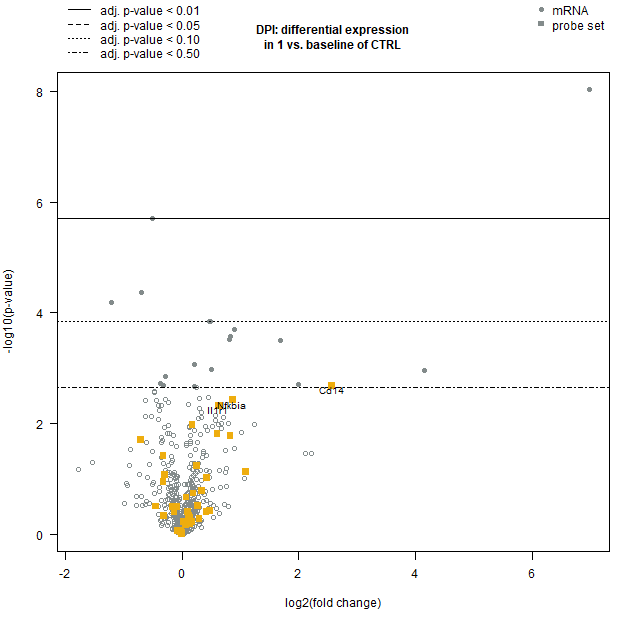

Supplement: Supplementary file 1 [file Data_Sheet_1.ZIP › Bp_final 2026-04-14 12-30/results/Gene set analysis/volcano plot - DPI1 - NF.kB.png]
