# Supplementary material for: Vascular-associated bacterial burden and neuroinflammatory transcriptional responses observed in models of pneumonic plague
Source: Front Microbiol. 2026 Jun 24;17:1865125. doi: 10.3389/fmicb.2026.1865125 (PMC13341613; doi:10.3389/fmicb.2026.1865125)
Supplement: Supplementary file 2 [file Presentation_1.PPTX]

## Slide 1
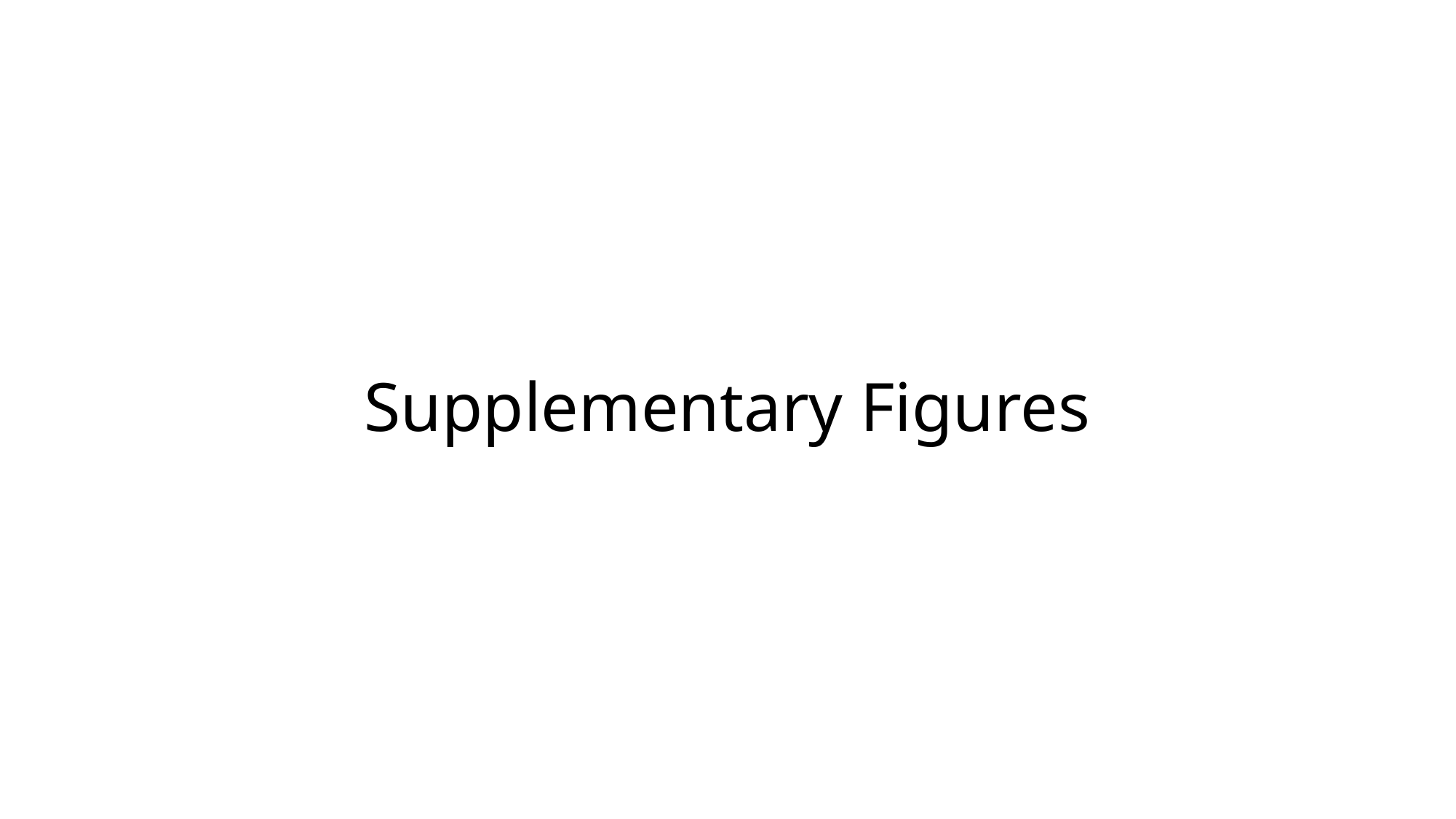

# Supplementary Figures

## Slide 2
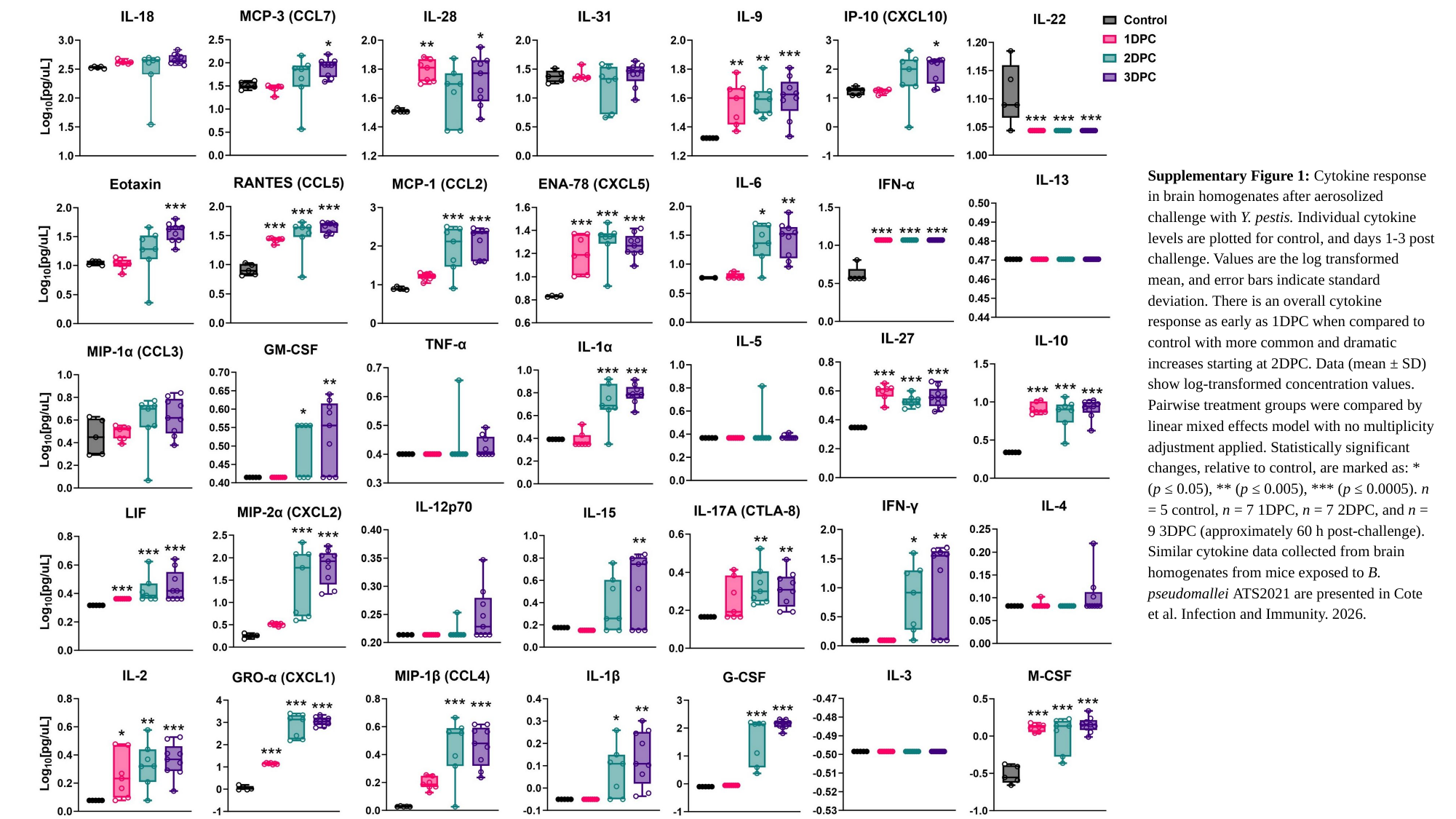

Supplementary Figure 1: Cytokine response in brain homogenates after aerosolized challenge with Y. pestis. Individual cytokine levels are plotted for control, and days 1-3 post challenge. Values are the log transformed mean, and error bars indicate standard deviation. There is an overall cytokine response as early as 1DPC when compared to control with more common and dramatic increases starting at 2DPC. Data (mean ± SD) show log-transformed concentration values. Pairwise treatment groups were compared by linear mixed effects model with no multiplicity adjustment applied. Statistically significant changes, relative to control, are marked as: * (p ≤ 0.05), ** (p ≤ 0.005), *** (p ≤ 0.0005). n = 5 control, n = 7 1DPC, n = 7 2DPC, and n = 9 3DPC (approximately 60 h post-challenge). Similar cytokine data collected from brain homogenates from mice exposed to B. pseudomallei ATS2021 are presented in Cote et al. Infection and Immunity. 2026.

## Slide 3
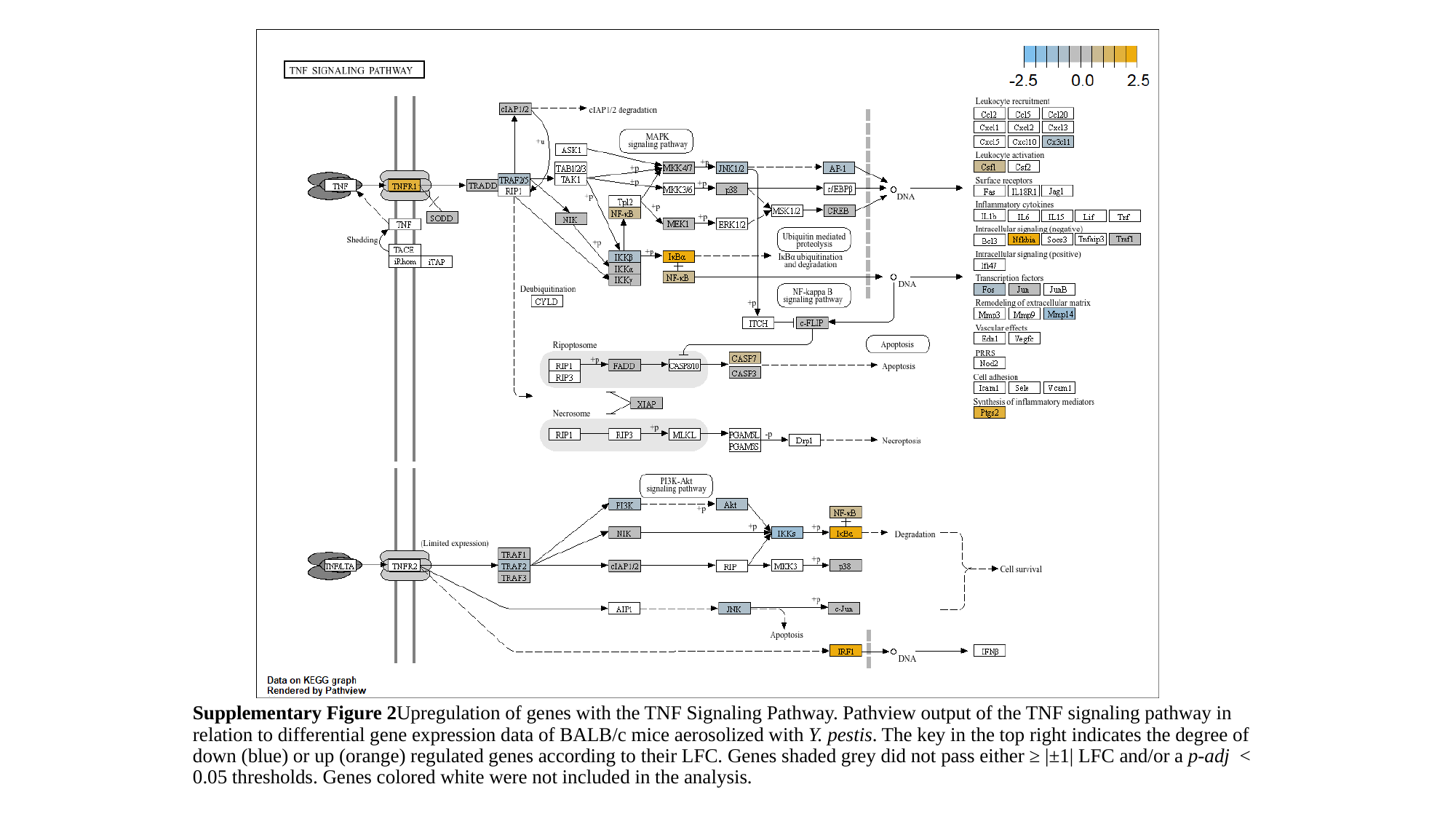

Supplementary Figure 2Upregulation of genes with the TNF Signaling Pathway. Pathview output of the TNF signaling pathway in relation to differential gene expression data of BALB/c mice aerosolized with Y. pestis. The key in the top right indicates the degree of down (blue) or up (orange) regulated genes according to their LFC. Genes shaded grey did not pass either ≥ |±1| LFC and/or a p-adj < 0.05 thresholds. Genes colored white were not included in the analysis.

## Slide 4
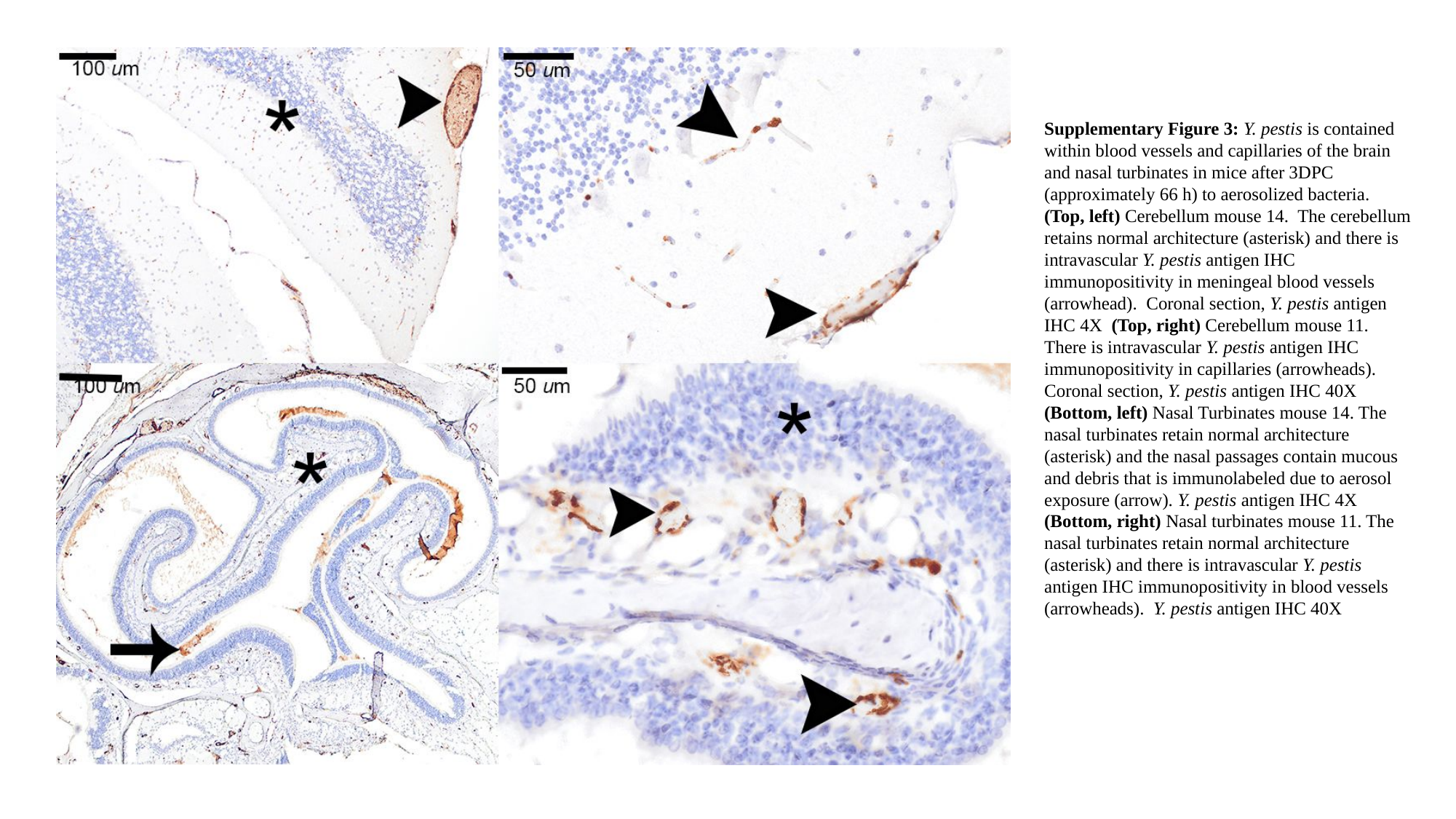

Supplementary Figure 3: Y. pestis is contained within blood vessels and capillaries of the brain and nasal turbinates in mice after 3DPC (approximately 66 h) to aerosolized bacteria. (Top, left) Cerebellum mouse 14. The cerebellum retains normal architecture (asterisk) and there is intravascular Y. pestis antigen IHC immunopositivity in meningeal blood vessels (arrowhead). Coronal section, Y. pestis antigen IHC 4X (Top, right) Cerebellum mouse 11. There is intravascular Y. pestis antigen IHC immunopositivity in capillaries (arrowheads). Coronal section, Y. pestis antigen IHC 40X (Bottom, left) Nasal Turbinates mouse 14. The nasal turbinates retain normal architecture (asterisk) and the nasal passages contain mucous and debris that is immunolabeled due to aerosol exposure (arrow). Y. pestis antigen IHC 4X (Bottom, right) Nasal turbinates mouse 11. The nasal turbinates retain normal architecture (asterisk) and there is intravascular Y. pestis antigen IHC immunopositivity in blood vessels (arrowheads). Y. pestis antigen IHC 40X

## Slide 5
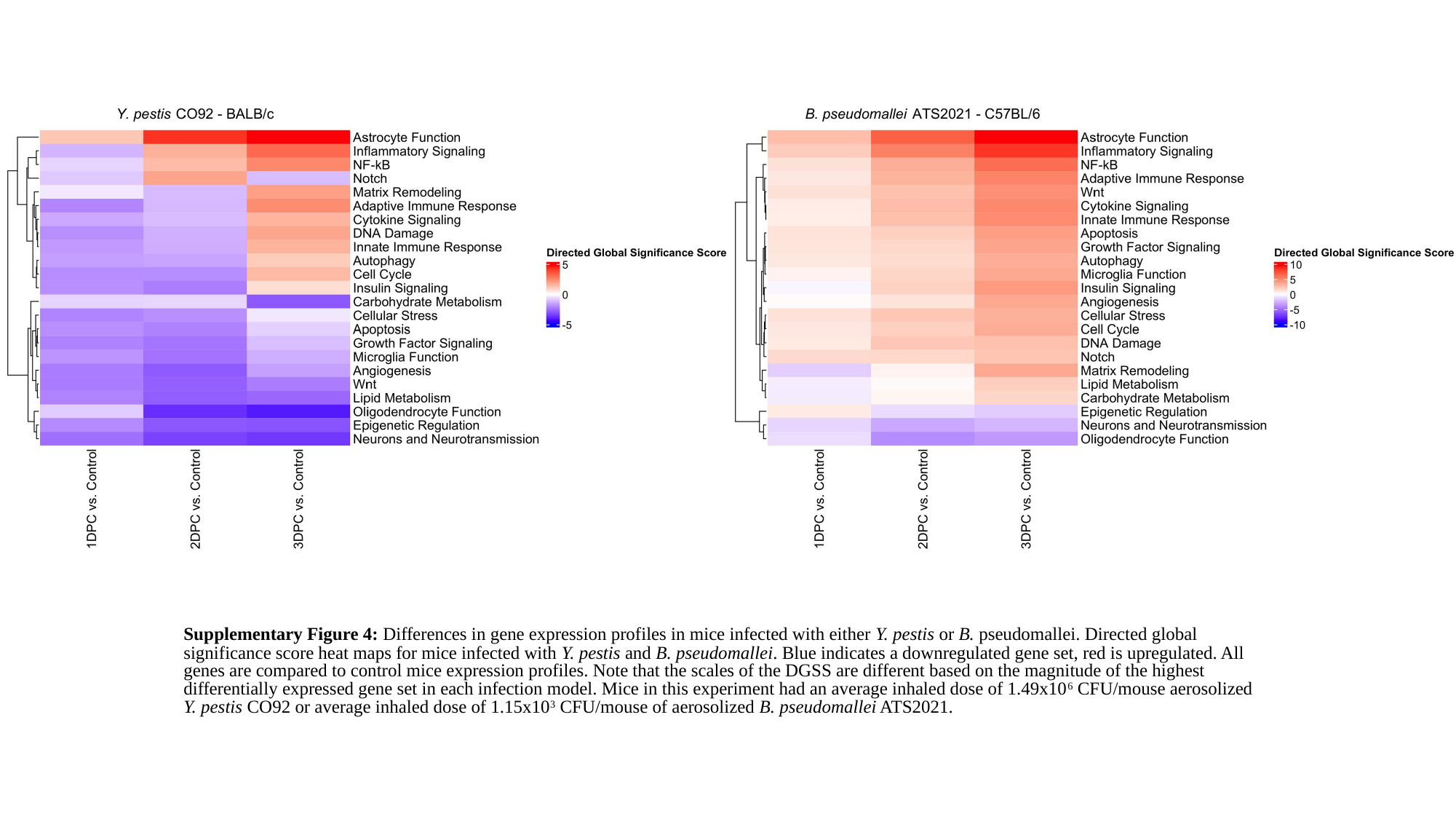

Supplementary Figure 4: Differences in gene expression profiles in mice infected with either Y. pestis or B. pseudomallei. Directed global significance score heat maps for mice infected with Y. pestis and B. pseudomallei. Blue indicates a downregulated gene set, red is upregulated. All genes are compared to control mice expression profiles. Note that the scales of the DGSS are different based on the magnitude of the highest differentially expressed gene set in each infection model. Mice in this experiment had an average inhaled dose of 1.49x10^6^ CFU/mouse aerosolized Y. pestis CO92 or average inhaled dose of 1.15x10^3^ CFU/mouse of aerosolized B. pseudomallei ATS2021.

## Slide 6
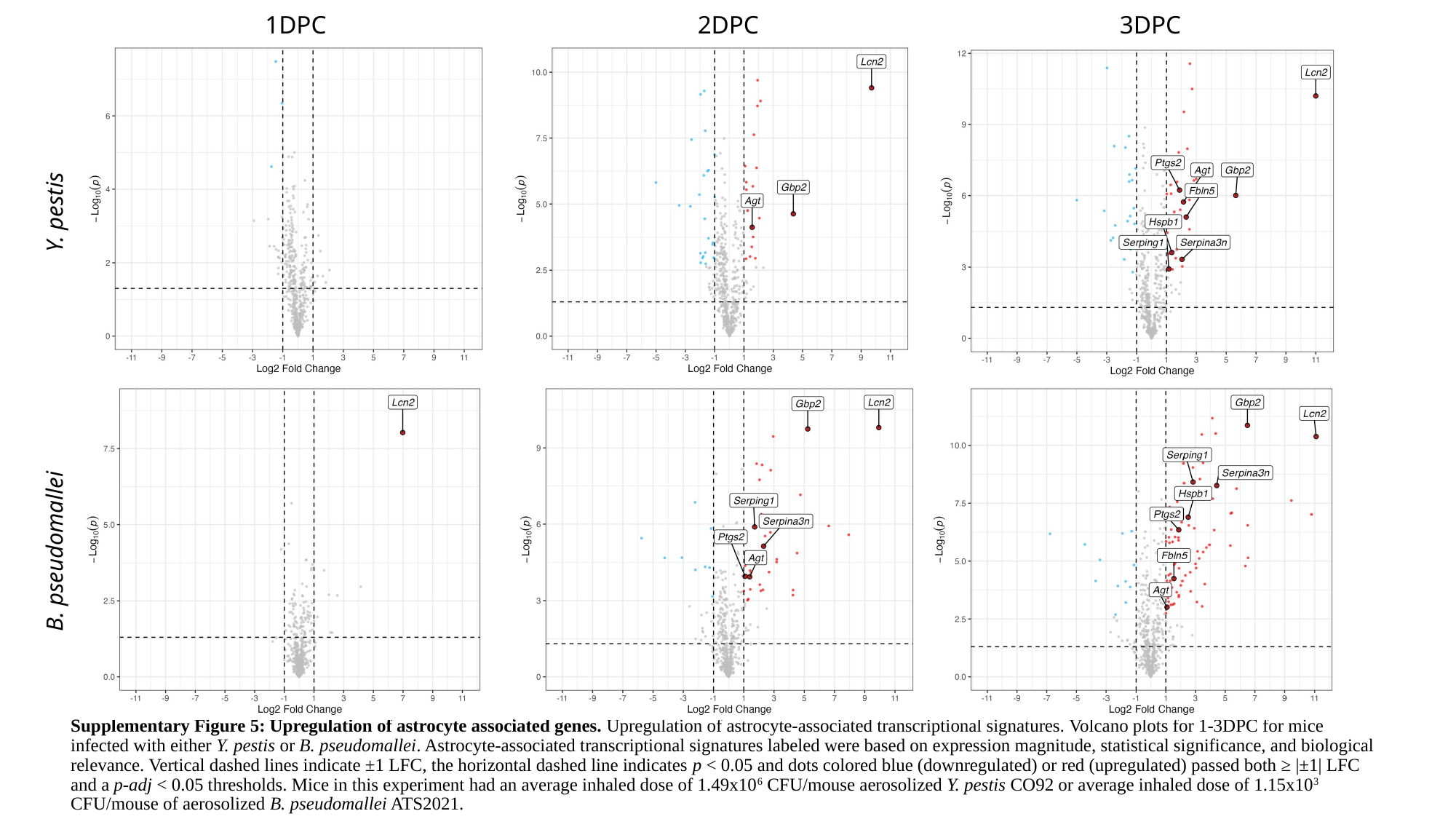

1DPC
2DPC
3DPC
Y. pestis
B. pseudomallei
Supplementary Figure 5: Upregulation of astrocyte associated genes. Upregulation of astrocyte-associated transcriptional signatures. Volcano plots for 1-3DPC for mice infected with either Y. pestis or B. pseudomallei. Astrocyte-associated transcriptional signatures labeled were based on expression magnitude, statistical significance, and biological relevance. Vertical dashed lines indicate ±1 LFC, the horizontal dashed line indicates p < 0.05 and dots colored blue (downregulated) or red (upregulated) passed both ≥ |±1| LFC and a p-adj < 0.05 thresholds. Mice in this experiment had an average inhaled dose of 1.49x10^6^ CFU/mouse aerosolized Y. pestis CO92 or average inhaled dose of 1.15x10^3^ CFU/mouse of aerosolized B. pseudomallei ATS2021.

## Slide 7
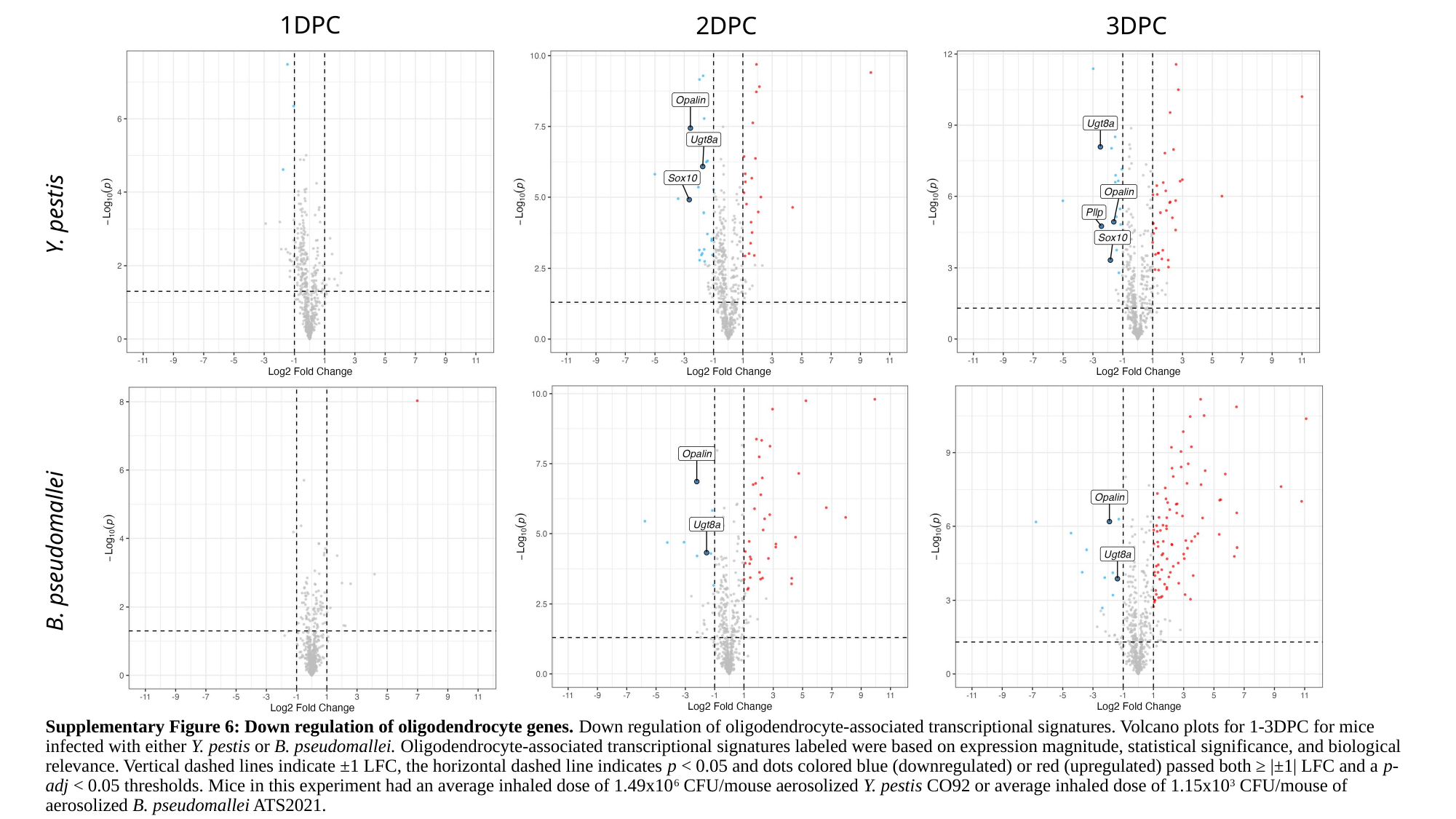

1DPC
2DPC
3DPC
Y. pestis
B. pseudomallei
Supplementary Figure 6: Down regulation of oligodendrocyte genes. Down regulation of oligodendrocyte-associated transcriptional signatures. Volcano plots for 1-3DPC for mice infected with either Y. pestis or B. pseudomallei. Oligodendrocyte-associated transcriptional signatures labeled were based on expression magnitude, statistical significance, and biological relevance. Vertical dashed lines indicate ±1 LFC, the horizontal dashed line indicates p < 0.05 and dots colored blue (downregulated) or red (upregulated) passed both ≥ |±1| LFC and a p-adj < 0.05 thresholds. Mice in this experiment had an average inhaled dose of 1.49x10^6^ CFU/mouse aerosolized Y. pestis CO92 or average inhaled dose of 1.15x10^3^ CFU/mouse of aerosolized B. pseudomallei ATS2021.

## Slide 8
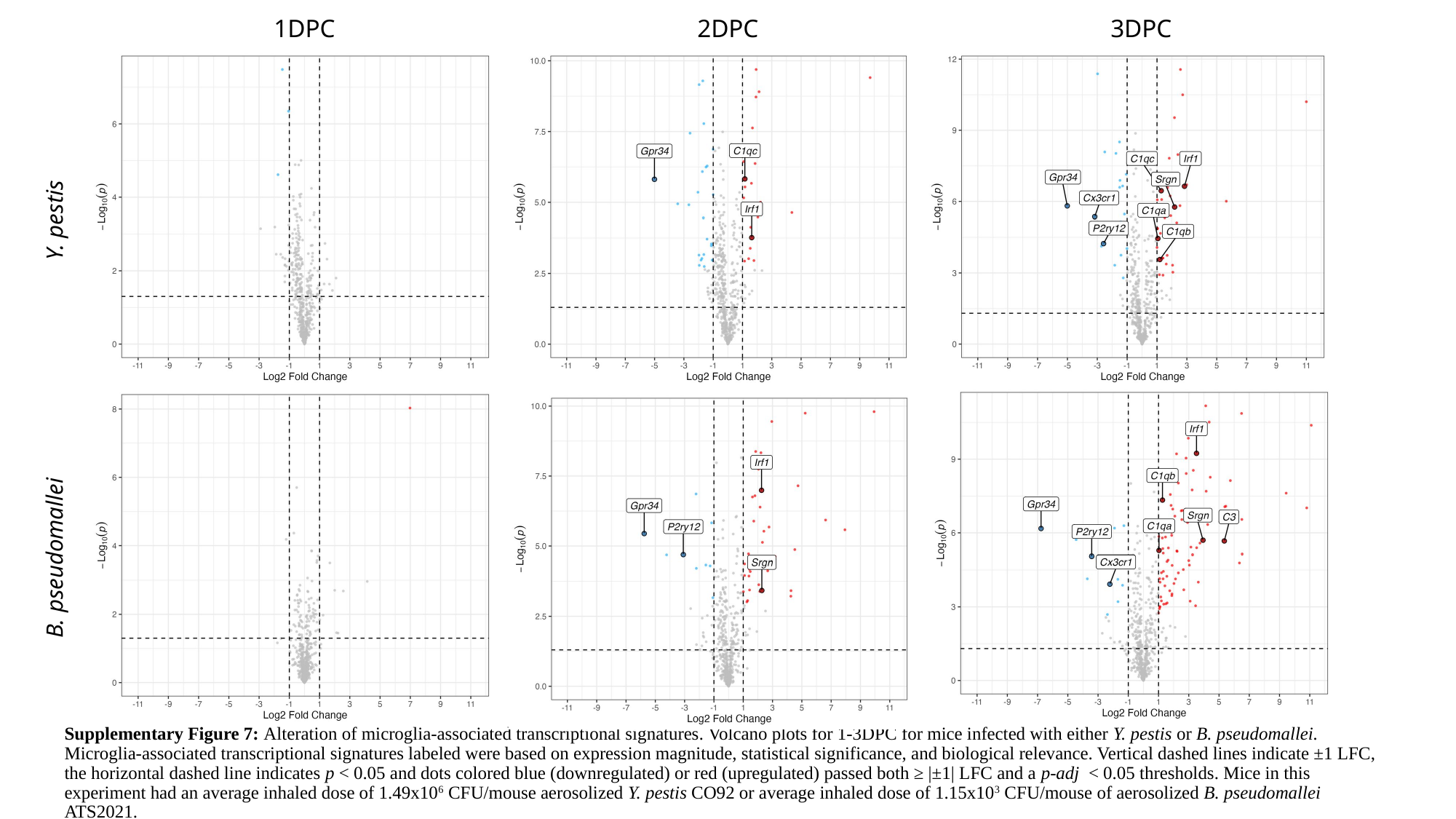

1DPC
3DPC
2DPC
Y. pestis
B. pseudomallei
Supplementary Figure 7: Alteration of microglia-associated transcriptional signatures. Volcano plots for 1-3DPC for mice infected with either Y. pestis or B. pseudomallei. Microglia-associated transcriptional signatures labeled were based on expression magnitude, statistical significance, and biological relevance. Vertical dashed lines indicate ±1 LFC, the horizontal dashed line indicates p < 0.05 and dots colored blue (downregulated) or red (upregulated) passed both ≥ |±1| LFC and a p-adj < 0.05 thresholds. Mice in this experiment had an average inhaled dose of 1.49x10^6^ CFU/mouse aerosolized Y. pestis CO92 or average inhaled dose of 1.15x10^3^ CFU/mouse of aerosolized B. pseudomallei ATS2021.
