# Supplementary material for: Vascular-associated bacterial burden and neuroinflammatory transcriptional responses observed in models of pneumonic plague
Source: Front Microbiol. 2026 Jun 24;17:1865125. doi: 10.3389/fmicb.2026.1865125 (PMC13341613; doi:10.3389/fmicb.2026.1865125)
Supplement: Supplementary file 1 [file Data_Sheet_1.ZIP › Yp_Final 2026-04-14 12-29/AdvAnalysisReport.html]

nCounter Advanced Analysis Report


- Overview
- Pathway Score
- Diff Expr
- GSA
- PathView
- Cell Type Profiling
- Analysis Parameters
- Share

This module generates a series of high level plots that describe the data overall and may be useful for identifying anomalous data and/or covariates.

Gene sets:

- Summary
- Adaptive Immune Response
- Angiogenesis
- Apoptosis
- Astrocyte Function
- Autophagy
- Carbohydrate Metabolism
- Cell Cycle
- Cellular Stress
- Cytokine Signaling
- DNA Damage
- Epigenetic Regulation
- Growth Factor Signaling
- Inflammatory Signaling
- Innate Immune Response
- Insulin Signaling
- Lipid Metabolism
- Matrix Remodeling
- Microglia Function
- Neurons and Neurotransmission
- NF-kB
- Notch
- Oligodendrocyte Function
- Wnt

- Heatmaps
- PCA
- Study design
- Other QC

- Heatmaps

- DPI

- Covariates

- Other QC

- **Heatmap of Raw Data  
  More Plot Information  Download detected/undetected calls data**


  ##### Heatmap of Raw Data

  Heatmap of the raw counts. The plot is meant to provide an overview of how robust the raw expression levels are across samples and gene sets. Datasets that entirely lack higher level expressions (e.g. counts > 100) may indicate experimental failure or low input. The detected/undetected calls links to a .csv file stating whether each probe is above background, with 0/1 indicating below/above background. If the user has not specified a detection threshold, probes are called detected if they have more than double the counts of the median negative control.
- **Heatmap of All Data  
  More Plot Information**


  ##### Heatmap of All Data

  Heatmap of the normalized data, scaled to give all genes equal variance, generated via unsupervised clustering. Orange indicates high expression; blue indicates low expression. This plot is meant to provide a high level exploratory view of the data.

- **Principal Components of All Data  
  More Plot Information**


  ##### Principal Components of All Data

  Principal component analysis maps high-dimensional datasets onto a smaller number of highly informative dimensions. Here, the first four principal components of the gene expression data are plotted against each other and colored by the values of the selected covariate. This plot may be used to identify clusters in the data and to identify variables associated with prominent signal in the data. Variables that are associated with these leading principal components should be considered in downstream analyses.
- **Outliers  
  More Plot Information**

  |  | Outlier on PC 1 | Outlier on PC 2 | Outlier on PC 3 | Outlier on PC 4 |
  | --- | --- | --- | --- | --- |
  | YP\_09.RCC | FALSE | FALSE | TRUE | FALSE |

  ##### Outliers

  Table identifying outliers in the first four PCs of the data.

- **More Plot Information**


  Pairwise comparisons of all covariates in the analysis. The type of plot is dependent on the types of variables compared; A categorical vs. categorical covariate plot is shown as a bar chart of counts (Y axis). Continuous vs. categorical covariates generate a boxplot with whiskers denoting 1.5 IQR. Continuous vs. continuous covariates are compared via a scatter plot. Variables that are correlated with a biological variable of interest are potential confounders that may influence downstream analyses. Additionally, bar plots and histograms show the distributions of categorical and continuous variables, respectively.

- **Variance vs. Mean normalized signal plot across all targets/probes  
  More Plot Information  Mean and Variance statistics across all genes**


  ##### Variance vs. Mean normalized signal plot across all targets/probes

  Each gene's variance in the log-scaled, normalized data is plotted against its mean value across all samples. Highly variable genes are indicated by gene name. Housekeeping genes are color coded according to their use in (or omission from) normalization.
- **p-value distribution plots  
  More Plot Information**


  ##### p-value distribution plots

  For each covariate included in the analysis, a histogram of p-values testing each gene's univariate association with the chosen covariate is displayed. Covariates with largely flat histograms have minimal association with gene expression; covariates with histograms with significantly more mass on the left are either associated with the expression of many genes or are confounded with a covariate that is associated with the expression. Low p-values indicate strong evidence for an association.

- Heatmaps
- PCA

- Heatmaps

- DPI

- **Heatmap of Adaptive Immune Response Data  
  More Plot Information**


  ##### Heatmap of Adaptive Immune Response Data

  Heatmap of the normalized data, scaled to give all genes equal variance, generated via unsupervised clustering. Orange indicates high expression; blue indicates low expression. This plot is meant to provide a high level exploratory view of the data.

- **Principal Components of Adaptive Immune Response Data  
  More Plot Information**


  ##### Principal Components of Adaptive Immune Response Data

  Principal component analysis maps high-dimensional datasets onto a smaller number of highly informative dimensions. Here, the first four principal components of the gene expression data are plotted against each other and colored by the values of the selected covariate. This plot may be used to identify clusters in the data and to identify variables associated with prominent signal in the data. Variables that are associated with these leading principal components should be considered in downstream analyses.
- **Outliers  
  More Plot Information**

  |  | Outlier on PC 1 | Outlier on PC 2 | Outlier on PC 3 | Outlier on PC 4 |
  | --- | --- | --- | --- | --- |
  | YP\_09.RCC | FALSE | FALSE | TRUE | FALSE |

  ##### Outliers

  Table identifying outliers in the first four PCs of the data.

- Heatmaps
- PCA

- Heatmaps

- DPI

- **Heatmap of Angiogenesis Data  
  More Plot Information**


  ##### Heatmap of Angiogenesis Data

  Heatmap of the normalized data, scaled to give all genes equal variance, generated via unsupervised clustering. Orange indicates high expression; blue indicates low expression. This plot is meant to provide a high level exploratory view of the data.

- **Principal Components of Angiogenesis Data  
  More Plot Information**


  ##### Principal Components of Angiogenesis Data

  Principal component analysis maps high-dimensional datasets onto a smaller number of highly informative dimensions. Here, the first four principal components of the gene expression data are plotted against each other and colored by the values of the selected covariate. This plot may be used to identify clusters in the data and to identify variables associated with prominent signal in the data. Variables that are associated with these leading principal components should be considered in downstream analyses.
- **Outliers  
  More Plot Information**

  |  | Outlier on PC 1 | Outlier on PC 2 | Outlier on PC 3 | Outlier on PC 4 |
  | --- | --- | --- | --- | --- |
  | YP\_09.RCC | FALSE | FALSE | TRUE | FALSE |

  ##### Outliers

  Table identifying outliers in the first four PCs of the data.

- Heatmaps
- PCA

- Heatmaps

- DPI

- **Heatmap of Apoptosis Data  
  More Plot Information**


  ##### Heatmap of Apoptosis Data

  Heatmap of the normalized data, scaled to give all genes equal variance, generated via unsupervised clustering. Orange indicates high expression; blue indicates low expression. This plot is meant to provide a high level exploratory view of the data.

- **Principal Components of Apoptosis Data  
  More Plot Information**


  ##### Principal Components of Apoptosis Data

  Principal component analysis maps high-dimensional datasets onto a smaller number of highly informative dimensions. Here, the first four principal components of the gene expression data are plotted against each other and colored by the values of the selected covariate. This plot may be used to identify clusters in the data and to identify variables associated with prominent signal in the data. Variables that are associated with these leading principal components should be considered in downstream analyses.
- **Outliers  
  More Plot Information**

  |  | Outlier on PC 1 | Outlier on PC 2 | Outlier on PC 3 | Outlier on PC 4 |
  | --- | --- | --- | --- | --- |
  | YP\_09.RCC | FALSE | FALSE | TRUE | FALSE |

  ##### Outliers

  Table identifying outliers in the first four PCs of the data.

- Heatmaps
- PCA

- Heatmaps

- DPI

- **Heatmap of Astrocyte Function Data  
  More Plot Information**


  ##### Heatmap of Astrocyte Function Data

  Heatmap of the normalized data, scaled to give all genes equal variance, generated via unsupervised clustering. Orange indicates high expression; blue indicates low expression. This plot is meant to provide a high level exploratory view of the data.

- **Principal Components of Astrocyte Function Data  
  More Plot Information**


  ##### Principal Components of Astrocyte Function Data

  Principal component analysis maps high-dimensional datasets onto a smaller number of highly informative dimensions. Here, the first four principal components of the gene expression data are plotted against each other and colored by the values of the selected covariate. This plot may be used to identify clusters in the data and to identify variables associated with prominent signal in the data. Variables that are associated with these leading principal components should be considered in downstream analyses.
- **Outliers  
  More Plot Information**

  |  | Outlier on PC 1 | Outlier on PC 2 | Outlier on PC 3 | Outlier on PC 4 |
  | --- | --- | --- | --- | --- |
  | YP\_09.RCC | FALSE | FALSE | TRUE | FALSE |

  ##### Outliers

  Table identifying outliers in the first four PCs of the data.

- Heatmaps
- PCA

- Heatmaps

- DPI

- **Heatmap of Autophagy Data  
  More Plot Information**


  ##### Heatmap of Autophagy Data

  Heatmap of the normalized data, scaled to give all genes equal variance, generated via unsupervised clustering. Orange indicates high expression; blue indicates low expression. This plot is meant to provide a high level exploratory view of the data.

- **Principal Components of Autophagy Data  
  More Plot Information**


  ##### Principal Components of Autophagy Data

  Principal component analysis maps high-dimensional datasets onto a smaller number of highly informative dimensions. Here, the first four principal components of the gene expression data are plotted against each other and colored by the values of the selected covariate. This plot may be used to identify clusters in the data and to identify variables associated with prominent signal in the data. Variables that are associated with these leading principal components should be considered in downstream analyses.
- **Outliers  
  More Plot Information**

  |  | Outlier on PC 1 | Outlier on PC 2 | Outlier on PC 3 | Outlier on PC 4 |
  | --- | --- | --- | --- | --- |
  | YP\_09.RCC | FALSE | FALSE | TRUE | FALSE |

  ##### Outliers

  Table identifying outliers in the first four PCs of the data.

- Heatmaps
- PCA

- Heatmaps

- DPI

- **Heatmap of Carbohydrate Metabolism Data  
  More Plot Information**


  ##### Heatmap of Carbohydrate Metabolism Data

  Heatmap of the normalized data, scaled to give all genes equal variance, generated via unsupervised clustering. Orange indicates high expression; blue indicates low expression. This plot is meant to provide a high level exploratory view of the data.

- **Principal Components of Carbohydrate Metabolism Data  
  More Plot Information**


  ##### Principal Components of Carbohydrate Metabolism Data

  Principal component analysis maps high-dimensional datasets onto a smaller number of highly informative dimensions. Here, the first four principal components of the gene expression data are plotted against each other and colored by the values of the selected covariate. This plot may be used to identify clusters in the data and to identify variables associated with prominent signal in the data. Variables that are associated with these leading principal components should be considered in downstream analyses.
- **Outliers  
  More Plot Information**

  |  | Outlier on PC 1 | Outlier on PC 2 | Outlier on PC 3 | Outlier on PC 4 |
  | --- | --- | --- | --- | --- |
  | YP\_09.RCC | FALSE | FALSE | TRUE | FALSE |

  ##### Outliers

  Table identifying outliers in the first four PCs of the data.

- Heatmaps
- PCA

- Heatmaps

- DPI

- **Heatmap of Cell Cycle Data  
  More Plot Information**


  ##### Heatmap of Cell Cycle Data

  Heatmap of the normalized data, scaled to give all genes equal variance, generated via unsupervised clustering. Orange indicates high expression; blue indicates low expression. This plot is meant to provide a high level exploratory view of the data.

- **Principal Components of Cell Cycle Data  
  More Plot Information**


  ##### Principal Components of Cell Cycle Data

  Principal component analysis maps high-dimensional datasets onto a smaller number of highly informative dimensions. Here, the first four principal components of the gene expression data are plotted against each other and colored by the values of the selected covariate. This plot may be used to identify clusters in the data and to identify variables associated with prominent signal in the data. Variables that are associated with these leading principal components should be considered in downstream analyses.
- **Outliers  
  More Plot Information**

  |  | Outlier on PC 1 | Outlier on PC 2 | Outlier on PC 3 | Outlier on PC 4 |
  | --- | --- | --- | --- | --- |
  | YP\_09.RCC | FALSE | FALSE | TRUE | FALSE |

  ##### Outliers

  Table identifying outliers in the first four PCs of the data.

- Heatmaps
- PCA

- Heatmaps

- DPI

- **Heatmap of Cellular Stress Data  
  More Plot Information**


  ##### Heatmap of Cellular Stress Data

  Heatmap of the normalized data, scaled to give all genes equal variance, generated via unsupervised clustering. Orange indicates high expression; blue indicates low expression. This plot is meant to provide a high level exploratory view of the data.

- **Principal Components of Cellular Stress Data  
  More Plot Information**


  ##### Principal Components of Cellular Stress Data

  Principal component analysis maps high-dimensional datasets onto a smaller number of highly informative dimensions. Here, the first four principal components of the gene expression data are plotted against each other and colored by the values of the selected covariate. This plot may be used to identify clusters in the data and to identify variables associated with prominent signal in the data. Variables that are associated with these leading principal components should be considered in downstream analyses.
- **Outliers  
  More Plot Information**

  |  | Outlier on PC 1 | Outlier on PC 2 | Outlier on PC 3 | Outlier on PC 4 |
  | --- | --- | --- | --- | --- |
  | YP\_09.RCC | FALSE | FALSE | TRUE | FALSE |

  ##### Outliers

  Table identifying outliers in the first four PCs of the data.

- Heatmaps
- PCA

- Heatmaps

- DPI

- **Heatmap of Cytokine Signaling Data  
  More Plot Information**


  ##### Heatmap of Cytokine Signaling Data

  Heatmap of the normalized data, scaled to give all genes equal variance, generated via unsupervised clustering. Orange indicates high expression; blue indicates low expression. This plot is meant to provide a high level exploratory view of the data.

- **Principal Components of Cytokine Signaling Data  
  More Plot Information**


  ##### Principal Components of Cytokine Signaling Data

  Principal component analysis maps high-dimensional datasets onto a smaller number of highly informative dimensions. Here, the first four principal components of the gene expression data are plotted against each other and colored by the values of the selected covariate. This plot may be used to identify clusters in the data and to identify variables associated with prominent signal in the data. Variables that are associated with these leading principal components should be considered in downstream analyses.
- **Outliers  
  More Plot Information**

  |  | Outlier on PC 1 | Outlier on PC 2 | Outlier on PC 3 | Outlier on PC 4 |
  | --- | --- | --- | --- | --- |
  | YP\_09.RCC | FALSE | FALSE | TRUE | FALSE |

  ##### Outliers

  Table identifying outliers in the first four PCs of the data.

- Heatmaps
- PCA

- Heatmaps

- DPI

- **Heatmap of DNA Damage Data  
  More Plot Information**


  ##### Heatmap of DNA Damage Data

  Heatmap of the normalized data, scaled to give all genes equal variance, generated via unsupervised clustering. Orange indicates high expression; blue indicates low expression. This plot is meant to provide a high level exploratory view of the data.

- **Principal Components of DNA Damage Data  
  More Plot Information**


  ##### Principal Components of DNA Damage Data

  Principal component analysis maps high-dimensional datasets onto a smaller number of highly informative dimensions. Here, the first four principal components of the gene expression data are plotted against each other and colored by the values of the selected covariate. This plot may be used to identify clusters in the data and to identify variables associated with prominent signal in the data. Variables that are associated with these leading principal components should be considered in downstream analyses.
- **Outliers  
  More Plot Information**

  |  | Outlier on PC 1 | Outlier on PC 2 | Outlier on PC 3 | Outlier on PC 4 |
  | --- | --- | --- | --- | --- |
  | YP\_09.RCC | FALSE | FALSE | TRUE | FALSE |

  ##### Outliers

  Table identifying outliers in the first four PCs of the data.

- Heatmaps
- PCA

- Heatmaps

- DPI

- **Heatmap of Epigenetic Regulation Data  
  More Plot Information**


  ##### Heatmap of Epigenetic Regulation Data

  Heatmap of the normalized data, scaled to give all genes equal variance, generated via unsupervised clustering. Orange indicates high expression; blue indicates low expression. This plot is meant to provide a high level exploratory view of the data.

- **Principal Components of Epigenetic Regulation Data  
  More Plot Information**


  ##### Principal Components of Epigenetic Regulation Data

  Principal component analysis maps high-dimensional datasets onto a smaller number of highly informative dimensions. Here, the first four principal components of the gene expression data are plotted against each other and colored by the values of the selected covariate. This plot may be used to identify clusters in the data and to identify variables associated with prominent signal in the data. Variables that are associated with these leading principal components should be considered in downstream analyses.
- **Outliers  
  More Plot Information**

  |  | Outlier on PC 1 | Outlier on PC 2 | Outlier on PC 3 | Outlier on PC 4 |
  | --- | --- | --- | --- | --- |
  | YP\_09.RCC | FALSE | FALSE | TRUE | FALSE |

  ##### Outliers

  Table identifying outliers in the first four PCs of the data.

- Heatmaps
- PCA

- Heatmaps

- DPI

- **Heatmap of Growth Factor Signaling Data  
  More Plot Information**


  ##### Heatmap of Growth Factor Signaling Data

  Heatmap of the normalized data, scaled to give all genes equal variance, generated via unsupervised clustering. Orange indicates high expression; blue indicates low expression. This plot is meant to provide a high level exploratory view of the data.

- **Principal Components of Growth Factor Signaling Data  
  More Plot Information**


  ##### Principal Components of Growth Factor Signaling Data

  Principal component analysis maps high-dimensional datasets onto a smaller number of highly informative dimensions. Here, the first four principal components of the gene expression data are plotted against each other and colored by the values of the selected covariate. This plot may be used to identify clusters in the data and to identify variables associated with prominent signal in the data. Variables that are associated with these leading principal components should be considered in downstream analyses.
- **Outliers  
  More Plot Information**

  |  | Outlier on PC 1 | Outlier on PC 2 | Outlier on PC 3 | Outlier on PC 4 |
  | --- | --- | --- | --- | --- |
  | YP\_09.RCC | FALSE | FALSE | TRUE | FALSE |

  ##### Outliers

  Table identifying outliers in the first four PCs of the data.

- Heatmaps
- PCA

- Heatmaps

- DPI

- **Heatmap of Inflammatory Signaling Data  
  More Plot Information**


  ##### Heatmap of Inflammatory Signaling Data

  Heatmap of the normalized data, scaled to give all genes equal variance, generated via unsupervised clustering. Orange indicates high expression; blue indicates low expression. This plot is meant to provide a high level exploratory view of the data.

- **Principal Components of Inflammatory Signaling Data  
  More Plot Information**


  ##### Principal Components of Inflammatory Signaling Data

  Principal component analysis maps high-dimensional datasets onto a smaller number of highly informative dimensions. Here, the first four principal components of the gene expression data are plotted against each other and colored by the values of the selected covariate. This plot may be used to identify clusters in the data and to identify variables associated with prominent signal in the data. Variables that are associated with these leading principal components should be considered in downstream analyses.
- **Outliers  
  More Plot Information**

  |  | Outlier on PC 1 | Outlier on PC 2 | Outlier on PC 3 | Outlier on PC 4 |
  | --- | --- | --- | --- | --- |
  | YP\_09.RCC | FALSE | FALSE | TRUE | FALSE |

  ##### Outliers

  Table identifying outliers in the first four PCs of the data.

- Heatmaps
- PCA

- Heatmaps

- DPI

- **Heatmap of Innate Immune Response Data  
  More Plot Information**


  ##### Heatmap of Innate Immune Response Data

  Heatmap of the normalized data, scaled to give all genes equal variance, generated via unsupervised clustering. Orange indicates high expression; blue indicates low expression. This plot is meant to provide a high level exploratory view of the data.

- **Principal Components of Innate Immune Response Data  
  More Plot Information**


  ##### Principal Components of Innate Immune Response Data

  Principal component analysis maps high-dimensional datasets onto a smaller number of highly informative dimensions. Here, the first four principal components of the gene expression data are plotted against each other and colored by the values of the selected covariate. This plot may be used to identify clusters in the data and to identify variables associated with prominent signal in the data. Variables that are associated with these leading principal components should be considered in downstream analyses.
- **Outliers  
  More Plot Information**

  |  | Outlier on PC 1 | Outlier on PC 2 | Outlier on PC 3 | Outlier on PC 4 |
  | --- | --- | --- | --- | --- |
  | YP\_09.RCC | FALSE | FALSE | TRUE | FALSE |

  ##### Outliers

  Table identifying outliers in the first four PCs of the data.

- Heatmaps
- PCA

- Heatmaps

- DPI

- **Heatmap of Insulin Signaling Data  
  More Plot Information**


  ##### Heatmap of Insulin Signaling Data

  Heatmap of the normalized data, scaled to give all genes equal variance, generated via unsupervised clustering. Orange indicates high expression; blue indicates low expression. This plot is meant to provide a high level exploratory view of the data.

- **Principal Components of Insulin Signaling Data  
  More Plot Information**


  ##### Principal Components of Insulin Signaling Data

  Principal component analysis maps high-dimensional datasets onto a smaller number of highly informative dimensions. Here, the first four principal components of the gene expression data are plotted against each other and colored by the values of the selected covariate. This plot may be used to identify clusters in the data and to identify variables associated with prominent signal in the data. Variables that are associated with these leading principal components should be considered in downstream analyses.
- **Outliers  
  More Plot Information**

  |  | Outlier on PC 1 | Outlier on PC 2 | Outlier on PC 3 | Outlier on PC 4 |
  | --- | --- | --- | --- | --- |
  | YP\_09.RCC | FALSE | FALSE | TRUE | FALSE |

  ##### Outliers

  Table identifying outliers in the first four PCs of the data.

- Heatmaps
- PCA

- Heatmaps

- DPI

- **Heatmap of Lipid Metabolism Data  
  More Plot Information**


  ##### Heatmap of Lipid Metabolism Data

  Heatmap of the normalized data, scaled to give all genes equal variance, generated via unsupervised clustering. Orange indicates high expression; blue indicates low expression. This plot is meant to provide a high level exploratory view of the data.

- **Principal Components of Lipid Metabolism Data  
  More Plot Information**


  ##### Principal Components of Lipid Metabolism Data

  Principal component analysis maps high-dimensional datasets onto a smaller number of highly informative dimensions. Here, the first four principal components of the gene expression data are plotted against each other and colored by the values of the selected covariate. This plot may be used to identify clusters in the data and to identify variables associated with prominent signal in the data. Variables that are associated with these leading principal components should be considered in downstream analyses.
- **Outliers  
  More Plot Information**

  |  | Outlier on PC 1 | Outlier on PC 2 | Outlier on PC 3 | Outlier on PC 4 |
  | --- | --- | --- | --- | --- |
  | YP\_09.RCC | FALSE | FALSE | TRUE | FALSE |

  ##### Outliers

  Table identifying outliers in the first four PCs of the data.

- Heatmaps
- PCA

- Heatmaps

- DPI

- **Heatmap of Matrix Remodeling Data  
  More Plot Information**


  ##### Heatmap of Matrix Remodeling Data

  Heatmap of the normalized data, scaled to give all genes equal variance, generated via unsupervised clustering. Orange indicates high expression; blue indicates low expression. This plot is meant to provide a high level exploratory view of the data.

- **Principal Components of Matrix Remodeling Data  
  More Plot Information**


  ##### Principal Components of Matrix Remodeling Data

  Principal component analysis maps high-dimensional datasets onto a smaller number of highly informative dimensions. Here, the first four principal components of the gene expression data are plotted against each other and colored by the values of the selected covariate. This plot may be used to identify clusters in the data and to identify variables associated with prominent signal in the data. Variables that are associated with these leading principal components should be considered in downstream analyses.
- **Outliers  
  More Plot Information**

  |  | Outlier on PC 1 | Outlier on PC 2 | Outlier on PC 3 | Outlier on PC 4 |
  | --- | --- | --- | --- | --- |
  | YP\_09.RCC | FALSE | FALSE | TRUE | FALSE |

  ##### Outliers

  Table identifying outliers in the first four PCs of the data.

- Heatmaps
- PCA

- Heatmaps

- DPI

- **Heatmap of Microglia Function Data  
  More Plot Information**


  ##### Heatmap of Microglia Function Data

  Heatmap of the normalized data, scaled to give all genes equal variance, generated via unsupervised clustering. Orange indicates high expression; blue indicates low expression. This plot is meant to provide a high level exploratory view of the data.

- **Principal Components of Microglia Function Data  
  More Plot Information**


  ##### Principal Components of Microglia Function Data

  Principal component analysis maps high-dimensional datasets onto a smaller number of highly informative dimensions. Here, the first four principal components of the gene expression data are plotted against each other and colored by the values of the selected covariate. This plot may be used to identify clusters in the data and to identify variables associated with prominent signal in the data. Variables that are associated with these leading principal components should be considered in downstream analyses.
- **Outliers  
  More Plot Information**

  |  | Outlier on PC 1 | Outlier on PC 2 | Outlier on PC 3 | Outlier on PC 4 |
  | --- | --- | --- | --- | --- |
  | YP\_09.RCC | FALSE | FALSE | TRUE | FALSE |

  ##### Outliers

  Table identifying outliers in the first four PCs of the data.

- Heatmaps
- PCA

- Heatmaps

- DPI

- **Heatmap of Neurons and Neurotransmission Data  
  More Plot Information**


  ##### Heatmap of Neurons and Neurotransmission Data

  Heatmap of the normalized data, scaled to give all genes equal variance, generated via unsupervised clustering. Orange indicates high expression; blue indicates low expression. This plot is meant to provide a high level exploratory view of the data.

- **Principal Components of Neurons and Neurotransmission Data  
  More Plot Information**


  ##### Principal Components of Neurons and Neurotransmission Data

  Principal component analysis maps high-dimensional datasets onto a smaller number of highly informative dimensions. Here, the first four principal components of the gene expression data are plotted against each other and colored by the values of the selected covariate. This plot may be used to identify clusters in the data and to identify variables associated with prominent signal in the data. Variables that are associated with these leading principal components should be considered in downstream analyses.
- **Outliers  
  More Plot Information**

  |  | Outlier on PC 1 | Outlier on PC 2 | Outlier on PC 3 | Outlier on PC 4 |
  | --- | --- | --- | --- | --- |
  | YP\_09.RCC | FALSE | FALSE | TRUE | FALSE |

  ##### Outliers

  Table identifying outliers in the first four PCs of the data.

- Heatmaps
- PCA

- Heatmaps

- DPI

- **Heatmap of NF-kB Data  
  More Plot Information**


  ##### Heatmap of NF-kB Data

  Heatmap of the normalized data, scaled to give all genes equal variance, generated via unsupervised clustering. Orange indicates high expression; blue indicates low expression. This plot is meant to provide a high level exploratory view of the data.

- **Principal Components of NF-kB Data  
  More Plot Information**


  ##### Principal Components of NF-kB Data

  Principal component analysis maps high-dimensional datasets onto a smaller number of highly informative dimensions. Here, the first four principal components of the gene expression data are plotted against each other and colored by the values of the selected covariate. This plot may be used to identify clusters in the data and to identify variables associated with prominent signal in the data. Variables that are associated with these leading principal components should be considered in downstream analyses.
- **Outliers  
  More Plot Information**

  |  | Outlier on PC 1 | Outlier on PC 2 | Outlier on PC 3 | Outlier on PC 4 |
  | --- | --- | --- | --- | --- |
  | YP\_09.RCC | FALSE | FALSE | TRUE | FALSE |

  ##### Outliers

  Table identifying outliers in the first four PCs of the data.

- Heatmaps
- PCA

- Heatmaps

- DPI

- **Heatmap of Notch Data  
  More Plot Information**


  ##### Heatmap of Notch Data

  Heatmap of the normalized data, scaled to give all genes equal variance, generated via unsupervised clustering. Orange indicates high expression; blue indicates low expression. This plot is meant to provide a high level exploratory view of the data.

- **Principal Components of Notch Data  
  More Plot Information**


  ##### Principal Components of Notch Data

  Principal component analysis maps high-dimensional datasets onto a smaller number of highly informative dimensions. Here, the first four principal components of the gene expression data are plotted against each other and colored by the values of the selected covariate. This plot may be used to identify clusters in the data and to identify variables associated with prominent signal in the data. Variables that are associated with these leading principal components should be considered in downstream analyses.
- **Outliers  
  More Plot Information**

  |  | Outlier on PC 1 | Outlier on PC 2 | Outlier on PC 3 | Outlier on PC 4 |
  | --- | --- | --- | --- | --- |
  | YP\_09.RCC | FALSE | FALSE | TRUE | FALSE |

  ##### Outliers

  Table identifying outliers in the first four PCs of the data.

- Heatmaps
- PCA

- Heatmaps

- DPI

- **Heatmap of Oligodendrocyte Function Data  
  More Plot Information**


  ##### Heatmap of Oligodendrocyte Function Data

  Heatmap of the normalized data, scaled to give all genes equal variance, generated via unsupervised clustering. Orange indicates high expression; blue indicates low expression. This plot is meant to provide a high level exploratory view of the data.

- **Principal Components of Oligodendrocyte Function Data  
  More Plot Information**


  ##### Principal Components of Oligodendrocyte Function Data

  Principal component analysis maps high-dimensional datasets onto a smaller number of highly informative dimensions. Here, the first four principal components of the gene expression data are plotted against each other and colored by the values of the selected covariate. This plot may be used to identify clusters in the data and to identify variables associated with prominent signal in the data. Variables that are associated with these leading principal components should be considered in downstream analyses.
- **Outliers  
  More Plot Information**

  |  | Outlier on PC 1 | Outlier on PC 2 | Outlier on PC 3 | Outlier on PC 4 |
  | --- | --- | --- | --- | --- |
  | YP\_09.RCC | FALSE | FALSE | TRUE | FALSE |

  ##### Outliers

  Table identifying outliers in the first four PCs of the data.

- Heatmaps
- PCA

- Heatmaps

- DPI

- **Heatmap of Wnt Data  
  More Plot Information**


  ##### Heatmap of Wnt Data

  Heatmap of the normalized data, scaled to give all genes equal variance, generated via unsupervised clustering. Orange indicates high expression; blue indicates low expression. This plot is meant to provide a high level exploratory view of the data.

- **Principal Components of Wnt Data  
  More Plot Information**


  ##### Principal Components of Wnt Data

  Principal component analysis maps high-dimensional datasets onto a smaller number of highly informative dimensions. Here, the first four principal components of the gene expression data are plotted against each other and colored by the values of the selected covariate. This plot may be used to identify clusters in the data and to identify variables associated with prominent signal in the data. Variables that are associated with these leading principal components should be considered in downstream analyses.
- **Outliers  
  More Plot Information**

  |  | Outlier on PC 1 | Outlier on PC 2 | Outlier on PC 3 | Outlier on PC 4 |
  | --- | --- | --- | --- | --- |
  | YP\_09.RCC | FALSE | FALSE | TRUE | FALSE |

  ##### Outliers

  Table identifying outliers in the first four PCs of the data.

Pathway scores condense each sample's gene expression profile into a small set of pathway scores. An experiment can then be explored through the lens of pathway scores instead of in the much higher-dimension lens of gene expression values. Pathway scores are fit using the first principal component of each gene set's data. They are oriented such that increasing score corresponds to mostly increasing expression (specifically, each pathway score has positive weights for at least half its genes).
Summary plots explore the joint behavior of pathways, and Covariates plots compare pathway scores to covariates.

- Summary
- Covariates

- score

- score

Choose a Pathway:

- Summary
- Adaptive Immune Response
- Angiogenesis
- Apoptosis
- Astrocyte Function
- Autophagy
- Carbohydrate Metabolism
- Cell Cycle
- Cellular Stress
- Cytokine Signaling
- DNA Damage
- Epigenetic Regulation
- Growth Factor Signaling
- Inflammatory Signaling
- Innate Immune Response
- Insulin Signaling
- Lipid Metabolism
- Matrix Remodeling
- Microglia Function
- Neurons and Neurotransmission
- NF-kB
- Notch
- Oligodendrocyte Function
- Wnt

- **Heatmap of Pathway scores  
  More Plot Information  Download Pathway scores**


  ##### Heatmap of Pathway scores

  Heatmap showing pathway score. This plot is a high level overview of how the pathway scores change across samples. Use it to understand how pathway scores cluster together and which samples exhibit similar pathway score profiles. Orange indicates high scores; blue indicates low scores. Scores are displayed on the same scale via a Z-transformation.
- **Heatmap of Correlation Matrix of Pathway scores  
  More Plot Information**


  ##### Heatmap of Correlation Matrix of Pathway scores

  Heatmap showing the correlation matrix of pathway scores. Orange and blue indicate positive and negative correlation, respectively.

- **Adaptive Immune Response measurements vs. other pathway scores  
  More Plot Information**


  ##### Adaptive Immune Response measurements vs. other pathway scores

  Each panel plots Adaptive Immune Response against another pathway score. Points are colored by DPI.

- **Angiogenesis measurements vs. other pathway scores  
  More Plot Information**


  ##### Angiogenesis measurements vs. other pathway scores

  Each panel plots Angiogenesis against another pathway score. Points are colored by DPI.

- **Apoptosis measurements vs. other pathway scores  
  More Plot Information**


  ##### Apoptosis measurements vs. other pathway scores

  Each panel plots Apoptosis against another pathway score. Points are colored by DPI.

- **Astrocyte Function measurements vs. other pathway scores  
  More Plot Information**


  ##### Astrocyte Function measurements vs. other pathway scores

  Each panel plots Astrocyte Function against another pathway score. Points are colored by DPI.

- **Autophagy measurements vs. other pathway scores  
  More Plot Information**


  ##### Autophagy measurements vs. other pathway scores

  Each panel plots Autophagy against another pathway score. Points are colored by DPI.

- **Carbohydrate Metabolism measurements vs. other pathway scores  
  More Plot Information**


  ##### Carbohydrate Metabolism measurements vs. other pathway scores

  Each panel plots Carbohydrate Metabolism against another pathway score. Points are colored by DPI.

- **Cell Cycle measurements vs. other pathway scores  
  More Plot Information**


  ##### Cell Cycle measurements vs. other pathway scores

  Each panel plots Cell Cycle against another pathway score. Points are colored by DPI.

- **Cellular Stress measurements vs. other pathway scores  
  More Plot Information**


  ##### Cellular Stress measurements vs. other pathway scores

  Each panel plots Cellular Stress against another pathway score. Points are colored by DPI.

- **Cytokine Signaling measurements vs. other pathway scores  
  More Plot Information**


  ##### Cytokine Signaling measurements vs. other pathway scores

  Each panel plots Cytokine Signaling against another pathway score. Points are colored by DPI.

- **DNA Damage measurements vs. other pathway scores  
  More Plot Information**


  ##### DNA Damage measurements vs. other pathway scores

  Each panel plots DNA Damage against another pathway score. Points are colored by DPI.

- **Epigenetic Regulation measurements vs. other pathway scores  
  More Plot Information**


  ##### Epigenetic Regulation measurements vs. other pathway scores

  Each panel plots Epigenetic Regulation against another pathway score. Points are colored by DPI.

- **Growth Factor Signaling measurements vs. other pathway scores  
  More Plot Information**


  ##### Growth Factor Signaling measurements vs. other pathway scores

  Each panel plots Growth Factor Signaling against another pathway score. Points are colored by DPI.

- **Inflammatory Signaling measurements vs. other pathway scores  
  More Plot Information**


  ##### Inflammatory Signaling measurements vs. other pathway scores

  Each panel plots Inflammatory Signaling against another pathway score. Points are colored by DPI.

- **Innate Immune Response measurements vs. other pathway scores  
  More Plot Information**


  ##### Innate Immune Response measurements vs. other pathway scores

  Each panel plots Innate Immune Response against another pathway score. Points are colored by DPI.

- **Insulin Signaling measurements vs. other pathway scores  
  More Plot Information**


  ##### Insulin Signaling measurements vs. other pathway scores

  Each panel plots Insulin Signaling against another pathway score. Points are colored by DPI.

- **Lipid Metabolism measurements vs. other pathway scores  
  More Plot Information**


  ##### Lipid Metabolism measurements vs. other pathway scores

  Each panel plots Lipid Metabolism against another pathway score. Points are colored by DPI.

- **Matrix Remodeling measurements vs. other pathway scores  
  More Plot Information**


  ##### Matrix Remodeling measurements vs. other pathway scores

  Each panel plots Matrix Remodeling against another pathway score. Points are colored by DPI.

- **Microglia Function measurements vs. other pathway scores  
  More Plot Information**


  ##### Microglia Function measurements vs. other pathway scores

  Each panel plots Microglia Function against another pathway score. Points are colored by DPI.

- **Neurons and Neurotransmission measurements vs. other pathway scores  
  More Plot Information**


  ##### Neurons and Neurotransmission measurements vs. other pathway scores

  Each panel plots Neurons and Neurotransmission against another pathway score. Points are colored by DPI.

- **NF-kB measurements vs. other pathway scores  
  More Plot Information**


  ##### NF-kB measurements vs. other pathway scores

  Each panel plots NF-kB against another pathway score. Points are colored by DPI.

- **Notch measurements vs. other pathway scores  
  More Plot Information**


  ##### Notch measurements vs. other pathway scores

  Each panel plots Notch against another pathway score. Points are colored by DPI.

- **Oligodendrocyte Function measurements vs. other pathway scores  
  More Plot Information**


  ##### Oligodendrocyte Function measurements vs. other pathway scores

  Each panel plots Oligodendrocyte Function against another pathway score. Points are colored by DPI.

- **Wnt measurements vs. other pathway scores  
  More Plot Information**


  ##### Wnt measurements vs. other pathway scores

  Each panel plots Wnt against another pathway score. Points are colored by DPI.

Choose a Pathway:

- Summary
- Adaptive Immune Response
- Angiogenesis
- Apoptosis
- Astrocyte Function
- Autophagy
- Carbohydrate Metabolism
- Cell Cycle
- Cellular Stress
- Cytokine Signaling
- DNA Damage
- Epigenetic Regulation
- Growth Factor Signaling
- Inflammatory Signaling
- Innate Immune Response
- Insulin Signaling
- Lipid Metabolism
- Matrix Remodeling
- Microglia Function
- Neurons and Neurotransmission
- NF-kB
- Notch
- Oligodendrocyte Function
- Wnt

- **Pathway scores vs. DPI  
  More Plot Information**


  ##### Pathway scores vs. DPI

  Plots the pathway scores against DPI. The pathway scores are plotted to show how they vary across different values of DPI conditions. Lines show each pathway's average score across values of DPI.

- **Adaptive Immune Response pathway scores vs. DPI  
  More Plot Information**


  ##### Adaptive Immune Response pathway scores vs. DPI

  Plots Adaptive Immune Response pathway scores against DPI.

- **Angiogenesis pathway scores vs. DPI  
  More Plot Information**


  ##### Angiogenesis pathway scores vs. DPI

  Plots Angiogenesis pathway scores against DPI.

- **Apoptosis pathway scores vs. DPI  
  More Plot Information**


  ##### Apoptosis pathway scores vs. DPI

  Plots Apoptosis pathway scores against DPI.

- **Astrocyte Function pathway scores vs. DPI  
  More Plot Information**


  ##### Astrocyte Function pathway scores vs. DPI

  Plots Astrocyte Function pathway scores against DPI.

- **Autophagy pathway scores vs. DPI  
  More Plot Information**


  ##### Autophagy pathway scores vs. DPI

  Plots Autophagy pathway scores against DPI.

- **Carbohydrate Metabolism pathway scores vs. DPI  
  More Plot Information**


  ##### Carbohydrate Metabolism pathway scores vs. DPI

  Plots Carbohydrate Metabolism pathway scores against DPI.

- **Cell Cycle pathway scores vs. DPI  
  More Plot Information**


  ##### Cell Cycle pathway scores vs. DPI

  Plots Cell Cycle pathway scores against DPI.

- **Cellular Stress pathway scores vs. DPI  
  More Plot Information**


  ##### Cellular Stress pathway scores vs. DPI

  Plots Cellular Stress pathway scores against DPI.

- **Cytokine Signaling pathway scores vs. DPI  
  More Plot Information**


  ##### Cytokine Signaling pathway scores vs. DPI

  Plots Cytokine Signaling pathway scores against DPI.

- **DNA Damage pathway scores vs. DPI  
  More Plot Information**


  ##### DNA Damage pathway scores vs. DPI

  Plots DNA Damage pathway scores against DPI.

- **Epigenetic Regulation pathway scores vs. DPI  
  More Plot Information**


  ##### Epigenetic Regulation pathway scores vs. DPI

  Plots Epigenetic Regulation pathway scores against DPI.

- **Growth Factor Signaling pathway scores vs. DPI  
  More Plot Information**


  ##### Growth Factor Signaling pathway scores vs. DPI

  Plots Growth Factor Signaling pathway scores against DPI.

- **Inflammatory Signaling pathway scores vs. DPI  
  More Plot Information**


  ##### Inflammatory Signaling pathway scores vs. DPI

  Plots Inflammatory Signaling pathway scores against DPI.

- **Innate Immune Response pathway scores vs. DPI  
  More Plot Information**


  ##### Innate Immune Response pathway scores vs. DPI

  Plots Innate Immune Response pathway scores against DPI.

- **Insulin Signaling pathway scores vs. DPI  
  More Plot Information**


  ##### Insulin Signaling pathway scores vs. DPI

  Plots Insulin Signaling pathway scores against DPI.

- **Lipid Metabolism pathway scores vs. DPI  
  More Plot Information**


  ##### Lipid Metabolism pathway scores vs. DPI

  Plots Lipid Metabolism pathway scores against DPI.

- **Matrix Remodeling pathway scores vs. DPI  
  More Plot Information**


  ##### Matrix Remodeling pathway scores vs. DPI

  Plots Matrix Remodeling pathway scores against DPI.

- **Microglia Function pathway scores vs. DPI  
  More Plot Information**


  ##### Microglia Function pathway scores vs. DPI

  Plots Microglia Function pathway scores against DPI.

- **Neurons and Neurotransmission pathway scores vs. DPI  
  More Plot Information**


  ##### Neurons and Neurotransmission pathway scores vs. DPI

  Plots Neurons and Neurotransmission pathway scores against DPI.

- **NF-kB pathway scores vs. DPI  
  More Plot Information**


  ##### NF-kB pathway scores vs. DPI

  Plots NF-kB pathway scores against DPI.

- **Notch pathway scores vs. DPI  
  More Plot Information**


  ##### Notch pathway scores vs. DPI

  Plots Notch pathway scores against DPI.

- **Oligodendrocyte Function pathway scores vs. DPI  
  More Plot Information**


  ##### Oligodendrocyte Function pathway scores vs. DPI

  Plots Oligodendrocyte Function pathway scores against DPI.

- **Wnt pathway scores vs. DPI  
  More Plot Information**


  ##### Wnt pathway scores vs. DPI

  Plots Wnt pathway scores against DPI.

Genes are tested for differential expression in response to each selected covariate. For each gene, a single linear
regression is fit using all selected covariates to predict expression. This approach eliminates confounding due to measured
covariates and isolates the independent association of each covariate with gene expression, measuring each variable's
association with a gene after holding all other variables constant.

DPI:
- DPI: differential expression in 1 vs. baseline of CTRL
- DPI: differential expression in 2 vs. baseline of CTRL
- DPI: differential expression in 3 vs. baseline of CTRL

- **Volcano Plot: DPI: 1 vs.CTRL  
  More Plot Information**


  ##### Volcano Plot: DPI: 1 vs.CTRL

  Volcano plot displaying each gene's -log10(p-value) and log2 fold change with the selected covariate. Highly statistically significant genes fall at the top of the plot above the horizontal lines, and highly differentially expressed genes fall to either side. Horizontal lines indicate various False Discovery Rate (FDR) thresholds or p-value thresholds if there is no adjustment to the p-values. Genes are colored if the resulting p-value is below the given FDR or p-value threshold. The 40 most statistically significant genes are labeled in the plot.
- **DE Results: DPI: 1 vs.CTRL  
  More Plot Information  Download CSV Data**

  |  | Log2 fold change | std error (log2) | Lower confidence limit (log2) | Upper confidence limit (log2) | Linear fold change | Lower confidence limit (linear) | Upper confidence limit (linear) | P-value | BY.p.value | method | Gene.sets | probe.ID |
  | --- | --- | --- | --- | --- | --- | --- | --- | --- | --- | --- | --- | --- |
  | Arc-mRNA | -1.46 | 0.148 | -1.75 | -1.17 | 0.363 | 0.297 | 0.444 | 3.28e-08 | 0.000116 | lm.nb | Neurons and Neurotransmission | NM\_018790.2:2715 |
  | Egr1-mRNA | -1.05 | 0.129 | -1.3 | -0.798 | 0.483 | 0.405 | 0.575 | 4.51e-07 | 0.000796 | lm.nb | Inflammatory Signaling, Neurons and Neurotransmission | NM\_007913.5:515 |
  | Rab6b-mRNA | -0.226 | 0.0357 | -0.296 | -0.156 | 0.855 | 0.814 | 0.897 | 9.93e-06 | 0.0093 | loglinear | Microglia Function | NM\_173781.4:715 |
  | Fscn1-mRNA | -0.612 | 0.0988 | -0.805 | -0.418 | 0.655 | 0.572 | 0.749 | 1.3e-05 | 0.0093 | loglinear | Microglia Function | NM\_007984.2:1645 |
  | Dusp7-mRNA | -0.378 | 0.0611 | -0.497 | -0.258 | 0.77 | 0.708 | 0.836 | 1.32e-05 | 0.0093 | loglinear | Adaptive Immune Response, Angiogenesis, Cytokine Signaling, Growth Factor Signaling, Innate Immune Response, Insulin Signaling, Microglia Function | NM\_153459.4:2094 |
  | Fos-mRNA | -1.75 | 0.299 | -2.34 | -1.17 | 0.297 | 0.198 | 0.446 | 2.43e-05 | 0.0143 | lm.nb | Adaptive Immune Response, Cellular Stress, Growth Factor Signaling, Innate Immune Response, Neurons and Neurotransmission | NM\_010234.2:1330 |
  | Syp-mRNA | 0.465 | 0.0859 | 0.296 | 0.633 | 1.38 | 1.23 | 1.55 | 5.76e-05 | 0.0291 | loglinear | Neurons and Neurotransmission | NM\_009305.2:732 |
  | Mapt-mRNA | -0.222 | 0.0425 | -0.306 | -0.139 | 0.857 | 0.809 | 0.908 | 8.22e-05 | 0.0321 | loglinear | Apoptosis, Cellular Stress, Growth Factor Signaling, Neurons and Neurotransmission | NM\_001038609.2:1202 |
  | Prkacb-mRNA | -0.327 | 0.0629 | -0.451 | -0.204 | 0.797 | 0.732 | 0.868 | 8.65e-05 | 0.0321 | loglinear | Adaptive Immune Response, Angiogenesis, Apoptosis, Autophagy, Growth Factor Signaling, Lipid Metabolism, Neurons and Neurotransmission, Wnt | NM\_011100.3:3754 |
  | Bdnf-mRNA | -0.736 | 0.143 | -1.02 | -0.456 | 0.6 | 0.494 | 0.729 | 9.57e-05 | 0.0321 | lm.nb | Growth Factor Signaling, Neurons and Neurotransmission | NM\_007540.4:3260 |
  | Pak1-mRNA | -0.284 | 0.0554 | -0.393 | -0.176 | 0.821 | 0.762 | 0.885 | 1e-04 | 0.0321 | loglinear | Adaptive Immune Response, Angiogenesis, Growth Factor Signaling, Innate Immune Response, Microglia Function | NM\_011035.2:1615 |
  | Rgl1-mRNA | -0.734 | 0.146 | -1.02 | -0.448 | 0.601 | 0.493 | 0.733 | 0.000121 | 0.0357 | lm.nb | Growth Factor Signaling, Microglia Function | NM\_016846.3:3320 |
  | Mdm2-mRNA | -0.317 | 0.0642 | -0.443 | -0.191 | 0.803 | 0.736 | 0.876 | 0.00015 | 0.0406 | loglinear | Adaptive Immune Response, Apoptosis, Cell Cycle, Cellular Stress, DNA Damage, Growth Factor Signaling, Neurons and Neurotransmission | NM\_010786.4:1664 |
  | Ppp3r1-mRNA | -0.28 | 0.0577 | -0.393 | -0.167 | 0.823 | 0.761 | 0.891 | 0.000175 | 0.0442 | loglinear | Adaptive Immune Response, Apoptosis, Growth Factor Signaling, Innate Immune Response, Wnt | NM\_024459.2:1390 |
  | Csf1r-mRNA | -0.687 | 0.144 | -0.969 | -0.405 | 0.621 | 0.511 | 0.755 | 0.000208 | 0.0464 | lm.nb | Cytokine Signaling, Growth Factor Signaling, Microglia Function | NM\_001037859.1:3655 |
  | Tbr1-mRNA | -0.58 | 0.122 | -0.818 | -0.341 | 0.669 | 0.567 | 0.789 | 0.00021 | 0.0464 | loglinear | Neurons and Neurotransmission | NM\_009322.3:2354 |
  | Cp-mRNA | 0.641 | 0.138 | 0.371 | 0.911 | 1.56 | 1.29 | 1.88 | 0.000262 | 0.0536 | loglinear | Astrocyte Function | NM\_001042611.1:1750 |
  | Smc1a-mRNA | -0.477 | 0.103 | -0.68 | -0.275 | 0.718 | 0.624 | 0.827 | 0.000287 | 0.0536 | lm.nb | Cell Cycle, DNA Damage | NM\_019710.2:1675 |
  | Kmt2a-mRNA | -0.591 | 0.129 | -0.844 | -0.339 | 0.664 | 0.557 | 0.791 | 0.000303 | 0.0536 | lm.nb | Epigenetic Regulation | NM\_001081049.1:2080 |
  | Amigo2-mRNA | 0.619 | 0.135 | 0.354 | 0.883 | 1.54 | 1.28 | 1.84 | 0.000304 | 0.0536 | lm.nb | Astrocyte Function | NM\_178114.4:2425 |

  ##### DE Results: DPI: 1 vs.CTRL

  Table presenting the most statistically significantly differentially expressed genes with the selected covariate. 'Estimated log fold-change' estimates a gene's differential expression. For categorical covariates, a gene is estimated to have 2^(log fold change) times its expression in baseline samples, holding all other variables in the analysis constant. If the covariate is continuous, for each unit increase in the selected covariate, a gene's expression is estimated to increase by 2^(log fold change)-fold, holding all other variables in the analysis constant. The 95% confidence interval for the log fold change is also presented, along with a p-value and an adjusted p-value or FDR if requested. Method column indicates the model used to estimate differential expression. Mixed negative binomial model uses the mle function to run the Wald test. The simplified negative binomial model uses the glm.nb function . The loglinear model uses the lm function.

- **Volcano Plot: DPI: 2 vs.CTRL  
  More Plot Information**


  ##### Volcano Plot: DPI: 2 vs.CTRL

  Volcano plot displaying each gene's -log10(p-value) and log2 fold change with the selected covariate. Highly statistically significant genes fall at the top of the plot above the horizontal lines, and highly differentially expressed genes fall to either side. Horizontal lines indicate various False Discovery Rate (FDR) thresholds or p-value thresholds if there is no adjustment to the p-values. Genes are colored if the resulting p-value is below the given FDR or p-value threshold. The 40 most statistically significant genes are labeled in the plot.
- **DE Results: DPI: 2 vs.CTRL  
  More Plot Information  Download CSV Data**

  |  | Log2 fold change | std error (log2) | Lower confidence limit (log2) | Upper confidence limit (log2) | Linear fold change | Lower confidence limit (linear) | Upper confidence limit (linear) | P-value | BY.p.value | method | Gene.sets | probe.ID |
  | --- | --- | --- | --- | --- | --- | --- | --- | --- | --- | --- | --- | --- |
  | Cp-mRNA | 1.93 | 0.138 | 1.66 | 2.2 | 3.82 | 3.17 | 4.6 | 2.03e-10 | 6.02e-07 | loglinear | Astrocyte Function | NM\_001042611.1:1750 |
  | Lcn2-mRNA | 9.71 | 0.723 | 8.3 | 11.1 | 839 | 314 | 2240 | 3.93e-10 | 6.02e-07 | lm.nb | Astrocyte Function, Inflammatory Signaling | NM\_008491.1:190 |
  | Egr1-mRNA | -1.71 | 0.13 | -1.97 | -1.46 | 0.305 | 0.255 | 0.363 | 5.12e-10 | 6.02e-07 | lm.nb | Inflammatory Signaling, Neurons and Neurotransmission | NM\_007913.5:515 |
  | Arc-mRNA | -1.96 | 0.152 | -2.26 | -1.66 | 0.257 | 0.209 | 0.316 | 6.95e-10 | 6.13e-07 | lm.nb | Neurons and Neurotransmission | NM\_018790.2:2715 |
  | Cdkn1a-mRNA | 2.13 | 0.171 | 1.79 | 2.46 | 4.37 | 3.47 | 5.52 | 1.24e-09 | 8.78e-07 | lm.nb | Adaptive Immune Response, Cell Cycle, Cellular Stress, Cytokine Signaling, DNA Damage, Growth Factor Signaling | NM\_007669.4:1670 |
  | Fkbp5-mRNA | 1.92 | 0.159 | 1.61 | 2.23 | 3.78 | 3.05 | 4.69 | 1.9e-09 | 1.12e-06 | lm.nb | Astrocyte Function | NM\_010220.3:2125 |
  | Bdnf-mRNA | -1.64 | 0.158 | -1.94 | -1.33 | 0.322 | 0.26 | 0.399 | 1.66e-08 | 8.35e-06 | lm.nb | Growth Factor Signaling, Neurons and Neurotransmission | NM\_007540.4:3260 |
  | Lfng-mRNA | 1.68 | 0.166 | 1.36 | 2.01 | 3.21 | 2.56 | 4.02 | 2.36e-08 | 1.04e-05 | lm.nb | Microglia Function, Notch | NM\_008494.3:1965 |
  | Rab6b-mRNA | -0.353 | 0.0357 | -0.423 | -0.283 | 0.783 | 0.746 | 0.822 | 3.25e-08 | 1.27e-05 | loglinear | Microglia Function | NM\_173781.4:715 |
  | Opalin-mRNA | -2.58 | 0.263 | -3.1 | -2.07 | 0.167 | 0.117 | 0.239 | 3.61e-08 | 1.27e-05 | lm.nb | Oligodendrocyte Function | NM\_153520.1:201 |
  | Fabp5-mRNA | -0.612 | 0.0681 | -0.746 | -0.479 | 0.654 | 0.596 | 0.718 | 1.19e-07 | 3.83e-05 | loglinear | Microglia Function | NM\_010634.3:430 |
  | Prkar2b-mRNA | -1.01 | 0.113 | -1.23 | -0.787 | 0.497 | 0.426 | 0.579 | 1.33e-07 | 3.91e-05 | loglinear | Apoptosis, Cell Cycle, Growth Factor Signaling | NM\_011158.3:918 |
  | Fscn1-mRNA | -0.872 | 0.0988 | -1.07 | -0.678 | 0.546 | 0.478 | 0.625 | 1.53e-07 | 4.14e-05 | loglinear | Microglia Function | NM\_007984.2:1645 |
  | Amigo2-mRNA | 1.08 | 0.131 | 0.826 | 1.34 | 2.12 | 1.77 | 2.53 | 3.65e-07 | 9.21e-05 | lm.nb | Astrocyte Function | NM\_178114.4:2425 |
  | Nfkbia-mRNA | 1.86 | 0.228 | 1.41 | 2.31 | 3.63 | 2.67 | 4.95 | 4.26e-07 | 9.99e-05 | lm.nb | Adaptive Immune Response, Apoptosis, Growth Factor Signaling, Inflammatory Signaling, Innate Immune Response, NF-kB | NM\_010907.2:646 |
  | Dusp7-mRNA | -0.496 | 0.0611 | -0.616 | -0.377 | 0.709 | 0.652 | 0.77 | 4.53e-07 | 9.99e-05 | loglinear | Adaptive Immune Response, Angiogenesis, Cytokine Signaling, Growth Factor Signaling, Innate Immune Response, Insulin Signaling, Microglia Function | NM\_153459.4:2094 |
  | Padi2-mRNA | -1.41 | 0.175 | -1.75 | -1.06 | 0.377 | 0.298 | 0.479 | 5.19e-07 | 0.000108 | lm.nb | Astrocyte Function, Epigenetic Regulation | NM\_008812.2:1016 |
  | Dock1-mRNA | -1.49 | 0.186 | -1.85 | -1.12 | 0.357 | 0.277 | 0.459 | 5.68e-07 | 0.000111 | loglinear | Angiogenesis, Autophagy | NM\_001033420.2:405 |
  | Prkar2a-mRNA | -0.311 | 0.0399 | -0.389 | -0.233 | 0.806 | 0.764 | 0.851 | 7.69e-07 | 0.000143 | loglinear | Apoptosis, Growth Factor Signaling | NM\_008924.2:2135 |
  | Ugt8a-mRNA | -1.74 | 0.224 | -2.18 | -1.3 | 0.3 | 0.221 | 0.407 | 8.23e-07 | 0.000145 | lm.nb | Lipid Metabolism, Oligodendrocyte Function | NM\_011674.4:138 |

  ##### DE Results: DPI: 2 vs.CTRL

  Table presenting the most statistically significantly differentially expressed genes with the selected covariate. 'Estimated log fold-change' estimates a gene's differential expression. For categorical covariates, a gene is estimated to have 2^(log fold change) times its expression in baseline samples, holding all other variables in the analysis constant. If the covariate is continuous, for each unit increase in the selected covariate, a gene's expression is estimated to increase by 2^(log fold change)-fold, holding all other variables in the analysis constant. The 95% confidence interval for the log fold change is also presented, along with a p-value and an adjusted p-value or FDR if requested. Method column indicates the model used to estimate differential expression. Mixed negative binomial model uses the mle function to run the Wald test. The simplified negative binomial model uses the glm.nb function . The loglinear model uses the lm function.

- **Volcano Plot: DPI: 3 vs.CTRL  
  More Plot Information**


  ##### Volcano Plot: DPI: 3 vs.CTRL

  Volcano plot displaying each gene's -log10(p-value) and log2 fold change with the selected covariate. Highly statistically significant genes fall at the top of the plot above the horizontal lines, and highly differentially expressed genes fall to either side. Horizontal lines indicate various False Discovery Rate (FDR) thresholds or p-value thresholds if there is no adjustment to the p-values. Genes are colored if the resulting p-value is below the given FDR or p-value threshold. The 40 most statistically significant genes are labeled in the plot.
- **DE Results: DPI: 3 vs.CTRL  
  More Plot Information  Download CSV Data**

  |  | Log2 fold change | std error (log2) | Lower confidence limit (log2) | Upper confidence limit (log2) | Linear fold change | Lower confidence limit (linear) | Upper confidence limit (linear) | P-value | BY.p.value | method | Gene.sets | probe.ID |
  | --- | --- | --- | --- | --- | --- | --- | --- | --- | --- | --- | --- | --- |
  | Cp-mRNA | 2.57 | 0.138 | 2.3 | 2.84 | 5.94 | 4.93 | 7.16 | 2.74e-12 | 7.36e-09 | loglinear | Astrocyte Function | NM\_001042611.1:1750 |
  | Arc-mRNA | -2.98 | 0.164 | -3.3 | -2.66 | 0.127 | 0.101 | 0.158 | 4.17e-12 | 7.36e-09 | lm.nb | Neurons and Neurotransmission | NM\_018790.2:2715 |
  | Cdkn1a-mRNA | 2.72 | 0.171 | 2.38 | 3.05 | 6.57 | 5.21 | 8.28 | 3.19e-11 | 3.76e-08 | lm.nb | Adaptive Immune Response, Cell Cycle, Cellular Stress, Cytokine Signaling, DNA Damage, Growth Factor Signaling | NM\_007669.4:1670 |
  | Lcn2-mRNA | 11 | 0.723 | 9.57 | 12.4 | 2030 | 759 | 5410 | 6.27e-11 | 5.53e-08 | lm.nb | Astrocyte Function, Inflammatory Signaling | NM\_008491.1:190 |
  | Fkbp5-mRNA | 2.17 | 0.158 | 1.86 | 2.47 | 4.48 | 3.62 | 5.56 | 2.94e-10 | 2.07e-07 | lm.nb | Astrocyte Function | NM\_010220.3:2125 |
  | Rab6b-mRNA | -0.441 | 0.0357 | -0.512 | -0.371 | 0.736 | 0.701 | 0.773 | 1.35e-09 | 7.96e-07 | loglinear | Microglia Function | NM\_173781.4:715 |
  | Egr1-mRNA | -1.51 | 0.13 | -1.77 | -1.26 | 0.351 | 0.294 | 0.418 | 3.11e-09 | 1.57e-06 | lm.nb | Inflammatory Signaling, Neurons and Neurotransmission | NM\_007913.5:515 |
  | Kdm5c-mRNA | -0.546 | 0.0494 | -0.643 | -0.449 | 0.685 | 0.64 | 0.732 | 6.61e-09 | 2.92e-06 | loglinear | Epigenetic Regulation | NM\_013668.3:750 |
  | Ugt8a-mRNA | -2.5 | 0.229 | -2.95 | -2.05 | 0.177 | 0.129 | 0.241 | 8.21e-09 | 3.12e-06 | lm.nb | Lipid Metabolism, Oligodendrocyte Function | NM\_011674.4:138 |
  | Sall1-mRNA | -1.75 | 0.162 | -2.06 | -1.43 | 0.298 | 0.239 | 0.371 | 9.43e-09 | 3.12e-06 | lm.nb | Epigenetic Regulation | NM\_021390.3:4875 |
  | Smarca4-mRNA | -0.495 | 0.046 | -0.585 | -0.405 | 0.71 | 0.667 | 0.755 | 9.76e-09 | 3.12e-06 | loglinear | Epigenetic Regulation, Wnt | NM\_011417.2:3540 |
  | Nfkbia-mRNA | 2.4 | 0.224 | 1.96 | 2.84 | 5.27 | 3.88 | 7.14 | 1.06e-08 | 3.12e-06 | lm.nb | Adaptive Immune Response, Apoptosis, Growth Factor Signaling, Inflammatory Signaling, Innate Immune Response, NF-kB | NM\_010907.2:646 |
  | Ifitm2-mRNA | 1.82 | 0.174 | 1.47 | 2.16 | 3.52 | 2.78 | 4.46 | 1.51e-08 | 4.09e-06 | lm.nb | Adaptive Immune Response, Inflammatory Signaling | NM\_030694.1:87 |
  | Tmem64-mRNA | -0.568 | 0.0558 | -0.677 | -0.459 | 0.675 | 0.625 | 0.728 | 2.13e-08 | 5.36e-06 | loglinear | Microglia Function | NM\_181401.3:1125 |
  | Mapt-mRNA | -0.413 | 0.0425 | -0.496 | -0.33 | 0.751 | 0.709 | 0.796 | 4.11e-08 | 9.67e-06 | loglinear | Apoptosis, Cellular Stress, Growth Factor Signaling, Neurons and Neurotransmission | NM\_001038609.2:1202 |
  | Kdm4b-mRNA | 0.563 | 0.0584 | 0.449 | 0.678 | 1.48 | 1.36 | 1.6 | 4.54e-08 | 1e-05 | loglinear | DNA Damage, Epigenetic Regulation | NM\_172132.1:3704 |
  | Stmn1-mRNA | -0.555 | 0.0593 | -0.671 | -0.438 | 0.681 | 0.628 | 0.738 | 6.89e-08 | 1.41e-05 | loglinear | Growth Factor Signaling, Microglia Function | NM\_019641.3:595 |
  | Prkar2b-mRNA | -1.06 | 0.113 | -1.28 | -0.834 | 0.481 | 0.412 | 0.561 | 7.17e-08 | 1.41e-05 | loglinear | Apoptosis, Cell Cycle, Growth Factor Signaling | NM\_011158.3:918 |
  | Dapk1-mRNA | 0.866 | 0.0943 | 0.681 | 1.05 | 1.82 | 1.6 | 2.07 | 8.86e-08 | 1.65e-05 | loglinear | Apoptosis, Autophagy | NM\_134062.1:4935 |
  | Irf3-mRNA | -1.48 | 0.165 | -1.8 | -1.15 | 0.359 | 0.287 | 0.45 | 1.3e-07 | 2.18e-05 | lm.nb | Apoptosis, Cytokine Signaling, Inflammatory Signaling, Innate Immune Response | NM\_016849.4:526 |

  ##### DE Results: DPI: 3 vs.CTRL

  Table presenting the most statistically significantly differentially expressed genes with the selected covariate. 'Estimated log fold-change' estimates a gene's differential expression. For categorical covariates, a gene is estimated to have 2^(log fold change) times its expression in baseline samples, holding all other variables in the analysis constant. If the covariate is continuous, for each unit increase in the selected covariate, a gene's expression is estimated to increase by 2^(log fold change)-fold, holding all other variables in the analysis constant. The 95% confidence interval for the log fold change is also presented, along with a p-value and an adjusted p-value or FDR if requested. Method column indicates the model used to estimate differential expression. Mixed negative binomial model uses the mle function to run the Wald test. The simplified negative binomial model uses the glm.nb function . The loglinear model uses the lm function.

The results of differential expression testing are summarized at the gene set level. Each gene set's most differentially expressed genes are identified, and the extent of differential expression in each gene set is summarized using a 'global significance score'.

Gene sets:

- Summary
- Adaptive Immune Response
- Angiogenesis
- Apoptosis
- Astrocyte Function
- Autophagy
- Carbohydrate Metabolism
- Cell Cycle
- Cellular Stress
- Cytokine Signaling
- DNA Damage
- Epigenetic Regulation
- Growth Factor Signaling
- Inflammatory Signaling
- Innate Immune Response
- Insulin Signaling
- Lipid Metabolism
- Matrix Remodeling
- Microglia Function
- Neurons and Neurotransmission
- NF-kB
- Notch
- Oligodendrocyte Function
- Wnt

- **Global Significance Scores  
  More Plot Information**


  ##### Global Significance Scores

  Heatmap displaying each sample's global significance scores. Global significance statistics measure the extent of differential expression of a gene set's genes with a covariate, ignoring whether each gene within the set is up- or down-regulated. Orange denotes gene sets whose genes exhibit extensive differential expression with the covariate, blue denotes gene sets with less differential expression.
- **Directed Global Significance Scores  
  More Plot Information**


  ##### Directed Global Significance Scores

  Heatmap displaying each sample's directed global significance scores. Directed global significance statistics measure the extent to which a gene set's genes are up- or down-regulated with the variable. Red denotes gene sets whose genes exhibit extensive over-expression with the covariate, blue denotes gene sets with extensive under-expression.

- **Undirected and Directed global significance scores table  
  More Plot Information  Download CSV Data**

  |  | Undirected DPI: differential expression in 1 vs. baseline of CTRL | Undirected DPI: differential expression in 2 vs. baseline of CTRL | Undirected DPI: differential expression in 3 vs. baseline of CTRL | Directed DPI: differential expression in 1 vs. baseline of CTRL | Directed DPI: differential expression in 2 vs. baseline of CTRL | Directed DPI: differential expression in 3 vs. baseline of CTRL |
  | --- | --- | --- | --- | --- | --- | --- |
  | Adaptive Immune Response | 2.622 | 3.764 | 4.069 | -2.083 | -1.192 | 2.46 |
  | Angiogenesis | 2.724 | 3.611 | 3.022 | -2.292 | -2.97 | -1.604 |
  | Apoptosis | 2.483 | 3.534 | 3.696 | -1.937 | -2.188 | -0.775 |
  | Astrocyte Function | 2.041 | 5.154 | 6.625 | 1.21 | 4.267 | 5.709 |
  | Autophagy | 2.23 | 3.317 | 3.174 | -1.633 | -1.554 | 1.085 |
  | Carbohydrate Metabolism | 2.095 | 2.776 | 3.516 | -0.714 | -0.682 | -2.978 |
  | Cell Cycle | 2.372 | 3.586 | 3.506 | -1.983 | -1.955 | 1.455 |
  | Cellular Stress | 2.366 | 3.458 | 3.71 | -2.127 | -1.933 | -0.376 |
  | Cytokine Signaling | 2.173 | 3.47 | 3.979 | -1.454 | -1.117 | 1.559 |
  | DNA Damage | 2.281 | 3.254 | 3.547 | -1.933 | -1.349 | 1.891 |
  | Epigenetic Regulation | 2.336 | 3.264 | 4.116 | -2.036 | -3.002 | -3.081 |
  | Growth Factor Signaling | 2.615 | 3.857 | 3.972 | -2.176 | -2.426 | -1.066 |
  | Inflammatory Signaling | 2.058 | 4.03 | 4.927 | -1.258 | 1.659 | 3.208 |
  | Innate Immune Response | 2.33 | 3.389 | 3.729 | -1.736 | -1.361 | 1.597 |
  | Insulin Signaling | 2.592 | 3.478 | 3.2 | -1.938 | -2.269 | 0.686 |
  | Lipid Metabolism | 2.262 | 3.438 | 4.21 | -2.182 | -2.857 | -2.682 |
  | Matrix Remodeling | 1.698 | 2.077 | 2.864 | -0.384 | -1.138 | 2.035 |
  | Microglia Function | 2.439 | 3.862 | 3.82 | -1.849 | -2.472 | -1.379 |
  | Neurons and Neurotransmission | 3.053 | 4.208 | 4.334 | -2.526 | -3.395 | -3.583 |
  | NF-kB | 1.878 | 3.189 | 4.252 | -0.702 | 1.427 | 2.558 |

  ##### Undirected and Directed global significance scores table

  Table displaying each sample's global significance scores and directed global significance scores as defined in the heatmaps above. The global significance score is calculated as the square root of the mean squared t-statistic for the genes in a gene set, with t-statistics coming from the linear regression underlying our differential expression analysis. The directed global significance score is calculated as the square root of the mean signed squared t-statistic for the genes in a gene set, with t-statistics coming from the linear regression underlying our differential expression analysis.

DPI:
- DPI: differential expression in 1 vs. baseline of CTRL
- DPI: differential expression in 2 vs. baseline of CTRL
- DPI: differential expression in 3 vs. baseline of CTRL

- **Volcano Plot: DPI: 1 vs.CTRL  
  More Plot Information**


  ##### Volcano Plot: DPI: 1 vs.CTRL

  Volcano plot displaying each gene's -log10(p-value) and log2 fold change for the selected covariate. Highly statistically significant genes fall at the top of the plot, and highly differentially expressed genes fall to either side. Genes within the selected gene set are highlighted in orange. Horizontal lines indicate various False Discovery Rate (FDR) thresholds.
- **DE Results - Adaptive Immune Response genes - DPI: 1 vs.CTRL  
  More Plot Information  Download CSV Data**

  | Probe Label | Log2 fold change | std error (log2) | Lower confidence limit (log2) | Upper confidence limit (log2) | Linear fold change | Lower confidence limit (linear) | Upper confidence limit (linear) | P-value | BY.p.value | method | Gene.sets | Codeset.Name | Probe.Label | Analyte.Type | Is.Control | Control.Type | Probe.Annotation | KEGG.Pathways | Cell.Type | Official.Gene.Name | volcanocol | volcanopch | tstats.all |
  | --- | --- | --- | --- | --- | --- | --- | --- | --- | --- | --- | --- | --- | --- | --- | --- | --- | --- | --- | --- | --- | --- | --- | --- |
  | Dusp7-mRNA | -0.378 | 0.0611 | -0.497 | -0.258 | 0.77 | 0.708 | 0.836 | 1.32e-05 | 0.0093 | loglinear | Adaptive Immune Response, Angiogenesis, Cytokine Signaling, Growth Factor Signaling, Innate Immune Response, Insulin Signaling, Microglia Function | NS\_MM\_NEUROINFLAM\_V1.0 | Dusp7 | mRNA | 0 |  | Microglia Function;Insulin Signaling;Innate Immune Response;Growth Factor Signaling;Cytokine Signaling;Angiogenesis;Adaptive Immune Response | mmu04010 |  | Dusp7 | slateblue2 | 16 | -6.18657937806874 |
  | Fos-mRNA | -1.75 | 0.299 | -2.34 | -1.17 | 0.297 | 0.198 | 0.446 | 2.43e-05 | 0.0143 | lm.nb | Adaptive Immune Response, Cellular Stress, Growth Factor Signaling, Innate Immune Response, Neurons and Neurotransmission | NS\_MM\_NEUROINFLAM\_V1.0 | Fos | mRNA | 0 |  | Neurons and Neurotransmission;Innate Immune Response;Growth Factor Signaling;Cellular Stress;Adaptive Immune Response | mmu04010;mmu04024;mmu04380;mmu04620;mmu04660;mmu04662;mmu04668;mmu04713;mmu04725;mmu04728;mmu04915;mmu04917;mmu04921;mmu05031;mmu05132;mmu05133;mmu05140;mmu05142;mmu05161;mmu05166;mmu05168;mmu05200;mmu05210;mmu05231;mmu05323 |  | Fos | slateblue2 | 16 | -5.85284280936455 |
  | Prkacb-mRNA | -0.327 | 0.0629 | -0.451 | -0.204 | 0.797 | 0.732 | 0.868 | 8.65e-05 | 0.0321 | loglinear | Adaptive Immune Response, Angiogenesis, Apoptosis, Autophagy, Growth Factor Signaling, Lipid Metabolism, Neurons and Neurotransmission, Wnt | NS\_MM\_NEUROINFLAM\_V1.0 | Prkacb | mRNA | 0 |  | Wnt;Neurons and Neurotransmission;Lipid Metabolism;Growth Factor Signaling;Autophagy;Apoptosis;Angiogenesis;Adaptive Immune Response | mmu04010;mmu04014;mmu04020;mmu04024;mmu04062;mmu04114;mmu04210;mmu04211;mmu04213;mmu04261;mmu04270;mmu04310;mmu04540;mmu04611;mmu04713;mmu04720;mmu04723;mmu04724;mmu04725;mmu04726;mmu04728;mmu04750;mmu04910;mmu04911;mmu04912;mmu04913;mmu04914;mmu04915;mmu04916;mmu04919;mmu04921;mmu04922;mmu04923;mmu04924;mmu04925;mmu05012;mmu05020;mmu05030;mmu05031;mmu05146;mmu05166;mmu05169;mmu05200;mmu05203;mmu05205;mmu05414 |  | Prkacb | slateblue2 | 16 | -5.19872813990461 |
  | Pak1-mRNA | -0.284 | 0.0554 | -0.393 | -0.176 | 0.821 | 0.762 | 0.885 | 1e-04 | 0.0321 | loglinear | Adaptive Immune Response, Angiogenesis, Growth Factor Signaling, Innate Immune Response, Microglia Function | NS\_MM\_NEUROINFLAM\_V1.0 | Pak1 | mRNA | 0 |  | Microglia Function;Innate Immune Response;Growth Factor Signaling;Angiogenesis;Adaptive Immune Response | mmu04010;mmu04012;mmu04014;mmu04024;mmu04062;mmu04360;mmu04510;mmu04650;mmu04660;mmu04666;mmu04810;mmu05205;mmu05211 |  | Pak1 | slateblue2 | 16 | -5.12635379061372 |
  | Mdm2-mRNA | -0.317 | 0.0642 | -0.443 | -0.191 | 0.803 | 0.736 | 0.876 | 0.00015 | 0.0406 | loglinear | Adaptive Immune Response, Apoptosis, Cell Cycle, Cellular Stress, DNA Damage, Growth Factor Signaling, Neurons and Neurotransmission | NS\_MM\_NEUROINFLAM\_V1.0 | Mdm2 | mRNA | 0 |  | Neurons and Neurotransmission;Growth Factor Signaling;DNA Damage;Cellular Stress;Cell Cycle;Apoptosis;Adaptive Immune Response | mmu04068;mmu04110;mmu04115;mmu04120;mmu04144;mmu04151;mmu04919;mmu05169;mmu05200;mmu05202;mmu05203;mmu05205;mmu05206;mmu05214;mmu05215;mmu05218;mmu05219;mmu05220 |  | Mdm2 | slateblue2 | 16 | -4.93769470404984 |
  | Ppp3r1-mRNA | -0.28 | 0.0577 | -0.393 | -0.167 | 0.823 | 0.761 | 0.891 | 0.000175 | 0.0442 | loglinear | Adaptive Immune Response, Apoptosis, Growth Factor Signaling, Innate Immune Response, Wnt | NS\_MM\_NEUROINFLAM\_V1.0 | Ppp3r1 | mRNA | 0 |  | Wnt;Innate Immune Response;Growth Factor Signaling;Apoptosis;Adaptive Immune Response | mmu04010;mmu04020;mmu04022;mmu04114;mmu04210;mmu04310;mmu04360;mmu04370;mmu04380;mmu04650;mmu04660;mmu04662;mmu04720;mmu04724;mmu04921;mmu04922;mmu04924;mmu05010;mmu05014;mmu05031;mmu05152;mmu05166 |  | Ppp3r1 | slateblue2 | 16 | -4.8526863084922 |
  | Lgmn-mRNA | 0.369 | 0.0832 | 0.206 | 0.532 | 1.29 | 1.15 | 1.45 | 0.000416 | 0.0611 | loglinear | Adaptive Immune Response, Innate Immune Response | NS\_MM\_NEUROINFLAM\_V1.0 | Lgmn | mRNA | 0 |  | Innate Immune Response;Adaptive Immune Response | mmu04142;mmu04612 |  | Lgmn | slateblue3 | 16 | 4.43509615384615 |
  | Ppp3cb-mRNA | -0.411 | 0.0979 | -0.602 | -0.219 | 0.752 | 0.659 | 0.859 | 0.000686 | 0.0806 | lm.nb | Adaptive Immune Response, Apoptosis, Growth Factor Signaling, Innate Immune Response, Wnt | NS\_MM\_NEUROINFLAM\_V1.0 | Ppp3cb | mRNA | 0 |  | Wnt;Innate Immune Response;Growth Factor Signaling;Apoptosis;Adaptive Immune Response | mmu04010;mmu04020;mmu04022;mmu04114;mmu04210;mmu04310;mmu04360;mmu04370;mmu04380;mmu04650;mmu04660;mmu04662;mmu04720;mmu04724;mmu04728;mmu04921;mmu04922;mmu04924;mmu05010;mmu05014;mmu05031;mmu05152;mmu05166 |  | Ppp3cb | slateblue3 | 16 | -4.19816138917262 |
  | Braf-mRNA | -0.641 | 0.154 | -0.943 | -0.339 | 0.641 | 0.52 | 0.791 | 0.000739 | 0.0815 | lm.nb | Adaptive Immune Response, Angiogenesis, Apoptosis, Cytokine Signaling, Growth Factor Signaling, Innate Immune Response, Insulin Signaling, Neurons and Neurotransmission | NS\_MM\_NEUROINFLAM\_V1.0 | Braf | mRNA | 0 |  | Neurons and Neurotransmission;Insulin Signaling;Innate Immune Response;Growth Factor Signaling;Cytokine Signaling;Apoptosis;Angiogenesis;Adaptive Immune Response | mmu04010;mmu04012;mmu04015;mmu04024;mmu04062;mmu04068;mmu04150;mmu04270;mmu04510;mmu04650;mmu04720;mmu04722;mmu04726;mmu04730;mmu04810;mmu04910;mmu04914;mmu05034;mmu05160;mmu05200;mmu05205;mmu05210;mmu05211;mmu05212;mmu05213;mmu05214;mmu05215;mmu05216;mmu05218;mmu05219;mmu05220;mmu05221;mmu05223 |  | Braf | slateblue3 | 16 | -4.16233766233766 |
  | Tubb4a-mRNA | -0.38 | 0.0956 | -0.567 | -0.192 | 0.769 | 0.675 | 0.875 | 0.00109 | 0.104 | loglinear | Adaptive Immune Response, Apoptosis, Cell Cycle, Neurons and Neurotransmission | NS\_MM\_NEUROINFLAM\_V1.0 | Tubb4a | mRNA | 0 |  | Neurons and Neurotransmission;Cell Cycle;Apoptosis;Adaptive Immune Response | mmu04145;mmu04540 |  | Tubb4a | slateblue4 | 16 | -3.97489539748954 |
  | Pten-mRNA | -0.296 | 0.0753 | -0.444 | -0.148 | 0.815 | 0.735 | 0.902 | 0.0012 | 0.109 | loglinear | Adaptive Immune Response, Autophagy, DNA Damage, Growth Factor Signaling, Lipid Metabolism | NS\_MM\_NEUROINFLAM\_V1.0 | Pten | mRNA | 0 |  | Lipid Metabolism;Growth Factor Signaling;DNA Damage;Autophagy;Adaptive Immune Response | mmu00562;mmu04068;mmu04070;mmu04071;mmu04115;mmu04150;mmu04151;mmu04510;mmu04530;mmu04931;mmu05161;mmu05200;mmu05206;mmu05213;mmu05214;mmu05215;mmu05218;mmu05222;mmu05230 |  | Pten | slateblue4 | 16 | -3.93094289508632 |
  | Mapk10-mRNA | -0.539 | 0.138 | -0.81 | -0.269 | 0.688 | 0.57 | 0.83 | 0.00126 | 0.111 | lm.nb | Adaptive Immune Response, Apoptosis, Autophagy, Cellular Stress, Growth Factor Signaling, Innate Immune Response, Wnt | NS\_MM\_NEUROINFLAM\_V1.0 | Mapk10 | mRNA | 0 |  | Wnt;Innate Immune Response;Growth Factor Signaling;Cellular Stress;Autophagy;Apoptosis;Adaptive Immune Response | mmu04010;mmu04012;mmu04014;mmu04024;mmu04068;mmu04071;mmu04141;mmu04310;mmu04380;mmu04510;mmu04620;mmu04621;mmu04622;mmu04664;mmu04668;mmu04722;mmu04723;mmu04728;mmu04750;mmu04910;mmu04912;mmu04914;mmu04917;mmu04920;mmu04930;mmu04931;mmu04932;mmu04933;mmu05132;mmu05133;mmu05142;mmu05145;mmu05152;mmu05160;mmu05161;mmu05164;mmu05168;mmu05169;mmu05200;mmu05210;mmu05212;mmu05231 |  | Mapk10 | slateblue4 | 16 | -3.90579710144928 |
  | Psmb8-mRNA | 1.36 | 0.365 | 0.649 | 2.08 | 2.57 | 1.57 | 4.23 | 0.00179 | 0.129 | lm.nb | Adaptive Immune Response, Angiogenesis, Apoptosis, Astrocyte Function, Cell Cycle, Cytokine Signaling, Growth Factor Signaling, Inflammatory Signaling, Insulin Signaling, Microglia Function, NF-kB, Wnt | NS\_MM\_NEUROINFLAM\_V1.0 | Psmb8 | mRNA | 0 |  | Wnt;NF-kB;Microglia Function;Insulin Signaling;Inflammatory Signaling;Growth Factor Signaling;Cytokine Signaling;Cell Cycle;Astrocyte Function;Apoptosis;Angiogenesis;Adaptive Immune Response |  |  | Psmb8 | slateblue4 | 16 | 3.72602739726027 |
  | Rac1-mRNA | -0.928 | 0.256 | -1.43 | -0.426 | 0.525 | 0.371 | 0.744 | 0.00229 | 0.149 | lm.nb | Adaptive Immune Response, Angiogenesis, Autophagy, Growth Factor Signaling, Innate Immune Response, Neurons and Neurotransmission, Wnt | NS\_MM\_NEUROINFLAM\_V1.0 | Rac1 | mRNA | 0 |  | Wnt;Neurons and Neurotransmission;Innate Immune Response;Growth Factor Signaling;Autophagy;Angiogenesis;Adaptive Immune Response | mmu04010;mmu04014;mmu04015;mmu04024;mmu04062;mmu04071;mmu04145;mmu04151;mmu04310;mmu04360;mmu04370;mmu04380;mmu04510;mmu04520;mmu04620;mmu04650;mmu04662;mmu04664;mmu04666;mmu04670;mmu04722;mmu04810;mmu04932;mmu04933;mmu05014;mmu05100;mmu05132;mmu05200;mmu05203;mmu05205;mmu05210;mmu05211;mmu05212;mmu05231;mmu05416 |  | Rac1 | slateblue4 | 16 | -3.625 |
  | Rapgef3-mRNA | -0.593 | 0.164 | -0.915 | -0.27 | 0.663 | 0.53 | 0.829 | 0.00238 | 0.15 | lm.nb | Adaptive Immune Response, Autophagy | NS\_MM\_NEUROINFLAM\_V1.0 | Rapgef3 | mRNA | 0 |  | Autophagy;Adaptive Immune Response | mmu04015;mmu04024;mmu04072;mmu04261;mmu04670;mmu04720;mmu04726 |  | Rapgef3 | slateblue4 | 16 | -3.61585365853659 |
  | Cdkn1a-mRNA | -0.625 | 0.18 | -0.979 | -0.272 | 0.648 | 0.507 | 0.828 | 0.00317 | 0.18 | lm.nb | Adaptive Immune Response, Cell Cycle, Cellular Stress, Cytokine Signaling, DNA Damage, Growth Factor Signaling | NS\_MM\_NEUROINFLAM\_V1.0 | Cdkn1a | mRNA | 0 |  | Growth Factor Signaling;DNA Damage;Cytokine Signaling;Cellular Stress;Cell Cycle;Adaptive Immune Response | mmu04012;mmu04066;mmu04068;mmu04110;mmu04115;mmu04151;mmu04630;mmu04921;mmu05160;mmu05161;mmu05166;mmu05169;mmu05200;mmu05202;mmu05203;mmu05205;mmu05206;mmu05214;mmu05215;mmu05218;mmu05219;mmu05220 |  | Cdkn1a | slateblue4 | 16 | -3.47222222222222 |
  | Casp9-mRNA | -1.44 | 0.433 | -2.29 | -0.59 | 0.369 | 0.205 | 0.664 | 0.00432 | 0.2 | lm.nb | Adaptive Immune Response, Apoptosis, DNA Damage, Growth Factor Signaling, Innate Immune Response | NS\_MM\_NEUROINFLAM\_V1.0 | Casp9 | mRNA | 0 |  | Innate Immune Response;Growth Factor Signaling;DNA Damage;Apoptosis;Adaptive Immune Response | mmu04115;mmu04151;mmu04210;mmu04370;mmu04919;mmu05010;mmu05012;mmu05014;mmu05016;mmu05134;mmu05145;mmu05152;mmu05161;mmu05164;mmu05200;mmu05210;mmu05212;mmu05213;mmu05215;mmu05222;mmu05223;mmu05416 |  | Casp9 | slateblue4 | 16 | -3.3256351039261 |
  | Tubb3-mRNA | -0.52 | 0.158 | -0.83 | -0.21 | 0.697 | 0.562 | 0.865 | 0.00464 | 0.206 | lm.nb | Adaptive Immune Response, Apoptosis, Cell Cycle, Neurons and Neurotransmission | NS\_MM\_NEUROINFLAM\_V1.0 | Tubb3 | mRNA | 0 |  | Neurons and Neurotransmission;Cell Cycle;Apoptosis;Adaptive Immune Response | mmu04145;mmu04540 |  | Tubb3 | slateblue4 | 16 | -3.29113924050633 |
  | Map2k4-mRNA | -0.433 | 0.133 | -0.693 | -0.173 | 0.741 | 0.619 | 0.887 | 0.00484 | 0.206 | lm.nb | Adaptive Immune Response, Apoptosis, Cellular Stress, Growth Factor Signaling, Innate Immune Response | NS\_MM\_NEUROINFLAM\_V1.0 | Map2k4 | mRNA | 0 |  | Innate Immune Response;Growth Factor Signaling;Cellular Stress;Apoptosis;Adaptive Immune Response | mmu04010;mmu04012;mmu04620;mmu04664;mmu04668;mmu04912;mmu05142;mmu05161;mmu05164;mmu05166;mmu05169 |  | Map2k4 | slateblue4 | 16 | -3.25563909774436 |
  | Traf6-mRNA | 0.546 | 0.169 | 0.215 | 0.877 | 1.46 | 1.16 | 1.84 | 0.00523 | 0.215 | lm.nb | Adaptive Immune Response, Apoptosis, Autophagy, Cytokine Signaling, Growth Factor Signaling, Innate Immune Response, NF-kB | NS\_MM\_NEUROINFLAM\_V1.0 | Traf6 | mRNA | 0 |  | NF-kB;Innate Immune Response;Growth Factor Signaling;Cytokine Signaling;Autophagy;Apoptosis;Adaptive Immune Response | mmu04010;mmu04064;mmu04120;mmu04144;mmu04380;mmu04620;mmu04621;mmu04622;mmu04722;mmu05133;mmu05140;mmu05142;mmu05145;mmu05152;mmu05160;mmu05162;mmu05168;mmu05169;mmu05200;mmu05222 |  | Traf6 | slateblue4 | 16 | 3.23076923076923 |

  ##### DE Results - Adaptive Immune Response genes - DPI: 1 vs.CTRL

  Table displaying each sample's global significance scores and directed global significance scores as defined in the heatmaps above. The global significance score is calculated as the square root of the mean squared t-statistic for the genes in a gene set, with t-statistics coming from the linear regression underlying our differential expression analysis. The directed global significance score is calculated as the square root of the mean signed squared t-statistic for the genes in a gene set, with t-statistics coming from the linear regression underlying our differential expression analysis.

- **Volcano Plot: DPI: 2 vs.CTRL  
  More Plot Information**


  ##### Volcano Plot: DPI: 2 vs.CTRL

  Volcano plot displaying each gene's -log10(p-value) and log2 fold change for the selected covariate. Highly statistically significant genes fall at the top of the plot, and highly differentially expressed genes fall to either side. Genes within the selected gene set are highlighted in orange. Horizontal lines indicate various False Discovery Rate (FDR) thresholds.
- **DE Results - Adaptive Immune Response genes - DPI: 2 vs.CTRL  
  More Plot Information  Download CSV Data**

  | Probe Label | Log2 fold change | std error (log2) | Lower confidence limit (log2) | Upper confidence limit (log2) | Linear fold change | Lower confidence limit (linear) | Upper confidence limit (linear) | P-value | BY.p.value | method | Gene.sets | Codeset.Name | Probe.Label | Analyte.Type | Is.Control | Control.Type | Probe.Annotation | KEGG.Pathways | Cell.Type | Official.Gene.Name | volcanocol | volcanopch | tstats.all |
  | --- | --- | --- | --- | --- | --- | --- | --- | --- | --- | --- | --- | --- | --- | --- | --- | --- | --- | --- | --- | --- | --- | --- | --- |
  | Cdkn1a-mRNA | 2.13 | 0.171 | 1.79 | 2.46 | 4.37 | 3.47 | 5.52 | 1.24e-09 | 8.78e-07 | lm.nb | Adaptive Immune Response, Cell Cycle, Cellular Stress, Cytokine Signaling, DNA Damage, Growth Factor Signaling | NS\_MM\_NEUROINFLAM\_V1.0 | Cdkn1a | mRNA | 0 |  | Growth Factor Signaling;DNA Damage;Cytokine Signaling;Cellular Stress;Cell Cycle;Adaptive Immune Response | mmu04012;mmu04066;mmu04068;mmu04110;mmu04115;mmu04151;mmu04630;mmu04921;mmu05160;mmu05161;mmu05166;mmu05169;mmu05200;mmu05202;mmu05203;mmu05205;mmu05206;mmu05214;mmu05215;mmu05218;mmu05219;mmu05220 |  | Cdkn1a | slateblue1 | 16 | 12.4561403508772 |
  | Nfkbia-mRNA | 1.86 | 0.228 | 1.41 | 2.31 | 3.63 | 2.67 | 4.95 | 4.26e-07 | 9.99e-05 | lm.nb | Adaptive Immune Response, Apoptosis, Growth Factor Signaling, Inflammatory Signaling, Innate Immune Response, NF-kB | NS\_MM\_NEUROINFLAM\_V1.0 | Nfkbia | mRNA | 0 |  | NF-kB;Innate Immune Response;Inflammatory Signaling;Growth Factor Signaling;Apoptosis;Adaptive Immune Response | mmu04024;mmu04062;mmu04064;mmu04210;mmu04380;mmu04620;mmu04621;mmu04622;mmu04623;mmu04660;mmu04662;mmu04668;mmu04722;mmu04920;mmu04931;mmu05134;mmu05140;mmu05142;mmu05145;mmu05160;mmu05161;mmu05162;mmu05164;mmu05166;mmu05168;mmu05169;mmu05200;mmu05203;mmu05215;mmu05220;mmu05222 |  | Nfkbia | slateblue1 | 16 | 8.15789473684211 |
  | Dusp7-mRNA | -0.496 | 0.0611 | -0.616 | -0.377 | 0.709 | 0.652 | 0.77 | 4.53e-07 | 9.99e-05 | loglinear | Adaptive Immune Response, Angiogenesis, Cytokine Signaling, Growth Factor Signaling, Innate Immune Response, Insulin Signaling, Microglia Function | NS\_MM\_NEUROINFLAM\_V1.0 | Dusp7 | mRNA | 0 |  | Microglia Function;Insulin Signaling;Innate Immune Response;Growth Factor Signaling;Cytokine Signaling;Angiogenesis;Adaptive Immune Response | mmu04010 |  | Dusp7 | slateblue1 | 16 | -8.11783960720131 |
  | Fos-mRNA | -2.04 | 0.3 | -2.62 | -1.45 | 0.244 | 0.162 | 0.367 | 4.41e-06 | 0.000585 | lm.nb | Adaptive Immune Response, Cellular Stress, Growth Factor Signaling, Innate Immune Response, Neurons and Neurotransmission | NS\_MM\_NEUROINFLAM\_V1.0 | Fos | mRNA | 0 |  | Neurons and Neurotransmission;Innate Immune Response;Growth Factor Signaling;Cellular Stress;Adaptive Immune Response | mmu04010;mmu04024;mmu04380;mmu04620;mmu04660;mmu04662;mmu04668;mmu04713;mmu04725;mmu04728;mmu04915;mmu04917;mmu04921;mmu05031;mmu05132;mmu05133;mmu05140;mmu05142;mmu05161;mmu05166;mmu05168;mmu05200;mmu05210;mmu05231;mmu05323 |  | Fos | slateblue1 | 16 | -6.8 |
  | Psmb8-mRNA | 2.23 | 0.352 | 1.54 | 2.92 | 4.7 | 2.91 | 7.59 | 9.89e-06 | 0.000997 | lm.nb | Adaptive Immune Response, Angiogenesis, Apoptosis, Astrocyte Function, Cell Cycle, Cytokine Signaling, Growth Factor Signaling, Inflammatory Signaling, Insulin Signaling, Microglia Function, NF-kB, Wnt | NS\_MM\_NEUROINFLAM\_V1.0 | Psmb8 | mRNA | 0 |  | Wnt;NF-kB;Microglia Function;Insulin Signaling;Inflammatory Signaling;Growth Factor Signaling;Cytokine Signaling;Cell Cycle;Astrocyte Function;Apoptosis;Angiogenesis;Adaptive Immune Response |  |  | Psmb8 | slateblue1 | 16 | 6.33522727272727 |
  | Fcgr2b-mRNA | 1.25 | 0.208 | 0.846 | 1.66 | 2.38 | 1.8 | 3.16 | 1.75e-05 | 0.00148 | lm.nb | Adaptive Immune Response, Autophagy | NS\_MM\_NEUROINFLAM\_V1.0 | Fcgr2b | mRNA | 0 |  | Autophagy;Adaptive Immune Response | mmu04145;mmu04380;mmu04662;mmu04666;mmu05150;mmu05152;mmu05162 |  | Fcgr2b | slateblue1 | 16 | 6.00961538461539 |
  | Ppp3r1-mRNA | -0.334 | 0.0577 | -0.447 | -0.221 | 0.793 | 0.734 | 0.858 | 2.79e-05 | 0.00214 | loglinear | Adaptive Immune Response, Apoptosis, Growth Factor Signaling, Innate Immune Response, Wnt | NS\_MM\_NEUROINFLAM\_V1.0 | Ppp3r1 | mRNA | 0 |  | Wnt;Innate Immune Response;Growth Factor Signaling;Apoptosis;Adaptive Immune Response | mmu04010;mmu04020;mmu04022;mmu04114;mmu04210;mmu04310;mmu04360;mmu04370;mmu04380;mmu04650;mmu04660;mmu04662;mmu04720;mmu04724;mmu04921;mmu04922;mmu04924;mmu05010;mmu05014;mmu05031;mmu05152;mmu05166 |  | Ppp3r1 | slateblue1 | 16 | -5.78856152512998 |
  | Fcgr3-mRNA | 2.05 | 0.36 | 1.34 | 2.75 | 4.14 | 2.54 | 6.74 | 3.32e-05 | 0.0024 | lm.nb | Adaptive Immune Response, Autophagy, Inflammatory Signaling, Innate Immune Response | NS\_MM\_NEUROINFLAM\_V1.0 | Fcgr3 | mRNA | 0 |  | Innate Immune Response;Inflammatory Signaling;Autophagy;Adaptive Immune Response | mmu04145;mmu04380;mmu05140;mmu05150;mmu05152 |  | Fcgr3 | slateblue1 | 16 | 5.69444444444444 |
  | Ppp3cb-mRNA | -0.554 | 0.098 | -0.747 | -0.362 | 0.681 | 0.596 | 0.778 | 3.56e-05 | 0.00246 | lm.nb | Adaptive Immune Response, Apoptosis, Growth Factor Signaling, Innate Immune Response, Wnt | NS\_MM\_NEUROINFLAM\_V1.0 | Ppp3cb | mRNA | 0 |  | Wnt;Innate Immune Response;Growth Factor Signaling;Apoptosis;Adaptive Immune Response | mmu04010;mmu04020;mmu04022;mmu04114;mmu04210;mmu04310;mmu04360;mmu04370;mmu04380;mmu04650;mmu04660;mmu04662;mmu04720;mmu04724;mmu04728;mmu04921;mmu04922;mmu04924;mmu05010;mmu05014;mmu05031;mmu05152;mmu05166 |  | Ppp3cb | slateblue1 | 16 | -5.6530612244898 |
  | Prkacb-mRNA | -0.322 | 0.0629 | -0.445 | -0.199 | 0.8 | 0.735 | 0.871 | 0.000104 | 0.00562 | loglinear | Adaptive Immune Response, Angiogenesis, Apoptosis, Autophagy, Growth Factor Signaling, Lipid Metabolism, Neurons and Neurotransmission, Wnt | NS\_MM\_NEUROINFLAM\_V1.0 | Prkacb | mRNA | 0 |  | Wnt;Neurons and Neurotransmission;Lipid Metabolism;Growth Factor Signaling;Autophagy;Apoptosis;Angiogenesis;Adaptive Immune Response | mmu04010;mmu04014;mmu04020;mmu04024;mmu04062;mmu04114;mmu04210;mmu04211;mmu04213;mmu04261;mmu04270;mmu04310;mmu04540;mmu04611;mmu04713;mmu04720;mmu04723;mmu04724;mmu04725;mmu04726;mmu04728;mmu04750;mmu04910;mmu04911;mmu04912;mmu04913;mmu04914;mmu04915;mmu04916;mmu04919;mmu04921;mmu04922;mmu04923;mmu04924;mmu04925;mmu05012;mmu05020;mmu05030;mmu05031;mmu05146;mmu05166;mmu05169;mmu05200;mmu05203;mmu05205;mmu05414 |  | Prkacb | slateblue1 | 16 | -5.11923688394277 |
  | Tubb3-mRNA | -0.799 | 0.158 | -1.11 | -0.489 | 0.575 | 0.463 | 0.713 | 0.000119 | 0.00609 | lm.nb | Adaptive Immune Response, Apoptosis, Cell Cycle, Neurons and Neurotransmission | NS\_MM\_NEUROINFLAM\_V1.0 | Tubb3 | mRNA | 0 |  | Neurons and Neurotransmission;Cell Cycle;Apoptosis;Adaptive Immune Response | mmu04145;mmu04540 |  | Tubb3 | slateblue1 | 16 | -5.05696202531646 |
  | Mapk10-mRNA | -0.673 | 0.138 | -0.943 | -0.402 | 0.627 | 0.52 | 0.757 | 0.000172 | 0.0082 | lm.nb | Adaptive Immune Response, Apoptosis, Autophagy, Cellular Stress, Growth Factor Signaling, Innate Immune Response, Wnt | NS\_MM\_NEUROINFLAM\_V1.0 | Mapk10 | mRNA | 0 |  | Wnt;Innate Immune Response;Growth Factor Signaling;Cellular Stress;Autophagy;Apoptosis;Adaptive Immune Response | mmu04010;mmu04012;mmu04014;mmu04024;mmu04068;mmu04071;mmu04141;mmu04310;mmu04380;mmu04510;mmu04620;mmu04621;mmu04622;mmu04664;mmu04668;mmu04722;mmu04723;mmu04728;mmu04750;mmu04910;mmu04912;mmu04914;mmu04917;mmu04920;mmu04930;mmu04931;mmu04932;mmu04933;mmu05132;mmu05133;mmu05142;mmu05145;mmu05152;mmu05160;mmu05161;mmu05164;mmu05168;mmu05169;mmu05200;mmu05210;mmu05212;mmu05231 |  | Mapk10 | slateblue1 | 16 | -4.8768115942029 |
  | Pak1-mRNA | -0.263 | 0.0554 | -0.372 | -0.155 | 0.833 | 0.773 | 0.898 | 0.000215 | 0.0092 | loglinear | Adaptive Immune Response, Angiogenesis, Growth Factor Signaling, Innate Immune Response, Microglia Function | NS\_MM\_NEUROINFLAM\_V1.0 | Pak1 | mRNA | 0 |  | Microglia Function;Innate Immune Response;Growth Factor Signaling;Angiogenesis;Adaptive Immune Response | mmu04010;mmu04012;mmu04014;mmu04024;mmu04062;mmu04360;mmu04510;mmu04650;mmu04660;mmu04666;mmu04810;mmu05205;mmu05211 |  | Pak1 | slateblue1 | 16 | -4.74729241877256 |
  | Prkcq-mRNA | -0.864 | 0.185 | -1.23 | -0.501 | 0.549 | 0.427 | 0.707 | 0.000258 | 0.0106 | lm.nb | Adaptive Immune Response, Apoptosis, Autophagy, NF-kB, Oligodendrocyte Function | NS\_MM\_NEUROINFLAM\_V1.0 | Prkcq | mRNA | 0 |  | Oligodendrocyte Function;NF-kB;Autophagy;Apoptosis;Adaptive Immune Response | mmu04064;mmu04270;mmu04530;mmu04660;mmu04750;mmu04920;mmu04931;mmu05162 | Oligodendrocytes | Prkcq | slateblue2 | 16 | -4.67027027027027 |
  | Traf6-mRNA | 0.756 | 0.167 | 0.428 | 1.08 | 1.69 | 1.35 | 2.12 | 0.000353 | 0.0135 | lm.nb | Adaptive Immune Response, Apoptosis, Autophagy, Cytokine Signaling, Growth Factor Signaling, Innate Immune Response, NF-kB | NS\_MM\_NEUROINFLAM\_V1.0 | Traf6 | mRNA | 0 |  | NF-kB;Innate Immune Response;Growth Factor Signaling;Cytokine Signaling;Autophagy;Apoptosis;Adaptive Immune Response | mmu04010;mmu04064;mmu04120;mmu04144;mmu04380;mmu04620;mmu04621;mmu04622;mmu04722;mmu05133;mmu05140;mmu05142;mmu05145;mmu05152;mmu05160;mmu05162;mmu05168;mmu05169;mmu05200;mmu05222 |  | Traf6 | slateblue2 | 16 | 4.52694610778443 |
  | Ifitm2-mRNA | 0.804 | 0.179 | 0.454 | 1.15 | 1.75 | 1.37 | 2.23 | 0.000363 | 0.0136 | lm.nb | Adaptive Immune Response, Inflammatory Signaling | NS\_MM\_NEUROINFLAM\_V1.0 | Ifitm2 | mRNA | 0 |  | Inflammatory Signaling;Adaptive Immune Response |  |  | Ifitm2 | slateblue2 | 16 | 4.49162011173184 |
  | Ifitm3-mRNA | 1.53 | 0.344 | 0.854 | 2.2 | 2.89 | 1.81 | 4.61 | 0.000411 | 0.0148 | lm.nb | Adaptive Immune Response, Inflammatory Signaling | NS\_MM\_NEUROINFLAM\_V1.0 | Ifitm3 | mRNA | 0 |  | Inflammatory Signaling;Adaptive Immune Response |  |  | Ifitm3 | slateblue2 | 16 | 4.44767441860465 |
  | Pik3r2-mRNA | -0.338 | 0.0793 | -0.494 | -0.183 | 0.791 | 0.71 | 0.881 | 0.000587 | 0.0197 | loglinear | Adaptive Immune Response, Angiogenesis, Apoptosis, Autophagy, Cytokine Signaling, Growth Factor Signaling, Innate Immune Response, Insulin Signaling | NS\_MM\_NEUROINFLAM\_V1.0 | Pik3r2 | mRNA | 0 |  | Insulin Signaling;Innate Immune Response;Growth Factor Signaling;Cytokine Signaling;Autophagy;Apoptosis;Angiogenesis;Adaptive Immune Response | mmu04012;mmu04014;mmu04015;mmu04022;mmu04024;mmu04062;mmu04066;mmu04068;mmu04070;mmu04071;mmu04072;mmu04150;mmu04151;mmu04152;mmu04210;mmu04211;mmu04213;mmu04261;mmu04370;mmu04380;mmu04510;mmu04550;mmu04611;mmu04620;mmu04630;mmu04650;mmu04660;mmu04662;mmu04664;mmu04666;mmu04668;mmu04670;mmu04722;mmu04725;mmu04750;mmu04810;mmu04910;mmu04914;mmu04915;mmu04917;mmu04919;mmu04921;mmu04923;mmu04930;mmu04931;mmu04932;mmu04933;mmu04960;mmu04973;mmu05100;mmu05142;mmu05145;mmu05146;mmu05160;mmu05161;mmu05162;mmu05164;mmu05166;mmu05169;mmu05200;mmu05203;mmu05205;mmu05210;mmu05211;mmu05212;mmu05213;mmu05214;mmu05215;mmu05218;mmu05220;mmu05221;mmu05222;mmu05223;mmu05230;mmu05231 |  | Pik3r2 | slateblue2 | 16 | -4.26229508196721 |
  | Nefl-mRNA | -0.522 | 0.124 | -0.764 | -0.28 | 0.696 | 0.589 | 0.824 | 0.000651 | 0.0215 | lm.nb | Adaptive Immune Response, Angiogenesis, Cytokine Signaling, Growth Factor Signaling, Insulin Signaling, Neurons and Neurotransmission | NS\_MM\_NEUROINFLAM\_V1.0 | Nefl | mRNA | 0 |  | Neurons and Neurotransmission;Insulin Signaling;Growth Factor Signaling;Cytokine Signaling;Angiogenesis;Adaptive Immune Response | mmu05014 |  | Nefl | slateblue2 | 16 | -4.20967741935484 |
  | Chuk-mRNA | -0.331 | 0.0787 | -0.485 | -0.176 | 0.795 | 0.715 | 0.885 | 0.000678 | 0.0218 | loglinear | Adaptive Immune Response, Apoptosis, Cytokine Signaling, Growth Factor Signaling, Inflammatory Signaling, Innate Immune Response, NF-kB | NS\_MM\_NEUROINFLAM\_V1.0 | Chuk | mRNA | 0 |  | NF-kB;Innate Immune Response;Inflammatory Signaling;Growth Factor Signaling;Cytokine Signaling;Apoptosis;Adaptive Immune Response | mmu04010;mmu04014;mmu04062;mmu04064;mmu04068;mmu04151;mmu04210;mmu04380;mmu04620;mmu04621;mmu04622;mmu04623;mmu04660;mmu04662;mmu04668;mmu04920;mmu05142;mmu05145;mmu05160;mmu05161;mmu05162;mmu05166;mmu05168;mmu05169;mmu05200;mmu05212;mmu05215;mmu05220;mmu05221;mmu05222 |  | Chuk | slateblue2 | 16 | -4.20584498094028 |

  ##### DE Results - Adaptive Immune Response genes - DPI: 2 vs.CTRL

  Table displaying each sample's global significance scores and directed global significance scores as defined in the heatmaps above. The global significance score is calculated as the square root of the mean squared t-statistic for the genes in a gene set, with t-statistics coming from the linear regression underlying our differential expression analysis. The directed global significance score is calculated as the square root of the mean signed squared t-statistic for the genes in a gene set, with t-statistics coming from the linear regression underlying our differential expression analysis.

- **Volcano Plot: DPI: 3 vs.CTRL  
  More Plot Information**


  ##### Volcano Plot: DPI: 3 vs.CTRL

  Volcano plot displaying each gene's -log10(p-value) and log2 fold change for the selected covariate. Highly statistically significant genes fall at the top of the plot, and highly differentially expressed genes fall to either side. Genes within the selected gene set are highlighted in orange. Horizontal lines indicate various False Discovery Rate (FDR) thresholds.
- **DE Results - Adaptive Immune Response genes - DPI: 3 vs.CTRL  
  More Plot Information  Download CSV Data**

  | Probe Label | Log2 fold change | std error (log2) | Lower confidence limit (log2) | Upper confidence limit (log2) | Linear fold change | Lower confidence limit (linear) | Upper confidence limit (linear) | P-value | BY.p.value | method | Gene.sets | Codeset.Name | Probe.Label | Analyte.Type | Is.Control | Control.Type | Probe.Annotation | KEGG.Pathways | Cell.Type | Official.Gene.Name | volcanocol | volcanopch | tstats.all |
  | --- | --- | --- | --- | --- | --- | --- | --- | --- | --- | --- | --- | --- | --- | --- | --- | --- | --- | --- | --- | --- | --- | --- | --- |
  | Cdkn1a-mRNA | 2.72 | 0.171 | 2.38 | 3.05 | 6.57 | 5.21 | 8.28 | 3.19e-11 | 3.76e-08 | lm.nb | Adaptive Immune Response, Cell Cycle, Cellular Stress, Cytokine Signaling, DNA Damage, Growth Factor Signaling | NS\_MM\_NEUROINFLAM\_V1.0 | Cdkn1a | mRNA | 0 |  | Growth Factor Signaling;DNA Damage;Cytokine Signaling;Cellular Stress;Cell Cycle;Adaptive Immune Response | mmu04012;mmu04066;mmu04068;mmu04110;mmu04115;mmu04151;mmu04630;mmu04921;mmu05160;mmu05161;mmu05166;mmu05169;mmu05200;mmu05202;mmu05203;mmu05205;mmu05206;mmu05214;mmu05215;mmu05218;mmu05219;mmu05220 |  | Cdkn1a | slateblue1 | 16 | 15.906432748538 |
  | Nfkbia-mRNA | 2.4 | 0.224 | 1.96 | 2.84 | 5.27 | 3.88 | 7.14 | 1.06e-08 | 3.12e-06 | lm.nb | Adaptive Immune Response, Apoptosis, Growth Factor Signaling, Inflammatory Signaling, Innate Immune Response, NF-kB | NS\_MM\_NEUROINFLAM\_V1.0 | Nfkbia | mRNA | 0 |  | NF-kB;Innate Immune Response;Inflammatory Signaling;Growth Factor Signaling;Apoptosis;Adaptive Immune Response | mmu04024;mmu04062;mmu04064;mmu04210;mmu04380;mmu04620;mmu04621;mmu04622;mmu04623;mmu04660;mmu04662;mmu04668;mmu04722;mmu04920;mmu04931;mmu05134;mmu05140;mmu05142;mmu05145;mmu05160;mmu05161;mmu05162;mmu05164;mmu05166;mmu05168;mmu05169;mmu05200;mmu05203;mmu05215;mmu05220;mmu05222 |  | Nfkbia | slateblue1 | 16 | 10.7142857142857 |
  | Ifitm2-mRNA | 1.82 | 0.174 | 1.47 | 2.16 | 3.52 | 2.78 | 4.46 | 1.51e-08 | 4.09e-06 | lm.nb | Adaptive Immune Response, Inflammatory Signaling | NS\_MM\_NEUROINFLAM\_V1.0 | Ifitm2 | mRNA | 0 |  | Inflammatory Signaling;Adaptive Immune Response |  |  | Ifitm2 | slateblue1 | 16 | 10.4597701149425 |
  | Psmb8-mRNA | 2.99 | 0.346 | 2.31 | 3.67 | 7.95 | 4.97 | 12.7 | 1.99e-07 | 3.2e-05 | lm.nb | Adaptive Immune Response, Angiogenesis, Apoptosis, Astrocyte Function, Cell Cycle, Cytokine Signaling, Growth Factor Signaling, Inflammatory Signaling, Insulin Signaling, Microglia Function, NF-kB, Wnt | NS\_MM\_NEUROINFLAM\_V1.0 | Psmb8 | mRNA | 0 |  | Wnt;NF-kB;Microglia Function;Insulin Signaling;Inflammatory Signaling;Growth Factor Signaling;Cytokine Signaling;Cell Cycle;Astrocyte Function;Apoptosis;Angiogenesis;Adaptive Immune Response |  |  | Psmb8 | slateblue1 | 16 | 8.64161849710983 |
  | Fcgr2b-mRNA | 1.71 | 0.202 | 1.31 | 2.11 | 3.27 | 2.49 | 4.31 | 2.63e-07 | 3.57e-05 | lm.nb | Adaptive Immune Response, Autophagy | NS\_MM\_NEUROINFLAM\_V1.0 | Fcgr2b | mRNA | 0 |  | Autophagy;Adaptive Immune Response | mmu04145;mmu04380;mmu04662;mmu04666;mmu05150;mmu05152;mmu05162 |  | Fcgr2b | slateblue1 | 16 | 8.46534653465346 |
  | Ifitm3-mRNA | 2.54 | 0.344 | 1.87 | 3.22 | 5.83 | 3.65 | 9.29 | 1.51e-06 | 0.000151 | lm.nb | Adaptive Immune Response, Inflammatory Signaling | NS\_MM\_NEUROINFLAM\_V1.0 | Ifitm3 | mRNA | 0 |  | Inflammatory Signaling;Adaptive Immune Response |  |  | Ifitm3 | slateblue1 | 16 | 7.38372093023256 |
  | Ikbkb-mRNA | -0.601 | 0.0868 | -0.772 | -0.431 | 0.659 | 0.586 | 0.742 | 3.42e-06 | 0.00028 | loglinear | Adaptive Immune Response, Apoptosis, Cytokine Signaling, Growth Factor Signaling, Inflammatory Signaling, Innate Immune Response, NF-kB | NS\_MM\_NEUROINFLAM\_V1.0 | Ikbkb | mRNA | 0 |  | NF-kB;Innate Immune Response;Inflammatory Signaling;Growth Factor Signaling;Cytokine Signaling;Apoptosis;Adaptive Immune Response | mmu04010;mmu04014;mmu04062;mmu04064;mmu04068;mmu04150;mmu04151;mmu04210;mmu04380;mmu04620;mmu04621;mmu04622;mmu04623;mmu04660;mmu04662;mmu04668;mmu04722;mmu04910;mmu04920;mmu04930;mmu04931;mmu04932;mmu05142;mmu05145;mmu05160;mmu05161;mmu05164;mmu05166;mmu05168;mmu05169;mmu05200;mmu05206;mmu05212;mmu05215;mmu05220;mmu05221;mmu05222 |  | Ikbkb | slateblue1 | 16 | -6.92396313364055 |
  | Creb1-mRNA | -0.443 | 0.0668 | -0.574 | -0.312 | 0.735 | 0.672 | 0.805 | 5.69e-06 | 0.000418 | loglinear | Adaptive Immune Response, Carbohydrate Metabolism, DNA Damage, Growth Factor Signaling, Innate Immune Response, Neurons and Neurotransmission, Notch | NS\_MM\_NEUROINFLAM\_V1.0 | Creb1 | mRNA | 0 |  | Notch;Neurons and Neurotransmission;Innate Immune Response;Growth Factor Signaling;DNA Damage;Carbohydrate Metabolism;Adaptive Immune Response | mmu04022;mmu04024;mmu04151;mmu04152;mmu04211;mmu04261;mmu04380;mmu04612;mmu04668;mmu04713;mmu04725;mmu04728;mmu04911;mmu04915;mmu04916;mmu04922;mmu04924;mmu04925;mmu04931;mmu05016;mmu05030;mmu05031;mmu05034;mmu05152;mmu05161;mmu05166;mmu05203;mmu05215 |  | Creb1 | slateblue1 | 16 | -6.63173652694611 |
  | Traf6-mRNA | 0.92 | 0.166 | 0.594 | 1.25 | 1.89 | 1.51 | 2.37 | 4.54e-05 | 0.00214 | lm.nb | Adaptive Immune Response, Apoptosis, Autophagy, Cytokine Signaling, Growth Factor Signaling, Innate Immune Response, NF-kB | NS\_MM\_NEUROINFLAM\_V1.0 | Traf6 | mRNA | 0 |  | NF-kB;Innate Immune Response;Growth Factor Signaling;Cytokine Signaling;Autophagy;Apoptosis;Adaptive Immune Response | mmu04010;mmu04064;mmu04120;mmu04144;mmu04380;mmu04620;mmu04621;mmu04622;mmu04722;mmu05133;mmu05140;mmu05142;mmu05145;mmu05152;mmu05160;mmu05162;mmu05168;mmu05169;mmu05200;mmu05222 |  | Traf6 | slateblue1 | 16 | 5.54216867469879 |
  | Akt2-mRNA | -0.766 | 0.142 | -1.04 | -0.488 | 0.588 | 0.485 | 0.713 | 5.85e-05 | 0.00265 | loglinear | Adaptive Immune Response, Angiogenesis, Apoptosis, Autophagy, Carbohydrate Metabolism, Cytokine Signaling, Growth Factor Signaling, Innate Immune Response, Insulin Signaling, Neurons and Neurotransmission, Wnt | NS\_MM\_NEUROINFLAM\_V1.0 | Akt2 | mRNA | 0 |  | Wnt;Neurons and Neurotransmission;Insulin Signaling;Innate Immune Response;Growth Factor Signaling;Cytokine Signaling;Carbohydrate Metabolism;Autophagy;Apoptosis;Angiogenesis;Adaptive Immune Response | mmu04010;mmu04012;mmu04014;mmu04015;mmu04022;mmu04024;mmu04062;mmu04066;mmu04068;mmu04071;mmu04072;mmu04150;mmu04151;mmu04152;mmu04210;mmu04211;mmu04213;mmu04261;mmu04370;mmu04380;mmu04510;mmu04530;mmu04550;mmu04611;mmu04620;mmu04630;mmu04660;mmu04662;mmu04664;mmu04666;mmu04668;mmu04722;mmu04725;mmu04728;mmu04910;mmu04914;mmu04915;mmu04917;mmu04919;mmu04920;mmu04922;mmu04923;mmu04931;mmu04932;mmu04933;mmu04973;mmu05142;mmu05145;mmu05152;mmu05160;mmu05161;mmu05162;mmu05164;mmu05166;mmu05169;mmu05200;mmu05205;mmu05210;mmu05211;mmu05212;mmu05213;mmu05214;mmu05215;mmu05218;mmu05220;mmu05221;mmu05222;mmu05223;mmu05230;mmu05231 |  | Akt2 | slateblue1 | 16 | -5.3943661971831 |
  | Chuk-mRNA | -0.423 | 0.0787 | -0.577 | -0.269 | 0.746 | 0.67 | 0.83 | 6.21e-05 | 0.00275 | loglinear | Adaptive Immune Response, Apoptosis, Cytokine Signaling, Growth Factor Signaling, Inflammatory Signaling, Innate Immune Response, NF-kB | NS\_MM\_NEUROINFLAM\_V1.0 | Chuk | mRNA | 0 |  | NF-kB;Innate Immune Response;Inflammatory Signaling;Growth Factor Signaling;Cytokine Signaling;Apoptosis;Adaptive Immune Response | mmu04010;mmu04014;mmu04062;mmu04064;mmu04068;mmu04151;mmu04210;mmu04380;mmu04620;mmu04621;mmu04622;mmu04623;mmu04660;mmu04662;mmu04668;mmu04920;mmu05142;mmu05145;mmu05160;mmu05161;mmu05162;mmu05166;mmu05168;mmu05169;mmu05200;mmu05212;mmu05215;mmu05220;mmu05221;mmu05222 |  | Chuk | slateblue1 | 16 | -5.37484116899619 |
  | Ago4-mRNA | -2.72 | 0.514 | -3.72 | -1.71 | 0.152 | 0.0757 | 0.306 | 7.46e-05 | 0.00321 | lm.nb | Adaptive Immune Response, Cellular Stress, Epigenetic Regulation, Growth Factor Signaling, Notch, Wnt | NS\_MM\_NEUROINFLAM\_V1.0 | Ago4 | mRNA | 0 |  | Wnt;Notch;Growth Factor Signaling;Epigenetic Regulation;Cellular Stress;Adaptive Immune Response |  |  | Ago4 | slateblue1 | 16 | -5.29182879377432 |
  | Ripk2-mRNA | 1 | 0.193 | 0.627 | 1.38 | 2.01 | 1.54 | 2.61 | 8.44e-05 | 0.00355 | loglinear | Adaptive Immune Response, Apoptosis, Cytokine Signaling, Growth Factor Signaling, Innate Immune Response | NS\_MM\_NEUROINFLAM\_V1.0 | Ripk2 | mRNA | 0 |  | Innate Immune Response;Growth Factor Signaling;Cytokine Signaling;Apoptosis;Adaptive Immune Response | mmu04621;mmu04722;mmu05152 |  | Ripk2 | slateblue1 | 16 | 5.18134715025907 |
  | Lgmn-mRNA | 0.371 | 0.0832 | 0.208 | 0.534 | 1.29 | 1.15 | 1.45 | 0.000401 | 0.0134 | loglinear | Adaptive Immune Response, Innate Immune Response | NS\_MM\_NEUROINFLAM\_V1.0 | Lgmn | mRNA | 0 |  | Innate Immune Response;Adaptive Immune Response | mmu04142;mmu04612 |  | Lgmn | slateblue2 | 16 | 4.45913461538462 |
  | Fcgr3-mRNA | 1.62 | 0.365 | 0.902 | 2.33 | 3.07 | 1.87 | 5.04 | 0.000421 | 0.0139 | lm.nb | Adaptive Immune Response, Autophagy, Inflammatory Signaling, Innate Immune Response | NS\_MM\_NEUROINFLAM\_V1.0 | Fcgr3 | mRNA | 0 |  | Innate Immune Response;Inflammatory Signaling;Autophagy;Adaptive Immune Response | mmu04145;mmu04380;mmu05140;mmu05150;mmu05152 |  | Fcgr3 | slateblue2 | 16 | 4.43835616438356 |
  | Tubb3-mRNA | -0.664 | 0.158 | -0.975 | -0.354 | 0.631 | 0.509 | 0.782 | 0.000683 | 0.021 | lm.nb | Adaptive Immune Response, Apoptosis, Cell Cycle, Neurons and Neurotransmission | NS\_MM\_NEUROINFLAM\_V1.0 | Tubb3 | mRNA | 0 |  | Neurons and Neurotransmission;Cell Cycle;Apoptosis;Adaptive Immune Response | mmu04145;mmu04540 |  | Tubb3 | slateblue2 | 16 | -4.20253164556962 |
  | Mapk10-mRNA | -0.571 | 0.138 | -0.842 | -0.3 | 0.673 | 0.558 | 0.812 | 0.000782 | 0.0231 | lm.nb | Adaptive Immune Response, Apoptosis, Autophagy, Cellular Stress, Growth Factor Signaling, Innate Immune Response, Wnt | NS\_MM\_NEUROINFLAM\_V1.0 | Mapk10 | mRNA | 0 |  | Wnt;Innate Immune Response;Growth Factor Signaling;Cellular Stress;Autophagy;Apoptosis;Adaptive Immune Response | mmu04010;mmu04012;mmu04014;mmu04024;mmu04068;mmu04071;mmu04141;mmu04310;mmu04380;mmu04510;mmu04620;mmu04621;mmu04622;mmu04664;mmu04668;mmu04722;mmu04723;mmu04728;mmu04750;mmu04910;mmu04912;mmu04914;mmu04917;mmu04920;mmu04930;mmu04931;mmu04932;mmu04933;mmu05132;mmu05133;mmu05142;mmu05145;mmu05152;mmu05160;mmu05161;mmu05164;mmu05168;mmu05169;mmu05200;mmu05210;mmu05212;mmu05231 |  | Mapk10 | slateblue2 | 16 | -4.13768115942029 |
  | Prkcq-mRNA | -0.735 | 0.181 | -1.09 | -0.379 | 0.601 | 0.47 | 0.769 | 0.000927 | 0.0267 | lm.nb | Adaptive Immune Response, Apoptosis, Autophagy, NF-kB, Oligodendrocyte Function | NS\_MM\_NEUROINFLAM\_V1.0 | Prkcq | mRNA | 0 |  | Oligodendrocyte Function;NF-kB;Autophagy;Apoptosis;Adaptive Immune Response | mmu04064;mmu04270;mmu04530;mmu04660;mmu04750;mmu04920;mmu04931;mmu05162 | Oligodendrocytes | Prkcq | slateblue2 | 16 | -4.06077348066298 |
  | Ctss-mRNA | 0.833 | 0.206 | 0.429 | 1.24 | 1.78 | 1.35 | 2.36 | 0.000938 | 0.0267 | loglinear | Adaptive Immune Response, Innate Immune Response, Matrix Remodeling, Microglia Function | NS\_MM\_NEUROINFLAM\_V1.0 | Ctss | mRNA | 0 |  | Microglia Function;Matrix Remodeling;Innate Immune Response;Adaptive Immune Response | mmu04142;mmu04145;mmu04612;mmu05152 |  | Ctss | slateblue2 | 16 | 4.04368932038835 |
  | Pik3cd-mRNA | -0.63 | 0.159 | -0.942 | -0.318 | 0.646 | 0.52 | 0.802 | 0.00114 | 0.0306 | lm.nb | Adaptive Immune Response, Apoptosis, Autophagy, Cytokine Signaling, Growth Factor Signaling, Innate Immune Response | NS\_MM\_NEUROINFLAM\_V1.0 | Pik3cd | mRNA | 0 |  | Innate Immune Response;Growth Factor Signaling;Cytokine Signaling;Autophagy;Apoptosis;Adaptive Immune Response |  |  |  | slateblue2 | 16 | -3.9622641509434 |

  ##### DE Results - Adaptive Immune Response genes - DPI: 3 vs.CTRL

  Table displaying each sample's global significance scores and directed global significance scores as defined in the heatmaps above. The global significance score is calculated as the square root of the mean squared t-statistic for the genes in a gene set, with t-statistics coming from the linear regression underlying our differential expression analysis. The directed global significance score is calculated as the square root of the mean signed squared t-statistic for the genes in a gene set, with t-statistics coming from the linear regression underlying our differential expression analysis.

DPI:
- DPI: differential expression in 1 vs. baseline of CTRL
- DPI: differential expression in 2 vs. baseline of CTRL
- DPI: differential expression in 3 vs. baseline of CTRL

- **Volcano Plot: DPI: 1 vs.CTRL  
  More Plot Information**


  ##### Volcano Plot: DPI: 1 vs.CTRL

  Volcano plot displaying each gene's -log10(p-value) and log2 fold change for the selected covariate. Highly statistically significant genes fall at the top of the plot, and highly differentially expressed genes fall to either side. Genes within the selected gene set are highlighted in orange. Horizontal lines indicate various False Discovery Rate (FDR) thresholds.
- **DE Results - Angiogenesis genes - DPI: 1 vs.CTRL  
  More Plot Information  Download CSV Data**

  | Probe Label | Log2 fold change | std error (log2) | Lower confidence limit (log2) | Upper confidence limit (log2) | Linear fold change | Lower confidence limit (linear) | Upper confidence limit (linear) | P-value | BY.p.value | method | Gene.sets | Codeset.Name | Probe.Label | Analyte.Type | Is.Control | Control.Type | Probe.Annotation | KEGG.Pathways | Cell.Type | Official.Gene.Name | volcanocol | volcanopch | tstats.all |
  | --- | --- | --- | --- | --- | --- | --- | --- | --- | --- | --- | --- | --- | --- | --- | --- | --- | --- | --- | --- | --- | --- | --- | --- |
  | Dusp7-mRNA | -0.378 | 0.0611 | -0.497 | -0.258 | 0.77 | 0.708 | 0.836 | 1.32e-05 | 0.0093 | loglinear | Adaptive Immune Response, Angiogenesis, Cytokine Signaling, Growth Factor Signaling, Innate Immune Response, Insulin Signaling, Microglia Function | NS\_MM\_NEUROINFLAM\_V1.0 | Dusp7 | mRNA | 0 |  | Microglia Function;Insulin Signaling;Innate Immune Response;Growth Factor Signaling;Cytokine Signaling;Angiogenesis;Adaptive Immune Response | mmu04010 |  | Dusp7 | slateblue2 | 16 | -6.18657937806874 |
  | Prkacb-mRNA | -0.327 | 0.0629 | -0.451 | -0.204 | 0.797 | 0.732 | 0.868 | 8.65e-05 | 0.0321 | loglinear | Adaptive Immune Response, Angiogenesis, Apoptosis, Autophagy, Growth Factor Signaling, Lipid Metabolism, Neurons and Neurotransmission, Wnt | NS\_MM\_NEUROINFLAM\_V1.0 | Prkacb | mRNA | 0 |  | Wnt;Neurons and Neurotransmission;Lipid Metabolism;Growth Factor Signaling;Autophagy;Apoptosis;Angiogenesis;Adaptive Immune Response | mmu04010;mmu04014;mmu04020;mmu04024;mmu04062;mmu04114;mmu04210;mmu04211;mmu04213;mmu04261;mmu04270;mmu04310;mmu04540;mmu04611;mmu04713;mmu04720;mmu04723;mmu04724;mmu04725;mmu04726;mmu04728;mmu04750;mmu04910;mmu04911;mmu04912;mmu04913;mmu04914;mmu04915;mmu04916;mmu04919;mmu04921;mmu04922;mmu04923;mmu04924;mmu04925;mmu05012;mmu05020;mmu05030;mmu05031;mmu05146;mmu05166;mmu05169;mmu05200;mmu05203;mmu05205;mmu05414 |  | Prkacb | slateblue2 | 16 | -5.19872813990461 |
  | Pak1-mRNA | -0.284 | 0.0554 | -0.393 | -0.176 | 0.821 | 0.762 | 0.885 | 1e-04 | 0.0321 | loglinear | Adaptive Immune Response, Angiogenesis, Growth Factor Signaling, Innate Immune Response, Microglia Function | NS\_MM\_NEUROINFLAM\_V1.0 | Pak1 | mRNA | 0 |  | Microglia Function;Innate Immune Response;Growth Factor Signaling;Angiogenesis;Adaptive Immune Response | mmu04010;mmu04012;mmu04014;mmu04024;mmu04062;mmu04360;mmu04510;mmu04650;mmu04660;mmu04666;mmu04810;mmu05205;mmu05211 |  | Pak1 | slateblue2 | 16 | -5.12635379061372 |
  | Braf-mRNA | -0.641 | 0.154 | -0.943 | -0.339 | 0.641 | 0.52 | 0.791 | 0.000739 | 0.0815 | lm.nb | Adaptive Immune Response, Angiogenesis, Apoptosis, Cytokine Signaling, Growth Factor Signaling, Innate Immune Response, Insulin Signaling, Neurons and Neurotransmission | NS\_MM\_NEUROINFLAM\_V1.0 | Braf | mRNA | 0 |  | Neurons and Neurotransmission;Insulin Signaling;Innate Immune Response;Growth Factor Signaling;Cytokine Signaling;Apoptosis;Angiogenesis;Adaptive Immune Response | mmu04010;mmu04012;mmu04015;mmu04024;mmu04062;mmu04068;mmu04150;mmu04270;mmu04510;mmu04650;mmu04720;mmu04722;mmu04726;mmu04730;mmu04810;mmu04910;mmu04914;mmu05034;mmu05160;mmu05200;mmu05205;mmu05210;mmu05211;mmu05212;mmu05213;mmu05214;mmu05215;mmu05216;mmu05218;mmu05219;mmu05220;mmu05221;mmu05223 |  | Braf | slateblue3 | 16 | -4.16233766233766 |
  | Psmb8-mRNA | 1.36 | 0.365 | 0.649 | 2.08 | 2.57 | 1.57 | 4.23 | 0.00179 | 0.129 | lm.nb | Adaptive Immune Response, Angiogenesis, Apoptosis, Astrocyte Function, Cell Cycle, Cytokine Signaling, Growth Factor Signaling, Inflammatory Signaling, Insulin Signaling, Microglia Function, NF-kB, Wnt | NS\_MM\_NEUROINFLAM\_V1.0 | Psmb8 | mRNA | 0 |  | Wnt;NF-kB;Microglia Function;Insulin Signaling;Inflammatory Signaling;Growth Factor Signaling;Cytokine Signaling;Cell Cycle;Astrocyte Function;Apoptosis;Angiogenesis;Adaptive Immune Response |  |  | Psmb8 | slateblue4 | 16 | 3.72602739726027 |
  | Rac1-mRNA | -0.928 | 0.256 | -1.43 | -0.426 | 0.525 | 0.371 | 0.744 | 0.00229 | 0.149 | lm.nb | Adaptive Immune Response, Angiogenesis, Autophagy, Growth Factor Signaling, Innate Immune Response, Neurons and Neurotransmission, Wnt | NS\_MM\_NEUROINFLAM\_V1.0 | Rac1 | mRNA | 0 |  | Wnt;Neurons and Neurotransmission;Innate Immune Response;Growth Factor Signaling;Autophagy;Angiogenesis;Adaptive Immune Response | mmu04010;mmu04014;mmu04015;mmu04024;mmu04062;mmu04071;mmu04145;mmu04151;mmu04310;mmu04360;mmu04370;mmu04380;mmu04510;mmu04520;mmu04620;mmu04650;mmu04662;mmu04664;mmu04666;mmu04670;mmu04722;mmu04810;mmu04932;mmu04933;mmu05014;mmu05100;mmu05132;mmu05200;mmu05203;mmu05205;mmu05210;mmu05211;mmu05212;mmu05231;mmu05416 |  | Rac1 | slateblue4 | 16 | -3.625 |
  | Rhoa-mRNA | -0.902 | 0.261 | -1.41 | -0.391 | 0.535 | 0.375 | 0.762 | 0.00322 | 0.18 | lm.nb | Angiogenesis, Autophagy, Growth Factor Signaling, Wnt | NS\_MM\_NEUROINFLAM\_V1.0 | Rhoa | mRNA | 0 |  | Wnt;Growth Factor Signaling;Autophagy;Angiogenesis | mmu04014;mmu04015;mmu04022;mmu04024;mmu04062;mmu04071;mmu04072;mmu04144;mmu04270;mmu04310;mmu04350;mmu04360;mmu04510;mmu04520;mmu04530;mmu04611;mmu04660;mmu04670;mmu04722;mmu04810;mmu04921;mmu05100;mmu05133;mmu05152;mmu05200;mmu05203;mmu05205;mmu05206;mmu05210 |  | Rhoa | slateblue4 | 16 | -3.45593869731801 |
  | Map2k1-mRNA | -1.03 | 0.32 | -1.65 | -0.399 | 0.491 | 0.318 | 0.759 | 0.00553 | 0.224 | lm.nb | Adaptive Immune Response, Angiogenesis, Autophagy, Cytokine Signaling, Growth Factor Signaling, Innate Immune Response, Insulin Signaling, Microglia Function | NS\_MM\_NEUROINFLAM\_V1.0 | Map2k1 | mRNA | 0 |  | Microglia Function;Insulin Signaling;Innate Immune Response;Growth Factor Signaling;Cytokine Signaling;Autophagy;Angiogenesis;Adaptive Immune Response | mmu04010;mmu04012;mmu04014;mmu04015;mmu04022;mmu04024;mmu04062;mmu04066;mmu04068;mmu04071;mmu04072;mmu04114;mmu04151;mmu04270;mmu04370;mmu04380;mmu04510;mmu04540;mmu04550;mmu04620;mmu04650;mmu04660;mmu04662;mmu04664;mmu04666;mmu04668;mmu04720;mmu04722;mmu04725;mmu04726;mmu04730;mmu04810;mmu04910;mmu04912;mmu04914;mmu04915;mmu04916;mmu04917;mmu04919;mmu04921;mmu05020;mmu05034;mmu05161;mmu05164;mmu05200;mmu05205;mmu05206;mmu05210;mmu05211;mmu05212;mmu05213;mmu05214;mmu05215;mmu05216;mmu05218;mmu05219;mmu05220;mmu05221;mmu05223;mmu05230;mmu05231 |  | Map2k1 | slateblue4 | 16 | -3.21875 |
  | Dlg4-mRNA | -0.175 | 0.056 | -0.284 | -0.0649 | 0.886 | 0.821 | 0.956 | 0.00662 | 0.246 | loglinear | Adaptive Immune Response, Angiogenesis, Cytokine Signaling, Growth Factor Signaling, Insulin Signaling, Neurons and Neurotransmission | NS\_MM\_NEUROINFLAM\_V1.0 | Dlg4 | mRNA | 0 |  | Neurons and Neurotransmission;Insulin Signaling;Growth Factor Signaling;Cytokine Signaling;Angiogenesis;Adaptive Immune Response | mmu04390;mmu04724;mmu05016;mmu05030 |  | Dlg4 | slateblue4 | 16 | -3.125 |
  | Nefl-mRNA | -0.382 | 0.123 | -0.624 | -0.141 | 0.767 | 0.649 | 0.907 | 0.00687 | 0.248 | lm.nb | Adaptive Immune Response, Angiogenesis, Cytokine Signaling, Growth Factor Signaling, Insulin Signaling, Neurons and Neurotransmission | NS\_MM\_NEUROINFLAM\_V1.0 | Nefl | mRNA | 0 |  | Neurons and Neurotransmission;Insulin Signaling;Growth Factor Signaling;Cytokine Signaling;Angiogenesis;Adaptive Immune Response | mmu05014 |  | Nefl | slateblue4 | 16 | -3.10569105691057 |
  | Mapk14-mRNA | -1.06 | 0.362 | -1.77 | -0.355 | 0.478 | 0.293 | 0.782 | 0.00961 | 0.3 | lm.nb | Angiogenesis, Autophagy, Cellular Stress, Growth Factor Signaling, Innate Immune Response | NS\_MM\_NEUROINFLAM\_V1.0 | Mapk14 | mRNA | 0 |  | Innate Immune Response;Growth Factor Signaling;Cellular Stress;Autophagy;Angiogenesis | mmu04010;mmu04015;mmu04068;mmu04071;mmu04261;mmu04370;mmu04380;mmu04550;mmu04611;mmu04620;mmu04621;mmu04622;mmu04660;mmu04664;mmu04668;mmu04670;mmu04722;mmu04723;mmu04728;mmu04750;mmu04912;mmu04914;mmu04917;mmu04933;mmu05014;mmu05132;mmu05133;mmu05140;mmu05142;mmu05145;mmu05152;mmu05160;mmu05164;mmu05169;mmu05205 |  | Mapk14 | slateblue4 | 16 | -2.92817679558011 |
  | Pik3r2-mRNA | -0.223 | 0.0793 | -0.378 | -0.0676 | 0.857 | 0.769 | 0.954 | 0.0125 | 0.357 | loglinear | Adaptive Immune Response, Angiogenesis, Apoptosis, Autophagy, Cytokine Signaling, Growth Factor Signaling, Innate Immune Response, Insulin Signaling | NS\_MM\_NEUROINFLAM\_V1.0 | Pik3r2 | mRNA | 0 |  | Insulin Signaling;Innate Immune Response;Growth Factor Signaling;Cytokine Signaling;Autophagy;Apoptosis;Angiogenesis;Adaptive Immune Response | mmu04012;mmu04014;mmu04015;mmu04022;mmu04024;mmu04062;mmu04066;mmu04068;mmu04070;mmu04071;mmu04072;mmu04150;mmu04151;mmu04152;mmu04210;mmu04211;mmu04213;mmu04261;mmu04370;mmu04380;mmu04510;mmu04550;mmu04611;mmu04620;mmu04630;mmu04650;mmu04660;mmu04662;mmu04664;mmu04666;mmu04668;mmu04670;mmu04722;mmu04725;mmu04750;mmu04810;mmu04910;mmu04914;mmu04915;mmu04917;mmu04919;mmu04921;mmu04923;mmu04930;mmu04931;mmu04932;mmu04933;mmu04960;mmu04973;mmu05100;mmu05142;mmu05145;mmu05146;mmu05160;mmu05161;mmu05162;mmu05164;mmu05166;mmu05169;mmu05200;mmu05203;mmu05205;mmu05210;mmu05211;mmu05212;mmu05213;mmu05214;mmu05215;mmu05218;mmu05220;mmu05221;mmu05222;mmu05223;mmu05230;mmu05231 |  | Pik3r2 | slateblue4 | 16 | -2.81210592686003 |
  | Pik3ca-mRNA | -0.287 | 0.107 | -0.497 | -0.0771 | 0.82 | 0.709 | 0.948 | 0.0164 | 0.417 | lm.nb | Adaptive Immune Response, Angiogenesis, Apoptosis, Autophagy, Carbohydrate Metabolism, Cytokine Signaling, Growth Factor Signaling, Innate Immune Response, Insulin Signaling, Lipid Metabolism | NS\_MM\_NEUROINFLAM\_V1.0 | Pik3ca | mRNA | 0 |  | Lipid Metabolism;Insulin Signaling;Innate Immune Response;Growth Factor Signaling;Cytokine Signaling;Carbohydrate Metabolism;Autophagy;Apoptosis;Angiogenesis;Adaptive Immune Response | mmu00562;mmu04012;mmu04014;mmu04015;mmu04022;mmu04024;mmu04062;mmu04066;mmu04068;mmu04070;mmu04071;mmu04072;mmu04150;mmu04151;mmu04152;mmu04210;mmu04211;mmu04213;mmu04261;mmu04370;mmu04380;mmu04510;mmu04550;mmu04611;mmu04620;mmu04630;mmu04650;mmu04660;mmu04662;mmu04664;mmu04666;mmu04668;mmu04670;mmu04722;mmu04725;mmu04750;mmu04810;mmu04910;mmu04914;mmu04915;mmu04917;mmu04919;mmu04921;mmu04923;mmu04930;mmu04931;mmu04932;mmu04933;mmu04960;mmu04973;mmu05100;mmu05142;mmu05145;mmu05146;mmu05160;mmu05161;mmu05162;mmu05164;mmu05166;mmu05169;mmu05200;mmu05203;mmu05205;mmu05210;mmu05211;mmu05212;mmu05213;mmu05214;mmu05215;mmu05218;mmu05220;mmu05221;mmu05222;mmu05223;mmu05230;mmu05231 |  | Pik3ca | slateblue4 | 16 | -2.68224299065421 |
  | Dock1-mRNA | -0.496 | 0.186 | -0.861 | -0.131 | 0.709 | 0.551 | 0.913 | 0.017 | 0.422 | loglinear | Angiogenesis, Autophagy | NS\_MM\_NEUROINFLAM\_V1.0 | Dock1 | mRNA | 0 |  | Autophagy;Angiogenesis | mmu04510;mmu04810;mmu05100 |  | Dock1 | slateblue4 | 16 | -2.66666666666667 |
  | Grin2b-mRNA | -0.254 | 0.101 | -0.451 | -0.057 | 0.839 | 0.731 | 0.961 | 0.0225 | 0.508 | lm.nb | Adaptive Immune Response, Angiogenesis, Cytokine Signaling, Growth Factor Signaling, Insulin Signaling, Neurons and Neurotransmission | NS\_MM\_NEUROINFLAM\_V1.0 | Grin2b | mRNA | 0 |  | Neurons and Neurotransmission;Insulin Signaling;Growth Factor Signaling;Cytokine Signaling;Angiogenesis;Adaptive Immune Response | mmu04014;mmu04015;mmu04024;mmu04080;mmu04713;mmu04720;mmu04724;mmu04728;mmu05010;mmu05014;mmu05016;mmu05030;mmu05031;mmu05033;mmu05034;mmu05322 |  | Grin2b | azure4 | 1 | -2.51485148514851 |
  | Vav1-mRNA | 1.65 | 0.659 | 0.363 | 2.95 | 3.15 | 1.29 | 7.7 | 0.0231 | 0.51 | lm.nb | Adaptive Immune Response, Angiogenesis, Autophagy, Cytokine Signaling, Growth Factor Signaling, Innate Immune Response | NS\_MM\_NEUROINFLAM\_V1.0 | Vav1 | mRNA | 0 |  | Innate Immune Response;Growth Factor Signaling;Cytokine Signaling;Autophagy;Angiogenesis;Adaptive Immune Response | mmu04024;mmu04062;mmu04510;mmu04650;mmu04660;mmu04662;mmu04664;mmu04666;mmu04670;mmu04810 |  | Vav1 | azure4 | 1 | 2.50379362670713 |
  | Pik3r1-mRNA | -0.169 | 0.0713 | -0.308 | -0.0288 | 0.89 | 0.808 | 0.98 | 0.0311 | 0.62 | loglinear | Adaptive Immune Response, Angiogenesis, Apoptosis, Autophagy, Carbohydrate Metabolism, Cytokine Signaling, Growth Factor Signaling, Innate Immune Response, Insulin Signaling, Lipid Metabolism | NS\_MM\_NEUROINFLAM\_V1.0 | Pik3r1 | mRNA | 0 |  | Lipid Metabolism;Insulin Signaling;Innate Immune Response;Growth Factor Signaling;Cytokine Signaling;Carbohydrate Metabolism;Autophagy;Apoptosis;Angiogenesis;Adaptive Immune Response | mmu04012;mmu04014;mmu04015;mmu04022;mmu04024;mmu04062;mmu04066;mmu04068;mmu04070;mmu04071;mmu04072;mmu04150;mmu04151;mmu04152;mmu04210;mmu04211;mmu04213;mmu04261;mmu04370;mmu04380;mmu04510;mmu04550;mmu04611;mmu04620;mmu04630;mmu04650;mmu04660;mmu04662;mmu04664;mmu04666;mmu04668;mmu04670;mmu04722;mmu04725;mmu04750;mmu04810;mmu04910;mmu04914;mmu04915;mmu04917;mmu04919;mmu04921;mmu04923;mmu04930;mmu04931;mmu04932;mmu04933;mmu04960;mmu04973;mmu05100;mmu05142;mmu05145;mmu05146;mmu05160;mmu05161;mmu05162;mmu05164;mmu05166;mmu05169;mmu05200;mmu05203;mmu05205;mmu05210;mmu05211;mmu05212;mmu05213;mmu05214;mmu05215;mmu05218;mmu05220;mmu05221;mmu05222;mmu05223;mmu05230;mmu05231 |  | Pik3r1 | azure4 | 1 | -2.37026647966339 |
  | Plxdc2-mRNA | 0.241 | 0.112 | 0.022 | 0.46 | 1.18 | 1.02 | 1.38 | 0.0466 | 0.798 | lm.nb | Angiogenesis | NS\_MM\_NEUROINFLAM\_V1.0 | Plxdc2 | mRNA | 0 |  | Angiogenesis |  |  | Plxdc2 | azure4 | 1 | 2.15178571428571 |
  | Akt2-mRNA | -0.291 | 0.142 | -0.568 | -0.0129 | 0.818 | 0.674 | 0.991 | 0.057 | 0.91 | loglinear | Adaptive Immune Response, Angiogenesis, Apoptosis, Autophagy, Carbohydrate Metabolism, Cytokine Signaling, Growth Factor Signaling, Innate Immune Response, Insulin Signaling, Neurons and Neurotransmission, Wnt | NS\_MM\_NEUROINFLAM\_V1.0 | Akt2 | mRNA | 0 |  | Wnt;Neurons and Neurotransmission;Insulin Signaling;Innate Immune Response;Growth Factor Signaling;Cytokine Signaling;Carbohydrate Metabolism;Autophagy;Apoptosis;Angiogenesis;Adaptive Immune Response | mmu04010;mmu04012;mmu04014;mmu04015;mmu04022;mmu04024;mmu04062;mmu04066;mmu04068;mmu04071;mmu04072;mmu04150;mmu04151;mmu04152;mmu04210;mmu04211;mmu04213;mmu04261;mmu04370;mmu04380;mmu04510;mmu04530;mmu04550;mmu04611;mmu04620;mmu04630;mmu04660;mmu04662;mmu04664;mmu04666;mmu04668;mmu04722;mmu04725;mmu04728;mmu04910;mmu04914;mmu04915;mmu04917;mmu04919;mmu04920;mmu04922;mmu04923;mmu04931;mmu04932;mmu04933;mmu04973;mmu05142;mmu05145;mmu05152;mmu05160;mmu05161;mmu05162;mmu05164;mmu05166;mmu05169;mmu05200;mmu05205;mmu05210;mmu05211;mmu05212;mmu05213;mmu05214;mmu05215;mmu05218;mmu05220;mmu05221;mmu05222;mmu05223;mmu05230;mmu05231 |  | Akt2 | azure4 | 1 | -2.04929577464789 |
  | Erbb3-mRNA | 1.01 | 0.526 | -0.0216 | 2.04 | 2.01 | 0.985 | 4.11 | 0.073 | 1 | lm.nb | Adaptive Immune Response, Angiogenesis, Cytokine Signaling, Growth Factor Signaling, Insulin Signaling, Oligodendrocyte Function | NS\_MM\_NEUROINFLAM\_V1.0 | Erbb3 | mRNA | 0 |  | Oligodendrocyte Function;Insulin Signaling;Growth Factor Signaling;Cytokine Signaling;Angiogenesis;Adaptive Immune Response | mmu04012;mmu04020;mmu04144;mmu05205;mmu05206 | Oligodendrocytes | Erbb3 | azure4 | 1 | 1.92015209125475 |

  ##### DE Results - Angiogenesis genes - DPI: 1 vs.CTRL

  Table displaying each sample's global significance scores and directed global significance scores as defined in the heatmaps above. The global significance score is calculated as the square root of the mean squared t-statistic for the genes in a gene set, with t-statistics coming from the linear regression underlying our differential expression analysis. The directed global significance score is calculated as the square root of the mean signed squared t-statistic for the genes in a gene set, with t-statistics coming from the linear regression underlying our differential expression analysis.

- **Volcano Plot: DPI: 2 vs.CTRL  
  More Plot Information**


  ##### Volcano Plot: DPI: 2 vs.CTRL

  Volcano plot displaying each gene's -log10(p-value) and log2 fold change for the selected covariate. Highly statistically significant genes fall at the top of the plot, and highly differentially expressed genes fall to either side. Genes within the selected gene set are highlighted in orange. Horizontal lines indicate various False Discovery Rate (FDR) thresholds.
- **DE Results - Angiogenesis genes - DPI: 2 vs.CTRL  
  More Plot Information  Download CSV Data**

  | Probe Label | Log2 fold change | std error (log2) | Lower confidence limit (log2) | Upper confidence limit (log2) | Linear fold change | Lower confidence limit (linear) | Upper confidence limit (linear) | P-value | BY.p.value | method | Gene.sets | Codeset.Name | Probe.Label | Analyte.Type | Is.Control | Control.Type | Probe.Annotation | KEGG.Pathways | Cell.Type | Official.Gene.Name | volcanocol | volcanopch | tstats.all |
  | --- | --- | --- | --- | --- | --- | --- | --- | --- | --- | --- | --- | --- | --- | --- | --- | --- | --- | --- | --- | --- | --- | --- | --- |
  | Dusp7-mRNA | -0.496 | 0.0611 | -0.616 | -0.377 | 0.709 | 0.652 | 0.77 | 4.53e-07 | 9.99e-05 | loglinear | Adaptive Immune Response, Angiogenesis, Cytokine Signaling, Growth Factor Signaling, Innate Immune Response, Insulin Signaling, Microglia Function | NS\_MM\_NEUROINFLAM\_V1.0 | Dusp7 | mRNA | 0 |  | Microglia Function;Insulin Signaling;Innate Immune Response;Growth Factor Signaling;Cytokine Signaling;Angiogenesis;Adaptive Immune Response | mmu04010 |  | Dusp7 | slateblue1 | 16 | -8.11783960720131 |
  | Dock1-mRNA | -1.49 | 0.186 | -1.85 | -1.12 | 0.357 | 0.277 | 0.459 | 5.68e-07 | 0.000111 | loglinear | Angiogenesis, Autophagy | NS\_MM\_NEUROINFLAM\_V1.0 | Dock1 | mRNA | 0 |  | Autophagy;Angiogenesis | mmu04510;mmu04810;mmu05100 |  | Dock1 | slateblue1 | 16 | -8.01075268817204 |
  | Psmb8-mRNA | 2.23 | 0.352 | 1.54 | 2.92 | 4.7 | 2.91 | 7.59 | 9.89e-06 | 0.000997 | lm.nb | Adaptive Immune Response, Angiogenesis, Apoptosis, Astrocyte Function, Cell Cycle, Cytokine Signaling, Growth Factor Signaling, Inflammatory Signaling, Insulin Signaling, Microglia Function, NF-kB, Wnt | NS\_MM\_NEUROINFLAM\_V1.0 | Psmb8 | mRNA | 0 |  | Wnt;NF-kB;Microglia Function;Insulin Signaling;Inflammatory Signaling;Growth Factor Signaling;Cytokine Signaling;Cell Cycle;Astrocyte Function;Apoptosis;Angiogenesis;Adaptive Immune Response |  |  | Psmb8 | slateblue1 | 16 | 6.33522727272727 |
  | Prkacb-mRNA | -0.322 | 0.0629 | -0.445 | -0.199 | 0.8 | 0.735 | 0.871 | 0.000104 | 0.00562 | loglinear | Adaptive Immune Response, Angiogenesis, Apoptosis, Autophagy, Growth Factor Signaling, Lipid Metabolism, Neurons and Neurotransmission, Wnt | NS\_MM\_NEUROINFLAM\_V1.0 | Prkacb | mRNA | 0 |  | Wnt;Neurons and Neurotransmission;Lipid Metabolism;Growth Factor Signaling;Autophagy;Apoptosis;Angiogenesis;Adaptive Immune Response | mmu04010;mmu04014;mmu04020;mmu04024;mmu04062;mmu04114;mmu04210;mmu04211;mmu04213;mmu04261;mmu04270;mmu04310;mmu04540;mmu04611;mmu04713;mmu04720;mmu04723;mmu04724;mmu04725;mmu04726;mmu04728;mmu04750;mmu04910;mmu04911;mmu04912;mmu04913;mmu04914;mmu04915;mmu04916;mmu04919;mmu04921;mmu04922;mmu04923;mmu04924;mmu04925;mmu05012;mmu05020;mmu05030;mmu05031;mmu05146;mmu05166;mmu05169;mmu05200;mmu05203;mmu05205;mmu05414 |  | Prkacb | slateblue1 | 16 | -5.11923688394277 |
  | Pak1-mRNA | -0.263 | 0.0554 | -0.372 | -0.155 | 0.833 | 0.773 | 0.898 | 0.000215 | 0.0092 | loglinear | Adaptive Immune Response, Angiogenesis, Growth Factor Signaling, Innate Immune Response, Microglia Function | NS\_MM\_NEUROINFLAM\_V1.0 | Pak1 | mRNA | 0 |  | Microglia Function;Innate Immune Response;Growth Factor Signaling;Angiogenesis;Adaptive Immune Response | mmu04010;mmu04012;mmu04014;mmu04024;mmu04062;mmu04360;mmu04510;mmu04650;mmu04660;mmu04666;mmu04810;mmu05205;mmu05211 |  | Pak1 | slateblue1 | 16 | -4.74729241877256 |
  | Pik3r2-mRNA | -0.338 | 0.0793 | -0.494 | -0.183 | 0.791 | 0.71 | 0.881 | 0.000587 | 0.0197 | loglinear | Adaptive Immune Response, Angiogenesis, Apoptosis, Autophagy, Cytokine Signaling, Growth Factor Signaling, Innate Immune Response, Insulin Signaling | NS\_MM\_NEUROINFLAM\_V1.0 | Pik3r2 | mRNA | 0 |  | Insulin Signaling;Innate Immune Response;Growth Factor Signaling;Cytokine Signaling;Autophagy;Apoptosis;Angiogenesis;Adaptive Immune Response | mmu04012;mmu04014;mmu04015;mmu04022;mmu04024;mmu04062;mmu04066;mmu04068;mmu04070;mmu04071;mmu04072;mmu04150;mmu04151;mmu04152;mmu04210;mmu04211;mmu04213;mmu04261;mmu04370;mmu04380;mmu04510;mmu04550;mmu04611;mmu04620;mmu04630;mmu04650;mmu04660;mmu04662;mmu04664;mmu04666;mmu04668;mmu04670;mmu04722;mmu04725;mmu04750;mmu04810;mmu04910;mmu04914;mmu04915;mmu04917;mmu04919;mmu04921;mmu04923;mmu04930;mmu04931;mmu04932;mmu04933;mmu04960;mmu04973;mmu05100;mmu05142;mmu05145;mmu05146;mmu05160;mmu05161;mmu05162;mmu05164;mmu05166;mmu05169;mmu05200;mmu05203;mmu05205;mmu05210;mmu05211;mmu05212;mmu05213;mmu05214;mmu05215;mmu05218;mmu05220;mmu05221;mmu05222;mmu05223;mmu05230;mmu05231 |  | Pik3r2 | slateblue2 | 16 | -4.26229508196721 |
  | Nefl-mRNA | -0.522 | 0.124 | -0.764 | -0.28 | 0.696 | 0.589 | 0.824 | 0.000651 | 0.0215 | lm.nb | Adaptive Immune Response, Angiogenesis, Cytokine Signaling, Growth Factor Signaling, Insulin Signaling, Neurons and Neurotransmission | NS\_MM\_NEUROINFLAM\_V1.0 | Nefl | mRNA | 0 |  | Neurons and Neurotransmission;Insulin Signaling;Growth Factor Signaling;Cytokine Signaling;Angiogenesis;Adaptive Immune Response | mmu05014 |  | Nefl | slateblue2 | 16 | -4.20967741935484 |
  | Akt2-mRNA | -0.592 | 0.142 | -0.869 | -0.314 | 0.664 | 0.547 | 0.804 | 0.000713 | 0.022 | loglinear | Adaptive Immune Response, Angiogenesis, Apoptosis, Autophagy, Carbohydrate Metabolism, Cytokine Signaling, Growth Factor Signaling, Innate Immune Response, Insulin Signaling, Neurons and Neurotransmission, Wnt | NS\_MM\_NEUROINFLAM\_V1.0 | Akt2 | mRNA | 0 |  | Wnt;Neurons and Neurotransmission;Insulin Signaling;Innate Immune Response;Growth Factor Signaling;Cytokine Signaling;Carbohydrate Metabolism;Autophagy;Apoptosis;Angiogenesis;Adaptive Immune Response | mmu04010;mmu04012;mmu04014;mmu04015;mmu04022;mmu04024;mmu04062;mmu04066;mmu04068;mmu04071;mmu04072;mmu04150;mmu04151;mmu04152;mmu04210;mmu04211;mmu04213;mmu04261;mmu04370;mmu04380;mmu04510;mmu04530;mmu04550;mmu04611;mmu04620;mmu04630;mmu04660;mmu04662;mmu04664;mmu04666;mmu04668;mmu04722;mmu04725;mmu04728;mmu04910;mmu04914;mmu04915;mmu04917;mmu04919;mmu04920;mmu04922;mmu04923;mmu04931;mmu04932;mmu04933;mmu04973;mmu05142;mmu05145;mmu05152;mmu05160;mmu05161;mmu05162;mmu05164;mmu05166;mmu05169;mmu05200;mmu05205;mmu05210;mmu05211;mmu05212;mmu05213;mmu05214;mmu05215;mmu05218;mmu05220;mmu05221;mmu05222;mmu05223;mmu05230;mmu05231 |  | Akt2 | slateblue2 | 16 | -4.16901408450704 |
  | Braf-mRNA | -0.643 | 0.154 | -0.945 | -0.341 | 0.64 | 0.519 | 0.789 | 0.000718 | 0.022 | lm.nb | Adaptive Immune Response, Angiogenesis, Apoptosis, Cytokine Signaling, Growth Factor Signaling, Innate Immune Response, Insulin Signaling, Neurons and Neurotransmission | NS\_MM\_NEUROINFLAM\_V1.0 | Braf | mRNA | 0 |  | Neurons and Neurotransmission;Insulin Signaling;Innate Immune Response;Growth Factor Signaling;Cytokine Signaling;Apoptosis;Angiogenesis;Adaptive Immune Response | mmu04010;mmu04012;mmu04015;mmu04024;mmu04062;mmu04068;mmu04150;mmu04270;mmu04510;mmu04650;mmu04720;mmu04722;mmu04726;mmu04730;mmu04810;mmu04910;mmu04914;mmu05034;mmu05160;mmu05200;mmu05205;mmu05210;mmu05211;mmu05212;mmu05213;mmu05214;mmu05215;mmu05216;mmu05218;mmu05219;mmu05220;mmu05221;mmu05223 |  | Braf | slateblue2 | 16 | -4.17532467532468 |
  | Rhoa-mRNA | -1.04 | 0.261 | -1.55 | -0.526 | 0.487 | 0.342 | 0.695 | 0.00109 | 0.0288 | lm.nb | Angiogenesis, Autophagy, Growth Factor Signaling, Wnt | NS\_MM\_NEUROINFLAM\_V1.0 | Rhoa | mRNA | 0 |  | Wnt;Growth Factor Signaling;Autophagy;Angiogenesis | mmu04014;mmu04015;mmu04022;mmu04024;mmu04062;mmu04071;mmu04072;mmu04144;mmu04270;mmu04310;mmu04350;mmu04360;mmu04510;mmu04520;mmu04530;mmu04611;mmu04660;mmu04670;mmu04722;mmu04810;mmu04921;mmu05100;mmu05133;mmu05152;mmu05200;mmu05203;mmu05205;mmu05206;mmu05210 |  | Rhoa | slateblue2 | 16 | -3.98467432950192 |
  | Dlg4-mRNA | -0.221 | 0.056 | -0.331 | -0.112 | 0.858 | 0.795 | 0.926 | 0.00114 | 0.0296 | loglinear | Adaptive Immune Response, Angiogenesis, Cytokine Signaling, Growth Factor Signaling, Insulin Signaling, Neurons and Neurotransmission | NS\_MM\_NEUROINFLAM\_V1.0 | Dlg4 | mRNA | 0 |  | Neurons and Neurotransmission;Insulin Signaling;Growth Factor Signaling;Cytokine Signaling;Angiogenesis;Adaptive Immune Response | mmu04390;mmu04724;mmu05016;mmu05030 |  | Dlg4 | slateblue2 | 16 | -3.94642857142857 |
  | Grin2b-mRNA | -0.395 | 0.101 | -0.592 | -0.197 | 0.761 | 0.663 | 0.872 | 0.00124 | 0.0314 | lm.nb | Adaptive Immune Response, Angiogenesis, Cytokine Signaling, Growth Factor Signaling, Insulin Signaling, Neurons and Neurotransmission | NS\_MM\_NEUROINFLAM\_V1.0 | Grin2b | mRNA | 0 |  | Neurons and Neurotransmission;Insulin Signaling;Growth Factor Signaling;Cytokine Signaling;Angiogenesis;Adaptive Immune Response | mmu04014;mmu04015;mmu04024;mmu04080;mmu04713;mmu04720;mmu04724;mmu04728;mmu05010;mmu05014;mmu05016;mmu05030;mmu05031;mmu05033;mmu05034;mmu05322 |  | Grin2b | slateblue2 | 16 | -3.91089108910891 |
  | Rac1-mRNA | -0.955 | 0.256 | -1.46 | -0.452 | 0.516 | 0.364 | 0.731 | 0.00184 | 0.0417 | lm.nb | Adaptive Immune Response, Angiogenesis, Autophagy, Growth Factor Signaling, Innate Immune Response, Neurons and Neurotransmission, Wnt | NS\_MM\_NEUROINFLAM\_V1.0 | Rac1 | mRNA | 0 |  | Wnt;Neurons and Neurotransmission;Innate Immune Response;Growth Factor Signaling;Autophagy;Angiogenesis;Adaptive Immune Response | mmu04010;mmu04014;mmu04015;mmu04024;mmu04062;mmu04071;mmu04145;mmu04151;mmu04310;mmu04360;mmu04370;mmu04380;mmu04510;mmu04520;mmu04620;mmu04650;mmu04662;mmu04664;mmu04666;mmu04670;mmu04722;mmu04810;mmu04932;mmu04933;mmu05014;mmu05100;mmu05132;mmu05200;mmu05203;mmu05205;mmu05210;mmu05211;mmu05212;mmu05231;mmu05416 |  | Rac1 | slateblue2 | 16 | -3.73046875 |
  | Vav1-mRNA | 2.33 | 0.652 | 1.06 | 3.61 | 5.04 | 2.08 | 12.2 | 0.00251 | 0.053 | lm.nb | Adaptive Immune Response, Angiogenesis, Autophagy, Cytokine Signaling, Growth Factor Signaling, Innate Immune Response | NS\_MM\_NEUROINFLAM\_V1.0 | Vav1 | mRNA | 0 |  | Innate Immune Response;Growth Factor Signaling;Cytokine Signaling;Autophagy;Angiogenesis;Adaptive Immune Response | mmu04024;mmu04062;mmu04510;mmu04650;mmu04660;mmu04662;mmu04664;mmu04666;mmu04670;mmu04810 |  | Vav1 | slateblue3 | 16 | 3.57361963190184 |
  | Map2k1-mRNA | -1.05 | 0.32 | -1.68 | -0.425 | 0.482 | 0.312 | 0.745 | 0.00463 | 0.0883 | lm.nb | Adaptive Immune Response, Angiogenesis, Autophagy, Cytokine Signaling, Growth Factor Signaling, Innate Immune Response, Insulin Signaling, Microglia Function | NS\_MM\_NEUROINFLAM\_V1.0 | Map2k1 | mRNA | 0 |  | Microglia Function;Insulin Signaling;Innate Immune Response;Growth Factor Signaling;Cytokine Signaling;Autophagy;Angiogenesis;Adaptive Immune Response | mmu04010;mmu04012;mmu04014;mmu04015;mmu04022;mmu04024;mmu04062;mmu04066;mmu04068;mmu04071;mmu04072;mmu04114;mmu04151;mmu04270;mmu04370;mmu04380;mmu04510;mmu04540;mmu04550;mmu04620;mmu04650;mmu04660;mmu04662;mmu04664;mmu04666;mmu04668;mmu04720;mmu04722;mmu04725;mmu04726;mmu04730;mmu04810;mmu04910;mmu04912;mmu04914;mmu04915;mmu04916;mmu04917;mmu04919;mmu04921;mmu05020;mmu05034;mmu05161;mmu05164;mmu05200;mmu05205;mmu05206;mmu05210;mmu05211;mmu05212;mmu05213;mmu05214;mmu05215;mmu05216;mmu05218;mmu05219;mmu05220;mmu05221;mmu05223;mmu05230;mmu05231 |  | Map2k1 | slateblue3 | 16 | -3.28125 |
  | Pik3cb-mRNA | -0.376 | 0.116 | -0.604 | -0.148 | 0.771 | 0.658 | 0.902 | 0.0052 | 0.0976 | loglinear | Adaptive Immune Response, Angiogenesis, Apoptosis, Autophagy, Carbohydrate Metabolism, Cytokine Signaling, Growth Factor Signaling, Innate Immune Response, Insulin Signaling, Lipid Metabolism | NS\_MM\_NEUROINFLAM\_V1.0 | Pik3cb | mRNA | 0 |  | Lipid Metabolism;Insulin Signaling;Innate Immune Response;Growth Factor Signaling;Cytokine Signaling;Carbohydrate Metabolism;Autophagy;Apoptosis;Angiogenesis;Adaptive Immune Response | mmu00562;mmu04012;mmu04014;mmu04015;mmu04022;mmu04024;mmu04062;mmu04066;mmu04068;mmu04070;mmu04071;mmu04072;mmu04150;mmu04151;mmu04152;mmu04210;mmu04211;mmu04213;mmu04261;mmu04370;mmu04380;mmu04510;mmu04550;mmu04611;mmu04620;mmu04630;mmu04650;mmu04660;mmu04662;mmu04664;mmu04666;mmu04668;mmu04670;mmu04722;mmu04725;mmu04750;mmu04810;mmu04910;mmu04914;mmu04915;mmu04917;mmu04919;mmu04921;mmu04923;mmu04930;mmu04931;mmu04932;mmu04933;mmu04960;mmu04973;mmu05100;mmu05142;mmu05145;mmu05146;mmu05160;mmu05161;mmu05162;mmu05164;mmu05166;mmu05169;mmu05200;mmu05203;mmu05205;mmu05210;mmu05211;mmu05212;mmu05213;mmu05214;mmu05215;mmu05218;mmu05220;mmu05221;mmu05222;mmu05223;mmu05230;mmu05231 |  | Pik3cb | slateblue4 | 16 | -3.24137931034483 |
  | Hspb1-mRNA | 0.891 | 0.295 | 0.314 | 1.47 | 1.85 | 1.24 | 2.77 | 0.00806 | 0.137 | lm.nb | Angiogenesis, Astrocyte Function, Cellular Stress, Growth Factor Signaling | NS\_MM\_NEUROINFLAM\_V1.0 | Hspb1 | mRNA | 0 |  | Growth Factor Signaling;Cellular Stress;Astrocyte Function;Angiogenesis | mmu04010;mmu04370;mmu05146;mmu05169 |  | Hspb1 | slateblue4 | 16 | 3.02033898305085 |
  | Prkaca-mRNA | -0.166 | 0.0577 | -0.279 | -0.0525 | 0.892 | 0.824 | 0.964 | 0.0111 | 0.179 | loglinear | Adaptive Immune Response, Angiogenesis, Apoptosis, Autophagy, Cell Cycle, Cytokine Signaling, Growth Factor Signaling, Lipid Metabolism, Neurons and Neurotransmission, Wnt | NS\_MM\_NEUROINFLAM\_V1.0 | Prkaca | mRNA | 0 |  | Wnt;Neurons and Neurotransmission;Lipid Metabolism;Growth Factor Signaling;Cytokine Signaling;Cell Cycle;Autophagy;Apoptosis;Angiogenesis;Adaptive Immune Response | mmu04010;mmu04014;mmu04020;mmu04024;mmu04062;mmu04114;mmu04210;mmu04211;mmu04213;mmu04261;mmu04270;mmu04310;mmu04540;mmu04611;mmu04713;mmu04720;mmu04723;mmu04724;mmu04725;mmu04726;mmu04728;mmu04750;mmu04910;mmu04911;mmu04912;mmu04913;mmu04914;mmu04915;mmu04916;mmu04919;mmu04921;mmu04922;mmu04923;mmu04924;mmu04925;mmu05012;mmu05020;mmu05030;mmu05031;mmu05034;mmu05146;mmu05166;mmu05169;mmu05200;mmu05203;mmu05205;mmu05414 |  | Prkaca | slateblue4 | 16 | -2.87694974003466 |
  | Mapk14-mRNA | -1.02 | 0.362 | -1.73 | -0.312 | 0.493 | 0.301 | 0.806 | 0.0123 | 0.193 | lm.nb | Angiogenesis, Autophagy, Cellular Stress, Growth Factor Signaling, Innate Immune Response | NS\_MM\_NEUROINFLAM\_V1.0 | Mapk14 | mRNA | 0 |  | Innate Immune Response;Growth Factor Signaling;Cellular Stress;Autophagy;Angiogenesis | mmu04010;mmu04015;mmu04068;mmu04071;mmu04261;mmu04370;mmu04380;mmu04550;mmu04611;mmu04620;mmu04621;mmu04622;mmu04660;mmu04664;mmu04668;mmu04670;mmu04722;mmu04723;mmu04728;mmu04750;mmu04912;mmu04914;mmu04917;mmu04933;mmu05014;mmu05132;mmu05133;mmu05140;mmu05142;mmu05145;mmu05152;mmu05160;mmu05164;mmu05169;mmu05205 |  | Mapk14 | slateblue4 | 16 | -2.81767955801105 |
  | Pik3ca-mRNA | -0.277 | 0.107 | -0.487 | -0.0672 | 0.825 | 0.714 | 0.954 | 0.0198 | 0.276 | lm.nb | Adaptive Immune Response, Angiogenesis, Apoptosis, Autophagy, Carbohydrate Metabolism, Cytokine Signaling, Growth Factor Signaling, Innate Immune Response, Insulin Signaling, Lipid Metabolism | NS\_MM\_NEUROINFLAM\_V1.0 | Pik3ca | mRNA | 0 |  | Lipid Metabolism;Insulin Signaling;Innate Immune Response;Growth Factor Signaling;Cytokine Signaling;Carbohydrate Metabolism;Autophagy;Apoptosis;Angiogenesis;Adaptive Immune Response | mmu00562;mmu04012;mmu04014;mmu04015;mmu04022;mmu04024;mmu04062;mmu04066;mmu04068;mmu04070;mmu04071;mmu04072;mmu04150;mmu04151;mmu04152;mmu04210;mmu04211;mmu04213;mmu04261;mmu04370;mmu04380;mmu04510;mmu04550;mmu04611;mmu04620;mmu04630;mmu04650;mmu04660;mmu04662;mmu04664;mmu04666;mmu04668;mmu04670;mmu04722;mmu04725;mmu04750;mmu04810;mmu04910;mmu04914;mmu04915;mmu04917;mmu04919;mmu04921;mmu04923;mmu04930;mmu04931;mmu04932;mmu04933;mmu04960;mmu04973;mmu05100;mmu05142;mmu05145;mmu05146;mmu05160;mmu05161;mmu05162;mmu05164;mmu05166;mmu05169;mmu05200;mmu05203;mmu05205;mmu05210;mmu05211;mmu05212;mmu05213;mmu05214;mmu05215;mmu05218;mmu05220;mmu05221;mmu05222;mmu05223;mmu05230;mmu05231 |  | Pik3ca | slateblue4 | 16 | -2.58878504672897 |

  ##### DE Results - Angiogenesis genes - DPI: 2 vs.CTRL

  Table displaying each sample's global significance scores and directed global significance scores as defined in the heatmaps above. The global significance score is calculated as the square root of the mean squared t-statistic for the genes in a gene set, with t-statistics coming from the linear regression underlying our differential expression analysis. The directed global significance score is calculated as the square root of the mean signed squared t-statistic for the genes in a gene set, with t-statistics coming from the linear regression underlying our differential expression analysis.

- **Volcano Plot: DPI: 3 vs.CTRL  
  More Plot Information**


  ##### Volcano Plot: DPI: 3 vs.CTRL

  Volcano plot displaying each gene's -log10(p-value) and log2 fold change for the selected covariate. Highly statistically significant genes fall at the top of the plot, and highly differentially expressed genes fall to either side. Genes within the selected gene set are highlighted in orange. Horizontal lines indicate various False Discovery Rate (FDR) thresholds.
- **DE Results - Angiogenesis genes - DPI: 3 vs.CTRL  
  More Plot Information  Download CSV Data**

  | Probe Label | Log2 fold change | std error (log2) | Lower confidence limit (log2) | Upper confidence limit (log2) | Linear fold change | Lower confidence limit (linear) | Upper confidence limit (linear) | P-value | BY.p.value | method | Gene.sets | Codeset.Name | Probe.Label | Analyte.Type | Is.Control | Control.Type | Probe.Annotation | KEGG.Pathways | Cell.Type | Official.Gene.Name | volcanocol | volcanopch | tstats.all |
  | --- | --- | --- | --- | --- | --- | --- | --- | --- | --- | --- | --- | --- | --- | --- | --- | --- | --- | --- | --- | --- | --- | --- | --- |
  | Psmb8-mRNA | 2.99 | 0.346 | 2.31 | 3.67 | 7.95 | 4.97 | 12.7 | 1.99e-07 | 3.2e-05 | lm.nb | Adaptive Immune Response, Angiogenesis, Apoptosis, Astrocyte Function, Cell Cycle, Cytokine Signaling, Growth Factor Signaling, Inflammatory Signaling, Insulin Signaling, Microglia Function, NF-kB, Wnt | NS\_MM\_NEUROINFLAM\_V1.0 | Psmb8 | mRNA | 0 |  | Wnt;NF-kB;Microglia Function;Insulin Signaling;Inflammatory Signaling;Growth Factor Signaling;Cytokine Signaling;Cell Cycle;Astrocyte Function;Apoptosis;Angiogenesis;Adaptive Immune Response |  |  | Psmb8 | slateblue1 | 16 | 8.64161849710983 |
  | Dock1-mRNA | -1.14 | 0.186 | -1.5 | -0.774 | 0.454 | 0.353 | 0.585 | 1.49e-05 | 0.000921 | loglinear | Angiogenesis, Autophagy | NS\_MM\_NEUROINFLAM\_V1.0 | Dock1 | mRNA | 0 |  | Autophagy;Angiogenesis | mmu04510;mmu04810;mmu05100 |  | Dock1 | slateblue1 | 16 | -6.12903225806452 |
  | Akt2-mRNA | -0.766 | 0.142 | -1.04 | -0.488 | 0.588 | 0.485 | 0.713 | 5.85e-05 | 0.00265 | loglinear | Adaptive Immune Response, Angiogenesis, Apoptosis, Autophagy, Carbohydrate Metabolism, Cytokine Signaling, Growth Factor Signaling, Innate Immune Response, Insulin Signaling, Neurons and Neurotransmission, Wnt | NS\_MM\_NEUROINFLAM\_V1.0 | Akt2 | mRNA | 0 |  | Wnt;Neurons and Neurotransmission;Insulin Signaling;Innate Immune Response;Growth Factor Signaling;Cytokine Signaling;Carbohydrate Metabolism;Autophagy;Apoptosis;Angiogenesis;Adaptive Immune Response | mmu04010;mmu04012;mmu04014;mmu04015;mmu04022;mmu04024;mmu04062;mmu04066;mmu04068;mmu04071;mmu04072;mmu04150;mmu04151;mmu04152;mmu04210;mmu04211;mmu04213;mmu04261;mmu04370;mmu04380;mmu04510;mmu04530;mmu04550;mmu04611;mmu04620;mmu04630;mmu04660;mmu04662;mmu04664;mmu04666;mmu04668;mmu04722;mmu04725;mmu04728;mmu04910;mmu04914;mmu04915;mmu04917;mmu04919;mmu04920;mmu04922;mmu04923;mmu04931;mmu04932;mmu04933;mmu04973;mmu05142;mmu05145;mmu05152;mmu05160;mmu05161;mmu05162;mmu05164;mmu05166;mmu05169;mmu05200;mmu05205;mmu05210;mmu05211;mmu05212;mmu05213;mmu05214;mmu05215;mmu05218;mmu05220;mmu05221;mmu05222;mmu05223;mmu05230;mmu05231 |  | Akt2 | slateblue1 | 16 | -5.3943661971831 |
  | Hspb1-mRNA | 1.36 | 0.29 | 0.795 | 1.93 | 2.57 | 1.73 | 3.82 | 0.000241 | 0.00894 | lm.nb | Angiogenesis, Astrocyte Function, Cellular Stress, Growth Factor Signaling | NS\_MM\_NEUROINFLAM\_V1.0 | Hspb1 | mRNA | 0 |  | Growth Factor Signaling;Cellular Stress;Astrocyte Function;Angiogenesis | mmu04010;mmu04370;mmu05146;mmu05169 |  | Hspb1 | slateblue1 | 16 | 4.68965517241379 |
  | Pak1-mRNA | -0.206 | 0.0554 | -0.314 | -0.0974 | 0.867 | 0.804 | 0.935 | 0.00187 | 0.046 | loglinear | Adaptive Immune Response, Angiogenesis, Growth Factor Signaling, Innate Immune Response, Microglia Function | NS\_MM\_NEUROINFLAM\_V1.0 | Pak1 | mRNA | 0 |  | Microglia Function;Innate Immune Response;Growth Factor Signaling;Angiogenesis;Adaptive Immune Response | mmu04010;mmu04012;mmu04014;mmu04024;mmu04062;mmu04360;mmu04510;mmu04650;mmu04660;mmu04666;mmu04810;mmu05205;mmu05211 |  | Pak1 | slateblue3 | 16 | -3.71841155234657 |
  | Dlg4-mRNA | -0.204 | 0.056 | -0.313 | -0.0938 | 0.868 | 0.805 | 0.937 | 0.00223 | 0.0534 | loglinear | Adaptive Immune Response, Angiogenesis, Cytokine Signaling, Growth Factor Signaling, Insulin Signaling, Neurons and Neurotransmission | NS\_MM\_NEUROINFLAM\_V1.0 | Dlg4 | mRNA | 0 |  | Neurons and Neurotransmission;Insulin Signaling;Growth Factor Signaling;Cytokine Signaling;Angiogenesis;Adaptive Immune Response | mmu04390;mmu04724;mmu05016;mmu05030 |  | Dlg4 | slateblue3 | 16 | -3.64285714285714 |
  | Csk-mRNA | -0.423 | 0.12 | -0.658 | -0.188 | 0.746 | 0.634 | 0.878 | 0.00282 | 0.063 | lm.nb | Adaptive Immune Response, Angiogenesis, Autophagy, Growth Factor Signaling, Insulin Signaling | NS\_MM\_NEUROINFLAM\_V1.0 | Csk | mRNA | 0 |  | Insulin Signaling;Growth Factor Signaling;Autophagy;Angiogenesis;Adaptive Immune Response |  |  | Csk | slateblue3 | 16 | -3.525 |
  | Vegfa-mRNA | -0.411 | 0.118 | -0.642 | -0.181 | 0.752 | 0.641 | 0.882 | 0.00299 | 0.0656 | loglinear | Angiogenesis, Cellular Stress, Cytokine Signaling, Growth Factor Signaling, Microglia Function | NS\_MM\_NEUROINFLAM\_V1.0 | Vegfa | mRNA | 0 |  | Microglia Function;Growth Factor Signaling;Cytokine Signaling;Cellular Stress;Angiogenesis | mmu04014;mmu04015;mmu04060;mmu04066;mmu04150;mmu04151;mmu04370;mmu04510;mmu04933;mmu05200;mmu05205;mmu05206;mmu05211;mmu05212;mmu05219;mmu05323 |  | Vegfa | slateblue3 | 16 | -3.48305084745763 |
  | Nefl-mRNA | -0.411 | 0.123 | -0.653 | -0.169 | 0.752 | 0.636 | 0.889 | 0.00423 | 0.0899 | lm.nb | Adaptive Immune Response, Angiogenesis, Cytokine Signaling, Growth Factor Signaling, Insulin Signaling, Neurons and Neurotransmission | NS\_MM\_NEUROINFLAM\_V1.0 | Nefl | mRNA | 0 |  | Neurons and Neurotransmission;Insulin Signaling;Growth Factor Signaling;Cytokine Signaling;Angiogenesis;Adaptive Immune Response | mmu05014 |  | Nefl | slateblue3 | 16 | -3.34146341463415 |
  | Pik3ca-mRNA | -0.344 | 0.107 | -0.554 | -0.134 | 0.788 | 0.681 | 0.911 | 0.00549 | 0.111 | lm.nb | Adaptive Immune Response, Angiogenesis, Apoptosis, Autophagy, Carbohydrate Metabolism, Cytokine Signaling, Growth Factor Signaling, Innate Immune Response, Insulin Signaling, Lipid Metabolism | NS\_MM\_NEUROINFLAM\_V1.0 | Pik3ca | mRNA | 0 |  | Lipid Metabolism;Insulin Signaling;Innate Immune Response;Growth Factor Signaling;Cytokine Signaling;Carbohydrate Metabolism;Autophagy;Apoptosis;Angiogenesis;Adaptive Immune Response | mmu00562;mmu04012;mmu04014;mmu04015;mmu04022;mmu04024;mmu04062;mmu04066;mmu04068;mmu04070;mmu04071;mmu04072;mmu04150;mmu04151;mmu04152;mmu04210;mmu04211;mmu04213;mmu04261;mmu04370;mmu04380;mmu04510;mmu04550;mmu04611;mmu04620;mmu04630;mmu04650;mmu04660;mmu04662;mmu04664;mmu04666;mmu04668;mmu04670;mmu04722;mmu04725;mmu04750;mmu04810;mmu04910;mmu04914;mmu04915;mmu04917;mmu04919;mmu04921;mmu04923;mmu04930;mmu04931;mmu04932;mmu04933;mmu04960;mmu04973;mmu05100;mmu05142;mmu05145;mmu05146;mmu05160;mmu05161;mmu05162;mmu05164;mmu05166;mmu05169;mmu05200;mmu05203;mmu05205;mmu05210;mmu05211;mmu05212;mmu05213;mmu05214;mmu05215;mmu05218;mmu05220;mmu05221;mmu05222;mmu05223;mmu05230;mmu05231 |  | Pik3ca | slateblue4 | 16 | -3.21495327102804 |
  | Grin2b-mRNA | -0.278 | 0.101 | -0.476 | -0.0811 | 0.825 | 0.719 | 0.945 | 0.0138 | 0.237 | lm.nb | Adaptive Immune Response, Angiogenesis, Cytokine Signaling, Growth Factor Signaling, Insulin Signaling, Neurons and Neurotransmission | NS\_MM\_NEUROINFLAM\_V1.0 | Grin2b | mRNA | 0 |  | Neurons and Neurotransmission;Insulin Signaling;Growth Factor Signaling;Cytokine Signaling;Angiogenesis;Adaptive Immune Response | mmu04014;mmu04015;mmu04024;mmu04080;mmu04713;mmu04720;mmu04724;mmu04728;mmu05010;mmu05014;mmu05016;mmu05030;mmu05031;mmu05033;mmu05034;mmu05322 |  | Grin2b | slateblue4 | 16 | -2.75247524752475 |
  | Nrp2-mRNA | -0.371 | 0.136 | -0.638 | -0.105 | 0.773 | 0.643 | 0.93 | 0.0149 | 0.254 | lm.nb | Angiogenesis, Microglia Function | NS\_MM\_NEUROINFLAM\_V1.0 | Nrp2 | mRNA | 0 |  | Microglia Function;Angiogenesis |  |  | Nrp2 | slateblue4 | 16 | -2.72794117647059 |
  | Dusp7-mRNA | -0.161 | 0.0611 | -0.281 | -0.0413 | 0.894 | 0.823 | 0.972 | 0.018 | 0.294 | loglinear | Adaptive Immune Response, Angiogenesis, Cytokine Signaling, Growth Factor Signaling, Innate Immune Response, Insulin Signaling, Microglia Function | NS\_MM\_NEUROINFLAM\_V1.0 | Dusp7 | mRNA | 0 |  | Microglia Function;Insulin Signaling;Innate Immune Response;Growth Factor Signaling;Cytokine Signaling;Angiogenesis;Adaptive Immune Response | mmu04010 |  | Dusp7 | slateblue4 | 16 | -2.63502454991817 |
  | Grin2a-mRNA | 0.253 | 0.0975 | 0.0617 | 0.444 | 1.19 | 1.04 | 1.36 | 0.0196 | 0.314 | loglinear | Adaptive Immune Response, Angiogenesis, Cytokine Signaling, Growth Factor Signaling, Insulin Signaling, Neurons and Neurotransmission | NS\_MM\_NEUROINFLAM\_V1.0 | Grin2a | mRNA | 0 |  | Neurons and Neurotransmission;Insulin Signaling;Growth Factor Signaling;Cytokine Signaling;Angiogenesis;Adaptive Immune Response | mmu04014;mmu04015;mmu04020;mmu04024;mmu04080;mmu04713;mmu04720;mmu04724;mmu04728;mmu05010;mmu05014;mmu05030;mmu05031;mmu05033;mmu05034;mmu05322 |  | Grin2a | slateblue4 | 16 | 2.59487179487179 |
  | Prkaca-mRNA | -0.149 | 0.0577 | -0.262 | -0.0355 | 0.902 | 0.834 | 0.976 | 0.0203 | 0.322 | loglinear | Adaptive Immune Response, Angiogenesis, Apoptosis, Autophagy, Cell Cycle, Cytokine Signaling, Growth Factor Signaling, Lipid Metabolism, Neurons and Neurotransmission, Wnt | NS\_MM\_NEUROINFLAM\_V1.0 | Prkaca | mRNA | 0 |  | Wnt;Neurons and Neurotransmission;Lipid Metabolism;Growth Factor Signaling;Cytokine Signaling;Cell Cycle;Autophagy;Apoptosis;Angiogenesis;Adaptive Immune Response | mmu04010;mmu04014;mmu04020;mmu04024;mmu04062;mmu04114;mmu04210;mmu04211;mmu04213;mmu04261;mmu04270;mmu04310;mmu04540;mmu04611;mmu04713;mmu04720;mmu04723;mmu04724;mmu04725;mmu04726;mmu04728;mmu04750;mmu04910;mmu04911;mmu04912;mmu04913;mmu04914;mmu04915;mmu04916;mmu04919;mmu04921;mmu04922;mmu04923;mmu04924;mmu04925;mmu05012;mmu05020;mmu05030;mmu05031;mmu05034;mmu05146;mmu05166;mmu05169;mmu05200;mmu05203;mmu05205;mmu05414 |  | Prkaca | slateblue4 | 16 | -2.58232235701906 |
  | Pik3r2-mRNA | -0.181 | 0.0793 | -0.337 | -0.0258 | 0.882 | 0.792 | 0.982 | 0.0363 | 0.503 | loglinear | Adaptive Immune Response, Angiogenesis, Apoptosis, Autophagy, Cytokine Signaling, Growth Factor Signaling, Innate Immune Response, Insulin Signaling | NS\_MM\_NEUROINFLAM\_V1.0 | Pik3r2 | mRNA | 0 |  | Insulin Signaling;Innate Immune Response;Growth Factor Signaling;Cytokine Signaling;Autophagy;Apoptosis;Angiogenesis;Adaptive Immune Response | mmu04012;mmu04014;mmu04015;mmu04022;mmu04024;mmu04062;mmu04066;mmu04068;mmu04070;mmu04071;mmu04072;mmu04150;mmu04151;mmu04152;mmu04210;mmu04211;mmu04213;mmu04261;mmu04370;mmu04380;mmu04510;mmu04550;mmu04611;mmu04620;mmu04630;mmu04650;mmu04660;mmu04662;mmu04664;mmu04666;mmu04668;mmu04670;mmu04722;mmu04725;mmu04750;mmu04810;mmu04910;mmu04914;mmu04915;mmu04917;mmu04919;mmu04921;mmu04923;mmu04930;mmu04931;mmu04932;mmu04933;mmu04960;mmu04973;mmu05100;mmu05142;mmu05145;mmu05146;mmu05160;mmu05161;mmu05162;mmu05164;mmu05166;mmu05169;mmu05200;mmu05203;mmu05205;mmu05210;mmu05211;mmu05212;mmu05213;mmu05214;mmu05215;mmu05218;mmu05220;mmu05221;mmu05222;mmu05223;mmu05230;mmu05231 |  | Pik3r2 | azure4 | 1 | -2.28247162673392 |
  | Fyn-mRNA | -0.123 | 0.0637 | -0.248 | 0.002 | 0.918 | 0.842 | 1 | 0.0717 | 0.896 | loglinear | Adaptive Immune Response, Angiogenesis, Autophagy, Cytokine Signaling, Growth Factor Signaling, Innate Immune Response, Insulin Signaling, Microglia Function | NS\_MM\_NEUROINFLAM\_V1.0 | Fyn | mRNA | 0 |  | Microglia Function;Insulin Signaling;Innate Immune Response;Growth Factor Signaling;Cytokine Signaling;Autophagy;Angiogenesis;Adaptive Immune Response | mmu04071;mmu04072;mmu04360;mmu04380;mmu04510;mmu04520;mmu04611;mmu04650;mmu04660;mmu04664;mmu04725;mmu05020;mmu05162;mmu05416 |  | Fyn | azure4 | 1 | -1.9309262166405 |
  | Vav1-mRNA | 1.27 | 0.664 | -0.0348 | 2.57 | 2.41 | 0.976 | 5.94 | 0.0745 | 0.919 | lm.nb | Adaptive Immune Response, Angiogenesis, Autophagy, Cytokine Signaling, Growth Factor Signaling, Innate Immune Response | NS\_MM\_NEUROINFLAM\_V1.0 | Vav1 | mRNA | 0 |  | Innate Immune Response;Growth Factor Signaling;Cytokine Signaling;Autophagy;Angiogenesis;Adaptive Immune Response | mmu04024;mmu04062;mmu04510;mmu04650;mmu04660;mmu04662;mmu04664;mmu04666;mmu04670;mmu04810 |  | Vav1 | azure4 | 1 | 1.91265060240964 |
  | Pik3cb-mRNA | -0.207 | 0.116 | -0.435 | 0.0205 | 0.866 | 0.74 | 1.01 | 0.0934 | 1 | loglinear | Adaptive Immune Response, Angiogenesis, Apoptosis, Autophagy, Carbohydrate Metabolism, Cytokine Signaling, Growth Factor Signaling, Innate Immune Response, Insulin Signaling, Lipid Metabolism | NS\_MM\_NEUROINFLAM\_V1.0 | Pik3cb | mRNA | 0 |  | Lipid Metabolism;Insulin Signaling;Innate Immune Response;Growth Factor Signaling;Cytokine Signaling;Carbohydrate Metabolism;Autophagy;Apoptosis;Angiogenesis;Adaptive Immune Response | mmu00562;mmu04012;mmu04014;mmu04015;mmu04022;mmu04024;mmu04062;mmu04066;mmu04068;mmu04070;mmu04071;mmu04072;mmu04150;mmu04151;mmu04152;mmu04210;mmu04211;mmu04213;mmu04261;mmu04370;mmu04380;mmu04510;mmu04550;mmu04611;mmu04620;mmu04630;mmu04650;mmu04660;mmu04662;mmu04664;mmu04666;mmu04668;mmu04670;mmu04722;mmu04725;mmu04750;mmu04810;mmu04910;mmu04914;mmu04915;mmu04917;mmu04919;mmu04921;mmu04923;mmu04930;mmu04931;mmu04932;mmu04933;mmu04960;mmu04973;mmu05100;mmu05142;mmu05145;mmu05146;mmu05160;mmu05161;mmu05162;mmu05164;mmu05166;mmu05169;mmu05200;mmu05203;mmu05205;mmu05210;mmu05211;mmu05212;mmu05213;mmu05214;mmu05215;mmu05218;mmu05220;mmu05221;mmu05222;mmu05223;mmu05230;mmu05231 |  | Pik3cb | azure4 | 1 | -1.78448275862069 |
  | Mapk14-mRNA | -0.632 | 0.361 | -1.34 | 0.076 | 0.645 | 0.395 | 1.05 | 0.0994 | 1 | lm.nb | Angiogenesis, Autophagy, Cellular Stress, Growth Factor Signaling, Innate Immune Response | NS\_MM\_NEUROINFLAM\_V1.0 | Mapk14 | mRNA | 0 |  | Innate Immune Response;Growth Factor Signaling;Cellular Stress;Autophagy;Angiogenesis | mmu04010;mmu04015;mmu04068;mmu04071;mmu04261;mmu04370;mmu04380;mmu04550;mmu04611;mmu04620;mmu04621;mmu04622;mmu04660;mmu04664;mmu04668;mmu04670;mmu04722;mmu04723;mmu04728;mmu04750;mmu04912;mmu04914;mmu04917;mmu04933;mmu05014;mmu05132;mmu05133;mmu05140;mmu05142;mmu05145;mmu05152;mmu05160;mmu05164;mmu05169;mmu05205 |  | Mapk14 | azure4 | 1 | -1.75069252077562 |

  ##### DE Results - Angiogenesis genes - DPI: 3 vs.CTRL

  Table displaying each sample's global significance scores and directed global significance scores as defined in the heatmaps above. The global significance score is calculated as the square root of the mean squared t-statistic for the genes in a gene set, with t-statistics coming from the linear regression underlying our differential expression analysis. The directed global significance score is calculated as the square root of the mean signed squared t-statistic for the genes in a gene set, with t-statistics coming from the linear regression underlying our differential expression analysis.

DPI:
- DPI: differential expression in 1 vs. baseline of CTRL
- DPI: differential expression in 2 vs. baseline of CTRL
- DPI: differential expression in 3 vs. baseline of CTRL

- **Volcano Plot: DPI: 1 vs.CTRL  
  More Plot Information**


  ##### Volcano Plot: DPI: 1 vs.CTRL

  Volcano plot displaying each gene's -log10(p-value) and log2 fold change for the selected covariate. Highly statistically significant genes fall at the top of the plot, and highly differentially expressed genes fall to either side. Genes within the selected gene set are highlighted in orange. Horizontal lines indicate various False Discovery Rate (FDR) thresholds.
- **DE Results - Apoptosis genes - DPI: 1 vs.CTRL  
  More Plot Information  Download CSV Data**

  | Probe Label | Log2 fold change | std error (log2) | Lower confidence limit (log2) | Upper confidence limit (log2) | Linear fold change | Lower confidence limit (linear) | Upper confidence limit (linear) | P-value | BY.p.value | method | Gene.sets | Codeset.Name | Probe.Label | Analyte.Type | Is.Control | Control.Type | Probe.Annotation | KEGG.Pathways | Cell.Type | Official.Gene.Name | volcanocol | volcanopch | tstats.all |
  | --- | --- | --- | --- | --- | --- | --- | --- | --- | --- | --- | --- | --- | --- | --- | --- | --- | --- | --- | --- | --- | --- | --- | --- |
  | Mapt-mRNA | -0.222 | 0.0425 | -0.306 | -0.139 | 0.857 | 0.809 | 0.908 | 8.22e-05 | 0.0321 | loglinear | Apoptosis, Cellular Stress, Growth Factor Signaling, Neurons and Neurotransmission | NS\_MM\_NEUROINFLAM\_V1.0 | Mapt | mRNA | 0 |  | Neurons and Neurotransmission;Growth Factor Signaling;Cellular Stress;Apoptosis | mmu04010;mmu05010 |  | Mapt | slateblue2 | 16 | -5.22352941176471 |
  | Prkacb-mRNA | -0.327 | 0.0629 | -0.451 | -0.204 | 0.797 | 0.732 | 0.868 | 8.65e-05 | 0.0321 | loglinear | Adaptive Immune Response, Angiogenesis, Apoptosis, Autophagy, Growth Factor Signaling, Lipid Metabolism, Neurons and Neurotransmission, Wnt | NS\_MM\_NEUROINFLAM\_V1.0 | Prkacb | mRNA | 0 |  | Wnt;Neurons and Neurotransmission;Lipid Metabolism;Growth Factor Signaling;Autophagy;Apoptosis;Angiogenesis;Adaptive Immune Response | mmu04010;mmu04014;mmu04020;mmu04024;mmu04062;mmu04114;mmu04210;mmu04211;mmu04213;mmu04261;mmu04270;mmu04310;mmu04540;mmu04611;mmu04713;mmu04720;mmu04723;mmu04724;mmu04725;mmu04726;mmu04728;mmu04750;mmu04910;mmu04911;mmu04912;mmu04913;mmu04914;mmu04915;mmu04916;mmu04919;mmu04921;mmu04922;mmu04923;mmu04924;mmu04925;mmu05012;mmu05020;mmu05030;mmu05031;mmu05146;mmu05166;mmu05169;mmu05200;mmu05203;mmu05205;mmu05414 |  | Prkacb | slateblue2 | 16 | -5.19872813990461 |
  | Mdm2-mRNA | -0.317 | 0.0642 | -0.443 | -0.191 | 0.803 | 0.736 | 0.876 | 0.00015 | 0.0406 | loglinear | Adaptive Immune Response, Apoptosis, Cell Cycle, Cellular Stress, DNA Damage, Growth Factor Signaling, Neurons and Neurotransmission | NS\_MM\_NEUROINFLAM\_V1.0 | Mdm2 | mRNA | 0 |  | Neurons and Neurotransmission;Growth Factor Signaling;DNA Damage;Cellular Stress;Cell Cycle;Apoptosis;Adaptive Immune Response | mmu04068;mmu04110;mmu04115;mmu04120;mmu04144;mmu04151;mmu04919;mmu05169;mmu05200;mmu05202;mmu05203;mmu05205;mmu05206;mmu05214;mmu05215;mmu05218;mmu05219;mmu05220 |  | Mdm2 | slateblue2 | 16 | -4.93769470404984 |
  | Ppp3r1-mRNA | -0.28 | 0.0577 | -0.393 | -0.167 | 0.823 | 0.761 | 0.891 | 0.000175 | 0.0442 | loglinear | Adaptive Immune Response, Apoptosis, Growth Factor Signaling, Innate Immune Response, Wnt | NS\_MM\_NEUROINFLAM\_V1.0 | Ppp3r1 | mRNA | 0 |  | Wnt;Innate Immune Response;Growth Factor Signaling;Apoptosis;Adaptive Immune Response | mmu04010;mmu04020;mmu04022;mmu04114;mmu04210;mmu04310;mmu04360;mmu04370;mmu04380;mmu04650;mmu04660;mmu04662;mmu04720;mmu04724;mmu04921;mmu04922;mmu04924;mmu05010;mmu05014;mmu05031;mmu05152;mmu05166 |  | Ppp3r1 | slateblue2 | 16 | -4.8526863084922 |
  | Parp1-mRNA | -0.49 | 0.108 | -0.703 | -0.278 | 0.712 | 0.614 | 0.825 | 0.000348 | 0.0564 | loglinear | Apoptosis, Cellular Stress, DNA Damage, NF-kB | NS\_MM\_NEUROINFLAM\_V1.0 | Parp1 | mRNA | 0 |  | NF-kB;DNA Damage;Cellular Stress;Apoptosis | mmu03410;mmu04064 |  | Parp1 | slateblue3 | 16 | -4.53703703703704 |
  | Prkar2b-mRNA | -0.5 | 0.113 | -0.722 | -0.278 | 0.707 | 0.606 | 0.825 | 0.000436 | 0.0616 | loglinear | Apoptosis, Cell Cycle, Growth Factor Signaling | NS\_MM\_NEUROINFLAM\_V1.0 | Prkar2b | mRNA | 0 |  | Growth Factor Signaling;Cell Cycle;Apoptosis | mmu04210;mmu04910 |  | Prkar2b | slateblue3 | 16 | -4.42477876106195 |
  | Hells-mRNA | -1.96 | 0.463 | -2.86 | -1.05 | 0.258 | 0.137 | 0.483 | 0.000639 | 0.0784 | lm.nb | Apoptosis | NS\_MM\_NEUROINFLAM\_V1.0 | Hells | mRNA | 0 |  | Apoptosis |  |  | Hells | slateblue3 | 16 | -4.23326133909287 |
  | Ppp3cb-mRNA | -0.411 | 0.0979 | -0.602 | -0.219 | 0.752 | 0.659 | 0.859 | 0.000686 | 0.0806 | lm.nb | Adaptive Immune Response, Apoptosis, Growth Factor Signaling, Innate Immune Response, Wnt | NS\_MM\_NEUROINFLAM\_V1.0 | Ppp3cb | mRNA | 0 |  | Wnt;Innate Immune Response;Growth Factor Signaling;Apoptosis;Adaptive Immune Response | mmu04010;mmu04020;mmu04022;mmu04114;mmu04210;mmu04310;mmu04360;mmu04370;mmu04380;mmu04650;mmu04660;mmu04662;mmu04720;mmu04724;mmu04728;mmu04921;mmu04922;mmu04924;mmu05010;mmu05014;mmu05031;mmu05152;mmu05166 |  | Ppp3cb | slateblue3 | 16 | -4.19816138917262 |
  | Braf-mRNA | -0.641 | 0.154 | -0.943 | -0.339 | 0.641 | 0.52 | 0.791 | 0.000739 | 0.0815 | lm.nb | Adaptive Immune Response, Angiogenesis, Apoptosis, Cytokine Signaling, Growth Factor Signaling, Innate Immune Response, Insulin Signaling, Neurons and Neurotransmission | NS\_MM\_NEUROINFLAM\_V1.0 | Braf | mRNA | 0 |  | Neurons and Neurotransmission;Insulin Signaling;Innate Immune Response;Growth Factor Signaling;Cytokine Signaling;Apoptosis;Angiogenesis;Adaptive Immune Response | mmu04010;mmu04012;mmu04015;mmu04024;mmu04062;mmu04068;mmu04150;mmu04270;mmu04510;mmu04650;mmu04720;mmu04722;mmu04726;mmu04730;mmu04810;mmu04910;mmu04914;mmu05034;mmu05160;mmu05200;mmu05205;mmu05210;mmu05211;mmu05212;mmu05213;mmu05214;mmu05215;mmu05216;mmu05218;mmu05219;mmu05220;mmu05221;mmu05223 |  | Braf | slateblue3 | 16 | -4.16233766233766 |
  | Tubb4a-mRNA | -0.38 | 0.0956 | -0.567 | -0.192 | 0.769 | 0.675 | 0.875 | 0.00109 | 0.104 | loglinear | Adaptive Immune Response, Apoptosis, Cell Cycle, Neurons and Neurotransmission | NS\_MM\_NEUROINFLAM\_V1.0 | Tubb4a | mRNA | 0 |  | Neurons and Neurotransmission;Cell Cycle;Apoptosis;Adaptive Immune Response | mmu04145;mmu04540 |  | Tubb4a | slateblue4 | 16 | -3.97489539748954 |
  | Mapk10-mRNA | -0.539 | 0.138 | -0.81 | -0.269 | 0.688 | 0.57 | 0.83 | 0.00126 | 0.111 | lm.nb | Adaptive Immune Response, Apoptosis, Autophagy, Cellular Stress, Growth Factor Signaling, Innate Immune Response, Wnt | NS\_MM\_NEUROINFLAM\_V1.0 | Mapk10 | mRNA | 0 |  | Wnt;Innate Immune Response;Growth Factor Signaling;Cellular Stress;Autophagy;Apoptosis;Adaptive Immune Response | mmu04010;mmu04012;mmu04014;mmu04024;mmu04068;mmu04071;mmu04141;mmu04310;mmu04380;mmu04510;mmu04620;mmu04621;mmu04622;mmu04664;mmu04668;mmu04722;mmu04723;mmu04728;mmu04750;mmu04910;mmu04912;mmu04914;mmu04917;mmu04920;mmu04930;mmu04931;mmu04932;mmu04933;mmu05132;mmu05133;mmu05142;mmu05145;mmu05152;mmu05160;mmu05161;mmu05164;mmu05168;mmu05169;mmu05200;mmu05210;mmu05212;mmu05231 |  | Mapk10 | slateblue4 | 16 | -3.90579710144928 |
  | Prkar1a-mRNA | -0.329 | 0.0856 | -0.497 | -0.162 | 0.796 | 0.709 | 0.894 | 0.00141 | 0.116 | loglinear | Apoptosis, Growth Factor Signaling | NS\_MM\_NEUROINFLAM\_V1.0 | Prkar1a | mRNA | 0 |  | Growth Factor Signaling;Apoptosis | mmu04210;mmu04910 |  | Prkar1a | slateblue4 | 16 | -3.84345794392523 |
  | Psmb8-mRNA | 1.36 | 0.365 | 0.649 | 2.08 | 2.57 | 1.57 | 4.23 | 0.00179 | 0.129 | lm.nb | Adaptive Immune Response, Angiogenesis, Apoptosis, Astrocyte Function, Cell Cycle, Cytokine Signaling, Growth Factor Signaling, Inflammatory Signaling, Insulin Signaling, Microglia Function, NF-kB, Wnt | NS\_MM\_NEUROINFLAM\_V1.0 | Psmb8 | mRNA | 0 |  | Wnt;NF-kB;Microglia Function;Insulin Signaling;Inflammatory Signaling;Growth Factor Signaling;Cytokine Signaling;Cell Cycle;Astrocyte Function;Apoptosis;Angiogenesis;Adaptive Immune Response |  |  | Psmb8 | slateblue4 | 16 | 3.72602739726027 |
  | Bnip3l-mRNA | -0.61 | 0.172 | -0.947 | -0.273 | 0.655 | 0.519 | 0.828 | 0.00267 | 0.163 | lm.nb | Apoptosis, Autophagy | NS\_MM\_NEUROINFLAM\_V1.0 | Bnip3l | mRNA | 0 |  | Autophagy;Apoptosis |  |  | Bnip3l | slateblue4 | 16 | -3.54651162790698 |
  | Hrk-mRNA | -0.468 | 0.134 | -0.73 | -0.205 | 0.723 | 0.603 | 0.867 | 0.00301 | 0.177 | lm.nb | Apoptosis | NS\_MM\_NEUROINFLAM\_V1.0 | Hrk | mRNA | 0 |  | Apoptosis |  |  | Hrk | slateblue4 | 16 | -3.49253731343284 |
  | Lmna-mRNA | -0.702 | 0.204 | -1.1 | -0.303 | 0.615 | 0.466 | 0.811 | 0.0033 | 0.18 | lm.nb | Apoptosis, Cell Cycle, Microglia Function | NS\_MM\_NEUROINFLAM\_V1.0 | Lmna | mRNA | 0 |  | Microglia Function;Cell Cycle;Apoptosis | mmu05410;mmu05412;mmu05414 |  | Lmna | slateblue4 | 16 | -3.44117647058824 |
  | Xiap-mRNA | -0.529 | 0.155 | -0.833 | -0.225 | 0.693 | 0.561 | 0.856 | 0.00359 | 0.184 | lm.nb | Apoptosis, Innate Immune Response, NF-kB, Wnt | NS\_MM\_NEUROINFLAM\_V1.0 | Xiap | mRNA | 0 |  | Wnt;NF-kB;Innate Immune Response;Apoptosis | mmu04064;mmu04120;mmu04210;mmu04510;mmu05145;mmu05166;mmu05200;mmu05222 |  | Xiap | slateblue4 | 16 | -3.41290322580645 |
  | Hprt-mRNA | -0.392 | 0.116 | -0.619 | -0.166 | 0.762 | 0.651 | 0.891 | 0.0037 | 0.184 | lm.nb | Apoptosis | NS\_MM\_NEUROINFLAM\_V1.0 | Hprt | mRNA | 0 |  | Apoptosis | mmu00230 |  | Hprt | slateblue4 | 16 | -3.37931034482759 |
  | Igf1r-mRNA | 0.523 | 0.156 | 0.216 | 0.83 | 1.44 | 1.16 | 1.78 | 0.00414 | 0.197 | lm.nb | Apoptosis, Autophagy, Carbohydrate Metabolism, Growth Factor Signaling, Insulin Signaling | NS\_MM\_NEUROINFLAM\_V1.0 | Igf1r | mRNA | 0 |  | Insulin Signaling;Growth Factor Signaling;Carbohydrate Metabolism;Autophagy;Apoptosis | mmu04014;mmu04015;mmu04066;mmu04068;mmu04114;mmu04144;mmu04151;mmu04152;mmu04211;mmu04213;mmu04510;mmu04520;mmu04550;mmu04730;mmu04913;mmu04914;mmu05200;mmu05202;mmu05205;mmu05214;mmu05215;mmu05218 |  | Igf1r | slateblue4 | 16 | 3.3525641025641 |
  | Casp9-mRNA | -1.44 | 0.433 | -2.29 | -0.59 | 0.369 | 0.205 | 0.664 | 0.00432 | 0.2 | lm.nb | Adaptive Immune Response, Apoptosis, DNA Damage, Growth Factor Signaling, Innate Immune Response | NS\_MM\_NEUROINFLAM\_V1.0 | Casp9 | mRNA | 0 |  | Innate Immune Response;Growth Factor Signaling;DNA Damage;Apoptosis;Adaptive Immune Response | mmu04115;mmu04151;mmu04210;mmu04370;mmu04919;mmu05010;mmu05012;mmu05014;mmu05016;mmu05134;mmu05145;mmu05152;mmu05161;mmu05164;mmu05200;mmu05210;mmu05212;mmu05213;mmu05215;mmu05222;mmu05223;mmu05416 |  | Casp9 | slateblue4 | 16 | -3.3256351039261 |

  ##### DE Results - Apoptosis genes - DPI: 1 vs.CTRL

  Table displaying each sample's global significance scores and directed global significance scores as defined in the heatmaps above. The global significance score is calculated as the square root of the mean squared t-statistic for the genes in a gene set, with t-statistics coming from the linear regression underlying our differential expression analysis. The directed global significance score is calculated as the square root of the mean signed squared t-statistic for the genes in a gene set, with t-statistics coming from the linear regression underlying our differential expression analysis.

- **Volcano Plot: DPI: 2 vs.CTRL  
  More Plot Information**


  ##### Volcano Plot: DPI: 2 vs.CTRL

  Volcano plot displaying each gene's -log10(p-value) and log2 fold change for the selected covariate. Highly statistically significant genes fall at the top of the plot, and highly differentially expressed genes fall to either side. Genes within the selected gene set are highlighted in orange. Horizontal lines indicate various False Discovery Rate (FDR) thresholds.
- **DE Results - Apoptosis genes - DPI: 2 vs.CTRL  
  More Plot Information  Download CSV Data**

  | Probe Label | Log2 fold change | std error (log2) | Lower confidence limit (log2) | Upper confidence limit (log2) | Linear fold change | Lower confidence limit (linear) | Upper confidence limit (linear) | P-value | BY.p.value | method | Gene.sets | Codeset.Name | Probe.Label | Analyte.Type | Is.Control | Control.Type | Probe.Annotation | KEGG.Pathways | Cell.Type | Official.Gene.Name | volcanocol | volcanopch | tstats.all |
  | --- | --- | --- | --- | --- | --- | --- | --- | --- | --- | --- | --- | --- | --- | --- | --- | --- | --- | --- | --- | --- | --- | --- | --- |
  | Prkar2b-mRNA | -1.01 | 0.113 | -1.23 | -0.787 | 0.497 | 0.426 | 0.579 | 1.33e-07 | 3.91e-05 | loglinear | Apoptosis, Cell Cycle, Growth Factor Signaling | NS\_MM\_NEUROINFLAM\_V1.0 | Prkar2b | mRNA | 0 |  | Growth Factor Signaling;Cell Cycle;Apoptosis | mmu04210;mmu04910 |  | Prkar2b | slateblue1 | 16 | -8.93805309734513 |
  | Nfkbia-mRNA | 1.86 | 0.228 | 1.41 | 2.31 | 3.63 | 2.67 | 4.95 | 4.26e-07 | 9.99e-05 | lm.nb | Adaptive Immune Response, Apoptosis, Growth Factor Signaling, Inflammatory Signaling, Innate Immune Response, NF-kB | NS\_MM\_NEUROINFLAM\_V1.0 | Nfkbia | mRNA | 0 |  | NF-kB;Innate Immune Response;Inflammatory Signaling;Growth Factor Signaling;Apoptosis;Adaptive Immune Response | mmu04024;mmu04062;mmu04064;mmu04210;mmu04380;mmu04620;mmu04621;mmu04622;mmu04623;mmu04660;mmu04662;mmu04668;mmu04722;mmu04920;mmu04931;mmu05134;mmu05140;mmu05142;mmu05145;mmu05160;mmu05161;mmu05162;mmu05164;mmu05166;mmu05168;mmu05169;mmu05200;mmu05203;mmu05215;mmu05220;mmu05222 |  | Nfkbia | slateblue1 | 16 | 8.15789473684211 |
  | Prkar2a-mRNA | -0.311 | 0.0399 | -0.389 | -0.233 | 0.806 | 0.764 | 0.851 | 7.69e-07 | 0.000143 | loglinear | Apoptosis, Growth Factor Signaling | NS\_MM\_NEUROINFLAM\_V1.0 | Prkar2a | mRNA | 0 |  | Growth Factor Signaling;Apoptosis | mmu04210;mmu04910 |  | Prkar2a | slateblue1 | 16 | -7.79448621553885 |
  | Mapt-mRNA | -0.314 | 0.0425 | -0.397 | -0.23 | 0.805 | 0.759 | 0.852 | 1.56e-06 | 0.000239 | loglinear | Apoptosis, Cellular Stress, Growth Factor Signaling, Neurons and Neurotransmission | NS\_MM\_NEUROINFLAM\_V1.0 | Mapt | mRNA | 0 |  | Neurons and Neurotransmission;Growth Factor Signaling;Cellular Stress;Apoptosis | mmu04010;mmu05010 |  | Mapt | slateblue1 | 16 | -7.38823529411765 |
  | Psmb8-mRNA | 2.23 | 0.352 | 1.54 | 2.92 | 4.7 | 2.91 | 7.59 | 9.89e-06 | 0.000997 | lm.nb | Adaptive Immune Response, Angiogenesis, Apoptosis, Astrocyte Function, Cell Cycle, Cytokine Signaling, Growth Factor Signaling, Inflammatory Signaling, Insulin Signaling, Microglia Function, NF-kB, Wnt | NS\_MM\_NEUROINFLAM\_V1.0 | Psmb8 | mRNA | 0 |  | Wnt;NF-kB;Microglia Function;Insulin Signaling;Inflammatory Signaling;Growth Factor Signaling;Cytokine Signaling;Cell Cycle;Astrocyte Function;Apoptosis;Angiogenesis;Adaptive Immune Response |  |  | Psmb8 | slateblue1 | 16 | 6.33522727272727 |
  | Dapk1-mRNA | 0.584 | 0.0943 | 0.399 | 0.769 | 1.5 | 1.32 | 1.7 | 1.28e-05 | 0.00113 | loglinear | Apoptosis, Autophagy | NS\_MM\_NEUROINFLAM\_V1.0 | Dapk1 | mRNA | 0 |  | Autophagy;Apoptosis | mmu05200;mmu05219 |  | Dapk1 | slateblue1 | 16 | 6.19300106044539 |
  | Ppp3r1-mRNA | -0.334 | 0.0577 | -0.447 | -0.221 | 0.793 | 0.734 | 0.858 | 2.79e-05 | 0.00214 | loglinear | Adaptive Immune Response, Apoptosis, Growth Factor Signaling, Innate Immune Response, Wnt | NS\_MM\_NEUROINFLAM\_V1.0 | Ppp3r1 | mRNA | 0 |  | Wnt;Innate Immune Response;Growth Factor Signaling;Apoptosis;Adaptive Immune Response | mmu04010;mmu04020;mmu04022;mmu04114;mmu04210;mmu04310;mmu04360;mmu04370;mmu04380;mmu04650;mmu04660;mmu04662;mmu04720;mmu04724;mmu04921;mmu04922;mmu04924;mmu05010;mmu05014;mmu05031;mmu05152;mmu05166 |  | Ppp3r1 | slateblue1 | 16 | -5.78856152512998 |
  | Ppp3cb-mRNA | -0.554 | 0.098 | -0.747 | -0.362 | 0.681 | 0.596 | 0.778 | 3.56e-05 | 0.00246 | lm.nb | Adaptive Immune Response, Apoptosis, Growth Factor Signaling, Innate Immune Response, Wnt | NS\_MM\_NEUROINFLAM\_V1.0 | Ppp3cb | mRNA | 0 |  | Wnt;Innate Immune Response;Growth Factor Signaling;Apoptosis;Adaptive Immune Response | mmu04010;mmu04020;mmu04022;mmu04114;mmu04210;mmu04310;mmu04360;mmu04370;mmu04380;mmu04650;mmu04660;mmu04662;mmu04720;mmu04724;mmu04728;mmu04921;mmu04922;mmu04924;mmu05010;mmu05014;mmu05031;mmu05152;mmu05166 |  | Ppp3cb | slateblue1 | 16 | -5.6530612244898 |
  | Bcl2l1-mRNA | 0.407 | 0.0734 | 0.263 | 0.551 | 1.33 | 1.2 | 1.46 | 4.51e-05 | 0.00306 | loglinear | Apoptosis, Autophagy, Cytokine Signaling, Growth Factor Signaling, Innate Immune Response, NF-kB | NS\_MM\_NEUROINFLAM\_V1.0 | Bcl2l1 | mRNA | 0 |  | NF-kB;Innate Immune Response;Growth Factor Signaling;Cytokine Signaling;Autophagy;Apoptosis | mmu04014;mmu04064;mmu04151;mmu04210;mmu04630;mmu05014;mmu05145;mmu05166;mmu05200;mmu05202;mmu05212;mmu05220;mmu05222 |  | Bcl2l1 | slateblue1 | 16 | 5.54495912806539 |
  | Apc-mRNA | -0.322 | 0.0593 | -0.438 | -0.206 | 0.8 | 0.738 | 0.867 | 5.61e-05 | 0.00362 | loglinear | Apoptosis, Astrocyte Function, Wnt | NS\_MM\_NEUROINFLAM\_V1.0 | Apc | mRNA | 0 |  | Wnt;Astrocyte Function;Apoptosis | mmu04310;mmu04390;mmu04550;mmu04810;mmu05166;mmu05200;mmu05206;mmu05210;mmu05213 |  | Apc | slateblue1 | 16 | -5.43001686340641 |
  | Prkar1a-mRNA | -0.452 | 0.0856 | -0.619 | -0.284 | 0.731 | 0.651 | 0.821 | 7.47e-05 | 0.00439 | loglinear | Apoptosis, Growth Factor Signaling | NS\_MM\_NEUROINFLAM\_V1.0 | Prkar1a | mRNA | 0 |  | Growth Factor Signaling;Apoptosis | mmu04210;mmu04910 |  | Prkar1a | slateblue1 | 16 | -5.2803738317757 |
  | Igf1r-mRNA | 0.813 | 0.155 | 0.51 | 1.12 | 1.76 | 1.42 | 2.17 | 7.74e-05 | 0.00441 | lm.nb | Apoptosis, Autophagy, Carbohydrate Metabolism, Growth Factor Signaling, Insulin Signaling | NS\_MM\_NEUROINFLAM\_V1.0 | Igf1r | mRNA | 0 |  | Insulin Signaling;Growth Factor Signaling;Carbohydrate Metabolism;Autophagy;Apoptosis | mmu04014;mmu04015;mmu04066;mmu04068;mmu04114;mmu04144;mmu04151;mmu04152;mmu04211;mmu04213;mmu04510;mmu04520;mmu04550;mmu04730;mmu04913;mmu04914;mmu05200;mmu05202;mmu05205;mmu05214;mmu05215;mmu05218 |  | Igf1r | slateblue1 | 16 | 5.24516129032258 |
  | Tfg-mRNA | -0.429 | 0.0822 | -0.59 | -0.268 | 0.743 | 0.664 | 0.831 | 8.41e-05 | 0.00464 | loglinear | Apoptosis, Neurons and Neurotransmission | NS\_MM\_NEUROINFLAM\_V1.0 | Tfg | mRNA | 0 |  | Neurons and Neurotransmission;Apoptosis | mmu05200;mmu05216 |  | Tfg | slateblue1 | 16 | -5.21897810218978 |
  | Prkacb-mRNA | -0.322 | 0.0629 | -0.445 | -0.199 | 0.8 | 0.735 | 0.871 | 0.000104 | 0.00562 | loglinear | Adaptive Immune Response, Angiogenesis, Apoptosis, Autophagy, Growth Factor Signaling, Lipid Metabolism, Neurons and Neurotransmission, Wnt | NS\_MM\_NEUROINFLAM\_V1.0 | Prkacb | mRNA | 0 |  | Wnt;Neurons and Neurotransmission;Lipid Metabolism;Growth Factor Signaling;Autophagy;Apoptosis;Angiogenesis;Adaptive Immune Response | mmu04010;mmu04014;mmu04020;mmu04024;mmu04062;mmu04114;mmu04210;mmu04211;mmu04213;mmu04261;mmu04270;mmu04310;mmu04540;mmu04611;mmu04713;mmu04720;mmu04723;mmu04724;mmu04725;mmu04726;mmu04728;mmu04750;mmu04910;mmu04911;mmu04912;mmu04913;mmu04914;mmu04915;mmu04916;mmu04919;mmu04921;mmu04922;mmu04923;mmu04924;mmu04925;mmu05012;mmu05020;mmu05030;mmu05031;mmu05146;mmu05166;mmu05169;mmu05200;mmu05203;mmu05205;mmu05414 |  | Prkacb | slateblue1 | 16 | -5.11923688394277 |
  | Tubb3-mRNA | -0.799 | 0.158 | -1.11 | -0.489 | 0.575 | 0.463 | 0.713 | 0.000119 | 0.00609 | lm.nb | Adaptive Immune Response, Apoptosis, Cell Cycle, Neurons and Neurotransmission | NS\_MM\_NEUROINFLAM\_V1.0 | Tubb3 | mRNA | 0 |  | Neurons and Neurotransmission;Cell Cycle;Apoptosis;Adaptive Immune Response | mmu04145;mmu04540 |  | Tubb3 | slateblue1 | 16 | -5.05696202531646 |
  | Bcl2l2-mRNA | -0.504 | 0.101 | -0.702 | -0.305 | 0.705 | 0.615 | 0.809 | 0.00014 | 0.0069 | loglinear | Apoptosis | NS\_MM\_NEUROINFLAM\_V1.0 | Bcl2l2 | mRNA | 0 |  | Apoptosis | mmu05206 |  | Bcl2l2 | slateblue1 | 16 | -4.99009900990099 |
  | Irf1-mRNA | 1.63 | 0.335 | 0.973 | 2.29 | 3.09 | 1.96 | 4.88 | 0.000172 | 0.0082 | lm.nb | Apoptosis, Inflammatory Signaling, Innate Immune Response | NS\_MM\_NEUROINFLAM\_V1.0 | Irf1 | mRNA | 0 |  | Innate Immune Response;Inflammatory Signaling;Apoptosis | mmu04917;mmu05133;mmu05160 |  | Irf1 | slateblue1 | 16 | 4.86567164179104 |
  | Mapk10-mRNA | -0.673 | 0.138 | -0.943 | -0.402 | 0.627 | 0.52 | 0.757 | 0.000172 | 0.0082 | lm.nb | Adaptive Immune Response, Apoptosis, Autophagy, Cellular Stress, Growth Factor Signaling, Innate Immune Response, Wnt | NS\_MM\_NEUROINFLAM\_V1.0 | Mapk10 | mRNA | 0 |  | Wnt;Innate Immune Response;Growth Factor Signaling;Cellular Stress;Autophagy;Apoptosis;Adaptive Immune Response | mmu04010;mmu04012;mmu04014;mmu04024;mmu04068;mmu04071;mmu04141;mmu04310;mmu04380;mmu04510;mmu04620;mmu04621;mmu04622;mmu04664;mmu04668;mmu04722;mmu04723;mmu04728;mmu04750;mmu04910;mmu04912;mmu04914;mmu04917;mmu04920;mmu04930;mmu04931;mmu04932;mmu04933;mmu05132;mmu05133;mmu05142;mmu05145;mmu05152;mmu05160;mmu05161;mmu05164;mmu05168;mmu05169;mmu05200;mmu05210;mmu05212;mmu05231 |  | Mapk10 | slateblue1 | 16 | -4.8768115942029 |
  | Lmna-mRNA | -0.993 | 0.205 | -1.39 | -0.592 | 0.502 | 0.38 | 0.664 | 0.000178 | 0.00829 | lm.nb | Apoptosis, Cell Cycle, Microglia Function | NS\_MM\_NEUROINFLAM\_V1.0 | Lmna | mRNA | 0 |  | Microglia Function;Cell Cycle;Apoptosis | mmu05410;mmu05412;mmu05414 |  | Lmna | slateblue1 | 16 | -4.84390243902439 |
  | Prkcq-mRNA | -0.864 | 0.185 | -1.23 | -0.501 | 0.549 | 0.427 | 0.707 | 0.000258 | 0.0106 | lm.nb | Adaptive Immune Response, Apoptosis, Autophagy, NF-kB, Oligodendrocyte Function | NS\_MM\_NEUROINFLAM\_V1.0 | Prkcq | mRNA | 0 |  | Oligodendrocyte Function;NF-kB;Autophagy;Apoptosis;Adaptive Immune Response | mmu04064;mmu04270;mmu04530;mmu04660;mmu04750;mmu04920;mmu04931;mmu05162 | Oligodendrocytes | Prkcq | slateblue2 | 16 | -4.67027027027027 |

  ##### DE Results - Apoptosis genes - DPI: 2 vs.CTRL

  Table displaying each sample's global significance scores and directed global significance scores as defined in the heatmaps above. The global significance score is calculated as the square root of the mean squared t-statistic for the genes in a gene set, with t-statistics coming from the linear regression underlying our differential expression analysis. The directed global significance score is calculated as the square root of the mean signed squared t-statistic for the genes in a gene set, with t-statistics coming from the linear regression underlying our differential expression analysis.

- **Volcano Plot: DPI: 3 vs.CTRL  
  More Plot Information**


  ##### Volcano Plot: DPI: 3 vs.CTRL

  Volcano plot displaying each gene's -log10(p-value) and log2 fold change for the selected covariate. Highly statistically significant genes fall at the top of the plot, and highly differentially expressed genes fall to either side. Genes within the selected gene set are highlighted in orange. Horizontal lines indicate various False Discovery Rate (FDR) thresholds.
- **DE Results - Apoptosis genes - DPI: 3 vs.CTRL  
  More Plot Information  Download CSV Data**

  | Probe Label | Log2 fold change | std error (log2) | Lower confidence limit (log2) | Upper confidence limit (log2) | Linear fold change | Lower confidence limit (linear) | Upper confidence limit (linear) | P-value | BY.p.value | method | Gene.sets | Codeset.Name | Probe.Label | Analyte.Type | Is.Control | Control.Type | Probe.Annotation | KEGG.Pathways | Cell.Type | Official.Gene.Name | volcanocol | volcanopch | tstats.all |
  | --- | --- | --- | --- | --- | --- | --- | --- | --- | --- | --- | --- | --- | --- | --- | --- | --- | --- | --- | --- | --- | --- | --- | --- |
  | Nfkbia-mRNA | 2.4 | 0.224 | 1.96 | 2.84 | 5.27 | 3.88 | 7.14 | 1.06e-08 | 3.12e-06 | lm.nb | Adaptive Immune Response, Apoptosis, Growth Factor Signaling, Inflammatory Signaling, Innate Immune Response, NF-kB | NS\_MM\_NEUROINFLAM\_V1.0 | Nfkbia | mRNA | 0 |  | NF-kB;Innate Immune Response;Inflammatory Signaling;Growth Factor Signaling;Apoptosis;Adaptive Immune Response | mmu04024;mmu04062;mmu04064;mmu04210;mmu04380;mmu04620;mmu04621;mmu04622;mmu04623;mmu04660;mmu04662;mmu04668;mmu04722;mmu04920;mmu04931;mmu05134;mmu05140;mmu05142;mmu05145;mmu05160;mmu05161;mmu05162;mmu05164;mmu05166;mmu05168;mmu05169;mmu05200;mmu05203;mmu05215;mmu05220;mmu05222 |  | Nfkbia | slateblue1 | 16 | 10.7142857142857 |
  | Mapt-mRNA | -0.413 | 0.0425 | -0.496 | -0.33 | 0.751 | 0.709 | 0.796 | 4.11e-08 | 9.67e-06 | loglinear | Apoptosis, Cellular Stress, Growth Factor Signaling, Neurons and Neurotransmission | NS\_MM\_NEUROINFLAM\_V1.0 | Mapt | mRNA | 0 |  | Neurons and Neurotransmission;Growth Factor Signaling;Cellular Stress;Apoptosis | mmu04010;mmu05010 |  | Mapt | slateblue1 | 16 | -9.71764705882353 |
  | Prkar2b-mRNA | -1.06 | 0.113 | -1.28 | -0.834 | 0.481 | 0.412 | 0.561 | 7.17e-08 | 1.41e-05 | loglinear | Apoptosis, Cell Cycle, Growth Factor Signaling | NS\_MM\_NEUROINFLAM\_V1.0 | Prkar2b | mRNA | 0 |  | Growth Factor Signaling;Cell Cycle;Apoptosis | mmu04210;mmu04910 |  | Prkar2b | slateblue1 | 16 | -9.38053097345133 |
  | Dapk1-mRNA | 0.866 | 0.0943 | 0.681 | 1.05 | 1.82 | 1.6 | 2.07 | 8.86e-08 | 1.65e-05 | loglinear | Apoptosis, Autophagy | NS\_MM\_NEUROINFLAM\_V1.0 | Dapk1 | mRNA | 0 |  | Autophagy;Apoptosis | mmu05200;mmu05219 |  | Dapk1 | slateblue1 | 16 | 9.18345705196182 |
  | Irf3-mRNA | -1.48 | 0.165 | -1.8 | -1.15 | 0.359 | 0.287 | 0.45 | 1.3e-07 | 2.18e-05 | lm.nb | Apoptosis, Cytokine Signaling, Inflammatory Signaling, Innate Immune Response | NS\_MM\_NEUROINFLAM\_V1.0 | Irf3 | mRNA | 0 |  | Innate Immune Response;Inflammatory Signaling;Cytokine Signaling;Apoptosis | mmu04620;mmu04622;mmu04623;mmu05133;mmu05160;mmu05161;mmu05162;mmu05164;mmu05168;mmu05169;mmu05203 |  | Irf3 | slateblue1 | 16 | -8.96969696969697 |
  | Psmb8-mRNA | 2.99 | 0.346 | 2.31 | 3.67 | 7.95 | 4.97 | 12.7 | 1.99e-07 | 3.2e-05 | lm.nb | Adaptive Immune Response, Angiogenesis, Apoptosis, Astrocyte Function, Cell Cycle, Cytokine Signaling, Growth Factor Signaling, Inflammatory Signaling, Insulin Signaling, Microglia Function, NF-kB, Wnt | NS\_MM\_NEUROINFLAM\_V1.0 | Psmb8 | mRNA | 0 |  | Wnt;NF-kB;Microglia Function;Insulin Signaling;Inflammatory Signaling;Growth Factor Signaling;Cytokine Signaling;Cell Cycle;Astrocyte Function;Apoptosis;Angiogenesis;Adaptive Immune Response |  |  | Psmb8 | slateblue1 | 16 | 8.64161849710983 |
  | Irf1-mRNA | 2.84 | 0.332 | 2.19 | 3.49 | 7.17 | 4.57 | 11.3 | 2.3e-07 | 3.39e-05 | lm.nb | Apoptosis, Inflammatory Signaling, Innate Immune Response | NS\_MM\_NEUROINFLAM\_V1.0 | Irf1 | mRNA | 0 |  | Innate Immune Response;Inflammatory Signaling;Apoptosis | mmu04917;mmu05133;mmu05160 |  | Irf1 | slateblue1 | 16 | 8.55421686746988 |
  | Ikbkb-mRNA | -0.601 | 0.0868 | -0.772 | -0.431 | 0.659 | 0.586 | 0.742 | 3.42e-06 | 0.00028 | loglinear | Adaptive Immune Response, Apoptosis, Cytokine Signaling, Growth Factor Signaling, Inflammatory Signaling, Innate Immune Response, NF-kB | NS\_MM\_NEUROINFLAM\_V1.0 | Ikbkb | mRNA | 0 |  | NF-kB;Innate Immune Response;Inflammatory Signaling;Growth Factor Signaling;Cytokine Signaling;Apoptosis;Adaptive Immune Response | mmu04010;mmu04014;mmu04062;mmu04064;mmu04068;mmu04150;mmu04151;mmu04210;mmu04380;mmu04620;mmu04621;mmu04622;mmu04623;mmu04660;mmu04662;mmu04668;mmu04722;mmu04910;mmu04920;mmu04930;mmu04931;mmu04932;mmu05142;mmu05145;mmu05160;mmu05161;mmu05164;mmu05166;mmu05168;mmu05169;mmu05200;mmu05206;mmu05212;mmu05215;mmu05220;mmu05221;mmu05222 |  | Ikbkb | slateblue1 | 16 | -6.92396313364055 |
  | Tnfrsf1a-mRNA | 1.92 | 0.281 | 1.37 | 2.47 | 3.79 | 2.59 | 5.56 | 3.96e-06 | 0.000317 | lm.nb | Apoptosis, Cytokine Signaling, Growth Factor Signaling, Innate Immune Response, NF-kB | NS\_MM\_NEUROINFLAM\_V1.0 | Tnfrsf1a | mRNA | 0 |  | NF-kB;Innate Immune Response;Growth Factor Signaling;Cytokine Signaling;Apoptosis | mmu04010;mmu04060;mmu04064;mmu04071;mmu04210;mmu04380;mmu04668;mmu04920;mmu04931;mmu04932;mmu05010;mmu05014;mmu05142;mmu05145;mmu05152;mmu05160;mmu05164;mmu05166;mmu05168 |  | Tnfrsf1a | slateblue1 | 16 | 6.83274021352313 |
  | Parp1-mRNA | -0.736 | 0.108 | -0.949 | -0.524 | 0.6 | 0.518 | 0.695 | 4.31e-06 | 0.000334 | loglinear | Apoptosis, Cellular Stress, DNA Damage, NF-kB | NS\_MM\_NEUROINFLAM\_V1.0 | Parp1 | mRNA | 0 |  | NF-kB;DNA Damage;Cellular Stress;Apoptosis | mmu03410;mmu04064 |  | Parp1 | slateblue1 | 16 | -6.81481481481481 |
  | Prkar2a-mRNA | -0.23 | 0.0399 | -0.308 | -0.152 | 0.853 | 0.808 | 0.9 | 2.9e-05 | 0.00153 | loglinear | Apoptosis, Growth Factor Signaling | NS\_MM\_NEUROINFLAM\_V1.0 | Prkar2a | mRNA | 0 |  | Growth Factor Signaling;Apoptosis | mmu04210;mmu04910 |  | Prkar2a | slateblue1 | 16 | -5.76441102756892 |
  | Bcl2l1-mRNA | 0.414 | 0.0734 | 0.27 | 0.558 | 1.33 | 1.21 | 1.47 | 3.75e-05 | 0.00184 | loglinear | Apoptosis, Autophagy, Cytokine Signaling, Growth Factor Signaling, Innate Immune Response, NF-kB | NS\_MM\_NEUROINFLAM\_V1.0 | Bcl2l1 | mRNA | 0 |  | NF-kB;Innate Immune Response;Growth Factor Signaling;Cytokine Signaling;Autophagy;Apoptosis | mmu04014;mmu04064;mmu04151;mmu04210;mmu04630;mmu05014;mmu05145;mmu05166;mmu05200;mmu05202;mmu05212;mmu05220;mmu05222 |  | Bcl2l1 | slateblue1 | 16 | 5.64032697547684 |
  | Traf6-mRNA | 0.92 | 0.166 | 0.594 | 1.25 | 1.89 | 1.51 | 2.37 | 4.54e-05 | 0.00214 | lm.nb | Adaptive Immune Response, Apoptosis, Autophagy, Cytokine Signaling, Growth Factor Signaling, Innate Immune Response, NF-kB | NS\_MM\_NEUROINFLAM\_V1.0 | Traf6 | mRNA | 0 |  | NF-kB;Innate Immune Response;Growth Factor Signaling;Cytokine Signaling;Autophagy;Apoptosis;Adaptive Immune Response | mmu04010;mmu04064;mmu04120;mmu04144;mmu04380;mmu04620;mmu04621;mmu04622;mmu04722;mmu05133;mmu05140;mmu05142;mmu05145;mmu05152;mmu05160;mmu05162;mmu05168;mmu05169;mmu05200;mmu05222 |  | Traf6 | slateblue1 | 16 | 5.54216867469879 |
  | Akt2-mRNA | -0.766 | 0.142 | -1.04 | -0.488 | 0.588 | 0.485 | 0.713 | 5.85e-05 | 0.00265 | loglinear | Adaptive Immune Response, Angiogenesis, Apoptosis, Autophagy, Carbohydrate Metabolism, Cytokine Signaling, Growth Factor Signaling, Innate Immune Response, Insulin Signaling, Neurons and Neurotransmission, Wnt | NS\_MM\_NEUROINFLAM\_V1.0 | Akt2 | mRNA | 0 |  | Wnt;Neurons and Neurotransmission;Insulin Signaling;Innate Immune Response;Growth Factor Signaling;Cytokine Signaling;Carbohydrate Metabolism;Autophagy;Apoptosis;Angiogenesis;Adaptive Immune Response | mmu04010;mmu04012;mmu04014;mmu04015;mmu04022;mmu04024;mmu04062;mmu04066;mmu04068;mmu04071;mmu04072;mmu04150;mmu04151;mmu04152;mmu04210;mmu04211;mmu04213;mmu04261;mmu04370;mmu04380;mmu04510;mmu04530;mmu04550;mmu04611;mmu04620;mmu04630;mmu04660;mmu04662;mmu04664;mmu04666;mmu04668;mmu04722;mmu04725;mmu04728;mmu04910;mmu04914;mmu04915;mmu04917;mmu04919;mmu04920;mmu04922;mmu04923;mmu04931;mmu04932;mmu04933;mmu04973;mmu05142;mmu05145;mmu05152;mmu05160;mmu05161;mmu05162;mmu05164;mmu05166;mmu05169;mmu05200;mmu05205;mmu05210;mmu05211;mmu05212;mmu05213;mmu05214;mmu05215;mmu05218;mmu05220;mmu05221;mmu05222;mmu05223;mmu05230;mmu05231 |  | Akt2 | slateblue1 | 16 | -5.3943661971831 |
  | Chuk-mRNA | -0.423 | 0.0787 | -0.577 | -0.269 | 0.746 | 0.67 | 0.83 | 6.21e-05 | 0.00275 | loglinear | Adaptive Immune Response, Apoptosis, Cytokine Signaling, Growth Factor Signaling, Inflammatory Signaling, Innate Immune Response, NF-kB | NS\_MM\_NEUROINFLAM\_V1.0 | Chuk | mRNA | 0 |  | NF-kB;Innate Immune Response;Inflammatory Signaling;Growth Factor Signaling;Cytokine Signaling;Apoptosis;Adaptive Immune Response | mmu04010;mmu04014;mmu04062;mmu04064;mmu04068;mmu04151;mmu04210;mmu04380;mmu04620;mmu04621;mmu04622;mmu04623;mmu04660;mmu04662;mmu04668;mmu04920;mmu05142;mmu05145;mmu05160;mmu05161;mmu05162;mmu05166;mmu05168;mmu05169;mmu05200;mmu05212;mmu05215;mmu05220;mmu05221;mmu05222 |  | Chuk | slateblue1 | 16 | -5.37484116899619 |
  | Ripk2-mRNA | 1 | 0.193 | 0.627 | 1.38 | 2.01 | 1.54 | 2.61 | 8.44e-05 | 0.00355 | loglinear | Adaptive Immune Response, Apoptosis, Cytokine Signaling, Growth Factor Signaling, Innate Immune Response | NS\_MM\_NEUROINFLAM\_V1.0 | Ripk2 | mRNA | 0 |  | Innate Immune Response;Growth Factor Signaling;Cytokine Signaling;Apoptosis;Adaptive Immune Response | mmu04621;mmu04722;mmu05152 |  | Ripk2 | slateblue1 | 16 | 5.18134715025907 |
  | Bnip3-mRNA | 0.275 | 0.0611 | 0.155 | 0.394 | 1.21 | 1.11 | 1.31 | 0.00037 | 0.0126 | loglinear | Apoptosis, Autophagy, Cellular Stress, Microglia Function | NS\_MM\_NEUROINFLAM\_V1.0 | Bnip3 | mRNA | 0 |  | Microglia Function;Cellular Stress;Autophagy;Apoptosis | mmu04068;mmu05134 |  | Bnip3 | slateblue2 | 16 | 4.50081833060556 |
  | Traf3-mRNA | -0.463 | 0.104 | -0.666 | -0.26 | 0.725 | 0.63 | 0.835 | 0.000386 | 0.013 | loglinear | Apoptosis, Cytokine Signaling, Inflammatory Signaling, Innate Immune Response, NF-kB | NS\_MM\_NEUROINFLAM\_V1.0 | Traf3 | mRNA | 0 |  | NF-kB;Innate Immune Response;Inflammatory Signaling;Cytokine Signaling;Apoptosis | mmu04064;mmu04620;mmu04622;mmu04668;mmu05160;mmu05168;mmu05169;mmu05200;mmu05203;mmu05222 |  | Traf3 | slateblue2 | 16 | -4.45192307692308 |
  | Traf2-mRNA | -0.552 | 0.129 | -0.805 | -0.298 | 0.682 | 0.572 | 0.813 | 0.000592 | 0.0183 | lm.nb | Apoptosis, Cytokine Signaling, Growth Factor Signaling, Inflammatory Signaling, Innate Immune Response, NF-kB | NS\_MM\_NEUROINFLAM\_V1.0 | Traf2 | mRNA | 0 |  | NF-kB;Innate Immune Response;Inflammatory Signaling;Growth Factor Signaling;Cytokine Signaling;Apoptosis | mmu04010;mmu04064;mmu04071;mmu04141;mmu04210;mmu04380;mmu04622;mmu04668;mmu04920;mmu04932;mmu05160;mmu05168;mmu05169;mmu05200;mmu05203;mmu05222 |  | Traf2 | slateblue2 | 16 | -4.27906976744186 |
  | Tubb3-mRNA | -0.664 | 0.158 | -0.975 | -0.354 | 0.631 | 0.509 | 0.782 | 0.000683 | 0.021 | lm.nb | Adaptive Immune Response, Apoptosis, Cell Cycle, Neurons and Neurotransmission | NS\_MM\_NEUROINFLAM\_V1.0 | Tubb3 | mRNA | 0 |  | Neurons and Neurotransmission;Cell Cycle;Apoptosis;Adaptive Immune Response | mmu04145;mmu04540 |  | Tubb3 | slateblue2 | 16 | -4.20253164556962 |

  ##### DE Results - Apoptosis genes - DPI: 3 vs.CTRL

  Table displaying each sample's global significance scores and directed global significance scores as defined in the heatmaps above. The global significance score is calculated as the square root of the mean squared t-statistic for the genes in a gene set, with t-statistics coming from the linear regression underlying our differential expression analysis. The directed global significance score is calculated as the square root of the mean signed squared t-statistic for the genes in a gene set, with t-statistics coming from the linear regression underlying our differential expression analysis.

DPI:
- DPI: differential expression in 1 vs. baseline of CTRL
- DPI: differential expression in 2 vs. baseline of CTRL
- DPI: differential expression in 3 vs. baseline of CTRL

- **Volcano Plot: DPI: 1 vs.CTRL  
  More Plot Information**


  ##### Volcano Plot: DPI: 1 vs.CTRL

  Volcano plot displaying each gene's -log10(p-value) and log2 fold change for the selected covariate. Highly statistically significant genes fall at the top of the plot, and highly differentially expressed genes fall to either side. Genes within the selected gene set are highlighted in orange. Horizontal lines indicate various False Discovery Rate (FDR) thresholds.
- **DE Results - Astrocyte Function genes - DPI: 1 vs.CTRL  
  More Plot Information  Download CSV Data**

  | Probe Label | Log2 fold change | std error (log2) | Lower confidence limit (log2) | Upper confidence limit (log2) | Linear fold change | Lower confidence limit (linear) | Upper confidence limit (linear) | P-value | BY.p.value | method | Gene.sets | Codeset.Name | Probe.Label | Analyte.Type | Is.Control | Control.Type | Probe.Annotation | KEGG.Pathways | Cell.Type | Official.Gene.Name | volcanocol | volcanopch | tstats.all |
  | --- | --- | --- | --- | --- | --- | --- | --- | --- | --- | --- | --- | --- | --- | --- | --- | --- | --- | --- | --- | --- | --- | --- | --- |
  | Cp-mRNA | 0.641 | 0.138 | 0.371 | 0.911 | 1.56 | 1.29 | 1.88 | 0.000262 | 0.0536 | loglinear | Astrocyte Function | NS\_MM\_NEUROINFLAM\_V1.0 | Cp | mRNA | 0 |  | Astrocyte Function |  |  | Cp | slateblue3 | 16 | 4.64492753623188 |
  | Amigo2-mRNA | 0.619 | 0.135 | 0.354 | 0.883 | 1.54 | 1.28 | 1.84 | 0.000304 | 0.0536 | lm.nb | Astrocyte Function | NS\_MM\_NEUROINFLAM\_V1.0 | Amigo2 | mRNA | 0 |  | Astrocyte Function |  |  | Amigo2 | slateblue3 | 16 | 4.58518518518518 |
  | Psmb8-mRNA | 1.36 | 0.365 | 0.649 | 2.08 | 2.57 | 1.57 | 4.23 | 0.00179 | 0.129 | lm.nb | Adaptive Immune Response, Angiogenesis, Apoptosis, Astrocyte Function, Cell Cycle, Cytokine Signaling, Growth Factor Signaling, Inflammatory Signaling, Insulin Signaling, Microglia Function, NF-kB, Wnt | NS\_MM\_NEUROINFLAM\_V1.0 | Psmb8 | mRNA | 0 |  | Wnt;NF-kB;Microglia Function;Insulin Signaling;Inflammatory Signaling;Growth Factor Signaling;Cytokine Signaling;Cell Cycle;Astrocyte Function;Apoptosis;Angiogenesis;Adaptive Immune Response |  |  | Psmb8 | slateblue4 | 16 | 3.72602739726027 |
  | Apc-mRNA | -0.195 | 0.0593 | -0.311 | -0.0783 | 0.874 | 0.806 | 0.947 | 0.00471 | 0.206 | loglinear | Apoptosis, Astrocyte Function, Wnt | NS\_MM\_NEUROINFLAM\_V1.0 | Apc | mRNA | 0 |  | Wnt;Astrocyte Function;Apoptosis | mmu04310;mmu04390;mmu04550;mmu04810;mmu05166;mmu05200;mmu05206;mmu05210;mmu05213 |  | Apc | slateblue4 | 16 | -3.28836424957841 |
  | B3gnt5-mRNA | 0.889 | 0.305 | 0.292 | 1.49 | 1.85 | 1.22 | 2.8 | 0.01 | 0.308 | lm.nb | Astrocyte Function | NS\_MM\_NEUROINFLAM\_V1.0 | B3gnt5 | mRNA | 0 |  | Astrocyte Function |  |  | B3gnt5 | slateblue4 | 16 | 2.91475409836066 |
  | Vim-mRNA | -0.843 | 0.292 | -1.41 | -0.27 | 0.558 | 0.375 | 0.829 | 0.0107 | 0.327 | lm.nb | Apoptosis, Astrocyte Function | NS\_MM\_NEUROINFLAM\_V1.0 | Vim | mRNA | 0 |  | Astrocyte Function;Apoptosis | mmu05169;mmu05206 |  | Vim | slateblue4 | 16 | -2.88698630136986 |
  | Gbp2-mRNA | 2.09 | 0.774 | 0.571 | 3.6 | 4.25 | 1.49 | 12.2 | 0.0158 | 0.41 | lm.nb | Astrocyte Function, Inflammatory Signaling | NS\_MM\_NEUROINFLAM\_V1.0 | Gbp2 | mRNA | 0 |  | Inflammatory Signaling;Astrocyte Function |  |  | Gbp2 | slateblue4 | 16 | 2.70025839793282 |
  | Padi2-mRNA | -0.42 | 0.16 | -0.733 | -0.107 | 0.747 | 0.601 | 0.928 | 0.0181 | 0.438 | lm.nb | Astrocyte Function, Epigenetic Regulation | NS\_MM\_NEUROINFLAM\_V1.0 | Padi2 | mRNA | 0 |  | Epigenetic Regulation;Astrocyte Function |  |  | Padi2 | slateblue4 | 16 | -2.625 |
  | Itga7-mRNA | -0.948 | 0.371 | -1.67 | -0.221 | 0.519 | 0.313 | 0.858 | 0.0212 | 0.485 | lm.nb | Astrocyte Function, Growth Factor Signaling, Matrix Remodeling | NS\_MM\_NEUROINFLAM\_V1.0 | Itga7 | mRNA | 0 |  | Matrix Remodeling;Growth Factor Signaling;Astrocyte Function | mmu04151;mmu04510;mmu04512;mmu04810;mmu05410;mmu05412;mmu05414 | Astrocytes | Itga7 | slateblue4 | 16 | -2.55525606469003 |
  | Gdpd2-mRNA | -0.807 | 0.337 | -1.47 | -0.147 | 0.571 | 0.361 | 0.903 | 0.0292 | 0.599 | lm.nb | Astrocyte Function, Lipid Metabolism | NS\_MM\_NEUROINFLAM\_V1.0 | Gdpd2 | mRNA | 0 |  | Lipid Metabolism;Astrocyte Function |  | Astrocytes | Gdpd2 | azure4 | 1 | -2.3946587537092 |
  | Lcn2-mRNA | 1.85 | 0.799 | 0.283 | 3.41 | 3.6 | 1.22 | 10.7 | 0.0343 | 0.654 | lm.nb | Astrocyte Function, Inflammatory Signaling | NS\_MM\_NEUROINFLAM\_V1.0 | Lcn2 | mRNA | 0 |  | Inflammatory Signaling;Astrocyte Function |  |  | Lcn2 | azure4 | 1 | 2.3153942428035 |
  | Aldh1l1-mRNA | 0.26 | 0.116 | 0.0318 | 0.488 | 1.2 | 1.02 | 1.4 | 0.0402 | 0.724 | loglinear | Astrocyte Function | NS\_MM\_NEUROINFLAM\_V1.0 | Aldh1l1 | mRNA | 0 |  | Astrocyte Function |  | Astrocytes | Aldh1l1 | azure4 | 1 | 2.24137931034483 |
  | Fbln5-mRNA | 0.828 | 0.384 | 0.0745 | 1.58 | 1.78 | 1.05 | 2.99 | 0.0468 | 0.799 | lm.nb | Astrocyte Function, Matrix Remodeling | NS\_MM\_NEUROINFLAM\_V1.0 | Fbln5 | mRNA | 0 |  | Matrix Remodeling;Astrocyte Function |  |  | Fbln5 | azure4 | 1 | 2.15625 |
  | Entpd2-mRNA | 1.13 | 0.559 | 0.0365 | 2.23 | 2.19 | 1.03 | 4.69 | 0.0598 | 0.93 | lm.nb | Astrocyte Function | NS\_MM\_NEUROINFLAM\_V1.0 | Entpd2 | mRNA | 0 |  | Astrocyte Function | mmu00230 | Astrocytes | Entpd2 | azure4 | 1 | 2.02146690518784 |
  | Egfr-mRNA | 0.542 | 0.287 | -0.0206 | 1.1 | 1.46 | 0.986 | 2.15 | 0.0773 | 1 | lm.nb | Adaptive Immune Response, Angiogenesis, Astrocyte Function, Cytokine Signaling, Growth Factor Signaling, Insulin Signaling | NS\_MM\_NEUROINFLAM\_V1.0 | Egfr | mRNA | 0 |  | Insulin Signaling;Growth Factor Signaling;Cytokine Signaling;Astrocyte Function;Angiogenesis;Adaptive Immune Response | mmu04010;mmu04012;mmu04014;mmu04015;mmu04020;mmu04060;mmu04066;mmu04068;mmu04072;mmu04144;mmu04151;mmu04510;mmu04520;mmu04540;mmu04810;mmu04912;mmu04915;mmu04921;mmu05160;mmu05200;mmu05205;mmu05206;mmu05212;mmu05213;mmu05214;mmu05215;mmu05218;mmu05219;mmu05223;mmu05230;mmu05231 | Astrocytes | Egfr | azure4 | 1 | 1.88850174216028 |
  | Fkbp5-mRNA | -0.29 | 0.181 | -0.645 | 0.0657 | 0.818 | 0.64 | 1.05 | 0.13 | 1 | lm.nb | Astrocyte Function | NS\_MM\_NEUROINFLAM\_V1.0 | Fkbp5 | mRNA | 0 |  | Astrocyte Function | mmu04915 |  | Fkbp5 | azure4 | 1 | -1.60220994475138 |
  | Kcnj10-mRNA | 0.186 | 0.119 | -0.0475 | 0.42 | 1.14 | 0.968 | 1.34 | 0.138 | 1 | lm.nb | Astrocyte Function, Neurons and Neurotransmission | NS\_MM\_NEUROINFLAM\_V1.0 | Kcnj10 | mRNA | 0 |  | Neurons and Neurotransmission;Astrocyte Function |  |  | Kcnj10 | azure4 | 1 | 1.56302521008403 |
  | Serping1-mRNA | -0.415 | 0.317 | -1.04 | 0.205 | 0.75 | 0.488 | 1.15 | 0.208 | 1 | lm.nb | Astrocyte Function | NS\_MM\_NEUROINFLAM\_V1.0 | Serping1 | mRNA | 0 |  | Astrocyte Function | mmu04610;mmu05133 |  | Serping1 | azure4 | 1 | -1.30914826498423 |
  | Gja1-mRNA | -0.112 | 0.0888 | -0.286 | 0.0619 | 0.925 | 0.82 | 1.04 | 0.225 | 1 | loglinear | Astrocyte Function, Neurons and Neurotransmission | NS\_MM\_NEUROINFLAM\_V1.0 | Gja1 | mRNA | 0 |  | Neurons and Neurotransmission;Astrocyte Function | mmu04540;mmu05412 |  | Gja1 | azure4 | 1 | -1.26126126126126 |
  | Apoe-mRNA | 0.1 | 0.0826 | -0.0615 | 0.262 | 1.07 | 0.958 | 1.2 | 0.242 | 1 | loglinear | Astrocyte Function, Cellular Stress, Lipid Metabolism, Microglia Function, Neurons and Neurotransmission | NS\_MM\_NEUROINFLAM\_V1.0 | Apoe | mRNA | 0 |  | Neurons and Neurotransmission;Microglia Function;Lipid Metabolism;Cellular Stress;Astrocyte Function | mmu05010 |  | Apoe | azure4 | 1 | 1.21065375302663 |

  ##### DE Results - Astrocyte Function genes - DPI: 1 vs.CTRL

  Table displaying each sample's global significance scores and directed global significance scores as defined in the heatmaps above. The global significance score is calculated as the square root of the mean squared t-statistic for the genes in a gene set, with t-statistics coming from the linear regression underlying our differential expression analysis. The directed global significance score is calculated as the square root of the mean signed squared t-statistic for the genes in a gene set, with t-statistics coming from the linear regression underlying our differential expression analysis.

- **Volcano Plot: DPI: 2 vs.CTRL  
  More Plot Information**


  ##### Volcano Plot: DPI: 2 vs.CTRL

  Volcano plot displaying each gene's -log10(p-value) and log2 fold change for the selected covariate. Highly statistically significant genes fall at the top of the plot, and highly differentially expressed genes fall to either side. Genes within the selected gene set are highlighted in orange. Horizontal lines indicate various False Discovery Rate (FDR) thresholds.
- **DE Results - Astrocyte Function genes - DPI: 2 vs.CTRL  
  More Plot Information  Download CSV Data**

  | Probe Label | Log2 fold change | std error (log2) | Lower confidence limit (log2) | Upper confidence limit (log2) | Linear fold change | Lower confidence limit (linear) | Upper confidence limit (linear) | P-value | BY.p.value | method | Gene.sets | Codeset.Name | Probe.Label | Analyte.Type | Is.Control | Control.Type | Probe.Annotation | KEGG.Pathways | Cell.Type | Official.Gene.Name | volcanocol | volcanopch | tstats.all |
  | --- | --- | --- | --- | --- | --- | --- | --- | --- | --- | --- | --- | --- | --- | --- | --- | --- | --- | --- | --- | --- | --- | --- | --- |
  | Cp-mRNA | 1.93 | 0.138 | 1.66 | 2.2 | 3.82 | 3.17 | 4.6 | 2.03e-10 | 6.02e-07 | loglinear | Astrocyte Function | NS\_MM\_NEUROINFLAM\_V1.0 | Cp | mRNA | 0 |  | Astrocyte Function |  |  | Cp | slateblue1 | 16 | 13.9855072463768 |
  | Lcn2-mRNA | 9.71 | 0.723 | 8.3 | 11.1 | 839 | 314 | 2240 | 3.93e-10 | 6.02e-07 | lm.nb | Astrocyte Function, Inflammatory Signaling | NS\_MM\_NEUROINFLAM\_V1.0 | Lcn2 | mRNA | 0 |  | Inflammatory Signaling;Astrocyte Function |  |  | Lcn2 | slateblue1 | 16 | 13.4301521438451 |
  | Fkbp5-mRNA | 1.92 | 0.159 | 1.61 | 2.23 | 3.78 | 3.05 | 4.69 | 1.9e-09 | 1.12e-06 | lm.nb | Astrocyte Function | NS\_MM\_NEUROINFLAM\_V1.0 | Fkbp5 | mRNA | 0 |  | Astrocyte Function | mmu04915 |  | Fkbp5 | slateblue1 | 16 | 12.0754716981132 |
  | Amigo2-mRNA | 1.08 | 0.131 | 0.826 | 1.34 | 2.12 | 1.77 | 2.53 | 3.65e-07 | 9.21e-05 | lm.nb | Astrocyte Function | NS\_MM\_NEUROINFLAM\_V1.0 | Amigo2 | mRNA | 0 |  | Astrocyte Function |  |  | Amigo2 | slateblue1 | 16 | 8.24427480916031 |
  | Padi2-mRNA | -1.41 | 0.175 | -1.75 | -1.06 | 0.377 | 0.298 | 0.479 | 5.19e-07 | 0.000108 | lm.nb | Astrocyte Function, Epigenetic Regulation | NS\_MM\_NEUROINFLAM\_V1.0 | Padi2 | mRNA | 0 |  | Epigenetic Regulation;Astrocyte Function |  |  | Padi2 | slateblue1 | 16 | -8.05714285714286 |
  | Psmb8-mRNA | 2.23 | 0.352 | 1.54 | 2.92 | 4.7 | 2.91 | 7.59 | 9.89e-06 | 0.000997 | lm.nb | Adaptive Immune Response, Angiogenesis, Apoptosis, Astrocyte Function, Cell Cycle, Cytokine Signaling, Growth Factor Signaling, Inflammatory Signaling, Insulin Signaling, Microglia Function, NF-kB, Wnt | NS\_MM\_NEUROINFLAM\_V1.0 | Psmb8 | mRNA | 0 |  | Wnt;NF-kB;Microglia Function;Insulin Signaling;Inflammatory Signaling;Growth Factor Signaling;Cytokine Signaling;Cell Cycle;Astrocyte Function;Apoptosis;Angiogenesis;Adaptive Immune Response |  |  | Psmb8 | slateblue1 | 16 | 6.33522727272727 |
  | Aldh1l1-mRNA | 0.702 | 0.116 | 0.474 | 0.93 | 1.63 | 1.39 | 1.91 | 1.76e-05 | 0.00148 | loglinear | Astrocyte Function | NS\_MM\_NEUROINFLAM\_V1.0 | Aldh1l1 | mRNA | 0 |  | Astrocyte Function |  | Astrocytes | Aldh1l1 | slateblue1 | 16 | 6.05172413793103 |
  | Gbp2-mRNA | 4.37 | 0.741 | 2.91 | 5.82 | 20.6 | 7.53 | 56.5 | 2.29e-05 | 0.00179 | lm.nb | Astrocyte Function, Inflammatory Signaling | NS\_MM\_NEUROINFLAM\_V1.0 | Gbp2 | mRNA | 0 |  | Inflammatory Signaling;Astrocyte Function |  |  | Gbp2 | slateblue1 | 16 | 5.8974358974359 |
  | Apc-mRNA | -0.322 | 0.0593 | -0.438 | -0.206 | 0.8 | 0.738 | 0.867 | 5.61e-05 | 0.00362 | loglinear | Apoptosis, Astrocyte Function, Wnt | NS\_MM\_NEUROINFLAM\_V1.0 | Apc | mRNA | 0 |  | Wnt;Astrocyte Function;Apoptosis | mmu04310;mmu04390;mmu04550;mmu04810;mmu05166;mmu05200;mmu05206;mmu05210;mmu05213 |  | Apc | slateblue1 | 16 | -5.43001686340641 |
  | Agt-mRNA | 1.56 | 0.296 | 0.984 | 2.14 | 2.96 | 1.98 | 4.42 | 7.42e-05 | 0.00439 | lm.nb | Astrocyte Function | NS\_MM\_NEUROINFLAM\_V1.0 | Agt | mRNA | 0 |  | Astrocyte Function | mmu04924 |  | Agt | slateblue1 | 16 | 5.27027027027027 |
  | Apoe-mRNA | 0.418 | 0.0826 | 0.256 | 0.58 | 1.34 | 1.19 | 1.49 | 0.000116 | 0.00609 | loglinear | Astrocyte Function, Cellular Stress, Lipid Metabolism, Microglia Function, Neurons and Neurotransmission | NS\_MM\_NEUROINFLAM\_V1.0 | Apoe | mRNA | 0 |  | Neurons and Neurotransmission;Microglia Function;Lipid Metabolism;Cellular Stress;Astrocyte Function | mmu05010 |  | Apoe | slateblue1 | 16 | 5.06053268765133 |
  | C4a-mRNA | 0.865 | 0.21 | 0.453 | 1.28 | 1.82 | 1.37 | 2.42 | 0.000805 | 0.0237 | lm.nb | Astrocyte Function | NS\_MM\_NEUROINFLAM\_V1.0 | C4a | mRNA | 0 |  | Astrocyte Function |  |  | C4a | slateblue2 | 16 | 4.11904761904762 |
  | Nwd1-mRNA | -0.5 | 0.134 | -0.763 | -0.237 | 0.707 | 0.589 | 0.849 | 0.00184 | 0.0417 | loglinear | Astrocyte Function | NS\_MM\_NEUROINFLAM\_V1.0 | Nwd1 | mRNA | 0 |  | Astrocyte Function |  | Astrocytes | Nwd1 | slateblue2 | 16 | -3.73134328358209 |
  | Grm3-mRNA | -0.327 | 0.0923 | -0.508 | -0.146 | 0.797 | 0.703 | 0.904 | 0.00272 | 0.056 | loglinear | Astrocyte Function, Neurons and Neurotransmission | NS\_MM\_NEUROINFLAM\_V1.0 | Grm3 | mRNA | 0 |  | Neurons and Neurotransmission;Astrocyte Function | mmu04072;mmu04080;mmu04724;mmu05030 |  | Grm3 | slateblue3 | 16 | -3.54279523293608 |
  | Tgfa-mRNA | -0.437 | 0.134 | -0.7 | -0.173 | 0.739 | 0.616 | 0.887 | 0.00503 | 0.0954 | loglinear | Astrocyte Function, Microglia Function, Neurons and Neurotransmission | NS\_MM\_NEUROINFLAM\_V1.0 | Tgfa | mRNA | 0 |  | Neurons and Neurotransmission;Microglia Function;Astrocyte Function | mmu04012;mmu05200;mmu05211;mmu05212;mmu05214;mmu05215;mmu05223 |  | Tgfa | slateblue3 | 16 | -3.26119402985075 |
  | Vim-mRNA | -0.919 | 0.292 | -1.49 | -0.346 | 0.529 | 0.356 | 0.787 | 0.00626 | 0.114 | lm.nb | Apoptosis, Astrocyte Function | NS\_MM\_NEUROINFLAM\_V1.0 | Vim | mRNA | 0 |  | Astrocyte Function;Apoptosis | mmu05169;mmu05206 |  | Vim | slateblue4 | 16 | -3.1472602739726 |
  | Hspb1-mRNA | 0.891 | 0.295 | 0.314 | 1.47 | 1.85 | 1.24 | 2.77 | 0.00806 | 0.137 | lm.nb | Angiogenesis, Astrocyte Function, Cellular Stress, Growth Factor Signaling | NS\_MM\_NEUROINFLAM\_V1.0 | Hspb1 | mRNA | 0 |  | Growth Factor Signaling;Cellular Stress;Astrocyte Function;Angiogenesis | mmu04010;mmu04370;mmu05146;mmu05169 |  | Hspb1 | slateblue4 | 16 | 3.02033898305085 |
  | Fbln5-mRNA | 1.14 | 0.377 | 0.399 | 1.88 | 2.2 | 1.32 | 3.67 | 0.00816 | 0.137 | lm.nb | Astrocyte Function, Matrix Remodeling | NS\_MM\_NEUROINFLAM\_V1.0 | Fbln5 | mRNA | 0 |  | Matrix Remodeling;Astrocyte Function |  |  | Fbln5 | slateblue4 | 16 | 3.02387267904509 |
  | Sox9-mRNA | -0.342 | 0.128 | -0.593 | -0.0906 | 0.789 | 0.663 | 0.939 | 0.0169 | 0.241 | lm.nb | Astrocyte Function, Wnt | NS\_MM\_NEUROINFLAM\_V1.0 | Sox9 | mRNA | 0 |  | Wnt;Astrocyte Function | mmu04024 | Astrocytes | Sox9 | slateblue4 | 16 | -2.671875 |
  | Entpd2-mRNA | 1.47 | 0.552 | 0.387 | 2.55 | 2.77 | 1.31 | 5.86 | 0.0171 | 0.243 | lm.nb | Astrocyte Function | NS\_MM\_NEUROINFLAM\_V1.0 | Entpd2 | mRNA | 0 |  | Astrocyte Function | mmu00230 | Astrocytes | Entpd2 | slateblue4 | 16 | 2.66304347826087 |

  ##### DE Results - Astrocyte Function genes - DPI: 2 vs.CTRL

  Table displaying each sample's global significance scores and directed global significance scores as defined in the heatmaps above. The global significance score is calculated as the square root of the mean squared t-statistic for the genes in a gene set, with t-statistics coming from the linear regression underlying our differential expression analysis. The directed global significance score is calculated as the square root of the mean signed squared t-statistic for the genes in a gene set, with t-statistics coming from the linear regression underlying our differential expression analysis.

- **Volcano Plot: DPI: 3 vs.CTRL  
  More Plot Information**


  ##### Volcano Plot: DPI: 3 vs.CTRL

  Volcano plot displaying each gene's -log10(p-value) and log2 fold change for the selected covariate. Highly statistically significant genes fall at the top of the plot, and highly differentially expressed genes fall to either side. Genes within the selected gene set are highlighted in orange. Horizontal lines indicate various False Discovery Rate (FDR) thresholds.
- **DE Results - Astrocyte Function genes - DPI: 3 vs.CTRL  
  More Plot Information  Download CSV Data**

  | Probe Label | Log2 fold change | std error (log2) | Lower confidence limit (log2) | Upper confidence limit (log2) | Linear fold change | Lower confidence limit (linear) | Upper confidence limit (linear) | P-value | BY.p.value | method | Gene.sets | Codeset.Name | Probe.Label | Analyte.Type | Is.Control | Control.Type | Probe.Annotation | KEGG.Pathways | Cell.Type | Official.Gene.Name | volcanocol | volcanopch | tstats.all |
  | --- | --- | --- | --- | --- | --- | --- | --- | --- | --- | --- | --- | --- | --- | --- | --- | --- | --- | --- | --- | --- | --- | --- | --- |
  | Cp-mRNA | 2.57 | 0.138 | 2.3 | 2.84 | 5.94 | 4.93 | 7.16 | 2.74e-12 | 7.36e-09 | loglinear | Astrocyte Function | NS\_MM\_NEUROINFLAM\_V1.0 | Cp | mRNA | 0 |  | Astrocyte Function |  |  | Cp | slateblue1 | 16 | 18.6231884057971 |
  | Lcn2-mRNA | 11 | 0.723 | 9.57 | 12.4 | 2030 | 759 | 5410 | 6.27e-11 | 5.53e-08 | lm.nb | Astrocyte Function, Inflammatory Signaling | NS\_MM\_NEUROINFLAM\_V1.0 | Lcn2 | mRNA | 0 |  | Inflammatory Signaling;Astrocyte Function |  |  | Lcn2 | slateblue1 | 16 | 15.2143845089903 |
  | Fkbp5-mRNA | 2.17 | 0.158 | 1.86 | 2.47 | 4.48 | 3.62 | 5.56 | 2.94e-10 | 2.07e-07 | lm.nb | Astrocyte Function | NS\_MM\_NEUROINFLAM\_V1.0 | Fkbp5 | mRNA | 0 |  | Astrocyte Function | mmu04915 |  | Fkbp5 | slateblue1 | 16 | 13.7341772151899 |
  | Psmb8-mRNA | 2.99 | 0.346 | 2.31 | 3.67 | 7.95 | 4.97 | 12.7 | 1.99e-07 | 3.2e-05 | lm.nb | Adaptive Immune Response, Angiogenesis, Apoptosis, Astrocyte Function, Cell Cycle, Cytokine Signaling, Growth Factor Signaling, Inflammatory Signaling, Insulin Signaling, Microglia Function, NF-kB, Wnt | NS\_MM\_NEUROINFLAM\_V1.0 | Psmb8 | mRNA | 0 |  | Wnt;NF-kB;Microglia Function;Insulin Signaling;Inflammatory Signaling;Growth Factor Signaling;Cytokine Signaling;Cell Cycle;Astrocyte Function;Apoptosis;Angiogenesis;Adaptive Immune Response |  |  | Psmb8 | slateblue1 | 16 | 8.64161849710983 |
  | Padi2-mRNA | -1.5 | 0.177 | -1.85 | -1.15 | 0.353 | 0.278 | 0.449 | 2.54e-07 | 3.57e-05 | lm.nb | Astrocyte Function, Epigenetic Regulation | NS\_MM\_NEUROINFLAM\_V1.0 | Padi2 | mRNA | 0 |  | Epigenetic Regulation;Astrocyte Function |  |  | Padi2 | slateblue1 | 16 | -8.47457627118644 |
  | Ptgs2-mRNA | 1.88 | 0.236 | 1.41 | 2.34 | 3.67 | 2.67 | 5.06 | 5.87e-07 | 7.14e-05 | lm.nb | Astrocyte Function, Cellular Stress, Innate Immune Response, Lipid Metabolism, Microglia Function, NF-kB | NS\_MM\_NEUROINFLAM\_V1.0 | Ptgs2 | mRNA | 0 |  | NF-kB;Microglia Function;Lipid Metabolism;Innate Immune Response;Cellular Stress;Astrocyte Function | mmu00590;mmu04064;mmu04370;mmu04668;mmu04723;mmu04726;mmu04913;mmu04921;mmu04923;mmu05140;mmu05200;mmu05206;mmu05222 |  | Ptgs2 | slateblue1 | 16 | 7.96610169491525 |
  | Amigo2-mRNA | 1.02 | 0.132 | 0.76 | 1.28 | 2.02 | 1.69 | 2.42 | 8.55e-07 | 9.43e-05 | lm.nb | Astrocyte Function | NS\_MM\_NEUROINFLAM\_V1.0 | Amigo2 | mRNA | 0 |  | Astrocyte Function |  |  | Amigo2 | slateblue1 | 16 | 7.72727272727273 |
  | Gbp2-mRNA | 5.64 | 0.736 | 4.19 | 7.08 | 49.7 | 18.3 | 135 | 9.8e-07 | 0.000105 | lm.nb | Astrocyte Function, Inflammatory Signaling | NS\_MM\_NEUROINFLAM\_V1.0 | Gbp2 | mRNA | 0 |  | Inflammatory Signaling;Astrocyte Function |  |  | Gbp2 | slateblue1 | 16 | 7.66304347826087 |
  | Srgn-mRNA | 2.18 | 0.297 | 1.59 | 2.76 | 4.52 | 3.02 | 6.77 | 1.72e-06 | 0.000164 | lm.nb | Astrocyte Function, Inflammatory Signaling | NS\_MM\_NEUROINFLAM\_V1.0 | Srgn | mRNA | 0 |  | Inflammatory Signaling;Astrocyte Function |  |  | Srgn | slateblue1 | 16 | 7.34006734006734 |
  | Agt-mRNA | 2.14 | 0.294 | 1.56 | 2.72 | 4.41 | 2.96 | 6.57 | 1.85e-06 | 0.000172 | lm.nb | Astrocyte Function | NS\_MM\_NEUROINFLAM\_V1.0 | Agt | mRNA | 0 |  | Astrocyte Function | mmu04924 |  | Agt | slateblue1 | 16 | 7.27891156462585 |
  | Aldh1l1-mRNA | 0.812 | 0.116 | 0.584 | 1.04 | 1.76 | 1.5 | 2.06 | 3.15e-06 | 0.000271 | loglinear | Astrocyte Function | NS\_MM\_NEUROINFLAM\_V1.0 | Aldh1l1 | mRNA | 0 |  | Astrocyte Function |  | Astrocytes | Aldh1l1 | slateblue1 | 16 | 7 |
  | Fbln5-mRNA | 2.32 | 0.359 | 1.62 | 3.03 | 5 | 3.07 | 8.14 | 7.74e-06 | 0.000535 | lm.nb | Astrocyte Function, Matrix Remodeling | NS\_MM\_NEUROINFLAM\_V1.0 | Fbln5 | mRNA | 0 |  | Matrix Remodeling;Astrocyte Function |  |  | Fbln5 | slateblue1 | 16 | 6.46239554317549 |
  | Grm3-mRNA | -0.553 | 0.0923 | -0.734 | -0.372 | 0.682 | 0.601 | 0.773 | 1.89e-05 | 0.00111 | loglinear | Astrocyte Function, Neurons and Neurotransmission | NS\_MM\_NEUROINFLAM\_V1.0 | Grm3 | mRNA | 0 |  | Neurons and Neurotransmission;Astrocyte Function | mmu04072;mmu04080;mmu04724;mmu05030 |  | Grm3 | slateblue1 | 16 | -5.99133261105092 |
  | C4a-mRNA | 1.23 | 0.207 | 0.82 | 1.63 | 2.34 | 1.77 | 3.1 | 2.14e-05 | 0.00124 | lm.nb | Astrocyte Function | NS\_MM\_NEUROINFLAM\_V1.0 | C4a | mRNA | 0 |  | Astrocyte Function |  |  | C4a | slateblue1 | 16 | 5.94202898550725 |
  | Tgfa-mRNA | -0.731 | 0.134 | -0.994 | -0.467 | 0.603 | 0.502 | 0.723 | 5.46e-05 | 0.00254 | loglinear | Astrocyte Function, Microglia Function, Neurons and Neurotransmission | NS\_MM\_NEUROINFLAM\_V1.0 | Tgfa | mRNA | 0 |  | Neurons and Neurotransmission;Microglia Function;Astrocyte Function | mmu04012;mmu05200;mmu05211;mmu05212;mmu05214;mmu05215;mmu05223 |  | Tgfa | slateblue1 | 16 | -5.45522388059701 |
  | Nwd1-mRNA | -0.714 | 0.134 | -0.977 | -0.452 | 0.609 | 0.508 | 0.731 | 6.83e-05 | 0.00298 | loglinear | Astrocyte Function | NS\_MM\_NEUROINFLAM\_V1.0 | Nwd1 | mRNA | 0 |  | Astrocyte Function |  | Astrocytes | Nwd1 | slateblue1 | 16 | -5.32835820895522 |
  | B3gnt5-mRNA | 1.4 | 0.298 | 0.818 | 1.99 | 2.64 | 1.76 | 3.96 | 0.000237 | 0.0089 | lm.nb | Astrocyte Function | NS\_MM\_NEUROINFLAM\_V1.0 | B3gnt5 | mRNA | 0 |  | Astrocyte Function |  |  | B3gnt5 | slateblue1 | 16 | 4.69798657718121 |
  | Hspb1-mRNA | 1.36 | 0.29 | 0.795 | 1.93 | 2.57 | 1.73 | 3.82 | 0.000241 | 0.00894 | lm.nb | Angiogenesis, Astrocyte Function, Cellular Stress, Growth Factor Signaling | NS\_MM\_NEUROINFLAM\_V1.0 | Hspb1 | mRNA | 0 |  | Growth Factor Signaling;Cellular Stress;Astrocyte Function;Angiogenesis | mmu04010;mmu04370;mmu05146;mmu05169 |  | Hspb1 | slateblue1 | 16 | 4.68965517241379 |
  | Serpina3n-mRNA | 2.04 | 0.467 | 1.13 | 2.96 | 4.12 | 2.19 | 7.78 | 0.000472 | 0.015 | lm.nb | Astrocyte Function | NS\_MM\_NEUROINFLAM\_V1.0 | Serpina3n | mRNA | 0 |  | Astrocyte Function |  |  | Serpina3n | slateblue2 | 16 | 4.36830835117773 |
  | Slc6a1-mRNA | 0.477 | 0.118 | 0.245 | 0.709 | 1.39 | 1.18 | 1.63 | 0.000971 | 0.0274 | lm.nb | Astrocyte Function, Neurons and Neurotransmission | NS\_MM\_NEUROINFLAM\_V1.0 | Slc6a1 | mRNA | 0 |  | Neurons and Neurotransmission;Astrocyte Function |  |  | Slc6a1 | slateblue2 | 16 | 4.04237288135593 |

  ##### DE Results - Astrocyte Function genes - DPI: 3 vs.CTRL

  Table displaying each sample's global significance scores and directed global significance scores as defined in the heatmaps above. The global significance score is calculated as the square root of the mean squared t-statistic for the genes in a gene set, with t-statistics coming from the linear regression underlying our differential expression analysis. The directed global significance score is calculated as the square root of the mean signed squared t-statistic for the genes in a gene set, with t-statistics coming from the linear regression underlying our differential expression analysis.

DPI:
- DPI: differential expression in 1 vs. baseline of CTRL
- DPI: differential expression in 2 vs. baseline of CTRL
- DPI: differential expression in 3 vs. baseline of CTRL

- **Volcano Plot: DPI: 1 vs.CTRL  
  More Plot Information**


  ##### Volcano Plot: DPI: 1 vs.CTRL

  Volcano plot displaying each gene's -log10(p-value) and log2 fold change for the selected covariate. Highly statistically significant genes fall at the top of the plot, and highly differentially expressed genes fall to either side. Genes within the selected gene set are highlighted in orange. Horizontal lines indicate various False Discovery Rate (FDR) thresholds.
- **DE Results - Autophagy genes - DPI: 1 vs.CTRL  
  More Plot Information  Download CSV Data**

  | Probe Label | Log2 fold change | std error (log2) | Lower confidence limit (log2) | Upper confidence limit (log2) | Linear fold change | Lower confidence limit (linear) | Upper confidence limit (linear) | P-value | BY.p.value | method | Gene.sets | Codeset.Name | Probe.Label | Analyte.Type | Is.Control | Control.Type | Probe.Annotation | KEGG.Pathways | Cell.Type | Official.Gene.Name | volcanocol | volcanopch | tstats.all |
  | --- | --- | --- | --- | --- | --- | --- | --- | --- | --- | --- | --- | --- | --- | --- | --- | --- | --- | --- | --- | --- | --- | --- | --- |
  | Prkacb-mRNA | -0.327 | 0.0629 | -0.451 | -0.204 | 0.797 | 0.732 | 0.868 | 8.65e-05 | 0.0321 | loglinear | Adaptive Immune Response, Angiogenesis, Apoptosis, Autophagy, Growth Factor Signaling, Lipid Metabolism, Neurons and Neurotransmission, Wnt | NS\_MM\_NEUROINFLAM\_V1.0 | Prkacb | mRNA | 0 |  | Wnt;Neurons and Neurotransmission;Lipid Metabolism;Growth Factor Signaling;Autophagy;Apoptosis;Angiogenesis;Adaptive Immune Response | mmu04010;mmu04014;mmu04020;mmu04024;mmu04062;mmu04114;mmu04210;mmu04211;mmu04213;mmu04261;mmu04270;mmu04310;mmu04540;mmu04611;mmu04713;mmu04720;mmu04723;mmu04724;mmu04725;mmu04726;mmu04728;mmu04750;mmu04910;mmu04911;mmu04912;mmu04913;mmu04914;mmu04915;mmu04916;mmu04919;mmu04921;mmu04922;mmu04923;mmu04924;mmu04925;mmu05012;mmu05020;mmu05030;mmu05031;mmu05146;mmu05166;mmu05169;mmu05200;mmu05203;mmu05205;mmu05414 |  | Prkacb | slateblue2 | 16 | -5.19872813990461 |
  | Prkce-mRNA | -0.427 | 0.0999 | -0.623 | -0.232 | 0.744 | 0.649 | 0.852 | 0.000577 | 0.0754 | loglinear | Autophagy, Growth Factor Signaling | NS\_MM\_NEUROINFLAM\_V1.0 | Prkce | mRNA | 0 |  | Growth Factor Signaling;Autophagy | mmu04022;mmu04071;mmu04270;mmu04530;mmu04666;mmu04750;mmu04925;mmu04930;mmu04931;mmu04933;mmu05206 |  | Prkce | slateblue3 | 16 | -4.27427427427427 |
  | Tgm2-mRNA | -2.9 | 0.693 | -4.26 | -1.54 | 0.134 | 0.0523 | 0.344 | 0.000708 | 0.0806 | lm.nb | Autophagy, Microglia Function | NS\_MM\_NEUROINFLAM\_V1.0 | Tgm2 | mRNA | 0 |  | Microglia Function;Autophagy | mmu05016 |  | Tgm2 | slateblue3 | 16 | -4.18470418470419 |
  | Pten-mRNA | -0.296 | 0.0753 | -0.444 | -0.148 | 0.815 | 0.735 | 0.902 | 0.0012 | 0.109 | loglinear | Adaptive Immune Response, Autophagy, DNA Damage, Growth Factor Signaling, Lipid Metabolism | NS\_MM\_NEUROINFLAM\_V1.0 | Pten | mRNA | 0 |  | Lipid Metabolism;Growth Factor Signaling;DNA Damage;Autophagy;Adaptive Immune Response | mmu00562;mmu04068;mmu04070;mmu04071;mmu04115;mmu04150;mmu04151;mmu04510;mmu04530;mmu04931;mmu05161;mmu05200;mmu05206;mmu05213;mmu05214;mmu05215;mmu05218;mmu05222;mmu05230 |  | Pten | slateblue4 | 16 | -3.93094289508632 |
  | Mapk10-mRNA | -0.539 | 0.138 | -0.81 | -0.269 | 0.688 | 0.57 | 0.83 | 0.00126 | 0.111 | lm.nb | Adaptive Immune Response, Apoptosis, Autophagy, Cellular Stress, Growth Factor Signaling, Innate Immune Response, Wnt | NS\_MM\_NEUROINFLAM\_V1.0 | Mapk10 | mRNA | 0 |  | Wnt;Innate Immune Response;Growth Factor Signaling;Cellular Stress;Autophagy;Apoptosis;Adaptive Immune Response | mmu04010;mmu04012;mmu04014;mmu04024;mmu04068;mmu04071;mmu04141;mmu04310;mmu04380;mmu04510;mmu04620;mmu04621;mmu04622;mmu04664;mmu04668;mmu04722;mmu04723;mmu04728;mmu04750;mmu04910;mmu04912;mmu04914;mmu04917;mmu04920;mmu04930;mmu04931;mmu04932;mmu04933;mmu05132;mmu05133;mmu05142;mmu05145;mmu05152;mmu05160;mmu05161;mmu05164;mmu05168;mmu05169;mmu05200;mmu05210;mmu05212;mmu05231 |  | Mapk10 | slateblue4 | 16 | -3.90579710144928 |
  | Rac1-mRNA | -0.928 | 0.256 | -1.43 | -0.426 | 0.525 | 0.371 | 0.744 | 0.00229 | 0.149 | lm.nb | Adaptive Immune Response, Angiogenesis, Autophagy, Growth Factor Signaling, Innate Immune Response, Neurons and Neurotransmission, Wnt | NS\_MM\_NEUROINFLAM\_V1.0 | Rac1 | mRNA | 0 |  | Wnt;Neurons and Neurotransmission;Innate Immune Response;Growth Factor Signaling;Autophagy;Angiogenesis;Adaptive Immune Response | mmu04010;mmu04014;mmu04015;mmu04024;mmu04062;mmu04071;mmu04145;mmu04151;mmu04310;mmu04360;mmu04370;mmu04380;mmu04510;mmu04520;mmu04620;mmu04650;mmu04662;mmu04664;mmu04666;mmu04670;mmu04722;mmu04810;mmu04932;mmu04933;mmu05014;mmu05100;mmu05132;mmu05200;mmu05203;mmu05205;mmu05210;mmu05211;mmu05212;mmu05231;mmu05416 |  | Rac1 | slateblue4 | 16 | -3.625 |
  | Rapgef3-mRNA | -0.593 | 0.164 | -0.915 | -0.27 | 0.663 | 0.53 | 0.829 | 0.00238 | 0.15 | lm.nb | Adaptive Immune Response, Autophagy | NS\_MM\_NEUROINFLAM\_V1.0 | Rapgef3 | mRNA | 0 |  | Autophagy;Adaptive Immune Response | mmu04015;mmu04024;mmu04072;mmu04261;mmu04670;mmu04720;mmu04726 |  | Rapgef3 | slateblue4 | 16 | -3.61585365853659 |
  | Bnip3l-mRNA | -0.61 | 0.172 | -0.947 | -0.273 | 0.655 | 0.519 | 0.828 | 0.00267 | 0.163 | lm.nb | Apoptosis, Autophagy | NS\_MM\_NEUROINFLAM\_V1.0 | Bnip3l | mRNA | 0 |  | Autophagy;Apoptosis |  |  | Bnip3l | slateblue4 | 16 | -3.54651162790698 |
  | Rhoa-mRNA | -0.902 | 0.261 | -1.41 | -0.391 | 0.535 | 0.375 | 0.762 | 0.00322 | 0.18 | lm.nb | Angiogenesis, Autophagy, Growth Factor Signaling, Wnt | NS\_MM\_NEUROINFLAM\_V1.0 | Rhoa | mRNA | 0 |  | Wnt;Growth Factor Signaling;Autophagy;Angiogenesis | mmu04014;mmu04015;mmu04022;mmu04024;mmu04062;mmu04071;mmu04072;mmu04144;mmu04270;mmu04310;mmu04350;mmu04360;mmu04510;mmu04520;mmu04530;mmu04611;mmu04660;mmu04670;mmu04722;mmu04810;mmu04921;mmu05100;mmu05133;mmu05152;mmu05200;mmu05203;mmu05205;mmu05206;mmu05210 |  | Rhoa | slateblue4 | 16 | -3.45593869731801 |
  | Igf1r-mRNA | 0.523 | 0.156 | 0.216 | 0.83 | 1.44 | 1.16 | 1.78 | 0.00414 | 0.197 | lm.nb | Apoptosis, Autophagy, Carbohydrate Metabolism, Growth Factor Signaling, Insulin Signaling | NS\_MM\_NEUROINFLAM\_V1.0 | Igf1r | mRNA | 0 |  | Insulin Signaling;Growth Factor Signaling;Carbohydrate Metabolism;Autophagy;Apoptosis | mmu04014;mmu04015;mmu04066;mmu04068;mmu04114;mmu04144;mmu04151;mmu04152;mmu04211;mmu04213;mmu04510;mmu04520;mmu04550;mmu04730;mmu04913;mmu04914;mmu05200;mmu05202;mmu05205;mmu05214;mmu05215;mmu05218 |  | Igf1r | slateblue4 | 16 | 3.3525641025641 |
  | Traf6-mRNA | 0.546 | 0.169 | 0.215 | 0.877 | 1.46 | 1.16 | 1.84 | 0.00523 | 0.215 | lm.nb | Adaptive Immune Response, Apoptosis, Autophagy, Cytokine Signaling, Growth Factor Signaling, Innate Immune Response, NF-kB | NS\_MM\_NEUROINFLAM\_V1.0 | Traf6 | mRNA | 0 |  | NF-kB;Innate Immune Response;Growth Factor Signaling;Cytokine Signaling;Autophagy;Apoptosis;Adaptive Immune Response | mmu04010;mmu04064;mmu04120;mmu04144;mmu04380;mmu04620;mmu04621;mmu04622;mmu04722;mmu05133;mmu05140;mmu05142;mmu05145;mmu05152;mmu05160;mmu05162;mmu05168;mmu05169;mmu05200;mmu05222 |  | Traf6 | slateblue4 | 16 | 3.23076923076923 |
  | Map2k1-mRNA | -1.03 | 0.32 | -1.65 | -0.399 | 0.491 | 0.318 | 0.759 | 0.00553 | 0.224 | lm.nb | Adaptive Immune Response, Angiogenesis, Autophagy, Cytokine Signaling, Growth Factor Signaling, Innate Immune Response, Insulin Signaling, Microglia Function | NS\_MM\_NEUROINFLAM\_V1.0 | Map2k1 | mRNA | 0 |  | Microglia Function;Insulin Signaling;Innate Immune Response;Growth Factor Signaling;Cytokine Signaling;Autophagy;Angiogenesis;Adaptive Immune Response | mmu04010;mmu04012;mmu04014;mmu04015;mmu04022;mmu04024;mmu04062;mmu04066;mmu04068;mmu04071;mmu04072;mmu04114;mmu04151;mmu04270;mmu04370;mmu04380;mmu04510;mmu04540;mmu04550;mmu04620;mmu04650;mmu04660;mmu04662;mmu04664;mmu04666;mmu04668;mmu04720;mmu04722;mmu04725;mmu04726;mmu04730;mmu04810;mmu04910;mmu04912;mmu04914;mmu04915;mmu04916;mmu04917;mmu04919;mmu04921;mmu05020;mmu05034;mmu05161;mmu05164;mmu05200;mmu05205;mmu05206;mmu05210;mmu05211;mmu05212;mmu05213;mmu05214;mmu05215;mmu05216;mmu05218;mmu05219;mmu05220;mmu05221;mmu05223;mmu05230;mmu05231 |  | Map2k1 | slateblue4 | 16 | -3.21875 |
  | Pink1-mRNA | -0.79 | 0.249 | -1.28 | -0.302 | 0.578 | 0.412 | 0.811 | 0.00592 | 0.235 | lm.nb | Autophagy, Cellular Stress | NS\_MM\_NEUROINFLAM\_V1.0 | Pink1 | mRNA | 0 |  | Cellular Stress;Autophagy | mmu05012 |  | Pink1 | slateblue4 | 16 | -3.17269076305221 |
  | Sqstm1-mRNA | -0.854 | 0.271 | -1.39 | -0.322 | 0.553 | 0.383 | 0.8 | 0.00626 | 0.244 | lm.nb | Autophagy, Cytokine Signaling, Growth Factor Signaling | NS\_MM\_NEUROINFLAM\_V1.0 | Sqstm1 | mRNA | 0 |  | Growth Factor Signaling;Cytokine Signaling;Autophagy | mmu04380 |  | Sqstm1 | slateblue4 | 16 | -3.15129151291513 |
  | Ambra1-mRNA | -0.503 | 0.165 | -0.826 | -0.179 | 0.706 | 0.564 | 0.883 | 0.00768 | 0.258 | lm.nb | Autophagy, Cellular Stress | NS\_MM\_NEUROINFLAM\_V1.0 | Ambra1 | mRNA | 0 |  | Cellular Stress;Autophagy |  |  | Ambra1 | slateblue4 | 16 | -3.04848484848485 |
  | Fcgr2b-mRNA | 0.656 | 0.218 | 0.228 | 1.08 | 1.58 | 1.17 | 2.12 | 0.00842 | 0.278 | lm.nb | Adaptive Immune Response, Autophagy | NS\_MM\_NEUROINFLAM\_V1.0 | Fcgr2b | mRNA | 0 |  | Autophagy;Adaptive Immune Response | mmu04145;mmu04380;mmu04662;mmu04666;mmu05150;mmu05152;mmu05162 |  | Fcgr2b | slateblue4 | 16 | 3.00917431192661 |
  | Mapk14-mRNA | -1.06 | 0.362 | -1.77 | -0.355 | 0.478 | 0.293 | 0.782 | 0.00961 | 0.3 | lm.nb | Angiogenesis, Autophagy, Cellular Stress, Growth Factor Signaling, Innate Immune Response | NS\_MM\_NEUROINFLAM\_V1.0 | Mapk14 | mRNA | 0 |  | Innate Immune Response;Growth Factor Signaling;Cellular Stress;Autophagy;Angiogenesis | mmu04010;mmu04015;mmu04068;mmu04071;mmu04261;mmu04370;mmu04380;mmu04550;mmu04611;mmu04620;mmu04621;mmu04622;mmu04660;mmu04664;mmu04668;mmu04670;mmu04722;mmu04723;mmu04728;mmu04750;mmu04912;mmu04914;mmu04917;mmu04933;mmu05014;mmu05132;mmu05133;mmu05140;mmu05142;mmu05145;mmu05152;mmu05160;mmu05164;mmu05169;mmu05205 |  | Mapk14 | slateblue4 | 16 | -2.92817679558011 |
  | Bad-mRNA | -0.533 | 0.188 | -0.901 | -0.164 | 0.691 | 0.535 | 0.892 | 0.012 | 0.351 | lm.nb | Adaptive Immune Response, Apoptosis, Autophagy, Cellular Stress, Growth Factor Signaling | NS\_MM\_NEUROINFLAM\_V1.0 | Bad | mRNA | 0 |  | Growth Factor Signaling;Cellular Stress;Autophagy;Apoptosis;Adaptive Immune Response | mmu04012;mmu04014;mmu04022;mmu04024;mmu04151;mmu04210;mmu04370;mmu04510;mmu04722;mmu04910;mmu04919;mmu05010;mmu05014;mmu05145;mmu05152;mmu05160;mmu05161;mmu05200;mmu05203;mmu05210;mmu05212;mmu05213;mmu05215;mmu05218;mmu05220;mmu05221;mmu05223 |  | Bad | slateblue4 | 16 | -2.83510638297872 |
  | Pik3r2-mRNA | -0.223 | 0.0793 | -0.378 | -0.0676 | 0.857 | 0.769 | 0.954 | 0.0125 | 0.357 | loglinear | Adaptive Immune Response, Angiogenesis, Apoptosis, Autophagy, Cytokine Signaling, Growth Factor Signaling, Innate Immune Response, Insulin Signaling | NS\_MM\_NEUROINFLAM\_V1.0 | Pik3r2 | mRNA | 0 |  | Insulin Signaling;Innate Immune Response;Growth Factor Signaling;Cytokine Signaling;Autophagy;Apoptosis;Angiogenesis;Adaptive Immune Response | mmu04012;mmu04014;mmu04015;mmu04022;mmu04024;mmu04062;mmu04066;mmu04068;mmu04070;mmu04071;mmu04072;mmu04150;mmu04151;mmu04152;mmu04210;mmu04211;mmu04213;mmu04261;mmu04370;mmu04380;mmu04510;mmu04550;mmu04611;mmu04620;mmu04630;mmu04650;mmu04660;mmu04662;mmu04664;mmu04666;mmu04668;mmu04670;mmu04722;mmu04725;mmu04750;mmu04810;mmu04910;mmu04914;mmu04915;mmu04917;mmu04919;mmu04921;mmu04923;mmu04930;mmu04931;mmu04932;mmu04933;mmu04960;mmu04973;mmu05100;mmu05142;mmu05145;mmu05146;mmu05160;mmu05161;mmu05162;mmu05164;mmu05166;mmu05169;mmu05200;mmu05203;mmu05205;mmu05210;mmu05211;mmu05212;mmu05213;mmu05214;mmu05215;mmu05218;mmu05220;mmu05221;mmu05222;mmu05223;mmu05230;mmu05231 |  | Pik3r2 | slateblue4 | 16 | -2.81210592686003 |
  | Pik3ca-mRNA | -0.287 | 0.107 | -0.497 | -0.0771 | 0.82 | 0.709 | 0.948 | 0.0164 | 0.417 | lm.nb | Adaptive Immune Response, Angiogenesis, Apoptosis, Autophagy, Carbohydrate Metabolism, Cytokine Signaling, Growth Factor Signaling, Innate Immune Response, Insulin Signaling, Lipid Metabolism | NS\_MM\_NEUROINFLAM\_V1.0 | Pik3ca | mRNA | 0 |  | Lipid Metabolism;Insulin Signaling;Innate Immune Response;Growth Factor Signaling;Cytokine Signaling;Carbohydrate Metabolism;Autophagy;Apoptosis;Angiogenesis;Adaptive Immune Response | mmu00562;mmu04012;mmu04014;mmu04015;mmu04022;mmu04024;mmu04062;mmu04066;mmu04068;mmu04070;mmu04071;mmu04072;mmu04150;mmu04151;mmu04152;mmu04210;mmu04211;mmu04213;mmu04261;mmu04370;mmu04380;mmu04510;mmu04550;mmu04611;mmu04620;mmu04630;mmu04650;mmu04660;mmu04662;mmu04664;mmu04666;mmu04668;mmu04670;mmu04722;mmu04725;mmu04750;mmu04810;mmu04910;mmu04914;mmu04915;mmu04917;mmu04919;mmu04921;mmu04923;mmu04930;mmu04931;mmu04932;mmu04933;mmu04960;mmu04973;mmu05100;mmu05142;mmu05145;mmu05146;mmu05160;mmu05161;mmu05162;mmu05164;mmu05166;mmu05169;mmu05200;mmu05203;mmu05205;mmu05210;mmu05211;mmu05212;mmu05213;mmu05214;mmu05215;mmu05218;mmu05220;mmu05221;mmu05222;mmu05223;mmu05230;mmu05231 |  | Pik3ca | slateblue4 | 16 | -2.68224299065421 |

  ##### DE Results - Autophagy genes - DPI: 1 vs.CTRL

  Table displaying each sample's global significance scores and directed global significance scores as defined in the heatmaps above. The global significance score is calculated as the square root of the mean squared t-statistic for the genes in a gene set, with t-statistics coming from the linear regression underlying our differential expression analysis. The directed global significance score is calculated as the square root of the mean signed squared t-statistic for the genes in a gene set, with t-statistics coming from the linear regression underlying our differential expression analysis.

- **Volcano Plot: DPI: 2 vs.CTRL  
  More Plot Information**


  ##### Volcano Plot: DPI: 2 vs.CTRL

  Volcano plot displaying each gene's -log10(p-value) and log2 fold change for the selected covariate. Highly statistically significant genes fall at the top of the plot, and highly differentially expressed genes fall to either side. Genes within the selected gene set are highlighted in orange. Horizontal lines indicate various False Discovery Rate (FDR) thresholds.
- **DE Results - Autophagy genes - DPI: 2 vs.CTRL  
  More Plot Information  Download CSV Data**

  | Probe Label | Log2 fold change | std error (log2) | Lower confidence limit (log2) | Upper confidence limit (log2) | Linear fold change | Lower confidence limit (linear) | Upper confidence limit (linear) | P-value | BY.p.value | method | Gene.sets | Codeset.Name | Probe.Label | Analyte.Type | Is.Control | Control.Type | Probe.Annotation | KEGG.Pathways | Cell.Type | Official.Gene.Name | volcanocol | volcanopch | tstats.all |
  | --- | --- | --- | --- | --- | --- | --- | --- | --- | --- | --- | --- | --- | --- | --- | --- | --- | --- | --- | --- | --- | --- | --- | --- |
  | Dock1-mRNA | -1.49 | 0.186 | -1.85 | -1.12 | 0.357 | 0.277 | 0.459 | 5.68e-07 | 0.000111 | loglinear | Angiogenesis, Autophagy | NS\_MM\_NEUROINFLAM\_V1.0 | Dock1 | mRNA | 0 |  | Autophagy;Angiogenesis | mmu04510;mmu04810;mmu05100 |  | Dock1 | slateblue1 | 16 | -8.01075268817204 |
  | Mertk-mRNA | 1.61 | 0.224 | 1.17 | 2.05 | 3.06 | 2.25 | 4.14 | 2.13e-06 | 0.000313 | lm.nb | Autophagy, Microglia Function | NS\_MM\_NEUROINFLAM\_V1.0 | Mertk | mRNA | 0 |  | Microglia Function;Autophagy |  |  | Mertk | slateblue1 | 16 | 7.1875 |
  | Atg3-mRNA | -0.467 | 0.0705 | -0.605 | -0.329 | 0.723 | 0.657 | 0.796 | 5.81e-06 | 0.000708 | loglinear | Autophagy, Cellular Stress | NS\_MM\_NEUROINFLAM\_V1.0 | Atg3 | mRNA | 0 |  | Cellular Stress;Autophagy | mmu04140 |  | Atg3 | slateblue1 | 16 | -6.62411347517731 |
  | Dapk1-mRNA | 0.584 | 0.0943 | 0.399 | 0.769 | 1.5 | 1.32 | 1.7 | 1.28e-05 | 0.00113 | loglinear | Apoptosis, Autophagy | NS\_MM\_NEUROINFLAM\_V1.0 | Dapk1 | mRNA | 0 |  | Autophagy;Apoptosis | mmu05200;mmu05219 |  | Dapk1 | slateblue1 | 16 | 6.19300106044539 |
  | Fcgr2b-mRNA | 1.25 | 0.208 | 0.846 | 1.66 | 2.38 | 1.8 | 3.16 | 1.75e-05 | 0.00148 | lm.nb | Adaptive Immune Response, Autophagy | NS\_MM\_NEUROINFLAM\_V1.0 | Fcgr2b | mRNA | 0 |  | Autophagy;Adaptive Immune Response | mmu04145;mmu04380;mmu04662;mmu04666;mmu05150;mmu05152;mmu05162 |  | Fcgr2b | slateblue1 | 16 | 6.00961538461539 |
  | Fcgr3-mRNA | 2.05 | 0.36 | 1.34 | 2.75 | 4.14 | 2.54 | 6.74 | 3.32e-05 | 0.0024 | lm.nb | Adaptive Immune Response, Autophagy, Inflammatory Signaling, Innate Immune Response | NS\_MM\_NEUROINFLAM\_V1.0 | Fcgr3 | mRNA | 0 |  | Innate Immune Response;Inflammatory Signaling;Autophagy;Adaptive Immune Response | mmu04145;mmu04380;mmu05140;mmu05150;mmu05152 |  | Fcgr3 | slateblue1 | 16 | 5.69444444444444 |
  | Bcl2l1-mRNA | 0.407 | 0.0734 | 0.263 | 0.551 | 1.33 | 1.2 | 1.46 | 4.51e-05 | 0.00306 | loglinear | Apoptosis, Autophagy, Cytokine Signaling, Growth Factor Signaling, Innate Immune Response, NF-kB | NS\_MM\_NEUROINFLAM\_V1.0 | Bcl2l1 | mRNA | 0 |  | NF-kB;Innate Immune Response;Growth Factor Signaling;Cytokine Signaling;Autophagy;Apoptosis | mmu04014;mmu04064;mmu04151;mmu04210;mmu04630;mmu05014;mmu05145;mmu05166;mmu05200;mmu05202;mmu05212;mmu05220;mmu05222 |  | Bcl2l1 | slateblue1 | 16 | 5.54495912806539 |
  | Igf1r-mRNA | 0.813 | 0.155 | 0.51 | 1.12 | 1.76 | 1.42 | 2.17 | 7.74e-05 | 0.00441 | lm.nb | Apoptosis, Autophagy, Carbohydrate Metabolism, Growth Factor Signaling, Insulin Signaling | NS\_MM\_NEUROINFLAM\_V1.0 | Igf1r | mRNA | 0 |  | Insulin Signaling;Growth Factor Signaling;Carbohydrate Metabolism;Autophagy;Apoptosis | mmu04014;mmu04015;mmu04066;mmu04068;mmu04114;mmu04144;mmu04151;mmu04152;mmu04211;mmu04213;mmu04510;mmu04520;mmu04550;mmu04730;mmu04913;mmu04914;mmu05200;mmu05202;mmu05205;mmu05214;mmu05215;mmu05218 |  | Igf1r | slateblue1 | 16 | 5.24516129032258 |
  | Prkacb-mRNA | -0.322 | 0.0629 | -0.445 | -0.199 | 0.8 | 0.735 | 0.871 | 0.000104 | 0.00562 | loglinear | Adaptive Immune Response, Angiogenesis, Apoptosis, Autophagy, Growth Factor Signaling, Lipid Metabolism, Neurons and Neurotransmission, Wnt | NS\_MM\_NEUROINFLAM\_V1.0 | Prkacb | mRNA | 0 |  | Wnt;Neurons and Neurotransmission;Lipid Metabolism;Growth Factor Signaling;Autophagy;Apoptosis;Angiogenesis;Adaptive Immune Response | mmu04010;mmu04014;mmu04020;mmu04024;mmu04062;mmu04114;mmu04210;mmu04211;mmu04213;mmu04261;mmu04270;mmu04310;mmu04540;mmu04611;mmu04713;mmu04720;mmu04723;mmu04724;mmu04725;mmu04726;mmu04728;mmu04750;mmu04910;mmu04911;mmu04912;mmu04913;mmu04914;mmu04915;mmu04916;mmu04919;mmu04921;mmu04922;mmu04923;mmu04924;mmu04925;mmu05012;mmu05020;mmu05030;mmu05031;mmu05146;mmu05166;mmu05169;mmu05200;mmu05203;mmu05205;mmu05414 |  | Prkacb | slateblue1 | 16 | -5.11923688394277 |
  | Ulk1-mRNA | 0.38 | 0.0766 | 0.23 | 0.531 | 1.3 | 1.17 | 1.44 | 0.000141 | 0.0069 | loglinear | Autophagy, Cellular Stress | NS\_MM\_NEUROINFLAM\_V1.0 | Ulk1 | mRNA | 0 |  | Cellular Stress;Autophagy | mmu04140;mmu04150;mmu04152;mmu04211 |  | Ulk1 | slateblue1 | 16 | 4.96083550913838 |
  | Mapk10-mRNA | -0.673 | 0.138 | -0.943 | -0.402 | 0.627 | 0.52 | 0.757 | 0.000172 | 0.0082 | lm.nb | Adaptive Immune Response, Apoptosis, Autophagy, Cellular Stress, Growth Factor Signaling, Innate Immune Response, Wnt | NS\_MM\_NEUROINFLAM\_V1.0 | Mapk10 | mRNA | 0 |  | Wnt;Innate Immune Response;Growth Factor Signaling;Cellular Stress;Autophagy;Apoptosis;Adaptive Immune Response | mmu04010;mmu04012;mmu04014;mmu04024;mmu04068;mmu04071;mmu04141;mmu04310;mmu04380;mmu04510;mmu04620;mmu04621;mmu04622;mmu04664;mmu04668;mmu04722;mmu04723;mmu04728;mmu04750;mmu04910;mmu04912;mmu04914;mmu04917;mmu04920;mmu04930;mmu04931;mmu04932;mmu04933;mmu05132;mmu05133;mmu05142;mmu05145;mmu05152;mmu05160;mmu05161;mmu05164;mmu05168;mmu05169;mmu05200;mmu05210;mmu05212;mmu05231 |  | Mapk10 | slateblue1 | 16 | -4.8768115942029 |
  | Prkcq-mRNA | -0.864 | 0.185 | -1.23 | -0.501 | 0.549 | 0.427 | 0.707 | 0.000258 | 0.0106 | lm.nb | Adaptive Immune Response, Apoptosis, Autophagy, NF-kB, Oligodendrocyte Function | NS\_MM\_NEUROINFLAM\_V1.0 | Prkcq | mRNA | 0 |  | Oligodendrocyte Function;NF-kB;Autophagy;Apoptosis;Adaptive Immune Response | mmu04064;mmu04270;mmu04530;mmu04660;mmu04750;mmu04920;mmu04931;mmu05162 | Oligodendrocytes | Prkcq | slateblue2 | 16 | -4.67027027027027 |
  | Bnip3l-mRNA | -0.797 | 0.172 | -1.13 | -0.46 | 0.575 | 0.455 | 0.727 | 0.000276 | 0.0112 | lm.nb | Apoptosis, Autophagy | NS\_MM\_NEUROINFLAM\_V1.0 | Bnip3l | mRNA | 0 |  | Autophagy;Apoptosis |  |  | Bnip3l | slateblue2 | 16 | -4.63372093023256 |
  | Traf6-mRNA | 0.756 | 0.167 | 0.428 | 1.08 | 1.69 | 1.35 | 2.12 | 0.000353 | 0.0135 | lm.nb | Adaptive Immune Response, Apoptosis, Autophagy, Cytokine Signaling, Growth Factor Signaling, Innate Immune Response, NF-kB | NS\_MM\_NEUROINFLAM\_V1.0 | Traf6 | mRNA | 0 |  | NF-kB;Innate Immune Response;Growth Factor Signaling;Cytokine Signaling;Autophagy;Apoptosis;Adaptive Immune Response | mmu04010;mmu04064;mmu04120;mmu04144;mmu04380;mmu04620;mmu04621;mmu04622;mmu04722;mmu05133;mmu05140;mmu05142;mmu05145;mmu05152;mmu05160;mmu05162;mmu05168;mmu05169;mmu05200;mmu05222 |  | Traf6 | slateblue2 | 16 | 4.52694610778443 |
  | Lamp1-mRNA | 0.268 | 0.0595 | 0.151 | 0.385 | 1.2 | 1.11 | 1.31 | 0.00036 | 0.0136 | loglinear | Autophagy, Microglia Function | NS\_MM\_NEUROINFLAM\_V1.0 | Lamp1 | mRNA | 0 |  | Microglia Function;Autophagy | mmu04142;mmu04145;mmu05152 |  | Lamp1 | slateblue2 | 16 | 4.50420168067227 |
  | Prkce-mRNA | -0.438 | 0.0999 | -0.634 | -0.242 | 0.738 | 0.645 | 0.846 | 0.000463 | 0.0161 | loglinear | Autophagy, Growth Factor Signaling | NS\_MM\_NEUROINFLAM\_V1.0 | Prkce | mRNA | 0 |  | Growth Factor Signaling;Autophagy | mmu04022;mmu04071;mmu04270;mmu04530;mmu04666;mmu04750;mmu04925;mmu04930;mmu04931;mmu04933;mmu05206 |  | Prkce | slateblue2 | 16 | -4.38438438438438 |
  | Pik3r2-mRNA | -0.338 | 0.0793 | -0.494 | -0.183 | 0.791 | 0.71 | 0.881 | 0.000587 | 0.0197 | loglinear | Adaptive Immune Response, Angiogenesis, Apoptosis, Autophagy, Cytokine Signaling, Growth Factor Signaling, Innate Immune Response, Insulin Signaling | NS\_MM\_NEUROINFLAM\_V1.0 | Pik3r2 | mRNA | 0 |  | Insulin Signaling;Innate Immune Response;Growth Factor Signaling;Cytokine Signaling;Autophagy;Apoptosis;Angiogenesis;Adaptive Immune Response | mmu04012;mmu04014;mmu04015;mmu04022;mmu04024;mmu04062;mmu04066;mmu04068;mmu04070;mmu04071;mmu04072;mmu04150;mmu04151;mmu04152;mmu04210;mmu04211;mmu04213;mmu04261;mmu04370;mmu04380;mmu04510;mmu04550;mmu04611;mmu04620;mmu04630;mmu04650;mmu04660;mmu04662;mmu04664;mmu04666;mmu04668;mmu04670;mmu04722;mmu04725;mmu04750;mmu04810;mmu04910;mmu04914;mmu04915;mmu04917;mmu04919;mmu04921;mmu04923;mmu04930;mmu04931;mmu04932;mmu04933;mmu04960;mmu04973;mmu05100;mmu05142;mmu05145;mmu05146;mmu05160;mmu05161;mmu05162;mmu05164;mmu05166;mmu05169;mmu05200;mmu05203;mmu05205;mmu05210;mmu05211;mmu05212;mmu05213;mmu05214;mmu05215;mmu05218;mmu05220;mmu05221;mmu05222;mmu05223;mmu05230;mmu05231 |  | Pik3r2 | slateblue2 | 16 | -4.26229508196721 |
  | Akt2-mRNA | -0.592 | 0.142 | -0.869 | -0.314 | 0.664 | 0.547 | 0.804 | 0.000713 | 0.022 | loglinear | Adaptive Immune Response, Angiogenesis, Apoptosis, Autophagy, Carbohydrate Metabolism, Cytokine Signaling, Growth Factor Signaling, Innate Immune Response, Insulin Signaling, Neurons and Neurotransmission, Wnt | NS\_MM\_NEUROINFLAM\_V1.0 | Akt2 | mRNA | 0 |  | Wnt;Neurons and Neurotransmission;Insulin Signaling;Innate Immune Response;Growth Factor Signaling;Cytokine Signaling;Carbohydrate Metabolism;Autophagy;Apoptosis;Angiogenesis;Adaptive Immune Response | mmu04010;mmu04012;mmu04014;mmu04015;mmu04022;mmu04024;mmu04062;mmu04066;mmu04068;mmu04071;mmu04072;mmu04150;mmu04151;mmu04152;mmu04210;mmu04211;mmu04213;mmu04261;mmu04370;mmu04380;mmu04510;mmu04530;mmu04550;mmu04611;mmu04620;mmu04630;mmu04660;mmu04662;mmu04664;mmu04666;mmu04668;mmu04722;mmu04725;mmu04728;mmu04910;mmu04914;mmu04915;mmu04917;mmu04919;mmu04920;mmu04922;mmu04923;mmu04931;mmu04932;mmu04933;mmu04973;mmu05142;mmu05145;mmu05152;mmu05160;mmu05161;mmu05162;mmu05164;mmu05166;mmu05169;mmu05200;mmu05205;mmu05210;mmu05211;mmu05212;mmu05213;mmu05214;mmu05215;mmu05218;mmu05220;mmu05221;mmu05222;mmu05223;mmu05230;mmu05231 |  | Akt2 | slateblue2 | 16 | -4.16901408450704 |
  | Hmgb1-mRNA | -0.277 | 0.0677 | -0.41 | -0.145 | 0.825 | 0.753 | 0.905 | 0.00084 | 0.0239 | loglinear | Apoptosis, Autophagy, Inflammatory Signaling, Innate Immune Response | NS\_MM\_NEUROINFLAM\_V1.0 | Hmgb1 | mRNA | 0 |  | Innate Immune Response;Inflammatory Signaling;Autophagy;Apoptosis | mmu03410 |  | Hmgb1 | slateblue2 | 16 | -4.09158050221566 |
  | Pten-mRNA | -0.304 | 0.0753 | -0.452 | -0.156 | 0.81 | 0.731 | 0.897 | 0.000959 | 0.0266 | loglinear | Adaptive Immune Response, Autophagy, DNA Damage, Growth Factor Signaling, Lipid Metabolism | NS\_MM\_NEUROINFLAM\_V1.0 | Pten | mRNA | 0 |  | Lipid Metabolism;Growth Factor Signaling;DNA Damage;Autophagy;Adaptive Immune Response | mmu00562;mmu04068;mmu04070;mmu04071;mmu04115;mmu04150;mmu04151;mmu04510;mmu04530;mmu04931;mmu05161;mmu05200;mmu05206;mmu05213;mmu05214;mmu05215;mmu05218;mmu05222;mmu05230 |  | Pten | slateblue2 | 16 | -4.03718459495352 |

  ##### DE Results - Autophagy genes - DPI: 2 vs.CTRL

  Table displaying each sample's global significance scores and directed global significance scores as defined in the heatmaps above. The global significance score is calculated as the square root of the mean squared t-statistic for the genes in a gene set, with t-statistics coming from the linear regression underlying our differential expression analysis. The directed global significance score is calculated as the square root of the mean signed squared t-statistic for the genes in a gene set, with t-statistics coming from the linear regression underlying our differential expression analysis.

- **Volcano Plot: DPI: 3 vs.CTRL  
  More Plot Information**


  ##### Volcano Plot: DPI: 3 vs.CTRL

  Volcano plot displaying each gene's -log10(p-value) and log2 fold change for the selected covariate. Highly statistically significant genes fall at the top of the plot, and highly differentially expressed genes fall to either side. Genes within the selected gene set are highlighted in orange. Horizontal lines indicate various False Discovery Rate (FDR) thresholds.
- **DE Results - Autophagy genes - DPI: 3 vs.CTRL  
  More Plot Information  Download CSV Data**

  | Probe Label | Log2 fold change | std error (log2) | Lower confidence limit (log2) | Upper confidence limit (log2) | Linear fold change | Lower confidence limit (linear) | Upper confidence limit (linear) | P-value | BY.p.value | method | Gene.sets | Codeset.Name | Probe.Label | Analyte.Type | Is.Control | Control.Type | Probe.Annotation | KEGG.Pathways | Cell.Type | Official.Gene.Name | volcanocol | volcanopch | tstats.all |
  | --- | --- | --- | --- | --- | --- | --- | --- | --- | --- | --- | --- | --- | --- | --- | --- | --- | --- | --- | --- | --- | --- | --- | --- |
  | Dapk1-mRNA | 0.866 | 0.0943 | 0.681 | 1.05 | 1.82 | 1.6 | 2.07 | 8.86e-08 | 1.65e-05 | loglinear | Apoptosis, Autophagy | NS\_MM\_NEUROINFLAM\_V1.0 | Dapk1 | mRNA | 0 |  | Autophagy;Apoptosis | mmu05200;mmu05219 |  | Dapk1 | slateblue1 | 16 | 9.18345705196182 |
  | Fcgr2b-mRNA | 1.71 | 0.202 | 1.31 | 2.11 | 3.27 | 2.49 | 4.31 | 2.63e-07 | 3.57e-05 | lm.nb | Adaptive Immune Response, Autophagy | NS\_MM\_NEUROINFLAM\_V1.0 | Fcgr2b | mRNA | 0 |  | Autophagy;Adaptive Immune Response | mmu04145;mmu04380;mmu04662;mmu04666;mmu05150;mmu05152;mmu05162 |  | Fcgr2b | slateblue1 | 16 | 8.46534653465346 |
  | Mertk-mRNA | 1.52 | 0.225 | 1.07 | 1.96 | 2.86 | 2.11 | 3.89 | 4.83e-06 | 0.000363 | lm.nb | Autophagy, Microglia Function | NS\_MM\_NEUROINFLAM\_V1.0 | Mertk | mRNA | 0 |  | Microglia Function;Autophagy |  |  | Mertk | slateblue1 | 16 | 6.75555555555556 |
  | Dock1-mRNA | -1.14 | 0.186 | -1.5 | -0.774 | 0.454 | 0.353 | 0.585 | 1.49e-05 | 0.000921 | loglinear | Angiogenesis, Autophagy | NS\_MM\_NEUROINFLAM\_V1.0 | Dock1 | mRNA | 0 |  | Autophagy;Angiogenesis | mmu04510;mmu04810;mmu05100 |  | Dock1 | slateblue1 | 16 | -6.12903225806452 |
  | Becn1-mRNA | -0.336 | 0.059 | -0.451 | -0.22 | 0.792 | 0.731 | 0.858 | 3.33e-05 | 0.00171 | loglinear | Autophagy, Cellular Stress | NS\_MM\_NEUROINFLAM\_V1.0 | Becn1 | mRNA | 0 |  | Cellular Stress;Autophagy | mmu04140 |  | Becn1 | slateblue1 | 16 | -5.69491525423729 |
  | Bcl2l1-mRNA | 0.414 | 0.0734 | 0.27 | 0.558 | 1.33 | 1.21 | 1.47 | 3.75e-05 | 0.00184 | loglinear | Apoptosis, Autophagy, Cytokine Signaling, Growth Factor Signaling, Innate Immune Response, NF-kB | NS\_MM\_NEUROINFLAM\_V1.0 | Bcl2l1 | mRNA | 0 |  | NF-kB;Innate Immune Response;Growth Factor Signaling;Cytokine Signaling;Autophagy;Apoptosis | mmu04014;mmu04064;mmu04151;mmu04210;mmu04630;mmu05014;mmu05145;mmu05166;mmu05200;mmu05202;mmu05212;mmu05220;mmu05222 |  | Bcl2l1 | slateblue1 | 16 | 5.64032697547684 |
  | Traf6-mRNA | 0.92 | 0.166 | 0.594 | 1.25 | 1.89 | 1.51 | 2.37 | 4.54e-05 | 0.00214 | lm.nb | Adaptive Immune Response, Apoptosis, Autophagy, Cytokine Signaling, Growth Factor Signaling, Innate Immune Response, NF-kB | NS\_MM\_NEUROINFLAM\_V1.0 | Traf6 | mRNA | 0 |  | NF-kB;Innate Immune Response;Growth Factor Signaling;Cytokine Signaling;Autophagy;Apoptosis;Adaptive Immune Response | mmu04010;mmu04064;mmu04120;mmu04144;mmu04380;mmu04620;mmu04621;mmu04622;mmu04722;mmu05133;mmu05140;mmu05142;mmu05145;mmu05152;mmu05160;mmu05162;mmu05168;mmu05169;mmu05200;mmu05222 |  | Traf6 | slateblue1 | 16 | 5.54216867469879 |
  | Akt2-mRNA | -0.766 | 0.142 | -1.04 | -0.488 | 0.588 | 0.485 | 0.713 | 5.85e-05 | 0.00265 | loglinear | Adaptive Immune Response, Angiogenesis, Apoptosis, Autophagy, Carbohydrate Metabolism, Cytokine Signaling, Growth Factor Signaling, Innate Immune Response, Insulin Signaling, Neurons and Neurotransmission, Wnt | NS\_MM\_NEUROINFLAM\_V1.0 | Akt2 | mRNA | 0 |  | Wnt;Neurons and Neurotransmission;Insulin Signaling;Innate Immune Response;Growth Factor Signaling;Cytokine Signaling;Carbohydrate Metabolism;Autophagy;Apoptosis;Angiogenesis;Adaptive Immune Response | mmu04010;mmu04012;mmu04014;mmu04015;mmu04022;mmu04024;mmu04062;mmu04066;mmu04068;mmu04071;mmu04072;mmu04150;mmu04151;mmu04152;mmu04210;mmu04211;mmu04213;mmu04261;mmu04370;mmu04380;mmu04510;mmu04530;mmu04550;mmu04611;mmu04620;mmu04630;mmu04660;mmu04662;mmu04664;mmu04666;mmu04668;mmu04722;mmu04725;mmu04728;mmu04910;mmu04914;mmu04915;mmu04917;mmu04919;mmu04920;mmu04922;mmu04923;mmu04931;mmu04932;mmu04933;mmu04973;mmu05142;mmu05145;mmu05152;mmu05160;mmu05161;mmu05162;mmu05164;mmu05166;mmu05169;mmu05200;mmu05205;mmu05210;mmu05211;mmu05212;mmu05213;mmu05214;mmu05215;mmu05218;mmu05220;mmu05221;mmu05222;mmu05223;mmu05230;mmu05231 |  | Akt2 | slateblue1 | 16 | -5.3943661971831 |
  | Lamp1-mRNA | 0.3 | 0.0595 | 0.183 | 0.416 | 1.23 | 1.14 | 1.33 | 0.000122 | 0.00498 | loglinear | Autophagy, Microglia Function | NS\_MM\_NEUROINFLAM\_V1.0 | Lamp1 | mRNA | 0 |  | Microglia Function;Autophagy | mmu04142;mmu04145;mmu05152 |  | Lamp1 | slateblue1 | 16 | 5.04201680672269 |
  | Epg5-mRNA | -0.459 | 0.101 | -0.657 | -0.262 | 0.727 | 0.634 | 0.834 | 0.000323 | 0.0112 | loglinear | Autophagy | NS\_MM\_NEUROINFLAM\_V1.0 | Epg5 | mRNA | 0 |  | Autophagy |  |  | Epg5 | slateblue2 | 16 | -4.54455445544554 |
  | Bnip3-mRNA | 0.275 | 0.0611 | 0.155 | 0.394 | 1.21 | 1.11 | 1.31 | 0.00037 | 0.0126 | loglinear | Apoptosis, Autophagy, Cellular Stress, Microglia Function | NS\_MM\_NEUROINFLAM\_V1.0 | Bnip3 | mRNA | 0 |  | Microglia Function;Cellular Stress;Autophagy;Apoptosis | mmu04068;mmu05134 |  | Bnip3 | slateblue2 | 16 | 4.50081833060556 |
  | Fcgr3-mRNA | 1.62 | 0.365 | 0.902 | 2.33 | 3.07 | 1.87 | 5.04 | 0.000421 | 0.0139 | lm.nb | Adaptive Immune Response, Autophagy, Inflammatory Signaling, Innate Immune Response | NS\_MM\_NEUROINFLAM\_V1.0 | Fcgr3 | mRNA | 0 |  | Innate Immune Response;Inflammatory Signaling;Autophagy;Adaptive Immune Response | mmu04145;mmu04380;mmu05140;mmu05150;mmu05152 |  | Fcgr3 | slateblue2 | 16 | 4.43835616438356 |
  | Lamp2-mRNA | 0.324 | 0.0738 | 0.18 | 0.469 | 1.25 | 1.13 | 1.38 | 0.000454 | 0.0148 | loglinear | Autophagy | NS\_MM\_NEUROINFLAM\_V1.0 | Lamp2 | mRNA | 0 |  | Autophagy | mmu04142;mmu04145;mmu05152 |  | Lamp2 | slateblue2 | 16 | 4.39024390243902 |
  | Bcl2-mRNA | -0.943 | 0.227 | -1.39 | -0.498 | 0.52 | 0.382 | 0.708 | 0.000747 | 0.0225 | lm.nb | Apoptosis, Autophagy, Cellular Stress, Cytokine Signaling, DNA Damage, Growth Factor Signaling, Innate Immune Response, Microglia Function, NF-kB | NS\_MM\_NEUROINFLAM\_V1.0 | Bcl2 | mRNA | 0 |  | NF-kB;Microglia Function;Innate Immune Response;Growth Factor Signaling;DNA Damage;Cytokine Signaling;Cellular Stress;Autophagy;Apoptosis | mmu04064;mmu04066;mmu04071;mmu04141;mmu04151;mmu04210;mmu04261;mmu04510;mmu04630;mmu04722;mmu04725;mmu04933;mmu05014;mmu05145;mmu05152;mmu05161;mmu05169;mmu05200;mmu05206;mmu05210;mmu05215;mmu05222 |  | Bcl2 | slateblue2 | 16 | -4.15418502202643 |
  | Mapk10-mRNA | -0.571 | 0.138 | -0.842 | -0.3 | 0.673 | 0.558 | 0.812 | 0.000782 | 0.0231 | lm.nb | Adaptive Immune Response, Apoptosis, Autophagy, Cellular Stress, Growth Factor Signaling, Innate Immune Response, Wnt | NS\_MM\_NEUROINFLAM\_V1.0 | Mapk10 | mRNA | 0 |  | Wnt;Innate Immune Response;Growth Factor Signaling;Cellular Stress;Autophagy;Apoptosis;Adaptive Immune Response | mmu04010;mmu04012;mmu04014;mmu04024;mmu04068;mmu04071;mmu04141;mmu04310;mmu04380;mmu04510;mmu04620;mmu04621;mmu04622;mmu04664;mmu04668;mmu04722;mmu04723;mmu04728;mmu04750;mmu04910;mmu04912;mmu04914;mmu04917;mmu04920;mmu04930;mmu04931;mmu04932;mmu04933;mmu05132;mmu05133;mmu05142;mmu05145;mmu05152;mmu05160;mmu05161;mmu05164;mmu05168;mmu05169;mmu05200;mmu05210;mmu05212;mmu05231 |  | Mapk10 | slateblue2 | 16 | -4.13768115942029 |
  | Prkcq-mRNA | -0.735 | 0.181 | -1.09 | -0.379 | 0.601 | 0.47 | 0.769 | 0.000927 | 0.0267 | lm.nb | Adaptive Immune Response, Apoptosis, Autophagy, NF-kB, Oligodendrocyte Function | NS\_MM\_NEUROINFLAM\_V1.0 | Prkcq | mRNA | 0 |  | Oligodendrocyte Function;NF-kB;Autophagy;Apoptosis;Adaptive Immune Response | mmu04064;mmu04270;mmu04530;mmu04660;mmu04750;mmu04920;mmu04931;mmu05162 | Oligodendrocytes | Prkcq | slateblue2 | 16 | -4.06077348066298 |
  | Pik3cd-mRNA | -0.63 | 0.159 | -0.942 | -0.318 | 0.646 | 0.52 | 0.802 | 0.00114 | 0.0306 | lm.nb | Adaptive Immune Response, Apoptosis, Autophagy, Cytokine Signaling, Growth Factor Signaling, Innate Immune Response | NS\_MM\_NEUROINFLAM\_V1.0 | Pik3cd | mRNA | 0 |  | Innate Immune Response;Growth Factor Signaling;Cytokine Signaling;Autophagy;Apoptosis;Adaptive Immune Response |  |  |  | slateblue2 | 16 | -3.9622641509434 |
  | Igf1r-mRNA | 0.603 | 0.156 | 0.297 | 0.908 | 1.52 | 1.23 | 1.88 | 0.00137 | 0.0347 | lm.nb | Apoptosis, Autophagy, Carbohydrate Metabolism, Growth Factor Signaling, Insulin Signaling | NS\_MM\_NEUROINFLAM\_V1.0 | Igf1r | mRNA | 0 |  | Insulin Signaling;Growth Factor Signaling;Carbohydrate Metabolism;Autophagy;Apoptosis | mmu04014;mmu04015;mmu04066;mmu04068;mmu04114;mmu04144;mmu04151;mmu04152;mmu04211;mmu04213;mmu04510;mmu04520;mmu04550;mmu04730;mmu04913;mmu04914;mmu05200;mmu05202;mmu05205;mmu05214;mmu05215;mmu05218 |  | Igf1r | slateblue2 | 16 | 3.86538461538462 |
  | Ulk1-mRNA | 0.296 | 0.0766 | 0.145 | 0.446 | 1.23 | 1.11 | 1.36 | 0.00139 | 0.0351 | loglinear | Autophagy, Cellular Stress | NS\_MM\_NEUROINFLAM\_V1.0 | Ulk1 | mRNA | 0 |  | Cellular Stress;Autophagy | mmu04140;mmu04150;mmu04152;mmu04211 |  | Ulk1 | slateblue2 | 16 | 3.86422976501305 |
  | Atg3-mRNA | -0.259 | 0.0705 | -0.397 | -0.121 | 0.836 | 0.759 | 0.92 | 0.00207 | 0.0504 | loglinear | Autophagy, Cellular Stress | NS\_MM\_NEUROINFLAM\_V1.0 | Atg3 | mRNA | 0 |  | Cellular Stress;Autophagy | mmu04140 |  | Atg3 | slateblue3 | 16 | -3.67375886524823 |

  ##### DE Results - Autophagy genes - DPI: 3 vs.CTRL

  Table displaying each sample's global significance scores and directed global significance scores as defined in the heatmaps above. The global significance score is calculated as the square root of the mean squared t-statistic for the genes in a gene set, with t-statistics coming from the linear regression underlying our differential expression analysis. The directed global significance score is calculated as the square root of the mean signed squared t-statistic for the genes in a gene set, with t-statistics coming from the linear regression underlying our differential expression analysis.

DPI:
- DPI: differential expression in 1 vs. baseline of CTRL
- DPI: differential expression in 2 vs. baseline of CTRL
- DPI: differential expression in 3 vs. baseline of CTRL

- **Volcano Plot: DPI: 1 vs.CTRL  
  More Plot Information**


  ##### Volcano Plot: DPI: 1 vs.CTRL

  Volcano plot displaying each gene's -log10(p-value) and log2 fold change for the selected covariate. Highly statistically significant genes fall at the top of the plot, and highly differentially expressed genes fall to either side. Genes within the selected gene set are highlighted in orange. Horizontal lines indicate various False Discovery Rate (FDR) thresholds.
- **DE Results - Carbohydrate Metabolism genes - DPI: 1 vs.CTRL  
  More Plot Information  Download CSV Data**

  | Probe Label | Log2 fold change | std error (log2) | Lower confidence limit (log2) | Upper confidence limit (log2) | Linear fold change | Lower confidence limit (linear) | Upper confidence limit (linear) | P-value | BY.p.value | method | Gene.sets | Codeset.Name | Probe.Label | Analyte.Type | Is.Control | Control.Type | Probe.Annotation | KEGG.Pathways | Cell.Type | Official.Gene.Name | volcanocol | volcanopch | tstats.all |
  | --- | --- | --- | --- | --- | --- | --- | --- | --- | --- | --- | --- | --- | --- | --- | --- | --- | --- | --- | --- | --- | --- | --- | --- |
  | Igf1r-mRNA | 0.523 | 0.156 | 0.216 | 0.83 | 1.44 | 1.16 | 1.78 | 0.00414 | 0.197 | lm.nb | Apoptosis, Autophagy, Carbohydrate Metabolism, Growth Factor Signaling, Insulin Signaling | NS\_MM\_NEUROINFLAM\_V1.0 | Igf1r | mRNA | 0 |  | Insulin Signaling;Growth Factor Signaling;Carbohydrate Metabolism;Autophagy;Apoptosis | mmu04014;mmu04015;mmu04066;mmu04068;mmu04114;mmu04144;mmu04151;mmu04152;mmu04211;mmu04213;mmu04510;mmu04520;mmu04550;mmu04730;mmu04913;mmu04914;mmu05200;mmu05202;mmu05205;mmu05214;mmu05215;mmu05218 |  | Igf1r | slateblue4 | 16 | 3.3525641025641 |
  | Pik3ca-mRNA | -0.287 | 0.107 | -0.497 | -0.0771 | 0.82 | 0.709 | 0.948 | 0.0164 | 0.417 | lm.nb | Adaptive Immune Response, Angiogenesis, Apoptosis, Autophagy, Carbohydrate Metabolism, Cytokine Signaling, Growth Factor Signaling, Innate Immune Response, Insulin Signaling, Lipid Metabolism | NS\_MM\_NEUROINFLAM\_V1.0 | Pik3ca | mRNA | 0 |  | Lipid Metabolism;Insulin Signaling;Innate Immune Response;Growth Factor Signaling;Cytokine Signaling;Carbohydrate Metabolism;Autophagy;Apoptosis;Angiogenesis;Adaptive Immune Response | mmu00562;mmu04012;mmu04014;mmu04015;mmu04022;mmu04024;mmu04062;mmu04066;mmu04068;mmu04070;mmu04071;mmu04072;mmu04150;mmu04151;mmu04152;mmu04210;mmu04211;mmu04213;mmu04261;mmu04370;mmu04380;mmu04510;mmu04550;mmu04611;mmu04620;mmu04630;mmu04650;mmu04660;mmu04662;mmu04664;mmu04666;mmu04668;mmu04670;mmu04722;mmu04725;mmu04750;mmu04810;mmu04910;mmu04914;mmu04915;mmu04917;mmu04919;mmu04921;mmu04923;mmu04930;mmu04931;mmu04932;mmu04933;mmu04960;mmu04973;mmu05100;mmu05142;mmu05145;mmu05146;mmu05160;mmu05161;mmu05162;mmu05164;mmu05166;mmu05169;mmu05200;mmu05203;mmu05205;mmu05210;mmu05211;mmu05212;mmu05213;mmu05214;mmu05215;mmu05218;mmu05220;mmu05221;mmu05222;mmu05223;mmu05230;mmu05231 |  | Pik3ca | slateblue4 | 16 | -2.68224299065421 |
  | Pik3r1-mRNA | -0.169 | 0.0713 | -0.308 | -0.0288 | 0.89 | 0.808 | 0.98 | 0.0311 | 0.62 | loglinear | Adaptive Immune Response, Angiogenesis, Apoptosis, Autophagy, Carbohydrate Metabolism, Cytokine Signaling, Growth Factor Signaling, Innate Immune Response, Insulin Signaling, Lipid Metabolism | NS\_MM\_NEUROINFLAM\_V1.0 | Pik3r1 | mRNA | 0 |  | Lipid Metabolism;Insulin Signaling;Innate Immune Response;Growth Factor Signaling;Cytokine Signaling;Carbohydrate Metabolism;Autophagy;Apoptosis;Angiogenesis;Adaptive Immune Response | mmu04012;mmu04014;mmu04015;mmu04022;mmu04024;mmu04062;mmu04066;mmu04068;mmu04070;mmu04071;mmu04072;mmu04150;mmu04151;mmu04152;mmu04210;mmu04211;mmu04213;mmu04261;mmu04370;mmu04380;mmu04510;mmu04550;mmu04611;mmu04620;mmu04630;mmu04650;mmu04660;mmu04662;mmu04664;mmu04666;mmu04668;mmu04670;mmu04722;mmu04725;mmu04750;mmu04810;mmu04910;mmu04914;mmu04915;mmu04917;mmu04919;mmu04921;mmu04923;mmu04930;mmu04931;mmu04932;mmu04933;mmu04960;mmu04973;mmu05100;mmu05142;mmu05145;mmu05146;mmu05160;mmu05161;mmu05162;mmu05164;mmu05166;mmu05169;mmu05200;mmu05203;mmu05205;mmu05210;mmu05211;mmu05212;mmu05213;mmu05214;mmu05215;mmu05218;mmu05220;mmu05221;mmu05222;mmu05223;mmu05230;mmu05231 |  | Pik3r1 | azure4 | 1 | -2.37026647966339 |
  | Akt2-mRNA | -0.291 | 0.142 | -0.568 | -0.0129 | 0.818 | 0.674 | 0.991 | 0.057 | 0.91 | loglinear | Adaptive Immune Response, Angiogenesis, Apoptosis, Autophagy, Carbohydrate Metabolism, Cytokine Signaling, Growth Factor Signaling, Innate Immune Response, Insulin Signaling, Neurons and Neurotransmission, Wnt | NS\_MM\_NEUROINFLAM\_V1.0 | Akt2 | mRNA | 0 |  | Wnt;Neurons and Neurotransmission;Insulin Signaling;Innate Immune Response;Growth Factor Signaling;Cytokine Signaling;Carbohydrate Metabolism;Autophagy;Apoptosis;Angiogenesis;Adaptive Immune Response | mmu04010;mmu04012;mmu04014;mmu04015;mmu04022;mmu04024;mmu04062;mmu04066;mmu04068;mmu04071;mmu04072;mmu04150;mmu04151;mmu04152;mmu04210;mmu04211;mmu04213;mmu04261;mmu04370;mmu04380;mmu04510;mmu04530;mmu04550;mmu04611;mmu04620;mmu04630;mmu04660;mmu04662;mmu04664;mmu04666;mmu04668;mmu04722;mmu04725;mmu04728;mmu04910;mmu04914;mmu04915;mmu04917;mmu04919;mmu04920;mmu04922;mmu04923;mmu04931;mmu04932;mmu04933;mmu04973;mmu05142;mmu05145;mmu05152;mmu05160;mmu05161;mmu05162;mmu05164;mmu05166;mmu05169;mmu05200;mmu05205;mmu05210;mmu05211;mmu05212;mmu05213;mmu05214;mmu05215;mmu05218;mmu05220;mmu05221;mmu05222;mmu05223;mmu05230;mmu05231 |  | Akt2 | azure4 | 1 | -2.04929577464789 |
  | Creb1-mRNA | -0.125 | 0.0668 | -0.255 | 0.00632 | 0.917 | 0.838 | 1 | 0.0806 | 1 | loglinear | Adaptive Immune Response, Carbohydrate Metabolism, DNA Damage, Growth Factor Signaling, Innate Immune Response, Neurons and Neurotransmission, Notch | NS\_MM\_NEUROINFLAM\_V1.0 | Creb1 | mRNA | 0 |  | Notch;Neurons and Neurotransmission;Innate Immune Response;Growth Factor Signaling;DNA Damage;Carbohydrate Metabolism;Adaptive Immune Response | mmu04022;mmu04024;mmu04151;mmu04152;mmu04211;mmu04261;mmu04380;mmu04612;mmu04668;mmu04713;mmu04725;mmu04728;mmu04911;mmu04915;mmu04916;mmu04922;mmu04924;mmu04925;mmu04931;mmu05016;mmu05030;mmu05031;mmu05034;mmu05152;mmu05161;mmu05166;mmu05203;mmu05215 |  | Creb1 | azure4 | 1 | -1.87125748502994 |
  | Slco2b1-mRNA | 0.675 | 0.388 | -0.0864 | 1.44 | 1.6 | 0.942 | 2.7 | 0.102 | 1 | lm.nb | Carbohydrate Metabolism | NS\_MM\_NEUROINFLAM\_V1.0 | Slco2b1 | mRNA | 0 |  | Carbohydrate Metabolism |  |  | Slco2b1 | azure4 | 1 | 1.73969072164948 |
  | Akt1-mRNA | 0.177 | 0.107 | -0.0326 | 0.387 | 1.13 | 0.978 | 1.31 | 0.117 | 1 | loglinear | Adaptive Immune Response, Angiogenesis, Apoptosis, Autophagy, Carbohydrate Metabolism, Cellular Stress, Cytokine Signaling, Growth Factor Signaling, Innate Immune Response, Insulin Signaling, Neurons and Neurotransmission, Wnt | NS\_MM\_NEUROINFLAM\_V1.0 | Akt1 | mRNA | 0 |  | Wnt;Neurons and Neurotransmission;Insulin Signaling;Innate Immune Response;Growth Factor Signaling;Cytokine Signaling;Cellular Stress;Carbohydrate Metabolism;Autophagy;Apoptosis;Angiogenesis;Adaptive Immune Response | mmu04010;mmu04012;mmu04014;mmu04015;mmu04022;mmu04024;mmu04062;mmu04066;mmu04068;mmu04071;mmu04072;mmu04150;mmu04151;mmu04152;mmu04210;mmu04211;mmu04213;mmu04261;mmu04370;mmu04380;mmu04510;mmu04530;mmu04550;mmu04611;mmu04620;mmu04630;mmu04660;mmu04662;mmu04664;mmu04666;mmu04668;mmu04722;mmu04725;mmu04728;mmu04910;mmu04914;mmu04915;mmu04917;mmu04919;mmu04920;mmu04922;mmu04923;mmu04931;mmu04932;mmu04933;mmu04973;mmu05142;mmu05145;mmu05152;mmu05160;mmu05161;mmu05162;mmu05164;mmu05166;mmu05169;mmu05200;mmu05205;mmu05210;mmu05211;mmu05212;mmu05213;mmu05214;mmu05215;mmu05218;mmu05220;mmu05221;mmu05222;mmu05223;mmu05230;mmu05231 |  | Akt1 | azure4 | 1 | 1.65420560747664 |
  | Pik3cb-mRNA | -0.144 | 0.116 | -0.372 | 0.0841 | 0.905 | 0.773 | 1.06 | 0.234 | 1 | loglinear | Adaptive Immune Response, Angiogenesis, Apoptosis, Autophagy, Carbohydrate Metabolism, Cytokine Signaling, Growth Factor Signaling, Innate Immune Response, Insulin Signaling, Lipid Metabolism | NS\_MM\_NEUROINFLAM\_V1.0 | Pik3cb | mRNA | 0 |  | Lipid Metabolism;Insulin Signaling;Innate Immune Response;Growth Factor Signaling;Cytokine Signaling;Carbohydrate Metabolism;Autophagy;Apoptosis;Angiogenesis;Adaptive Immune Response | mmu00562;mmu04012;mmu04014;mmu04015;mmu04022;mmu04024;mmu04062;mmu04066;mmu04068;mmu04070;mmu04071;mmu04072;mmu04150;mmu04151;mmu04152;mmu04210;mmu04211;mmu04213;mmu04261;mmu04370;mmu04380;mmu04510;mmu04550;mmu04611;mmu04620;mmu04630;mmu04650;mmu04660;mmu04662;mmu04664;mmu04666;mmu04668;mmu04670;mmu04722;mmu04725;mmu04750;mmu04810;mmu04910;mmu04914;mmu04915;mmu04917;mmu04919;mmu04921;mmu04923;mmu04930;mmu04931;mmu04932;mmu04933;mmu04960;mmu04973;mmu05100;mmu05142;mmu05145;mmu05146;mmu05160;mmu05161;mmu05162;mmu05164;mmu05166;mmu05169;mmu05200;mmu05203;mmu05205;mmu05210;mmu05211;mmu05212;mmu05213;mmu05214;mmu05215;mmu05218;mmu05220;mmu05221;mmu05222;mmu05223;mmu05230;mmu05231 |  | Pik3cb | azure4 | 1 | -1.24137931034483 |
  | Sirt1-mRNA | 0.0739 | 0.109 | -0.139 | 0.287 | 1.05 | 0.908 | 1.22 | 0.507 | 1 | loglinear | Carbohydrate Metabolism, Cellular Stress, Epigenetic Regulation | NS\_MM\_NEUROINFLAM\_V1.0 | Sirt1 | mRNA | 0 |  | Epigenetic Regulation;Cellular Stress;Carbohydrate Metabolism | mmu04068;mmu04152;mmu04211;mmu04213;mmu04922;mmu05031;mmu05206 |  | Sirt1 | azure4 | 1 | 0.677981651376147 |

  ##### DE Results - Carbohydrate Metabolism genes - DPI: 1 vs.CTRL

  Table displaying each sample's global significance scores and directed global significance scores as defined in the heatmaps above. The global significance score is calculated as the square root of the mean squared t-statistic for the genes in a gene set, with t-statistics coming from the linear regression underlying our differential expression analysis. The directed global significance score is calculated as the square root of the mean signed squared t-statistic for the genes in a gene set, with t-statistics coming from the linear regression underlying our differential expression analysis.

- **Volcano Plot: DPI: 2 vs.CTRL  
  More Plot Information**


  ##### Volcano Plot: DPI: 2 vs.CTRL

  Volcano plot displaying each gene's -log10(p-value) and log2 fold change for the selected covariate. Highly statistically significant genes fall at the top of the plot, and highly differentially expressed genes fall to either side. Genes within the selected gene set are highlighted in orange. Horizontal lines indicate various False Discovery Rate (FDR) thresholds.
- **DE Results - Carbohydrate Metabolism genes - DPI: 2 vs.CTRL  
  More Plot Information  Download CSV Data**

  | Probe Label | Log2 fold change | std error (log2) | Lower confidence limit (log2) | Upper confidence limit (log2) | Linear fold change | Lower confidence limit (linear) | Upper confidence limit (linear) | P-value | BY.p.value | method | Gene.sets | Codeset.Name | Probe.Label | Analyte.Type | Is.Control | Control.Type | Probe.Annotation | KEGG.Pathways | Cell.Type | Official.Gene.Name | volcanocol | volcanopch | tstats.all |
  | --- | --- | --- | --- | --- | --- | --- | --- | --- | --- | --- | --- | --- | --- | --- | --- | --- | --- | --- | --- | --- | --- | --- | --- |
  | Igf1r-mRNA | 0.813 | 0.155 | 0.51 | 1.12 | 1.76 | 1.42 | 2.17 | 7.74e-05 | 0.00441 | lm.nb | Apoptosis, Autophagy, Carbohydrate Metabolism, Growth Factor Signaling, Insulin Signaling | NS\_MM\_NEUROINFLAM\_V1.0 | Igf1r | mRNA | 0 |  | Insulin Signaling;Growth Factor Signaling;Carbohydrate Metabolism;Autophagy;Apoptosis | mmu04014;mmu04015;mmu04066;mmu04068;mmu04114;mmu04144;mmu04151;mmu04152;mmu04211;mmu04213;mmu04510;mmu04520;mmu04550;mmu04730;mmu04913;mmu04914;mmu05200;mmu05202;mmu05205;mmu05214;mmu05215;mmu05218 |  | Igf1r | slateblue1 | 16 | 5.24516129032258 |
  | Akt2-mRNA | -0.592 | 0.142 | -0.869 | -0.314 | 0.664 | 0.547 | 0.804 | 0.000713 | 0.022 | loglinear | Adaptive Immune Response, Angiogenesis, Apoptosis, Autophagy, Carbohydrate Metabolism, Cytokine Signaling, Growth Factor Signaling, Innate Immune Response, Insulin Signaling, Neurons and Neurotransmission, Wnt | NS\_MM\_NEUROINFLAM\_V1.0 | Akt2 | mRNA | 0 |  | Wnt;Neurons and Neurotransmission;Insulin Signaling;Innate Immune Response;Growth Factor Signaling;Cytokine Signaling;Carbohydrate Metabolism;Autophagy;Apoptosis;Angiogenesis;Adaptive Immune Response | mmu04010;mmu04012;mmu04014;mmu04015;mmu04022;mmu04024;mmu04062;mmu04066;mmu04068;mmu04071;mmu04072;mmu04150;mmu04151;mmu04152;mmu04210;mmu04211;mmu04213;mmu04261;mmu04370;mmu04380;mmu04510;mmu04530;mmu04550;mmu04611;mmu04620;mmu04630;mmu04660;mmu04662;mmu04664;mmu04666;mmu04668;mmu04722;mmu04725;mmu04728;mmu04910;mmu04914;mmu04915;mmu04917;mmu04919;mmu04920;mmu04922;mmu04923;mmu04931;mmu04932;mmu04933;mmu04973;mmu05142;mmu05145;mmu05152;mmu05160;mmu05161;mmu05162;mmu05164;mmu05166;mmu05169;mmu05200;mmu05205;mmu05210;mmu05211;mmu05212;mmu05213;mmu05214;mmu05215;mmu05218;mmu05220;mmu05221;mmu05222;mmu05223;mmu05230;mmu05231 |  | Akt2 | slateblue2 | 16 | -4.16901408450704 |
  | Pik3cb-mRNA | -0.376 | 0.116 | -0.604 | -0.148 | 0.771 | 0.658 | 0.902 | 0.0052 | 0.0976 | loglinear | Adaptive Immune Response, Angiogenesis, Apoptosis, Autophagy, Carbohydrate Metabolism, Cytokine Signaling, Growth Factor Signaling, Innate Immune Response, Insulin Signaling, Lipid Metabolism | NS\_MM\_NEUROINFLAM\_V1.0 | Pik3cb | mRNA | 0 |  | Lipid Metabolism;Insulin Signaling;Innate Immune Response;Growth Factor Signaling;Cytokine Signaling;Carbohydrate Metabolism;Autophagy;Apoptosis;Angiogenesis;Adaptive Immune Response | mmu00562;mmu04012;mmu04014;mmu04015;mmu04022;mmu04024;mmu04062;mmu04066;mmu04068;mmu04070;mmu04071;mmu04072;mmu04150;mmu04151;mmu04152;mmu04210;mmu04211;mmu04213;mmu04261;mmu04370;mmu04380;mmu04510;mmu04550;mmu04611;mmu04620;mmu04630;mmu04650;mmu04660;mmu04662;mmu04664;mmu04666;mmu04668;mmu04670;mmu04722;mmu04725;mmu04750;mmu04810;mmu04910;mmu04914;mmu04915;mmu04917;mmu04919;mmu04921;mmu04923;mmu04930;mmu04931;mmu04932;mmu04933;mmu04960;mmu04973;mmu05100;mmu05142;mmu05145;mmu05146;mmu05160;mmu05161;mmu05162;mmu05164;mmu05166;mmu05169;mmu05200;mmu05203;mmu05205;mmu05210;mmu05211;mmu05212;mmu05213;mmu05214;mmu05215;mmu05218;mmu05220;mmu05221;mmu05222;mmu05223;mmu05230;mmu05231 |  | Pik3cb | slateblue4 | 16 | -3.24137931034483 |
  | Pik3ca-mRNA | -0.277 | 0.107 | -0.487 | -0.0672 | 0.825 | 0.714 | 0.954 | 0.0198 | 0.276 | lm.nb | Adaptive Immune Response, Angiogenesis, Apoptosis, Autophagy, Carbohydrate Metabolism, Cytokine Signaling, Growth Factor Signaling, Innate Immune Response, Insulin Signaling, Lipid Metabolism | NS\_MM\_NEUROINFLAM\_V1.0 | Pik3ca | mRNA | 0 |  | Lipid Metabolism;Insulin Signaling;Innate Immune Response;Growth Factor Signaling;Cytokine Signaling;Carbohydrate Metabolism;Autophagy;Apoptosis;Angiogenesis;Adaptive Immune Response | mmu00562;mmu04012;mmu04014;mmu04015;mmu04022;mmu04024;mmu04062;mmu04066;mmu04068;mmu04070;mmu04071;mmu04072;mmu04150;mmu04151;mmu04152;mmu04210;mmu04211;mmu04213;mmu04261;mmu04370;mmu04380;mmu04510;mmu04550;mmu04611;mmu04620;mmu04630;mmu04650;mmu04660;mmu04662;mmu04664;mmu04666;mmu04668;mmu04670;mmu04722;mmu04725;mmu04750;mmu04810;mmu04910;mmu04914;mmu04915;mmu04917;mmu04919;mmu04921;mmu04923;mmu04930;mmu04931;mmu04932;mmu04933;mmu04960;mmu04973;mmu05100;mmu05142;mmu05145;mmu05146;mmu05160;mmu05161;mmu05162;mmu05164;mmu05166;mmu05169;mmu05200;mmu05203;mmu05205;mmu05210;mmu05211;mmu05212;mmu05213;mmu05214;mmu05215;mmu05218;mmu05220;mmu05221;mmu05222;mmu05223;mmu05230;mmu05231 |  | Pik3ca | slateblue4 | 16 | -2.58878504672897 |
  | Akt1-mRNA | 0.203 | 0.107 | -0.00674 | 0.413 | 1.15 | 0.995 | 1.33 | 0.076 | 0.823 | loglinear | Adaptive Immune Response, Angiogenesis, Apoptosis, Autophagy, Carbohydrate Metabolism, Cellular Stress, Cytokine Signaling, Growth Factor Signaling, Innate Immune Response, Insulin Signaling, Neurons and Neurotransmission, Wnt | NS\_MM\_NEUROINFLAM\_V1.0 | Akt1 | mRNA | 0 |  | Wnt;Neurons and Neurotransmission;Insulin Signaling;Innate Immune Response;Growth Factor Signaling;Cytokine Signaling;Cellular Stress;Carbohydrate Metabolism;Autophagy;Apoptosis;Angiogenesis;Adaptive Immune Response | mmu04010;mmu04012;mmu04014;mmu04015;mmu04022;mmu04024;mmu04062;mmu04066;mmu04068;mmu04071;mmu04072;mmu04150;mmu04151;mmu04152;mmu04210;mmu04211;mmu04213;mmu04261;mmu04370;mmu04380;mmu04510;mmu04530;mmu04550;mmu04611;mmu04620;mmu04630;mmu04660;mmu04662;mmu04664;mmu04666;mmu04668;mmu04722;mmu04725;mmu04728;mmu04910;mmu04914;mmu04915;mmu04917;mmu04919;mmu04920;mmu04922;mmu04923;mmu04931;mmu04932;mmu04933;mmu04973;mmu05142;mmu05145;mmu05152;mmu05160;mmu05161;mmu05162;mmu05164;mmu05166;mmu05169;mmu05200;mmu05205;mmu05210;mmu05211;mmu05212;mmu05213;mmu05214;mmu05215;mmu05218;mmu05220;mmu05221;mmu05222;mmu05223;mmu05230;mmu05231 |  | Akt1 | azure4 | 1 | 1.89719626168224 |
  | Creb1-mRNA | -0.0939 | 0.0668 | -0.225 | 0.037 | 0.937 | 0.856 | 1.03 | 0.179 | 1 | loglinear | Adaptive Immune Response, Carbohydrate Metabolism, DNA Damage, Growth Factor Signaling, Innate Immune Response, Neurons and Neurotransmission, Notch | NS\_MM\_NEUROINFLAM\_V1.0 | Creb1 | mRNA | 0 |  | Notch;Neurons and Neurotransmission;Innate Immune Response;Growth Factor Signaling;DNA Damage;Carbohydrate Metabolism;Adaptive Immune Response | mmu04022;mmu04024;mmu04151;mmu04152;mmu04211;mmu04261;mmu04380;mmu04612;mmu04668;mmu04713;mmu04725;mmu04728;mmu04911;mmu04915;mmu04916;mmu04922;mmu04924;mmu04925;mmu04931;mmu05016;mmu05030;mmu05031;mmu05034;mmu05152;mmu05161;mmu05166;mmu05203;mmu05215 |  | Creb1 | azure4 | 1 | -1.40568862275449 |
  | Pik3r1-mRNA | 0.0869 | 0.0713 | -0.0529 | 0.227 | 1.06 | 0.964 | 1.17 | 0.241 | 1 | loglinear | Adaptive Immune Response, Angiogenesis, Apoptosis, Autophagy, Carbohydrate Metabolism, Cytokine Signaling, Growth Factor Signaling, Innate Immune Response, Insulin Signaling, Lipid Metabolism | NS\_MM\_NEUROINFLAM\_V1.0 | Pik3r1 | mRNA | 0 |  | Lipid Metabolism;Insulin Signaling;Innate Immune Response;Growth Factor Signaling;Cytokine Signaling;Carbohydrate Metabolism;Autophagy;Apoptosis;Angiogenesis;Adaptive Immune Response | mmu04012;mmu04014;mmu04015;mmu04022;mmu04024;mmu04062;mmu04066;mmu04068;mmu04070;mmu04071;mmu04072;mmu04150;mmu04151;mmu04152;mmu04210;mmu04211;mmu04213;mmu04261;mmu04370;mmu04380;mmu04510;mmu04550;mmu04611;mmu04620;mmu04630;mmu04650;mmu04660;mmu04662;mmu04664;mmu04666;mmu04668;mmu04670;mmu04722;mmu04725;mmu04750;mmu04810;mmu04910;mmu04914;mmu04915;mmu04917;mmu04919;mmu04921;mmu04923;mmu04930;mmu04931;mmu04932;mmu04933;mmu04960;mmu04973;mmu05100;mmu05142;mmu05145;mmu05146;mmu05160;mmu05161;mmu05162;mmu05164;mmu05166;mmu05169;mmu05200;mmu05203;mmu05205;mmu05210;mmu05211;mmu05212;mmu05213;mmu05214;mmu05215;mmu05218;mmu05220;mmu05221;mmu05222;mmu05223;mmu05230;mmu05231 |  | Pik3r1 | azure4 | 1 | 1.21879382889201 |
  | Slco2b1-mRNA | -0.177 | 0.413 | -0.987 | 0.633 | 0.885 | 0.504 | 1.55 | 0.674 | 1 | lm.nb | Carbohydrate Metabolism | NS\_MM\_NEUROINFLAM\_V1.0 | Slco2b1 | mRNA | 0 |  | Carbohydrate Metabolism |  |  | Slco2b1 | azure4 | 1 | -0.428571428571429 |
  | Sirt1-mRNA | -0.0185 | 0.109 | -0.232 | 0.195 | 0.987 | 0.852 | 1.14 | 0.867 | 1 | loglinear | Carbohydrate Metabolism, Cellular Stress, Epigenetic Regulation | NS\_MM\_NEUROINFLAM\_V1.0 | Sirt1 | mRNA | 0 |  | Epigenetic Regulation;Cellular Stress;Carbohydrate Metabolism | mmu04068;mmu04152;mmu04211;mmu04213;mmu04922;mmu05031;mmu05206 |  | Sirt1 | azure4 | 1 | -0.169724770642202 |

  ##### DE Results - Carbohydrate Metabolism genes - DPI: 2 vs.CTRL

  Table displaying each sample's global significance scores and directed global significance scores as defined in the heatmaps above. The global significance score is calculated as the square root of the mean squared t-statistic for the genes in a gene set, with t-statistics coming from the linear regression underlying our differential expression analysis. The directed global significance score is calculated as the square root of the mean signed squared t-statistic for the genes in a gene set, with t-statistics coming from the linear regression underlying our differential expression analysis.

- **Volcano Plot: DPI: 3 vs.CTRL  
  More Plot Information**


  ##### Volcano Plot: DPI: 3 vs.CTRL

  Volcano plot displaying each gene's -log10(p-value) and log2 fold change for the selected covariate. Highly statistically significant genes fall at the top of the plot, and highly differentially expressed genes fall to either side. Genes within the selected gene set are highlighted in orange. Horizontal lines indicate various False Discovery Rate (FDR) thresholds.
- **DE Results - Carbohydrate Metabolism genes - DPI: 3 vs.CTRL  
  More Plot Information  Download CSV Data**

  | Probe Label | Log2 fold change | std error (log2) | Lower confidence limit (log2) | Upper confidence limit (log2) | Linear fold change | Lower confidence limit (linear) | Upper confidence limit (linear) | P-value | BY.p.value | method | Gene.sets | Codeset.Name | Probe.Label | Analyte.Type | Is.Control | Control.Type | Probe.Annotation | KEGG.Pathways | Cell.Type | Official.Gene.Name | volcanocol | volcanopch | tstats.all |
  | --- | --- | --- | --- | --- | --- | --- | --- | --- | --- | --- | --- | --- | --- | --- | --- | --- | --- | --- | --- | --- | --- | --- | --- |
  | Creb1-mRNA | -0.443 | 0.0668 | -0.574 | -0.312 | 0.735 | 0.672 | 0.805 | 5.69e-06 | 0.000418 | loglinear | Adaptive Immune Response, Carbohydrate Metabolism, DNA Damage, Growth Factor Signaling, Innate Immune Response, Neurons and Neurotransmission, Notch | NS\_MM\_NEUROINFLAM\_V1.0 | Creb1 | mRNA | 0 |  | Notch;Neurons and Neurotransmission;Innate Immune Response;Growth Factor Signaling;DNA Damage;Carbohydrate Metabolism;Adaptive Immune Response | mmu04022;mmu04024;mmu04151;mmu04152;mmu04211;mmu04261;mmu04380;mmu04612;mmu04668;mmu04713;mmu04725;mmu04728;mmu04911;mmu04915;mmu04916;mmu04922;mmu04924;mmu04925;mmu04931;mmu05016;mmu05030;mmu05031;mmu05034;mmu05152;mmu05161;mmu05166;mmu05203;mmu05215 |  | Creb1 | slateblue1 | 16 | -6.63173652694611 |
  | Akt2-mRNA | -0.766 | 0.142 | -1.04 | -0.488 | 0.588 | 0.485 | 0.713 | 5.85e-05 | 0.00265 | loglinear | Adaptive Immune Response, Angiogenesis, Apoptosis, Autophagy, Carbohydrate Metabolism, Cytokine Signaling, Growth Factor Signaling, Innate Immune Response, Insulin Signaling, Neurons and Neurotransmission, Wnt | NS\_MM\_NEUROINFLAM\_V1.0 | Akt2 | mRNA | 0 |  | Wnt;Neurons and Neurotransmission;Insulin Signaling;Innate Immune Response;Growth Factor Signaling;Cytokine Signaling;Carbohydrate Metabolism;Autophagy;Apoptosis;Angiogenesis;Adaptive Immune Response | mmu04010;mmu04012;mmu04014;mmu04015;mmu04022;mmu04024;mmu04062;mmu04066;mmu04068;mmu04071;mmu04072;mmu04150;mmu04151;mmu04152;mmu04210;mmu04211;mmu04213;mmu04261;mmu04370;mmu04380;mmu04510;mmu04530;mmu04550;mmu04611;mmu04620;mmu04630;mmu04660;mmu04662;mmu04664;mmu04666;mmu04668;mmu04722;mmu04725;mmu04728;mmu04910;mmu04914;mmu04915;mmu04917;mmu04919;mmu04920;mmu04922;mmu04923;mmu04931;mmu04932;mmu04933;mmu04973;mmu05142;mmu05145;mmu05152;mmu05160;mmu05161;mmu05162;mmu05164;mmu05166;mmu05169;mmu05200;mmu05205;mmu05210;mmu05211;mmu05212;mmu05213;mmu05214;mmu05215;mmu05218;mmu05220;mmu05221;mmu05222;mmu05223;mmu05230;mmu05231 |  | Akt2 | slateblue1 | 16 | -5.3943661971831 |
  | Igf1r-mRNA | 0.603 | 0.156 | 0.297 | 0.908 | 1.52 | 1.23 | 1.88 | 0.00137 | 0.0347 | lm.nb | Apoptosis, Autophagy, Carbohydrate Metabolism, Growth Factor Signaling, Insulin Signaling | NS\_MM\_NEUROINFLAM\_V1.0 | Igf1r | mRNA | 0 |  | Insulin Signaling;Growth Factor Signaling;Carbohydrate Metabolism;Autophagy;Apoptosis | mmu04014;mmu04015;mmu04066;mmu04068;mmu04114;mmu04144;mmu04151;mmu04152;mmu04211;mmu04213;mmu04510;mmu04520;mmu04550;mmu04730;mmu04913;mmu04914;mmu05200;mmu05202;mmu05205;mmu05214;mmu05215;mmu05218 |  | Igf1r | slateblue2 | 16 | 3.86538461538462 |
  | Pik3ca-mRNA | -0.344 | 0.107 | -0.554 | -0.134 | 0.788 | 0.681 | 0.911 | 0.00549 | 0.111 | lm.nb | Adaptive Immune Response, Angiogenesis, Apoptosis, Autophagy, Carbohydrate Metabolism, Cytokine Signaling, Growth Factor Signaling, Innate Immune Response, Insulin Signaling, Lipid Metabolism | NS\_MM\_NEUROINFLAM\_V1.0 | Pik3ca | mRNA | 0 |  | Lipid Metabolism;Insulin Signaling;Innate Immune Response;Growth Factor Signaling;Cytokine Signaling;Carbohydrate Metabolism;Autophagy;Apoptosis;Angiogenesis;Adaptive Immune Response | mmu00562;mmu04012;mmu04014;mmu04015;mmu04022;mmu04024;mmu04062;mmu04066;mmu04068;mmu04070;mmu04071;mmu04072;mmu04150;mmu04151;mmu04152;mmu04210;mmu04211;mmu04213;mmu04261;mmu04370;mmu04380;mmu04510;mmu04550;mmu04611;mmu04620;mmu04630;mmu04650;mmu04660;mmu04662;mmu04664;mmu04666;mmu04668;mmu04670;mmu04722;mmu04725;mmu04750;mmu04810;mmu04910;mmu04914;mmu04915;mmu04917;mmu04919;mmu04921;mmu04923;mmu04930;mmu04931;mmu04932;mmu04933;mmu04960;mmu04973;mmu05100;mmu05142;mmu05145;mmu05146;mmu05160;mmu05161;mmu05162;mmu05164;mmu05166;mmu05169;mmu05200;mmu05203;mmu05205;mmu05210;mmu05211;mmu05212;mmu05213;mmu05214;mmu05215;mmu05218;mmu05220;mmu05221;mmu05222;mmu05223;mmu05230;mmu05231 |  | Pik3ca | slateblue4 | 16 | -3.21495327102804 |
  | Slco2b1-mRNA | -1.45 | 0.485 | -2.4 | -0.502 | 0.365 | 0.189 | 0.706 | 0.00857 | 0.16 | lm.nb | Carbohydrate Metabolism | NS\_MM\_NEUROINFLAM\_V1.0 | Slco2b1 | mRNA | 0 |  | Carbohydrate Metabolism |  |  | Slco2b1 | slateblue4 | 16 | -2.98969072164948 |
  | Pik3cb-mRNA | -0.207 | 0.116 | -0.435 | 0.0205 | 0.866 | 0.74 | 1.01 | 0.0934 | 1 | loglinear | Adaptive Immune Response, Angiogenesis, Apoptosis, Autophagy, Carbohydrate Metabolism, Cytokine Signaling, Growth Factor Signaling, Innate Immune Response, Insulin Signaling, Lipid Metabolism | NS\_MM\_NEUROINFLAM\_V1.0 | Pik3cb | mRNA | 0 |  | Lipid Metabolism;Insulin Signaling;Innate Immune Response;Growth Factor Signaling;Cytokine Signaling;Carbohydrate Metabolism;Autophagy;Apoptosis;Angiogenesis;Adaptive Immune Response | mmu00562;mmu04012;mmu04014;mmu04015;mmu04022;mmu04024;mmu04062;mmu04066;mmu04068;mmu04070;mmu04071;mmu04072;mmu04150;mmu04151;mmu04152;mmu04210;mmu04211;mmu04213;mmu04261;mmu04370;mmu04380;mmu04510;mmu04550;mmu04611;mmu04620;mmu04630;mmu04650;mmu04660;mmu04662;mmu04664;mmu04666;mmu04668;mmu04670;mmu04722;mmu04725;mmu04750;mmu04810;mmu04910;mmu04914;mmu04915;mmu04917;mmu04919;mmu04921;mmu04923;mmu04930;mmu04931;mmu04932;mmu04933;mmu04960;mmu04973;mmu05100;mmu05142;mmu05145;mmu05146;mmu05160;mmu05161;mmu05162;mmu05164;mmu05166;mmu05169;mmu05200;mmu05203;mmu05205;mmu05210;mmu05211;mmu05212;mmu05213;mmu05214;mmu05215;mmu05218;mmu05220;mmu05221;mmu05222;mmu05223;mmu05230;mmu05231 |  | Pik3cb | azure4 | 1 | -1.78448275862069 |
  | Pik3r1-mRNA | 0.0533 | 0.0713 | -0.0865 | 0.193 | 1.04 | 0.942 | 1.14 | 0.466 | 1 | loglinear | Adaptive Immune Response, Angiogenesis, Apoptosis, Autophagy, Carbohydrate Metabolism, Cytokine Signaling, Growth Factor Signaling, Innate Immune Response, Insulin Signaling, Lipid Metabolism | NS\_MM\_NEUROINFLAM\_V1.0 | Pik3r1 | mRNA | 0 |  | Lipid Metabolism;Insulin Signaling;Innate Immune Response;Growth Factor Signaling;Cytokine Signaling;Carbohydrate Metabolism;Autophagy;Apoptosis;Angiogenesis;Adaptive Immune Response | mmu04012;mmu04014;mmu04015;mmu04022;mmu04024;mmu04062;mmu04066;mmu04068;mmu04070;mmu04071;mmu04072;mmu04150;mmu04151;mmu04152;mmu04210;mmu04211;mmu04213;mmu04261;mmu04370;mmu04380;mmu04510;mmu04550;mmu04611;mmu04620;mmu04630;mmu04650;mmu04660;mmu04662;mmu04664;mmu04666;mmu04668;mmu04670;mmu04722;mmu04725;mmu04750;mmu04810;mmu04910;mmu04914;mmu04915;mmu04917;mmu04919;mmu04921;mmu04923;mmu04930;mmu04931;mmu04932;mmu04933;mmu04960;mmu04973;mmu05100;mmu05142;mmu05145;mmu05146;mmu05160;mmu05161;mmu05162;mmu05164;mmu05166;mmu05169;mmu05200;mmu05203;mmu05205;mmu05210;mmu05211;mmu05212;mmu05213;mmu05214;mmu05215;mmu05218;mmu05220;mmu05221;mmu05222;mmu05223;mmu05230;mmu05231 |  | Pik3r1 | azure4 | 1 | 0.747545582047686 |
  | Akt1-mRNA | 0.0379 | 0.107 | -0.172 | 0.248 | 1.03 | 0.888 | 1.19 | 0.728 | 1 | loglinear | Adaptive Immune Response, Angiogenesis, Apoptosis, Autophagy, Carbohydrate Metabolism, Cellular Stress, Cytokine Signaling, Growth Factor Signaling, Innate Immune Response, Insulin Signaling, Neurons and Neurotransmission, Wnt | NS\_MM\_NEUROINFLAM\_V1.0 | Akt1 | mRNA | 0 |  | Wnt;Neurons and Neurotransmission;Insulin Signaling;Innate Immune Response;Growth Factor Signaling;Cytokine Signaling;Cellular Stress;Carbohydrate Metabolism;Autophagy;Apoptosis;Angiogenesis;Adaptive Immune Response | mmu04010;mmu04012;mmu04014;mmu04015;mmu04022;mmu04024;mmu04062;mmu04066;mmu04068;mmu04071;mmu04072;mmu04150;mmu04151;mmu04152;mmu04210;mmu04211;mmu04213;mmu04261;mmu04370;mmu04380;mmu04510;mmu04530;mmu04550;mmu04611;mmu04620;mmu04630;mmu04660;mmu04662;mmu04664;mmu04666;mmu04668;mmu04722;mmu04725;mmu04728;mmu04910;mmu04914;mmu04915;mmu04917;mmu04919;mmu04920;mmu04922;mmu04923;mmu04931;mmu04932;mmu04933;mmu04973;mmu05142;mmu05145;mmu05152;mmu05160;mmu05161;mmu05162;mmu05164;mmu05166;mmu05169;mmu05200;mmu05205;mmu05210;mmu05211;mmu05212;mmu05213;mmu05214;mmu05215;mmu05218;mmu05220;mmu05221;mmu05222;mmu05223;mmu05230;mmu05231 |  | Akt1 | azure4 | 1 | 0.354205607476636 |
  | Sirt1-mRNA | 0.0367 | 0.109 | -0.177 | 0.25 | 1.03 | 0.885 | 1.19 | 0.74 | 1 | loglinear | Carbohydrate Metabolism, Cellular Stress, Epigenetic Regulation | NS\_MM\_NEUROINFLAM\_V1.0 | Sirt1 | mRNA | 0 |  | Epigenetic Regulation;Cellular Stress;Carbohydrate Metabolism | mmu04068;mmu04152;mmu04211;mmu04213;mmu04922;mmu05031;mmu05206 |  | Sirt1 | azure4 | 1 | 0.336697247706422 |

  ##### DE Results - Carbohydrate Metabolism genes - DPI: 3 vs.CTRL

  Table displaying each sample's global significance scores and directed global significance scores as defined in the heatmaps above. The global significance score is calculated as the square root of the mean squared t-statistic for the genes in a gene set, with t-statistics coming from the linear regression underlying our differential expression analysis. The directed global significance score is calculated as the square root of the mean signed squared t-statistic for the genes in a gene set, with t-statistics coming from the linear regression underlying our differential expression analysis.

DPI:
- DPI: differential expression in 1 vs. baseline of CTRL
- DPI: differential expression in 2 vs. baseline of CTRL
- DPI: differential expression in 3 vs. baseline of CTRL

- **Volcano Plot: DPI: 1 vs.CTRL  
  More Plot Information**


  ##### Volcano Plot: DPI: 1 vs.CTRL

  Volcano plot displaying each gene's -log10(p-value) and log2 fold change for the selected covariate. Highly statistically significant genes fall at the top of the plot, and highly differentially expressed genes fall to either side. Genes within the selected gene set are highlighted in orange. Horizontal lines indicate various False Discovery Rate (FDR) thresholds.
- **DE Results - Cell Cycle genes - DPI: 1 vs.CTRL  
  More Plot Information  Download CSV Data**

  | Probe Label | Log2 fold change | std error (log2) | Lower confidence limit (log2) | Upper confidence limit (log2) | Linear fold change | Lower confidence limit (linear) | Upper confidence limit (linear) | P-value | BY.p.value | method | Gene.sets | Codeset.Name | Probe.Label | Analyte.Type | Is.Control | Control.Type | Probe.Annotation | KEGG.Pathways | Cell.Type | Official.Gene.Name | volcanocol | volcanopch | tstats.all |
  | --- | --- | --- | --- | --- | --- | --- | --- | --- | --- | --- | --- | --- | --- | --- | --- | --- | --- | --- | --- | --- | --- | --- | --- |
  | Mdm2-mRNA | -0.317 | 0.0642 | -0.443 | -0.191 | 0.803 | 0.736 | 0.876 | 0.00015 | 0.0406 | loglinear | Adaptive Immune Response, Apoptosis, Cell Cycle, Cellular Stress, DNA Damage, Growth Factor Signaling, Neurons and Neurotransmission | NS\_MM\_NEUROINFLAM\_V1.0 | Mdm2 | mRNA | 0 |  | Neurons and Neurotransmission;Growth Factor Signaling;DNA Damage;Cellular Stress;Cell Cycle;Apoptosis;Adaptive Immune Response | mmu04068;mmu04110;mmu04115;mmu04120;mmu04144;mmu04151;mmu04919;mmu05169;mmu05200;mmu05202;mmu05203;mmu05205;mmu05206;mmu05214;mmu05215;mmu05218;mmu05219;mmu05220 |  | Mdm2 | slateblue2 | 16 | -4.93769470404984 |
  | Smc1a-mRNA | -0.477 | 0.103 | -0.68 | -0.275 | 0.718 | 0.624 | 0.827 | 0.000287 | 0.0536 | lm.nb | Cell Cycle, DNA Damage | NS\_MM\_NEUROINFLAM\_V1.0 | Smc1a | mRNA | 0 |  | DNA Damage;Cell Cycle | mmu04110;mmu04114 |  | Smc1a | slateblue3 | 16 | -4.63106796116505 |
  | Prkar2b-mRNA | -0.5 | 0.113 | -0.722 | -0.278 | 0.707 | 0.606 | 0.825 | 0.000436 | 0.0616 | loglinear | Apoptosis, Cell Cycle, Growth Factor Signaling | NS\_MM\_NEUROINFLAM\_V1.0 | Prkar2b | mRNA | 0 |  | Growth Factor Signaling;Cell Cycle;Apoptosis | mmu04210;mmu04910 |  | Prkar2b | slateblue3 | 16 | -4.42477876106195 |
  | Tubb4a-mRNA | -0.38 | 0.0956 | -0.567 | -0.192 | 0.769 | 0.675 | 0.875 | 0.00109 | 0.104 | loglinear | Adaptive Immune Response, Apoptosis, Cell Cycle, Neurons and Neurotransmission | NS\_MM\_NEUROINFLAM\_V1.0 | Tubb4a | mRNA | 0 |  | Neurons and Neurotransmission;Cell Cycle;Apoptosis;Adaptive Immune Response | mmu04145;mmu04540 |  | Tubb4a | slateblue4 | 16 | -3.97489539748954 |
  | Rad9a-mRNA | -0.968 | 0.244 | -1.45 | -0.489 | 0.511 | 0.367 | 0.713 | 0.00112 | 0.104 | lm.nb | Cell Cycle, DNA Damage | NS\_MM\_NEUROINFLAM\_V1.0 | Rad9a | mRNA | 0 |  | DNA Damage;Cell Cycle |  |  | Rad9a | slateblue4 | 16 | -3.9672131147541 |
  | Gadd45a-mRNA | -0.491 | 0.13 | -0.746 | -0.237 | 0.711 | 0.596 | 0.849 | 0.00162 | 0.127 | lm.nb | Cell Cycle, DNA Damage, Growth Factor Signaling | NS\_MM\_NEUROINFLAM\_V1.0 | Gadd45a | mRNA | 0 |  | Growth Factor Signaling;DNA Damage;Cell Cycle | mmu04010;mmu04068;mmu04110;mmu04115 |  | Gadd45a | slateblue4 | 16 | -3.77692307692308 |
  | Nbn-mRNA | -0.429 | 0.115 | -0.654 | -0.205 | 0.743 | 0.636 | 0.868 | 0.00176 | 0.129 | loglinear | Cell Cycle, Cellular Stress, DNA Damage | NS\_MM\_NEUROINFLAM\_V1.0 | Nbn | mRNA | 0 |  | DNA Damage;Cellular Stress;Cell Cycle | mmu03440 |  | Nbn | slateblue4 | 16 | -3.7304347826087 |
  | Psmb8-mRNA | 1.36 | 0.365 | 0.649 | 2.08 | 2.57 | 1.57 | 4.23 | 0.00179 | 0.129 | lm.nb | Adaptive Immune Response, Angiogenesis, Apoptosis, Astrocyte Function, Cell Cycle, Cytokine Signaling, Growth Factor Signaling, Inflammatory Signaling, Insulin Signaling, Microglia Function, NF-kB, Wnt | NS\_MM\_NEUROINFLAM\_V1.0 | Psmb8 | mRNA | 0 |  | Wnt;NF-kB;Microglia Function;Insulin Signaling;Inflammatory Signaling;Growth Factor Signaling;Cytokine Signaling;Cell Cycle;Astrocyte Function;Apoptosis;Angiogenesis;Adaptive Immune Response |  |  | Psmb8 | slateblue4 | 16 | 3.72602739726027 |
  | Sumo1-mRNA | -0.424 | 0.115 | -0.65 | -0.198 | 0.745 | 0.637 | 0.871 | 0.00202 | 0.136 | lm.nb | Cell Cycle, Cytokine Signaling, DNA Damage, Inflammatory Signaling | NS\_MM\_NEUROINFLAM\_V1.0 | Sumo1 | mRNA | 0 |  | Inflammatory Signaling;DNA Damage;Cytokine Signaling;Cell Cycle |  |  | Sumo1 | slateblue4 | 16 | -3.68695652173913 |
  | Cdkn1a-mRNA | -0.625 | 0.18 | -0.979 | -0.272 | 0.648 | 0.507 | 0.828 | 0.00317 | 0.18 | lm.nb | Adaptive Immune Response, Cell Cycle, Cellular Stress, Cytokine Signaling, DNA Damage, Growth Factor Signaling | NS\_MM\_NEUROINFLAM\_V1.0 | Cdkn1a | mRNA | 0 |  | Growth Factor Signaling;DNA Damage;Cytokine Signaling;Cellular Stress;Cell Cycle;Adaptive Immune Response | mmu04012;mmu04066;mmu04068;mmu04110;mmu04115;mmu04151;mmu04630;mmu04921;mmu05160;mmu05161;mmu05166;mmu05169;mmu05200;mmu05202;mmu05203;mmu05205;mmu05206;mmu05214;mmu05215;mmu05218;mmu05219;mmu05220 |  | Cdkn1a | slateblue4 | 16 | -3.47222222222222 |
  | Lmna-mRNA | -0.702 | 0.204 | -1.1 | -0.303 | 0.615 | 0.466 | 0.811 | 0.0033 | 0.18 | lm.nb | Apoptosis, Cell Cycle, Microglia Function | NS\_MM\_NEUROINFLAM\_V1.0 | Lmna | mRNA | 0 |  | Microglia Function;Cell Cycle;Apoptosis | mmu05410;mmu05412;mmu05414 |  | Lmna | slateblue4 | 16 | -3.44117647058824 |
  | Blm-mRNA | -1.87 | 0.546 | -2.94 | -0.796 | 0.274 | 0.131 | 0.576 | 0.00353 | 0.184 | loglinear | Cell Cycle, DNA Damage | NS\_MM\_NEUROINFLAM\_V1.0 | Blm | mRNA | 0 |  | DNA Damage;Cell Cycle | mmu03440;mmu03460 |  | Blm | slateblue4 | 16 | -3.42490842490842 |
  | Tubb3-mRNA | -0.52 | 0.158 | -0.83 | -0.21 | 0.697 | 0.562 | 0.865 | 0.00464 | 0.206 | lm.nb | Adaptive Immune Response, Apoptosis, Cell Cycle, Neurons and Neurotransmission | NS\_MM\_NEUROINFLAM\_V1.0 | Tubb3 | mRNA | 0 |  | Neurons and Neurotransmission;Cell Cycle;Apoptosis;Adaptive Immune Response | mmu04145;mmu04540 |  | Tubb3 | slateblue4 | 16 | -3.29113924050633 |
  | Smarca5-mRNA | -0.143 | 0.0451 | -0.232 | -0.0549 | 0.905 | 0.852 | 0.963 | 0.00583 | 0.234 | loglinear | Cell Cycle, DNA Damage, Epigenetic Regulation | NS\_MM\_NEUROINFLAM\_V1.0 | Smarca5 | mRNA | 0 |  | Epigenetic Regulation;DNA Damage;Cell Cycle |  |  | Smarca5 | slateblue4 | 16 | -3.17073170731707 |
  | Hus1-mRNA | -0.528 | 0.172 | -0.866 | -0.191 | 0.693 | 0.549 | 0.876 | 0.00735 | 0.254 | lm.nb | Cell Cycle, DNA Damage | NS\_MM\_NEUROINFLAM\_V1.0 | Hus1 | mRNA | 0 |  | DNA Damage;Cell Cycle |  |  | Hus1 | slateblue4 | 16 | -3.06976744186047 |
  | Cks1b-mRNA | -0.758 | 0.255 | -1.26 | -0.258 | 0.591 | 0.418 | 0.836 | 0.00905 | 0.286 | lm.nb | Cell Cycle | NS\_MM\_NEUROINFLAM\_V1.0 | Cks1b | mRNA | 0 |  | Cell Cycle | mmu05200;mmu05222 |  | Cks1b | slateblue4 | 16 | -2.97254901960784 |
  | Ccni-mRNA | 0.291 | 0.102 | 0.0914 | 0.49 | 1.22 | 1.07 | 1.4 | 0.0114 | 0.34 | lm.nb | Cell Cycle | NS\_MM\_NEUROINFLAM\_V1.0 | Ccni | mRNA | 0 |  | Cell Cycle |  |  | Ccni | slateblue4 | 16 | 2.85294117647059 |
  | Rpa1-mRNA | -0.732 | 0.276 | -1.27 | -0.19 | 0.602 | 0.414 | 0.877 | 0.0175 | 0.43 | lm.nb | Cell Cycle, Cellular Stress, DNA Damage | NS\_MM\_NEUROINFLAM\_V1.0 | Rpa1 | mRNA | 0 |  | DNA Damage;Cellular Stress;Cell Cycle | mmu03030;mmu03420;mmu03430;mmu03440;mmu03460 |  | Rpa1 | slateblue4 | 16 | -2.65217391304348 |
  | Mcm2-mRNA | -0.981 | 0.389 | -1.74 | -0.218 | 0.507 | 0.299 | 0.86 | 0.0227 | 0.508 | lm.nb | Cell Cycle, Inflammatory Signaling | NS\_MM\_NEUROINFLAM\_V1.0 | Mcm2 | mRNA | 0 |  | Inflammatory Signaling;Cell Cycle | mmu03030;mmu04110 |  | Mcm2 | azure4 | 1 | -2.52185089974293 |
  | Rnf8-mRNA | -0.228 | 0.0965 | -0.417 | -0.0389 | 0.854 | 0.749 | 0.973 | 0.0311 | 0.62 | loglinear | Cell Cycle, DNA Damage | NS\_MM\_NEUROINFLAM\_V1.0 | Rnf8 | mRNA | 0 |  | DNA Damage;Cell Cycle |  |  | Rnf8 | azure4 | 1 | -2.36269430051813 |

  ##### DE Results - Cell Cycle genes - DPI: 1 vs.CTRL

  Table displaying each sample's global significance scores and directed global significance scores as defined in the heatmaps above. The global significance score is calculated as the square root of the mean squared t-statistic for the genes in a gene set, with t-statistics coming from the linear regression underlying our differential expression analysis. The directed global significance score is calculated as the square root of the mean signed squared t-statistic for the genes in a gene set, with t-statistics coming from the linear regression underlying our differential expression analysis.

- **Volcano Plot: DPI: 2 vs.CTRL  
  More Plot Information**


  ##### Volcano Plot: DPI: 2 vs.CTRL

  Volcano plot displaying each gene's -log10(p-value) and log2 fold change for the selected covariate. Highly statistically significant genes fall at the top of the plot, and highly differentially expressed genes fall to either side. Genes within the selected gene set are highlighted in orange. Horizontal lines indicate various False Discovery Rate (FDR) thresholds.
- **DE Results - Cell Cycle genes - DPI: 2 vs.CTRL  
  More Plot Information  Download CSV Data**

  | Probe Label | Log2 fold change | std error (log2) | Lower confidence limit (log2) | Upper confidence limit (log2) | Linear fold change | Lower confidence limit (linear) | Upper confidence limit (linear) | P-value | BY.p.value | method | Gene.sets | Codeset.Name | Probe.Label | Analyte.Type | Is.Control | Control.Type | Probe.Annotation | KEGG.Pathways | Cell.Type | Official.Gene.Name | volcanocol | volcanopch | tstats.all |
  | --- | --- | --- | --- | --- | --- | --- | --- | --- | --- | --- | --- | --- | --- | --- | --- | --- | --- | --- | --- | --- | --- | --- | --- |
  | Cdkn1a-mRNA | 2.13 | 0.171 | 1.79 | 2.46 | 4.37 | 3.47 | 5.52 | 1.24e-09 | 8.78e-07 | lm.nb | Adaptive Immune Response, Cell Cycle, Cellular Stress, Cytokine Signaling, DNA Damage, Growth Factor Signaling | NS\_MM\_NEUROINFLAM\_V1.0 | Cdkn1a | mRNA | 0 |  | Growth Factor Signaling;DNA Damage;Cytokine Signaling;Cellular Stress;Cell Cycle;Adaptive Immune Response | mmu04012;mmu04066;mmu04068;mmu04110;mmu04115;mmu04151;mmu04630;mmu04921;mmu05160;mmu05161;mmu05166;mmu05169;mmu05200;mmu05202;mmu05203;mmu05205;mmu05206;mmu05214;mmu05215;mmu05218;mmu05219;mmu05220 |  | Cdkn1a | slateblue1 | 16 | 12.4561403508772 |
  | Prkar2b-mRNA | -1.01 | 0.113 | -1.23 | -0.787 | 0.497 | 0.426 | 0.579 | 1.33e-07 | 3.91e-05 | loglinear | Apoptosis, Cell Cycle, Growth Factor Signaling | NS\_MM\_NEUROINFLAM\_V1.0 | Prkar2b | mRNA | 0 |  | Growth Factor Signaling;Cell Cycle;Apoptosis | mmu04210;mmu04910 |  | Prkar2b | slateblue1 | 16 | -8.93805309734513 |
  | Psmb8-mRNA | 2.23 | 0.352 | 1.54 | 2.92 | 4.7 | 2.91 | 7.59 | 9.89e-06 | 0.000997 | lm.nb | Adaptive Immune Response, Angiogenesis, Apoptosis, Astrocyte Function, Cell Cycle, Cytokine Signaling, Growth Factor Signaling, Inflammatory Signaling, Insulin Signaling, Microglia Function, NF-kB, Wnt | NS\_MM\_NEUROINFLAM\_V1.0 | Psmb8 | mRNA | 0 |  | Wnt;NF-kB;Microglia Function;Insulin Signaling;Inflammatory Signaling;Growth Factor Signaling;Cytokine Signaling;Cell Cycle;Astrocyte Function;Apoptosis;Angiogenesis;Adaptive Immune Response |  |  | Psmb8 | slateblue1 | 16 | 6.33522727272727 |
  | Blm-mRNA | -3.42 | 0.546 | -4.49 | -2.35 | 0.0933 | 0.0444 | 0.196 | 1.13e-05 | 0.0011 | loglinear | Cell Cycle, DNA Damage | NS\_MM\_NEUROINFLAM\_V1.0 | Blm | mRNA | 0 |  | DNA Damage;Cell Cycle | mmu03440;mmu03460 |  | Blm | slateblue1 | 16 | -6.26373626373626 |
  | Smarca5-mRNA | -0.281 | 0.0451 | -0.369 | -0.192 | 0.823 | 0.774 | 0.875 | 1.21e-05 | 0.0011 | loglinear | Cell Cycle, DNA Damage, Epigenetic Regulation | NS\_MM\_NEUROINFLAM\_V1.0 | Smarca5 | mRNA | 0 |  | Epigenetic Regulation;DNA Damage;Cell Cycle |  |  | Smarca5 | slateblue1 | 16 | -6.23059866962306 |
  | Smc1a-mRNA | -0.615 | 0.104 | -0.819 | -0.412 | 0.653 | 0.567 | 0.752 | 2.17e-05 | 0.00178 | lm.nb | Cell Cycle, DNA Damage | NS\_MM\_NEUROINFLAM\_V1.0 | Smc1a | mRNA | 0 |  | DNA Damage;Cell Cycle | mmu04110;mmu04114 |  | Smc1a | slateblue1 | 16 | -5.91346153846154 |
  | Cdk20-mRNA | 0.456 | 0.0865 | 0.286 | 0.625 | 1.37 | 1.22 | 1.54 | 7.63e-05 | 0.00441 | loglinear | Cell Cycle | NS\_MM\_NEUROINFLAM\_V1.0 | Cdk20 | mRNA | 0 |  | Cell Cycle |  |  | Cdk20 | slateblue1 | 16 | 5.27167630057804 |
  | Tubb3-mRNA | -0.799 | 0.158 | -1.11 | -0.489 | 0.575 | 0.463 | 0.713 | 0.000119 | 0.00609 | lm.nb | Adaptive Immune Response, Apoptosis, Cell Cycle, Neurons and Neurotransmission | NS\_MM\_NEUROINFLAM\_V1.0 | Tubb3 | mRNA | 0 |  | Neurons and Neurotransmission;Cell Cycle;Apoptosis;Adaptive Immune Response | mmu04145;mmu04540 |  | Tubb3 | slateblue1 | 16 | -5.05696202531646 |
  | Lmna-mRNA | -0.993 | 0.205 | -1.39 | -0.592 | 0.502 | 0.38 | 0.664 | 0.000178 | 0.00829 | lm.nb | Apoptosis, Cell Cycle, Microglia Function | NS\_MM\_NEUROINFLAM\_V1.0 | Lmna | mRNA | 0 |  | Microglia Function;Cell Cycle;Apoptosis | mmu05410;mmu05412;mmu05414 |  | Lmna | slateblue1 | 16 | -4.84390243902439 |
  | Sumo1-mRNA | -0.529 | 0.115 | -0.755 | -0.303 | 0.693 | 0.593 | 0.811 | 0.000305 | 0.0121 | lm.nb | Cell Cycle, Cytokine Signaling, DNA Damage, Inflammatory Signaling | NS\_MM\_NEUROINFLAM\_V1.0 | Sumo1 | mRNA | 0 |  | Inflammatory Signaling;DNA Damage;Cytokine Signaling;Cell Cycle |  |  | Sumo1 | slateblue2 | 16 | -4.6 |
  | Rad9a-mRNA | -1.13 | 0.249 | -1.62 | -0.642 | 0.457 | 0.326 | 0.641 | 0.000333 | 0.0129 | lm.nb | Cell Cycle, DNA Damage | NS\_MM\_NEUROINFLAM\_V1.0 | Rad9a | mRNA | 0 |  | DNA Damage;Cell Cycle |  |  | Rad9a | slateblue2 | 16 | -4.53815261044177 |
  | Gadd45a-mRNA | -0.561 | 0.13 | -0.817 | -0.305 | 0.678 | 0.568 | 0.809 | 0.000551 | 0.0187 | lm.nb | Cell Cycle, DNA Damage, Growth Factor Signaling | NS\_MM\_NEUROINFLAM\_V1.0 | Gadd45a | mRNA | 0 |  | Growth Factor Signaling;DNA Damage;Cell Cycle | mmu04010;mmu04068;mmu04110;mmu04115 |  | Gadd45a | slateblue2 | 16 | -4.31538461538462 |
  | Nbn-mRNA | -0.476 | 0.115 | -0.7 | -0.251 | 0.719 | 0.616 | 0.84 | 0.00075 | 0.0225 | loglinear | Cell Cycle, Cellular Stress, DNA Damage | NS\_MM\_NEUROINFLAM\_V1.0 | Nbn | mRNA | 0 |  | DNA Damage;Cellular Stress;Cell Cycle | mmu03440 |  | Nbn | slateblue2 | 16 | -4.13913043478261 |
  | Mcm2-mRNA | -1.77 | 0.437 | -2.63 | -0.912 | 0.293 | 0.162 | 0.531 | 0.000938 | 0.0265 | lm.nb | Cell Cycle, Inflammatory Signaling | NS\_MM\_NEUROINFLAM\_V1.0 | Mcm2 | mRNA | 0 |  | Inflammatory Signaling;Cell Cycle | mmu03030;mmu04110 |  | Mcm2 | slateblue2 | 16 | -4.05034324942792 |
  | H2afx-mRNA | -0.214 | 0.0562 | -0.325 | -0.104 | 0.862 | 0.799 | 0.93 | 0.00154 | 0.0372 | loglinear | Cell Cycle, Cellular Stress, DNA Damage, Epigenetic Regulation, Wnt | NS\_MM\_NEUROINFLAM\_V1.0 | H2afx | mRNA | 0 |  | Wnt;Epigenetic Regulation;DNA Damage;Cellular Stress;Cell Cycle | mmu05034;mmu05322 |  | H2afx | slateblue2 | 16 | -3.80782918149466 |
  | Hus1-mRNA | -0.655 | 0.174 | -0.996 | -0.314 | 0.635 | 0.501 | 0.804 | 0.00169 | 0.0397 | lm.nb | Cell Cycle, DNA Damage | NS\_MM\_NEUROINFLAM\_V1.0 | Hus1 | mRNA | 0 |  | DNA Damage;Cell Cycle |  |  | Hus1 | slateblue2 | 16 | -3.76436781609195 |
  | Tubb4a-mRNA | -0.354 | 0.0956 | -0.541 | -0.166 | 0.783 | 0.687 | 0.891 | 0.00195 | 0.0433 | loglinear | Adaptive Immune Response, Apoptosis, Cell Cycle, Neurons and Neurotransmission | NS\_MM\_NEUROINFLAM\_V1.0 | Tubb4a | mRNA | 0 |  | Neurons and Neurotransmission;Cell Cycle;Apoptosis;Adaptive Immune Response | mmu04145;mmu04540 |  | Tubb4a | slateblue2 | 16 | -3.70292887029289 |
  | Anapc15-mRNA | -0.663 | 0.18 | -1.01 | -0.31 | 0.632 | 0.495 | 0.807 | 0.002 | 0.0441 | lm.nb | Cell Cycle, Cellular Stress | NS\_MM\_NEUROINFLAM\_V1.0 | Anapc15 | mRNA | 0 |  | Cellular Stress;Cell Cycle |  |  | Anapc15 | slateblue2 | 16 | -3.68333333333333 |
  | Hdac2-mRNA | -0.184 | 0.057 | -0.295 | -0.0718 | 0.881 | 0.815 | 0.951 | 0.00537 | 0.1 | loglinear | Cell Cycle, Cellular Stress, Epigenetic Regulation, Growth Factor Signaling, Notch | NS\_MM\_NEUROINFLAM\_V1.0 | Hdac2 | mRNA | 0 |  | Notch;Growth Factor Signaling;Epigenetic Regulation;Cellular Stress;Cell Cycle | mmu04110;mmu04213;mmu04330;mmu04919;mmu05016;mmu05034;mmu05169;mmu05200;mmu05202;mmu05203;mmu05220 |  | Hdac2 | slateblue4 | 16 | -3.2280701754386 |
  | Rnf8-mRNA | -0.298 | 0.0965 | -0.487 | -0.109 | 0.813 | 0.713 | 0.927 | 0.0071 | 0.123 | loglinear | Cell Cycle, DNA Damage | NS\_MM\_NEUROINFLAM\_V1.0 | Rnf8 | mRNA | 0 |  | DNA Damage;Cell Cycle |  |  | Rnf8 | slateblue4 | 16 | -3.0880829015544 |

  ##### DE Results - Cell Cycle genes - DPI: 2 vs.CTRL

  Table displaying each sample's global significance scores and directed global significance scores as defined in the heatmaps above. The global significance score is calculated as the square root of the mean squared t-statistic for the genes in a gene set, with t-statistics coming from the linear regression underlying our differential expression analysis. The directed global significance score is calculated as the square root of the mean signed squared t-statistic for the genes in a gene set, with t-statistics coming from the linear regression underlying our differential expression analysis.

- **Volcano Plot: DPI: 3 vs.CTRL  
  More Plot Information**


  ##### Volcano Plot: DPI: 3 vs.CTRL

  Volcano plot displaying each gene's -log10(p-value) and log2 fold change for the selected covariate. Highly statistically significant genes fall at the top of the plot, and highly differentially expressed genes fall to either side. Genes within the selected gene set are highlighted in orange. Horizontal lines indicate various False Discovery Rate (FDR) thresholds.
- **DE Results - Cell Cycle genes - DPI: 3 vs.CTRL  
  More Plot Information  Download CSV Data**

  | Probe Label | Log2 fold change | std error (log2) | Lower confidence limit (log2) | Upper confidence limit (log2) | Linear fold change | Lower confidence limit (linear) | Upper confidence limit (linear) | P-value | BY.p.value | method | Gene.sets | Codeset.Name | Probe.Label | Analyte.Type | Is.Control | Control.Type | Probe.Annotation | KEGG.Pathways | Cell.Type | Official.Gene.Name | volcanocol | volcanopch | tstats.all |
  | --- | --- | --- | --- | --- | --- | --- | --- | --- | --- | --- | --- | --- | --- | --- | --- | --- | --- | --- | --- | --- | --- | --- | --- |
  | Cdkn1a-mRNA | 2.72 | 0.171 | 2.38 | 3.05 | 6.57 | 5.21 | 8.28 | 3.19e-11 | 3.76e-08 | lm.nb | Adaptive Immune Response, Cell Cycle, Cellular Stress, Cytokine Signaling, DNA Damage, Growth Factor Signaling | NS\_MM\_NEUROINFLAM\_V1.0 | Cdkn1a | mRNA | 0 |  | Growth Factor Signaling;DNA Damage;Cytokine Signaling;Cellular Stress;Cell Cycle;Adaptive Immune Response | mmu04012;mmu04066;mmu04068;mmu04110;mmu04115;mmu04151;mmu04630;mmu04921;mmu05160;mmu05161;mmu05166;mmu05169;mmu05200;mmu05202;mmu05203;mmu05205;mmu05206;mmu05214;mmu05215;mmu05218;mmu05219;mmu05220 |  | Cdkn1a | slateblue1 | 16 | 15.906432748538 |
  | Prkar2b-mRNA | -1.06 | 0.113 | -1.28 | -0.834 | 0.481 | 0.412 | 0.561 | 7.17e-08 | 1.41e-05 | loglinear | Apoptosis, Cell Cycle, Growth Factor Signaling | NS\_MM\_NEUROINFLAM\_V1.0 | Prkar2b | mRNA | 0 |  | Growth Factor Signaling;Cell Cycle;Apoptosis | mmu04210;mmu04910 |  | Prkar2b | slateblue1 | 16 | -9.38053097345133 |
  | Psmb8-mRNA | 2.99 | 0.346 | 2.31 | 3.67 | 7.95 | 4.97 | 12.7 | 1.99e-07 | 3.2e-05 | lm.nb | Adaptive Immune Response, Angiogenesis, Apoptosis, Astrocyte Function, Cell Cycle, Cytokine Signaling, Growth Factor Signaling, Inflammatory Signaling, Insulin Signaling, Microglia Function, NF-kB, Wnt | NS\_MM\_NEUROINFLAM\_V1.0 | Psmb8 | mRNA | 0 |  | Wnt;NF-kB;Microglia Function;Insulin Signaling;Inflammatory Signaling;Growth Factor Signaling;Cytokine Signaling;Cell Cycle;Astrocyte Function;Apoptosis;Angiogenesis;Adaptive Immune Response |  |  | Psmb8 | slateblue1 | 16 | 8.64161849710983 |
  | Hdac2-mRNA | -0.452 | 0.057 | -0.564 | -0.341 | 0.731 | 0.676 | 0.79 | 6.19e-07 | 7.29e-05 | loglinear | Cell Cycle, Cellular Stress, Epigenetic Regulation, Growth Factor Signaling, Notch | NS\_MM\_NEUROINFLAM\_V1.0 | Hdac2 | mRNA | 0 |  | Notch;Growth Factor Signaling;Epigenetic Regulation;Cellular Stress;Cell Cycle | mmu04110;mmu04213;mmu04330;mmu04919;mmu05016;mmu05034;mmu05169;mmu05200;mmu05202;mmu05203;mmu05220 |  | Hdac2 | slateblue1 | 16 | -7.92982456140351 |
  | Smc1a-mRNA | -0.597 | 0.104 | -0.8 | -0.393 | 0.661 | 0.574 | 0.762 | 3.03e-05 | 0.00157 | lm.nb | Cell Cycle, DNA Damage | NS\_MM\_NEUROINFLAM\_V1.0 | Smc1a | mRNA | 0 |  | DNA Damage;Cell Cycle | mmu04110;mmu04114 |  | Smc1a | slateblue1 | 16 | -5.74038461538461 |
  | Cdc25a-mRNA | 0.548 | 0.12 | 0.313 | 0.782 | 1.46 | 1.24 | 1.72 | 0.000307 | 0.0108 | lm.nb | Cell Cycle, DNA Damage | NS\_MM\_NEUROINFLAM\_V1.0 | Cdc25a | mRNA | 0 |  | DNA Damage;Cell Cycle | mmu04110;mmu04914;mmu05206 |  | Cdc25a | slateblue2 | 16 | 4.56666666666667 |
  | Fen1-mRNA | 0.646 | 0.141 | 0.369 | 0.922 | 1.56 | 1.29 | 1.9 | 0.000309 | 0.0108 | loglinear | Cell Cycle, DNA Damage | NS\_MM\_NEUROINFLAM\_V1.0 | Fen1 | mRNA | 0 |  | DNA Damage;Cell Cycle | mmu03030;mmu03410;mmu03450 |  | Fen1 | slateblue2 | 16 | 4.58156028368794 |
  | Tubb3-mRNA | -0.664 | 0.158 | -0.975 | -0.354 | 0.631 | 0.509 | 0.782 | 0.000683 | 0.021 | lm.nb | Adaptive Immune Response, Apoptosis, Cell Cycle, Neurons and Neurotransmission | NS\_MM\_NEUROINFLAM\_V1.0 | Tubb3 | mRNA | 0 |  | Neurons and Neurotransmission;Cell Cycle;Apoptosis;Adaptive Immune Response | mmu04145;mmu04540 |  | Tubb3 | slateblue2 | 16 | -4.20253164556962 |
  | Gadd45g-mRNA | 0.993 | 0.244 | 0.515 | 1.47 | 1.99 | 1.43 | 2.77 | 0.000882 | 0.0257 | lm.nb | Cell Cycle, DNA Damage, Growth Factor Signaling | NS\_MM\_NEUROINFLAM\_V1.0 | Gadd45g | mRNA | 0 |  | Growth Factor Signaling;DNA Damage;Cell Cycle | mmu04010;mmu04068;mmu04110;mmu04115 |  | Gadd45g | slateblue2 | 16 | 4.06967213114754 |
  | Rad9a-mRNA | -0.981 | 0.245 | -1.46 | -0.501 | 0.507 | 0.364 | 0.707 | 0.00101 | 0.0279 | lm.nb | Cell Cycle, DNA Damage | NS\_MM\_NEUROINFLAM\_V1.0 | Rad9a | mRNA | 0 |  | DNA Damage;Cell Cycle |  |  | Rad9a | slateblue2 | 16 | -4.00408163265306 |
  | Cdk20-mRNA | 0.282 | 0.0865 | 0.113 | 0.452 | 1.22 | 1.08 | 1.37 | 0.00488 | 0.101 | loglinear | Cell Cycle | NS\_MM\_NEUROINFLAM\_V1.0 | Cdk20 | mRNA | 0 |  | Cell Cycle |  |  | Cdk20 | slateblue4 | 16 | 3.26011560693642 |
  | Tubb4a-mRNA | -0.3 | 0.0956 | -0.488 | -0.113 | 0.812 | 0.713 | 0.925 | 0.00632 | 0.125 | loglinear | Adaptive Immune Response, Apoptosis, Cell Cycle, Neurons and Neurotransmission | NS\_MM\_NEUROINFLAM\_V1.0 | Tubb4a | mRNA | 0 |  | Neurons and Neurotransmission;Cell Cycle;Apoptosis;Adaptive Immune Response | mmu04145;mmu04540 |  | Tubb4a | slateblue4 | 16 | -3.13807531380753 |
  | Rad1-mRNA | -0.413 | 0.134 | -0.674 | -0.151 | 0.751 | 0.627 | 0.901 | 0.00704 | 0.139 | loglinear | Cell Cycle, DNA Damage | NS\_MM\_NEUROINFLAM\_V1.0 | Rad1 | mRNA | 0 |  | DNA Damage;Cell Cycle |  |  | Rad1 | slateblue4 | 16 | -3.08208955223881 |
  | Rad51c-mRNA | -0.675 | 0.249 | -1.16 | -0.187 | 0.626 | 0.446 | 0.878 | 0.0154 | 0.261 | loglinear | Cell Cycle, DNA Damage | NS\_MM\_NEUROINFLAM\_V1.0 | Rad51c | mRNA | 0 |  | DNA Damage;Cell Cycle | mmu03440;mmu03460 |  | Rad51c | slateblue4 | 16 | -2.71084337349398 |
  | Tgfb1-mRNA | 1.02 | 0.383 | 0.269 | 1.77 | 2.03 | 1.2 | 3.41 | 0.017 | 0.281 | lm.nb | Cell Cycle, Cytokine Signaling, Growth Factor Signaling, Matrix Remodeling, Microglia Function | NS\_MM\_NEUROINFLAM\_V1.0 | Tgfb1 | mRNA | 0 |  | Microglia Function;Matrix Remodeling;Growth Factor Signaling;Cytokine Signaling;Cell Cycle | mmu04010;mmu04060;mmu04068;mmu04110;mmu04144;mmu04350;mmu04380;mmu04390;mmu04672;mmu04932;mmu04933;mmu05140;mmu05142;mmu05144;mmu05145;mmu05146;mmu05152;mmu05161;mmu05166;mmu05200;mmu05205;mmu05210;mmu05211;mmu05212;mmu05220;mmu05321;mmu05323;mmu05410;mmu05414 |  | Tgfb1 | slateblue4 | 16 | 2.66318537859008 |
  | Prkaca-mRNA | -0.149 | 0.0577 | -0.262 | -0.0355 | 0.902 | 0.834 | 0.976 | 0.0203 | 0.322 | loglinear | Adaptive Immune Response, Angiogenesis, Apoptosis, Autophagy, Cell Cycle, Cytokine Signaling, Growth Factor Signaling, Lipid Metabolism, Neurons and Neurotransmission, Wnt | NS\_MM\_NEUROINFLAM\_V1.0 | Prkaca | mRNA | 0 |  | Wnt;Neurons and Neurotransmission;Lipid Metabolism;Growth Factor Signaling;Cytokine Signaling;Cell Cycle;Autophagy;Apoptosis;Angiogenesis;Adaptive Immune Response | mmu04010;mmu04014;mmu04020;mmu04024;mmu04062;mmu04114;mmu04210;mmu04211;mmu04213;mmu04261;mmu04270;mmu04310;mmu04540;mmu04611;mmu04713;mmu04720;mmu04723;mmu04724;mmu04725;mmu04726;mmu04728;mmu04750;mmu04910;mmu04911;mmu04912;mmu04913;mmu04914;mmu04915;mmu04916;mmu04919;mmu04921;mmu04922;mmu04923;mmu04924;mmu04925;mmu05012;mmu05020;mmu05030;mmu05031;mmu05034;mmu05146;mmu05166;mmu05169;mmu05200;mmu05203;mmu05205;mmu05414 |  | Prkaca | slateblue4 | 16 | -2.58232235701906 |
  | Ccng2-mRNA | -0.306 | 0.12 | -0.541 | -0.0717 | 0.809 | 0.687 | 0.951 | 0.021 | 0.33 | loglinear | Cell Cycle, DNA Damage | NS\_MM\_NEUROINFLAM\_V1.0 | Ccng2 | mRNA | 0 |  | DNA Damage;Cell Cycle | mmu04068;mmu04115 |  | Ccng2 | slateblue4 | 16 | -2.55 |
  | Bard1-mRNA | -0.922 | 0.378 | -1.66 | -0.181 | 0.528 | 0.316 | 0.882 | 0.0267 | 0.399 | lm.nb | Cell Cycle, DNA Damage | NS\_MM\_NEUROINFLAM\_V1.0 | Bard1 | mRNA | 0 |  | DNA Damage;Cell Cycle |  |  | Bard1 | slateblue4 | 16 | -2.43915343915344 |
  | Hus1-mRNA | -0.359 | 0.17 | -0.692 | -0.0255 | 0.78 | 0.619 | 0.982 | 0.0509 | 0.659 | lm.nb | Cell Cycle, DNA Damage | NS\_MM\_NEUROINFLAM\_V1.0 | Hus1 | mRNA | 0 |  | DNA Damage;Cell Cycle |  |  | Hus1 | azure4 | 1 | -2.11176470588235 |
  | Myc-mRNA | 0.677 | 0.331 | 0.0278 | 1.33 | 1.6 | 1.02 | 2.51 | 0.0578 | 0.734 | lm.nb | Apoptosis, Cell Cycle, Cytokine Signaling, Growth Factor Signaling, Notch, Wnt | NS\_MM\_NEUROINFLAM\_V1.0 | Myc | mRNA | 0 |  | Wnt;Notch;Growth Factor Signaling;Cytokine Signaling;Cell Cycle;Apoptosis | mmu04010;mmu04012;mmu04110;mmu04151;mmu04310;mmu04350;mmu04390;mmu04550;mmu04630;mmu04919;mmu05161;mmu05166;mmu05169;mmu05200;mmu05202;mmu05205;mmu05206;mmu05210;mmu05213;mmu05216;mmu05219;mmu05220;mmu05221;mmu05222;mmu05230 |  | Myc | azure4 | 1 | 2.04531722054381 |

  ##### DE Results - Cell Cycle genes - DPI: 3 vs.CTRL

  Table displaying each sample's global significance scores and directed global significance scores as defined in the heatmaps above. The global significance score is calculated as the square root of the mean squared t-statistic for the genes in a gene set, with t-statistics coming from the linear regression underlying our differential expression analysis. The directed global significance score is calculated as the square root of the mean signed squared t-statistic for the genes in a gene set, with t-statistics coming from the linear regression underlying our differential expression analysis.

DPI:
- DPI: differential expression in 1 vs. baseline of CTRL
- DPI: differential expression in 2 vs. baseline of CTRL
- DPI: differential expression in 3 vs. baseline of CTRL

- **Volcano Plot: DPI: 1 vs.CTRL  
  More Plot Information**


  ##### Volcano Plot: DPI: 1 vs.CTRL

  Volcano plot displaying each gene's -log10(p-value) and log2 fold change for the selected covariate. Highly statistically significant genes fall at the top of the plot, and highly differentially expressed genes fall to either side. Genes within the selected gene set are highlighted in orange. Horizontal lines indicate various False Discovery Rate (FDR) thresholds.
- **DE Results - Cellular Stress genes - DPI: 1 vs.CTRL  
  More Plot Information  Download CSV Data**

  | Probe Label | Log2 fold change | std error (log2) | Lower confidence limit (log2) | Upper confidence limit (log2) | Linear fold change | Lower confidence limit (linear) | Upper confidence limit (linear) | P-value | BY.p.value | method | Gene.sets | Codeset.Name | Probe.Label | Analyte.Type | Is.Control | Control.Type | Probe.Annotation | KEGG.Pathways | Cell.Type | Official.Gene.Name | volcanocol | volcanopch | tstats.all |
  | --- | --- | --- | --- | --- | --- | --- | --- | --- | --- | --- | --- | --- | --- | --- | --- | --- | --- | --- | --- | --- | --- | --- | --- |
  | Fos-mRNA | -1.75 | 0.299 | -2.34 | -1.17 | 0.297 | 0.198 | 0.446 | 2.43e-05 | 0.0143 | lm.nb | Adaptive Immune Response, Cellular Stress, Growth Factor Signaling, Innate Immune Response, Neurons and Neurotransmission | NS\_MM\_NEUROINFLAM\_V1.0 | Fos | mRNA | 0 |  | Neurons and Neurotransmission;Innate Immune Response;Growth Factor Signaling;Cellular Stress;Adaptive Immune Response | mmu04010;mmu04024;mmu04380;mmu04620;mmu04660;mmu04662;mmu04668;mmu04713;mmu04725;mmu04728;mmu04915;mmu04917;mmu04921;mmu05031;mmu05132;mmu05133;mmu05140;mmu05142;mmu05161;mmu05166;mmu05168;mmu05200;mmu05210;mmu05231;mmu05323 |  | Fos | slateblue2 | 16 | -5.85284280936455 |
  | Mapt-mRNA | -0.222 | 0.0425 | -0.306 | -0.139 | 0.857 | 0.809 | 0.908 | 8.22e-05 | 0.0321 | loglinear | Apoptosis, Cellular Stress, Growth Factor Signaling, Neurons and Neurotransmission | NS\_MM\_NEUROINFLAM\_V1.0 | Mapt | mRNA | 0 |  | Neurons and Neurotransmission;Growth Factor Signaling;Cellular Stress;Apoptosis | mmu04010;mmu05010 |  | Mapt | slateblue2 | 16 | -5.22352941176471 |
  | Mdm2-mRNA | -0.317 | 0.0642 | -0.443 | -0.191 | 0.803 | 0.736 | 0.876 | 0.00015 | 0.0406 | loglinear | Adaptive Immune Response, Apoptosis, Cell Cycle, Cellular Stress, DNA Damage, Growth Factor Signaling, Neurons and Neurotransmission | NS\_MM\_NEUROINFLAM\_V1.0 | Mdm2 | mRNA | 0 |  | Neurons and Neurotransmission;Growth Factor Signaling;DNA Damage;Cellular Stress;Cell Cycle;Apoptosis;Adaptive Immune Response | mmu04068;mmu04110;mmu04115;mmu04120;mmu04144;mmu04151;mmu04919;mmu05169;mmu05200;mmu05202;mmu05203;mmu05205;mmu05206;mmu05214;mmu05215;mmu05218;mmu05219;mmu05220 |  | Mdm2 | slateblue2 | 16 | -4.93769470404984 |
  | Parp1-mRNA | -0.49 | 0.108 | -0.703 | -0.278 | 0.712 | 0.614 | 0.825 | 0.000348 | 0.0564 | loglinear | Apoptosis, Cellular Stress, DNA Damage, NF-kB | NS\_MM\_NEUROINFLAM\_V1.0 | Parp1 | mRNA | 0 |  | NF-kB;DNA Damage;Cellular Stress;Apoptosis | mmu03410;mmu04064 |  | Parp1 | slateblue3 | 16 | -4.53703703703704 |
  | Mapk10-mRNA | -0.539 | 0.138 | -0.81 | -0.269 | 0.688 | 0.57 | 0.83 | 0.00126 | 0.111 | lm.nb | Adaptive Immune Response, Apoptosis, Autophagy, Cellular Stress, Growth Factor Signaling, Innate Immune Response, Wnt | NS\_MM\_NEUROINFLAM\_V1.0 | Mapk10 | mRNA | 0 |  | Wnt;Innate Immune Response;Growth Factor Signaling;Cellular Stress;Autophagy;Apoptosis;Adaptive Immune Response | mmu04010;mmu04012;mmu04014;mmu04024;mmu04068;mmu04071;mmu04141;mmu04310;mmu04380;mmu04510;mmu04620;mmu04621;mmu04622;mmu04664;mmu04668;mmu04722;mmu04723;mmu04728;mmu04750;mmu04910;mmu04912;mmu04914;mmu04917;mmu04920;mmu04930;mmu04931;mmu04932;mmu04933;mmu05132;mmu05133;mmu05142;mmu05145;mmu05152;mmu05160;mmu05161;mmu05164;mmu05168;mmu05169;mmu05200;mmu05210;mmu05212;mmu05231 |  | Mapk10 | slateblue4 | 16 | -3.90579710144928 |
  | Nbn-mRNA | -0.429 | 0.115 | -0.654 | -0.205 | 0.743 | 0.636 | 0.868 | 0.00176 | 0.129 | loglinear | Cell Cycle, Cellular Stress, DNA Damage | NS\_MM\_NEUROINFLAM\_V1.0 | Nbn | mRNA | 0 |  | DNA Damage;Cellular Stress;Cell Cycle | mmu03440 |  | Nbn | slateblue4 | 16 | -3.7304347826087 |
  | Cdkn1a-mRNA | -0.625 | 0.18 | -0.979 | -0.272 | 0.648 | 0.507 | 0.828 | 0.00317 | 0.18 | lm.nb | Adaptive Immune Response, Cell Cycle, Cellular Stress, Cytokine Signaling, DNA Damage, Growth Factor Signaling | NS\_MM\_NEUROINFLAM\_V1.0 | Cdkn1a | mRNA | 0 |  | Growth Factor Signaling;DNA Damage;Cytokine Signaling;Cellular Stress;Cell Cycle;Adaptive Immune Response | mmu04012;mmu04066;mmu04068;mmu04110;mmu04115;mmu04151;mmu04630;mmu04921;mmu05160;mmu05161;mmu05166;mmu05169;mmu05200;mmu05202;mmu05203;mmu05205;mmu05206;mmu05214;mmu05215;mmu05218;mmu05219;mmu05220 |  | Cdkn1a | slateblue4 | 16 | -3.47222222222222 |
  | Mmp14-mRNA | -1.57 | 0.459 | -2.47 | -0.668 | 0.338 | 0.181 | 0.629 | 0.00354 | 0.184 | lm.nb | Cellular Stress, Innate Immune Response, Matrix Remodeling, Microglia Function | NS\_MM\_NEUROINFLAM\_V1.0 | Mmp14 | mRNA | 0 |  | Microglia Function;Matrix Remodeling;Innate Immune Response;Cellular Stress | mmu04668;mmu04912 |  | Mmp14 | slateblue4 | 16 | -3.42047930283224 |
  | Suz12-mRNA | -0.439 | 0.13 | -0.693 | -0.185 | 0.738 | 0.619 | 0.88 | 0.00375 | 0.184 | lm.nb | Cellular Stress, Epigenetic Regulation | NS\_MM\_NEUROINFLAM\_V1.0 | Suz12 | mRNA | 0 |  | Epigenetic Regulation;Cellular Stress |  |  | Suz12 | slateblue4 | 16 | -3.37692307692308 |
  | Map2k4-mRNA | -0.433 | 0.133 | -0.693 | -0.173 | 0.741 | 0.619 | 0.887 | 0.00484 | 0.206 | lm.nb | Adaptive Immune Response, Apoptosis, Cellular Stress, Growth Factor Signaling, Innate Immune Response | NS\_MM\_NEUROINFLAM\_V1.0 | Map2k4 | mRNA | 0 |  | Innate Immune Response;Growth Factor Signaling;Cellular Stress;Apoptosis;Adaptive Immune Response | mmu04010;mmu04012;mmu04620;mmu04664;mmu04668;mmu04912;mmu05142;mmu05161;mmu05164;mmu05166;mmu05169 |  | Map2k4 | slateblue4 | 16 | -3.25563909774436 |
  | Bag4-mRNA | -0.576 | 0.178 | -0.925 | -0.227 | 0.671 | 0.527 | 0.855 | 0.00523 | 0.215 | lm.nb | Apoptosis, Cellular Stress, Innate Immune Response | NS\_MM\_NEUROINFLAM\_V1.0 | Bag4 | mRNA | 0 |  | Innate Immune Response;Cellular Stress;Apoptosis | mmu04668 |  | Bag4 | slateblue4 | 16 | -3.23595505617978 |
  | Pink1-mRNA | -0.79 | 0.249 | -1.28 | -0.302 | 0.578 | 0.412 | 0.811 | 0.00592 | 0.235 | lm.nb | Autophagy, Cellular Stress | NS\_MM\_NEUROINFLAM\_V1.0 | Pink1 | mRNA | 0 |  | Cellular Stress;Autophagy | mmu05012 |  | Pink1 | slateblue4 | 16 | -3.17269076305221 |
  | Ambra1-mRNA | -0.503 | 0.165 | -0.826 | -0.179 | 0.706 | 0.564 | 0.883 | 0.00768 | 0.258 | lm.nb | Autophagy, Cellular Stress | NS\_MM\_NEUROINFLAM\_V1.0 | Ambra1 | mRNA | 0 |  | Cellular Stress;Autophagy |  |  | Ambra1 | slateblue4 | 16 | -3.04848484848485 |
  | Mapk14-mRNA | -1.06 | 0.362 | -1.77 | -0.355 | 0.478 | 0.293 | 0.782 | 0.00961 | 0.3 | lm.nb | Angiogenesis, Autophagy, Cellular Stress, Growth Factor Signaling, Innate Immune Response | NS\_MM\_NEUROINFLAM\_V1.0 | Mapk14 | mRNA | 0 |  | Innate Immune Response;Growth Factor Signaling;Cellular Stress;Autophagy;Angiogenesis | mmu04010;mmu04015;mmu04068;mmu04071;mmu04261;mmu04370;mmu04380;mmu04550;mmu04611;mmu04620;mmu04621;mmu04622;mmu04660;mmu04664;mmu04668;mmu04670;mmu04722;mmu04723;mmu04728;mmu04750;mmu04912;mmu04914;mmu04917;mmu04933;mmu05014;mmu05132;mmu05133;mmu05140;mmu05142;mmu05145;mmu05152;mmu05160;mmu05164;mmu05169;mmu05205 |  | Mapk14 | slateblue4 | 16 | -2.92817679558011 |
  | Bad-mRNA | -0.533 | 0.188 | -0.901 | -0.164 | 0.691 | 0.535 | 0.892 | 0.012 | 0.351 | lm.nb | Adaptive Immune Response, Apoptosis, Autophagy, Cellular Stress, Growth Factor Signaling | NS\_MM\_NEUROINFLAM\_V1.0 | Bad | mRNA | 0 |  | Growth Factor Signaling;Cellular Stress;Autophagy;Apoptosis;Adaptive Immune Response | mmu04012;mmu04014;mmu04022;mmu04024;mmu04151;mmu04210;mmu04370;mmu04510;mmu04722;mmu04910;mmu04919;mmu05010;mmu05014;mmu05145;mmu05152;mmu05160;mmu05161;mmu05200;mmu05203;mmu05210;mmu05212;mmu05213;mmu05215;mmu05218;mmu05220;mmu05221;mmu05223 |  | Bad | slateblue4 | 16 | -2.83510638297872 |
  | Sod2-mRNA | -0.226 | 0.0804 | -0.383 | -0.0684 | 0.855 | 0.767 | 0.954 | 0.0125 | 0.357 | loglinear | Cellular Stress, Microglia Function | NS\_MM\_NEUROINFLAM\_V1.0 | Sod2 | mRNA | 0 |  | Microglia Function;Cellular Stress | mmu04068;mmu04211;mmu04213;mmu05016 |  | Sod2 | slateblue4 | 16 | -2.81094527363184 |
  | Hdac6-mRNA | -0.503 | 0.18 | -0.856 | -0.151 | 0.705 | 0.552 | 0.901 | 0.0129 | 0.364 | lm.nb | Cellular Stress, Epigenetic Regulation, Notch | NS\_MM\_NEUROINFLAM\_V1.0 | Hdac6 | mRNA | 0 |  | Notch;Epigenetic Regulation;Cellular Stress | mmu05034;mmu05203 |  | Hdac6 | slateblue4 | 16 | -2.79444444444444 |
  | Txnrd1-mRNA | -0.714 | 0.256 | -1.22 | -0.213 | 0.61 | 0.431 | 0.863 | 0.013 | 0.364 | lm.nb | Cellular Stress | NS\_MM\_NEUROINFLAM\_V1.0 | Txnrd1 | mRNA | 0 |  | Cellular Stress |  |  | Txnrd1 | slateblue4 | 16 | -2.7890625 |
  | Eed-mRNA | -0.131 | 0.0478 | -0.225 | -0.0377 | 0.913 | 0.855 | 0.974 | 0.0143 | 0.385 | loglinear | Cellular Stress, Epigenetic Regulation | NS\_MM\_NEUROINFLAM\_V1.0 | Eed | mRNA | 0 |  | Epigenetic Regulation;Cellular Stress |  |  | Eed | slateblue4 | 16 | -2.74058577405858 |
  | Casp3-mRNA | -0.413 | 0.154 | -0.716 | -0.111 | 0.751 | 0.609 | 0.926 | 0.0165 | 0.417 | lm.nb | Apoptosis, Cellular Stress, DNA Damage, Growth Factor Signaling, Innate Immune Response, Matrix Remodeling | NS\_MM\_NEUROINFLAM\_V1.0 | Casp3 | mRNA | 0 |  | Matrix Remodeling;Innate Immune Response;Growth Factor Signaling;DNA Damage;Cellular Stress;Apoptosis | mmu04010;mmu04115;mmu04210;mmu04650;mmu04668;mmu04726;mmu04932;mmu04933;mmu05010;mmu05012;mmu05014;mmu05016;mmu05133;mmu05134;mmu05145;mmu05146;mmu05152;mmu05161;mmu05168;mmu05200;mmu05203;mmu05205;mmu05206;mmu05210;mmu05416 |  | Casp3 | slateblue4 | 16 | -2.68181818181818 |

  ##### DE Results - Cellular Stress genes - DPI: 1 vs.CTRL

  Table displaying each sample's global significance scores and directed global significance scores as defined in the heatmaps above. The global significance score is calculated as the square root of the mean squared t-statistic for the genes in a gene set, with t-statistics coming from the linear regression underlying our differential expression analysis. The directed global significance score is calculated as the square root of the mean signed squared t-statistic for the genes in a gene set, with t-statistics coming from the linear regression underlying our differential expression analysis.

- **Volcano Plot: DPI: 2 vs.CTRL  
  More Plot Information**


  ##### Volcano Plot: DPI: 2 vs.CTRL

  Volcano plot displaying each gene's -log10(p-value) and log2 fold change for the selected covariate. Highly statistically significant genes fall at the top of the plot, and highly differentially expressed genes fall to either side. Genes within the selected gene set are highlighted in orange. Horizontal lines indicate various False Discovery Rate (FDR) thresholds.
- **DE Results - Cellular Stress genes - DPI: 2 vs.CTRL  
  More Plot Information  Download CSV Data**

  | Probe Label | Log2 fold change | std error (log2) | Lower confidence limit (log2) | Upper confidence limit (log2) | Linear fold change | Lower confidence limit (linear) | Upper confidence limit (linear) | P-value | BY.p.value | method | Gene.sets | Codeset.Name | Probe.Label | Analyte.Type | Is.Control | Control.Type | Probe.Annotation | KEGG.Pathways | Cell.Type | Official.Gene.Name | volcanocol | volcanopch | tstats.all |
  | --- | --- | --- | --- | --- | --- | --- | --- | --- | --- | --- | --- | --- | --- | --- | --- | --- | --- | --- | --- | --- | --- | --- | --- |
  | Cdkn1a-mRNA | 2.13 | 0.171 | 1.79 | 2.46 | 4.37 | 3.47 | 5.52 | 1.24e-09 | 8.78e-07 | lm.nb | Adaptive Immune Response, Cell Cycle, Cellular Stress, Cytokine Signaling, DNA Damage, Growth Factor Signaling | NS\_MM\_NEUROINFLAM\_V1.0 | Cdkn1a | mRNA | 0 |  | Growth Factor Signaling;DNA Damage;Cytokine Signaling;Cellular Stress;Cell Cycle;Adaptive Immune Response | mmu04012;mmu04066;mmu04068;mmu04110;mmu04115;mmu04151;mmu04630;mmu04921;mmu05160;mmu05161;mmu05166;mmu05169;mmu05200;mmu05202;mmu05203;mmu05205;mmu05206;mmu05214;mmu05215;mmu05218;mmu05219;mmu05220 |  | Cdkn1a | slateblue1 | 16 | 12.4561403508772 |
  | Mapt-mRNA | -0.314 | 0.0425 | -0.397 | -0.23 | 0.805 | 0.759 | 0.852 | 1.56e-06 | 0.000239 | loglinear | Apoptosis, Cellular Stress, Growth Factor Signaling, Neurons and Neurotransmission | NS\_MM\_NEUROINFLAM\_V1.0 | Mapt | mRNA | 0 |  | Neurons and Neurotransmission;Growth Factor Signaling;Cellular Stress;Apoptosis | mmu04010;mmu05010 |  | Mapt | slateblue1 | 16 | -7.38823529411765 |
  | Fos-mRNA | -2.04 | 0.3 | -2.62 | -1.45 | 0.244 | 0.162 | 0.367 | 4.41e-06 | 0.000585 | lm.nb | Adaptive Immune Response, Cellular Stress, Growth Factor Signaling, Innate Immune Response, Neurons and Neurotransmission | NS\_MM\_NEUROINFLAM\_V1.0 | Fos | mRNA | 0 |  | Neurons and Neurotransmission;Innate Immune Response;Growth Factor Signaling;Cellular Stress;Adaptive Immune Response | mmu04010;mmu04024;mmu04380;mmu04620;mmu04660;mmu04662;mmu04668;mmu04713;mmu04725;mmu04728;mmu04915;mmu04917;mmu04921;mmu05031;mmu05132;mmu05133;mmu05140;mmu05142;mmu05161;mmu05166;mmu05168;mmu05200;mmu05210;mmu05231;mmu05323 |  | Fos | slateblue1 | 16 | -6.8 |
  | Atg3-mRNA | -0.467 | 0.0705 | -0.605 | -0.329 | 0.723 | 0.657 | 0.796 | 5.81e-06 | 0.000708 | loglinear | Autophagy, Cellular Stress | NS\_MM\_NEUROINFLAM\_V1.0 | Atg3 | mRNA | 0 |  | Cellular Stress;Autophagy | mmu04140 |  | Atg3 | slateblue1 | 16 | -6.62411347517731 |
  | Apoe-mRNA | 0.418 | 0.0826 | 0.256 | 0.58 | 1.34 | 1.19 | 1.49 | 0.000116 | 0.00609 | loglinear | Astrocyte Function, Cellular Stress, Lipid Metabolism, Microglia Function, Neurons and Neurotransmission | NS\_MM\_NEUROINFLAM\_V1.0 | Apoe | mRNA | 0 |  | Neurons and Neurotransmission;Microglia Function;Lipid Metabolism;Cellular Stress;Astrocyte Function | mmu05010 |  | Apoe | slateblue1 | 16 | 5.06053268765133 |
  | Ulk1-mRNA | 0.38 | 0.0766 | 0.23 | 0.531 | 1.3 | 1.17 | 1.44 | 0.000141 | 0.0069 | loglinear | Autophagy, Cellular Stress | NS\_MM\_NEUROINFLAM\_V1.0 | Ulk1 | mRNA | 0 |  | Cellular Stress;Autophagy | mmu04140;mmu04150;mmu04152;mmu04211 |  | Ulk1 | slateblue1 | 16 | 4.96083550913838 |
  | Mapk10-mRNA | -0.673 | 0.138 | -0.943 | -0.402 | 0.627 | 0.52 | 0.757 | 0.000172 | 0.0082 | lm.nb | Adaptive Immune Response, Apoptosis, Autophagy, Cellular Stress, Growth Factor Signaling, Innate Immune Response, Wnt | NS\_MM\_NEUROINFLAM\_V1.0 | Mapk10 | mRNA | 0 |  | Wnt;Innate Immune Response;Growth Factor Signaling;Cellular Stress;Autophagy;Apoptosis;Adaptive Immune Response | mmu04010;mmu04012;mmu04014;mmu04024;mmu04068;mmu04071;mmu04141;mmu04310;mmu04380;mmu04510;mmu04620;mmu04621;mmu04622;mmu04664;mmu04668;mmu04722;mmu04723;mmu04728;mmu04750;mmu04910;mmu04912;mmu04914;mmu04917;mmu04920;mmu04930;mmu04931;mmu04932;mmu04933;mmu05132;mmu05133;mmu05142;mmu05145;mmu05152;mmu05160;mmu05161;mmu05164;mmu05168;mmu05169;mmu05200;mmu05210;mmu05212;mmu05231 |  | Mapk10 | slateblue1 | 16 | -4.8768115942029 |
  | Ets2-mRNA | 0.438 | 0.0908 | 0.26 | 0.616 | 1.36 | 1.2 | 1.53 | 0.000186 | 0.00851 | loglinear | Cellular Stress, Growth Factor Signaling, Microglia Function | NS\_MM\_NEUROINFLAM\_V1.0 | Ets2 | mRNA | 0 |  | Microglia Function;Growth Factor Signaling;Cellular Stress | mmu04014;mmu05166 |  | Ets2 | slateblue1 | 16 | 4.82378854625551 |
  | Suz12-mRNA | -0.578 | 0.13 | -0.832 | -0.323 | 0.67 | 0.562 | 0.799 | 0.000406 | 0.0148 | lm.nb | Cellular Stress, Epigenetic Regulation | NS\_MM\_NEUROINFLAM\_V1.0 | Suz12 | mRNA | 0 |  | Epigenetic Regulation;Cellular Stress |  |  | Suz12 | slateblue2 | 16 | -4.44615384615385 |
  | Mmp14-mRNA | -1.97 | 0.472 | -2.9 | -1.05 | 0.255 | 0.134 | 0.484 | 0.000713 | 0.022 | lm.nb | Cellular Stress, Innate Immune Response, Matrix Remodeling, Microglia Function | NS\_MM\_NEUROINFLAM\_V1.0 | Mmp14 | mRNA | 0 |  | Microglia Function;Matrix Remodeling;Innate Immune Response;Cellular Stress | mmu04668;mmu04912 |  | Mmp14 | slateblue2 | 16 | -4.17372881355932 |
  | Ehmt2-mRNA | -0.211 | 0.0508 | -0.311 | -0.112 | 0.864 | 0.806 | 0.925 | 0.000732 | 0.0223 | loglinear | Cellular Stress, Epigenetic Regulation | NS\_MM\_NEUROINFLAM\_V1.0 | Ehmt2 | mRNA | 0 |  | Epigenetic Regulation;Cellular Stress | mmu00310;mmu04211 |  | Ehmt2 | slateblue2 | 16 | -4.15354330708661 |
  | Nbn-mRNA | -0.476 | 0.115 | -0.7 | -0.251 | 0.719 | 0.616 | 0.84 | 0.00075 | 0.0225 | loglinear | Cell Cycle, Cellular Stress, DNA Damage | NS\_MM\_NEUROINFLAM\_V1.0 | Nbn | mRNA | 0 |  | DNA Damage;Cellular Stress;Cell Cycle | mmu03440 |  | Nbn | slateblue2 | 16 | -4.13913043478261 |
  | Casp3-mRNA | -0.652 | 0.157 | -0.96 | -0.344 | 0.636 | 0.514 | 0.788 | 0.000753 | 0.0225 | lm.nb | Apoptosis, Cellular Stress, DNA Damage, Growth Factor Signaling, Innate Immune Response, Matrix Remodeling | NS\_MM\_NEUROINFLAM\_V1.0 | Casp3 | mRNA | 0 |  | Matrix Remodeling;Innate Immune Response;Growth Factor Signaling;DNA Damage;Cellular Stress;Apoptosis | mmu04010;mmu04115;mmu04210;mmu04650;mmu04668;mmu04726;mmu04932;mmu04933;mmu05010;mmu05012;mmu05014;mmu05016;mmu05133;mmu05134;mmu05145;mmu05146;mmu05152;mmu05161;mmu05168;mmu05200;mmu05203;mmu05205;mmu05206;mmu05210;mmu05416 |  | Casp3 | slateblue2 | 16 | -4.15286624203822 |
  | Map1lc3a-mRNA | -0.333 | 0.083 | -0.495 | -0.17 | 0.794 | 0.709 | 0.889 | 0.00102 | 0.0278 | loglinear | Autophagy, Cellular Stress | NS\_MM\_NEUROINFLAM\_V1.0 | Map1lc3a | mRNA | 0 |  | Cellular Stress;Autophagy |  |  | Map1lc3a | slateblue2 | 16 | -4.01204819277108 |
  | Eed-mRNA | -0.19 | 0.0478 | -0.284 | -0.0967 | 0.876 | 0.821 | 0.935 | 0.00107 | 0.0287 | loglinear | Cellular Stress, Epigenetic Regulation | NS\_MM\_NEUROINFLAM\_V1.0 | Eed | mRNA | 0 |  | Epigenetic Regulation;Cellular Stress |  |  | Eed | slateblue2 | 16 | -3.97489539748954 |
  | Bcl2-mRNA | -0.88 | 0.225 | -1.32 | -0.439 | 0.543 | 0.4 | 0.737 | 0.00123 | 0.0313 | lm.nb | Apoptosis, Autophagy, Cellular Stress, Cytokine Signaling, DNA Damage, Growth Factor Signaling, Innate Immune Response, Microglia Function, NF-kB | NS\_MM\_NEUROINFLAM\_V1.0 | Bcl2 | mRNA | 0 |  | NF-kB;Microglia Function;Innate Immune Response;Growth Factor Signaling;DNA Damage;Cytokine Signaling;Cellular Stress;Autophagy;Apoptosis | mmu04064;mmu04066;mmu04071;mmu04141;mmu04151;mmu04210;mmu04261;mmu04510;mmu04630;mmu04722;mmu04725;mmu04933;mmu05014;mmu05145;mmu05152;mmu05161;mmu05169;mmu05200;mmu05206;mmu05210;mmu05215;mmu05222 |  | Bcl2 | slateblue2 | 16 | -3.91111111111111 |
  | H2afx-mRNA | -0.214 | 0.0562 | -0.325 | -0.104 | 0.862 | 0.799 | 0.93 | 0.00154 | 0.0372 | loglinear | Cell Cycle, Cellular Stress, DNA Damage, Epigenetic Regulation, Wnt | NS\_MM\_NEUROINFLAM\_V1.0 | H2afx | mRNA | 0 |  | Wnt;Epigenetic Regulation;DNA Damage;Cellular Stress;Cell Cycle | mmu05034;mmu05322 |  | H2afx | slateblue2 | 16 | -3.80782918149466 |
  | Parp1-mRNA | -0.406 | 0.108 | -0.619 | -0.194 | 0.754 | 0.651 | 0.874 | 0.00175 | 0.0406 | loglinear | Apoptosis, Cellular Stress, DNA Damage, NF-kB | NS\_MM\_NEUROINFLAM\_V1.0 | Parp1 | mRNA | 0 |  | NF-kB;DNA Damage;Cellular Stress;Apoptosis | mmu03410;mmu04064 |  | Parp1 | slateblue2 | 16 | -3.75925925925926 |
  | Hdac6-mRNA | -0.68 | 0.181 | -1.04 | -0.325 | 0.624 | 0.488 | 0.799 | 0.00175 | 0.0406 | lm.nb | Cellular Stress, Epigenetic Regulation, Notch | NS\_MM\_NEUROINFLAM\_V1.0 | Hdac6 | mRNA | 0 |  | Notch;Epigenetic Regulation;Cellular Stress | mmu05034;mmu05203 |  | Hdac6 | slateblue2 | 16 | -3.75690607734807 |
  | Anapc15-mRNA | -0.663 | 0.18 | -1.01 | -0.31 | 0.632 | 0.495 | 0.807 | 0.002 | 0.0441 | lm.nb | Cell Cycle, Cellular Stress | NS\_MM\_NEUROINFLAM\_V1.0 | Anapc15 | mRNA | 0 |  | Cellular Stress;Cell Cycle |  |  | Anapc15 | slateblue2 | 16 | -3.68333333333333 |

  ##### DE Results - Cellular Stress genes - DPI: 2 vs.CTRL

  Table displaying each sample's global significance scores and directed global significance scores as defined in the heatmaps above. The global significance score is calculated as the square root of the mean squared t-statistic for the genes in a gene set, with t-statistics coming from the linear regression underlying our differential expression analysis. The directed global significance score is calculated as the square root of the mean signed squared t-statistic for the genes in a gene set, with t-statistics coming from the linear regression underlying our differential expression analysis.

- **Volcano Plot: DPI: 3 vs.CTRL  
  More Plot Information**


  ##### Volcano Plot: DPI: 3 vs.CTRL

  Volcano plot displaying each gene's -log10(p-value) and log2 fold change for the selected covariate. Highly statistically significant genes fall at the top of the plot, and highly differentially expressed genes fall to either side. Genes within the selected gene set are highlighted in orange. Horizontal lines indicate various False Discovery Rate (FDR) thresholds.
- **DE Results - Cellular Stress genes - DPI: 3 vs.CTRL  
  More Plot Information  Download CSV Data**

  | Probe Label | Log2 fold change | std error (log2) | Lower confidence limit (log2) | Upper confidence limit (log2) | Linear fold change | Lower confidence limit (linear) | Upper confidence limit (linear) | P-value | BY.p.value | method | Gene.sets | Codeset.Name | Probe.Label | Analyte.Type | Is.Control | Control.Type | Probe.Annotation | KEGG.Pathways | Cell.Type | Official.Gene.Name | volcanocol | volcanopch | tstats.all |
  | --- | --- | --- | --- | --- | --- | --- | --- | --- | --- | --- | --- | --- | --- | --- | --- | --- | --- | --- | --- | --- | --- | --- | --- |
  | Cdkn1a-mRNA | 2.72 | 0.171 | 2.38 | 3.05 | 6.57 | 5.21 | 8.28 | 3.19e-11 | 3.76e-08 | lm.nb | Adaptive Immune Response, Cell Cycle, Cellular Stress, Cytokine Signaling, DNA Damage, Growth Factor Signaling | NS\_MM\_NEUROINFLAM\_V1.0 | Cdkn1a | mRNA | 0 |  | Growth Factor Signaling;DNA Damage;Cytokine Signaling;Cellular Stress;Cell Cycle;Adaptive Immune Response | mmu04012;mmu04066;mmu04068;mmu04110;mmu04115;mmu04151;mmu04630;mmu04921;mmu05160;mmu05161;mmu05166;mmu05169;mmu05200;mmu05202;mmu05203;mmu05205;mmu05206;mmu05214;mmu05215;mmu05218;mmu05219;mmu05220 |  | Cdkn1a | slateblue1 | 16 | 15.906432748538 |
  | Mapt-mRNA | -0.413 | 0.0425 | -0.496 | -0.33 | 0.751 | 0.709 | 0.796 | 4.11e-08 | 9.67e-06 | loglinear | Apoptosis, Cellular Stress, Growth Factor Signaling, Neurons and Neurotransmission | NS\_MM\_NEUROINFLAM\_V1.0 | Mapt | mRNA | 0 |  | Neurons and Neurotransmission;Growth Factor Signaling;Cellular Stress;Apoptosis | mmu04010;mmu05010 |  | Mapt | slateblue1 | 16 | -9.71764705882353 |
  | Ptgs2-mRNA | 1.88 | 0.236 | 1.41 | 2.34 | 3.67 | 2.67 | 5.06 | 5.87e-07 | 7.14e-05 | lm.nb | Astrocyte Function, Cellular Stress, Innate Immune Response, Lipid Metabolism, Microglia Function, NF-kB | NS\_MM\_NEUROINFLAM\_V1.0 | Ptgs2 | mRNA | 0 |  | NF-kB;Microglia Function;Lipid Metabolism;Innate Immune Response;Cellular Stress;Astrocyte Function | mmu00590;mmu04064;mmu04370;mmu04668;mmu04723;mmu04726;mmu04913;mmu04921;mmu04923;mmu05140;mmu05200;mmu05206;mmu05222 |  | Ptgs2 | slateblue1 | 16 | 7.96610169491525 |
  | Hdac2-mRNA | -0.452 | 0.057 | -0.564 | -0.341 | 0.731 | 0.676 | 0.79 | 6.19e-07 | 7.29e-05 | loglinear | Cell Cycle, Cellular Stress, Epigenetic Regulation, Growth Factor Signaling, Notch | NS\_MM\_NEUROINFLAM\_V1.0 | Hdac2 | mRNA | 0 |  | Notch;Growth Factor Signaling;Epigenetic Regulation;Cellular Stress;Cell Cycle | mmu04110;mmu04213;mmu04330;mmu04919;mmu05016;mmu05034;mmu05169;mmu05200;mmu05202;mmu05203;mmu05220 |  | Hdac2 | slateblue1 | 16 | -7.92982456140351 |
  | Ets2-mRNA | 0.65 | 0.0908 | 0.472 | 0.828 | 1.57 | 1.39 | 1.78 | 2.28e-06 | 0.000206 | loglinear | Cellular Stress, Growth Factor Signaling, Microglia Function | NS\_MM\_NEUROINFLAM\_V1.0 | Ets2 | mRNA | 0 |  | Microglia Function;Growth Factor Signaling;Cellular Stress | mmu04014;mmu05166 |  | Ets2 | slateblue1 | 16 | 7.15859030837004 |
  | Parp1-mRNA | -0.736 | 0.108 | -0.949 | -0.524 | 0.6 | 0.518 | 0.695 | 4.31e-06 | 0.000334 | loglinear | Apoptosis, Cellular Stress, DNA Damage, NF-kB | NS\_MM\_NEUROINFLAM\_V1.0 | Parp1 | mRNA | 0 |  | NF-kB;DNA Damage;Cellular Stress;Apoptosis | mmu03410;mmu04064 |  | Parp1 | slateblue1 | 16 | -6.81481481481481 |
  | Ehmt2-mRNA | -0.329 | 0.0508 | -0.429 | -0.23 | 0.796 | 0.743 | 0.853 | 7.46e-06 | 0.000527 | loglinear | Cellular Stress, Epigenetic Regulation | NS\_MM\_NEUROINFLAM\_V1.0 | Ehmt2 | mRNA | 0 |  | Epigenetic Regulation;Cellular Stress | mmu00310;mmu04211 |  | Ehmt2 | slateblue1 | 16 | -6.47637795275591 |
  | Becn1-mRNA | -0.336 | 0.059 | -0.451 | -0.22 | 0.792 | 0.731 | 0.858 | 3.33e-05 | 0.00171 | loglinear | Autophagy, Cellular Stress | NS\_MM\_NEUROINFLAM\_V1.0 | Becn1 | mRNA | 0 |  | Cellular Stress;Autophagy | mmu04140 |  | Becn1 | slateblue1 | 16 | -5.69491525423729 |
  | Ago4-mRNA | -2.72 | 0.514 | -3.72 | -1.71 | 0.152 | 0.0757 | 0.306 | 7.46e-05 | 0.00321 | lm.nb | Adaptive Immune Response, Cellular Stress, Epigenetic Regulation, Growth Factor Signaling, Notch, Wnt | NS\_MM\_NEUROINFLAM\_V1.0 | Ago4 | mRNA | 0 |  | Wnt;Notch;Growth Factor Signaling;Epigenetic Regulation;Cellular Stress;Adaptive Immune Response |  |  | Ago4 | slateblue1 | 16 | -5.29182879377432 |
  | Eed-mRNA | -0.239 | 0.0478 | -0.333 | -0.145 | 0.847 | 0.794 | 0.904 | 0.000131 | 0.00525 | loglinear | Cellular Stress, Epigenetic Regulation | NS\_MM\_NEUROINFLAM\_V1.0 | Eed | mRNA | 0 |  | Epigenetic Regulation;Cellular Stress |  |  | Eed | slateblue1 | 16 | -5 |
  | Hspb1-mRNA | 1.36 | 0.29 | 0.795 | 1.93 | 2.57 | 1.73 | 3.82 | 0.000241 | 0.00894 | lm.nb | Angiogenesis, Astrocyte Function, Cellular Stress, Growth Factor Signaling | NS\_MM\_NEUROINFLAM\_V1.0 | Hspb1 | mRNA | 0 |  | Growth Factor Signaling;Cellular Stress;Astrocyte Function;Angiogenesis | mmu04010;mmu04370;mmu05146;mmu05169 |  | Hspb1 | slateblue1 | 16 | 4.68965517241379 |
  | Bnip3-mRNA | 0.275 | 0.0611 | 0.155 | 0.394 | 1.21 | 1.11 | 1.31 | 0.00037 | 0.0126 | loglinear | Apoptosis, Autophagy, Cellular Stress, Microglia Function | NS\_MM\_NEUROINFLAM\_V1.0 | Bnip3 | mRNA | 0 |  | Microglia Function;Cellular Stress;Autophagy;Apoptosis | mmu04068;mmu05134 |  | Bnip3 | slateblue2 | 16 | 4.50081833060556 |
  | Bcl2-mRNA | -0.943 | 0.227 | -1.39 | -0.498 | 0.52 | 0.382 | 0.708 | 0.000747 | 0.0225 | lm.nb | Apoptosis, Autophagy, Cellular Stress, Cytokine Signaling, DNA Damage, Growth Factor Signaling, Innate Immune Response, Microglia Function, NF-kB | NS\_MM\_NEUROINFLAM\_V1.0 | Bcl2 | mRNA | 0 |  | NF-kB;Microglia Function;Innate Immune Response;Growth Factor Signaling;DNA Damage;Cytokine Signaling;Cellular Stress;Autophagy;Apoptosis | mmu04064;mmu04066;mmu04071;mmu04141;mmu04151;mmu04210;mmu04261;mmu04510;mmu04630;mmu04722;mmu04725;mmu04933;mmu05014;mmu05145;mmu05152;mmu05161;mmu05169;mmu05200;mmu05206;mmu05210;mmu05215;mmu05222 |  | Bcl2 | slateblue2 | 16 | -4.15418502202643 |
  | Mapk10-mRNA | -0.571 | 0.138 | -0.842 | -0.3 | 0.673 | 0.558 | 0.812 | 0.000782 | 0.0231 | lm.nb | Adaptive Immune Response, Apoptosis, Autophagy, Cellular Stress, Growth Factor Signaling, Innate Immune Response, Wnt | NS\_MM\_NEUROINFLAM\_V1.0 | Mapk10 | mRNA | 0 |  | Wnt;Innate Immune Response;Growth Factor Signaling;Cellular Stress;Autophagy;Apoptosis;Adaptive Immune Response | mmu04010;mmu04012;mmu04014;mmu04024;mmu04068;mmu04071;mmu04141;mmu04310;mmu04380;mmu04510;mmu04620;mmu04621;mmu04622;mmu04664;mmu04668;mmu04722;mmu04723;mmu04728;mmu04750;mmu04910;mmu04912;mmu04914;mmu04917;mmu04920;mmu04930;mmu04931;mmu04932;mmu04933;mmu05132;mmu05133;mmu05142;mmu05145;mmu05152;mmu05160;mmu05161;mmu05164;mmu05168;mmu05169;mmu05200;mmu05210;mmu05212;mmu05231 |  | Mapk10 | slateblue2 | 16 | -4.13768115942029 |
  | Ulk1-mRNA | 0.296 | 0.0766 | 0.145 | 0.446 | 1.23 | 1.11 | 1.36 | 0.00139 | 0.0351 | loglinear | Autophagy, Cellular Stress | NS\_MM\_NEUROINFLAM\_V1.0 | Ulk1 | mRNA | 0 |  | Cellular Stress;Autophagy | mmu04140;mmu04150;mmu04152;mmu04211 |  | Ulk1 | slateblue2 | 16 | 3.86422976501305 |
  | Atg3-mRNA | -0.259 | 0.0705 | -0.397 | -0.121 | 0.836 | 0.759 | 0.92 | 0.00207 | 0.0504 | loglinear | Autophagy, Cellular Stress | NS\_MM\_NEUROINFLAM\_V1.0 | Atg3 | mRNA | 0 |  | Cellular Stress;Autophagy | mmu04140 |  | Atg3 | slateblue3 | 16 | -3.67375886524823 |
  | Apoe-mRNA | 0.298 | 0.0826 | 0.136 | 0.46 | 1.23 | 1.1 | 1.38 | 0.00237 | 0.055 | loglinear | Astrocyte Function, Cellular Stress, Lipid Metabolism, Microglia Function, Neurons and Neurotransmission | NS\_MM\_NEUROINFLAM\_V1.0 | Apoe | mRNA | 0 |  | Neurons and Neurotransmission;Microglia Function;Lipid Metabolism;Cellular Stress;Astrocyte Function | mmu05010 |  | Apoe | slateblue3 | 16 | 3.60774818401937 |
  | Atg5-mRNA | -0.325 | 0.0921 | -0.505 | -0.144 | 0.799 | 0.705 | 0.905 | 0.00282 | 0.063 | loglinear | Autophagy, Cellular Stress, Innate Immune Response | NS\_MM\_NEUROINFLAM\_V1.0 | Atg5 | mRNA | 0 |  | Innate Immune Response;Cellular Stress;Autophagy | mmu04140;mmu04211;mmu04213;mmu04622 |  | Atg5 | slateblue3 | 16 | -3.52877307274701 |
  | Snca-mRNA | -0.351 | 0.0996 | -0.546 | -0.155 | 0.784 | 0.685 | 0.898 | 0.00284 | 0.0631 | loglinear | Cellular Stress, Microglia Function | NS\_MM\_NEUROINFLAM\_V1.0 | Snca | mRNA | 0 |  | Microglia Function;Cellular Stress | mmu05010;mmu05012 |  | Snca | slateblue3 | 16 | -3.52409638554217 |
  | Vegfa-mRNA | -0.411 | 0.118 | -0.642 | -0.181 | 0.752 | 0.641 | 0.882 | 0.00299 | 0.0656 | loglinear | Angiogenesis, Cellular Stress, Cytokine Signaling, Growth Factor Signaling, Microglia Function | NS\_MM\_NEUROINFLAM\_V1.0 | Vegfa | mRNA | 0 |  | Microglia Function;Growth Factor Signaling;Cytokine Signaling;Cellular Stress;Angiogenesis | mmu04014;mmu04015;mmu04060;mmu04066;mmu04150;mmu04151;mmu04370;mmu04510;mmu04933;mmu05200;mmu05205;mmu05206;mmu05211;mmu05212;mmu05219;mmu05323 |  | Vegfa | slateblue3 | 16 | -3.48305084745763 |

  ##### DE Results - Cellular Stress genes - DPI: 3 vs.CTRL

  Table displaying each sample's global significance scores and directed global significance scores as defined in the heatmaps above. The global significance score is calculated as the square root of the mean squared t-statistic for the genes in a gene set, with t-statistics coming from the linear regression underlying our differential expression analysis. The directed global significance score is calculated as the square root of the mean signed squared t-statistic for the genes in a gene set, with t-statistics coming from the linear regression underlying our differential expression analysis.

DPI:
- DPI: differential expression in 1 vs. baseline of CTRL
- DPI: differential expression in 2 vs. baseline of CTRL
- DPI: differential expression in 3 vs. baseline of CTRL

- **Volcano Plot: DPI: 1 vs.CTRL  
  More Plot Information**


  ##### Volcano Plot: DPI: 1 vs.CTRL

  Volcano plot displaying each gene's -log10(p-value) and log2 fold change for the selected covariate. Highly statistically significant genes fall at the top of the plot, and highly differentially expressed genes fall to either side. Genes within the selected gene set are highlighted in orange. Horizontal lines indicate various False Discovery Rate (FDR) thresholds.
- **DE Results - Cytokine Signaling genes - DPI: 1 vs.CTRL  
  More Plot Information  Download CSV Data**

  | Probe Label | Log2 fold change | std error (log2) | Lower confidence limit (log2) | Upper confidence limit (log2) | Linear fold change | Lower confidence limit (linear) | Upper confidence limit (linear) | P-value | BY.p.value | method | Gene.sets | Codeset.Name | Probe.Label | Analyte.Type | Is.Control | Control.Type | Probe.Annotation | KEGG.Pathways | Cell.Type | Official.Gene.Name | volcanocol | volcanopch | tstats.all |
  | --- | --- | --- | --- | --- | --- | --- | --- | --- | --- | --- | --- | --- | --- | --- | --- | --- | --- | --- | --- | --- | --- | --- | --- |
  | Dusp7-mRNA | -0.378 | 0.0611 | -0.497 | -0.258 | 0.77 | 0.708 | 0.836 | 1.32e-05 | 0.0093 | loglinear | Adaptive Immune Response, Angiogenesis, Cytokine Signaling, Growth Factor Signaling, Innate Immune Response, Insulin Signaling, Microglia Function | NS\_MM\_NEUROINFLAM\_V1.0 | Dusp7 | mRNA | 0 |  | Microglia Function;Insulin Signaling;Innate Immune Response;Growth Factor Signaling;Cytokine Signaling;Angiogenesis;Adaptive Immune Response | mmu04010 |  | Dusp7 | slateblue2 | 16 | -6.18657937806874 |
  | Csf1r-mRNA | -0.687 | 0.144 | -0.969 | -0.405 | 0.621 | 0.511 | 0.755 | 0.000208 | 0.0464 | lm.nb | Cytokine Signaling, Growth Factor Signaling, Microglia Function | NS\_MM\_NEUROINFLAM\_V1.0 | Csf1r | mRNA | 0 |  | Microglia Function;Growth Factor Signaling;Cytokine Signaling | mmu04014;mmu04015;mmu04060;mmu04144;mmu04151;mmu04380;mmu04640;mmu05200;mmu05202 |  | Csf1r | slateblue2 | 16 | -4.77083333333333 |
  | Braf-mRNA | -0.641 | 0.154 | -0.943 | -0.339 | 0.641 | 0.52 | 0.791 | 0.000739 | 0.0815 | lm.nb | Adaptive Immune Response, Angiogenesis, Apoptosis, Cytokine Signaling, Growth Factor Signaling, Innate Immune Response, Insulin Signaling, Neurons and Neurotransmission | NS\_MM\_NEUROINFLAM\_V1.0 | Braf | mRNA | 0 |  | Neurons and Neurotransmission;Insulin Signaling;Innate Immune Response;Growth Factor Signaling;Cytokine Signaling;Apoptosis;Angiogenesis;Adaptive Immune Response | mmu04010;mmu04012;mmu04015;mmu04024;mmu04062;mmu04068;mmu04150;mmu04270;mmu04510;mmu04650;mmu04720;mmu04722;mmu04726;mmu04730;mmu04810;mmu04910;mmu04914;mmu05034;mmu05160;mmu05200;mmu05205;mmu05210;mmu05211;mmu05212;mmu05213;mmu05214;mmu05215;mmu05216;mmu05218;mmu05219;mmu05220;mmu05221;mmu05223 |  | Braf | slateblue3 | 16 | -4.16233766233766 |
  | Psmb8-mRNA | 1.36 | 0.365 | 0.649 | 2.08 | 2.57 | 1.57 | 4.23 | 0.00179 | 0.129 | lm.nb | Adaptive Immune Response, Angiogenesis, Apoptosis, Astrocyte Function, Cell Cycle, Cytokine Signaling, Growth Factor Signaling, Inflammatory Signaling, Insulin Signaling, Microglia Function, NF-kB, Wnt | NS\_MM\_NEUROINFLAM\_V1.0 | Psmb8 | mRNA | 0 |  | Wnt;NF-kB;Microglia Function;Insulin Signaling;Inflammatory Signaling;Growth Factor Signaling;Cytokine Signaling;Cell Cycle;Astrocyte Function;Apoptosis;Angiogenesis;Adaptive Immune Response |  |  | Psmb8 | slateblue4 | 16 | 3.72602739726027 |
  | Sumo1-mRNA | -0.424 | 0.115 | -0.65 | -0.198 | 0.745 | 0.637 | 0.871 | 0.00202 | 0.136 | lm.nb | Cell Cycle, Cytokine Signaling, DNA Damage, Inflammatory Signaling | NS\_MM\_NEUROINFLAM\_V1.0 | Sumo1 | mRNA | 0 |  | Inflammatory Signaling;DNA Damage;Cytokine Signaling;Cell Cycle |  |  | Sumo1 | slateblue4 | 16 | -3.68695652173913 |
  | Cdkn1a-mRNA | -0.625 | 0.18 | -0.979 | -0.272 | 0.648 | 0.507 | 0.828 | 0.00317 | 0.18 | lm.nb | Adaptive Immune Response, Cell Cycle, Cellular Stress, Cytokine Signaling, DNA Damage, Growth Factor Signaling | NS\_MM\_NEUROINFLAM\_V1.0 | Cdkn1a | mRNA | 0 |  | Growth Factor Signaling;DNA Damage;Cytokine Signaling;Cellular Stress;Cell Cycle;Adaptive Immune Response | mmu04012;mmu04066;mmu04068;mmu04110;mmu04115;mmu04151;mmu04630;mmu04921;mmu05160;mmu05161;mmu05166;mmu05169;mmu05200;mmu05202;mmu05203;mmu05205;mmu05206;mmu05214;mmu05215;mmu05218;mmu05219;mmu05220 |  | Cdkn1a | slateblue4 | 16 | -3.47222222222222 |
  | Traf6-mRNA | 0.546 | 0.169 | 0.215 | 0.877 | 1.46 | 1.16 | 1.84 | 0.00523 | 0.215 | lm.nb | Adaptive Immune Response, Apoptosis, Autophagy, Cytokine Signaling, Growth Factor Signaling, Innate Immune Response, NF-kB | NS\_MM\_NEUROINFLAM\_V1.0 | Traf6 | mRNA | 0 |  | NF-kB;Innate Immune Response;Growth Factor Signaling;Cytokine Signaling;Autophagy;Apoptosis;Adaptive Immune Response | mmu04010;mmu04064;mmu04120;mmu04144;mmu04380;mmu04620;mmu04621;mmu04622;mmu04722;mmu05133;mmu05140;mmu05142;mmu05145;mmu05152;mmu05160;mmu05162;mmu05168;mmu05169;mmu05200;mmu05222 |  | Traf6 | slateblue4 | 16 | 3.23076923076923 |
  | Map2k1-mRNA | -1.03 | 0.32 | -1.65 | -0.399 | 0.491 | 0.318 | 0.759 | 0.00553 | 0.224 | lm.nb | Adaptive Immune Response, Angiogenesis, Autophagy, Cytokine Signaling, Growth Factor Signaling, Innate Immune Response, Insulin Signaling, Microglia Function | NS\_MM\_NEUROINFLAM\_V1.0 | Map2k1 | mRNA | 0 |  | Microglia Function;Insulin Signaling;Innate Immune Response;Growth Factor Signaling;Cytokine Signaling;Autophagy;Angiogenesis;Adaptive Immune Response | mmu04010;mmu04012;mmu04014;mmu04015;mmu04022;mmu04024;mmu04062;mmu04066;mmu04068;mmu04071;mmu04072;mmu04114;mmu04151;mmu04270;mmu04370;mmu04380;mmu04510;mmu04540;mmu04550;mmu04620;mmu04650;mmu04660;mmu04662;mmu04664;mmu04666;mmu04668;mmu04720;mmu04722;mmu04725;mmu04726;mmu04730;mmu04810;mmu04910;mmu04912;mmu04914;mmu04915;mmu04916;mmu04917;mmu04919;mmu04921;mmu05020;mmu05034;mmu05161;mmu05164;mmu05200;mmu05205;mmu05206;mmu05210;mmu05211;mmu05212;mmu05213;mmu05214;mmu05215;mmu05216;mmu05218;mmu05219;mmu05220;mmu05221;mmu05223;mmu05230;mmu05231 |  | Map2k1 | slateblue4 | 16 | -3.21875 |
  | Sqstm1-mRNA | -0.854 | 0.271 | -1.39 | -0.322 | 0.553 | 0.383 | 0.8 | 0.00626 | 0.244 | lm.nb | Autophagy, Cytokine Signaling, Growth Factor Signaling | NS\_MM\_NEUROINFLAM\_V1.0 | Sqstm1 | mRNA | 0 |  | Growth Factor Signaling;Cytokine Signaling;Autophagy | mmu04380 |  | Sqstm1 | slateblue4 | 16 | -3.15129151291513 |
  | Dlg4-mRNA | -0.175 | 0.056 | -0.284 | -0.0649 | 0.886 | 0.821 | 0.956 | 0.00662 | 0.246 | loglinear | Adaptive Immune Response, Angiogenesis, Cytokine Signaling, Growth Factor Signaling, Insulin Signaling, Neurons and Neurotransmission | NS\_MM\_NEUROINFLAM\_V1.0 | Dlg4 | mRNA | 0 |  | Neurons and Neurotransmission;Insulin Signaling;Growth Factor Signaling;Cytokine Signaling;Angiogenesis;Adaptive Immune Response | mmu04390;mmu04724;mmu05016;mmu05030 |  | Dlg4 | slateblue4 | 16 | -3.125 |
  | Nefl-mRNA | -0.382 | 0.123 | -0.624 | -0.141 | 0.767 | 0.649 | 0.907 | 0.00687 | 0.248 | lm.nb | Adaptive Immune Response, Angiogenesis, Cytokine Signaling, Growth Factor Signaling, Insulin Signaling, Neurons and Neurotransmission | NS\_MM\_NEUROINFLAM\_V1.0 | Nefl | mRNA | 0 |  | Neurons and Neurotransmission;Insulin Signaling;Growth Factor Signaling;Cytokine Signaling;Angiogenesis;Adaptive Immune Response | mmu05014 |  | Nefl | slateblue4 | 16 | -3.10569105691057 |
  | Pik3r2-mRNA | -0.223 | 0.0793 | -0.378 | -0.0676 | 0.857 | 0.769 | 0.954 | 0.0125 | 0.357 | loglinear | Adaptive Immune Response, Angiogenesis, Apoptosis, Autophagy, Cytokine Signaling, Growth Factor Signaling, Innate Immune Response, Insulin Signaling | NS\_MM\_NEUROINFLAM\_V1.0 | Pik3r2 | mRNA | 0 |  | Insulin Signaling;Innate Immune Response;Growth Factor Signaling;Cytokine Signaling;Autophagy;Apoptosis;Angiogenesis;Adaptive Immune Response | mmu04012;mmu04014;mmu04015;mmu04022;mmu04024;mmu04062;mmu04066;mmu04068;mmu04070;mmu04071;mmu04072;mmu04150;mmu04151;mmu04152;mmu04210;mmu04211;mmu04213;mmu04261;mmu04370;mmu04380;mmu04510;mmu04550;mmu04611;mmu04620;mmu04630;mmu04650;mmu04660;mmu04662;mmu04664;mmu04666;mmu04668;mmu04670;mmu04722;mmu04725;mmu04750;mmu04810;mmu04910;mmu04914;mmu04915;mmu04917;mmu04919;mmu04921;mmu04923;mmu04930;mmu04931;mmu04932;mmu04933;mmu04960;mmu04973;mmu05100;mmu05142;mmu05145;mmu05146;mmu05160;mmu05161;mmu05162;mmu05164;mmu05166;mmu05169;mmu05200;mmu05203;mmu05205;mmu05210;mmu05211;mmu05212;mmu05213;mmu05214;mmu05215;mmu05218;mmu05220;mmu05221;mmu05222;mmu05223;mmu05230;mmu05231 |  | Pik3r2 | slateblue4 | 16 | -2.81210592686003 |
  | Irak1-mRNA | 0.531 | 0.191 | 0.157 | 0.904 | 1.44 | 1.11 | 1.87 | 0.0133 | 0.367 | lm.nb | Apoptosis, Cytokine Signaling, Growth Factor Signaling, Innate Immune Response, NF-kB | NS\_MM\_NEUROINFLAM\_V1.0 | Irak1 | mRNA | 0 |  | NF-kB;Innate Immune Response;Growth Factor Signaling;Cytokine Signaling;Apoptosis | mmu04064;mmu04210;mmu04620;mmu04722;mmu05133;mmu05140;mmu05142;mmu05145;mmu05152;mmu05162;mmu05169 |  | Irak1 | slateblue4 | 16 | 2.78010471204188 |
  | Ifnar1-mRNA | 0.313 | 0.116 | 0.0868 | 0.54 | 1.24 | 1.06 | 1.45 | 0.0154 | 0.41 | loglinear | Cytokine Signaling, Growth Factor Signaling, Inflammatory Signaling, Innate Immune Response | NS\_MM\_NEUROINFLAM\_V1.0 | Ifnar1 | mRNA | 0 |  | Innate Immune Response;Inflammatory Signaling;Growth Factor Signaling;Cytokine Signaling | mmu04060;mmu04151;mmu04380;mmu04620;mmu04630;mmu04650;mmu05160;mmu05161;mmu05162;mmu05164;mmu05168 |  | Ifnar1 | slateblue4 | 16 | 2.69827586206897 |
  | Cx3cl1-mRNA | -0.472 | 0.174 | -0.813 | -0.13 | 0.721 | 0.569 | 0.914 | 0.0157 | 0.41 | lm.nb | Cytokine Signaling, Inflammatory Signaling, Innate Immune Response | NS\_MM\_NEUROINFLAM\_V1.0 | Cx3cl1 | mRNA | 0 |  | Innate Immune Response;Inflammatory Signaling;Cytokine Signaling | mmu04060;mmu04062;mmu04668 |  | Cx3cl1 | slateblue4 | 16 | -2.71264367816092 |
  | Pik3ca-mRNA | -0.287 | 0.107 | -0.497 | -0.0771 | 0.82 | 0.709 | 0.948 | 0.0164 | 0.417 | lm.nb | Adaptive Immune Response, Angiogenesis, Apoptosis, Autophagy, Carbohydrate Metabolism, Cytokine Signaling, Growth Factor Signaling, Innate Immune Response, Insulin Signaling, Lipid Metabolism | NS\_MM\_NEUROINFLAM\_V1.0 | Pik3ca | mRNA | 0 |  | Lipid Metabolism;Insulin Signaling;Innate Immune Response;Growth Factor Signaling;Cytokine Signaling;Carbohydrate Metabolism;Autophagy;Apoptosis;Angiogenesis;Adaptive Immune Response | mmu00562;mmu04012;mmu04014;mmu04015;mmu04022;mmu04024;mmu04062;mmu04066;mmu04068;mmu04070;mmu04071;mmu04072;mmu04150;mmu04151;mmu04152;mmu04210;mmu04211;mmu04213;mmu04261;mmu04370;mmu04380;mmu04510;mmu04550;mmu04611;mmu04620;mmu04630;mmu04650;mmu04660;mmu04662;mmu04664;mmu04666;mmu04668;mmu04670;mmu04722;mmu04725;mmu04750;mmu04810;mmu04910;mmu04914;mmu04915;mmu04917;mmu04919;mmu04921;mmu04923;mmu04930;mmu04931;mmu04932;mmu04933;mmu04960;mmu04973;mmu05100;mmu05142;mmu05145;mmu05146;mmu05160;mmu05161;mmu05162;mmu05164;mmu05166;mmu05169;mmu05200;mmu05203;mmu05205;mmu05210;mmu05211;mmu05212;mmu05213;mmu05214;mmu05215;mmu05218;mmu05220;mmu05221;mmu05222;mmu05223;mmu05230;mmu05231 |  | Pik3ca | slateblue4 | 16 | -2.68224299065421 |
  | Grin2b-mRNA | -0.254 | 0.101 | -0.451 | -0.057 | 0.839 | 0.731 | 0.961 | 0.0225 | 0.508 | lm.nb | Adaptive Immune Response, Angiogenesis, Cytokine Signaling, Growth Factor Signaling, Insulin Signaling, Neurons and Neurotransmission | NS\_MM\_NEUROINFLAM\_V1.0 | Grin2b | mRNA | 0 |  | Neurons and Neurotransmission;Insulin Signaling;Growth Factor Signaling;Cytokine Signaling;Angiogenesis;Adaptive Immune Response | mmu04014;mmu04015;mmu04024;mmu04080;mmu04713;mmu04720;mmu04724;mmu04728;mmu05010;mmu05014;mmu05016;mmu05030;mmu05031;mmu05033;mmu05034;mmu05322 |  | Grin2b | azure4 | 1 | -2.51485148514851 |
  | Irak3-mRNA | 1.3 | 0.518 | 0.288 | 2.32 | 2.47 | 1.22 | 4.99 | 0.0229 | 0.509 | lm.nb | Apoptosis, Cytokine Signaling, Innate Immune Response | NS\_MM\_NEUROINFLAM\_V1.0 | Irak3 | mRNA | 0 |  | Innate Immune Response;Cytokine Signaling;Apoptosis | mmu04210;mmu04722 |  | Irak3 | azure4 | 1 | 2.50965250965251 |
  | Vav1-mRNA | 1.65 | 0.659 | 0.363 | 2.95 | 3.15 | 1.29 | 7.7 | 0.0231 | 0.51 | lm.nb | Adaptive Immune Response, Angiogenesis, Autophagy, Cytokine Signaling, Growth Factor Signaling, Innate Immune Response | NS\_MM\_NEUROINFLAM\_V1.0 | Vav1 | mRNA | 0 |  | Innate Immune Response;Growth Factor Signaling;Cytokine Signaling;Autophagy;Angiogenesis;Adaptive Immune Response | mmu04024;mmu04062;mmu04510;mmu04650;mmu04660;mmu04662;mmu04664;mmu04666;mmu04670;mmu04810 |  | Vav1 | azure4 | 1 | 2.50379362670713 |
  | Nfkb1-mRNA | -0.581 | 0.234 | -1.04 | -0.123 | 0.669 | 0.487 | 0.918 | 0.0244 | 0.533 | lm.nb | Adaptive Immune Response, Apoptosis, Cellular Stress, Cytokine Signaling, Growth Factor Signaling, Inflammatory Signaling, Innate Immune Response, NF-kB | NS\_MM\_NEUROINFLAM\_V1.0 | Nfkb1 | mRNA | 0 |  | NF-kB;Innate Immune Response;Inflammatory Signaling;Growth Factor Signaling;Cytokine Signaling;Cellular Stress;Apoptosis;Adaptive Immune Response | mmu04010;mmu04014;mmu04024;mmu04062;mmu04064;mmu04066;mmu04071;mmu04151;mmu04210;mmu04211;mmu04380;mmu04620;mmu04621;mmu04622;mmu04623;mmu04660;mmu04662;mmu04668;mmu04722;mmu04917;mmu04920;mmu04931;mmu04932;mmu04933;mmu05030;mmu05132;mmu05133;mmu05134;mmu05140;mmu05142;mmu05145;mmu05146;mmu05152;mmu05160;mmu05161;mmu05162;mmu05164;mmu05166;mmu05168;mmu05169;mmu05200;mmu05202;mmu05203;mmu05206;mmu05212;mmu05215;mmu05220;mmu05221;mmu05222;mmu05321 |  | Nfkb1 | azure4 | 1 | -2.48290598290598 |

  ##### DE Results - Cytokine Signaling genes - DPI: 1 vs.CTRL

  Table displaying each sample's global significance scores and directed global significance scores as defined in the heatmaps above. The global significance score is calculated as the square root of the mean squared t-statistic for the genes in a gene set, with t-statistics coming from the linear regression underlying our differential expression analysis. The directed global significance score is calculated as the square root of the mean signed squared t-statistic for the genes in a gene set, with t-statistics coming from the linear regression underlying our differential expression analysis.

- **Volcano Plot: DPI: 2 vs.CTRL  
  More Plot Information**


  ##### Volcano Plot: DPI: 2 vs.CTRL

  Volcano plot displaying each gene's -log10(p-value) and log2 fold change for the selected covariate. Highly statistically significant genes fall at the top of the plot, and highly differentially expressed genes fall to either side. Genes within the selected gene set are highlighted in orange. Horizontal lines indicate various False Discovery Rate (FDR) thresholds.
- **DE Results - Cytokine Signaling genes - DPI: 2 vs.CTRL  
  More Plot Information  Download CSV Data**

  | Probe Label | Log2 fold change | std error (log2) | Lower confidence limit (log2) | Upper confidence limit (log2) | Linear fold change | Lower confidence limit (linear) | Upper confidence limit (linear) | P-value | BY.p.value | method | Gene.sets | Codeset.Name | Probe.Label | Analyte.Type | Is.Control | Control.Type | Probe.Annotation | KEGG.Pathways | Cell.Type | Official.Gene.Name | volcanocol | volcanopch | tstats.all |
  | --- | --- | --- | --- | --- | --- | --- | --- | --- | --- | --- | --- | --- | --- | --- | --- | --- | --- | --- | --- | --- | --- | --- | --- |
  | Cdkn1a-mRNA | 2.13 | 0.171 | 1.79 | 2.46 | 4.37 | 3.47 | 5.52 | 1.24e-09 | 8.78e-07 | lm.nb | Adaptive Immune Response, Cell Cycle, Cellular Stress, Cytokine Signaling, DNA Damage, Growth Factor Signaling | NS\_MM\_NEUROINFLAM\_V1.0 | Cdkn1a | mRNA | 0 |  | Growth Factor Signaling;DNA Damage;Cytokine Signaling;Cellular Stress;Cell Cycle;Adaptive Immune Response | mmu04012;mmu04066;mmu04068;mmu04110;mmu04115;mmu04151;mmu04630;mmu04921;mmu05160;mmu05161;mmu05166;mmu05169;mmu05200;mmu05202;mmu05203;mmu05205;mmu05206;mmu05214;mmu05215;mmu05218;mmu05219;mmu05220 |  | Cdkn1a | slateblue1 | 16 | 12.4561403508772 |
  | Dusp7-mRNA | -0.496 | 0.0611 | -0.616 | -0.377 | 0.709 | 0.652 | 0.77 | 4.53e-07 | 9.99e-05 | loglinear | Adaptive Immune Response, Angiogenesis, Cytokine Signaling, Growth Factor Signaling, Innate Immune Response, Insulin Signaling, Microglia Function | NS\_MM\_NEUROINFLAM\_V1.0 | Dusp7 | mRNA | 0 |  | Microglia Function;Insulin Signaling;Innate Immune Response;Growth Factor Signaling;Cytokine Signaling;Angiogenesis;Adaptive Immune Response | mmu04010 |  | Dusp7 | slateblue1 | 16 | -8.11783960720131 |
  | Csf1r-mRNA | -1 | 0.15 | -1.29 | -0.706 | 0.5 | 0.408 | 0.613 | 5.38e-06 | 0.000678 | lm.nb | Cytokine Signaling, Growth Factor Signaling, Microglia Function | NS\_MM\_NEUROINFLAM\_V1.0 | Csf1r | mRNA | 0 |  | Microglia Function;Growth Factor Signaling;Cytokine Signaling | mmu04014;mmu04015;mmu04060;mmu04144;mmu04151;mmu04380;mmu04640;mmu05200;mmu05202 |  | Csf1r | slateblue1 | 16 | -6.66666666666667 |
  | Psmb8-mRNA | 2.23 | 0.352 | 1.54 | 2.92 | 4.7 | 2.91 | 7.59 | 9.89e-06 | 0.000997 | lm.nb | Adaptive Immune Response, Angiogenesis, Apoptosis, Astrocyte Function, Cell Cycle, Cytokine Signaling, Growth Factor Signaling, Inflammatory Signaling, Insulin Signaling, Microglia Function, NF-kB, Wnt | NS\_MM\_NEUROINFLAM\_V1.0 | Psmb8 | mRNA | 0 |  | Wnt;NF-kB;Microglia Function;Insulin Signaling;Inflammatory Signaling;Growth Factor Signaling;Cytokine Signaling;Cell Cycle;Astrocyte Function;Apoptosis;Angiogenesis;Adaptive Immune Response |  |  | Psmb8 | slateblue1 | 16 | 6.33522727272727 |
  | Bcl2l1-mRNA | 0.407 | 0.0734 | 0.263 | 0.551 | 1.33 | 1.2 | 1.46 | 4.51e-05 | 0.00306 | loglinear | Apoptosis, Autophagy, Cytokine Signaling, Growth Factor Signaling, Innate Immune Response, NF-kB | NS\_MM\_NEUROINFLAM\_V1.0 | Bcl2l1 | mRNA | 0 |  | NF-kB;Innate Immune Response;Growth Factor Signaling;Cytokine Signaling;Autophagy;Apoptosis | mmu04014;mmu04064;mmu04151;mmu04210;mmu04630;mmu05014;mmu05145;mmu05166;mmu05200;mmu05202;mmu05212;mmu05220;mmu05222 |  | Bcl2l1 | slateblue1 | 16 | 5.54495912806539 |
  | Tgfbr1-mRNA | -0.546 | 0.102 | -0.745 | -0.347 | 0.685 | 0.596 | 0.786 | 6.2e-05 | 0.00387 | loglinear | Cytokine Signaling, Growth Factor Signaling | NS\_MM\_NEUROINFLAM\_V1.0 | Tgfbr1 | mRNA | 0 |  | Growth Factor Signaling;Cytokine Signaling | mmu04010;mmu04060;mmu04068;mmu04144;mmu04350;mmu04380;mmu04390;mmu04520;mmu04933;mmu05142;mmu05161;mmu05166;mmu05200;mmu05210;mmu05212;mmu05220 |  | Tgfbr1 | slateblue1 | 16 | -5.35294117647059 |
  | Sumo1-mRNA | -0.529 | 0.115 | -0.755 | -0.303 | 0.693 | 0.593 | 0.811 | 0.000305 | 0.0121 | lm.nb | Cell Cycle, Cytokine Signaling, DNA Damage, Inflammatory Signaling | NS\_MM\_NEUROINFLAM\_V1.0 | Sumo1 | mRNA | 0 |  | Inflammatory Signaling;DNA Damage;Cytokine Signaling;Cell Cycle |  |  | Sumo1 | slateblue2 | 16 | -4.6 |
  | Traf6-mRNA | 0.756 | 0.167 | 0.428 | 1.08 | 1.69 | 1.35 | 2.12 | 0.000353 | 0.0135 | lm.nb | Adaptive Immune Response, Apoptosis, Autophagy, Cytokine Signaling, Growth Factor Signaling, Innate Immune Response, NF-kB | NS\_MM\_NEUROINFLAM\_V1.0 | Traf6 | mRNA | 0 |  | NF-kB;Innate Immune Response;Growth Factor Signaling;Cytokine Signaling;Autophagy;Apoptosis;Adaptive Immune Response | mmu04010;mmu04064;mmu04120;mmu04144;mmu04380;mmu04620;mmu04621;mmu04622;mmu04722;mmu05133;mmu05140;mmu05142;mmu05145;mmu05152;mmu05160;mmu05162;mmu05168;mmu05169;mmu05200;mmu05222 |  | Traf6 | slateblue2 | 16 | 4.52694610778443 |
  | Pik3r2-mRNA | -0.338 | 0.0793 | -0.494 | -0.183 | 0.791 | 0.71 | 0.881 | 0.000587 | 0.0197 | loglinear | Adaptive Immune Response, Angiogenesis, Apoptosis, Autophagy, Cytokine Signaling, Growth Factor Signaling, Innate Immune Response, Insulin Signaling | NS\_MM\_NEUROINFLAM\_V1.0 | Pik3r2 | mRNA | 0 |  | Insulin Signaling;Innate Immune Response;Growth Factor Signaling;Cytokine Signaling;Autophagy;Apoptosis;Angiogenesis;Adaptive Immune Response | mmu04012;mmu04014;mmu04015;mmu04022;mmu04024;mmu04062;mmu04066;mmu04068;mmu04070;mmu04071;mmu04072;mmu04150;mmu04151;mmu04152;mmu04210;mmu04211;mmu04213;mmu04261;mmu04370;mmu04380;mmu04510;mmu04550;mmu04611;mmu04620;mmu04630;mmu04650;mmu04660;mmu04662;mmu04664;mmu04666;mmu04668;mmu04670;mmu04722;mmu04725;mmu04750;mmu04810;mmu04910;mmu04914;mmu04915;mmu04917;mmu04919;mmu04921;mmu04923;mmu04930;mmu04931;mmu04932;mmu04933;mmu04960;mmu04973;mmu05100;mmu05142;mmu05145;mmu05146;mmu05160;mmu05161;mmu05162;mmu05164;mmu05166;mmu05169;mmu05200;mmu05203;mmu05205;mmu05210;mmu05211;mmu05212;mmu05213;mmu05214;mmu05215;mmu05218;mmu05220;mmu05221;mmu05222;mmu05223;mmu05230;mmu05231 |  | Pik3r2 | slateblue2 | 16 | -4.26229508196721 |
  | Nefl-mRNA | -0.522 | 0.124 | -0.764 | -0.28 | 0.696 | 0.589 | 0.824 | 0.000651 | 0.0215 | lm.nb | Adaptive Immune Response, Angiogenesis, Cytokine Signaling, Growth Factor Signaling, Insulin Signaling, Neurons and Neurotransmission | NS\_MM\_NEUROINFLAM\_V1.0 | Nefl | mRNA | 0 |  | Neurons and Neurotransmission;Insulin Signaling;Growth Factor Signaling;Cytokine Signaling;Angiogenesis;Adaptive Immune Response | mmu05014 |  | Nefl | slateblue2 | 16 | -4.20967741935484 |
  | Chuk-mRNA | -0.331 | 0.0787 | -0.485 | -0.176 | 0.795 | 0.715 | 0.885 | 0.000678 | 0.0218 | loglinear | Adaptive Immune Response, Apoptosis, Cytokine Signaling, Growth Factor Signaling, Inflammatory Signaling, Innate Immune Response, NF-kB | NS\_MM\_NEUROINFLAM\_V1.0 | Chuk | mRNA | 0 |  | NF-kB;Innate Immune Response;Inflammatory Signaling;Growth Factor Signaling;Cytokine Signaling;Apoptosis;Adaptive Immune Response | mmu04010;mmu04014;mmu04062;mmu04064;mmu04068;mmu04151;mmu04210;mmu04380;mmu04620;mmu04621;mmu04622;mmu04623;mmu04660;mmu04662;mmu04668;mmu04920;mmu05142;mmu05145;mmu05160;mmu05161;mmu05162;mmu05166;mmu05168;mmu05169;mmu05200;mmu05212;mmu05215;mmu05220;mmu05221;mmu05222 |  | Chuk | slateblue2 | 16 | -4.20584498094028 |
  | Akt2-mRNA | -0.592 | 0.142 | -0.869 | -0.314 | 0.664 | 0.547 | 0.804 | 0.000713 | 0.022 | loglinear | Adaptive Immune Response, Angiogenesis, Apoptosis, Autophagy, Carbohydrate Metabolism, Cytokine Signaling, Growth Factor Signaling, Innate Immune Response, Insulin Signaling, Neurons and Neurotransmission, Wnt | NS\_MM\_NEUROINFLAM\_V1.0 | Akt2 | mRNA | 0 |  | Wnt;Neurons and Neurotransmission;Insulin Signaling;Innate Immune Response;Growth Factor Signaling;Cytokine Signaling;Carbohydrate Metabolism;Autophagy;Apoptosis;Angiogenesis;Adaptive Immune Response | mmu04010;mmu04012;mmu04014;mmu04015;mmu04022;mmu04024;mmu04062;mmu04066;mmu04068;mmu04071;mmu04072;mmu04150;mmu04151;mmu04152;mmu04210;mmu04211;mmu04213;mmu04261;mmu04370;mmu04380;mmu04510;mmu04530;mmu04550;mmu04611;mmu04620;mmu04630;mmu04660;mmu04662;mmu04664;mmu04666;mmu04668;mmu04722;mmu04725;mmu04728;mmu04910;mmu04914;mmu04915;mmu04917;mmu04919;mmu04920;mmu04922;mmu04923;mmu04931;mmu04932;mmu04933;mmu04973;mmu05142;mmu05145;mmu05152;mmu05160;mmu05161;mmu05162;mmu05164;mmu05166;mmu05169;mmu05200;mmu05205;mmu05210;mmu05211;mmu05212;mmu05213;mmu05214;mmu05215;mmu05218;mmu05220;mmu05221;mmu05222;mmu05223;mmu05230;mmu05231 |  | Akt2 | slateblue2 | 16 | -4.16901408450704 |
  | Braf-mRNA | -0.643 | 0.154 | -0.945 | -0.341 | 0.64 | 0.519 | 0.789 | 0.000718 | 0.022 | lm.nb | Adaptive Immune Response, Angiogenesis, Apoptosis, Cytokine Signaling, Growth Factor Signaling, Innate Immune Response, Insulin Signaling, Neurons and Neurotransmission | NS\_MM\_NEUROINFLAM\_V1.0 | Braf | mRNA | 0 |  | Neurons and Neurotransmission;Insulin Signaling;Innate Immune Response;Growth Factor Signaling;Cytokine Signaling;Apoptosis;Angiogenesis;Adaptive Immune Response | mmu04010;mmu04012;mmu04015;mmu04024;mmu04062;mmu04068;mmu04150;mmu04270;mmu04510;mmu04650;mmu04720;mmu04722;mmu04726;mmu04730;mmu04810;mmu04910;mmu04914;mmu05034;mmu05160;mmu05200;mmu05205;mmu05210;mmu05211;mmu05212;mmu05213;mmu05214;mmu05215;mmu05216;mmu05218;mmu05219;mmu05220;mmu05221;mmu05223 |  | Braf | slateblue2 | 16 | -4.17532467532468 |
  | Il6ra-mRNA | 1.42 | 0.352 | 0.731 | 2.11 | 2.68 | 1.66 | 4.32 | 0.000954 | 0.0266 | lm.nb | Cytokine Signaling, Growth Factor Signaling, Microglia Function | NS\_MM\_NEUROINFLAM\_V1.0 | Il6ra | mRNA | 0 |  | Microglia Function;Growth Factor Signaling;Cytokine Signaling | mmu04060;mmu04066;mmu04151;mmu04630;mmu04640;mmu04932 |  | Il6ra | slateblue2 | 16 | 4.03409090909091 |
  | Dlg4-mRNA | -0.221 | 0.056 | -0.331 | -0.112 | 0.858 | 0.795 | 0.926 | 0.00114 | 0.0296 | loglinear | Adaptive Immune Response, Angiogenesis, Cytokine Signaling, Growth Factor Signaling, Insulin Signaling, Neurons and Neurotransmission | NS\_MM\_NEUROINFLAM\_V1.0 | Dlg4 | mRNA | 0 |  | Neurons and Neurotransmission;Insulin Signaling;Growth Factor Signaling;Cytokine Signaling;Angiogenesis;Adaptive Immune Response | mmu04390;mmu04724;mmu05016;mmu05030 |  | Dlg4 | slateblue2 | 16 | -3.94642857142857 |
  | Tnfrsf1a-mRNA | 1.14 | 0.29 | 0.575 | 1.71 | 2.21 | 1.49 | 3.27 | 0.00116 | 0.0296 | lm.nb | Apoptosis, Cytokine Signaling, Growth Factor Signaling, Innate Immune Response, NF-kB | NS\_MM\_NEUROINFLAM\_V1.0 | Tnfrsf1a | mRNA | 0 |  | NF-kB;Innate Immune Response;Growth Factor Signaling;Cytokine Signaling;Apoptosis | mmu04010;mmu04060;mmu04064;mmu04071;mmu04210;mmu04380;mmu04668;mmu04920;mmu04931;mmu04932;mmu05010;mmu05014;mmu05142;mmu05145;mmu05152;mmu05160;mmu05164;mmu05166;mmu05168 |  | Tnfrsf1a | slateblue2 | 16 | 3.93103448275862 |
  | Bcl2-mRNA | -0.88 | 0.225 | -1.32 | -0.439 | 0.543 | 0.4 | 0.737 | 0.00123 | 0.0313 | lm.nb | Apoptosis, Autophagy, Cellular Stress, Cytokine Signaling, DNA Damage, Growth Factor Signaling, Innate Immune Response, Microglia Function, NF-kB | NS\_MM\_NEUROINFLAM\_V1.0 | Bcl2 | mRNA | 0 |  | NF-kB;Microglia Function;Innate Immune Response;Growth Factor Signaling;DNA Damage;Cytokine Signaling;Cellular Stress;Autophagy;Apoptosis | mmu04064;mmu04066;mmu04071;mmu04141;mmu04151;mmu04210;mmu04261;mmu04510;mmu04630;mmu04722;mmu04725;mmu04933;mmu05014;mmu05145;mmu05152;mmu05161;mmu05169;mmu05200;mmu05206;mmu05210;mmu05215;mmu05222 |  | Bcl2 | slateblue2 | 16 | -3.91111111111111 |
  | Grin2b-mRNA | -0.395 | 0.101 | -0.592 | -0.197 | 0.761 | 0.663 | 0.872 | 0.00124 | 0.0314 | lm.nb | Adaptive Immune Response, Angiogenesis, Cytokine Signaling, Growth Factor Signaling, Insulin Signaling, Neurons and Neurotransmission | NS\_MM\_NEUROINFLAM\_V1.0 | Grin2b | mRNA | 0 |  | Neurons and Neurotransmission;Insulin Signaling;Growth Factor Signaling;Cytokine Signaling;Angiogenesis;Adaptive Immune Response | mmu04014;mmu04015;mmu04024;mmu04080;mmu04713;mmu04720;mmu04724;mmu04728;mmu05010;mmu05014;mmu05016;mmu05030;mmu05031;mmu05033;mmu05034;mmu05322 |  | Grin2b | slateblue2 | 16 | -3.91089108910891 |
  | Irak3-mRNA | 1.83 | 0.51 | 0.833 | 2.83 | 3.56 | 1.78 | 7.13 | 0.00244 | 0.0523 | lm.nb | Apoptosis, Cytokine Signaling, Innate Immune Response | NS\_MM\_NEUROINFLAM\_V1.0 | Irak3 | mRNA | 0 |  | Innate Immune Response;Cytokine Signaling;Apoptosis | mmu04210;mmu04722 |  | Irak3 | slateblue3 | 16 | 3.58823529411765 |
  | Vav1-mRNA | 2.33 | 0.652 | 1.06 | 3.61 | 5.04 | 2.08 | 12.2 | 0.00251 | 0.053 | lm.nb | Adaptive Immune Response, Angiogenesis, Autophagy, Cytokine Signaling, Growth Factor Signaling, Innate Immune Response | NS\_MM\_NEUROINFLAM\_V1.0 | Vav1 | mRNA | 0 |  | Innate Immune Response;Growth Factor Signaling;Cytokine Signaling;Autophagy;Angiogenesis;Adaptive Immune Response | mmu04024;mmu04062;mmu04510;mmu04650;mmu04660;mmu04662;mmu04664;mmu04666;mmu04670;mmu04810 |  | Vav1 | slateblue3 | 16 | 3.57361963190184 |

  ##### DE Results - Cytokine Signaling genes - DPI: 2 vs.CTRL

  Table displaying each sample's global significance scores and directed global significance scores as defined in the heatmaps above. The global significance score is calculated as the square root of the mean squared t-statistic for the genes in a gene set, with t-statistics coming from the linear regression underlying our differential expression analysis. The directed global significance score is calculated as the square root of the mean signed squared t-statistic for the genes in a gene set, with t-statistics coming from the linear regression underlying our differential expression analysis.

- **Volcano Plot: DPI: 3 vs.CTRL  
  More Plot Information**


  ##### Volcano Plot: DPI: 3 vs.CTRL

  Volcano plot displaying each gene's -log10(p-value) and log2 fold change for the selected covariate. Highly statistically significant genes fall at the top of the plot, and highly differentially expressed genes fall to either side. Genes within the selected gene set are highlighted in orange. Horizontal lines indicate various False Discovery Rate (FDR) thresholds.
- **DE Results - Cytokine Signaling genes - DPI: 3 vs.CTRL  
  More Plot Information  Download CSV Data**

  | Probe Label | Log2 fold change | std error (log2) | Lower confidence limit (log2) | Upper confidence limit (log2) | Linear fold change | Lower confidence limit (linear) | Upper confidence limit (linear) | P-value | BY.p.value | method | Gene.sets | Codeset.Name | Probe.Label | Analyte.Type | Is.Control | Control.Type | Probe.Annotation | KEGG.Pathways | Cell.Type | Official.Gene.Name | volcanocol | volcanopch | tstats.all |
  | --- | --- | --- | --- | --- | --- | --- | --- | --- | --- | --- | --- | --- | --- | --- | --- | --- | --- | --- | --- | --- | --- | --- | --- |
  | Cdkn1a-mRNA | 2.72 | 0.171 | 2.38 | 3.05 | 6.57 | 5.21 | 8.28 | 3.19e-11 | 3.76e-08 | lm.nb | Adaptive Immune Response, Cell Cycle, Cellular Stress, Cytokine Signaling, DNA Damage, Growth Factor Signaling | NS\_MM\_NEUROINFLAM\_V1.0 | Cdkn1a | mRNA | 0 |  | Growth Factor Signaling;DNA Damage;Cytokine Signaling;Cellular Stress;Cell Cycle;Adaptive Immune Response | mmu04012;mmu04066;mmu04068;mmu04110;mmu04115;mmu04151;mmu04630;mmu04921;mmu05160;mmu05161;mmu05166;mmu05169;mmu05200;mmu05202;mmu05203;mmu05205;mmu05206;mmu05214;mmu05215;mmu05218;mmu05219;mmu05220 |  | Cdkn1a | slateblue1 | 16 | 15.906432748538 |
  | Irf3-mRNA | -1.48 | 0.165 | -1.8 | -1.15 | 0.359 | 0.287 | 0.45 | 1.3e-07 | 2.18e-05 | lm.nb | Apoptosis, Cytokine Signaling, Inflammatory Signaling, Innate Immune Response | NS\_MM\_NEUROINFLAM\_V1.0 | Irf3 | mRNA | 0 |  | Innate Immune Response;Inflammatory Signaling;Cytokine Signaling;Apoptosis | mmu04620;mmu04622;mmu04623;mmu05133;mmu05160;mmu05161;mmu05162;mmu05164;mmu05168;mmu05169;mmu05203 |  | Irf3 | slateblue1 | 16 | -8.96969696969697 |
  | Psmb8-mRNA | 2.99 | 0.346 | 2.31 | 3.67 | 7.95 | 4.97 | 12.7 | 1.99e-07 | 3.2e-05 | lm.nb | Adaptive Immune Response, Angiogenesis, Apoptosis, Astrocyte Function, Cell Cycle, Cytokine Signaling, Growth Factor Signaling, Inflammatory Signaling, Insulin Signaling, Microglia Function, NF-kB, Wnt | NS\_MM\_NEUROINFLAM\_V1.0 | Psmb8 | mRNA | 0 |  | Wnt;NF-kB;Microglia Function;Insulin Signaling;Inflammatory Signaling;Growth Factor Signaling;Cytokine Signaling;Cell Cycle;Astrocyte Function;Apoptosis;Angiogenesis;Adaptive Immune Response |  |  | Psmb8 | slateblue1 | 16 | 8.64161849710983 |
  | Ikbkb-mRNA | -0.601 | 0.0868 | -0.772 | -0.431 | 0.659 | 0.586 | 0.742 | 3.42e-06 | 0.00028 | loglinear | Adaptive Immune Response, Apoptosis, Cytokine Signaling, Growth Factor Signaling, Inflammatory Signaling, Innate Immune Response, NF-kB | NS\_MM\_NEUROINFLAM\_V1.0 | Ikbkb | mRNA | 0 |  | NF-kB;Innate Immune Response;Inflammatory Signaling;Growth Factor Signaling;Cytokine Signaling;Apoptosis;Adaptive Immune Response | mmu04010;mmu04014;mmu04062;mmu04064;mmu04068;mmu04150;mmu04151;mmu04210;mmu04380;mmu04620;mmu04621;mmu04622;mmu04623;mmu04660;mmu04662;mmu04668;mmu04722;mmu04910;mmu04920;mmu04930;mmu04931;mmu04932;mmu05142;mmu05145;mmu05160;mmu05161;mmu05164;mmu05166;mmu05168;mmu05169;mmu05200;mmu05206;mmu05212;mmu05215;mmu05220;mmu05221;mmu05222 |  | Ikbkb | slateblue1 | 16 | -6.92396313364055 |
  | Tnfrsf1a-mRNA | 1.92 | 0.281 | 1.37 | 2.47 | 3.79 | 2.59 | 5.56 | 3.96e-06 | 0.000317 | lm.nb | Apoptosis, Cytokine Signaling, Growth Factor Signaling, Innate Immune Response, NF-kB | NS\_MM\_NEUROINFLAM\_V1.0 | Tnfrsf1a | mRNA | 0 |  | NF-kB;Innate Immune Response;Growth Factor Signaling;Cytokine Signaling;Apoptosis | mmu04010;mmu04060;mmu04064;mmu04071;mmu04210;mmu04380;mmu04668;mmu04920;mmu04931;mmu04932;mmu05010;mmu05014;mmu05142;mmu05145;mmu05152;mmu05160;mmu05164;mmu05166;mmu05168 |  | Tnfrsf1a | slateblue1 | 16 | 6.83274021352313 |
  | Cx3cr1-mRNA | -3.17 | 0.467 | -4.08 | -2.25 | 0.111 | 0.059 | 0.21 | 4.35e-06 | 0.000334 | loglinear | Cytokine Signaling, Microglia Function | NS\_MM\_NEUROINFLAM\_V1.0 | Cx3cr1 | mRNA | 0 |  | Microglia Function;Cytokine Signaling | mmu04060;mmu04062 |  | Cx3cr1 | slateblue1 | 16 | -6.78800856531049 |
  | Bcl2l1-mRNA | 0.414 | 0.0734 | 0.27 | 0.558 | 1.33 | 1.21 | 1.47 | 3.75e-05 | 0.00184 | loglinear | Apoptosis, Autophagy, Cytokine Signaling, Growth Factor Signaling, Innate Immune Response, NF-kB | NS\_MM\_NEUROINFLAM\_V1.0 | Bcl2l1 | mRNA | 0 |  | NF-kB;Innate Immune Response;Growth Factor Signaling;Cytokine Signaling;Autophagy;Apoptosis | mmu04014;mmu04064;mmu04151;mmu04210;mmu04630;mmu05014;mmu05145;mmu05166;mmu05200;mmu05202;mmu05212;mmu05220;mmu05222 |  | Bcl2l1 | slateblue1 | 16 | 5.64032697547684 |
  | Traf6-mRNA | 0.92 | 0.166 | 0.594 | 1.25 | 1.89 | 1.51 | 2.37 | 4.54e-05 | 0.00214 | lm.nb | Adaptive Immune Response, Apoptosis, Autophagy, Cytokine Signaling, Growth Factor Signaling, Innate Immune Response, NF-kB | NS\_MM\_NEUROINFLAM\_V1.0 | Traf6 | mRNA | 0 |  | NF-kB;Innate Immune Response;Growth Factor Signaling;Cytokine Signaling;Autophagy;Apoptosis;Adaptive Immune Response | mmu04010;mmu04064;mmu04120;mmu04144;mmu04380;mmu04620;mmu04621;mmu04622;mmu04722;mmu05133;mmu05140;mmu05142;mmu05145;mmu05152;mmu05160;mmu05162;mmu05168;mmu05169;mmu05200;mmu05222 |  | Traf6 | slateblue1 | 16 | 5.54216867469879 |
  | Akt2-mRNA | -0.766 | 0.142 | -1.04 | -0.488 | 0.588 | 0.485 | 0.713 | 5.85e-05 | 0.00265 | loglinear | Adaptive Immune Response, Angiogenesis, Apoptosis, Autophagy, Carbohydrate Metabolism, Cytokine Signaling, Growth Factor Signaling, Innate Immune Response, Insulin Signaling, Neurons and Neurotransmission, Wnt | NS\_MM\_NEUROINFLAM\_V1.0 | Akt2 | mRNA | 0 |  | Wnt;Neurons and Neurotransmission;Insulin Signaling;Innate Immune Response;Growth Factor Signaling;Cytokine Signaling;Carbohydrate Metabolism;Autophagy;Apoptosis;Angiogenesis;Adaptive Immune Response | mmu04010;mmu04012;mmu04014;mmu04015;mmu04022;mmu04024;mmu04062;mmu04066;mmu04068;mmu04071;mmu04072;mmu04150;mmu04151;mmu04152;mmu04210;mmu04211;mmu04213;mmu04261;mmu04370;mmu04380;mmu04510;mmu04530;mmu04550;mmu04611;mmu04620;mmu04630;mmu04660;mmu04662;mmu04664;mmu04666;mmu04668;mmu04722;mmu04725;mmu04728;mmu04910;mmu04914;mmu04915;mmu04917;mmu04919;mmu04920;mmu04922;mmu04923;mmu04931;mmu04932;mmu04933;mmu04973;mmu05142;mmu05145;mmu05152;mmu05160;mmu05161;mmu05162;mmu05164;mmu05166;mmu05169;mmu05200;mmu05205;mmu05210;mmu05211;mmu05212;mmu05213;mmu05214;mmu05215;mmu05218;mmu05220;mmu05221;mmu05222;mmu05223;mmu05230;mmu05231 |  | Akt2 | slateblue1 | 16 | -5.3943661971831 |
  | Chuk-mRNA | -0.423 | 0.0787 | -0.577 | -0.269 | 0.746 | 0.67 | 0.83 | 6.21e-05 | 0.00275 | loglinear | Adaptive Immune Response, Apoptosis, Cytokine Signaling, Growth Factor Signaling, Inflammatory Signaling, Innate Immune Response, NF-kB | NS\_MM\_NEUROINFLAM\_V1.0 | Chuk | mRNA | 0 |  | NF-kB;Innate Immune Response;Inflammatory Signaling;Growth Factor Signaling;Cytokine Signaling;Apoptosis;Adaptive Immune Response | mmu04010;mmu04014;mmu04062;mmu04064;mmu04068;mmu04151;mmu04210;mmu04380;mmu04620;mmu04621;mmu04622;mmu04623;mmu04660;mmu04662;mmu04668;mmu04920;mmu05142;mmu05145;mmu05160;mmu05161;mmu05162;mmu05166;mmu05168;mmu05169;mmu05200;mmu05212;mmu05215;mmu05220;mmu05221;mmu05222 |  | Chuk | slateblue1 | 16 | -5.37484116899619 |
  | Ripk2-mRNA | 1 | 0.193 | 0.627 | 1.38 | 2.01 | 1.54 | 2.61 | 8.44e-05 | 0.00355 | loglinear | Adaptive Immune Response, Apoptosis, Cytokine Signaling, Growth Factor Signaling, Innate Immune Response | NS\_MM\_NEUROINFLAM\_V1.0 | Ripk2 | mRNA | 0 |  | Innate Immune Response;Growth Factor Signaling;Cytokine Signaling;Apoptosis;Adaptive Immune Response | mmu04621;mmu04722;mmu05152 |  | Ripk2 | slateblue1 | 16 | 5.18134715025907 |
  | Tgfbr1-mRNA | -0.53 | 0.102 | -0.729 | -0.331 | 0.692 | 0.603 | 0.795 | 8.46e-05 | 0.00355 | loglinear | Cytokine Signaling, Growth Factor Signaling | NS\_MM\_NEUROINFLAM\_V1.0 | Tgfbr1 | mRNA | 0 |  | Growth Factor Signaling;Cytokine Signaling | mmu04010;mmu04060;mmu04068;mmu04144;mmu04350;mmu04380;mmu04390;mmu04520;mmu04933;mmu05142;mmu05161;mmu05166;mmu05200;mmu05210;mmu05212;mmu05220 |  | Tgfbr1 | slateblue1 | 16 | -5.19607843137255 |
  | Csf1r-mRNA | -0.702 | 0.144 | -0.985 | -0.419 | 0.615 | 0.505 | 0.748 | 0.000171 | 0.0067 | lm.nb | Cytokine Signaling, Growth Factor Signaling, Microglia Function | NS\_MM\_NEUROINFLAM\_V1.0 | Csf1r | mRNA | 0 |  | Microglia Function;Growth Factor Signaling;Cytokine Signaling | mmu04014;mmu04015;mmu04060;mmu04144;mmu04151;mmu04380;mmu04640;mmu05200;mmu05202 |  | Csf1r | slateblue1 | 16 | -4.875 |
  | Il6ra-mRNA | 1.68 | 0.346 | 0.997 | 2.35 | 3.19 | 2 | 5.11 | 0.000181 | 0.00695 | lm.nb | Cytokine Signaling, Growth Factor Signaling, Microglia Function | NS\_MM\_NEUROINFLAM\_V1.0 | Il6ra | mRNA | 0 |  | Microglia Function;Growth Factor Signaling;Cytokine Signaling | mmu04060;mmu04066;mmu04151;mmu04630;mmu04640;mmu04932 |  | Il6ra | slateblue1 | 16 | 4.85549132947977 |
  | Traf3-mRNA | -0.463 | 0.104 | -0.666 | -0.26 | 0.725 | 0.63 | 0.835 | 0.000386 | 0.013 | loglinear | Apoptosis, Cytokine Signaling, Inflammatory Signaling, Innate Immune Response, NF-kB | NS\_MM\_NEUROINFLAM\_V1.0 | Traf3 | mRNA | 0 |  | NF-kB;Innate Immune Response;Inflammatory Signaling;Cytokine Signaling;Apoptosis | mmu04064;mmu04620;mmu04622;mmu04668;mmu05160;mmu05168;mmu05169;mmu05200;mmu05203;mmu05222 |  | Traf3 | slateblue2 | 16 | -4.45192307692308 |
  | Traf2-mRNA | -0.552 | 0.129 | -0.805 | -0.298 | 0.682 | 0.572 | 0.813 | 0.000592 | 0.0183 | lm.nb | Apoptosis, Cytokine Signaling, Growth Factor Signaling, Inflammatory Signaling, Innate Immune Response, NF-kB | NS\_MM\_NEUROINFLAM\_V1.0 | Traf2 | mRNA | 0 |  | NF-kB;Innate Immune Response;Inflammatory Signaling;Growth Factor Signaling;Cytokine Signaling;Apoptosis | mmu04010;mmu04064;mmu04071;mmu04141;mmu04210;mmu04380;mmu04622;mmu04668;mmu04920;mmu04932;mmu05160;mmu05168;mmu05169;mmu05200;mmu05203;mmu05222 |  | Traf2 | slateblue2 | 16 | -4.27906976744186 |
  | Bcl2-mRNA | -0.943 | 0.227 | -1.39 | -0.498 | 0.52 | 0.382 | 0.708 | 0.000747 | 0.0225 | lm.nb | Apoptosis, Autophagy, Cellular Stress, Cytokine Signaling, DNA Damage, Growth Factor Signaling, Innate Immune Response, Microglia Function, NF-kB | NS\_MM\_NEUROINFLAM\_V1.0 | Bcl2 | mRNA | 0 |  | NF-kB;Microglia Function;Innate Immune Response;Growth Factor Signaling;DNA Damage;Cytokine Signaling;Cellular Stress;Autophagy;Apoptosis | mmu04064;mmu04066;mmu04071;mmu04141;mmu04151;mmu04210;mmu04261;mmu04510;mmu04630;mmu04722;mmu04725;mmu04933;mmu05014;mmu05145;mmu05152;mmu05161;mmu05169;mmu05200;mmu05206;mmu05210;mmu05215;mmu05222 |  | Bcl2 | slateblue2 | 16 | -4.15418502202643 |
  | Irak3-mRNA | 2.06 | 0.508 | 1.06 | 3.05 | 4.16 | 2.08 | 8.29 | 0.000934 | 0.0267 | lm.nb | Apoptosis, Cytokine Signaling, Innate Immune Response | NS\_MM\_NEUROINFLAM\_V1.0 | Irak3 | mRNA | 0 |  | Innate Immune Response;Cytokine Signaling;Apoptosis | mmu04210;mmu04722 |  | Irak3 | slateblue2 | 16 | 4.05511811023622 |
  | Pik3cd-mRNA | -0.63 | 0.159 | -0.942 | -0.318 | 0.646 | 0.52 | 0.802 | 0.00114 | 0.0306 | lm.nb | Adaptive Immune Response, Apoptosis, Autophagy, Cytokine Signaling, Growth Factor Signaling, Innate Immune Response | NS\_MM\_NEUROINFLAM\_V1.0 | Pik3cd | mRNA | 0 |  | Innate Immune Response;Growth Factor Signaling;Cytokine Signaling;Autophagy;Apoptosis;Adaptive Immune Response |  |  |  | slateblue2 | 16 | -3.9622641509434 |
  | Dlg4-mRNA | -0.204 | 0.056 | -0.313 | -0.0938 | 0.868 | 0.805 | 0.937 | 0.00223 | 0.0534 | loglinear | Adaptive Immune Response, Angiogenesis, Cytokine Signaling, Growth Factor Signaling, Insulin Signaling, Neurons and Neurotransmission | NS\_MM\_NEUROINFLAM\_V1.0 | Dlg4 | mRNA | 0 |  | Neurons and Neurotransmission;Insulin Signaling;Growth Factor Signaling;Cytokine Signaling;Angiogenesis;Adaptive Immune Response | mmu04390;mmu04724;mmu05016;mmu05030 |  | Dlg4 | slateblue3 | 16 | -3.64285714285714 |

  ##### DE Results - Cytokine Signaling genes - DPI: 3 vs.CTRL

  Table displaying each sample's global significance scores and directed global significance scores as defined in the heatmaps above. The global significance score is calculated as the square root of the mean squared t-statistic for the genes in a gene set, with t-statistics coming from the linear regression underlying our differential expression analysis. The directed global significance score is calculated as the square root of the mean signed squared t-statistic for the genes in a gene set, with t-statistics coming from the linear regression underlying our differential expression analysis.

DPI:
- DPI: differential expression in 1 vs. baseline of CTRL
- DPI: differential expression in 2 vs. baseline of CTRL
- DPI: differential expression in 3 vs. baseline of CTRL

- **Volcano Plot: DPI: 1 vs.CTRL  
  More Plot Information**


  ##### Volcano Plot: DPI: 1 vs.CTRL

  Volcano plot displaying each gene's -log10(p-value) and log2 fold change for the selected covariate. Highly statistically significant genes fall at the top of the plot, and highly differentially expressed genes fall to either side. Genes within the selected gene set are highlighted in orange. Horizontal lines indicate various False Discovery Rate (FDR) thresholds.
- **DE Results - DNA Damage genes - DPI: 1 vs.CTRL  
  More Plot Information  Download CSV Data**

  | Probe Label | Log2 fold change | std error (log2) | Lower confidence limit (log2) | Upper confidence limit (log2) | Linear fold change | Lower confidence limit (linear) | Upper confidence limit (linear) | P-value | BY.p.value | method | Gene.sets | Codeset.Name | Probe.Label | Analyte.Type | Is.Control | Control.Type | Probe.Annotation | KEGG.Pathways | Cell.Type | Official.Gene.Name | volcanocol | volcanopch | tstats.all |
  | --- | --- | --- | --- | --- | --- | --- | --- | --- | --- | --- | --- | --- | --- | --- | --- | --- | --- | --- | --- | --- | --- | --- | --- |
  | Mdm2-mRNA | -0.317 | 0.0642 | -0.443 | -0.191 | 0.803 | 0.736 | 0.876 | 0.00015 | 0.0406 | loglinear | Adaptive Immune Response, Apoptosis, Cell Cycle, Cellular Stress, DNA Damage, Growth Factor Signaling, Neurons and Neurotransmission | NS\_MM\_NEUROINFLAM\_V1.0 | Mdm2 | mRNA | 0 |  | Neurons and Neurotransmission;Growth Factor Signaling;DNA Damage;Cellular Stress;Cell Cycle;Apoptosis;Adaptive Immune Response | mmu04068;mmu04110;mmu04115;mmu04120;mmu04144;mmu04151;mmu04919;mmu05169;mmu05200;mmu05202;mmu05203;mmu05205;mmu05206;mmu05214;mmu05215;mmu05218;mmu05219;mmu05220 |  | Mdm2 | slateblue2 | 16 | -4.93769470404984 |
  | Smc1a-mRNA | -0.477 | 0.103 | -0.68 | -0.275 | 0.718 | 0.624 | 0.827 | 0.000287 | 0.0536 | lm.nb | Cell Cycle, DNA Damage | NS\_MM\_NEUROINFLAM\_V1.0 | Smc1a | mRNA | 0 |  | DNA Damage;Cell Cycle | mmu04110;mmu04114 |  | Smc1a | slateblue3 | 16 | -4.63106796116505 |
  | Parp1-mRNA | -0.49 | 0.108 | -0.703 | -0.278 | 0.712 | 0.614 | 0.825 | 0.000348 | 0.0564 | loglinear | Apoptosis, Cellular Stress, DNA Damage, NF-kB | NS\_MM\_NEUROINFLAM\_V1.0 | Parp1 | mRNA | 0 |  | NF-kB;DNA Damage;Cellular Stress;Apoptosis | mmu03410;mmu04064 |  | Parp1 | slateblue3 | 16 | -4.53703703703704 |
  | Rad9a-mRNA | -0.968 | 0.244 | -1.45 | -0.489 | 0.511 | 0.367 | 0.713 | 0.00112 | 0.104 | lm.nb | Cell Cycle, DNA Damage | NS\_MM\_NEUROINFLAM\_V1.0 | Rad9a | mRNA | 0 |  | DNA Damage;Cell Cycle |  |  | Rad9a | slateblue4 | 16 | -3.9672131147541 |
  | Pten-mRNA | -0.296 | 0.0753 | -0.444 | -0.148 | 0.815 | 0.735 | 0.902 | 0.0012 | 0.109 | loglinear | Adaptive Immune Response, Autophagy, DNA Damage, Growth Factor Signaling, Lipid Metabolism | NS\_MM\_NEUROINFLAM\_V1.0 | Pten | mRNA | 0 |  | Lipid Metabolism;Growth Factor Signaling;DNA Damage;Autophagy;Adaptive Immune Response | mmu00562;mmu04068;mmu04070;mmu04071;mmu04115;mmu04150;mmu04151;mmu04510;mmu04530;mmu04931;mmu05161;mmu05200;mmu05206;mmu05213;mmu05214;mmu05215;mmu05218;mmu05222;mmu05230 |  | Pten | slateblue4 | 16 | -3.93094289508632 |
  | Gadd45a-mRNA | -0.491 | 0.13 | -0.746 | -0.237 | 0.711 | 0.596 | 0.849 | 0.00162 | 0.127 | lm.nb | Cell Cycle, DNA Damage, Growth Factor Signaling | NS\_MM\_NEUROINFLAM\_V1.0 | Gadd45a | mRNA | 0 |  | Growth Factor Signaling;DNA Damage;Cell Cycle | mmu04010;mmu04068;mmu04110;mmu04115 |  | Gadd45a | slateblue4 | 16 | -3.77692307692308 |
  | Nbn-mRNA | -0.429 | 0.115 | -0.654 | -0.205 | 0.743 | 0.636 | 0.868 | 0.00176 | 0.129 | loglinear | Cell Cycle, Cellular Stress, DNA Damage | NS\_MM\_NEUROINFLAM\_V1.0 | Nbn | mRNA | 0 |  | DNA Damage;Cellular Stress;Cell Cycle | mmu03440 |  | Nbn | slateblue4 | 16 | -3.7304347826087 |
  | Sumo1-mRNA | -0.424 | 0.115 | -0.65 | -0.198 | 0.745 | 0.637 | 0.871 | 0.00202 | 0.136 | lm.nb | Cell Cycle, Cytokine Signaling, DNA Damage, Inflammatory Signaling | NS\_MM\_NEUROINFLAM\_V1.0 | Sumo1 | mRNA | 0 |  | Inflammatory Signaling;DNA Damage;Cytokine Signaling;Cell Cycle |  |  | Sumo1 | slateblue4 | 16 | -3.68695652173913 |
  | Cdkn1a-mRNA | -0.625 | 0.18 | -0.979 | -0.272 | 0.648 | 0.507 | 0.828 | 0.00317 | 0.18 | lm.nb | Adaptive Immune Response, Cell Cycle, Cellular Stress, Cytokine Signaling, DNA Damage, Growth Factor Signaling | NS\_MM\_NEUROINFLAM\_V1.0 | Cdkn1a | mRNA | 0 |  | Growth Factor Signaling;DNA Damage;Cytokine Signaling;Cellular Stress;Cell Cycle;Adaptive Immune Response | mmu04012;mmu04066;mmu04068;mmu04110;mmu04115;mmu04151;mmu04630;mmu04921;mmu05160;mmu05161;mmu05166;mmu05169;mmu05200;mmu05202;mmu05203;mmu05205;mmu05206;mmu05214;mmu05215;mmu05218;mmu05219;mmu05220 |  | Cdkn1a | slateblue4 | 16 | -3.47222222222222 |
  | Blm-mRNA | -1.87 | 0.546 | -2.94 | -0.796 | 0.274 | 0.131 | 0.576 | 0.00353 | 0.184 | loglinear | Cell Cycle, DNA Damage | NS\_MM\_NEUROINFLAM\_V1.0 | Blm | mRNA | 0 |  | DNA Damage;Cell Cycle | mmu03440;mmu03460 |  | Blm | slateblue4 | 16 | -3.42490842490842 |
  | Topbp1-mRNA | -0.646 | 0.193 | -1.02 | -0.268 | 0.639 | 0.492 | 0.83 | 0.00406 | 0.196 | lm.nb | DNA Damage | NS\_MM\_NEUROINFLAM\_V1.0 | Topbp1 | mRNA | 0 |  | DNA Damage |  |  | Topbp1 | slateblue4 | 16 | -3.34715025906736 |
  | Casp9-mRNA | -1.44 | 0.433 | -2.29 | -0.59 | 0.369 | 0.205 | 0.664 | 0.00432 | 0.2 | lm.nb | Adaptive Immune Response, Apoptosis, DNA Damage, Growth Factor Signaling, Innate Immune Response | NS\_MM\_NEUROINFLAM\_V1.0 | Casp9 | mRNA | 0 |  | Innate Immune Response;Growth Factor Signaling;DNA Damage;Apoptosis;Adaptive Immune Response | mmu04115;mmu04151;mmu04210;mmu04370;mmu04919;mmu05010;mmu05012;mmu05014;mmu05016;mmu05134;mmu05145;mmu05152;mmu05161;mmu05164;mmu05200;mmu05210;mmu05212;mmu05213;mmu05215;mmu05222;mmu05223;mmu05416 |  | Casp9 | slateblue4 | 16 | -3.3256351039261 |
  | Smarca5-mRNA | -0.143 | 0.0451 | -0.232 | -0.0549 | 0.905 | 0.852 | 0.963 | 0.00583 | 0.234 | loglinear | Cell Cycle, DNA Damage, Epigenetic Regulation | NS\_MM\_NEUROINFLAM\_V1.0 | Smarca5 | mRNA | 0 |  | Epigenetic Regulation;DNA Damage;Cell Cycle |  |  | Smarca5 | slateblue4 | 16 | -3.17073170731707 |
  | Hus1-mRNA | -0.528 | 0.172 | -0.866 | -0.191 | 0.693 | 0.549 | 0.876 | 0.00735 | 0.254 | lm.nb | Cell Cycle, DNA Damage | NS\_MM\_NEUROINFLAM\_V1.0 | Hus1 | mRNA | 0 |  | DNA Damage;Cell Cycle |  |  | Hus1 | slateblue4 | 16 | -3.06976744186047 |
  | Msh2-mRNA | -0.334 | 0.112 | -0.553 | -0.115 | 0.793 | 0.682 | 0.923 | 0.00863 | 0.282 | loglinear | DNA Damage | NS\_MM\_NEUROINFLAM\_V1.0 | Msh2 | mRNA | 0 |  | DNA Damage | mmu03430;mmu05200;mmu05210 |  | Msh2 | slateblue4 | 16 | -2.98214285714286 |
  | Ddb2-mRNA | -0.605 | 0.204 | -1 | -0.206 | 0.657 | 0.499 | 0.867 | 0.00905 | 0.286 | loglinear | DNA Damage | NS\_MM\_NEUROINFLAM\_V1.0 | Ddb2 | mRNA | 0 |  | DNA Damage | mmu03420;mmu04115;mmu04120;mmu05161 |  | Ddb2 | slateblue4 | 16 | -2.9656862745098 |
  | Nthl1-mRNA | 0.519 | 0.186 | 0.154 | 0.883 | 1.43 | 1.11 | 1.84 | 0.0131 | 0.364 | loglinear | DNA Damage | NS\_MM\_NEUROINFLAM\_V1.0 | Nthl1 | mRNA | 0 |  | DNA Damage | mmu03410 |  | Nthl1 | slateblue4 | 16 | 2.79032258064516 |
  | Casp3-mRNA | -0.413 | 0.154 | -0.716 | -0.111 | 0.751 | 0.609 | 0.926 | 0.0165 | 0.417 | lm.nb | Apoptosis, Cellular Stress, DNA Damage, Growth Factor Signaling, Innate Immune Response, Matrix Remodeling | NS\_MM\_NEUROINFLAM\_V1.0 | Casp3 | mRNA | 0 |  | Matrix Remodeling;Innate Immune Response;Growth Factor Signaling;DNA Damage;Cellular Stress;Apoptosis | mmu04010;mmu04115;mmu04210;mmu04650;mmu04668;mmu04726;mmu04932;mmu04933;mmu05010;mmu05012;mmu05014;mmu05016;mmu05133;mmu05134;mmu05145;mmu05146;mmu05152;mmu05161;mmu05168;mmu05200;mmu05203;mmu05205;mmu05206;mmu05210;mmu05416 |  | Casp3 | slateblue4 | 16 | -2.68181818181818 |
  | Kdm4b-mRNA | 0.156 | 0.0584 | 0.0416 | 0.271 | 1.11 | 1.03 | 1.21 | 0.0167 | 0.418 | loglinear | DNA Damage, Epigenetic Regulation | NS\_MM\_NEUROINFLAM\_V1.0 | Kdm4b | mRNA | 0 |  | Epigenetic Regulation;DNA Damage |  |  | Kdm4b | slateblue4 | 16 | 2.67123287671233 |
  | Pms2-mRNA | -0.359 | 0.135 | -0.624 | -0.0933 | 0.78 | 0.649 | 0.937 | 0.0175 | 0.43 | lm.nb | DNA Damage | NS\_MM\_NEUROINFLAM\_V1.0 | Pms2 | mRNA | 0 |  | DNA Damage | mmu03430;mmu03460 |  | Pms2 | slateblue4 | 16 | -2.65925925925926 |

  ##### DE Results - DNA Damage genes - DPI: 1 vs.CTRL

  Table displaying each sample's global significance scores and directed global significance scores as defined in the heatmaps above. The global significance score is calculated as the square root of the mean squared t-statistic for the genes in a gene set, with t-statistics coming from the linear regression underlying our differential expression analysis. The directed global significance score is calculated as the square root of the mean signed squared t-statistic for the genes in a gene set, with t-statistics coming from the linear regression underlying our differential expression analysis.

- **Volcano Plot: DPI: 2 vs.CTRL  
  More Plot Information**


  ##### Volcano Plot: DPI: 2 vs.CTRL

  Volcano plot displaying each gene's -log10(p-value) and log2 fold change for the selected covariate. Highly statistically significant genes fall at the top of the plot, and highly differentially expressed genes fall to either side. Genes within the selected gene set are highlighted in orange. Horizontal lines indicate various False Discovery Rate (FDR) thresholds.
- **DE Results - DNA Damage genes - DPI: 2 vs.CTRL  
  More Plot Information  Download CSV Data**

  | Probe Label | Log2 fold change | std error (log2) | Lower confidence limit (log2) | Upper confidence limit (log2) | Linear fold change | Lower confidence limit (linear) | Upper confidence limit (linear) | P-value | BY.p.value | method | Gene.sets | Codeset.Name | Probe.Label | Analyte.Type | Is.Control | Control.Type | Probe.Annotation | KEGG.Pathways | Cell.Type | Official.Gene.Name | volcanocol | volcanopch | tstats.all |
  | --- | --- | --- | --- | --- | --- | --- | --- | --- | --- | --- | --- | --- | --- | --- | --- | --- | --- | --- | --- | --- | --- | --- | --- |
  | Cdkn1a-mRNA | 2.13 | 0.171 | 1.79 | 2.46 | 4.37 | 3.47 | 5.52 | 1.24e-09 | 8.78e-07 | lm.nb | Adaptive Immune Response, Cell Cycle, Cellular Stress, Cytokine Signaling, DNA Damage, Growth Factor Signaling | NS\_MM\_NEUROINFLAM\_V1.0 | Cdkn1a | mRNA | 0 |  | Growth Factor Signaling;DNA Damage;Cytokine Signaling;Cellular Stress;Cell Cycle;Adaptive Immune Response | mmu04012;mmu04066;mmu04068;mmu04110;mmu04115;mmu04151;mmu04630;mmu04921;mmu05160;mmu05161;mmu05166;mmu05169;mmu05200;mmu05202;mmu05203;mmu05205;mmu05206;mmu05214;mmu05215;mmu05218;mmu05219;mmu05220 |  | Cdkn1a | slateblue1 | 16 | 12.4561403508772 |
  | Sesn1-mRNA | 0.59 | 0.0913 | 0.412 | 0.769 | 1.51 | 1.33 | 1.7 | 7.77e-06 | 0.000831 | loglinear | DNA Damage | NS\_MM\_NEUROINFLAM\_V1.0 | Sesn1 | mRNA | 0 |  | DNA Damage | mmu04115;mmu04211 |  | Sesn1 | slateblue1 | 16 | 6.46221248630887 |
  | Blm-mRNA | -3.42 | 0.546 | -4.49 | -2.35 | 0.0933 | 0.0444 | 0.196 | 1.13e-05 | 0.0011 | loglinear | Cell Cycle, DNA Damage | NS\_MM\_NEUROINFLAM\_V1.0 | Blm | mRNA | 0 |  | DNA Damage;Cell Cycle | mmu03440;mmu03460 |  | Blm | slateblue1 | 16 | -6.26373626373626 |
  | Smarca5-mRNA | -0.281 | 0.0451 | -0.369 | -0.192 | 0.823 | 0.774 | 0.875 | 1.21e-05 | 0.0011 | loglinear | Cell Cycle, DNA Damage, Epigenetic Regulation | NS\_MM\_NEUROINFLAM\_V1.0 | Smarca5 | mRNA | 0 |  | Epigenetic Regulation;DNA Damage;Cell Cycle |  |  | Smarca5 | slateblue1 | 16 | -6.23059866962306 |
  | Smc1a-mRNA | -0.615 | 0.104 | -0.819 | -0.412 | 0.653 | 0.567 | 0.752 | 2.17e-05 | 0.00178 | lm.nb | Cell Cycle, DNA Damage | NS\_MM\_NEUROINFLAM\_V1.0 | Smc1a | mRNA | 0 |  | DNA Damage;Cell Cycle | mmu04110;mmu04114 |  | Smc1a | slateblue1 | 16 | -5.91346153846154 |
  | Kdm4b-mRNA | 0.314 | 0.0584 | 0.199 | 0.428 | 1.24 | 1.15 | 1.35 | 6.26e-05 | 0.00387 | loglinear | DNA Damage, Epigenetic Regulation | NS\_MM\_NEUROINFLAM\_V1.0 | Kdm4b | mRNA | 0 |  | Epigenetic Regulation;DNA Damage |  |  | Kdm4b | slateblue1 | 16 | 5.37671232876712 |
  | Sumo1-mRNA | -0.529 | 0.115 | -0.755 | -0.303 | 0.693 | 0.593 | 0.811 | 0.000305 | 0.0121 | lm.nb | Cell Cycle, Cytokine Signaling, DNA Damage, Inflammatory Signaling | NS\_MM\_NEUROINFLAM\_V1.0 | Sumo1 | mRNA | 0 |  | Inflammatory Signaling;DNA Damage;Cytokine Signaling;Cell Cycle |  |  | Sumo1 | slateblue2 | 16 | -4.6 |
  | Rad9a-mRNA | -1.13 | 0.249 | -1.62 | -0.642 | 0.457 | 0.326 | 0.641 | 0.000333 | 0.0129 | lm.nb | Cell Cycle, DNA Damage | NS\_MM\_NEUROINFLAM\_V1.0 | Rad9a | mRNA | 0 |  | DNA Damage;Cell Cycle |  |  | Rad9a | slateblue2 | 16 | -4.53815261044177 |
  | Gadd45a-mRNA | -0.561 | 0.13 | -0.817 | -0.305 | 0.678 | 0.568 | 0.809 | 0.000551 | 0.0187 | lm.nb | Cell Cycle, DNA Damage, Growth Factor Signaling | NS\_MM\_NEUROINFLAM\_V1.0 | Gadd45a | mRNA | 0 |  | Growth Factor Signaling;DNA Damage;Cell Cycle | mmu04010;mmu04068;mmu04110;mmu04115 |  | Gadd45a | slateblue2 | 16 | -4.31538461538462 |
  | Nbn-mRNA | -0.476 | 0.115 | -0.7 | -0.251 | 0.719 | 0.616 | 0.84 | 0.00075 | 0.0225 | loglinear | Cell Cycle, Cellular Stress, DNA Damage | NS\_MM\_NEUROINFLAM\_V1.0 | Nbn | mRNA | 0 |  | DNA Damage;Cellular Stress;Cell Cycle | mmu03440 |  | Nbn | slateblue2 | 16 | -4.13913043478261 |
  | Casp3-mRNA | -0.652 | 0.157 | -0.96 | -0.344 | 0.636 | 0.514 | 0.788 | 0.000753 | 0.0225 | lm.nb | Apoptosis, Cellular Stress, DNA Damage, Growth Factor Signaling, Innate Immune Response, Matrix Remodeling | NS\_MM\_NEUROINFLAM\_V1.0 | Casp3 | mRNA | 0 |  | Matrix Remodeling;Innate Immune Response;Growth Factor Signaling;DNA Damage;Cellular Stress;Apoptosis | mmu04010;mmu04115;mmu04210;mmu04650;mmu04668;mmu04726;mmu04932;mmu04933;mmu05010;mmu05012;mmu05014;mmu05016;mmu05133;mmu05134;mmu05145;mmu05146;mmu05152;mmu05161;mmu05168;mmu05200;mmu05203;mmu05205;mmu05206;mmu05210;mmu05416 |  | Casp3 | slateblue2 | 16 | -4.15286624203822 |
  | Pten-mRNA | -0.304 | 0.0753 | -0.452 | -0.156 | 0.81 | 0.731 | 0.897 | 0.000959 | 0.0266 | loglinear | Adaptive Immune Response, Autophagy, DNA Damage, Growth Factor Signaling, Lipid Metabolism | NS\_MM\_NEUROINFLAM\_V1.0 | Pten | mRNA | 0 |  | Lipid Metabolism;Growth Factor Signaling;DNA Damage;Autophagy;Adaptive Immune Response | mmu00562;mmu04068;mmu04070;mmu04071;mmu04115;mmu04150;mmu04151;mmu04510;mmu04530;mmu04931;mmu05161;mmu05200;mmu05206;mmu05213;mmu05214;mmu05215;mmu05218;mmu05222;mmu05230 |  | Pten | slateblue2 | 16 | -4.03718459495352 |
  | Bcl2-mRNA | -0.88 | 0.225 | -1.32 | -0.439 | 0.543 | 0.4 | 0.737 | 0.00123 | 0.0313 | lm.nb | Apoptosis, Autophagy, Cellular Stress, Cytokine Signaling, DNA Damage, Growth Factor Signaling, Innate Immune Response, Microglia Function, NF-kB | NS\_MM\_NEUROINFLAM\_V1.0 | Bcl2 | mRNA | 0 |  | NF-kB;Microglia Function;Innate Immune Response;Growth Factor Signaling;DNA Damage;Cytokine Signaling;Cellular Stress;Autophagy;Apoptosis | mmu04064;mmu04066;mmu04071;mmu04141;mmu04151;mmu04210;mmu04261;mmu04510;mmu04630;mmu04722;mmu04725;mmu04933;mmu05014;mmu05145;mmu05152;mmu05161;mmu05169;mmu05200;mmu05206;mmu05210;mmu05215;mmu05222 |  | Bcl2 | slateblue2 | 16 | -3.91111111111111 |
  | H2afx-mRNA | -0.214 | 0.0562 | -0.325 | -0.104 | 0.862 | 0.799 | 0.93 | 0.00154 | 0.0372 | loglinear | Cell Cycle, Cellular Stress, DNA Damage, Epigenetic Regulation, Wnt | NS\_MM\_NEUROINFLAM\_V1.0 | H2afx | mRNA | 0 |  | Wnt;Epigenetic Regulation;DNA Damage;Cellular Stress;Cell Cycle | mmu05034;mmu05322 |  | H2afx | slateblue2 | 16 | -3.80782918149466 |
  | Hus1-mRNA | -0.655 | 0.174 | -0.996 | -0.314 | 0.635 | 0.501 | 0.804 | 0.00169 | 0.0397 | lm.nb | Cell Cycle, DNA Damage | NS\_MM\_NEUROINFLAM\_V1.0 | Hus1 | mRNA | 0 |  | DNA Damage;Cell Cycle |  |  | Hus1 | slateblue2 | 16 | -3.76436781609195 |
  | Parp1-mRNA | -0.406 | 0.108 | -0.619 | -0.194 | 0.754 | 0.651 | 0.874 | 0.00175 | 0.0406 | loglinear | Apoptosis, Cellular Stress, DNA Damage, NF-kB | NS\_MM\_NEUROINFLAM\_V1.0 | Parp1 | mRNA | 0 |  | NF-kB;DNA Damage;Cellular Stress;Apoptosis | mmu03410;mmu04064 |  | Parp1 | slateblue2 | 16 | -3.75925925925926 |
  | Casp9-mRNA | -1.57 | 0.435 | -2.43 | -0.721 | 0.336 | 0.186 | 0.607 | 0.00231 | 0.0504 | lm.nb | Adaptive Immune Response, Apoptosis, DNA Damage, Growth Factor Signaling, Innate Immune Response | NS\_MM\_NEUROINFLAM\_V1.0 | Casp9 | mRNA | 0 |  | Innate Immune Response;Growth Factor Signaling;DNA Damage;Apoptosis;Adaptive Immune Response | mmu04115;mmu04151;mmu04210;mmu04370;mmu04919;mmu05010;mmu05012;mmu05014;mmu05016;mmu05134;mmu05145;mmu05152;mmu05161;mmu05164;mmu05200;mmu05210;mmu05212;mmu05213;mmu05215;mmu05222;mmu05223;mmu05416 |  | Casp9 | slateblue3 | 16 | -3.60919540229885 |
  | Rnf8-mRNA | -0.298 | 0.0965 | -0.487 | -0.109 | 0.813 | 0.713 | 0.927 | 0.0071 | 0.123 | loglinear | Cell Cycle, DNA Damage | NS\_MM\_NEUROINFLAM\_V1.0 | Rnf8 | mRNA | 0 |  | DNA Damage;Cell Cycle |  |  | Rnf8 | slateblue4 | 16 | -3.0880829015544 |
  | Ep300-mRNA | -0.33 | 0.111 | -0.547 | -0.114 | 0.795 | 0.684 | 0.924 | 0.0087 | 0.144 | loglinear | Cell Cycle, Cellular Stress, Cytokine Signaling, DNA Damage, Epigenetic Regulation, Inflammatory Signaling, Innate Immune Response, Lipid Metabolism, Notch, Wnt | NS\_MM\_NEUROINFLAM\_V1.0 | Ep300 | mRNA | 0 |  | Wnt;Notch;Lipid Metabolism;Innate Immune Response;Inflammatory Signaling;Epigenetic Regulation;DNA Damage;Cytokine Signaling;Cellular Stress;Cell Cycle | mmu04024;mmu04066;mmu04068;mmu04110;mmu04310;mmu04330;mmu04350;mmu04520;mmu04630;mmu04720;mmu04916;mmu04919;mmu04922;mmu05016;mmu05152;mmu05161;mmu05164;mmu05166;mmu05168;mmu05169;mmu05200;mmu05203;mmu05206;mmu05211;mmu05215 |  | Ep300 | slateblue4 | 16 | -2.97297297297297 |
  | Mre11a-mRNA | -0.261 | 0.0903 | -0.438 | -0.0836 | 0.835 | 0.738 | 0.944 | 0.0107 | 0.175 | loglinear | DNA Damage | NS\_MM\_NEUROINFLAM\_V1.0 | Mre11a | mRNA | 0 |  | DNA Damage | mmu03440;mmu03450 |  | Mre11a | slateblue4 | 16 | -2.89036544850498 |

  ##### DE Results - DNA Damage genes - DPI: 2 vs.CTRL

  Table displaying each sample's global significance scores and directed global significance scores as defined in the heatmaps above. The global significance score is calculated as the square root of the mean squared t-statistic for the genes in a gene set, with t-statistics coming from the linear regression underlying our differential expression analysis. The directed global significance score is calculated as the square root of the mean signed squared t-statistic for the genes in a gene set, with t-statistics coming from the linear regression underlying our differential expression analysis.

- **Volcano Plot: DPI: 3 vs.CTRL  
  More Plot Information**


  ##### Volcano Plot: DPI: 3 vs.CTRL

  Volcano plot displaying each gene's -log10(p-value) and log2 fold change for the selected covariate. Highly statistically significant genes fall at the top of the plot, and highly differentially expressed genes fall to either side. Genes within the selected gene set are highlighted in orange. Horizontal lines indicate various False Discovery Rate (FDR) thresholds.
- **DE Results - DNA Damage genes - DPI: 3 vs.CTRL  
  More Plot Information  Download CSV Data**

  | Probe Label | Log2 fold change | std error (log2) | Lower confidence limit (log2) | Upper confidence limit (log2) | Linear fold change | Lower confidence limit (linear) | Upper confidence limit (linear) | P-value | BY.p.value | method | Gene.sets | Codeset.Name | Probe.Label | Analyte.Type | Is.Control | Control.Type | Probe.Annotation | KEGG.Pathways | Cell.Type | Official.Gene.Name | volcanocol | volcanopch | tstats.all |
  | --- | --- | --- | --- | --- | --- | --- | --- | --- | --- | --- | --- | --- | --- | --- | --- | --- | --- | --- | --- | --- | --- | --- | --- |
  | Cdkn1a-mRNA | 2.72 | 0.171 | 2.38 | 3.05 | 6.57 | 5.21 | 8.28 | 3.19e-11 | 3.76e-08 | lm.nb | Adaptive Immune Response, Cell Cycle, Cellular Stress, Cytokine Signaling, DNA Damage, Growth Factor Signaling | NS\_MM\_NEUROINFLAM\_V1.0 | Cdkn1a | mRNA | 0 |  | Growth Factor Signaling;DNA Damage;Cytokine Signaling;Cellular Stress;Cell Cycle;Adaptive Immune Response | mmu04012;mmu04066;mmu04068;mmu04110;mmu04115;mmu04151;mmu04630;mmu04921;mmu05160;mmu05161;mmu05166;mmu05169;mmu05200;mmu05202;mmu05203;mmu05205;mmu05206;mmu05214;mmu05215;mmu05218;mmu05219;mmu05220 |  | Cdkn1a | slateblue1 | 16 | 15.906432748538 |
  | Kdm4b-mRNA | 0.563 | 0.0584 | 0.449 | 0.678 | 1.48 | 1.36 | 1.6 | 4.54e-08 | 1e-05 | loglinear | DNA Damage, Epigenetic Regulation | NS\_MM\_NEUROINFLAM\_V1.0 | Kdm4b | mRNA | 0 |  | Epigenetic Regulation;DNA Damage |  |  | Kdm4b | slateblue1 | 16 | 9.64041095890411 |
  | Sesn1-mRNA | 0.75 | 0.0913 | 0.571 | 0.929 | 1.68 | 1.49 | 1.9 | 3.93e-07 | 4.96e-05 | loglinear | DNA Damage | NS\_MM\_NEUROINFLAM\_V1.0 | Sesn1 | mRNA | 0 |  | DNA Damage | mmu04115;mmu04211 |  | Sesn1 | slateblue1 | 16 | 8.21467688937568 |
  | Parp1-mRNA | -0.736 | 0.108 | -0.949 | -0.524 | 0.6 | 0.518 | 0.695 | 4.31e-06 | 0.000334 | loglinear | Apoptosis, Cellular Stress, DNA Damage, NF-kB | NS\_MM\_NEUROINFLAM\_V1.0 | Parp1 | mRNA | 0 |  | NF-kB;DNA Damage;Cellular Stress;Apoptosis | mmu03410;mmu04064 |  | Parp1 | slateblue1 | 16 | -6.81481481481481 |
  | Creb1-mRNA | -0.443 | 0.0668 | -0.574 | -0.312 | 0.735 | 0.672 | 0.805 | 5.69e-06 | 0.000418 | loglinear | Adaptive Immune Response, Carbohydrate Metabolism, DNA Damage, Growth Factor Signaling, Innate Immune Response, Neurons and Neurotransmission, Notch | NS\_MM\_NEUROINFLAM\_V1.0 | Creb1 | mRNA | 0 |  | Notch;Neurons and Neurotransmission;Innate Immune Response;Growth Factor Signaling;DNA Damage;Carbohydrate Metabolism;Adaptive Immune Response | mmu04022;mmu04024;mmu04151;mmu04152;mmu04211;mmu04261;mmu04380;mmu04612;mmu04668;mmu04713;mmu04725;mmu04728;mmu04911;mmu04915;mmu04916;mmu04922;mmu04924;mmu04925;mmu04931;mmu05016;mmu05030;mmu05031;mmu05034;mmu05152;mmu05161;mmu05166;mmu05203;mmu05215 |  | Creb1 | slateblue1 | 16 | -6.63173652694611 |
  | Smc1a-mRNA | -0.597 | 0.104 | -0.8 | -0.393 | 0.661 | 0.574 | 0.762 | 3.03e-05 | 0.00157 | lm.nb | Cell Cycle, DNA Damage | NS\_MM\_NEUROINFLAM\_V1.0 | Smc1a | mRNA | 0 |  | DNA Damage;Cell Cycle | mmu04110;mmu04114 |  | Smc1a | slateblue1 | 16 | -5.74038461538461 |
  | Cdc25a-mRNA | 0.548 | 0.12 | 0.313 | 0.782 | 1.46 | 1.24 | 1.72 | 0.000307 | 0.0108 | lm.nb | Cell Cycle, DNA Damage | NS\_MM\_NEUROINFLAM\_V1.0 | Cdc25a | mRNA | 0 |  | DNA Damage;Cell Cycle | mmu04110;mmu04914;mmu05206 |  | Cdc25a | slateblue2 | 16 | 4.56666666666667 |
  | Fen1-mRNA | 0.646 | 0.141 | 0.369 | 0.922 | 1.56 | 1.29 | 1.9 | 0.000309 | 0.0108 | loglinear | Cell Cycle, DNA Damage | NS\_MM\_NEUROINFLAM\_V1.0 | Fen1 | mRNA | 0 |  | DNA Damage;Cell Cycle | mmu03030;mmu03410;mmu03450 |  | Fen1 | slateblue2 | 16 | 4.58156028368794 |
  | Msh2-mRNA | -0.508 | 0.112 | -0.727 | -0.289 | 0.703 | 0.604 | 0.819 | 0.00033 | 0.0113 | loglinear | DNA Damage | NS\_MM\_NEUROINFLAM\_V1.0 | Msh2 | mRNA | 0 |  | DNA Damage | mmu03430;mmu05200;mmu05210 |  | Msh2 | slateblue2 | 16 | -4.53571428571429 |
  | Bcl2-mRNA | -0.943 | 0.227 | -1.39 | -0.498 | 0.52 | 0.382 | 0.708 | 0.000747 | 0.0225 | lm.nb | Apoptosis, Autophagy, Cellular Stress, Cytokine Signaling, DNA Damage, Growth Factor Signaling, Innate Immune Response, Microglia Function, NF-kB | NS\_MM\_NEUROINFLAM\_V1.0 | Bcl2 | mRNA | 0 |  | NF-kB;Microglia Function;Innate Immune Response;Growth Factor Signaling;DNA Damage;Cytokine Signaling;Cellular Stress;Autophagy;Apoptosis | mmu04064;mmu04066;mmu04071;mmu04141;mmu04151;mmu04210;mmu04261;mmu04510;mmu04630;mmu04722;mmu04725;mmu04933;mmu05014;mmu05145;mmu05152;mmu05161;mmu05169;mmu05200;mmu05206;mmu05210;mmu05215;mmu05222 |  | Bcl2 | slateblue2 | 16 | -4.15418502202643 |
  | Gadd45g-mRNA | 0.993 | 0.244 | 0.515 | 1.47 | 1.99 | 1.43 | 2.77 | 0.000882 | 0.0257 | lm.nb | Cell Cycle, DNA Damage, Growth Factor Signaling | NS\_MM\_NEUROINFLAM\_V1.0 | Gadd45g | mRNA | 0 |  | Growth Factor Signaling;DNA Damage;Cell Cycle | mmu04010;mmu04068;mmu04110;mmu04115 |  | Gadd45g | slateblue2 | 16 | 4.06967213114754 |
  | Rad9a-mRNA | -0.981 | 0.245 | -1.46 | -0.501 | 0.507 | 0.364 | 0.707 | 0.00101 | 0.0279 | lm.nb | Cell Cycle, DNA Damage | NS\_MM\_NEUROINFLAM\_V1.0 | Rad9a | mRNA | 0 |  | DNA Damage;Cell Cycle |  |  | Rad9a | slateblue2 | 16 | -4.00408163265306 |
  | Bid-mRNA | 0.854 | 0.216 | 0.431 | 1.28 | 1.81 | 1.35 | 2.42 | 0.00112 | 0.0305 | lm.nb | Apoptosis, DNA Damage, Innate Immune Response | NS\_MM\_NEUROINFLAM\_V1.0 | Bid | mRNA | 0 |  | Innate Immune Response;DNA Damage;Apoptosis | mmu04071;mmu04115;mmu04210;mmu04650;mmu04932;mmu05010;mmu05014;mmu05152;mmu05200;mmu05416 |  | Bid | slateblue2 | 16 | 3.9537037037037 |
  | Parp2-mRNA | -0.318 | 0.0827 | -0.481 | -0.156 | 0.802 | 0.717 | 0.897 | 0.00141 | 0.0353 | loglinear | DNA Damage | NS\_MM\_NEUROINFLAM\_V1.0 | Parp2 | mRNA | 0 |  | DNA Damage | mmu03410 |  | Parp2 | slateblue2 | 16 | -3.84522370012092 |
  | Sesn2-mRNA | 0.706 | 0.196 | 0.322 | 1.09 | 1.63 | 1.25 | 2.13 | 0.00238 | 0.055 | lm.nb | DNA Damage | NS\_MM\_NEUROINFLAM\_V1.0 | Sesn2 | mRNA | 0 |  | DNA Damage | mmu04115;mmu04211 |  | Sesn2 | slateblue3 | 16 | 3.60204081632653 |
  | Rad1-mRNA | -0.413 | 0.134 | -0.674 | -0.151 | 0.751 | 0.627 | 0.901 | 0.00704 | 0.139 | loglinear | Cell Cycle, DNA Damage | NS\_MM\_NEUROINFLAM\_V1.0 | Rad1 | mRNA | 0 |  | DNA Damage;Cell Cycle |  |  | Rad1 | slateblue4 | 16 | -3.08208955223881 |
  | Fancg-mRNA | -0.639 | 0.208 | -1.05 | -0.23 | 0.642 | 0.484 | 0.852 | 0.00739 | 0.144 | lm.nb | DNA Damage | NS\_MM\_NEUROINFLAM\_V1.0 | Fancg | mRNA | 0 |  | DNA Damage | mmu03460 |  | Fancg | slateblue4 | 16 | -3.07211538461539 |
  | Casp3-mRNA | -0.463 | 0.155 | -0.766 | -0.159 | 0.726 | 0.588 | 0.896 | 0.00872 | 0.162 | lm.nb | Apoptosis, Cellular Stress, DNA Damage, Growth Factor Signaling, Innate Immune Response, Matrix Remodeling | NS\_MM\_NEUROINFLAM\_V1.0 | Casp3 | mRNA | 0 |  | Matrix Remodeling;Innate Immune Response;Growth Factor Signaling;DNA Damage;Cellular Stress;Apoptosis | mmu04010;mmu04115;mmu04210;mmu04650;mmu04668;mmu04726;mmu04932;mmu04933;mmu05010;mmu05012;mmu05014;mmu05016;mmu05133;mmu05134;mmu05145;mmu05146;mmu05152;mmu05161;mmu05168;mmu05200;mmu05203;mmu05205;mmu05206;mmu05210;mmu05416 |  | Casp3 | slateblue4 | 16 | -2.98709677419355 |
  | Xrcc6-mRNA | -0.585 | 0.214 | -1 | -0.166 | 0.667 | 0.499 | 0.891 | 0.0146 | 0.249 | lm.nb | DNA Damage, Innate Immune Response | NS\_MM\_NEUROINFLAM\_V1.0 | Xrcc6 | mRNA | 0 |  | Innate Immune Response;DNA Damage | mmu03450 |  | Xrcc6 | slateblue4 | 16 | -2.73364485981308 |
  | Rad51c-mRNA | -0.675 | 0.249 | -1.16 | -0.187 | 0.626 | 0.446 | 0.878 | 0.0154 | 0.261 | loglinear | Cell Cycle, DNA Damage | NS\_MM\_NEUROINFLAM\_V1.0 | Rad51c | mRNA | 0 |  | DNA Damage;Cell Cycle | mmu03440;mmu03460 |  | Rad51c | slateblue4 | 16 | -2.71084337349398 |

  ##### DE Results - DNA Damage genes - DPI: 3 vs.CTRL

  Table displaying each sample's global significance scores and directed global significance scores as defined in the heatmaps above. The global significance score is calculated as the square root of the mean squared t-statistic for the genes in a gene set, with t-statistics coming from the linear regression underlying our differential expression analysis. The directed global significance score is calculated as the square root of the mean signed squared t-statistic for the genes in a gene set, with t-statistics coming from the linear regression underlying our differential expression analysis.

DPI:
- DPI: differential expression in 1 vs. baseline of CTRL
- DPI: differential expression in 2 vs. baseline of CTRL
- DPI: differential expression in 3 vs. baseline of CTRL

- **Volcano Plot: DPI: 1 vs.CTRL  
  More Plot Information**


  ##### Volcano Plot: DPI: 1 vs.CTRL

  Volcano plot displaying each gene's -log10(p-value) and log2 fold change for the selected covariate. Highly statistically significant genes fall at the top of the plot, and highly differentially expressed genes fall to either side. Genes within the selected gene set are highlighted in orange. Horizontal lines indicate various False Discovery Rate (FDR) thresholds.
- **DE Results - Epigenetic Regulation genes - DPI: 1 vs.CTRL  
  More Plot Information  Download CSV Data**

  | Probe Label | Log2 fold change | std error (log2) | Lower confidence limit (log2) | Upper confidence limit (log2) | Linear fold change | Lower confidence limit (linear) | Upper confidence limit (linear) | P-value | BY.p.value | method | Gene.sets | Codeset.Name | Probe.Label | Analyte.Type | Is.Control | Control.Type | Probe.Annotation | KEGG.Pathways | Cell.Type | Official.Gene.Name | volcanocol | volcanopch | tstats.all |
  | --- | --- | --- | --- | --- | --- | --- | --- | --- | --- | --- | --- | --- | --- | --- | --- | --- | --- | --- | --- | --- | --- | --- | --- |
  | Kmt2a-mRNA | -0.591 | 0.129 | -0.844 | -0.339 | 0.664 | 0.557 | 0.791 | 0.000303 | 0.0536 | lm.nb | Epigenetic Regulation | NS\_MM\_NEUROINFLAM\_V1.0 | Kmt2a | mRNA | 0 |  | Epigenetic Regulation | mmu00310;mmu05202 |  | Kmt2a | slateblue3 | 16 | -4.58139534883721 |
  | Brd4-mRNA | -0.197 | 0.0443 | -0.284 | -0.11 | 0.873 | 0.822 | 0.927 | 0.000412 | 0.0611 | loglinear | Epigenetic Regulation | NS\_MM\_NEUROINFLAM\_V1.0 | Brd4 | mRNA | 0 |  | Epigenetic Regulation |  |  | Brd4 | slateblue3 | 16 | -4.44695259593679 |
  | Setd2-mRNA | -0.41 | 0.0997 | -0.605 | -0.214 | 0.753 | 0.657 | 0.862 | 0.000818 | 0.0875 | lm.nb | Epigenetic Regulation | NS\_MM\_NEUROINFLAM\_V1.0 | Setd2 | mRNA | 0 |  | Epigenetic Regulation | mmu00310 |  | Setd2 | slateblue3 | 16 | -4.1123370110331 |
  | Mbd2-mRNA | -0.355 | 0.0877 | -0.527 | -0.183 | 0.782 | 0.694 | 0.881 | 0.000939 | 0.0956 | loglinear | Epigenetic Regulation | NS\_MM\_NEUROINFLAM\_V1.0 | Mbd2 | mRNA | 0 |  | Epigenetic Regulation |  |  | Mbd2 | slateblue3 | 16 | -4.0478905359179 |
  | Setd7-mRNA | -0.784 | 0.194 | -1.17 | -0.403 | 0.581 | 0.446 | 0.756 | 0.00096 | 0.0956 | lm.nb | Epigenetic Regulation | NS\_MM\_NEUROINFLAM\_V1.0 | Setd7 | mRNA | 0 |  | Epigenetic Regulation | mmu00310;mmu04068 |  | Setd7 | slateblue3 | 16 | -4.04123711340206 |
  | Brd2-mRNA | -0.122 | 0.0318 | -0.185 | -0.0598 | 0.919 | 0.88 | 0.959 | 0.00145 | 0.116 | loglinear | Epigenetic Regulation | NS\_MM\_NEUROINFLAM\_V1.0 | Brd2 | mRNA | 0 |  | Epigenetic Regulation |  |  | Brd2 | slateblue4 | 16 | -3.83647798742138 |
  | Kat2a-mRNA | -0.787 | 0.21 | -1.2 | -0.375 | 0.579 | 0.436 | 0.771 | 0.00177 | 0.129 | lm.nb | Epigenetic Regulation, Notch | NS\_MM\_NEUROINFLAM\_V1.0 | Kat2a | mRNA | 0 |  | Notch;Epigenetic Regulation | mmu04330;mmu04919;mmu05166;mmu05203 |  | Kat2a | slateblue4 | 16 | -3.74761904761905 |
  | Smarcd1-mRNA | -0.399 | 0.107 | -0.609 | -0.19 | 0.758 | 0.656 | 0.877 | 0.00178 | 0.129 | loglinear | Epigenetic Regulation | NS\_MM\_NEUROINFLAM\_V1.0 | Smarcd1 | mRNA | 0 |  | Epigenetic Regulation |  |  | Smarcd1 | slateblue4 | 16 | -3.72897196261682 |
  | Kdm4c-mRNA | 0.542 | 0.157 | 0.234 | 0.85 | 1.46 | 1.18 | 1.8 | 0.00331 | 0.18 | lm.nb | Epigenetic Regulation | NS\_MM\_NEUROINFLAM\_V1.0 | Kdm4c | mRNA | 0 |  | Epigenetic Regulation |  |  | Kdm4c | slateblue4 | 16 | 3.45222929936306 |
  | Suz12-mRNA | -0.439 | 0.13 | -0.693 | -0.185 | 0.738 | 0.619 | 0.88 | 0.00375 | 0.184 | lm.nb | Cellular Stress, Epigenetic Regulation | NS\_MM\_NEUROINFLAM\_V1.0 | Suz12 | mRNA | 0 |  | Epigenetic Regulation;Cellular Stress |  |  | Suz12 | slateblue4 | 16 | -3.37692307692308 |
  | Brd3-mRNA | -0.314 | 0.096 | -0.502 | -0.126 | 0.804 | 0.706 | 0.916 | 0.00479 | 0.206 | loglinear | Epigenetic Regulation | NS\_MM\_NEUROINFLAM\_V1.0 | Brd3 | mRNA | 0 |  | Epigenetic Regulation |  |  | Brd3 | slateblue4 | 16 | -3.27083333333333 |
  | Smarca5-mRNA | -0.143 | 0.0451 | -0.232 | -0.0549 | 0.905 | 0.852 | 0.963 | 0.00583 | 0.234 | loglinear | Cell Cycle, DNA Damage, Epigenetic Regulation | NS\_MM\_NEUROINFLAM\_V1.0 | Smarca5 | mRNA | 0 |  | Epigenetic Regulation;DNA Damage;Cell Cycle |  |  | Smarca5 | slateblue4 | 16 | -3.17073170731707 |
  | Kdm5a-mRNA | -0.375 | 0.119 | -0.609 | -0.141 | 0.771 | 0.656 | 0.907 | 0.00629 | 0.244 | loglinear | Epigenetic Regulation | NS\_MM\_NEUROINFLAM\_V1.0 | Kdm5a | mRNA | 0 |  | Epigenetic Regulation |  |  | Kdm5a | slateblue4 | 16 | -3.15126050420168 |
  | Kmt2c-mRNA | -0.534 | 0.171 | -0.869 | -0.2 | 0.691 | 0.548 | 0.871 | 0.00647 | 0.246 | lm.nb | Epigenetic Regulation | NS\_MM\_NEUROINFLAM\_V1.0 | Kmt2c | mRNA | 0 |  | Epigenetic Regulation | mmu00310 |  | Kmt2c | slateblue4 | 16 | -3.12280701754386 |
  | Tarbp2-mRNA | -0.853 | 0.274 | -1.39 | -0.316 | 0.554 | 0.381 | 0.803 | 0.00671 | 0.247 | lm.nb | Epigenetic Regulation | NS\_MM\_NEUROINFLAM\_V1.0 | Tarbp2 | mRNA | 0 |  | Epigenetic Regulation |  |  | Tarbp2 | slateblue4 | 16 | -3.11313868613139 |
  | Sall1-mRNA | -0.408 | 0.137 | -0.677 | -0.138 | 0.754 | 0.626 | 0.909 | 0.00908 | 0.286 | lm.nb | Epigenetic Regulation | NS\_MM\_NEUROINFLAM\_V1.0 | Sall1 | mRNA | 0 |  | Epigenetic Regulation |  |  | Sall1 | slateblue4 | 16 | -2.97810218978102 |
  | Setd1a-mRNA | -0.515 | 0.182 | -0.873 | -0.158 | 0.7 | 0.546 | 0.896 | 0.0122 | 0.355 | lm.nb | Epigenetic Regulation | NS\_MM\_NEUROINFLAM\_V1.0 | Setd1a | mRNA | 0 |  | Epigenetic Regulation | mmu00310 |  | Setd1a | slateblue4 | 16 | -2.82967032967033 |
  | Hdac6-mRNA | -0.503 | 0.18 | -0.856 | -0.151 | 0.705 | 0.552 | 0.901 | 0.0129 | 0.364 | lm.nb | Cellular Stress, Epigenetic Regulation, Notch | NS\_MM\_NEUROINFLAM\_V1.0 | Hdac6 | mRNA | 0 |  | Notch;Epigenetic Regulation;Cellular Stress | mmu05034;mmu05203 |  | Hdac6 | slateblue4 | 16 | -2.79444444444444 |
  | Eed-mRNA | -0.131 | 0.0478 | -0.225 | -0.0377 | 0.913 | 0.855 | 0.974 | 0.0143 | 0.385 | loglinear | Cellular Stress, Epigenetic Regulation | NS\_MM\_NEUROINFLAM\_V1.0 | Eed | mRNA | 0 |  | Epigenetic Regulation;Cellular Stress |  |  | Eed | slateblue4 | 16 | -2.74058577405858 |
  | Kdm4b-mRNA | 0.156 | 0.0584 | 0.0416 | 0.271 | 1.11 | 1.03 | 1.21 | 0.0167 | 0.418 | loglinear | DNA Damage, Epigenetic Regulation | NS\_MM\_NEUROINFLAM\_V1.0 | Kdm4b | mRNA | 0 |  | Epigenetic Regulation;DNA Damage |  |  | Kdm4b | slateblue4 | 16 | 2.67123287671233 |

  ##### DE Results - Epigenetic Regulation genes - DPI: 1 vs.CTRL

  Table displaying each sample's global significance scores and directed global significance scores as defined in the heatmaps above. The global significance score is calculated as the square root of the mean squared t-statistic for the genes in a gene set, with t-statistics coming from the linear regression underlying our differential expression analysis. The directed global significance score is calculated as the square root of the mean signed squared t-statistic for the genes in a gene set, with t-statistics coming from the linear regression underlying our differential expression analysis.

- **Volcano Plot: DPI: 2 vs.CTRL  
  More Plot Information**


  ##### Volcano Plot: DPI: 2 vs.CTRL

  Volcano plot displaying each gene's -log10(p-value) and log2 fold change for the selected covariate. Highly statistically significant genes fall at the top of the plot, and highly differentially expressed genes fall to either side. Genes within the selected gene set are highlighted in orange. Horizontal lines indicate various False Discovery Rate (FDR) thresholds.
- **DE Results - Epigenetic Regulation genes - DPI: 2 vs.CTRL  
  More Plot Information  Download CSV Data**

  | Probe Label | Log2 fold change | std error (log2) | Lower confidence limit (log2) | Upper confidence limit (log2) | Linear fold change | Lower confidence limit (linear) | Upper confidence limit (linear) | P-value | BY.p.value | method | Gene.sets | Codeset.Name | Probe.Label | Analyte.Type | Is.Control | Control.Type | Probe.Annotation | KEGG.Pathways | Cell.Type | Official.Gene.Name | volcanocol | volcanopch | tstats.all |
  | --- | --- | --- | --- | --- | --- | --- | --- | --- | --- | --- | --- | --- | --- | --- | --- | --- | --- | --- | --- | --- | --- | --- | --- |
  | Padi2-mRNA | -1.41 | 0.175 | -1.75 | -1.06 | 0.377 | 0.298 | 0.479 | 5.19e-07 | 0.000108 | lm.nb | Astrocyte Function, Epigenetic Regulation | NS\_MM\_NEUROINFLAM\_V1.0 | Padi2 | mRNA | 0 |  | Epigenetic Regulation;Astrocyte Function |  |  | Padi2 | slateblue1 | 16 | -8.05714285714286 |
  | Brd4-mRNA | -0.289 | 0.0443 | -0.376 | -0.202 | 0.818 | 0.77 | 0.869 | 6.94e-06 | 0.000793 | loglinear | Epigenetic Regulation | NS\_MM\_NEUROINFLAM\_V1.0 | Brd4 | mRNA | 0 |  | Epigenetic Regulation |  |  | Brd4 | slateblue1 | 16 | -6.52370203160271 |
  | Sall1-mRNA | -0.944 | 0.145 | -1.23 | -0.659 | 0.52 | 0.427 | 0.633 | 7.21e-06 | 0.000795 | lm.nb | Epigenetic Regulation | NS\_MM\_NEUROINFLAM\_V1.0 | Sall1 | mRNA | 0 |  | Epigenetic Regulation |  |  | Sall1 | slateblue1 | 16 | -6.51034482758621 |
  | Smarca5-mRNA | -0.281 | 0.0451 | -0.369 | -0.192 | 0.823 | 0.774 | 0.875 | 1.21e-05 | 0.0011 | loglinear | Cell Cycle, DNA Damage, Epigenetic Regulation | NS\_MM\_NEUROINFLAM\_V1.0 | Smarca5 | mRNA | 0 |  | Epigenetic Regulation;DNA Damage;Cell Cycle |  |  | Smarca5 | slateblue1 | 16 | -6.23059866962306 |
  | Kmt2a-mRNA | -0.808 | 0.13 | -1.06 | -0.554 | 0.571 | 0.479 | 0.681 | 1.21e-05 | 0.0011 | lm.nb | Epigenetic Regulation | NS\_MM\_NEUROINFLAM\_V1.0 | Kmt2a | mRNA | 0 |  | Epigenetic Regulation | mmu00310;mmu05202 |  | Kmt2a | slateblue1 | 16 | -6.21538461538462 |
  | Setd2-mRNA | -0.55 | 0.1 | -0.746 | -0.353 | 0.683 | 0.596 | 0.783 | 4.96e-05 | 0.0033 | lm.nb | Epigenetic Regulation | NS\_MM\_NEUROINFLAM\_V1.0 | Setd2 | mRNA | 0 |  | Epigenetic Regulation | mmu00310 |  | Setd2 | slateblue1 | 16 | -5.5 |
  | Kdm4b-mRNA | 0.314 | 0.0584 | 0.199 | 0.428 | 1.24 | 1.15 | 1.35 | 6.26e-05 | 0.00387 | loglinear | DNA Damage, Epigenetic Regulation | NS\_MM\_NEUROINFLAM\_V1.0 | Kdm4b | mRNA | 0 |  | Epigenetic Regulation;DNA Damage |  |  | Kdm4b | slateblue1 | 16 | 5.37671232876712 |
  | Dnmt3a-mRNA | -0.685 | 0.145 | -0.969 | -0.401 | 0.622 | 0.511 | 0.757 | 0.000226 | 0.00951 | lm.nb | Epigenetic Regulation | NS\_MM\_NEUROINFLAM\_V1.0 | Dnmt3a | mRNA | 0 |  | Epigenetic Regulation | mmu00270;mmu05206 |  | Dnmt3a | slateblue2 | 16 | -4.72413793103448 |
  | Suz12-mRNA | -0.578 | 0.13 | -0.832 | -0.323 | 0.67 | 0.562 | 0.799 | 0.000406 | 0.0148 | lm.nb | Cellular Stress, Epigenetic Regulation | NS\_MM\_NEUROINFLAM\_V1.0 | Suz12 | mRNA | 0 |  | Epigenetic Regulation;Cellular Stress |  |  | Suz12 | slateblue2 | 16 | -4.44615384615385 |
  | Brd3-mRNA | -0.423 | 0.096 | -0.611 | -0.235 | 0.746 | 0.655 | 0.85 | 0.000443 | 0.0156 | loglinear | Epigenetic Regulation | NS\_MM\_NEUROINFLAM\_V1.0 | Brd3 | mRNA | 0 |  | Epigenetic Regulation |  |  | Brd3 | slateblue2 | 16 | -4.40625 |
  | Mbd2-mRNA | -0.371 | 0.0877 | -0.543 | -0.199 | 0.773 | 0.686 | 0.871 | 0.000633 | 0.0211 | loglinear | Epigenetic Regulation | NS\_MM\_NEUROINFLAM\_V1.0 | Mbd2 | mRNA | 0 |  | Epigenetic Regulation |  |  | Mbd2 | slateblue2 | 16 | -4.230330672748 |
  | Kat2a-mRNA | -0.885 | 0.211 | -1.3 | -0.472 | 0.542 | 0.407 | 0.721 | 0.000674 | 0.0218 | lm.nb | Epigenetic Regulation, Notch | NS\_MM\_NEUROINFLAM\_V1.0 | Kat2a | mRNA | 0 |  | Notch;Epigenetic Regulation | mmu04330;mmu04919;mmu05166;mmu05203 |  | Kat2a | slateblue2 | 16 | -4.19431279620853 |
  | Kmt2c-mRNA | -0.718 | 0.172 | -1.06 | -0.381 | 0.608 | 0.481 | 0.768 | 0.000716 | 0.022 | lm.nb | Epigenetic Regulation | NS\_MM\_NEUROINFLAM\_V1.0 | Kmt2c | mRNA | 0 |  | Epigenetic Regulation | mmu00310 |  | Kmt2c | slateblue2 | 16 | -4.17441860465116 |
  | Ehmt2-mRNA | -0.211 | 0.0508 | -0.311 | -0.112 | 0.864 | 0.806 | 0.925 | 0.000732 | 0.0223 | loglinear | Cellular Stress, Epigenetic Regulation | NS\_MM\_NEUROINFLAM\_V1.0 | Ehmt2 | mRNA | 0 |  | Epigenetic Regulation;Cellular Stress | mmu00310;mmu04211 |  | Ehmt2 | slateblue2 | 16 | -4.15354330708661 |
  | Ash2l-mRNA | -0.451 | 0.109 | -0.665 | -0.237 | 0.731 | 0.631 | 0.848 | 0.000784 | 0.0233 | loglinear | Epigenetic Regulation, Wnt | NS\_MM\_NEUROINFLAM\_V1.0 | Ash2l | mRNA | 0 |  | Wnt;Epigenetic Regulation |  |  | Ash2l | slateblue2 | 16 | -4.13761467889908 |
  | Eed-mRNA | -0.19 | 0.0478 | -0.284 | -0.0967 | 0.876 | 0.821 | 0.935 | 0.00107 | 0.0287 | loglinear | Cellular Stress, Epigenetic Regulation | NS\_MM\_NEUROINFLAM\_V1.0 | Eed | mRNA | 0 |  | Epigenetic Regulation;Cellular Stress |  |  | Eed | slateblue2 | 16 | -3.97489539748954 |
  | Smarcd1-mRNA | -0.421 | 0.107 | -0.631 | -0.212 | 0.747 | 0.646 | 0.863 | 0.00116 | 0.0296 | loglinear | Epigenetic Regulation | NS\_MM\_NEUROINFLAM\_V1.0 | Smarcd1 | mRNA | 0 |  | Epigenetic Regulation |  |  | Smarcd1 | slateblue2 | 16 | -3.93457943925234 |
  | Brd2-mRNA | -0.124 | 0.0318 | -0.186 | -0.0616 | 0.918 | 0.879 | 0.958 | 0.00128 | 0.0321 | loglinear | Epigenetic Regulation | NS\_MM\_NEUROINFLAM\_V1.0 | Brd2 | mRNA | 0 |  | Epigenetic Regulation |  |  | Brd2 | slateblue2 | 16 | -3.89937106918239 |
  | Suv39h2-mRNA | -0.801 | 0.207 | -1.21 | -0.394 | 0.574 | 0.433 | 0.761 | 0.00138 | 0.034 | lm.nb | Epigenetic Regulation | NS\_MM\_NEUROINFLAM\_V1.0 | Suv39h2 | mRNA | 0 |  | Epigenetic Regulation | mmu00310 |  | Suv39h2 | slateblue2 | 16 | -3.8695652173913 |
  | H2afx-mRNA | -0.214 | 0.0562 | -0.325 | -0.104 | 0.862 | 0.799 | 0.93 | 0.00154 | 0.0372 | loglinear | Cell Cycle, Cellular Stress, DNA Damage, Epigenetic Regulation, Wnt | NS\_MM\_NEUROINFLAM\_V1.0 | H2afx | mRNA | 0 |  | Wnt;Epigenetic Regulation;DNA Damage;Cellular Stress;Cell Cycle | mmu05034;mmu05322 |  | H2afx | slateblue2 | 16 | -3.80782918149466 |

  ##### DE Results - Epigenetic Regulation genes - DPI: 2 vs.CTRL

  Table displaying each sample's global significance scores and directed global significance scores as defined in the heatmaps above. The global significance score is calculated as the square root of the mean squared t-statistic for the genes in a gene set, with t-statistics coming from the linear regression underlying our differential expression analysis. The directed global significance score is calculated as the square root of the mean signed squared t-statistic for the genes in a gene set, with t-statistics coming from the linear regression underlying our differential expression analysis.

- **Volcano Plot: DPI: 3 vs.CTRL  
  More Plot Information**


  ##### Volcano Plot: DPI: 3 vs.CTRL

  Volcano plot displaying each gene's -log10(p-value) and log2 fold change for the selected covariate. Highly statistically significant genes fall at the top of the plot, and highly differentially expressed genes fall to either side. Genes within the selected gene set are highlighted in orange. Horizontal lines indicate various False Discovery Rate (FDR) thresholds.
- **DE Results - Epigenetic Regulation genes - DPI: 3 vs.CTRL  
  More Plot Information  Download CSV Data**

  | Probe Label | Log2 fold change | std error (log2) | Lower confidence limit (log2) | Upper confidence limit (log2) | Linear fold change | Lower confidence limit (linear) | Upper confidence limit (linear) | P-value | BY.p.value | method | Gene.sets | Codeset.Name | Probe.Label | Analyte.Type | Is.Control | Control.Type | Probe.Annotation | KEGG.Pathways | Cell.Type | Official.Gene.Name | volcanocol | volcanopch | tstats.all |
  | --- | --- | --- | --- | --- | --- | --- | --- | --- | --- | --- | --- | --- | --- | --- | --- | --- | --- | --- | --- | --- | --- | --- | --- |
  | Kdm5c-mRNA | -0.546 | 0.0494 | -0.643 | -0.449 | 0.685 | 0.64 | 0.732 | 6.61e-09 | 2.92e-06 | loglinear | Epigenetic Regulation | NS\_MM\_NEUROINFLAM\_V1.0 | Kdm5c | mRNA | 0 |  | Epigenetic Regulation |  |  | Kdm5c | slateblue1 | 16 | -11.0526315789474 |
  | Sall1-mRNA | -1.75 | 0.162 | -2.06 | -1.43 | 0.298 | 0.239 | 0.371 | 9.43e-09 | 3.12e-06 | lm.nb | Epigenetic Regulation | NS\_MM\_NEUROINFLAM\_V1.0 | Sall1 | mRNA | 0 |  | Epigenetic Regulation |  |  | Sall1 | slateblue1 | 16 | -10.8024691358025 |
  | Smarca4-mRNA | -0.495 | 0.046 | -0.585 | -0.405 | 0.71 | 0.667 | 0.755 | 9.76e-09 | 3.12e-06 | loglinear | Epigenetic Regulation, Wnt | NS\_MM\_NEUROINFLAM\_V1.0 | Smarca4 | mRNA | 0 |  | Wnt;Epigenetic Regulation |  |  | Smarca4 | slateblue1 | 16 | -10.7608695652174 |
  | Kdm4b-mRNA | 0.563 | 0.0584 | 0.449 | 0.678 | 1.48 | 1.36 | 1.6 | 4.54e-08 | 1e-05 | loglinear | DNA Damage, Epigenetic Regulation | NS\_MM\_NEUROINFLAM\_V1.0 | Kdm4b | mRNA | 0 |  | Epigenetic Regulation;DNA Damage |  |  | Kdm4b | slateblue1 | 16 | 9.64041095890411 |
  | Eif1-mRNA | 0.37 | 0.0414 | 0.289 | 0.451 | 1.29 | 1.22 | 1.37 | 1.3e-07 | 2.18e-05 | loglinear | Epigenetic Regulation | NS\_MM\_NEUROINFLAM\_V1.0 | Eif1 | mRNA | 0 |  | Epigenetic Regulation |  |  | Eif1 | slateblue1 | 16 | 8.93719806763285 |
  | Padi2-mRNA | -1.5 | 0.177 | -1.85 | -1.15 | 0.353 | 0.278 | 0.449 | 2.54e-07 | 3.57e-05 | lm.nb | Astrocyte Function, Epigenetic Regulation | NS\_MM\_NEUROINFLAM\_V1.0 | Padi2 | mRNA | 0 |  | Epigenetic Regulation;Astrocyte Function |  |  | Padi2 | slateblue1 | 16 | -8.47457627118644 |
  | Hdac2-mRNA | -0.452 | 0.057 | -0.564 | -0.341 | 0.731 | 0.676 | 0.79 | 6.19e-07 | 7.29e-05 | loglinear | Cell Cycle, Cellular Stress, Epigenetic Regulation, Growth Factor Signaling, Notch | NS\_MM\_NEUROINFLAM\_V1.0 | Hdac2 | mRNA | 0 |  | Notch;Growth Factor Signaling;Epigenetic Regulation;Cellular Stress;Cell Cycle | mmu04110;mmu04213;mmu04330;mmu04919;mmu05016;mmu05034;mmu05169;mmu05200;mmu05202;mmu05203;mmu05220 |  | Hdac2 | slateblue1 | 16 | -7.92982456140351 |
  | Ehmt2-mRNA | -0.329 | 0.0508 | -0.429 | -0.23 | 0.796 | 0.743 | 0.853 | 7.46e-06 | 0.000527 | loglinear | Cellular Stress, Epigenetic Regulation | NS\_MM\_NEUROINFLAM\_V1.0 | Ehmt2 | mRNA | 0 |  | Epigenetic Regulation;Cellular Stress | mmu00310;mmu04211 |  | Ehmt2 | slateblue1 | 16 | -6.47637795275591 |
  | Ash2l-mRNA | -0.668 | 0.109 | -0.882 | -0.454 | 0.629 | 0.542 | 0.73 | 1.48e-05 | 0.000921 | loglinear | Epigenetic Regulation, Wnt | NS\_MM\_NEUROINFLAM\_V1.0 | Ash2l | mRNA | 0 |  | Wnt;Epigenetic Regulation |  |  | Ash2l | slateblue1 | 16 | -6.12844036697248 |
  | Kdm2a-mRNA | -0.531 | 0.0952 | -0.718 | -0.345 | 0.692 | 0.608 | 0.788 | 4.14e-05 | 0.002 | loglinear | Epigenetic Regulation | NS\_MM\_NEUROINFLAM\_V1.0 | Kdm2a | mRNA | 0 |  | Epigenetic Regulation |  |  | Kdm2a | slateblue1 | 16 | -5.57773109243697 |
  | Brd2-mRNA | 0.177 | 0.0318 | 0.115 | 0.24 | 1.13 | 1.08 | 1.18 | 4.25e-05 | 0.00203 | loglinear | Epigenetic Regulation | NS\_MM\_NEUROINFLAM\_V1.0 | Brd2 | mRNA | 0 |  | Epigenetic Regulation |  |  | Brd2 | slateblue1 | 16 | 5.56603773584906 |
  | Ago4-mRNA | -2.72 | 0.514 | -3.72 | -1.71 | 0.152 | 0.0757 | 0.306 | 7.46e-05 | 0.00321 | lm.nb | Adaptive Immune Response, Cellular Stress, Epigenetic Regulation, Growth Factor Signaling, Notch, Wnt | NS\_MM\_NEUROINFLAM\_V1.0 | Ago4 | mRNA | 0 |  | Wnt;Notch;Growth Factor Signaling;Epigenetic Regulation;Cellular Stress;Adaptive Immune Response |  |  | Ago4 | slateblue1 | 16 | -5.29182879377432 |
  | Setd7-mRNA | -1.01 | 0.195 | -1.39 | -0.625 | 0.498 | 0.382 | 0.649 | 9.37e-05 | 0.00389 | lm.nb | Epigenetic Regulation | NS\_MM\_NEUROINFLAM\_V1.0 | Setd7 | mRNA | 0 |  | Epigenetic Regulation | mmu00310;mmu04068 |  | Setd7 | slateblue1 | 16 | -5.17948717948718 |
  | Eed-mRNA | -0.239 | 0.0478 | -0.333 | -0.145 | 0.847 | 0.794 | 0.904 | 0.000131 | 0.00525 | loglinear | Cellular Stress, Epigenetic Regulation | NS\_MM\_NEUROINFLAM\_V1.0 | Eed | mRNA | 0 |  | Epigenetic Regulation;Cellular Stress |  |  | Eed | slateblue1 | 16 | -5 |
  | Kdm1b-mRNA | -0.787 | 0.161 | -1.1 | -0.471 | 0.58 | 0.466 | 0.722 | 0.000167 | 0.00663 | lm.nb | Epigenetic Regulation | NS\_MM\_NEUROINFLAM\_V1.0 | Kdm1b | mRNA | 0 |  | Epigenetic Regulation |  |  | Kdm1b | slateblue1 | 16 | -4.88819875776398 |
  | Suv39h2-mRNA | -0.974 | 0.212 | -1.39 | -0.557 | 0.509 | 0.382 | 0.68 | 0.000306 | 0.0108 | lm.nb | Epigenetic Regulation | NS\_MM\_NEUROINFLAM\_V1.0 | Suv39h2 | mRNA | 0 |  | Epigenetic Regulation | mmu00310 |  | Suv39h2 | slateblue2 | 16 | -4.59433962264151 |
  | Kmt2c-mRNA | -0.71 | 0.172 | -1.05 | -0.373 | 0.611 | 0.484 | 0.772 | 0.000786 | 0.0231 | lm.nb | Epigenetic Regulation | NS\_MM\_NEUROINFLAM\_V1.0 | Kmt2c | mRNA | 0 |  | Epigenetic Regulation | mmu00310 |  | Kmt2c | slateblue2 | 16 | -4.12790697674419 |
  | Kmt2a-mRNA | -0.512 | 0.129 | -0.764 | -0.26 | 0.701 | 0.589 | 0.835 | 0.00106 | 0.0291 | lm.nb | Epigenetic Regulation | NS\_MM\_NEUROINFLAM\_V1.0 | Kmt2a | mRNA | 0 |  | Epigenetic Regulation | mmu00310;mmu05202 |  | Kmt2a | slateblue2 | 16 | -3.96899224806202 |
  | Suv39h1-mRNA | 0.278 | 0.0757 | 0.13 | 0.426 | 1.21 | 1.09 | 1.34 | 0.00205 | 0.0502 | loglinear | Epigenetic Regulation | NS\_MM\_NEUROINFLAM\_V1.0 | Suv39h1 | mRNA | 0 |  | Epigenetic Regulation | mmu00310 |  | Suv39h1 | slateblue3 | 16 | 3.67239101717305 |
  | Setdb1-mRNA | -0.796 | 0.22 | -1.23 | -0.365 | 0.576 | 0.427 | 0.776 | 0.00228 | 0.0539 | lm.nb | Epigenetic Regulation | NS\_MM\_NEUROINFLAM\_V1.0 | Setdb1 | mRNA | 0 |  | Epigenetic Regulation | mmu00310;mmu04550 |  | Setdb1 | slateblue3 | 16 | -3.61818181818182 |

  ##### DE Results - Epigenetic Regulation genes - DPI: 3 vs.CTRL

  Table displaying each sample's global significance scores and directed global significance scores as defined in the heatmaps above. The global significance score is calculated as the square root of the mean squared t-statistic for the genes in a gene set, with t-statistics coming from the linear regression underlying our differential expression analysis. The directed global significance score is calculated as the square root of the mean signed squared t-statistic for the genes in a gene set, with t-statistics coming from the linear regression underlying our differential expression analysis.

DPI:
- DPI: differential expression in 1 vs. baseline of CTRL
- DPI: differential expression in 2 vs. baseline of CTRL
- DPI: differential expression in 3 vs. baseline of CTRL

- **Volcano Plot: DPI: 1 vs.CTRL  
  More Plot Information**


  ##### Volcano Plot: DPI: 1 vs.CTRL

  Volcano plot displaying each gene's -log10(p-value) and log2 fold change for the selected covariate. Highly statistically significant genes fall at the top of the plot, and highly differentially expressed genes fall to either side. Genes within the selected gene set are highlighted in orange. Horizontal lines indicate various False Discovery Rate (FDR) thresholds.
- **DE Results - Growth Factor Signaling genes - DPI: 1 vs.CTRL  
  More Plot Information  Download CSV Data**

  | Probe Label | Log2 fold change | std error (log2) | Lower confidence limit (log2) | Upper confidence limit (log2) | Linear fold change | Lower confidence limit (linear) | Upper confidence limit (linear) | P-value | BY.p.value | method | Gene.sets | Codeset.Name | Probe.Label | Analyte.Type | Is.Control | Control.Type | Probe.Annotation | KEGG.Pathways | Cell.Type | Official.Gene.Name | volcanocol | volcanopch | tstats.all |
  | --- | --- | --- | --- | --- | --- | --- | --- | --- | --- | --- | --- | --- | --- | --- | --- | --- | --- | --- | --- | --- | --- | --- | --- |
  | Dusp7-mRNA | -0.378 | 0.0611 | -0.497 | -0.258 | 0.77 | 0.708 | 0.836 | 1.32e-05 | 0.0093 | loglinear | Adaptive Immune Response, Angiogenesis, Cytokine Signaling, Growth Factor Signaling, Innate Immune Response, Insulin Signaling, Microglia Function | NS\_MM\_NEUROINFLAM\_V1.0 | Dusp7 | mRNA | 0 |  | Microglia Function;Insulin Signaling;Innate Immune Response;Growth Factor Signaling;Cytokine Signaling;Angiogenesis;Adaptive Immune Response | mmu04010 |  | Dusp7 | slateblue2 | 16 | -6.18657937806874 |
  | Fos-mRNA | -1.75 | 0.299 | -2.34 | -1.17 | 0.297 | 0.198 | 0.446 | 2.43e-05 | 0.0143 | lm.nb | Adaptive Immune Response, Cellular Stress, Growth Factor Signaling, Innate Immune Response, Neurons and Neurotransmission | NS\_MM\_NEUROINFLAM\_V1.0 | Fos | mRNA | 0 |  | Neurons and Neurotransmission;Innate Immune Response;Growth Factor Signaling;Cellular Stress;Adaptive Immune Response | mmu04010;mmu04024;mmu04380;mmu04620;mmu04660;mmu04662;mmu04668;mmu04713;mmu04725;mmu04728;mmu04915;mmu04917;mmu04921;mmu05031;mmu05132;mmu05133;mmu05140;mmu05142;mmu05161;mmu05166;mmu05168;mmu05200;mmu05210;mmu05231;mmu05323 |  | Fos | slateblue2 | 16 | -5.85284280936455 |
  | Mapt-mRNA | -0.222 | 0.0425 | -0.306 | -0.139 | 0.857 | 0.809 | 0.908 | 8.22e-05 | 0.0321 | loglinear | Apoptosis, Cellular Stress, Growth Factor Signaling, Neurons and Neurotransmission | NS\_MM\_NEUROINFLAM\_V1.0 | Mapt | mRNA | 0 |  | Neurons and Neurotransmission;Growth Factor Signaling;Cellular Stress;Apoptosis | mmu04010;mmu05010 |  | Mapt | slateblue2 | 16 | -5.22352941176471 |
  | Prkacb-mRNA | -0.327 | 0.0629 | -0.451 | -0.204 | 0.797 | 0.732 | 0.868 | 8.65e-05 | 0.0321 | loglinear | Adaptive Immune Response, Angiogenesis, Apoptosis, Autophagy, Growth Factor Signaling, Lipid Metabolism, Neurons and Neurotransmission, Wnt | NS\_MM\_NEUROINFLAM\_V1.0 | Prkacb | mRNA | 0 |  | Wnt;Neurons and Neurotransmission;Lipid Metabolism;Growth Factor Signaling;Autophagy;Apoptosis;Angiogenesis;Adaptive Immune Response | mmu04010;mmu04014;mmu04020;mmu04024;mmu04062;mmu04114;mmu04210;mmu04211;mmu04213;mmu04261;mmu04270;mmu04310;mmu04540;mmu04611;mmu04713;mmu04720;mmu04723;mmu04724;mmu04725;mmu04726;mmu04728;mmu04750;mmu04910;mmu04911;mmu04912;mmu04913;mmu04914;mmu04915;mmu04916;mmu04919;mmu04921;mmu04922;mmu04923;mmu04924;mmu04925;mmu05012;mmu05020;mmu05030;mmu05031;mmu05146;mmu05166;mmu05169;mmu05200;mmu05203;mmu05205;mmu05414 |  | Prkacb | slateblue2 | 16 | -5.19872813990461 |
  | Bdnf-mRNA | -0.736 | 0.143 | -1.02 | -0.456 | 0.6 | 0.494 | 0.729 | 9.57e-05 | 0.0321 | lm.nb | Growth Factor Signaling, Neurons and Neurotransmission | NS\_MM\_NEUROINFLAM\_V1.0 | Bdnf | mRNA | 0 |  | Neurons and Neurotransmission;Growth Factor Signaling | mmu04010;mmu04024;mmu04722;mmu05016;mmu05030;mmu05034 |  | Bdnf | slateblue2 | 16 | -5.14685314685315 |
  | Pak1-mRNA | -0.284 | 0.0554 | -0.393 | -0.176 | 0.821 | 0.762 | 0.885 | 1e-04 | 0.0321 | loglinear | Adaptive Immune Response, Angiogenesis, Growth Factor Signaling, Innate Immune Response, Microglia Function | NS\_MM\_NEUROINFLAM\_V1.0 | Pak1 | mRNA | 0 |  | Microglia Function;Innate Immune Response;Growth Factor Signaling;Angiogenesis;Adaptive Immune Response | mmu04010;mmu04012;mmu04014;mmu04024;mmu04062;mmu04360;mmu04510;mmu04650;mmu04660;mmu04666;mmu04810;mmu05205;mmu05211 |  | Pak1 | slateblue2 | 16 | -5.12635379061372 |
  | Rgl1-mRNA | -0.734 | 0.146 | -1.02 | -0.448 | 0.601 | 0.493 | 0.733 | 0.000121 | 0.0357 | lm.nb | Growth Factor Signaling, Microglia Function | NS\_MM\_NEUROINFLAM\_V1.0 | Rgl1 | mRNA | 0 |  | Microglia Function;Growth Factor Signaling | mmu04014 |  | Rgl1 | slateblue2 | 16 | -5.02739726027397 |
  | Mdm2-mRNA | -0.317 | 0.0642 | -0.443 | -0.191 | 0.803 | 0.736 | 0.876 | 0.00015 | 0.0406 | loglinear | Adaptive Immune Response, Apoptosis, Cell Cycle, Cellular Stress, DNA Damage, Growth Factor Signaling, Neurons and Neurotransmission | NS\_MM\_NEUROINFLAM\_V1.0 | Mdm2 | mRNA | 0 |  | Neurons and Neurotransmission;Growth Factor Signaling;DNA Damage;Cellular Stress;Cell Cycle;Apoptosis;Adaptive Immune Response | mmu04068;mmu04110;mmu04115;mmu04120;mmu04144;mmu04151;mmu04919;mmu05169;mmu05200;mmu05202;mmu05203;mmu05205;mmu05206;mmu05214;mmu05215;mmu05218;mmu05219;mmu05220 |  | Mdm2 | slateblue2 | 16 | -4.93769470404984 |
  | Ppp3r1-mRNA | -0.28 | 0.0577 | -0.393 | -0.167 | 0.823 | 0.761 | 0.891 | 0.000175 | 0.0442 | loglinear | Adaptive Immune Response, Apoptosis, Growth Factor Signaling, Innate Immune Response, Wnt | NS\_MM\_NEUROINFLAM\_V1.0 | Ppp3r1 | mRNA | 0 |  | Wnt;Innate Immune Response;Growth Factor Signaling;Apoptosis;Adaptive Immune Response | mmu04010;mmu04020;mmu04022;mmu04114;mmu04210;mmu04310;mmu04360;mmu04370;mmu04380;mmu04650;mmu04660;mmu04662;mmu04720;mmu04724;mmu04921;mmu04922;mmu04924;mmu05010;mmu05014;mmu05031;mmu05152;mmu05166 |  | Ppp3r1 | slateblue2 | 16 | -4.8526863084922 |
  | Csf1r-mRNA | -0.687 | 0.144 | -0.969 | -0.405 | 0.621 | 0.511 | 0.755 | 0.000208 | 0.0464 | lm.nb | Cytokine Signaling, Growth Factor Signaling, Microglia Function | NS\_MM\_NEUROINFLAM\_V1.0 | Csf1r | mRNA | 0 |  | Microglia Function;Growth Factor Signaling;Cytokine Signaling | mmu04014;mmu04015;mmu04060;mmu04144;mmu04151;mmu04380;mmu04640;mmu05200;mmu05202 |  | Csf1r | slateblue2 | 16 | -4.77083333333333 |
  | Prkar2b-mRNA | -0.5 | 0.113 | -0.722 | -0.278 | 0.707 | 0.606 | 0.825 | 0.000436 | 0.0616 | loglinear | Apoptosis, Cell Cycle, Growth Factor Signaling | NS\_MM\_NEUROINFLAM\_V1.0 | Prkar2b | mRNA | 0 |  | Growth Factor Signaling;Cell Cycle;Apoptosis | mmu04210;mmu04910 |  | Prkar2b | slateblue3 | 16 | -4.42477876106195 |
  | Prkce-mRNA | -0.427 | 0.0999 | -0.623 | -0.232 | 0.744 | 0.649 | 0.852 | 0.000577 | 0.0754 | loglinear | Autophagy, Growth Factor Signaling | NS\_MM\_NEUROINFLAM\_V1.0 | Prkce | mRNA | 0 |  | Growth Factor Signaling;Autophagy | mmu04022;mmu04071;mmu04270;mmu04530;mmu04666;mmu04750;mmu04925;mmu04930;mmu04931;mmu04933;mmu05206 |  | Prkce | slateblue3 | 16 | -4.27427427427427 |
  | Ppp3cb-mRNA | -0.411 | 0.0979 | -0.602 | -0.219 | 0.752 | 0.659 | 0.859 | 0.000686 | 0.0806 | lm.nb | Adaptive Immune Response, Apoptosis, Growth Factor Signaling, Innate Immune Response, Wnt | NS\_MM\_NEUROINFLAM\_V1.0 | Ppp3cb | mRNA | 0 |  | Wnt;Innate Immune Response;Growth Factor Signaling;Apoptosis;Adaptive Immune Response | mmu04010;mmu04020;mmu04022;mmu04114;mmu04210;mmu04310;mmu04360;mmu04370;mmu04380;mmu04650;mmu04660;mmu04662;mmu04720;mmu04724;mmu04728;mmu04921;mmu04922;mmu04924;mmu05010;mmu05014;mmu05031;mmu05152;mmu05166 |  | Ppp3cb | slateblue3 | 16 | -4.19816138917262 |
  | Braf-mRNA | -0.641 | 0.154 | -0.943 | -0.339 | 0.641 | 0.52 | 0.791 | 0.000739 | 0.0815 | lm.nb | Adaptive Immune Response, Angiogenesis, Apoptosis, Cytokine Signaling, Growth Factor Signaling, Innate Immune Response, Insulin Signaling, Neurons and Neurotransmission | NS\_MM\_NEUROINFLAM\_V1.0 | Braf | mRNA | 0 |  | Neurons and Neurotransmission;Insulin Signaling;Innate Immune Response;Growth Factor Signaling;Cytokine Signaling;Apoptosis;Angiogenesis;Adaptive Immune Response | mmu04010;mmu04012;mmu04015;mmu04024;mmu04062;mmu04068;mmu04150;mmu04270;mmu04510;mmu04650;mmu04720;mmu04722;mmu04726;mmu04730;mmu04810;mmu04910;mmu04914;mmu05034;mmu05160;mmu05200;mmu05205;mmu05210;mmu05211;mmu05212;mmu05213;mmu05214;mmu05215;mmu05216;mmu05218;mmu05219;mmu05220;mmu05221;mmu05223 |  | Braf | slateblue3 | 16 | -4.16233766233766 |
  | Fgf13-mRNA | -0.545 | 0.135 | -0.81 | -0.28 | 0.685 | 0.57 | 0.824 | 0.000975 | 0.0956 | lm.nb | Growth Factor Signaling, Microglia Function | NS\_MM\_NEUROINFLAM\_V1.0 | Fgf13 | mRNA | 0 |  | Microglia Function;Growth Factor Signaling | mmu04010;mmu04014;mmu04015;mmu04151;mmu04810;mmu05200;mmu05218 |  | Fgf13 | slateblue4 | 16 | -4.03703703703704 |
  | Pten-mRNA | -0.296 | 0.0753 | -0.444 | -0.148 | 0.815 | 0.735 | 0.902 | 0.0012 | 0.109 | loglinear | Adaptive Immune Response, Autophagy, DNA Damage, Growth Factor Signaling, Lipid Metabolism | NS\_MM\_NEUROINFLAM\_V1.0 | Pten | mRNA | 0 |  | Lipid Metabolism;Growth Factor Signaling;DNA Damage;Autophagy;Adaptive Immune Response | mmu00562;mmu04068;mmu04070;mmu04071;mmu04115;mmu04150;mmu04151;mmu04510;mmu04530;mmu04931;mmu05161;mmu05200;mmu05206;mmu05213;mmu05214;mmu05215;mmu05218;mmu05222;mmu05230 |  | Pten | slateblue4 | 16 | -3.93094289508632 |
  | Mapk10-mRNA | -0.539 | 0.138 | -0.81 | -0.269 | 0.688 | 0.57 | 0.83 | 0.00126 | 0.111 | lm.nb | Adaptive Immune Response, Apoptosis, Autophagy, Cellular Stress, Growth Factor Signaling, Innate Immune Response, Wnt | NS\_MM\_NEUROINFLAM\_V1.0 | Mapk10 | mRNA | 0 |  | Wnt;Innate Immune Response;Growth Factor Signaling;Cellular Stress;Autophagy;Apoptosis;Adaptive Immune Response | mmu04010;mmu04012;mmu04014;mmu04024;mmu04068;mmu04071;mmu04141;mmu04310;mmu04380;mmu04510;mmu04620;mmu04621;mmu04622;mmu04664;mmu04668;mmu04722;mmu04723;mmu04728;mmu04750;mmu04910;mmu04912;mmu04914;mmu04917;mmu04920;mmu04930;mmu04931;mmu04932;mmu04933;mmu05132;mmu05133;mmu05142;mmu05145;mmu05152;mmu05160;mmu05161;mmu05164;mmu05168;mmu05169;mmu05200;mmu05210;mmu05212;mmu05231 |  | Mapk10 | slateblue4 | 16 | -3.90579710144928 |
  | Camk4-mRNA | -0.766 | 0.197 | -1.15 | -0.379 | 0.588 | 0.45 | 0.769 | 0.00131 | 0.113 | lm.nb | Growth Factor Signaling, Neurons and Neurotransmission | NS\_MM\_NEUROINFLAM\_V1.0 | Camk4 | mRNA | 0 |  | Neurons and Neurotransmission;Growth Factor Signaling | mmu04020;mmu04024;mmu04211;mmu04380;mmu04720;mmu04722;mmu04725;mmu04921;mmu04925;mmu05031;mmu05034 |  | Camk4 | slateblue4 | 16 | -3.88832487309645 |
  | Prkar1a-mRNA | -0.329 | 0.0856 | -0.497 | -0.162 | 0.796 | 0.709 | 0.894 | 0.00141 | 0.116 | loglinear | Apoptosis, Growth Factor Signaling | NS\_MM\_NEUROINFLAM\_V1.0 | Prkar1a | mRNA | 0 |  | Growth Factor Signaling;Apoptosis | mmu04210;mmu04910 |  | Prkar1a | slateblue4 | 16 | -3.84345794392523 |
  | Gadd45a-mRNA | -0.491 | 0.13 | -0.746 | -0.237 | 0.711 | 0.596 | 0.849 | 0.00162 | 0.127 | lm.nb | Cell Cycle, DNA Damage, Growth Factor Signaling | NS\_MM\_NEUROINFLAM\_V1.0 | Gadd45a | mRNA | 0 |  | Growth Factor Signaling;DNA Damage;Cell Cycle | mmu04010;mmu04068;mmu04110;mmu04115 |  | Gadd45a | slateblue4 | 16 | -3.77692307692308 |

  ##### DE Results - Growth Factor Signaling genes - DPI: 1 vs.CTRL

  Table displaying each sample's global significance scores and directed global significance scores as defined in the heatmaps above. The global significance score is calculated as the square root of the mean squared t-statistic for the genes in a gene set, with t-statistics coming from the linear regression underlying our differential expression analysis. The directed global significance score is calculated as the square root of the mean signed squared t-statistic for the genes in a gene set, with t-statistics coming from the linear regression underlying our differential expression analysis.

- **Volcano Plot: DPI: 2 vs.CTRL  
  More Plot Information**


  ##### Volcano Plot: DPI: 2 vs.CTRL

  Volcano plot displaying each gene's -log10(p-value) and log2 fold change for the selected covariate. Highly statistically significant genes fall at the top of the plot, and highly differentially expressed genes fall to either side. Genes within the selected gene set are highlighted in orange. Horizontal lines indicate various False Discovery Rate (FDR) thresholds.
- **DE Results - Growth Factor Signaling genes - DPI: 2 vs.CTRL  
  More Plot Information  Download CSV Data**

  | Probe Label | Log2 fold change | std error (log2) | Lower confidence limit (log2) | Upper confidence limit (log2) | Linear fold change | Lower confidence limit (linear) | Upper confidence limit (linear) | P-value | BY.p.value | method | Gene.sets | Codeset.Name | Probe.Label | Analyte.Type | Is.Control | Control.Type | Probe.Annotation | KEGG.Pathways | Cell.Type | Official.Gene.Name | volcanocol | volcanopch | tstats.all |
  | --- | --- | --- | --- | --- | --- | --- | --- | --- | --- | --- | --- | --- | --- | --- | --- | --- | --- | --- | --- | --- | --- | --- | --- |
  | Cdkn1a-mRNA | 2.13 | 0.171 | 1.79 | 2.46 | 4.37 | 3.47 | 5.52 | 1.24e-09 | 8.78e-07 | lm.nb | Adaptive Immune Response, Cell Cycle, Cellular Stress, Cytokine Signaling, DNA Damage, Growth Factor Signaling | NS\_MM\_NEUROINFLAM\_V1.0 | Cdkn1a | mRNA | 0 |  | Growth Factor Signaling;DNA Damage;Cytokine Signaling;Cellular Stress;Cell Cycle;Adaptive Immune Response | mmu04012;mmu04066;mmu04068;mmu04110;mmu04115;mmu04151;mmu04630;mmu04921;mmu05160;mmu05161;mmu05166;mmu05169;mmu05200;mmu05202;mmu05203;mmu05205;mmu05206;mmu05214;mmu05215;mmu05218;mmu05219;mmu05220 |  | Cdkn1a | slateblue1 | 16 | 12.4561403508772 |
  | Bdnf-mRNA | -1.64 | 0.158 | -1.94 | -1.33 | 0.322 | 0.26 | 0.399 | 1.66e-08 | 8.35e-06 | lm.nb | Growth Factor Signaling, Neurons and Neurotransmission | NS\_MM\_NEUROINFLAM\_V1.0 | Bdnf | mRNA | 0 |  | Neurons and Neurotransmission;Growth Factor Signaling | mmu04010;mmu04024;mmu04722;mmu05016;mmu05030;mmu05034 |  | Bdnf | slateblue1 | 16 | -10.379746835443 |
  | Prkar2b-mRNA | -1.01 | 0.113 | -1.23 | -0.787 | 0.497 | 0.426 | 0.579 | 1.33e-07 | 3.91e-05 | loglinear | Apoptosis, Cell Cycle, Growth Factor Signaling | NS\_MM\_NEUROINFLAM\_V1.0 | Prkar2b | mRNA | 0 |  | Growth Factor Signaling;Cell Cycle;Apoptosis | mmu04210;mmu04910 |  | Prkar2b | slateblue1 | 16 | -8.93805309734513 |
  | Nfkbia-mRNA | 1.86 | 0.228 | 1.41 | 2.31 | 3.63 | 2.67 | 4.95 | 4.26e-07 | 9.99e-05 | lm.nb | Adaptive Immune Response, Apoptosis, Growth Factor Signaling, Inflammatory Signaling, Innate Immune Response, NF-kB | NS\_MM\_NEUROINFLAM\_V1.0 | Nfkbia | mRNA | 0 |  | NF-kB;Innate Immune Response;Inflammatory Signaling;Growth Factor Signaling;Apoptosis;Adaptive Immune Response | mmu04024;mmu04062;mmu04064;mmu04210;mmu04380;mmu04620;mmu04621;mmu04622;mmu04623;mmu04660;mmu04662;mmu04668;mmu04722;mmu04920;mmu04931;mmu05134;mmu05140;mmu05142;mmu05145;mmu05160;mmu05161;mmu05162;mmu05164;mmu05166;mmu05168;mmu05169;mmu05200;mmu05203;mmu05215;mmu05220;mmu05222 |  | Nfkbia | slateblue1 | 16 | 8.15789473684211 |
  | Dusp7-mRNA | -0.496 | 0.0611 | -0.616 | -0.377 | 0.709 | 0.652 | 0.77 | 4.53e-07 | 9.99e-05 | loglinear | Adaptive Immune Response, Angiogenesis, Cytokine Signaling, Growth Factor Signaling, Innate Immune Response, Insulin Signaling, Microglia Function | NS\_MM\_NEUROINFLAM\_V1.0 | Dusp7 | mRNA | 0 |  | Microglia Function;Insulin Signaling;Innate Immune Response;Growth Factor Signaling;Cytokine Signaling;Angiogenesis;Adaptive Immune Response | mmu04010 |  | Dusp7 | slateblue1 | 16 | -8.11783960720131 |
  | Prkar2a-mRNA | -0.311 | 0.0399 | -0.389 | -0.233 | 0.806 | 0.764 | 0.851 | 7.69e-07 | 0.000143 | loglinear | Apoptosis, Growth Factor Signaling | NS\_MM\_NEUROINFLAM\_V1.0 | Prkar2a | mRNA | 0 |  | Growth Factor Signaling;Apoptosis | mmu04210;mmu04910 |  | Prkar2a | slateblue1 | 16 | -7.79448621553885 |
  | Mapt-mRNA | -0.314 | 0.0425 | -0.397 | -0.23 | 0.805 | 0.759 | 0.852 | 1.56e-06 | 0.000239 | loglinear | Apoptosis, Cellular Stress, Growth Factor Signaling, Neurons and Neurotransmission | NS\_MM\_NEUROINFLAM\_V1.0 | Mapt | mRNA | 0 |  | Neurons and Neurotransmission;Growth Factor Signaling;Cellular Stress;Apoptosis | mmu04010;mmu05010 |  | Mapt | slateblue1 | 16 | -7.38823529411765 |
  | Fos-mRNA | -2.04 | 0.3 | -2.62 | -1.45 | 0.244 | 0.162 | 0.367 | 4.41e-06 | 0.000585 | lm.nb | Adaptive Immune Response, Cellular Stress, Growth Factor Signaling, Innate Immune Response, Neurons and Neurotransmission | NS\_MM\_NEUROINFLAM\_V1.0 | Fos | mRNA | 0 |  | Neurons and Neurotransmission;Innate Immune Response;Growth Factor Signaling;Cellular Stress;Adaptive Immune Response | mmu04010;mmu04024;mmu04380;mmu04620;mmu04660;mmu04662;mmu04668;mmu04713;mmu04725;mmu04728;mmu04915;mmu04917;mmu04921;mmu05031;mmu05132;mmu05133;mmu05140;mmu05142;mmu05161;mmu05166;mmu05168;mmu05200;mmu05210;mmu05231;mmu05323 |  | Fos | slateblue1 | 16 | -6.8 |
  | Csf1r-mRNA | -1 | 0.15 | -1.29 | -0.706 | 0.5 | 0.408 | 0.613 | 5.38e-06 | 0.000678 | lm.nb | Cytokine Signaling, Growth Factor Signaling, Microglia Function | NS\_MM\_NEUROINFLAM\_V1.0 | Csf1r | mRNA | 0 |  | Microglia Function;Growth Factor Signaling;Cytokine Signaling | mmu04014;mmu04015;mmu04060;mmu04144;mmu04151;mmu04380;mmu04640;mmu05200;mmu05202 |  | Csf1r | slateblue1 | 16 | -6.66666666666667 |
  | Fgf13-mRNA | -0.862 | 0.136 | -1.13 | -0.596 | 0.55 | 0.457 | 0.661 | 9.62e-06 | 0.000997 | lm.nb | Growth Factor Signaling, Microglia Function | NS\_MM\_NEUROINFLAM\_V1.0 | Fgf13 | mRNA | 0 |  | Microglia Function;Growth Factor Signaling | mmu04010;mmu04014;mmu04015;mmu04151;mmu04810;mmu05200;mmu05218 |  | Fgf13 | slateblue1 | 16 | -6.33823529411765 |
  | Psmb8-mRNA | 2.23 | 0.352 | 1.54 | 2.92 | 4.7 | 2.91 | 7.59 | 9.89e-06 | 0.000997 | lm.nb | Adaptive Immune Response, Angiogenesis, Apoptosis, Astrocyte Function, Cell Cycle, Cytokine Signaling, Growth Factor Signaling, Inflammatory Signaling, Insulin Signaling, Microglia Function, NF-kB, Wnt | NS\_MM\_NEUROINFLAM\_V1.0 | Psmb8 | mRNA | 0 |  | Wnt;NF-kB;Microglia Function;Insulin Signaling;Inflammatory Signaling;Growth Factor Signaling;Cytokine Signaling;Cell Cycle;Astrocyte Function;Apoptosis;Angiogenesis;Adaptive Immune Response |  |  | Psmb8 | slateblue1 | 16 | 6.33522727272727 |
  | Ppp3r1-mRNA | -0.334 | 0.0577 | -0.447 | -0.221 | 0.793 | 0.734 | 0.858 | 2.79e-05 | 0.00214 | loglinear | Adaptive Immune Response, Apoptosis, Growth Factor Signaling, Innate Immune Response, Wnt | NS\_MM\_NEUROINFLAM\_V1.0 | Ppp3r1 | mRNA | 0 |  | Wnt;Innate Immune Response;Growth Factor Signaling;Apoptosis;Adaptive Immune Response | mmu04010;mmu04020;mmu04022;mmu04114;mmu04210;mmu04310;mmu04360;mmu04370;mmu04380;mmu04650;mmu04660;mmu04662;mmu04720;mmu04724;mmu04921;mmu04922;mmu04924;mmu05010;mmu05014;mmu05031;mmu05152;mmu05166 |  | Ppp3r1 | slateblue1 | 16 | -5.78856152512998 |
  | Ppp3cb-mRNA | -0.554 | 0.098 | -0.747 | -0.362 | 0.681 | 0.596 | 0.778 | 3.56e-05 | 0.00246 | lm.nb | Adaptive Immune Response, Apoptosis, Growth Factor Signaling, Innate Immune Response, Wnt | NS\_MM\_NEUROINFLAM\_V1.0 | Ppp3cb | mRNA | 0 |  | Wnt;Innate Immune Response;Growth Factor Signaling;Apoptosis;Adaptive Immune Response | mmu04010;mmu04020;mmu04022;mmu04114;mmu04210;mmu04310;mmu04360;mmu04370;mmu04380;mmu04650;mmu04660;mmu04662;mmu04720;mmu04724;mmu04728;mmu04921;mmu04922;mmu04924;mmu05010;mmu05014;mmu05031;mmu05152;mmu05166 |  | Ppp3cb | slateblue1 | 16 | -5.6530612244898 |
  | Bcl2l1-mRNA | 0.407 | 0.0734 | 0.263 | 0.551 | 1.33 | 1.2 | 1.46 | 4.51e-05 | 0.00306 | loglinear | Apoptosis, Autophagy, Cytokine Signaling, Growth Factor Signaling, Innate Immune Response, NF-kB | NS\_MM\_NEUROINFLAM\_V1.0 | Bcl2l1 | mRNA | 0 |  | NF-kB;Innate Immune Response;Growth Factor Signaling;Cytokine Signaling;Autophagy;Apoptosis | mmu04014;mmu04064;mmu04151;mmu04210;mmu04630;mmu05014;mmu05145;mmu05166;mmu05200;mmu05202;mmu05212;mmu05220;mmu05222 |  | Bcl2l1 | slateblue1 | 16 | 5.54495912806539 |
  | Tgfbr1-mRNA | -0.546 | 0.102 | -0.745 | -0.347 | 0.685 | 0.596 | 0.786 | 6.2e-05 | 0.00387 | loglinear | Cytokine Signaling, Growth Factor Signaling | NS\_MM\_NEUROINFLAM\_V1.0 | Tgfbr1 | mRNA | 0 |  | Growth Factor Signaling;Cytokine Signaling | mmu04010;mmu04060;mmu04068;mmu04144;mmu04350;mmu04380;mmu04390;mmu04520;mmu04933;mmu05142;mmu05161;mmu05166;mmu05200;mmu05210;mmu05212;mmu05220 |  | Tgfbr1 | slateblue1 | 16 | -5.35294117647059 |
  | Prkar1a-mRNA | -0.452 | 0.0856 | -0.619 | -0.284 | 0.731 | 0.651 | 0.821 | 7.47e-05 | 0.00439 | loglinear | Apoptosis, Growth Factor Signaling | NS\_MM\_NEUROINFLAM\_V1.0 | Prkar1a | mRNA | 0 |  | Growth Factor Signaling;Apoptosis | mmu04210;mmu04910 |  | Prkar1a | slateblue1 | 16 | -5.2803738317757 |
  | Igf1r-mRNA | 0.813 | 0.155 | 0.51 | 1.12 | 1.76 | 1.42 | 2.17 | 7.74e-05 | 0.00441 | lm.nb | Apoptosis, Autophagy, Carbohydrate Metabolism, Growth Factor Signaling, Insulin Signaling | NS\_MM\_NEUROINFLAM\_V1.0 | Igf1r | mRNA | 0 |  | Insulin Signaling;Growth Factor Signaling;Carbohydrate Metabolism;Autophagy;Apoptosis | mmu04014;mmu04015;mmu04066;mmu04068;mmu04114;mmu04144;mmu04151;mmu04152;mmu04211;mmu04213;mmu04510;mmu04520;mmu04550;mmu04730;mmu04913;mmu04914;mmu05200;mmu05202;mmu05205;mmu05214;mmu05215;mmu05218 |  | Igf1r | slateblue1 | 16 | 5.24516129032258 |
  | Prkacb-mRNA | -0.322 | 0.0629 | -0.445 | -0.199 | 0.8 | 0.735 | 0.871 | 0.000104 | 0.00562 | loglinear | Adaptive Immune Response, Angiogenesis, Apoptosis, Autophagy, Growth Factor Signaling, Lipid Metabolism, Neurons and Neurotransmission, Wnt | NS\_MM\_NEUROINFLAM\_V1.0 | Prkacb | mRNA | 0 |  | Wnt;Neurons and Neurotransmission;Lipid Metabolism;Growth Factor Signaling;Autophagy;Apoptosis;Angiogenesis;Adaptive Immune Response | mmu04010;mmu04014;mmu04020;mmu04024;mmu04062;mmu04114;mmu04210;mmu04211;mmu04213;mmu04261;mmu04270;mmu04310;mmu04540;mmu04611;mmu04713;mmu04720;mmu04723;mmu04724;mmu04725;mmu04726;mmu04728;mmu04750;mmu04910;mmu04911;mmu04912;mmu04913;mmu04914;mmu04915;mmu04916;mmu04919;mmu04921;mmu04922;mmu04923;mmu04924;mmu04925;mmu05012;mmu05020;mmu05030;mmu05031;mmu05146;mmu05166;mmu05169;mmu05200;mmu05203;mmu05205;mmu05414 |  | Prkacb | slateblue1 | 16 | -5.11923688394277 |
  | Stmn1-mRNA | -0.298 | 0.0593 | -0.414 | -0.181 | 0.814 | 0.751 | 0.882 | 0.000125 | 0.00632 | loglinear | Growth Factor Signaling, Microglia Function | NS\_MM\_NEUROINFLAM\_V1.0 | Stmn1 | mRNA | 0 |  | Microglia Function;Growth Factor Signaling | mmu04010;mmu05206 |  | Stmn1 | slateblue1 | 16 | -5.02529510961214 |
  | Mapk10-mRNA | -0.673 | 0.138 | -0.943 | -0.402 | 0.627 | 0.52 | 0.757 | 0.000172 | 0.0082 | lm.nb | Adaptive Immune Response, Apoptosis, Autophagy, Cellular Stress, Growth Factor Signaling, Innate Immune Response, Wnt | NS\_MM\_NEUROINFLAM\_V1.0 | Mapk10 | mRNA | 0 |  | Wnt;Innate Immune Response;Growth Factor Signaling;Cellular Stress;Autophagy;Apoptosis;Adaptive Immune Response | mmu04010;mmu04012;mmu04014;mmu04024;mmu04068;mmu04071;mmu04141;mmu04310;mmu04380;mmu04510;mmu04620;mmu04621;mmu04622;mmu04664;mmu04668;mmu04722;mmu04723;mmu04728;mmu04750;mmu04910;mmu04912;mmu04914;mmu04917;mmu04920;mmu04930;mmu04931;mmu04932;mmu04933;mmu05132;mmu05133;mmu05142;mmu05145;mmu05152;mmu05160;mmu05161;mmu05164;mmu05168;mmu05169;mmu05200;mmu05210;mmu05212;mmu05231 |  | Mapk10 | slateblue1 | 16 | -4.8768115942029 |

  ##### DE Results - Growth Factor Signaling genes - DPI: 2 vs.CTRL

  Table displaying each sample's global significance scores and directed global significance scores as defined in the heatmaps above. The global significance score is calculated as the square root of the mean squared t-statistic for the genes in a gene set, with t-statistics coming from the linear regression underlying our differential expression analysis. The directed global significance score is calculated as the square root of the mean signed squared t-statistic for the genes in a gene set, with t-statistics coming from the linear regression underlying our differential expression analysis.

- **Volcano Plot: DPI: 3 vs.CTRL  
  More Plot Information**


  ##### Volcano Plot: DPI: 3 vs.CTRL

  Volcano plot displaying each gene's -log10(p-value) and log2 fold change for the selected covariate. Highly statistically significant genes fall at the top of the plot, and highly differentially expressed genes fall to either side. Genes within the selected gene set are highlighted in orange. Horizontal lines indicate various False Discovery Rate (FDR) thresholds.
- **DE Results - Growth Factor Signaling genes - DPI: 3 vs.CTRL  
  More Plot Information  Download CSV Data**

  | Probe Label | Log2 fold change | std error (log2) | Lower confidence limit (log2) | Upper confidence limit (log2) | Linear fold change | Lower confidence limit (linear) | Upper confidence limit (linear) | P-value | BY.p.value | method | Gene.sets | Codeset.Name | Probe.Label | Analyte.Type | Is.Control | Control.Type | Probe.Annotation | KEGG.Pathways | Cell.Type | Official.Gene.Name | volcanocol | volcanopch | tstats.all |
  | --- | --- | --- | --- | --- | --- | --- | --- | --- | --- | --- | --- | --- | --- | --- | --- | --- | --- | --- | --- | --- | --- | --- | --- |
  | Cdkn1a-mRNA | 2.72 | 0.171 | 2.38 | 3.05 | 6.57 | 5.21 | 8.28 | 3.19e-11 | 3.76e-08 | lm.nb | Adaptive Immune Response, Cell Cycle, Cellular Stress, Cytokine Signaling, DNA Damage, Growth Factor Signaling | NS\_MM\_NEUROINFLAM\_V1.0 | Cdkn1a | mRNA | 0 |  | Growth Factor Signaling;DNA Damage;Cytokine Signaling;Cellular Stress;Cell Cycle;Adaptive Immune Response | mmu04012;mmu04066;mmu04068;mmu04110;mmu04115;mmu04151;mmu04630;mmu04921;mmu05160;mmu05161;mmu05166;mmu05169;mmu05200;mmu05202;mmu05203;mmu05205;mmu05206;mmu05214;mmu05215;mmu05218;mmu05219;mmu05220 |  | Cdkn1a | slateblue1 | 16 | 15.906432748538 |
  | Nfkbia-mRNA | 2.4 | 0.224 | 1.96 | 2.84 | 5.27 | 3.88 | 7.14 | 1.06e-08 | 3.12e-06 | lm.nb | Adaptive Immune Response, Apoptosis, Growth Factor Signaling, Inflammatory Signaling, Innate Immune Response, NF-kB | NS\_MM\_NEUROINFLAM\_V1.0 | Nfkbia | mRNA | 0 |  | NF-kB;Innate Immune Response;Inflammatory Signaling;Growth Factor Signaling;Apoptosis;Adaptive Immune Response | mmu04024;mmu04062;mmu04064;mmu04210;mmu04380;mmu04620;mmu04621;mmu04622;mmu04623;mmu04660;mmu04662;mmu04668;mmu04722;mmu04920;mmu04931;mmu05134;mmu05140;mmu05142;mmu05145;mmu05160;mmu05161;mmu05162;mmu05164;mmu05166;mmu05168;mmu05169;mmu05200;mmu05203;mmu05215;mmu05220;mmu05222 |  | Nfkbia | slateblue1 | 16 | 10.7142857142857 |
  | Mapt-mRNA | -0.413 | 0.0425 | -0.496 | -0.33 | 0.751 | 0.709 | 0.796 | 4.11e-08 | 9.67e-06 | loglinear | Apoptosis, Cellular Stress, Growth Factor Signaling, Neurons and Neurotransmission | NS\_MM\_NEUROINFLAM\_V1.0 | Mapt | mRNA | 0 |  | Neurons and Neurotransmission;Growth Factor Signaling;Cellular Stress;Apoptosis | mmu04010;mmu05010 |  | Mapt | slateblue1 | 16 | -9.71764705882353 |
  | Stmn1-mRNA | -0.555 | 0.0593 | -0.671 | -0.438 | 0.681 | 0.628 | 0.738 | 6.89e-08 | 1.41e-05 | loglinear | Growth Factor Signaling, Microglia Function | NS\_MM\_NEUROINFLAM\_V1.0 | Stmn1 | mRNA | 0 |  | Microglia Function;Growth Factor Signaling | mmu04010;mmu05206 |  | Stmn1 | slateblue1 | 16 | -9.35919055649241 |
  | Prkar2b-mRNA | -1.06 | 0.113 | -1.28 | -0.834 | 0.481 | 0.412 | 0.561 | 7.17e-08 | 1.41e-05 | loglinear | Apoptosis, Cell Cycle, Growth Factor Signaling | NS\_MM\_NEUROINFLAM\_V1.0 | Prkar2b | mRNA | 0 |  | Growth Factor Signaling;Cell Cycle;Apoptosis | mmu04210;mmu04910 |  | Prkar2b | slateblue1 | 16 | -9.38053097345133 |
  | Psmb8-mRNA | 2.99 | 0.346 | 2.31 | 3.67 | 7.95 | 4.97 | 12.7 | 1.99e-07 | 3.2e-05 | lm.nb | Adaptive Immune Response, Angiogenesis, Apoptosis, Astrocyte Function, Cell Cycle, Cytokine Signaling, Growth Factor Signaling, Inflammatory Signaling, Insulin Signaling, Microglia Function, NF-kB, Wnt | NS\_MM\_NEUROINFLAM\_V1.0 | Psmb8 | mRNA | 0 |  | Wnt;NF-kB;Microglia Function;Insulin Signaling;Inflammatory Signaling;Growth Factor Signaling;Cytokine Signaling;Cell Cycle;Astrocyte Function;Apoptosis;Angiogenesis;Adaptive Immune Response |  |  | Psmb8 | slateblue1 | 16 | 8.64161849710983 |
  | Bdnf-mRNA | -1.3 | 0.151 | -1.59 | -1 | 0.407 | 0.332 | 0.5 | 2.23e-07 | 3.39e-05 | lm.nb | Growth Factor Signaling, Neurons and Neurotransmission | NS\_MM\_NEUROINFLAM\_V1.0 | Bdnf | mRNA | 0 |  | Neurons and Neurotransmission;Growth Factor Signaling | mmu04010;mmu04024;mmu04722;mmu05016;mmu05030;mmu05034 |  | Bdnf | slateblue1 | 16 | -8.60927152317881 |
  | Hdac2-mRNA | -0.452 | 0.057 | -0.564 | -0.341 | 0.731 | 0.676 | 0.79 | 6.19e-07 | 7.29e-05 | loglinear | Cell Cycle, Cellular Stress, Epigenetic Regulation, Growth Factor Signaling, Notch | NS\_MM\_NEUROINFLAM\_V1.0 | Hdac2 | mRNA | 0 |  | Notch;Growth Factor Signaling;Epigenetic Regulation;Cellular Stress;Cell Cycle | mmu04110;mmu04213;mmu04330;mmu04919;mmu05016;mmu05034;mmu05169;mmu05200;mmu05202;mmu05203;mmu05220 |  | Hdac2 | slateblue1 | 16 | -7.92982456140351 |
  | Ets2-mRNA | 0.65 | 0.0908 | 0.472 | 0.828 | 1.57 | 1.39 | 1.78 | 2.28e-06 | 0.000206 | loglinear | Cellular Stress, Growth Factor Signaling, Microglia Function | NS\_MM\_NEUROINFLAM\_V1.0 | Ets2 | mRNA | 0 |  | Microglia Function;Growth Factor Signaling;Cellular Stress | mmu04014;mmu05166 |  | Ets2 | slateblue1 | 16 | 7.15859030837004 |
  | Ikbkb-mRNA | -0.601 | 0.0868 | -0.772 | -0.431 | 0.659 | 0.586 | 0.742 | 3.42e-06 | 0.00028 | loglinear | Adaptive Immune Response, Apoptosis, Cytokine Signaling, Growth Factor Signaling, Inflammatory Signaling, Innate Immune Response, NF-kB | NS\_MM\_NEUROINFLAM\_V1.0 | Ikbkb | mRNA | 0 |  | NF-kB;Innate Immune Response;Inflammatory Signaling;Growth Factor Signaling;Cytokine Signaling;Apoptosis;Adaptive Immune Response | mmu04010;mmu04014;mmu04062;mmu04064;mmu04068;mmu04150;mmu04151;mmu04210;mmu04380;mmu04620;mmu04621;mmu04622;mmu04623;mmu04660;mmu04662;mmu04668;mmu04722;mmu04910;mmu04920;mmu04930;mmu04931;mmu04932;mmu05142;mmu05145;mmu05160;mmu05161;mmu05164;mmu05166;mmu05168;mmu05169;mmu05200;mmu05206;mmu05212;mmu05215;mmu05220;mmu05221;mmu05222 |  | Ikbkb | slateblue1 | 16 | -6.92396313364055 |
  | Tnfrsf1a-mRNA | 1.92 | 0.281 | 1.37 | 2.47 | 3.79 | 2.59 | 5.56 | 3.96e-06 | 0.000317 | lm.nb | Apoptosis, Cytokine Signaling, Growth Factor Signaling, Innate Immune Response, NF-kB | NS\_MM\_NEUROINFLAM\_V1.0 | Tnfrsf1a | mRNA | 0 |  | NF-kB;Innate Immune Response;Growth Factor Signaling;Cytokine Signaling;Apoptosis | mmu04010;mmu04060;mmu04064;mmu04071;mmu04210;mmu04380;mmu04668;mmu04920;mmu04931;mmu04932;mmu05010;mmu05014;mmu05142;mmu05145;mmu05152;mmu05160;mmu05164;mmu05166;mmu05168 |  | Tnfrsf1a | slateblue1 | 16 | 6.83274021352313 |
  | Creb1-mRNA | -0.443 | 0.0668 | -0.574 | -0.312 | 0.735 | 0.672 | 0.805 | 5.69e-06 | 0.000418 | loglinear | Adaptive Immune Response, Carbohydrate Metabolism, DNA Damage, Growth Factor Signaling, Innate Immune Response, Neurons and Neurotransmission, Notch | NS\_MM\_NEUROINFLAM\_V1.0 | Creb1 | mRNA | 0 |  | Notch;Neurons and Neurotransmission;Innate Immune Response;Growth Factor Signaling;DNA Damage;Carbohydrate Metabolism;Adaptive Immune Response | mmu04022;mmu04024;mmu04151;mmu04152;mmu04211;mmu04261;mmu04380;mmu04612;mmu04668;mmu04713;mmu04725;mmu04728;mmu04911;mmu04915;mmu04916;mmu04922;mmu04924;mmu04925;mmu04931;mmu05016;mmu05030;mmu05031;mmu05034;mmu05152;mmu05161;mmu05166;mmu05203;mmu05215 |  | Creb1 | slateblue1 | 16 | -6.63173652694611 |
  | Atp6v0e-mRNA | 0.678 | 0.117 | 0.448 | 0.907 | 1.6 | 1.36 | 1.88 | 2.78e-05 | 0.00149 | loglinear | Growth Factor Signaling, Insulin Signaling, Microglia Function | NS\_MM\_NEUROINFLAM\_V1.0 | Atp6v0e | mRNA | 0 |  | Microglia Function;Insulin Signaling;Growth Factor Signaling | mmu04145;mmu04721;mmu05323 |  | Atp6v0e | slateblue1 | 16 | 5.79487179487179 |
  | Prkar2a-mRNA | -0.23 | 0.0399 | -0.308 | -0.152 | 0.853 | 0.808 | 0.9 | 2.9e-05 | 0.00153 | loglinear | Apoptosis, Growth Factor Signaling | NS\_MM\_NEUROINFLAM\_V1.0 | Prkar2a | mRNA | 0 |  | Growth Factor Signaling;Apoptosis | mmu04210;mmu04910 |  | Prkar2a | slateblue1 | 16 | -5.76441102756892 |
  | Bcl2l1-mRNA | 0.414 | 0.0734 | 0.27 | 0.558 | 1.33 | 1.21 | 1.47 | 3.75e-05 | 0.00184 | loglinear | Apoptosis, Autophagy, Cytokine Signaling, Growth Factor Signaling, Innate Immune Response, NF-kB | NS\_MM\_NEUROINFLAM\_V1.0 | Bcl2l1 | mRNA | 0 |  | NF-kB;Innate Immune Response;Growth Factor Signaling;Cytokine Signaling;Autophagy;Apoptosis | mmu04014;mmu04064;mmu04151;mmu04210;mmu04630;mmu05014;mmu05145;mmu05166;mmu05200;mmu05202;mmu05212;mmu05220;mmu05222 |  | Bcl2l1 | slateblue1 | 16 | 5.64032697547684 |
  | Traf6-mRNA | 0.92 | 0.166 | 0.594 | 1.25 | 1.89 | 1.51 | 2.37 | 4.54e-05 | 0.00214 | lm.nb | Adaptive Immune Response, Apoptosis, Autophagy, Cytokine Signaling, Growth Factor Signaling, Innate Immune Response, NF-kB | NS\_MM\_NEUROINFLAM\_V1.0 | Traf6 | mRNA | 0 |  | NF-kB;Innate Immune Response;Growth Factor Signaling;Cytokine Signaling;Autophagy;Apoptosis;Adaptive Immune Response | mmu04010;mmu04064;mmu04120;mmu04144;mmu04380;mmu04620;mmu04621;mmu04622;mmu04722;mmu05133;mmu05140;mmu05142;mmu05145;mmu05152;mmu05160;mmu05162;mmu05168;mmu05169;mmu05200;mmu05222 |  | Traf6 | slateblue1 | 16 | 5.54216867469879 |
  | Akt2-mRNA | -0.766 | 0.142 | -1.04 | -0.488 | 0.588 | 0.485 | 0.713 | 5.85e-05 | 0.00265 | loglinear | Adaptive Immune Response, Angiogenesis, Apoptosis, Autophagy, Carbohydrate Metabolism, Cytokine Signaling, Growth Factor Signaling, Innate Immune Response, Insulin Signaling, Neurons and Neurotransmission, Wnt | NS\_MM\_NEUROINFLAM\_V1.0 | Akt2 | mRNA | 0 |  | Wnt;Neurons and Neurotransmission;Insulin Signaling;Innate Immune Response;Growth Factor Signaling;Cytokine Signaling;Carbohydrate Metabolism;Autophagy;Apoptosis;Angiogenesis;Adaptive Immune Response | mmu04010;mmu04012;mmu04014;mmu04015;mmu04022;mmu04024;mmu04062;mmu04066;mmu04068;mmu04071;mmu04072;mmu04150;mmu04151;mmu04152;mmu04210;mmu04211;mmu04213;mmu04261;mmu04370;mmu04380;mmu04510;mmu04530;mmu04550;mmu04611;mmu04620;mmu04630;mmu04660;mmu04662;mmu04664;mmu04666;mmu04668;mmu04722;mmu04725;mmu04728;mmu04910;mmu04914;mmu04915;mmu04917;mmu04919;mmu04920;mmu04922;mmu04923;mmu04931;mmu04932;mmu04933;mmu04973;mmu05142;mmu05145;mmu05152;mmu05160;mmu05161;mmu05162;mmu05164;mmu05166;mmu05169;mmu05200;mmu05205;mmu05210;mmu05211;mmu05212;mmu05213;mmu05214;mmu05215;mmu05218;mmu05220;mmu05221;mmu05222;mmu05223;mmu05230;mmu05231 |  | Akt2 | slateblue1 | 16 | -5.3943661971831 |
  | Chuk-mRNA | -0.423 | 0.0787 | -0.577 | -0.269 | 0.746 | 0.67 | 0.83 | 6.21e-05 | 0.00275 | loglinear | Adaptive Immune Response, Apoptosis, Cytokine Signaling, Growth Factor Signaling, Inflammatory Signaling, Innate Immune Response, NF-kB | NS\_MM\_NEUROINFLAM\_V1.0 | Chuk | mRNA | 0 |  | NF-kB;Innate Immune Response;Inflammatory Signaling;Growth Factor Signaling;Cytokine Signaling;Apoptosis;Adaptive Immune Response | mmu04010;mmu04014;mmu04062;mmu04064;mmu04068;mmu04151;mmu04210;mmu04380;mmu04620;mmu04621;mmu04622;mmu04623;mmu04660;mmu04662;mmu04668;mmu04920;mmu05142;mmu05145;mmu05160;mmu05161;mmu05162;mmu05166;mmu05168;mmu05169;mmu05200;mmu05212;mmu05215;mmu05220;mmu05221;mmu05222 |  | Chuk | slateblue1 | 16 | -5.37484116899619 |
  | Ago4-mRNA | -2.72 | 0.514 | -3.72 | -1.71 | 0.152 | 0.0757 | 0.306 | 7.46e-05 | 0.00321 | lm.nb | Adaptive Immune Response, Cellular Stress, Epigenetic Regulation, Growth Factor Signaling, Notch, Wnt | NS\_MM\_NEUROINFLAM\_V1.0 | Ago4 | mRNA | 0 |  | Wnt;Notch;Growth Factor Signaling;Epigenetic Regulation;Cellular Stress;Adaptive Immune Response |  |  | Ago4 | slateblue1 | 16 | -5.29182879377432 |
  | Ripk2-mRNA | 1 | 0.193 | 0.627 | 1.38 | 2.01 | 1.54 | 2.61 | 8.44e-05 | 0.00355 | loglinear | Adaptive Immune Response, Apoptosis, Cytokine Signaling, Growth Factor Signaling, Innate Immune Response | NS\_MM\_NEUROINFLAM\_V1.0 | Ripk2 | mRNA | 0 |  | Innate Immune Response;Growth Factor Signaling;Cytokine Signaling;Apoptosis;Adaptive Immune Response | mmu04621;mmu04722;mmu05152 |  | Ripk2 | slateblue1 | 16 | 5.18134715025907 |

  ##### DE Results - Growth Factor Signaling genes - DPI: 3 vs.CTRL

  Table displaying each sample's global significance scores and directed global significance scores as defined in the heatmaps above. The global significance score is calculated as the square root of the mean squared t-statistic for the genes in a gene set, with t-statistics coming from the linear regression underlying our differential expression analysis. The directed global significance score is calculated as the square root of the mean signed squared t-statistic for the genes in a gene set, with t-statistics coming from the linear regression underlying our differential expression analysis.

DPI:
- DPI: differential expression in 1 vs. baseline of CTRL
- DPI: differential expression in 2 vs. baseline of CTRL
- DPI: differential expression in 3 vs. baseline of CTRL

- **Volcano Plot: DPI: 1 vs.CTRL  
  More Plot Information**


  ##### Volcano Plot: DPI: 1 vs.CTRL

  Volcano plot displaying each gene's -log10(p-value) and log2 fold change for the selected covariate. Highly statistically significant genes fall at the top of the plot, and highly differentially expressed genes fall to either side. Genes within the selected gene set are highlighted in orange. Horizontal lines indicate various False Discovery Rate (FDR) thresholds.
- **DE Results - Inflammatory Signaling genes - DPI: 1 vs.CTRL  
  More Plot Information  Download CSV Data**

  | Probe Label | Log2 fold change | std error (log2) | Lower confidence limit (log2) | Upper confidence limit (log2) | Linear fold change | Lower confidence limit (linear) | Upper confidence limit (linear) | P-value | BY.p.value | method | Gene.sets | Codeset.Name | Probe.Label | Analyte.Type | Is.Control | Control.Type | Probe.Annotation | KEGG.Pathways | Cell.Type | Official.Gene.Name | volcanocol | volcanopch | tstats.all |
  | --- | --- | --- | --- | --- | --- | --- | --- | --- | --- | --- | --- | --- | --- | --- | --- | --- | --- | --- | --- | --- | --- | --- | --- |
  | Egr1-mRNA | -1.05 | 0.129 | -1.3 | -0.798 | 0.483 | 0.405 | 0.575 | 4.51e-07 | 0.000796 | lm.nb | Inflammatory Signaling, Neurons and Neurotransmission | NS\_MM\_NEUROINFLAM\_V1.0 | Egr1 | mRNA | 0 |  | Neurons and Neurotransmission;Inflammatory Signaling | mmu04933;mmu05020;mmu05166 |  | Egr1 | slateblue1 | 16 | -8.13953488372093 |
  | Psmb8-mRNA | 1.36 | 0.365 | 0.649 | 2.08 | 2.57 | 1.57 | 4.23 | 0.00179 | 0.129 | lm.nb | Adaptive Immune Response, Angiogenesis, Apoptosis, Astrocyte Function, Cell Cycle, Cytokine Signaling, Growth Factor Signaling, Inflammatory Signaling, Insulin Signaling, Microglia Function, NF-kB, Wnt | NS\_MM\_NEUROINFLAM\_V1.0 | Psmb8 | mRNA | 0 |  | Wnt;NF-kB;Microglia Function;Insulin Signaling;Inflammatory Signaling;Growth Factor Signaling;Cytokine Signaling;Cell Cycle;Astrocyte Function;Apoptosis;Angiogenesis;Adaptive Immune Response |  |  | Psmb8 | slateblue4 | 16 | 3.72602739726027 |
  | Sumo1-mRNA | -0.424 | 0.115 | -0.65 | -0.198 | 0.745 | 0.637 | 0.871 | 0.00202 | 0.136 | lm.nb | Cell Cycle, Cytokine Signaling, DNA Damage, Inflammatory Signaling | NS\_MM\_NEUROINFLAM\_V1.0 | Sumo1 | mRNA | 0 |  | Inflammatory Signaling;DNA Damage;Cytokine Signaling;Cell Cycle |  |  | Sumo1 | slateblue4 | 16 | -3.68695652173913 |
  | Gclc-mRNA | -0.495 | 0.138 | -0.766 | -0.225 | 0.709 | 0.588 | 0.856 | 0.00245 | 0.152 | lm.nb | Inflammatory Signaling | NS\_MM\_NEUROINFLAM\_V1.0 | Gclc | mRNA | 0 |  | Inflammatory Signaling | mmu00270 |  | Gclc | slateblue4 | 16 | -3.58695652173913 |
  | Rsad2-mRNA | 1.49 | 0.456 | 0.597 | 2.38 | 2.81 | 1.51 | 5.22 | 0.0048 | 0.206 | lm.nb | Inflammatory Signaling, Microglia Function | NS\_MM\_NEUROINFLAM\_V1.0 | Rsad2 | mRNA | 0 |  | Microglia Function;Inflammatory Signaling | mmu05164 |  | Rsad2 | slateblue4 | 16 | 3.26754385964912 |
  | Mavs-mRNA | -1.29 | 0.465 | -2.2 | -0.374 | 0.41 | 0.218 | 0.772 | 0.0139 | 0.377 | lm.nb | Inflammatory Signaling, Innate Immune Response | NS\_MM\_NEUROINFLAM\_V1.0 | Mavs | mRNA | 0 |  | Innate Immune Response;Inflammatory Signaling | mmu04622;mmu04623;mmu05160;mmu05161;mmu05162;mmu05164;mmu05168 |  | Mavs | slateblue4 | 16 | -2.7741935483871 |
  | Ifnar1-mRNA | 0.313 | 0.116 | 0.0868 | 0.54 | 1.24 | 1.06 | 1.45 | 0.0154 | 0.41 | loglinear | Cytokine Signaling, Growth Factor Signaling, Inflammatory Signaling, Innate Immune Response | NS\_MM\_NEUROINFLAM\_V1.0 | Ifnar1 | mRNA | 0 |  | Innate Immune Response;Inflammatory Signaling;Growth Factor Signaling;Cytokine Signaling | mmu04060;mmu04151;mmu04380;mmu04620;mmu04630;mmu04650;mmu05160;mmu05161;mmu05162;mmu05164;mmu05168 |  | Ifnar1 | slateblue4 | 16 | 2.69827586206897 |
  | Cx3cl1-mRNA | -0.472 | 0.174 | -0.813 | -0.13 | 0.721 | 0.569 | 0.914 | 0.0157 | 0.41 | lm.nb | Cytokine Signaling, Inflammatory Signaling, Innate Immune Response | NS\_MM\_NEUROINFLAM\_V1.0 | Cx3cl1 | mRNA | 0 |  | Innate Immune Response;Inflammatory Signaling;Cytokine Signaling | mmu04060;mmu04062;mmu04668 |  | Cx3cl1 | slateblue4 | 16 | -2.71264367816092 |
  | Gbp2-mRNA | 2.09 | 0.774 | 0.571 | 3.6 | 4.25 | 1.49 | 12.2 | 0.0158 | 0.41 | lm.nb | Astrocyte Function, Inflammatory Signaling | NS\_MM\_NEUROINFLAM\_V1.0 | Gbp2 | mRNA | 0 |  | Inflammatory Signaling;Astrocyte Function |  |  | Gbp2 | slateblue4 | 16 | 2.70025839793282 |
  | Mcm2-mRNA | -0.981 | 0.389 | -1.74 | -0.218 | 0.507 | 0.299 | 0.86 | 0.0227 | 0.508 | lm.nb | Cell Cycle, Inflammatory Signaling | NS\_MM\_NEUROINFLAM\_V1.0 | Mcm2 | mRNA | 0 |  | Inflammatory Signaling;Cell Cycle | mmu03030;mmu04110 |  | Mcm2 | azure4 | 1 | -2.52185089974293 |
  | Nfkb1-mRNA | -0.581 | 0.234 | -1.04 | -0.123 | 0.669 | 0.487 | 0.918 | 0.0244 | 0.533 | lm.nb | Adaptive Immune Response, Apoptosis, Cellular Stress, Cytokine Signaling, Growth Factor Signaling, Inflammatory Signaling, Innate Immune Response, NF-kB | NS\_MM\_NEUROINFLAM\_V1.0 | Nfkb1 | mRNA | 0 |  | NF-kB;Innate Immune Response;Inflammatory Signaling;Growth Factor Signaling;Cytokine Signaling;Cellular Stress;Apoptosis;Adaptive Immune Response | mmu04010;mmu04014;mmu04024;mmu04062;mmu04064;mmu04066;mmu04071;mmu04151;mmu04210;mmu04211;mmu04380;mmu04620;mmu04621;mmu04622;mmu04623;mmu04660;mmu04662;mmu04668;mmu04722;mmu04917;mmu04920;mmu04931;mmu04932;mmu04933;mmu05030;mmu05132;mmu05133;mmu05134;mmu05140;mmu05142;mmu05145;mmu05146;mmu05152;mmu05160;mmu05161;mmu05162;mmu05164;mmu05166;mmu05168;mmu05169;mmu05200;mmu05202;mmu05203;mmu05206;mmu05212;mmu05215;mmu05220;mmu05221;mmu05222;mmu05321 |  | Nfkb1 | azure4 | 1 | -2.48290598290598 |
  | Fcgr3-mRNA | 0.933 | 0.378 | 0.192 | 1.67 | 1.91 | 1.14 | 3.19 | 0.0252 | 0.539 | lm.nb | Adaptive Immune Response, Autophagy, Inflammatory Signaling, Innate Immune Response | NS\_MM\_NEUROINFLAM\_V1.0 | Fcgr3 | mRNA | 0 |  | Innate Immune Response;Inflammatory Signaling;Autophagy;Adaptive Immune Response | mmu04145;mmu04380;mmu05140;mmu05150;mmu05152 |  | Fcgr3 | azure4 | 1 | 2.46825396825397 |
  | Lcn2-mRNA | 1.85 | 0.799 | 0.283 | 3.41 | 3.6 | 1.22 | 10.7 | 0.0343 | 0.654 | lm.nb | Astrocyte Function, Inflammatory Signaling | NS\_MM\_NEUROINFLAM\_V1.0 | Lcn2 | mRNA | 0 |  | Inflammatory Signaling;Astrocyte Function |  |  | Lcn2 | azure4 | 1 | 2.3153942428035 |
  | Gstm1-mRNA | -0.418 | 0.197 | -0.805 | -0.0323 | 0.748 | 0.573 | 0.978 | 0.0496 | 0.834 | loglinear | Cellular Stress, Inflammatory Signaling | NS\_MM\_NEUROINFLAM\_V1.0 | Gstm1 | mRNA | 0 |  | Inflammatory Signaling;Cellular Stress |  |  | Gstm1 | azure4 | 1 | -2.12182741116751 |
  | Traf2-mRNA | -0.257 | 0.126 | -0.505 | -0.0103 | 0.837 | 0.705 | 0.993 | 0.058 | 0.918 | lm.nb | Apoptosis, Cytokine Signaling, Growth Factor Signaling, Inflammatory Signaling, Innate Immune Response, NF-kB | NS\_MM\_NEUROINFLAM\_V1.0 | Traf2 | mRNA | 0 |  | NF-kB;Innate Immune Response;Inflammatory Signaling;Growth Factor Signaling;Cytokine Signaling;Apoptosis | mmu04010;mmu04064;mmu04071;mmu04141;mmu04210;mmu04380;mmu04622;mmu04668;mmu04920;mmu04932;mmu05160;mmu05168;mmu05169;mmu05200;mmu05203;mmu05222 |  | Traf2 | azure4 | 1 | -2.03968253968254 |
  | Tnfrsf12a-mRNA | -0.72 | 0.359 | -1.42 | -0.0165 | 0.607 | 0.373 | 0.989 | 0.0621 | 0.948 | lm.nb | Cytokine Signaling, Inflammatory Signaling, NF-kB | NS\_MM\_NEUROINFLAM\_V1.0 | Tnfrsf12a | mRNA | 0 |  | NF-kB;Inflammatory Signaling;Cytokine Signaling | mmu04060 |  | Tnfrsf12a | azure4 | 1 | -2.00557103064067 |
  | Cyp7b1-mRNA | 0.333 | 0.176 | -0.0119 | 0.677 | 1.26 | 0.992 | 1.6 | 0.0767 | 1 | lm.nb | Inflammatory Signaling | NS\_MM\_NEUROINFLAM\_V1.0 | Cyp7b1 | mRNA | 0 |  | Inflammatory Signaling |  |  | Cyp7b1 | azure4 | 1 | 1.89204545454545 |
  | Ep300-mRNA | -0.209 | 0.111 | -0.425 | 0.008 | 0.865 | 0.745 | 1.01 | 0.0774 | 1 | loglinear | Cell Cycle, Cellular Stress, Cytokine Signaling, DNA Damage, Epigenetic Regulation, Inflammatory Signaling, Innate Immune Response, Lipid Metabolism, Notch, Wnt | NS\_MM\_NEUROINFLAM\_V1.0 | Ep300 | mRNA | 0 |  | Wnt;Notch;Lipid Metabolism;Innate Immune Response;Inflammatory Signaling;Epigenetic Regulation;DNA Damage;Cytokine Signaling;Cellular Stress;Cell Cycle | mmu04024;mmu04066;mmu04068;mmu04110;mmu04310;mmu04330;mmu04350;mmu04520;mmu04630;mmu04720;mmu04916;mmu04919;mmu04922;mmu05016;mmu05152;mmu05161;mmu05164;mmu05166;mmu05168;mmu05169;mmu05200;mmu05203;mmu05206;mmu05211;mmu05215 |  | Ep300 | azure4 | 1 | -1.88288288288288 |
  | Traf3-mRNA | -0.195 | 0.104 | -0.398 | 0.00834 | 0.874 | 0.759 | 1.01 | 0.0785 | 1 | loglinear | Apoptosis, Cytokine Signaling, Inflammatory Signaling, Innate Immune Response, NF-kB | NS\_MM\_NEUROINFLAM\_V1.0 | Traf3 | mRNA | 0 |  | NF-kB;Innate Immune Response;Inflammatory Signaling;Cytokine Signaling;Apoptosis | mmu04064;mmu04620;mmu04622;mmu04668;mmu05160;mmu05168;mmu05169;mmu05200;mmu05203;mmu05222 |  | Traf3 | azure4 | 1 | -1.875 |
  | Crebbp-mRNA | -0.0726 | 0.0406 | -0.152 | 0.00705 | 0.951 | 0.9 | 1 | 0.093 | 1 | loglinear | Cell Cycle, Cellular Stress, Cytokine Signaling, Epigenetic Regulation, Inflammatory Signaling, Innate Immune Response, Lipid Metabolism, Notch, Wnt | NS\_MM\_NEUROINFLAM\_V1.0 | Crebbp | mRNA | 0 |  | Wnt;Notch;Lipid Metabolism;Innate Immune Response;Inflammatory Signaling;Epigenetic Regulation;Cytokine Signaling;Cellular Stress;Cell Cycle | mmu04024;mmu04066;mmu04068;mmu04110;mmu04310;mmu04330;mmu04350;mmu04520;mmu04630;mmu04720;mmu04916;mmu04919;mmu04922;mmu05016;mmu05152;mmu05161;mmu05164;mmu05166;mmu05168;mmu05169;mmu05200;mmu05203;mmu05206;mmu05211;mmu05215 |  | Crebbp | azure4 | 1 | -1.78817733990148 |

  ##### DE Results - Inflammatory Signaling genes - DPI: 1 vs.CTRL

  Table displaying each sample's global significance scores and directed global significance scores as defined in the heatmaps above. The global significance score is calculated as the square root of the mean squared t-statistic for the genes in a gene set, with t-statistics coming from the linear regression underlying our differential expression analysis. The directed global significance score is calculated as the square root of the mean signed squared t-statistic for the genes in a gene set, with t-statistics coming from the linear regression underlying our differential expression analysis.

- **Volcano Plot: DPI: 2 vs.CTRL  
  More Plot Information**


  ##### Volcano Plot: DPI: 2 vs.CTRL

  Volcano plot displaying each gene's -log10(p-value) and log2 fold change for the selected covariate. Highly statistically significant genes fall at the top of the plot, and highly differentially expressed genes fall to either side. Genes within the selected gene set are highlighted in orange. Horizontal lines indicate various False Discovery Rate (FDR) thresholds.
- **DE Results - Inflammatory Signaling genes - DPI: 2 vs.CTRL  
  More Plot Information  Download CSV Data**

  | Probe Label | Log2 fold change | std error (log2) | Lower confidence limit (log2) | Upper confidence limit (log2) | Linear fold change | Lower confidence limit (linear) | Upper confidence limit (linear) | P-value | BY.p.value | method | Gene.sets | Codeset.Name | Probe.Label | Analyte.Type | Is.Control | Control.Type | Probe.Annotation | KEGG.Pathways | Cell.Type | Official.Gene.Name | volcanocol | volcanopch | tstats.all |
  | --- | --- | --- | --- | --- | --- | --- | --- | --- | --- | --- | --- | --- | --- | --- | --- | --- | --- | --- | --- | --- | --- | --- | --- |
  | Lcn2-mRNA | 9.71 | 0.723 | 8.3 | 11.1 | 839 | 314 | 2240 | 3.93e-10 | 6.02e-07 | lm.nb | Astrocyte Function, Inflammatory Signaling | NS\_MM\_NEUROINFLAM\_V1.0 | Lcn2 | mRNA | 0 |  | Inflammatory Signaling;Astrocyte Function |  |  | Lcn2 | slateblue1 | 16 | 13.4301521438451 |
  | Egr1-mRNA | -1.71 | 0.13 | -1.97 | -1.46 | 0.305 | 0.255 | 0.363 | 5.12e-10 | 6.02e-07 | lm.nb | Inflammatory Signaling, Neurons and Neurotransmission | NS\_MM\_NEUROINFLAM\_V1.0 | Egr1 | mRNA | 0 |  | Neurons and Neurotransmission;Inflammatory Signaling | mmu04933;mmu05020;mmu05166 |  | Egr1 | slateblue1 | 16 | -13.1538461538462 |
  | Nfkbia-mRNA | 1.86 | 0.228 | 1.41 | 2.31 | 3.63 | 2.67 | 4.95 | 4.26e-07 | 9.99e-05 | lm.nb | Adaptive Immune Response, Apoptosis, Growth Factor Signaling, Inflammatory Signaling, Innate Immune Response, NF-kB | NS\_MM\_NEUROINFLAM\_V1.0 | Nfkbia | mRNA | 0 |  | NF-kB;Innate Immune Response;Inflammatory Signaling;Growth Factor Signaling;Apoptosis;Adaptive Immune Response | mmu04024;mmu04062;mmu04064;mmu04210;mmu04380;mmu04620;mmu04621;mmu04622;mmu04623;mmu04660;mmu04662;mmu04668;mmu04722;mmu04920;mmu04931;mmu05134;mmu05140;mmu05142;mmu05145;mmu05160;mmu05161;mmu05162;mmu05164;mmu05166;mmu05168;mmu05169;mmu05200;mmu05203;mmu05215;mmu05220;mmu05222 |  | Nfkbia | slateblue1 | 16 | 8.15789473684211 |
  | Psmb8-mRNA | 2.23 | 0.352 | 1.54 | 2.92 | 4.7 | 2.91 | 7.59 | 9.89e-06 | 0.000997 | lm.nb | Adaptive Immune Response, Angiogenesis, Apoptosis, Astrocyte Function, Cell Cycle, Cytokine Signaling, Growth Factor Signaling, Inflammatory Signaling, Insulin Signaling, Microglia Function, NF-kB, Wnt | NS\_MM\_NEUROINFLAM\_V1.0 | Psmb8 | mRNA | 0 |  | Wnt;NF-kB;Microglia Function;Insulin Signaling;Inflammatory Signaling;Growth Factor Signaling;Cytokine Signaling;Cell Cycle;Astrocyte Function;Apoptosis;Angiogenesis;Adaptive Immune Response |  |  | Psmb8 | slateblue1 | 16 | 6.33522727272727 |
  | Gbp2-mRNA | 4.37 | 0.741 | 2.91 | 5.82 | 20.6 | 7.53 | 56.5 | 2.29e-05 | 0.00179 | lm.nb | Astrocyte Function, Inflammatory Signaling | NS\_MM\_NEUROINFLAM\_V1.0 | Gbp2 | mRNA | 0 |  | Inflammatory Signaling;Astrocyte Function |  |  | Gbp2 | slateblue1 | 16 | 5.8974358974359 |
  | Fcgr3-mRNA | 2.05 | 0.36 | 1.34 | 2.75 | 4.14 | 2.54 | 6.74 | 3.32e-05 | 0.0024 | lm.nb | Adaptive Immune Response, Autophagy, Inflammatory Signaling, Innate Immune Response | NS\_MM\_NEUROINFLAM\_V1.0 | Fcgr3 | mRNA | 0 |  | Innate Immune Response;Inflammatory Signaling;Autophagy;Adaptive Immune Response | mmu04145;mmu04380;mmu05140;mmu05150;mmu05152 |  | Fcgr3 | slateblue1 | 16 | 5.69444444444444 |
  | Irf1-mRNA | 1.63 | 0.335 | 0.973 | 2.29 | 3.09 | 1.96 | 4.88 | 0.000172 | 0.0082 | lm.nb | Apoptosis, Inflammatory Signaling, Innate Immune Response | NS\_MM\_NEUROINFLAM\_V1.0 | Irf1 | mRNA | 0 |  | Innate Immune Response;Inflammatory Signaling;Apoptosis | mmu04917;mmu05133;mmu05160 |  | Irf1 | slateblue1 | 16 | 4.86567164179104 |
  | Sumo1-mRNA | -0.529 | 0.115 | -0.755 | -0.303 | 0.693 | 0.593 | 0.811 | 0.000305 | 0.0121 | lm.nb | Cell Cycle, Cytokine Signaling, DNA Damage, Inflammatory Signaling | NS\_MM\_NEUROINFLAM\_V1.0 | Sumo1 | mRNA | 0 |  | Inflammatory Signaling;DNA Damage;Cytokine Signaling;Cell Cycle |  |  | Sumo1 | slateblue2 | 16 | -4.6 |
  | Ifitm2-mRNA | 0.804 | 0.179 | 0.454 | 1.15 | 1.75 | 1.37 | 2.23 | 0.000363 | 0.0136 | lm.nb | Adaptive Immune Response, Inflammatory Signaling | NS\_MM\_NEUROINFLAM\_V1.0 | Ifitm2 | mRNA | 0 |  | Inflammatory Signaling;Adaptive Immune Response |  |  | Ifitm2 | slateblue2 | 16 | 4.49162011173184 |
  | Ifitm3-mRNA | 1.53 | 0.344 | 0.854 | 2.2 | 2.89 | 1.81 | 4.61 | 0.000411 | 0.0148 | lm.nb | Adaptive Immune Response, Inflammatory Signaling | NS\_MM\_NEUROINFLAM\_V1.0 | Ifitm3 | mRNA | 0 |  | Inflammatory Signaling;Adaptive Immune Response |  |  | Ifitm3 | slateblue2 | 16 | 4.44767441860465 |
  | Chuk-mRNA | -0.331 | 0.0787 | -0.485 | -0.176 | 0.795 | 0.715 | 0.885 | 0.000678 | 0.0218 | loglinear | Adaptive Immune Response, Apoptosis, Cytokine Signaling, Growth Factor Signaling, Inflammatory Signaling, Innate Immune Response, NF-kB | NS\_MM\_NEUROINFLAM\_V1.0 | Chuk | mRNA | 0 |  | NF-kB;Innate Immune Response;Inflammatory Signaling;Growth Factor Signaling;Cytokine Signaling;Apoptosis;Adaptive Immune Response | mmu04010;mmu04014;mmu04062;mmu04064;mmu04068;mmu04151;mmu04210;mmu04380;mmu04620;mmu04621;mmu04622;mmu04623;mmu04660;mmu04662;mmu04668;mmu04920;mmu05142;mmu05145;mmu05160;mmu05161;mmu05162;mmu05166;mmu05168;mmu05169;mmu05200;mmu05212;mmu05215;mmu05220;mmu05221;mmu05222 |  | Chuk | slateblue2 | 16 | -4.20584498094028 |
  | Hmgb1-mRNA | -0.277 | 0.0677 | -0.41 | -0.145 | 0.825 | 0.753 | 0.905 | 0.00084 | 0.0239 | loglinear | Apoptosis, Autophagy, Inflammatory Signaling, Innate Immune Response | NS\_MM\_NEUROINFLAM\_V1.0 | Hmgb1 | mRNA | 0 |  | Innate Immune Response;Inflammatory Signaling;Autophagy;Apoptosis | mmu03410 |  | Hmgb1 | slateblue2 | 16 | -4.09158050221566 |
  | Mcm2-mRNA | -1.77 | 0.437 | -2.63 | -0.912 | 0.293 | 0.162 | 0.531 | 0.000938 | 0.0265 | lm.nb | Cell Cycle, Inflammatory Signaling | NS\_MM\_NEUROINFLAM\_V1.0 | Mcm2 | mRNA | 0 |  | Inflammatory Signaling;Cell Cycle | mmu03030;mmu04110 |  | Mcm2 | slateblue2 | 16 | -4.05034324942792 |
  | Rsad2-mRNA | 1.78 | 0.448 | 0.899 | 2.66 | 3.43 | 1.86 | 6.31 | 0.00111 | 0.0293 | lm.nb | Inflammatory Signaling, Microglia Function | NS\_MM\_NEUROINFLAM\_V1.0 | Rsad2 | mRNA | 0 |  | Microglia Function;Inflammatory Signaling | mmu05164 |  | Rsad2 | slateblue2 | 16 | 3.97321428571429 |
  | Cyp7b1-mRNA | 0.659 | 0.171 | 0.323 | 0.994 | 1.58 | 1.25 | 1.99 | 0.00143 | 0.035 | lm.nb | Inflammatory Signaling | NS\_MM\_NEUROINFLAM\_V1.0 | Cyp7b1 | mRNA | 0 |  | Inflammatory Signaling |  |  | Cyp7b1 | slateblue2 | 16 | 3.85380116959064 |
  | Relb-mRNA | -0.486 | 0.137 | -0.755 | -0.218 | 0.714 | 0.593 | 0.86 | 0.00268 | 0.0557 | loglinear | Cytokine Signaling, Growth Factor Signaling, Inflammatory Signaling, NF-kB | NS\_MM\_NEUROINFLAM\_V1.0 | Relb | mRNA | 0 |  | NF-kB;Inflammatory Signaling;Growth Factor Signaling;Cytokine Signaling | mmu04010;mmu04064;mmu04380;mmu05166;mmu05169 |  | Relb | slateblue3 | 16 | -3.54744525547445 |
  | Cx3cl1-mRNA | -0.617 | 0.175 | -0.96 | -0.274 | 0.652 | 0.514 | 0.827 | 0.00283 | 0.0581 | lm.nb | Cytokine Signaling, Inflammatory Signaling, Innate Immune Response | NS\_MM\_NEUROINFLAM\_V1.0 | Cx3cl1 | mRNA | 0 |  | Innate Immune Response;Inflammatory Signaling;Cytokine Signaling | mmu04060;mmu04062;mmu04668 |  | Cx3cl1 | slateblue3 | 16 | -3.52571428571429 |
  | Ikbkb-mRNA | -0.299 | 0.0868 | -0.47 | -0.129 | 0.813 | 0.722 | 0.914 | 0.00331 | 0.0665 | loglinear | Adaptive Immune Response, Apoptosis, Cytokine Signaling, Growth Factor Signaling, Inflammatory Signaling, Innate Immune Response, NF-kB | NS\_MM\_NEUROINFLAM\_V1.0 | Ikbkb | mRNA | 0 |  | NF-kB;Innate Immune Response;Inflammatory Signaling;Growth Factor Signaling;Cytokine Signaling;Apoptosis;Adaptive Immune Response | mmu04010;mmu04014;mmu04062;mmu04064;mmu04068;mmu04150;mmu04151;mmu04210;mmu04380;mmu04620;mmu04621;mmu04622;mmu04623;mmu04660;mmu04662;mmu04668;mmu04722;mmu04910;mmu04920;mmu04930;mmu04931;mmu04932;mmu05142;mmu05145;mmu05160;mmu05161;mmu05164;mmu05166;mmu05168;mmu05169;mmu05200;mmu05206;mmu05212;mmu05215;mmu05220;mmu05221;mmu05222 |  | Ikbkb | slateblue3 | 16 | -3.44470046082949 |
  | Irf2-mRNA | 0.216 | 0.0696 | 0.0795 | 0.352 | 1.16 | 1.06 | 1.28 | 0.00685 | 0.121 | loglinear | Apoptosis, Inflammatory Signaling, Innate Immune Response | NS\_MM\_NEUROINFLAM\_V1.0 | Irf2 | mRNA | 0 |  | Innate Immune Response;Inflammatory Signaling;Apoptosis |  |  | Irf2 | slateblue4 | 16 | 3.10344827586207 |
  | Traf2-mRNA | -0.396 | 0.128 | -0.646 | -0.146 | 0.76 | 0.639 | 0.904 | 0.00685 | 0.121 | lm.nb | Apoptosis, Cytokine Signaling, Growth Factor Signaling, Inflammatory Signaling, Innate Immune Response, NF-kB | NS\_MM\_NEUROINFLAM\_V1.0 | Traf2 | mRNA | 0 |  | NF-kB;Innate Immune Response;Inflammatory Signaling;Growth Factor Signaling;Cytokine Signaling;Apoptosis | mmu04010;mmu04064;mmu04071;mmu04141;mmu04210;mmu04380;mmu04622;mmu04668;mmu04920;mmu04932;mmu05160;mmu05168;mmu05169;mmu05200;mmu05203;mmu05222 |  | Traf2 | slateblue4 | 16 | -3.09375 |

  ##### DE Results - Inflammatory Signaling genes - DPI: 2 vs.CTRL

  Table displaying each sample's global significance scores and directed global significance scores as defined in the heatmaps above. The global significance score is calculated as the square root of the mean squared t-statistic for the genes in a gene set, with t-statistics coming from the linear regression underlying our differential expression analysis. The directed global significance score is calculated as the square root of the mean signed squared t-statistic for the genes in a gene set, with t-statistics coming from the linear regression underlying our differential expression analysis.

- **Volcano Plot: DPI: 3 vs.CTRL  
  More Plot Information**


  ##### Volcano Plot: DPI: 3 vs.CTRL

  Volcano plot displaying each gene's -log10(p-value) and log2 fold change for the selected covariate. Highly statistically significant genes fall at the top of the plot, and highly differentially expressed genes fall to either side. Genes within the selected gene set are highlighted in orange. Horizontal lines indicate various False Discovery Rate (FDR) thresholds.
- **DE Results - Inflammatory Signaling genes - DPI: 3 vs.CTRL  
  More Plot Information  Download CSV Data**

  | Probe Label | Log2 fold change | std error (log2) | Lower confidence limit (log2) | Upper confidence limit (log2) | Linear fold change | Lower confidence limit (linear) | Upper confidence limit (linear) | P-value | BY.p.value | method | Gene.sets | Codeset.Name | Probe.Label | Analyte.Type | Is.Control | Control.Type | Probe.Annotation | KEGG.Pathways | Cell.Type | Official.Gene.Name | volcanocol | volcanopch | tstats.all |
  | --- | --- | --- | --- | --- | --- | --- | --- | --- | --- | --- | --- | --- | --- | --- | --- | --- | --- | --- | --- | --- | --- | --- | --- |
  | Lcn2-mRNA | 11 | 0.723 | 9.57 | 12.4 | 2030 | 759 | 5410 | 6.27e-11 | 5.53e-08 | lm.nb | Astrocyte Function, Inflammatory Signaling | NS\_MM\_NEUROINFLAM\_V1.0 | Lcn2 | mRNA | 0 |  | Inflammatory Signaling;Astrocyte Function |  |  | Lcn2 | slateblue1 | 16 | 15.2143845089903 |
  | Egr1-mRNA | -1.51 | 0.13 | -1.77 | -1.26 | 0.351 | 0.294 | 0.418 | 3.11e-09 | 1.57e-06 | lm.nb | Inflammatory Signaling, Neurons and Neurotransmission | NS\_MM\_NEUROINFLAM\_V1.0 | Egr1 | mRNA | 0 |  | Neurons and Neurotransmission;Inflammatory Signaling | mmu04933;mmu05020;mmu05166 |  | Egr1 | slateblue1 | 16 | -11.6153846153846 |
  | Nfkbia-mRNA | 2.4 | 0.224 | 1.96 | 2.84 | 5.27 | 3.88 | 7.14 | 1.06e-08 | 3.12e-06 | lm.nb | Adaptive Immune Response, Apoptosis, Growth Factor Signaling, Inflammatory Signaling, Innate Immune Response, NF-kB | NS\_MM\_NEUROINFLAM\_V1.0 | Nfkbia | mRNA | 0 |  | NF-kB;Innate Immune Response;Inflammatory Signaling;Growth Factor Signaling;Apoptosis;Adaptive Immune Response | mmu04024;mmu04062;mmu04064;mmu04210;mmu04380;mmu04620;mmu04621;mmu04622;mmu04623;mmu04660;mmu04662;mmu04668;mmu04722;mmu04920;mmu04931;mmu05134;mmu05140;mmu05142;mmu05145;mmu05160;mmu05161;mmu05162;mmu05164;mmu05166;mmu05168;mmu05169;mmu05200;mmu05203;mmu05215;mmu05220;mmu05222 |  | Nfkbia | slateblue1 | 16 | 10.7142857142857 |
  | Ifitm2-mRNA | 1.82 | 0.174 | 1.47 | 2.16 | 3.52 | 2.78 | 4.46 | 1.51e-08 | 4.09e-06 | lm.nb | Adaptive Immune Response, Inflammatory Signaling | NS\_MM\_NEUROINFLAM\_V1.0 | Ifitm2 | mRNA | 0 |  | Inflammatory Signaling;Adaptive Immune Response |  |  | Ifitm2 | slateblue1 | 16 | 10.4597701149425 |
  | Irf3-mRNA | -1.48 | 0.165 | -1.8 | -1.15 | 0.359 | 0.287 | 0.45 | 1.3e-07 | 2.18e-05 | lm.nb | Apoptosis, Cytokine Signaling, Inflammatory Signaling, Innate Immune Response | NS\_MM\_NEUROINFLAM\_V1.0 | Irf3 | mRNA | 0 |  | Innate Immune Response;Inflammatory Signaling;Cytokine Signaling;Apoptosis | mmu04620;mmu04622;mmu04623;mmu05133;mmu05160;mmu05161;mmu05162;mmu05164;mmu05168;mmu05169;mmu05203 |  | Irf3 | slateblue1 | 16 | -8.96969696969697 |
  | Psmb8-mRNA | 2.99 | 0.346 | 2.31 | 3.67 | 7.95 | 4.97 | 12.7 | 1.99e-07 | 3.2e-05 | lm.nb | Adaptive Immune Response, Angiogenesis, Apoptosis, Astrocyte Function, Cell Cycle, Cytokine Signaling, Growth Factor Signaling, Inflammatory Signaling, Insulin Signaling, Microglia Function, NF-kB, Wnt | NS\_MM\_NEUROINFLAM\_V1.0 | Psmb8 | mRNA | 0 |  | Wnt;NF-kB;Microglia Function;Insulin Signaling;Inflammatory Signaling;Growth Factor Signaling;Cytokine Signaling;Cell Cycle;Astrocyte Function;Apoptosis;Angiogenesis;Adaptive Immune Response |  |  | Psmb8 | slateblue1 | 16 | 8.64161849710983 |
  | Irf1-mRNA | 2.84 | 0.332 | 2.19 | 3.49 | 7.17 | 4.57 | 11.3 | 2.3e-07 | 3.39e-05 | lm.nb | Apoptosis, Inflammatory Signaling, Innate Immune Response | NS\_MM\_NEUROINFLAM\_V1.0 | Irf1 | mRNA | 0 |  | Innate Immune Response;Inflammatory Signaling;Apoptosis | mmu04917;mmu05133;mmu05160 |  | Irf1 | slateblue1 | 16 | 8.55421686746988 |
  | Gbp2-mRNA | 5.64 | 0.736 | 4.19 | 7.08 | 49.7 | 18.3 | 135 | 9.8e-07 | 0.000105 | lm.nb | Astrocyte Function, Inflammatory Signaling | NS\_MM\_NEUROINFLAM\_V1.0 | Gbp2 | mRNA | 0 |  | Inflammatory Signaling;Astrocyte Function |  |  | Gbp2 | slateblue1 | 16 | 7.66304347826087 |
  | Ifitm3-mRNA | 2.54 | 0.344 | 1.87 | 3.22 | 5.83 | 3.65 | 9.29 | 1.51e-06 | 0.000151 | lm.nb | Adaptive Immune Response, Inflammatory Signaling | NS\_MM\_NEUROINFLAM\_V1.0 | Ifitm3 | mRNA | 0 |  | Inflammatory Signaling;Adaptive Immune Response |  |  | Ifitm3 | slateblue1 | 16 | 7.38372093023256 |
  | Srgn-mRNA | 2.18 | 0.297 | 1.59 | 2.76 | 4.52 | 3.02 | 6.77 | 1.72e-06 | 0.000164 | lm.nb | Astrocyte Function, Inflammatory Signaling | NS\_MM\_NEUROINFLAM\_V1.0 | Srgn | mRNA | 0 |  | Inflammatory Signaling;Astrocyte Function |  |  | Srgn | slateblue1 | 16 | 7.34006734006734 |
  | Ikbkb-mRNA | -0.601 | 0.0868 | -0.772 | -0.431 | 0.659 | 0.586 | 0.742 | 3.42e-06 | 0.00028 | loglinear | Adaptive Immune Response, Apoptosis, Cytokine Signaling, Growth Factor Signaling, Inflammatory Signaling, Innate Immune Response, NF-kB | NS\_MM\_NEUROINFLAM\_V1.0 | Ikbkb | mRNA | 0 |  | NF-kB;Innate Immune Response;Inflammatory Signaling;Growth Factor Signaling;Cytokine Signaling;Apoptosis;Adaptive Immune Response | mmu04010;mmu04014;mmu04062;mmu04064;mmu04068;mmu04150;mmu04151;mmu04210;mmu04380;mmu04620;mmu04621;mmu04622;mmu04623;mmu04660;mmu04662;mmu04668;mmu04722;mmu04910;mmu04920;mmu04930;mmu04931;mmu04932;mmu05142;mmu05145;mmu05160;mmu05161;mmu05164;mmu05166;mmu05168;mmu05169;mmu05200;mmu05206;mmu05212;mmu05215;mmu05220;mmu05221;mmu05222 |  | Ikbkb | slateblue1 | 16 | -6.92396313364055 |
  | Rsad2-mRNA | 2.54 | 0.435 | 1.69 | 3.39 | 5.81 | 3.22 | 10.5 | 2.54e-05 | 0.00141 | lm.nb | Inflammatory Signaling, Microglia Function | NS\_MM\_NEUROINFLAM\_V1.0 | Rsad2 | mRNA | 0 |  | Microglia Function;Inflammatory Signaling | mmu05164 |  | Rsad2 | slateblue1 | 16 | 5.83908045977012 |
  | Chuk-mRNA | -0.423 | 0.0787 | -0.577 | -0.269 | 0.746 | 0.67 | 0.83 | 6.21e-05 | 0.00275 | loglinear | Adaptive Immune Response, Apoptosis, Cytokine Signaling, Growth Factor Signaling, Inflammatory Signaling, Innate Immune Response, NF-kB | NS\_MM\_NEUROINFLAM\_V1.0 | Chuk | mRNA | 0 |  | NF-kB;Innate Immune Response;Inflammatory Signaling;Growth Factor Signaling;Cytokine Signaling;Apoptosis;Adaptive Immune Response | mmu04010;mmu04014;mmu04062;mmu04064;mmu04068;mmu04151;mmu04210;mmu04380;mmu04620;mmu04621;mmu04622;mmu04623;mmu04660;mmu04662;mmu04668;mmu04920;mmu05142;mmu05145;mmu05160;mmu05161;mmu05162;mmu05166;mmu05168;mmu05169;mmu05200;mmu05212;mmu05215;mmu05220;mmu05221;mmu05222 |  | Chuk | slateblue1 | 16 | -5.37484116899619 |
  | Traf3-mRNA | -0.463 | 0.104 | -0.666 | -0.26 | 0.725 | 0.63 | 0.835 | 0.000386 | 0.013 | loglinear | Apoptosis, Cytokine Signaling, Inflammatory Signaling, Innate Immune Response, NF-kB | NS\_MM\_NEUROINFLAM\_V1.0 | Traf3 | mRNA | 0 |  | NF-kB;Innate Immune Response;Inflammatory Signaling;Cytokine Signaling;Apoptosis | mmu04064;mmu04620;mmu04622;mmu04668;mmu05160;mmu05168;mmu05169;mmu05200;mmu05203;mmu05222 |  | Traf3 | slateblue2 | 16 | -4.45192307692308 |
  | Fcgr3-mRNA | 1.62 | 0.365 | 0.902 | 2.33 | 3.07 | 1.87 | 5.04 | 0.000421 | 0.0139 | lm.nb | Adaptive Immune Response, Autophagy, Inflammatory Signaling, Innate Immune Response | NS\_MM\_NEUROINFLAM\_V1.0 | Fcgr3 | mRNA | 0 |  | Innate Immune Response;Inflammatory Signaling;Autophagy;Adaptive Immune Response | mmu04145;mmu04380;mmu05140;mmu05150;mmu05152 |  | Fcgr3 | slateblue2 | 16 | 4.43835616438356 |
  | Traf2-mRNA | -0.552 | 0.129 | -0.805 | -0.298 | 0.682 | 0.572 | 0.813 | 0.000592 | 0.0183 | lm.nb | Apoptosis, Cytokine Signaling, Growth Factor Signaling, Inflammatory Signaling, Innate Immune Response, NF-kB | NS\_MM\_NEUROINFLAM\_V1.0 | Traf2 | mRNA | 0 |  | NF-kB;Innate Immune Response;Inflammatory Signaling;Growth Factor Signaling;Cytokine Signaling;Apoptosis | mmu04010;mmu04064;mmu04071;mmu04141;mmu04210;mmu04380;mmu04622;mmu04668;mmu04920;mmu04932;mmu05160;mmu05168;mmu05169;mmu05200;mmu05203;mmu05222 |  | Traf2 | slateblue2 | 16 | -4.27906976744186 |
  | Ddx58-mRNA | 1.4 | 0.357 | 0.697 | 2.1 | 2.63 | 1.62 | 4.27 | 0.00124 | 0.0324 | lm.nb | Inflammatory Signaling, Innate Immune Response, NF-kB | NS\_MM\_NEUROINFLAM\_V1.0 | Ddx58 | mRNA | 0 |  | NF-kB;Innate Immune Response;Inflammatory Signaling | mmu04064;mmu04622;mmu04623;mmu05160;mmu05161;mmu05162;mmu05164;mmu05168;mmu05169 |  | Ddx58 | slateblue2 | 16 | 3.92156862745098 |
  | Ifnar1-mRNA | 0.42 | 0.116 | 0.193 | 0.647 | 1.34 | 1.14 | 1.57 | 0.00224 | 0.0534 | loglinear | Cytokine Signaling, Growth Factor Signaling, Inflammatory Signaling, Innate Immune Response | NS\_MM\_NEUROINFLAM\_V1.0 | Ifnar1 | mRNA | 0 |  | Innate Immune Response;Inflammatory Signaling;Growth Factor Signaling;Cytokine Signaling | mmu04060;mmu04151;mmu04380;mmu04620;mmu04630;mmu04650;mmu05160;mmu05161;mmu05162;mmu05164;mmu05168 |  | Ifnar1 | slateblue3 | 16 | 3.62068965517241 |
  | Cx3cl1-mRNA | -0.594 | 0.175 | -0.937 | -0.251 | 0.662 | 0.522 | 0.84 | 0.0037 | 0.0797 | lm.nb | Cytokine Signaling, Inflammatory Signaling, Innate Immune Response | NS\_MM\_NEUROINFLAM\_V1.0 | Cx3cl1 | mRNA | 0 |  | Innate Immune Response;Inflammatory Signaling;Cytokine Signaling | mmu04060;mmu04062;mmu04668 |  | Cx3cl1 | slateblue3 | 16 | -3.39428571428571 |
  | Rela-mRNA | 0.505 | 0.157 | 0.197 | 0.812 | 1.42 | 1.15 | 1.76 | 0.00535 | 0.109 | lm.nb | Adaptive Immune Response, Apoptosis, Cellular Stress, Cytokine Signaling, Growth Factor Signaling, Inflammatory Signaling, Innate Immune Response, NF-kB | NS\_MM\_NEUROINFLAM\_V1.0 | Rela | mRNA | 0 |  | NF-kB;Innate Immune Response;Inflammatory Signaling;Growth Factor Signaling;Cytokine Signaling;Cellular Stress;Apoptosis;Adaptive Immune Response | mmu04010;mmu04014;mmu04024;mmu04062;mmu04064;mmu04066;mmu04071;mmu04151;mmu04210;mmu04211;mmu04380;mmu04620;mmu04621;mmu04622;mmu04623;mmu04660;mmu04662;mmu04668;mmu04722;mmu04917;mmu04920;mmu04931;mmu04932;mmu04933;mmu05030;mmu05132;mmu05133;mmu05134;mmu05140;mmu05142;mmu05145;mmu05146;mmu05152;mmu05160;mmu05161;mmu05162;mmu05164;mmu05166;mmu05168;mmu05169;mmu05200;mmu05202;mmu05203;mmu05212;mmu05215;mmu05220;mmu05221;mmu05222;mmu05321 |  | Rela | slateblue4 | 16 | 3.21656050955414 |

  ##### DE Results - Inflammatory Signaling genes - DPI: 3 vs.CTRL

  Table displaying each sample's global significance scores and directed global significance scores as defined in the heatmaps above. The global significance score is calculated as the square root of the mean squared t-statistic for the genes in a gene set, with t-statistics coming from the linear regression underlying our differential expression analysis. The directed global significance score is calculated as the square root of the mean signed squared t-statistic for the genes in a gene set, with t-statistics coming from the linear regression underlying our differential expression analysis.

DPI:
- DPI: differential expression in 1 vs. baseline of CTRL
- DPI: differential expression in 2 vs. baseline of CTRL
- DPI: differential expression in 3 vs. baseline of CTRL

- **Volcano Plot: DPI: 1 vs.CTRL  
  More Plot Information**


  ##### Volcano Plot: DPI: 1 vs.CTRL

  Volcano plot displaying each gene's -log10(p-value) and log2 fold change for the selected covariate. Highly statistically significant genes fall at the top of the plot, and highly differentially expressed genes fall to either side. Genes within the selected gene set are highlighted in orange. Horizontal lines indicate various False Discovery Rate (FDR) thresholds.
- **DE Results - Innate Immune Response genes - DPI: 1 vs.CTRL  
  More Plot Information  Download CSV Data**

  | Probe Label | Log2 fold change | std error (log2) | Lower confidence limit (log2) | Upper confidence limit (log2) | Linear fold change | Lower confidence limit (linear) | Upper confidence limit (linear) | P-value | BY.p.value | method | Gene.sets | Codeset.Name | Probe.Label | Analyte.Type | Is.Control | Control.Type | Probe.Annotation | KEGG.Pathways | Cell.Type | Official.Gene.Name | volcanocol | volcanopch | tstats.all |
  | --- | --- | --- | --- | --- | --- | --- | --- | --- | --- | --- | --- | --- | --- | --- | --- | --- | --- | --- | --- | --- | --- | --- | --- |
  | Dusp7-mRNA | -0.378 | 0.0611 | -0.497 | -0.258 | 0.77 | 0.708 | 0.836 | 1.32e-05 | 0.0093 | loglinear | Adaptive Immune Response, Angiogenesis, Cytokine Signaling, Growth Factor Signaling, Innate Immune Response, Insulin Signaling, Microglia Function | NS\_MM\_NEUROINFLAM\_V1.0 | Dusp7 | mRNA | 0 |  | Microglia Function;Insulin Signaling;Innate Immune Response;Growth Factor Signaling;Cytokine Signaling;Angiogenesis;Adaptive Immune Response | mmu04010 |  | Dusp7 | slateblue2 | 16 | -6.18657937806874 |
  | Fos-mRNA | -1.75 | 0.299 | -2.34 | -1.17 | 0.297 | 0.198 | 0.446 | 2.43e-05 | 0.0143 | lm.nb | Adaptive Immune Response, Cellular Stress, Growth Factor Signaling, Innate Immune Response, Neurons and Neurotransmission | NS\_MM\_NEUROINFLAM\_V1.0 | Fos | mRNA | 0 |  | Neurons and Neurotransmission;Innate Immune Response;Growth Factor Signaling;Cellular Stress;Adaptive Immune Response | mmu04010;mmu04024;mmu04380;mmu04620;mmu04660;mmu04662;mmu04668;mmu04713;mmu04725;mmu04728;mmu04915;mmu04917;mmu04921;mmu05031;mmu05132;mmu05133;mmu05140;mmu05142;mmu05161;mmu05166;mmu05168;mmu05200;mmu05210;mmu05231;mmu05323 |  | Fos | slateblue2 | 16 | -5.85284280936455 |
  | Pak1-mRNA | -0.284 | 0.0554 | -0.393 | -0.176 | 0.821 | 0.762 | 0.885 | 1e-04 | 0.0321 | loglinear | Adaptive Immune Response, Angiogenesis, Growth Factor Signaling, Innate Immune Response, Microglia Function | NS\_MM\_NEUROINFLAM\_V1.0 | Pak1 | mRNA | 0 |  | Microglia Function;Innate Immune Response;Growth Factor Signaling;Angiogenesis;Adaptive Immune Response | mmu04010;mmu04012;mmu04014;mmu04024;mmu04062;mmu04360;mmu04510;mmu04650;mmu04660;mmu04666;mmu04810;mmu05205;mmu05211 |  | Pak1 | slateblue2 | 16 | -5.12635379061372 |
  | Ppp3r1-mRNA | -0.28 | 0.0577 | -0.393 | -0.167 | 0.823 | 0.761 | 0.891 | 0.000175 | 0.0442 | loglinear | Adaptive Immune Response, Apoptosis, Growth Factor Signaling, Innate Immune Response, Wnt | NS\_MM\_NEUROINFLAM\_V1.0 | Ppp3r1 | mRNA | 0 |  | Wnt;Innate Immune Response;Growth Factor Signaling;Apoptosis;Adaptive Immune Response | mmu04010;mmu04020;mmu04022;mmu04114;mmu04210;mmu04310;mmu04360;mmu04370;mmu04380;mmu04650;mmu04660;mmu04662;mmu04720;mmu04724;mmu04921;mmu04922;mmu04924;mmu05010;mmu05014;mmu05031;mmu05152;mmu05166 |  | Ppp3r1 | slateblue2 | 16 | -4.8526863084922 |
  | Lgmn-mRNA | 0.369 | 0.0832 | 0.206 | 0.532 | 1.29 | 1.15 | 1.45 | 0.000416 | 0.0611 | loglinear | Adaptive Immune Response, Innate Immune Response | NS\_MM\_NEUROINFLAM\_V1.0 | Lgmn | mRNA | 0 |  | Innate Immune Response;Adaptive Immune Response | mmu04142;mmu04612 |  | Lgmn | slateblue3 | 16 | 4.43509615384615 |
  | Ppp3cb-mRNA | -0.411 | 0.0979 | -0.602 | -0.219 | 0.752 | 0.659 | 0.859 | 0.000686 | 0.0806 | lm.nb | Adaptive Immune Response, Apoptosis, Growth Factor Signaling, Innate Immune Response, Wnt | NS\_MM\_NEUROINFLAM\_V1.0 | Ppp3cb | mRNA | 0 |  | Wnt;Innate Immune Response;Growth Factor Signaling;Apoptosis;Adaptive Immune Response | mmu04010;mmu04020;mmu04022;mmu04114;mmu04210;mmu04310;mmu04360;mmu04370;mmu04380;mmu04650;mmu04660;mmu04662;mmu04720;mmu04724;mmu04728;mmu04921;mmu04922;mmu04924;mmu05010;mmu05014;mmu05031;mmu05152;mmu05166 |  | Ppp3cb | slateblue3 | 16 | -4.19816138917262 |
  | Braf-mRNA | -0.641 | 0.154 | -0.943 | -0.339 | 0.641 | 0.52 | 0.791 | 0.000739 | 0.0815 | lm.nb | Adaptive Immune Response, Angiogenesis, Apoptosis, Cytokine Signaling, Growth Factor Signaling, Innate Immune Response, Insulin Signaling, Neurons and Neurotransmission | NS\_MM\_NEUROINFLAM\_V1.0 | Braf | mRNA | 0 |  | Neurons and Neurotransmission;Insulin Signaling;Innate Immune Response;Growth Factor Signaling;Cytokine Signaling;Apoptosis;Angiogenesis;Adaptive Immune Response | mmu04010;mmu04012;mmu04015;mmu04024;mmu04062;mmu04068;mmu04150;mmu04270;mmu04510;mmu04650;mmu04720;mmu04722;mmu04726;mmu04730;mmu04810;mmu04910;mmu04914;mmu05034;mmu05160;mmu05200;mmu05205;mmu05210;mmu05211;mmu05212;mmu05213;mmu05214;mmu05215;mmu05216;mmu05218;mmu05219;mmu05220;mmu05221;mmu05223 |  | Braf | slateblue3 | 16 | -4.16233766233766 |
  | Mapk10-mRNA | -0.539 | 0.138 | -0.81 | -0.269 | 0.688 | 0.57 | 0.83 | 0.00126 | 0.111 | lm.nb | Adaptive Immune Response, Apoptosis, Autophagy, Cellular Stress, Growth Factor Signaling, Innate Immune Response, Wnt | NS\_MM\_NEUROINFLAM\_V1.0 | Mapk10 | mRNA | 0 |  | Wnt;Innate Immune Response;Growth Factor Signaling;Cellular Stress;Autophagy;Apoptosis;Adaptive Immune Response | mmu04010;mmu04012;mmu04014;mmu04024;mmu04068;mmu04071;mmu04141;mmu04310;mmu04380;mmu04510;mmu04620;mmu04621;mmu04622;mmu04664;mmu04668;mmu04722;mmu04723;mmu04728;mmu04750;mmu04910;mmu04912;mmu04914;mmu04917;mmu04920;mmu04930;mmu04931;mmu04932;mmu04933;mmu05132;mmu05133;mmu05142;mmu05145;mmu05152;mmu05160;mmu05161;mmu05164;mmu05168;mmu05169;mmu05200;mmu05210;mmu05212;mmu05231 |  | Mapk10 | slateblue4 | 16 | -3.90579710144928 |
  | Rac1-mRNA | -0.928 | 0.256 | -1.43 | -0.426 | 0.525 | 0.371 | 0.744 | 0.00229 | 0.149 | lm.nb | Adaptive Immune Response, Angiogenesis, Autophagy, Growth Factor Signaling, Innate Immune Response, Neurons and Neurotransmission, Wnt | NS\_MM\_NEUROINFLAM\_V1.0 | Rac1 | mRNA | 0 |  | Wnt;Neurons and Neurotransmission;Innate Immune Response;Growth Factor Signaling;Autophagy;Angiogenesis;Adaptive Immune Response | mmu04010;mmu04014;mmu04015;mmu04024;mmu04062;mmu04071;mmu04145;mmu04151;mmu04310;mmu04360;mmu04370;mmu04380;mmu04510;mmu04520;mmu04620;mmu04650;mmu04662;mmu04664;mmu04666;mmu04670;mmu04722;mmu04810;mmu04932;mmu04933;mmu05014;mmu05100;mmu05132;mmu05200;mmu05203;mmu05205;mmu05210;mmu05211;mmu05212;mmu05231;mmu05416 |  | Rac1 | slateblue4 | 16 | -3.625 |
  | Mmp14-mRNA | -1.57 | 0.459 | -2.47 | -0.668 | 0.338 | 0.181 | 0.629 | 0.00354 | 0.184 | lm.nb | Cellular Stress, Innate Immune Response, Matrix Remodeling, Microglia Function | NS\_MM\_NEUROINFLAM\_V1.0 | Mmp14 | mRNA | 0 |  | Microglia Function;Matrix Remodeling;Innate Immune Response;Cellular Stress | mmu04668;mmu04912 |  | Mmp14 | slateblue4 | 16 | -3.42047930283224 |
  | Xiap-mRNA | -0.529 | 0.155 | -0.833 | -0.225 | 0.693 | 0.561 | 0.856 | 0.00359 | 0.184 | lm.nb | Apoptosis, Innate Immune Response, NF-kB, Wnt | NS\_MM\_NEUROINFLAM\_V1.0 | Xiap | mRNA | 0 |  | Wnt;NF-kB;Innate Immune Response;Apoptosis | mmu04064;mmu04120;mmu04210;mmu04510;mmu05145;mmu05166;mmu05200;mmu05222 |  | Xiap | slateblue4 | 16 | -3.41290322580645 |
  | Casp9-mRNA | -1.44 | 0.433 | -2.29 | -0.59 | 0.369 | 0.205 | 0.664 | 0.00432 | 0.2 | lm.nb | Adaptive Immune Response, Apoptosis, DNA Damage, Growth Factor Signaling, Innate Immune Response | NS\_MM\_NEUROINFLAM\_V1.0 | Casp9 | mRNA | 0 |  | Innate Immune Response;Growth Factor Signaling;DNA Damage;Apoptosis;Adaptive Immune Response | mmu04115;mmu04151;mmu04210;mmu04370;mmu04919;mmu05010;mmu05012;mmu05014;mmu05016;mmu05134;mmu05145;mmu05152;mmu05161;mmu05164;mmu05200;mmu05210;mmu05212;mmu05213;mmu05215;mmu05222;mmu05223;mmu05416 |  | Casp9 | slateblue4 | 16 | -3.3256351039261 |
  | Map2k4-mRNA | -0.433 | 0.133 | -0.693 | -0.173 | 0.741 | 0.619 | 0.887 | 0.00484 | 0.206 | lm.nb | Adaptive Immune Response, Apoptosis, Cellular Stress, Growth Factor Signaling, Innate Immune Response | NS\_MM\_NEUROINFLAM\_V1.0 | Map2k4 | mRNA | 0 |  | Innate Immune Response;Growth Factor Signaling;Cellular Stress;Apoptosis;Adaptive Immune Response | mmu04010;mmu04012;mmu04620;mmu04664;mmu04668;mmu04912;mmu05142;mmu05161;mmu05164;mmu05166;mmu05169 |  | Map2k4 | slateblue4 | 16 | -3.25563909774436 |
  | Bag4-mRNA | -0.576 | 0.178 | -0.925 | -0.227 | 0.671 | 0.527 | 0.855 | 0.00523 | 0.215 | lm.nb | Apoptosis, Cellular Stress, Innate Immune Response | NS\_MM\_NEUROINFLAM\_V1.0 | Bag4 | mRNA | 0 |  | Innate Immune Response;Cellular Stress;Apoptosis | mmu04668 |  | Bag4 | slateblue4 | 16 | -3.23595505617978 |
  | Traf6-mRNA | 0.546 | 0.169 | 0.215 | 0.877 | 1.46 | 1.16 | 1.84 | 0.00523 | 0.215 | lm.nb | Adaptive Immune Response, Apoptosis, Autophagy, Cytokine Signaling, Growth Factor Signaling, Innate Immune Response, NF-kB | NS\_MM\_NEUROINFLAM\_V1.0 | Traf6 | mRNA | 0 |  | NF-kB;Innate Immune Response;Growth Factor Signaling;Cytokine Signaling;Autophagy;Apoptosis;Adaptive Immune Response | mmu04010;mmu04064;mmu04120;mmu04144;mmu04380;mmu04620;mmu04621;mmu04622;mmu04722;mmu05133;mmu05140;mmu05142;mmu05145;mmu05152;mmu05160;mmu05162;mmu05168;mmu05169;mmu05200;mmu05222 |  | Traf6 | slateblue4 | 16 | 3.23076923076923 |
  | Map2k1-mRNA | -1.03 | 0.32 | -1.65 | -0.399 | 0.491 | 0.318 | 0.759 | 0.00553 | 0.224 | lm.nb | Adaptive Immune Response, Angiogenesis, Autophagy, Cytokine Signaling, Growth Factor Signaling, Innate Immune Response, Insulin Signaling, Microglia Function | NS\_MM\_NEUROINFLAM\_V1.0 | Map2k1 | mRNA | 0 |  | Microglia Function;Insulin Signaling;Innate Immune Response;Growth Factor Signaling;Cytokine Signaling;Autophagy;Angiogenesis;Adaptive Immune Response | mmu04010;mmu04012;mmu04014;mmu04015;mmu04022;mmu04024;mmu04062;mmu04066;mmu04068;mmu04071;mmu04072;mmu04114;mmu04151;mmu04270;mmu04370;mmu04380;mmu04510;mmu04540;mmu04550;mmu04620;mmu04650;mmu04660;mmu04662;mmu04664;mmu04666;mmu04668;mmu04720;mmu04722;mmu04725;mmu04726;mmu04730;mmu04810;mmu04910;mmu04912;mmu04914;mmu04915;mmu04916;mmu04917;mmu04919;mmu04921;mmu05020;mmu05034;mmu05161;mmu05164;mmu05200;mmu05205;mmu05206;mmu05210;mmu05211;mmu05212;mmu05213;mmu05214;mmu05215;mmu05216;mmu05218;mmu05219;mmu05220;mmu05221;mmu05223;mmu05230;mmu05231 |  | Map2k1 | slateblue4 | 16 | -3.21875 |
  | Mapk14-mRNA | -1.06 | 0.362 | -1.77 | -0.355 | 0.478 | 0.293 | 0.782 | 0.00961 | 0.3 | lm.nb | Angiogenesis, Autophagy, Cellular Stress, Growth Factor Signaling, Innate Immune Response | NS\_MM\_NEUROINFLAM\_V1.0 | Mapk14 | mRNA | 0 |  | Innate Immune Response;Growth Factor Signaling;Cellular Stress;Autophagy;Angiogenesis | mmu04010;mmu04015;mmu04068;mmu04071;mmu04261;mmu04370;mmu04380;mmu04550;mmu04611;mmu04620;mmu04621;mmu04622;mmu04660;mmu04664;mmu04668;mmu04670;mmu04722;mmu04723;mmu04728;mmu04750;mmu04912;mmu04914;mmu04917;mmu04933;mmu05014;mmu05132;mmu05133;mmu05140;mmu05142;mmu05145;mmu05152;mmu05160;mmu05164;mmu05169;mmu05205 |  | Mapk14 | slateblue4 | 16 | -2.92817679558011 |
  | Pik3r2-mRNA | -0.223 | 0.0793 | -0.378 | -0.0676 | 0.857 | 0.769 | 0.954 | 0.0125 | 0.357 | loglinear | Adaptive Immune Response, Angiogenesis, Apoptosis, Autophagy, Cytokine Signaling, Growth Factor Signaling, Innate Immune Response, Insulin Signaling | NS\_MM\_NEUROINFLAM\_V1.0 | Pik3r2 | mRNA | 0 |  | Insulin Signaling;Innate Immune Response;Growth Factor Signaling;Cytokine Signaling;Autophagy;Apoptosis;Angiogenesis;Adaptive Immune Response | mmu04012;mmu04014;mmu04015;mmu04022;mmu04024;mmu04062;mmu04066;mmu04068;mmu04070;mmu04071;mmu04072;mmu04150;mmu04151;mmu04152;mmu04210;mmu04211;mmu04213;mmu04261;mmu04370;mmu04380;mmu04510;mmu04550;mmu04611;mmu04620;mmu04630;mmu04650;mmu04660;mmu04662;mmu04664;mmu04666;mmu04668;mmu04670;mmu04722;mmu04725;mmu04750;mmu04810;mmu04910;mmu04914;mmu04915;mmu04917;mmu04919;mmu04921;mmu04923;mmu04930;mmu04931;mmu04932;mmu04933;mmu04960;mmu04973;mmu05100;mmu05142;mmu05145;mmu05146;mmu05160;mmu05161;mmu05162;mmu05164;mmu05166;mmu05169;mmu05200;mmu05203;mmu05205;mmu05210;mmu05211;mmu05212;mmu05213;mmu05214;mmu05215;mmu05218;mmu05220;mmu05221;mmu05222;mmu05223;mmu05230;mmu05231 |  | Pik3r2 | slateblue4 | 16 | -2.81210592686003 |
  | Irak1-mRNA | 0.531 | 0.191 | 0.157 | 0.904 | 1.44 | 1.11 | 1.87 | 0.0133 | 0.367 | lm.nb | Apoptosis, Cytokine Signaling, Growth Factor Signaling, Innate Immune Response, NF-kB | NS\_MM\_NEUROINFLAM\_V1.0 | Irak1 | mRNA | 0 |  | NF-kB;Innate Immune Response;Growth Factor Signaling;Cytokine Signaling;Apoptosis | mmu04064;mmu04210;mmu04620;mmu04722;mmu05133;mmu05140;mmu05142;mmu05145;mmu05152;mmu05162;mmu05169 |  | Irak1 | slateblue4 | 16 | 2.78010471204188 |
  | Mavs-mRNA | -1.29 | 0.465 | -2.2 | -0.374 | 0.41 | 0.218 | 0.772 | 0.0139 | 0.377 | lm.nb | Inflammatory Signaling, Innate Immune Response | NS\_MM\_NEUROINFLAM\_V1.0 | Mavs | mRNA | 0 |  | Innate Immune Response;Inflammatory Signaling | mmu04622;mmu04623;mmu05160;mmu05161;mmu05162;mmu05164;mmu05168 |  | Mavs | slateblue4 | 16 | -2.7741935483871 |

  ##### DE Results - Innate Immune Response genes - DPI: 1 vs.CTRL

  Table displaying each sample's global significance scores and directed global significance scores as defined in the heatmaps above. The global significance score is calculated as the square root of the mean squared t-statistic for the genes in a gene set, with t-statistics coming from the linear regression underlying our differential expression analysis. The directed global significance score is calculated as the square root of the mean signed squared t-statistic for the genes in a gene set, with t-statistics coming from the linear regression underlying our differential expression analysis.

- **Volcano Plot: DPI: 2 vs.CTRL  
  More Plot Information**


  ##### Volcano Plot: DPI: 2 vs.CTRL

  Volcano plot displaying each gene's -log10(p-value) and log2 fold change for the selected covariate. Highly statistically significant genes fall at the top of the plot, and highly differentially expressed genes fall to either side. Genes within the selected gene set are highlighted in orange. Horizontal lines indicate various False Discovery Rate (FDR) thresholds.
- **DE Results - Innate Immune Response genes - DPI: 2 vs.CTRL  
  More Plot Information  Download CSV Data**

  | Probe Label | Log2 fold change | std error (log2) | Lower confidence limit (log2) | Upper confidence limit (log2) | Linear fold change | Lower confidence limit (linear) | Upper confidence limit (linear) | P-value | BY.p.value | method | Gene.sets | Codeset.Name | Probe.Label | Analyte.Type | Is.Control | Control.Type | Probe.Annotation | KEGG.Pathways | Cell.Type | Official.Gene.Name | volcanocol | volcanopch | tstats.all |
  | --- | --- | --- | --- | --- | --- | --- | --- | --- | --- | --- | --- | --- | --- | --- | --- | --- | --- | --- | --- | --- | --- | --- | --- |
  | Nfkbia-mRNA | 1.86 | 0.228 | 1.41 | 2.31 | 3.63 | 2.67 | 4.95 | 4.26e-07 | 9.99e-05 | lm.nb | Adaptive Immune Response, Apoptosis, Growth Factor Signaling, Inflammatory Signaling, Innate Immune Response, NF-kB | NS\_MM\_NEUROINFLAM\_V1.0 | Nfkbia | mRNA | 0 |  | NF-kB;Innate Immune Response;Inflammatory Signaling;Growth Factor Signaling;Apoptosis;Adaptive Immune Response | mmu04024;mmu04062;mmu04064;mmu04210;mmu04380;mmu04620;mmu04621;mmu04622;mmu04623;mmu04660;mmu04662;mmu04668;mmu04722;mmu04920;mmu04931;mmu05134;mmu05140;mmu05142;mmu05145;mmu05160;mmu05161;mmu05162;mmu05164;mmu05166;mmu05168;mmu05169;mmu05200;mmu05203;mmu05215;mmu05220;mmu05222 |  | Nfkbia | slateblue1 | 16 | 8.15789473684211 |
  | Dusp7-mRNA | -0.496 | 0.0611 | -0.616 | -0.377 | 0.709 | 0.652 | 0.77 | 4.53e-07 | 9.99e-05 | loglinear | Adaptive Immune Response, Angiogenesis, Cytokine Signaling, Growth Factor Signaling, Innate Immune Response, Insulin Signaling, Microglia Function | NS\_MM\_NEUROINFLAM\_V1.0 | Dusp7 | mRNA | 0 |  | Microglia Function;Insulin Signaling;Innate Immune Response;Growth Factor Signaling;Cytokine Signaling;Angiogenesis;Adaptive Immune Response | mmu04010 |  | Dusp7 | slateblue1 | 16 | -8.11783960720131 |
  | C1qc-mRNA | 1.16 | 0.156 | 0.85 | 1.46 | 2.23 | 1.8 | 2.75 | 1.49e-06 | 0.000239 | lm.nb | Innate Immune Response | NS\_MM\_NEUROINFLAM\_V1.0 | C1qc | mRNA | 0 |  | Innate Immune Response | mmu04610;mmu05020;mmu05133;mmu05142;mmu05150;mmu05322 |  | C1qc | slateblue1 | 16 | 7.43589743589744 |
  | Fos-mRNA | -2.04 | 0.3 | -2.62 | -1.45 | 0.244 | 0.162 | 0.367 | 4.41e-06 | 0.000585 | lm.nb | Adaptive Immune Response, Cellular Stress, Growth Factor Signaling, Innate Immune Response, Neurons and Neurotransmission | NS\_MM\_NEUROINFLAM\_V1.0 | Fos | mRNA | 0 |  | Neurons and Neurotransmission;Innate Immune Response;Growth Factor Signaling;Cellular Stress;Adaptive Immune Response | mmu04010;mmu04024;mmu04380;mmu04620;mmu04660;mmu04662;mmu04668;mmu04713;mmu04725;mmu04728;mmu04915;mmu04917;mmu04921;mmu05031;mmu05132;mmu05133;mmu05140;mmu05142;mmu05161;mmu05166;mmu05168;mmu05200;mmu05210;mmu05231;mmu05323 |  | Fos | slateblue1 | 16 | -6.8 |
  | Ppp3r1-mRNA | -0.334 | 0.0577 | -0.447 | -0.221 | 0.793 | 0.734 | 0.858 | 2.79e-05 | 0.00214 | loglinear | Adaptive Immune Response, Apoptosis, Growth Factor Signaling, Innate Immune Response, Wnt | NS\_MM\_NEUROINFLAM\_V1.0 | Ppp3r1 | mRNA | 0 |  | Wnt;Innate Immune Response;Growth Factor Signaling;Apoptosis;Adaptive Immune Response | mmu04010;mmu04020;mmu04022;mmu04114;mmu04210;mmu04310;mmu04360;mmu04370;mmu04380;mmu04650;mmu04660;mmu04662;mmu04720;mmu04724;mmu04921;mmu04922;mmu04924;mmu05010;mmu05014;mmu05031;mmu05152;mmu05166 |  | Ppp3r1 | slateblue1 | 16 | -5.78856152512998 |
  | Fcgr3-mRNA | 2.05 | 0.36 | 1.34 | 2.75 | 4.14 | 2.54 | 6.74 | 3.32e-05 | 0.0024 | lm.nb | Adaptive Immune Response, Autophagy, Inflammatory Signaling, Innate Immune Response | NS\_MM\_NEUROINFLAM\_V1.0 | Fcgr3 | mRNA | 0 |  | Innate Immune Response;Inflammatory Signaling;Autophagy;Adaptive Immune Response | mmu04145;mmu04380;mmu05140;mmu05150;mmu05152 |  | Fcgr3 | slateblue1 | 16 | 5.69444444444444 |
  | Ppp3cb-mRNA | -0.554 | 0.098 | -0.747 | -0.362 | 0.681 | 0.596 | 0.778 | 3.56e-05 | 0.00246 | lm.nb | Adaptive Immune Response, Apoptosis, Growth Factor Signaling, Innate Immune Response, Wnt | NS\_MM\_NEUROINFLAM\_V1.0 | Ppp3cb | mRNA | 0 |  | Wnt;Innate Immune Response;Growth Factor Signaling;Apoptosis;Adaptive Immune Response | mmu04010;mmu04020;mmu04022;mmu04114;mmu04210;mmu04310;mmu04360;mmu04370;mmu04380;mmu04650;mmu04660;mmu04662;mmu04720;mmu04724;mmu04728;mmu04921;mmu04922;mmu04924;mmu05010;mmu05014;mmu05031;mmu05152;mmu05166 |  | Ppp3cb | slateblue1 | 16 | -5.6530612244898 |
  | Bcl2l1-mRNA | 0.407 | 0.0734 | 0.263 | 0.551 | 1.33 | 1.2 | 1.46 | 4.51e-05 | 0.00306 | loglinear | Apoptosis, Autophagy, Cytokine Signaling, Growth Factor Signaling, Innate Immune Response, NF-kB | NS\_MM\_NEUROINFLAM\_V1.0 | Bcl2l1 | mRNA | 0 |  | NF-kB;Innate Immune Response;Growth Factor Signaling;Cytokine Signaling;Autophagy;Apoptosis | mmu04014;mmu04064;mmu04151;mmu04210;mmu04630;mmu05014;mmu05145;mmu05166;mmu05200;mmu05202;mmu05212;mmu05220;mmu05222 |  | Bcl2l1 | slateblue1 | 16 | 5.54495912806539 |
  | C1qa-mRNA | 0.944 | 0.187 | 0.578 | 1.31 | 1.92 | 1.49 | 2.48 | 0.000119 | 0.00609 | lm.nb | Innate Immune Response | NS\_MM\_NEUROINFLAM\_V1.0 | C1qa | mRNA | 0 |  | Innate Immune Response | mmu04610;mmu05020;mmu05133;mmu05142;mmu05150;mmu05322 |  | C1qa | slateblue1 | 16 | 5.04812834224599 |
  | Irf1-mRNA | 1.63 | 0.335 | 0.973 | 2.29 | 3.09 | 1.96 | 4.88 | 0.000172 | 0.0082 | lm.nb | Apoptosis, Inflammatory Signaling, Innate Immune Response | NS\_MM\_NEUROINFLAM\_V1.0 | Irf1 | mRNA | 0 |  | Innate Immune Response;Inflammatory Signaling;Apoptosis | mmu04917;mmu05133;mmu05160 |  | Irf1 | slateblue1 | 16 | 4.86567164179104 |
  | Mapk10-mRNA | -0.673 | 0.138 | -0.943 | -0.402 | 0.627 | 0.52 | 0.757 | 0.000172 | 0.0082 | lm.nb | Adaptive Immune Response, Apoptosis, Autophagy, Cellular Stress, Growth Factor Signaling, Innate Immune Response, Wnt | NS\_MM\_NEUROINFLAM\_V1.0 | Mapk10 | mRNA | 0 |  | Wnt;Innate Immune Response;Growth Factor Signaling;Cellular Stress;Autophagy;Apoptosis;Adaptive Immune Response | mmu04010;mmu04012;mmu04014;mmu04024;mmu04068;mmu04071;mmu04141;mmu04310;mmu04380;mmu04510;mmu04620;mmu04621;mmu04622;mmu04664;mmu04668;mmu04722;mmu04723;mmu04728;mmu04750;mmu04910;mmu04912;mmu04914;mmu04917;mmu04920;mmu04930;mmu04931;mmu04932;mmu04933;mmu05132;mmu05133;mmu05142;mmu05145;mmu05152;mmu05160;mmu05161;mmu05164;mmu05168;mmu05169;mmu05200;mmu05210;mmu05212;mmu05231 |  | Mapk10 | slateblue1 | 16 | -4.8768115942029 |
  | Pak1-mRNA | -0.263 | 0.0554 | -0.372 | -0.155 | 0.833 | 0.773 | 0.898 | 0.000215 | 0.0092 | loglinear | Adaptive Immune Response, Angiogenesis, Growth Factor Signaling, Innate Immune Response, Microglia Function | NS\_MM\_NEUROINFLAM\_V1.0 | Pak1 | mRNA | 0 |  | Microglia Function;Innate Immune Response;Growth Factor Signaling;Angiogenesis;Adaptive Immune Response | mmu04010;mmu04012;mmu04014;mmu04024;mmu04062;mmu04360;mmu04510;mmu04650;mmu04660;mmu04666;mmu04810;mmu05205;mmu05211 |  | Pak1 | slateblue1 | 16 | -4.74729241877256 |
  | Traf6-mRNA | 0.756 | 0.167 | 0.428 | 1.08 | 1.69 | 1.35 | 2.12 | 0.000353 | 0.0135 | lm.nb | Adaptive Immune Response, Apoptosis, Autophagy, Cytokine Signaling, Growth Factor Signaling, Innate Immune Response, NF-kB | NS\_MM\_NEUROINFLAM\_V1.0 | Traf6 | mRNA | 0 |  | NF-kB;Innate Immune Response;Growth Factor Signaling;Cytokine Signaling;Autophagy;Apoptosis;Adaptive Immune Response | mmu04010;mmu04064;mmu04120;mmu04144;mmu04380;mmu04620;mmu04621;mmu04622;mmu04722;mmu05133;mmu05140;mmu05142;mmu05145;mmu05152;mmu05160;mmu05162;mmu05168;mmu05169;mmu05200;mmu05222 |  | Traf6 | slateblue2 | 16 | 4.52694610778443 |
  | Mef2c-mRNA | -0.355 | 0.0801 | -0.512 | -0.198 | 0.782 | 0.701 | 0.872 | 0.000422 | 0.015 | loglinear | Growth Factor Signaling, Innate Immune Response, Microglia Function | NS\_MM\_NEUROINFLAM\_V1.0 | Mef2c | mRNA | 0 |  | Microglia Function;Innate Immune Response;Growth Factor Signaling | mmu04010;mmu04022;mmu04921;mmu05202 |  | Mef2c | slateblue2 | 16 | -4.43196004993758 |
  | Pik3r2-mRNA | -0.338 | 0.0793 | -0.494 | -0.183 | 0.791 | 0.71 | 0.881 | 0.000587 | 0.0197 | loglinear | Adaptive Immune Response, Angiogenesis, Apoptosis, Autophagy, Cytokine Signaling, Growth Factor Signaling, Innate Immune Response, Insulin Signaling | NS\_MM\_NEUROINFLAM\_V1.0 | Pik3r2 | mRNA | 0 |  | Insulin Signaling;Innate Immune Response;Growth Factor Signaling;Cytokine Signaling;Autophagy;Apoptosis;Angiogenesis;Adaptive Immune Response | mmu04012;mmu04014;mmu04015;mmu04022;mmu04024;mmu04062;mmu04066;mmu04068;mmu04070;mmu04071;mmu04072;mmu04150;mmu04151;mmu04152;mmu04210;mmu04211;mmu04213;mmu04261;mmu04370;mmu04380;mmu04510;mmu04550;mmu04611;mmu04620;mmu04630;mmu04650;mmu04660;mmu04662;mmu04664;mmu04666;mmu04668;mmu04670;mmu04722;mmu04725;mmu04750;mmu04810;mmu04910;mmu04914;mmu04915;mmu04917;mmu04919;mmu04921;mmu04923;mmu04930;mmu04931;mmu04932;mmu04933;mmu04960;mmu04973;mmu05100;mmu05142;mmu05145;mmu05146;mmu05160;mmu05161;mmu05162;mmu05164;mmu05166;mmu05169;mmu05200;mmu05203;mmu05205;mmu05210;mmu05211;mmu05212;mmu05213;mmu05214;mmu05215;mmu05218;mmu05220;mmu05221;mmu05222;mmu05223;mmu05230;mmu05231 |  | Pik3r2 | slateblue2 | 16 | -4.26229508196721 |
  | Chuk-mRNA | -0.331 | 0.0787 | -0.485 | -0.176 | 0.795 | 0.715 | 0.885 | 0.000678 | 0.0218 | loglinear | Adaptive Immune Response, Apoptosis, Cytokine Signaling, Growth Factor Signaling, Inflammatory Signaling, Innate Immune Response, NF-kB | NS\_MM\_NEUROINFLAM\_V1.0 | Chuk | mRNA | 0 |  | NF-kB;Innate Immune Response;Inflammatory Signaling;Growth Factor Signaling;Cytokine Signaling;Apoptosis;Adaptive Immune Response | mmu04010;mmu04014;mmu04062;mmu04064;mmu04068;mmu04151;mmu04210;mmu04380;mmu04620;mmu04621;mmu04622;mmu04623;mmu04660;mmu04662;mmu04668;mmu04920;mmu05142;mmu05145;mmu05160;mmu05161;mmu05162;mmu05166;mmu05168;mmu05169;mmu05200;mmu05212;mmu05215;mmu05220;mmu05221;mmu05222 |  | Chuk | slateblue2 | 16 | -4.20584498094028 |
  | Akt2-mRNA | -0.592 | 0.142 | -0.869 | -0.314 | 0.664 | 0.547 | 0.804 | 0.000713 | 0.022 | loglinear | Adaptive Immune Response, Angiogenesis, Apoptosis, Autophagy, Carbohydrate Metabolism, Cytokine Signaling, Growth Factor Signaling, Innate Immune Response, Insulin Signaling, Neurons and Neurotransmission, Wnt | NS\_MM\_NEUROINFLAM\_V1.0 | Akt2 | mRNA | 0 |  | Wnt;Neurons and Neurotransmission;Insulin Signaling;Innate Immune Response;Growth Factor Signaling;Cytokine Signaling;Carbohydrate Metabolism;Autophagy;Apoptosis;Angiogenesis;Adaptive Immune Response | mmu04010;mmu04012;mmu04014;mmu04015;mmu04022;mmu04024;mmu04062;mmu04066;mmu04068;mmu04071;mmu04072;mmu04150;mmu04151;mmu04152;mmu04210;mmu04211;mmu04213;mmu04261;mmu04370;mmu04380;mmu04510;mmu04530;mmu04550;mmu04611;mmu04620;mmu04630;mmu04660;mmu04662;mmu04664;mmu04666;mmu04668;mmu04722;mmu04725;mmu04728;mmu04910;mmu04914;mmu04915;mmu04917;mmu04919;mmu04920;mmu04922;mmu04923;mmu04931;mmu04932;mmu04933;mmu04973;mmu05142;mmu05145;mmu05152;mmu05160;mmu05161;mmu05162;mmu05164;mmu05166;mmu05169;mmu05200;mmu05205;mmu05210;mmu05211;mmu05212;mmu05213;mmu05214;mmu05215;mmu05218;mmu05220;mmu05221;mmu05222;mmu05223;mmu05230;mmu05231 |  | Akt2 | slateblue2 | 16 | -4.16901408450704 |
  | Mmp14-mRNA | -1.97 | 0.472 | -2.9 | -1.05 | 0.255 | 0.134 | 0.484 | 0.000713 | 0.022 | lm.nb | Cellular Stress, Innate Immune Response, Matrix Remodeling, Microglia Function | NS\_MM\_NEUROINFLAM\_V1.0 | Mmp14 | mRNA | 0 |  | Microglia Function;Matrix Remodeling;Innate Immune Response;Cellular Stress | mmu04668;mmu04912 |  | Mmp14 | slateblue2 | 16 | -4.17372881355932 |
  | Braf-mRNA | -0.643 | 0.154 | -0.945 | -0.341 | 0.64 | 0.519 | 0.789 | 0.000718 | 0.022 | lm.nb | Adaptive Immune Response, Angiogenesis, Apoptosis, Cytokine Signaling, Growth Factor Signaling, Innate Immune Response, Insulin Signaling, Neurons and Neurotransmission | NS\_MM\_NEUROINFLAM\_V1.0 | Braf | mRNA | 0 |  | Neurons and Neurotransmission;Insulin Signaling;Innate Immune Response;Growth Factor Signaling;Cytokine Signaling;Apoptosis;Angiogenesis;Adaptive Immune Response | mmu04010;mmu04012;mmu04015;mmu04024;mmu04062;mmu04068;mmu04150;mmu04270;mmu04510;mmu04650;mmu04720;mmu04722;mmu04726;mmu04730;mmu04810;mmu04910;mmu04914;mmu05034;mmu05160;mmu05200;mmu05205;mmu05210;mmu05211;mmu05212;mmu05213;mmu05214;mmu05215;mmu05216;mmu05218;mmu05219;mmu05220;mmu05221;mmu05223 |  | Braf | slateblue2 | 16 | -4.17532467532468 |
  | Casp3-mRNA | -0.652 | 0.157 | -0.96 | -0.344 | 0.636 | 0.514 | 0.788 | 0.000753 | 0.0225 | lm.nb | Apoptosis, Cellular Stress, DNA Damage, Growth Factor Signaling, Innate Immune Response, Matrix Remodeling | NS\_MM\_NEUROINFLAM\_V1.0 | Casp3 | mRNA | 0 |  | Matrix Remodeling;Innate Immune Response;Growth Factor Signaling;DNA Damage;Cellular Stress;Apoptosis | mmu04010;mmu04115;mmu04210;mmu04650;mmu04668;mmu04726;mmu04932;mmu04933;mmu05010;mmu05012;mmu05014;mmu05016;mmu05133;mmu05134;mmu05145;mmu05146;mmu05152;mmu05161;mmu05168;mmu05200;mmu05203;mmu05205;mmu05206;mmu05210;mmu05416 |  | Casp3 | slateblue2 | 16 | -4.15286624203822 |

  ##### DE Results - Innate Immune Response genes - DPI: 2 vs.CTRL

  Table displaying each sample's global significance scores and directed global significance scores as defined in the heatmaps above. The global significance score is calculated as the square root of the mean squared t-statistic for the genes in a gene set, with t-statistics coming from the linear regression underlying our differential expression analysis. The directed global significance score is calculated as the square root of the mean signed squared t-statistic for the genes in a gene set, with t-statistics coming from the linear regression underlying our differential expression analysis.

- **Volcano Plot: DPI: 3 vs.CTRL  
  More Plot Information**


  ##### Volcano Plot: DPI: 3 vs.CTRL

  Volcano plot displaying each gene's -log10(p-value) and log2 fold change for the selected covariate. Highly statistically significant genes fall at the top of the plot, and highly differentially expressed genes fall to either side. Genes within the selected gene set are highlighted in orange. Horizontal lines indicate various False Discovery Rate (FDR) thresholds.
- **DE Results - Innate Immune Response genes - DPI: 3 vs.CTRL  
  More Plot Information  Download CSV Data**

  | Probe Label | Log2 fold change | std error (log2) | Lower confidence limit (log2) | Upper confidence limit (log2) | Linear fold change | Lower confidence limit (linear) | Upper confidence limit (linear) | P-value | BY.p.value | method | Gene.sets | Codeset.Name | Probe.Label | Analyte.Type | Is.Control | Control.Type | Probe.Annotation | KEGG.Pathways | Cell.Type | Official.Gene.Name | volcanocol | volcanopch | tstats.all |
  | --- | --- | --- | --- | --- | --- | --- | --- | --- | --- | --- | --- | --- | --- | --- | --- | --- | --- | --- | --- | --- | --- | --- | --- |
  | Nfkbia-mRNA | 2.4 | 0.224 | 1.96 | 2.84 | 5.27 | 3.88 | 7.14 | 1.06e-08 | 3.12e-06 | lm.nb | Adaptive Immune Response, Apoptosis, Growth Factor Signaling, Inflammatory Signaling, Innate Immune Response, NF-kB | NS\_MM\_NEUROINFLAM\_V1.0 | Nfkbia | mRNA | 0 |  | NF-kB;Innate Immune Response;Inflammatory Signaling;Growth Factor Signaling;Apoptosis;Adaptive Immune Response | mmu04024;mmu04062;mmu04064;mmu04210;mmu04380;mmu04620;mmu04621;mmu04622;mmu04623;mmu04660;mmu04662;mmu04668;mmu04722;mmu04920;mmu04931;mmu05134;mmu05140;mmu05142;mmu05145;mmu05160;mmu05161;mmu05162;mmu05164;mmu05166;mmu05168;mmu05169;mmu05200;mmu05203;mmu05215;mmu05220;mmu05222 |  | Nfkbia | slateblue1 | 16 | 10.7142857142857 |
  | Irf3-mRNA | -1.48 | 0.165 | -1.8 | -1.15 | 0.359 | 0.287 | 0.45 | 1.3e-07 | 2.18e-05 | lm.nb | Apoptosis, Cytokine Signaling, Inflammatory Signaling, Innate Immune Response | NS\_MM\_NEUROINFLAM\_V1.0 | Irf3 | mRNA | 0 |  | Innate Immune Response;Inflammatory Signaling;Cytokine Signaling;Apoptosis | mmu04620;mmu04622;mmu04623;mmu05133;mmu05160;mmu05161;mmu05162;mmu05164;mmu05168;mmu05169;mmu05203 |  | Irf3 | slateblue1 | 16 | -8.96969696969697 |
  | Irf1-mRNA | 2.84 | 0.332 | 2.19 | 3.49 | 7.17 | 4.57 | 11.3 | 2.3e-07 | 3.39e-05 | lm.nb | Apoptosis, Inflammatory Signaling, Innate Immune Response | NS\_MM\_NEUROINFLAM\_V1.0 | Irf1 | mRNA | 0 |  | Innate Immune Response;Inflammatory Signaling;Apoptosis | mmu04917;mmu05133;mmu05160 |  | Irf1 | slateblue1 | 16 | 8.55421686746988 |
  | C1qc-mRNA | 1.28 | 0.155 | 0.979 | 1.59 | 2.43 | 1.97 | 3 | 3.54e-07 | 4.63e-05 | lm.nb | Innate Immune Response | NS\_MM\_NEUROINFLAM\_V1.0 | C1qc | mRNA | 0 |  | Innate Immune Response | mmu04610;mmu05020;mmu05133;mmu05142;mmu05150;mmu05322 |  | C1qc | slateblue1 | 16 | 8.25806451612903 |
  | Ptgs2-mRNA | 1.88 | 0.236 | 1.41 | 2.34 | 3.67 | 2.67 | 5.06 | 5.87e-07 | 7.14e-05 | lm.nb | Astrocyte Function, Cellular Stress, Innate Immune Response, Lipid Metabolism, Microglia Function, NF-kB | NS\_MM\_NEUROINFLAM\_V1.0 | Ptgs2 | mRNA | 0 |  | NF-kB;Microglia Function;Lipid Metabolism;Innate Immune Response;Cellular Stress;Astrocyte Function | mmu00590;mmu04064;mmu04370;mmu04668;mmu04723;mmu04726;mmu04913;mmu04921;mmu04923;mmu05140;mmu05200;mmu05206;mmu05222 |  | Ptgs2 | slateblue1 | 16 | 7.96610169491525 |
  | Ikbkb-mRNA | -0.601 | 0.0868 | -0.772 | -0.431 | 0.659 | 0.586 | 0.742 | 3.42e-06 | 0.00028 | loglinear | Adaptive Immune Response, Apoptosis, Cytokine Signaling, Growth Factor Signaling, Inflammatory Signaling, Innate Immune Response, NF-kB | NS\_MM\_NEUROINFLAM\_V1.0 | Ikbkb | mRNA | 0 |  | NF-kB;Innate Immune Response;Inflammatory Signaling;Growth Factor Signaling;Cytokine Signaling;Apoptosis;Adaptive Immune Response | mmu04010;mmu04014;mmu04062;mmu04064;mmu04068;mmu04150;mmu04151;mmu04210;mmu04380;mmu04620;mmu04621;mmu04622;mmu04623;mmu04660;mmu04662;mmu04668;mmu04722;mmu04910;mmu04920;mmu04930;mmu04931;mmu04932;mmu05142;mmu05145;mmu05160;mmu05161;mmu05164;mmu05166;mmu05168;mmu05169;mmu05200;mmu05206;mmu05212;mmu05215;mmu05220;mmu05221;mmu05222 |  | Ikbkb | slateblue1 | 16 | -6.92396313364055 |
  | Tnfrsf1a-mRNA | 1.92 | 0.281 | 1.37 | 2.47 | 3.79 | 2.59 | 5.56 | 3.96e-06 | 0.000317 | lm.nb | Apoptosis, Cytokine Signaling, Growth Factor Signaling, Innate Immune Response, NF-kB | NS\_MM\_NEUROINFLAM\_V1.0 | Tnfrsf1a | mRNA | 0 |  | NF-kB;Innate Immune Response;Growth Factor Signaling;Cytokine Signaling;Apoptosis | mmu04010;mmu04060;mmu04064;mmu04071;mmu04210;mmu04380;mmu04668;mmu04920;mmu04931;mmu04932;mmu05010;mmu05014;mmu05142;mmu05145;mmu05152;mmu05160;mmu05164;mmu05166;mmu05168 |  | Tnfrsf1a | slateblue1 | 16 | 6.83274021352313 |
  | Creb1-mRNA | -0.443 | 0.0668 | -0.574 | -0.312 | 0.735 | 0.672 | 0.805 | 5.69e-06 | 0.000418 | loglinear | Adaptive Immune Response, Carbohydrate Metabolism, DNA Damage, Growth Factor Signaling, Innate Immune Response, Neurons and Neurotransmission, Notch | NS\_MM\_NEUROINFLAM\_V1.0 | Creb1 | mRNA | 0 |  | Notch;Neurons and Neurotransmission;Innate Immune Response;Growth Factor Signaling;DNA Damage;Carbohydrate Metabolism;Adaptive Immune Response | mmu04022;mmu04024;mmu04151;mmu04152;mmu04211;mmu04261;mmu04380;mmu04612;mmu04668;mmu04713;mmu04725;mmu04728;mmu04911;mmu04915;mmu04916;mmu04922;mmu04924;mmu04925;mmu04931;mmu05016;mmu05030;mmu05031;mmu05034;mmu05152;mmu05161;mmu05166;mmu05203;mmu05215 |  | Creb1 | slateblue1 | 16 | -6.63173652694611 |
  | C1qa-mRNA | 1.06 | 0.186 | 0.69 | 1.42 | 2.08 | 1.61 | 2.68 | 3.53e-05 | 0.00178 | lm.nb | Innate Immune Response | NS\_MM\_NEUROINFLAM\_V1.0 | C1qa | mRNA | 0 |  | Innate Immune Response | mmu04610;mmu05020;mmu05133;mmu05142;mmu05150;mmu05322 |  | C1qa | slateblue1 | 16 | 5.6989247311828 |
  | Bcl2l1-mRNA | 0.414 | 0.0734 | 0.27 | 0.558 | 1.33 | 1.21 | 1.47 | 3.75e-05 | 0.00184 | loglinear | Apoptosis, Autophagy, Cytokine Signaling, Growth Factor Signaling, Innate Immune Response, NF-kB | NS\_MM\_NEUROINFLAM\_V1.0 | Bcl2l1 | mRNA | 0 |  | NF-kB;Innate Immune Response;Growth Factor Signaling;Cytokine Signaling;Autophagy;Apoptosis | mmu04014;mmu04064;mmu04151;mmu04210;mmu04630;mmu05014;mmu05145;mmu05166;mmu05200;mmu05202;mmu05212;mmu05220;mmu05222 |  | Bcl2l1 | slateblue1 | 16 | 5.64032697547684 |
  | Traf6-mRNA | 0.92 | 0.166 | 0.594 | 1.25 | 1.89 | 1.51 | 2.37 | 4.54e-05 | 0.00214 | lm.nb | Adaptive Immune Response, Apoptosis, Autophagy, Cytokine Signaling, Growth Factor Signaling, Innate Immune Response, NF-kB | NS\_MM\_NEUROINFLAM\_V1.0 | Traf6 | mRNA | 0 |  | NF-kB;Innate Immune Response;Growth Factor Signaling;Cytokine Signaling;Autophagy;Apoptosis;Adaptive Immune Response | mmu04010;mmu04064;mmu04120;mmu04144;mmu04380;mmu04620;mmu04621;mmu04622;mmu04722;mmu05133;mmu05140;mmu05142;mmu05145;mmu05152;mmu05160;mmu05162;mmu05168;mmu05169;mmu05200;mmu05222 |  | Traf6 | slateblue1 | 16 | 5.54216867469879 |
  | Akt2-mRNA | -0.766 | 0.142 | -1.04 | -0.488 | 0.588 | 0.485 | 0.713 | 5.85e-05 | 0.00265 | loglinear | Adaptive Immune Response, Angiogenesis, Apoptosis, Autophagy, Carbohydrate Metabolism, Cytokine Signaling, Growth Factor Signaling, Innate Immune Response, Insulin Signaling, Neurons and Neurotransmission, Wnt | NS\_MM\_NEUROINFLAM\_V1.0 | Akt2 | mRNA | 0 |  | Wnt;Neurons and Neurotransmission;Insulin Signaling;Innate Immune Response;Growth Factor Signaling;Cytokine Signaling;Carbohydrate Metabolism;Autophagy;Apoptosis;Angiogenesis;Adaptive Immune Response | mmu04010;mmu04012;mmu04014;mmu04015;mmu04022;mmu04024;mmu04062;mmu04066;mmu04068;mmu04071;mmu04072;mmu04150;mmu04151;mmu04152;mmu04210;mmu04211;mmu04213;mmu04261;mmu04370;mmu04380;mmu04510;mmu04530;mmu04550;mmu04611;mmu04620;mmu04630;mmu04660;mmu04662;mmu04664;mmu04666;mmu04668;mmu04722;mmu04725;mmu04728;mmu04910;mmu04914;mmu04915;mmu04917;mmu04919;mmu04920;mmu04922;mmu04923;mmu04931;mmu04932;mmu04933;mmu04973;mmu05142;mmu05145;mmu05152;mmu05160;mmu05161;mmu05162;mmu05164;mmu05166;mmu05169;mmu05200;mmu05205;mmu05210;mmu05211;mmu05212;mmu05213;mmu05214;mmu05215;mmu05218;mmu05220;mmu05221;mmu05222;mmu05223;mmu05230;mmu05231 |  | Akt2 | slateblue1 | 16 | -5.3943661971831 |
  | Chuk-mRNA | -0.423 | 0.0787 | -0.577 | -0.269 | 0.746 | 0.67 | 0.83 | 6.21e-05 | 0.00275 | loglinear | Adaptive Immune Response, Apoptosis, Cytokine Signaling, Growth Factor Signaling, Inflammatory Signaling, Innate Immune Response, NF-kB | NS\_MM\_NEUROINFLAM\_V1.0 | Chuk | mRNA | 0 |  | NF-kB;Innate Immune Response;Inflammatory Signaling;Growth Factor Signaling;Cytokine Signaling;Apoptosis;Adaptive Immune Response | mmu04010;mmu04014;mmu04062;mmu04064;mmu04068;mmu04151;mmu04210;mmu04380;mmu04620;mmu04621;mmu04622;mmu04623;mmu04660;mmu04662;mmu04668;mmu04920;mmu05142;mmu05145;mmu05160;mmu05161;mmu05162;mmu05166;mmu05168;mmu05169;mmu05200;mmu05212;mmu05215;mmu05220;mmu05221;mmu05222 |  | Chuk | slateblue1 | 16 | -5.37484116899619 |
  | Ripk2-mRNA | 1 | 0.193 | 0.627 | 1.38 | 2.01 | 1.54 | 2.61 | 8.44e-05 | 0.00355 | loglinear | Adaptive Immune Response, Apoptosis, Cytokine Signaling, Growth Factor Signaling, Innate Immune Response | NS\_MM\_NEUROINFLAM\_V1.0 | Ripk2 | mRNA | 0 |  | Innate Immune Response;Growth Factor Signaling;Cytokine Signaling;Apoptosis;Adaptive Immune Response | mmu04621;mmu04722;mmu05152 |  | Ripk2 | slateblue1 | 16 | 5.18134715025907 |
  | C1qb-mRNA | 1.19 | 0.257 | 0.69 | 1.7 | 2.29 | 1.61 | 3.25 | 0.000272 | 0.0098 | lm.nb | Innate Immune Response | NS\_MM\_NEUROINFLAM\_V1.0 | C1qb | mRNA | 0 |  | Innate Immune Response | mmu04610;mmu05020;mmu05133;mmu05142;mmu05150;mmu05322 |  | C1qb | slateblue2 | 16 | 4.63035019455253 |
  | Traf3-mRNA | -0.463 | 0.104 | -0.666 | -0.26 | 0.725 | 0.63 | 0.835 | 0.000386 | 0.013 | loglinear | Apoptosis, Cytokine Signaling, Inflammatory Signaling, Innate Immune Response, NF-kB | NS\_MM\_NEUROINFLAM\_V1.0 | Traf3 | mRNA | 0 |  | NF-kB;Innate Immune Response;Inflammatory Signaling;Cytokine Signaling;Apoptosis | mmu04064;mmu04620;mmu04622;mmu04668;mmu05160;mmu05168;mmu05169;mmu05200;mmu05203;mmu05222 |  | Traf3 | slateblue2 | 16 | -4.45192307692308 |
  | Lgmn-mRNA | 0.371 | 0.0832 | 0.208 | 0.534 | 1.29 | 1.15 | 1.45 | 0.000401 | 0.0134 | loglinear | Adaptive Immune Response, Innate Immune Response | NS\_MM\_NEUROINFLAM\_V1.0 | Lgmn | mRNA | 0 |  | Innate Immune Response;Adaptive Immune Response | mmu04142;mmu04612 |  | Lgmn | slateblue2 | 16 | 4.45913461538462 |
  | Fcgr3-mRNA | 1.62 | 0.365 | 0.902 | 2.33 | 3.07 | 1.87 | 5.04 | 0.000421 | 0.0139 | lm.nb | Adaptive Immune Response, Autophagy, Inflammatory Signaling, Innate Immune Response | NS\_MM\_NEUROINFLAM\_V1.0 | Fcgr3 | mRNA | 0 |  | Innate Immune Response;Inflammatory Signaling;Autophagy;Adaptive Immune Response | mmu04145;mmu04380;mmu05140;mmu05150;mmu05152 |  | Fcgr3 | slateblue2 | 16 | 4.43835616438356 |
  | Traf2-mRNA | -0.552 | 0.129 | -0.805 | -0.298 | 0.682 | 0.572 | 0.813 | 0.000592 | 0.0183 | lm.nb | Apoptosis, Cytokine Signaling, Growth Factor Signaling, Inflammatory Signaling, Innate Immune Response, NF-kB | NS\_MM\_NEUROINFLAM\_V1.0 | Traf2 | mRNA | 0 |  | NF-kB;Innate Immune Response;Inflammatory Signaling;Growth Factor Signaling;Cytokine Signaling;Apoptosis | mmu04010;mmu04064;mmu04071;mmu04141;mmu04210;mmu04380;mmu04622;mmu04668;mmu04920;mmu04932;mmu05160;mmu05168;mmu05169;mmu05200;mmu05203;mmu05222 |  | Traf2 | slateblue2 | 16 | -4.27906976744186 |
  | Bcl2-mRNA | -0.943 | 0.227 | -1.39 | -0.498 | 0.52 | 0.382 | 0.708 | 0.000747 | 0.0225 | lm.nb | Apoptosis, Autophagy, Cellular Stress, Cytokine Signaling, DNA Damage, Growth Factor Signaling, Innate Immune Response, Microglia Function, NF-kB | NS\_MM\_NEUROINFLAM\_V1.0 | Bcl2 | mRNA | 0 |  | NF-kB;Microglia Function;Innate Immune Response;Growth Factor Signaling;DNA Damage;Cytokine Signaling;Cellular Stress;Autophagy;Apoptosis | mmu04064;mmu04066;mmu04071;mmu04141;mmu04151;mmu04210;mmu04261;mmu04510;mmu04630;mmu04722;mmu04725;mmu04933;mmu05014;mmu05145;mmu05152;mmu05161;mmu05169;mmu05200;mmu05206;mmu05210;mmu05215;mmu05222 |  | Bcl2 | slateblue2 | 16 | -4.15418502202643 |

  ##### DE Results - Innate Immune Response genes - DPI: 3 vs.CTRL

  Table displaying each sample's global significance scores and directed global significance scores as defined in the heatmaps above. The global significance score is calculated as the square root of the mean squared t-statistic for the genes in a gene set, with t-statistics coming from the linear regression underlying our differential expression analysis. The directed global significance score is calculated as the square root of the mean signed squared t-statistic for the genes in a gene set, with t-statistics coming from the linear regression underlying our differential expression analysis.

DPI:
- DPI: differential expression in 1 vs. baseline of CTRL
- DPI: differential expression in 2 vs. baseline of CTRL
- DPI: differential expression in 3 vs. baseline of CTRL

- **Volcano Plot: DPI: 1 vs.CTRL  
  More Plot Information**


  ##### Volcano Plot: DPI: 1 vs.CTRL

  Volcano plot displaying each gene's -log10(p-value) and log2 fold change for the selected covariate. Highly statistically significant genes fall at the top of the plot, and highly differentially expressed genes fall to either side. Genes within the selected gene set are highlighted in orange. Horizontal lines indicate various False Discovery Rate (FDR) thresholds.
- **DE Results - Insulin Signaling genes - DPI: 1 vs.CTRL  
  More Plot Information  Download CSV Data**

  | Probe Label | Log2 fold change | std error (log2) | Lower confidence limit (log2) | Upper confidence limit (log2) | Linear fold change | Lower confidence limit (linear) | Upper confidence limit (linear) | P-value | BY.p.value | method | Gene.sets | Codeset.Name | Probe.Label | Analyte.Type | Is.Control | Control.Type | Probe.Annotation | KEGG.Pathways | Cell.Type | Official.Gene.Name | volcanocol | volcanopch | tstats.all |
  | --- | --- | --- | --- | --- | --- | --- | --- | --- | --- | --- | --- | --- | --- | --- | --- | --- | --- | --- | --- | --- | --- | --- | --- |
  | Dusp7-mRNA | -0.378 | 0.0611 | -0.497 | -0.258 | 0.77 | 0.708 | 0.836 | 1.32e-05 | 0.0093 | loglinear | Adaptive Immune Response, Angiogenesis, Cytokine Signaling, Growth Factor Signaling, Innate Immune Response, Insulin Signaling, Microglia Function | NS\_MM\_NEUROINFLAM\_V1.0 | Dusp7 | mRNA | 0 |  | Microglia Function;Insulin Signaling;Innate Immune Response;Growth Factor Signaling;Cytokine Signaling;Angiogenesis;Adaptive Immune Response | mmu04010 |  | Dusp7 | slateblue2 | 16 | -6.18657937806874 |
  | Braf-mRNA | -0.641 | 0.154 | -0.943 | -0.339 | 0.641 | 0.52 | 0.791 | 0.000739 | 0.0815 | lm.nb | Adaptive Immune Response, Angiogenesis, Apoptosis, Cytokine Signaling, Growth Factor Signaling, Innate Immune Response, Insulin Signaling, Neurons and Neurotransmission | NS\_MM\_NEUROINFLAM\_V1.0 | Braf | mRNA | 0 |  | Neurons and Neurotransmission;Insulin Signaling;Innate Immune Response;Growth Factor Signaling;Cytokine Signaling;Apoptosis;Angiogenesis;Adaptive Immune Response | mmu04010;mmu04012;mmu04015;mmu04024;mmu04062;mmu04068;mmu04150;mmu04270;mmu04510;mmu04650;mmu04720;mmu04722;mmu04726;mmu04730;mmu04810;mmu04910;mmu04914;mmu05034;mmu05160;mmu05200;mmu05205;mmu05210;mmu05211;mmu05212;mmu05213;mmu05214;mmu05215;mmu05216;mmu05218;mmu05219;mmu05220;mmu05221;mmu05223 |  | Braf | slateblue3 | 16 | -4.16233766233766 |
  | Psmb8-mRNA | 1.36 | 0.365 | 0.649 | 2.08 | 2.57 | 1.57 | 4.23 | 0.00179 | 0.129 | lm.nb | Adaptive Immune Response, Angiogenesis, Apoptosis, Astrocyte Function, Cell Cycle, Cytokine Signaling, Growth Factor Signaling, Inflammatory Signaling, Insulin Signaling, Microglia Function, NF-kB, Wnt | NS\_MM\_NEUROINFLAM\_V1.0 | Psmb8 | mRNA | 0 |  | Wnt;NF-kB;Microglia Function;Insulin Signaling;Inflammatory Signaling;Growth Factor Signaling;Cytokine Signaling;Cell Cycle;Astrocyte Function;Apoptosis;Angiogenesis;Adaptive Immune Response |  |  | Psmb8 | slateblue4 | 16 | 3.72602739726027 |
  | Igf1r-mRNA | 0.523 | 0.156 | 0.216 | 0.83 | 1.44 | 1.16 | 1.78 | 0.00414 | 0.197 | lm.nb | Apoptosis, Autophagy, Carbohydrate Metabolism, Growth Factor Signaling, Insulin Signaling | NS\_MM\_NEUROINFLAM\_V1.0 | Igf1r | mRNA | 0 |  | Insulin Signaling;Growth Factor Signaling;Carbohydrate Metabolism;Autophagy;Apoptosis | mmu04014;mmu04015;mmu04066;mmu04068;mmu04114;mmu04144;mmu04151;mmu04152;mmu04211;mmu04213;mmu04510;mmu04520;mmu04550;mmu04730;mmu04913;mmu04914;mmu05200;mmu05202;mmu05205;mmu05214;mmu05215;mmu05218 |  | Igf1r | slateblue4 | 16 | 3.3525641025641 |
  | Map2k1-mRNA | -1.03 | 0.32 | -1.65 | -0.399 | 0.491 | 0.318 | 0.759 | 0.00553 | 0.224 | lm.nb | Adaptive Immune Response, Angiogenesis, Autophagy, Cytokine Signaling, Growth Factor Signaling, Innate Immune Response, Insulin Signaling, Microglia Function | NS\_MM\_NEUROINFLAM\_V1.0 | Map2k1 | mRNA | 0 |  | Microglia Function;Insulin Signaling;Innate Immune Response;Growth Factor Signaling;Cytokine Signaling;Autophagy;Angiogenesis;Adaptive Immune Response | mmu04010;mmu04012;mmu04014;mmu04015;mmu04022;mmu04024;mmu04062;mmu04066;mmu04068;mmu04071;mmu04072;mmu04114;mmu04151;mmu04270;mmu04370;mmu04380;mmu04510;mmu04540;mmu04550;mmu04620;mmu04650;mmu04660;mmu04662;mmu04664;mmu04666;mmu04668;mmu04720;mmu04722;mmu04725;mmu04726;mmu04730;mmu04810;mmu04910;mmu04912;mmu04914;mmu04915;mmu04916;mmu04917;mmu04919;mmu04921;mmu05020;mmu05034;mmu05161;mmu05164;mmu05200;mmu05205;mmu05206;mmu05210;mmu05211;mmu05212;mmu05213;mmu05214;mmu05215;mmu05216;mmu05218;mmu05219;mmu05220;mmu05221;mmu05223;mmu05230;mmu05231 |  | Map2k1 | slateblue4 | 16 | -3.21875 |
  | Dlg4-mRNA | -0.175 | 0.056 | -0.284 | -0.0649 | 0.886 | 0.821 | 0.956 | 0.00662 | 0.246 | loglinear | Adaptive Immune Response, Angiogenesis, Cytokine Signaling, Growth Factor Signaling, Insulin Signaling, Neurons and Neurotransmission | NS\_MM\_NEUROINFLAM\_V1.0 | Dlg4 | mRNA | 0 |  | Neurons and Neurotransmission;Insulin Signaling;Growth Factor Signaling;Cytokine Signaling;Angiogenesis;Adaptive Immune Response | mmu04390;mmu04724;mmu05016;mmu05030 |  | Dlg4 | slateblue4 | 16 | -3.125 |
  | Nefl-mRNA | -0.382 | 0.123 | -0.624 | -0.141 | 0.767 | 0.649 | 0.907 | 0.00687 | 0.248 | lm.nb | Adaptive Immune Response, Angiogenesis, Cytokine Signaling, Growth Factor Signaling, Insulin Signaling, Neurons and Neurotransmission | NS\_MM\_NEUROINFLAM\_V1.0 | Nefl | mRNA | 0 |  | Neurons and Neurotransmission;Insulin Signaling;Growth Factor Signaling;Cytokine Signaling;Angiogenesis;Adaptive Immune Response | mmu05014 |  | Nefl | slateblue4 | 16 | -3.10569105691057 |
  | Pik3r2-mRNA | -0.223 | 0.0793 | -0.378 | -0.0676 | 0.857 | 0.769 | 0.954 | 0.0125 | 0.357 | loglinear | Adaptive Immune Response, Angiogenesis, Apoptosis, Autophagy, Cytokine Signaling, Growth Factor Signaling, Innate Immune Response, Insulin Signaling | NS\_MM\_NEUROINFLAM\_V1.0 | Pik3r2 | mRNA | 0 |  | Insulin Signaling;Innate Immune Response;Growth Factor Signaling;Cytokine Signaling;Autophagy;Apoptosis;Angiogenesis;Adaptive Immune Response | mmu04012;mmu04014;mmu04015;mmu04022;mmu04024;mmu04062;mmu04066;mmu04068;mmu04070;mmu04071;mmu04072;mmu04150;mmu04151;mmu04152;mmu04210;mmu04211;mmu04213;mmu04261;mmu04370;mmu04380;mmu04510;mmu04550;mmu04611;mmu04620;mmu04630;mmu04650;mmu04660;mmu04662;mmu04664;mmu04666;mmu04668;mmu04670;mmu04722;mmu04725;mmu04750;mmu04810;mmu04910;mmu04914;mmu04915;mmu04917;mmu04919;mmu04921;mmu04923;mmu04930;mmu04931;mmu04932;mmu04933;mmu04960;mmu04973;mmu05100;mmu05142;mmu05145;mmu05146;mmu05160;mmu05161;mmu05162;mmu05164;mmu05166;mmu05169;mmu05200;mmu05203;mmu05205;mmu05210;mmu05211;mmu05212;mmu05213;mmu05214;mmu05215;mmu05218;mmu05220;mmu05221;mmu05222;mmu05223;mmu05230;mmu05231 |  | Pik3r2 | slateblue4 | 16 | -2.81210592686003 |
  | Pik3ca-mRNA | -0.287 | 0.107 | -0.497 | -0.0771 | 0.82 | 0.709 | 0.948 | 0.0164 | 0.417 | lm.nb | Adaptive Immune Response, Angiogenesis, Apoptosis, Autophagy, Carbohydrate Metabolism, Cytokine Signaling, Growth Factor Signaling, Innate Immune Response, Insulin Signaling, Lipid Metabolism | NS\_MM\_NEUROINFLAM\_V1.0 | Pik3ca | mRNA | 0 |  | Lipid Metabolism;Insulin Signaling;Innate Immune Response;Growth Factor Signaling;Cytokine Signaling;Carbohydrate Metabolism;Autophagy;Apoptosis;Angiogenesis;Adaptive Immune Response | mmu00562;mmu04012;mmu04014;mmu04015;mmu04022;mmu04024;mmu04062;mmu04066;mmu04068;mmu04070;mmu04071;mmu04072;mmu04150;mmu04151;mmu04152;mmu04210;mmu04211;mmu04213;mmu04261;mmu04370;mmu04380;mmu04510;mmu04550;mmu04611;mmu04620;mmu04630;mmu04650;mmu04660;mmu04662;mmu04664;mmu04666;mmu04668;mmu04670;mmu04722;mmu04725;mmu04750;mmu04810;mmu04910;mmu04914;mmu04915;mmu04917;mmu04919;mmu04921;mmu04923;mmu04930;mmu04931;mmu04932;mmu04933;mmu04960;mmu04973;mmu05100;mmu05142;mmu05145;mmu05146;mmu05160;mmu05161;mmu05162;mmu05164;mmu05166;mmu05169;mmu05200;mmu05203;mmu05205;mmu05210;mmu05211;mmu05212;mmu05213;mmu05214;mmu05215;mmu05218;mmu05220;mmu05221;mmu05222;mmu05223;mmu05230;mmu05231 |  | Pik3ca | slateblue4 | 16 | -2.68224299065421 |
  | Grin2b-mRNA | -0.254 | 0.101 | -0.451 | -0.057 | 0.839 | 0.731 | 0.961 | 0.0225 | 0.508 | lm.nb | Adaptive Immune Response, Angiogenesis, Cytokine Signaling, Growth Factor Signaling, Insulin Signaling, Neurons and Neurotransmission | NS\_MM\_NEUROINFLAM\_V1.0 | Grin2b | mRNA | 0 |  | Neurons and Neurotransmission;Insulin Signaling;Growth Factor Signaling;Cytokine Signaling;Angiogenesis;Adaptive Immune Response | mmu04014;mmu04015;mmu04024;mmu04080;mmu04713;mmu04720;mmu04724;mmu04728;mmu05010;mmu05014;mmu05016;mmu05030;mmu05031;mmu05033;mmu05034;mmu05322 |  | Grin2b | azure4 | 1 | -2.51485148514851 |
  | Pik3r1-mRNA | -0.169 | 0.0713 | -0.308 | -0.0288 | 0.89 | 0.808 | 0.98 | 0.0311 | 0.62 | loglinear | Adaptive Immune Response, Angiogenesis, Apoptosis, Autophagy, Carbohydrate Metabolism, Cytokine Signaling, Growth Factor Signaling, Innate Immune Response, Insulin Signaling, Lipid Metabolism | NS\_MM\_NEUROINFLAM\_V1.0 | Pik3r1 | mRNA | 0 |  | Lipid Metabolism;Insulin Signaling;Innate Immune Response;Growth Factor Signaling;Cytokine Signaling;Carbohydrate Metabolism;Autophagy;Apoptosis;Angiogenesis;Adaptive Immune Response | mmu04012;mmu04014;mmu04015;mmu04022;mmu04024;mmu04062;mmu04066;mmu04068;mmu04070;mmu04071;mmu04072;mmu04150;mmu04151;mmu04152;mmu04210;mmu04211;mmu04213;mmu04261;mmu04370;mmu04380;mmu04510;mmu04550;mmu04611;mmu04620;mmu04630;mmu04650;mmu04660;mmu04662;mmu04664;mmu04666;mmu04668;mmu04670;mmu04722;mmu04725;mmu04750;mmu04810;mmu04910;mmu04914;mmu04915;mmu04917;mmu04919;mmu04921;mmu04923;mmu04930;mmu04931;mmu04932;mmu04933;mmu04960;mmu04973;mmu05100;mmu05142;mmu05145;mmu05146;mmu05160;mmu05161;mmu05162;mmu05164;mmu05166;mmu05169;mmu05200;mmu05203;mmu05205;mmu05210;mmu05211;mmu05212;mmu05213;mmu05214;mmu05215;mmu05218;mmu05220;mmu05221;mmu05222;mmu05223;mmu05230;mmu05231 |  | Pik3r1 | azure4 | 1 | -2.37026647966339 |
  | Eef2k-mRNA | -0.592 | 0.27 | -1.12 | -0.0629 | 0.663 | 0.459 | 0.957 | 0.0434 | 0.761 | lm.nb | Growth Factor Signaling, Insulin Signaling, Microglia Function | NS\_MM\_NEUROINFLAM\_V1.0 | Eef2k | mRNA | 0 |  | Microglia Function;Insulin Signaling;Growth Factor Signaling | mmu04152;mmu04921 |  | Eef2k | azure4 | 1 | -2.19259259259259 |
  | Akt2-mRNA | -0.291 | 0.142 | -0.568 | -0.0129 | 0.818 | 0.674 | 0.991 | 0.057 | 0.91 | loglinear | Adaptive Immune Response, Angiogenesis, Apoptosis, Autophagy, Carbohydrate Metabolism, Cytokine Signaling, Growth Factor Signaling, Innate Immune Response, Insulin Signaling, Neurons and Neurotransmission, Wnt | NS\_MM\_NEUROINFLAM\_V1.0 | Akt2 | mRNA | 0 |  | Wnt;Neurons and Neurotransmission;Insulin Signaling;Innate Immune Response;Growth Factor Signaling;Cytokine Signaling;Carbohydrate Metabolism;Autophagy;Apoptosis;Angiogenesis;Adaptive Immune Response | mmu04010;mmu04012;mmu04014;mmu04015;mmu04022;mmu04024;mmu04062;mmu04066;mmu04068;mmu04071;mmu04072;mmu04150;mmu04151;mmu04152;mmu04210;mmu04211;mmu04213;mmu04261;mmu04370;mmu04380;mmu04510;mmu04530;mmu04550;mmu04611;mmu04620;mmu04630;mmu04660;mmu04662;mmu04664;mmu04666;mmu04668;mmu04722;mmu04725;mmu04728;mmu04910;mmu04914;mmu04915;mmu04917;mmu04919;mmu04920;mmu04922;mmu04923;mmu04931;mmu04932;mmu04933;mmu04973;mmu05142;mmu05145;mmu05152;mmu05160;mmu05161;mmu05162;mmu05164;mmu05166;mmu05169;mmu05200;mmu05205;mmu05210;mmu05211;mmu05212;mmu05213;mmu05214;mmu05215;mmu05218;mmu05220;mmu05221;mmu05222;mmu05223;mmu05230;mmu05231 |  | Akt2 | azure4 | 1 | -2.04929577464789 |
  | Erbb3-mRNA | 1.01 | 0.526 | -0.0216 | 2.04 | 2.01 | 0.985 | 4.11 | 0.073 | 1 | lm.nb | Adaptive Immune Response, Angiogenesis, Cytokine Signaling, Growth Factor Signaling, Insulin Signaling, Oligodendrocyte Function | NS\_MM\_NEUROINFLAM\_V1.0 | Erbb3 | mRNA | 0 |  | Oligodendrocyte Function;Insulin Signaling;Growth Factor Signaling;Cytokine Signaling;Angiogenesis;Adaptive Immune Response | mmu04012;mmu04020;mmu04144;mmu05205;mmu05206 | Oligodendrocytes | Erbb3 | azure4 | 1 | 1.92015209125475 |
  | Atp6v1a-mRNA | -0.119 | 0.0628 | -0.242 | 0.00424 | 0.921 | 0.846 | 1 | 0.0767 | 1 | loglinear | Growth Factor Signaling, Insulin Signaling, Microglia Function | NS\_MM\_NEUROINFLAM\_V1.0 | Atp6v1a | mRNA | 0 |  | Microglia Function;Insulin Signaling;Growth Factor Signaling | mmu04145;mmu04721;mmu05323 |  | Atp6v1a | azure4 | 1 | -1.89490445859873 |
  | Egfr-mRNA | 0.542 | 0.287 | -0.0206 | 1.1 | 1.46 | 0.986 | 2.15 | 0.0773 | 1 | lm.nb | Adaptive Immune Response, Angiogenesis, Astrocyte Function, Cytokine Signaling, Growth Factor Signaling, Insulin Signaling | NS\_MM\_NEUROINFLAM\_V1.0 | Egfr | mRNA | 0 |  | Insulin Signaling;Growth Factor Signaling;Cytokine Signaling;Astrocyte Function;Angiogenesis;Adaptive Immune Response | mmu04010;mmu04012;mmu04014;mmu04015;mmu04020;mmu04060;mmu04066;mmu04068;mmu04072;mmu04144;mmu04151;mmu04510;mmu04520;mmu04540;mmu04810;mmu04912;mmu04915;mmu04921;mmu05160;mmu05200;mmu05205;mmu05206;mmu05212;mmu05213;mmu05214;mmu05215;mmu05218;mmu05219;mmu05223;mmu05230;mmu05231 | Astrocytes | Egfr | azure4 | 1 | 1.88850174216028 |
  | Mafb-mRNA | -0.219 | 0.126 | -0.467 | 0.0282 | 0.859 | 0.724 | 1.02 | 0.102 | 1 | loglinear | Insulin Signaling | NS\_MM\_NEUROINFLAM\_V1.0 | Mafb | mRNA | 0 |  | Insulin Signaling |  |  | Mafb | azure4 | 1 | -1.73809523809524 |
  | Akt1-mRNA | 0.177 | 0.107 | -0.0326 | 0.387 | 1.13 | 0.978 | 1.31 | 0.117 | 1 | loglinear | Adaptive Immune Response, Angiogenesis, Apoptosis, Autophagy, Carbohydrate Metabolism, Cellular Stress, Cytokine Signaling, Growth Factor Signaling, Innate Immune Response, Insulin Signaling, Neurons and Neurotransmission, Wnt | NS\_MM\_NEUROINFLAM\_V1.0 | Akt1 | mRNA | 0 |  | Wnt;Neurons and Neurotransmission;Insulin Signaling;Innate Immune Response;Growth Factor Signaling;Cytokine Signaling;Cellular Stress;Carbohydrate Metabolism;Autophagy;Apoptosis;Angiogenesis;Adaptive Immune Response | mmu04010;mmu04012;mmu04014;mmu04015;mmu04022;mmu04024;mmu04062;mmu04066;mmu04068;mmu04071;mmu04072;mmu04150;mmu04151;mmu04152;mmu04210;mmu04211;mmu04213;mmu04261;mmu04370;mmu04380;mmu04510;mmu04530;mmu04550;mmu04611;mmu04620;mmu04630;mmu04660;mmu04662;mmu04664;mmu04666;mmu04668;mmu04722;mmu04725;mmu04728;mmu04910;mmu04914;mmu04915;mmu04917;mmu04919;mmu04920;mmu04922;mmu04923;mmu04931;mmu04932;mmu04933;mmu04973;mmu05142;mmu05145;mmu05152;mmu05160;mmu05161;mmu05162;mmu05164;mmu05166;mmu05169;mmu05200;mmu05205;mmu05210;mmu05211;mmu05212;mmu05213;mmu05214;mmu05215;mmu05218;mmu05220;mmu05221;mmu05222;mmu05223;mmu05230;mmu05231 |  | Akt1 | azure4 | 1 | 1.65420560747664 |
  | Pik3cb-mRNA | -0.144 | 0.116 | -0.372 | 0.0841 | 0.905 | 0.773 | 1.06 | 0.234 | 1 | loglinear | Adaptive Immune Response, Angiogenesis, Apoptosis, Autophagy, Carbohydrate Metabolism, Cytokine Signaling, Growth Factor Signaling, Innate Immune Response, Insulin Signaling, Lipid Metabolism | NS\_MM\_NEUROINFLAM\_V1.0 | Pik3cb | mRNA | 0 |  | Lipid Metabolism;Insulin Signaling;Innate Immune Response;Growth Factor Signaling;Cytokine Signaling;Carbohydrate Metabolism;Autophagy;Apoptosis;Angiogenesis;Adaptive Immune Response | mmu00562;mmu04012;mmu04014;mmu04015;mmu04022;mmu04024;mmu04062;mmu04066;mmu04068;mmu04070;mmu04071;mmu04072;mmu04150;mmu04151;mmu04152;mmu04210;mmu04211;mmu04213;mmu04261;mmu04370;mmu04380;mmu04510;mmu04550;mmu04611;mmu04620;mmu04630;mmu04650;mmu04660;mmu04662;mmu04664;mmu04666;mmu04668;mmu04670;mmu04722;mmu04725;mmu04750;mmu04810;mmu04910;mmu04914;mmu04915;mmu04917;mmu04919;mmu04921;mmu04923;mmu04930;mmu04931;mmu04932;mmu04933;mmu04960;mmu04973;mmu05100;mmu05142;mmu05145;mmu05146;mmu05160;mmu05161;mmu05162;mmu05164;mmu05166;mmu05169;mmu05200;mmu05203;mmu05205;mmu05210;mmu05211;mmu05212;mmu05213;mmu05214;mmu05215;mmu05218;mmu05220;mmu05221;mmu05222;mmu05223;mmu05230;mmu05231 |  | Pik3cb | azure4 | 1 | -1.24137931034483 |
  | Kit-mRNA | -0.194 | 0.207 | -0.601 | 0.212 | 0.874 | 0.659 | 1.16 | 0.363 | 1 | lm.nb | Adaptive Immune Response, Angiogenesis, Cytokine Signaling, Growth Factor Signaling, Insulin Signaling | NS\_MM\_NEUROINFLAM\_V1.0 | Kit | mRNA | 0 |  | Insulin Signaling;Growth Factor Signaling;Cytokine Signaling;Angiogenesis;Adaptive Immune Response | mmu04014;mmu04015;mmu04060;mmu04072;mmu04144;mmu04151;mmu04640;mmu04916;mmu05200;mmu05221;mmu05230 |  | Kit | azure4 | 1 | -0.93719806763285 |

  ##### DE Results - Insulin Signaling genes - DPI: 1 vs.CTRL

  Table displaying each sample's global significance scores and directed global significance scores as defined in the heatmaps above. The global significance score is calculated as the square root of the mean squared t-statistic for the genes in a gene set, with t-statistics coming from the linear regression underlying our differential expression analysis. The directed global significance score is calculated as the square root of the mean signed squared t-statistic for the genes in a gene set, with t-statistics coming from the linear regression underlying our differential expression analysis.

- **Volcano Plot: DPI: 2 vs.CTRL  
  More Plot Information**


  ##### Volcano Plot: DPI: 2 vs.CTRL

  Volcano plot displaying each gene's -log10(p-value) and log2 fold change for the selected covariate. Highly statistically significant genes fall at the top of the plot, and highly differentially expressed genes fall to either side. Genes within the selected gene set are highlighted in orange. Horizontal lines indicate various False Discovery Rate (FDR) thresholds.
- **DE Results - Insulin Signaling genes - DPI: 2 vs.CTRL  
  More Plot Information  Download CSV Data**

  | Probe Label | Log2 fold change | std error (log2) | Lower confidence limit (log2) | Upper confidence limit (log2) | Linear fold change | Lower confidence limit (linear) | Upper confidence limit (linear) | P-value | BY.p.value | method | Gene.sets | Codeset.Name | Probe.Label | Analyte.Type | Is.Control | Control.Type | Probe.Annotation | KEGG.Pathways | Cell.Type | Official.Gene.Name | volcanocol | volcanopch | tstats.all |
  | --- | --- | --- | --- | --- | --- | --- | --- | --- | --- | --- | --- | --- | --- | --- | --- | --- | --- | --- | --- | --- | --- | --- | --- |
  | Dusp7-mRNA | -0.496 | 0.0611 | -0.616 | -0.377 | 0.709 | 0.652 | 0.77 | 4.53e-07 | 9.99e-05 | loglinear | Adaptive Immune Response, Angiogenesis, Cytokine Signaling, Growth Factor Signaling, Innate Immune Response, Insulin Signaling, Microglia Function | NS\_MM\_NEUROINFLAM\_V1.0 | Dusp7 | mRNA | 0 |  | Microglia Function;Insulin Signaling;Innate Immune Response;Growth Factor Signaling;Cytokine Signaling;Angiogenesis;Adaptive Immune Response | mmu04010 |  | Dusp7 | slateblue1 | 16 | -8.11783960720131 |
  | Psmb8-mRNA | 2.23 | 0.352 | 1.54 | 2.92 | 4.7 | 2.91 | 7.59 | 9.89e-06 | 0.000997 | lm.nb | Adaptive Immune Response, Angiogenesis, Apoptosis, Astrocyte Function, Cell Cycle, Cytokine Signaling, Growth Factor Signaling, Inflammatory Signaling, Insulin Signaling, Microglia Function, NF-kB, Wnt | NS\_MM\_NEUROINFLAM\_V1.0 | Psmb8 | mRNA | 0 |  | Wnt;NF-kB;Microglia Function;Insulin Signaling;Inflammatory Signaling;Growth Factor Signaling;Cytokine Signaling;Cell Cycle;Astrocyte Function;Apoptosis;Angiogenesis;Adaptive Immune Response |  |  | Psmb8 | slateblue1 | 16 | 6.33522727272727 |
  | Igf1r-mRNA | 0.813 | 0.155 | 0.51 | 1.12 | 1.76 | 1.42 | 2.17 | 7.74e-05 | 0.00441 | lm.nb | Apoptosis, Autophagy, Carbohydrate Metabolism, Growth Factor Signaling, Insulin Signaling | NS\_MM\_NEUROINFLAM\_V1.0 | Igf1r | mRNA | 0 |  | Insulin Signaling;Growth Factor Signaling;Carbohydrate Metabolism;Autophagy;Apoptosis | mmu04014;mmu04015;mmu04066;mmu04068;mmu04114;mmu04144;mmu04151;mmu04152;mmu04211;mmu04213;mmu04510;mmu04520;mmu04550;mmu04730;mmu04913;mmu04914;mmu05200;mmu05202;mmu05205;mmu05214;mmu05215;mmu05218 |  | Igf1r | slateblue1 | 16 | 5.24516129032258 |
  | Pik3r2-mRNA | -0.338 | 0.0793 | -0.494 | -0.183 | 0.791 | 0.71 | 0.881 | 0.000587 | 0.0197 | loglinear | Adaptive Immune Response, Angiogenesis, Apoptosis, Autophagy, Cytokine Signaling, Growth Factor Signaling, Innate Immune Response, Insulin Signaling | NS\_MM\_NEUROINFLAM\_V1.0 | Pik3r2 | mRNA | 0 |  | Insulin Signaling;Innate Immune Response;Growth Factor Signaling;Cytokine Signaling;Autophagy;Apoptosis;Angiogenesis;Adaptive Immune Response | mmu04012;mmu04014;mmu04015;mmu04022;mmu04024;mmu04062;mmu04066;mmu04068;mmu04070;mmu04071;mmu04072;mmu04150;mmu04151;mmu04152;mmu04210;mmu04211;mmu04213;mmu04261;mmu04370;mmu04380;mmu04510;mmu04550;mmu04611;mmu04620;mmu04630;mmu04650;mmu04660;mmu04662;mmu04664;mmu04666;mmu04668;mmu04670;mmu04722;mmu04725;mmu04750;mmu04810;mmu04910;mmu04914;mmu04915;mmu04917;mmu04919;mmu04921;mmu04923;mmu04930;mmu04931;mmu04932;mmu04933;mmu04960;mmu04973;mmu05100;mmu05142;mmu05145;mmu05146;mmu05160;mmu05161;mmu05162;mmu05164;mmu05166;mmu05169;mmu05200;mmu05203;mmu05205;mmu05210;mmu05211;mmu05212;mmu05213;mmu05214;mmu05215;mmu05218;mmu05220;mmu05221;mmu05222;mmu05223;mmu05230;mmu05231 |  | Pik3r2 | slateblue2 | 16 | -4.26229508196721 |
  | Nefl-mRNA | -0.522 | 0.124 | -0.764 | -0.28 | 0.696 | 0.589 | 0.824 | 0.000651 | 0.0215 | lm.nb | Adaptive Immune Response, Angiogenesis, Cytokine Signaling, Growth Factor Signaling, Insulin Signaling, Neurons and Neurotransmission | NS\_MM\_NEUROINFLAM\_V1.0 | Nefl | mRNA | 0 |  | Neurons and Neurotransmission;Insulin Signaling;Growth Factor Signaling;Cytokine Signaling;Angiogenesis;Adaptive Immune Response | mmu05014 |  | Nefl | slateblue2 | 16 | -4.20967741935484 |
  | Akt2-mRNA | -0.592 | 0.142 | -0.869 | -0.314 | 0.664 | 0.547 | 0.804 | 0.000713 | 0.022 | loglinear | Adaptive Immune Response, Angiogenesis, Apoptosis, Autophagy, Carbohydrate Metabolism, Cytokine Signaling, Growth Factor Signaling, Innate Immune Response, Insulin Signaling, Neurons and Neurotransmission, Wnt | NS\_MM\_NEUROINFLAM\_V1.0 | Akt2 | mRNA | 0 |  | Wnt;Neurons and Neurotransmission;Insulin Signaling;Innate Immune Response;Growth Factor Signaling;Cytokine Signaling;Carbohydrate Metabolism;Autophagy;Apoptosis;Angiogenesis;Adaptive Immune Response | mmu04010;mmu04012;mmu04014;mmu04015;mmu04022;mmu04024;mmu04062;mmu04066;mmu04068;mmu04071;mmu04072;mmu04150;mmu04151;mmu04152;mmu04210;mmu04211;mmu04213;mmu04261;mmu04370;mmu04380;mmu04510;mmu04530;mmu04550;mmu04611;mmu04620;mmu04630;mmu04660;mmu04662;mmu04664;mmu04666;mmu04668;mmu04722;mmu04725;mmu04728;mmu04910;mmu04914;mmu04915;mmu04917;mmu04919;mmu04920;mmu04922;mmu04923;mmu04931;mmu04932;mmu04933;mmu04973;mmu05142;mmu05145;mmu05152;mmu05160;mmu05161;mmu05162;mmu05164;mmu05166;mmu05169;mmu05200;mmu05205;mmu05210;mmu05211;mmu05212;mmu05213;mmu05214;mmu05215;mmu05218;mmu05220;mmu05221;mmu05222;mmu05223;mmu05230;mmu05231 |  | Akt2 | slateblue2 | 16 | -4.16901408450704 |
  | Braf-mRNA | -0.643 | 0.154 | -0.945 | -0.341 | 0.64 | 0.519 | 0.789 | 0.000718 | 0.022 | lm.nb | Adaptive Immune Response, Angiogenesis, Apoptosis, Cytokine Signaling, Growth Factor Signaling, Innate Immune Response, Insulin Signaling, Neurons and Neurotransmission | NS\_MM\_NEUROINFLAM\_V1.0 | Braf | mRNA | 0 |  | Neurons and Neurotransmission;Insulin Signaling;Innate Immune Response;Growth Factor Signaling;Cytokine Signaling;Apoptosis;Angiogenesis;Adaptive Immune Response | mmu04010;mmu04012;mmu04015;mmu04024;mmu04062;mmu04068;mmu04150;mmu04270;mmu04510;mmu04650;mmu04720;mmu04722;mmu04726;mmu04730;mmu04810;mmu04910;mmu04914;mmu05034;mmu05160;mmu05200;mmu05205;mmu05210;mmu05211;mmu05212;mmu05213;mmu05214;mmu05215;mmu05216;mmu05218;mmu05219;mmu05220;mmu05221;mmu05223 |  | Braf | slateblue2 | 16 | -4.17532467532468 |
  | Dlg4-mRNA | -0.221 | 0.056 | -0.331 | -0.112 | 0.858 | 0.795 | 0.926 | 0.00114 | 0.0296 | loglinear | Adaptive Immune Response, Angiogenesis, Cytokine Signaling, Growth Factor Signaling, Insulin Signaling, Neurons and Neurotransmission | NS\_MM\_NEUROINFLAM\_V1.0 | Dlg4 | mRNA | 0 |  | Neurons and Neurotransmission;Insulin Signaling;Growth Factor Signaling;Cytokine Signaling;Angiogenesis;Adaptive Immune Response | mmu04390;mmu04724;mmu05016;mmu05030 |  | Dlg4 | slateblue2 | 16 | -3.94642857142857 |
  | Grin2b-mRNA | -0.395 | 0.101 | -0.592 | -0.197 | 0.761 | 0.663 | 0.872 | 0.00124 | 0.0314 | lm.nb | Adaptive Immune Response, Angiogenesis, Cytokine Signaling, Growth Factor Signaling, Insulin Signaling, Neurons and Neurotransmission | NS\_MM\_NEUROINFLAM\_V1.0 | Grin2b | mRNA | 0 |  | Neurons and Neurotransmission;Insulin Signaling;Growth Factor Signaling;Cytokine Signaling;Angiogenesis;Adaptive Immune Response | mmu04014;mmu04015;mmu04024;mmu04080;mmu04713;mmu04720;mmu04724;mmu04728;mmu05010;mmu05014;mmu05016;mmu05030;mmu05031;mmu05033;mmu05034;mmu05322 |  | Grin2b | slateblue2 | 16 | -3.91089108910891 |
  | Map2k1-mRNA | -1.05 | 0.32 | -1.68 | -0.425 | 0.482 | 0.312 | 0.745 | 0.00463 | 0.0883 | lm.nb | Adaptive Immune Response, Angiogenesis, Autophagy, Cytokine Signaling, Growth Factor Signaling, Innate Immune Response, Insulin Signaling, Microglia Function | NS\_MM\_NEUROINFLAM\_V1.0 | Map2k1 | mRNA | 0 |  | Microglia Function;Insulin Signaling;Innate Immune Response;Growth Factor Signaling;Cytokine Signaling;Autophagy;Angiogenesis;Adaptive Immune Response | mmu04010;mmu04012;mmu04014;mmu04015;mmu04022;mmu04024;mmu04062;mmu04066;mmu04068;mmu04071;mmu04072;mmu04114;mmu04151;mmu04270;mmu04370;mmu04380;mmu04510;mmu04540;mmu04550;mmu04620;mmu04650;mmu04660;mmu04662;mmu04664;mmu04666;mmu04668;mmu04720;mmu04722;mmu04725;mmu04726;mmu04730;mmu04810;mmu04910;mmu04912;mmu04914;mmu04915;mmu04916;mmu04917;mmu04919;mmu04921;mmu05020;mmu05034;mmu05161;mmu05164;mmu05200;mmu05205;mmu05206;mmu05210;mmu05211;mmu05212;mmu05213;mmu05214;mmu05215;mmu05216;mmu05218;mmu05219;mmu05220;mmu05221;mmu05223;mmu05230;mmu05231 |  | Map2k1 | slateblue3 | 16 | -3.28125 |
  | Pik3cb-mRNA | -0.376 | 0.116 | -0.604 | -0.148 | 0.771 | 0.658 | 0.902 | 0.0052 | 0.0976 | loglinear | Adaptive Immune Response, Angiogenesis, Apoptosis, Autophagy, Carbohydrate Metabolism, Cytokine Signaling, Growth Factor Signaling, Innate Immune Response, Insulin Signaling, Lipid Metabolism | NS\_MM\_NEUROINFLAM\_V1.0 | Pik3cb | mRNA | 0 |  | Lipid Metabolism;Insulin Signaling;Innate Immune Response;Growth Factor Signaling;Cytokine Signaling;Carbohydrate Metabolism;Autophagy;Apoptosis;Angiogenesis;Adaptive Immune Response | mmu00562;mmu04012;mmu04014;mmu04015;mmu04022;mmu04024;mmu04062;mmu04066;mmu04068;mmu04070;mmu04071;mmu04072;mmu04150;mmu04151;mmu04152;mmu04210;mmu04211;mmu04213;mmu04261;mmu04370;mmu04380;mmu04510;mmu04550;mmu04611;mmu04620;mmu04630;mmu04650;mmu04660;mmu04662;mmu04664;mmu04666;mmu04668;mmu04670;mmu04722;mmu04725;mmu04750;mmu04810;mmu04910;mmu04914;mmu04915;mmu04917;mmu04919;mmu04921;mmu04923;mmu04930;mmu04931;mmu04932;mmu04933;mmu04960;mmu04973;mmu05100;mmu05142;mmu05145;mmu05146;mmu05160;mmu05161;mmu05162;mmu05164;mmu05166;mmu05169;mmu05200;mmu05203;mmu05205;mmu05210;mmu05211;mmu05212;mmu05213;mmu05214;mmu05215;mmu05218;mmu05220;mmu05221;mmu05222;mmu05223;mmu05230;mmu05231 |  | Pik3cb | slateblue4 | 16 | -3.24137931034483 |
  | Atp6v0e-mRNA | 0.372 | 0.117 | 0.142 | 0.601 | 1.29 | 1.1 | 1.52 | 0.0059 | 0.109 | loglinear | Growth Factor Signaling, Insulin Signaling, Microglia Function | NS\_MM\_NEUROINFLAM\_V1.0 | Atp6v0e | mRNA | 0 |  | Microglia Function;Insulin Signaling;Growth Factor Signaling | mmu04145;mmu04721;mmu05323 |  | Atp6v0e | slateblue4 | 16 | 3.17948717948718 |
  | Atp6v1a-mRNA | -0.192 | 0.0628 | -0.315 | -0.069 | 0.875 | 0.804 | 0.953 | 0.00749 | 0.13 | loglinear | Growth Factor Signaling, Insulin Signaling, Microglia Function | NS\_MM\_NEUROINFLAM\_V1.0 | Atp6v1a | mRNA | 0 |  | Microglia Function;Insulin Signaling;Growth Factor Signaling | mmu04145;mmu04721;mmu05323 |  | Atp6v1a | slateblue4 | 16 | -3.05732484076433 |
  | Pik3ca-mRNA | -0.277 | 0.107 | -0.487 | -0.0672 | 0.825 | 0.714 | 0.954 | 0.0198 | 0.276 | lm.nb | Adaptive Immune Response, Angiogenesis, Apoptosis, Autophagy, Carbohydrate Metabolism, Cytokine Signaling, Growth Factor Signaling, Innate Immune Response, Insulin Signaling, Lipid Metabolism | NS\_MM\_NEUROINFLAM\_V1.0 | Pik3ca | mRNA | 0 |  | Lipid Metabolism;Insulin Signaling;Innate Immune Response;Growth Factor Signaling;Cytokine Signaling;Carbohydrate Metabolism;Autophagy;Apoptosis;Angiogenesis;Adaptive Immune Response | mmu00562;mmu04012;mmu04014;mmu04015;mmu04022;mmu04024;mmu04062;mmu04066;mmu04068;mmu04070;mmu04071;mmu04072;mmu04150;mmu04151;mmu04152;mmu04210;mmu04211;mmu04213;mmu04261;mmu04370;mmu04380;mmu04510;mmu04550;mmu04611;mmu04620;mmu04630;mmu04650;mmu04660;mmu04662;mmu04664;mmu04666;mmu04668;mmu04670;mmu04722;mmu04725;mmu04750;mmu04810;mmu04910;mmu04914;mmu04915;mmu04917;mmu04919;mmu04921;mmu04923;mmu04930;mmu04931;mmu04932;mmu04933;mmu04960;mmu04973;mmu05100;mmu05142;mmu05145;mmu05146;mmu05160;mmu05161;mmu05162;mmu05164;mmu05166;mmu05169;mmu05200;mmu05203;mmu05205;mmu05210;mmu05211;mmu05212;mmu05213;mmu05214;mmu05215;mmu05218;mmu05220;mmu05221;mmu05222;mmu05223;mmu05230;mmu05231 |  | Pik3ca | slateblue4 | 16 | -2.58878504672897 |
  | Akt1-mRNA | 0.203 | 0.107 | -0.00674 | 0.413 | 1.15 | 0.995 | 1.33 | 0.076 | 0.823 | loglinear | Adaptive Immune Response, Angiogenesis, Apoptosis, Autophagy, Carbohydrate Metabolism, Cellular Stress, Cytokine Signaling, Growth Factor Signaling, Innate Immune Response, Insulin Signaling, Neurons and Neurotransmission, Wnt | NS\_MM\_NEUROINFLAM\_V1.0 | Akt1 | mRNA | 0 |  | Wnt;Neurons and Neurotransmission;Insulin Signaling;Innate Immune Response;Growth Factor Signaling;Cytokine Signaling;Cellular Stress;Carbohydrate Metabolism;Autophagy;Apoptosis;Angiogenesis;Adaptive Immune Response | mmu04010;mmu04012;mmu04014;mmu04015;mmu04022;mmu04024;mmu04062;mmu04066;mmu04068;mmu04071;mmu04072;mmu04150;mmu04151;mmu04152;mmu04210;mmu04211;mmu04213;mmu04261;mmu04370;mmu04380;mmu04510;mmu04530;mmu04550;mmu04611;mmu04620;mmu04630;mmu04660;mmu04662;mmu04664;mmu04666;mmu04668;mmu04722;mmu04725;mmu04728;mmu04910;mmu04914;mmu04915;mmu04917;mmu04919;mmu04920;mmu04922;mmu04923;mmu04931;mmu04932;mmu04933;mmu04973;mmu05142;mmu05145;mmu05152;mmu05160;mmu05161;mmu05162;mmu05164;mmu05166;mmu05169;mmu05200;mmu05205;mmu05210;mmu05211;mmu05212;mmu05213;mmu05214;mmu05215;mmu05218;mmu05220;mmu05221;mmu05222;mmu05223;mmu05230;mmu05231 |  | Akt1 | azure4 | 1 | 1.89719626168224 |
  | Egfr-mRNA | 0.527 | 0.287 | -0.036 | 1.09 | 1.44 | 0.975 | 2.13 | 0.0852 | 0.909 | lm.nb | Adaptive Immune Response, Angiogenesis, Astrocyte Function, Cytokine Signaling, Growth Factor Signaling, Insulin Signaling | NS\_MM\_NEUROINFLAM\_V1.0 | Egfr | mRNA | 0 |  | Insulin Signaling;Growth Factor Signaling;Cytokine Signaling;Astrocyte Function;Angiogenesis;Adaptive Immune Response | mmu04010;mmu04012;mmu04014;mmu04015;mmu04020;mmu04060;mmu04066;mmu04068;mmu04072;mmu04144;mmu04151;mmu04510;mmu04520;mmu04540;mmu04810;mmu04912;mmu04915;mmu04921;mmu05160;mmu05200;mmu05205;mmu05206;mmu05212;mmu05213;mmu05214;mmu05215;mmu05218;mmu05219;mmu05223;mmu05230;mmu05231 | Astrocytes | Egfr | azure4 | 1 | 1.83623693379791 |
  | Csk-mRNA | -0.205 | 0.118 | -0.436 | 0.0271 | 0.868 | 0.739 | 1.02 | 0.103 | 1 | lm.nb | Adaptive Immune Response, Angiogenesis, Autophagy, Growth Factor Signaling, Insulin Signaling | NS\_MM\_NEUROINFLAM\_V1.0 | Csk | mRNA | 0 |  | Insulin Signaling;Growth Factor Signaling;Autophagy;Angiogenesis;Adaptive Immune Response |  |  | Csk | azure4 | 1 | -1.73728813559322 |
  | Eef2k-mRNA | -0.433 | 0.269 | -0.961 | 0.0941 | 0.741 | 0.514 | 1.07 | 0.127 | 1 | lm.nb | Growth Factor Signaling, Insulin Signaling, Microglia Function | NS\_MM\_NEUROINFLAM\_V1.0 | Eef2k | mRNA | 0 |  | Microglia Function;Insulin Signaling;Growth Factor Signaling | mmu04152;mmu04921 |  | Eef2k | azure4 | 1 | -1.60966542750929 |
  | Mafb-mRNA | -0.185 | 0.126 | -0.432 | 0.0629 | 0.88 | 0.741 | 1.04 | 0.163 | 1 | loglinear | Insulin Signaling | NS\_MM\_NEUROINFLAM\_V1.0 | Mafb | mRNA | 0 |  | Insulin Signaling |  |  | Mafb | azure4 | 1 | -1.46825396825397 |
  | Fyn-mRNA | -0.0834 | 0.0637 | -0.208 | 0.0415 | 0.944 | 0.866 | 1.03 | 0.209 | 1 | loglinear | Adaptive Immune Response, Angiogenesis, Autophagy, Cytokine Signaling, Growth Factor Signaling, Innate Immune Response, Insulin Signaling, Microglia Function | NS\_MM\_NEUROINFLAM\_V1.0 | Fyn | mRNA | 0 |  | Microglia Function;Insulin Signaling;Innate Immune Response;Growth Factor Signaling;Cytokine Signaling;Autophagy;Angiogenesis;Adaptive Immune Response | mmu04071;mmu04072;mmu04360;mmu04380;mmu04510;mmu04520;mmu04611;mmu04650;mmu04660;mmu04664;mmu04725;mmu05020;mmu05162;mmu05416 |  | Fyn | azure4 | 1 | -1.30926216640502 |

  ##### DE Results - Insulin Signaling genes - DPI: 2 vs.CTRL

  Table displaying each sample's global significance scores and directed global significance scores as defined in the heatmaps above. The global significance score is calculated as the square root of the mean squared t-statistic for the genes in a gene set, with t-statistics coming from the linear regression underlying our differential expression analysis. The directed global significance score is calculated as the square root of the mean signed squared t-statistic for the genes in a gene set, with t-statistics coming from the linear regression underlying our differential expression analysis.

- **Volcano Plot: DPI: 3 vs.CTRL  
  More Plot Information**


  ##### Volcano Plot: DPI: 3 vs.CTRL

  Volcano plot displaying each gene's -log10(p-value) and log2 fold change for the selected covariate. Highly statistically significant genes fall at the top of the plot, and highly differentially expressed genes fall to either side. Genes within the selected gene set are highlighted in orange. Horizontal lines indicate various False Discovery Rate (FDR) thresholds.
- **DE Results - Insulin Signaling genes - DPI: 3 vs.CTRL  
  More Plot Information  Download CSV Data**

  | Probe Label | Log2 fold change | std error (log2) | Lower confidence limit (log2) | Upper confidence limit (log2) | Linear fold change | Lower confidence limit (linear) | Upper confidence limit (linear) | P-value | BY.p.value | method | Gene.sets | Codeset.Name | Probe.Label | Analyte.Type | Is.Control | Control.Type | Probe.Annotation | KEGG.Pathways | Cell.Type | Official.Gene.Name | volcanocol | volcanopch | tstats.all |
  | --- | --- | --- | --- | --- | --- | --- | --- | --- | --- | --- | --- | --- | --- | --- | --- | --- | --- | --- | --- | --- | --- | --- | --- |
  | Psmb8-mRNA | 2.99 | 0.346 | 2.31 | 3.67 | 7.95 | 4.97 | 12.7 | 1.99e-07 | 3.2e-05 | lm.nb | Adaptive Immune Response, Angiogenesis, Apoptosis, Astrocyte Function, Cell Cycle, Cytokine Signaling, Growth Factor Signaling, Inflammatory Signaling, Insulin Signaling, Microglia Function, NF-kB, Wnt | NS\_MM\_NEUROINFLAM\_V1.0 | Psmb8 | mRNA | 0 |  | Wnt;NF-kB;Microglia Function;Insulin Signaling;Inflammatory Signaling;Growth Factor Signaling;Cytokine Signaling;Cell Cycle;Astrocyte Function;Apoptosis;Angiogenesis;Adaptive Immune Response |  |  | Psmb8 | slateblue1 | 16 | 8.64161849710983 |
  | Atp6v0e-mRNA | 0.678 | 0.117 | 0.448 | 0.907 | 1.6 | 1.36 | 1.88 | 2.78e-05 | 0.00149 | loglinear | Growth Factor Signaling, Insulin Signaling, Microglia Function | NS\_MM\_NEUROINFLAM\_V1.0 | Atp6v0e | mRNA | 0 |  | Microglia Function;Insulin Signaling;Growth Factor Signaling | mmu04145;mmu04721;mmu05323 |  | Atp6v0e | slateblue1 | 16 | 5.79487179487179 |
  | Akt2-mRNA | -0.766 | 0.142 | -1.04 | -0.488 | 0.588 | 0.485 | 0.713 | 5.85e-05 | 0.00265 | loglinear | Adaptive Immune Response, Angiogenesis, Apoptosis, Autophagy, Carbohydrate Metabolism, Cytokine Signaling, Growth Factor Signaling, Innate Immune Response, Insulin Signaling, Neurons and Neurotransmission, Wnt | NS\_MM\_NEUROINFLAM\_V1.0 | Akt2 | mRNA | 0 |  | Wnt;Neurons and Neurotransmission;Insulin Signaling;Innate Immune Response;Growth Factor Signaling;Cytokine Signaling;Carbohydrate Metabolism;Autophagy;Apoptosis;Angiogenesis;Adaptive Immune Response | mmu04010;mmu04012;mmu04014;mmu04015;mmu04022;mmu04024;mmu04062;mmu04066;mmu04068;mmu04071;mmu04072;mmu04150;mmu04151;mmu04152;mmu04210;mmu04211;mmu04213;mmu04261;mmu04370;mmu04380;mmu04510;mmu04530;mmu04550;mmu04611;mmu04620;mmu04630;mmu04660;mmu04662;mmu04664;mmu04666;mmu04668;mmu04722;mmu04725;mmu04728;mmu04910;mmu04914;mmu04915;mmu04917;mmu04919;mmu04920;mmu04922;mmu04923;mmu04931;mmu04932;mmu04933;mmu04973;mmu05142;mmu05145;mmu05152;mmu05160;mmu05161;mmu05162;mmu05164;mmu05166;mmu05169;mmu05200;mmu05205;mmu05210;mmu05211;mmu05212;mmu05213;mmu05214;mmu05215;mmu05218;mmu05220;mmu05221;mmu05222;mmu05223;mmu05230;mmu05231 |  | Akt2 | slateblue1 | 16 | -5.3943661971831 |
  | Igf1r-mRNA | 0.603 | 0.156 | 0.297 | 0.908 | 1.52 | 1.23 | 1.88 | 0.00137 | 0.0347 | lm.nb | Apoptosis, Autophagy, Carbohydrate Metabolism, Growth Factor Signaling, Insulin Signaling | NS\_MM\_NEUROINFLAM\_V1.0 | Igf1r | mRNA | 0 |  | Insulin Signaling;Growth Factor Signaling;Carbohydrate Metabolism;Autophagy;Apoptosis | mmu04014;mmu04015;mmu04066;mmu04068;mmu04114;mmu04144;mmu04151;mmu04152;mmu04211;mmu04213;mmu04510;mmu04520;mmu04550;mmu04730;mmu04913;mmu04914;mmu05200;mmu05202;mmu05205;mmu05214;mmu05215;mmu05218 |  | Igf1r | slateblue2 | 16 | 3.86538461538462 |
  | Dlg4-mRNA | -0.204 | 0.056 | -0.313 | -0.0938 | 0.868 | 0.805 | 0.937 | 0.00223 | 0.0534 | loglinear | Adaptive Immune Response, Angiogenesis, Cytokine Signaling, Growth Factor Signaling, Insulin Signaling, Neurons and Neurotransmission | NS\_MM\_NEUROINFLAM\_V1.0 | Dlg4 | mRNA | 0 |  | Neurons and Neurotransmission;Insulin Signaling;Growth Factor Signaling;Cytokine Signaling;Angiogenesis;Adaptive Immune Response | mmu04390;mmu04724;mmu05016;mmu05030 |  | Dlg4 | slateblue3 | 16 | -3.64285714285714 |
  | Csk-mRNA | -0.423 | 0.12 | -0.658 | -0.188 | 0.746 | 0.634 | 0.878 | 0.00282 | 0.063 | lm.nb | Adaptive Immune Response, Angiogenesis, Autophagy, Growth Factor Signaling, Insulin Signaling | NS\_MM\_NEUROINFLAM\_V1.0 | Csk | mRNA | 0 |  | Insulin Signaling;Growth Factor Signaling;Autophagy;Angiogenesis;Adaptive Immune Response |  |  | Csk | slateblue3 | 16 | -3.525 |
  | Nefl-mRNA | -0.411 | 0.123 | -0.653 | -0.169 | 0.752 | 0.636 | 0.889 | 0.00423 | 0.0899 | lm.nb | Adaptive Immune Response, Angiogenesis, Cytokine Signaling, Growth Factor Signaling, Insulin Signaling, Neurons and Neurotransmission | NS\_MM\_NEUROINFLAM\_V1.0 | Nefl | mRNA | 0 |  | Neurons and Neurotransmission;Insulin Signaling;Growth Factor Signaling;Cytokine Signaling;Angiogenesis;Adaptive Immune Response | mmu05014 |  | Nefl | slateblue3 | 16 | -3.34146341463415 |
  | Pik3ca-mRNA | -0.344 | 0.107 | -0.554 | -0.134 | 0.788 | 0.681 | 0.911 | 0.00549 | 0.111 | lm.nb | Adaptive Immune Response, Angiogenesis, Apoptosis, Autophagy, Carbohydrate Metabolism, Cytokine Signaling, Growth Factor Signaling, Innate Immune Response, Insulin Signaling, Lipid Metabolism | NS\_MM\_NEUROINFLAM\_V1.0 | Pik3ca | mRNA | 0 |  | Lipid Metabolism;Insulin Signaling;Innate Immune Response;Growth Factor Signaling;Cytokine Signaling;Carbohydrate Metabolism;Autophagy;Apoptosis;Angiogenesis;Adaptive Immune Response | mmu00562;mmu04012;mmu04014;mmu04015;mmu04022;mmu04024;mmu04062;mmu04066;mmu04068;mmu04070;mmu04071;mmu04072;mmu04150;mmu04151;mmu04152;mmu04210;mmu04211;mmu04213;mmu04261;mmu04370;mmu04380;mmu04510;mmu04550;mmu04611;mmu04620;mmu04630;mmu04650;mmu04660;mmu04662;mmu04664;mmu04666;mmu04668;mmu04670;mmu04722;mmu04725;mmu04750;mmu04810;mmu04910;mmu04914;mmu04915;mmu04917;mmu04919;mmu04921;mmu04923;mmu04930;mmu04931;mmu04932;mmu04933;mmu04960;mmu04973;mmu05100;mmu05142;mmu05145;mmu05146;mmu05160;mmu05161;mmu05162;mmu05164;mmu05166;mmu05169;mmu05200;mmu05203;mmu05205;mmu05210;mmu05211;mmu05212;mmu05213;mmu05214;mmu05215;mmu05218;mmu05220;mmu05221;mmu05222;mmu05223;mmu05230;mmu05231 |  | Pik3ca | slateblue4 | 16 | -3.21495327102804 |
  | Mafb-mRNA | -0.35 | 0.126 | -0.597 | -0.102 | 0.785 | 0.661 | 0.931 | 0.0136 | 0.237 | loglinear | Insulin Signaling | NS\_MM\_NEUROINFLAM\_V1.0 | Mafb | mRNA | 0 |  | Insulin Signaling |  |  | Mafb | slateblue4 | 16 | -2.77777777777778 |
  | Grin2b-mRNA | -0.278 | 0.101 | -0.476 | -0.0811 | 0.825 | 0.719 | 0.945 | 0.0138 | 0.237 | lm.nb | Adaptive Immune Response, Angiogenesis, Cytokine Signaling, Growth Factor Signaling, Insulin Signaling, Neurons and Neurotransmission | NS\_MM\_NEUROINFLAM\_V1.0 | Grin2b | mRNA | 0 |  | Neurons and Neurotransmission;Insulin Signaling;Growth Factor Signaling;Cytokine Signaling;Angiogenesis;Adaptive Immune Response | mmu04014;mmu04015;mmu04024;mmu04080;mmu04713;mmu04720;mmu04724;mmu04728;mmu05010;mmu05014;mmu05016;mmu05030;mmu05031;mmu05033;mmu05034;mmu05322 |  | Grin2b | slateblue4 | 16 | -2.75247524752475 |
  | Dusp7-mRNA | -0.161 | 0.0611 | -0.281 | -0.0413 | 0.894 | 0.823 | 0.972 | 0.018 | 0.294 | loglinear | Adaptive Immune Response, Angiogenesis, Cytokine Signaling, Growth Factor Signaling, Innate Immune Response, Insulin Signaling, Microglia Function | NS\_MM\_NEUROINFLAM\_V1.0 | Dusp7 | mRNA | 0 |  | Microglia Function;Insulin Signaling;Innate Immune Response;Growth Factor Signaling;Cytokine Signaling;Angiogenesis;Adaptive Immune Response | mmu04010 |  | Dusp7 | slateblue4 | 16 | -2.63502454991817 |
  | Grin2a-mRNA | 0.253 | 0.0975 | 0.0617 | 0.444 | 1.19 | 1.04 | 1.36 | 0.0196 | 0.314 | loglinear | Adaptive Immune Response, Angiogenesis, Cytokine Signaling, Growth Factor Signaling, Insulin Signaling, Neurons and Neurotransmission | NS\_MM\_NEUROINFLAM\_V1.0 | Grin2a | mRNA | 0 |  | Neurons and Neurotransmission;Insulin Signaling;Growth Factor Signaling;Cytokine Signaling;Angiogenesis;Adaptive Immune Response | mmu04014;mmu04015;mmu04020;mmu04024;mmu04080;mmu04713;mmu04720;mmu04724;mmu04728;mmu05010;mmu05014;mmu05030;mmu05031;mmu05033;mmu05034;mmu05322 |  | Grin2a | slateblue4 | 16 | 2.59487179487179 |
  | Pik3r2-mRNA | -0.181 | 0.0793 | -0.337 | -0.0258 | 0.882 | 0.792 | 0.982 | 0.0363 | 0.503 | loglinear | Adaptive Immune Response, Angiogenesis, Apoptosis, Autophagy, Cytokine Signaling, Growth Factor Signaling, Innate Immune Response, Insulin Signaling | NS\_MM\_NEUROINFLAM\_V1.0 | Pik3r2 | mRNA | 0 |  | Insulin Signaling;Innate Immune Response;Growth Factor Signaling;Cytokine Signaling;Autophagy;Apoptosis;Angiogenesis;Adaptive Immune Response | mmu04012;mmu04014;mmu04015;mmu04022;mmu04024;mmu04062;mmu04066;mmu04068;mmu04070;mmu04071;mmu04072;mmu04150;mmu04151;mmu04152;mmu04210;mmu04211;mmu04213;mmu04261;mmu04370;mmu04380;mmu04510;mmu04550;mmu04611;mmu04620;mmu04630;mmu04650;mmu04660;mmu04662;mmu04664;mmu04666;mmu04668;mmu04670;mmu04722;mmu04725;mmu04750;mmu04810;mmu04910;mmu04914;mmu04915;mmu04917;mmu04919;mmu04921;mmu04923;mmu04930;mmu04931;mmu04932;mmu04933;mmu04960;mmu04973;mmu05100;mmu05142;mmu05145;mmu05146;mmu05160;mmu05161;mmu05162;mmu05164;mmu05166;mmu05169;mmu05200;mmu05203;mmu05205;mmu05210;mmu05211;mmu05212;mmu05213;mmu05214;mmu05215;mmu05218;mmu05220;mmu05221;mmu05222;mmu05223;mmu05230;mmu05231 |  | Pik3r2 | azure4 | 1 | -2.28247162673392 |
  | Atp6v1a-mRNA | -0.14 | 0.0628 | -0.263 | -0.0171 | 0.907 | 0.833 | 0.988 | 0.0403 | 0.544 | loglinear | Growth Factor Signaling, Insulin Signaling, Microglia Function | NS\_MM\_NEUROINFLAM\_V1.0 | Atp6v1a | mRNA | 0 |  | Microglia Function;Insulin Signaling;Growth Factor Signaling | mmu04145;mmu04721;mmu05323 |  | Atp6v1a | azure4 | 1 | -2.22929936305733 |
  | Fyn-mRNA | -0.123 | 0.0637 | -0.248 | 0.002 | 0.918 | 0.842 | 1 | 0.0717 | 0.896 | loglinear | Adaptive Immune Response, Angiogenesis, Autophagy, Cytokine Signaling, Growth Factor Signaling, Innate Immune Response, Insulin Signaling, Microglia Function | NS\_MM\_NEUROINFLAM\_V1.0 | Fyn | mRNA | 0 |  | Microglia Function;Insulin Signaling;Innate Immune Response;Growth Factor Signaling;Cytokine Signaling;Autophagy;Angiogenesis;Adaptive Immune Response | mmu04071;mmu04072;mmu04360;mmu04380;mmu04510;mmu04520;mmu04611;mmu04650;mmu04660;mmu04664;mmu04725;mmu05020;mmu05162;mmu05416 |  | Fyn | azure4 | 1 | -1.9309262166405 |
  | Pik3cb-mRNA | -0.207 | 0.116 | -0.435 | 0.0205 | 0.866 | 0.74 | 1.01 | 0.0934 | 1 | loglinear | Adaptive Immune Response, Angiogenesis, Apoptosis, Autophagy, Carbohydrate Metabolism, Cytokine Signaling, Growth Factor Signaling, Innate Immune Response, Insulin Signaling, Lipid Metabolism | NS\_MM\_NEUROINFLAM\_V1.0 | Pik3cb | mRNA | 0 |  | Lipid Metabolism;Insulin Signaling;Innate Immune Response;Growth Factor Signaling;Cytokine Signaling;Carbohydrate Metabolism;Autophagy;Apoptosis;Angiogenesis;Adaptive Immune Response | mmu00562;mmu04012;mmu04014;mmu04015;mmu04022;mmu04024;mmu04062;mmu04066;mmu04068;mmu04070;mmu04071;mmu04072;mmu04150;mmu04151;mmu04152;mmu04210;mmu04211;mmu04213;mmu04261;mmu04370;mmu04380;mmu04510;mmu04550;mmu04611;mmu04620;mmu04630;mmu04650;mmu04660;mmu04662;mmu04664;mmu04666;mmu04668;mmu04670;mmu04722;mmu04725;mmu04750;mmu04810;mmu04910;mmu04914;mmu04915;mmu04917;mmu04919;mmu04921;mmu04923;mmu04930;mmu04931;mmu04932;mmu04933;mmu04960;mmu04973;mmu05100;mmu05142;mmu05145;mmu05146;mmu05160;mmu05161;mmu05162;mmu05164;mmu05166;mmu05169;mmu05200;mmu05203;mmu05205;mmu05210;mmu05211;mmu05212;mmu05213;mmu05214;mmu05215;mmu05218;mmu05220;mmu05221;mmu05222;mmu05223;mmu05230;mmu05231 |  | Pik3cb | azure4 | 1 | -1.78448275862069 |
  | Eef2k-mRNA | 0.452 | 0.265 | -0.0676 | 0.971 | 1.37 | 0.954 | 1.96 | 0.108 | 1 | lm.nb | Growth Factor Signaling, Insulin Signaling, Microglia Function | NS\_MM\_NEUROINFLAM\_V1.0 | Eef2k | mRNA | 0 |  | Microglia Function;Insulin Signaling;Growth Factor Signaling | mmu04152;mmu04921 |  | Eef2k | azure4 | 1 | 1.70566037735849 |
  | Map2k1-mRNA | -0.524 | 0.32 | -1.15 | 0.104 | 0.696 | 0.45 | 1.07 | 0.121 | 1 | lm.nb | Adaptive Immune Response, Angiogenesis, Autophagy, Cytokine Signaling, Growth Factor Signaling, Innate Immune Response, Insulin Signaling, Microglia Function | NS\_MM\_NEUROINFLAM\_V1.0 | Map2k1 | mRNA | 0 |  | Microglia Function;Insulin Signaling;Innate Immune Response;Growth Factor Signaling;Cytokine Signaling;Autophagy;Angiogenesis;Adaptive Immune Response | mmu04010;mmu04012;mmu04014;mmu04015;mmu04022;mmu04024;mmu04062;mmu04066;mmu04068;mmu04071;mmu04072;mmu04114;mmu04151;mmu04270;mmu04370;mmu04380;mmu04510;mmu04540;mmu04550;mmu04620;mmu04650;mmu04660;mmu04662;mmu04664;mmu04666;mmu04668;mmu04720;mmu04722;mmu04725;mmu04726;mmu04730;mmu04810;mmu04910;mmu04912;mmu04914;mmu04915;mmu04916;mmu04917;mmu04919;mmu04921;mmu05020;mmu05034;mmu05161;mmu05164;mmu05200;mmu05205;mmu05206;mmu05210;mmu05211;mmu05212;mmu05213;mmu05214;mmu05215;mmu05216;mmu05218;mmu05219;mmu05220;mmu05221;mmu05223;mmu05230;mmu05231 |  | Map2k1 | azure4 | 1 | -1.6375 |
  | Braf-mRNA | -0.251 | 0.154 | -0.552 | 0.0506 | 0.841 | 0.682 | 1.04 | 0.122 | 1 | lm.nb | Adaptive Immune Response, Angiogenesis, Apoptosis, Cytokine Signaling, Growth Factor Signaling, Innate Immune Response, Insulin Signaling, Neurons and Neurotransmission | NS\_MM\_NEUROINFLAM\_V1.0 | Braf | mRNA | 0 |  | Neurons and Neurotransmission;Insulin Signaling;Innate Immune Response;Growth Factor Signaling;Cytokine Signaling;Apoptosis;Angiogenesis;Adaptive Immune Response | mmu04010;mmu04012;mmu04015;mmu04024;mmu04062;mmu04068;mmu04150;mmu04270;mmu04510;mmu04650;mmu04720;mmu04722;mmu04726;mmu04730;mmu04810;mmu04910;mmu04914;mmu05034;mmu05160;mmu05200;mmu05205;mmu05210;mmu05211;mmu05212;mmu05213;mmu05214;mmu05215;mmu05216;mmu05218;mmu05219;mmu05220;mmu05221;mmu05223 |  | Braf | azure4 | 1 | -1.62987012987013 |
  | Erbb3-mRNA | -0.527 | 0.547 | -1.6 | 0.546 | 0.694 | 0.33 | 1.46 | 0.35 | 1 | lm.nb | Adaptive Immune Response, Angiogenesis, Cytokine Signaling, Growth Factor Signaling, Insulin Signaling, Oligodendrocyte Function | NS\_MM\_NEUROINFLAM\_V1.0 | Erbb3 | mRNA | 0 |  | Oligodendrocyte Function;Insulin Signaling;Growth Factor Signaling;Cytokine Signaling;Angiogenesis;Adaptive Immune Response | mmu04012;mmu04020;mmu04144;mmu05205;mmu05206 | Oligodendrocytes | Erbb3 | azure4 | 1 | -0.963436928702011 |

  ##### DE Results - Insulin Signaling genes - DPI: 3 vs.CTRL

  Table displaying each sample's global significance scores and directed global significance scores as defined in the heatmaps above. The global significance score is calculated as the square root of the mean squared t-statistic for the genes in a gene set, with t-statistics coming from the linear regression underlying our differential expression analysis. The directed global significance score is calculated as the square root of the mean signed squared t-statistic for the genes in a gene set, with t-statistics coming from the linear regression underlying our differential expression analysis.

DPI:
- DPI: differential expression in 1 vs. baseline of CTRL
- DPI: differential expression in 2 vs. baseline of CTRL
- DPI: differential expression in 3 vs. baseline of CTRL

- **Volcano Plot: DPI: 1 vs.CTRL  
  More Plot Information**


  ##### Volcano Plot: DPI: 1 vs.CTRL

  Volcano plot displaying each gene's -log10(p-value) and log2 fold change for the selected covariate. Highly statistically significant genes fall at the top of the plot, and highly differentially expressed genes fall to either side. Genes within the selected gene set are highlighted in orange. Horizontal lines indicate various False Discovery Rate (FDR) thresholds.
- **DE Results - Lipid Metabolism genes - DPI: 1 vs.CTRL  
  More Plot Information  Download CSV Data**

  | Probe Label | Log2 fold change | std error (log2) | Lower confidence limit (log2) | Upper confidence limit (log2) | Linear fold change | Lower confidence limit (linear) | Upper confidence limit (linear) | P-value | BY.p.value | method | Gene.sets | Codeset.Name | Probe.Label | Analyte.Type | Is.Control | Control.Type | Probe.Annotation | KEGG.Pathways | Cell.Type | Official.Gene.Name | volcanocol | volcanopch | tstats.all |
  | --- | --- | --- | --- | --- | --- | --- | --- | --- | --- | --- | --- | --- | --- | --- | --- | --- | --- | --- | --- | --- | --- | --- | --- |
  | Prkacb-mRNA | -0.327 | 0.0629 | -0.451 | -0.204 | 0.797 | 0.732 | 0.868 | 8.65e-05 | 0.0321 | loglinear | Adaptive Immune Response, Angiogenesis, Apoptosis, Autophagy, Growth Factor Signaling, Lipid Metabolism, Neurons and Neurotransmission, Wnt | NS\_MM\_NEUROINFLAM\_V1.0 | Prkacb | mRNA | 0 |  | Wnt;Neurons and Neurotransmission;Lipid Metabolism;Growth Factor Signaling;Autophagy;Apoptosis;Angiogenesis;Adaptive Immune Response | mmu04010;mmu04014;mmu04020;mmu04024;mmu04062;mmu04114;mmu04210;mmu04211;mmu04213;mmu04261;mmu04270;mmu04310;mmu04540;mmu04611;mmu04713;mmu04720;mmu04723;mmu04724;mmu04725;mmu04726;mmu04728;mmu04750;mmu04910;mmu04911;mmu04912;mmu04913;mmu04914;mmu04915;mmu04916;mmu04919;mmu04921;mmu04922;mmu04923;mmu04924;mmu04925;mmu05012;mmu05020;mmu05030;mmu05031;mmu05146;mmu05166;mmu05169;mmu05200;mmu05203;mmu05205;mmu05414 |  | Prkacb | slateblue2 | 16 | -5.19872813990461 |
  | Pten-mRNA | -0.296 | 0.0753 | -0.444 | -0.148 | 0.815 | 0.735 | 0.902 | 0.0012 | 0.109 | loglinear | Adaptive Immune Response, Autophagy, DNA Damage, Growth Factor Signaling, Lipid Metabolism | NS\_MM\_NEUROINFLAM\_V1.0 | Pten | mRNA | 0 |  | Lipid Metabolism;Growth Factor Signaling;DNA Damage;Autophagy;Adaptive Immune Response | mmu00562;mmu04068;mmu04070;mmu04071;mmu04115;mmu04150;mmu04151;mmu04510;mmu04530;mmu04931;mmu05161;mmu05200;mmu05206;mmu05213;mmu05214;mmu05215;mmu05218;mmu05222;mmu05230 |  | Pten | slateblue4 | 16 | -3.93094289508632 |
  | Pik3ca-mRNA | -0.287 | 0.107 | -0.497 | -0.0771 | 0.82 | 0.709 | 0.948 | 0.0164 | 0.417 | lm.nb | Adaptive Immune Response, Angiogenesis, Apoptosis, Autophagy, Carbohydrate Metabolism, Cytokine Signaling, Growth Factor Signaling, Innate Immune Response, Insulin Signaling, Lipid Metabolism | NS\_MM\_NEUROINFLAM\_V1.0 | Pik3ca | mRNA | 0 |  | Lipid Metabolism;Insulin Signaling;Innate Immune Response;Growth Factor Signaling;Cytokine Signaling;Carbohydrate Metabolism;Autophagy;Apoptosis;Angiogenesis;Adaptive Immune Response | mmu00562;mmu04012;mmu04014;mmu04015;mmu04022;mmu04024;mmu04062;mmu04066;mmu04068;mmu04070;mmu04071;mmu04072;mmu04150;mmu04151;mmu04152;mmu04210;mmu04211;mmu04213;mmu04261;mmu04370;mmu04380;mmu04510;mmu04550;mmu04611;mmu04620;mmu04630;mmu04650;mmu04660;mmu04662;mmu04664;mmu04666;mmu04668;mmu04670;mmu04722;mmu04725;mmu04750;mmu04810;mmu04910;mmu04914;mmu04915;mmu04917;mmu04919;mmu04921;mmu04923;mmu04930;mmu04931;mmu04932;mmu04933;mmu04960;mmu04973;mmu05100;mmu05142;mmu05145;mmu05146;mmu05160;mmu05161;mmu05162;mmu05164;mmu05166;mmu05169;mmu05200;mmu05203;mmu05205;mmu05210;mmu05211;mmu05212;mmu05213;mmu05214;mmu05215;mmu05218;mmu05220;mmu05221;mmu05222;mmu05223;mmu05230;mmu05231 |  | Pik3ca | slateblue4 | 16 | -2.68224299065421 |
  | Ugt8a-mRNA | -0.542 | 0.219 | -0.972 | -0.113 | 0.687 | 0.51 | 0.925 | 0.025 | 0.539 | lm.nb | Lipid Metabolism, Oligodendrocyte Function | NS\_MM\_NEUROINFLAM\_V1.0 | Ugt8a | mRNA | 0 |  | Oligodendrocyte Function;Lipid Metabolism | mmu00565 | Oligodendrocytes | Ugt8a | azure4 | 1 | -2.47488584474886 |
  | Gdpd2-mRNA | -0.807 | 0.337 | -1.47 | -0.147 | 0.571 | 0.361 | 0.903 | 0.0292 | 0.599 | lm.nb | Astrocyte Function, Lipid Metabolism | NS\_MM\_NEUROINFLAM\_V1.0 | Gdpd2 | mRNA | 0 |  | Lipid Metabolism;Astrocyte Function |  | Astrocytes | Gdpd2 | azure4 | 1 | -2.3946587537092 |
  | Pik3r1-mRNA | -0.169 | 0.0713 | -0.308 | -0.0288 | 0.89 | 0.808 | 0.98 | 0.0311 | 0.62 | loglinear | Adaptive Immune Response, Angiogenesis, Apoptosis, Autophagy, Carbohydrate Metabolism, Cytokine Signaling, Growth Factor Signaling, Innate Immune Response, Insulin Signaling, Lipid Metabolism | NS\_MM\_NEUROINFLAM\_V1.0 | Pik3r1 | mRNA | 0 |  | Lipid Metabolism;Insulin Signaling;Innate Immune Response;Growth Factor Signaling;Cytokine Signaling;Carbohydrate Metabolism;Autophagy;Apoptosis;Angiogenesis;Adaptive Immune Response | mmu04012;mmu04014;mmu04015;mmu04022;mmu04024;mmu04062;mmu04066;mmu04068;mmu04070;mmu04071;mmu04072;mmu04150;mmu04151;mmu04152;mmu04210;mmu04211;mmu04213;mmu04261;mmu04370;mmu04380;mmu04510;mmu04550;mmu04611;mmu04620;mmu04630;mmu04650;mmu04660;mmu04662;mmu04664;mmu04666;mmu04668;mmu04670;mmu04722;mmu04725;mmu04750;mmu04810;mmu04910;mmu04914;mmu04915;mmu04917;mmu04919;mmu04921;mmu04923;mmu04930;mmu04931;mmu04932;mmu04933;mmu04960;mmu04973;mmu05100;mmu05142;mmu05145;mmu05146;mmu05160;mmu05161;mmu05162;mmu05164;mmu05166;mmu05169;mmu05200;mmu05203;mmu05205;mmu05210;mmu05211;mmu05212;mmu05213;mmu05214;mmu05215;mmu05218;mmu05220;mmu05221;mmu05222;mmu05223;mmu05230;mmu05231 |  | Pik3r1 | azure4 | 1 | -2.37026647966339 |
  | Ep300-mRNA | -0.209 | 0.111 | -0.425 | 0.008 | 0.865 | 0.745 | 1.01 | 0.0774 | 1 | loglinear | Cell Cycle, Cellular Stress, Cytokine Signaling, DNA Damage, Epigenetic Regulation, Inflammatory Signaling, Innate Immune Response, Lipid Metabolism, Notch, Wnt | NS\_MM\_NEUROINFLAM\_V1.0 | Ep300 | mRNA | 0 |  | Wnt;Notch;Lipid Metabolism;Innate Immune Response;Inflammatory Signaling;Epigenetic Regulation;DNA Damage;Cytokine Signaling;Cellular Stress;Cell Cycle | mmu04024;mmu04066;mmu04068;mmu04110;mmu04310;mmu04330;mmu04350;mmu04520;mmu04630;mmu04720;mmu04916;mmu04919;mmu04922;mmu05016;mmu05152;mmu05161;mmu05164;mmu05166;mmu05168;mmu05169;mmu05200;mmu05203;mmu05206;mmu05211;mmu05215 |  | Ep300 | azure4 | 1 | -1.88288288288288 |
  | Crebbp-mRNA | -0.0726 | 0.0406 | -0.152 | 0.00705 | 0.951 | 0.9 | 1 | 0.093 | 1 | loglinear | Cell Cycle, Cellular Stress, Cytokine Signaling, Epigenetic Regulation, Inflammatory Signaling, Innate Immune Response, Lipid Metabolism, Notch, Wnt | NS\_MM\_NEUROINFLAM\_V1.0 | Crebbp | mRNA | 0 |  | Wnt;Notch;Lipid Metabolism;Innate Immune Response;Inflammatory Signaling;Epigenetic Regulation;Cytokine Signaling;Cellular Stress;Cell Cycle | mmu04024;mmu04066;mmu04068;mmu04110;mmu04310;mmu04330;mmu04350;mmu04520;mmu04630;mmu04720;mmu04916;mmu04919;mmu04922;mmu05016;mmu05152;mmu05161;mmu05164;mmu05166;mmu05168;mmu05169;mmu05200;mmu05203;mmu05206;mmu05211;mmu05215 |  | Crebbp | azure4 | 1 | -1.78817733990148 |
  | Pik3cb-mRNA | -0.144 | 0.116 | -0.372 | 0.0841 | 0.905 | 0.773 | 1.06 | 0.234 | 1 | loglinear | Adaptive Immune Response, Angiogenesis, Apoptosis, Autophagy, Carbohydrate Metabolism, Cytokine Signaling, Growth Factor Signaling, Innate Immune Response, Insulin Signaling, Lipid Metabolism | NS\_MM\_NEUROINFLAM\_V1.0 | Pik3cb | mRNA | 0 |  | Lipid Metabolism;Insulin Signaling;Innate Immune Response;Growth Factor Signaling;Cytokine Signaling;Carbohydrate Metabolism;Autophagy;Apoptosis;Angiogenesis;Adaptive Immune Response | mmu00562;mmu04012;mmu04014;mmu04015;mmu04022;mmu04024;mmu04062;mmu04066;mmu04068;mmu04070;mmu04071;mmu04072;mmu04150;mmu04151;mmu04152;mmu04210;mmu04211;mmu04213;mmu04261;mmu04370;mmu04380;mmu04510;mmu04550;mmu04611;mmu04620;mmu04630;mmu04650;mmu04660;mmu04662;mmu04664;mmu04666;mmu04668;mmu04670;mmu04722;mmu04725;mmu04750;mmu04810;mmu04910;mmu04914;mmu04915;mmu04917;mmu04919;mmu04921;mmu04923;mmu04930;mmu04931;mmu04932;mmu04933;mmu04960;mmu04973;mmu05100;mmu05142;mmu05145;mmu05146;mmu05160;mmu05161;mmu05162;mmu05164;mmu05166;mmu05169;mmu05200;mmu05203;mmu05205;mmu05210;mmu05211;mmu05212;mmu05213;mmu05214;mmu05215;mmu05218;mmu05220;mmu05221;mmu05222;mmu05223;mmu05230;mmu05231 |  | Pik3cb | azure4 | 1 | -1.24137931034483 |
  | Apoe-mRNA | 0.1 | 0.0826 | -0.0615 | 0.262 | 1.07 | 0.958 | 1.2 | 0.242 | 1 | loglinear | Astrocyte Function, Cellular Stress, Lipid Metabolism, Microglia Function, Neurons and Neurotransmission | NS\_MM\_NEUROINFLAM\_V1.0 | Apoe | mRNA | 0 |  | Neurons and Neurotransmission;Microglia Function;Lipid Metabolism;Cellular Stress;Astrocyte Function | mmu05010 |  | Apoe | azure4 | 1 | 1.21065375302663 |
  | Ptgs2-mRNA | -0.304 | 0.251 | -0.795 | 0.188 | 0.81 | 0.576 | 1.14 | 0.243 | 1 | lm.nb | Astrocyte Function, Cellular Stress, Innate Immune Response, Lipid Metabolism, Microglia Function, NF-kB | NS\_MM\_NEUROINFLAM\_V1.0 | Ptgs2 | mRNA | 0 |  | NF-kB;Microglia Function;Lipid Metabolism;Innate Immune Response;Cellular Stress;Astrocyte Function | mmu00590;mmu04064;mmu04370;mmu04668;mmu04723;mmu04726;mmu04913;mmu04921;mmu04923;mmu05140;mmu05200;mmu05206;mmu05222 |  | Ptgs2 | azure4 | 1 | -1.21115537848606 |
  | Fa2h-mRNA | 0.161 | 0.145 | -0.123 | 0.446 | 1.12 | 0.918 | 1.36 | 0.284 | 1 | lm.nb | Lipid Metabolism, Oligodendrocyte Function | NS\_MM\_NEUROINFLAM\_V1.0 | Fa2h | mRNA | 0 |  | Oligodendrocyte Function;Lipid Metabolism |  | Oligodendrocytes | Fa2h | azure4 | 1 | 1.11034482758621 |
  | Gba-mRNA | -0.255 | 0.239 | -0.723 | 0.213 | 0.838 | 0.606 | 1.16 | 0.302 | 1 | lm.nb | Lipid Metabolism, Microglia Function | NS\_MM\_NEUROINFLAM\_V1.0 | Gba | mRNA | 0 |  | Microglia Function;Lipid Metabolism | mmu04142 |  | Gba | azure4 | 1 | -1.06694560669456 |
  | Prkaca-mRNA | -0.0568 | 0.0577 | -0.17 | 0.0563 | 0.961 | 0.889 | 1.04 | 0.34 | 1 | loglinear | Adaptive Immune Response, Angiogenesis, Apoptosis, Autophagy, Cell Cycle, Cytokine Signaling, Growth Factor Signaling, Lipid Metabolism, Neurons and Neurotransmission, Wnt | NS\_MM\_NEUROINFLAM\_V1.0 | Prkaca | mRNA | 0 |  | Wnt;Neurons and Neurotransmission;Lipid Metabolism;Growth Factor Signaling;Cytokine Signaling;Cell Cycle;Autophagy;Apoptosis;Angiogenesis;Adaptive Immune Response | mmu04010;mmu04014;mmu04020;mmu04024;mmu04062;mmu04114;mmu04210;mmu04211;mmu04213;mmu04261;mmu04270;mmu04310;mmu04540;mmu04611;mmu04713;mmu04720;mmu04723;mmu04724;mmu04725;mmu04726;mmu04728;mmu04750;mmu04910;mmu04911;mmu04912;mmu04913;mmu04914;mmu04915;mmu04916;mmu04919;mmu04921;mmu04922;mmu04923;mmu04924;mmu04925;mmu05012;mmu05020;mmu05030;mmu05031;mmu05034;mmu05146;mmu05166;mmu05169;mmu05200;mmu05203;mmu05205;mmu05414 |  | Prkaca | azure4 | 1 | -0.984402079722704 |
  | Lsr-mRNA | 0.105 | 0.274 | -0.433 | 0.643 | 1.08 | 0.741 | 1.56 | 0.707 | 1 | lm.nb | Lipid Metabolism | NS\_MM\_NEUROINFLAM\_V1.0 | Lsr | mRNA | 0 |  | Lipid Metabolism |  | Endothelial Cells | Lsr | azure4 | 1 | 0.383211678832117 |
  | Gal3st1-mRNA | 0.0199 | 0.151 | -0.276 | 0.315 | 1.01 | 0.826 | 1.24 | 0.897 | 1 | lm.nb | Lipid Metabolism, Oligodendrocyte Function | NS\_MM\_NEUROINFLAM\_V1.0 | Gal3st1 | mRNA | 0 |  | Oligodendrocyte Function;Lipid Metabolism | mmu00565 | Oligodendrocytes | Gal3st1 | azure4 | 1 | 0.131788079470199 |

  ##### DE Results - Lipid Metabolism genes - DPI: 1 vs.CTRL

  Table displaying each sample's global significance scores and directed global significance scores as defined in the heatmaps above. The global significance score is calculated as the square root of the mean squared t-statistic for the genes in a gene set, with t-statistics coming from the linear regression underlying our differential expression analysis. The directed global significance score is calculated as the square root of the mean signed squared t-statistic for the genes in a gene set, with t-statistics coming from the linear regression underlying our differential expression analysis.

- **Volcano Plot: DPI: 2 vs.CTRL  
  More Plot Information**


  ##### Volcano Plot: DPI: 2 vs.CTRL

  Volcano plot displaying each gene's -log10(p-value) and log2 fold change for the selected covariate. Highly statistically significant genes fall at the top of the plot, and highly differentially expressed genes fall to either side. Genes within the selected gene set are highlighted in orange. Horizontal lines indicate various False Discovery Rate (FDR) thresholds.
- **DE Results - Lipid Metabolism genes - DPI: 2 vs.CTRL  
  More Plot Information  Download CSV Data**

  | Probe Label | Log2 fold change | std error (log2) | Lower confidence limit (log2) | Upper confidence limit (log2) | Linear fold change | Lower confidence limit (linear) | Upper confidence limit (linear) | P-value | BY.p.value | method | Gene.sets | Codeset.Name | Probe.Label | Analyte.Type | Is.Control | Control.Type | Probe.Annotation | KEGG.Pathways | Cell.Type | Official.Gene.Name | volcanocol | volcanopch | tstats.all |
  | --- | --- | --- | --- | --- | --- | --- | --- | --- | --- | --- | --- | --- | --- | --- | --- | --- | --- | --- | --- | --- | --- | --- | --- |
  | Ugt8a-mRNA | -1.74 | 0.224 | -2.18 | -1.3 | 0.3 | 0.221 | 0.407 | 8.23e-07 | 0.000145 | lm.nb | Lipid Metabolism, Oligodendrocyte Function | NS\_MM\_NEUROINFLAM\_V1.0 | Ugt8a | mRNA | 0 |  | Oligodendrocyte Function;Lipid Metabolism | mmu00565 | Oligodendrocytes | Ugt8a | slateblue1 | 16 | -7.76785714285714 |
  | Prkacb-mRNA | -0.322 | 0.0629 | -0.445 | -0.199 | 0.8 | 0.735 | 0.871 | 0.000104 | 0.00562 | loglinear | Adaptive Immune Response, Angiogenesis, Apoptosis, Autophagy, Growth Factor Signaling, Lipid Metabolism, Neurons and Neurotransmission, Wnt | NS\_MM\_NEUROINFLAM\_V1.0 | Prkacb | mRNA | 0 |  | Wnt;Neurons and Neurotransmission;Lipid Metabolism;Growth Factor Signaling;Autophagy;Apoptosis;Angiogenesis;Adaptive Immune Response | mmu04010;mmu04014;mmu04020;mmu04024;mmu04062;mmu04114;mmu04210;mmu04211;mmu04213;mmu04261;mmu04270;mmu04310;mmu04540;mmu04611;mmu04713;mmu04720;mmu04723;mmu04724;mmu04725;mmu04726;mmu04728;mmu04750;mmu04910;mmu04911;mmu04912;mmu04913;mmu04914;mmu04915;mmu04916;mmu04919;mmu04921;mmu04922;mmu04923;mmu04924;mmu04925;mmu05012;mmu05020;mmu05030;mmu05031;mmu05146;mmu05166;mmu05169;mmu05200;mmu05203;mmu05205;mmu05414 |  | Prkacb | slateblue1 | 16 | -5.11923688394277 |
  | Apoe-mRNA | 0.418 | 0.0826 | 0.256 | 0.58 | 1.34 | 1.19 | 1.49 | 0.000116 | 0.00609 | loglinear | Astrocyte Function, Cellular Stress, Lipid Metabolism, Microglia Function, Neurons and Neurotransmission | NS\_MM\_NEUROINFLAM\_V1.0 | Apoe | mRNA | 0 |  | Neurons and Neurotransmission;Microglia Function;Lipid Metabolism;Cellular Stress;Astrocyte Function | mmu05010 |  | Apoe | slateblue1 | 16 | 5.06053268765133 |
  | Pten-mRNA | -0.304 | 0.0753 | -0.452 | -0.156 | 0.81 | 0.731 | 0.897 | 0.000959 | 0.0266 | loglinear | Adaptive Immune Response, Autophagy, DNA Damage, Growth Factor Signaling, Lipid Metabolism | NS\_MM\_NEUROINFLAM\_V1.0 | Pten | mRNA | 0 |  | Lipid Metabolism;Growth Factor Signaling;DNA Damage;Autophagy;Adaptive Immune Response | mmu00562;mmu04068;mmu04070;mmu04071;mmu04115;mmu04150;mmu04151;mmu04510;mmu04530;mmu04931;mmu05161;mmu05200;mmu05206;mmu05213;mmu05214;mmu05215;mmu05218;mmu05222;mmu05230 |  | Pten | slateblue2 | 16 | -4.03718459495352 |
  | Fa2h-mRNA | -0.622 | 0.155 | -0.925 | -0.318 | 0.65 | 0.527 | 0.802 | 0.00101 | 0.0278 | lm.nb | Lipid Metabolism, Oligodendrocyte Function | NS\_MM\_NEUROINFLAM\_V1.0 | Fa2h | mRNA | 0 |  | Oligodendrocyte Function;Lipid Metabolism |  | Oligodendrocytes | Fa2h | slateblue2 | 16 | -4.01290322580645 |
  | Pik3cb-mRNA | -0.376 | 0.116 | -0.604 | -0.148 | 0.771 | 0.658 | 0.902 | 0.0052 | 0.0976 | loglinear | Adaptive Immune Response, Angiogenesis, Apoptosis, Autophagy, Carbohydrate Metabolism, Cytokine Signaling, Growth Factor Signaling, Innate Immune Response, Insulin Signaling, Lipid Metabolism | NS\_MM\_NEUROINFLAM\_V1.0 | Pik3cb | mRNA | 0 |  | Lipid Metabolism;Insulin Signaling;Innate Immune Response;Growth Factor Signaling;Cytokine Signaling;Carbohydrate Metabolism;Autophagy;Apoptosis;Angiogenesis;Adaptive Immune Response | mmu00562;mmu04012;mmu04014;mmu04015;mmu04022;mmu04024;mmu04062;mmu04066;mmu04068;mmu04070;mmu04071;mmu04072;mmu04150;mmu04151;mmu04152;mmu04210;mmu04211;mmu04213;mmu04261;mmu04370;mmu04380;mmu04510;mmu04550;mmu04611;mmu04620;mmu04630;mmu04650;mmu04660;mmu04662;mmu04664;mmu04666;mmu04668;mmu04670;mmu04722;mmu04725;mmu04750;mmu04810;mmu04910;mmu04914;mmu04915;mmu04917;mmu04919;mmu04921;mmu04923;mmu04930;mmu04931;mmu04932;mmu04933;mmu04960;mmu04973;mmu05100;mmu05142;mmu05145;mmu05146;mmu05160;mmu05161;mmu05162;mmu05164;mmu05166;mmu05169;mmu05200;mmu05203;mmu05205;mmu05210;mmu05211;mmu05212;mmu05213;mmu05214;mmu05215;mmu05218;mmu05220;mmu05221;mmu05222;mmu05223;mmu05230;mmu05231 |  | Pik3cb | slateblue4 | 16 | -3.24137931034483 |
  | Ep300-mRNA | -0.33 | 0.111 | -0.547 | -0.114 | 0.795 | 0.684 | 0.924 | 0.0087 | 0.144 | loglinear | Cell Cycle, Cellular Stress, Cytokine Signaling, DNA Damage, Epigenetic Regulation, Inflammatory Signaling, Innate Immune Response, Lipid Metabolism, Notch, Wnt | NS\_MM\_NEUROINFLAM\_V1.0 | Ep300 | mRNA | 0 |  | Wnt;Notch;Lipid Metabolism;Innate Immune Response;Inflammatory Signaling;Epigenetic Regulation;DNA Damage;Cytokine Signaling;Cellular Stress;Cell Cycle | mmu04024;mmu04066;mmu04068;mmu04110;mmu04310;mmu04330;mmu04350;mmu04520;mmu04630;mmu04720;mmu04916;mmu04919;mmu04922;mmu05016;mmu05152;mmu05161;mmu05164;mmu05166;mmu05168;mmu05169;mmu05200;mmu05203;mmu05206;mmu05211;mmu05215 |  | Ep300 | slateblue4 | 16 | -2.97297297297297 |
  | Prkaca-mRNA | -0.166 | 0.0577 | -0.279 | -0.0525 | 0.892 | 0.824 | 0.964 | 0.0111 | 0.179 | loglinear | Adaptive Immune Response, Angiogenesis, Apoptosis, Autophagy, Cell Cycle, Cytokine Signaling, Growth Factor Signaling, Lipid Metabolism, Neurons and Neurotransmission, Wnt | NS\_MM\_NEUROINFLAM\_V1.0 | Prkaca | mRNA | 0 |  | Wnt;Neurons and Neurotransmission;Lipid Metabolism;Growth Factor Signaling;Cytokine Signaling;Cell Cycle;Autophagy;Apoptosis;Angiogenesis;Adaptive Immune Response | mmu04010;mmu04014;mmu04020;mmu04024;mmu04062;mmu04114;mmu04210;mmu04211;mmu04213;mmu04261;mmu04270;mmu04310;mmu04540;mmu04611;mmu04713;mmu04720;mmu04723;mmu04724;mmu04725;mmu04726;mmu04728;mmu04750;mmu04910;mmu04911;mmu04912;mmu04913;mmu04914;mmu04915;mmu04916;mmu04919;mmu04921;mmu04922;mmu04923;mmu04924;mmu04925;mmu05012;mmu05020;mmu05030;mmu05031;mmu05034;mmu05146;mmu05166;mmu05169;mmu05200;mmu05203;mmu05205;mmu05414 |  | Prkaca | slateblue4 | 16 | -2.87694974003466 |
  | Pik3ca-mRNA | -0.277 | 0.107 | -0.487 | -0.0672 | 0.825 | 0.714 | 0.954 | 0.0198 | 0.276 | lm.nb | Adaptive Immune Response, Angiogenesis, Apoptosis, Autophagy, Carbohydrate Metabolism, Cytokine Signaling, Growth Factor Signaling, Innate Immune Response, Insulin Signaling, Lipid Metabolism | NS\_MM\_NEUROINFLAM\_V1.0 | Pik3ca | mRNA | 0 |  | Lipid Metabolism;Insulin Signaling;Innate Immune Response;Growth Factor Signaling;Cytokine Signaling;Carbohydrate Metabolism;Autophagy;Apoptosis;Angiogenesis;Adaptive Immune Response | mmu00562;mmu04012;mmu04014;mmu04015;mmu04022;mmu04024;mmu04062;mmu04066;mmu04068;mmu04070;mmu04071;mmu04072;mmu04150;mmu04151;mmu04152;mmu04210;mmu04211;mmu04213;mmu04261;mmu04370;mmu04380;mmu04510;mmu04550;mmu04611;mmu04620;mmu04630;mmu04650;mmu04660;mmu04662;mmu04664;mmu04666;mmu04668;mmu04670;mmu04722;mmu04725;mmu04750;mmu04810;mmu04910;mmu04914;mmu04915;mmu04917;mmu04919;mmu04921;mmu04923;mmu04930;mmu04931;mmu04932;mmu04933;mmu04960;mmu04973;mmu05100;mmu05142;mmu05145;mmu05146;mmu05160;mmu05161;mmu05162;mmu05164;mmu05166;mmu05169;mmu05200;mmu05203;mmu05205;mmu05210;mmu05211;mmu05212;mmu05213;mmu05214;mmu05215;mmu05218;mmu05220;mmu05221;mmu05222;mmu05223;mmu05230;mmu05231 |  | Pik3ca | slateblue4 | 16 | -2.58878504672897 |
  | Gal3st1-mRNA | -0.356 | 0.158 | -0.665 | -0.0472 | 0.781 | 0.631 | 0.968 | 0.0381 | 0.474 | lm.nb | Lipid Metabolism, Oligodendrocyte Function | NS\_MM\_NEUROINFLAM\_V1.0 | Gal3st1 | mRNA | 0 |  | Oligodendrocyte Function;Lipid Metabolism | mmu00565 | Oligodendrocytes | Gal3st1 | slateblue4 | 16 | -2.25316455696203 |
  | Lsr-mRNA | 0.349 | 0.269 | -0.179 | 0.876 | 1.27 | 0.884 | 1.84 | 0.213 | 1 | lm.nb | Lipid Metabolism | NS\_MM\_NEUROINFLAM\_V1.0 | Lsr | mRNA | 0 |  | Lipid Metabolism |  | Endothelial Cells | Lsr | azure4 | 1 | 1.29739776951673 |
  | Pik3r1-mRNA | 0.0869 | 0.0713 | -0.0529 | 0.227 | 1.06 | 0.964 | 1.17 | 0.241 | 1 | loglinear | Adaptive Immune Response, Angiogenesis, Apoptosis, Autophagy, Carbohydrate Metabolism, Cytokine Signaling, Growth Factor Signaling, Innate Immune Response, Insulin Signaling, Lipid Metabolism | NS\_MM\_NEUROINFLAM\_V1.0 | Pik3r1 | mRNA | 0 |  | Lipid Metabolism;Insulin Signaling;Innate Immune Response;Growth Factor Signaling;Cytokine Signaling;Carbohydrate Metabolism;Autophagy;Apoptosis;Angiogenesis;Adaptive Immune Response | mmu04012;mmu04014;mmu04015;mmu04022;mmu04024;mmu04062;mmu04066;mmu04068;mmu04070;mmu04071;mmu04072;mmu04150;mmu04151;mmu04152;mmu04210;mmu04211;mmu04213;mmu04261;mmu04370;mmu04380;mmu04510;mmu04550;mmu04611;mmu04620;mmu04630;mmu04650;mmu04660;mmu04662;mmu04664;mmu04666;mmu04668;mmu04670;mmu04722;mmu04725;mmu04750;mmu04810;mmu04910;mmu04914;mmu04915;mmu04917;mmu04919;mmu04921;mmu04923;mmu04930;mmu04931;mmu04932;mmu04933;mmu04960;mmu04973;mmu05100;mmu05142;mmu05145;mmu05146;mmu05160;mmu05161;mmu05162;mmu05164;mmu05166;mmu05169;mmu05200;mmu05203;mmu05205;mmu05210;mmu05211;mmu05212;mmu05213;mmu05214;mmu05215;mmu05218;mmu05220;mmu05221;mmu05222;mmu05223;mmu05230;mmu05231 |  | Pik3r1 | azure4 | 1 | 1.21879382889201 |
  | Gdpd2-mRNA | -0.268 | 0.322 | -0.899 | 0.362 | 0.83 | 0.536 | 1.29 | 0.417 | 1 | lm.nb | Astrocyte Function, Lipid Metabolism | NS\_MM\_NEUROINFLAM\_V1.0 | Gdpd2 | mRNA | 0 |  | Lipid Metabolism;Astrocyte Function |  | Astrocytes | Gdpd2 | azure4 | 1 | -0.832298136645963 |
  | Crebbp-mRNA | -0.0334 | 0.0406 | -0.113 | 0.0462 | 0.977 | 0.925 | 1.03 | 0.423 | 1 | loglinear | Cell Cycle, Cellular Stress, Cytokine Signaling, Epigenetic Regulation, Inflammatory Signaling, Innate Immune Response, Lipid Metabolism, Notch, Wnt | NS\_MM\_NEUROINFLAM\_V1.0 | Crebbp | mRNA | 0 |  | Wnt;Notch;Lipid Metabolism;Innate Immune Response;Inflammatory Signaling;Epigenetic Regulation;Cytokine Signaling;Cellular Stress;Cell Cycle | mmu04024;mmu04066;mmu04068;mmu04110;mmu04310;mmu04330;mmu04350;mmu04520;mmu04630;mmu04720;mmu04916;mmu04919;mmu04922;mmu05016;mmu05152;mmu05161;mmu05164;mmu05166;mmu05168;mmu05169;mmu05200;mmu05203;mmu05206;mmu05211;mmu05215 |  | Crebbp | azure4 | 1 | -0.822660098522167 |
  | Ptgs2-mRNA | 0.169 | 0.245 | -0.312 | 0.65 | 1.12 | 0.805 | 1.57 | 0.501 | 1 | lm.nb | Astrocyte Function, Cellular Stress, Innate Immune Response, Lipid Metabolism, Microglia Function, NF-kB | NS\_MM\_NEUROINFLAM\_V1.0 | Ptgs2 | mRNA | 0 |  | NF-kB;Microglia Function;Lipid Metabolism;Innate Immune Response;Cellular Stress;Astrocyte Function | mmu00590;mmu04064;mmu04370;mmu04668;mmu04723;mmu04726;mmu04913;mmu04921;mmu04923;mmu05140;mmu05200;mmu05206;mmu05222 |  | Ptgs2 | azure4 | 1 | 0.689795918367347 |
  | Gba-mRNA | -0.0904 | 0.237 | -0.556 | 0.375 | 0.939 | 0.68 | 1.3 | 0.708 | 1 | lm.nb | Lipid Metabolism, Microglia Function | NS\_MM\_NEUROINFLAM\_V1.0 | Gba | mRNA | 0 |  | Microglia Function;Lipid Metabolism | mmu04142 |  | Gba | azure4 | 1 | -0.381434599156118 |

  ##### DE Results - Lipid Metabolism genes - DPI: 2 vs.CTRL

  Table displaying each sample's global significance scores and directed global significance scores as defined in the heatmaps above. The global significance score is calculated as the square root of the mean squared t-statistic for the genes in a gene set, with t-statistics coming from the linear regression underlying our differential expression analysis. The directed global significance score is calculated as the square root of the mean signed squared t-statistic for the genes in a gene set, with t-statistics coming from the linear regression underlying our differential expression analysis.

- **Volcano Plot: DPI: 3 vs.CTRL  
  More Plot Information**


  ##### Volcano Plot: DPI: 3 vs.CTRL

  Volcano plot displaying each gene's -log10(p-value) and log2 fold change for the selected covariate. Highly statistically significant genes fall at the top of the plot, and highly differentially expressed genes fall to either side. Genes within the selected gene set are highlighted in orange. Horizontal lines indicate various False Discovery Rate (FDR) thresholds.
- **DE Results - Lipid Metabolism genes - DPI: 3 vs.CTRL  
  More Plot Information  Download CSV Data**

  | Probe Label | Log2 fold change | std error (log2) | Lower confidence limit (log2) | Upper confidence limit (log2) | Linear fold change | Lower confidence limit (linear) | Upper confidence limit (linear) | P-value | BY.p.value | method | Gene.sets | Codeset.Name | Probe.Label | Analyte.Type | Is.Control | Control.Type | Probe.Annotation | KEGG.Pathways | Cell.Type | Official.Gene.Name | volcanocol | volcanopch | tstats.all |
  | --- | --- | --- | --- | --- | --- | --- | --- | --- | --- | --- | --- | --- | --- | --- | --- | --- | --- | --- | --- | --- | --- | --- | --- |
  | Ugt8a-mRNA | -2.5 | 0.229 | -2.95 | -2.05 | 0.177 | 0.129 | 0.241 | 8.21e-09 | 3.12e-06 | lm.nb | Lipid Metabolism, Oligodendrocyte Function | NS\_MM\_NEUROINFLAM\_V1.0 | Ugt8a | mRNA | 0 |  | Oligodendrocyte Function;Lipid Metabolism | mmu00565 | Oligodendrocytes | Ugt8a | slateblue1 | 16 | -10.9170305676856 |
  | Ptgs2-mRNA | 1.88 | 0.236 | 1.41 | 2.34 | 3.67 | 2.67 | 5.06 | 5.87e-07 | 7.14e-05 | lm.nb | Astrocyte Function, Cellular Stress, Innate Immune Response, Lipid Metabolism, Microglia Function, NF-kB | NS\_MM\_NEUROINFLAM\_V1.0 | Ptgs2 | mRNA | 0 |  | NF-kB;Microglia Function;Lipid Metabolism;Innate Immune Response;Cellular Stress;Astrocyte Function | mmu00590;mmu04064;mmu04370;mmu04668;mmu04723;mmu04726;mmu04913;mmu04921;mmu04923;mmu05140;mmu05200;mmu05206;mmu05222 |  | Ptgs2 | slateblue1 | 16 | 7.96610169491525 |
  | Fa2h-mRNA | -0.935 | 0.16 | -1.25 | -0.621 | 0.523 | 0.421 | 0.65 | 2.55e-05 | 0.00141 | lm.nb | Lipid Metabolism, Oligodendrocyte Function | NS\_MM\_NEUROINFLAM\_V1.0 | Fa2h | mRNA | 0 |  | Oligodendrocyte Function;Lipid Metabolism |  | Oligodendrocytes | Fa2h | slateblue1 | 16 | -5.84375 |
  | Gal3st1-mRNA | -0.71 | 0.166 | -1.04 | -0.386 | 0.611 | 0.488 | 0.765 | 0.000561 | 0.0175 | lm.nb | Lipid Metabolism, Oligodendrocyte Function | NS\_MM\_NEUROINFLAM\_V1.0 | Gal3st1 | mRNA | 0 |  | Oligodendrocyte Function;Lipid Metabolism | mmu00565 | Oligodendrocytes | Gal3st1 | slateblue2 | 16 | -4.27710843373494 |
  | Apoe-mRNA | 0.298 | 0.0826 | 0.136 | 0.46 | 1.23 | 1.1 | 1.38 | 0.00237 | 0.055 | loglinear | Astrocyte Function, Cellular Stress, Lipid Metabolism, Microglia Function, Neurons and Neurotransmission | NS\_MM\_NEUROINFLAM\_V1.0 | Apoe | mRNA | 0 |  | Neurons and Neurotransmission;Microglia Function;Lipid Metabolism;Cellular Stress;Astrocyte Function | mmu05010 |  | Apoe | slateblue3 | 16 | 3.60774818401937 |
  | Pik3ca-mRNA | -0.344 | 0.107 | -0.554 | -0.134 | 0.788 | 0.681 | 0.911 | 0.00549 | 0.111 | lm.nb | Adaptive Immune Response, Angiogenesis, Apoptosis, Autophagy, Carbohydrate Metabolism, Cytokine Signaling, Growth Factor Signaling, Innate Immune Response, Insulin Signaling, Lipid Metabolism | NS\_MM\_NEUROINFLAM\_V1.0 | Pik3ca | mRNA | 0 |  | Lipid Metabolism;Insulin Signaling;Innate Immune Response;Growth Factor Signaling;Cytokine Signaling;Carbohydrate Metabolism;Autophagy;Apoptosis;Angiogenesis;Adaptive Immune Response | mmu00562;mmu04012;mmu04014;mmu04015;mmu04022;mmu04024;mmu04062;mmu04066;mmu04068;mmu04070;mmu04071;mmu04072;mmu04150;mmu04151;mmu04152;mmu04210;mmu04211;mmu04213;mmu04261;mmu04370;mmu04380;mmu04510;mmu04550;mmu04611;mmu04620;mmu04630;mmu04650;mmu04660;mmu04662;mmu04664;mmu04666;mmu04668;mmu04670;mmu04722;mmu04725;mmu04750;mmu04810;mmu04910;mmu04914;mmu04915;mmu04917;mmu04919;mmu04921;mmu04923;mmu04930;mmu04931;mmu04932;mmu04933;mmu04960;mmu04973;mmu05100;mmu05142;mmu05145;mmu05146;mmu05160;mmu05161;mmu05162;mmu05164;mmu05166;mmu05169;mmu05200;mmu05203;mmu05205;mmu05210;mmu05211;mmu05212;mmu05213;mmu05214;mmu05215;mmu05218;mmu05220;mmu05221;mmu05222;mmu05223;mmu05230;mmu05231 |  | Pik3ca | slateblue4 | 16 | -3.21495327102804 |
  | Prkaca-mRNA | -0.149 | 0.0577 | -0.262 | -0.0355 | 0.902 | 0.834 | 0.976 | 0.0203 | 0.322 | loglinear | Adaptive Immune Response, Angiogenesis, Apoptosis, Autophagy, Cell Cycle, Cytokine Signaling, Growth Factor Signaling, Lipid Metabolism, Neurons and Neurotransmission, Wnt | NS\_MM\_NEUROINFLAM\_V1.0 | Prkaca | mRNA | 0 |  | Wnt;Neurons and Neurotransmission;Lipid Metabolism;Growth Factor Signaling;Cytokine Signaling;Cell Cycle;Autophagy;Apoptosis;Angiogenesis;Adaptive Immune Response | mmu04010;mmu04014;mmu04020;mmu04024;mmu04062;mmu04114;mmu04210;mmu04211;mmu04213;mmu04261;mmu04270;mmu04310;mmu04540;mmu04611;mmu04713;mmu04720;mmu04723;mmu04724;mmu04725;mmu04726;mmu04728;mmu04750;mmu04910;mmu04911;mmu04912;mmu04913;mmu04914;mmu04915;mmu04916;mmu04919;mmu04921;mmu04922;mmu04923;mmu04924;mmu04925;mmu05012;mmu05020;mmu05030;mmu05031;mmu05034;mmu05146;mmu05166;mmu05169;mmu05200;mmu05203;mmu05205;mmu05414 |  | Prkaca | slateblue4 | 16 | -2.58232235701906 |
  | Lsr-mRNA | 0.621 | 0.264 | 0.103 | 1.14 | 1.54 | 1.07 | 2.2 | 0.0319 | 0.458 | lm.nb | Lipid Metabolism | NS\_MM\_NEUROINFLAM\_V1.0 | Lsr | mRNA | 0 |  | Lipid Metabolism |  | Endothelial Cells | Lsr | slateblue4 | 16 | 2.35227272727273 |
  | Gdpd2-mRNA | -0.72 | 0.334 | -1.37 | -0.0648 | 0.607 | 0.386 | 0.956 | 0.0468 | 0.614 | lm.nb | Astrocyte Function, Lipid Metabolism | NS\_MM\_NEUROINFLAM\_V1.0 | Gdpd2 | mRNA | 0 |  | Lipid Metabolism;Astrocyte Function |  | Astrocytes | Gdpd2 | azure4 | 1 | -2.15568862275449 |
  | Pik3cb-mRNA | -0.207 | 0.116 | -0.435 | 0.0205 | 0.866 | 0.74 | 1.01 | 0.0934 | 1 | loglinear | Adaptive Immune Response, Angiogenesis, Apoptosis, Autophagy, Carbohydrate Metabolism, Cytokine Signaling, Growth Factor Signaling, Innate Immune Response, Insulin Signaling, Lipid Metabolism | NS\_MM\_NEUROINFLAM\_V1.0 | Pik3cb | mRNA | 0 |  | Lipid Metabolism;Insulin Signaling;Innate Immune Response;Growth Factor Signaling;Cytokine Signaling;Carbohydrate Metabolism;Autophagy;Apoptosis;Angiogenesis;Adaptive Immune Response | mmu00562;mmu04012;mmu04014;mmu04015;mmu04022;mmu04024;mmu04062;mmu04066;mmu04068;mmu04070;mmu04071;mmu04072;mmu04150;mmu04151;mmu04152;mmu04210;mmu04211;mmu04213;mmu04261;mmu04370;mmu04380;mmu04510;mmu04550;mmu04611;mmu04620;mmu04630;mmu04650;mmu04660;mmu04662;mmu04664;mmu04666;mmu04668;mmu04670;mmu04722;mmu04725;mmu04750;mmu04810;mmu04910;mmu04914;mmu04915;mmu04917;mmu04919;mmu04921;mmu04923;mmu04930;mmu04931;mmu04932;mmu04933;mmu04960;mmu04973;mmu05100;mmu05142;mmu05145;mmu05146;mmu05160;mmu05161;mmu05162;mmu05164;mmu05166;mmu05169;mmu05200;mmu05203;mmu05205;mmu05210;mmu05211;mmu05212;mmu05213;mmu05214;mmu05215;mmu05218;mmu05220;mmu05221;mmu05222;mmu05223;mmu05230;mmu05231 |  | Pik3cb | azure4 | 1 | -1.78448275862069 |
  | Prkacb-mRNA | -0.104 | 0.0629 | -0.227 | 0.0196 | 0.931 | 0.854 | 1.01 | 0.119 | 1 | loglinear | Adaptive Immune Response, Angiogenesis, Apoptosis, Autophagy, Growth Factor Signaling, Lipid Metabolism, Neurons and Neurotransmission, Wnt | NS\_MM\_NEUROINFLAM\_V1.0 | Prkacb | mRNA | 0 |  | Wnt;Neurons and Neurotransmission;Lipid Metabolism;Growth Factor Signaling;Autophagy;Apoptosis;Angiogenesis;Adaptive Immune Response | mmu04010;mmu04014;mmu04020;mmu04024;mmu04062;mmu04114;mmu04210;mmu04211;mmu04213;mmu04261;mmu04270;mmu04310;mmu04540;mmu04611;mmu04713;mmu04720;mmu04723;mmu04724;mmu04725;mmu04726;mmu04728;mmu04750;mmu04910;mmu04911;mmu04912;mmu04913;mmu04914;mmu04915;mmu04916;mmu04919;mmu04921;mmu04922;mmu04923;mmu04924;mmu04925;mmu05012;mmu05020;mmu05030;mmu05031;mmu05146;mmu05166;mmu05169;mmu05200;mmu05203;mmu05205;mmu05414 |  | Prkacb | azure4 | 1 | -1.65341812400636 |
  | Gba-mRNA | 0.213 | 0.235 | -0.247 | 0.674 | 1.16 | 0.842 | 1.6 | 0.377 | 1 | lm.nb | Lipid Metabolism, Microglia Function | NS\_MM\_NEUROINFLAM\_V1.0 | Gba | mRNA | 0 |  | Microglia Function;Lipid Metabolism | mmu04142 |  | Gba | azure4 | 1 | 0.906382978723404 |
  | Pten-mRNA | 0.0677 | 0.0753 | -0.0799 | 0.215 | 1.05 | 0.946 | 1.16 | 0.382 | 1 | loglinear | Adaptive Immune Response, Autophagy, DNA Damage, Growth Factor Signaling, Lipid Metabolism | NS\_MM\_NEUROINFLAM\_V1.0 | Pten | mRNA | 0 |  | Lipid Metabolism;Growth Factor Signaling;DNA Damage;Autophagy;Adaptive Immune Response | mmu00562;mmu04068;mmu04070;mmu04071;mmu04115;mmu04150;mmu04151;mmu04510;mmu04530;mmu04931;mmu05161;mmu05200;mmu05206;mmu05213;mmu05214;mmu05215;mmu05218;mmu05222;mmu05230 |  | Pten | azure4 | 1 | 0.899070385126162 |
  | Pik3r1-mRNA | 0.0533 | 0.0713 | -0.0865 | 0.193 | 1.04 | 0.942 | 1.14 | 0.466 | 1 | loglinear | Adaptive Immune Response, Angiogenesis, Apoptosis, Autophagy, Carbohydrate Metabolism, Cytokine Signaling, Growth Factor Signaling, Innate Immune Response, Insulin Signaling, Lipid Metabolism | NS\_MM\_NEUROINFLAM\_V1.0 | Pik3r1 | mRNA | 0 |  | Lipid Metabolism;Insulin Signaling;Innate Immune Response;Growth Factor Signaling;Cytokine Signaling;Carbohydrate Metabolism;Autophagy;Apoptosis;Angiogenesis;Adaptive Immune Response | mmu04012;mmu04014;mmu04015;mmu04022;mmu04024;mmu04062;mmu04066;mmu04068;mmu04070;mmu04071;mmu04072;mmu04150;mmu04151;mmu04152;mmu04210;mmu04211;mmu04213;mmu04261;mmu04370;mmu04380;mmu04510;mmu04550;mmu04611;mmu04620;mmu04630;mmu04650;mmu04660;mmu04662;mmu04664;mmu04666;mmu04668;mmu04670;mmu04722;mmu04725;mmu04750;mmu04810;mmu04910;mmu04914;mmu04915;mmu04917;mmu04919;mmu04921;mmu04923;mmu04930;mmu04931;mmu04932;mmu04933;mmu04960;mmu04973;mmu05100;mmu05142;mmu05145;mmu05146;mmu05160;mmu05161;mmu05162;mmu05164;mmu05166;mmu05169;mmu05200;mmu05203;mmu05205;mmu05210;mmu05211;mmu05212;mmu05213;mmu05214;mmu05215;mmu05218;mmu05220;mmu05221;mmu05222;mmu05223;mmu05230;mmu05231 |  | Pik3r1 | azure4 | 1 | 0.747545582047686 |
  | Ep300-mRNA | -0.0339 | 0.111 | -0.251 | 0.183 | 0.977 | 0.841 | 1.14 | 0.763 | 1 | loglinear | Cell Cycle, Cellular Stress, Cytokine Signaling, DNA Damage, Epigenetic Regulation, Inflammatory Signaling, Innate Immune Response, Lipid Metabolism, Notch, Wnt | NS\_MM\_NEUROINFLAM\_V1.0 | Ep300 | mRNA | 0 |  | Wnt;Notch;Lipid Metabolism;Innate Immune Response;Inflammatory Signaling;Epigenetic Regulation;DNA Damage;Cytokine Signaling;Cellular Stress;Cell Cycle | mmu04024;mmu04066;mmu04068;mmu04110;mmu04310;mmu04330;mmu04350;mmu04520;mmu04630;mmu04720;mmu04916;mmu04919;mmu04922;mmu05016;mmu05152;mmu05161;mmu05164;mmu05166;mmu05168;mmu05169;mmu05200;mmu05203;mmu05206;mmu05211;mmu05215 |  | Ep300 | azure4 | 1 | -0.305405405405405 |
  | Crebbp-mRNA | 0.00879 | 0.0406 | -0.0708 | 0.0884 | 1.01 | 0.952 | 1.06 | 0.831 | 1 | loglinear | Cell Cycle, Cellular Stress, Cytokine Signaling, Epigenetic Regulation, Inflammatory Signaling, Innate Immune Response, Lipid Metabolism, Notch, Wnt | NS\_MM\_NEUROINFLAM\_V1.0 | Crebbp | mRNA | 0 |  | Wnt;Notch;Lipid Metabolism;Innate Immune Response;Inflammatory Signaling;Epigenetic Regulation;Cytokine Signaling;Cellular Stress;Cell Cycle | mmu04024;mmu04066;mmu04068;mmu04110;mmu04310;mmu04330;mmu04350;mmu04520;mmu04630;mmu04720;mmu04916;mmu04919;mmu04922;mmu05016;mmu05152;mmu05161;mmu05164;mmu05166;mmu05168;mmu05169;mmu05200;mmu05203;mmu05206;mmu05211;mmu05215 |  | Crebbp | azure4 | 1 | 0.216502463054187 |

  ##### DE Results - Lipid Metabolism genes - DPI: 3 vs.CTRL

  Table displaying each sample's global significance scores and directed global significance scores as defined in the heatmaps above. The global significance score is calculated as the square root of the mean squared t-statistic for the genes in a gene set, with t-statistics coming from the linear regression underlying our differential expression analysis. The directed global significance score is calculated as the square root of the mean signed squared t-statistic for the genes in a gene set, with t-statistics coming from the linear regression underlying our differential expression analysis.

DPI:
- DPI: differential expression in 1 vs. baseline of CTRL
- DPI: differential expression in 2 vs. baseline of CTRL
- DPI: differential expression in 3 vs. baseline of CTRL

- **Volcano Plot: DPI: 1 vs.CTRL  
  More Plot Information**


  ##### Volcano Plot: DPI: 1 vs.CTRL

  Volcano plot displaying each gene's -log10(p-value) and log2 fold change for the selected covariate. Highly statistically significant genes fall at the top of the plot, and highly differentially expressed genes fall to either side. Genes within the selected gene set are highlighted in orange. Horizontal lines indicate various False Discovery Rate (FDR) thresholds.
- **DE Results - Matrix Remodeling genes - DPI: 1 vs.CTRL  
  More Plot Information  Download CSV Data**

  | Probe Label | Log2 fold change | std error (log2) | Lower confidence limit (log2) | Upper confidence limit (log2) | Linear fold change | Lower confidence limit (linear) | Upper confidence limit (linear) | P-value | BY.p.value | method | Gene.sets | Codeset.Name | Probe.Label | Analyte.Type | Is.Control | Control.Type | Probe.Annotation | KEGG.Pathways | Cell.Type | Official.Gene.Name | volcanocol | volcanopch | tstats.all |
  | --- | --- | --- | --- | --- | --- | --- | --- | --- | --- | --- | --- | --- | --- | --- | --- | --- | --- | --- | --- | --- | --- | --- | --- |
  | Mmp14-mRNA | -1.57 | 0.459 | -2.47 | -0.668 | 0.338 | 0.181 | 0.629 | 0.00354 | 0.184 | lm.nb | Cellular Stress, Innate Immune Response, Matrix Remodeling, Microglia Function | NS\_MM\_NEUROINFLAM\_V1.0 | Mmp14 | mRNA | 0 |  | Microglia Function;Matrix Remodeling;Innate Immune Response;Cellular Stress | mmu04668;mmu04912 |  | Mmp14 | slateblue4 | 16 | -3.42047930283224 |
  | Casp3-mRNA | -0.413 | 0.154 | -0.716 | -0.111 | 0.751 | 0.609 | 0.926 | 0.0165 | 0.417 | lm.nb | Apoptosis, Cellular Stress, DNA Damage, Growth Factor Signaling, Innate Immune Response, Matrix Remodeling | NS\_MM\_NEUROINFLAM\_V1.0 | Casp3 | mRNA | 0 |  | Matrix Remodeling;Innate Immune Response;Growth Factor Signaling;DNA Damage;Cellular Stress;Apoptosis | mmu04010;mmu04115;mmu04210;mmu04650;mmu04668;mmu04726;mmu04932;mmu04933;mmu05010;mmu05012;mmu05014;mmu05016;mmu05133;mmu05134;mmu05145;mmu05146;mmu05152;mmu05161;mmu05168;mmu05200;mmu05203;mmu05205;mmu05206;mmu05210;mmu05416 |  | Casp3 | slateblue4 | 16 | -2.68181818181818 |
  | Itga7-mRNA | -0.948 | 0.371 | -1.67 | -0.221 | 0.519 | 0.313 | 0.858 | 0.0212 | 0.485 | lm.nb | Astrocyte Function, Growth Factor Signaling, Matrix Remodeling | NS\_MM\_NEUROINFLAM\_V1.0 | Itga7 | mRNA | 0 |  | Matrix Remodeling;Growth Factor Signaling;Astrocyte Function | mmu04151;mmu04510;mmu04512;mmu04810;mmu05410;mmu05412;mmu05414 | Astrocytes | Itga7 | slateblue4 | 16 | -2.55525606469003 |
  | Pecam1-mRNA | 1.11 | 0.478 | 0.175 | 2.05 | 2.16 | 1.13 | 4.14 | 0.0335 | 0.649 | lm.nb | Matrix Remodeling | NS\_MM\_NEUROINFLAM\_V1.0 | Pecam1 | mRNA | 0 |  | Matrix Remodeling | mmu04514;mmu04670;mmu05144 |  | Pecam1 | azure4 | 1 | 2.32217573221757 |
  | Itga6-mRNA | 0.434 | 0.19 | 0.0611 | 0.806 | 1.35 | 1.04 | 1.75 | 0.0365 | 0.679 | loglinear | Growth Factor Signaling, Matrix Remodeling, Microglia Function | NS\_MM\_NEUROINFLAM\_V1.0 | Itga6 | mRNA | 0 |  | Microglia Function;Matrix Remodeling;Growth Factor Signaling | mmu04151;mmu04510;mmu04512;mmu04514;mmu04640;mmu04810;mmu05145;mmu05200;mmu05222;mmu05410;mmu05412;mmu05414 |  | Itga6 | azure4 | 1 | 2.28421052631579 |
  | Dst-mRNA | 0.382 | 0.172 | 0.0456 | 0.718 | 1.3 | 1.03 | 1.64 | 0.0408 | 0.73 | lm.nb | Matrix Remodeling, Microglia Function | NS\_MM\_NEUROINFLAM\_V1.0 | Dst | mRNA | 0 |  | Microglia Function;Matrix Remodeling |  |  | Dst | azure4 | 1 | 2.22093023255814 |
  | Tgfb1-mRNA | 0.852 | 0.388 | 0.0926 | 1.61 | 1.81 | 1.07 | 3.06 | 0.0429 | 0.758 | lm.nb | Cell Cycle, Cytokine Signaling, Growth Factor Signaling, Matrix Remodeling, Microglia Function | NS\_MM\_NEUROINFLAM\_V1.0 | Tgfb1 | mRNA | 0 |  | Microglia Function;Matrix Remodeling;Growth Factor Signaling;Cytokine Signaling;Cell Cycle | mmu04010;mmu04060;mmu04068;mmu04110;mmu04144;mmu04350;mmu04380;mmu04390;mmu04672;mmu04932;mmu04933;mmu05140;mmu05142;mmu05144;mmu05145;mmu05146;mmu05152;mmu05161;mmu05166;mmu05200;mmu05205;mmu05210;mmu05211;mmu05212;mmu05220;mmu05321;mmu05323;mmu05410;mmu05414 |  | Tgfb1 | azure4 | 1 | 2.19587628865979 |
  | Fbln5-mRNA | 0.828 | 0.384 | 0.0745 | 1.58 | 1.78 | 1.05 | 2.99 | 0.0468 | 0.799 | lm.nb | Astrocyte Function, Matrix Remodeling | NS\_MM\_NEUROINFLAM\_V1.0 | Fbln5 | mRNA | 0 |  | Matrix Remodeling;Astrocyte Function |  |  | Fbln5 | azure4 | 1 | 2.15625 |
  | Cd47-mRNA | -0.579 | 0.277 | -1.12 | -0.0359 | 0.67 | 0.46 | 0.975 | 0.053 | 0.865 | lm.nb | Autophagy, Matrix Remodeling | NS\_MM\_NEUROINFLAM\_V1.0 | Cd47 | mRNA | 0 |  | Matrix Remodeling;Autophagy | mmu04512 |  | Cd47 | azure4 | 1 | -2.09025270758123 |
  | Cntnap2-mRNA | -0.271 | 0.155 | -0.573 | 0.0323 | 0.829 | 0.672 | 1.02 | 0.0991 | 1 | lm.nb | Matrix Remodeling, Neurons and Neurotransmission | NS\_MM\_NEUROINFLAM\_V1.0 | Cntnap2 | mRNA | 0 |  | Neurons and Neurotransmission;Matrix Remodeling | mmu04514 |  | Cntnap2 | azure4 | 1 | -1.74838709677419 |
  | Nlgn1-mRNA | -0.066 | 0.0536 | -0.171 | 0.039 | 0.955 | 0.888 | 1.03 | 0.236 | 1 | loglinear | Matrix Remodeling, Neurons and Neurotransmission | NS\_MM\_NEUROINFLAM\_V1.0 | Nlgn1 | mRNA | 0 |  | Neurons and Neurotransmission;Matrix Remodeling | mmu04514 |  | Nlgn1 | azure4 | 1 | -1.23134328358209 |
  | Jam2-mRNA | 0.174 | 0.142 | -0.104 | 0.453 | 1.13 | 0.93 | 1.37 | 0.238 | 1 | lm.nb | Matrix Remodeling, Microglia Function | NS\_MM\_NEUROINFLAM\_V1.0 | Jam2 | mRNA | 0 |  | Microglia Function;Matrix Remodeling | mmu04514;mmu04530;mmu04670 |  | Jam2 | azure4 | 1 | 1.22535211267606 |
  | Ttr-mRNA | 0.325 | 0.276 | -0.217 | 0.866 | 1.25 | 0.861 | 1.82 | 0.257 | 1 | lm.nb | Matrix Remodeling | NS\_MM\_NEUROINFLAM\_V1.0 | Ttr | mRNA | 0 |  | Matrix Remodeling |  |  | Ttr | azure4 | 1 | 1.17753623188406 |
  | Itgb5-mRNA | 0.132 | 0.12 | -0.103 | 0.367 | 1.1 | 0.931 | 1.29 | 0.287 | 1 | lm.nb | Adaptive Immune Response, Growth Factor Signaling, Matrix Remodeling | NS\_MM\_NEUROINFLAM\_V1.0 | Itgb5 | mRNA | 0 |  | Matrix Remodeling;Growth Factor Signaling;Adaptive Immune Response | mmu04145;mmu04151;mmu04510;mmu04512;mmu04810;mmu05205;mmu05410;mmu05412;mmu05414 |  | Itgb5 | azure4 | 1 | 1.1 |
  | Itgav-mRNA | 0.143 | 0.135 | -0.122 | 0.408 | 1.1 | 0.919 | 1.33 | 0.306 | 1 | loglinear | Adaptive Immune Response, Angiogenesis, Autophagy, Growth Factor Signaling, Matrix Remodeling | NS\_MM\_NEUROINFLAM\_V1.0 | Itgav | mRNA | 0 |  | Matrix Remodeling;Growth Factor Signaling;Autophagy;Angiogenesis;Adaptive Immune Response | mmu04145;mmu04151;mmu04510;mmu04512;mmu04514;mmu04810;mmu04919;mmu05200;mmu05205;mmu05222;mmu05410;mmu05412;mmu05414 |  | Itgav | azure4 | 1 | 1.05925925925926 |
  | Mag-mRNA | 0.105 | 0.108 | -0.106 | 0.317 | 1.08 | 0.929 | 1.25 | 0.344 | 1 | loglinear | Growth Factor Signaling, Matrix Remodeling, Oligodendrocyte Function | NS\_MM\_NEUROINFLAM\_V1.0 | Mag | mRNA | 0 |  | Oligodendrocyte Function;Matrix Remodeling;Growth Factor Signaling | mmu04514 |  | Mag | azure4 | 1 | 0.972222222222222 |
  | Cldn5-mRNA | -0.233 | 0.245 | -0.713 | 0.248 | 0.851 | 0.61 | 1.19 | 0.356 | 1 | lm.nb | Matrix Remodeling | NS\_MM\_NEUROINFLAM\_V1.0 | Cldn5 | mRNA | 0 |  | Matrix Remodeling | mmu04514;mmu04530;mmu04670;mmu05160 | Endothelial Cells | Cldn5 | azure4 | 1 | -0.951020408163265 |
  | Nlgn2-mRNA | -0.0924 | 0.106 | -0.299 | 0.114 | 0.938 | 0.813 | 1.08 | 0.394 | 1 | loglinear | Matrix Remodeling, Neurons and Neurotransmission | NS\_MM\_NEUROINFLAM\_V1.0 | Nlgn2 | mRNA | 0 |  | Neurons and Neurotransmission;Matrix Remodeling | mmu04514 |  | Nlgn2 | azure4 | 1 | -0.871698113207547 |
  | Pdpn-mRNA | 0.149 | 0.175 | -0.195 | 0.493 | 1.11 | 0.874 | 1.41 | 0.408 | 1 | lm.nb | Matrix Remodeling | NS\_MM\_NEUROINFLAM\_V1.0 | Pdpn | mRNA | 0 |  | Matrix Remodeling |  |  | Pdpn | azure4 | 1 | 0.851428571428571 |
  | Olfml3-mRNA | 0.168 | 0.234 | -0.29 | 0.626 | 1.12 | 0.818 | 1.54 | 0.483 | 1 | lm.nb | Matrix Remodeling | NS\_MM\_NEUROINFLAM\_V1.0 | Olfml3 | mRNA | 0 |  | Matrix Remodeling |  |  | Olfml3 | azure4 | 1 | 0.717948717948718 |

  ##### DE Results - Matrix Remodeling genes - DPI: 1 vs.CTRL

  Table displaying each sample's global significance scores and directed global significance scores as defined in the heatmaps above. The global significance score is calculated as the square root of the mean squared t-statistic for the genes in a gene set, with t-statistics coming from the linear regression underlying our differential expression analysis. The directed global significance score is calculated as the square root of the mean signed squared t-statistic for the genes in a gene set, with t-statistics coming from the linear regression underlying our differential expression analysis.

- **Volcano Plot: DPI: 2 vs.CTRL  
  More Plot Information**


  ##### Volcano Plot: DPI: 2 vs.CTRL

  Volcano plot displaying each gene's -log10(p-value) and log2 fold change for the selected covariate. Highly statistically significant genes fall at the top of the plot, and highly differentially expressed genes fall to either side. Genes within the selected gene set are highlighted in orange. Horizontal lines indicate various False Discovery Rate (FDR) thresholds.
- **DE Results - Matrix Remodeling genes - DPI: 2 vs.CTRL  
  More Plot Information  Download CSV Data**

  | Probe Label | Log2 fold change | std error (log2) | Lower confidence limit (log2) | Upper confidence limit (log2) | Linear fold change | Lower confidence limit (linear) | Upper confidence limit (linear) | P-value | BY.p.value | method | Gene.sets | Codeset.Name | Probe.Label | Analyte.Type | Is.Control | Control.Type | Probe.Annotation | KEGG.Pathways | Cell.Type | Official.Gene.Name | volcanocol | volcanopch | tstats.all |
  | --- | --- | --- | --- | --- | --- | --- | --- | --- | --- | --- | --- | --- | --- | --- | --- | --- | --- | --- | --- | --- | --- | --- | --- |
  | Mmp14-mRNA | -1.97 | 0.472 | -2.9 | -1.05 | 0.255 | 0.134 | 0.484 | 0.000713 | 0.022 | lm.nb | Cellular Stress, Innate Immune Response, Matrix Remodeling, Microglia Function | NS\_MM\_NEUROINFLAM\_V1.0 | Mmp14 | mRNA | 0 |  | Microglia Function;Matrix Remodeling;Innate Immune Response;Cellular Stress | mmu04668;mmu04912 |  | Mmp14 | slateblue2 | 16 | -4.17372881355932 |
  | Casp3-mRNA | -0.652 | 0.157 | -0.96 | -0.344 | 0.636 | 0.514 | 0.788 | 0.000753 | 0.0225 | lm.nb | Apoptosis, Cellular Stress, DNA Damage, Growth Factor Signaling, Innate Immune Response, Matrix Remodeling | NS\_MM\_NEUROINFLAM\_V1.0 | Casp3 | mRNA | 0 |  | Matrix Remodeling;Innate Immune Response;Growth Factor Signaling;DNA Damage;Cellular Stress;Apoptosis | mmu04010;mmu04115;mmu04210;mmu04650;mmu04668;mmu04726;mmu04932;mmu04933;mmu05010;mmu05012;mmu05014;mmu05016;mmu05133;mmu05134;mmu05145;mmu05146;mmu05152;mmu05161;mmu05168;mmu05200;mmu05203;mmu05205;mmu05206;mmu05210;mmu05416 |  | Casp3 | slateblue2 | 16 | -4.15286624203822 |
  | Cldn5-mRNA | -0.955 | 0.253 | -1.45 | -0.458 | 0.516 | 0.366 | 0.728 | 0.00169 | 0.0397 | lm.nb | Matrix Remodeling | NS\_MM\_NEUROINFLAM\_V1.0 | Cldn5 | mRNA | 0 |  | Matrix Remodeling | mmu04514;mmu04530;mmu04670;mmu05160 | Endothelial Cells | Cldn5 | slateblue2 | 16 | -3.77470355731225 |
  | Itgb5-mRNA | 0.401 | 0.118 | 0.169 | 0.633 | 1.32 | 1.12 | 1.55 | 0.00372 | 0.0729 | lm.nb | Adaptive Immune Response, Growth Factor Signaling, Matrix Remodeling | NS\_MM\_NEUROINFLAM\_V1.0 | Itgb5 | mRNA | 0 |  | Matrix Remodeling;Growth Factor Signaling;Adaptive Immune Response | mmu04145;mmu04151;mmu04510;mmu04512;mmu04810;mmu05205;mmu05410;mmu05412;mmu05414 |  | Itgb5 | slateblue3 | 16 | 3.39830508474576 |
  | Fbln5-mRNA | 1.14 | 0.377 | 0.399 | 1.88 | 2.2 | 1.32 | 3.67 | 0.00816 | 0.137 | lm.nb | Astrocyte Function, Matrix Remodeling | NS\_MM\_NEUROINFLAM\_V1.0 | Fbln5 | mRNA | 0 |  | Matrix Remodeling;Astrocyte Function |  |  | Fbln5 | slateblue4 | 16 | 3.02387267904509 |
  | Pecam1-mRNA | 1.18 | 0.477 | 0.248 | 2.12 | 2.27 | 1.19 | 4.34 | 0.0246 | 0.319 | lm.nb | Matrix Remodeling | NS\_MM\_NEUROINFLAM\_V1.0 | Pecam1 | mRNA | 0 |  | Matrix Remodeling | mmu04514;mmu04670;mmu05144 |  | Pecam1 | slateblue4 | 16 | 2.47379454926625 |
  | Cd47-mRNA | -0.634 | 0.277 | -1.18 | -0.091 | 0.644 | 0.442 | 0.939 | 0.0361 | 0.451 | lm.nb | Autophagy, Matrix Remodeling | NS\_MM\_NEUROINFLAM\_V1.0 | Cd47 | mRNA | 0 |  | Matrix Remodeling;Autophagy | mmu04512 |  | Cd47 | slateblue4 | 16 | -2.28880866425993 |
  | Nlgn2-mRNA | -0.216 | 0.106 | -0.423 | -0.00901 | 0.861 | 0.746 | 0.994 | 0.0576 | 0.663 | loglinear | Matrix Remodeling, Neurons and Neurotransmission | NS\_MM\_NEUROINFLAM\_V1.0 | Nlgn2 | mRNA | 0 |  | Neurons and Neurotransmission;Matrix Remodeling | mmu04514 |  | Nlgn2 | azure4 | 1 | -2.0377358490566 |
  | Jam2-mRNA | -0.291 | 0.147 | -0.579 | -0.00339 | 0.817 | 0.669 | 0.998 | 0.0648 | 0.724 | lm.nb | Matrix Remodeling, Microglia Function | NS\_MM\_NEUROINFLAM\_V1.0 | Jam2 | mRNA | 0 |  | Microglia Function;Matrix Remodeling | mmu04514;mmu04530;mmu04670 |  | Jam2 | azure4 | 1 | -1.97959183673469 |
  | Cntnap2-mRNA | -0.261 | 0.154 | -0.563 | 0.0422 | 0.835 | 0.677 | 1.03 | 0.111 | 1 | lm.nb | Matrix Remodeling, Neurons and Neurotransmission | NS\_MM\_NEUROINFLAM\_V1.0 | Cntnap2 | mRNA | 0 |  | Neurons and Neurotransmission;Matrix Remodeling | mmu04514 |  | Cntnap2 | azure4 | 1 | -1.69480519480519 |
  | Dst-mRNA | 0.281 | 0.172 | -0.0562 | 0.617 | 1.21 | 0.962 | 1.53 | 0.122 | 1 | lm.nb | Matrix Remodeling, Microglia Function | NS\_MM\_NEUROINFLAM\_V1.0 | Dst | mRNA | 0 |  | Microglia Function;Matrix Remodeling |  |  | Dst | azure4 | 1 | 1.63372093023256 |
  | Pdpn-mRNA | 0.249 | 0.174 | -0.093 | 0.591 | 1.19 | 0.938 | 1.51 | 0.173 | 1 | lm.nb | Matrix Remodeling | NS\_MM\_NEUROINFLAM\_V1.0 | Pdpn | mRNA | 0 |  | Matrix Remodeling |  |  | Pdpn | azure4 | 1 | 1.43103448275862 |
  | Spp1-mRNA | 0.323 | 0.249 | -0.164 | 0.811 | 1.25 | 0.892 | 1.75 | 0.212 | 1 | lm.nb | Growth Factor Signaling, Matrix Remodeling, Microglia Function | NS\_MM\_NEUROINFLAM\_V1.0 | Spp1 | mRNA | 0 |  | Microglia Function;Matrix Remodeling;Growth Factor Signaling | mmu04151;mmu04510;mmu04512;mmu04620 |  | Spp1 | azure4 | 1 | 1.29718875502008 |
  | Mag-mRNA | -0.133 | 0.108 | -0.345 | 0.0794 | 0.912 | 0.788 | 1.06 | 0.238 | 1 | loglinear | Growth Factor Signaling, Matrix Remodeling, Oligodendrocyte Function | NS\_MM\_NEUROINFLAM\_V1.0 | Mag | mRNA | 0 |  | Oligodendrocyte Function;Matrix Remodeling;Growth Factor Signaling | mmu04514 |  | Mag | azure4 | 1 | -1.23148148148148 |
  | Olfml3-mRNA | 0.249 | 0.232 | -0.207 | 0.704 | 1.19 | 0.867 | 1.63 | 0.3 | 1 | lm.nb | Matrix Remodeling | NS\_MM\_NEUROINFLAM\_V1.0 | Olfml3 | mRNA | 0 |  | Matrix Remodeling |  |  | Olfml3 | azure4 | 1 | 1.07327586206897 |
  | Tgfb1-mRNA | 0.354 | 0.404 | -0.439 | 1.15 | 1.28 | 0.738 | 2.21 | 0.395 | 1 | lm.nb | Cell Cycle, Cytokine Signaling, Growth Factor Signaling, Matrix Remodeling, Microglia Function | NS\_MM\_NEUROINFLAM\_V1.0 | Tgfb1 | mRNA | 0 |  | Microglia Function;Matrix Remodeling;Growth Factor Signaling;Cytokine Signaling;Cell Cycle | mmu04010;mmu04060;mmu04068;mmu04110;mmu04144;mmu04350;mmu04380;mmu04390;mmu04672;mmu04932;mmu04933;mmu05140;mmu05142;mmu05144;mmu05145;mmu05146;mmu05152;mmu05161;mmu05166;mmu05200;mmu05205;mmu05210;mmu05211;mmu05212;mmu05220;mmu05321;mmu05323;mmu05410;mmu05414 |  | Tgfb1 | azure4 | 1 | 0.876237623762376 |
  | Ctss-mRNA | 0.135 | 0.206 | -0.269 | 0.538 | 1.1 | 0.83 | 1.45 | 0.523 | 1 | loglinear | Adaptive Immune Response, Innate Immune Response, Matrix Remodeling, Microglia Function | NS\_MM\_NEUROINFLAM\_V1.0 | Ctss | mRNA | 0 |  | Microglia Function;Matrix Remodeling;Innate Immune Response;Adaptive Immune Response | mmu04142;mmu04145;mmu04612;mmu05152 |  | Ctss | azure4 | 1 | 0.655339805825243 |
  | Itga6-mRNA | -0.116 | 0.19 | -0.489 | 0.256 | 0.923 | 0.713 | 1.19 | 0.55 | 1 | loglinear | Growth Factor Signaling, Matrix Remodeling, Microglia Function | NS\_MM\_NEUROINFLAM\_V1.0 | Itga6 | mRNA | 0 |  | Microglia Function;Matrix Remodeling;Growth Factor Signaling | mmu04151;mmu04510;mmu04512;mmu04514;mmu04640;mmu04810;mmu05145;mmu05200;mmu05222;mmu05410;mmu05412;mmu05414 |  | Itga6 | azure4 | 1 | -0.610526315789474 |
  | Itgav-mRNA | 0.0787 | 0.135 | -0.187 | 0.344 | 1.06 | 0.879 | 1.27 | 0.569 | 1 | loglinear | Adaptive Immune Response, Angiogenesis, Autophagy, Growth Factor Signaling, Matrix Remodeling | NS\_MM\_NEUROINFLAM\_V1.0 | Itgav | mRNA | 0 |  | Matrix Remodeling;Growth Factor Signaling;Autophagy;Angiogenesis;Adaptive Immune Response | mmu04145;mmu04151;mmu04510;mmu04512;mmu04514;mmu04810;mmu04919;mmu05200;mmu05205;mmu05222;mmu05410;mmu05412;mmu05414 |  | Itgav | azure4 | 1 | 0.582962962962963 |
  | Esam-mRNA | -0.213 | 0.375 | -0.947 | 0.521 | 0.863 | 0.519 | 1.44 | 0.578 | 1 | lm.nb | Matrix Remodeling | NS\_MM\_NEUROINFLAM\_V1.0 | Esam | mRNA | 0 |  | Matrix Remodeling | mmu04514;mmu04670 | Endothelial Cells | Esam | azure4 | 1 | -0.568 |

  ##### DE Results - Matrix Remodeling genes - DPI: 2 vs.CTRL

  Table displaying each sample's global significance scores and directed global significance scores as defined in the heatmaps above. The global significance score is calculated as the square root of the mean squared t-statistic for the genes in a gene set, with t-statistics coming from the linear regression underlying our differential expression analysis. The directed global significance score is calculated as the square root of the mean signed squared t-statistic for the genes in a gene set, with t-statistics coming from the linear regression underlying our differential expression analysis.

- **Volcano Plot: DPI: 3 vs.CTRL  
  More Plot Information**


  ##### Volcano Plot: DPI: 3 vs.CTRL

  Volcano plot displaying each gene's -log10(p-value) and log2 fold change for the selected covariate. Highly statistically significant genes fall at the top of the plot, and highly differentially expressed genes fall to either side. Genes within the selected gene set are highlighted in orange. Horizontal lines indicate various False Discovery Rate (FDR) thresholds.
- **DE Results - Matrix Remodeling genes - DPI: 3 vs.CTRL  
  More Plot Information  Download CSV Data**

  | Probe Label | Log2 fold change | std error (log2) | Lower confidence limit (log2) | Upper confidence limit (log2) | Linear fold change | Lower confidence limit (linear) | Upper confidence limit (linear) | P-value | BY.p.value | method | Gene.sets | Codeset.Name | Probe.Label | Analyte.Type | Is.Control | Control.Type | Probe.Annotation | KEGG.Pathways | Cell.Type | Official.Gene.Name | volcanocol | volcanopch | tstats.all |
  | --- | --- | --- | --- | --- | --- | --- | --- | --- | --- | --- | --- | --- | --- | --- | --- | --- | --- | --- | --- | --- | --- | --- | --- |
  | Fbln5-mRNA | 2.32 | 0.359 | 1.62 | 3.03 | 5 | 3.07 | 8.14 | 7.74e-06 | 0.000535 | lm.nb | Astrocyte Function, Matrix Remodeling | NS\_MM\_NEUROINFLAM\_V1.0 | Fbln5 | mRNA | 0 |  | Matrix Remodeling;Astrocyte Function |  |  | Fbln5 | slateblue1 | 16 | 6.46239554317549 |
  | Pdpn-mRNA | 1.04 | 0.169 | 0.709 | 1.37 | 2.06 | 1.63 | 2.59 | 1.37e-05 | 0.000877 | lm.nb | Matrix Remodeling | NS\_MM\_NEUROINFLAM\_V1.0 | Pdpn | mRNA | 0 |  | Matrix Remodeling |  |  | Pdpn | slateblue1 | 16 | 6.15384615384615 |
  | Mag-mRNA | -0.474 | 0.108 | -0.686 | -0.262 | 0.72 | 0.621 | 0.834 | 0.000459 | 0.0149 | loglinear | Growth Factor Signaling, Matrix Remodeling, Oligodendrocyte Function | NS\_MM\_NEUROINFLAM\_V1.0 | Mag | mRNA | 0 |  | Oligodendrocyte Function;Matrix Remodeling;Growth Factor Signaling | mmu04514 |  | Mag | slateblue2 | 16 | -4.38888888888889 |
  | Ctss-mRNA | 0.833 | 0.206 | 0.429 | 1.24 | 1.78 | 1.35 | 2.36 | 0.000938 | 0.0267 | loglinear | Adaptive Immune Response, Innate Immune Response, Matrix Remodeling, Microglia Function | NS\_MM\_NEUROINFLAM\_V1.0 | Ctss | mRNA | 0 |  | Microglia Function;Matrix Remodeling;Innate Immune Response;Adaptive Immune Response | mmu04142;mmu04145;mmu04612;mmu05152 |  | Ctss | slateblue2 | 16 | 4.04368932038835 |
  | Esam-mRNA | 1.06 | 0.336 | 0.398 | 1.71 | 2.08 | 1.32 | 3.28 | 0.00627 | 0.125 | lm.nb | Matrix Remodeling | NS\_MM\_NEUROINFLAM\_V1.0 | Esam | mRNA | 0 |  | Matrix Remodeling | mmu04514;mmu04670 | Endothelial Cells | Esam | slateblue4 | 16 | 3.1547619047619 |
  | Itga7-mRNA | 0.889 | 0.29 | 0.321 | 1.46 | 1.85 | 1.25 | 2.75 | 0.00736 | 0.144 | lm.nb | Astrocyte Function, Growth Factor Signaling, Matrix Remodeling | NS\_MM\_NEUROINFLAM\_V1.0 | Itga7 | mRNA | 0 |  | Matrix Remodeling;Growth Factor Signaling;Astrocyte Function | mmu04151;mmu04510;mmu04512;mmu04810;mmu05410;mmu05412;mmu05414 | Astrocytes | Itga7 | slateblue4 | 16 | 3.06551724137931 |
  | Casp3-mRNA | -0.463 | 0.155 | -0.766 | -0.159 | 0.726 | 0.588 | 0.896 | 0.00872 | 0.162 | lm.nb | Apoptosis, Cellular Stress, DNA Damage, Growth Factor Signaling, Innate Immune Response, Matrix Remodeling | NS\_MM\_NEUROINFLAM\_V1.0 | Casp3 | mRNA | 0 |  | Matrix Remodeling;Innate Immune Response;Growth Factor Signaling;DNA Damage;Cellular Stress;Apoptosis | mmu04010;mmu04115;mmu04210;mmu04650;mmu04668;mmu04726;mmu04932;mmu04933;mmu05010;mmu05012;mmu05014;mmu05016;mmu05133;mmu05134;mmu05145;mmu05146;mmu05152;mmu05161;mmu05168;mmu05200;mmu05203;mmu05205;mmu05206;mmu05210;mmu05416 |  | Casp3 | slateblue4 | 16 | -2.98709677419355 |
  | Jam2-mRNA | -0.428 | 0.149 | -0.719 | -0.137 | 0.743 | 0.607 | 0.909 | 0.0108 | 0.196 | lm.nb | Matrix Remodeling, Microglia Function | NS\_MM\_NEUROINFLAM\_V1.0 | Jam2 | mRNA | 0 |  | Microglia Function;Matrix Remodeling | mmu04514;mmu04530;mmu04670 |  | Jam2 | slateblue4 | 16 | -2.87248322147651 |
  | Pecam1-mRNA | 1.35 | 0.474 | 0.416 | 2.27 | 2.54 | 1.33 | 4.84 | 0.0119 | 0.214 | lm.nb | Matrix Remodeling | NS\_MM\_NEUROINFLAM\_V1.0 | Pecam1 | mRNA | 0 |  | Matrix Remodeling | mmu04514;mmu04670;mmu05144 |  | Pecam1 | slateblue4 | 16 | 2.84810126582279 |
  | Nlgn1-mRNA | 0.143 | 0.0536 | 0.038 | 0.248 | 1.1 | 1.03 | 1.19 | 0.0168 | 0.28 | loglinear | Matrix Remodeling, Neurons and Neurotransmission | NS\_MM\_NEUROINFLAM\_V1.0 | Nlgn1 | mRNA | 0 |  | Neurons and Neurotransmission;Matrix Remodeling | mmu04514 |  | Nlgn1 | slateblue4 | 16 | 2.66791044776119 |
  | Tgfb1-mRNA | 1.02 | 0.383 | 0.269 | 1.77 | 2.03 | 1.2 | 3.41 | 0.017 | 0.281 | lm.nb | Cell Cycle, Cytokine Signaling, Growth Factor Signaling, Matrix Remodeling, Microglia Function | NS\_MM\_NEUROINFLAM\_V1.0 | Tgfb1 | mRNA | 0 |  | Microglia Function;Matrix Remodeling;Growth Factor Signaling;Cytokine Signaling;Cell Cycle | mmu04010;mmu04060;mmu04068;mmu04110;mmu04144;mmu04350;mmu04380;mmu04390;mmu04672;mmu04932;mmu04933;mmu05140;mmu05142;mmu05144;mmu05145;mmu05146;mmu05152;mmu05161;mmu05166;mmu05200;mmu05205;mmu05210;mmu05211;mmu05212;mmu05220;mmu05321;mmu05323;mmu05410;mmu05414 |  | Tgfb1 | slateblue4 | 16 | 2.66318537859008 |
  | Cldn5-mRNA | -0.612 | 0.249 | -1.1 | -0.124 | 0.654 | 0.466 | 0.918 | 0.0257 | 0.388 | lm.nb | Matrix Remodeling | NS\_MM\_NEUROINFLAM\_V1.0 | Cldn5 | mRNA | 0 |  | Matrix Remodeling | mmu04514;mmu04530;mmu04670;mmu05160 | Endothelial Cells | Cldn5 | slateblue4 | 16 | -2.4578313253012 |
  | Mmp14-mRNA | -1.03 | 0.445 | -1.9 | -0.155 | 0.49 | 0.268 | 0.898 | 0.0346 | 0.488 | lm.nb | Cellular Stress, Innate Immune Response, Matrix Remodeling, Microglia Function | NS\_MM\_NEUROINFLAM\_V1.0 | Mmp14 | mRNA | 0 |  | Microglia Function;Matrix Remodeling;Innate Immune Response;Cellular Stress | mmu04668;mmu04912 |  | Mmp14 | slateblue4 | 16 | -2.31460674157303 |
  | Itgb5-mRNA | 0.261 | 0.119 | 0.0274 | 0.494 | 1.2 | 1.02 | 1.41 | 0.0437 | 0.584 | lm.nb | Adaptive Immune Response, Growth Factor Signaling, Matrix Remodeling | NS\_MM\_NEUROINFLAM\_V1.0 | Itgb5 | mRNA | 0 |  | Matrix Remodeling;Growth Factor Signaling;Adaptive Immune Response | mmu04145;mmu04151;mmu04510;mmu04512;mmu04810;mmu05205;mmu05410;mmu05412;mmu05414 |  | Itgb5 | azure4 | 1 | 2.19327731092437 |
  | Nlgn2-mRNA | 0.182 | 0.106 | -0.0244 | 0.389 | 1.13 | 0.983 | 1.31 | 0.103 | 1 | loglinear | Matrix Remodeling, Neurons and Neurotransmission | NS\_MM\_NEUROINFLAM\_V1.0 | Nlgn2 | mRNA | 0 |  | Neurons and Neurotransmission;Matrix Remodeling | mmu04514 |  | Nlgn2 | azure4 | 1 | 1.71698113207547 |
  | Dst-mRNA | 0.218 | 0.172 | -0.119 | 0.555 | 1.16 | 0.921 | 1.47 | 0.224 | 1 | lm.nb | Matrix Remodeling, Microglia Function | NS\_MM\_NEUROINFLAM\_V1.0 | Dst | mRNA | 0 |  | Microglia Function;Matrix Remodeling |  |  | Dst | azure4 | 1 | 1.26744186046512 |
  | Spp1-mRNA | 0.235 | 0.249 | -0.253 | 0.723 | 1.18 | 0.839 | 1.65 | 0.359 | 1 | lm.nb | Growth Factor Signaling, Matrix Remodeling, Microglia Function | NS\_MM\_NEUROINFLAM\_V1.0 | Spp1 | mRNA | 0 |  | Microglia Function;Matrix Remodeling;Growth Factor Signaling | mmu04151;mmu04510;mmu04512;mmu04620 |  | Spp1 | azure4 | 1 | 0.943775100401606 |
  | Olfml3-mRNA | -0.19 | 0.241 | -0.662 | 0.282 | 0.877 | 0.632 | 1.22 | 0.442 | 1 | lm.nb | Matrix Remodeling | NS\_MM\_NEUROINFLAM\_V1.0 | Olfml3 | mRNA | 0 |  | Matrix Remodeling |  |  | Olfml3 | azure4 | 1 | -0.788381742738589 |
  | Cd47-mRNA | -0.138 | 0.277 | -0.68 | 0.404 | 0.909 | 0.624 | 1.32 | 0.624 | 1 | lm.nb | Autophagy, Matrix Remodeling | NS\_MM\_NEUROINFLAM\_V1.0 | Cd47 | mRNA | 0 |  | Matrix Remodeling;Autophagy | mmu04512 |  | Cd47 | azure4 | 1 | -0.498194945848375 |
  | Itgav-mRNA | 0.0374 | 0.135 | -0.228 | 0.303 | 1.03 | 0.854 | 1.23 | 0.786 | 1 | loglinear | Adaptive Immune Response, Angiogenesis, Autophagy, Growth Factor Signaling, Matrix Remodeling | NS\_MM\_NEUROINFLAM\_V1.0 | Itgav | mRNA | 0 |  | Matrix Remodeling;Growth Factor Signaling;Autophagy;Angiogenesis;Adaptive Immune Response | mmu04145;mmu04151;mmu04510;mmu04512;mmu04514;mmu04810;mmu04919;mmu05200;mmu05205;mmu05222;mmu05410;mmu05412;mmu05414 |  | Itgav | azure4 | 1 | 0.277037037037037 |

  ##### DE Results - Matrix Remodeling genes - DPI: 3 vs.CTRL

  Table displaying each sample's global significance scores and directed global significance scores as defined in the heatmaps above. The global significance score is calculated as the square root of the mean squared t-statistic for the genes in a gene set, with t-statistics coming from the linear regression underlying our differential expression analysis. The directed global significance score is calculated as the square root of the mean signed squared t-statistic for the genes in a gene set, with t-statistics coming from the linear regression underlying our differential expression analysis.

DPI:
- DPI: differential expression in 1 vs. baseline of CTRL
- DPI: differential expression in 2 vs. baseline of CTRL
- DPI: differential expression in 3 vs. baseline of CTRL

- **Volcano Plot: DPI: 1 vs.CTRL  
  More Plot Information**


  ##### Volcano Plot: DPI: 1 vs.CTRL

  Volcano plot displaying each gene's -log10(p-value) and log2 fold change for the selected covariate. Highly statistically significant genes fall at the top of the plot, and highly differentially expressed genes fall to either side. Genes within the selected gene set are highlighted in orange. Horizontal lines indicate various False Discovery Rate (FDR) thresholds.
- **DE Results - Microglia Function genes - DPI: 1 vs.CTRL  
  More Plot Information  Download CSV Data**

  | Probe Label | Log2 fold change | std error (log2) | Lower confidence limit (log2) | Upper confidence limit (log2) | Linear fold change | Lower confidence limit (linear) | Upper confidence limit (linear) | P-value | BY.p.value | method | Gene.sets | Codeset.Name | Probe.Label | Analyte.Type | Is.Control | Control.Type | Probe.Annotation | KEGG.Pathways | Cell.Type | Official.Gene.Name | volcanocol | volcanopch | tstats.all |
  | --- | --- | --- | --- | --- | --- | --- | --- | --- | --- | --- | --- | --- | --- | --- | --- | --- | --- | --- | --- | --- | --- | --- | --- |
  | Rab6b-mRNA | -0.226 | 0.0357 | -0.296 | -0.156 | 0.855 | 0.814 | 0.897 | 9.93e-06 | 0.0093 | loglinear | Microglia Function | NS\_MM\_NEUROINFLAM\_V1.0 | Rab6b | mRNA | 0 |  | Microglia Function |  |  | Rab6b | slateblue1 | 16 | -6.33053221288515 |
  | Fscn1-mRNA | -0.612 | 0.0988 | -0.805 | -0.418 | 0.655 | 0.572 | 0.749 | 1.3e-05 | 0.0093 | loglinear | Microglia Function | NS\_MM\_NEUROINFLAM\_V1.0 | Fscn1 | mRNA | 0 |  | Microglia Function |  |  | Fscn1 | slateblue1 | 16 | -6.19433198380567 |
  | Dusp7-mRNA | -0.378 | 0.0611 | -0.497 | -0.258 | 0.77 | 0.708 | 0.836 | 1.32e-05 | 0.0093 | loglinear | Adaptive Immune Response, Angiogenesis, Cytokine Signaling, Growth Factor Signaling, Innate Immune Response, Insulin Signaling, Microglia Function | NS\_MM\_NEUROINFLAM\_V1.0 | Dusp7 | mRNA | 0 |  | Microglia Function;Insulin Signaling;Innate Immune Response;Growth Factor Signaling;Cytokine Signaling;Angiogenesis;Adaptive Immune Response | mmu04010 |  | Dusp7 | slateblue2 | 16 | -6.18657937806874 |
  | Pak1-mRNA | -0.284 | 0.0554 | -0.393 | -0.176 | 0.821 | 0.762 | 0.885 | 1e-04 | 0.0321 | loglinear | Adaptive Immune Response, Angiogenesis, Growth Factor Signaling, Innate Immune Response, Microglia Function | NS\_MM\_NEUROINFLAM\_V1.0 | Pak1 | mRNA | 0 |  | Microglia Function;Innate Immune Response;Growth Factor Signaling;Angiogenesis;Adaptive Immune Response | mmu04010;mmu04012;mmu04014;mmu04024;mmu04062;mmu04360;mmu04510;mmu04650;mmu04660;mmu04666;mmu04810;mmu05205;mmu05211 |  | Pak1 | slateblue2 | 16 | -5.12635379061372 |
  | Rgl1-mRNA | -0.734 | 0.146 | -1.02 | -0.448 | 0.601 | 0.493 | 0.733 | 0.000121 | 0.0357 | lm.nb | Growth Factor Signaling, Microglia Function | NS\_MM\_NEUROINFLAM\_V1.0 | Rgl1 | mRNA | 0 |  | Microglia Function;Growth Factor Signaling | mmu04014 |  | Rgl1 | slateblue2 | 16 | -5.02739726027397 |
  | Csf1r-mRNA | -0.687 | 0.144 | -0.969 | -0.405 | 0.621 | 0.511 | 0.755 | 0.000208 | 0.0464 | lm.nb | Cytokine Signaling, Growth Factor Signaling, Microglia Function | NS\_MM\_NEUROINFLAM\_V1.0 | Csf1r | mRNA | 0 |  | Microglia Function;Growth Factor Signaling;Cytokine Signaling | mmu04014;mmu04015;mmu04060;mmu04144;mmu04151;mmu04380;mmu04640;mmu05200;mmu05202 |  | Csf1r | slateblue2 | 16 | -4.77083333333333 |
  | Fabp5-mRNA | -0.308 | 0.0681 | -0.441 | -0.174 | 0.808 | 0.736 | 0.886 | 0.000352 | 0.0564 | loglinear | Microglia Function | NS\_MM\_NEUROINFLAM\_V1.0 | Fabp5 | mRNA | 0 |  | Microglia Function |  |  | Fabp5 | slateblue3 | 16 | -4.52276064610866 |
  | Tgm2-mRNA | -2.9 | 0.693 | -4.26 | -1.54 | 0.134 | 0.0523 | 0.344 | 0.000708 | 0.0806 | lm.nb | Autophagy, Microglia Function | NS\_MM\_NEUROINFLAM\_V1.0 | Tgm2 | mRNA | 0 |  | Microglia Function;Autophagy | mmu05016 |  | Tgm2 | slateblue3 | 16 | -4.18470418470419 |
  | Fgf13-mRNA | -0.545 | 0.135 | -0.81 | -0.28 | 0.685 | 0.57 | 0.824 | 0.000975 | 0.0956 | lm.nb | Growth Factor Signaling, Microglia Function | NS\_MM\_NEUROINFLAM\_V1.0 | Fgf13 | mRNA | 0 |  | Microglia Function;Growth Factor Signaling | mmu04010;mmu04014;mmu04015;mmu04151;mmu04810;mmu05200;mmu05218 |  | Fgf13 | slateblue4 | 16 | -4.03703703703704 |
  | Rpl9-mRNA | -0.39 | 0.101 | -0.588 | -0.192 | 0.763 | 0.665 | 0.875 | 0.00137 | 0.115 | lm.nb | Microglia Function | NS\_MM\_NEUROINFLAM\_V1.0 | Rpl9 | mRNA | 0 |  | Microglia Function | mmu03010 |  | Rpl9 | slateblue4 | 16 | -3.86138613861386 |
  | Psmb8-mRNA | 1.36 | 0.365 | 0.649 | 2.08 | 2.57 | 1.57 | 4.23 | 0.00179 | 0.129 | lm.nb | Adaptive Immune Response, Angiogenesis, Apoptosis, Astrocyte Function, Cell Cycle, Cytokine Signaling, Growth Factor Signaling, Inflammatory Signaling, Insulin Signaling, Microglia Function, NF-kB, Wnt | NS\_MM\_NEUROINFLAM\_V1.0 | Psmb8 | mRNA | 0 |  | Wnt;NF-kB;Microglia Function;Insulin Signaling;Inflammatory Signaling;Growth Factor Signaling;Cytokine Signaling;Cell Cycle;Astrocyte Function;Apoptosis;Angiogenesis;Adaptive Immune Response |  |  | Psmb8 | slateblue4 | 16 | 3.72602739726027 |
  | Rps3-mRNA | 0.521 | 0.141 | 0.244 | 0.798 | 1.44 | 1.18 | 1.74 | 0.00201 | 0.136 | lm.nb | Microglia Function | NS\_MM\_NEUROINFLAM\_V1.0 | Rps3 | mRNA | 0 |  | Microglia Function | mmu03010 |  | Rps3 | slateblue4 | 16 | 3.69503546099291 |
  | Crip1-mRNA | -1.05 | 0.287 | -1.62 | -0.493 | 0.481 | 0.326 | 0.71 | 0.00202 | 0.136 | lm.nb | Microglia Function | NS\_MM\_NEUROINFLAM\_V1.0 | Crip1 | mRNA | 0 |  | Microglia Function |  |  | Crip1 | slateblue4 | 16 | -3.65853658536585 |
  | Rpl29-mRNA | -0.362 | 0.1 | -0.559 | -0.165 | 0.778 | 0.679 | 0.892 | 0.00238 | 0.15 | loglinear | Microglia Function | NS\_MM\_NEUROINFLAM\_V1.0 | Rpl29 | mRNA | 0 |  | Microglia Function | mmu03010 |  | Rpl29 | slateblue4 | 16 | -3.62 |
  | Lmna-mRNA | -0.702 | 0.204 | -1.1 | -0.303 | 0.615 | 0.466 | 0.811 | 0.0033 | 0.18 | lm.nb | Apoptosis, Cell Cycle, Microglia Function | NS\_MM\_NEUROINFLAM\_V1.0 | Lmna | mRNA | 0 |  | Microglia Function;Cell Cycle;Apoptosis | mmu05410;mmu05412;mmu05414 |  | Lmna | slateblue4 | 16 | -3.44117647058824 |
  | Slc44a1-mRNA | 0.255 | 0.0742 | 0.11 | 0.4 | 1.19 | 1.08 | 1.32 | 0.0034 | 0.182 | loglinear | Microglia Function | NS\_MM\_NEUROINFLAM\_V1.0 | Slc44a1 | mRNA | 0 |  | Microglia Function | mmu05231 |  | Slc44a1 | slateblue4 | 16 | 3.4366576819407 |
  | Mmp14-mRNA | -1.57 | 0.459 | -2.47 | -0.668 | 0.338 | 0.181 | 0.629 | 0.00354 | 0.184 | lm.nb | Cellular Stress, Innate Immune Response, Matrix Remodeling, Microglia Function | NS\_MM\_NEUROINFLAM\_V1.0 | Mmp14 | mRNA | 0 |  | Microglia Function;Matrix Remodeling;Innate Immune Response;Cellular Stress | mmu04668;mmu04912 |  | Mmp14 | slateblue4 | 16 | -3.42047930283224 |
  | Ldha-mRNA | -0.55 | 0.162 | -0.868 | -0.232 | 0.683 | 0.548 | 0.851 | 0.00372 | 0.184 | lm.nb | Microglia Function | NS\_MM\_NEUROINFLAM\_V1.0 | Ldha | mRNA | 0 |  | Microglia Function | mmu00270;mmu04066;mmu04922;mmu05230 |  | Ldha | slateblue4 | 16 | -3.39506172839506 |
  | Pacsin1-mRNA | -0.275 | 0.0827 | -0.437 | -0.113 | 0.827 | 0.739 | 0.925 | 0.00429 | 0.2 | loglinear | Microglia Function | NS\_MM\_NEUROINFLAM\_V1.0 | Pacsin1 | mRNA | 0 |  | Microglia Function |  |  | Pacsin1 | slateblue4 | 16 | -3.32527206771463 |
  | Ptms-mRNA | -1.28 | 0.39 | -2.05 | -0.516 | 0.412 | 0.242 | 0.699 | 0.00468 | 0.206 | lm.nb | Microglia Function | NS\_MM\_NEUROINFLAM\_V1.0 | Ptms | mRNA | 0 |  | Microglia Function |  |  | Ptms | slateblue4 | 16 | -3.28205128205128 |

  ##### DE Results - Microglia Function genes - DPI: 1 vs.CTRL

  Table displaying each sample's global significance scores and directed global significance scores as defined in the heatmaps above. The global significance score is calculated as the square root of the mean squared t-statistic for the genes in a gene set, with t-statistics coming from the linear regression underlying our differential expression analysis. The directed global significance score is calculated as the square root of the mean signed squared t-statistic for the genes in a gene set, with t-statistics coming from the linear regression underlying our differential expression analysis.

- **Volcano Plot: DPI: 2 vs.CTRL  
  More Plot Information**


  ##### Volcano Plot: DPI: 2 vs.CTRL

  Volcano plot displaying each gene's -log10(p-value) and log2 fold change for the selected covariate. Highly statistically significant genes fall at the top of the plot, and highly differentially expressed genes fall to either side. Genes within the selected gene set are highlighted in orange. Horizontal lines indicate various False Discovery Rate (FDR) thresholds.
- **DE Results - Microglia Function genes - DPI: 2 vs.CTRL  
  More Plot Information  Download CSV Data**

  | Probe Label | Log2 fold change | std error (log2) | Lower confidence limit (log2) | Upper confidence limit (log2) | Linear fold change | Lower confidence limit (linear) | Upper confidence limit (linear) | P-value | BY.p.value | method | Gene.sets | Codeset.Name | Probe.Label | Analyte.Type | Is.Control | Control.Type | Probe.Annotation | KEGG.Pathways | Cell.Type | Official.Gene.Name | volcanocol | volcanopch | tstats.all |
  | --- | --- | --- | --- | --- | --- | --- | --- | --- | --- | --- | --- | --- | --- | --- | --- | --- | --- | --- | --- | --- | --- | --- | --- |
  | Lfng-mRNA | 1.68 | 0.166 | 1.36 | 2.01 | 3.21 | 2.56 | 4.02 | 2.36e-08 | 1.04e-05 | lm.nb | Microglia Function, Notch | NS\_MM\_NEUROINFLAM\_V1.0 | Lfng | mRNA | 0 |  | Notch;Microglia Function | mmu04330 |  | Lfng | slateblue1 | 16 | 10.1204819277108 |
  | Rab6b-mRNA | -0.353 | 0.0357 | -0.423 | -0.283 | 0.783 | 0.746 | 0.822 | 3.25e-08 | 1.27e-05 | loglinear | Microglia Function | NS\_MM\_NEUROINFLAM\_V1.0 | Rab6b | mRNA | 0 |  | Microglia Function |  |  | Rab6b | slateblue1 | 16 | -9.88795518207283 |
  | Fabp5-mRNA | -0.612 | 0.0681 | -0.746 | -0.479 | 0.654 | 0.596 | 0.718 | 1.19e-07 | 3.83e-05 | loglinear | Microglia Function | NS\_MM\_NEUROINFLAM\_V1.0 | Fabp5 | mRNA | 0 |  | Microglia Function |  |  | Fabp5 | slateblue1 | 16 | -8.98678414096916 |
  | Fscn1-mRNA | -0.872 | 0.0988 | -1.07 | -0.678 | 0.546 | 0.478 | 0.625 | 1.53e-07 | 4.14e-05 | loglinear | Microglia Function | NS\_MM\_NEUROINFLAM\_V1.0 | Fscn1 | mRNA | 0 |  | Microglia Function |  |  | Fscn1 | slateblue1 | 16 | -8.82591093117409 |
  | Dusp7-mRNA | -0.496 | 0.0611 | -0.616 | -0.377 | 0.709 | 0.652 | 0.77 | 4.53e-07 | 9.99e-05 | loglinear | Adaptive Immune Response, Angiogenesis, Cytokine Signaling, Growth Factor Signaling, Innate Immune Response, Insulin Signaling, Microglia Function | NS\_MM\_NEUROINFLAM\_V1.0 | Dusp7 | mRNA | 0 |  | Microglia Function;Insulin Signaling;Innate Immune Response;Growth Factor Signaling;Cytokine Signaling;Angiogenesis;Adaptive Immune Response | mmu04010 |  | Dusp7 | slateblue1 | 16 | -8.11783960720131 |
  | Gpr34-mRNA | -5.01 | 0.678 | -6.34 | -3.68 | 0.0311 | 0.0124 | 0.0781 | 1.54e-06 | 0.000239 | lm.nb | Microglia Function | NS\_MM\_NEUROINFLAM\_V1.0 | Gpr34 | mRNA | 0 |  | Microglia Function |  |  | Gpr34 | slateblue1 | 16 | -7.38938053097345 |
  | Mertk-mRNA | 1.61 | 0.224 | 1.17 | 2.05 | 3.06 | 2.25 | 4.14 | 2.13e-06 | 0.000313 | lm.nb | Autophagy, Microglia Function | NS\_MM\_NEUROINFLAM\_V1.0 | Mertk | mRNA | 0 |  | Microglia Function;Autophagy |  |  | Mertk | slateblue1 | 16 | 7.1875 |
  | Csf1r-mRNA | -1 | 0.15 | -1.29 | -0.706 | 0.5 | 0.408 | 0.613 | 5.38e-06 | 0.000678 | lm.nb | Cytokine Signaling, Growth Factor Signaling, Microglia Function | NS\_MM\_NEUROINFLAM\_V1.0 | Csf1r | mRNA | 0 |  | Microglia Function;Growth Factor Signaling;Cytokine Signaling | mmu04014;mmu04015;mmu04060;mmu04144;mmu04151;mmu04380;mmu04640;mmu05200;mmu05202 |  | Csf1r | slateblue1 | 16 | -6.66666666666667 |
  | F3-mRNA | 1.08 | 0.166 | 0.758 | 1.41 | 2.12 | 1.69 | 2.65 | 6.96e-06 | 0.000793 | lm.nb | Microglia Function | NS\_MM\_NEUROINFLAM\_V1.0 | F3 | mRNA | 0 |  | Microglia Function | mmu04610;mmu04933 |  | F3 | slateblue1 | 16 | 6.50602409638554 |
  | Fgf13-mRNA | -0.862 | 0.136 | -1.13 | -0.596 | 0.55 | 0.457 | 0.661 | 9.62e-06 | 0.000997 | lm.nb | Growth Factor Signaling, Microglia Function | NS\_MM\_NEUROINFLAM\_V1.0 | Fgf13 | mRNA | 0 |  | Microglia Function;Growth Factor Signaling | mmu04010;mmu04014;mmu04015;mmu04151;mmu04810;mmu05200;mmu05218 |  | Fgf13 | slateblue1 | 16 | -6.33823529411765 |
  | Psmb8-mRNA | 2.23 | 0.352 | 1.54 | 2.92 | 4.7 | 2.91 | 7.59 | 9.89e-06 | 0.000997 | lm.nb | Adaptive Immune Response, Angiogenesis, Apoptosis, Astrocyte Function, Cell Cycle, Cytokine Signaling, Growth Factor Signaling, Inflammatory Signaling, Insulin Signaling, Microglia Function, NF-kB, Wnt | NS\_MM\_NEUROINFLAM\_V1.0 | Psmb8 | mRNA | 0 |  | Wnt;NF-kB;Microglia Function;Insulin Signaling;Inflammatory Signaling;Growth Factor Signaling;Cytokine Signaling;Cell Cycle;Astrocyte Function;Apoptosis;Angiogenesis;Adaptive Immune Response |  |  | Psmb8 | slateblue1 | 16 | 6.33522727272727 |
  | Bola2-mRNA | -0.259 | 0.0439 | -0.345 | -0.173 | 0.836 | 0.787 | 0.887 | 2.24e-05 | 0.00179 | loglinear | Microglia Function | NS\_MM\_NEUROINFLAM\_V1.0 | Bola2 | mRNA | 0 |  | Microglia Function |  |  | Bola2 | slateblue1 | 16 | -5.8997722095672 |
  | Pacsin1-mRNA | -0.476 | 0.0827 | -0.638 | -0.314 | 0.719 | 0.643 | 0.804 | 2.93e-05 | 0.0022 | loglinear | Microglia Function | NS\_MM\_NEUROINFLAM\_V1.0 | Pacsin1 | mRNA | 0 |  | Microglia Function |  |  | Pacsin1 | slateblue1 | 16 | -5.75574365175332 |
  | Crip1-mRNA | -1.67 | 0.294 | -2.24 | -1.09 | 0.315 | 0.211 | 0.47 | 3.51e-05 | 0.00246 | lm.nb | Microglia Function | NS\_MM\_NEUROINFLAM\_V1.0 | Crip1 | mRNA | 0 |  | Microglia Function |  |  | Crip1 | slateblue1 | 16 | -5.68027210884354 |
  | Cox5b-mRNA | -0.185 | 0.0346 | -0.252 | -0.117 | 0.88 | 0.84 | 0.922 | 6.7e-05 | 0.00407 | loglinear | Microglia Function | NS\_MM\_NEUROINFLAM\_V1.0 | Cox5b | mRNA | 0 |  | Microglia Function | mmu04932;mmu05010;mmu05012;mmu05016 |  | Cox5b | slateblue1 | 16 | -5.34682080924856 |
  | Rps21-mRNA | -0.402 | 0.0768 | -0.552 | -0.251 | 0.757 | 0.682 | 0.84 | 8.21e-05 | 0.0046 | loglinear | Microglia Function | NS\_MM\_NEUROINFLAM\_V1.0 | Rps21 | mRNA | 0 |  | Microglia Function | mmu03010 |  | Rps21 | slateblue1 | 16 | -5.234375 |
  | Tmem64-mRNA | -0.282 | 0.0558 | -0.392 | -0.173 | 0.822 | 0.762 | 0.887 | 0.000115 | 0.00609 | loglinear | Microglia Function | NS\_MM\_NEUROINFLAM\_V1.0 | Tmem64 | mRNA | 0 |  | Microglia Function |  |  | Tmem64 | slateblue1 | 16 | -5.05376344086021 |
  | Apoe-mRNA | 0.418 | 0.0826 | 0.256 | 0.58 | 1.34 | 1.19 | 1.49 | 0.000116 | 0.00609 | loglinear | Astrocyte Function, Cellular Stress, Lipid Metabolism, Microglia Function, Neurons and Neurotransmission | NS\_MM\_NEUROINFLAM\_V1.0 | Apoe | mRNA | 0 |  | Neurons and Neurotransmission;Microglia Function;Lipid Metabolism;Cellular Stress;Astrocyte Function | mmu05010 |  | Apoe | slateblue1 | 16 | 5.06053268765133 |
  | Stmn1-mRNA | -0.298 | 0.0593 | -0.414 | -0.181 | 0.814 | 0.751 | 0.882 | 0.000125 | 0.00632 | loglinear | Growth Factor Signaling, Microglia Function | NS\_MM\_NEUROINFLAM\_V1.0 | Stmn1 | mRNA | 0 |  | Microglia Function;Growth Factor Signaling | mmu04010;mmu05206 |  | Stmn1 | slateblue1 | 16 | -5.02529510961214 |
  | Lmna-mRNA | -0.993 | 0.205 | -1.39 | -0.592 | 0.502 | 0.38 | 0.664 | 0.000178 | 0.00829 | lm.nb | Apoptosis, Cell Cycle, Microglia Function | NS\_MM\_NEUROINFLAM\_V1.0 | Lmna | mRNA | 0 |  | Microglia Function;Cell Cycle;Apoptosis | mmu05410;mmu05412;mmu05414 |  | Lmna | slateblue1 | 16 | -4.84390243902439 |

  ##### DE Results - Microglia Function genes - DPI: 2 vs.CTRL

  Table displaying each sample's global significance scores and directed global significance scores as defined in the heatmaps above. The global significance score is calculated as the square root of the mean squared t-statistic for the genes in a gene set, with t-statistics coming from the linear regression underlying our differential expression analysis. The directed global significance score is calculated as the square root of the mean signed squared t-statistic for the genes in a gene set, with t-statistics coming from the linear regression underlying our differential expression analysis.

- **Volcano Plot: DPI: 3 vs.CTRL  
  More Plot Information**


  ##### Volcano Plot: DPI: 3 vs.CTRL

  Volcano plot displaying each gene's -log10(p-value) and log2 fold change for the selected covariate. Highly statistically significant genes fall at the top of the plot, and highly differentially expressed genes fall to either side. Genes within the selected gene set are highlighted in orange. Horizontal lines indicate various False Discovery Rate (FDR) thresholds.
- **DE Results - Microglia Function genes - DPI: 3 vs.CTRL  
  More Plot Information  Download CSV Data**

  | Probe Label | Log2 fold change | std error (log2) | Lower confidence limit (log2) | Upper confidence limit (log2) | Linear fold change | Lower confidence limit (linear) | Upper confidence limit (linear) | P-value | BY.p.value | method | Gene.sets | Codeset.Name | Probe.Label | Analyte.Type | Is.Control | Control.Type | Probe.Annotation | KEGG.Pathways | Cell.Type | Official.Gene.Name | volcanocol | volcanopch | tstats.all |
  | --- | --- | --- | --- | --- | --- | --- | --- | --- | --- | --- | --- | --- | --- | --- | --- | --- | --- | --- | --- | --- | --- | --- | --- |
  | Rab6b-mRNA | -0.441 | 0.0357 | -0.512 | -0.371 | 0.736 | 0.701 | 0.773 | 1.35e-09 | 7.96e-07 | loglinear | Microglia Function | NS\_MM\_NEUROINFLAM\_V1.0 | Rab6b | mRNA | 0 |  | Microglia Function |  |  | Rab6b | slateblue1 | 16 | -12.3529411764706 |
  | Tmem64-mRNA | -0.568 | 0.0558 | -0.677 | -0.459 | 0.675 | 0.625 | 0.728 | 2.13e-08 | 5.36e-06 | loglinear | Microglia Function | NS\_MM\_NEUROINFLAM\_V1.0 | Tmem64 | mRNA | 0 |  | Microglia Function |  |  | Tmem64 | slateblue1 | 16 | -10.179211469534 |
  | Stmn1-mRNA | -0.555 | 0.0593 | -0.671 | -0.438 | 0.681 | 0.628 | 0.738 | 6.89e-08 | 1.41e-05 | loglinear | Growth Factor Signaling, Microglia Function | NS\_MM\_NEUROINFLAM\_V1.0 | Stmn1 | mRNA | 0 |  | Microglia Function;Growth Factor Signaling | mmu04010;mmu05206 |  | Stmn1 | slateblue1 | 16 | -9.35919055649241 |
  | Psmb8-mRNA | 2.99 | 0.346 | 2.31 | 3.67 | 7.95 | 4.97 | 12.7 | 1.99e-07 | 3.2e-05 | lm.nb | Adaptive Immune Response, Angiogenesis, Apoptosis, Astrocyte Function, Cell Cycle, Cytokine Signaling, Growth Factor Signaling, Inflammatory Signaling, Insulin Signaling, Microglia Function, NF-kB, Wnt | NS\_MM\_NEUROINFLAM\_V1.0 | Psmb8 | mRNA | 0 |  | Wnt;NF-kB;Microglia Function;Insulin Signaling;Inflammatory Signaling;Growth Factor Signaling;Cytokine Signaling;Cell Cycle;Astrocyte Function;Apoptosis;Angiogenesis;Adaptive Immune Response |  |  | Psmb8 | slateblue1 | 16 | 8.64161849710983 |
  | Ptgs2-mRNA | 1.88 | 0.236 | 1.41 | 2.34 | 3.67 | 2.67 | 5.06 | 5.87e-07 | 7.14e-05 | lm.nb | Astrocyte Function, Cellular Stress, Innate Immune Response, Lipid Metabolism, Microglia Function, NF-kB | NS\_MM\_NEUROINFLAM\_V1.0 | Ptgs2 | mRNA | 0 |  | NF-kB;Microglia Function;Lipid Metabolism;Innate Immune Response;Cellular Stress;Astrocyte Function | mmu00590;mmu04064;mmu04370;mmu04668;mmu04723;mmu04726;mmu04913;mmu04921;mmu04923;mmu05140;mmu05200;mmu05206;mmu05222 |  | Ptgs2 | slateblue1 | 16 | 7.96610169491525 |
  | Lfng-mRNA | 1.31 | 0.168 | 0.975 | 1.64 | 2.47 | 1.97 | 3.11 | 8.33e-07 | 9.43e-05 | lm.nb | Microglia Function, Notch | NS\_MM\_NEUROINFLAM\_V1.0 | Lfng | mRNA | 0 |  | Notch;Microglia Function | mmu04330 |  | Lfng | slateblue1 | 16 | 7.79761904761905 |
  | Gpr34-mRNA | -5.01 | 0.678 | -6.34 | -3.68 | 0.0311 | 0.0124 | 0.0781 | 1.54e-06 | 0.000151 | lm.nb | Microglia Function | NS\_MM\_NEUROINFLAM\_V1.0 | Gpr34 | mRNA | 0 |  | Microglia Function |  |  | Gpr34 | slateblue1 | 16 | -7.38938053097345 |
  | Ets2-mRNA | 0.65 | 0.0908 | 0.472 | 0.828 | 1.57 | 1.39 | 1.78 | 2.28e-06 | 0.000206 | loglinear | Cellular Stress, Growth Factor Signaling, Microglia Function | NS\_MM\_NEUROINFLAM\_V1.0 | Ets2 | mRNA | 0 |  | Microglia Function;Growth Factor Signaling;Cellular Stress | mmu04014;mmu05166 |  | Ets2 | slateblue1 | 16 | 7.15859030837004 |
  | Tle3-mRNA | 0.865 | 0.124 | 0.622 | 1.11 | 1.82 | 1.54 | 2.16 | 3.15e-06 | 0.000271 | loglinear | Microglia Function, Notch, Wnt | NS\_MM\_NEUROINFLAM\_V1.0 | Tle3 | mRNA | 0 |  | Wnt;Notch;Microglia Function |  |  | Tle3 | slateblue1 | 16 | 6.9758064516129 |
  | Cx3cr1-mRNA | -3.17 | 0.467 | -4.08 | -2.25 | 0.111 | 0.059 | 0.21 | 4.35e-06 | 0.000334 | loglinear | Cytokine Signaling, Microglia Function | NS\_MM\_NEUROINFLAM\_V1.0 | Cx3cr1 | mRNA | 0 |  | Microglia Function;Cytokine Signaling | mmu04060;mmu04062 |  | Cx3cr1 | slateblue1 | 16 | -6.78800856531049 |
  | Mertk-mRNA | 1.52 | 0.225 | 1.07 | 1.96 | 2.86 | 2.11 | 3.89 | 4.83e-06 | 0.000363 | lm.nb | Autophagy, Microglia Function | NS\_MM\_NEUROINFLAM\_V1.0 | Mertk | mRNA | 0 |  | Microglia Function;Autophagy |  |  | Mertk | slateblue1 | 16 | 6.75555555555556 |
  | F3-mRNA | 1.03 | 0.166 | 0.702 | 1.35 | 2.04 | 1.63 | 2.55 | 1.3e-05 | 0.000852 | lm.nb | Microglia Function | NS\_MM\_NEUROINFLAM\_V1.0 | F3 | mRNA | 0 |  | Microglia Function | mmu04610;mmu04933 |  | F3 | slateblue1 | 16 | 6.20481927710843 |
  | Fabp5-mRNA | -0.413 | 0.0681 | -0.547 | -0.279 | 0.751 | 0.685 | 0.824 | 1.65e-05 | 0.001 | loglinear | Microglia Function | NS\_MM\_NEUROINFLAM\_V1.0 | Fabp5 | mRNA | 0 |  | Microglia Function |  |  | Fabp5 | slateblue1 | 16 | -6.06461086637298 |
  | Rsad2-mRNA | 2.54 | 0.435 | 1.69 | 3.39 | 5.81 | 3.22 | 10.5 | 2.54e-05 | 0.00141 | lm.nb | Inflammatory Signaling, Microglia Function | NS\_MM\_NEUROINFLAM\_V1.0 | Rsad2 | mRNA | 0 |  | Microglia Function;Inflammatory Signaling | mmu05164 |  | Rsad2 | slateblue1 | 16 | 5.83908045977012 |
  | Cox5b-mRNA | -0.202 | 0.0346 | -0.27 | -0.134 | 0.87 | 0.83 | 0.911 | 2.55e-05 | 0.00141 | loglinear | Microglia Function | NS\_MM\_NEUROINFLAM\_V1.0 | Cox5b | mRNA | 0 |  | Microglia Function | mmu04932;mmu05010;mmu05012;mmu05016 |  | Cox5b | slateblue1 | 16 | -5.83815028901734 |
  | Atp6v0e-mRNA | 0.678 | 0.117 | 0.448 | 0.907 | 1.6 | 1.36 | 1.88 | 2.78e-05 | 0.00149 | loglinear | Growth Factor Signaling, Insulin Signaling, Microglia Function | NS\_MM\_NEUROINFLAM\_V1.0 | Atp6v0e | mRNA | 0 |  | Microglia Function;Insulin Signaling;Growth Factor Signaling | mmu04145;mmu04721;mmu05323 |  | Atp6v0e | slateblue1 | 16 | 5.79487179487179 |
  | Tgfa-mRNA | -0.731 | 0.134 | -0.994 | -0.467 | 0.603 | 0.502 | 0.723 | 5.46e-05 | 0.00254 | loglinear | Astrocyte Function, Microglia Function, Neurons and Neurotransmission | NS\_MM\_NEUROINFLAM\_V1.0 | Tgfa | mRNA | 0 |  | Neurons and Neurotransmission;Microglia Function;Astrocyte Function | mmu04012;mmu05200;mmu05211;mmu05212;mmu05214;mmu05215;mmu05223 |  | Tgfa | slateblue1 | 16 | -5.45522388059701 |
  | P2ry12-mRNA | -2.58 | 0.478 | -3.52 | -1.65 | 0.167 | 0.0871 | 0.319 | 5.8e-05 | 0.00265 | lm.nb | Microglia Function | NS\_MM\_NEUROINFLAM\_V1.0 | P2ry12 | mRNA | 0 |  | Microglia Function | mmu04611 |  | P2ry12 | slateblue1 | 16 | -5.39748953974895 |
  | Pacsin1-mRNA | -0.444 | 0.0827 | -0.606 | -0.282 | 0.735 | 0.657 | 0.822 | 6.24e-05 | 0.00275 | loglinear | Microglia Function | NS\_MM\_NEUROINFLAM\_V1.0 | Pacsin1 | mRNA | 0 |  | Microglia Function |  |  | Pacsin1 | slateblue1 | 16 | -5.36880290205562 |
  | Lamp1-mRNA | 0.3 | 0.0595 | 0.183 | 0.416 | 1.23 | 1.14 | 1.33 | 0.000122 | 0.00498 | loglinear | Autophagy, Microglia Function | NS\_MM\_NEUROINFLAM\_V1.0 | Lamp1 | mRNA | 0 |  | Microglia Function;Autophagy | mmu04142;mmu04145;mmu05152 |  | Lamp1 | slateblue1 | 16 | 5.04201680672269 |

  ##### DE Results - Microglia Function genes - DPI: 3 vs.CTRL

  Table displaying each sample's global significance scores and directed global significance scores as defined in the heatmaps above. The global significance score is calculated as the square root of the mean squared t-statistic for the genes in a gene set, with t-statistics coming from the linear regression underlying our differential expression analysis. The directed global significance score is calculated as the square root of the mean signed squared t-statistic for the genes in a gene set, with t-statistics coming from the linear regression underlying our differential expression analysis.

DPI:
- DPI: differential expression in 1 vs. baseline of CTRL
- DPI: differential expression in 2 vs. baseline of CTRL
- DPI: differential expression in 3 vs. baseline of CTRL

- **Volcano Plot: DPI: 1 vs.CTRL  
  More Plot Information**


  ##### Volcano Plot: DPI: 1 vs.CTRL

  Volcano plot displaying each gene's -log10(p-value) and log2 fold change for the selected covariate. Highly statistically significant genes fall at the top of the plot, and highly differentially expressed genes fall to either side. Genes within the selected gene set are highlighted in orange. Horizontal lines indicate various False Discovery Rate (FDR) thresholds.
- **DE Results - Neurons and Neurotransmission genes - DPI: 1 vs.CTRL  
  More Plot Information  Download CSV Data**

  | Probe Label | Log2 fold change | std error (log2) | Lower confidence limit (log2) | Upper confidence limit (log2) | Linear fold change | Lower confidence limit (linear) | Upper confidence limit (linear) | P-value | BY.p.value | method | Gene.sets | Codeset.Name | Probe.Label | Analyte.Type | Is.Control | Control.Type | Probe.Annotation | KEGG.Pathways | Cell.Type | Official.Gene.Name | volcanocol | volcanopch | tstats.all |
  | --- | --- | --- | --- | --- | --- | --- | --- | --- | --- | --- | --- | --- | --- | --- | --- | --- | --- | --- | --- | --- | --- | --- | --- |
  | Arc-mRNA | -1.46 | 0.148 | -1.75 | -1.17 | 0.363 | 0.297 | 0.444 | 3.28e-08 | 0.000116 | lm.nb | Neurons and Neurotransmission | NS\_MM\_NEUROINFLAM\_V1.0 | Arc | mRNA | 0 |  | Neurons and Neurotransmission | mmu05031 |  | Arc | slateblue1 | 16 | -9.86486486486486 |
  | Egr1-mRNA | -1.05 | 0.129 | -1.3 | -0.798 | 0.483 | 0.405 | 0.575 | 4.51e-07 | 0.000796 | lm.nb | Inflammatory Signaling, Neurons and Neurotransmission | NS\_MM\_NEUROINFLAM\_V1.0 | Egr1 | mRNA | 0 |  | Neurons and Neurotransmission;Inflammatory Signaling | mmu04933;mmu05020;mmu05166 |  | Egr1 | slateblue1 | 16 | -8.13953488372093 |
  | Fos-mRNA | -1.75 | 0.299 | -2.34 | -1.17 | 0.297 | 0.198 | 0.446 | 2.43e-05 | 0.0143 | lm.nb | Adaptive Immune Response, Cellular Stress, Growth Factor Signaling, Innate Immune Response, Neurons and Neurotransmission | NS\_MM\_NEUROINFLAM\_V1.0 | Fos | mRNA | 0 |  | Neurons and Neurotransmission;Innate Immune Response;Growth Factor Signaling;Cellular Stress;Adaptive Immune Response | mmu04010;mmu04024;mmu04380;mmu04620;mmu04660;mmu04662;mmu04668;mmu04713;mmu04725;mmu04728;mmu04915;mmu04917;mmu04921;mmu05031;mmu05132;mmu05133;mmu05140;mmu05142;mmu05161;mmu05166;mmu05168;mmu05200;mmu05210;mmu05231;mmu05323 |  | Fos | slateblue2 | 16 | -5.85284280936455 |
  | Syp-mRNA | 0.465 | 0.0859 | 0.296 | 0.633 | 1.38 | 1.23 | 1.55 | 5.76e-05 | 0.0291 | loglinear | Neurons and Neurotransmission | NS\_MM\_NEUROINFLAM\_V1.0 | Syp | mRNA | 0 |  | Neurons and Neurotransmission |  |  | Syp | slateblue2 | 16 | 5.41327124563446 |
  | Mapt-mRNA | -0.222 | 0.0425 | -0.306 | -0.139 | 0.857 | 0.809 | 0.908 | 8.22e-05 | 0.0321 | loglinear | Apoptosis, Cellular Stress, Growth Factor Signaling, Neurons and Neurotransmission | NS\_MM\_NEUROINFLAM\_V1.0 | Mapt | mRNA | 0 |  | Neurons and Neurotransmission;Growth Factor Signaling;Cellular Stress;Apoptosis | mmu04010;mmu05010 |  | Mapt | slateblue2 | 16 | -5.22352941176471 |
  | Prkacb-mRNA | -0.327 | 0.0629 | -0.451 | -0.204 | 0.797 | 0.732 | 0.868 | 8.65e-05 | 0.0321 | loglinear | Adaptive Immune Response, Angiogenesis, Apoptosis, Autophagy, Growth Factor Signaling, Lipid Metabolism, Neurons and Neurotransmission, Wnt | NS\_MM\_NEUROINFLAM\_V1.0 | Prkacb | mRNA | 0 |  | Wnt;Neurons and Neurotransmission;Lipid Metabolism;Growth Factor Signaling;Autophagy;Apoptosis;Angiogenesis;Adaptive Immune Response | mmu04010;mmu04014;mmu04020;mmu04024;mmu04062;mmu04114;mmu04210;mmu04211;mmu04213;mmu04261;mmu04270;mmu04310;mmu04540;mmu04611;mmu04713;mmu04720;mmu04723;mmu04724;mmu04725;mmu04726;mmu04728;mmu04750;mmu04910;mmu04911;mmu04912;mmu04913;mmu04914;mmu04915;mmu04916;mmu04919;mmu04921;mmu04922;mmu04923;mmu04924;mmu04925;mmu05012;mmu05020;mmu05030;mmu05031;mmu05146;mmu05166;mmu05169;mmu05200;mmu05203;mmu05205;mmu05414 |  | Prkacb | slateblue2 | 16 | -5.19872813990461 |
  | Bdnf-mRNA | -0.736 | 0.143 | -1.02 | -0.456 | 0.6 | 0.494 | 0.729 | 9.57e-05 | 0.0321 | lm.nb | Growth Factor Signaling, Neurons and Neurotransmission | NS\_MM\_NEUROINFLAM\_V1.0 | Bdnf | mRNA | 0 |  | Neurons and Neurotransmission;Growth Factor Signaling | mmu04010;mmu04024;mmu04722;mmu05016;mmu05030;mmu05034 |  | Bdnf | slateblue2 | 16 | -5.14685314685315 |
  | Mdm2-mRNA | -0.317 | 0.0642 | -0.443 | -0.191 | 0.803 | 0.736 | 0.876 | 0.00015 | 0.0406 | loglinear | Adaptive Immune Response, Apoptosis, Cell Cycle, Cellular Stress, DNA Damage, Growth Factor Signaling, Neurons and Neurotransmission | NS\_MM\_NEUROINFLAM\_V1.0 | Mdm2 | mRNA | 0 |  | Neurons and Neurotransmission;Growth Factor Signaling;DNA Damage;Cellular Stress;Cell Cycle;Apoptosis;Adaptive Immune Response | mmu04068;mmu04110;mmu04115;mmu04120;mmu04144;mmu04151;mmu04919;mmu05169;mmu05200;mmu05202;mmu05203;mmu05205;mmu05206;mmu05214;mmu05215;mmu05218;mmu05219;mmu05220 |  | Mdm2 | slateblue2 | 16 | -4.93769470404984 |
  | Tbr1-mRNA | -0.58 | 0.122 | -0.818 | -0.341 | 0.669 | 0.567 | 0.789 | 0.00021 | 0.0464 | loglinear | Neurons and Neurotransmission | NS\_MM\_NEUROINFLAM\_V1.0 | Tbr1 | mRNA | 0 |  | Neurons and Neurotransmission |  | Neuron | Tbr1 | slateblue3 | 16 | -4.75409836065574 |
  | Nrgn-mRNA | -1.04 | 0.242 | -1.51 | -0.563 | 0.487 | 0.351 | 0.677 | 0.000567 | 0.0754 | lm.nb | Neurons and Neurotransmission | NS\_MM\_NEUROINFLAM\_V1.0 | Nrgn | mRNA | 0 |  | Neurons and Neurotransmission |  |  | Nrgn | slateblue3 | 16 | -4.29752066115702 |
  | Nptx1-mRNA | 0.701 | 0.166 | 0.376 | 1.03 | 1.63 | 1.3 | 2.04 | 0.000644 | 0.0784 | lm.nb | Neurons and Neurotransmission | NS\_MM\_NEUROINFLAM\_V1.0 | Nptx1 | mRNA | 0 |  | Neurons and Neurotransmission |  |  | Nptx1 | slateblue3 | 16 | 4.22289156626506 |
  | Braf-mRNA | -0.641 | 0.154 | -0.943 | -0.339 | 0.641 | 0.52 | 0.791 | 0.000739 | 0.0815 | lm.nb | Adaptive Immune Response, Angiogenesis, Apoptosis, Cytokine Signaling, Growth Factor Signaling, Innate Immune Response, Insulin Signaling, Neurons and Neurotransmission | NS\_MM\_NEUROINFLAM\_V1.0 | Braf | mRNA | 0 |  | Neurons and Neurotransmission;Insulin Signaling;Innate Immune Response;Growth Factor Signaling;Cytokine Signaling;Apoptosis;Angiogenesis;Adaptive Immune Response | mmu04010;mmu04012;mmu04015;mmu04024;mmu04062;mmu04068;mmu04150;mmu04270;mmu04510;mmu04650;mmu04720;mmu04722;mmu04726;mmu04730;mmu04810;mmu04910;mmu04914;mmu05034;mmu05160;mmu05200;mmu05205;mmu05210;mmu05211;mmu05212;mmu05213;mmu05214;mmu05215;mmu05216;mmu05218;mmu05219;mmu05220;mmu05221;mmu05223 |  | Braf | slateblue3 | 16 | -4.16233766233766 |
  | Tubb4a-mRNA | -0.38 | 0.0956 | -0.567 | -0.192 | 0.769 | 0.675 | 0.875 | 0.00109 | 0.104 | loglinear | Adaptive Immune Response, Apoptosis, Cell Cycle, Neurons and Neurotransmission | NS\_MM\_NEUROINFLAM\_V1.0 | Tubb4a | mRNA | 0 |  | Neurons and Neurotransmission;Cell Cycle;Apoptosis;Adaptive Immune Response | mmu04145;mmu04540 |  | Tubb4a | slateblue4 | 16 | -3.97489539748954 |
  | Camk4-mRNA | -0.766 | 0.197 | -1.15 | -0.379 | 0.588 | 0.45 | 0.769 | 0.00131 | 0.113 | lm.nb | Growth Factor Signaling, Neurons and Neurotransmission | NS\_MM\_NEUROINFLAM\_V1.0 | Camk4 | mRNA | 0 |  | Neurons and Neurotransmission;Growth Factor Signaling | mmu04020;mmu04024;mmu04211;mmu04380;mmu04720;mmu04722;mmu04725;mmu04921;mmu04925;mmu05031;mmu05034 |  | Camk4 | slateblue4 | 16 | -3.88832487309645 |
  | Rac1-mRNA | -0.928 | 0.256 | -1.43 | -0.426 | 0.525 | 0.371 | 0.744 | 0.00229 | 0.149 | lm.nb | Adaptive Immune Response, Angiogenesis, Autophagy, Growth Factor Signaling, Innate Immune Response, Neurons and Neurotransmission, Wnt | NS\_MM\_NEUROINFLAM\_V1.0 | Rac1 | mRNA | 0 |  | Wnt;Neurons and Neurotransmission;Innate Immune Response;Growth Factor Signaling;Autophagy;Angiogenesis;Adaptive Immune Response | mmu04010;mmu04014;mmu04015;mmu04024;mmu04062;mmu04071;mmu04145;mmu04151;mmu04310;mmu04360;mmu04370;mmu04380;mmu04510;mmu04520;mmu04620;mmu04650;mmu04662;mmu04664;mmu04666;mmu04670;mmu04722;mmu04810;mmu04932;mmu04933;mmu05014;mmu05100;mmu05132;mmu05200;mmu05203;mmu05205;mmu05210;mmu05211;mmu05212;mmu05231;mmu05416 |  | Rac1 | slateblue4 | 16 | -3.625 |
  | Rbfox3-mRNA | -0.388 | 0.11 | -0.604 | -0.172 | 0.764 | 0.658 | 0.887 | 0.0028 | 0.167 | lm.nb | Neurons and Neurotransmission | NS\_MM\_NEUROINFLAM\_V1.0 | Rbfox3 | mRNA | 0 |  | Neurons and Neurotransmission |  |  | Rbfox3 | slateblue4 | 16 | -3.52727272727273 |
  | Tubb3-mRNA | -0.52 | 0.158 | -0.83 | -0.21 | 0.697 | 0.562 | 0.865 | 0.00464 | 0.206 | lm.nb | Adaptive Immune Response, Apoptosis, Cell Cycle, Neurons and Neurotransmission | NS\_MM\_NEUROINFLAM\_V1.0 | Tubb3 | mRNA | 0 |  | Neurons and Neurotransmission;Cell Cycle;Apoptosis;Adaptive Immune Response | mmu04145;mmu04540 |  | Tubb3 | slateblue4 | 16 | -3.29113924050633 |
  | Dlg4-mRNA | -0.175 | 0.056 | -0.284 | -0.0649 | 0.886 | 0.821 | 0.956 | 0.00662 | 0.246 | loglinear | Adaptive Immune Response, Angiogenesis, Cytokine Signaling, Growth Factor Signaling, Insulin Signaling, Neurons and Neurotransmission | NS\_MM\_NEUROINFLAM\_V1.0 | Dlg4 | mRNA | 0 |  | Neurons and Neurotransmission;Insulin Signaling;Growth Factor Signaling;Cytokine Signaling;Angiogenesis;Adaptive Immune Response | mmu04390;mmu04724;mmu05016;mmu05030 |  | Dlg4 | slateblue4 | 16 | -3.125 |
  | Nefl-mRNA | -0.382 | 0.123 | -0.624 | -0.141 | 0.767 | 0.649 | 0.907 | 0.00687 | 0.248 | lm.nb | Adaptive Immune Response, Angiogenesis, Cytokine Signaling, Growth Factor Signaling, Insulin Signaling, Neurons and Neurotransmission | NS\_MM\_NEUROINFLAM\_V1.0 | Nefl | mRNA | 0 |  | Neurons and Neurotransmission;Insulin Signaling;Growth Factor Signaling;Cytokine Signaling;Angiogenesis;Adaptive Immune Response | mmu05014 |  | Nefl | slateblue4 | 16 | -3.10569105691057 |
  | Tfg-mRNA | -0.252 | 0.0822 | -0.413 | -0.0905 | 0.84 | 0.751 | 0.939 | 0.00745 | 0.255 | loglinear | Apoptosis, Neurons and Neurotransmission | NS\_MM\_NEUROINFLAM\_V1.0 | Tfg | mRNA | 0 |  | Neurons and Neurotransmission;Apoptosis | mmu05200;mmu05216 |  | Tfg | slateblue4 | 16 | -3.06569343065693 |

  ##### DE Results - Neurons and Neurotransmission genes - DPI: 1 vs.CTRL

  Table displaying each sample's global significance scores and directed global significance scores as defined in the heatmaps above. The global significance score is calculated as the square root of the mean squared t-statistic for the genes in a gene set, with t-statistics coming from the linear regression underlying our differential expression analysis. The directed global significance score is calculated as the square root of the mean signed squared t-statistic for the genes in a gene set, with t-statistics coming from the linear regression underlying our differential expression analysis.

- **Volcano Plot: DPI: 2 vs.CTRL  
  More Plot Information**


  ##### Volcano Plot: DPI: 2 vs.CTRL

  Volcano plot displaying each gene's -log10(p-value) and log2 fold change for the selected covariate. Highly statistically significant genes fall at the top of the plot, and highly differentially expressed genes fall to either side. Genes within the selected gene set are highlighted in orange. Horizontal lines indicate various False Discovery Rate (FDR) thresholds.
- **DE Results - Neurons and Neurotransmission genes - DPI: 2 vs.CTRL  
  More Plot Information  Download CSV Data**

  | Probe Label | Log2 fold change | std error (log2) | Lower confidence limit (log2) | Upper confidence limit (log2) | Linear fold change | Lower confidence limit (linear) | Upper confidence limit (linear) | P-value | BY.p.value | method | Gene.sets | Codeset.Name | Probe.Label | Analyte.Type | Is.Control | Control.Type | Probe.Annotation | KEGG.Pathways | Cell.Type | Official.Gene.Name | volcanocol | volcanopch | tstats.all |
  | --- | --- | --- | --- | --- | --- | --- | --- | --- | --- | --- | --- | --- | --- | --- | --- | --- | --- | --- | --- | --- | --- | --- | --- |
  | Egr1-mRNA | -1.71 | 0.13 | -1.97 | -1.46 | 0.305 | 0.255 | 0.363 | 5.12e-10 | 6.02e-07 | lm.nb | Inflammatory Signaling, Neurons and Neurotransmission | NS\_MM\_NEUROINFLAM\_V1.0 | Egr1 | mRNA | 0 |  | Neurons and Neurotransmission;Inflammatory Signaling | mmu04933;mmu05020;mmu05166 |  | Egr1 | slateblue1 | 16 | -13.1538461538462 |
  | Arc-mRNA | -1.96 | 0.152 | -2.26 | -1.66 | 0.257 | 0.209 | 0.316 | 6.95e-10 | 6.13e-07 | lm.nb | Neurons and Neurotransmission | NS\_MM\_NEUROINFLAM\_V1.0 | Arc | mRNA | 0 |  | Neurons and Neurotransmission | mmu05031 |  | Arc | slateblue1 | 16 | -12.8947368421053 |
  | Bdnf-mRNA | -1.64 | 0.158 | -1.94 | -1.33 | 0.322 | 0.26 | 0.399 | 1.66e-08 | 8.35e-06 | lm.nb | Growth Factor Signaling, Neurons and Neurotransmission | NS\_MM\_NEUROINFLAM\_V1.0 | Bdnf | mRNA | 0 |  | Neurons and Neurotransmission;Growth Factor Signaling | mmu04010;mmu04024;mmu04722;mmu05016;mmu05030;mmu05034 |  | Bdnf | slateblue1 | 16 | -10.379746835443 |
  | Mapt-mRNA | -0.314 | 0.0425 | -0.397 | -0.23 | 0.805 | 0.759 | 0.852 | 1.56e-06 | 0.000239 | loglinear | Apoptosis, Cellular Stress, Growth Factor Signaling, Neurons and Neurotransmission | NS\_MM\_NEUROINFLAM\_V1.0 | Mapt | mRNA | 0 |  | Neurons and Neurotransmission;Growth Factor Signaling;Cellular Stress;Apoptosis | mmu04010;mmu05010 |  | Mapt | slateblue1 | 16 | -7.38823529411765 |
  | Nptx1-mRNA | 1.17 | 0.166 | 0.84 | 1.49 | 2.24 | 1.79 | 2.81 | 2.87e-06 | 0.000405 | lm.nb | Neurons and Neurotransmission | NS\_MM\_NEUROINFLAM\_V1.0 | Nptx1 | mRNA | 0 |  | Neurons and Neurotransmission |  |  | Nptx1 | slateblue1 | 16 | 7.04819277108434 |
  | Fos-mRNA | -2.04 | 0.3 | -2.62 | -1.45 | 0.244 | 0.162 | 0.367 | 4.41e-06 | 0.000585 | lm.nb | Adaptive Immune Response, Cellular Stress, Growth Factor Signaling, Innate Immune Response, Neurons and Neurotransmission | NS\_MM\_NEUROINFLAM\_V1.0 | Fos | mRNA | 0 |  | Neurons and Neurotransmission;Innate Immune Response;Growth Factor Signaling;Cellular Stress;Adaptive Immune Response | mmu04010;mmu04024;mmu04380;mmu04620;mmu04660;mmu04662;mmu04668;mmu04713;mmu04725;mmu04728;mmu04915;mmu04917;mmu04921;mmu05031;mmu05132;mmu05133;mmu05140;mmu05142;mmu05161;mmu05166;mmu05168;mmu05200;mmu05210;mmu05231;mmu05323 |  | Fos | slateblue1 | 16 | -6.8 |
  | Islr2-mRNA | 0.795 | 0.117 | 0.565 | 1.03 | 1.74 | 1.48 | 2.03 | 4.48e-06 | 0.000585 | lm.nb | Neurons and Neurotransmission | NS\_MM\_NEUROINFLAM\_V1.0 | Islr2 | mRNA | 0 |  | Neurons and Neurotransmission |  | Neuron | Islr2 | slateblue1 | 16 | 6.79487179487179 |
  | Syp-mRNA | 0.466 | 0.0859 | 0.297 | 0.634 | 1.38 | 1.23 | 1.55 | 5.64e-05 | 0.00362 | loglinear | Neurons and Neurotransmission | NS\_MM\_NEUROINFLAM\_V1.0 | Syp | mRNA | 0 |  | Neurons and Neurotransmission |  |  | Syp | slateblue1 | 16 | 5.42491268917346 |
  | Tfg-mRNA | -0.429 | 0.0822 | -0.59 | -0.268 | 0.743 | 0.664 | 0.831 | 8.41e-05 | 0.00464 | loglinear | Apoptosis, Neurons and Neurotransmission | NS\_MM\_NEUROINFLAM\_V1.0 | Tfg | mRNA | 0 |  | Neurons and Neurotransmission;Apoptosis | mmu05200;mmu05216 |  | Tfg | slateblue1 | 16 | -5.21897810218978 |
  | Prkacb-mRNA | -0.322 | 0.0629 | -0.445 | -0.199 | 0.8 | 0.735 | 0.871 | 0.000104 | 0.00562 | loglinear | Adaptive Immune Response, Angiogenesis, Apoptosis, Autophagy, Growth Factor Signaling, Lipid Metabolism, Neurons and Neurotransmission, Wnt | NS\_MM\_NEUROINFLAM\_V1.0 | Prkacb | mRNA | 0 |  | Wnt;Neurons and Neurotransmission;Lipid Metabolism;Growth Factor Signaling;Autophagy;Apoptosis;Angiogenesis;Adaptive Immune Response | mmu04010;mmu04014;mmu04020;mmu04024;mmu04062;mmu04114;mmu04210;mmu04211;mmu04213;mmu04261;mmu04270;mmu04310;mmu04540;mmu04611;mmu04713;mmu04720;mmu04723;mmu04724;mmu04725;mmu04726;mmu04728;mmu04750;mmu04910;mmu04911;mmu04912;mmu04913;mmu04914;mmu04915;mmu04916;mmu04919;mmu04921;mmu04922;mmu04923;mmu04924;mmu04925;mmu05012;mmu05020;mmu05030;mmu05031;mmu05146;mmu05166;mmu05169;mmu05200;mmu05203;mmu05205;mmu05414 |  | Prkacb | slateblue1 | 16 | -5.11923688394277 |
  | Apoe-mRNA | 0.418 | 0.0826 | 0.256 | 0.58 | 1.34 | 1.19 | 1.49 | 0.000116 | 0.00609 | loglinear | Astrocyte Function, Cellular Stress, Lipid Metabolism, Microglia Function, Neurons and Neurotransmission | NS\_MM\_NEUROINFLAM\_V1.0 | Apoe | mRNA | 0 |  | Neurons and Neurotransmission;Microglia Function;Lipid Metabolism;Cellular Stress;Astrocyte Function | mmu05010 |  | Apoe | slateblue1 | 16 | 5.06053268765133 |
  | Tubb3-mRNA | -0.799 | 0.158 | -1.11 | -0.489 | 0.575 | 0.463 | 0.713 | 0.000119 | 0.00609 | lm.nb | Adaptive Immune Response, Apoptosis, Cell Cycle, Neurons and Neurotransmission | NS\_MM\_NEUROINFLAM\_V1.0 | Tubb3 | mRNA | 0 |  | Neurons and Neurotransmission;Cell Cycle;Apoptosis;Adaptive Immune Response | mmu04145;mmu04540 |  | Tubb3 | slateblue1 | 16 | -5.05696202531646 |
  | Homer1-mRNA | -0.458 | 0.0952 | -0.645 | -0.271 | 0.728 | 0.64 | 0.828 | 0.000192 | 0.00868 | lm.nb | Neurons and Neurotransmission | NS\_MM\_NEUROINFLAM\_V1.0 | Homer1 | mRNA | 0 |  | Neurons and Neurotransmission | mmu04068;mmu04724 |  | Homer1 | slateblue1 | 16 | -4.8109243697479 |
  | Nrgn-mRNA | -1.12 | 0.242 | -1.59 | -0.643 | 0.461 | 0.332 | 0.64 | 0.000286 | 0.0115 | lm.nb | Neurons and Neurotransmission | NS\_MM\_NEUROINFLAM\_V1.0 | Nrgn | mRNA | 0 |  | Neurons and Neurotransmission |  |  | Nrgn | slateblue2 | 16 | -4.62809917355372 |
  | Gjb1-mRNA | -0.959 | 0.214 | -1.38 | -0.54 | 0.514 | 0.385 | 0.688 | 0.000377 | 0.014 | lm.nb | Neurons and Neurotransmission, Oligodendrocyte Function | NS\_MM\_NEUROINFLAM\_V1.0 | Gjb1 | mRNA | 0 |  | Oligodendrocyte Function;Neurons and Neurotransmission |  | Oligodendrocytes | Gjb1 | slateblue2 | 16 | -4.48130841121495 |
  | Tbr1-mRNA | -0.541 | 0.122 | -0.779 | -0.302 | 0.687 | 0.583 | 0.811 | 0.000406 | 0.0148 | loglinear | Neurons and Neurotransmission | NS\_MM\_NEUROINFLAM\_V1.0 | Tbr1 | mRNA | 0 |  | Neurons and Neurotransmission |  | Neuron | Tbr1 | slateblue2 | 16 | -4.4344262295082 |
  | Nefl-mRNA | -0.522 | 0.124 | -0.764 | -0.28 | 0.696 | 0.589 | 0.824 | 0.000651 | 0.0215 | lm.nb | Adaptive Immune Response, Angiogenesis, Cytokine Signaling, Growth Factor Signaling, Insulin Signaling, Neurons and Neurotransmission | NS\_MM\_NEUROINFLAM\_V1.0 | Nefl | mRNA | 0 |  | Neurons and Neurotransmission;Insulin Signaling;Growth Factor Signaling;Cytokine Signaling;Angiogenesis;Adaptive Immune Response | mmu05014 |  | Nefl | slateblue2 | 16 | -4.20967741935484 |
  | Camk4-mRNA | -0.824 | 0.197 | -1.21 | -0.437 | 0.565 | 0.432 | 0.738 | 0.000709 | 0.022 | lm.nb | Growth Factor Signaling, Neurons and Neurotransmission | NS\_MM\_NEUROINFLAM\_V1.0 | Camk4 | mRNA | 0 |  | Neurons and Neurotransmission;Growth Factor Signaling | mmu04020;mmu04024;mmu04211;mmu04380;mmu04720;mmu04722;mmu04725;mmu04921;mmu04925;mmu05031;mmu05034 |  | Camk4 | slateblue2 | 16 | -4.18274111675127 |
  | Akt2-mRNA | -0.592 | 0.142 | -0.869 | -0.314 | 0.664 | 0.547 | 0.804 | 0.000713 | 0.022 | loglinear | Adaptive Immune Response, Angiogenesis, Apoptosis, Autophagy, Carbohydrate Metabolism, Cytokine Signaling, Growth Factor Signaling, Innate Immune Response, Insulin Signaling, Neurons and Neurotransmission, Wnt | NS\_MM\_NEUROINFLAM\_V1.0 | Akt2 | mRNA | 0 |  | Wnt;Neurons and Neurotransmission;Insulin Signaling;Innate Immune Response;Growth Factor Signaling;Cytokine Signaling;Carbohydrate Metabolism;Autophagy;Apoptosis;Angiogenesis;Adaptive Immune Response | mmu04010;mmu04012;mmu04014;mmu04015;mmu04022;mmu04024;mmu04062;mmu04066;mmu04068;mmu04071;mmu04072;mmu04150;mmu04151;mmu04152;mmu04210;mmu04211;mmu04213;mmu04261;mmu04370;mmu04380;mmu04510;mmu04530;mmu04550;mmu04611;mmu04620;mmu04630;mmu04660;mmu04662;mmu04664;mmu04666;mmu04668;mmu04722;mmu04725;mmu04728;mmu04910;mmu04914;mmu04915;mmu04917;mmu04919;mmu04920;mmu04922;mmu04923;mmu04931;mmu04932;mmu04933;mmu04973;mmu05142;mmu05145;mmu05152;mmu05160;mmu05161;mmu05162;mmu05164;mmu05166;mmu05169;mmu05200;mmu05205;mmu05210;mmu05211;mmu05212;mmu05213;mmu05214;mmu05215;mmu05218;mmu05220;mmu05221;mmu05222;mmu05223;mmu05230;mmu05231 |  | Akt2 | slateblue2 | 16 | -4.16901408450704 |
  | Braf-mRNA | -0.643 | 0.154 | -0.945 | -0.341 | 0.64 | 0.519 | 0.789 | 0.000718 | 0.022 | lm.nb | Adaptive Immune Response, Angiogenesis, Apoptosis, Cytokine Signaling, Growth Factor Signaling, Innate Immune Response, Insulin Signaling, Neurons and Neurotransmission | NS\_MM\_NEUROINFLAM\_V1.0 | Braf | mRNA | 0 |  | Neurons and Neurotransmission;Insulin Signaling;Innate Immune Response;Growth Factor Signaling;Cytokine Signaling;Apoptosis;Angiogenesis;Adaptive Immune Response | mmu04010;mmu04012;mmu04015;mmu04024;mmu04062;mmu04068;mmu04150;mmu04270;mmu04510;mmu04650;mmu04720;mmu04722;mmu04726;mmu04730;mmu04810;mmu04910;mmu04914;mmu05034;mmu05160;mmu05200;mmu05205;mmu05210;mmu05211;mmu05212;mmu05213;mmu05214;mmu05215;mmu05216;mmu05218;mmu05219;mmu05220;mmu05221;mmu05223 |  | Braf | slateblue2 | 16 | -4.17532467532468 |

  ##### DE Results - Neurons and Neurotransmission genes - DPI: 2 vs.CTRL

  Table displaying each sample's global significance scores and directed global significance scores as defined in the heatmaps above. The global significance score is calculated as the square root of the mean squared t-statistic for the genes in a gene set, with t-statistics coming from the linear regression underlying our differential expression analysis. The directed global significance score is calculated as the square root of the mean signed squared t-statistic for the genes in a gene set, with t-statistics coming from the linear regression underlying our differential expression analysis.

- **Volcano Plot: DPI: 3 vs.CTRL  
  More Plot Information**


  ##### Volcano Plot: DPI: 3 vs.CTRL

  Volcano plot displaying each gene's -log10(p-value) and log2 fold change for the selected covariate. Highly statistically significant genes fall at the top of the plot, and highly differentially expressed genes fall to either side. Genes within the selected gene set are highlighted in orange. Horizontal lines indicate various False Discovery Rate (FDR) thresholds.
- **DE Results - Neurons and Neurotransmission genes - DPI: 3 vs.CTRL  
  More Plot Information  Download CSV Data**

  | Probe Label | Log2 fold change | std error (log2) | Lower confidence limit (log2) | Upper confidence limit (log2) | Linear fold change | Lower confidence limit (linear) | Upper confidence limit (linear) | P-value | BY.p.value | method | Gene.sets | Codeset.Name | Probe.Label | Analyte.Type | Is.Control | Control.Type | Probe.Annotation | KEGG.Pathways | Cell.Type | Official.Gene.Name | volcanocol | volcanopch | tstats.all |
  | --- | --- | --- | --- | --- | --- | --- | --- | --- | --- | --- | --- | --- | --- | --- | --- | --- | --- | --- | --- | --- | --- | --- | --- |
  | Arc-mRNA | -2.98 | 0.164 | -3.3 | -2.66 | 0.127 | 0.101 | 0.158 | 4.17e-12 | 7.36e-09 | lm.nb | Neurons and Neurotransmission | NS\_MM\_NEUROINFLAM\_V1.0 | Arc | mRNA | 0 |  | Neurons and Neurotransmission | mmu05031 |  | Arc | slateblue1 | 16 | -18.1707317073171 |
  | Egr1-mRNA | -1.51 | 0.13 | -1.77 | -1.26 | 0.351 | 0.294 | 0.418 | 3.11e-09 | 1.57e-06 | lm.nb | Inflammatory Signaling, Neurons and Neurotransmission | NS\_MM\_NEUROINFLAM\_V1.0 | Egr1 | mRNA | 0 |  | Neurons and Neurotransmission;Inflammatory Signaling | mmu04933;mmu05020;mmu05166 |  | Egr1 | slateblue1 | 16 | -11.6153846153846 |
  | Mapt-mRNA | -0.413 | 0.0425 | -0.496 | -0.33 | 0.751 | 0.709 | 0.796 | 4.11e-08 | 9.67e-06 | loglinear | Apoptosis, Cellular Stress, Growth Factor Signaling, Neurons and Neurotransmission | NS\_MM\_NEUROINFLAM\_V1.0 | Mapt | mRNA | 0 |  | Neurons and Neurotransmission;Growth Factor Signaling;Cellular Stress;Apoptosis | mmu04010;mmu05010 |  | Mapt | slateblue1 | 16 | -9.71764705882353 |
  | Bdnf-mRNA | -1.3 | 0.151 | -1.59 | -1 | 0.407 | 0.332 | 0.5 | 2.23e-07 | 3.39e-05 | lm.nb | Growth Factor Signaling, Neurons and Neurotransmission | NS\_MM\_NEUROINFLAM\_V1.0 | Bdnf | mRNA | 0 |  | Neurons and Neurotransmission;Growth Factor Signaling | mmu04010;mmu04024;mmu04722;mmu05016;mmu05030;mmu05034 |  | Bdnf | slateblue1 | 16 | -8.60927152317881 |
  | Islr2-mRNA | 0.871 | 0.117 | 0.642 | 1.1 | 1.83 | 1.56 | 2.14 | 1.41e-06 | 0.000146 | lm.nb | Neurons and Neurotransmission | NS\_MM\_NEUROINFLAM\_V1.0 | Islr2 | mRNA | 0 |  | Neurons and Neurotransmission |  | Neuron | Islr2 | slateblue1 | 16 | 7.44444444444444 |
  | Creb1-mRNA | -0.443 | 0.0668 | -0.574 | -0.312 | 0.735 | 0.672 | 0.805 | 5.69e-06 | 0.000418 | loglinear | Adaptive Immune Response, Carbohydrate Metabolism, DNA Damage, Growth Factor Signaling, Innate Immune Response, Neurons and Neurotransmission, Notch | NS\_MM\_NEUROINFLAM\_V1.0 | Creb1 | mRNA | 0 |  | Notch;Neurons and Neurotransmission;Innate Immune Response;Growth Factor Signaling;DNA Damage;Carbohydrate Metabolism;Adaptive Immune Response | mmu04022;mmu04024;mmu04151;mmu04152;mmu04211;mmu04261;mmu04380;mmu04612;mmu04668;mmu04713;mmu04725;mmu04728;mmu04911;mmu04915;mmu04916;mmu04922;mmu04924;mmu04925;mmu04931;mmu05016;mmu05030;mmu05031;mmu05034;mmu05152;mmu05161;mmu05166;mmu05203;mmu05215 |  | Creb1 | slateblue1 | 16 | -6.63173652694611 |
  | Gjb1-mRNA | -1.43 | 0.219 | -1.86 | -1 | 0.371 | 0.276 | 0.5 | 7.05e-06 | 0.000507 | lm.nb | Neurons and Neurotransmission, Oligodendrocyte Function | NS\_MM\_NEUROINFLAM\_V1.0 | Gjb1 | mRNA | 0 |  | Oligodendrocyte Function;Neurons and Neurotransmission |  | Oligodendrocytes | Gjb1 | slateblue1 | 16 | -6.5296803652968 |
  | Homer1-mRNA | -0.595 | 0.0954 | -0.782 | -0.408 | 0.662 | 0.582 | 0.754 | 1.18e-05 | 0.000788 | lm.nb | Neurons and Neurotransmission | NS\_MM\_NEUROINFLAM\_V1.0 | Homer1 | mRNA | 0 |  | Neurons and Neurotransmission | mmu04068;mmu04724 |  | Homer1 | slateblue1 | 16 | -6.23689727463312 |
  | Grm3-mRNA | -0.553 | 0.0923 | -0.734 | -0.372 | 0.682 | 0.601 | 0.773 | 1.89e-05 | 0.00111 | loglinear | Astrocyte Function, Neurons and Neurotransmission | NS\_MM\_NEUROINFLAM\_V1.0 | Grm3 | mRNA | 0 |  | Neurons and Neurotransmission;Astrocyte Function | mmu04072;mmu04080;mmu04724;mmu05030 |  | Grm3 | slateblue1 | 16 | -5.99133261105092 |
  | Syp-mRNA | 0.498 | 0.0859 | 0.329 | 0.666 | 1.41 | 1.26 | 1.59 | 2.74e-05 | 0.00149 | loglinear | Neurons and Neurotransmission | NS\_MM\_NEUROINFLAM\_V1.0 | Syp | mRNA | 0 |  | Neurons and Neurotransmission |  |  | Syp | slateblue1 | 16 | 5.79743888242142 |
  | Nptx1-mRNA | 0.936 | 0.166 | 0.611 | 1.26 | 1.91 | 1.53 | 2.4 | 3.67e-05 | 0.00182 | lm.nb | Neurons and Neurotransmission | NS\_MM\_NEUROINFLAM\_V1.0 | Nptx1 | mRNA | 0 |  | Neurons and Neurotransmission |  |  | Nptx1 | slateblue1 | 16 | 5.63855421686747 |
  | Tgfa-mRNA | -0.731 | 0.134 | -0.994 | -0.467 | 0.603 | 0.502 | 0.723 | 5.46e-05 | 0.00254 | loglinear | Astrocyte Function, Microglia Function, Neurons and Neurotransmission | NS\_MM\_NEUROINFLAM\_V1.0 | Tgfa | mRNA | 0 |  | Neurons and Neurotransmission;Microglia Function;Astrocyte Function | mmu04012;mmu05200;mmu05211;mmu05212;mmu05214;mmu05215;mmu05223 |  | Tgfa | slateblue1 | 16 | -5.45522388059701 |
  | Akt2-mRNA | -0.766 | 0.142 | -1.04 | -0.488 | 0.588 | 0.485 | 0.713 | 5.85e-05 | 0.00265 | loglinear | Adaptive Immune Response, Angiogenesis, Apoptosis, Autophagy, Carbohydrate Metabolism, Cytokine Signaling, Growth Factor Signaling, Innate Immune Response, Insulin Signaling, Neurons and Neurotransmission, Wnt | NS\_MM\_NEUROINFLAM\_V1.0 | Akt2 | mRNA | 0 |  | Wnt;Neurons and Neurotransmission;Insulin Signaling;Innate Immune Response;Growth Factor Signaling;Cytokine Signaling;Carbohydrate Metabolism;Autophagy;Apoptosis;Angiogenesis;Adaptive Immune Response | mmu04010;mmu04012;mmu04014;mmu04015;mmu04022;mmu04024;mmu04062;mmu04066;mmu04068;mmu04071;mmu04072;mmu04150;mmu04151;mmu04152;mmu04210;mmu04211;mmu04213;mmu04261;mmu04370;mmu04380;mmu04510;mmu04530;mmu04550;mmu04611;mmu04620;mmu04630;mmu04660;mmu04662;mmu04664;mmu04666;mmu04668;mmu04722;mmu04725;mmu04728;mmu04910;mmu04914;mmu04915;mmu04917;mmu04919;mmu04920;mmu04922;mmu04923;mmu04931;mmu04932;mmu04933;mmu04973;mmu05142;mmu05145;mmu05152;mmu05160;mmu05161;mmu05162;mmu05164;mmu05166;mmu05169;mmu05200;mmu05205;mmu05210;mmu05211;mmu05212;mmu05213;mmu05214;mmu05215;mmu05218;mmu05220;mmu05221;mmu05222;mmu05223;mmu05230;mmu05231 |  | Akt2 | slateblue1 | 16 | -5.3943661971831 |
  | Syn2-mRNA | -0.218 | 0.0465 | -0.31 | -0.127 | 0.86 | 0.807 | 0.916 | 0.000243 | 0.00894 | loglinear | Neurons and Neurotransmission | NS\_MM\_NEUROINFLAM\_V1.0 | Syn2 | mRNA | 0 |  | Neurons and Neurotransmission |  |  | Syn2 | slateblue1 | 16 | -4.68817204301075 |
  | Tubb3-mRNA | -0.664 | 0.158 | -0.975 | -0.354 | 0.631 | 0.509 | 0.782 | 0.000683 | 0.021 | lm.nb | Adaptive Immune Response, Apoptosis, Cell Cycle, Neurons and Neurotransmission | NS\_MM\_NEUROINFLAM\_V1.0 | Tubb3 | mRNA | 0 |  | Neurons and Neurotransmission;Cell Cycle;Apoptosis;Adaptive Immune Response | mmu04145;mmu04540 |  | Tubb3 | slateblue2 | 16 | -4.20253164556962 |
  | Slc6a1-mRNA | 0.477 | 0.118 | 0.245 | 0.709 | 1.39 | 1.18 | 1.63 | 0.000971 | 0.0274 | lm.nb | Astrocyte Function, Neurons and Neurotransmission | NS\_MM\_NEUROINFLAM\_V1.0 | Slc6a1 | mRNA | 0 |  | Neurons and Neurotransmission;Astrocyte Function |  |  | Slc6a1 | slateblue2 | 16 | 4.04237288135593 |
  | P2rx7-mRNA | -1.26 | 0.332 | -1.91 | -0.606 | 0.419 | 0.267 | 0.657 | 0.00162 | 0.0403 | lm.nb | Innate Immune Response, Microglia Function, Neurons and Neurotransmission | NS\_MM\_NEUROINFLAM\_V1.0 | P2rx7 | mRNA | 0 |  | Neurons and Neurotransmission;Microglia Function;Innate Immune Response | mmu04020;mmu04080 |  | P2rx7 | slateblue2 | 16 | -3.79518072289157 |
  | Dlg4-mRNA | -0.204 | 0.056 | -0.313 | -0.0938 | 0.868 | 0.805 | 0.937 | 0.00223 | 0.0534 | loglinear | Adaptive Immune Response, Angiogenesis, Cytokine Signaling, Growth Factor Signaling, Insulin Signaling, Neurons and Neurotransmission | NS\_MM\_NEUROINFLAM\_V1.0 | Dlg4 | mRNA | 0 |  | Neurons and Neurotransmission;Insulin Signaling;Growth Factor Signaling;Cytokine Signaling;Angiogenesis;Adaptive Immune Response | mmu04390;mmu04724;mmu05016;mmu05030 |  | Dlg4 | slateblue3 | 16 | -3.64285714285714 |
  | Apoe-mRNA | 0.298 | 0.0826 | 0.136 | 0.46 | 1.23 | 1.1 | 1.38 | 0.00237 | 0.055 | loglinear | Astrocyte Function, Cellular Stress, Lipid Metabolism, Microglia Function, Neurons and Neurotransmission | NS\_MM\_NEUROINFLAM\_V1.0 | Apoe | mRNA | 0 |  | Neurons and Neurotransmission;Microglia Function;Lipid Metabolism;Cellular Stress;Astrocyte Function | mmu05010 |  | Apoe | slateblue3 | 16 | 3.60774818401937 |
  | Rala-mRNA | -0.395 | 0.11 | -0.61 | -0.18 | 0.761 | 0.655 | 0.883 | 0.00238 | 0.055 | loglinear | Autophagy, Growth Factor Signaling, Neurons and Neurotransmission | NS\_MM\_NEUROINFLAM\_V1.0 | Rala | mRNA | 0 |  | Neurons and Neurotransmission;Growth Factor Signaling;Autophagy | mmu04014;mmu04015;mmu04072;mmu05200;mmu05212 |  | Rala | slateblue3 | 16 | -3.59090909090909 |

  ##### DE Results - Neurons and Neurotransmission genes - DPI: 3 vs.CTRL

  Table displaying each sample's global significance scores and directed global significance scores as defined in the heatmaps above. The global significance score is calculated as the square root of the mean squared t-statistic for the genes in a gene set, with t-statistics coming from the linear regression underlying our differential expression analysis. The directed global significance score is calculated as the square root of the mean signed squared t-statistic for the genes in a gene set, with t-statistics coming from the linear regression underlying our differential expression analysis.

DPI:
- DPI: differential expression in 1 vs. baseline of CTRL
- DPI: differential expression in 2 vs. baseline of CTRL
- DPI: differential expression in 3 vs. baseline of CTRL

- **Volcano Plot: DPI: 1 vs.CTRL  
  More Plot Information**


  ##### Volcano Plot: DPI: 1 vs.CTRL

  Volcano plot displaying each gene's -log10(p-value) and log2 fold change for the selected covariate. Highly statistically significant genes fall at the top of the plot, and highly differentially expressed genes fall to either side. Genes within the selected gene set are highlighted in orange. Horizontal lines indicate various False Discovery Rate (FDR) thresholds.
- **DE Results - NF-kB genes - DPI: 1 vs.CTRL  
  More Plot Information  Download CSV Data**

  | Probe Label | Log2 fold change | std error (log2) | Lower confidence limit (log2) | Upper confidence limit (log2) | Linear fold change | Lower confidence limit (linear) | Upper confidence limit (linear) | P-value | BY.p.value | method | Gene.sets | Codeset.Name | Probe.Label | Analyte.Type | Is.Control | Control.Type | Probe.Annotation | KEGG.Pathways | Cell.Type | Official.Gene.Name | volcanocol | volcanopch | tstats.all |
  | --- | --- | --- | --- | --- | --- | --- | --- | --- | --- | --- | --- | --- | --- | --- | --- | --- | --- | --- | --- | --- | --- | --- | --- |
  | Parp1-mRNA | -0.49 | 0.108 | -0.703 | -0.278 | 0.712 | 0.614 | 0.825 | 0.000348 | 0.0564 | loglinear | Apoptosis, Cellular Stress, DNA Damage, NF-kB | NS\_MM\_NEUROINFLAM\_V1.0 | Parp1 | mRNA | 0 |  | NF-kB;DNA Damage;Cellular Stress;Apoptosis | mmu03410;mmu04064 |  | Parp1 | slateblue3 | 16 | -4.53703703703704 |
  | Psmb8-mRNA | 1.36 | 0.365 | 0.649 | 2.08 | 2.57 | 1.57 | 4.23 | 0.00179 | 0.129 | lm.nb | Adaptive Immune Response, Angiogenesis, Apoptosis, Astrocyte Function, Cell Cycle, Cytokine Signaling, Growth Factor Signaling, Inflammatory Signaling, Insulin Signaling, Microglia Function, NF-kB, Wnt | NS\_MM\_NEUROINFLAM\_V1.0 | Psmb8 | mRNA | 0 |  | Wnt;NF-kB;Microglia Function;Insulin Signaling;Inflammatory Signaling;Growth Factor Signaling;Cytokine Signaling;Cell Cycle;Astrocyte Function;Apoptosis;Angiogenesis;Adaptive Immune Response |  |  | Psmb8 | slateblue4 | 16 | 3.72602739726027 |
  | Xiap-mRNA | -0.529 | 0.155 | -0.833 | -0.225 | 0.693 | 0.561 | 0.856 | 0.00359 | 0.184 | lm.nb | Apoptosis, Innate Immune Response, NF-kB, Wnt | NS\_MM\_NEUROINFLAM\_V1.0 | Xiap | mRNA | 0 |  | Wnt;NF-kB;Innate Immune Response;Apoptosis | mmu04064;mmu04120;mmu04210;mmu04510;mmu05145;mmu05166;mmu05200;mmu05222 |  | Xiap | slateblue4 | 16 | -3.41290322580645 |
  | Traf6-mRNA | 0.546 | 0.169 | 0.215 | 0.877 | 1.46 | 1.16 | 1.84 | 0.00523 | 0.215 | lm.nb | Adaptive Immune Response, Apoptosis, Autophagy, Cytokine Signaling, Growth Factor Signaling, Innate Immune Response, NF-kB | NS\_MM\_NEUROINFLAM\_V1.0 | Traf6 | mRNA | 0 |  | NF-kB;Innate Immune Response;Growth Factor Signaling;Cytokine Signaling;Autophagy;Apoptosis;Adaptive Immune Response | mmu04010;mmu04064;mmu04120;mmu04144;mmu04380;mmu04620;mmu04621;mmu04622;mmu04722;mmu05133;mmu05140;mmu05142;mmu05145;mmu05152;mmu05160;mmu05162;mmu05168;mmu05169;mmu05200;mmu05222 |  | Traf6 | slateblue4 | 16 | 3.23076923076923 |
  | Irak1-mRNA | 0.531 | 0.191 | 0.157 | 0.904 | 1.44 | 1.11 | 1.87 | 0.0133 | 0.367 | lm.nb | Apoptosis, Cytokine Signaling, Growth Factor Signaling, Innate Immune Response, NF-kB | NS\_MM\_NEUROINFLAM\_V1.0 | Irak1 | mRNA | 0 |  | NF-kB;Innate Immune Response;Growth Factor Signaling;Cytokine Signaling;Apoptosis | mmu04064;mmu04210;mmu04620;mmu04722;mmu05133;mmu05140;mmu05142;mmu05145;mmu05152;mmu05162;mmu05169 |  | Irak1 | slateblue4 | 16 | 2.78010471204188 |
  | Bcl10-mRNA | -1.3 | 0.502 | -2.28 | -0.311 | 0.407 | 0.206 | 0.806 | 0.0201 | 0.468 | lm.nb | Adaptive Immune Response, Apoptosis, NF-kB | NS\_MM\_NEUROINFLAM\_V1.0 | Bcl10 | mRNA | 0 |  | NF-kB;Apoptosis;Adaptive Immune Response | mmu04064;mmu04660;mmu04662;mmu05152 |  | Bcl10 | slateblue4 | 16 | -2.58964143426295 |
  | Nfkb1-mRNA | -0.581 | 0.234 | -1.04 | -0.123 | 0.669 | 0.487 | 0.918 | 0.0244 | 0.533 | lm.nb | Adaptive Immune Response, Apoptosis, Cellular Stress, Cytokine Signaling, Growth Factor Signaling, Inflammatory Signaling, Innate Immune Response, NF-kB | NS\_MM\_NEUROINFLAM\_V1.0 | Nfkb1 | mRNA | 0 |  | NF-kB;Innate Immune Response;Inflammatory Signaling;Growth Factor Signaling;Cytokine Signaling;Cellular Stress;Apoptosis;Adaptive Immune Response | mmu04010;mmu04014;mmu04024;mmu04062;mmu04064;mmu04066;mmu04071;mmu04151;mmu04210;mmu04211;mmu04380;mmu04620;mmu04621;mmu04622;mmu04623;mmu04660;mmu04662;mmu04668;mmu04722;mmu04917;mmu04920;mmu04931;mmu04932;mmu04933;mmu05030;mmu05132;mmu05133;mmu05134;mmu05140;mmu05142;mmu05145;mmu05146;mmu05152;mmu05160;mmu05161;mmu05162;mmu05164;mmu05166;mmu05168;mmu05169;mmu05200;mmu05202;mmu05203;mmu05206;mmu05212;mmu05215;mmu05220;mmu05221;mmu05222;mmu05321 |  | Nfkb1 | azure4 | 1 | -2.48290598290598 |
  | Plcg2-mRNA | 0.903 | 0.414 | 0.0904 | 1.71 | 1.87 | 1.06 | 3.28 | 0.0447 | 0.777 | lm.nb | Adaptive Immune Response, Growth Factor Signaling, Innate Immune Response, NF-kB | NS\_MM\_NEUROINFLAM\_V1.0 | Plcg2 | mRNA | 0 |  | NF-kB;Innate Immune Response;Growth Factor Signaling;Adaptive Immune Response | mmu00562;mmu04012;mmu04014;mmu04020;mmu04064;mmu04066;mmu04070;mmu04072;mmu04370;mmu04380;mmu04611;mmu04650;mmu04662;mmu04664;mmu04666;mmu04670;mmu04722;mmu04750;mmu04919;mmu04933;mmu05169;mmu05200;mmu05205;mmu05206;mmu05214;mmu05223 |  | Plcg2 | azure4 | 1 | 2.18115942028986 |
  | Traf2-mRNA | -0.257 | 0.126 | -0.505 | -0.0103 | 0.837 | 0.705 | 0.993 | 0.058 | 0.918 | lm.nb | Apoptosis, Cytokine Signaling, Growth Factor Signaling, Inflammatory Signaling, Innate Immune Response, NF-kB | NS\_MM\_NEUROINFLAM\_V1.0 | Traf2 | mRNA | 0 |  | NF-kB;Innate Immune Response;Inflammatory Signaling;Growth Factor Signaling;Cytokine Signaling;Apoptosis | mmu04010;mmu04064;mmu04071;mmu04141;mmu04210;mmu04380;mmu04622;mmu04668;mmu04920;mmu04932;mmu05160;mmu05168;mmu05169;mmu05200;mmu05203;mmu05222 |  | Traf2 | azure4 | 1 | -2.03968253968254 |
  | Tnfrsf12a-mRNA | -0.72 | 0.359 | -1.42 | -0.0165 | 0.607 | 0.373 | 0.989 | 0.0621 | 0.948 | lm.nb | Cytokine Signaling, Inflammatory Signaling, NF-kB | NS\_MM\_NEUROINFLAM\_V1.0 | Tnfrsf12a | mRNA | 0 |  | NF-kB;Inflammatory Signaling;Cytokine Signaling | mmu04060 |  | Tnfrsf12a | azure4 | 1 | -2.00557103064067 |
  | Atm-mRNA | 0.353 | 0.186 | -0.0106 | 0.717 | 1.28 | 0.993 | 1.64 | 0.0752 | 1 | lm.nb | Apoptosis, Cell Cycle, Cellular Stress, DNA Damage, NF-kB | NS\_MM\_NEUROINFLAM\_V1.0 | Atm | mRNA | 0 |  | NF-kB;DNA Damage;Cellular Stress;Cell Cycle;Apoptosis | mmu04064;mmu04068;mmu04110;mmu04115;mmu04210;mmu05166;mmu05202;mmu05206 |  | Atm | azure4 | 1 | 1.89784946236559 |
  | Traf3-mRNA | -0.195 | 0.104 | -0.398 | 0.00834 | 0.874 | 0.759 | 1.01 | 0.0785 | 1 | loglinear | Apoptosis, Cytokine Signaling, Inflammatory Signaling, Innate Immune Response, NF-kB | NS\_MM\_NEUROINFLAM\_V1.0 | Traf3 | mRNA | 0 |  | NF-kB;Innate Immune Response;Inflammatory Signaling;Cytokine Signaling;Apoptosis | mmu04064;mmu04620;mmu04622;mmu04668;mmu05160;mmu05168;mmu05169;mmu05200;mmu05203;mmu05222 |  | Traf3 | azure4 | 1 | -1.875 |
  | Bcl2l1-mRNA | 0.135 | 0.0734 | -0.0091 | 0.279 | 1.1 | 0.994 | 1.21 | 0.085 | 1 | loglinear | Apoptosis, Autophagy, Cytokine Signaling, Growth Factor Signaling, Innate Immune Response, NF-kB | NS\_MM\_NEUROINFLAM\_V1.0 | Bcl2l1 | mRNA | 0 |  | NF-kB;Innate Immune Response;Growth Factor Signaling;Cytokine Signaling;Autophagy;Apoptosis | mmu04014;mmu04064;mmu04151;mmu04210;mmu04630;mmu05014;mmu05145;mmu05166;mmu05200;mmu05202;mmu05212;mmu05220;mmu05222 |  | Bcl2l1 | azure4 | 1 | 1.83923705722071 |
  | Prkcq-mRNA | -0.287 | 0.17 | -0.62 | 0.0467 | 0.82 | 0.651 | 1.03 | 0.111 | 1 | lm.nb | Adaptive Immune Response, Apoptosis, Autophagy, NF-kB, Oligodendrocyte Function | NS\_MM\_NEUROINFLAM\_V1.0 | Prkcq | mRNA | 0 |  | Oligodendrocyte Function;NF-kB;Autophagy;Apoptosis;Adaptive Immune Response | mmu04064;mmu04270;mmu04530;mmu04660;mmu04750;mmu04920;mmu04931;mmu05162 | Oligodendrocytes | Prkcq | azure4 | 1 | -1.68823529411765 |
  | Birc2-mRNA | -0.208 | 0.127 | -0.457 | 0.0399 | 0.866 | 0.729 | 1.03 | 0.119 | 1 | lm.nb | Apoptosis, Cytokine Signaling, Innate Immune Response, NF-kB | NS\_MM\_NEUROINFLAM\_V1.0 | Birc2 | mRNA | 0 |  | NF-kB;Innate Immune Response;Cytokine Signaling;Apoptosis | mmu04064;mmu04120;mmu04210;mmu04390;mmu04510;mmu04621;mmu04668;mmu05145;mmu05200;mmu05222 |  | Birc2 | azure4 | 1 | -1.63779527559055 |
  | Tnfrsf1a-mRNA | 0.488 | 0.301 | -0.101 | 1.08 | 1.4 | 0.932 | 2.11 | 0.124 | 1 | lm.nb | Apoptosis, Cytokine Signaling, Growth Factor Signaling, Innate Immune Response, NF-kB | NS\_MM\_NEUROINFLAM\_V1.0 | Tnfrsf1a | mRNA | 0 |  | NF-kB;Innate Immune Response;Growth Factor Signaling;Cytokine Signaling;Apoptosis | mmu04010;mmu04060;mmu04064;mmu04071;mmu04210;mmu04380;mmu04668;mmu04920;mmu04931;mmu04932;mmu05010;mmu05014;mmu05142;mmu05145;mmu05152;mmu05160;mmu05164;mmu05166;mmu05168 |  | Tnfrsf1a | azure4 | 1 | 1.62126245847176 |
  | Bcl2-mRNA | -0.326 | 0.208 | -0.735 | 0.0825 | 0.798 | 0.601 | 1.06 | 0.137 | 1 | lm.nb | Apoptosis, Autophagy, Cellular Stress, Cytokine Signaling, DNA Damage, Growth Factor Signaling, Innate Immune Response, Microglia Function, NF-kB | NS\_MM\_NEUROINFLAM\_V1.0 | Bcl2 | mRNA | 0 |  | NF-kB;Microglia Function;Innate Immune Response;Growth Factor Signaling;DNA Damage;Cytokine Signaling;Cellular Stress;Autophagy;Apoptosis | mmu04064;mmu04066;mmu04071;mmu04141;mmu04151;mmu04210;mmu04261;mmu04510;mmu04630;mmu04722;mmu04725;mmu04933;mmu05014;mmu05145;mmu05152;mmu05161;mmu05169;mmu05200;mmu05206;mmu05210;mmu05215;mmu05222 |  | Bcl2 | azure4 | 1 | -1.56730769230769 |
  | Ltbr-mRNA | 0.381 | 0.278 | -0.164 | 0.925 | 1.3 | 0.892 | 1.9 | 0.19 | 1 | lm.nb | Apoptosis, Cytokine Signaling, Inflammatory Signaling, NF-kB | NS\_MM\_NEUROINFLAM\_V1.0 | Ltbr | mRNA | 0 |  | NF-kB;Inflammatory Signaling;Cytokine Signaling;Apoptosis | mmu04060;mmu04064;mmu04066;mmu04672;mmu05166;mmu05203 |  | Ltbr | azure4 | 1 | 1.3705035971223 |
  | Tnfsf12-mRNA | 0.479 | 0.368 | -0.242 | 1.2 | 1.39 | 0.846 | 2.3 | 0.211 | 1 | lm.nb | Cytokine Signaling, Inflammatory Signaling, NF-kB | NS\_MM\_NEUROINFLAM\_V1.0 | Tnfsf12 | mRNA | 0 |  | NF-kB;Inflammatory Signaling;Cytokine Signaling | mmu04060 |  | Tnfsf12 | azure4 | 1 | 1.30163043478261 |
  | Ptgs2-mRNA | -0.304 | 0.251 | -0.795 | 0.188 | 0.81 | 0.576 | 1.14 | 0.243 | 1 | lm.nb | Astrocyte Function, Cellular Stress, Innate Immune Response, Lipid Metabolism, Microglia Function, NF-kB | NS\_MM\_NEUROINFLAM\_V1.0 | Ptgs2 | mRNA | 0 |  | NF-kB;Microglia Function;Lipid Metabolism;Innate Immune Response;Cellular Stress;Astrocyte Function | mmu00590;mmu04064;mmu04370;mmu04668;mmu04723;mmu04726;mmu04913;mmu04921;mmu04923;mmu05140;mmu05200;mmu05206;mmu05222 |  | Ptgs2 | azure4 | 1 | -1.21115537848606 |

  ##### DE Results - NF-kB genes - DPI: 1 vs.CTRL

  Table displaying each sample's global significance scores and directed global significance scores as defined in the heatmaps above. The global significance score is calculated as the square root of the mean squared t-statistic for the genes in a gene set, with t-statistics coming from the linear regression underlying our differential expression analysis. The directed global significance score is calculated as the square root of the mean signed squared t-statistic for the genes in a gene set, with t-statistics coming from the linear regression underlying our differential expression analysis.

- **Volcano Plot: DPI: 2 vs.CTRL  
  More Plot Information**


  ##### Volcano Plot: DPI: 2 vs.CTRL

  Volcano plot displaying each gene's -log10(p-value) and log2 fold change for the selected covariate. Highly statistically significant genes fall at the top of the plot, and highly differentially expressed genes fall to either side. Genes within the selected gene set are highlighted in orange. Horizontal lines indicate various False Discovery Rate (FDR) thresholds.
- **DE Results - NF-kB genes - DPI: 2 vs.CTRL  
  More Plot Information  Download CSV Data**

  | Probe Label | Log2 fold change | std error (log2) | Lower confidence limit (log2) | Upper confidence limit (log2) | Linear fold change | Lower confidence limit (linear) | Upper confidence limit (linear) | P-value | BY.p.value | method | Gene.sets | Codeset.Name | Probe.Label | Analyte.Type | Is.Control | Control.Type | Probe.Annotation | KEGG.Pathways | Cell.Type | Official.Gene.Name | volcanocol | volcanopch | tstats.all |
  | --- | --- | --- | --- | --- | --- | --- | --- | --- | --- | --- | --- | --- | --- | --- | --- | --- | --- | --- | --- | --- | --- | --- | --- |
  | Nfkbia-mRNA | 1.86 | 0.228 | 1.41 | 2.31 | 3.63 | 2.67 | 4.95 | 4.26e-07 | 9.99e-05 | lm.nb | Adaptive Immune Response, Apoptosis, Growth Factor Signaling, Inflammatory Signaling, Innate Immune Response, NF-kB | NS\_MM\_NEUROINFLAM\_V1.0 | Nfkbia | mRNA | 0 |  | NF-kB;Innate Immune Response;Inflammatory Signaling;Growth Factor Signaling;Apoptosis;Adaptive Immune Response | mmu04024;mmu04062;mmu04064;mmu04210;mmu04380;mmu04620;mmu04621;mmu04622;mmu04623;mmu04660;mmu04662;mmu04668;mmu04722;mmu04920;mmu04931;mmu05134;mmu05140;mmu05142;mmu05145;mmu05160;mmu05161;mmu05162;mmu05164;mmu05166;mmu05168;mmu05169;mmu05200;mmu05203;mmu05215;mmu05220;mmu05222 |  | Nfkbia | slateblue1 | 16 | 8.15789473684211 |
  | Psmb8-mRNA | 2.23 | 0.352 | 1.54 | 2.92 | 4.7 | 2.91 | 7.59 | 9.89e-06 | 0.000997 | lm.nb | Adaptive Immune Response, Angiogenesis, Apoptosis, Astrocyte Function, Cell Cycle, Cytokine Signaling, Growth Factor Signaling, Inflammatory Signaling, Insulin Signaling, Microglia Function, NF-kB, Wnt | NS\_MM\_NEUROINFLAM\_V1.0 | Psmb8 | mRNA | 0 |  | Wnt;NF-kB;Microglia Function;Insulin Signaling;Inflammatory Signaling;Growth Factor Signaling;Cytokine Signaling;Cell Cycle;Astrocyte Function;Apoptosis;Angiogenesis;Adaptive Immune Response |  |  | Psmb8 | slateblue1 | 16 | 6.33522727272727 |
  | Bcl2l1-mRNA | 0.407 | 0.0734 | 0.263 | 0.551 | 1.33 | 1.2 | 1.46 | 4.51e-05 | 0.00306 | loglinear | Apoptosis, Autophagy, Cytokine Signaling, Growth Factor Signaling, Innate Immune Response, NF-kB | NS\_MM\_NEUROINFLAM\_V1.0 | Bcl2l1 | mRNA | 0 |  | NF-kB;Innate Immune Response;Growth Factor Signaling;Cytokine Signaling;Autophagy;Apoptosis | mmu04014;mmu04064;mmu04151;mmu04210;mmu04630;mmu05014;mmu05145;mmu05166;mmu05200;mmu05202;mmu05212;mmu05220;mmu05222 |  | Bcl2l1 | slateblue1 | 16 | 5.54495912806539 |
  | Prkcq-mRNA | -0.864 | 0.185 | -1.23 | -0.501 | 0.549 | 0.427 | 0.707 | 0.000258 | 0.0106 | lm.nb | Adaptive Immune Response, Apoptosis, Autophagy, NF-kB, Oligodendrocyte Function | NS\_MM\_NEUROINFLAM\_V1.0 | Prkcq | mRNA | 0 |  | Oligodendrocyte Function;NF-kB;Autophagy;Apoptosis;Adaptive Immune Response | mmu04064;mmu04270;mmu04530;mmu04660;mmu04750;mmu04920;mmu04931;mmu05162 | Oligodendrocytes | Prkcq | slateblue2 | 16 | -4.67027027027027 |
  | Traf6-mRNA | 0.756 | 0.167 | 0.428 | 1.08 | 1.69 | 1.35 | 2.12 | 0.000353 | 0.0135 | lm.nb | Adaptive Immune Response, Apoptosis, Autophagy, Cytokine Signaling, Growth Factor Signaling, Innate Immune Response, NF-kB | NS\_MM\_NEUROINFLAM\_V1.0 | Traf6 | mRNA | 0 |  | NF-kB;Innate Immune Response;Growth Factor Signaling;Cytokine Signaling;Autophagy;Apoptosis;Adaptive Immune Response | mmu04010;mmu04064;mmu04120;mmu04144;mmu04380;mmu04620;mmu04621;mmu04622;mmu04722;mmu05133;mmu05140;mmu05142;mmu05145;mmu05152;mmu05160;mmu05162;mmu05168;mmu05169;mmu05200;mmu05222 |  | Traf6 | slateblue2 | 16 | 4.52694610778443 |
  | Chuk-mRNA | -0.331 | 0.0787 | -0.485 | -0.176 | 0.795 | 0.715 | 0.885 | 0.000678 | 0.0218 | loglinear | Adaptive Immune Response, Apoptosis, Cytokine Signaling, Growth Factor Signaling, Inflammatory Signaling, Innate Immune Response, NF-kB | NS\_MM\_NEUROINFLAM\_V1.0 | Chuk | mRNA | 0 |  | NF-kB;Innate Immune Response;Inflammatory Signaling;Growth Factor Signaling;Cytokine Signaling;Apoptosis;Adaptive Immune Response | mmu04010;mmu04014;mmu04062;mmu04064;mmu04068;mmu04151;mmu04210;mmu04380;mmu04620;mmu04621;mmu04622;mmu04623;mmu04660;mmu04662;mmu04668;mmu04920;mmu05142;mmu05145;mmu05160;mmu05161;mmu05162;mmu05166;mmu05168;mmu05169;mmu05200;mmu05212;mmu05215;mmu05220;mmu05221;mmu05222 |  | Chuk | slateblue2 | 16 | -4.20584498094028 |
  | Tnfrsf1a-mRNA | 1.14 | 0.29 | 0.575 | 1.71 | 2.21 | 1.49 | 3.27 | 0.00116 | 0.0296 | lm.nb | Apoptosis, Cytokine Signaling, Growth Factor Signaling, Innate Immune Response, NF-kB | NS\_MM\_NEUROINFLAM\_V1.0 | Tnfrsf1a | mRNA | 0 |  | NF-kB;Innate Immune Response;Growth Factor Signaling;Cytokine Signaling;Apoptosis | mmu04010;mmu04060;mmu04064;mmu04071;mmu04210;mmu04380;mmu04668;mmu04920;mmu04931;mmu04932;mmu05010;mmu05014;mmu05142;mmu05145;mmu05152;mmu05160;mmu05164;mmu05166;mmu05168 |  | Tnfrsf1a | slateblue2 | 16 | 3.93103448275862 |
  | Bcl2-mRNA | -0.88 | 0.225 | -1.32 | -0.439 | 0.543 | 0.4 | 0.737 | 0.00123 | 0.0313 | lm.nb | Apoptosis, Autophagy, Cellular Stress, Cytokine Signaling, DNA Damage, Growth Factor Signaling, Innate Immune Response, Microglia Function, NF-kB | NS\_MM\_NEUROINFLAM\_V1.0 | Bcl2 | mRNA | 0 |  | NF-kB;Microglia Function;Innate Immune Response;Growth Factor Signaling;DNA Damage;Cytokine Signaling;Cellular Stress;Autophagy;Apoptosis | mmu04064;mmu04066;mmu04071;mmu04141;mmu04151;mmu04210;mmu04261;mmu04510;mmu04630;mmu04722;mmu04725;mmu04933;mmu05014;mmu05145;mmu05152;mmu05161;mmu05169;mmu05200;mmu05206;mmu05210;mmu05215;mmu05222 |  | Bcl2 | slateblue2 | 16 | -3.91111111111111 |
  | Parp1-mRNA | -0.406 | 0.108 | -0.619 | -0.194 | 0.754 | 0.651 | 0.874 | 0.00175 | 0.0406 | loglinear | Apoptosis, Cellular Stress, DNA Damage, NF-kB | NS\_MM\_NEUROINFLAM\_V1.0 | Parp1 | mRNA | 0 |  | NF-kB;DNA Damage;Cellular Stress;Apoptosis | mmu03410;mmu04064 |  | Parp1 | slateblue2 | 16 | -3.75925925925926 |
  | Xiap-mRNA | -0.58 | 0.155 | -0.884 | -0.276 | 0.669 | 0.542 | 0.826 | 0.0018 | 0.0414 | lm.nb | Apoptosis, Innate Immune Response, NF-kB, Wnt | NS\_MM\_NEUROINFLAM\_V1.0 | Xiap | mRNA | 0 |  | Wnt;NF-kB;Innate Immune Response;Apoptosis | mmu04064;mmu04120;mmu04210;mmu04510;mmu05145;mmu05166;mmu05200;mmu05222 |  | Xiap | slateblue2 | 16 | -3.74193548387097 |
  | Relb-mRNA | -0.486 | 0.137 | -0.755 | -0.218 | 0.714 | 0.593 | 0.86 | 0.00268 | 0.0557 | loglinear | Cytokine Signaling, Growth Factor Signaling, Inflammatory Signaling, NF-kB | NS\_MM\_NEUROINFLAM\_V1.0 | Relb | mRNA | 0 |  | NF-kB;Inflammatory Signaling;Growth Factor Signaling;Cytokine Signaling | mmu04010;mmu04064;mmu04380;mmu05166;mmu05169 |  | Relb | slateblue3 | 16 | -3.54744525547445 |
  | Ikbkb-mRNA | -0.299 | 0.0868 | -0.47 | -0.129 | 0.813 | 0.722 | 0.914 | 0.00331 | 0.0665 | loglinear | Adaptive Immune Response, Apoptosis, Cytokine Signaling, Growth Factor Signaling, Inflammatory Signaling, Innate Immune Response, NF-kB | NS\_MM\_NEUROINFLAM\_V1.0 | Ikbkb | mRNA | 0 |  | NF-kB;Innate Immune Response;Inflammatory Signaling;Growth Factor Signaling;Cytokine Signaling;Apoptosis;Adaptive Immune Response | mmu04010;mmu04014;mmu04062;mmu04064;mmu04068;mmu04150;mmu04151;mmu04210;mmu04380;mmu04620;mmu04621;mmu04622;mmu04623;mmu04660;mmu04662;mmu04668;mmu04722;mmu04910;mmu04920;mmu04930;mmu04931;mmu04932;mmu05142;mmu05145;mmu05160;mmu05161;mmu05164;mmu05166;mmu05168;mmu05169;mmu05200;mmu05206;mmu05212;mmu05215;mmu05220;mmu05221;mmu05222 |  | Ikbkb | slateblue3 | 16 | -3.44470046082949 |
  | Irak1-mRNA | 0.604 | 0.19 | 0.231 | 0.976 | 1.52 | 1.17 | 1.97 | 0.00587 | 0.108 | lm.nb | Apoptosis, Cytokine Signaling, Growth Factor Signaling, Innate Immune Response, NF-kB | NS\_MM\_NEUROINFLAM\_V1.0 | Irak1 | mRNA | 0 |  | NF-kB;Innate Immune Response;Growth Factor Signaling;Cytokine Signaling;Apoptosis | mmu04064;mmu04210;mmu04620;mmu04722;mmu05133;mmu05140;mmu05142;mmu05145;mmu05152;mmu05162;mmu05169 |  | Irak1 | slateblue4 | 16 | 3.17894736842105 |
  | Traf2-mRNA | -0.396 | 0.128 | -0.646 | -0.146 | 0.76 | 0.639 | 0.904 | 0.00685 | 0.121 | lm.nb | Apoptosis, Cytokine Signaling, Growth Factor Signaling, Inflammatory Signaling, Innate Immune Response, NF-kB | NS\_MM\_NEUROINFLAM\_V1.0 | Traf2 | mRNA | 0 |  | NF-kB;Innate Immune Response;Inflammatory Signaling;Growth Factor Signaling;Cytokine Signaling;Apoptosis | mmu04010;mmu04064;mmu04071;mmu04141;mmu04210;mmu04380;mmu04622;mmu04668;mmu04920;mmu04932;mmu05160;mmu05168;mmu05169;mmu05200;mmu05203;mmu05222 |  | Traf2 | slateblue4 | 16 | -3.09375 |
  | Tnfrsf12a-mRNA | -1.1 | 0.363 | -1.81 | -0.384 | 0.468 | 0.286 | 0.766 | 0.00814 | 0.137 | lm.nb | Cytokine Signaling, Inflammatory Signaling, NF-kB | NS\_MM\_NEUROINFLAM\_V1.0 | Tnfrsf12a | mRNA | 0 |  | NF-kB;Inflammatory Signaling;Cytokine Signaling | mmu04060 |  | Tnfrsf12a | slateblue4 | 16 | -3.03030303030303 |
  | Plcg2-mRNA | 1.2 | 0.409 | 0.403 | 2.01 | 2.3 | 1.32 | 4.02 | 0.0095 | 0.157 | lm.nb | Adaptive Immune Response, Growth Factor Signaling, Innate Immune Response, NF-kB | NS\_MM\_NEUROINFLAM\_V1.0 | Plcg2 | mRNA | 0 |  | NF-kB;Innate Immune Response;Growth Factor Signaling;Adaptive Immune Response | mmu00562;mmu04012;mmu04014;mmu04020;mmu04064;mmu04066;mmu04070;mmu04072;mmu04370;mmu04380;mmu04611;mmu04650;mmu04662;mmu04664;mmu04666;mmu04670;mmu04722;mmu04750;mmu04919;mmu04933;mmu05169;mmu05200;mmu05205;mmu05206;mmu05214;mmu05223 |  | Plcg2 | slateblue4 | 16 | 2.93398533007335 |
  | Bcl10-mRNA | -1.33 | 0.502 | -2.31 | -0.341 | 0.399 | 0.202 | 0.789 | 0.0178 | 0.252 | lm.nb | Adaptive Immune Response, Apoptosis, NF-kB | NS\_MM\_NEUROINFLAM\_V1.0 | Bcl10 | mRNA | 0 |  | NF-kB;Apoptosis;Adaptive Immune Response | mmu04064;mmu04660;mmu04662;mmu05152 |  | Bcl10 | slateblue4 | 16 | -2.64940239043825 |
  | Map3k14-mRNA | 0.942 | 0.37 | 0.216 | 1.67 | 1.92 | 1.16 | 3.18 | 0.0217 | 0.287 | lm.nb | Adaptive Immune Response, Apoptosis, Cytokine Signaling, Growth Factor Signaling, Inflammatory Signaling, Innate Immune Response, NF-kB | NS\_MM\_NEUROINFLAM\_V1.0 | Map3k14 | mRNA | 0 |  | NF-kB;Innate Immune Response;Inflammatory Signaling;Growth Factor Signaling;Cytokine Signaling;Apoptosis;Adaptive Immune Response | mmu04010;mmu04064;mmu04210;mmu04380;mmu04660;mmu04668;mmu04672;mmu05166;mmu05169 |  | Map3k14 | slateblue4 | 16 | 2.54594594594595 |
  | Ikbkg-mRNA | 0.296 | 0.139 | 0.0237 | 0.568 | 1.23 | 1.02 | 1.48 | 0.049 | 0.586 | loglinear | Adaptive Immune Response, Apoptosis, Cytokine Signaling, Growth Factor Signaling, Inflammatory Signaling, Innate Immune Response, NF-kB | NS\_MM\_NEUROINFLAM\_V1.0 | Ikbkg | mRNA | 0 |  | NF-kB;Innate Immune Response;Inflammatory Signaling;Growth Factor Signaling;Cytokine Signaling;Apoptosis;Adaptive Immune Response | mmu04010;mmu04014;mmu04062;mmu04064;mmu04151;mmu04210;mmu04380;mmu04620;mmu04621;mmu04622;mmu04623;mmu04660;mmu04662;mmu04668;mmu04920;mmu05142;mmu05145;mmu05160;mmu05161;mmu05166;mmu05168;mmu05169;mmu05200;mmu05203;mmu05212;mmu05215;mmu05220;mmu05221;mmu05222;mmu05340 |  | Ikbkg | azure4 | 1 | 2.1294964028777 |
  | Atm-mRNA | 0.38 | 0.185 | 0.0169 | 0.743 | 1.3 | 1.01 | 1.67 | 0.057 | 0.661 | lm.nb | Apoptosis, Cell Cycle, Cellular Stress, DNA Damage, NF-kB | NS\_MM\_NEUROINFLAM\_V1.0 | Atm | mRNA | 0 |  | NF-kB;DNA Damage;Cellular Stress;Cell Cycle;Apoptosis | mmu04064;mmu04068;mmu04110;mmu04115;mmu04210;mmu05166;mmu05202;mmu05206 |  | Atm | azure4 | 1 | 2.05405405405405 |

  ##### DE Results - NF-kB genes - DPI: 2 vs.CTRL

  Table displaying each sample's global significance scores and directed global significance scores as defined in the heatmaps above. The global significance score is calculated as the square root of the mean squared t-statistic for the genes in a gene set, with t-statistics coming from the linear regression underlying our differential expression analysis. The directed global significance score is calculated as the square root of the mean signed squared t-statistic for the genes in a gene set, with t-statistics coming from the linear regression underlying our differential expression analysis.

- **Volcano Plot: DPI: 3 vs.CTRL  
  More Plot Information**


  ##### Volcano Plot: DPI: 3 vs.CTRL

  Volcano plot displaying each gene's -log10(p-value) and log2 fold change for the selected covariate. Highly statistically significant genes fall at the top of the plot, and highly differentially expressed genes fall to either side. Genes within the selected gene set are highlighted in orange. Horizontal lines indicate various False Discovery Rate (FDR) thresholds.
- **DE Results - NF-kB genes - DPI: 3 vs.CTRL  
  More Plot Information  Download CSV Data**

  | Probe Label | Log2 fold change | std error (log2) | Lower confidence limit (log2) | Upper confidence limit (log2) | Linear fold change | Lower confidence limit (linear) | Upper confidence limit (linear) | P-value | BY.p.value | method | Gene.sets | Codeset.Name | Probe.Label | Analyte.Type | Is.Control | Control.Type | Probe.Annotation | KEGG.Pathways | Cell.Type | Official.Gene.Name | volcanocol | volcanopch | tstats.all |
  | --- | --- | --- | --- | --- | --- | --- | --- | --- | --- | --- | --- | --- | --- | --- | --- | --- | --- | --- | --- | --- | --- | --- | --- |
  | Nfkbia-mRNA | 2.4 | 0.224 | 1.96 | 2.84 | 5.27 | 3.88 | 7.14 | 1.06e-08 | 3.12e-06 | lm.nb | Adaptive Immune Response, Apoptosis, Growth Factor Signaling, Inflammatory Signaling, Innate Immune Response, NF-kB | NS\_MM\_NEUROINFLAM\_V1.0 | Nfkbia | mRNA | 0 |  | NF-kB;Innate Immune Response;Inflammatory Signaling;Growth Factor Signaling;Apoptosis;Adaptive Immune Response | mmu04024;mmu04062;mmu04064;mmu04210;mmu04380;mmu04620;mmu04621;mmu04622;mmu04623;mmu04660;mmu04662;mmu04668;mmu04722;mmu04920;mmu04931;mmu05134;mmu05140;mmu05142;mmu05145;mmu05160;mmu05161;mmu05162;mmu05164;mmu05166;mmu05168;mmu05169;mmu05200;mmu05203;mmu05215;mmu05220;mmu05222 |  | Nfkbia | slateblue1 | 16 | 10.7142857142857 |
  | Psmb8-mRNA | 2.99 | 0.346 | 2.31 | 3.67 | 7.95 | 4.97 | 12.7 | 1.99e-07 | 3.2e-05 | lm.nb | Adaptive Immune Response, Angiogenesis, Apoptosis, Astrocyte Function, Cell Cycle, Cytokine Signaling, Growth Factor Signaling, Inflammatory Signaling, Insulin Signaling, Microglia Function, NF-kB, Wnt | NS\_MM\_NEUROINFLAM\_V1.0 | Psmb8 | mRNA | 0 |  | Wnt;NF-kB;Microglia Function;Insulin Signaling;Inflammatory Signaling;Growth Factor Signaling;Cytokine Signaling;Cell Cycle;Astrocyte Function;Apoptosis;Angiogenesis;Adaptive Immune Response |  |  | Psmb8 | slateblue1 | 16 | 8.64161849710983 |
  | Ptgs2-mRNA | 1.88 | 0.236 | 1.41 | 2.34 | 3.67 | 2.67 | 5.06 | 5.87e-07 | 7.14e-05 | lm.nb | Astrocyte Function, Cellular Stress, Innate Immune Response, Lipid Metabolism, Microglia Function, NF-kB | NS\_MM\_NEUROINFLAM\_V1.0 | Ptgs2 | mRNA | 0 |  | NF-kB;Microglia Function;Lipid Metabolism;Innate Immune Response;Cellular Stress;Astrocyte Function | mmu00590;mmu04064;mmu04370;mmu04668;mmu04723;mmu04726;mmu04913;mmu04921;mmu04923;mmu05140;mmu05200;mmu05206;mmu05222 |  | Ptgs2 | slateblue1 | 16 | 7.96610169491525 |
  | Ikbkb-mRNA | -0.601 | 0.0868 | -0.772 | -0.431 | 0.659 | 0.586 | 0.742 | 3.42e-06 | 0.00028 | loglinear | Adaptive Immune Response, Apoptosis, Cytokine Signaling, Growth Factor Signaling, Inflammatory Signaling, Innate Immune Response, NF-kB | NS\_MM\_NEUROINFLAM\_V1.0 | Ikbkb | mRNA | 0 |  | NF-kB;Innate Immune Response;Inflammatory Signaling;Growth Factor Signaling;Cytokine Signaling;Apoptosis;Adaptive Immune Response | mmu04010;mmu04014;mmu04062;mmu04064;mmu04068;mmu04150;mmu04151;mmu04210;mmu04380;mmu04620;mmu04621;mmu04622;mmu04623;mmu04660;mmu04662;mmu04668;mmu04722;mmu04910;mmu04920;mmu04930;mmu04931;mmu04932;mmu05142;mmu05145;mmu05160;mmu05161;mmu05164;mmu05166;mmu05168;mmu05169;mmu05200;mmu05206;mmu05212;mmu05215;mmu05220;mmu05221;mmu05222 |  | Ikbkb | slateblue1 | 16 | -6.92396313364055 |
  | Tnfrsf1a-mRNA | 1.92 | 0.281 | 1.37 | 2.47 | 3.79 | 2.59 | 5.56 | 3.96e-06 | 0.000317 | lm.nb | Apoptosis, Cytokine Signaling, Growth Factor Signaling, Innate Immune Response, NF-kB | NS\_MM\_NEUROINFLAM\_V1.0 | Tnfrsf1a | mRNA | 0 |  | NF-kB;Innate Immune Response;Growth Factor Signaling;Cytokine Signaling;Apoptosis | mmu04010;mmu04060;mmu04064;mmu04071;mmu04210;mmu04380;mmu04668;mmu04920;mmu04931;mmu04932;mmu05010;mmu05014;mmu05142;mmu05145;mmu05152;mmu05160;mmu05164;mmu05166;mmu05168 |  | Tnfrsf1a | slateblue1 | 16 | 6.83274021352313 |
  | Parp1-mRNA | -0.736 | 0.108 | -0.949 | -0.524 | 0.6 | 0.518 | 0.695 | 4.31e-06 | 0.000334 | loglinear | Apoptosis, Cellular Stress, DNA Damage, NF-kB | NS\_MM\_NEUROINFLAM\_V1.0 | Parp1 | mRNA | 0 |  | NF-kB;DNA Damage;Cellular Stress;Apoptosis | mmu03410;mmu04064 |  | Parp1 | slateblue1 | 16 | -6.81481481481481 |
  | Bcl2l1-mRNA | 0.414 | 0.0734 | 0.27 | 0.558 | 1.33 | 1.21 | 1.47 | 3.75e-05 | 0.00184 | loglinear | Apoptosis, Autophagy, Cytokine Signaling, Growth Factor Signaling, Innate Immune Response, NF-kB | NS\_MM\_NEUROINFLAM\_V1.0 | Bcl2l1 | mRNA | 0 |  | NF-kB;Innate Immune Response;Growth Factor Signaling;Cytokine Signaling;Autophagy;Apoptosis | mmu04014;mmu04064;mmu04151;mmu04210;mmu04630;mmu05014;mmu05145;mmu05166;mmu05200;mmu05202;mmu05212;mmu05220;mmu05222 |  | Bcl2l1 | slateblue1 | 16 | 5.64032697547684 |
  | Traf6-mRNA | 0.92 | 0.166 | 0.594 | 1.25 | 1.89 | 1.51 | 2.37 | 4.54e-05 | 0.00214 | lm.nb | Adaptive Immune Response, Apoptosis, Autophagy, Cytokine Signaling, Growth Factor Signaling, Innate Immune Response, NF-kB | NS\_MM\_NEUROINFLAM\_V1.0 | Traf6 | mRNA | 0 |  | NF-kB;Innate Immune Response;Growth Factor Signaling;Cytokine Signaling;Autophagy;Apoptosis;Adaptive Immune Response | mmu04010;mmu04064;mmu04120;mmu04144;mmu04380;mmu04620;mmu04621;mmu04622;mmu04722;mmu05133;mmu05140;mmu05142;mmu05145;mmu05152;mmu05160;mmu05162;mmu05168;mmu05169;mmu05200;mmu05222 |  | Traf6 | slateblue1 | 16 | 5.54216867469879 |
  | Chuk-mRNA | -0.423 | 0.0787 | -0.577 | -0.269 | 0.746 | 0.67 | 0.83 | 6.21e-05 | 0.00275 | loglinear | Adaptive Immune Response, Apoptosis, Cytokine Signaling, Growth Factor Signaling, Inflammatory Signaling, Innate Immune Response, NF-kB | NS\_MM\_NEUROINFLAM\_V1.0 | Chuk | mRNA | 0 |  | NF-kB;Innate Immune Response;Inflammatory Signaling;Growth Factor Signaling;Cytokine Signaling;Apoptosis;Adaptive Immune Response | mmu04010;mmu04014;mmu04062;mmu04064;mmu04068;mmu04151;mmu04210;mmu04380;mmu04620;mmu04621;mmu04622;mmu04623;mmu04660;mmu04662;mmu04668;mmu04920;mmu05142;mmu05145;mmu05160;mmu05161;mmu05162;mmu05166;mmu05168;mmu05169;mmu05200;mmu05212;mmu05215;mmu05220;mmu05221;mmu05222 |  | Chuk | slateblue1 | 16 | -5.37484116899619 |
  | Traf3-mRNA | -0.463 | 0.104 | -0.666 | -0.26 | 0.725 | 0.63 | 0.835 | 0.000386 | 0.013 | loglinear | Apoptosis, Cytokine Signaling, Inflammatory Signaling, Innate Immune Response, NF-kB | NS\_MM\_NEUROINFLAM\_V1.0 | Traf3 | mRNA | 0 |  | NF-kB;Innate Immune Response;Inflammatory Signaling;Cytokine Signaling;Apoptosis | mmu04064;mmu04620;mmu04622;mmu04668;mmu05160;mmu05168;mmu05169;mmu05200;mmu05203;mmu05222 |  | Traf3 | slateblue2 | 16 | -4.45192307692308 |
  | Traf2-mRNA | -0.552 | 0.129 | -0.805 | -0.298 | 0.682 | 0.572 | 0.813 | 0.000592 | 0.0183 | lm.nb | Apoptosis, Cytokine Signaling, Growth Factor Signaling, Inflammatory Signaling, Innate Immune Response, NF-kB | NS\_MM\_NEUROINFLAM\_V1.0 | Traf2 | mRNA | 0 |  | NF-kB;Innate Immune Response;Inflammatory Signaling;Growth Factor Signaling;Cytokine Signaling;Apoptosis | mmu04010;mmu04064;mmu04071;mmu04141;mmu04210;mmu04380;mmu04622;mmu04668;mmu04920;mmu04932;mmu05160;mmu05168;mmu05169;mmu05200;mmu05203;mmu05222 |  | Traf2 | slateblue2 | 16 | -4.27906976744186 |
  | Bcl2-mRNA | -0.943 | 0.227 | -1.39 | -0.498 | 0.52 | 0.382 | 0.708 | 0.000747 | 0.0225 | lm.nb | Apoptosis, Autophagy, Cellular Stress, Cytokine Signaling, DNA Damage, Growth Factor Signaling, Innate Immune Response, Microglia Function, NF-kB | NS\_MM\_NEUROINFLAM\_V1.0 | Bcl2 | mRNA | 0 |  | NF-kB;Microglia Function;Innate Immune Response;Growth Factor Signaling;DNA Damage;Cytokine Signaling;Cellular Stress;Autophagy;Apoptosis | mmu04064;mmu04066;mmu04071;mmu04141;mmu04151;mmu04210;mmu04261;mmu04510;mmu04630;mmu04722;mmu04725;mmu04933;mmu05014;mmu05145;mmu05152;mmu05161;mmu05169;mmu05200;mmu05206;mmu05210;mmu05215;mmu05222 |  | Bcl2 | slateblue2 | 16 | -4.15418502202643 |
  | Prkcq-mRNA | -0.735 | 0.181 | -1.09 | -0.379 | 0.601 | 0.47 | 0.769 | 0.000927 | 0.0267 | lm.nb | Adaptive Immune Response, Apoptosis, Autophagy, NF-kB, Oligodendrocyte Function | NS\_MM\_NEUROINFLAM\_V1.0 | Prkcq | mRNA | 0 |  | Oligodendrocyte Function;NF-kB;Autophagy;Apoptosis;Adaptive Immune Response | mmu04064;mmu04270;mmu04530;mmu04660;mmu04750;mmu04920;mmu04931;mmu05162 | Oligodendrocytes | Prkcq | slateblue2 | 16 | -4.06077348066298 |
  | Ddx58-mRNA | 1.4 | 0.357 | 0.697 | 2.1 | 2.63 | 1.62 | 4.27 | 0.00124 | 0.0324 | lm.nb | Inflammatory Signaling, Innate Immune Response, NF-kB | NS\_MM\_NEUROINFLAM\_V1.0 | Ddx58 | mRNA | 0 |  | NF-kB;Innate Immune Response;Inflammatory Signaling | mmu04064;mmu04622;mmu04623;mmu05160;mmu05161;mmu05162;mmu05164;mmu05168;mmu05169 |  | Ddx58 | slateblue2 | 16 | 3.92156862745098 |
  | Rela-mRNA | 0.505 | 0.157 | 0.197 | 0.812 | 1.42 | 1.15 | 1.76 | 0.00535 | 0.109 | lm.nb | Adaptive Immune Response, Apoptosis, Cellular Stress, Cytokine Signaling, Growth Factor Signaling, Inflammatory Signaling, Innate Immune Response, NF-kB | NS\_MM\_NEUROINFLAM\_V1.0 | Rela | mRNA | 0 |  | NF-kB;Innate Immune Response;Inflammatory Signaling;Growth Factor Signaling;Cytokine Signaling;Cellular Stress;Apoptosis;Adaptive Immune Response | mmu04010;mmu04014;mmu04024;mmu04062;mmu04064;mmu04066;mmu04071;mmu04151;mmu04210;mmu04211;mmu04380;mmu04620;mmu04621;mmu04622;mmu04623;mmu04660;mmu04662;mmu04668;mmu04722;mmu04917;mmu04920;mmu04931;mmu04932;mmu04933;mmu05030;mmu05132;mmu05133;mmu05134;mmu05140;mmu05142;mmu05145;mmu05146;mmu05152;mmu05160;mmu05161;mmu05162;mmu05164;mmu05166;mmu05168;mmu05169;mmu05200;mmu05202;mmu05203;mmu05212;mmu05215;mmu05220;mmu05221;mmu05222;mmu05321 |  | Rela | slateblue4 | 16 | 3.21656050955414 |
  | Blnk-mRNA | 0.63 | 0.197 | 0.244 | 1.02 | 1.55 | 1.18 | 2.02 | 0.00564 | 0.114 | lm.nb | Adaptive Immune Response, Microglia Function, NF-kB | NS\_MM\_NEUROINFLAM\_V1.0 | Blnk | mRNA | 0 |  | NF-kB;Microglia Function;Adaptive Immune Response | mmu04064;mmu04380;mmu04662;mmu05340 |  | Blnk | slateblue4 | 16 | 3.19796954314721 |
  | Il1r1-mRNA | 1.31 | 0.411 | 0.506 | 2.12 | 2.48 | 1.42 | 4.33 | 0.00568 | 0.114 | lm.nb | Apoptosis, Cellular Stress, Cytokine Signaling, Growth Factor Signaling, Microglia Function, NF-kB | NS\_MM\_NEUROINFLAM\_V1.0 | Il1r1 | mRNA | 0 |  | NF-kB;Microglia Function;Growth Factor Signaling;Cytokine Signaling;Cellular Stress;Apoptosis | mmu04010;mmu04060;mmu04064;mmu04210;mmu04380;mmu04640;mmu04750;mmu05146;mmu05166 |  | Il1r1 | slateblue4 | 16 | 3.18734793187348 |
  | Ltbr-mRNA | 0.827 | 0.271 | 0.296 | 1.36 | 1.77 | 1.23 | 2.56 | 0.00758 | 0.146 | lm.nb | Apoptosis, Cytokine Signaling, Inflammatory Signaling, NF-kB | NS\_MM\_NEUROINFLAM\_V1.0 | Ltbr | mRNA | 0 |  | NF-kB;Inflammatory Signaling;Cytokine Signaling;Apoptosis | mmu04060;mmu04064;mmu04066;mmu04672;mmu05166;mmu05203 |  | Ltbr | slateblue4 | 16 | 3.05166051660517 |
  | Nfkb2-mRNA | 1.86 | 0.669 | 0.545 | 3.17 | 3.62 | 1.46 | 8.98 | 0.0135 | 0.236 | lm.nb | Apoptosis, Cytokine Signaling, Growth Factor Signaling, Inflammatory Signaling, Innate Immune Response, NF-kB | NS\_MM\_NEUROINFLAM\_V1.0 | Nfkb2 | mRNA | 0 |  | NF-kB;Innate Immune Response;Inflammatory Signaling;Growth Factor Signaling;Cytokine Signaling;Apoptosis | mmu04010;mmu04064;mmu04380;mmu05134;mmu05166;mmu05169;mmu05200;mmu05203 |  | Nfkb2 | slateblue4 | 16 | 2.78026905829596 |
  | Myd88-mRNA | 0.795 | 0.318 | 0.173 | 1.42 | 1.74 | 1.13 | 2.67 | 0.0235 | 0.361 | lm.nb | Apoptosis, Cytokine Signaling, Growth Factor Signaling, Inflammatory Signaling, Innate Immune Response, NF-kB | NS\_MM\_NEUROINFLAM\_V1.0 | Myd88 | mRNA | 0 |  | NF-kB;Innate Immune Response;Inflammatory Signaling;Growth Factor Signaling;Cytokine Signaling;Apoptosis | mmu04064;mmu04210;mmu04620;mmu05132;mmu05133;mmu05134;mmu05140;mmu05142;mmu05143;mmu05144;mmu05145;mmu05152;mmu05161;mmu05162;mmu05164;mmu05168 |  | Myd88 | slateblue4 | 16 | 2.5 |

  ##### DE Results - NF-kB genes - DPI: 3 vs.CTRL

  Table displaying each sample's global significance scores and directed global significance scores as defined in the heatmaps above. The global significance score is calculated as the square root of the mean squared t-statistic for the genes in a gene set, with t-statistics coming from the linear regression underlying our differential expression analysis. The directed global significance score is calculated as the square root of the mean signed squared t-statistic for the genes in a gene set, with t-statistics coming from the linear regression underlying our differential expression analysis.

DPI:
- DPI: differential expression in 1 vs. baseline of CTRL
- DPI: differential expression in 2 vs. baseline of CTRL
- DPI: differential expression in 3 vs. baseline of CTRL

- **Volcano Plot: DPI: 1 vs.CTRL  
  More Plot Information**


  ##### Volcano Plot: DPI: 1 vs.CTRL

  Volcano plot displaying each gene's -log10(p-value) and log2 fold change for the selected covariate. Highly statistically significant genes fall at the top of the plot, and highly differentially expressed genes fall to either side. Genes within the selected gene set are highlighted in orange. Horizontal lines indicate various False Discovery Rate (FDR) thresholds.
- **DE Results - Notch genes - DPI: 1 vs.CTRL  
  More Plot Information  Download CSV Data**

  | Probe Label | Log2 fold change | std error (log2) | Lower confidence limit (log2) | Upper confidence limit (log2) | Linear fold change | Lower confidence limit (linear) | Upper confidence limit (linear) | P-value | BY.p.value | method | Gene.sets | Codeset.Name | Probe.Label | Analyte.Type | Is.Control | Control.Type | Probe.Annotation | KEGG.Pathways | Cell.Type | Official.Gene.Name | volcanocol | volcanopch | tstats.all |
  | --- | --- | --- | --- | --- | --- | --- | --- | --- | --- | --- | --- | --- | --- | --- | --- | --- | --- | --- | --- | --- | --- | --- | --- |
  | Kat2a-mRNA | -0.787 | 0.21 | -1.2 | -0.375 | 0.579 | 0.436 | 0.771 | 0.00177 | 0.129 | lm.nb | Epigenetic Regulation, Notch | NS\_MM\_NEUROINFLAM\_V1.0 | Kat2a | mRNA | 0 |  | Notch;Epigenetic Regulation | mmu04330;mmu04919;mmu05166;mmu05203 |  | Kat2a | slateblue4 | 16 | -3.74761904761905 |
  | Hdac6-mRNA | -0.503 | 0.18 | -0.856 | -0.151 | 0.705 | 0.552 | 0.901 | 0.0129 | 0.364 | lm.nb | Cellular Stress, Epigenetic Regulation, Notch | NS\_MM\_NEUROINFLAM\_V1.0 | Hdac6 | mRNA | 0 |  | Notch;Epigenetic Regulation;Cellular Stress | mmu05034;mmu05203 |  | Hdac6 | slateblue4 | 16 | -2.79444444444444 |
  | Psen2-mRNA | 0.373 | 0.135 | 0.108 | 0.639 | 1.3 | 1.08 | 1.56 | 0.0139 | 0.377 | loglinear | Growth Factor Signaling, Microglia Function, Notch | NS\_MM\_NEUROINFLAM\_V1.0 | Psen2 | mRNA | 0 |  | Notch;Microglia Function;Growth Factor Signaling | mmu04330;mmu04722;mmu05010 |  | Psen2 | slateblue4 | 16 | 2.76296296296296 |
  | Lfng-mRNA | 0.433 | 0.176 | 0.0887 | 0.777 | 1.35 | 1.06 | 1.71 | 0.0254 | 0.54 | lm.nb | Microglia Function, Notch | NS\_MM\_NEUROINFLAM\_V1.0 | Lfng | mRNA | 0 |  | Notch;Microglia Function | mmu04330 |  | Lfng | azure4 | 1 | 2.46022727272727 |
  | Myc-mRNA | -0.803 | 0.346 | -1.48 | -0.124 | 0.573 | 0.358 | 0.918 | 0.034 | 0.652 | lm.nb | Apoptosis, Cell Cycle, Cytokine Signaling, Growth Factor Signaling, Notch, Wnt | NS\_MM\_NEUROINFLAM\_V1.0 | Myc | mRNA | 0 |  | Wnt;Notch;Growth Factor Signaling;Cytokine Signaling;Cell Cycle;Apoptosis | mmu04010;mmu04012;mmu04110;mmu04151;mmu04310;mmu04350;mmu04390;mmu04550;mmu04630;mmu04919;mmu05161;mmu05166;mmu05169;mmu05200;mmu05202;mmu05205;mmu05206;mmu05210;mmu05213;mmu05216;mmu05219;mmu05220;mmu05221;mmu05222;mmu05230 |  | Myc | azure4 | 1 | -2.32080924855491 |
  | St3gal6-mRNA | 0.428 | 0.194 | 0.0487 | 0.808 | 1.35 | 1.03 | 1.75 | 0.0419 | 0.743 | lm.nb | Microglia Function, Notch | NS\_MM\_NEUROINFLAM\_V1.0 | St3gal6 | mRNA | 0 |  | Notch;Microglia Function |  |  | St3gal6 | azure4 | 1 | 2.20618556701031 |
  | Hif1a-mRNA | -0.239 | 0.117 | -0.469 | -0.00993 | 0.847 | 0.723 | 0.993 | 0.0577 | 0.917 | lm.nb | Autophagy, Cellular Stress, Microglia Function, Notch | NS\_MM\_NEUROINFLAM\_V1.0 | Hif1a | mRNA | 0 |  | Notch;Microglia Function;Cellular Stress;Autophagy | mmu04066;mmu04150;mmu04919;mmu05200;mmu05205;mmu05211;mmu05230;mmu05231 |  | Hif1a | azure4 | 1 | -2.04273504273504 |
  | Ep300-mRNA | -0.209 | 0.111 | -0.425 | 0.008 | 0.865 | 0.745 | 1.01 | 0.0774 | 1 | loglinear | Cell Cycle, Cellular Stress, Cytokine Signaling, DNA Damage, Epigenetic Regulation, Inflammatory Signaling, Innate Immune Response, Lipid Metabolism, Notch, Wnt | NS\_MM\_NEUROINFLAM\_V1.0 | Ep300 | mRNA | 0 |  | Wnt;Notch;Lipid Metabolism;Innate Immune Response;Inflammatory Signaling;Epigenetic Regulation;DNA Damage;Cytokine Signaling;Cellular Stress;Cell Cycle | mmu04024;mmu04066;mmu04068;mmu04110;mmu04310;mmu04330;mmu04350;mmu04520;mmu04630;mmu04720;mmu04916;mmu04919;mmu04922;mmu05016;mmu05152;mmu05161;mmu05164;mmu05166;mmu05168;mmu05169;mmu05200;mmu05203;mmu05206;mmu05211;mmu05215 |  | Ep300 | azure4 | 1 | -1.88288288288288 |
  | Creb1-mRNA | -0.125 | 0.0668 | -0.255 | 0.00632 | 0.917 | 0.838 | 1 | 0.0806 | 1 | loglinear | Adaptive Immune Response, Carbohydrate Metabolism, DNA Damage, Growth Factor Signaling, Innate Immune Response, Neurons and Neurotransmission, Notch | NS\_MM\_NEUROINFLAM\_V1.0 | Creb1 | mRNA | 0 |  | Notch;Neurons and Neurotransmission;Innate Immune Response;Growth Factor Signaling;DNA Damage;Carbohydrate Metabolism;Adaptive Immune Response | mmu04022;mmu04024;mmu04151;mmu04152;mmu04211;mmu04261;mmu04380;mmu04612;mmu04668;mmu04713;mmu04725;mmu04728;mmu04911;mmu04915;mmu04916;mmu04922;mmu04924;mmu04925;mmu04931;mmu05016;mmu05030;mmu05031;mmu05034;mmu05152;mmu05161;mmu05166;mmu05203;mmu05215 |  | Creb1 | azure4 | 1 | -1.87125748502994 |
  | Crebbp-mRNA | -0.0726 | 0.0406 | -0.152 | 0.00705 | 0.951 | 0.9 | 1 | 0.093 | 1 | loglinear | Cell Cycle, Cellular Stress, Cytokine Signaling, Epigenetic Regulation, Inflammatory Signaling, Innate Immune Response, Lipid Metabolism, Notch, Wnt | NS\_MM\_NEUROINFLAM\_V1.0 | Crebbp | mRNA | 0 |  | Wnt;Notch;Lipid Metabolism;Innate Immune Response;Inflammatory Signaling;Epigenetic Regulation;Cytokine Signaling;Cellular Stress;Cell Cycle | mmu04024;mmu04066;mmu04068;mmu04110;mmu04310;mmu04330;mmu04350;mmu04520;mmu04630;mmu04720;mmu04916;mmu04919;mmu04922;mmu05016;mmu05152;mmu05161;mmu05164;mmu05166;mmu05168;mmu05169;mmu05200;mmu05203;mmu05206;mmu05211;mmu05215 |  | Crebbp | azure4 | 1 | -1.78817733990148 |
  | Kat2b-mRNA | 0.176 | 0.131 | -0.08 | 0.432 | 1.13 | 0.946 | 1.35 | 0.197 | 1 | lm.nb | Epigenetic Regulation, Notch | NS\_MM\_NEUROINFLAM\_V1.0 | Kat2b | mRNA | 0 |  | Notch;Epigenetic Regulation | mmu04330;mmu04919;mmu05166;mmu05203 |  | Kat2b | azure4 | 1 | 1.34351145038168 |
  | Trp53-mRNA | 0.257 | 0.2 | -0.134 | 0.649 | 1.2 | 0.911 | 1.57 | 0.216 | 1 | lm.nb | Apoptosis, Cell Cycle, Cellular Stress, DNA Damage, Growth Factor Signaling, Notch, Wnt | NS\_MM\_NEUROINFLAM\_V1.0 | Trp53 | mRNA | 0 |  | Wnt;Notch;Growth Factor Signaling;DNA Damage;Cellular Stress;Cell Cycle;Apoptosis | mmu04010;mmu04071;mmu04110;mmu04115;mmu04151;mmu04210;mmu04211;mmu04310;mmu04722;mmu04919;mmu05014;mmu05016;mmu05160;mmu05161;mmu05162;mmu05166;mmu05168;mmu05169;mmu05200;mmu05202;mmu05203;mmu05205;mmu05206;mmu05210;mmu05212;mmu05213;mmu05214;mmu05215;mmu05216;mmu05218;mmu05219;mmu05220;mmu05222;mmu05223;mmu05230 |  | Trp53 | azure4 | 1 | 1.285 |
  | Hdac1-mRNA | 0.105 | 0.0891 | -0.0698 | 0.279 | 1.08 | 0.953 | 1.21 | 0.257 | 1 | loglinear | Cell Cycle, Epigenetic Regulation, Growth Factor Signaling, Notch, Wnt | NS\_MM\_NEUROINFLAM\_V1.0 | Hdac1 | mRNA | 0 |  | Wnt;Notch;Growth Factor Signaling;Epigenetic Regulation;Cell Cycle | mmu04110;mmu04213;mmu04330;mmu04919;mmu05016;mmu05031;mmu05034;mmu05169;mmu05200;mmu05202;mmu05203;mmu05220 |  | Hdac1 | azure4 | 1 | 1.17845117845118 |
  | Tle3-mRNA | 0.111 | 0.124 | -0.133 | 0.354 | 1.08 | 0.912 | 1.28 | 0.386 | 1 | loglinear | Microglia Function, Notch, Wnt | NS\_MM\_NEUROINFLAM\_V1.0 | Tle3 | mRNA | 0 |  | Wnt;Notch;Microglia Function |  |  | Tle3 | azure4 | 1 | 0.895161290322581 |
  | Ncor1-mRNA | 0.0861 | 0.103 | -0.115 | 0.287 | 1.06 | 0.923 | 1.22 | 0.414 | 1 | loglinear | Epigenetic Regulation, Growth Factor Signaling, Notch | NS\_MM\_NEUROINFLAM\_V1.0 | Ncor1 | mRNA | 0 |  | Notch;Growth Factor Signaling;Epigenetic Regulation | mmu04919;mmu05202 |  | Ncor1 | azure4 | 1 | 0.835922330097087 |
  | E2f1-mRNA | -0.203 | 0.272 | -0.735 | 0.33 | 0.869 | 0.601 | 1.26 | 0.466 | 1 | lm.nb | Apoptosis, Cell Cycle, Cellular Stress, Notch | NS\_MM\_NEUROINFLAM\_V1.0 | E2f1 | mRNA | 0 |  | Notch;Cellular Stress;Cell Cycle;Apoptosis | mmu04110;mmu05161;mmu05166;mmu05200;mmu05206;mmu05212;mmu05214;mmu05215;mmu05218;mmu05219;mmu05220;mmu05222;mmu05223 |  | E2f1 | azure4 | 1 | -0.746323529411765 |
  | Ago4-mRNA | 0.231 | 0.368 | -0.489 | 0.952 | 1.17 | 0.712 | 1.93 | 0.538 | 1 | lm.nb | Adaptive Immune Response, Cellular Stress, Epigenetic Regulation, Growth Factor Signaling, Notch, Wnt | NS\_MM\_NEUROINFLAM\_V1.0 | Ago4 | mRNA | 0 |  | Wnt;Notch;Growth Factor Signaling;Epigenetic Regulation;Cellular Stress;Adaptive Immune Response |  |  | Ago4 | azure4 | 1 | 0.627717391304348 |
  | Jun-mRNA | 0.066 | 0.12 | -0.17 | 0.302 | 1.05 | 0.889 | 1.23 | 0.591 | 1 | lm.nb | Adaptive Immune Response, Apoptosis, Cellular Stress, Growth Factor Signaling, Innate Immune Response, Notch, Wnt | NS\_MM\_NEUROINFLAM\_V1.0 | Jun | mRNA | 0 |  | Wnt;Notch;Innate Immune Response;Growth Factor Signaling;Cellular Stress;Apoptosis;Adaptive Immune Response | mmu04010;mmu04012;mmu04024;mmu04310;mmu04380;mmu04510;mmu04620;mmu04660;mmu04662;mmu04668;mmu04722;mmu04912;mmu04915;mmu04921;mmu04932;mmu04933;mmu05030;mmu05031;mmu05132;mmu05133;mmu05140;mmu05142;mmu05161;mmu05164;mmu05166;mmu05168;mmu05169;mmu05200;mmu05203;mmu05210;mmu05211;mmu05231;mmu05321;mmu05323 |  | Jun | azure4 | 1 | 0.55 |
  | Hdac2-mRNA | 0.0235 | 0.057 | -0.0884 | 0.135 | 1.02 | 0.941 | 1.1 | 0.686 | 1 | loglinear | Cell Cycle, Cellular Stress, Epigenetic Regulation, Growth Factor Signaling, Notch | NS\_MM\_NEUROINFLAM\_V1.0 | Hdac2 | mRNA | 0 |  | Notch;Growth Factor Signaling;Epigenetic Regulation;Cellular Stress;Cell Cycle | mmu04110;mmu04213;mmu04330;mmu04919;mmu05016;mmu05034;mmu05169;mmu05200;mmu05202;mmu05203;mmu05220 |  | Hdac2 | azure4 | 1 | 0.412280701754386 |
  | Ncor2-mRNA | -0.0143 | 0.0819 | -0.175 | 0.146 | 0.99 | 0.886 | 1.11 | 0.864 | 1 | loglinear | Epigenetic Regulation, Notch | NS\_MM\_NEUROINFLAM\_V1.0 | Ncor2 | mRNA | 0 |  | Notch;Epigenetic Regulation | mmu04330;mmu05169 |  | Ncor2 | azure4 | 1 | -0.174603174603175 |

  ##### DE Results - Notch genes - DPI: 1 vs.CTRL

  Table displaying each sample's global significance scores and directed global significance scores as defined in the heatmaps above. The global significance score is calculated as the square root of the mean squared t-statistic for the genes in a gene set, with t-statistics coming from the linear regression underlying our differential expression analysis. The directed global significance score is calculated as the square root of the mean signed squared t-statistic for the genes in a gene set, with t-statistics coming from the linear regression underlying our differential expression analysis.

- **Volcano Plot: DPI: 2 vs.CTRL  
  More Plot Information**


  ##### Volcano Plot: DPI: 2 vs.CTRL

  Volcano plot displaying each gene's -log10(p-value) and log2 fold change for the selected covariate. Highly statistically significant genes fall at the top of the plot, and highly differentially expressed genes fall to either side. Genes within the selected gene set are highlighted in orange. Horizontal lines indicate various False Discovery Rate (FDR) thresholds.
- **DE Results - Notch genes - DPI: 2 vs.CTRL  
  More Plot Information  Download CSV Data**

  | Probe Label | Log2 fold change | std error (log2) | Lower confidence limit (log2) | Upper confidence limit (log2) | Linear fold change | Lower confidence limit (linear) | Upper confidence limit (linear) | P-value | BY.p.value | method | Gene.sets | Codeset.Name | Probe.Label | Analyte.Type | Is.Control | Control.Type | Probe.Annotation | KEGG.Pathways | Cell.Type | Official.Gene.Name | volcanocol | volcanopch | tstats.all |
  | --- | --- | --- | --- | --- | --- | --- | --- | --- | --- | --- | --- | --- | --- | --- | --- | --- | --- | --- | --- | --- | --- | --- | --- |
  | Lfng-mRNA | 1.68 | 0.166 | 1.36 | 2.01 | 3.21 | 2.56 | 4.02 | 2.36e-08 | 1.04e-05 | lm.nb | Microglia Function, Notch | NS\_MM\_NEUROINFLAM\_V1.0 | Lfng | mRNA | 0 |  | Notch;Microglia Function | mmu04330 |  | Lfng | slateblue1 | 16 | 10.1204819277108 |
  | Tle3-mRNA | 0.592 | 0.124 | 0.349 | 0.836 | 1.51 | 1.27 | 1.78 | 0.000207 | 0.00902 | loglinear | Microglia Function, Notch, Wnt | NS\_MM\_NEUROINFLAM\_V1.0 | Tle3 | mRNA | 0 |  | Wnt;Notch;Microglia Function |  |  | Tle3 | slateblue1 | 16 | 4.7741935483871 |
  | Kat2a-mRNA | -0.885 | 0.211 | -1.3 | -0.472 | 0.542 | 0.407 | 0.721 | 0.000674 | 0.0218 | lm.nb | Epigenetic Regulation, Notch | NS\_MM\_NEUROINFLAM\_V1.0 | Kat2a | mRNA | 0 |  | Notch;Epigenetic Regulation | mmu04330;mmu04919;mmu05166;mmu05203 |  | Kat2a | slateblue2 | 16 | -4.19431279620853 |
  | Hdac6-mRNA | -0.68 | 0.181 | -1.04 | -0.325 | 0.624 | 0.488 | 0.799 | 0.00175 | 0.0406 | lm.nb | Cellular Stress, Epigenetic Regulation, Notch | NS\_MM\_NEUROINFLAM\_V1.0 | Hdac6 | mRNA | 0 |  | Notch;Epigenetic Regulation;Cellular Stress | mmu05034;mmu05203 |  | Hdac6 | slateblue2 | 16 | -3.75690607734807 |
  | Hif1a-mRNA | -0.425 | 0.117 | -0.655 | -0.196 | 0.745 | 0.635 | 0.873 | 0.00226 | 0.0495 | lm.nb | Autophagy, Cellular Stress, Microglia Function, Notch | NS\_MM\_NEUROINFLAM\_V1.0 | Hif1a | mRNA | 0 |  | Notch;Microglia Function;Cellular Stress;Autophagy | mmu04066;mmu04150;mmu04919;mmu05200;mmu05205;mmu05211;mmu05230;mmu05231 |  | Hif1a | slateblue3 | 16 | -3.63247863247863 |
  | Hdac2-mRNA | -0.184 | 0.057 | -0.295 | -0.0718 | 0.881 | 0.815 | 0.951 | 0.00537 | 0.1 | loglinear | Cell Cycle, Cellular Stress, Epigenetic Regulation, Growth Factor Signaling, Notch | NS\_MM\_NEUROINFLAM\_V1.0 | Hdac2 | mRNA | 0 |  | Notch;Growth Factor Signaling;Epigenetic Regulation;Cellular Stress;Cell Cycle | mmu04110;mmu04213;mmu04330;mmu04919;mmu05016;mmu05034;mmu05169;mmu05200;mmu05202;mmu05203;mmu05220 |  | Hdac2 | slateblue4 | 16 | -3.2280701754386 |
  | Ep300-mRNA | -0.33 | 0.111 | -0.547 | -0.114 | 0.795 | 0.684 | 0.924 | 0.0087 | 0.144 | loglinear | Cell Cycle, Cellular Stress, Cytokine Signaling, DNA Damage, Epigenetic Regulation, Inflammatory Signaling, Innate Immune Response, Lipid Metabolism, Notch, Wnt | NS\_MM\_NEUROINFLAM\_V1.0 | Ep300 | mRNA | 0 |  | Wnt;Notch;Lipid Metabolism;Innate Immune Response;Inflammatory Signaling;Epigenetic Regulation;DNA Damage;Cytokine Signaling;Cellular Stress;Cell Cycle | mmu04024;mmu04066;mmu04068;mmu04110;mmu04310;mmu04330;mmu04350;mmu04520;mmu04630;mmu04720;mmu04916;mmu04919;mmu04922;mmu05016;mmu05152;mmu05161;mmu05164;mmu05166;mmu05168;mmu05169;mmu05200;mmu05203;mmu05206;mmu05211;mmu05215 |  | Ep300 | slateblue4 | 16 | -2.97297297297297 |
  | Psen2-mRNA | 0.381 | 0.135 | 0.116 | 0.646 | 1.3 | 1.08 | 1.56 | 0.0124 | 0.194 | loglinear | Growth Factor Signaling, Microglia Function, Notch | NS\_MM\_NEUROINFLAM\_V1.0 | Psen2 | mRNA | 0 |  | Notch;Microglia Function;Growth Factor Signaling | mmu04330;mmu04722;mmu05010 |  | Psen2 | slateblue4 | 16 | 2.82222222222222 |
  | Ago4-mRNA | -0.969 | 0.4 | -1.75 | -0.185 | 0.511 | 0.297 | 0.88 | 0.0277 | 0.356 | lm.nb | Adaptive Immune Response, Cellular Stress, Epigenetic Regulation, Growth Factor Signaling, Notch, Wnt | NS\_MM\_NEUROINFLAM\_V1.0 | Ago4 | mRNA | 0 |  | Wnt;Notch;Growth Factor Signaling;Epigenetic Regulation;Cellular Stress;Adaptive Immune Response |  |  | Ago4 | slateblue4 | 16 | -2.4225 |
  | St3gal6-mRNA | 0.42 | 0.194 | 0.0405 | 0.8 | 1.34 | 1.03 | 1.74 | 0.0455 | 0.55 | lm.nb | Microglia Function, Notch | NS\_MM\_NEUROINFLAM\_V1.0 | St3gal6 | mRNA | 0 |  | Notch;Microglia Function |  |  | St3gal6 | azure4 | 1 | 2.16494845360825 |
  | Kat2b-mRNA | 0.264 | 0.13 | 0.00945 | 0.519 | 1.2 | 1.01 | 1.43 | 0.059 | 0.676 | lm.nb | Epigenetic Regulation, Notch | NS\_MM\_NEUROINFLAM\_V1.0 | Kat2b | mRNA | 0 |  | Notch;Epigenetic Regulation | mmu04330;mmu04919;mmu05166;mmu05203 |  | Kat2b | azure4 | 1 | 2.03076923076923 |
  | Jun-mRNA | 0.242 | 0.12 | 0.0077 | 0.477 | 1.18 | 1.01 | 1.39 | 0.06 | 0.68 | lm.nb | Adaptive Immune Response, Apoptosis, Cellular Stress, Growth Factor Signaling, Innate Immune Response, Notch, Wnt | NS\_MM\_NEUROINFLAM\_V1.0 | Jun | mRNA | 0 |  | Wnt;Notch;Innate Immune Response;Growth Factor Signaling;Cellular Stress;Apoptosis;Adaptive Immune Response | mmu04010;mmu04012;mmu04024;mmu04310;mmu04380;mmu04510;mmu04620;mmu04660;mmu04662;mmu04668;mmu04722;mmu04912;mmu04915;mmu04921;mmu04932;mmu04933;mmu05030;mmu05031;mmu05132;mmu05133;mmu05140;mmu05142;mmu05161;mmu05164;mmu05166;mmu05168;mmu05169;mmu05200;mmu05203;mmu05210;mmu05211;mmu05231;mmu05321;mmu05323 |  | Jun | azure4 | 1 | 2.01666666666667 |
  | Trp53-mRNA | 0.368 | 0.198 | -0.0207 | 0.756 | 1.29 | 0.986 | 1.69 | 0.082 | 0.877 | lm.nb | Apoptosis, Cell Cycle, Cellular Stress, DNA Damage, Growth Factor Signaling, Notch, Wnt | NS\_MM\_NEUROINFLAM\_V1.0 | Trp53 | mRNA | 0 |  | Wnt;Notch;Growth Factor Signaling;DNA Damage;Cellular Stress;Cell Cycle;Apoptosis | mmu04010;mmu04071;mmu04110;mmu04115;mmu04151;mmu04210;mmu04211;mmu04310;mmu04722;mmu04919;mmu05014;mmu05016;mmu05160;mmu05161;mmu05162;mmu05166;mmu05168;mmu05169;mmu05200;mmu05202;mmu05203;mmu05205;mmu05206;mmu05210;mmu05212;mmu05213;mmu05214;mmu05215;mmu05216;mmu05218;mmu05219;mmu05220;mmu05222;mmu05223;mmu05230 |  | Trp53 | azure4 | 1 | 1.85858585858586 |
  | Creb1-mRNA | -0.0939 | 0.0668 | -0.225 | 0.037 | 0.937 | 0.856 | 1.03 | 0.179 | 1 | loglinear | Adaptive Immune Response, Carbohydrate Metabolism, DNA Damage, Growth Factor Signaling, Innate Immune Response, Neurons and Neurotransmission, Notch | NS\_MM\_NEUROINFLAM\_V1.0 | Creb1 | mRNA | 0 |  | Notch;Neurons and Neurotransmission;Innate Immune Response;Growth Factor Signaling;DNA Damage;Carbohydrate Metabolism;Adaptive Immune Response | mmu04022;mmu04024;mmu04151;mmu04152;mmu04211;mmu04261;mmu04380;mmu04612;mmu04668;mmu04713;mmu04725;mmu04728;mmu04911;mmu04915;mmu04916;mmu04922;mmu04924;mmu04925;mmu04931;mmu05016;mmu05030;mmu05031;mmu05034;mmu05152;mmu05161;mmu05166;mmu05203;mmu05215 |  | Creb1 | azure4 | 1 | -1.40568862275449 |
  | Hdac4-mRNA | 0.0826 | 0.074 | -0.0624 | 0.228 | 1.06 | 0.958 | 1.17 | 0.28 | 1 | loglinear | Epigenetic Regulation, Notch | NS\_MM\_NEUROINFLAM\_V1.0 | Hdac4 | mRNA | 0 |  | Notch;Epigenetic Regulation | mmu05034;mmu05169;mmu05203 |  | Hdac4 | azure4 | 1 | 1.11621621621622 |
  | Myc-mRNA | -0.311 | 0.34 | -0.977 | 0.355 | 0.806 | 0.508 | 1.28 | 0.373 | 1 | lm.nb | Apoptosis, Cell Cycle, Cytokine Signaling, Growth Factor Signaling, Notch, Wnt | NS\_MM\_NEUROINFLAM\_V1.0 | Myc | mRNA | 0 |  | Wnt;Notch;Growth Factor Signaling;Cytokine Signaling;Cell Cycle;Apoptosis | mmu04010;mmu04012;mmu04110;mmu04151;mmu04310;mmu04350;mmu04390;mmu04550;mmu04630;mmu04919;mmu05161;mmu05166;mmu05169;mmu05200;mmu05202;mmu05205;mmu05206;mmu05210;mmu05213;mmu05216;mmu05219;mmu05220;mmu05221;mmu05222;mmu05230 |  | Myc | azure4 | 1 | -0.914705882352941 |
  | Crebbp-mRNA | -0.0334 | 0.0406 | -0.113 | 0.0462 | 0.977 | 0.925 | 1.03 | 0.423 | 1 | loglinear | Cell Cycle, Cellular Stress, Cytokine Signaling, Epigenetic Regulation, Inflammatory Signaling, Innate Immune Response, Lipid Metabolism, Notch, Wnt | NS\_MM\_NEUROINFLAM\_V1.0 | Crebbp | mRNA | 0 |  | Wnt;Notch;Lipid Metabolism;Innate Immune Response;Inflammatory Signaling;Epigenetic Regulation;Cytokine Signaling;Cellular Stress;Cell Cycle | mmu04024;mmu04066;mmu04068;mmu04110;mmu04310;mmu04330;mmu04350;mmu04520;mmu04630;mmu04720;mmu04916;mmu04919;mmu04922;mmu05016;mmu05152;mmu05161;mmu05164;mmu05166;mmu05168;mmu05169;mmu05200;mmu05203;mmu05206;mmu05211;mmu05215 |  | Crebbp | azure4 | 1 | -0.822660098522167 |
  | Ncor2-mRNA | 0.0496 | 0.0819 | -0.111 | 0.21 | 1.03 | 0.926 | 1.16 | 0.554 | 1 | loglinear | Epigenetic Regulation, Notch | NS\_MM\_NEUROINFLAM\_V1.0 | Ncor2 | mRNA | 0 |  | Notch;Epigenetic Regulation | mmu04330;mmu05169 |  | Ncor2 | azure4 | 1 | 0.605616605616606 |
  | Hdac1-mRNA | 0.0347 | 0.0891 | -0.14 | 0.209 | 1.02 | 0.908 | 1.16 | 0.702 | 1 | loglinear | Cell Cycle, Epigenetic Regulation, Growth Factor Signaling, Notch, Wnt | NS\_MM\_NEUROINFLAM\_V1.0 | Hdac1 | mRNA | 0 |  | Wnt;Notch;Growth Factor Signaling;Epigenetic Regulation;Cell Cycle | mmu04110;mmu04213;mmu04330;mmu04919;mmu05016;mmu05031;mmu05034;mmu05169;mmu05200;mmu05202;mmu05203;mmu05220 |  | Hdac1 | azure4 | 1 | 0.389450056116723 |
  | E2f1-mRNA | 0.0556 | 0.268 | -0.47 | 0.582 | 1.04 | 0.722 | 1.5 | 0.838 | 1 | lm.nb | Apoptosis, Cell Cycle, Cellular Stress, Notch | NS\_MM\_NEUROINFLAM\_V1.0 | E2f1 | mRNA | 0 |  | Notch;Cellular Stress;Cell Cycle;Apoptosis | mmu04110;mmu05161;mmu05166;mmu05200;mmu05206;mmu05212;mmu05214;mmu05215;mmu05218;mmu05219;mmu05220;mmu05222;mmu05223 |  | E2f1 | azure4 | 1 | 0.207462686567164 |

  ##### DE Results - Notch genes - DPI: 2 vs.CTRL

  Table displaying each sample's global significance scores and directed global significance scores as defined in the heatmaps above. The global significance score is calculated as the square root of the mean squared t-statistic for the genes in a gene set, with t-statistics coming from the linear regression underlying our differential expression analysis. The directed global significance score is calculated as the square root of the mean signed squared t-statistic for the genes in a gene set, with t-statistics coming from the linear regression underlying our differential expression analysis.

- **Volcano Plot: DPI: 3 vs.CTRL  
  More Plot Information**


  ##### Volcano Plot: DPI: 3 vs.CTRL

  Volcano plot displaying each gene's -log10(p-value) and log2 fold change for the selected covariate. Highly statistically significant genes fall at the top of the plot, and highly differentially expressed genes fall to either side. Genes within the selected gene set are highlighted in orange. Horizontal lines indicate various False Discovery Rate (FDR) thresholds.
- **DE Results - Notch genes - DPI: 3 vs.CTRL  
  More Plot Information  Download CSV Data**

  | Probe Label | Log2 fold change | std error (log2) | Lower confidence limit (log2) | Upper confidence limit (log2) | Linear fold change | Lower confidence limit (linear) | Upper confidence limit (linear) | P-value | BY.p.value | method | Gene.sets | Codeset.Name | Probe.Label | Analyte.Type | Is.Control | Control.Type | Probe.Annotation | KEGG.Pathways | Cell.Type | Official.Gene.Name | volcanocol | volcanopch | tstats.all |
  | --- | --- | --- | --- | --- | --- | --- | --- | --- | --- | --- | --- | --- | --- | --- | --- | --- | --- | --- | --- | --- | --- | --- | --- |
  | Hdac2-mRNA | -0.452 | 0.057 | -0.564 | -0.341 | 0.731 | 0.676 | 0.79 | 6.19e-07 | 7.29e-05 | loglinear | Cell Cycle, Cellular Stress, Epigenetic Regulation, Growth Factor Signaling, Notch | NS\_MM\_NEUROINFLAM\_V1.0 | Hdac2 | mRNA | 0 |  | Notch;Growth Factor Signaling;Epigenetic Regulation;Cellular Stress;Cell Cycle | mmu04110;mmu04213;mmu04330;mmu04919;mmu05016;mmu05034;mmu05169;mmu05200;mmu05202;mmu05203;mmu05220 |  | Hdac2 | slateblue1 | 16 | -7.92982456140351 |
  | Lfng-mRNA | 1.31 | 0.168 | 0.975 | 1.64 | 2.47 | 1.97 | 3.11 | 8.33e-07 | 9.43e-05 | lm.nb | Microglia Function, Notch | NS\_MM\_NEUROINFLAM\_V1.0 | Lfng | mRNA | 0 |  | Notch;Microglia Function | mmu04330 |  | Lfng | slateblue1 | 16 | 7.79761904761905 |
  | Tle3-mRNA | 0.865 | 0.124 | 0.622 | 1.11 | 1.82 | 1.54 | 2.16 | 3.15e-06 | 0.000271 | loglinear | Microglia Function, Notch, Wnt | NS\_MM\_NEUROINFLAM\_V1.0 | Tle3 | mRNA | 0 |  | Wnt;Notch;Microglia Function |  |  | Tle3 | slateblue1 | 16 | 6.9758064516129 |
  | Creb1-mRNA | -0.443 | 0.0668 | -0.574 | -0.312 | 0.735 | 0.672 | 0.805 | 5.69e-06 | 0.000418 | loglinear | Adaptive Immune Response, Carbohydrate Metabolism, DNA Damage, Growth Factor Signaling, Innate Immune Response, Neurons and Neurotransmission, Notch | NS\_MM\_NEUROINFLAM\_V1.0 | Creb1 | mRNA | 0 |  | Notch;Neurons and Neurotransmission;Innate Immune Response;Growth Factor Signaling;DNA Damage;Carbohydrate Metabolism;Adaptive Immune Response | mmu04022;mmu04024;mmu04151;mmu04152;mmu04211;mmu04261;mmu04380;mmu04612;mmu04668;mmu04713;mmu04725;mmu04728;mmu04911;mmu04915;mmu04916;mmu04922;mmu04924;mmu04925;mmu04931;mmu05016;mmu05030;mmu05031;mmu05034;mmu05152;mmu05161;mmu05166;mmu05203;mmu05215 |  | Creb1 | slateblue1 | 16 | -6.63173652694611 |
  | Ago4-mRNA | -2.72 | 0.514 | -3.72 | -1.71 | 0.152 | 0.0757 | 0.306 | 7.46e-05 | 0.00321 | lm.nb | Adaptive Immune Response, Cellular Stress, Epigenetic Regulation, Growth Factor Signaling, Notch, Wnt | NS\_MM\_NEUROINFLAM\_V1.0 | Ago4 | mRNA | 0 |  | Wnt;Notch;Growth Factor Signaling;Epigenetic Regulation;Cellular Stress;Adaptive Immune Response |  |  | Ago4 | slateblue1 | 16 | -5.29182879377432 |
  | Kat2b-mRNA | 0.331 | 0.13 | 0.0772 | 0.585 | 1.26 | 1.05 | 1.5 | 0.0212 | 0.33 | lm.nb | Epigenetic Regulation, Notch | NS\_MM\_NEUROINFLAM\_V1.0 | Kat2b | mRNA | 0 |  | Notch;Epigenetic Regulation | mmu04330;mmu04919;mmu05166;mmu05203 |  | Kat2b | slateblue4 | 16 | 2.54615384615385 |
  | Hdac4-mRNA | -0.176 | 0.074 | -0.321 | -0.0309 | 0.885 | 0.801 | 0.979 | 0.0303 | 0.441 | loglinear | Epigenetic Regulation, Notch | NS\_MM\_NEUROINFLAM\_V1.0 | Hdac4 | mRNA | 0 |  | Notch;Epigenetic Regulation | mmu05034;mmu05169;mmu05203 |  | Hdac4 | slateblue4 | 16 | -2.37837837837838 |
  | Psen2-mRNA | 0.294 | 0.135 | 0.0292 | 0.559 | 1.23 | 1.02 | 1.47 | 0.0449 | 0.593 | loglinear | Growth Factor Signaling, Microglia Function, Notch | NS\_MM\_NEUROINFLAM\_V1.0 | Psen2 | mRNA | 0 |  | Notch;Microglia Function;Growth Factor Signaling | mmu04330;mmu04722;mmu05010 |  | Psen2 | azure4 | 1 | 2.17777777777778 |
  | Kat2a-mRNA | -0.437 | 0.209 | -0.847 | -0.0273 | 0.738 | 0.556 | 0.981 | 0.0529 | 0.679 | lm.nb | Epigenetic Regulation, Notch | NS\_MM\_NEUROINFLAM\_V1.0 | Kat2a | mRNA | 0 |  | Notch;Epigenetic Regulation | mmu04330;mmu04919;mmu05166;mmu05203 |  | Kat2a | azure4 | 1 | -2.09090909090909 |
  | Myc-mRNA | 0.677 | 0.331 | 0.0278 | 1.33 | 1.6 | 1.02 | 2.51 | 0.0578 | 0.734 | lm.nb | Apoptosis, Cell Cycle, Cytokine Signaling, Growth Factor Signaling, Notch, Wnt | NS\_MM\_NEUROINFLAM\_V1.0 | Myc | mRNA | 0 |  | Wnt;Notch;Growth Factor Signaling;Cytokine Signaling;Cell Cycle;Apoptosis | mmu04010;mmu04012;mmu04110;mmu04151;mmu04310;mmu04350;mmu04390;mmu04550;mmu04630;mmu04919;mmu05161;mmu05166;mmu05169;mmu05200;mmu05202;mmu05205;mmu05206;mmu05210;mmu05213;mmu05216;mmu05219;mmu05220;mmu05221;mmu05222;mmu05230 |  | Myc | azure4 | 1 | 2.04531722054381 |
  | Jun-mRNA | -0.217 | 0.121 | -0.455 | 0.0202 | 0.86 | 0.73 | 1.01 | 0.0918 | 1 | lm.nb | Adaptive Immune Response, Apoptosis, Cellular Stress, Growth Factor Signaling, Innate Immune Response, Notch, Wnt | NS\_MM\_NEUROINFLAM\_V1.0 | Jun | mRNA | 0 |  | Wnt;Notch;Innate Immune Response;Growth Factor Signaling;Cellular Stress;Apoptosis;Adaptive Immune Response | mmu04010;mmu04012;mmu04024;mmu04310;mmu04380;mmu04510;mmu04620;mmu04660;mmu04662;mmu04668;mmu04722;mmu04912;mmu04915;mmu04921;mmu04932;mmu04933;mmu05030;mmu05031;mmu05132;mmu05133;mmu05140;mmu05142;mmu05161;mmu05164;mmu05166;mmu05168;mmu05169;mmu05200;mmu05203;mmu05210;mmu05211;mmu05231;mmu05321;mmu05323 |  | Jun | azure4 | 1 | -1.79338842975207 |
  | Hdac1-mRNA | -0.128 | 0.0891 | -0.303 | 0.0462 | 0.915 | 0.811 | 1.03 | 0.169 | 1 | loglinear | Cell Cycle, Epigenetic Regulation, Growth Factor Signaling, Notch, Wnt | NS\_MM\_NEUROINFLAM\_V1.0 | Hdac1 | mRNA | 0 |  | Wnt;Notch;Growth Factor Signaling;Epigenetic Regulation;Cell Cycle | mmu04110;mmu04213;mmu04330;mmu04919;mmu05016;mmu05031;mmu05034;mmu05169;mmu05200;mmu05202;mmu05203;mmu05220 |  | Hdac1 | azure4 | 1 | -1.43658810325477 |
  | Hdac6-mRNA | -0.176 | 0.178 | -0.524 | 0.172 | 0.885 | 0.695 | 1.13 | 0.336 | 1 | lm.nb | Cellular Stress, Epigenetic Regulation, Notch | NS\_MM\_NEUROINFLAM\_V1.0 | Hdac6 | mRNA | 0 |  | Notch;Epigenetic Regulation;Cellular Stress | mmu05034;mmu05203 |  | Hdac6 | azure4 | 1 | -0.98876404494382 |
  | Trp53-mRNA | 0.168 | 0.201 | -0.226 | 0.563 | 1.12 | 0.855 | 1.48 | 0.415 | 1 | lm.nb | Apoptosis, Cell Cycle, Cellular Stress, DNA Damage, Growth Factor Signaling, Notch, Wnt | NS\_MM\_NEUROINFLAM\_V1.0 | Trp53 | mRNA | 0 |  | Wnt;Notch;Growth Factor Signaling;DNA Damage;Cellular Stress;Cell Cycle;Apoptosis | mmu04010;mmu04071;mmu04110;mmu04115;mmu04151;mmu04210;mmu04211;mmu04310;mmu04722;mmu04919;mmu05014;mmu05016;mmu05160;mmu05161;mmu05162;mmu05166;mmu05168;mmu05169;mmu05200;mmu05202;mmu05203;mmu05205;mmu05206;mmu05210;mmu05212;mmu05213;mmu05214;mmu05215;mmu05216;mmu05218;mmu05219;mmu05220;mmu05222;mmu05223;mmu05230 |  | Trp53 | azure4 | 1 | 0.835820895522388 |
  | Ncor2-mRNA | 0.06 | 0.0819 | -0.1 | 0.22 | 1.04 | 0.933 | 1.17 | 0.474 | 1 | loglinear | Epigenetic Regulation, Notch | NS\_MM\_NEUROINFLAM\_V1.0 | Ncor2 | mRNA | 0 |  | Notch;Epigenetic Regulation | mmu04330;mmu05169 |  | Ncor2 | azure4 | 1 | 0.732600732600733 |
  | St3gal6-mRNA | 0.141 | 0.2 | -0.251 | 0.533 | 1.1 | 0.841 | 1.45 | 0.49 | 1 | lm.nb | Microglia Function, Notch | NS\_MM\_NEUROINFLAM\_V1.0 | St3gal6 | mRNA | 0 |  | Notch;Microglia Function |  |  | St3gal6 | azure4 | 1 | 0.705 |
  | E2f1-mRNA | -0.104 | 0.27 | -0.634 | 0.425 | 0.93 | 0.644 | 1.34 | 0.704 | 1 | lm.nb | Apoptosis, Cell Cycle, Cellular Stress, Notch | NS\_MM\_NEUROINFLAM\_V1.0 | E2f1 | mRNA | 0 |  | Notch;Cellular Stress;Cell Cycle;Apoptosis | mmu04110;mmu05161;mmu05166;mmu05200;mmu05206;mmu05212;mmu05214;mmu05215;mmu05218;mmu05219;mmu05220;mmu05222;mmu05223 |  | E2f1 | azure4 | 1 | -0.385185185185185 |
  | Ep300-mRNA | -0.0339 | 0.111 | -0.251 | 0.183 | 0.977 | 0.841 | 1.14 | 0.763 | 1 | loglinear | Cell Cycle, Cellular Stress, Cytokine Signaling, DNA Damage, Epigenetic Regulation, Inflammatory Signaling, Innate Immune Response, Lipid Metabolism, Notch, Wnt | NS\_MM\_NEUROINFLAM\_V1.0 | Ep300 | mRNA | 0 |  | Wnt;Notch;Lipid Metabolism;Innate Immune Response;Inflammatory Signaling;Epigenetic Regulation;DNA Damage;Cytokine Signaling;Cellular Stress;Cell Cycle | mmu04024;mmu04066;mmu04068;mmu04110;mmu04310;mmu04330;mmu04350;mmu04520;mmu04630;mmu04720;mmu04916;mmu04919;mmu04922;mmu05016;mmu05152;mmu05161;mmu05164;mmu05166;mmu05168;mmu05169;mmu05200;mmu05203;mmu05206;mmu05211;mmu05215 |  | Ep300 | azure4 | 1 | -0.305405405405405 |
  | Crebbp-mRNA | 0.00879 | 0.0406 | -0.0708 | 0.0884 | 1.01 | 0.952 | 1.06 | 0.831 | 1 | loglinear | Cell Cycle, Cellular Stress, Cytokine Signaling, Epigenetic Regulation, Inflammatory Signaling, Innate Immune Response, Lipid Metabolism, Notch, Wnt | NS\_MM\_NEUROINFLAM\_V1.0 | Crebbp | mRNA | 0 |  | Wnt;Notch;Lipid Metabolism;Innate Immune Response;Inflammatory Signaling;Epigenetic Regulation;Cytokine Signaling;Cellular Stress;Cell Cycle | mmu04024;mmu04066;mmu04068;mmu04110;mmu04310;mmu04330;mmu04350;mmu04520;mmu04630;mmu04720;mmu04916;mmu04919;mmu04922;mmu05016;mmu05152;mmu05161;mmu05164;mmu05166;mmu05168;mmu05169;mmu05200;mmu05203;mmu05206;mmu05211;mmu05215 |  | Crebbp | azure4 | 1 | 0.216502463054187 |
  | Hif1a-mRNA | 0.014 | 0.117 | -0.215 | 0.243 | 1.01 | 0.862 | 1.18 | 0.906 | 1 | lm.nb | Autophagy, Cellular Stress, Microglia Function, Notch | NS\_MM\_NEUROINFLAM\_V1.0 | Hif1a | mRNA | 0 |  | Notch;Microglia Function;Cellular Stress;Autophagy | mmu04066;mmu04150;mmu04919;mmu05200;mmu05205;mmu05211;mmu05230;mmu05231 |  | Hif1a | azure4 | 1 | 0.11965811965812 |

  ##### DE Results - Notch genes - DPI: 3 vs.CTRL

  Table displaying each sample's global significance scores and directed global significance scores as defined in the heatmaps above. The global significance score is calculated as the square root of the mean squared t-statistic for the genes in a gene set, with t-statistics coming from the linear regression underlying our differential expression analysis. The directed global significance score is calculated as the square root of the mean signed squared t-statistic for the genes in a gene set, with t-statistics coming from the linear regression underlying our differential expression analysis.

DPI:
- DPI: differential expression in 1 vs. baseline of CTRL
- DPI: differential expression in 2 vs. baseline of CTRL
- DPI: differential expression in 3 vs. baseline of CTRL

- **Volcano Plot: DPI: 1 vs.CTRL  
  More Plot Information**


  ##### Volcano Plot: DPI: 1 vs.CTRL

  Volcano plot displaying each gene's -log10(p-value) and log2 fold change for the selected covariate. Highly statistically significant genes fall at the top of the plot, and highly differentially expressed genes fall to either side. Genes within the selected gene set are highlighted in orange. Horizontal lines indicate various False Discovery Rate (FDR) thresholds.
- **DE Results - Oligodendrocyte Function genes - DPI: 1 vs.CTRL  
  More Plot Information  Download CSV Data**

  | Probe Label | Log2 fold change | std error (log2) | Lower confidence limit (log2) | Upper confidence limit (log2) | Linear fold change | Lower confidence limit (linear) | Upper confidence limit (linear) | P-value | BY.p.value | method | Gene.sets | Codeset.Name | Probe.Label | Analyte.Type | Is.Control | Control.Type | Probe.Annotation | KEGG.Pathways | Cell.Type | Official.Gene.Name | volcanocol | volcanopch | tstats.all |
  | --- | --- | --- | --- | --- | --- | --- | --- | --- | --- | --- | --- | --- | --- | --- | --- | --- | --- | --- | --- | --- | --- | --- | --- |
  | Mog-mRNA | 0.437 | 0.119 | 0.204 | 0.67 | 1.35 | 1.15 | 1.59 | 0.00205 | 0.136 | lm.nb | Oligodendrocyte Function | NS\_MM\_NEUROINFLAM\_V1.0 | Mog | mRNA | 0 |  | Oligodendrocyte Function |  |  | Mog | slateblue4 | 16 | 3.67226890756303 |
  | Cnp-mRNA | -0.557 | 0.16 | -0.871 | -0.243 | 0.68 | 0.547 | 0.845 | 0.00311 | 0.18 | lm.nb | Oligodendrocyte Function | NS\_MM\_NEUROINFLAM\_V1.0 | Cnp | mRNA | 0 |  | Oligodendrocyte Function |  |  | Cnp | slateblue4 | 16 | -3.48125 |
  | Gsn-mRNA | 0.453 | 0.144 | 0.17 | 0.736 | 1.37 | 1.12 | 1.67 | 0.00641 | 0.246 | lm.nb | Apoptosis, Microglia Function, Oligodendrocyte Function | NS\_MM\_NEUROINFLAM\_V1.0 | Gsn | mRNA | 0 |  | Oligodendrocyte Function;Microglia Function;Apoptosis | mmu04666;mmu04810;mmu05203 | Oligodendrocytes | Gsn | slateblue4 | 16 | 3.14583333333333 |
  | Sox10-mRNA | -1.29 | 0.416 | -2.1 | -0.474 | 0.409 | 0.233 | 0.72 | 0.00687 | 0.248 | lm.nb | Oligodendrocyte Function | NS\_MM\_NEUROINFLAM\_V1.0 | Sox10 | mRNA | 0 |  | Oligodendrocyte Function |  | Oligodendrocytes |  | slateblue4 | 16 | -3.10096153846154 |
  | Lingo1-mRNA | -1.19 | 0.39 | -1.96 | -0.427 | 0.438 | 0.258 | 0.744 | 0.00753 | 0.255 | lm.nb | Growth Factor Signaling, Oligodendrocyte Function | NS\_MM\_NEUROINFLAM\_V1.0 | Lingo1 | mRNA | 0 |  | Oligodendrocyte Function;Growth Factor Signaling |  |  | Lingo1 | slateblue4 | 16 | -3.05128205128205 |
  | Opalin-mRNA | -0.724 | 0.252 | -1.22 | -0.229 | 0.605 | 0.43 | 0.853 | 0.0111 | 0.336 | lm.nb | Oligodendrocyte Function | NS\_MM\_NEUROINFLAM\_V1.0 | Opalin | mRNA | 0 |  | Oligodendrocyte Function |  |  | Opalin | slateblue4 | 16 | -2.87301587301587 |
  | Mobp-mRNA | -0.325 | 0.129 | -0.578 | -0.0724 | 0.798 | 0.67 | 0.951 | 0.0226 | 0.508 | lm.nb | Oligodendrocyte Function | NS\_MM\_NEUROINFLAM\_V1.0 | Mobp | mRNA | 0 |  | Oligodendrocyte Function |  |  | Mobp | azure4 | 1 | -2.51937984496124 |
  | Ugt8a-mRNA | -0.542 | 0.219 | -0.972 | -0.113 | 0.687 | 0.51 | 0.925 | 0.025 | 0.539 | lm.nb | Lipid Metabolism, Oligodendrocyte Function | NS\_MM\_NEUROINFLAM\_V1.0 | Ugt8a | mRNA | 0 |  | Oligodendrocyte Function;Lipid Metabolism | mmu00565 | Oligodendrocytes | Ugt8a | azure4 | 1 | -2.47488584474886 |
  | Plp1-mRNA | -0.371 | 0.157 | -0.679 | -0.0626 | 0.773 | 0.625 | 0.958 | 0.0314 | 0.623 | lm.nb | Oligodendrocyte Function | NS\_MM\_NEUROINFLAM\_V1.0 | Plp1 | mRNA | 0 |  | Oligodendrocyte Function |  |  | Plp1 | azure4 | 1 | -2.36305732484076 |
  | Myrf-mRNA | 0.263 | 0.115 | 0.0379 | 0.487 | 1.2 | 1.03 | 1.4 | 0.0359 | 0.67 | loglinear | Oligodendrocyte Function | NS\_MM\_NEUROINFLAM\_V1.0 | Myrf | mRNA | 0 |  | Oligodendrocyte Function |  | Oligodendrocytes | Myrf | azure4 | 1 | 2.28695652173913 |
  | S1pr5-mRNA | 0.562 | 0.26 | 0.0527 | 1.07 | 1.48 | 1.04 | 2.1 | 0.046 | 0.795 | lm.nb | Neurons and Neurotransmission, Oligodendrocyte Function | NS\_MM\_NEUROINFLAM\_V1.0 | S1pr5 | mRNA | 0 |  | Oligodendrocyte Function;Neurons and Neurotransmission | mmu04071;mmu04080 |  | S1pr5 | azure4 | 1 | 2.16153846153846 |
  | Erbb3-mRNA | 1.01 | 0.526 | -0.0216 | 2.04 | 2.01 | 0.985 | 4.11 | 0.073 | 1 | lm.nb | Adaptive Immune Response, Angiogenesis, Cytokine Signaling, Growth Factor Signaling, Insulin Signaling, Oligodendrocyte Function | NS\_MM\_NEUROINFLAM\_V1.0 | Erbb3 | mRNA | 0 |  | Oligodendrocyte Function;Insulin Signaling;Growth Factor Signaling;Cytokine Signaling;Angiogenesis;Adaptive Immune Response | mmu04012;mmu04020;mmu04144;mmu05205;mmu05206 | Oligodendrocytes | Erbb3 | azure4 | 1 | 1.92015209125475 |
  | Enpp6-mRNA | 0.436 | 0.254 | -0.0618 | 0.935 | 1.35 | 0.958 | 1.91 | 0.105 | 1 | lm.nb | Oligodendrocyte Function | NS\_MM\_NEUROINFLAM\_V1.0 | Enpp6 | mRNA | 0 |  | Oligodendrocyte Function | mmu00565 |  | Enpp6 | azure4 | 1 | 1.71653543307087 |
  | Prkcq-mRNA | -0.287 | 0.17 | -0.62 | 0.0467 | 0.82 | 0.651 | 1.03 | 0.111 | 1 | lm.nb | Adaptive Immune Response, Apoptosis, Autophagy, NF-kB, Oligodendrocyte Function | NS\_MM\_NEUROINFLAM\_V1.0 | Prkcq | mRNA | 0 |  | Oligodendrocyte Function;NF-kB;Autophagy;Apoptosis;Adaptive Immune Response | mmu04064;mmu04270;mmu04530;mmu04660;mmu04750;mmu04920;mmu04931;mmu05162 | Oligodendrocytes | Prkcq | azure4 | 1 | -1.68823529411765 |
  | Mal-mRNA | -0.169 | 0.132 | -0.428 | 0.0896 | 0.889 | 0.743 | 1.06 | 0.218 | 1 | lm.nb | Oligodendrocyte Function | NS\_MM\_NEUROINFLAM\_V1.0 | Mal | mRNA | 0 |  | Oligodendrocyte Function |  |  | Mal | azure4 | 1 | -1.28030303030303 |
  | Pllp-mRNA | 0.428 | 0.335 | -0.23 | 1.09 | 1.35 | 0.853 | 2.12 | 0.22 | 1 | lm.nb | Oligodendrocyte Function | NS\_MM\_NEUROINFLAM\_V1.0 | Pllp | mRNA | 0 |  | Oligodendrocyte Function |  | Oligodendrocytes | Pllp | azure4 | 1 | 1.27761194029851 |
  | Plekhm1-mRNA | 0.153 | 0.134 | -0.109 | 0.416 | 1.11 | 0.927 | 1.33 | 0.27 | 1 | lm.nb | Oligodendrocyte Function | NS\_MM\_NEUROINFLAM\_V1.0 | Plekhm1 | mRNA | 0 |  | Oligodendrocyte Function |  |  | Plekhm1 | azure4 | 1 | 1.14179104477612 |
  | Fa2h-mRNA | 0.161 | 0.145 | -0.123 | 0.446 | 1.12 | 0.918 | 1.36 | 0.284 | 1 | lm.nb | Lipid Metabolism, Oligodendrocyte Function | NS\_MM\_NEUROINFLAM\_V1.0 | Fa2h | mRNA | 0 |  | Oligodendrocyte Function;Lipid Metabolism |  | Oligodendrocytes | Fa2h | azure4 | 1 | 1.11034482758621 |
  | Mag-mRNA | 0.105 | 0.108 | -0.106 | 0.317 | 1.08 | 0.929 | 1.25 | 0.344 | 1 | loglinear | Growth Factor Signaling, Matrix Remodeling, Oligodendrocyte Function | NS\_MM\_NEUROINFLAM\_V1.0 | Mag | mRNA | 0 |  | Oligodendrocyte Function;Matrix Remodeling;Growth Factor Signaling | mmu04514 |  | Mag | azure4 | 1 | 0.972222222222222 |
  | Gjb1-mRNA | -0.147 | 0.208 | -0.555 | 0.261 | 0.903 | 0.681 | 1.2 | 0.49 | 1 | lm.nb | Neurons and Neurotransmission, Oligodendrocyte Function | NS\_MM\_NEUROINFLAM\_V1.0 | Gjb1 | mRNA | 0 |  | Oligodendrocyte Function;Neurons and Neurotransmission |  | Oligodendrocytes | Gjb1 | azure4 | 1 | -0.706730769230769 |

  ##### DE Results - Oligodendrocyte Function genes - DPI: 1 vs.CTRL

  Table displaying each sample's global significance scores and directed global significance scores as defined in the heatmaps above. The global significance score is calculated as the square root of the mean squared t-statistic for the genes in a gene set, with t-statistics coming from the linear regression underlying our differential expression analysis. The directed global significance score is calculated as the square root of the mean signed squared t-statistic for the genes in a gene set, with t-statistics coming from the linear regression underlying our differential expression analysis.

- **Volcano Plot: DPI: 2 vs.CTRL  
  More Plot Information**


  ##### Volcano Plot: DPI: 2 vs.CTRL

  Volcano plot displaying each gene's -log10(p-value) and log2 fold change for the selected covariate. Highly statistically significant genes fall at the top of the plot, and highly differentially expressed genes fall to either side. Genes within the selected gene set are highlighted in orange. Horizontal lines indicate various False Discovery Rate (FDR) thresholds.
- **DE Results - Oligodendrocyte Function genes - DPI: 2 vs.CTRL  
  More Plot Information  Download CSV Data**

  | Probe Label | Log2 fold change | std error (log2) | Lower confidence limit (log2) | Upper confidence limit (log2) | Linear fold change | Lower confidence limit (linear) | Upper confidence limit (linear) | P-value | BY.p.value | method | Gene.sets | Codeset.Name | Probe.Label | Analyte.Type | Is.Control | Control.Type | Probe.Annotation | KEGG.Pathways | Cell.Type | Official.Gene.Name | volcanocol | volcanopch | tstats.all |
  | --- | --- | --- | --- | --- | --- | --- | --- | --- | --- | --- | --- | --- | --- | --- | --- | --- | --- | --- | --- | --- | --- | --- | --- |
  | Opalin-mRNA | -2.58 | 0.263 | -3.1 | -2.07 | 0.167 | 0.117 | 0.239 | 3.61e-08 | 1.27e-05 | lm.nb | Oligodendrocyte Function | NS\_MM\_NEUROINFLAM\_V1.0 | Opalin | mRNA | 0 |  | Oligodendrocyte Function |  |  | Opalin | slateblue1 | 16 | -9.80988593155893 |
  | Ugt8a-mRNA | -1.74 | 0.224 | -2.18 | -1.3 | 0.3 | 0.221 | 0.407 | 8.23e-07 | 0.000145 | lm.nb | Lipid Metabolism, Oligodendrocyte Function | NS\_MM\_NEUROINFLAM\_V1.0 | Ugt8a | mRNA | 0 |  | Oligodendrocyte Function;Lipid Metabolism | mmu00565 | Oligodendrocytes | Ugt8a | slateblue1 | 16 | -7.76785714285714 |
  | Sox10-mRNA | -2.66 | 0.427 | -3.49 | -1.82 | 0.158 | 0.0887 | 0.283 | 1.22e-05 | 0.0011 | lm.nb | Oligodendrocyte Function | NS\_MM\_NEUROINFLAM\_V1.0 | Sox10 | mRNA | 0 |  | Oligodendrocyte Function |  | Oligodendrocytes |  | slateblue1 | 16 | -6.22950819672131 |
  | Cnp-mRNA | -0.912 | 0.16 | -1.23 | -0.598 | 0.531 | 0.427 | 0.661 | 3.34e-05 | 0.0024 | lm.nb | Oligodendrocyte Function | NS\_MM\_NEUROINFLAM\_V1.0 | Cnp | mRNA | 0 |  | Oligodendrocyte Function |  |  | Cnp | slateblue1 | 16 | -5.7 |
  | Plp1-mRNA | -0.753 | 0.157 | -1.06 | -0.445 | 0.593 | 0.479 | 0.734 | 2e-04 | 0.00882 | lm.nb | Oligodendrocyte Function | NS\_MM\_NEUROINFLAM\_V1.0 | Plp1 | mRNA | 0 |  | Oligodendrocyte Function |  |  | Plp1 | slateblue1 | 16 | -4.79617834394904 |
  | Prkcq-mRNA | -0.864 | 0.185 | -1.23 | -0.501 | 0.549 | 0.427 | 0.707 | 0.000258 | 0.0106 | lm.nb | Adaptive Immune Response, Apoptosis, Autophagy, NF-kB, Oligodendrocyte Function | NS\_MM\_NEUROINFLAM\_V1.0 | Prkcq | mRNA | 0 |  | Oligodendrocyte Function;NF-kB;Autophagy;Apoptosis;Adaptive Immune Response | mmu04064;mmu04270;mmu04530;mmu04660;mmu04750;mmu04920;mmu04931;mmu05162 | Oligodendrocytes | Prkcq | slateblue2 | 16 | -4.67027027027027 |
  | Gjb1-mRNA | -0.959 | 0.214 | -1.38 | -0.54 | 0.514 | 0.385 | 0.688 | 0.000377 | 0.014 | lm.nb | Neurons and Neurotransmission, Oligodendrocyte Function | NS\_MM\_NEUROINFLAM\_V1.0 | Gjb1 | mRNA | 0 |  | Oligodendrocyte Function;Neurons and Neurotransmission |  | Oligodendrocytes | Gjb1 | slateblue2 | 16 | -4.48130841121495 |
  | Lingo1-mRNA | -1.64 | 0.39 | -2.4 | -0.874 | 0.321 | 0.189 | 0.546 | 0.000681 | 0.0218 | lm.nb | Growth Factor Signaling, Oligodendrocyte Function | NS\_MM\_NEUROINFLAM\_V1.0 | Lingo1 | mRNA | 0 |  | Oligodendrocyte Function;Growth Factor Signaling |  |  | Lingo1 | slateblue2 | 16 | -4.2051282051282 |
  | Fa2h-mRNA | -0.622 | 0.155 | -0.925 | -0.318 | 0.65 | 0.527 | 0.802 | 0.00101 | 0.0278 | lm.nb | Lipid Metabolism, Oligodendrocyte Function | NS\_MM\_NEUROINFLAM\_V1.0 | Fa2h | mRNA | 0 |  | Oligodendrocyte Function;Lipid Metabolism |  | Oligodendrocytes | Fa2h | slateblue2 | 16 | -4.01290322580645 |
  | Pllp-mRNA | -1.3 | 0.362 | -2.01 | -0.587 | 0.407 | 0.249 | 0.666 | 0.00249 | 0.053 | lm.nb | Oligodendrocyte Function | NS\_MM\_NEUROINFLAM\_V1.0 | Pllp | mRNA | 0 |  | Oligodendrocyte Function |  | Oligodendrocytes | Pllp | slateblue3 | 16 | -3.59116022099448 |
  | Enpp6-mRNA | -0.781 | 0.273 | -1.32 | -0.245 | 0.582 | 0.401 | 0.844 | 0.0114 | 0.183 | lm.nb | Oligodendrocyte Function | NS\_MM\_NEUROINFLAM\_V1.0 | Enpp6 | mRNA | 0 |  | Oligodendrocyte Function | mmu00565 |  | Enpp6 | slateblue4 | 16 | -2.86080586080586 |
  | S1pr5-mRNA | 0.716 | 0.256 | 0.214 | 1.22 | 1.64 | 1.16 | 2.33 | 0.013 | 0.202 | lm.nb | Neurons and Neurotransmission, Oligodendrocyte Function | NS\_MM\_NEUROINFLAM\_V1.0 | S1pr5 | mRNA | 0 |  | Oligodendrocyte Function;Neurons and Neurotransmission | mmu04071;mmu04080 |  | S1pr5 | slateblue4 | 16 | 2.796875 |
  | Gal3st1-mRNA | -0.356 | 0.158 | -0.665 | -0.0472 | 0.781 | 0.631 | 0.968 | 0.0381 | 0.474 | lm.nb | Lipid Metabolism, Oligodendrocyte Function | NS\_MM\_NEUROINFLAM\_V1.0 | Gal3st1 | mRNA | 0 |  | Oligodendrocyte Function;Lipid Metabolism | mmu00565 | Oligodendrocytes | Gal3st1 | slateblue4 | 16 | -2.25316455696203 |
  | Gsn-mRNA | 0.323 | 0.145 | 0.0394 | 0.607 | 1.25 | 1.03 | 1.52 | 0.0403 | 0.495 | lm.nb | Apoptosis, Microglia Function, Oligodendrocyte Function | NS\_MM\_NEUROINFLAM\_V1.0 | Gsn | mRNA | 0 |  | Oligodendrocyte Function;Microglia Function;Apoptosis | mmu04666;mmu04810;mmu05203 | Oligodendrocytes | Gsn | azure4 | 1 | 2.22758620689655 |
  | Myrf-mRNA | 0.242 | 0.115 | 0.0173 | 0.466 | 1.18 | 1.01 | 1.38 | 0.0509 | 0.602 | loglinear | Oligodendrocyte Function | NS\_MM\_NEUROINFLAM\_V1.0 | Myrf | mRNA | 0 |  | Oligodendrocyte Function |  | Oligodendrocytes | Myrf | azure4 | 1 | 2.10434782608696 |
  | Plekhm1-mRNA | 0.27 | 0.133 | 0.00906 | 0.53 | 1.21 | 1.01 | 1.44 | 0.0595 | 0.678 | lm.nb | Oligodendrocyte Function | NS\_MM\_NEUROINFLAM\_V1.0 | Plekhm1 | mRNA | 0 |  | Oligodendrocyte Function |  |  | Plekhm1 | azure4 | 1 | 2.03007518796993 |
  | Gpr62-mRNA | -0.266 | 0.141 | -0.542 | 0.0104 | 0.832 | 0.687 | 1.01 | 0.0775 | 0.837 | lm.nb | Oligodendrocyte Function | NS\_MM\_NEUROINFLAM\_V1.0 | Gpr62 | mRNA | 0 |  | Oligodendrocyte Function |  |  | Gpr62 | azure4 | 1 | -1.88652482269504 |
  | Mal-mRNA | -0.227 | 0.132 | -0.486 | 0.0316 | 0.854 | 0.714 | 1.02 | 0.105 | 1 | lm.nb | Oligodendrocyte Function | NS\_MM\_NEUROINFLAM\_V1.0 | Mal | mRNA | 0 |  | Oligodendrocyte Function |  |  | Mal | azure4 | 1 | -1.71969696969697 |
  | Bcas1-mRNA | -0.198 | 0.123 | -0.439 | 0.0441 | 0.872 | 0.738 | 1.03 | 0.129 | 1 | loglinear | Oligodendrocyte Function | NS\_MM\_NEUROINFLAM\_V1.0 | Bcas1 | mRNA | 0 |  | Oligodendrocyte Function |  | Oligodendrocytes | Bcas1 | azure4 | 1 | -1.60975609756098 |
  | Plxnb3-mRNA | -0.274 | 0.191 | -0.648 | 0.101 | 0.827 | 0.638 | 1.07 | 0.172 | 1 | lm.nb | Oligodendrocyte Function | NS\_MM\_NEUROINFLAM\_V1.0 | Plxnb3 | mRNA | 0 |  | Oligodendrocyte Function | mmu04360 | Oligodendrocytes | Plxnb3 | azure4 | 1 | -1.43455497382199 |

  ##### DE Results - Oligodendrocyte Function genes - DPI: 2 vs.CTRL

  Table displaying each sample's global significance scores and directed global significance scores as defined in the heatmaps above. The global significance score is calculated as the square root of the mean squared t-statistic for the genes in a gene set, with t-statistics coming from the linear regression underlying our differential expression analysis. The directed global significance score is calculated as the square root of the mean signed squared t-statistic for the genes in a gene set, with t-statistics coming from the linear regression underlying our differential expression analysis.

- **Volcano Plot: DPI: 3 vs.CTRL  
  More Plot Information**


  ##### Volcano Plot: DPI: 3 vs.CTRL

  Volcano plot displaying each gene's -log10(p-value) and log2 fold change for the selected covariate. Highly statistically significant genes fall at the top of the plot, and highly differentially expressed genes fall to either side. Genes within the selected gene set are highlighted in orange. Horizontal lines indicate various False Discovery Rate (FDR) thresholds.
- **DE Results - Oligodendrocyte Function genes - DPI: 3 vs.CTRL  
  More Plot Information  Download CSV Data**

  | Probe Label | Log2 fold change | std error (log2) | Lower confidence limit (log2) | Upper confidence limit (log2) | Linear fold change | Lower confidence limit (linear) | Upper confidence limit (linear) | P-value | BY.p.value | method | Gene.sets | Codeset.Name | Probe.Label | Analyte.Type | Is.Control | Control.Type | Probe.Annotation | KEGG.Pathways | Cell.Type | Official.Gene.Name | volcanocol | volcanopch | tstats.all |
  | --- | --- | --- | --- | --- | --- | --- | --- | --- | --- | --- | --- | --- | --- | --- | --- | --- | --- | --- | --- | --- | --- | --- | --- |
  | Ugt8a-mRNA | -2.5 | 0.229 | -2.95 | -2.05 | 0.177 | 0.129 | 0.241 | 8.21e-09 | 3.12e-06 | lm.nb | Lipid Metabolism, Oligodendrocyte Function | NS\_MM\_NEUROINFLAM\_V1.0 | Ugt8a | mRNA | 0 |  | Oligodendrocyte Function;Lipid Metabolism | mmu00565 | Oligodendrocytes | Ugt8a | slateblue1 | 16 | -10.9170305676856 |
  | Gjb1-mRNA | -1.43 | 0.219 | -1.86 | -1 | 0.371 | 0.276 | 0.5 | 7.05e-06 | 0.000507 | lm.nb | Neurons and Neurotransmission, Oligodendrocyte Function | NS\_MM\_NEUROINFLAM\_V1.0 | Gjb1 | mRNA | 0 |  | Oligodendrocyte Function;Neurons and Neurotransmission |  | Oligodendrocytes | Gjb1 | slateblue1 | 16 | -6.5296803652968 |
  | Opalin-mRNA | -1.6 | 0.256 | -2.1 | -1.1 | 0.33 | 0.233 | 0.467 | 1.15e-05 | 0.000782 | lm.nb | Oligodendrocyte Function | NS\_MM\_NEUROINFLAM\_V1.0 | Opalin | mRNA | 0 |  | Oligodendrocyte Function |  |  | Opalin | slateblue1 | 16 | -6.25 |
  | Pllp-mRNA | -2.43 | 0.403 | -3.22 | -1.64 | 0.186 | 0.107 | 0.321 | 1.75e-05 | 0.00105 | lm.nb | Oligodendrocyte Function | NS\_MM\_NEUROINFLAM\_V1.0 | Pllp | mRNA | 0 |  | Oligodendrocyte Function |  | Oligodendrocytes | Pllp | slateblue1 | 16 | -6.02977667493797 |
  | Fa2h-mRNA | -0.935 | 0.16 | -1.25 | -0.621 | 0.523 | 0.421 | 0.65 | 2.55e-05 | 0.00141 | lm.nb | Lipid Metabolism, Oligodendrocyte Function | NS\_MM\_NEUROINFLAM\_V1.0 | Fa2h | mRNA | 0 |  | Oligodendrocyte Function;Lipid Metabolism |  | Oligodendrocytes | Fa2h | slateblue1 | 16 | -5.84375 |
  | Plxnb3-mRNA | -0.983 | 0.207 | -1.39 | -0.577 | 0.506 | 0.382 | 0.671 | 0.00022 | 0.00837 | lm.nb | Oligodendrocyte Function | NS\_MM\_NEUROINFLAM\_V1.0 | Plxnb3 | mRNA | 0 |  | Oligodendrocyte Function | mmu04360 | Oligodendrocytes | Plxnb3 | slateblue1 | 16 | -4.7487922705314 |
  | Mag-mRNA | -0.474 | 0.108 | -0.686 | -0.262 | 0.72 | 0.621 | 0.834 | 0.000459 | 0.0149 | loglinear | Growth Factor Signaling, Matrix Remodeling, Oligodendrocyte Function | NS\_MM\_NEUROINFLAM\_V1.0 | Mag | mRNA | 0 |  | Oligodendrocyte Function;Matrix Remodeling;Growth Factor Signaling | mmu04514 |  | Mag | slateblue2 | 16 | -4.38888888888889 |
  | Sox10-mRNA | -1.83 | 0.419 | -2.65 | -1.01 | 0.281 | 0.159 | 0.496 | 0.000473 | 0.015 | lm.nb | Oligodendrocyte Function | NS\_MM\_NEUROINFLAM\_V1.0 | Sox10 | mRNA | 0 |  | Oligodendrocyte Function |  | Oligodendrocytes |  | slateblue2 | 16 | -4.36754176610979 |
  | Gal3st1-mRNA | -0.71 | 0.166 | -1.04 | -0.386 | 0.611 | 0.488 | 0.765 | 0.000561 | 0.0175 | lm.nb | Lipid Metabolism, Oligodendrocyte Function | NS\_MM\_NEUROINFLAM\_V1.0 | Gal3st1 | mRNA | 0 |  | Oligodendrocyte Function;Lipid Metabolism | mmu00565 | Oligodendrocytes | Gal3st1 | slateblue2 | 16 | -4.27710843373494 |
  | Prkcq-mRNA | -0.735 | 0.181 | -1.09 | -0.379 | 0.601 | 0.47 | 0.769 | 0.000927 | 0.0267 | lm.nb | Adaptive Immune Response, Apoptosis, Autophagy, NF-kB, Oligodendrocyte Function | NS\_MM\_NEUROINFLAM\_V1.0 | Prkcq | mRNA | 0 |  | Oligodendrocyte Function;NF-kB;Autophagy;Apoptosis;Adaptive Immune Response | mmu04064;mmu04270;mmu04530;mmu04660;mmu04750;mmu04920;mmu04931;mmu05162 | Oligodendrocytes | Prkcq | slateblue2 | 16 | -4.06077348066298 |
  | Plp1-mRNA | -0.621 | 0.157 | -0.929 | -0.312 | 0.65 | 0.525 | 0.805 | 0.00116 | 0.0309 | lm.nb | Oligodendrocyte Function | NS\_MM\_NEUROINFLAM\_V1.0 | Plp1 | mRNA | 0 |  | Oligodendrocyte Function |  |  | Plp1 | slateblue2 | 16 | -3.95541401273885 |
  | Cnp-mRNA | -0.629 | 0.16 | -0.943 | -0.315 | 0.647 | 0.52 | 0.804 | 0.0012 | 0.0317 | lm.nb | Oligodendrocyte Function | NS\_MM\_NEUROINFLAM\_V1.0 | Cnp | mRNA | 0 |  | Oligodendrocyte Function |  |  | Cnp | slateblue2 | 16 | -3.93125 |
  | Enpp6-mRNA | -1.02 | 0.279 | -1.57 | -0.474 | 0.493 | 0.337 | 0.72 | 0.00212 | 0.0512 | lm.nb | Oligodendrocyte Function | NS\_MM\_NEUROINFLAM\_V1.0 | Enpp6 | mRNA | 0 |  | Oligodendrocyte Function | mmu00565 |  | Enpp6 | slateblue3 | 16 | -3.65591397849462 |
  | Myrf-mRNA | 0.269 | 0.115 | 0.044 | 0.493 | 1.2 | 1.03 | 1.41 | 0.0323 | 0.462 | loglinear | Oligodendrocyte Function | NS\_MM\_NEUROINFLAM\_V1.0 | Myrf | mRNA | 0 |  | Oligodendrocyte Function |  | Oligodendrocytes | Myrf | slateblue4 | 16 | 2.33913043478261 |
  | Mobp-mRNA | 0.288 | 0.128 | 0.0369 | 0.539 | 1.22 | 1.03 | 1.45 | 0.039 | 0.534 | lm.nb | Oligodendrocyte Function | NS\_MM\_NEUROINFLAM\_V1.0 | Mobp | mRNA | 0 |  | Oligodendrocyte Function |  |  | Mobp | azure4 | 1 | 2.25 |
  | Lingo1-mRNA | -0.639 | 0.389 | -1.4 | 0.123 | 0.642 | 0.378 | 1.09 | 0.12 | 1 | lm.nb | Growth Factor Signaling, Oligodendrocyte Function | NS\_MM\_NEUROINFLAM\_V1.0 | Lingo1 | mRNA | 0 |  | Oligodendrocyte Function;Growth Factor Signaling |  |  | Lingo1 | azure4 | 1 | -1.6426735218509 |
  | Plekhm1-mRNA | -0.185 | 0.138 | -0.455 | 0.0851 | 0.88 | 0.729 | 1.06 | 0.198 | 1 | lm.nb | Oligodendrocyte Function | NS\_MM\_NEUROINFLAM\_V1.0 | Plekhm1 | mRNA | 0 |  | Oligodendrocyte Function |  |  | Plekhm1 | azure4 | 1 | -1.34057971014493 |
  | Mog-mRNA | -0.16 | 0.121 | -0.396 | 0.0766 | 0.895 | 0.76 | 1.05 | 0.204 | 1 | lm.nb | Oligodendrocyte Function | NS\_MM\_NEUROINFLAM\_V1.0 | Mog | mRNA | 0 |  | Oligodendrocyte Function |  |  | Mog | azure4 | 1 | -1.32231404958678 |
  | Bcas1-mRNA | -0.136 | 0.123 | -0.377 | 0.106 | 0.91 | 0.77 | 1.08 | 0.287 | 1 | loglinear | Oligodendrocyte Function | NS\_MM\_NEUROINFLAM\_V1.0 | Bcas1 | mRNA | 0 |  | Oligodendrocyte Function |  | Oligodendrocytes | Bcas1 | azure4 | 1 | -1.10569105691057 |
  | Erbb3-mRNA | -0.527 | 0.547 | -1.6 | 0.546 | 0.694 | 0.33 | 1.46 | 0.35 | 1 | lm.nb | Adaptive Immune Response, Angiogenesis, Cytokine Signaling, Growth Factor Signaling, Insulin Signaling, Oligodendrocyte Function | NS\_MM\_NEUROINFLAM\_V1.0 | Erbb3 | mRNA | 0 |  | Oligodendrocyte Function;Insulin Signaling;Growth Factor Signaling;Cytokine Signaling;Angiogenesis;Adaptive Immune Response | mmu04012;mmu04020;mmu04144;mmu05205;mmu05206 | Oligodendrocytes | Erbb3 | azure4 | 1 | -0.963436928702011 |

  ##### DE Results - Oligodendrocyte Function genes - DPI: 3 vs.CTRL

  Table displaying each sample's global significance scores and directed global significance scores as defined in the heatmaps above. The global significance score is calculated as the square root of the mean squared t-statistic for the genes in a gene set, with t-statistics coming from the linear regression underlying our differential expression analysis. The directed global significance score is calculated as the square root of the mean signed squared t-statistic for the genes in a gene set, with t-statistics coming from the linear regression underlying our differential expression analysis.

DPI:
- DPI: differential expression in 1 vs. baseline of CTRL
- DPI: differential expression in 2 vs. baseline of CTRL
- DPI: differential expression in 3 vs. baseline of CTRL

- **Volcano Plot: DPI: 1 vs.CTRL  
  More Plot Information**


  ##### Volcano Plot: DPI: 1 vs.CTRL

  Volcano plot displaying each gene's -log10(p-value) and log2 fold change for the selected covariate. Highly statistically significant genes fall at the top of the plot, and highly differentially expressed genes fall to either side. Genes within the selected gene set are highlighted in orange. Horizontal lines indicate various False Discovery Rate (FDR) thresholds.
- **DE Results - Wnt genes - DPI: 1 vs.CTRL  
  More Plot Information  Download CSV Data**

  | Probe Label | Log2 fold change | std error (log2) | Lower confidence limit (log2) | Upper confidence limit (log2) | Linear fold change | Lower confidence limit (linear) | Upper confidence limit (linear) | P-value | BY.p.value | method | Gene.sets | Codeset.Name | Probe.Label | Analyte.Type | Is.Control | Control.Type | Probe.Annotation | KEGG.Pathways | Cell.Type | Official.Gene.Name | volcanocol | volcanopch | tstats.all |
  | --- | --- | --- | --- | --- | --- | --- | --- | --- | --- | --- | --- | --- | --- | --- | --- | --- | --- | --- | --- | --- | --- | --- | --- |
  | Prkacb-mRNA | -0.327 | 0.0629 | -0.451 | -0.204 | 0.797 | 0.732 | 0.868 | 8.65e-05 | 0.0321 | loglinear | Adaptive Immune Response, Angiogenesis, Apoptosis, Autophagy, Growth Factor Signaling, Lipid Metabolism, Neurons and Neurotransmission, Wnt | NS\_MM\_NEUROINFLAM\_V1.0 | Prkacb | mRNA | 0 |  | Wnt;Neurons and Neurotransmission;Lipid Metabolism;Growth Factor Signaling;Autophagy;Apoptosis;Angiogenesis;Adaptive Immune Response | mmu04010;mmu04014;mmu04020;mmu04024;mmu04062;mmu04114;mmu04210;mmu04211;mmu04213;mmu04261;mmu04270;mmu04310;mmu04540;mmu04611;mmu04713;mmu04720;mmu04723;mmu04724;mmu04725;mmu04726;mmu04728;mmu04750;mmu04910;mmu04911;mmu04912;mmu04913;mmu04914;mmu04915;mmu04916;mmu04919;mmu04921;mmu04922;mmu04923;mmu04924;mmu04925;mmu05012;mmu05020;mmu05030;mmu05031;mmu05146;mmu05166;mmu05169;mmu05200;mmu05203;mmu05205;mmu05414 |  | Prkacb | slateblue2 | 16 | -5.19872813990461 |
  | Ppp3r1-mRNA | -0.28 | 0.0577 | -0.393 | -0.167 | 0.823 | 0.761 | 0.891 | 0.000175 | 0.0442 | loglinear | Adaptive Immune Response, Apoptosis, Growth Factor Signaling, Innate Immune Response, Wnt | NS\_MM\_NEUROINFLAM\_V1.0 | Ppp3r1 | mRNA | 0 |  | Wnt;Innate Immune Response;Growth Factor Signaling;Apoptosis;Adaptive Immune Response | mmu04010;mmu04020;mmu04022;mmu04114;mmu04210;mmu04310;mmu04360;mmu04370;mmu04380;mmu04650;mmu04660;mmu04662;mmu04720;mmu04724;mmu04921;mmu04922;mmu04924;mmu05010;mmu05014;mmu05031;mmu05152;mmu05166 |  | Ppp3r1 | slateblue2 | 16 | -4.8526863084922 |
  | Ppp3cb-mRNA | -0.411 | 0.0979 | -0.602 | -0.219 | 0.752 | 0.659 | 0.859 | 0.000686 | 0.0806 | lm.nb | Adaptive Immune Response, Apoptosis, Growth Factor Signaling, Innate Immune Response, Wnt | NS\_MM\_NEUROINFLAM\_V1.0 | Ppp3cb | mRNA | 0 |  | Wnt;Innate Immune Response;Growth Factor Signaling;Apoptosis;Adaptive Immune Response | mmu04010;mmu04020;mmu04022;mmu04114;mmu04210;mmu04310;mmu04360;mmu04370;mmu04380;mmu04650;mmu04660;mmu04662;mmu04720;mmu04724;mmu04728;mmu04921;mmu04922;mmu04924;mmu05010;mmu05014;mmu05031;mmu05152;mmu05166 |  | Ppp3cb | slateblue3 | 16 | -4.19816138917262 |
  | Mapk10-mRNA | -0.539 | 0.138 | -0.81 | -0.269 | 0.688 | 0.57 | 0.83 | 0.00126 | 0.111 | lm.nb | Adaptive Immune Response, Apoptosis, Autophagy, Cellular Stress, Growth Factor Signaling, Innate Immune Response, Wnt | NS\_MM\_NEUROINFLAM\_V1.0 | Mapk10 | mRNA | 0 |  | Wnt;Innate Immune Response;Growth Factor Signaling;Cellular Stress;Autophagy;Apoptosis;Adaptive Immune Response | mmu04010;mmu04012;mmu04014;mmu04024;mmu04068;mmu04071;mmu04141;mmu04310;mmu04380;mmu04510;mmu04620;mmu04621;mmu04622;mmu04664;mmu04668;mmu04722;mmu04723;mmu04728;mmu04750;mmu04910;mmu04912;mmu04914;mmu04917;mmu04920;mmu04930;mmu04931;mmu04932;mmu04933;mmu05132;mmu05133;mmu05142;mmu05145;mmu05152;mmu05160;mmu05161;mmu05164;mmu05168;mmu05169;mmu05200;mmu05210;mmu05212;mmu05231 |  | Mapk10 | slateblue4 | 16 | -3.90579710144928 |
  | Psmb8-mRNA | 1.36 | 0.365 | 0.649 | 2.08 | 2.57 | 1.57 | 4.23 | 0.00179 | 0.129 | lm.nb | Adaptive Immune Response, Angiogenesis, Apoptosis, Astrocyte Function, Cell Cycle, Cytokine Signaling, Growth Factor Signaling, Inflammatory Signaling, Insulin Signaling, Microglia Function, NF-kB, Wnt | NS\_MM\_NEUROINFLAM\_V1.0 | Psmb8 | mRNA | 0 |  | Wnt;NF-kB;Microglia Function;Insulin Signaling;Inflammatory Signaling;Growth Factor Signaling;Cytokine Signaling;Cell Cycle;Astrocyte Function;Apoptosis;Angiogenesis;Adaptive Immune Response |  |  | Psmb8 | slateblue4 | 16 | 3.72602739726027 |
  | Rac1-mRNA | -0.928 | 0.256 | -1.43 | -0.426 | 0.525 | 0.371 | 0.744 | 0.00229 | 0.149 | lm.nb | Adaptive Immune Response, Angiogenesis, Autophagy, Growth Factor Signaling, Innate Immune Response, Neurons and Neurotransmission, Wnt | NS\_MM\_NEUROINFLAM\_V1.0 | Rac1 | mRNA | 0 |  | Wnt;Neurons and Neurotransmission;Innate Immune Response;Growth Factor Signaling;Autophagy;Angiogenesis;Adaptive Immune Response | mmu04010;mmu04014;mmu04015;mmu04024;mmu04062;mmu04071;mmu04145;mmu04151;mmu04310;mmu04360;mmu04370;mmu04380;mmu04510;mmu04520;mmu04620;mmu04650;mmu04662;mmu04664;mmu04666;mmu04670;mmu04722;mmu04810;mmu04932;mmu04933;mmu05014;mmu05100;mmu05132;mmu05200;mmu05203;mmu05205;mmu05210;mmu05211;mmu05212;mmu05231;mmu05416 |  | Rac1 | slateblue4 | 16 | -3.625 |
  | Rhoa-mRNA | -0.902 | 0.261 | -1.41 | -0.391 | 0.535 | 0.375 | 0.762 | 0.00322 | 0.18 | lm.nb | Angiogenesis, Autophagy, Growth Factor Signaling, Wnt | NS\_MM\_NEUROINFLAM\_V1.0 | Rhoa | mRNA | 0 |  | Wnt;Growth Factor Signaling;Autophagy;Angiogenesis | mmu04014;mmu04015;mmu04022;mmu04024;mmu04062;mmu04071;mmu04072;mmu04144;mmu04270;mmu04310;mmu04350;mmu04360;mmu04510;mmu04520;mmu04530;mmu04611;mmu04660;mmu04670;mmu04722;mmu04810;mmu04921;mmu05100;mmu05133;mmu05152;mmu05200;mmu05203;mmu05205;mmu05206;mmu05210 |  | Rhoa | slateblue4 | 16 | -3.45593869731801 |
  | Xiap-mRNA | -0.529 | 0.155 | -0.833 | -0.225 | 0.693 | 0.561 | 0.856 | 0.00359 | 0.184 | lm.nb | Apoptosis, Innate Immune Response, NF-kB, Wnt | NS\_MM\_NEUROINFLAM\_V1.0 | Xiap | mRNA | 0 |  | Wnt;NF-kB;Innate Immune Response;Apoptosis | mmu04064;mmu04120;mmu04210;mmu04510;mmu05145;mmu05166;mmu05200;mmu05222 |  | Xiap | slateblue4 | 16 | -3.41290322580645 |
  | Apc-mRNA | -0.195 | 0.0593 | -0.311 | -0.0783 | 0.874 | 0.806 | 0.947 | 0.00471 | 0.206 | loglinear | Apoptosis, Astrocyte Function, Wnt | NS\_MM\_NEUROINFLAM\_V1.0 | Apc | mRNA | 0 |  | Wnt;Astrocyte Function;Apoptosis | mmu04310;mmu04390;mmu04550;mmu04810;mmu05166;mmu05200;mmu05206;mmu05210;mmu05213 |  | Apc | slateblue4 | 16 | -3.28836424957841 |
  | Sox4-mRNA | -0.561 | 0.198 | -0.948 | -0.173 | 0.678 | 0.518 | 0.887 | 0.0119 | 0.351 | lm.nb | Microglia Function, Wnt | NS\_MM\_NEUROINFLAM\_V1.0 | Sox4 | mRNA | 0 |  | Wnt;Microglia Function |  |  | Sox4 | slateblue4 | 16 | -2.83333333333333 |
  | Ash2l-mRNA | -0.264 | 0.109 | -0.478 | -0.0497 | 0.833 | 0.718 | 0.966 | 0.0281 | 0.59 | loglinear | Epigenetic Regulation, Wnt | NS\_MM\_NEUROINFLAM\_V1.0 | Ash2l | mRNA | 0 |  | Wnt;Epigenetic Regulation |  |  | Ash2l | azure4 | 1 | -2.42201834862385 |
  | Myc-mRNA | -0.803 | 0.346 | -1.48 | -0.124 | 0.573 | 0.358 | 0.918 | 0.034 | 0.652 | lm.nb | Apoptosis, Cell Cycle, Cytokine Signaling, Growth Factor Signaling, Notch, Wnt | NS\_MM\_NEUROINFLAM\_V1.0 | Myc | mRNA | 0 |  | Wnt;Notch;Growth Factor Signaling;Cytokine Signaling;Cell Cycle;Apoptosis | mmu04010;mmu04012;mmu04110;mmu04151;mmu04310;mmu04350;mmu04390;mmu04550;mmu04630;mmu04919;mmu05161;mmu05166;mmu05169;mmu05200;mmu05202;mmu05205;mmu05206;mmu05210;mmu05213;mmu05216;mmu05219;mmu05220;mmu05221;mmu05222;mmu05230 |  | Myc | azure4 | 1 | -2.32080924855491 |
  | Akt2-mRNA | -0.291 | 0.142 | -0.568 | -0.0129 | 0.818 | 0.674 | 0.991 | 0.057 | 0.91 | loglinear | Adaptive Immune Response, Angiogenesis, Apoptosis, Autophagy, Carbohydrate Metabolism, Cytokine Signaling, Growth Factor Signaling, Innate Immune Response, Insulin Signaling, Neurons and Neurotransmission, Wnt | NS\_MM\_NEUROINFLAM\_V1.0 | Akt2 | mRNA | 0 |  | Wnt;Neurons and Neurotransmission;Insulin Signaling;Innate Immune Response;Growth Factor Signaling;Cytokine Signaling;Carbohydrate Metabolism;Autophagy;Apoptosis;Angiogenesis;Adaptive Immune Response | mmu04010;mmu04012;mmu04014;mmu04015;mmu04022;mmu04024;mmu04062;mmu04066;mmu04068;mmu04071;mmu04072;mmu04150;mmu04151;mmu04152;mmu04210;mmu04211;mmu04213;mmu04261;mmu04370;mmu04380;mmu04510;mmu04530;mmu04550;mmu04611;mmu04620;mmu04630;mmu04660;mmu04662;mmu04664;mmu04666;mmu04668;mmu04722;mmu04725;mmu04728;mmu04910;mmu04914;mmu04915;mmu04917;mmu04919;mmu04920;mmu04922;mmu04923;mmu04931;mmu04932;mmu04933;mmu04973;mmu05142;mmu05145;mmu05152;mmu05160;mmu05161;mmu05162;mmu05164;mmu05166;mmu05169;mmu05200;mmu05205;mmu05210;mmu05211;mmu05212;mmu05213;mmu05214;mmu05215;mmu05218;mmu05220;mmu05221;mmu05222;mmu05223;mmu05230;mmu05231 |  | Akt2 | azure4 | 1 | -2.04929577464789 |
  | Ep300-mRNA | -0.209 | 0.111 | -0.425 | 0.008 | 0.865 | 0.745 | 1.01 | 0.0774 | 1 | loglinear | Cell Cycle, Cellular Stress, Cytokine Signaling, DNA Damage, Epigenetic Regulation, Inflammatory Signaling, Innate Immune Response, Lipid Metabolism, Notch, Wnt | NS\_MM\_NEUROINFLAM\_V1.0 | Ep300 | mRNA | 0 |  | Wnt;Notch;Lipid Metabolism;Innate Immune Response;Inflammatory Signaling;Epigenetic Regulation;DNA Damage;Cytokine Signaling;Cellular Stress;Cell Cycle | mmu04024;mmu04066;mmu04068;mmu04110;mmu04310;mmu04330;mmu04350;mmu04520;mmu04630;mmu04720;mmu04916;mmu04919;mmu04922;mmu05016;mmu05152;mmu05161;mmu05164;mmu05166;mmu05168;mmu05169;mmu05200;mmu05203;mmu05206;mmu05211;mmu05215 |  | Ep300 | azure4 | 1 | -1.88288288288288 |
  | Crebbp-mRNA | -0.0726 | 0.0406 | -0.152 | 0.00705 | 0.951 | 0.9 | 1 | 0.093 | 1 | loglinear | Cell Cycle, Cellular Stress, Cytokine Signaling, Epigenetic Regulation, Inflammatory Signaling, Innate Immune Response, Lipid Metabolism, Notch, Wnt | NS\_MM\_NEUROINFLAM\_V1.0 | Crebbp | mRNA | 0 |  | Wnt;Notch;Lipid Metabolism;Innate Immune Response;Inflammatory Signaling;Epigenetic Regulation;Cytokine Signaling;Cellular Stress;Cell Cycle | mmu04024;mmu04066;mmu04068;mmu04110;mmu04310;mmu04330;mmu04350;mmu04520;mmu04630;mmu04720;mmu04916;mmu04919;mmu04922;mmu05016;mmu05152;mmu05161;mmu05164;mmu05166;mmu05168;mmu05169;mmu05200;mmu05203;mmu05206;mmu05211;mmu05215 |  | Crebbp | azure4 | 1 | -1.78817733990148 |
  | Akt1-mRNA | 0.177 | 0.107 | -0.0326 | 0.387 | 1.13 | 0.978 | 1.31 | 0.117 | 1 | loglinear | Adaptive Immune Response, Angiogenesis, Apoptosis, Autophagy, Carbohydrate Metabolism, Cellular Stress, Cytokine Signaling, Growth Factor Signaling, Innate Immune Response, Insulin Signaling, Neurons and Neurotransmission, Wnt | NS\_MM\_NEUROINFLAM\_V1.0 | Akt1 | mRNA | 0 |  | Wnt;Neurons and Neurotransmission;Insulin Signaling;Innate Immune Response;Growth Factor Signaling;Cytokine Signaling;Cellular Stress;Carbohydrate Metabolism;Autophagy;Apoptosis;Angiogenesis;Adaptive Immune Response | mmu04010;mmu04012;mmu04014;mmu04015;mmu04022;mmu04024;mmu04062;mmu04066;mmu04068;mmu04071;mmu04072;mmu04150;mmu04151;mmu04152;mmu04210;mmu04211;mmu04213;mmu04261;mmu04370;mmu04380;mmu04510;mmu04530;mmu04550;mmu04611;mmu04620;mmu04630;mmu04660;mmu04662;mmu04664;mmu04666;mmu04668;mmu04722;mmu04725;mmu04728;mmu04910;mmu04914;mmu04915;mmu04917;mmu04919;mmu04920;mmu04922;mmu04923;mmu04931;mmu04932;mmu04933;mmu04973;mmu05142;mmu05145;mmu05152;mmu05160;mmu05161;mmu05162;mmu05164;mmu05166;mmu05169;mmu05200;mmu05205;mmu05210;mmu05211;mmu05212;mmu05213;mmu05214;mmu05215;mmu05218;mmu05220;mmu05221;mmu05222;mmu05223;mmu05230;mmu05231 |  | Akt1 | azure4 | 1 | 1.65420560747664 |
  | Trp53-mRNA | 0.257 | 0.2 | -0.134 | 0.649 | 1.2 | 0.911 | 1.57 | 0.216 | 1 | lm.nb | Apoptosis, Cell Cycle, Cellular Stress, DNA Damage, Growth Factor Signaling, Notch, Wnt | NS\_MM\_NEUROINFLAM\_V1.0 | Trp53 | mRNA | 0 |  | Wnt;Notch;Growth Factor Signaling;DNA Damage;Cellular Stress;Cell Cycle;Apoptosis | mmu04010;mmu04071;mmu04110;mmu04115;mmu04151;mmu04210;mmu04211;mmu04310;mmu04722;mmu04919;mmu05014;mmu05016;mmu05160;mmu05161;mmu05162;mmu05166;mmu05168;mmu05169;mmu05200;mmu05202;mmu05203;mmu05205;mmu05206;mmu05210;mmu05212;mmu05213;mmu05214;mmu05215;mmu05216;mmu05218;mmu05219;mmu05220;mmu05222;mmu05223;mmu05230 |  | Trp53 | azure4 | 1 | 1.285 |
  | Serpinf1-mRNA | -0.234 | 0.196 | -0.619 | 0.151 | 0.85 | 0.651 | 1.11 | 0.251 | 1 | lm.nb | Microglia Function, Wnt | NS\_MM\_NEUROINFLAM\_V1.0 | Serpinf1 | mRNA | 0 |  | Wnt;Microglia Function | mmu04310 |  | Serpinf1 | azure4 | 1 | -1.19387755102041 |
  | Hdac1-mRNA | 0.105 | 0.0891 | -0.0698 | 0.279 | 1.08 | 0.953 | 1.21 | 0.257 | 1 | loglinear | Cell Cycle, Epigenetic Regulation, Growth Factor Signaling, Notch, Wnt | NS\_MM\_NEUROINFLAM\_V1.0 | Hdac1 | mRNA | 0 |  | Wnt;Notch;Growth Factor Signaling;Epigenetic Regulation;Cell Cycle | mmu04110;mmu04213;mmu04330;mmu04919;mmu05016;mmu05031;mmu05034;mmu05169;mmu05200;mmu05202;mmu05203;mmu05220 |  | Hdac1 | azure4 | 1 | 1.17845117845118 |
  | Prkaca-mRNA | -0.0568 | 0.0577 | -0.17 | 0.0563 | 0.961 | 0.889 | 1.04 | 0.34 | 1 | loglinear | Adaptive Immune Response, Angiogenesis, Apoptosis, Autophagy, Cell Cycle, Cytokine Signaling, Growth Factor Signaling, Lipid Metabolism, Neurons and Neurotransmission, Wnt | NS\_MM\_NEUROINFLAM\_V1.0 | Prkaca | mRNA | 0 |  | Wnt;Neurons and Neurotransmission;Lipid Metabolism;Growth Factor Signaling;Cytokine Signaling;Cell Cycle;Autophagy;Apoptosis;Angiogenesis;Adaptive Immune Response | mmu04010;mmu04014;mmu04020;mmu04024;mmu04062;mmu04114;mmu04210;mmu04211;mmu04213;mmu04261;mmu04270;mmu04310;mmu04540;mmu04611;mmu04713;mmu04720;mmu04723;mmu04724;mmu04725;mmu04726;mmu04728;mmu04750;mmu04910;mmu04911;mmu04912;mmu04913;mmu04914;mmu04915;mmu04916;mmu04919;mmu04921;mmu04922;mmu04923;mmu04924;mmu04925;mmu05012;mmu05020;mmu05030;mmu05031;mmu05034;mmu05146;mmu05166;mmu05169;mmu05200;mmu05203;mmu05205;mmu05414 |  | Prkaca | azure4 | 1 | -0.984402079722704 |

  ##### DE Results - Wnt genes - DPI: 1 vs.CTRL

  Table displaying each sample's global significance scores and directed global significance scores as defined in the heatmaps above. The global significance score is calculated as the square root of the mean squared t-statistic for the genes in a gene set, with t-statistics coming from the linear regression underlying our differential expression analysis. The directed global significance score is calculated as the square root of the mean signed squared t-statistic for the genes in a gene set, with t-statistics coming from the linear regression underlying our differential expression analysis.

- **Volcano Plot: DPI: 2 vs.CTRL  
  More Plot Information**


  ##### Volcano Plot: DPI: 2 vs.CTRL

  Volcano plot displaying each gene's -log10(p-value) and log2 fold change for the selected covariate. Highly statistically significant genes fall at the top of the plot, and highly differentially expressed genes fall to either side. Genes within the selected gene set are highlighted in orange. Horizontal lines indicate various False Discovery Rate (FDR) thresholds.
- **DE Results - Wnt genes - DPI: 2 vs.CTRL  
  More Plot Information  Download CSV Data**

  | Probe Label | Log2 fold change | std error (log2) | Lower confidence limit (log2) | Upper confidence limit (log2) | Linear fold change | Lower confidence limit (linear) | Upper confidence limit (linear) | P-value | BY.p.value | method | Gene.sets | Codeset.Name | Probe.Label | Analyte.Type | Is.Control | Control.Type | Probe.Annotation | KEGG.Pathways | Cell.Type | Official.Gene.Name | volcanocol | volcanopch | tstats.all |
  | --- | --- | --- | --- | --- | --- | --- | --- | --- | --- | --- | --- | --- | --- | --- | --- | --- | --- | --- | --- | --- | --- | --- | --- |
  | Psmb8-mRNA | 2.23 | 0.352 | 1.54 | 2.92 | 4.7 | 2.91 | 7.59 | 9.89e-06 | 0.000997 | lm.nb | Adaptive Immune Response, Angiogenesis, Apoptosis, Astrocyte Function, Cell Cycle, Cytokine Signaling, Growth Factor Signaling, Inflammatory Signaling, Insulin Signaling, Microglia Function, NF-kB, Wnt | NS\_MM\_NEUROINFLAM\_V1.0 | Psmb8 | mRNA | 0 |  | Wnt;NF-kB;Microglia Function;Insulin Signaling;Inflammatory Signaling;Growth Factor Signaling;Cytokine Signaling;Cell Cycle;Astrocyte Function;Apoptosis;Angiogenesis;Adaptive Immune Response |  |  | Psmb8 | slateblue1 | 16 | 6.33522727272727 |
  | Ppp3r1-mRNA | -0.334 | 0.0577 | -0.447 | -0.221 | 0.793 | 0.734 | 0.858 | 2.79e-05 | 0.00214 | loglinear | Adaptive Immune Response, Apoptosis, Growth Factor Signaling, Innate Immune Response, Wnt | NS\_MM\_NEUROINFLAM\_V1.0 | Ppp3r1 | mRNA | 0 |  | Wnt;Innate Immune Response;Growth Factor Signaling;Apoptosis;Adaptive Immune Response | mmu04010;mmu04020;mmu04022;mmu04114;mmu04210;mmu04310;mmu04360;mmu04370;mmu04380;mmu04650;mmu04660;mmu04662;mmu04720;mmu04724;mmu04921;mmu04922;mmu04924;mmu05010;mmu05014;mmu05031;mmu05152;mmu05166 |  | Ppp3r1 | slateblue1 | 16 | -5.78856152512998 |
  | Ppp3cb-mRNA | -0.554 | 0.098 | -0.747 | -0.362 | 0.681 | 0.596 | 0.778 | 3.56e-05 | 0.00246 | lm.nb | Adaptive Immune Response, Apoptosis, Growth Factor Signaling, Innate Immune Response, Wnt | NS\_MM\_NEUROINFLAM\_V1.0 | Ppp3cb | mRNA | 0 |  | Wnt;Innate Immune Response;Growth Factor Signaling;Apoptosis;Adaptive Immune Response | mmu04010;mmu04020;mmu04022;mmu04114;mmu04210;mmu04310;mmu04360;mmu04370;mmu04380;mmu04650;mmu04660;mmu04662;mmu04720;mmu04724;mmu04728;mmu04921;mmu04922;mmu04924;mmu05010;mmu05014;mmu05031;mmu05152;mmu05166 |  | Ppp3cb | slateblue1 | 16 | -5.6530612244898 |
  | Apc-mRNA | -0.322 | 0.0593 | -0.438 | -0.206 | 0.8 | 0.738 | 0.867 | 5.61e-05 | 0.00362 | loglinear | Apoptosis, Astrocyte Function, Wnt | NS\_MM\_NEUROINFLAM\_V1.0 | Apc | mRNA | 0 |  | Wnt;Astrocyte Function;Apoptosis | mmu04310;mmu04390;mmu04550;mmu04810;mmu05166;mmu05200;mmu05206;mmu05210;mmu05213 |  | Apc | slateblue1 | 16 | -5.43001686340641 |
  | Prkacb-mRNA | -0.322 | 0.0629 | -0.445 | -0.199 | 0.8 | 0.735 | 0.871 | 0.000104 | 0.00562 | loglinear | Adaptive Immune Response, Angiogenesis, Apoptosis, Autophagy, Growth Factor Signaling, Lipid Metabolism, Neurons and Neurotransmission, Wnt | NS\_MM\_NEUROINFLAM\_V1.0 | Prkacb | mRNA | 0 |  | Wnt;Neurons and Neurotransmission;Lipid Metabolism;Growth Factor Signaling;Autophagy;Apoptosis;Angiogenesis;Adaptive Immune Response | mmu04010;mmu04014;mmu04020;mmu04024;mmu04062;mmu04114;mmu04210;mmu04211;mmu04213;mmu04261;mmu04270;mmu04310;mmu04540;mmu04611;mmu04713;mmu04720;mmu04723;mmu04724;mmu04725;mmu04726;mmu04728;mmu04750;mmu04910;mmu04911;mmu04912;mmu04913;mmu04914;mmu04915;mmu04916;mmu04919;mmu04921;mmu04922;mmu04923;mmu04924;mmu04925;mmu05012;mmu05020;mmu05030;mmu05031;mmu05146;mmu05166;mmu05169;mmu05200;mmu05203;mmu05205;mmu05414 |  | Prkacb | slateblue1 | 16 | -5.11923688394277 |
  | Mapk10-mRNA | -0.673 | 0.138 | -0.943 | -0.402 | 0.627 | 0.52 | 0.757 | 0.000172 | 0.0082 | lm.nb | Adaptive Immune Response, Apoptosis, Autophagy, Cellular Stress, Growth Factor Signaling, Innate Immune Response, Wnt | NS\_MM\_NEUROINFLAM\_V1.0 | Mapk10 | mRNA | 0 |  | Wnt;Innate Immune Response;Growth Factor Signaling;Cellular Stress;Autophagy;Apoptosis;Adaptive Immune Response | mmu04010;mmu04012;mmu04014;mmu04024;mmu04068;mmu04071;mmu04141;mmu04310;mmu04380;mmu04510;mmu04620;mmu04621;mmu04622;mmu04664;mmu04668;mmu04722;mmu04723;mmu04728;mmu04750;mmu04910;mmu04912;mmu04914;mmu04917;mmu04920;mmu04930;mmu04931;mmu04932;mmu04933;mmu05132;mmu05133;mmu05142;mmu05145;mmu05152;mmu05160;mmu05161;mmu05164;mmu05168;mmu05169;mmu05200;mmu05210;mmu05212;mmu05231 |  | Mapk10 | slateblue1 | 16 | -4.8768115942029 |
  | Tle3-mRNA | 0.592 | 0.124 | 0.349 | 0.836 | 1.51 | 1.27 | 1.78 | 0.000207 | 0.00902 | loglinear | Microglia Function, Notch, Wnt | NS\_MM\_NEUROINFLAM\_V1.0 | Tle3 | mRNA | 0 |  | Wnt;Notch;Microglia Function |  |  | Tle3 | slateblue1 | 16 | 4.7741935483871 |
  | Akt2-mRNA | -0.592 | 0.142 | -0.869 | -0.314 | 0.664 | 0.547 | 0.804 | 0.000713 | 0.022 | loglinear | Adaptive Immune Response, Angiogenesis, Apoptosis, Autophagy, Carbohydrate Metabolism, Cytokine Signaling, Growth Factor Signaling, Innate Immune Response, Insulin Signaling, Neurons and Neurotransmission, Wnt | NS\_MM\_NEUROINFLAM\_V1.0 | Akt2 | mRNA | 0 |  | Wnt;Neurons and Neurotransmission;Insulin Signaling;Innate Immune Response;Growth Factor Signaling;Cytokine Signaling;Carbohydrate Metabolism;Autophagy;Apoptosis;Angiogenesis;Adaptive Immune Response | mmu04010;mmu04012;mmu04014;mmu04015;mmu04022;mmu04024;mmu04062;mmu04066;mmu04068;mmu04071;mmu04072;mmu04150;mmu04151;mmu04152;mmu04210;mmu04211;mmu04213;mmu04261;mmu04370;mmu04380;mmu04510;mmu04530;mmu04550;mmu04611;mmu04620;mmu04630;mmu04660;mmu04662;mmu04664;mmu04666;mmu04668;mmu04722;mmu04725;mmu04728;mmu04910;mmu04914;mmu04915;mmu04917;mmu04919;mmu04920;mmu04922;mmu04923;mmu04931;mmu04932;mmu04933;mmu04973;mmu05142;mmu05145;mmu05152;mmu05160;mmu05161;mmu05162;mmu05164;mmu05166;mmu05169;mmu05200;mmu05205;mmu05210;mmu05211;mmu05212;mmu05213;mmu05214;mmu05215;mmu05218;mmu05220;mmu05221;mmu05222;mmu05223;mmu05230;mmu05231 |  | Akt2 | slateblue2 | 16 | -4.16901408450704 |
  | Ash2l-mRNA | -0.451 | 0.109 | -0.665 | -0.237 | 0.731 | 0.631 | 0.848 | 0.000784 | 0.0233 | loglinear | Epigenetic Regulation, Wnt | NS\_MM\_NEUROINFLAM\_V1.0 | Ash2l | mRNA | 0 |  | Wnt;Epigenetic Regulation |  |  | Ash2l | slateblue2 | 16 | -4.13761467889908 |
  | Sox4-mRNA | -0.822 | 0.2 | -1.21 | -0.429 | 0.566 | 0.431 | 0.743 | 0.000839 | 0.0239 | lm.nb | Microglia Function, Wnt | NS\_MM\_NEUROINFLAM\_V1.0 | Sox4 | mRNA | 0 |  | Wnt;Microglia Function |  |  | Sox4 | slateblue2 | 16 | -4.11 |
  | Rhoa-mRNA | -1.04 | 0.261 | -1.55 | -0.526 | 0.487 | 0.342 | 0.695 | 0.00109 | 0.0288 | lm.nb | Angiogenesis, Autophagy, Growth Factor Signaling, Wnt | NS\_MM\_NEUROINFLAM\_V1.0 | Rhoa | mRNA | 0 |  | Wnt;Growth Factor Signaling;Autophagy;Angiogenesis | mmu04014;mmu04015;mmu04022;mmu04024;mmu04062;mmu04071;mmu04072;mmu04144;mmu04270;mmu04310;mmu04350;mmu04360;mmu04510;mmu04520;mmu04530;mmu04611;mmu04660;mmu04670;mmu04722;mmu04810;mmu04921;mmu05100;mmu05133;mmu05152;mmu05200;mmu05203;mmu05205;mmu05206;mmu05210 |  | Rhoa | slateblue2 | 16 | -3.98467432950192 |
  | H2afx-mRNA | -0.214 | 0.0562 | -0.325 | -0.104 | 0.862 | 0.799 | 0.93 | 0.00154 | 0.0372 | loglinear | Cell Cycle, Cellular Stress, DNA Damage, Epigenetic Regulation, Wnt | NS\_MM\_NEUROINFLAM\_V1.0 | H2afx | mRNA | 0 |  | Wnt;Epigenetic Regulation;DNA Damage;Cellular Stress;Cell Cycle | mmu05034;mmu05322 |  | H2afx | slateblue2 | 16 | -3.80782918149466 |
  | Xiap-mRNA | -0.58 | 0.155 | -0.884 | -0.276 | 0.669 | 0.542 | 0.826 | 0.0018 | 0.0414 | lm.nb | Apoptosis, Innate Immune Response, NF-kB, Wnt | NS\_MM\_NEUROINFLAM\_V1.0 | Xiap | mRNA | 0 |  | Wnt;NF-kB;Innate Immune Response;Apoptosis | mmu04064;mmu04120;mmu04210;mmu04510;mmu05145;mmu05166;mmu05200;mmu05222 |  | Xiap | slateblue2 | 16 | -3.74193548387097 |
  | Rac1-mRNA | -0.955 | 0.256 | -1.46 | -0.452 | 0.516 | 0.364 | 0.731 | 0.00184 | 0.0417 | lm.nb | Adaptive Immune Response, Angiogenesis, Autophagy, Growth Factor Signaling, Innate Immune Response, Neurons and Neurotransmission, Wnt | NS\_MM\_NEUROINFLAM\_V1.0 | Rac1 | mRNA | 0 |  | Wnt;Neurons and Neurotransmission;Innate Immune Response;Growth Factor Signaling;Autophagy;Angiogenesis;Adaptive Immune Response | mmu04010;mmu04014;mmu04015;mmu04024;mmu04062;mmu04071;mmu04145;mmu04151;mmu04310;mmu04360;mmu04370;mmu04380;mmu04510;mmu04520;mmu04620;mmu04650;mmu04662;mmu04664;mmu04666;mmu04670;mmu04722;mmu04810;mmu04932;mmu04933;mmu05014;mmu05100;mmu05132;mmu05200;mmu05203;mmu05205;mmu05210;mmu05211;mmu05212;mmu05231;mmu05416 |  | Rac1 | slateblue2 | 16 | -3.73046875 |
  | Ep300-mRNA | -0.33 | 0.111 | -0.547 | -0.114 | 0.795 | 0.684 | 0.924 | 0.0087 | 0.144 | loglinear | Cell Cycle, Cellular Stress, Cytokine Signaling, DNA Damage, Epigenetic Regulation, Inflammatory Signaling, Innate Immune Response, Lipid Metabolism, Notch, Wnt | NS\_MM\_NEUROINFLAM\_V1.0 | Ep300 | mRNA | 0 |  | Wnt;Notch;Lipid Metabolism;Innate Immune Response;Inflammatory Signaling;Epigenetic Regulation;DNA Damage;Cytokine Signaling;Cellular Stress;Cell Cycle | mmu04024;mmu04066;mmu04068;mmu04110;mmu04310;mmu04330;mmu04350;mmu04520;mmu04630;mmu04720;mmu04916;mmu04919;mmu04922;mmu05016;mmu05152;mmu05161;mmu05164;mmu05166;mmu05168;mmu05169;mmu05200;mmu05203;mmu05206;mmu05211;mmu05215 |  | Ep300 | slateblue4 | 16 | -2.97297297297297 |
  | Prkaca-mRNA | -0.166 | 0.0577 | -0.279 | -0.0525 | 0.892 | 0.824 | 0.964 | 0.0111 | 0.179 | loglinear | Adaptive Immune Response, Angiogenesis, Apoptosis, Autophagy, Cell Cycle, Cytokine Signaling, Growth Factor Signaling, Lipid Metabolism, Neurons and Neurotransmission, Wnt | NS\_MM\_NEUROINFLAM\_V1.0 | Prkaca | mRNA | 0 |  | Wnt;Neurons and Neurotransmission;Lipid Metabolism;Growth Factor Signaling;Cytokine Signaling;Cell Cycle;Autophagy;Apoptosis;Angiogenesis;Adaptive Immune Response | mmu04010;mmu04014;mmu04020;mmu04024;mmu04062;mmu04114;mmu04210;mmu04211;mmu04213;mmu04261;mmu04270;mmu04310;mmu04540;mmu04611;mmu04713;mmu04720;mmu04723;mmu04724;mmu04725;mmu04726;mmu04728;mmu04750;mmu04910;mmu04911;mmu04912;mmu04913;mmu04914;mmu04915;mmu04916;mmu04919;mmu04921;mmu04922;mmu04923;mmu04924;mmu04925;mmu05012;mmu05020;mmu05030;mmu05031;mmu05034;mmu05146;mmu05166;mmu05169;mmu05200;mmu05203;mmu05205;mmu05414 |  | Prkaca | slateblue4 | 16 | -2.87694974003466 |
  | Sox9-mRNA | -0.342 | 0.128 | -0.593 | -0.0906 | 0.789 | 0.663 | 0.939 | 0.0169 | 0.241 | lm.nb | Astrocyte Function, Wnt | NS\_MM\_NEUROINFLAM\_V1.0 | Sox9 | mRNA | 0 |  | Wnt;Astrocyte Function | mmu04024 | Astrocytes | Sox9 | slateblue4 | 16 | -2.671875 |
  | Ago4-mRNA | -0.969 | 0.4 | -1.75 | -0.185 | 0.511 | 0.297 | 0.88 | 0.0277 | 0.356 | lm.nb | Adaptive Immune Response, Cellular Stress, Epigenetic Regulation, Growth Factor Signaling, Notch, Wnt | NS\_MM\_NEUROINFLAM\_V1.0 | Ago4 | mRNA | 0 |  | Wnt;Notch;Growth Factor Signaling;Epigenetic Regulation;Cellular Stress;Adaptive Immune Response |  |  | Ago4 | slateblue4 | 16 | -2.4225 |
  | Serpinf1-mRNA | -0.409 | 0.199 | -0.798 | -0.0195 | 0.753 | 0.575 | 0.987 | 0.0562 | 0.655 | lm.nb | Microglia Function, Wnt | NS\_MM\_NEUROINFLAM\_V1.0 | Serpinf1 | mRNA | 0 |  | Wnt;Microglia Function | mmu04310 |  | Serpinf1 | azure4 | 1 | -2.05527638190955 |
  | Jun-mRNA | 0.242 | 0.12 | 0.0077 | 0.477 | 1.18 | 1.01 | 1.39 | 0.06 | 0.68 | lm.nb | Adaptive Immune Response, Apoptosis, Cellular Stress, Growth Factor Signaling, Innate Immune Response, Notch, Wnt | NS\_MM\_NEUROINFLAM\_V1.0 | Jun | mRNA | 0 |  | Wnt;Notch;Innate Immune Response;Growth Factor Signaling;Cellular Stress;Apoptosis;Adaptive Immune Response | mmu04010;mmu04012;mmu04024;mmu04310;mmu04380;mmu04510;mmu04620;mmu04660;mmu04662;mmu04668;mmu04722;mmu04912;mmu04915;mmu04921;mmu04932;mmu04933;mmu05030;mmu05031;mmu05132;mmu05133;mmu05140;mmu05142;mmu05161;mmu05164;mmu05166;mmu05168;mmu05169;mmu05200;mmu05203;mmu05210;mmu05211;mmu05231;mmu05321;mmu05323 |  | Jun | azure4 | 1 | 2.01666666666667 |

  ##### DE Results - Wnt genes - DPI: 2 vs.CTRL

  Table displaying each sample's global significance scores and directed global significance scores as defined in the heatmaps above. The global significance score is calculated as the square root of the mean squared t-statistic for the genes in a gene set, with t-statistics coming from the linear regression underlying our differential expression analysis. The directed global significance score is calculated as the square root of the mean signed squared t-statistic for the genes in a gene set, with t-statistics coming from the linear regression underlying our differential expression analysis.

- **Volcano Plot: DPI: 3 vs.CTRL  
  More Plot Information**


  ##### Volcano Plot: DPI: 3 vs.CTRL

  Volcano plot displaying each gene's -log10(p-value) and log2 fold change for the selected covariate. Highly statistically significant genes fall at the top of the plot, and highly differentially expressed genes fall to either side. Genes within the selected gene set are highlighted in orange. Horizontal lines indicate various False Discovery Rate (FDR) thresholds.
- **DE Results - Wnt genes - DPI: 3 vs.CTRL  
  More Plot Information  Download CSV Data**

  | Probe Label | Log2 fold change | std error (log2) | Lower confidence limit (log2) | Upper confidence limit (log2) | Linear fold change | Lower confidence limit (linear) | Upper confidence limit (linear) | P-value | BY.p.value | method | Gene.sets | Codeset.Name | Probe.Label | Analyte.Type | Is.Control | Control.Type | Probe.Annotation | KEGG.Pathways | Cell.Type | Official.Gene.Name | volcanocol | volcanopch | tstats.all |
  | --- | --- | --- | --- | --- | --- | --- | --- | --- | --- | --- | --- | --- | --- | --- | --- | --- | --- | --- | --- | --- | --- | --- | --- |
  | Smarca4-mRNA | -0.495 | 0.046 | -0.585 | -0.405 | 0.71 | 0.667 | 0.755 | 9.76e-09 | 3.12e-06 | loglinear | Epigenetic Regulation, Wnt | NS\_MM\_NEUROINFLAM\_V1.0 | Smarca4 | mRNA | 0 |  | Wnt;Epigenetic Regulation |  |  | Smarca4 | slateblue1 | 16 | -10.7608695652174 |
  | Psmb8-mRNA | 2.99 | 0.346 | 2.31 | 3.67 | 7.95 | 4.97 | 12.7 | 1.99e-07 | 3.2e-05 | lm.nb | Adaptive Immune Response, Angiogenesis, Apoptosis, Astrocyte Function, Cell Cycle, Cytokine Signaling, Growth Factor Signaling, Inflammatory Signaling, Insulin Signaling, Microglia Function, NF-kB, Wnt | NS\_MM\_NEUROINFLAM\_V1.0 | Psmb8 | mRNA | 0 |  | Wnt;NF-kB;Microglia Function;Insulin Signaling;Inflammatory Signaling;Growth Factor Signaling;Cytokine Signaling;Cell Cycle;Astrocyte Function;Apoptosis;Angiogenesis;Adaptive Immune Response |  |  | Psmb8 | slateblue1 | 16 | 8.64161849710983 |
  | Tle3-mRNA | 0.865 | 0.124 | 0.622 | 1.11 | 1.82 | 1.54 | 2.16 | 3.15e-06 | 0.000271 | loglinear | Microglia Function, Notch, Wnt | NS\_MM\_NEUROINFLAM\_V1.0 | Tle3 | mRNA | 0 |  | Wnt;Notch;Microglia Function |  |  | Tle3 | slateblue1 | 16 | 6.9758064516129 |
  | Ash2l-mRNA | -0.668 | 0.109 | -0.882 | -0.454 | 0.629 | 0.542 | 0.73 | 1.48e-05 | 0.000921 | loglinear | Epigenetic Regulation, Wnt | NS\_MM\_NEUROINFLAM\_V1.0 | Ash2l | mRNA | 0 |  | Wnt;Epigenetic Regulation |  |  | Ash2l | slateblue1 | 16 | -6.12844036697248 |
  | Akt2-mRNA | -0.766 | 0.142 | -1.04 | -0.488 | 0.588 | 0.485 | 0.713 | 5.85e-05 | 0.00265 | loglinear | Adaptive Immune Response, Angiogenesis, Apoptosis, Autophagy, Carbohydrate Metabolism, Cytokine Signaling, Growth Factor Signaling, Innate Immune Response, Insulin Signaling, Neurons and Neurotransmission, Wnt | NS\_MM\_NEUROINFLAM\_V1.0 | Akt2 | mRNA | 0 |  | Wnt;Neurons and Neurotransmission;Insulin Signaling;Innate Immune Response;Growth Factor Signaling;Cytokine Signaling;Carbohydrate Metabolism;Autophagy;Apoptosis;Angiogenesis;Adaptive Immune Response | mmu04010;mmu04012;mmu04014;mmu04015;mmu04022;mmu04024;mmu04062;mmu04066;mmu04068;mmu04071;mmu04072;mmu04150;mmu04151;mmu04152;mmu04210;mmu04211;mmu04213;mmu04261;mmu04370;mmu04380;mmu04510;mmu04530;mmu04550;mmu04611;mmu04620;mmu04630;mmu04660;mmu04662;mmu04664;mmu04666;mmu04668;mmu04722;mmu04725;mmu04728;mmu04910;mmu04914;mmu04915;mmu04917;mmu04919;mmu04920;mmu04922;mmu04923;mmu04931;mmu04932;mmu04933;mmu04973;mmu05142;mmu05145;mmu05152;mmu05160;mmu05161;mmu05162;mmu05164;mmu05166;mmu05169;mmu05200;mmu05205;mmu05210;mmu05211;mmu05212;mmu05213;mmu05214;mmu05215;mmu05218;mmu05220;mmu05221;mmu05222;mmu05223;mmu05230;mmu05231 |  | Akt2 | slateblue1 | 16 | -5.3943661971831 |
  | Ago4-mRNA | -2.72 | 0.514 | -3.72 | -1.71 | 0.152 | 0.0757 | 0.306 | 7.46e-05 | 0.00321 | lm.nb | Adaptive Immune Response, Cellular Stress, Epigenetic Regulation, Growth Factor Signaling, Notch, Wnt | NS\_MM\_NEUROINFLAM\_V1.0 | Ago4 | mRNA | 0 |  | Wnt;Notch;Growth Factor Signaling;Epigenetic Regulation;Cellular Stress;Adaptive Immune Response |  |  | Ago4 | slateblue1 | 16 | -5.29182879377432 |
  | Mapk10-mRNA | -0.571 | 0.138 | -0.842 | -0.3 | 0.673 | 0.558 | 0.812 | 0.000782 | 0.0231 | lm.nb | Adaptive Immune Response, Apoptosis, Autophagy, Cellular Stress, Growth Factor Signaling, Innate Immune Response, Wnt | NS\_MM\_NEUROINFLAM\_V1.0 | Mapk10 | mRNA | 0 |  | Wnt;Innate Immune Response;Growth Factor Signaling;Cellular Stress;Autophagy;Apoptosis;Adaptive Immune Response | mmu04010;mmu04012;mmu04014;mmu04024;mmu04068;mmu04071;mmu04141;mmu04310;mmu04380;mmu04510;mmu04620;mmu04621;mmu04622;mmu04664;mmu04668;mmu04722;mmu04723;mmu04728;mmu04750;mmu04910;mmu04912;mmu04914;mmu04917;mmu04920;mmu04930;mmu04931;mmu04932;mmu04933;mmu05132;mmu05133;mmu05142;mmu05145;mmu05152;mmu05160;mmu05161;mmu05164;mmu05168;mmu05169;mmu05200;mmu05210;mmu05212;mmu05231 |  | Mapk10 | slateblue2 | 16 | -4.13768115942029 |
  | Apc-mRNA | -0.238 | 0.0593 | -0.354 | -0.122 | 0.848 | 0.782 | 0.919 | 0.00101 | 0.0279 | loglinear | Apoptosis, Astrocyte Function, Wnt | NS\_MM\_NEUROINFLAM\_V1.0 | Apc | mRNA | 0 |  | Wnt;Astrocyte Function;Apoptosis | mmu04310;mmu04390;mmu04550;mmu04810;mmu05166;mmu05200;mmu05206;mmu05210;mmu05213 |  | Apc | slateblue2 | 16 | -4.01349072512648 |
  | Sox4-mRNA | 0.575 | 0.19 | 0.202 | 0.947 | 1.49 | 1.15 | 1.93 | 0.00807 | 0.152 | lm.nb | Microglia Function, Wnt | NS\_MM\_NEUROINFLAM\_V1.0 | Sox4 | mRNA | 0 |  | Wnt;Microglia Function |  |  | Sox4 | slateblue4 | 16 | 3.02631578947368 |
  | Ppp3cb-mRNA | -0.263 | 0.0978 | -0.454 | -0.0711 | 0.834 | 0.73 | 0.952 | 0.0162 | 0.273 | lm.nb | Adaptive Immune Response, Apoptosis, Growth Factor Signaling, Innate Immune Response, Wnt | NS\_MM\_NEUROINFLAM\_V1.0 | Ppp3cb | mRNA | 0 |  | Wnt;Innate Immune Response;Growth Factor Signaling;Apoptosis;Adaptive Immune Response | mmu04010;mmu04020;mmu04022;mmu04114;mmu04210;mmu04310;mmu04360;mmu04370;mmu04380;mmu04650;mmu04660;mmu04662;mmu04720;mmu04724;mmu04728;mmu04921;mmu04922;mmu04924;mmu05010;mmu05014;mmu05031;mmu05152;mmu05166 |  | Ppp3cb | slateblue4 | 16 | -2.68916155419223 |
  | Prkaca-mRNA | -0.149 | 0.0577 | -0.262 | -0.0355 | 0.902 | 0.834 | 0.976 | 0.0203 | 0.322 | loglinear | Adaptive Immune Response, Angiogenesis, Apoptosis, Autophagy, Cell Cycle, Cytokine Signaling, Growth Factor Signaling, Lipid Metabolism, Neurons and Neurotransmission, Wnt | NS\_MM\_NEUROINFLAM\_V1.0 | Prkaca | mRNA | 0 |  | Wnt;Neurons and Neurotransmission;Lipid Metabolism;Growth Factor Signaling;Cytokine Signaling;Cell Cycle;Autophagy;Apoptosis;Angiogenesis;Adaptive Immune Response | mmu04010;mmu04014;mmu04020;mmu04024;mmu04062;mmu04114;mmu04210;mmu04211;mmu04213;mmu04261;mmu04270;mmu04310;mmu04540;mmu04611;mmu04713;mmu04720;mmu04723;mmu04724;mmu04725;mmu04726;mmu04728;mmu04750;mmu04910;mmu04911;mmu04912;mmu04913;mmu04914;mmu04915;mmu04916;mmu04919;mmu04921;mmu04922;mmu04923;mmu04924;mmu04925;mmu05012;mmu05020;mmu05030;mmu05031;mmu05034;mmu05146;mmu05166;mmu05169;mmu05200;mmu05203;mmu05205;mmu05414 |  | Prkaca | slateblue4 | 16 | -2.58232235701906 |
  | Myc-mRNA | 0.677 | 0.331 | 0.0278 | 1.33 | 1.6 | 1.02 | 2.51 | 0.0578 | 0.734 | lm.nb | Apoptosis, Cell Cycle, Cytokine Signaling, Growth Factor Signaling, Notch, Wnt | NS\_MM\_NEUROINFLAM\_V1.0 | Myc | mRNA | 0 |  | Wnt;Notch;Growth Factor Signaling;Cytokine Signaling;Cell Cycle;Apoptosis | mmu04010;mmu04012;mmu04110;mmu04151;mmu04310;mmu04350;mmu04390;mmu04550;mmu04630;mmu04919;mmu05161;mmu05166;mmu05169;mmu05200;mmu05202;mmu05205;mmu05206;mmu05210;mmu05213;mmu05216;mmu05219;mmu05220;mmu05221;mmu05222;mmu05230 |  | Myc | azure4 | 1 | 2.04531722054381 |
  | Xiap-mRNA | -0.299 | 0.155 | -0.602 | 0.00504 | 0.813 | 0.659 | 1 | 0.0719 | 0.896 | lm.nb | Apoptosis, Innate Immune Response, NF-kB, Wnt | NS\_MM\_NEUROINFLAM\_V1.0 | Xiap | mRNA | 0 |  | Wnt;NF-kB;Innate Immune Response;Apoptosis | mmu04064;mmu04120;mmu04210;mmu04510;mmu05145;mmu05166;mmu05200;mmu05222 |  | Xiap | azure4 | 1 | -1.92903225806452 |
  | Jun-mRNA | -0.217 | 0.121 | -0.455 | 0.0202 | 0.86 | 0.73 | 1.01 | 0.0918 | 1 | lm.nb | Adaptive Immune Response, Apoptosis, Cellular Stress, Growth Factor Signaling, Innate Immune Response, Notch, Wnt | NS\_MM\_NEUROINFLAM\_V1.0 | Jun | mRNA | 0 |  | Wnt;Notch;Innate Immune Response;Growth Factor Signaling;Cellular Stress;Apoptosis;Adaptive Immune Response | mmu04010;mmu04012;mmu04024;mmu04310;mmu04380;mmu04510;mmu04620;mmu04660;mmu04662;mmu04668;mmu04722;mmu04912;mmu04915;mmu04921;mmu04932;mmu04933;mmu05030;mmu05031;mmu05132;mmu05133;mmu05140;mmu05142;mmu05161;mmu05164;mmu05166;mmu05168;mmu05169;mmu05200;mmu05203;mmu05210;mmu05211;mmu05231;mmu05321;mmu05323 |  | Jun | azure4 | 1 | -1.79338842975207 |
  | Sox9-mRNA | 0.217 | 0.124 | -0.0267 | 0.46 | 1.16 | 0.982 | 1.38 | 0.1 | 1 | lm.nb | Astrocyte Function, Wnt | NS\_MM\_NEUROINFLAM\_V1.0 | Sox9 | mRNA | 0 |  | Wnt;Astrocyte Function | mmu04024 | Astrocytes | Sox9 | azure4 | 1 | 1.75 |
  | Prkacb-mRNA | -0.104 | 0.0629 | -0.227 | 0.0196 | 0.931 | 0.854 | 1.01 | 0.119 | 1 | loglinear | Adaptive Immune Response, Angiogenesis, Apoptosis, Autophagy, Growth Factor Signaling, Lipid Metabolism, Neurons and Neurotransmission, Wnt | NS\_MM\_NEUROINFLAM\_V1.0 | Prkacb | mRNA | 0 |  | Wnt;Neurons and Neurotransmission;Lipid Metabolism;Growth Factor Signaling;Autophagy;Apoptosis;Angiogenesis;Adaptive Immune Response | mmu04010;mmu04014;mmu04020;mmu04024;mmu04062;mmu04114;mmu04210;mmu04211;mmu04213;mmu04261;mmu04270;mmu04310;mmu04540;mmu04611;mmu04713;mmu04720;mmu04723;mmu04724;mmu04725;mmu04726;mmu04728;mmu04750;mmu04910;mmu04911;mmu04912;mmu04913;mmu04914;mmu04915;mmu04916;mmu04919;mmu04921;mmu04922;mmu04923;mmu04924;mmu04925;mmu05012;mmu05020;mmu05030;mmu05031;mmu05146;mmu05166;mmu05169;mmu05200;mmu05203;mmu05205;mmu05414 |  | Prkacb | azure4 | 1 | -1.65341812400636 |
  | Ppp3r1-mRNA | -0.0936 | 0.0577 | -0.207 | 0.0195 | 0.937 | 0.867 | 1.01 | 0.124 | 1 | loglinear | Adaptive Immune Response, Apoptosis, Growth Factor Signaling, Innate Immune Response, Wnt | NS\_MM\_NEUROINFLAM\_V1.0 | Ppp3r1 | mRNA | 0 |  | Wnt;Innate Immune Response;Growth Factor Signaling;Apoptosis;Adaptive Immune Response | mmu04010;mmu04020;mmu04022;mmu04114;mmu04210;mmu04310;mmu04360;mmu04370;mmu04380;mmu04650;mmu04660;mmu04662;mmu04720;mmu04724;mmu04921;mmu04922;mmu04924;mmu05010;mmu05014;mmu05031;mmu05152;mmu05166 |  | Ppp3r1 | azure4 | 1 | -1.62218370883882 |
  | Rac1-mRNA | -0.405 | 0.256 | -0.907 | 0.0972 | 0.755 | 0.533 | 1.07 | 0.134 | 1 | lm.nb | Adaptive Immune Response, Angiogenesis, Autophagy, Growth Factor Signaling, Innate Immune Response, Neurons and Neurotransmission, Wnt | NS\_MM\_NEUROINFLAM\_V1.0 | Rac1 | mRNA | 0 |  | Wnt;Neurons and Neurotransmission;Innate Immune Response;Growth Factor Signaling;Autophagy;Angiogenesis;Adaptive Immune Response | mmu04010;mmu04014;mmu04015;mmu04024;mmu04062;mmu04071;mmu04145;mmu04151;mmu04310;mmu04360;mmu04370;mmu04380;mmu04510;mmu04520;mmu04620;mmu04650;mmu04662;mmu04664;mmu04666;mmu04670;mmu04722;mmu04810;mmu04932;mmu04933;mmu05014;mmu05100;mmu05132;mmu05200;mmu05203;mmu05205;mmu05210;mmu05211;mmu05212;mmu05231;mmu05416 |  | Rac1 | azure4 | 1 | -1.58203125 |
  | Rhoa-mRNA | -0.377 | 0.26 | -0.888 | 0.133 | 0.77 | 0.54 | 1.1 | 0.166 | 1 | lm.nb | Angiogenesis, Autophagy, Growth Factor Signaling, Wnt | NS\_MM\_NEUROINFLAM\_V1.0 | Rhoa | mRNA | 0 |  | Wnt;Growth Factor Signaling;Autophagy;Angiogenesis | mmu04014;mmu04015;mmu04022;mmu04024;mmu04062;mmu04071;mmu04072;mmu04144;mmu04270;mmu04310;mmu04350;mmu04360;mmu04510;mmu04520;mmu04530;mmu04611;mmu04660;mmu04670;mmu04722;mmu04810;mmu04921;mmu05100;mmu05133;mmu05152;mmu05200;mmu05203;mmu05205;mmu05206;mmu05210 |  | Rhoa | azure4 | 1 | -1.45 |
  | Hdac1-mRNA | -0.128 | 0.0891 | -0.303 | 0.0462 | 0.915 | 0.811 | 1.03 | 0.169 | 1 | loglinear | Cell Cycle, Epigenetic Regulation, Growth Factor Signaling, Notch, Wnt | NS\_MM\_NEUROINFLAM\_V1.0 | Hdac1 | mRNA | 0 |  | Wnt;Notch;Growth Factor Signaling;Epigenetic Regulation;Cell Cycle | mmu04110;mmu04213;mmu04330;mmu04919;mmu05016;mmu05031;mmu05034;mmu05169;mmu05200;mmu05202;mmu05203;mmu05220 |  | Hdac1 | azure4 | 1 | -1.43658810325477 |

  ##### DE Results - Wnt genes - DPI: 3 vs.CTRL

  Table displaying each sample's global significance scores and directed global significance scores as defined in the heatmaps above. The global significance score is calculated as the square root of the mean squared t-statistic for the genes in a gene set, with t-statistics coming from the linear regression underlying our differential expression analysis. The directed global significance score is calculated as the square root of the mean signed squared t-statistic for the genes in a gene set, with t-statistics coming from the linear regression underlying our differential expression analysis.

Pathview is used to overlay differential expression results on KEGG pathways

DPI:
- DPI: differential expression in 1 vs. baseline of CTRL
- DPI: differential expression in 2 vs. baseline of CTRL
- DPI: differential expression in 3 vs. baseline of CTRL

Pathways:

- Influenza A
- Hepatitis B
- TNF signaling pathway
- Pathways in cancer
- Tuberculosis
- Osteoclast differentiation
- MAPK signaling pathway
- Toll-like receptor signaling pathway
- HTLV-I infection
- Apoptosis
- PI3K-Akt signaling pathway
- Chagas disease (American trypanosomiasis)
- Herpes simplex infection
- Hepatitis C
- Epstein-Barr virus infection
- NF-kappa B signaling pathway
- Measles
- Ras signaling pathway
- Cytokine-cytokine receptor interaction
- Viral carcinogenesis

- **More Plot Information**


  For each KEGG pathway, genes within the panel are mapped to the pathway and differential expression information is overlaid on the protein-based KEGG pathway image. Pathway nodes shown in white have no genes in the panel that map to them. Pathway nodes in grey have corresponding genes in the panel, however no significant differential expression is observed. Nodes in blue denote downregulation relative to the selected baseline, whereas nodes in orange denote upregulation relative to the selected baseline.

- **More Plot Information**


  For each KEGG pathway, genes within the panel are mapped to the pathway and differential expression information is overlaid on the protein-based KEGG pathway image. Pathway nodes shown in white have no genes in the panel that map to them. Pathway nodes in grey have corresponding genes in the panel, however no significant differential expression is observed. Nodes in blue denote downregulation relative to the selected baseline, whereas nodes in orange denote upregulation relative to the selected baseline.

- **More Plot Information**


  For each KEGG pathway, genes within the panel are mapped to the pathway and differential expression information is overlaid on the protein-based KEGG pathway image. Pathway nodes shown in white have no genes in the panel that map to them. Pathway nodes in grey have corresponding genes in the panel, however no significant differential expression is observed. Nodes in blue denote downregulation relative to the selected baseline, whereas nodes in orange denote upregulation relative to the selected baseline.

- **More Plot Information**


  For each KEGG pathway, genes within the panel are mapped to the pathway and differential expression information is overlaid on the protein-based KEGG pathway image. Pathway nodes shown in white have no genes in the panel that map to them. Pathway nodes in grey have corresponding genes in the panel, however no significant differential expression is observed. Nodes in blue denote downregulation relative to the selected baseline, whereas nodes in orange denote upregulation relative to the selected baseline.

- **More Plot Information**


  For each KEGG pathway, genes within the panel are mapped to the pathway and differential expression information is overlaid on the protein-based KEGG pathway image. Pathway nodes shown in white have no genes in the panel that map to them. Pathway nodes in grey have corresponding genes in the panel, however no significant differential expression is observed. Nodes in blue denote downregulation relative to the selected baseline, whereas nodes in orange denote upregulation relative to the selected baseline.

- **More Plot Information**


  For each KEGG pathway, genes within the panel are mapped to the pathway and differential expression information is overlaid on the protein-based KEGG pathway image. Pathway nodes shown in white have no genes in the panel that map to them. Pathway nodes in grey have corresponding genes in the panel, however no significant differential expression is observed. Nodes in blue denote downregulation relative to the selected baseline, whereas nodes in orange denote upregulation relative to the selected baseline.

- **More Plot Information**


  For each KEGG pathway, genes within the panel are mapped to the pathway and differential expression information is overlaid on the protein-based KEGG pathway image. Pathway nodes shown in white have no genes in the panel that map to them. Pathway nodes in grey have corresponding genes in the panel, however no significant differential expression is observed. Nodes in blue denote downregulation relative to the selected baseline, whereas nodes in orange denote upregulation relative to the selected baseline.

- **More Plot Information**


  For each KEGG pathway, genes within the panel are mapped to the pathway and differential expression information is overlaid on the protein-based KEGG pathway image. Pathway nodes shown in white have no genes in the panel that map to them. Pathway nodes in grey have corresponding genes in the panel, however no significant differential expression is observed. Nodes in blue denote downregulation relative to the selected baseline, whereas nodes in orange denote upregulation relative to the selected baseline.

- **More Plot Information**


  For each KEGG pathway, genes within the panel are mapped to the pathway and differential expression information is overlaid on the protein-based KEGG pathway image. Pathway nodes shown in white have no genes in the panel that map to them. Pathway nodes in grey have corresponding genes in the panel, however no significant differential expression is observed. Nodes in blue denote downregulation relative to the selected baseline, whereas nodes in orange denote upregulation relative to the selected baseline.

- **More Plot Information**


  For each KEGG pathway, genes within the panel are mapped to the pathway and differential expression information is overlaid on the protein-based KEGG pathway image. Pathway nodes shown in white have no genes in the panel that map to them. Pathway nodes in grey have corresponding genes in the panel, however no significant differential expression is observed. Nodes in blue denote downregulation relative to the selected baseline, whereas nodes in orange denote upregulation relative to the selected baseline.

- **More Plot Information**


  For each KEGG pathway, genes within the panel are mapped to the pathway and differential expression information is overlaid on the protein-based KEGG pathway image. Pathway nodes shown in white have no genes in the panel that map to them. Pathway nodes in grey have corresponding genes in the panel, however no significant differential expression is observed. Nodes in blue denote downregulation relative to the selected baseline, whereas nodes in orange denote upregulation relative to the selected baseline.

- **More Plot Information**


  For each KEGG pathway, genes within the panel are mapped to the pathway and differential expression information is overlaid on the protein-based KEGG pathway image. Pathway nodes shown in white have no genes in the panel that map to them. Pathway nodes in grey have corresponding genes in the panel, however no significant differential expression is observed. Nodes in blue denote downregulation relative to the selected baseline, whereas nodes in orange denote upregulation relative to the selected baseline.

- **More Plot Information**


  For each KEGG pathway, genes within the panel are mapped to the pathway and differential expression information is overlaid on the protein-based KEGG pathway image. Pathway nodes shown in white have no genes in the panel that map to them. Pathway nodes in grey have corresponding genes in the panel, however no significant differential expression is observed. Nodes in blue denote downregulation relative to the selected baseline, whereas nodes in orange denote upregulation relative to the selected baseline.

- **More Plot Information**


  For each KEGG pathway, genes within the panel are mapped to the pathway and differential expression information is overlaid on the protein-based KEGG pathway image. Pathway nodes shown in white have no genes in the panel that map to them. Pathway nodes in grey have corresponding genes in the panel, however no significant differential expression is observed. Nodes in blue denote downregulation relative to the selected baseline, whereas nodes in orange denote upregulation relative to the selected baseline.

- **More Plot Information**


  For each KEGG pathway, genes within the panel are mapped to the pathway and differential expression information is overlaid on the protein-based KEGG pathway image. Pathway nodes shown in white have no genes in the panel that map to them. Pathway nodes in grey have corresponding genes in the panel, however no significant differential expression is observed. Nodes in blue denote downregulation relative to the selected baseline, whereas nodes in orange denote upregulation relative to the selected baseline.

- **More Plot Information**


  For each KEGG pathway, genes within the panel are mapped to the pathway and differential expression information is overlaid on the protein-based KEGG pathway image. Pathway nodes shown in white have no genes in the panel that map to them. Pathway nodes in grey have corresponding genes in the panel, however no significant differential expression is observed. Nodes in blue denote downregulation relative to the selected baseline, whereas nodes in orange denote upregulation relative to the selected baseline.

- **More Plot Information**


  For each KEGG pathway, genes within the panel are mapped to the pathway and differential expression information is overlaid on the protein-based KEGG pathway image. Pathway nodes shown in white have no genes in the panel that map to them. Pathway nodes in grey have corresponding genes in the panel, however no significant differential expression is observed. Nodes in blue denote downregulation relative to the selected baseline, whereas nodes in orange denote upregulation relative to the selected baseline.

- **More Plot Information**


  For each KEGG pathway, genes within the panel are mapped to the pathway and differential expression information is overlaid on the protein-based KEGG pathway image. Pathway nodes shown in white have no genes in the panel that map to them. Pathway nodes in grey have corresponding genes in the panel, however no significant differential expression is observed. Nodes in blue denote downregulation relative to the selected baseline, whereas nodes in orange denote upregulation relative to the selected baseline.

- **More Plot Information**


  For each KEGG pathway, genes within the panel are mapped to the pathway and differential expression information is overlaid on the protein-based KEGG pathway image. Pathway nodes shown in white have no genes in the panel that map to them. Pathway nodes in grey have corresponding genes in the panel, however no significant differential expression is observed. Nodes in blue denote downregulation relative to the selected baseline, whereas nodes in orange denote upregulation relative to the selected baseline.

- **More Plot Information**


  For each KEGG pathway, genes within the panel are mapped to the pathway and differential expression information is overlaid on the protein-based KEGG pathway image. Pathway nodes shown in white have no genes in the panel that map to them. Pathway nodes in grey have corresponding genes in the panel, however no significant differential expression is observed. Nodes in blue denote downregulation relative to the selected baseline, whereas nodes in orange denote upregulation relative to the selected baseline.

Pathways:

- Osteoclast differentiation
- Pathways in cancer
- Tuberculosis
- Influenza A
- HTLV-I infection
- Hepatitis B
- TNF signaling pathway
- Hepatitis C
- Apoptosis
- Cytokine-cytokine receptor interaction
- MAPK signaling pathway
- Chagas disease (American trypanosomiasis)
- PI3K-Akt signaling pathway
- Epstein-Barr virus infection
- Toll-like receptor signaling pathway
- NF-kappa B signaling pathway
- Herpes simplex infection
- Viral carcinogenesis
- Measles
- Ras signaling pathway

- **More Plot Information**


  For each KEGG pathway, genes within the panel are mapped to the pathway and differential expression information is overlaid on the protein-based KEGG pathway image. Pathway nodes shown in white have no genes in the panel that map to them. Pathway nodes in grey have corresponding genes in the panel, however no significant differential expression is observed. Nodes in blue denote downregulation relative to the selected baseline, whereas nodes in orange denote upregulation relative to the selected baseline.

- **More Plot Information**


  For each KEGG pathway, genes within the panel are mapped to the pathway and differential expression information is overlaid on the protein-based KEGG pathway image. Pathway nodes shown in white have no genes in the panel that map to them. Pathway nodes in grey have corresponding genes in the panel, however no significant differential expression is observed. Nodes in blue denote downregulation relative to the selected baseline, whereas nodes in orange denote upregulation relative to the selected baseline.

- **More Plot Information**


  For each KEGG pathway, genes within the panel are mapped to the pathway and differential expression information is overlaid on the protein-based KEGG pathway image. Pathway nodes shown in white have no genes in the panel that map to them. Pathway nodes in grey have corresponding genes in the panel, however no significant differential expression is observed. Nodes in blue denote downregulation relative to the selected baseline, whereas nodes in orange denote upregulation relative to the selected baseline.

- **More Plot Information**


  For each KEGG pathway, genes within the panel are mapped to the pathway and differential expression information is overlaid on the protein-based KEGG pathway image. Pathway nodes shown in white have no genes in the panel that map to them. Pathway nodes in grey have corresponding genes in the panel, however no significant differential expression is observed. Nodes in blue denote downregulation relative to the selected baseline, whereas nodes in orange denote upregulation relative to the selected baseline.

- **More Plot Information**


  For each KEGG pathway, genes within the panel are mapped to the pathway and differential expression information is overlaid on the protein-based KEGG pathway image. Pathway nodes shown in white have no genes in the panel that map to them. Pathway nodes in grey have corresponding genes in the panel, however no significant differential expression is observed. Nodes in blue denote downregulation relative to the selected baseline, whereas nodes in orange denote upregulation relative to the selected baseline.

- **More Plot Information**


  For each KEGG pathway, genes within the panel are mapped to the pathway and differential expression information is overlaid on the protein-based KEGG pathway image. Pathway nodes shown in white have no genes in the panel that map to them. Pathway nodes in grey have corresponding genes in the panel, however no significant differential expression is observed. Nodes in blue denote downregulation relative to the selected baseline, whereas nodes in orange denote upregulation relative to the selected baseline.

- **More Plot Information**


  For each KEGG pathway, genes within the panel are mapped to the pathway and differential expression information is overlaid on the protein-based KEGG pathway image. Pathway nodes shown in white have no genes in the panel that map to them. Pathway nodes in grey have corresponding genes in the panel, however no significant differential expression is observed. Nodes in blue denote downregulation relative to the selected baseline, whereas nodes in orange denote upregulation relative to the selected baseline.

- **More Plot Information**


  For each KEGG pathway, genes within the panel are mapped to the pathway and differential expression information is overlaid on the protein-based KEGG pathway image. Pathway nodes shown in white have no genes in the panel that map to them. Pathway nodes in grey have corresponding genes in the panel, however no significant differential expression is observed. Nodes in blue denote downregulation relative to the selected baseline, whereas nodes in orange denote upregulation relative to the selected baseline.

- **More Plot Information**


  For each KEGG pathway, genes within the panel are mapped to the pathway and differential expression information is overlaid on the protein-based KEGG pathway image. Pathway nodes shown in white have no genes in the panel that map to them. Pathway nodes in grey have corresponding genes in the panel, however no significant differential expression is observed. Nodes in blue denote downregulation relative to the selected baseline, whereas nodes in orange denote upregulation relative to the selected baseline.

- **More Plot Information**


  For each KEGG pathway, genes within the panel are mapped to the pathway and differential expression information is overlaid on the protein-based KEGG pathway image. Pathway nodes shown in white have no genes in the panel that map to them. Pathway nodes in grey have corresponding genes in the panel, however no significant differential expression is observed. Nodes in blue denote downregulation relative to the selected baseline, whereas nodes in orange denote upregulation relative to the selected baseline.

- **More Plot Information**


  For each KEGG pathway, genes within the panel are mapped to the pathway and differential expression information is overlaid on the protein-based KEGG pathway image. Pathway nodes shown in white have no genes in the panel that map to them. Pathway nodes in grey have corresponding genes in the panel, however no significant differential expression is observed. Nodes in blue denote downregulation relative to the selected baseline, whereas nodes in orange denote upregulation relative to the selected baseline.

- **More Plot Information**


  For each KEGG pathway, genes within the panel are mapped to the pathway and differential expression information is overlaid on the protein-based KEGG pathway image. Pathway nodes shown in white have no genes in the panel that map to them. Pathway nodes in grey have corresponding genes in the panel, however no significant differential expression is observed. Nodes in blue denote downregulation relative to the selected baseline, whereas nodes in orange denote upregulation relative to the selected baseline.

- **More Plot Information**


  For each KEGG pathway, genes within the panel are mapped to the pathway and differential expression information is overlaid on the protein-based KEGG pathway image. Pathway nodes shown in white have no genes in the panel that map to them. Pathway nodes in grey have corresponding genes in the panel, however no significant differential expression is observed. Nodes in blue denote downregulation relative to the selected baseline, whereas nodes in orange denote upregulation relative to the selected baseline.

- **More Plot Information**


  For each KEGG pathway, genes within the panel are mapped to the pathway and differential expression information is overlaid on the protein-based KEGG pathway image. Pathway nodes shown in white have no genes in the panel that map to them. Pathway nodes in grey have corresponding genes in the panel, however no significant differential expression is observed. Nodes in blue denote downregulation relative to the selected baseline, whereas nodes in orange denote upregulation relative to the selected baseline.

- **More Plot Information**


  For each KEGG pathway, genes within the panel are mapped to the pathway and differential expression information is overlaid on the protein-based KEGG pathway image. Pathway nodes shown in white have no genes in the panel that map to them. Pathway nodes in grey have corresponding genes in the panel, however no significant differential expression is observed. Nodes in blue denote downregulation relative to the selected baseline, whereas nodes in orange denote upregulation relative to the selected baseline.

- **More Plot Information**


  For each KEGG pathway, genes within the panel are mapped to the pathway and differential expression information is overlaid on the protein-based KEGG pathway image. Pathway nodes shown in white have no genes in the panel that map to them. Pathway nodes in grey have corresponding genes in the panel, however no significant differential expression is observed. Nodes in blue denote downregulation relative to the selected baseline, whereas nodes in orange denote upregulation relative to the selected baseline.

- **More Plot Information**


  For each KEGG pathway, genes within the panel are mapped to the pathway and differential expression information is overlaid on the protein-based KEGG pathway image. Pathway nodes shown in white have no genes in the panel that map to them. Pathway nodes in grey have corresponding genes in the panel, however no significant differential expression is observed. Nodes in blue denote downregulation relative to the selected baseline, whereas nodes in orange denote upregulation relative to the selected baseline.

- **More Plot Information**


  For each KEGG pathway, genes within the panel are mapped to the pathway and differential expression information is overlaid on the protein-based KEGG pathway image. Pathway nodes shown in white have no genes in the panel that map to them. Pathway nodes in grey have corresponding genes in the panel, however no significant differential expression is observed. Nodes in blue denote downregulation relative to the selected baseline, whereas nodes in orange denote upregulation relative to the selected baseline.

- **More Plot Information**


  For each KEGG pathway, genes within the panel are mapped to the pathway and differential expression information is overlaid on the protein-based KEGG pathway image. Pathway nodes shown in white have no genes in the panel that map to them. Pathway nodes in grey have corresponding genes in the panel, however no significant differential expression is observed. Nodes in blue denote downregulation relative to the selected baseline, whereas nodes in orange denote upregulation relative to the selected baseline.

- **More Plot Information**


  For each KEGG pathway, genes within the panel are mapped to the pathway and differential expression information is overlaid on the protein-based KEGG pathway image. Pathway nodes shown in white have no genes in the panel that map to them. Pathway nodes in grey have corresponding genes in the panel, however no significant differential expression is observed. Nodes in blue denote downregulation relative to the selected baseline, whereas nodes in orange denote upregulation relative to the selected baseline.

Pathways:

- HTLV-I infection
- Osteoclast differentiation
- Hepatitis C
- MAPK signaling pathway
- Hepatitis B
- Epstein-Barr virus infection
- Influenza A
- NF-kappa B signaling pathway
- Chagas disease (American trypanosomiasis)
- Pathways in cancer
- Apoptosis
- Cytokine-cytokine receptor interaction
- Tuberculosis
- Measles
- Herpes simplex infection
- TNF signaling pathway
- Viral carcinogenesis
- PI3K-Akt signaling pathway
- Toll-like receptor signaling pathway
- Ras signaling pathway

- **More Plot Information**


  For each KEGG pathway, genes within the panel are mapped to the pathway and differential expression information is overlaid on the protein-based KEGG pathway image. Pathway nodes shown in white have no genes in the panel that map to them. Pathway nodes in grey have corresponding genes in the panel, however no significant differential expression is observed. Nodes in blue denote downregulation relative to the selected baseline, whereas nodes in orange denote upregulation relative to the selected baseline.

- **More Plot Information**


  For each KEGG pathway, genes within the panel are mapped to the pathway and differential expression information is overlaid on the protein-based KEGG pathway image. Pathway nodes shown in white have no genes in the panel that map to them. Pathway nodes in grey have corresponding genes in the panel, however no significant differential expression is observed. Nodes in blue denote downregulation relative to the selected baseline, whereas nodes in orange denote upregulation relative to the selected baseline.

- **More Plot Information**


  For each KEGG pathway, genes within the panel are mapped to the pathway and differential expression information is overlaid on the protein-based KEGG pathway image. Pathway nodes shown in white have no genes in the panel that map to them. Pathway nodes in grey have corresponding genes in the panel, however no significant differential expression is observed. Nodes in blue denote downregulation relative to the selected baseline, whereas nodes in orange denote upregulation relative to the selected baseline.

- **More Plot Information**


  For each KEGG pathway, genes within the panel are mapped to the pathway and differential expression information is overlaid on the protein-based KEGG pathway image. Pathway nodes shown in white have no genes in the panel that map to them. Pathway nodes in grey have corresponding genes in the panel, however no significant differential expression is observed. Nodes in blue denote downregulation relative to the selected baseline, whereas nodes in orange denote upregulation relative to the selected baseline.

- **More Plot Information**


  For each KEGG pathway, genes within the panel are mapped to the pathway and differential expression information is overlaid on the protein-based KEGG pathway image. Pathway nodes shown in white have no genes in the panel that map to them. Pathway nodes in grey have corresponding genes in the panel, however no significant differential expression is observed. Nodes in blue denote downregulation relative to the selected baseline, whereas nodes in orange denote upregulation relative to the selected baseline.

- **More Plot Information**


  For each KEGG pathway, genes within the panel are mapped to the pathway and differential expression information is overlaid on the protein-based KEGG pathway image. Pathway nodes shown in white have no genes in the panel that map to them. Pathway nodes in grey have corresponding genes in the panel, however no significant differential expression is observed. Nodes in blue denote downregulation relative to the selected baseline, whereas nodes in orange denote upregulation relative to the selected baseline.

- **More Plot Information**


  For each KEGG pathway, genes within the panel are mapped to the pathway and differential expression information is overlaid on the protein-based KEGG pathway image. Pathway nodes shown in white have no genes in the panel that map to them. Pathway nodes in grey have corresponding genes in the panel, however no significant differential expression is observed. Nodes in blue denote downregulation relative to the selected baseline, whereas nodes in orange denote upregulation relative to the selected baseline.

- **More Plot Information**


  For each KEGG pathway, genes within the panel are mapped to the pathway and differential expression information is overlaid on the protein-based KEGG pathway image. Pathway nodes shown in white have no genes in the panel that map to them. Pathway nodes in grey have corresponding genes in the panel, however no significant differential expression is observed. Nodes in blue denote downregulation relative to the selected baseline, whereas nodes in orange denote upregulation relative to the selected baseline.

- **More Plot Information**


  For each KEGG pathway, genes within the panel are mapped to the pathway and differential expression information is overlaid on the protein-based KEGG pathway image. Pathway nodes shown in white have no genes in the panel that map to them. Pathway nodes in grey have corresponding genes in the panel, however no significant differential expression is observed. Nodes in blue denote downregulation relative to the selected baseline, whereas nodes in orange denote upregulation relative to the selected baseline.

- **More Plot Information**


  For each KEGG pathway, genes within the panel are mapped to the pathway and differential expression information is overlaid on the protein-based KEGG pathway image. Pathway nodes shown in white have no genes in the panel that map to them. Pathway nodes in grey have corresponding genes in the panel, however no significant differential expression is observed. Nodes in blue denote downregulation relative to the selected baseline, whereas nodes in orange denote upregulation relative to the selected baseline.

- **More Plot Information**


  For each KEGG pathway, genes within the panel are mapped to the pathway and differential expression information is overlaid on the protein-based KEGG pathway image. Pathway nodes shown in white have no genes in the panel that map to them. Pathway nodes in grey have corresponding genes in the panel, however no significant differential expression is observed. Nodes in blue denote downregulation relative to the selected baseline, whereas nodes in orange denote upregulation relative to the selected baseline.

- **More Plot Information**


  For each KEGG pathway, genes within the panel are mapped to the pathway and differential expression information is overlaid on the protein-based KEGG pathway image. Pathway nodes shown in white have no genes in the panel that map to them. Pathway nodes in grey have corresponding genes in the panel, however no significant differential expression is observed. Nodes in blue denote downregulation relative to the selected baseline, whereas nodes in orange denote upregulation relative to the selected baseline.

- **More Plot Information**


  For each KEGG pathway, genes within the panel are mapped to the pathway and differential expression information is overlaid on the protein-based KEGG pathway image. Pathway nodes shown in white have no genes in the panel that map to them. Pathway nodes in grey have corresponding genes in the panel, however no significant differential expression is observed. Nodes in blue denote downregulation relative to the selected baseline, whereas nodes in orange denote upregulation relative to the selected baseline.

- **More Plot Information**


  For each KEGG pathway, genes within the panel are mapped to the pathway and differential expression information is overlaid on the protein-based KEGG pathway image. Pathway nodes shown in white have no genes in the panel that map to them. Pathway nodes in grey have corresponding genes in the panel, however no significant differential expression is observed. Nodes in blue denote downregulation relative to the selected baseline, whereas nodes in orange denote upregulation relative to the selected baseline.

- **More Plot Information**


  For each KEGG pathway, genes within the panel are mapped to the pathway and differential expression information is overlaid on the protein-based KEGG pathway image. Pathway nodes shown in white have no genes in the panel that map to them. Pathway nodes in grey have corresponding genes in the panel, however no significant differential expression is observed. Nodes in blue denote downregulation relative to the selected baseline, whereas nodes in orange denote upregulation relative to the selected baseline.

- **More Plot Information**


  For each KEGG pathway, genes within the panel are mapped to the pathway and differential expression information is overlaid on the protein-based KEGG pathway image. Pathway nodes shown in white have no genes in the panel that map to them. Pathway nodes in grey have corresponding genes in the panel, however no significant differential expression is observed. Nodes in blue denote downregulation relative to the selected baseline, whereas nodes in orange denote upregulation relative to the selected baseline.

- **More Plot Information**


  For each KEGG pathway, genes within the panel are mapped to the pathway and differential expression information is overlaid on the protein-based KEGG pathway image. Pathway nodes shown in white have no genes in the panel that map to them. Pathway nodes in grey have corresponding genes in the panel, however no significant differential expression is observed. Nodes in blue denote downregulation relative to the selected baseline, whereas nodes in orange denote upregulation relative to the selected baseline.

- **More Plot Information**


  For each KEGG pathway, genes within the panel are mapped to the pathway and differential expression information is overlaid on the protein-based KEGG pathway image. Pathway nodes shown in white have no genes in the panel that map to them. Pathway nodes in grey have corresponding genes in the panel, however no significant differential expression is observed. Nodes in blue denote downregulation relative to the selected baseline, whereas nodes in orange denote upregulation relative to the selected baseline.

- **More Plot Information**


  For each KEGG pathway, genes within the panel are mapped to the pathway and differential expression information is overlaid on the protein-based KEGG pathway image. Pathway nodes shown in white have no genes in the panel that map to them. Pathway nodes in grey have corresponding genes in the panel, however no significant differential expression is observed. Nodes in blue denote downregulation relative to the selected baseline, whereas nodes in orange denote upregulation relative to the selected baseline.

- **More Plot Information**


  For each KEGG pathway, genes within the panel are mapped to the pathway and differential expression information is overlaid on the protein-based KEGG pathway image. Pathway nodes shown in white have no genes in the panel that map to them. Pathway nodes in grey have corresponding genes in the panel, however no significant differential expression is observed. Nodes in blue denote downregulation relative to the selected baseline, whereas nodes in orange denote upregulation relative to the selected baseline.

Genes previously shown to be characteristic of various cell populations are used to measure these populations' abundance.
QC plots explore the validity of each cell type's measurements, Summary plots explore the joint behavior of cell types, and Covariates plots compare cell type measurements to covariates.
In addition to raw cell type abundance measurements, relative abundances measuring various contrasts between cell types are reported.

- QC
- Summary
- Covariates

- QC

- Raw

- Raw

Choose a Cell Type:

- Summary
- Astrocytes
- DC
- Endothelial Cells
- Exhausted CD8
- Macrophages
- Neuron
- Oligodendrocytes
- Treg

- **Barplot of p-values across cell types  
  More Plot Information**


  ##### Barplot of p-values across cell types

  Barplot of p-values across cell types. p-values are -log10 transformed. Bars above the solid black line indicate statistically significant cell types at an alpha level of 0.01. Bars above the dashed black line indicate statistically significant cell types at an alpha level of 0.001.

- **QC Plot for Cell Type Measurements: Astrocytes  
  More Plot Information**


  ##### QC Plot for Cell Type Measurements: Astrocytes

  Normalized expression of the genes characteristic of Astrocytes.
  If a cell type's characteristic genes are specific to the cell type and stably expressed within it, they will be strongly correlated with a slope of 1. Substantial departures from this pattern indicate noisier quantification of cell type abundance.

- **QC Plot for Cell Type Measurements: DC  
  More Plot Information**


  ##### QC Plot for Cell Type Measurements: DC

  Normalized expression of the genes characteristic of DC.
  If a cell type's characteristic genes are specific to the cell type and stably expressed within it, they will be strongly correlated with a slope of 1. Substantial departures from this pattern indicate noisier quantification of cell type abundance.

- **QC Plot for Cell Type Measurements: Endothelial Cells  
  More Plot Information**


  ##### QC Plot for Cell Type Measurements: Endothelial Cells

  Normalized expression of the genes characteristic of Endothelial Cells.
  If a cell type's characteristic genes are specific to the cell type and stably expressed within it, they will be strongly correlated with a slope of 1. Substantial departures from this pattern indicate noisier quantification of cell type abundance.

- **QC Plot for Cell Type Measurements: Exhausted CD8  
  More Plot Information**


  ##### QC Plot for Cell Type Measurements: Exhausted CD8

  Normalized expression of the genes characteristic of Exhausted CD8.
  If a cell type's characteristic genes are specific to the cell type and stably expressed within it, they will be strongly correlated with a slope of 1. Substantial departures from this pattern indicate noisier quantification of cell type abundance.

- **QC Plot for Cell Type Measurements: Macrophages  
  More Plot Information**


  ##### QC Plot for Cell Type Measurements: Macrophages

  Normalized expression of the genes characteristic of Macrophages.
  If a cell type's characteristic genes are specific to the cell type and stably expressed within it, they will be strongly correlated with a slope of 1. Substantial departures from this pattern indicate noisier quantification of cell type abundance.

- **QC Plot for Cell Type Measurements: Neuron  
  More Plot Information**


  ##### QC Plot for Cell Type Measurements: Neuron

  Normalized expression of the genes characteristic of Neuron.
  If a cell type's characteristic genes are specific to the cell type and stably expressed within it, they will be strongly correlated with a slope of 1. Substantial departures from this pattern indicate noisier quantification of cell type abundance.

- **QC Plot for Cell Type Measurements: Oligodendrocytes  
  More Plot Information**


  ##### QC Plot for Cell Type Measurements: Oligodendrocytes

  Normalized expression of the genes characteristic of Oligodendrocytes.
  If a cell type's characteristic genes are specific to the cell type and stably expressed within it, they will be strongly correlated with a slope of 1. Substantial departures from this pattern indicate noisier quantification of cell type abundance.

- **QC Plot for Cell Type Measurements: Treg  
  More Plot Information**


  ##### QC Plot for Cell Type Measurements: Treg

  Normalized expression of the genes characteristic of Treg.
  If a cell type's characteristic genes are specific to the cell type and stably expressed within it, they will be strongly correlated with a slope of 1. Substantial departures from this pattern indicate noisier quantification of cell type abundance.

Choose a Cell Type:

- Summary
- Macrophages
- DC
- Endothelial Cells
- Neuron
- Exhausted CD8
- Oligodendrocytes
- Astrocytes
- Treg

- **Heatmap of Raw cell type measurements  
  More Plot Information  Download raw cell type measurements**


  ##### Heatmap of Raw cell type measurements

  Heatmap showing raw abundance of cell types. Orange indicates high abundance; blue indicates low abundance. Note that our methods do not support the conclusion that one cell type is more abundant than another. Rather, they support the claim that a given cell type is more abundant in one sample than in another.
- **Heatmap of Correlation Matrix of Raw cell type measurements  
  More Plot Information**


  ##### Heatmap of Correlation Matrix of Raw cell type measurements

  Heatmap showing the correlation matrix of raw cell abundance. Orange and blue indicate positive and negative correlation, respectively.

- **Macrophages measurements vs. other cell type measurements  
  More Plot Information**


  ##### Macrophages measurements vs. other cell type measurements

  Each panel plots Macrophages against another raw abundance measurements. Points are colored by DPI.

- **DC measurements vs. other cell type measurements  
  More Plot Information**


  ##### DC measurements vs. other cell type measurements

  Each panel plots DC against another raw abundance measurements. Points are colored by DPI.

- **Endothelial Cells measurements vs. other cell type measurements  
  More Plot Information**


  ##### Endothelial Cells measurements vs. other cell type measurements

  Each panel plots Endothelial Cells against another raw abundance measurements. Points are colored by DPI.

- **Neuron measurements vs. other cell type measurements  
  More Plot Information**


  ##### Neuron measurements vs. other cell type measurements

  Each panel plots Neuron against another raw abundance measurements. Points are colored by DPI.

- **Exhausted CD8 measurements vs. other cell type measurements  
  More Plot Information**


  ##### Exhausted CD8 measurements vs. other cell type measurements

  Each panel plots Exhausted CD8 against another raw abundance measurements. Points are colored by DPI.

- **Oligodendrocytes measurements vs. other cell type measurements  
  More Plot Information**


  ##### Oligodendrocytes measurements vs. other cell type measurements

  Each panel plots Oligodendrocytes against another raw abundance measurements. Points are colored by DPI.

- **Astrocytes measurements vs. other cell type measurements  
  More Plot Information**


  ##### Astrocytes measurements vs. other cell type measurements

  Each panel plots Astrocytes against another raw abundance measurements. Points are colored by DPI.

- **Treg measurements vs. other cell type measurements  
  More Plot Information**


  ##### Treg measurements vs. other cell type measurements

  Each panel plots Treg against another raw abundance measurements. Points are colored by DPI.

Choose a Cell Type:

- Summary
- Macrophages
- DC
- Endothelial Cells
- Neuron
- Exhausted CD8
- Oligodendrocytes
- Astrocytes
- Treg

- **Raw cell type measurements vs. DPI  
  More Plot Information**


  ##### Raw cell type measurements vs. DPI

  Plots the raw cell type abundance measurements against DPI.

- **Macrophages Measurement vs. DPI  
  More Plot Information**


  ##### Macrophages Measurement vs. DPI

  Plots Macrophages measurements against DPI.

- **DC Measurement vs. DPI  
  More Plot Information**


  ##### DC Measurement vs. DPI

  Plots DC measurements against DPI.

- **Endothelial Cells Measurement vs. DPI  
  More Plot Information**


  ##### Endothelial Cells Measurement vs. DPI

  Plots Endothelial Cells measurements against DPI.

- **Neuron Measurement vs. DPI  
  More Plot Information**


  ##### Neuron Measurement vs. DPI

  Plots Neuron measurements against DPI.

- **Exhausted CD8 Measurement vs. DPI  
  More Plot Information**


  ##### Exhausted CD8 Measurement vs. DPI

  Plots Exhausted CD8 measurements against DPI.

- **Oligodendrocytes Measurement vs. DPI  
  More Plot Information**


  ##### Oligodendrocytes Measurement vs. DPI

  Plots Oligodendrocytes measurements against DPI.

- **Astrocytes Measurement vs. DPI  
  More Plot Information**


  ##### Astrocytes Measurement vs. DPI

  Plots Astrocytes measurements against DPI.

- **Treg Measurement vs. DPI  
  More Plot Information**


  ##### Treg Measurement vs. DPI

  Plots Treg measurements against DPI.

Summary of the options that were chosen when running this analysis.

##### **Analysis Details**

Analysis name: Yp\_Final   
 Location of results: C:\Users\basmi\OneDrive\Documents\Army\RIID\Analysis   
 File type of plots: png

##### **Sample Annotation Details**

Unique sample identifier: File.Name   
Covariate1: DPI; Variable Type: categorical; Reference Level: CTRL

##### **Gene Annotation Details**

Column defining probe set membership: Probe.Annotation   
 Probe annotation column giving cell type-specific probes: Cell.Type

##### **Fusion Analysis Details**

Perform fusion analysis: No   
 p-value end probe threshold: 0.05   
 p-value junction probe threshold: 0.05

##### **SNV Analysis Details**

Perform snv analysis: No   
 Fold change threshold(s): 1, 2   
 p-value threshold(s): 0.01, 0.01   
 Reference samples:  
 NA   
 Use EM post processing: Yes   
 Use debiasing post processing: Yes

##### **Normalization Details**

Perform normalization on: none   
 Number of normalization probes: NA

##### **Pathway Scoring Analysis Details**

Perform pathway scoring analysis: TRUE   
 Covariates to include in plots: DPI

##### **Differential Expression Analysis Details**

Perform differential expression testing: TRUE   
 Predictors: DPI   
 Confounders: none   
 P-value adjustment: BY   
 Run gene set analysis TRUE

##### **Pathview Details**

Display results using Pathview: TRUE   
 Color Pathview pathview plots by: Fold-change   
 P-value threshold: 0.05   
 Additional KEGG IDs: none

##### **Cell Type Profiling Details**

Perform cell type profiling: TRUE   
 Covariates to include in plots: DPI   
 Use all cell type genes or dynamically select a subset:   
 P-value threshold for reporting a cell type's results: 1   
 Show results at the level of raw cell type abundance: TRUE   
 Show results at the level of relative cell type abundance: TRUE   
 Use default contrasts for calculating relative cell type abundance: TRUE

##### **Probe Descriptive Analysis Details**

Run Probe Descriptive analysis: FALSE

##### **Related Analytes Analysis Details**

Related Analytes Analysis: FALSE

##### **Warnings**

T-cells, CD8 T cells, Microglia, Cytotoxic cells, Mast cells, Neutrophils, CD45, NK CD56dim cells, B-cells, Th1 cells, NK cells cell types dropped from Cell Type Profiling as all probes were discarded due to signals below background threshold expression.  
  
Warning: the following cell types in the cell types contrast file were not found in the probe annotation file's cell type column:  
 B-cells, CD45, Cytotoxic cells, Mast cells, Neutrophils, NK CD56dim cells, NK cells, T-cells, Th1 cells, CD8 T cells   
 Therefore the following contrasts have been discarded:  
 Total.TILs  
B.cells.vs..TILs  
Cytotoxic.cells.vs..TILs  
DC.vs..TILs  
Exhausted.CD8.vs..TILs  
Macrophages.vs..TILs  
Mast.cells.vs..TILs  
Neutrophils.vs..TILs  
NK.CD56dim.cells.vs..TILs  
NK.cells.vs..TILs  
T.cells.vs..TILs  
Th1.cells.vs..TILs  
Treg.vs..TILs  
CD8.T.cells.vs..TILs  
CD8.vs..Exhasuted.CD8  
CD8.vs..Treg  
CD8.vs..T.cells   
 Warning: no cell type contrasts remained with valid data. Only raw cell type abundance will be reported.
